# Putatively novel serotypes and the potential for reduced vaccine effectiveness: capsular locus diversity revealed among 5405 pneumococcal genomes

**DOI:** 10.1099/mgen.0.000090

**Published:** 2016-10-21

**Authors:** Andries J. van Tonder, James E. Bray, Sigríður J. Quirk, Gunnsteinn Haraldsson, Keith A. Jolley, Martin C. J. Maiden, Steen Hoffmann, Stephen D. Bentley, Ásgeir Haraldsson, Helga Erlendsdóttir, Karl G. Kristinsson, Angela B. Brueggemann

**Affiliations:** ^1^​ Nuffield Department of Medicine, University of Oxford, Oxford, United Kingdom; ^2^​ Department of Zoology, University of Oxford, Oxford, United Kingdom; ^3^​ Clinical Microbiology, University of Iceland and Landspitali University Hospital, Reykjavik, Iceland; ^4^​ Department of Microbiology and Infection Control, Statens Serum Institut, Copenhagen, Denmark; ^5^​ Pathogen Genomics, Wellcome Trust Sanger Institute, Hinxton, United Kingdom

**Keywords:** pneumococcal capsular locus, molecular epidemiology, vaccine impact, sequence-based serotyping

## Abstract

The pneumococcus is a leading global pathogen and a key virulence factor possessed by the majority of pneumococci is an antigenic polysaccharide capsule (‘serotype’), which is encoded by the capsular (*cps*) locus. Approximately 100 different serotypes are known, but the extent of sequence diversity within the *cps* loci of individual serotypes is not well understood. Investigating serotype-specific sequence variation is crucial to the design of sequence-based serotyping methodology, understanding pneumococcal conjugate vaccine (PCV) effectiveness and the design of future PCVs. The availability of large genome datasets makes it possible to assess population-level variation among pneumococcal serotypes and in this study 5405 pneumococcal genomes were used to investigate *cps* locus diversity among 49 different serotypes. Pneumococci had been recovered between 1916 and 2014 from people of all ages living in 51 countries. Serotypes were deduced bioinformatically, *cps* locus sequences were extracted and variation was assessed within the *cps* locus, in the context of pneumococcal genetic lineages. Overall, *cps* locus sequence diversity varied markedly: low to moderate diversity was revealed among serogroups/types 1, 3, 7, 9, 11 and 22; whereas serogroups/types 6, 19, 23, 14, 15, 18, 33 and 35 displayed high diversity. Putative novel and/or hybrid *cps* loci were identified among all serogroups/types apart from 1, 3 and 9. This study demonstrated that *cps* locus sequence diversity varied widely between serogroups/types. Investigation of the biochemical structure of the polysaccharide capsule of major variants, particularly PCV-related serotypes and those that appear to be novel or hybrids, is warranted.

## Data Summary

1. Table S2 and Figs S2–S15 (available in the online Supplementary Material), have been deposited in FigShare: DOI: 10.6084/m9.figshare.3839898
2. Raw sequencing data for all study genomes have been deposited in the European Nucleotide Archive (ENA). Accession numbers are detailed in Table S2, which has been deposited in FigShare: FigShare: DOI: 10.6084/m9.figshare.3839898
3. All assembled genomes and associated metadata are available in the PubMLST database: http://pubmlst.org/spneumoniae/
4. seqSerotyper.R is available from GitHub under GNU GPL v2: (https://github.com/avantonder/seqSerotyper)

### Significance as a BioResource to the community

A key pneumococcal virulence factor is the antigenic polysaccharide capsule that surrounds the cell and protects it from the host immune system. The capsule is the target for vaccine-mediated protection from pneumococcal infection. Many capsular types (‘serotypes’) are recognized, but diversity within the capsular locus is not well understood. We interrogated a large genome dataset to investigate population-level diversity within capsular genes and revealed that some genes were highly conserved whereas others were diverse. Putatively novel and hybrid capsular sequences were identified. Knowledge of the extent and location of sequence variation informs our view about vaccine effectiveness and vaccine design, and the accuracy of sequence-based serotyping methodology depends on understanding capsular variation. Therefore, this study provides a major advance in our knowledge of the biology and evolution of polysaccharide capsules among a major global pathogen. Investigators interested in molecular biology, bacterial evolution, serotyping, vaccine impact, vaccine design and large-scale genomic analyses will find these data and methods useful. Raw sequencing data are available from the European Nucleotide Archive (ENA) and the assembled genomes are available from the PubMLST website. Pneumococcal strain requests should be directed to the laboratories within which the pneumococci were originally recovered, as detailed in the online Supplementary Material.

## Introduction

*Streptococcus pneumoniae* (the pneumococcus) is a leading global pathogen. Every year diseases like pneumonia, meningitis and bacteraemia claim the lives of an estimated 800 000 children <5 years of age (O'Brien *et al.*, 2009). Safe and effective pneumococcal conjugate vaccines (PCVs) are available and ensuring widespread access to these life-saving vaccines is a global health priority (WHO, 2012). The target for PCV-mediated protection is the antigenic polysaccharide capsule (‘serotype’) that surrounds the bacterial cell wall and inhibits phagocytosis by the human immune system. Antigenically similar serotypes are categorized in serogroups.

Assessing serotype-specific epidemiology pre- and post-PCV introduction is a fundamental component of surveillance programmes and vaccine impact studies. The first PCV (PCV7) was licensed for use in 2000 and included seven serotypes: 4, 6B, 9V, 14, 18C, 19F and 23F. PCV7 was superseded by PCV10, which added serotypes 1, 5 and 7F, and PCV13, which added serotypes 1, 3, 5, 6A, 7F and 19A (Hausdorff *et al.*, 2015). It is now well established that widespread introduction of PCVs alters the distribution of circulating serotypes in both nasopharyngeal colonization (‘carriage’) and disease (Weinberger *et al.*, 2011; Feikin *et al.*, 2013; Hausdorff *et al.*, 2015).

The standard phenotypic methodologies for determining the pneumococcal serotype are the Quellung reaction and latex agglutination; however, PCR-based and microarray-based serotyping have increased in popularity in recent years (Satzke *et al.*, 2015). Furthermore, there is growing interest in deducing the serotype directly from the genome sequence, given that genome sequencing is now relatively straightforward and increasingly utilized. Genome-sequence-based serotyping has the added advantage that putatively novel serotypes can be identified directly from the sequence, whereas all other methods result in a negative or equivocal test result that requires repeat testing. However, confirmation of putatively novel serotypes requires a biochemical assessment of the polysaccharide capsule to confirm novelty, which is not a trivial task.

Every serotyping method has its challenges, but for sequence-based methods a key consideration is the extent of sequence diversity within the *cps* locus (e.g. within the PCR primer-binding region) that might lead to erroneous results. Sequence-based methods are also reliant on having a representative reference set of *cps* loci with which to design the test method and ensure accurate serotype prediction. The first detailed genetic analysis of a single example of each of 90 pneumococcal serotypes was published in 2006, and since then new serotypes have been discovered (Bentley *et al.*, 2006; Calix & Nahm, 2010; Calix *et al.*, 2012). Whether or not the original reference set of 90 *cps* loci are the best representatives of each serotype is unknown, even though they form the basis for the design of many sequence-based serotyping assays or algorithms.

A surprising amount of *cps* locus diversity was recently revealed among serogroup 6 pneumococci (van Tonder *et al.*, 2015). Therefore, in order to further assess pneumococcal serotype diversity, we compiled a large and diverse pneumococcal dataset of 5405 genomes of 14 major serogroups/types, which corresponded to 49 different serotypes. Serogroups/types were selected based upon their overall prevalence, inclusion in current or future PCVs, and invasive disease potential (Brueggemann *et al.*, 2003). The aim of the study was to assess the level of sequence diversity at the *cps* locus for each serogroup/type and identify any unusual serotypes within each serogroup, in the context of the pneumococcal genetic lineages.

## Methods

### Pneumococcal genome sequences and datasets.

Datasets of assembled pneumococcal genomes (*n*=5405; Table 1) from 14 serogroups/types were compiled using previously published genome datasets (Croucher *et al.*, 2011, 2013a, 2013b; Wyres *et al.*, 2012; Chewapreecha *et al.*, 2014; van Tonder *et al.*, 2014; Gladstone *et al.*, 2015; Cornick *et al.*, 2015), sequence data from GenBank (http://www.ncbi.nlm.nih.gov/genbank/), a new set of historical (1937–1996) pneumococcal genomes and new genome data from an ongoing Icelandic vaccine impact study (van Tonder *et al.*, 2015). The genome datasets included 21 Pneumococcal Molecular Epidemiology Network (PMEN) reference strains sequenced by us or downloaded from GenBank (Table S1; McGee *et al.*, 2001).

**Table 1.** Pneumococcal genomes included in *cps* locus analyses

		Serogroup/type													
Genomes^*^	*n*	1	3	6	7	9	11	14	15	18	19	22	23	33	35
Thailand	1875	6	35	396	25	42	49	147	161	32	482	30	325	59	86
Iceland	1536	6	60	338	15	49	78	59	87	22	448	50	255	27	42
USA (MA)	540	0	11	97	14	10	49	4	82	5	104	20	73	5	66
UK	428	2	8	126	6	8	37	7	50	1	50	27	62	16	28
Serotype 1	317	317	0	0	0	0	0	0	0	0	0	0	0	0	0
PMEN2	172	0	0	172	0	0	0	0	0	0	0	0	0	0	0
PMEN1	126	0	0	2	0	0	0	0	0	0	0	0	124	0	0
Historical	100	5	6	2	11	12	5	8	5	7	15	3	9	6	6
Serotype 3	75	0	75	0	0	0	0	0	0	0	0	0	0	0	0
PMEN ref	21	4	0	5	1	1	0	3	0	1	3	0	3	0	0
GenBank	215	8	10	44	4	4	4	30	0	3	83	0	23	2	0
Total n	5405	348	205	1182	76	126	222	258	385	71	1185	130	874	115	228

MA, Massachusetts; PMEN, Pneumococcal Molecular Epidemiology Network.

*Pneumococcal genome studies from which genome sequences were obtained.

Genome sequences downloaded from GenBank were assembled by the original submitter. All other genome sequences were downloaded as raw sequence reads from the European Nucleotide Archive (http://www.ebi.ac.uk/ena), assembled using Velvet (Zerbino & Birney, 2008), assessed for quality and deposited in the Ribosomal Multilocus Sequence Typing (rMLST) database, which is publicly available and powered using BIGSdb (http://pubmlst.org/rmlst/; Jolley & Maiden, 2010; Jolley *et al.*, 2012). Genomes were confirmed to be pneumococci based on analyses of the ribosomal genes and MLST profiles and the presence of a *cps* locus. Metadata for the isolates were manually extracted from the original publications (Table S2). Summaries of the metadata, including demographics, carriage/disease status and whether the pneumococci were recovered pre- or post-PCV are included in Tables 2 and 3.

**Table 2.** Demographic and epidemiological information for serogroups/types with low to moderate diversity in the *cps* locus

Serogroup/type		Years	Countries (*n*)	Genomes (*n*)	Carriage/Disease*			PCV status*		
					Carr	Dis	Unk	Pre	Post	Unk
1	–	1943–2012	22	348	16	260	72	20	5	323
3	–	1961–2014	10	205	88	111	6	167	36	2
7	7A	1937	1	1	0	0	1	1	0	0
	7B	1952–2010	3	13	11	0	2	13	0	0
	7C	1939–2011	4	10	8	0	2	7	2	1
	7F	1962–2014	5	36	16	17	3	6	25	5
	7Hybrid	1952–2009	4	16	11	2	3	14	2	0
9	9A	1962–2009	2	3	0	2	1	2	1	0
	9L	1941–2010	3	11	8	0	3	11	0	0
	9Lii	2008–2010	1	7	7	0	0	7	0	0
	9N	1938–2014	5	36	24	7	5	18	14	4
	9V	1968–2014	6	58	30	25	3	44	11	3
	9Vii	2008–2011	2	11	11	0	0	10	0	1
11	11A	1939–2014	6	166	140	25	1	33	94	39
	11B	1940, 2010	2	2	0	1	1	2	0	0
	11C	1957	1	1	0	0	1	1	0	0
	11D	1986	1	1	0	0	1	1	0	0
	11E	2012	1	1	0	1	0	0	1	0
	11F	1952	1	1	0	0	1	1	0	0
	11Hybrid	2001	1	1	1	0	0	1	0	0
	11X	2008–2010	1	49	49	0	0	49	0	0
22	22A	1939–2009	2	19	17	0	2	19	0	0
	22F	1940–2014	4	98	75	22	1	7	64	27
	22Hybrid	2008–2010	1	13	13	0	0	13	0	0

*Carr, carriage; Dis, disease; Unk, unknown; Pre, recovered pre-PCV; Post, recovered post-PCV.

**Table 3.** Demographic and epidemiological information for serogroups/types with high diversity in the *cps* locus

Serogroup/type		Years	Countries (*n*)	Genomes (*n*)	Carriage/Disease*			PCV status*		
					Carr	Dis	Unk	Pre	Post	Unk
6	6A	1952–2014	5	283	212	68	3	140	110	33
	6Bi	1939–2014	4	171	136	34	1	79	49	43
	6Bii (6E)	1981–2014	16	420	230	164	26	374	37	9
	6C	2001–2014	3	106	88	18	0	8	56	42
	6D	2006–2011	2	5	5	0	0	4	0	1
	6F	2006–2011	1	1	1	0	0	0	0	1
	6Hybrid	1978–2014	5	196	180	13	3	166	21	9
19	19A	1939–2014	5	210	141	65	4	56	144	10
	19B	1971	1	1	0	0	1	1	0	0
	19C	1939	1	1	0	0	1	1	0	0
	19F	1952–2014	9	745	470	271	4	555	178	12
	19Hybrid	1952–2014	9	222	166	43	13	154	33	35
	19A/F	2012–2014	1	6	4	2	0	0	6	0
23	23A	1945–2014	4	84	70	13	1	17	52	15
	23B	1941–2014	4	76	64	11	1	2	54	20
	23F	1940–2014	22	375	158	171	46	253	82	40
	23Fii	1983–2010	2	49	48	0	1	48	1	0
	23Hybrid	1997–2011	5	261	256	4	1	248	13	0
	23X	1996–2014	5	29	26	2	1	17	10	2
14	14i	1952–2013	8	58	19	33	6	35	17	6
	14wciY-fs	1967–2013	9	48	15	30	3	32	11	5
	14ii	2008–2010	1	88	88	0	0	88	0	0
	14Hybrid	1939–2011	4	64	59	2	3	63	1	0
15	15A	1939–2014	5	62	59	2	1	53	3	6
	15BC	1939–2013	4	20	15	2	3	6	7	7
	15F	1963	1	1	0	0	1	1	0	0
	15Hybrid	2001–2014	4	210	183	27	0	41	132	37
	15X	2008–2010	1	92	92	0	0	92	0	0
18	18A	1952–2010	2	4	3	0	1	4	0	0
	18B	1941, 2011	2	2	0	1	1	1	1	0
	18C	1939–2013	5	31	20	8	3	21	9	1
	18F	1961	1	1	0	0	1	1	0	0
	18Hybrid	1945–2010	4	33	32	0	1	33	0	0
33	33A	1937–2011	3	4	1	0	3	3	0	1
	33B	1962	1	1	0	0	1	1	0	0
	33C	2007–2010	1	11	11	0	0	11	0	0
	33D	1979	1	2	0	0	2	2	0	0
	33F	1999–2014	3	40	23	17	0	11	20	9
	33Hybrid	2008–2014	3	52	52	0	0	43	3	6
	33X	2008–2010	1	5	5	0	0	5	0	0
35	35A	1939–2010	2	11	10	0	1	11	0	0
	35B	1939–2014	5	108	97	9	2	42	61	5
	35C	1941–2009	2	8	6	0	2	8	0	0
	35F	1939–2014	5	73	63	9	1	18	32	23
	35Hybrid	2007–2010	2	28	28	0	0	25	3	0

*Carr, carriage; Dis, disease; Unk, unknown; Pre, recovered pre-PCV; Post, recovered post-PCV.

The serogroup/types selected for investigation in this study were 1, 3, 6, 7, 9, 11, 14, 15, 18, 19, 22, 23, 33 and 35, corresponding to 49 serotypes in total. Each genome sequence was screened against the relevant *cps* reference for the stated serotype and any genomes without full-length *cps* sequences were removed from further analyses. Genome sequences and associated metadata for all the pneumococci analysed in this study were deposited in the pneumococcal PubMLST database (http://pubmlst.org/spneumoniae/).

### Sequence-based serotyping.

With the exception of serotypes 3 and 37 (although serotype 37 was not investigated here), the polysaccharide capsule in pneumococcus is synthesized by the Wzy-dependent pathway. All Wzy-dependent serotypes have a *cps* locus located between *dexB* and *aliA*. Four conserved genes, *wzg*, *wzh*, *wzd* and *wze,* (also known as *cpsA–D*) at the start of the *cps* locus are responsible for the regulation of capsular synthesis while the remaining genes in the *cps* locus are serotype-specific and are involved in the production of the polysaccharide capsule. The serotype 3 *cps* locus shares a similar structure except that *wzg*, *wzh* and *wze* are truncated or frame-shifted (Yother, 2011).

Serotypes for each of the pneumococci included in the study were assigned based on the genome sequence, using a bespoke serotyping script called seqSerotyper.R (https://github.com/avantonder/seqSerotyper). The genome sequences were screened against published serotype reference sequences downloaded from GenBank, including the recently identified serotypes 6F, 6G and 11E (Table S3). Following the initial screen, where necessary, further differentiation of serotypes within serogroups was achieved using serotype-specific markers such as key amino acid residues and/or alleles unique to a particular serotype.

Serotypes 15B and 15C are difficult to accurately differentiate using phenotypic methods. A published method for separating serotypes 15B and 15C based upon counting the number of TA repeats in *wciZ* was attempted (van Selm *et al.*, 2003); however, this methodology failed to unambiguously resolve the two serotypes (Fig. S1) and thus any pneumococci with a 15B- or 15C-like sequence were reported here as 15B/C.

### Extraction and analysis of the *cps* locus sequences within each serogroup/type.

For serotypes 1, 3 and 14 the *cps* locus sequences were extracted from the corresponding pneumococcal genome sequences and aligned using muscle, excluding frameshifted genes and transposons (Edgar, 2004). To investigate the serogroups that contained multiple serotypes (6, 7, 9, 11, 15, 18, 19, 22, 23, 33 and 35), within each individual serogroup cd-hit was used to identify coding sequences (CDS) that were common to all serotypes in that serogroup at a sequence identity threshold of ≥70 %; (Li & Godzik, 2006; Table S4). A representative sequence from one of the serotype references within the serogroup was then BLASTed against the pneumococcal genome dataset to extract sequences for each of the shared CDS of that serogroup (Altschul *et al.*, 1997). These extracted *cps* sequences were aligned gene-by-gene using muscle before being concatenated to obtain a *cps* locus alignment for each serogroup.

Phylogenetic trees were reconstructed from the respective concatenated alignments using FastTreeMP and a general time-reversible nucleotide model (Price *et al.*, 2010). ClonalFrameML was implemented to reconstruct final phylogenetic trees adjusted for recombination (Didelot & Wilson, 2015). iTOL was used to annotate and colour the resulting diagrams (Letunic & Bork, 2011).

### Investigation of sequence diversity among serogroups/types.

To examine the level of *cps* locus sequence diversity amongst the 49 different serotypes included in this study, the nucleotide sequence of each reference serotype was BLASTed against the pneumococcal genomes in BIGSdb and the corresponding sequences were exported. Using mega6, each set of serotype-specific nucleotide sequences was aligned, converted to amino acid sequences and the variable amino acids were exported (Tamura *et al.*, 2013). The variable amino acid alignments were then exported to MS Excel and annotated: across the alignment amino acids that were identical to the reference serotype were coloured the same colour as the reference whilst alternative amino acids were shaded grey.

Putative novel (*cps* sequence with unknown origin) and hybrid (sequence combinations of two or more different serotypes) *cps* loci were identifiable in the phylogenetic trees on the basis of distinct clustering and in the variable amino acid alignments on the basis of significant divergence from the reference sequence. Variable regions of sequence were manually extracted and investigated (e.g. via blast searches to the study database, the rMLST database and/or GenBank) to determine the likely origin of the alternative sequence.

### Determination of pneumococcal genetic lineages.

Prokka was used to predict coding CDS in each genome and add annotation using a bespoke pneumococcal sequence database compiled for this study based upon the available gene annotation data from all pneumococcal genomes in GenBank (Seemann, 2014). The resulting annotation files in gff format were then input into Roary and clustered using a sequence identity threshold of 90 % (Page *et al.*, 2015). A core genome threshold of 100 % (i.e. those genes present in every genome were considered core genes) was implemented to select the core genes for each serogroup/type and these gene sequences were aligned. FastTreeMP and ClonalFrameML were used to reconstruct phylogenetic trees. Multilocus sequence type (MLST) data were automatically extracted from each genome using BIGSdb and STs were clustered into clonal complexes (CCs) using Phyloviz (Francisco *et al.*, 2012). Each core gene phylogenetic tree was annotated using iTOL.

## Results

### Serogroups/types with low to moderate diversity in the *cps* locus

#### Serotype 1.

**Fig. 1.**
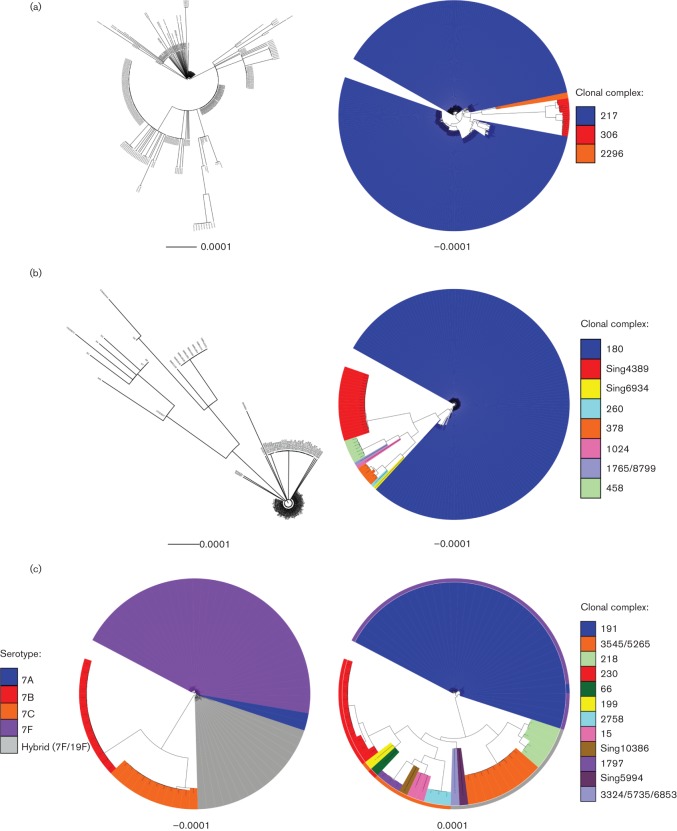
Phylogenetic trees illustrating *cps* locus sequence diversity and genetic relationships among pneumococci of serotypes 1 and 3 and serogroup 7. (a) Phylogenetic trees generated based on 15 *cps* locus genes amongst 348 serotype 1 pneumococcal genomes (left) and reconstructed using the concatenated sequence of 1000 full-length coding loci found in all 348 serotype 1 genomes (right). (b) Phylogenetic trees generated based on 4 *cps* locus genes amongst 205 serotype 3 pneumococcal genomes (left) and reconstructed using the concatenated sequence of 1160 full-length coding loci found in all 205 serotype 3 genomes (right). (c) Phylogenetic trees generated based on 10 *cps* locus genes amongst 76 serogroup 7 pneumococcal genomes (left) and reconstructed using the concatenated sequence of 1378 full-length coding loci found in all 76 serogroup 7 genomes (right). The outer ring of the tree on the right indicates the serotype by colour as detailed in the corresponding tree on the left.

Serotype 1 pneumococci (*n*=348) were recovered in 22 countries from 1943 to 2012 (Table 2). Minor serotype 1 *cps* locus variation was revealed among 12 of 14 genes, but patterns of variation were most commonly identified in *wzd* and *wze* (*cpsC* and *cpsD*, regulation) and *ugd*, *rmlA* and *rmlB* (sugar biosynthesis) (Fig. S2; Yother, 2011). Three genetic lineages were represented although most serotype 1 pneumococci in this study were within CC217, given that many serotype 1 genomes were from a recent African study (Cornick *et al.*, 2015; Fig. 1a).

#### Serotype 3.

Serotype 3 pneumococci (*n*=205) were collected in 10 countries from 1961 to 2014 (Table 2) and 81 % of serotype 3 pneumococci were in CC180, a widespread serotype 3 lineage (Fig. 1b; http://pubmlst.org/spneumoniae/). Little variation within the *cps* locus was revealed and what was noted was mainly due to the fact that the serotype 3 reference sequence differed at five amino acids in *ugd* (sugar biosynthesis), *wchE* and *galU* (glycosyltransferases) from the majority of the serotype 3 pneumococci (Fig. S3).

#### Serogroup 7.

Distinct clusters of serotype-specific *cps* loci were observed among serogroup 7 pneumococci (total *n*=76; Fig. 1c). Nearly half (*n*=36) were serotype 7F pneumococci of three major genetic lineages, CC191, CC218 and CC3545/5265, plus two singletons (single genotypes with no closely related variants) that dated from 1962 to 2014. The *cps* locus genes were generally highly conserved, although minor variation at amino acids in *wzd* (*cpsC*, regulation), *wcwA* (glycosyltransferase) and *rmlD* (sugar biosynthesis) was noted (Fig. S4). A total of 13 serotype 7B pneumococci were characterized and dated from 1952 to 2010 (Table 2), most of which were from Thailand (*n*=11; CC230; Fig. 1). Serotype 7C pneumococci (*n*=10; five different CCs) were recovered from 1939 to 2011 in four countries. The serotype 7B and 7C *cps* loci were highly conserved (Fig. S4). Serotype 7A (differentiated from 7F by a frameshift in *wcwD*) was rare: only one example (CC191) from 1937 was detected.

Additionally, a hybrid *cps* locus comprised of *wzg* (*cpsA*, regulation) sequence that matched both serotypes 7F and 19F was identified (Fig. S4). Carriage and disease-associated pneumococci with this hybrid locus (*n*=16) were isolated in four countries from 1952 to 2009 (Table 2). The pneumococci were of four genetic lineages, but mainly CC3545/5265 (*n*=9) from Thailand and CC218 (*n*=5), which is widespread (http://pubmlst.org/spneumoniae/).

#### Serogroup 9.

**Fig. 2.**
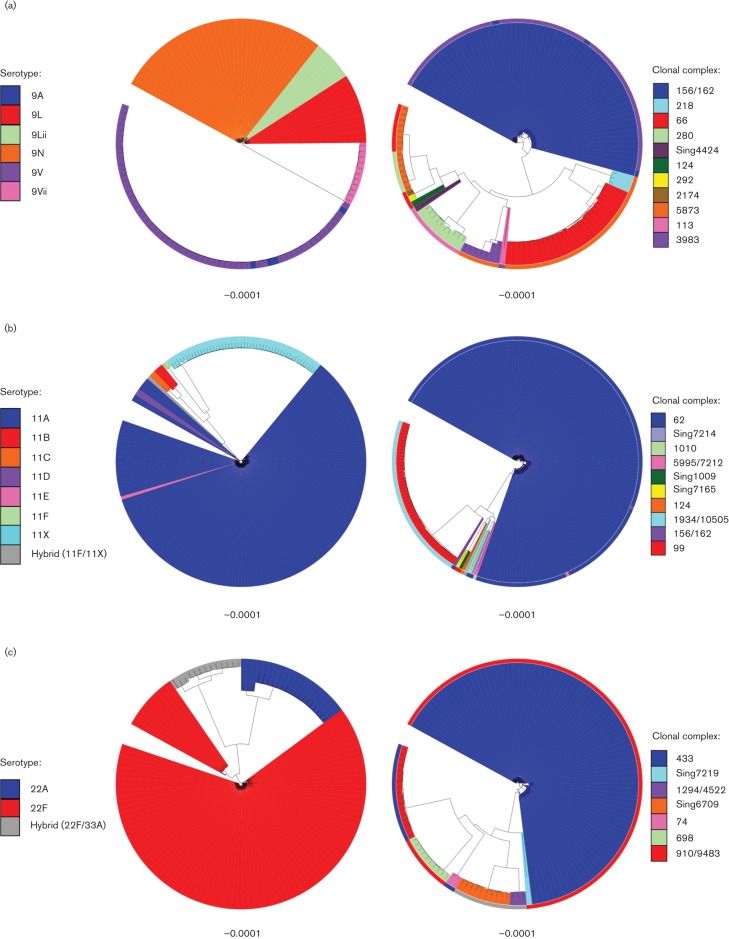
Phylogenetic trees illustrating *cps* locus sequence diversity and genetic relationships among pneumococci of serogroups 9, 11 and 22. (a) Phylogenetic trees generated based on 12 *cps* locus genes amongst 126 serogroup 9 pneumococcal genomes (left) and reconstructed using the concatenated sequence of 1259 full-length coding loci found in all 126 serogroup 9 genomes (right). (b) Phylogenetic trees generated based on 10 *cps* locus genes amongst 222 serogroup 11 pneumococcal genomes (left) and reconstructed using the concatenated sequence of 1204 full-length coding loci found in all 222 serogroup 11 genomes (right). (c) Phylogenetic trees generated based on 16 *cps* locus genes amongst 130 serogroup 22 pneumococcal genomes (left) and reconstructed using the concatenated sequence of 1390 full-length coding loci found in all 130 serogroup 22 genomes (right). The outer rings of the trees on the right indicate the serotype by colour as detailed in the corresponding trees on the left.

Distinct clusters of serotype-specific *cps* loci were observed among 126 serogroup 9 pneumococci (Fig. 2a). Serotype 9V pneumococci were the most common (*n*=69), dated from 1968 to 2014, and 83 % were members of CC156/162. One major serotype 9V *cps* locus sequence predominated, although eight minor Icelandic variants were identified with changes in *wzg, wze* and *wchO* (glycosyltransferase; Fig. S5). In addition, a variant serotype 9V *cps* locus (serotype 9Vii, Figs 2a and S5) with divergent alleles at 7 of 14 *cps* locus genes was revealed among 11 serotype 9V isolates (10 CC280 genomes from Thailand plus one singleton genome from the UK). Also of note was that the serotype 9V reference sequence differed at one amino acid in *wcjB* from all but one serotype 9V pneumococcus. The *cps* locus of serotype 9A is similar to that of 9V apart from a disrupted *wcjE* gene (Bentley *et al.*, 2006), but serotype 9A was uncommon in this study (*n*=3).

Serotype 9N pneumococci were also frequently identified (*n*=36), were recovered from 1938 to 2014 in five countries (Table 2), and were mainly of two genetic lineages (CC66, *n*=26; CC3983, *n*=7). The serotype 9N *cps* locus was generally highly conserved apart from isolated amino acid changes in *wze*, *wchA*, *wchO* and *wcjC* (the latter among Thailand pneumococci only; Fig. S5). Most serotype 9L pneumococci (*n*=18) were identified within the Thai dataset (*n*=15; CC5873) except for three historical isolates isolated from 1941 to 1968. No variable amino acids were observed among 10 of 13 serotype 9L *cps* locus genes although a distinct *wzg* allele was observed among seven Thai pneumococci (serotype 9Lii; Fig. S5).

#### Serogroup 11.

Serogroup 11 consists of six known serotypes but most were uncommon in this study. Serotypes 11C, 11D, 11E and 11F were represented by single pneumococci from 1957, 1986, 2012 and 1952, respectively, and only two serotype 11B pneumococci from 1940 and 2010 were identified (Table 2; Fig. 2b). Of the 222 serogroup 11 isolates 75 % were serotype 11A and 92 % of those were in CC62. Excluding unusual variants (see below), 7 of 16 genes within the *cps* locus of the serotype 11A pneumococci were identical to the serotype 11A reference, whilst five genes were differentiated from the reference by various single amino acid changes (Fig. S6). Most serotype 11A pneumococci consistently differed from the reference in genes *wchA* (glycosyltransferase), *wcwC* and *wcjE* (acetyl transferases).

A putative novel *cps* locus (11X) was identified among 49 pneumococci (CC99) from Thailand (Fig. 2b). The sequence was most similar to serotype 11F, although the nucleotide sequence similarity for each gene ranged from 85.5 to 99.6 % (Fig. S6). Notably, the novel *cps* locus had distinctive *wcwC* and *wcrL* (glycosyltransferase) genes (91.3 and 85.5 % similarity to serotype 11A) that did not match any other serotype sequences in GenBank. The novel locus also had intact *gct* (CDP-glycerol pathway) and *wcjE* genes, similar to serotype 11A. Interrogation of the PubMLST database revealed that pneumococci within CC99 were also identified in South Korea and China, but whether they possess the novel *cps* locus is unknown. Finally, a single hybrid combination of serotype 11A/11X was identified in one pneumococcus from Massachusetts (Figs 2b and S6).

#### Serogroup 22.

A total of 130 serogroup 22 pneumococci were analysed and serotypes 22F, 22A and a hybrid were identified (Fig. 2c). Serotype 22F pneumococci (*n*=98) were recovered from 1940 to 2014 in four countries (Table 2), and 89 % were in CC433. Overall, one major and one minor cluster of the serotype 22F *cps* locus were observed: all 19 genes differed from the reference by two or fewer amino acids (Figs 2c and S7). Apart from two historical pneumococci from 1939 (CC74), serotype 22A pneumococci (CC910/9483) were from Thailand (*n*=17). The serotype 22A *cps* locus was also conserved: 16 out of 19 genes were identical to the serotype 22A reference. Finally, a serotype 22F/33A hybrid *cps* locus was identified among 13 Thai pneumococci (Figs 2c and S7).

### Serogroups/types with high diversity in the *cps* locus

#### Serogroup 6.

We recently analysed a set of 974 serogroup 6 pneumococci and this work demonstrated a high level of *cps* locus diversity within the serogroup, and revealed that the vast majority of isolates that had previously been phenotypically typed as serotype 6B were in fact serotype 6E, which were approximately 7 % divergent at a nucleotide level in the *cps* locus (van Tonder *et al.*, 2015). Subsequent to that published work, other investigators reported that the serotype 6B and 6E polysaccharide capsule structures were identical (Burton *et al.*, 2016). Consequently, here we will refer to serotype 6E sequences as serotype 6Bii (serotype 6B class 2), as originally described by Mavroidi and colleagues, whilst serotype 6B class 1 sequences will be annotated as serotype 6B (Mavroidi *et al.*, 2004). In this study we reanalysed the original 974 genomes and added 208 new genomes, so that we could compare the serogroup 6 *cps* locus diversity to other high-prevalence serogroups, in particular serogroups 19 and 23.

**Fig. 3.**
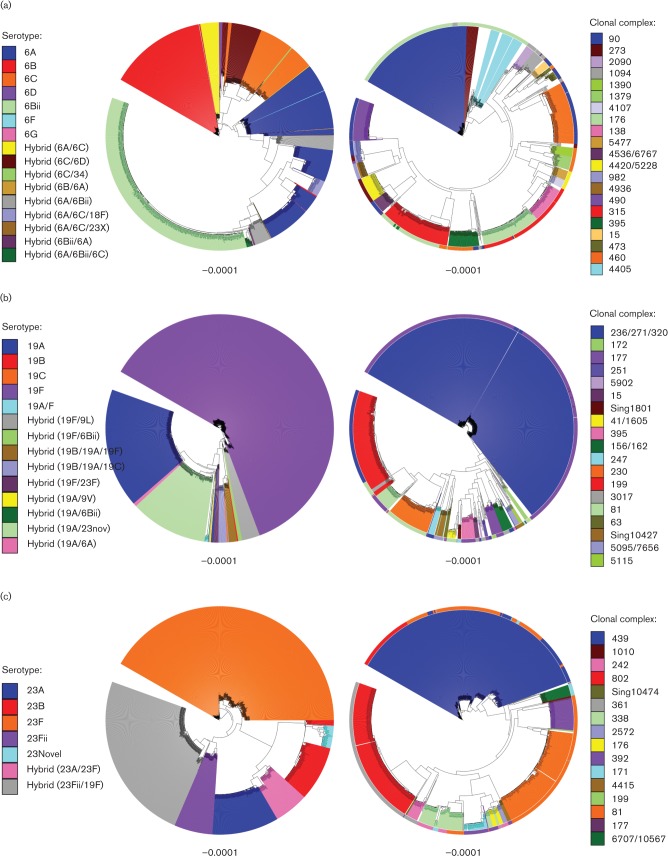
Phylogenetic trees illustrating *cps* locus sequence diversity and genetic relationships among pneumococci of serogroups 6, 19 and 23. (a) Phylogenetic trees generated based on 13 *cps* locus genes amongst 1182 serogroup 6 pneumococcal genomes (left) and reconstructed using the concatenated sequence of 470 full-length coding loci found in all 1182 serogroup 6 genomes (right). CCs with ten or more isolates were coloured as listed in the key. (b) Phylogenetic trees generated based on 13 *cps* locus genes amongst 1185 serogroup 19 pneumococcal genomes (left) and reconstructed using the concatenated sequence of 505 full-length coding loci found in all 1185 serogroup 19 genomes (right). CCs with five or more isolates were coloured as listed in the key. (c) Phylogenetic trees generated based on 17 *cps* locus genes amongst 874 serogroup 23 pneumococcal genomes (left) and reconstructed using the concatenated sequence of 628 full-length coding loci found in all 874 serogroup 23 genomes (right). The outer rings of the trees on the right indicate the serotype by colour as detailed in the corresponding trees on the left.

Analyses of the 1182 serogroup 6 *cps* locus sequences revealed seven serotypes and nine different hybrid serotypes (Fig. 3a). Serotype 6Bii pneumococci were the most prevalent (*n*=420), were collected from 1981 to 2014 in 16 countries (Table 3), and four major lineages were represented: CC90 (*n*=200); CC315 (*n*=104); CC4405 (*n*=54); and CC273 (*n*=21). The serotype 6Bii *cps* locus sequence was conserved (Fig. S8). Serotype 6A pneumococci (*n*=283) were also frequently recovered. They were members of 16 different CCs but the two major lineages were CC460 (*n*=109) and CC490 (*n*=74). There were five major clusters of serotype 6A *cps* loci and sequence variation occurred across the *cps* locus (Figs 3a and S8).

A total of 171 serotype 6B pneumococci collected from 1939 to 2014 in four countries were characterized and these were mainly of CC176 (*n*=103) or CC138 (*n*=62; Fig. 3a). The serotype 6B reference was not representative of the majority of serotype 6B pneumococci and was probably a capsular switch from serotype 6A to 6B. If the 6B reference sequence was excluded from the alignment then the *cps* locus was generally conserved among serotype 6B pneumococci (Fig. S8). A total of 106 serotype 6C pneumococci were revealed and were collected from 2001 to 2014 in three countries. Eight CCs were represented, but four were the most common: CC395 (*n*=44); CC1379 (*n*=24); CC1390 (*n*=160); and CC315 (*n*=13). Four distinct variants were observed in the *cps* loci sequence alignment and the variation occurred across the *cps* locus (Fig. S8).

Serotypes 6D and 6F were rare and serotypes 6G and 6H were not identified; however, hybrid serogroup 6 *cps* loci were common. Combinations of serogroup 6 pneumococci were most often observed, although the import of *cps* locus sequences from other serotypes was also revealed (Figs 3a and S8).

#### Serogroup 19.

1185 pneumococci within serogroup 19 were analysed, the majority of which were unequivocal serotypes 19F (*n*=745) and 19A (*n*=210; Table 3). Of the serotype 19F pneumococci 88 % (*n*=658) were recovered in nine countries from 1995 to 2014 and were members of CC236/271/320. Three other major serotype 19F lineages included CC81 (*n*=29), CC395 (*n*=24) and CC177 (*n*=13). Four different clusters of serotype 19F *cps* locus sequences were identified and amino acid variation occurred across the *cps* locus (Table 3; Figs 3b and S9).

Serotype 19A pneumococci were recovered in five countries from 1939 to 2014 (Table 3). Of these, 78 % (*n*=164) were in CC199, a widely-distributed lineage (http://pubmlst.org/spneumoniae/; Fig. 3b). Two other major lineages were CC236/271/320 (*n*=17) and CC247 (*n*=11), the latter being the earliest reported vaccine escape pneumococci (Brueggemann *et al.*, 2007). Alignments of the unequivocal serotype 19A *cps* loci to the 19A reference sequence demonstrated identical sequences between the 19A reference and three historical 19A pneumococci from 1939, 1952 and 1968. However, modern serotype 19A pneumococci differed considerably from the 19A reference across the *cps* locus (e.g. the nucleotide sequences of *rmlC*, *rmlB* and *rmlD* diverged from the reference sequence by up to 8, 15 and 1 %, respectively; Fig. S9). The *cps* loci of modern 19A pneumococci were highly similar. Serotype 19B and 19C pneumococci were rare (*n*=1 for each).

The remaining 222 serogroup 19 pneumococci revealed nine different types of hybrid *cps* loci (Figs 3b and S9). Hybrids containing 19A *cps* locus sequences (*n*=145) were most common and 93 % of these pneumococci were a combination of 19A/23X (novel serotype 23, see below) that resulted in serotype 23-like changes in *rmlC*, *rmlB* and *rmlD*. Three major CCs described 82 % of the 19A/23X hybrids: CC230 (*n*=76); CC199 (*n*=21); and CC41/1605 (*n*=14).

A total of 47 pneumococci contained a hybrid *cps* locus containing serotypes 19F/9L (*n*=31), 19F/23F (*n*=9) or 19F/6Bii (*n*=7) and six pneumococci revealed a combination 19A/19F *cps* locus that has been reported previously (Fig. S9; Pimenta *et al.*, 2009; Menezes *et al.*, 2013; Dunne *et al.*, 2015). A set of 30 pneumococci from Thailand possessed a *cps* locus that was a hybrid of either 19B/19A/19C (*n*=16) or 19B/19A/19F (*n*=14), each with divergent *wzg*, *wzh*, *wzd and wze* genes and evidence for multiple instances of recombination, while the rest of the *cps* locus of these pneumococci was similar to that of serotype 19B.

#### Serogroup 23.

A total of 874 pneumococci within serogroup 23 were characterized and 43 % (*n*=375) were unequivocal serotype 23F pneumococci recovered in 22 countries from 1940 to 2014 (Table 3). The major genetic lineages were CC439 (*n*=160), CC81 (*n*=142) and CC338 (*n*=20). There were two major serotype 23F *cps* locus clusters (Fig. 3c) and sequence variation occurred across the *cps* locus, but was mainly concentrated in *wzg*, *wchA*, *wchF*, *rmlA*, *rmlC*, *rmlB* and *rmlD* (Fig. S10). A variant serotype 23F (23Fii; *n*=49) *cps* locus was also revealed, which differed significantly at *wzg* and *wzh* from the serotype 23F reference. Pneumococci with this locus were recovered in Thailand and the USA from 1983 to 2010 and were mainly of CC171 (*n*=30). The serotype 23Fii variant was also detected as part of a hybrid *cps* locus with 23Fii and 19F sequences (Fig. 3c). A total of 217 pneumococci collected from 1997 to 2010 in four countries possessed this hybrid *cps* locus and two major lineages were revealed, CC802 (*n*=193) and CC242 (*n*=15). Serotype 19F-like sequence was identified in *wzg* and part of *wzh* but otherwise the *cps* locus was highly similar to serotype 23F.

A total of 84 pneumococci from four countries (1945–2014) typed as serotype 23A (Table 3). All were members of CC439 and the *cps* locus sequence was conserved (Figs 3c and S10). There were also 44 pneumococci from three countries (2008–2011) with a hybrid serotype 23A/23F *cps* locus and 80 % of these pneumococci were in CC338. The *cps* locus changes were mainly in *wzg*, although *rmlA* and *rmlD* also included some variable amino acids that matched the serotype 23B sequence.

There were 76 serotype 23B pneumococci collected from 1941 to 2014 (Table 3). All were members of CC439 except for one pneumococcus from 1941 and the *cps* loci were highly conserved (Figs 3c and S10). In addition, 29 pneumococci possessed some serotype 23B sequence, but the nucleotide sequences of eight genes, *wchX* through *rmlD*, differed by 14 % (*rmlB*) to 22 % (*rmlD*) from the serotype 23B reference (Fig. S10). Unusually, *rmlD* was inverted, which suggested a genome rearrangement (Fig. S10). A search of all available *cps* locus reference sequences failed to identify the possible source of this sequence. This putative novel serotype 23X was present in 29 pneumococci collected from 1996 to 2014 in five countries. 15 pneumococci were from a Thai lineage and the others were mainly in CC338 (*n*=7) and CC439 (*n*=5).

#### Serotype 14.

**Fig. 4.**
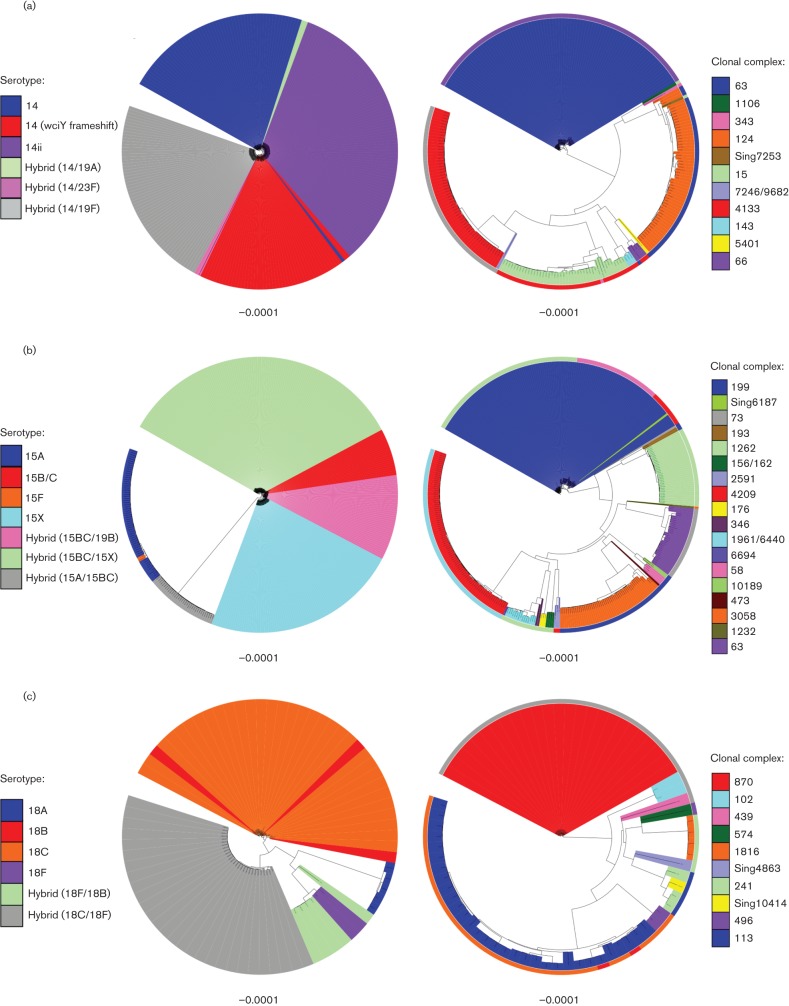
Phylogenetic trees illustrating *cps* locus sequence diversity and genetic relationships among pneumococci of serotype 14 and serogroups 15 and 18. (a) Phylogenetic trees generated based on 12 *cps* locus genes amongst 258 serotype 14 pneumococcal genomes (left) and reconstructed using the concatenated sequence of 911 full-length coding loci found in all 258 serotype 14 genomes (right). (b) Phylogenetic trees generated based on 13 *cps* locus genes amongst 385 serogroup 15 pneumococcal genomes (left) and reconstructed using the concatenated sequence of 893 full-length coding loci found in all 385 serogroup 15 genomes (right). (c) Phylogenetic trees generated based on 17 *cps* locus genes amongst 71 serogroup 18 pneumococcal genomes (left) and reconstructed using the concatenated sequence of 1302 full-length coding loci found in all 71 serogroup 18 genomes (right). The outer rings of the trees on the right indicate the serotype by colour as detailed in the corresponding trees on the left.

Four major *cps* locus clusters were clearly identified among a collection of 258 serotype 14 pneumococci collected from 1939 to 2013 (Fig. 4a). Of these, 58 pneumococci possessed *cps* loci that were identical or nearly identical to the serotype 14 reference sequence and all but two were in CC124. A total of 48 pneumococci, predominantly of CC15, possessed *cps* loci with a documented frameshift in *wciY* that is not believed to disrupt capsule production (Kolkman *et al.*, 1997; Ding *et al.*, 2009). Pneumococci with the frameshifted *wciY* also possessed a divergent *Lrp*, a large repetitive protein which varies in size across the entire serotype 14 collection. CC124 and CC15 are among the most common serotype 14 lineages circulating worldwide (http://pubmlst.org/spneumoniae/).

A set of 88 pneumococci from Thailand possessed a serotype 14 variant (14ii) *cps* locus with significant amino acid differences across seven *cps* locus genes and these pneumococci were all members of CC63, a widely-distributed lineage (http://pubmlst.org/spneumoniae/; Fig. 4). In addition, three different hybrid *cps* loci were identified, which were combinations of serotypes: 14/19F (*n*=60); 14/23F (*n*=2); and 14/19A (*n*=2; Figs 4 and S11). The serotype 14/19F hybrid *cps* locus was found in one South African pneumococcus from 1988 and in 59 recent pneumococci from Thailand (CC4133).

#### Serogroup 15.

Serotype clusters within serogroup 15 pneumococci (*n*=385) were clearly differentiated (Fig. 4b). A total of 62 serotype 15A pneumococci were recovered in five countries from 1939 to 2014 (Table 3) and 84 % were members of CC3058. The *cps* loci of serotype 15A pneumococci were similar to the 15A reference sequence: relatively few amino acids varied across the 16 *cps* locus genes and the variation was predominantly among 51 serotype 15A pneumococci from Thailand (Fig. S12). A single example of serotype 15F was collected in 1963.

The serotype 15B/C *cps* loci were diverse. Only 20 pneumococci possessed a *cps* locus similar to the serotype 15B and 15C references and 80 % were of CC199. Initially typed as serotype 15B/C, a putative novel *cps* locus (15X) was found in 92 pneumococci of CC4209 isolated in Thailand. Gene order was identical to that of the 15A and 15B/C *cps* loci; however, there were distinct differences in the amino acid sequences of *wzg*, *wzh* and *wzy* (polymerase), and other genes displayed evidence of recombination with serotype 15A (*wchA*, *gtp1* to *gtp3*; sugar biosynthesis); Fig. S12).

Finally, three large groups of hybrid *cps* loci were identified (Figs 4b and S12). Of these, 114 pneumococci were members of CC199 and possessed a hybrid *cps* locus combination of either 15BC/15X (*n*=74) or 15BC/19B (*n*=40). The 15BC/15X hybrid was also identified among pneumococci from six other genetic lineages. A hybrid combination of 15A/15BC was also revealed among 34 pneumococci in CC63.

#### Serogroup 18.

A total of 71 pneumococci of serogroup 18 were investigated and the predominant serotype was 18C (*n*=31; Table 3). Nearly all 18C pneumococci were in CC113, a widespread 18C lineage (http://pubmlst.org/spneumoniae/), and the serotype 18C *cps* locus was conserved (Figs 4c and S13). Serotypes 18A and 18F were uncommon (*n*=4 and *n*=1, respectively) and only found in Thailand. Two pneumococci had a serotype 18B *cps* locus, which differs from serotype 18C by a frameshift in *wciX* (acetyl transferase).

Two hybrid *cps* loci were revealed: 18C/18F (*n*=28) and 18F/18B (*n*=5). The 18C/18F hybrid pneumococci (CC870) were mainly from Thailand, although three were from Iceland (CC102) and the USA (CC439); these pneumococci possessed variable amino acids at 12 *cps* locus genes, including four genes involved in sugar biosynthesis (Fig. S13).

#### Serogroup 33.

**Fig. 5.**
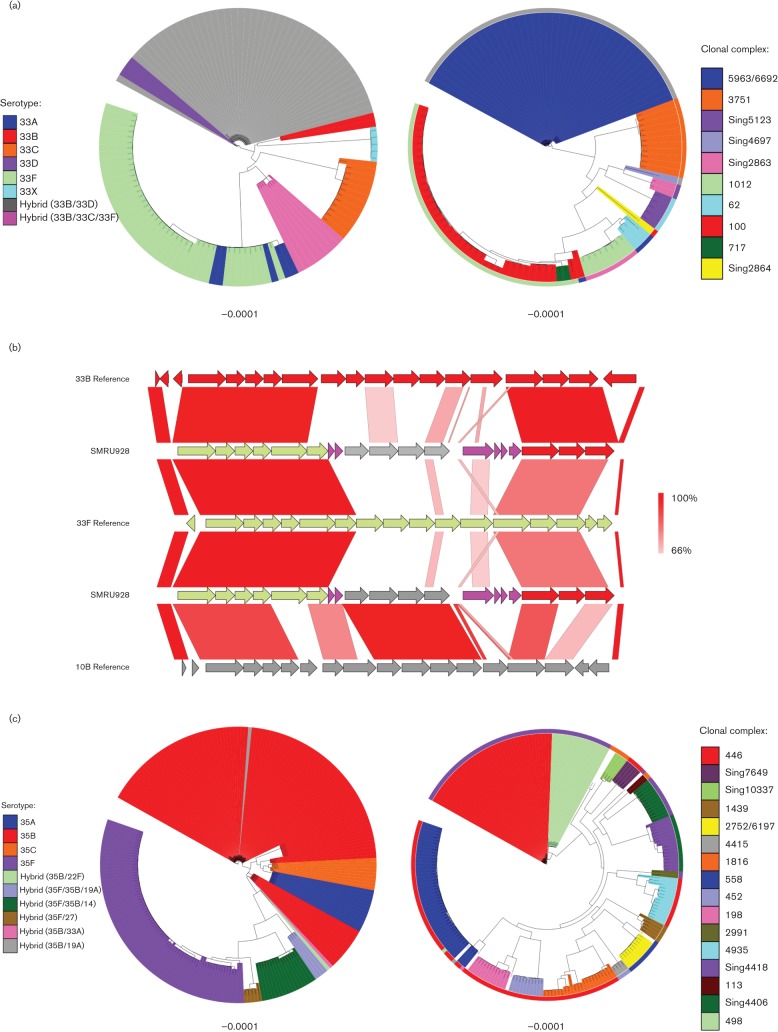
Phylogenetic trees and a comparative diagram illustrating *cps* locus sequence diversity and genetic relationships among pneumococci of serogroups 33 and 35. (a) Phylogenetic trees generated based on five *cps* locus genes amongst 115 serogroup 33 pneumococcal genomes (left) and reconstructed using the concatenated sequence of 1218 full-length coding loci found in all 115 serogroup 33 genomes (right). (b) Comparison of the putative serogroup 33X *cps* locus sequence to the serotype 33B, 33F and 10B reference sequences using Easyfig (Sullivan *et al.*, 2011). The results of pairwise blast nucleotide sequence comparisons are shown: darker red highlights greater sequence conservation between the pair of sequences. In order to generate pair-wise comparisons between all three reference sequences the putative serogroup 33X *cps* locus sequence was included twice. Coding loci in the putative serogroup 33X *cps* locus sequence were coloured according to the level of sequence similarity to coding loci from one of the reference sequences: light green for serotype 33F; grey for serotype 10B; red for serotype 33B; and disrupted loci are highlighted in pink. (c) Phylogenetic trees generated based on seven *cps* locus genes amongst 228 serogroup 35 pneumococcal genomes (left) and reconstructed using the concatenated sequence of 993 full-length coding loci found in all 228 serogroup 35 genomes (right). The outer rings of the trees on the right indicate the serotype by colour as detailed in the corresponding trees on the left.

A total of 115 pneumococcal genomes were analysed and this serogroup was unusual in that only five *cps* locus genes shared ≥70 % sequence identity across the serotypes within serogroup 33, even though the genes that comprised each *cps* locus were broadly similar (Table S4; Bentley *et al.*, 2006). Serotype clusters were clearly delineated within the tree except for serotypes 33A and 33F, which differ only by a frameshift in *wcjE* (Fig. 5a). Serotype 33F (*n*=40) pneumococci were frequently identified in three countries from 1999 to 2014, and all but two were in CC100. Three out of four serotype 33A pneumococci were historical (1937–1946, CC62 and CC100), two serotype 33D were from 1979, and one historical serotype 33B from 1962 was detected. A total of 11 serotype 33C pneumococci from Thailand were identified, all of CC3751. Sequence alignments of serotype 33F, 33A and 33C *cps* loci revealed conserved sequences when compared to each respective reference sequence (Fig. S14).

With the exception of two pneumococci from 1979, all capsular sequences extracted from pneumococci initially typed as serotype 33D revealed evidence of recombination with serotype 33B, which probably altered six or more *cps* locus genes (*n*=44; Figs 5a and S14). All but one of these hybrid pneumococci were from Thailand. A second hybrid *cps* locus comprised of sequence from serotypes 33B/33C/33F was revealed in eight pneumococci of CC62 from the UK and Iceland. Finally, five Thai pneumococci possessed a novel 33X *cps* locus, which consisted of a complex arrangement of nucleotide sequences from serotypes 33B, 33F and 10A/B *cps* locus genes (Fig. 5b).

#### Serogroup 35.

A total of 228 pneumococci within serogroup 35 were analysed and four known serotypes were identified, in addition to several hybrid *cps* loci (Fig. 5c). Two distinct clusters of serotype 35B (*n*=108) pneumococci were identified. The pneumococci were recovered in five countries from 1939 to 2014 and five major and three minor CCs were recognized (Table 3; Fig. 5c). A total of 81 serotype 35B *cps* locus sequences were conserved relative to the 35B reference, with the exception of some scattered variation (Fig. S15). The remaining 35B *cps* loci possessed many variable amino acids relative to the reference sequence and these were of two major groups from Iceland (*n*=10) and Thailand (*n*=17). Of these, 22 were members of CC1816 and the rest were a unique Thai lineage.

Single clusters of 35F (*n*=73), 35A (*n*=11) and 35C (*n*=8) were also revealed (Fig. 5c). Serotype 35F pneumococci were identified in five countries from 1939 to 2014 and three major lineages were detected: CC446 (*n*=41; Iceland, UK); CC498 (*n*=17; USA); and CC4406 (*n*=12; Thailand). Serotype 35A and 35C sequences were mainly identified among Thai pneumococci, apart from historical isolates from 1939 (35A) and 1941–1943 (35C), and these pneumococci were members of one or one predominant CC, respectively (Fig. 5c). The *cps* loci of each serotype were generally highly conserved (Fig. S15).

Finally, three hybrid *cps* loci of different serotype combinations were identified in Thailand (35F/14/35B, *n*=16; 35F/27, *n*=5; and 35F/19A/35B, *n*=4). Three additional hybrid combinations were identified in the USA (35B/22F, 35B/33A and 35B/19A, *n*=1 each; Fig. S15).

## Discussion

Genomics has revolutionized microbiological research: thousands of bacterial genomes are now in the public domain and the deluge of new data continues. Maximizing the utility of the vast quantities of genome sequence data from well-sampled studies is advancing our understanding of fundamental aspects of microbiology, epidemiology, population biology and microbial evolution. Given the significance of the polysaccharide capsule to pneumococcal biology it is essential to understand the variation (or lack thereof) that occurs within and between different serotypes. In this study we assessed the level of sequence diversity at the *cps* locus for each serogroup/type and identified hybrid or putatively novel serotypes within each serogroup.

We revealed serogroups/types that were largely conserved and others that were rather diverse. Diversity among high-prevalence serogroups like 6, 19 and 23 was expected, but variation among serotypes like 14 was more surprising. To what extent the variation is biologically informative remains to be deciphered, but is the essential next step in gaining a deeper understanding of pneumococcal capsular biology. Furthermore, unusual variation at the level of the *cps* locus and the circulating genetic lineages was frequently noted among the pneumococci from Thailand. Whether this reflects an unusual collection of pneumococci or whether it is simply that collections of pneumococci from resource-poor countries differ significantly from other geographical regions remains to be determined. Finally, the selection of reference strains of pneumococci must be made carefully, since some *cps* locus reference strains (Bentley *et al.*, 2006) are not particularly good representatives of modern circulating pneumococci. Sequencing the 90 *cps* loci some years ago was a big step forward at a time when sequencing costs were still very high and limited *cps* loci data were available, but some revisions to the selections of reference pneumococci would now be useful.

Investigating serotype-specific sequence variation is crucial to the design of sequence-based serotyping methodology. Sequence diversity within regions targeted by PCR or microarray could lead to erroneous test results and subsequently, a misinformed view about the circulating serotypes. Since many countries are now using PCVs, pre- and post-PCV introduction surveillance is essential and correctly identifying the serotypes of circulating pneumococci is central to surveillance.

Understanding *cps* locus diversity is also fundamental to assessing PCV effectiveness. Longer-term effectiveness depends (at least in part) on the PCV-specific polysaccharides representing the circulating pneumococci in order to elicit an appropriate vaccine-mediated antigenic response, and on a lack of subsequent capsular polysaccharide changes that could lead to a reduction in the protective capacity and a potential for vaccine failure. The emergence of *cps* locus variants appears to be part of the normal biological processes of pneumococci and is unlikely to be driven primarily by the introduction of vaccines (Wyres *et al.*, 2012; van Tonder *et al.*, 2015), but the selective pressure of vaccine use may result in a population-level increase in the variants that do emerge and this could affect overall vaccine effectiveness.

It is known that small changes to genes within the *cps* locus can lead to changes in the polysaccharide structure, e.g. single amino acid changes in the *wciP* of serogroup 6 lead to the different serotype 6A and 6B polysaccharides (Mavroidi *et al.*, 2004), but equally the large genetic differences between serotypes 6B and 6Bii (6E) apparently do not confer a difference in the polysaccharide (Burton *et al.*, 2016). Future work should investigate precisely which sequence-based changes revealed in this work result in a biologically relevant change, particularly among high-prevalence serotypes and those included in current PCVs. Equally, any future higher-valency formulations of PCVs that utilize the polysaccharide capsule to elicit a protective immune response will undoubtedly benefit from an assessment of *cps* locus diversity for each vaccine serotype, to assess the pre-PCV introduction capsular diversity and the potential for reduced vaccine effectiveness.

We also revealed many hybrid *cps* loci and putatively novel serotypes. The hybrid *cps* loci are unsurprising, given the genome-wide level of recombination that occurs among pneumococci, including at the *cps* locus. Confirmation of putatively novel serotypes requires a biochemical assessment of the polysaccharide capsule to confirm novelty, which is not a trivial task. One might take the view that rare serotype variants are not important enough to warrant complicated and costly biochemical exploration; however, putatively novel serotypes that are common in a population arguably are worth further assessment.

Genomic analyses of a very large and diverse dataset have allowed for a fine-scale investigation of pneumococcal *cps* diversity. This work provides a major advance in our understanding of pneumococcal serotypes and sequence diversity at the *cps* locus.

## Supplementary Data

21926 Supplementary File 1
