# Supplementary material for: Putatively novel serotypes and the potential for reduced vaccine effectiveness: capsular locus diversity revealed among 5405 pneumococcal genomes
Source: Microb Genom. 2016 Oct 21;2(10):e000090. doi: 10.1099/mgen.0.000090 (PMC5266551; doi:10.1099/mgen.0.000090)
Supplement: Supplementary File 1 [file mgen-02-90-s001.pdf]

Serogroup 15

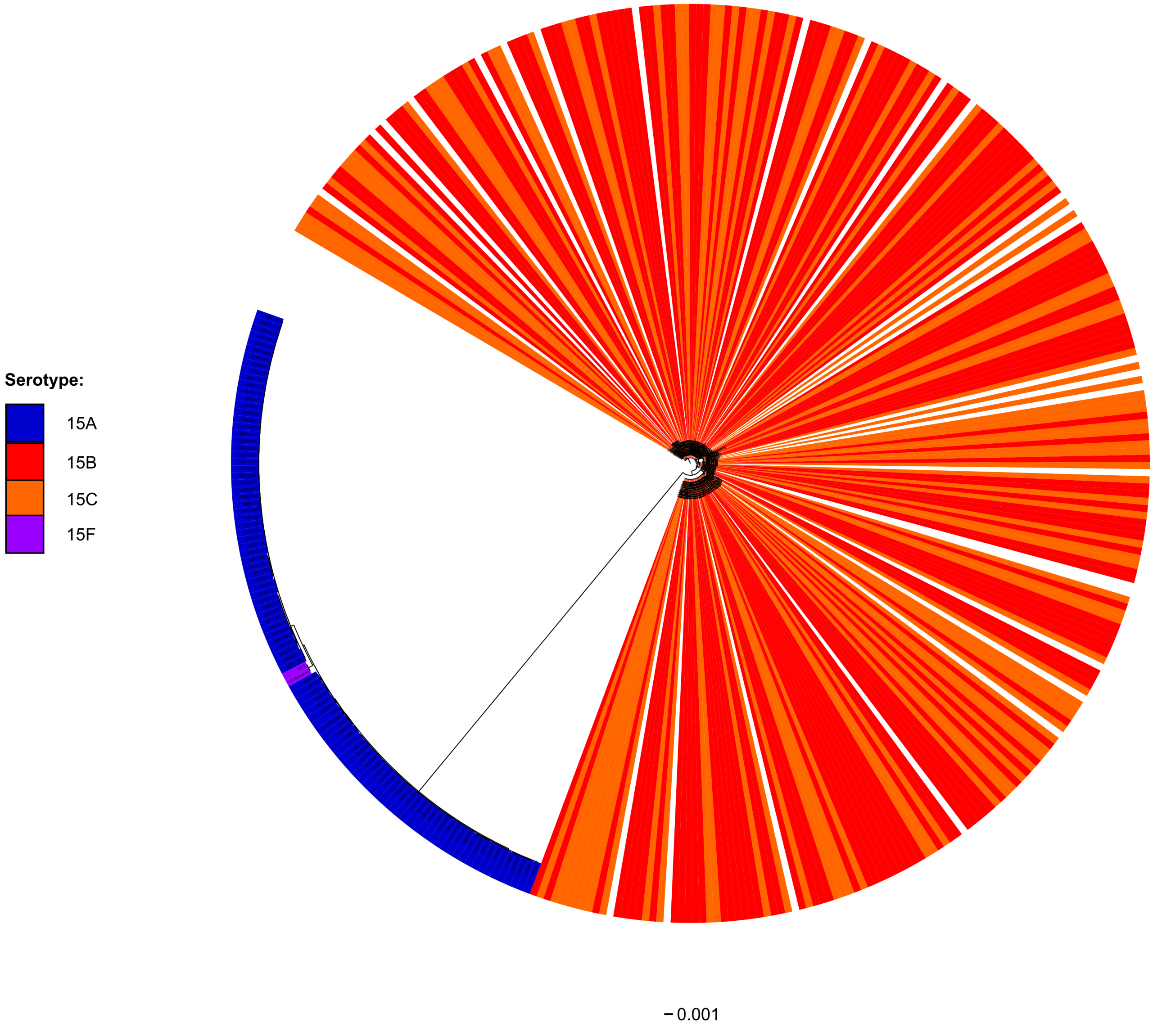

rmlst ID	PubMLST id	ENA	Name	ST	CC	Orig_sero	Orig_sero_method	Seq_sero
N/A	35812	N/A	1/2	615	2296	1	Quellung	1
N/A	36289	N/A	1/4	5316	306	1	Quellung	1
N/A	35813	N/A	1/5	5316	306	1	Quellung	1
N/A	15482	N/A	1/6	5316	306	1	Quellung	1
N/A	35814	N/A	1/7	250	306	1	Quellung	1
18140	32737	ERR018044	ERR018044	217	217	1	PCR/Sequence based	1
32767	32900	ERR023321	ERR023321	217	217	1	PCR/Sequence based	1
115753	33730	ERR023322	ERR023322	217	217	1	PCR/Sequence based	1
33755	32944	ERR023325	ERR023325	217	217	1	PCR/Sequence based	1
33756	32945	ERR023326	ERR023326	SLV217	217	1	PCR/Sequence based	1
31870	32837	ERR023328	ERR023328	217	217	1	PCR/Sequence based	1
31871	32838	ERR023329	ERR023329	217	217	1	PCR/Sequence based	1
32768	32901	ERR023330	ERR023330	217	217	1	PCR/Sequence based	1
44801	33048	ERR023331	ERR023331	217	217	1	PCR/Sequence based	1
39992	32985	ERR023332	ERR023332	217	217	1	PCR/Sequence based	1
39993	32986	ERR023334	ERR023334	217	217	1	PCR/Sequence based	1
33757	32946	ERR023335	ERR023335	217	217	1	PCR/Sequence based	1
39994	32987	ERR023336	ERR023336	217	217	1	PCR/Sequence based	1
32769	32902	ERR023337	ERR023337	217	217	1	PCR/Sequence based	1
55120	33062	ERR023338	ERR023338	SLV217	217	1	PCR/Sequence based	1
115754	33731	ERR023339	ERR023339	217	217	1	PCR/Sequence based	1
129690	34334	ERR023340	ERR023340	217	217	1	PCR/Sequence based	1
32770	32903	ERR023342	ERR023342	SLV217	217	1	PCR/Sequence based	1
115755	33732	ERR023343	ERR023343	SLV217	217	1	PCR/Sequence based	1
39995	32988	ERR023344	ERR023344	217	217	1	PCR/Sequence based	1
39996	32989	ERR023345	ERR023345	217	217	1	PCR/Sequence based	1
44137	33045	ERR023351	ERR023351	217	217	1	PCR/Sequence based	1
39998	32990	ERR023354	ERR023354	217	217	1	PCR/Sequence based	1
33759	32947	ERR023357	ERR023357	217	217	1	PCR/Sequence based	1
129700	34341	ERR023358	ERR023358	217	217	1	PCR/Sequence based	1
33760	32948	ERR023376	ERR023376	217	217	1	PCR/Sequence based	1
129701	34342	ERR023381	ERR023381	217	217	1	PCR/Sequence based	1
115759	33733	ERR023393	ERR023393	303	217	1	PCR/Sequence based	1
33761	32949	ERR023394	ERR023394	217	217	1	PCR/Sequence based	1
44802	33049	ERR023569	ERR023569	217	217	1	PCR/Sequence based	1
33766	32950	ERR023570	ERR023570	217	217	1	PCR/Sequence based	1
33767	32951	ERR023572	ERR023572	217	217	1	PCR/Sequence based	1
44803	33050	ERR023573	ERR023573	217	217	1	PCR/Sequence based	1
115765	33734	ERR023575	ERR023575	217	217	1	PCR/Sequence based	1
44804	33051	ERR023577	ERR023577	217	217	1	PCR/Sequence based	1
40006	32991	ERR023578	ERR023578	612	217	1	PCR/Sequence based	1
115766	33735	ERR023579	ERR023579	217	217	1	PCR/Sequence based	1
129702	34343	ERR023585	ERR023585	217	217	1	PCR/Sequence based	1
32783	32904	ERR023589	ERR023589	217	217	1	PCR/Sequence based	1
129689	34333	ERR023592	ERR023592	217	217	1	PCR/Sequence based	1
129211	34318	ERR023595	ERR023595	217	217	1	PCR/Sequence based	1
129696	34338	ERR023601	ERR023601	612	217	1	PCR/Sequence based	1
40009	32992	ERR023605	ERR023605	217	217	1	PCR/Sequence based	1
44805	33052	ERR023608	ERR023608	217	217	1	PCR/Sequence based	1
32789	32905	ERR023610	ERR023610	217	217	1	PCR/Sequence based	1
40074	32996	ERR028396	ERR028396	217	217	1	PCR/Sequence based	1
40075	32997	ERR028400	ERR028400	217	217	1	PCR/Sequence based	1
31206	32795	ERR028406	ERR028406	217	217	1	PCR/Sequence based	1
18335	32764	ERR028411	ERR028411	217	217	1	PCR/Sequence based	1
40081	32998	ERR028423	ERR028423	217	217	1	PCR/Sequence based	1
40084	32999	ERR028438	ERR028438	217	217	1	PCR/Sequence based	1
32863	32906	ERR028439	ERR028439	217	217	1	PCR/Sequence based	1
33818	32952	ERR028440	ERR028440	SLV217	217	1	PCR/Sequence based	1

40085	33000	ERR028444	ERR028444	217	217	1	PCR/Sequence based	1
40086	33001	ERR028447	ERR028447	SLV217	217	1	PCR/Sequence based	1
18154	32739	ERR028451	ERR028451	SLV217	217	1	PCR/Sequence based	1
55914	33071	ERR028452	ERR028452	217	217	1	PCR/Sequence based	1
32867	32907	ERR028458	ERR028458	217	217	1	PCR/Sequence based	1
31218	32796	ERR033114	ERR033114	217	217	1	PCR/Sequence based	1
18081	32734	ERR033120	ERR033120	217	217	1	PCR/Sequence based	1
18334	32763	ERR033122	ERR033122	217	217	1	PCR/Sequence based	1
18319	32760	ERR033123	ERR033123	217	217	1	PCR/Sequence based	1
18326	32761	ERR033141	ERR033141	217	217	1	PCR/Sequence based	1
31220	32797	ERR033146	ERR033146	217	217	1	PCR/Sequence based	1
18261	32748	ERR033151	ERR033151	217	217	1	PCR/Sequence based	1
44144	33046	ERR042138	ERR042138	3081	217	1	PCR/Sequence based	1
32903	32908	ERR042139	ERR042139	3081	217	1	PCR/Sequence based	1
31948	32843	ERR042140	ERR042140	SLV2084	217	1	PCR/Sequence based	1
32904	32909	ERR042141	ERR042141	3081	217	1	PCR/Sequence based	1
32905	32910	ERR042142	ERR042142	SLV217	217	1	PCR/Sequence based	1
32906	32911	ERR042143	ERR042143	217	217	1	PCR/Sequence based	1
31949	32844	ERR042144	ERR042144	3081	217	1	PCR/Sequence based	1
40120	33002	ERR042145	ERR042145	3081	217	1	PCR/Sequence based	1
31950	32845	ERR042146	ERR042146	3081	217	1	PCR/Sequence based	1
31951	32846	ERR042147	ERR042147	3081	217	1	PCR/Sequence based	1
31952	32847	ERR042148	ERR042148	3081	217	1	PCR/Sequence based	1
31953	32848	ERR042149	ERR042149	3081	217	1	PCR/Sequence based	1
31954	32849	ERR042150	ERR042150	303	217	1	PCR/Sequence based	1
33846	32953	ERR042151	ERR042151	303	217	1	PCR/Sequence based	1
31955	32850	ERR042152	ERR042152	303	217	1	PCR/Sequence based	1
32907	32912	ERR042153	ERR042153	303	217	1	PCR/Sequence based	1
31956	32851	ERR042154	ERR042154	303	217	1	PCR/Sequence based	1
31957	32852	ERR042155	ERR042155	303	217	1	PCR/Sequence based	1
40121	33003	ERR042156	ERR042156	303	217	1	PCR/Sequence based	1
40122	33004	ERR042157	ERR042157	303	217	1	PCR/Sequence based	1
31958	32853	ERR042158	ERR042158	3081	217	1	PCR/Sequence based	1
31959	32854	ERR042159	ERR042159	3081	217	1	PCR/Sequence based	1
32908	32913	ERR042160	ERR042160	3081	217	1	PCR/Sequence based	1
32909	32914	ERR042161	ERR042161	3081	217	1	PCR/Sequence based	1
31960	32855	ERR042162	ERR042162	217	217	1	PCR/Sequence based	1
33847	32954	ERR042163	ERR042163	217	217	1	PCR/Sequence based	1
33848	32955	ERR042164	ERR042164	3081	217	1	PCR/Sequence based	1
40123	33005	ERR042165	ERR042165	3081	217	1	PCR/Sequence based	1
31961	32856	ERR042166	ERR042166	3081	217	1	PCR/Sequence based	1
31962	32857	ERR042167	ERR042167	618	217	1	PCR/Sequence based	1
31221	32798	ERR042168	ERR042168	3081	217	1	PCR/Sequence based	1
40124	33006	ERR042169	ERR042169	3081	217	1	PCR/Sequence based	1
40125	33007	ERR042170	ERR042170	3081	217	1	PCR/Sequence based	1
18446	32783	ERR042173	ERR042173	3081	217	1	PCR/Sequence based	1
18350	32767	ERR042174	ERR042174	3081	217	1	PCR/Sequence based	1
18462	32784	ERR042175	ERR042175	3081	217	1	PCR/Sequence based	1
18362	32770	ERR042176	ERR042176	3081	217	1	PCR/Sequence based	1
18464	32785	ERR042177	ERR042177	618	217	1	PCR/Sequence based	1
18371	32772	ERR042178	ERR042178	618	217	1	PCR/Sequence based	1
18421	32777	ERR042179	ERR042179	3081	217	1	PCR/Sequence based	1
18346	32766	ERR042180	ERR042180	3081	217	1	PCR/Sequence based	1
31964	32858	ERR042181	ERR042181	3081	217	1	PCR/Sequence based	1
18444	32782	ERR042182	ERR042182	3081	217	1	PCR/Sequence based	1
18442	32781	ERR042183	ERR042183	217	217	1	PCR/Sequence based	1
32910	32915	ERR042184	ERR042184	618	217	1	PCR/Sequence based	1
31965	32859	ERR042185	ERR042185	618	217	1	PCR/Sequence based	1
31966	32860	ERR042186	ERR042186	217	217	1	PCR/Sequence based	1

32911	32916	ERR042187	ERR042187	618	217	1	PCR/Sequence based	1
32912	32917	ERR042188	ERR042188	3081	217	1	PCR/Sequence based	1
31967	32861	ERR042189	ERR042189	3081	217	1	PCR/Sequence based	1
18316	32759	ERR042190	ERR042190	3081	217	1	PCR/Sequence based	1
33849	32956	ERR042191	ERR042191	3081	217	1	PCR/Sequence based	1
40126	33008	ERR042192	ERR042192	3081	217	1	PCR/Sequence based	1
32913	32918	ERR042193	ERR042193	303	217	1	PCR/Sequence based	1
33850	32957	ERR042195	ERR042195	303	217	1	PCR/Sequence based	1
18398	32776	ERR042197	ERR042197	3081	217	1	PCR/Sequence based	1
31223	32799	ERR042198	ERR042198	3081	217	1	PCR/Sequence based	1
18268	32751	ERR042199	ERR042199	3081	217	1	PCR/Sequence based	1
18227	32743	ERR042200	ERR042200	3081	217	1	PCR/Sequence based	1
18375	32773	ERR042201	ERR042201	3081	217	1	PCR/Sequence based	1
18188	32740	ERR042203	ERR042203	217	217	1	PCR/Sequence based	1
40128	33009	ERR042204	ERR042204	3081	217	1	PCR/Sequence based	1
18440	32780	ERR042206	ERR042206	3081	217	1	PCR/Sequence based	1
31968	32862	ERR042207	ERR042207	3081	217	1	PCR/Sequence based	1
115841	33779	ERR045783	ERR045783	217	217	1	PCR/Sequence based	1
33853	32960	ERR045786	ERR045786	8158	217	1	PCR/Sequence based	1
32915	32920	ERR045787	ERR045787	217	217	1	PCR/Sequence based	1
129697	34339	ERR045789	ERR045789	217	217	1	PCR/Sequence based	1
129743	34383	ERR045790	ERR045790	217	217	1	PCR/Sequence based	1
129687	34331	ERR045791	ERR045791	217	217	1	PCR/Sequence based	1
115842	33780	ERR045792	ERR045792	217	217	1	PCR/Sequence based	1
115850	33787	ERR046710	ERR046710	2206	217	1	PCR/Sequence based	1
40142	33023	ERR046711	ERR046711	2206	217	1	PCR/Sequence based	1
40143	33024	ERR046712	ERR046712	303	217	1	PCR/Sequence based	1
40144	33025	ERR046713	ERR046713	303	217	1	PCR/Sequence based	1
55922	33074	ERR046714	ERR046714	303	217	1	PCR/Sequence based	1
33862	32969	ERR046715	ERR046715	217	217	1	PCR/Sequence based	1
33863	32970	ERR046716	ERR046716	217	217	1	PCR/Sequence based	1
33864	32971	ERR046717	ERR046717	217	217	1	PCR/Sequence based	1
33865	32972	ERR046718	ERR046718	303	217	1	PCR/Sequence based	1
40145	33026	ERR046719	ERR046719	217	217	1	PCR/Sequence based	1
55127	33065	ERR046720	ERR046720	303	217	1	PCR/Sequence based	1
115851	33788	ERR046721	ERR046721	217	217	1	PCR/Sequence based	1
40146	33027	ERR046722	ERR046722	303	217	1	PCR/Sequence based	1
40147	33028	ERR046723	ERR046723	217	217	1	PCR/Sequence based	1
55128	33066	ERR046724	ERR046724	303	217	1	PCR/Sequence based	1
55923	33075	ERR046725	ERR046725	217	217	1	PCR/Sequence based	1
40148	33029	ERR046726	ERR046726	303	217	1	PCR/Sequence based	1
44829	33059	ERR046727	ERR046727	303	217	1	PCR/Sequence based	1
40149	33030	ERR046728	ERR046728	217	217	1	PCR/Sequence based	1
129744	34384	ERR059358	ERR059358	217	217	1	PCR/Sequence based	1
129752	34392	ERR059359	ERR059359	217	217	1	PCR/Sequence based	1
129754	34394	ERR059360	ERR059360	217	217	1	PCR/Sequence based	1
129741	34381	ERR059361	ERR059361	303	217	1	PCR/Sequence based	1
129753	34393	ERR059362	ERR059362	217	217	1	PCR/Sequence based	1
129707	34348	ERR059363	ERR059363	2839	217	1	PCR/Sequence based	1
129731	34371	ERR059364	ERR059364	217	217	1	PCR/Sequence based	1
129751	34391	ERR059365	ERR059365	217	217	1	PCR/Sequence based	1
129691	34335	ERR059366	ERR059366	303	217	1	PCR/Sequence based	1
129699	34340	ERR059367	ERR059367	217	217	1	PCR/Sequence based	1
129722	34362	ERR059368	ERR059368	217	217	1	PCR/Sequence based	1
129705	34346	ERR059369	ERR059369	217	217	1	PCR/Sequence based	1
129749	34389	ERR059370	ERR059370	303	217	1	PCR/Sequence based	1
129750	34390	ERR059371	ERR059371	217	217	1	PCR/Sequence based	1
129738	34378	ERR059372	ERR059372	303	217	1	PCR/Sequence based	1
129740	34380	ERR059373	ERR059373	217	217	1	PCR/Sequence based	1

129724	34364	ERR059375	ERR059375	217	217	1	PCR/Sequence based	1
129695	34337	ERR059376	ERR059376	217	217	1	PCR/Sequence based	1
129714	34354	ERR128823	ERR128823	303	217	1	PCR/Sequence based	1
129704	34345	ERR128824	ERR128824	303	217	1	PCR/Sequence based	1
129709	34350	ERR128825	ERR128825	2084	217	1	PCR/Sequence based	1
129725	34365	ERR128826	ERR128826	303	217	1	PCR/Sequence based	1
129726	34366	ERR128827	ERR128827	303	217	1	PCR/Sequence based	1
129713	34353	ERR128828	ERR128828	217	217	1	PCR/Sequence based	1
129728	34368	ERR128829	ERR128829	303	217	1	PCR/Sequence based	1
129716	34356	ERR128830	ERR128830	303	217	1	PCR/Sequence based	1
129748	34388	ERR128831	ERR128831	303	217	1	PCR/Sequence based	1
129746	34386	ERR128832	ERR128832	217	217	1	PCR/Sequence based	1
129737	34377	ERR128833	ERR128833	303	217	1	PCR/Sequence based	1
129739	34379	ERR128834	ERR128834	SLV303	217	1	PCR/Sequence based	1
129708	34349	ERR128835	ERR128835	303	217	1	PCR/Sequence based	1
129734	34374	ERR128836	ERR128836	303	217	1	PCR/Sequence based	1
129715	34355	ERR128837	ERR128837	2084	217	1	PCR/Sequence based	1
129712	34352	ERR128838	ERR128838	303	217	1	PCR/Sequence based	1
129711	34351	ERR128839	ERR128839	4755	217	1	PCR/Sequence based	1
129735	34375	ERR128840	ERR128840	303	217	1	PCR/Sequence based	1
129742	34382	ERR128841	ERR128841	4755	217	1	PCR/Sequence based	1
129745	34385	ERR128842	ERR128842	303	217	1	PCR/Sequence based	1
129721	34361	ERR128843	ERR128843	303	217	1	PCR/Sequence based	1
129212	34319	ERR128844	ERR128844	217	217	1	PCR/Sequence based	1
129727	34367	ERR128845	ERR128845	303	217	1	PCR/Sequence based	1
129747	34387	ERR128846	ERR128846	303	217	1	PCR/Sequence based	1
129706	34347	ERR128847	ERR128847	303	217	1	PCR/Sequence based	1
129719	34359	ERR128848	ERR128848	303	217	1	PCR/Sequence based	1
129718	34358	ERR128849	ERR128849	303	217	1	PCR/Sequence based	1
129730	34370	ERR128850	ERR128850	303	217	1	PCR/Sequence based	1
129732	34372	ERR128851	ERR128851	303	217	1	PCR/Sequence based	1
129729	34369	ERR128852	ERR128852	303	217	1	PCR/Sequence based	1
129723	34363	ERR128853	ERR128853	303	217	1	PCR/Sequence based	1
129717	34357	ERR128854	ERR128854	303	217	1	PCR/Sequence based	1
129733	34373	ERR128855	ERR128855	303	217	1	PCR/Sequence based	1
129736	34376	ERR128856	ERR128856	303	217	1	PCR/Sequence based	1
129720	34360	ERR128857	ERR128857	303	217	1	PCR/Sequence based	1
115977	33795	ERR222951	ERR222951	217	217	1	PCR/Sequence based	1
115978	33796	ERR222952	ERR222952	217	217	1	PCR/Sequence based	1
55170	33069	ERR222953	ERR222953	217	217	1	PCR/Sequence based	1
32537	32865	ERR222954	ERR222954	217	217	1	PCR/Sequence based	1
34408	32976	ERR222955	ERR222955	217	217	1	PCR/Sequence based	1
129694	34336	ERR222958	ERR222958	217	217	1	PCR/Sequence based	1
115979	33797	ERR222960	ERR222960	217	217	1	PCR/Sequence based	1
31574	32801	ERR222961	ERR222961	217	217	1	PCR/Sequence based	1
32539	32866	ERR222962	ERR222962	217	217	1	PCR/Sequence based	1
34410	32977	ERR222965	ERR222965	217	217	1	PCR/Sequence based	1
31575	32802	ERR222966	ERR222966	217	217	1	PCR/Sequence based	1
31576	32803	ERR222967	ERR222967	217	217	1	PCR/Sequence based	1
31577	32804	ERR222968	ERR222968	217	217	1	PCR/Sequence based	1
32540	32867	ERR222969	ERR222969	5044	217	1	PCR/Sequence based	1
32541	32868	ERR222970	ERR222970	5672	217	1	PCR/Sequence based	1
32542	32869	ERR222971	ERR222971	217	217	1	PCR/Sequence based	1
129213	34320	ERR222972	ERR222972	5044	217	1	PCR/Sequence based	1
115980	33798	ERR222973	ERR222973	5044	217	1	PCR/Sequence based	1
129688	34332	ERR222974	ERR222974	5044	217	1	PCR/Sequence based	1
31578	32805	ERR222975	ERR222975	5672	217	1	PCR/Sequence based	1
129703	34344	ERR222976	ERR222976	5672	217	1	PCR/Sequence based	1
32543	32870	ERR222977	ERR222977	5044	217	1	PCR/Sequence based	1

55171	33070	ERR222978	ERR222978	303	217	1	PCR/Sequence based	1
31579	32806	ERR222979	ERR222979	303	217	1	PCR/Sequence based	1
34411	32978	ERR222980	ERR222980	303	217	1	PCR/Sequence based	1
31580	32807	ERR222981	ERR222981	303	217	1	PCR/Sequence based	1
40702	33035	ERR222990	ERR222990	303	217	1	PCR/Sequence based	1
32545	32871	ERR222991	ERR222991	303	217	1	PCR/Sequence based	1
33510	32928	ERR222992	ERR222992	303	217	1	PCR/Sequence based	1
31583	32808	ERR222993	ERR222993	303	217	1	PCR/Sequence based	1
32546	32872	ERR222994	ERR222994	303	217	1	PCR/Sequence based	1
33511	32929	ERR222995	ERR222995	303	217	1	PCR/Sequence based	1
40703	33036	ERR222996	ERR222996	303	217	1	PCR/Sequence based	1
33512	32930	ERR222999	ERR222999	217	217	1	PCR/Sequence based	1
40704	33037	ERR223002	ERR223002	303	217	1	PCR/Sequence based	1
115981	33799	ERR223003	ERR223003	303	217	1	PCR/Sequence based	1
32549	32873	ERR223007	ERR223007	217	217	1	PCR/Sequence based	1
32550	32874	ERR223008	ERR223008	217	217	1	PCR/Sequence based	1
40705	33038	ERR223009	ERR223009	5002	217	1	PCR/Sequence based	1
33516	32931	ERR223021	ERR223021	2839	217	1	PCR/Sequence based	1
32553	32875	ERR223022	ERR223022	5191	217	1	PCR/Sequence based	1
40708	33039	ERR223023	ERR223023	217	217	1	PCR/Sequence based	1
33517	32932	ERR223024	ERR223024	217	217	1	PCR/Sequence based	1
40709	33040	ERR223026	ERR223026	217	217	1	PCR/Sequence based	1
32554	32876	ERR223027	ERR223027	217	217	1	PCR/Sequence based	1
31588	32809	ERR223028	ERR223028	217	217	1	PCR/Sequence based	1
31589	32810	ERR223030	ERR223030	217	217	1	PCR/Sequence based	1
33519	32933	ERR223031	ERR223031	7112	217	1	PCR/Sequence based	1
129683	34328	ERR223032	ERR223032	217	217	1	PCR/Sequence based	1
31590	32811	ERR223033	ERR223033	217	217	1	PCR/Sequence based	1
18152	32738	ERR223034	ERR223034	217	217	1	PCR/Sequence based	1
18098	32735	ERR223035	ERR223035	SLV217	217	1	PCR/Sequence based	1
40710	33041	ERR223037	ERR223037	217	217	1	PCR/Sequence based	1
31595	32812	ERR226333	ERR226333	217	217	1	PCR/Sequence based	1
31596	32813	ERR226334	ERR226334	217	217	1	PCR/Sequence based	1
32558	32877	ERR226335	ERR226335	217	217	1	PCR/Sequence based	1
33520	32934	ERR226336	ERR226336	5044	217	1	PCR/Sequence based	1
32559	32878	ERR226337	ERR226337	5002	217	1	PCR/Sequence based	1
32560	32879	ERR226338	ERR226338	217	217	1	PCR/Sequence based	1
32561	32880	ERR226340	ERR226340	2019	217	1	PCR/Sequence based	1
33521	32935	ERR226341	ERR226341	618	217	1	PCR/Sequence based	1
31597	32814	ERR226342	ERR226342	2034	217	1	PCR/Sequence based	1
32562	32881	ERR226343	ERR226343	618	217	1	PCR/Sequence based	1
31598	32815	ERR226344	ERR226344	618	217	1	PCR/Sequence based	1
32563	32882	ERR226345	ERR226345	618	217	1	PCR/Sequence based	1
33522	32936	ERR226346	ERR226346	217	217	1	PCR/Sequence based	1
32564	32883	ERR226347	ERR226347	217	217	1	PCR/Sequence based	1
129685	34330	ERR226348	ERR226348	217	217	1	PCR/Sequence based	1
33523	32937	ERR226349	ERR226349	217	217	1	PCR/Sequence based	1
32565	32884	ERR226350	ERR226350	217	217	1	PCR/Sequence based	1
129684	34329	ERR226351	ERR226351	217	217	1	PCR/Sequence based	1
31599	32816	ERR226352	ERR226352	217	217	1	PCR/Sequence based	1
32566	32885	ERR226353	ERR226353	217	217	1	PCR/Sequence based	1
32567	32886	ERR226354	ERR226354	217	217	1	PCR/Sequence based	1
31600	32817	ERR226355	ERR226355	217	217	1	PCR/Sequence based	1
31601	32818	ERR226356	ERR226356	217	217	1	PCR/Sequence based	1
32568	32887	ERR226357	ERR226357	217	217	1	PCR/Sequence based	1
31602	32819	ERR226358	ERR226358	217	217	1	PCR/Sequence based	1
31603	32820	ERR226359	ERR226359	217	217	1	PCR/Sequence based	1
40711	33042	ERR226360	ERR226360	217	217	1	PCR/Sequence based	1
33524	32938	ERR226361	ERR226361	217	217	1	PCR/Sequence based	1

33525	32939	ERR226362	ERR226362	217	217	1	PCR/Sequence based	1
32569	32888	ERR226363	ERR226363	217	217	1	PCR/Sequence based	1
32570	32889	ERR226364	ERR226364	217	217	1	PCR/Sequence based	1
32571	32890	ERR226365	ERR226365	217	217	1	PCR/Sequence based	1
33526	32940	ERR226366	ERR226366	217	217	1	PCR/Sequence based	1
31604	32821	ERR226367	ERR226367	217	217	1	PCR/Sequence based	1
32572	32891	ERR226368	ERR226368	217	217	1	PCR/Sequence based	1
33527	32941	ERR226369	ERR226369	217	217	1	PCR/Sequence based	1
32573	32892	ERR226370	ERR226370	217	217	1	PCR/Sequence based	1
31605	32822	ERR226371	ERR226371	217	217	1	PCR/Sequence based	1
32574	32893	ERR226372	ERR226372	217	217	1	PCR/Sequence based	1
32575	32894	ERR226373	ERR226373	217	217	1	PCR/Sequence based	1
31606	32823	ERR226374	ERR226374	217	217	1	PCR/Sequence based	1
31607	32824	ERR226376	ERR226376	217	217	1	PCR/Sequence based	1
31608	32825	ERR226377	ERR226377	217	217	1	PCR/Sequence based	1
40712	33043	ERR226378	ERR226378	217	217	1	PCR/Sequence based	1
33529	32942	ERR226379	ERR226379	217	217	1	PCR/Sequence based	1
32576	32895	ERR226380	ERR226380	217	217	1	PCR/Sequence based	1
31609	32826	ERR226381	ERR226381	217	217	1	PCR/Sequence based	1
31610	32827	ERR226382	ERR226382	217	217	1	PCR/Sequence based	1
32577	32896	ERR226383	ERR226383	217	217	1	PCR/Sequence based	1
32578	32897	ERR226384	ERR226384	5632	217	1	PCR/Sequence based	1
33530	32943	ERR226385	ERR226385	217	217	1	PCR/Sequence based	1
40713	33044	ERR226386	ERR226386	217	217	1	PCR/Sequence based	1
31611	32828	ERR226387	ERR226387	217	217	1	PCR/Sequence based	1
31612	32829	ERR226388	ERR226388	217	217	1	PCR/Sequence based	1
31613	32830	ERR226389	ERR226389	217	217	1	PCR/Sequence based	1
50355	33061	ERR357214	ERR357214	612	217	1	PCR/Sequence based	1
N/A	35988	N/A	GA47901	304	306	1	unknown	1
N/A	1685	N/A	NCTC7465	615	2296	1	unknown	1
N/A	3210	N/A	P1031	303	217	1	unknown	1
N/A	3217	N/A	P1041	303	217	1	unknown	1
45000	26546	ERR072224	SMRU3075	217	217	1	latex & Quellung	1
82121	26542	ERR048064	SMRU339	217	217	1	latex & Quellung	1
82144	26543	ERR048189	SMRU512	217	217	1	latex & Quellung	1
32957	26544	ERR051484	SMRU674	217	217	1	latex & Quellung	1
82230	26545	ERR052621	SMRU764	217	217	1	latex & Quellung	1
40118	26541	ERR039645	SMRU86	217	217	1	latex & Quellung	1
N/A	35937	N/A	Sp03_2672	306	306	1	unknown	1
N/A	35938	N/A	Sp03-3038	306	306	1	unknown	1
N/A	35994	N/A	Sp06-1370	306	306	1	unknown	1
N/A	35882	N/A	SPnINV104B	227	306	1	unknown	1
N/A	35870	N/A	Sweden1-27	217	217	1	Quellung	1
N/A	35871	N/A	Sweden1-28	306	306	1	Quellung	1
N/A	35876	N/A	Sweden1-40	304	306	1	Quellung	1
121472	33916	ERR046104	UoS3220	306	306	unknown	WGS-based	1
121525	33969	ERR045430	UoS5157	227	306	unknown	WGS-based	1
N/A	35872	N/A	USA1-29	615	2296	1	Quellung	1
N/A	34518	ERR540441	VICE0151	304	306	1	Latex/PCR	1
N/A	34209	ERR388884	VICE0194	306	306	1	Latex/PCR	1
N/A	33085	ERR388913	VICE0244	306	306	1	Latex/PCR	1
N/A	33098	ERR388914	VICE0246a	306	306	1	Latex/PCR	1
N/A	33097	ERR388915	VICE0246b	306	306	1	Latex/PCR	1
N/A	34584	ERR388829	VICE0256	306	306	1	Latex/PCR	1

Hybrid	Hybrid type	Country	Region	carriage/disease	Source	Year
No	N/A	USA	unknown	unknown	unknown	1965
No	N/A	Denmark	unknown	unknown	unknown	1943
No	N/A	Denmark	unknown	unknown	unknown	1943
No	N/A	Denmark	unknown	unknown	unknown	1944
No	N/A	Denmark	unknown	unknown	unknown	1991
No	N/A	Malawi	unknown	disease	unknown	unknown
No	N/A	Malawi	unknown	disease	unknown	unknown
No	N/A	Malawi	unknown	disease	unknown	unknown
No	N/A	Malawi	unknown	disease	unknown	unknown
No	N/A	Malawi	unknown	disease	unknown	unknown
No	N/A	Malawi	unknown	disease	unknown	unknown
No	N/A	Malawi	unknown	disease	unknown	unknown
No	N/A	Malawi	unknown	disease	unknown	unknown
No	N/A	Malawi	unknown	disease	unknown	unknown
No	N/A	Malawi	unknown	disease	unknown	unknown
No	N/A	Malawi	unknown	disease	unknown	unknown
No	N/A	Malawi	unknown	disease	unknown	unknown
No	N/A	Malawi	unknown	disease	unknown	unknown
No	N/A	Malawi	unknown	disease	unknown	unknown
No	N/A	Malawi	unknown	disease	unknown	unknown
No	N/A	Malawi	unknown	disease	unknown	unknown
No	N/A	Malawi	unknown	disease	unknown	unknown
No	N/A	Malawi	unknown	disease	unknown	unknown
No	N/A	Malawi	unknown	disease	unknown	unknown
No	N/A	Malawi	unknown	disease	unknown	unknown
No	N/A	Malawi	unknown	disease	unknown	unknown
No	N/A	Malawi	unknown	disease	unknown	unknown
No	N/A	South Africa	unknown	disease	unknown	unknown
No	N/A	Malawi	unknown	disease	unknown	unknown
No	N/A	Malawi	unknown	disease	unknown	unknown
No	N/A	Malawi	unknown	disease	unknown	unknown
No	N/A	Malawi	unknown	disease	unknown	unknown
No	N/A	Malawi	unknown	disease	unknown	unknown
No	N/A	Malawi	unknown	disease	unknown	unknown
No	N/A	South Africa	unknown	disease	unknown	unknown
No	N/A	South Africa	unknown	disease	unknown	unknown
No	N/A	South Africa	unknown	disease	unknown	unknown
No	N/A	South Africa	unknown	disease	unknown	unknown
No	N/A	South Africa	unknown	disease	unknown	unknown
No	N/A	South Africa	unknown	disease	unknown	unknown
No	N/A	South Africa	unknown	disease	unknown	unknown
No	N/A	South Africa	unknown	disease	unknown	unknown
No	N/A	South Africa	unknown	disease	unknown	unknown
No	N/A	South Africa	unknown	disease	unknown	unknown
No	N/A	South Africa	unknown	disease	unknown	unknown
No	N/A	Mozambique	unknown	disease	unknown	unknown
No	N/A	South Africa	unknown	disease	unknown	unknown
No	N/A	South Africa	unknown	disease	unknown	unknown
No	N/A	South Africa	unknown	disease	unknown	unknown
No	N/A	South Africa	unknown	disease	unknown	unknown
No	N/A	Malawi	unknown	disease	unknown	unknown
No	N/A	Malawi	unknown	disease	unknown	unknown
No	N/A	Malawi	unknown	disease	unknown	unknown
No	N/A	Malawi	unknown	disease	unknown	unknown
No	N/A	Malawi	unknown	disease	unknown	unknown
No	N/A	Malawi	unknown	disease	unknown	unknown
No	N/A	Malawi	unknown	disease	unknown	unknown
No	N/A	Malawi	unknown	disease	unknown	unknown

No	N/A	Malawi	unknown	disease	unknown	unknown
No	N/A	Malawi	unknown	disease	unknown	unknown
No	N/A	Malawi	unknown	disease	unknown	unknown
No	N/A	Malawi	unknown	disease	unknown	unknown
No	N/A	Malawi	unknown	disease	unknown	unknown
No	N/A	Malawi	unknown	disease	unknown	unknown
No	N/A	Malawi	unknown	disease	unknown	unknown
No	N/A	Malawi	unknown	disease	unknown	unknown
No	N/A	Malawi	unknown	disease	unknown	unknown
No	N/A	Malawi	unknown	disease	unknown	unknown
No	N/A	Malawi	unknown	disease	unknown	unknown
No	N/A	Malawi	unknown	disease	unknown	unknown
No	N/A	The Gambia	unknown	unknown	unknown	unknown
No	N/A	The Gambia	unknown	unknown	unknown	unknown
No	N/A	The Gambia	unknown	unknown	unknown	unknown
No	N/A	The Gambia	unknown	unknown	unknown	unknown
No	N/A	The Gambia	unknown	unknown	unknown	unknown
No	N/A	The Gambia	unknown	unknown	unknown	unknown
No	N/A	The Gambia	unknown	unknown	unknown	unknown
No	N/A	The Gambia	unknown	unknown	unknown	unknown
No	N/A	The Gambia	unknown	unknown	unknown	unknown
No	N/A	The Gambia	unknown	unknown	unknown	unknown
No	N/A	The Gambia	unknown	unknown	unknown	unknown
No	N/A	The Gambia	unknown	unknown	unknown	unknown
No	N/A	Togo	unknown	disease	CSF	unknown
No	N/A	Togo	unknown	disease	CSF	unknown
No	N/A	Togo	unknown	disease	CSF	unknown
No	N/A	Togo	unknown	disease	CSF	unknown
No	N/A	Togo	unknown	disease	CSF	unknown
No	N/A	Togo	unknown	disease	CSF	unknown
No	N/A	Niger	unknown	disease	CSF	unknown
No	N/A	Niger	unknown	disease	CSF	unknown
No	N/A	Senegal	unknown	disease	CSF	unknown
No	N/A	Senegal	unknown	disease	CSF	unknown
No	N/A	Senegal	unknown	disease	CSF	unknown
No	N/A	Senegal	unknown	disease	CSF	unknown
No	N/A	Nigeria	unknown	carriage	nasopharynx	unknown
No	N/A	Nigeria	unknown	carriage	nasopharynx	unknown
No	N/A	The Gambia	unknown	unknown	unknown	unknown
No	N/A	The Gambia	unknown	unknown	unknown	unknown
No	N/A	The Gambia	unknown	unknown	unknown	unknown
No	N/A	The Gambia	unknown	unknown	unknown	unknown
No	N/A	The Gambia	unknown	unknown	unknown	unknown
No	N/A	The Gambia	unknown	unknown	unknown	unknown
No	N/A	The Gambia	unknown	unknown	unknown	unknown
No	N/A	The Gambia	unknown	unknown	unknown	unknown
No	N/A	The Gambia	unknown	unknown	unknown	unknown
No	N/A	The Gambia	unknown	unknown	unknown	unknown
No	N/A	The Gambia	unknown	unknown	unknown	unknown
No	N/A	The Gambia	unknown	unknown	unknown	unknown
No	N/A	The Gambia	unknown	unknown	unknown	unknown
No	N/A	The Gambia	unknown	unknown	unknown	unknown
No	N/A	The Gambia	unknown	unknown	unknown	unknown
No	N/A	The Gambia	unknown	unknown	unknown	unknown
No	N/A	The Gambia	unknown	unknown	unknown	unknown
No	N/A	The Gambia	unknown	unknown	unknown	unknown
No	N/A	The Gambia	unknown	unknown	unknown	unknown
No	N/A	The Gambia	unknown	unknown	unknown	unknown
No	N/A	The Gambia	unknown	unknown	unknown	unknown

No	N/A	The Gambia	unknown	unknown	unknown	unknown
No	N/A	The Gambia	unknown	unknown	unknown	unknown
No	N/A	The Gambia	unknown	unknown	unknown	unknown
No	N/A	The Gambia	unknown	unknown	unknown	unknown
No	N/A	The Gambia	unknown	unknown	unknown	unknown
No	N/A	The Gambia	unknown	unknown	unknown	unknown
No	N/A	The Gambia	unknown	unknown	unknown	unknown
No	N/A	The Gambia	unknown	unknown	unknown	unknown
No	N/A	The Gambia	unknown	unknown	unknown	unknown
No	N/A	The Gambia	unknown	unknown	unknown	unknown
No	N/A	The Gambia	unknown	unknown	unknown	unknown
No	N/A	The Gambia	unknown	unknown	unknown	unknown
No	N/A	The Gambia	unknown	unknown	unknown	unknown
No	N/A	The Gambia	unknown	unknown	unknown	unknown
No	N/A	The Gambia	unknown	unknown	unknown	unknown
No	N/A	The Gambia	unknown	unknown	unknown	unknown
No	N/A	The Gambia	unknown	unknown	unknown	unknown
No	N/A	Malawi	unknown	disease	unknown	unknown
No	N/A	Malawi	unknown	disease	unknown	unknown
No	N/A	Malawi	unknown	disease	unknown	unknown
No	N/A	Malawi	unknown	disease	unknown	unknown
No	N/A	Malawi	unknown	disease	unknown	unknown
No	N/A	Malawi	unknown	disease	unknown	unknown
No	N/A	Malawi	unknown	disease	unknown	unknown
No	N/A	Niger	unknown	disease	CSF	unknown
No	N/A	Niger	unknown	disease	CSF	unknown
No	N/A	Niger	unknown	disease	CSF	unknown
No	N/A	Niger	unknown	disease	CSF	unknown
No	N/A	Niger	unknown	disease	CSF	unknown
No	N/A	Niger	unknown	disease	CSF	unknown
No	N/A	Niger	unknown	disease	CSF	unknown
No	N/A	Niger	unknown	disease	CSF	unknown
No	N/A	Niger	unknown	disease	CSF	unknown
No	N/A	Niger	unknown	disease	CSF	unknown
No	N/A	Niger	unknown	disease	CSF	unknown
No	N/A	Niger	unknown	disease	CSF	unknown
No	N/A	Niger	unknown	disease	CSF	unknown
No	N/A	Niger	unknown	disease	CSF	unknown
No	N/A	Niger	unknown	disease	CSF	unknown
No	N/A	Niger	unknown	disease	CSF	unknown
No	N/A	Niger	unknown	disease	CSF	unknown
No	N/A	Niger	unknown	disease	CSF	unknown
No	N/A	Niger	unknown	disease	CSF	unknown
No	N/A	Niger	unknown	disease	CSF	unknown
No	N/A	Niger	unknown	disease	CSF	unknown
No	N/A	Niger	unknown	disease	CSF	unknown
No	N/A	Niger	unknown	disease	CSF	unknown
No	N/A	Niger	unknown	disease	CSF	unknown
No	N/A	Niger	unknown	disease	CSF	unknown
No	N/A	Niger	unknown	disease	CSF	unknown
No	N/A	Niger	unknown	disease	CSF	unknown
No	N/A	Niger	unknown	disease	CSF	unknown
No	N/A	Niger	unknown	disease	CSF	unknown
No	N/A	Niger	unknown	disease	CSF	unknown
No	N/A	Niger	unknown	disease	CSF	unknown
No	N/A	Niger	unknown	disease	CSF	unknown
No	N/A	Niger	unknown	disease	CSF	unknown
No	N/A	Niger	unknown	disease	CSF	unknown
No	N/A	Niger	unknown	disease	CSF	unknown

No	N/A	Niger	unknown	disease	CSF	unknown
No	N/A	Niger	unknown	disease	CSF	unknown
No	N/A	Ghana	unknown	disease	CSF	unknown
No	N/A	Ghana	unknown	disease	CSF	unknown
No	N/A	Ghana	unknown	disease	CSF	unknown
No	N/A	Ghana	unknown	disease	CSF	unknown
No	N/A	Ghana	unknown	disease	CSF	unknown
No	N/A	Ghana	unknown	disease	CSF	unknown
No	N/A	Ghana	unknown	disease	CSF	unknown
No	N/A	Ghana	unknown	disease	CSF	unknown
No	N/A	Ghana	unknown	disease	CSF	unknown
No	N/A	Ghana	unknown	disease	CSF	unknown
No	N/A	Ghana	unknown	disease	CSF	unknown
No	N/A	Ghana	unknown	disease	CSF	unknown
No	N/A	Ghana	unknown	disease	CSF	unknown
No	N/A	Ghana	unknown	disease	CSF	unknown
No	N/A	Ghana	unknown	disease	CSF	unknown
No	N/A	Ghana	unknown	disease	CSF	unknown
No	N/A	Ghana	unknown	disease	CSF	unknown
No	N/A	Ghana	unknown	disease	CSF	unknown
No	N/A	Ghana	unknown	disease	CSF	unknown
No	N/A	Ghana	unknown	disease	CSF	unknown
No	N/A	Ghana	unknown	disease	CSF	unknown
No	N/A	Ghana	unknown	disease	CSF	unknown
No	N/A	Ghana	unknown	disease	CSF	unknown
No	N/A	Ghana	unknown	disease	CSF	unknown
No	N/A	Ghana	unknown	disease	CSF	unknown
No	N/A	Ghana	unknown	disease	CSF	unknown
No	N/A	Ghana	unknown	disease	CSF	unknown
No	N/A	Ghana	unknown	disease	CSF	unknown
No	N/A	Ghana	unknown	disease	CSF	unknown
No	N/A	Ghana	unknown	disease	CSF	unknown
No	N/A	Ghana	unknown	disease	CSF	unknown
No	N/A	Ghana	unknown	disease	CSF	unknown
No	N/A	Ghana	unknown	disease	CSF	unknown
No	N/A	Ghana	unknown	disease	CSF	unknown
No	N/A	Ghana	unknown	disease	CSF	unknown
No	N/A	Thailand	unknown	disease	unknown	unknown
No	N/A	Thailand	unknown	disease	unknown	unknown
No	N/A	Thailand	unknown	disease	unknown	unknown
No	N/A	Thailand	unknown	disease	unknown	unknown
No	N/A	Thailand	unknown	disease	unknown	unknown
No	N/A	Thailand	unknown	disease	unknown	unknown
No	N/A	Thailand	unknown	disease	unknown	unknown
No	N/A	Thailand	unknown	disease	unknown	unknown
No	N/A	Thailand	unknown	disease	unknown	unknown
No	N/A	Thailand	unknown	disease	unknown	unknown
No	N/A	Thailand	unknown	disease	unknown	unknown
No	N/A	Thailand	unknown	disease	unknown	unknown
No	N/A	Thailand	unknown	disease	unknown	unknown
No	N/A	India	unknown	unknown	unknown	unknown
No	N/A	India	unknown	unknown	unknown	unknown
No	N/A	India	unknown	unknown	unknown	unknown
No	N/A	India	unknown	unknown	unknown	unknown
No	N/A	India	unknown	unknown	unknown	unknown
No	N/A	India	unknown	unknown	unknown	unknown
No	N/A	India	unknown	unknown	unknown	unknown
No	N/A	India	unknown	unknown	unknown	unknown
No	N/A	India	unknown	unknown	unknown	unknown

No	N/A	Nepal	unknown	carriage	nasopharynx	unknown
No	N/A	Nepal	unknown	carriage	nasopharynx	unknown
No	N/A	Nepal	unknown	carriage	nasopharynx	unknown
No	N/A	Nepal	unknown	carriage	nasopharynx	unknown
No	N/A	Bangladesh	unknown	disease	blood	unknown
No	N/A	Bangladesh	unknown	disease	blood	unknown
No	N/A	Bangladesh	unknown	disease	blood	unknown
No	N/A	Bangladesh	unknown	disease	blood	unknown
No	N/A	Bangladesh	unknown	disease	blood	unknown
No	N/A	Bangladesh	unknown	disease	blood	unknown
No	N/A	Bangladesh	unknown	disease	blood	unknown
No	N/A	Qatar	unknown	disease	blood	unknown
No	N/A	Qatar	unknown	disease	blood	unknown
No	N/A	Qatar	unknown	disease	blood	unknown
No	N/A	Ethiopia	unknown	carriage	nasopharynx	unknown
No	N/A	Ethiopia	unknown	carriage	nasopharynx	unknown
No	N/A	India	unknown	unknown	unknown	unknown
No	N/A	India	unknown	unknown	unknown	unknown
No	N/A	India	unknown	unknown	unknown	unknown
No	N/A	Philippines	unknown	disease	unknown	unknown
No	N/A	Philippines	unknown	disease	unknown	unknown
No	N/A	Thailand	unknown	disease	unknown	unknown
No	N/A	Thailand	unknown	disease	unknown	unknown
No	N/A	Thailand	unknown	disease	unknown	unknown
No	N/A	Mozambique	unknown	disease	unknown	unknown
No	N/A	Mozambique	unknown	disease	unknown	unknown
No	N/A	Mozambique	unknown	disease	unknown	unknown
No	N/A	Mozambique	unknown	disease	unknown	unknown
No	N/A	Mozambique	unknown	disease	unknown	unknown
No	N/A	Mozambique	unknown	disease	unknown	unknown
No	N/A	Mozambique	unknown	disease	unknown	unknown
No	N/A	India	unknown	unknown	unknown	unknown
No	N/A	India	unknown	unknown	unknown	unknown
No	N/A	India	unknown	unknown	unknown	unknown
No	N/A	India	unknown	unknown	unknown	unknown
No	N/A	India	unknown	unknown	unknown	unknown
No	N/A	Egypt	unknown	disease	CSF	unknown
No	N/A	Egypt	unknown	disease	CSF	unknown
No	N/A	Egypt	unknown	disease	CSF	unknown
No	N/A	Egypt	unknown	disease	CSF	unknown
No	N/A	Egypt	unknown	disease	CSF	unknown
No	N/A	Egypt	unknown	disease	CSF	unknown
No	N/A	Egypt	unknown	disease	CSF	unknown
No	N/A	Mozambique	unknown	disease	unknown	unknown
No	N/A	Mozambique	unknown	disease	unknown	unknown
No	N/A	Mozambique	unknown	disease	unknown	unknown
No	N/A	Mozambique	unknown	disease	unknown	unknown
No	N/A	Mozambique	unknown	disease	unknown	unknown
No	N/A	Mozambique	unknown	disease	unknown	unknown
No	N/A	Mozambique	unknown	disease	unknown	unknown
No	N/A	Mozambique	unknown	disease	unknown	unknown
No	N/A	Mozambique	unknown	disease	unknown	unknown
No	N/A	Mozambique	unknown	disease	unknown	unknown
No	N/A	Mozambique	unknown	disease	unknown	unknown
No	N/A	Mozambique	unknown	disease	unknown	unknown
No	N/A	Mozambique	unknown	disease	unknown	unknown
No	N/A	Mozambique	unknown	disease	unknown	unknown
No	N/A	Mozambique	unknown	disease	unknown	unknown
No	N/A	Mozambique	unknown	disease	unknown	unknown

No	N/A	Mozambique	unknown	disease	unknown	unknown
No	N/A	Mozambique	unknown	disease	unknown	unknown
No	N/A	Mozambique	unknown	disease	unknown	unknown
No	N/A	Mozambique	unknown	disease	unknown	unknown
No	N/A	Mozambique	unknown	disease	unknown	unknown
No	N/A	Mozambique	unknown	disease	unknown	unknown
No	N/A	Mozambique	unknown	disease	unknown	unknown
No	N/A	Mozambique	unknown	disease	unknown	unknown
No	N/A	Mozambique	unknown	disease	unknown	unknown
No	N/A	Mozambique	unknown	disease	unknown	unknown
No	N/A	Mozambique	unknown	disease	unknown	unknown
No	N/A	Mozambique	unknown	disease	unknown	unknown
No	N/A	Mozambique	unknown	disease	unknown	unknown
No	N/A	Mozambique	unknown	disease	unknown	unknown
No	N/A	Mozambique	unknown	disease	unknown	unknown
No	N/A	Mozambique	unknown	disease	unknown	unknown
No	N/A	Mozambique	unknown	disease	unknown	unknown
No	N/A	Mozambique	unknown	disease	unknown	unknown
No	N/A	Mozambique	unknown	disease	unknown	unknown
No	N/A	Mozambique	unknown	disease	unknown	unknown
No	N/A	Mozambique	unknown	disease	unknown	unknown
No	N/A	Mozambique	unknown	disease	unknown	unknown
No	N/A	Mozambique	unknown	disease	unknown	unknown
No	N/A	Mozambique	unknown	disease	unknown	unknown
No	N/A	Mozambique	unknown	disease	unknown	unknown
No	N/A	Mozambique	unknown	disease	unknown	unknown
No	N/A	Thailand	unknown	disease	unknown	unknown
No	N/A	South Africa	unknown	disease	unknown	unknown
No	N/A	USA	Georgia	disease	blood	2006
No	N/A	USA	unknown	disease	unknown	1948
No	N/A	Ghana	Kassena Nankana	disease	CSF	2002
No	N/A	Ghana	Kassena Nankana	disease	CSF	2002
No	N/A	Thailand	Maela	carriage	nasopharynx	2010
No	N/A	Thailand	Maela	carriage	nasopharynx	2008
No	N/A	Thailand	Maela	carriage	nasopharynx	2008
No	N/A	Thailand	Maela	carriage	nasopharynx	2008
No	N/A	Thailand	Maela	carriage	nasopharynx	2008
No	N/A	Thailand	Maela	carriage	nasopharynx	2008
No	N/A	United Kingdom	Scotland	disease	unknown	unknown
No	N/A	United Kingdom	Scotland	disease	unknown	unknown
No	N/A	United Kingdom	unknown	disease	unknown	unknown
No	N/A	United Kingdom	Oxford	disease	blood	1998
No	N/A	Sweden	unknown	disease	blood	1992
No	N/A	Sweden	unknown	disease	blood	1997
No	N/A	Sweden	unknown	disease	unknown	unknown
No	N/A	United Kingdom	unknown	carriage	nasopharynx	unknown
No	N/A	United Kingdom	unknown	carriage	nasopharynx	unknown
No	N/A	USA	unknown	disease	unknown	1948
No	N/A	Iceland	Borgarnes	disease	blood	2009
No	N/A	Iceland	Hafnarfjörður	disease	blood	2010
No	N/A	Iceland	Reykjavík	disease	blood	2012
No	N/A	Iceland	Reykjavík	disease	blood	2012
No	N/A	Iceland	unknown	disease	blood	2012
No	N/A	Iceland	Reykjavík	disease	blood	2012

Publication	Reference	vaccine status
Historical	This publication	pre
Historical	This publication	pre
Historical	This publication	pre
Historical	This publication	pre
Historical	This publication	pre
Cornick Serotype 1	Microbial Genomics, 2015 1. doi: 10.1099/mgen.0.000027	unknown
Cornick Serotype 1	Microbial Genomics, 2015 1. doi: 10.1099/mgen.0.000027	unknown
Cornick Serotype 1	Microbial Genomics, 2015 1. doi: 10.1099/mgen.0.000027	unknown
Cornick Serotype 1	Microbial Genomics, 2015 1. doi: 10.1099/mgen.0.000027	unknown
Cornick Serotype 1	Microbial Genomics, 2015 1. doi: 10.1099/mgen.0.000027	unknown
Cornick Serotype 1	Microbial Genomics, 2015 1. doi: 10.1099/mgen.0.000027	unknown
Cornick Serotype 1	Microbial Genomics, 2015 1. doi: 10.1099/mgen.0.000027	unknown
Cornick Serotype 1	Microbial Genomics, 2015 1. doi: 10.1099/mgen.0.000027	unknown
Cornick Serotype 1	Microbial Genomics, 2015 1. doi: 10.1099/mgen.0.000027	unknown
Cornick Serotype 1	Microbial Genomics, 2015 1. doi: 10.1099/mgen.0.000027	unknown
Cornick Serotype 1	Microbial Genomics, 2015 1. doi: 10.1099/mgen.0.000027	unknown
Cornick Serotype 1	Microbial Genomics, 2015 1. doi: 10.1099/mgen.0.000027	unknown
Cornick Serotype 1	Microbial Genomics, 2015 1. doi: 10.1099/mgen.0.000027	unknown
Cornick Serotype 1	Microbial Genomics, 2015 1. doi: 10.1099/mgen.0.000027	unknown
Cornick Serotype 1	Microbial Genomics, 2015 1. doi: 10.1099/mgen.0.000027	unknown
Cornick Serotype 1	Microbial Genomics, 2015 1. doi: 10.1099/mgen.0.000027	unknown
Cornick Serotype 1	Microbial Genomics, 2015 1. doi: 10.1099/mgen.0.000027	unknown
Cornick Serotype 1	Microbial Genomics, 2015 1. doi: 10.1099/mgen.0.000027	unknown
Cornick Serotype 1	Microbial Genomics, 2015 1. doi: 10.1099/mgen.0.000027	unknown
Cornick Serotype 1	Microbial Genomics, 2015 1. doi: 10.1099/mgen.0.000027	unknown
Cornick Serotype 1	Microbial Genomics, 2015 1. doi: 10.1099/mgen.0.000027	unknown
Cornick Serotype 1	Microbial Genomics, 2015 1. doi: 10.1099/mgen.0.000027	unknown
Cornick Serotype 1	Microbial Genomics, 2015 1. doi: 10.1099/mgen.0.000027	unknown
Cornick Serotype 1	Microbial Genomics, 2015 1. doi: 10.1099/mgen.0.000027	unknown
Cornick Serotype 1	Microbial Genomics, 2015 1. doi: 10.1099/mgen.0.000027	unknown
Cornick Serotype 1	Microbial Genomics, 2015 1. doi: 10.1099/mgen.0.000027	unknown
Cornick Serotype 1	Microbial Genomics, 2015 1. doi: 10.1099/mgen.0.000027	unknown
Cornick Serotype 1	Microbial Genomics, 2015 1. doi: 10.1099/mgen.0.000027	unknown
Cornick Serotype 1	Microbial Genomics, 2015 1. doi: 10.1099/mgen.0.000027	unknown
Cornick Serotype 1	Microbial Genomics, 2015 1. doi: 10.1099/mgen.0.000027	unknown
Cornick Serotype 1	Microbial Genomics, 2015 1. doi: 10.1099/mgen.0.000027	unknown
Cornick Serotype 1	Microbial Genomics, 2015 1. doi: 10.1099/mgen.0.000027	unknown
Cornick Serotype 1	Microbial Genomics, 2015 1. doi: 10.1099/mgen.0.000027	unknown
Cornick Serotype 1	Microbial Genomics, 2015 1. doi: 10.1099/mgen.0.000027	unknown
Cornick Serotype 1	Microbial Genomics, 2015 1. doi: 10.1099/mgen.0.000027	unknown
Cornick Serotype 1	Microbial Genomics, 2015 1. doi: 10.1099/mgen.0.000027	unknown
Cornick Serotype 1	Microbial Genomics, 2015 1. doi: 10.1099/mgen.0.000027	unknown
Cornick Serotype 1	Microbial Genomics, 2015 1. doi: 10.1099/mgen.0.000027	unknown
Cornick Serotype 1	Microbial Genomics, 2015 1. doi: 10.1099/mgen.0.000027	unknown
Cornick Serotype 1	Microbial Genomics, 2015 1. doi: 10.1099/mgen.0.000027	unknown
Cornick Serotype 1	Microbial Genomics, 2015 1. doi: 10.1099/mgen.0.000027	unknown
Cornick Serotype 1	Microbial Genomics, 2015 1. doi: 10.1099/mgen.0.000027	unknown
Cornick Serotype 1	Microbial Genomics, 2015 1. doi: 10.1099/mgen.0.000027	unknown
Cornick Serotype 1	Microbial Genomics, 2015 1. doi: 10.1099/mgen.0.000027	unknown
Cornick Serotype 1	Microbial Genomics, 2015 1. doi: 10.1099/mgen.0.000027	unknown
Cornick Serotype 1	Microbial Genomics, 2015 1. doi: 10.1099/mgen.0.000027	unknown
Cornick Serotype 1	Microbial Genomics, 2015 1. doi: 10.1099/mgen.0.000027	unknown
Cornick Serotype 1	Microbial Genomics, 2015 1. doi: 10.1099/mgen.0.000027	unknown
Cornick Serotype 1	Microbial Genomics, 2015 1. doi: 10.1099/mgen.0.000027	unknown
Cornick Serotype 1	Microbial Genomics, 2015 1. doi: 10.1099/mgen.0.000027	unknown
Cornick Serotype 1	Microbial Genomics, 2015 1. doi: 10.1099/mgen.0.000027	unknown
Cornick Serotype 1	Microbial Genomics, 2015 1. doi: 10.1099/mgen.0.000027	unknown
Cornick Serotype 1	Microbial Genomics, 2015 1. doi: 10.1099/mgen.0.000027	unknown
Cornick Serotype 1	Microbial Genomics, 2015 1. doi: 10.1099/mgen.0.000027	unknown

Cornick Serotype 1	Microbial Genomics, 2015 1. doi: 10.1099/mgen.0.000027	unknown
Cornick Serotype 1	Microbial Genomics, 2015 1. doi: 10.1099/mgen.0.000027	unknown
Cornick Serotype 1	Microbial Genomics, 2015 1. doi: 10.1099/mgen.0.000027	unknown
Cornick Serotype 1	Microbial Genomics, 2015 1. doi: 10.1099/mgen.0.000027	unknown
Cornick Serotype 1	Microbial Genomics, 2015 1. doi: 10.1099/mgen.0.000027	unknown
Cornick Serotype 1	Microbial Genomics, 2015 1. doi: 10.1099/mgen.0.000027	unknown
Cornick Serotype 1	Microbial Genomics, 2015 1. doi: 10.1099/mgen.0.000027	unknown
Cornick Serotype 1	Microbial Genomics, 2015 1. doi: 10.1099/mgen.0.000027	unknown
Cornick Serotype 1	Microbial Genomics, 2015 1. doi: 10.1099/mgen.0.000027	unknown
Cornick Serotype 1	Microbial Genomics, 2015 1. doi: 10.1099/mgen.0.000027	unknown
Cornick Serotype 1	Microbial Genomics, 2015 1. doi: 10.1099/mgen.0.000027	unknown
Cornick Serotype 1	Microbial Genomics, 2015 1. doi: 10.1099/mgen.0.000027	unknown
Cornick Serotype 1	Microbial Genomics, 2015 1. doi: 10.1099/mgen.0.000027	unknown
Cornick Serotype 1	Microbial Genomics, 2015 1. doi: 10.1099/mgen.0.000027	unknown
Cornick Serotype 1	Microbial Genomics, 2015 1. doi: 10.1099/mgen.0.000027	unknown
Cornick Serotype 1	Microbial Genomics, 2015 1. doi: 10.1099/mgen.0.000027	unknown
Cornick Serotype 1	Microbial Genomics, 2015 1. doi: 10.1099/mgen.0.000027	unknown
Cornick Serotype 1	Microbial Genomics, 2015 1. doi: 10.1099/mgen.0.000027	unknown
Cornick Serotype 1	Microbial Genomics, 2015 1. doi: 10.1099/mgen.0.000027	unknown
Cornick Serotype 1	Microbial Genomics, 2015 1. doi: 10.1099/mgen.0.000027	unknown
Cornick Serotype 1	Microbial Genomics, 2015 1. doi: 10.1099/mgen.0.000027	unknown
Cornick Serotype 1	Microbial Genomics, 2015 1. doi: 10.1099/mgen.0.000027	unknown
Cornick Serotype 1	Microbial Genomics, 2015 1. doi: 10.1099/mgen.0.000027	unknown
Cornick Serotype 1	Microbial Genomics, 2015 1. doi: 10.1099/mgen.0.000027	unknown
Cornick Serotype 1	Microbial Genomics, 2015 1. doi: 10.1099/mgen.0.000027	unknown
Cornick Serotype 1	Microbial Genomics, 2015 1. doi: 10.1099/mgen.0.000027	unknown
Cornick Serotype 1	Microbial Genomics, 2015 1. doi: 10.1099/mgen.0.000027	unknown
Cornick Serotype 1	Microbial Genomics, 2015 1. doi: 10.1099/mgen.0.000027	unknown
Cornick Serotype 1	Microbial Genomics, 2015 1. doi: 10.1099/mgen.0.000027	unknown
Cornick Serotype 1	Microbial Genomics, 2015 1. doi: 10.1099/mgen.0.000027	unknown
Cornick Serotype 1	Microbial Genomics, 2015 1. doi: 10.1099/mgen.0.000027	unknown
Cornick Serotype 1	Microbial Genomics, 2015 1. doi: 10.1099/mgen.0.000027	unknown
Cornick Serotype 1	Microbial Genomics, 2015 1. doi: 10.1099/mgen.0.000027	unknown
Cornick Serotype 1	Microbial Genomics, 2015 1. doi: 10.1099/mgen.0.000027	unknown
Cornick Serotype 1	Microbial Genomics, 2015 1. doi: 10.1099/mgen.0.000027	unknown
Cornick Serotype 1	Microbial Genomics, 2015 1. doi: 10.1099/mgen.0.000027	unknown
Cornick Serotype 1	Microbial Genomics, 2015 1. doi: 10.1099/mgen.0.000027	unknown
Cornick Serotype 1	Microbial Genomics, 2015 1. doi: 10.1099/mgen.0.000027	unknown
Cornick Serotype 1	Microbial Genomics, 2015 1. doi: 10.1099/mgen.0.000027	unknown
Cornick Serotype 1	Microbial Genomics, 2015 1. doi: 10.1099/mgen.0.000027	unknown
Cornick Serotype 1	Microbial Genomics, 2015 1. doi: 10.1099/mgen.0.000027	unknown
Cornick Serotype 1	Microbial Genomics, 2015 1. doi: 10.1099/mgen.0.000027	unknown
Cornick Serotype 1	Microbial Genomics, 2015 1. doi: 10.1099/mgen.0.000027	unknown
Cornick Serotype 1	Microbial Genomics, 2015 1. doi: 10.1099/mgen.0.000027	unknown
Cornick Serotype 1	Microbial Genomics, 2015 1. doi: 10.1099/mgen.0.000027	unknown
Cornick Serotype 1	Microbial Genomics, 2015 1. doi: 10.1099/mgen.0.000027	unknown
Cornick Serotype 1	Microbial Genomics, 2015 1. doi: 10.1099/mgen.0.000027	unknown
Cornick Serotype 1	Microbial Genomics, 2015 1. doi: 10.1099/mgen.0.000027	unknown
Cornick Serotype 1	Microbial Genomics, 2015 1. doi: 10.1099/mgen.0.000027	unknown
Cornick Serotype 1	Microbial Genomics, 2015 1. doi: 10.1099/mgen.0.000027	unknown
Cornick Serotype 1	Microbial Genomics, 2015 1. doi: 10.1099/mgen.0.000027	unknown
Cornick Serotype 1	Microbial Genomics, 2015 1. doi: 10.1099/mgen.0.000027	unknown
Cornick Serotype 1	Microbial Genomics, 2015 1. doi: 10.1099/mgen.0.000027	unknown
Cornick Serotype 1	Microbial Genomics, 2015 1. doi: 10.1099/mgen.0.000027	unknown
Cornick Serotype 1	Microbial Genomics, 2015 1. doi: 10.1099/mgen.0.000027	unknown
Cornick Serotype 1	Microbial Genomics, 2015 1. doi: 10.1099/mgen.0.000027	unknown
Cornick Serotype 1	Microbial Genomics, 2015 1. doi: 10.1099/mgen.0.000027	unknown
Cornick Serotype 1	Microbial Genomics, 2015 1. doi: 10.1099/mgen.0.000027	unknown
Cornick Serotype 1	Microbial Genomics, 2015 1. doi: 10.1099/mgen.0.000027	unknown

Cornick Serotype 1	Microbial Genomics, 2015 1. doi: 10.1099/mgen.0.000027	unknown
Cornick Serotype 1	Microbial Genomics, 2015 1. doi: 10.1099/mgen.0.000027	unknown
Cornick Serotype 1	Microbial Genomics, 2015 1. doi: 10.1099/mgen.0.000027	unknown
Cornick Serotype 1	Microbial Genomics, 2015 1. doi: 10.1099/mgen.0.000027	unknown
Cornick Serotype 1	Microbial Genomics, 2015 1. doi: 10.1099/mgen.0.000027	unknown
Cornick Serotype 1	Microbial Genomics, 2015 1. doi: 10.1099/mgen.0.000027	unknown
Cornick Serotype 1	Microbial Genomics, 2015 1. doi: 10.1099/mgen.0.000027	unknown
Cornick Serotype 1	Microbial Genomics, 2015 1. doi: 10.1099/mgen.0.000027	unknown
Cornick Serotype 1	Microbial Genomics, 2015 1. doi: 10.1099/mgen.0.000027	unknown
Cornick Serotype 1	Microbial Genomics, 2015 1. doi: 10.1099/mgen.0.000027	unknown
Cornick Serotype 1	Microbial Genomics, 2015 1. doi: 10.1099/mgen.0.000027	unknown
Cornick Serotype 1	Microbial Genomics, 2015 1. doi: 10.1099/mgen.0.000027	unknown
Cornick Serotype 1	Microbial Genomics, 2015 1. doi: 10.1099/mgen.0.000027	unknown
Cornick Serotype 1	Microbial Genomics, 2015 1. doi: 10.1099/mgen.0.000027	unknown
Cornick Serotype 1	Microbial Genomics, 2015 1. doi: 10.1099/mgen.0.000027	unknown
Cornick Serotype 1	Microbial Genomics, 2015 1. doi: 10.1099/mgen.0.000027	unknown
Cornick Serotype 1	Microbial Genomics, 2015 1. doi: 10.1099/mgen.0.000027	unknown
Cornick Serotype 1	Microbial Genomics, 2015 1. doi: 10.1099/mgen.0.000027	unknown
Cornick Serotype 1	Microbial Genomics, 2015 1. doi: 10.1099/mgen.0.000027	unknown
Cornick Serotype 1	Microbial Genomics, 2015 1. doi: 10.1099/mgen.0.000027	unknown
Cornick Serotype 1	Microbial Genomics, 2015 1. doi: 10.1099/mgen.0.000027	unknown
Cornick Serotype 1	Microbial Genomics, 2015 1. doi: 10.1099/mgen.0.000027	unknown
Cornick Serotype 1	Microbial Genomics, 2015 1. doi: 10.1099/mgen.0.000027	unknown
Cornick Serotype 1	Microbial Genomics, 2015 1. doi: 10.1099/mgen.0.000027	unknown
Cornick Serotype 1	Microbial Genomics, 2015 1. doi: 10.1099/mgen.0.000027	unknown
Cornick Serotype 1	Microbial Genomics, 2015 1. doi: 10.1099/mgen.0.000027	unknown
Cornick Serotype 1	Microbial Genomics, 2015 1. doi: 10.1099/mgen.0.000027	unknown
Cornick Serotype 1	Microbial Genomics, 2015 1. doi: 10.1099/mgen.0.000027	unknown
Cornick Serotype 1	Microbial Genomics, 2015 1. doi: 10.1099/mgen.0.000027	unknown
Cornick Serotype 1	Microbial Genomics, 2015 1. doi: 10.1099/mgen.0.000027	unknown
Cornick Serotype 1	Microbial Genomics, 2015 1. doi: 10.1099/mgen.0.000027	unknown
Cornick Serotype 1	Microbial Genomics, 2015 1. doi: 10.1099/mgen.0.000027	unknown
Cornick Serotype 1	Microbial Genomics, 2015 1. doi: 10.1099/mgen.0.000027	unknown
Cornick Serotype 1	Microbial Genomics, 2015 1. doi: 10.1099/mgen.0.000027	unknown
Cornick Serotype 1	Microbial Genomics, 2015 1. doi: 10.1099/mgen.0.000027	unknown
Cornick Serotype 1	Microbial Genomics, 2015 1. doi: 10.1099/mgen.0.000027	unknown
Cornick Serotype 1	Microbial Genomics, 2015 1. doi: 10.1099/mgen.0.000027	unknown
Cornick Serotype 1	Microbial Genomics, 2015 1. doi: 10.1099/mgen.0.000027	unknown
Cornick Serotype 1	Microbial Genomics, 2015 1. doi: 10.1099/mgen.0.000027	unknown
Cornick Serotype 1	Microbial Genomics, 2015 1. doi: 10.1099/mgen.0.000027	unknown
Cornick Serotype 1	Microbial Genomics, 2015 1. doi: 10.1099/mgen.0.000027	unknown
Cornick Serotype 1	Microbial Genomics, 2015 1. doi: 10.1099/mgen.0.000027	unknown
Cornick Serotype 1	Microbial Genomics, 2015 1. doi: 10.1099/mgen.0.000027	unknown
Cornick Serotype 1	Microbial Genomics, 2015 1. doi: 10.1099/mgen.0.000027	unknown
Cornick Serotype 1	Microbial Genomics, 2015 1. doi: 10.1099/mgen.0.000027	unknown
Cornick Serotype 1	Microbial Genomics, 2015 1. doi: 10.1099/mgen.0.000027	unknown
Cornick Serotype 1	Microbial Genomics, 2015 1. doi: 10.1099/mgen.0.000027	unknown
Cornick Serotype 1	Microbial Genomics, 2015 1. doi: 10.1099/mgen.0.000027	unknown
Cornick Serotype 1	Microbial Genomics, 2015 1. doi: 10.1099/mgen.0.000027	unknown
Cornick Serotype 1	Microbial Genomics, 2015 1. doi: 10.1099/mgen.0.000027	unknown
Cornick Serotype 1	Microbial Genomics, 2015 1. doi: 10.1099/mgen.0.000027	unknown
Cornick Serotype 1	Microbial Genomics, 2015 1. doi: 10.1099/mgen.0.000027	unknown
Cornick Serotype 1	Microbial Genomics, 2015 1. doi: 10.1099/mgen.0.000027	unknown
Cornick Serotype 1	Microbial Genomics, 2015 1. doi: 10.1099/mgen.0.000027	unknown
Cornick Serotype 1	Microbial Genomics, 2015 1. doi: 10.1099/mgen.0.000027	unknown
Cornick Serotype 1	Microbial Genomics, 2015 1. doi: 10.1099/mgen.0.000027	unknown
Cornick Serotype 1	Microbial Genomics, 2015 1. doi: 10.1099/mgen.0.000027	unknown
Cornick Serotype 1	Microbial Genomics, 2015 1. doi: 10.1099/mgen.0.000027	unknown
Cornick Serotype 1	Microbial Genomics, 2015 1. doi: 10.1099/mgen.0.000027	unknown

Cornick Serotype 1	Microbial Genomics, 2015 1. doi: 10.1099/mgen.0.000027	unknown
Cornick Serotype 1	Microbial Genomics, 2015 1. doi: 10.1099/mgen.0.000027	unknown
Cornick Serotype 1	Microbial Genomics, 2015 1. doi: 10.1099/mgen.0.000027	unknown
Cornick Serotype 1	Microbial Genomics, 2015 1. doi: 10.1099/mgen.0.000027	unknown
Cornick Serotype 1	Microbial Genomics, 2015 1. doi: 10.1099/mgen.0.000027	unknown
Cornick Serotype 1	Microbial Genomics, 2015 1. doi: 10.1099/mgen.0.000027	unknown
Cornick Serotype 1	Microbial Genomics, 2015 1. doi: 10.1099/mgen.0.000027	unknown
Cornick Serotype 1	Microbial Genomics, 2015 1. doi: 10.1099/mgen.0.000027	unknown
Cornick Serotype 1	Microbial Genomics, 2015 1. doi: 10.1099/mgen.0.000027	unknown
Cornick Serotype 1	Microbial Genomics, 2015 1. doi: 10.1099/mgen.0.000027	unknown
Cornick Serotype 1	Microbial Genomics, 2015 1. doi: 10.1099/mgen.0.000027	unknown
Cornick Serotype 1	Microbial Genomics, 2015 1. doi: 10.1099/mgen.0.000027	unknown
Cornick Serotype 1	Microbial Genomics, 2015 1. doi: 10.1099/mgen.0.000027	unknown
Cornick Serotype 1	Microbial Genomics, 2015 1. doi: 10.1099/mgen.0.000027	unknown
Cornick Serotype 1	Microbial Genomics, 2015 1. doi: 10.1099/mgen.0.000027	unknown
Cornick Serotype 1	Microbial Genomics, 2015 1. doi: 10.1099/mgen.0.000027	unknown
Cornick Serotype 1	Microbial Genomics, 2015 1. doi: 10.1099/mgen.0.000027	unknown
Cornick Serotype 1	Microbial Genomics, 2015 1. doi: 10.1099/mgen.0.000027	unknown
Cornick Serotype 1	Microbial Genomics, 2015 1. doi: 10.1099/mgen.0.000027	unknown
Cornick Serotype 1	Microbial Genomics, 2015 1. doi: 10.1099/mgen.0.000027	unknown
Cornick Serotype 1	Microbial Genomics, 2015 1. doi: 10.1099/mgen.0.000027	unknown
Cornick Serotype 1	Microbial Genomics, 2015 1. doi: 10.1099/mgen.0.000027	unknown
Cornick Serotype 1	Microbial Genomics, 2015 1. doi: 10.1099/mgen.0.000027	unknown
Cornick Serotype 1	Microbial Genomics, 2015 1. doi: 10.1099/mgen.0.000027	unknown
Cornick Serotype 1	Microbial Genomics, 2015 1. doi: 10.1099/mgen.0.000027	unknown
Cornick Serotype 1	Microbial Genomics, 2015 1. doi: 10.1099/mgen.0.000027	unknown
Cornick Serotype 1	Microbial Genomics, 2015 1. doi: 10.1099/mgen.0.000027	unknown
Cornick Serotype 1	Microbial Genomics, 2015 1. doi: 10.1099/mgen.0.000027	unknown
Cornick Serotype 1	Microbial Genomics, 2015 1. doi: 10.1099/mgen.0.000027	unknown
Cornick Serotype 1	Microbial Genomics, 2015 1. doi: 10.1099/mgen.0.000027	unknown
Cornick Serotype 1	Microbial Genomics, 2015 1. doi: 10.1099/mgen.0.000027	unknown
Cornick Serotype 1	Microbial Genomics, 2015 1. doi: 10.1099/mgen.0.000027	unknown
Cornick Serotype 1	Microbial Genomics, 2015 1. doi: 10.1099/mgen.0.000027	unknown
Cornick Serotype 1	Microbial Genomics, 2015 1. doi: 10.1099/mgen.0.000027	unknown
Cornick Serotype 1	Microbial Genomics, 2015 1. doi: 10.1099/mgen.0.000027	unknown
Cornick Serotype 1	Microbial Genomics, 2015 1. doi: 10.1099/mgen.0.000027	unknown
Cornick Serotype 1	Microbial Genomics, 2015 1. doi: 10.1099/mgen.0.000027	unknown
Cornick Serotype 1	Microbial Genomics, 2015 1. doi: 10.1099/mgen.0.000027	unknown
Cornick Serotype 1	Microbial Genomics, 2015 1. doi: 10.1099/mgen.0.000027	unknown
Cornick Serotype 1	Microbial Genomics, 2015 1. doi: 10.1099/mgen.0.000027	unknown
Cornick Serotype 1	Microbial Genomics, 2015 1. doi: 10.1099/mgen.0.000027	unknown
Cornick Serotype 1	Microbial Genomics, 2015 1. doi: 10.1099/mgen.0.000027	unknown
Cornick Serotype 1	Microbial Genomics, 2015 1. doi: 10.1099/mgen.0.000027	unknown
Cornick Serotype 1	Microbial Genomics, 2015 1. doi: 10.1099/mgen.0.000027	unknown
Cornick Serotype 1	Microbial Genomics, 2015 1. doi: 10.1099/mgen.0.000027	unknown
Cornick Serotype 1	Microbial Genomics, 2015 1. doi: 10.1099/mgen.0.000027	unknown
Cornick Serotype 1	Microbial Genomics, 2015 1. doi: 10.1099/mgen.0.000027	unknown
Cornick Serotype 1	Microbial Genomics, 2015 1. doi: 10.1099/mgen.0.000027	unknown
Cornick Serotype 1	Microbial Genomics, 2015 1. doi: 10.1099/mgen.0.000027	unknown
Cornick Serotype 1	Microbial Genomics, 2015 1. doi: 10.1099/mgen.0.000027	unknown
Cornick Serotype 1	Microbial Genomics, 2015 1. doi: 10.1099/mgen.0.000027	unknown
Cornick Serotype 1	Microbial Genomics, 2015 1. doi: 10.1099/mgen.0.000027	unknown
Cornick Serotype 1	Microbial Genomics, 2015 1. doi: 10.1099/mgen.0.000027	unknown
Cornick Serotype 1	Microbial Genomics, 2015 1. doi: 10.1099/mgen.0.000027	unknown
Cornick Serotype 1	Microbial Genomics, 2015 1. doi: 10.1099/mgen.0.000027	unknown
Cornick Serotype 1	Microbial Genomics, 2015 1. doi: 10.1099/mgen.0.000027	unknown
Cornick Serotype 1	Microbial Genomics, 2015 1. doi: 10.1099/mgen.0.000027	unknown
Cornick Serotype 1	Microbial Genomics, 2015 1. doi: 10.1099/mgen.0.000027	unknown
Cornick Serotype 1	Microbial Genomics, 2015 1. doi: 10.1099/mgen.0.000027	unknown

Cornick Serotype 1	Microbial Genomics, 2015 1. doi: 10.1099/mgen.0.000027	unknown
Cornick Serotype 1	Microbial Genomics, 2015 1. doi: 10.1099/mgen.0.000027	unknown
Cornick Serotype 1	Microbial Genomics, 2015 1. doi: 10.1099/mgen.0.000027	unknown
Cornick Serotype 1	Microbial Genomics, 2015 1. doi: 10.1099/mgen.0.000027	unknown
Cornick Serotype 1	Microbial Genomics, 2015 1. doi: 10.1099/mgen.0.000027	unknown
Cornick Serotype 1	Microbial Genomics, 2015 1. doi: 10.1099/mgen.0.000027	unknown
Cornick Serotype 1	Microbial Genomics, 2015 1. doi: 10.1099/mgen.0.000027	unknown
Cornick Serotype 1	Microbial Genomics, 2015 1. doi: 10.1099/mgen.0.000027	unknown
Cornick Serotype 1	Microbial Genomics, 2015 1. doi: 10.1099/mgen.0.000027	unknown
Cornick Serotype 1	Microbial Genomics, 2015 1. doi: 10.1099/mgen.0.000027	unknown
Cornick Serotype 1	Microbial Genomics, 2015 1. doi: 10.1099/mgen.0.000027	unknown
Cornick Serotype 1	Microbial Genomics, 2015 1. doi: 10.1099/mgen.0.000027	unknown
Cornick Serotype 1	Microbial Genomics, 2015 1. doi: 10.1099/mgen.0.000027	unknown
Cornick Serotype 1	Microbial Genomics, 2015 1. doi: 10.1099/mgen.0.000027	unknown
Cornick Serotype 1	Microbial Genomics, 2015 1. doi: 10.1099/mgen.0.000027	unknown
Cornick Serotype 1	Microbial Genomics, 2015 1. doi: 10.1099/mgen.0.000027	unknown
Cornick Serotype 1	Microbial Genomics, 2015 1. doi: 10.1099/mgen.0.000027	unknown
Cornick Serotype 1	Microbial Genomics, 2015 1. doi: 10.1099/mgen.0.000027	unknown
Cornick Serotype 1	Microbial Genomics, 2015 1. doi: 10.1099/mgen.0.000027	unknown
Cornick Serotype 1	Microbial Genomics, 2015 1. doi: 10.1099/mgen.0.000027	unknown
Cornick Serotype 1	Microbial Genomics, 2015 1. doi: 10.1099/mgen.0.000027	unknown
Cornick Serotype 1	Microbial Genomics, 2015 1. doi: 10.1099/mgen.0.000027	unknown
Cornick Serotype 1	Microbial Genomics, 2015 1. doi: 10.1099/mgen.0.000027	unknown
Cornick Serotype 1	Microbial Genomics, 2015 1. doi: 10.1099/mgen.0.000027	unknown
Cornick Serotype 1	Microbial Genomics, 2015 1. doi: 10.1099/mgen.0.000027	unknown
Cornick Serotype 1	Microbial Genomics, 2015 1. doi: 10.1099/mgen.0.000027	unknown
Cornick Serotype 1	Microbial Genomics, 2015 1. doi: 10.1099/mgen.0.000027	unknown
Cornick Serotype 1	Microbial Genomics, 2015 1. doi: 10.1099/mgen.0.000027	unknown
Cornick Serotype 1	Microbial Genomics, 2015 1. doi: 10.1099/mgen.0.000027	unknown
Cornick Serotype 1	Microbial Genomics, 2015 1. doi: 10.1099/mgen.0.000027	unknown
Cornick Serotype 1	Microbial Genomics, 2015 1. doi: 10.1099/mgen.0.000027	unknown
Cornick Serotype 1	Microbial Genomics, 2015 1. doi: 10.1099/mgen.0.000027	unknown
Cornick Serotype 1	Microbial Genomics, 2015 1. doi: 10.1099/mgen.0.000027	unknown
Cornick Serotype 1	Microbial Genomics, 2015 1. doi: 10.1099/mgen.0.000027	unknown
Cornick Serotype 1	Microbial Genomics, 2015 1. doi: 10.1099/mgen.0.000027	unknown
Cornick Serotype 1	Microbial Genomics, 2015 1. doi: 10.1099/mgen.0.000027	unknown
Cornick Serotype 1	Microbial Genomics, 2015 1. doi: 10.1099/mgen.0.000027	unknown
Cornick Serotype 1	Microbial Genomics, 2015 1. doi: 10.1099/mgen.0.000027	unknown
Cornick Serotype 1	Microbial Genomics, 2015 1. doi: 10.1099/mgen.0.000027	unknown
Cornick Serotype 1	Microbial Genomics, 2015 1. doi: 10.1099/mgen.0.000027	unknown
Cornick Serotype 1	Microbial Genomics, 2015 1. doi: 10.1099/mgen.0.000027	unknown
Cornick Serotype 1	Microbial Genomics, 2015 1. doi: 10.1099/mgen.0.000027	unknown
Cornick Serotype 1	Microbial Genomics, 2015 1. doi: 10.1099/mgen.0.000027	unknown
Cornick Serotype 1	Microbial Genomics, 2015 1. doi: 10.1099/mgen.0.000027	unknown
Cornick Serotype 1	Microbial Genomics, 2015 1. doi: 10.1099/mgen.0.000027	unknown
Cornick Serotype 1	Microbial Genomics, 2015 1. doi: 10.1099/mgen.0.000027	unknown
Cornick Serotype 1	Microbial Genomics, 2015 1. doi: 10.1099/mgen.0.000027	unknown
Cornick Serotype 1	Microbial Genomics, 2015 1. doi: 10.1099/mgen.0.000027	unknown
Cornick Serotype 1	Microbial Genomics, 2015 1. doi: 10.1099/mgen.0.000027	unknown
Cornick Serotype 1	Microbial Genomics, 2015 1. doi: 10.1099/mgen.0.000027	unknown
Cornick Serotype 1	Microbial Genomics, 2015 1. doi: 10.1099/mgen.0.000027	unknown
Cornick Serotype 1	Microbial Genomics, 2015 1. doi: 10.1099/mgen.0.000027	unknown
Cornick Serotype 1	Microbial Genomics, 2015 1. doi: 10.1099/mgen.0.000027	unknown
Cornick Serotype 1	Microbial Genomics, 2015 1. doi: 10.1099/mgen.0.000027	unknown
Cornick Serotype 1	Microbial Genomics, 2015 1. doi: 10.1099/mgen.0.000027	unknown
Cornick Serotype 1	Microbial Genomics, 2015 1. doi: 10.1099/mgen.0.000027	unknown
Cornick Serotype 1	Microbial Genomics, 2015 1. doi: 10.1099/mgen.0.000027	unknown
Cornick Serotype 1	Microbial Genomics, 2015 1. doi: 10.1099/mgen.0.000027	unknown
Cornick Serotype 1	Microbial Genomics, 2015 1. doi: 10.1099/mgen.0.000027	unknown

Cornick Serotype 1	Microbial Genomics, 2015 1. doi: 10.1099/mgen.0.000027	unknown
Cornick Serotype 1	Microbial Genomics, 2015 1. doi: 10.1099/mgen.0.000027	unknown
Cornick Serotype 1	Microbial Genomics, 2015 1. doi: 10.1099/mgen.0.000027	unknown
Cornick Serotype 1	Microbial Genomics, 2015 1. doi: 10.1099/mgen.0.000027	unknown
Cornick Serotype 1	Microbial Genomics, 2015 1. doi: 10.1099/mgen.0.000027	unknown
Cornick Serotype 1	Microbial Genomics, 2015 1. doi: 10.1099/mgen.0.000027	unknown
Cornick Serotype 1	Microbial Genomics, 2015 1. doi: 10.1099/mgen.0.000027	unknown
Cornick Serotype 1	Microbial Genomics, 2015 1. doi: 10.1099/mgen.0.000027	unknown
Cornick Serotype 1	Microbial Genomics, 2015 1. doi: 10.1099/mgen.0.000027	unknown
Cornick Serotype 1	Microbial Genomics, 2015 1. doi: 10.1099/mgen.0.000027	unknown
Cornick Serotype 1	Microbial Genomics, 2015 1. doi: 10.1099/mgen.0.000027	unknown
Cornick Serotype 1	Microbial Genomics, 2015 1. doi: 10.1099/mgen.0.000027	unknown
Cornick Serotype 1	Microbial Genomics, 2015 1. doi: 10.1099/mgen.0.000027	unknown
Cornick Serotype 1	Microbial Genomics, 2015 1. doi: 10.1099/mgen.0.000027	unknown
Cornick Serotype 1	Microbial Genomics, 2015 1. doi: 10.1099/mgen.0.000027	unknown
Cornick Serotype 1	Microbial Genomics, 2015 1. doi: 10.1099/mgen.0.000027	unknown
Cornick Serotype 1	Microbial Genomics, 2015 1. doi: 10.1099/mgen.0.000027	unknown
Cornick Serotype 1	Microbial Genomics, 2015 1. doi: 10.1099/mgen.0.000027	unknown
Cornick Serotype 1	Microbial Genomics, 2015 1. doi: 10.1099/mgen.0.000027	unknown
Cornick Serotype 1	Microbial Genomics, 2015 1. doi: 10.1099/mgen.0.000027	unknown
Cornick Serotype 1	Microbial Genomics, 2015 1. doi: 10.1099/mgen.0.000027	unknown
Cornick Serotype 1	Microbial Genomics, 2015 1. doi: 10.1099/mgen.0.000027	unknown
Cornick Serotype 1	Microbial Genomics, 2015 1. doi: 10.1099/mgen.0.000027	unknown
Cornick Serotype 1	Microbial Genomics, 2015 1. doi: 10.1099/mgen.0.000027	unknown
Cornick Serotype 1	Microbial Genomics, 2015 1. doi: 10.1099/mgen.0.000027	unknown
Cornick Serotype 1	Microbial Genomics, 2015 1. doi: 10.1099/mgen.0.000027	unknown
Cornick Serotype 1	Microbial Genomics, 2015 1. doi: 10.1099/mgen.0.000027	unknown
Cornick Serotype 1	Microbial Genomics, 2015 1. doi: 10.1099/mgen.0.000027	unknown
Genbank (336)	N/A	post
Genbank (336)	N/A	pre
Genbank (336)	N/A	pre
Genbank (336)	N/A	pre
Maela	PMID: 24509479	pre
Maela	PMID: 24509479	pre
Maela	PMID: 24509479	pre
Maela	PMID: 24509479	pre
Maela	PMID: 24509479	pre
Maela	PMID: 24509479	pre
Genbank (336)	N/A	unknown
Genbank (336)	N/A	unknown
Genbank (336)	N/A	unknown
Genbank (336)	N/A	pre
PMEN clones	This publication	pre
PMEN clones	This publication	pre
PMEN clones	This publication	unknown
Gladstone UK PCV	PMID: 25776920	unknown
Gladstone UK PCV	PMID: 25776920	unknown
PMEN clones	This publication	pre
VICE	This publication	pre
VICE	This publication	pre
VICE	This publication	post
VICE	This publication	post
VICE	This publication	post
VICE	This publication	post

Serotype 1 lineage
C
A
A
A
A
B
B
B
B
B
B
B
B
B
B
B
B
B
B
B
B
B
B
B
B
B
B
B
B
B
B
B
B
B
B
B
B
B
B
B
B
B
B
B
B
B
B
B
B
B
B
B
B
B
B
B
B
B

B
B
B
B
B
B
B
B
B
B
B
B
B
B
B
B
B
B
B
B
B
B
B
B
B
B
B
B
B
B
B
B
B
B
B
B
B
B
B
B
B
B
B
B
B
B
B
B
B
B
B
B
B
B
B
B
B
B
B

B
B
B
B
B
B
B
B
B
B
B
B
B
B
B
B
B
B
B
B
B
B
B
B
B
B
B
B
B
B
B
B
B
B
B
B
B
B
B
B
B
B
B
B
B
B
B
B
B
B
B
B
B
B
B
B
B
B
B

B
B
B
B
B
B
B
B
B
B
B
B
B
B
B
B
B
B
B
B
B
B
B
B
B
B
B
B
B
B
B
B
B
B
B
B
B
B
B
B
B
B
B
B
B
B
B
B
B
B
B
B
B
B
B
B
B
B
B

B
B
B
B
B
B
B
B
B
B
B
B
B
B
B
B
B
B
B
B
B
B
B
B
B
B
B
B
B
B
B
B
B
B
B
B
B
B
B
B
B
B
B
B
B
B
B
B
B
B
B
B
B
B
B
B
B
B
B

B
B
B
B
B
B
B
B
B
B
B
B
B
B
B
B
B
B
B
B
B
B
B
B
B
B
B
B
A
C
B
B
B
B
B
B
B
B
A
A
A
A
B
A
A
A
A
C
A
A
A
A
A
A

SEROTYPE 1 ALL ISOLATES

rmlst ID	BIGS id	Name	CC	Seq_sero	wzg			wzh		
					95	142	338	27	56	168
		1 reference			Q	E	A	A	A	S
18140		ERR018044	217	1	Q	E	A	A	A	S
32767		ERR023321	217	1	Q	E	A	A	A	S
115753		ERR023322	217	1	Q	E	A	A	A	S
33755		ERR023325	217	1	Q	E	A	A	A	S
33756		ERR023326	217	1	Q	E	A	A	A	S
31870		ERR023328	217	1	Q	E	A	A	A	S
31871		ERR023329	217	1	Q	E	A	A	A	S
32768		ERR023330	217	1	Q	E	A	A	A	S
44801		ERR023331	217	1	Q	E	A	A	A	S
39992		ERR023332	217	1	Q	E	A	A	A	S
39993		ERR023334	217	1	Q	E	A	A	A	S
33757		ERR023335	217	1	Q	E	A	A	A	S
39994		ERR023336	217	1	Q	E	A	A	A	S
32769		ERR023337	217	1	Q	E	A	A	A	S
55120		ERR023338	217	1	Q	E	A	A	A	S
115754		ERR023339	217	1	Q	E	A	A	A	S
129690		ERR023340	217	1	Q	E	A	A	A	S
32770		ERR023342	217	1	Q	E	A	A	A	S
115755		ERR023343	217	1	Q	E	A	A	A	S
39995		ERR023344	217	1	Q	E	A	A	A	S
39996		ERR023345	217	1	Q	E	A	A	A	S
44137		ERR023351	217	1	Q	E	A	A	A	S
39998		ERR023354	217	1	Q	E	A	A	A	S
33759		ERR023357	217	1	Q	E	A	A	A	S
129700		ERR023358	217	1	Q	E	A	A	A	S
33760		ERR023376	217	1	Q	E	A	A	A	S
129701		ERR023381	217	1	Q	E	A	A	A	S
115759		ERR023393	217	1	Q	E	A	A	A	S
33761		ERR023394	217	1	Q	E	A	A	A	S
44802		ERR023569	217	1	Q	E	A	A	A	S
33766		ERR023570	217	1	Q	E	A	A	A	S
33767		ERR023572	217	1	Q	E	A	A	A	S
44803		ERR023573	217	1	Q	E	A	A	A	S
115765		ERR023575	217	1	Q	E	A	A	A	S
44804		ERR023577	217	1	Q	E	A	T	A	S
40006		ERR023578	217	1	Q	E	A	A	A	S
115766		ERR023579	217	1	Q	E	A	A	A	S
129702		ERR023585	217	1	Q	E	A	A	A	S
32783		ERR023589	217	1	Q	E	A	A	A	S
129689		ERR023592	217	1	Q	E	A	A	A	S
129211		ERR023595	217	1	Q	E	A	A	A	S
129696		ERR023601	217	1	Q	E	A	A	A	S
40009		ERR023605	217	1	Q	E	A	A	A	S
44805		ERR023608	217	1	Q	E	A	A	A	S
32789		ERR023610	217	1	Q	E	A	A	A	S
40074		ERR028396	217	1	Q	E	A	A	A	S
40075		ERR028400	217	1	Q	E	A	A	A	S
31206		ERR028406	217	1	Q	E	A	A	A	S
18335		ERR028411	217	1	Q	E	A	A	A	S
40081		ERR028423	217	1	Q	E	A	A	A	S
40084		ERR028438	217	1	Q	E	A	A	A	S
32863		ERR028439	217	1	Q	E	A	A	A	S
33818		ERR028440	217	1	Q	E	A	A	A	S
40085		ERR028444	217	1	Q	E	A	A	A	S
40086		ERR028447	217	1	Q	E	A	A	A	S
18154		ERR028451	217	1	Q	E	A	A	A	S
55914		ERR028452	217	1	Q	E	A	A	A	S
32867		ERR028458	217	1	Q	E	A	A	A	S
31218		ERR033114	217	1	Q	E	A	A	A	S
18081		ERR033120	217	1	Q	E	A	A	A	S
18334		ERR033122	217	1	Q	E	A	A	A	S
18319		ERR033123	217	1	Q	E	A	A	A	S

18326	ERR033141	217	1	Q	E	A	A	A	S
31220	ERR033146	217	1	Q	E	A	A	A	S
18261	ERR033151	217	1	Q	E	A	A	A	S
44144	ERR042138	217	1	Q	E	A	A	A	S
32903	ERR042139	217	1	Q	E	A	A	A	S
31948	ERR042140	217	1	Q	E	A	A	A	S
32904	ERR042141	217	1	Q	E	A	A	A	S
32905	ERR042142	217	1	Q	K	A	A	A	S
32906	ERR042143	217	1	Q	E	A	A	A	S
31949	ERR042144	217	1	Q	E	A	A	A	S
40120	ERR042145	217	1	Q	E	A	A	A	S
31950	ERR042146	217	1	Q	E	A	A	A	S
31951	ERR042147	217	1	Q	E	A	A	A	S
31952	ERR042148	217	1	Q	E	A	A	A	S
31953	ERR042149	217	1	Q	E	A	A	A	S
31954	ERR042150	217	1	Q	E	A	A	A	S
33846	ERR042151	217	1	Q	E	A	A	A	S
31955	ERR042152	217	1	Q	E	A	A	A	S
32907	ERR042153	217	1	Q	E	A	A	A	S
31956	ERR042154	217	1	Q	E	A	A	A	S
31957	ERR042155	217	1	Q	E	A	A	A	S
40121	ERR042156	217	1	Q	E	A	A	A	S
40122	ERR042157	217	1	Q	E	A	A	A	S
31958	ERR042158	217	1	Q	E	A	A	A	S
31959	ERR042159	217	1	Q	E	A	A	A	S
32908	ERR042160	217	1	Q	E	A	A	A	S
32909	ERR042161	217	1	Q	E	A	A	A	S
31960	ERR042162	217	1	Q	E	A	A	A	S
33847	ERR042163	217	1	Q	E	A	A	A	S
33848	ERR042164	217	1	Q	E	A	A	A	S
40123	ERR042165	217	1	Q	E	A	A	A	S
31961	ERR042166	217	1	Q	E	A	A	A	S
31962	ERR042167	217	1	Q	E	A	A	A	S
31221	ERR042168	217	1	Q	E	A	A	A	S
40124	ERR042169	217	1	Q	E	A	A	A	S
40125	ERR042170	217	1	Q	E	A	A	A	S
18446	ERR042173	217	1	Q	E	A	A	A	S
18350	ERR042174	217	1	Q	E	A	A	A	S
18462	ERR042175	217	1	Q	E	A	A	A	S
18362	ERR042176	217	1	Q	E	A	A	A	S
18464	ERR042177	217	1	Q	E	A	A	A	S
18371	ERR042178	217	1	Q	E	A	A	A	S
18421	ERR042179	217	1	Q	E	A	A	A	S
18346	ERR042180	217	1	Q	E	A	A	A	S
31964	ERR042181	217	1	Q	E	A	A	A	S
18444	ERR042182	217	1	Q	E	A	A	A	S
18442	ERR042183	217	1	Q	E	A	A	A	S
32910	ERR042184	217	1	Q	E	A	A	A	S
31965	ERR042185	217	1	Q	E	A	A	A	S
31966	ERR042186	217	1	Q	E	A	A	A	S
32911	ERR042187	217	1	Q	E	A	A	A	S
32912	ERR042188	217	1	Q	E	A	A	A	S
31967	ERR042189	217	1	Q	E	A	A	A	S
18316	ERR042190	217	1	Q	E	A	A	A	S
33849	ERR042191	217	1	Q	E	A	A	A	S
40126	ERR042192	217	1	Q	E	A	A	A	S
32913	ERR042193	217	1	Q	E	A	A	A	S
33850	ERR042195	217	1	Q	E	A	A	A	S
18398	ERR042197	217	1	Q	E	A	A	A	S
31223	ERR042198	217	1	Q	E	A	A	A	S
18268	ERR042199	217	1	Q	E	A	A	A	S
18227	ERR042200	217	1	Q	E	A	A	A	S
18375	ERR042201	217	1	Q	E	A	A	A	S
18188	ERR042203	217	1	Q	E	A	A	A	S
40128	ERR042204	217	1	Q	E	A	A	A	S
18440	ERR042206	217	1	Q	E	A	A	A	S
31968	ERR042207	217	1	Q	E	A	A	A	S

115841	ERR045783	217	1	Q	E	A		A	A	S
33853	ERR045786	217	1	Q	E	A		A	A	S
32915	ERR045787	217	1	Q	E	A		A	A	S
129697	ERR045789	217	1	Q	E	A		A	A	S
129743	ERR045790	217	1	Q	E	A		A	A	S
129687	ERR045791	217	1	Q	E	A		A	A	S
115842	ERR045792	217	1	Q	E	A		A	A	S
115850	ERR046710	217	1	Q	E	A		A	A	S
40142	ERR046711	217	1	Q	E	A		A	A	S
40143	ERR046712	217	1	Q	E	A		A	A	S
40144	ERR046713	217	1	Q	E	A		A	A	S
55922	ERR046714	217	1	Q	E	A		A	A	S
33862	ERR046715	217	1	Q	E	A		A	A	S
33863	ERR046716	217	1	Q	E	A		A	A	S
33864	ERR046717	217	1	R	E	A		A	A	S
33865	ERR046718	217	1	Q	E	A		A	A	L
40145	ERR046719	217	1	Q	E	A		A	A	S
55127	ERR046720	217	1	Q	E	A		A	A	S
115851	ERR046721	217	1	Q	E	A		A	A	S
40146	ERR046722	217	1	Q	E	A		A	A	S
40147	ERR046723	217	1	Q	E	A		A	A	S
55128	ERR046724	217	1	Q	E	A		A	A	S
55923	ERR046725	217	1	Q	E	A		A	A	S
40148	ERR046726	217	1	Q	E	A		A	A	S
44829	ERR046727	217	1	Q	E	A		A	A	S
40149	ERR046728	217	1	Q	E	A		A	A	S
129744	ERR059358	217	1	Q	E	A		A	A	S
129752	ERR059359	217	1	Q	E	A		A	A	S
129754	ERR059360	217	1	Q	E	A		A	A	S
129741	ERR059361	217	1	Q	E	A		A	A	S
129753	ERR059362	217	1	Q	E	A		A	A	S
129707	ERR059363	217	1	Q	E	A		A	A	S
129731	ERR059364	217	1	Q	E	A		A	A	S
129751	ERR059365	217	1	Q	E	A		A	A	S
129691	ERR059366	217	1	Q	E	A		A	A	L
129699	ERR059367	217	1	Q	E	A		A	A	S
129722	ERR059368	217	1	Q	E	A		A	A	S
129705	ERR059369	217	1	Q	E	A		A	A	S
129749	ERR059370	217	1	Q	E	A		A	A	S
129750	ERR059371	217	1	Q	E	A		A	A	S
129738	ERR059372	217	1	Q	E	A		A	A	S
129740	ERR059373	217	1	Q	E	A		A	A	S
129724	ERR059375	217	1	Q	E	A		A	A	S
129695	ERR059376	217	1	Q	E	A		A	A	S
129714	ERR128823	217	1	Q	E	A		A	A	S
129704	ERR128824	217	1	Q	E	A		A	A	S
129709	ERR128825	217	1	Q	E	A		A	A	S
129725	ERR128826	217	1	Q	E	A		A	A	S
129726	ERR128827	217	1	Q	E	A		A	A	S
129713	ERR128828	217	1	Q	E	A		A	A	S
129728	ERR128829	217	1	Q	E	A		A	A	S
129716	ERR128830	217	1	Q	E	A		A	A	S
129748	ERR128831	217	1	Q	E	A		A	A	S
129746	ERR128832	217	1	Q	E	A		A	A	S
129737	ERR128833	217	1	Q	E	A		A	A	S
129739	ERR128834	217	1	Q	E	A		A	A	S
129708	ERR128835	217	1	Q	E	A		A	A	S
129734	ERR128836	217	1	Q	E	A		A	A	S
129715	ERR128837	217	1	Q	E	A		A	A	S
129712	ERR128838	217	1	Q	E	A		A	A	S
129711	ERR128839	217	1	Q	E	A		A	A	S
129735	ERR128840	217	1	Q	E	A		A	A	S
129742	ERR128841	217	1	Q	E	A		A	A	S
129745	ERR128842	217	1	Q	E	A		A	A	S
129721	ERR128843	217	1	Q	E	A		A	A	S
129212	ERR128844	217	1	Q	E	A		A	A	S
129727	ERR128845	217	1	Q	E	A		A	A	S

129747	ERR128846	217	1	Q	E	A	A	A	S
129706	ERR128847	217	1	Q	E	A	A	A	S
129719	ERR128848	217	1	Q	E	A	A	A	S
129718	ERR128849	217	1	Q	E	A	A	A	S
129730	ERR128850	217	1	Q	E	A	A	A	S
129732	ERR128851	217	1	Q	E	A	A	A	S
129729	ERR128852	217	1	Q	E	A	A	A	S
129723	ERR128853	217	1	Q	E	A	A	A	S
129717	ERR128854	217	1	Q	E	A	A	A	S
129733	ERR128855	217	1	Q	E	A	A	A	S
129736	ERR128856	217	1	Q	E	A	A	A	S
129720	ERR128857	217	1	Q	E	A	A	A	S
115977	ERR222951	217	1	Q	E	A	A	A	S
115978	ERR222952	217	1	Q	E	A	A	A	S
55170	ERR222953	217	1	Q	E	A	A	A	S
32537	ERR222954	217	1	Q	E	A	A	A	S
34408	ERR222955	217	1	Q	E	A	A	A	S
129694	ERR222958	217	1	Q	E	A	A	A	S
115979	ERR222960	217	1	Q	E	A	A	A	S
31574	ERR222961	217	1	Q	E	A	A	A	S
32539	ERR222962	217	1	Q	E	A	A	A	S
34410	ERR222965	217	1	Q	E	A	A	A	S
31575	ERR222966	217	1	Q	E	A	A	A	S
31576	ERR222967	217	1	Q	E	A	A	A	S
31577	ERR222968	217	1	Q	E	A	A	A	S
32540	ERR222969	217	1	Q	E	A	A	A	S
32541	ERR222970	217	1	Q	E	A	A	A	S
32542	ERR222971	217	1	Q	E	A	A	A	S
129213	ERR222972	217	1	Q	E	A	A	A	S
115980	ERR222973	217	1	Q	E	A	A	A	S
129688	ERR222974	217	1	Q	E	A	A	A	S
31578	ERR222975	217	1	Q	E	A	A	A	S
129703	ERR222976	217	1	Q	E	A	A	A	S
32543	ERR222977	217	1	Q	E	A	A	A	S
55171	ERR222978	217	1	Q	E	A	A	A	S
31579	ERR222979	217	1	Q	E	A	A	A	S
34411	ERR222980	217	1	Q	E	A	A	A	S
31580	ERR222981	217	1	Q	E	A	A	A	S
40702	ERR222990	217	1	Q	E	A	A	A	S
32545	ERR222991	217	1	Q	E	A	A	A	S
33510	ERR222992	217	1	Q	E	A	A	A	S
31583	ERR222993	217	1	Q	E	A	A	A	S
32546	ERR222994	217	1	Q	E	A	A	A	S
33511	ERR222995	217	1	Q	E	A	A	A	S
40703	ERR222996	217	1	Q	E	A	A	A	S
33512	ERR222999	217	1	Q	E	A	A	A	S
40704	ERR223002	217	1	Q	E	A	A	A	S
115981	ERR223003	217	1	Q	E	A	A	A	S
32549	ERR223007	217	1	Q	E	A	A	A	S
32550	ERR223008	217	1	Q	E	A	A	A	S
40705	ERR223009	217	1	Q	E	A	A	V	S
33516	ERR223021	217	1	Q	E	A	A	A	S
32553	ERR223022	217	1	Q	E	A	A	A	S
40708	ERR223023	217	1	Q	E	A	A	A	S
33517	ERR223024	217	1	Q	E	A	A	A	S
40709	ERR223026	217	1	Q	E	A	A	A	S
32554	ERR223027	217	1	Q	E	A	A	A	S
31588	ERR223028	217	1	Q	E	A	A	A	S
31589	ERR223030	217	1	Q	E	A	A	A	S
33519	ERR223031	217	1	Q	E	A	A	A	S
129683	ERR223032	217	1	Q	E	A	A	A	S
31590	ERR223033	217	1	Q	E	A	A	A	S
18152	ERR223034	217	1	Q	E	A	A	A	S
18098	ERR223035	217	1	Q	E	A	A	A	S
40710	ERR223037	217	1	Q	E	A	A	A	S
31595	ERR226333	217	1	Q	E	A	A	A	S
31596	ERR226334	217	1	Q	E	A	A	A	S

32558		ERR226335	217	1	Q	E	A	A	A	S
33520		ERR226336	217	1	Q	E	A	A	A	S
32559		ERR226337	217	1	Q	E	A	A	V	S
32560		ERR226338	217	1	Q	E	A	A	A	S
32561		ERR226340	217	1	Q	E	V	A	A	S
33521		ERR226341	217	1	Q	E	A	A	A	S
31597		ERR226342	217	1	Q	E	A	A	A	S
32562		ERR226343	217	1	Q	E	A	A	A	S
31598		ERR226344	217	1	Q	E	A	A	A	S
32563		ERR226345	217	1	Q	E	A	A	A	S
33522		ERR226346	217	1	Q	E	A	A	A	S
32564		ERR226347	217	1	Q	E	A	A	A	S
129685		ERR226348	217	1	Q	E	A	A	A	S
33523		ERR226349	217	1	Q	E	A	A	A	S
32565		ERR226350	217	1	Q	E	A	A	A	S
129684		ERR226351	217	1	Q	E	A	A	A	S
31599		ERR226352	217	1	Q	E	A	A	A	S
32566		ERR226353	217	1	Q	E	A	A	A	S
32567		ERR226354	217	1	Q	E	A	A	A	S
31600		ERR226355	217	1	Q	E	A	A	A	S
31601		ERR226356	217	1	Q	E	A	A	A	S
32568		ERR226357	217	1	Q	E	A	A	A	S
31602		ERR226358	217	1	Q	E	A	A	A	S
31603		ERR226359	217	1	Q	E	A	A	A	S
40711		ERR226360	217	1	Q	E	A	A	A	S
33524		ERR226361	217	1	Q	E	A	A	A	S
33525		ERR226362	217	1	Q	E	A	A	A	S
32569		ERR226363	217	1	Q	E	A	A	A	S
32570		ERR226364	217	1	Q	E	A	A	A	S
32571		ERR226365	217	1	Q	E	A	A	A	S
33526		ERR226366	217	1	Q	E	A	A	A	S
31604		ERR226367	217	1	Q	E	A	A	A	S
32572		ERR226368	217	1	Q	E	A	A	A	S
33527		ERR226369	217	1	Q	E	A	A	A	S
32573		ERR226370	217	1	Q	E	A	A	A	S
31605		ERR226371	217	1	Q	E	A	A	A	S
32574		ERR226372	217	1	Q	E	A	A	A	S
32575		ERR226373	217	1	Q	E	A	A	A	S
31606		ERR226374	217	1	Q	E	A	A	A	S
31607		ERR226376	217	1	Q	E	A	A	A	S
31608		ERR226377	217	1	Q	E	A	A	A	S
40712		ERR226378	217	1	Q	E	A	A	A	S
33529		ERR226379	217	1	Q	E	A	A	A	S
32576		ERR226380	217	1	Q	E	A	A	A	S
31609		ERR226381	217	1	Q	E	A	A	A	S
31610		ERR226382	217	1	Q	E	A	A	A	S
32577		ERR226383	217	1	Q	E	A	A	A	S
32578		ERR226384	217	1	Q	E	A	A	A	S
33530		ERR226385	217	1	Q	E	A	A	A	S
40713		ERR226386	217	1	Q	E	A	A	A	S
31611		ERR226387	217	1	Q	E	A	A	A	S
31612		ERR226388	217	1	Q	E	A	A	A	S
31613		ERR226389	217	1	Q	E	A	A	A	S
50355		ERR357214	217	1	Q	E	A	A	A	S
N/A	319	P1031	217	1	Q	E	A	A	A	S
N/A	1197	P1041	217	1	Q	E	A	A	A	S
45000		SMRU3075	217	1	Q	E	A	A	A	S
82121		SMRU339	217	1	Q	E	A	A	A	S
82144		SMRU512	217	1	Q	E	A	A	A	S
32957		SMRU674	217	1	Q	E	A	A	A	S
82230		SMRU764	217	1	Q	E	A	A	A	S
40118		SMRU86	217	1	Q	E	A	A	A	S
N/A	287	Sweden1-27	217	1	Q	E	A	A	A	S
N/A	2886	1/4	306	1	Q	E	A	A	A	S
N/A	4	1/5	306	1	Q	E	A	A	A	S
N/A	5	1/6	306	1	Q	E	A	A	A	S
N/A	6	1/7	306	1	Q	E	A	A	A	S

N/A	1180	GA47901	306	1	Q	E	A	A	A	S
N/A	1114	Sp03_2672	306	1	Q	E	A	A	A	S
N/A	1115	Sp03-3038	306	1	Q	E	A	A	A	S
N/A	1199	Sp06-1370	306	1	Q	E	A	A	A	S
N/A	315	SPnINV104B	306	1	Q	E	A	A	A	S
N/A	288	Sweden1-28	306	1	Q	E	A	A	A	S
N/A	300	Sweden1-40	306	1	Q	E	A	A	A	S
121472		UoS3220	306	1	Q	E	A	A	A	S
121525		UoS5157	306	1	Q	E	A	A	A	S
N/A	472	VICE0151	306	1	Q	E	A	A	A	S
N/A	515	VICE0194	306	1	Q	E	A	A	A	S
N/A	565	VICE0244	306	1	Q	E	A	A	A	S
N/A	567	VICE0246a	306	1	Q	E	A	A	A	S
N/A	568	VICE0246b	306	1	Q	E	A	A	A	S
N/A	578	VICE0256	306	1	Q	E	A	A	A	S
N/A	1	1/2	2296	1	Q	E	A	A	A	S
N/A	1198	NCTC7465	2296	1	Q	E	A	A	A	S
N/A	289	USA1-29	2296	1	Q	E	A	A	A	S

wzd																				
6	17	18	41	49	56	65	73	74	104	129	151	172	204	207	216	217	220	228	44	50
T	A	L	T	T	V	P	Q	A	V	D	I	S	R	R	Q	M	L	G	N	T
T	A	L	T	T	V	P	Q	A	M	D	I	S	R	R	Q	M	L	G	N	T
T	A	L	T	T	V	P	Q	A	M	D	I	S	R	R	Q	M	L	G	N	T
T	A	L	T	T	V	P	Q	A	M	D	I	S	R	R	Q	M	L	G	N	T
T	A	L	T	T	V	P	Q	A	M	D	I	S	R	R	Q	M	L	G	N	T
T	A	L	T	T	V	P	Q	A	M	D	I	S	R	R	Q	M	L	G	N	T
T	A	L	T	T	V	P	Q	A	M	D	I	S	R	R	Q	M	F	G	N	T
T	A	L	T	T	V	P	Q	A	M	D	I	S	R	R	Q	M	L	G	N	T
T	A	L	T	T	V	P	Q	A	M	D	I	S	R	R	Q	M	L	G	N	T
T	A	L	T	T	V	P	Q	A	M	D	I	S	R	R	Q	M	L	G	N	T
T	A	L	T	T	V	P	Q	A	M	D	I	S	R	R	Q	M	L	G	N	T
T	A	L	T	T	V	P	Q	A	M	D	I	S	R	R	Q	M	L	G	N	T
T	A	L	T	T	V	S	Q	V	M	D	I	S	R	R	Q	M	L	G	N	T
T	A	L	T	T	V	P	Q	A	M	D	I	S	R	R	Q	M	L	G	N	T
T	A	L	T	T	V	P	Q	A	M	D	I	S	R	R	Q	M	L	G	N	T
T	A	L	T	T	V	P	Q	A	M	D	I	S	R	R	Q	M	L	G	N	T
T	A	L	T	T	V	P	Q	A	M	D	I	S	R	R	Q	M	L	G	N	T
T	A	L	T	T	V	P	Q	A	M	D	I	S	R	R	Q	M	L	G	N	T
T	A	L	T	T	V	P	Q	A	M	D	I	S	R	R	Q	M	L	G	N	T
T	A	L	T	T	V	P	Q	A	M	D	I	S	R	R	Q	M	L	G	N	T
T	A	L	T	T	V	P	Q	A	M	D	I	S	R	R	Q	M	L	G	N	T
T	A	L	T	T	V	P	Q	A	M	D	I	S	R	R	Q	M	L	G	N	T
T	A	L	T	T	V	S	Q	V	M	D	I	S	R	R	Q	M	L	G	N	T
T	A	L	T	T	V	P	Q	A	M	D	I	S	R	R	Q	M	L	G	N	T
T	A	L	T	T	V	P	Q	A	M	D	I	S	R	R	Q	M	L	G	N	T
T	A	L	T	T	V	P	Q	A	M	D	I	S	R	R	Q	M	L	G	N	T
T	A	L	T	T	V	P	Q	A	M	D	I	S	R	R	Q	M	L	G	N	T
T	A	L	T	T	V	P	Q	A	M	D	I	S	R	R	Q	M	L	G	N	T
T	A	L	T	T	V	P	Q	V	M	D	I	S	R	R	Q	M	L	G	N	T
T	A	L	T	T	V	P	Q	A	M	D	I	S	R	R	Q	M	L	G	N	T
T	A	L	T	T	V	P	Q	A	M	D	I	S	R	R	Q	M	L	G	N	T
T	A	L	T	T	V	P	Q	A	M	D	I	S	R	R	Q	M	L	G	N	T
T	A	L	T	T	V	P	Q	A	M	D	I	S	R	R	Q	M	L	G	N	T
T	A	L	T	T	V	P	Q	A	M	D	I	S	R	R	Q	M	L	G	N	T
T	A	L	T	T	V	P	Q	A	M	D	I	S	R	R	Q	M	L	G	N	T
T	A	L	T	T	V	P	Q	A	M	D	I	S	R	R	Q	M	L	G	N	T
T	A	L	T	T	V	S	Q	A	M	D	I	S	R	R	Q	M	L	G	N	T
T	A	L	T	T	V	P	Q	A	M	D	I	S	R	R	Q	M	L	G	N	T
T	A	L	T	T	V	P	Q	A	M	D	I	S	R	R	Q	M	L	G	N	T
T	A	L	T	T	V	P	Q	A	M	D	I	S	R	R	Q	M	L	G	N	T
T	A	L	T	T	V	P	Q	A	M	D	I	S	R	R	Q	M	L	G	N	T
T	A	L	T	T	V	P	Q	A	M	D	I	S	R	R	Q	M	L	G	N	T
T	A	L	T	T	V	S	Q	A	M	D	I	S	R	R	Q	M	L	G	N	T
T	A	L	T	T	V	P	Q	A	M	D	I	S	R	R	Q	M	L	G	N	T
T	A	L	T	T	V	P	Q	A	M	D	I	S	R	R	Q	M	L	G	N	T
T	A	L	T	T	V	P	Q	A	M	D	I	S	R	R	Q	M	L	G	N	T
T	A	L	T	T	V	P	Q	A	M	D	I	S	R	R	Q	M	L	G	N	T
T	A	L	T	T	V	P	Q	A	M	D	I	S	R	R	Q	M	L	G	N	T
T	A	L	T	T	V	P	Q	A	M	D	I	S	R	R	Q	M	L	G	N	T
T	A	L	T	T	V	P	Q	A	M	D	I	S	R	R	Q	M	L	G	N	T
T	A	L	T	T	V	P	Q	A	M	D	I	S	R	R	Q	M	L	G	N	T
T	A	L	T	T	V	P	Q	A	M	D	I	S	R	R	Q	M	L	G	N	T
T	A	L	T	T	V	P	Q	A	M	D	I	S	R	R	Q	M	L	G	N	T
T	A	L	T	T	V	P	Q	A	M	D	I	S	R	R	Q	M	L	G	N	T
T	A	L	T	T	V	S	Q	A	M	D	I	S	R	R	Q	M	L	G	N	T
T	A	L	T	T	V	P	Q	A	M	D	I	S	R	R	Q	M	L	G	N	T
T	A	L	T	T	V	P	Q	A	M	D	I	S	R	R	Q	M	L	G	N	T
T	A	L	T	T	V	P	Q	A	M	D	I	S	R	R	Q	M	L	G	N	T
T	A	L	T	T	V	P	Q	A	M	D	I	S	R	R	Q	M	L	G	N	T
T	A	L	T	T	V	P	Q	A	M	D	I	S	R	R	Q	M	L	G	N	T
T	A	L	T	T	V	P	Q	A	M	D	I	S	R	R	Q	M	L	G	N	T
T	A	L	T	T	V	P	Q	A	M	D	I	S	R	R	Q	M	L	G	N	T
T	A	L	T	T	V	P	Q	A	M	D	I	S	R	R	Q	M	L	G	N	T

T	A	L	T	T	V	P	Q	A	M	D	I	S	R	R	Q	M	L	G	N	T
T	A	L	T	T	V	P	Q	A	M	D	I	S	R	R	Q	M	L	G	N	T
T	A	L	T	T	V	P	Q	A	M	D	I	S	R	R	Q	M	L	G	N	T
T	A	L	T	T	V	P	Q	A	M	D	I	S	R	R	Q	M	L	G	N	T
T	A	L	T	T	V	P	Q	A	M	D	I	S	R	R	Q	M	L	G	N	T
T	A	L	T	T	V	P	Q	A	M	D	I	S	R	R	Q	M	L	G	N	T
T	A	L	T	T	V	P	Q	A	M	D	I	S	R	R	Q	M	L	G	N	T
T	A	L	T	T	V	P	Q	A	M	D	I	S	R	R	Q	M	L	G	N	T
T	A	L	T	T	V	P	Q	A	M	D	I	S	R	R	Q	M	L	G	N	T
T	A	L	T	T	V	P	Q	A	M	D	I	S	R	R	Q	M	L	G	N	T
T	A	L	T	T	V	P	Q	A	M	D	I	S	R	R	Q	M	L	G	N	T
T	A	L	T	T	V	P	Q	A	M	D	I	S	R	R	Q	M	L	G	N	T
T	A	L	T	T	V	P	Q	A	M	D	I	S	R	R	Q	M	L	G	N	T
T	A	L	T	T	V	P	Q	A	M	D	I	S	R	R	Q	M	L	G	N	T
T	A	L	T	T	V	P	Q	A	M	D	I	S	R	R	Q	M	L	G	N	T
T	A	L	T	T	V	P	Q	A	M	D	I	S	R	R	Q	M	L	G	N	T
I	A	L	T	T	V	P	Q	A	M	D	I	S	R	R	Q	M	L	G	N	T
I	A	L	T	T	V	P	Q	A	M	D	I	S	R	R	Q	M	L	G	N	T
I	A	L	T	T	V	P	Q	A	M	D	I	S	R	R	Q	M	L	G	N	T
I	A	L	T	T	V	P	Q	A	M	D	I	S	R	R	Q	M	L	G	N	T
T	A	L	T	T	V	P	Q	A	M	D	I	S	R	R	Q	M	L	G	N	T
T	A	L	T	T	V	P	Q	A	M	D	I	S	R	R	Q	M	L	G	N	T
T	A	L	T	T	V	P	Q	A	M	D	I	S	R	R	Q	M	L	G	N	T
T	A	L	T	T	V	P	Q	A	M	D	I	S	R	R	Q	M	L	G	N	T
T	A	L	T	T	V	P	Q	A	M	D	I	S	R	R	Q	M	L	G	N	T
T	A	L	T	T	V	P	Q	A	M	D	I	S	R	R	Q	M	L	G	N	T
T	A	L	T	T	V	P	Q	A	M	D	I	S	R	R	Q	M	L	G	N	T
T	A	L	T	T	V	P	Q	A	M	D	I	S	R	R	Q	M	L	G	N	T
T	A	L	T	T	V	P	Q	A	M	D	I	S	R	R	Q	M	L	G	N	T
T	A	L	T	T	V	P	Q	A	M	D	I	S	R	R	Q	M	L	G	N	T
T	A	L	T	T	V	P	Q	A	M	D	I	S	R	R	Q	M	L	G	N	T
T	A	L	T	T	V	P	Q	A	M	D	I	S	R	R	Q	M	L	G	N	T
T	A	F	T	T	V	P	Q	A	M	D	I	S	R	R	Q	M	L	G	N	T
T	A	L	T	T	V	P	Q	A	M	D	I	S	R	R	Q	M	L	G	N	T
T	A	L	T	T	V	P	Q	A	M	D	I	S	R	R	Q	M	L	G	N	T
T	A	L	T	T	V	P	Q	A	M	D	I	S	R	R	Q	M	L	G	N	T
T	A	L	T	T	V	P	Q	A	M	D	I	S	R	R	Q	M	L	G	N	T
T	A	L	T	T	V	P	Q	A	M	D	I	S	R	R	Q	M	L	G	N	T
T	A	L	T	T	V	P	Q	A	M	D	I	S	R	R	Q	M	L	G	N	T
T	A	L	T	T	V	P	Q	A	M	D	I	S	R	R	Q	M	L	G	N	T
T	A	F	T	T	V	P	Q	A	M	D	I	S	R	R	Q	M	L	G	N	T
T	A	L	T	T	V	P	Q	A	M	D	I	S	R	R	Q	M	L	G	N	T
T	A	L	T	T	V	P	Q	A	M	D	I	S	R	R	Q	M	L	G	N	T
T	A	L	T	T	V	P	Q	A	M	D	I	S	R	R	Q	M	L	G	N	T
T	A	L	T	T	V	P	Q	A	M	D	I	S	R	R	Q	M	L	G	N	T
T	A	L	T	T	V	P	Q	A	M	D	I	S	R	R	Q	M	L	G	N	T
T	A	L	T	T	V	P	Q	A	M	D	I	S	R	R	Q	M	L	G	N	T
T	A	L	T	T	V	P	Q	A	M	D	I	F	R	R	Q	M	L	G	N	T
T	A	F	T	T	V	P	Q	A	M	D	I	S	R	R	Q	M	L	G	N	T
T	A	L	T	T	V	P	Q	A	M	D	I	S	R	R	Q	M	L	G	N	T
T	A	L	T	T	V	P	Q	A	M	D	I	S	R	R	Q	M	L	G	N	T
T	A	L	T	T	V	P	Q	A	M	D	I	S	R	R	Q	M	L	G	N	T
T	A	L	T	T	V	P	Q	A	M	D	I	S	R	R	Q	M	L	G	N	T
T	A	L	T	T	V	P	Q	A	M	D	I	S	R	R	Q	M	L	G	N	T
T	A	L	T	T	V	P	Q	A	M	D	I	S	R	R	Q	M	L	G	N	T
T	A	L	T	T	V	P	Q	A	M	D	I	S	R	R	Q	M	L	G	N	T
T	A	L	T	T	V	P	Q	A	M	D	I	S	R	R	Q	M	L	G	N	T
T	A	L	T	T	V	P	Q	A	M	D	I	S	R	R	Q	M	L	G	N	T
T	A	L	T	T	V	P	Q	A	M	D	I	S	R	R	Q	M	L	G	N	T
T	A	L	T	T	V	P	Q	A	M	D	I	S	R	R	Q	M	L	G	N	T
T	A	L	T	T	V	P	Q	A	M	D	I	S	R	R	Q	M	L	G	N	T
T	A	L	T	T	V	P	Q	A	M	D	I	S	R	R	Q	M	L	G	N	T
T	A	L	T	T	V	P	Q	A	M	D	I	S	R	R	Q	M	L	G	N	T
T	A	L	T	T	V	P	Q	A	M	D	I	S	R	R	Q	M	L	G	N	T
T	A	L	T	T	V	P	Q	A	M	D	I	S	R	R	Q	M	L	G	N	T
T	A	L	T	T	V	P	Q	A	M	D	I	S	R	R	Q	M	L	G	N	T
T	A	L	T	T	V	P	Q	A	M	D	I	S	R	R	Q	M	L	G	N	T

T	A	L	T	T	V	P	Q	A	M	D	I	S	R	R	Q	M	L	G	N	T
T	A	L	T	T	V	P	Q	A	M	D	I	S	R	R	Q	M	L	G	N	T
T	A	L	T	T	V	P	Q	A	M	D	I	S	R	R	Q	M	L	G	N	T
T	A	L	T	T	V	P	Q	A	M	D	I	S	R	R	Q	M	L	G	N	T
T	A	L	T	T	V	P	Q	A	M	D	I	S	R	R	Q	M	L	G	N	T
T	A	L	T	T	V	P	Q	A	M	D	I	S	R	R	Q	M	F	G	N	T
T	A	L	T	T	V	P	Q	A	M	D	I	S	R	R	Q	M	L	G	N	T
T	A	L	T	T	V	P	Q	A	M	D	V	S	R	R	Q	M	L	G	N	T
T	A	L	T	T	V	P	Q	A	M	D	V	S	R	R	Q	M	L	G	N	T
T	A	L	T	T	V	P	Q	A	M	D	I	S	R	R	Q	M	L	G	S	T
T	A	L	T	T	V	P	Q	A	M	D	I	S	R	L	Q	M	L	G	N	T
T	A	L	T	T	V	P	Q	A	M	D	I	S	R	R	Q	M	L	G	N	T
T	A	L	T	T	V	P	Q	A	M	D	I	S	R	R	Q	M	L	G	N	T
T	A	L	T	T	V	P	Q	A	M	D	I	S	R	R	Q	M	L	G	N	T
T	A	L	T	T	V	P	Q	A	M	D	I	S	R	R	Q	M	L	G	N	T
T	A	L	T	T	V	P	Q	A	M	D	I	S	R	R	Q	M	L	G	N	T
T	A	L	T	T	V	P	Q	A	M	D	I	S	R	R	Q	M	L	G	N	T
T	A	L	T	T	V	P	Q	A	M	D	I	S	R	R	Q	M	L	G	N	T
T	A	L	T	T	V	P	Q	A	M	D	I	S	R	R	Q	M	L	G	N	T
T	A	L	T	T	V	P	Q	A	M	D	I	S	R	R	Q	M	L	G	N	T
T	A	L	T	T	V	P	Q	A	M	D	I	S	R	R	Q	M	L	G	N	T
T	A	L	T	T	V	P	Q	A	M	D	I	S	R	R	Q	M	L	G	N	T
T	A	L	T	T	V	P	Q	A	M	D	I	S	R	R	Q	M	L	G	N	T
T	A	L	T	T	V	P	Q	A	M	D	I	S	R	R	Q	M	L	G	N	T
T	A	L	T	T	V	P	Q	A	M	D	I	S	C	R	Q	M	L	G	N	T
T	A	L	T	T	V	P	Q	A	M	D	I	S	R	R	Q	M	L	G	N	T
T	A	L	T	T	V	P	Q	A	M	D	I	S	R	R	Q	M	L	G	N	T
T	A	L	T	T	V	P	Q	A	M	D	I	S	R	R	Q	M	L	G	N	T
T	A	L	T	T	V	P	Q	A	M	D	I	S	R	R	Q	M	L	G	N	T
T	A	L	T	T	V	P	Q	A	M	D	I	S	R	R	Q	M	L	G	N	T
T	A	L	T	T	V	P	Q	A	M	D	I	S	R	R	Q	M	L	G	N	T
T	A	L	T	T	V	P	Q	A	M	D	I	S	R	R	Q	M	L	G	N	T
T	A	L	T	T	V	P	Q	A	M	D	I	S	R	R	Q	M	L	G	N	T
T	V	L	T	T	V	P	Q	A	M	D	I	S	R	R	Q	M	L	G	N	T
T	A	L	T	T	V	P	Q	A	M	D	I	S	R	R	Q	M	L	G	N	T
T	A	L	T	T	V	P	Q	A	M	D	I	S	R	R	Q	M	L	G	N	T
T	A	L	T	T	V	P	Q	A	M	D	I	S	R	R	Q	M	L	G	N	T
T	A	L	T	T	V	P	Q	A	M	D	I	S	R	R	Q	M	L	G	N	T
T	A	L	T	T	V	H	Q	A	M	D	I	S	R	R	Q	M	L	G	N	T
T	A	L	T	T	V	P	Q	A	M	D	I	S	R	R	Q	T	L	G	N	T
T	A	L	T	T	V	P	Q	A	M	D	I	S	R	R	Q	M	L	G	N	T
T	A	L	T	T	V	P	Q	A	M	D	I	S	R	R	Q	M	L	G	N	T
T	A	L	T	T	V	P	Q	A	M	D	I	S	R	R	Q	M	L	G	N	T
T	A	L	T	T	V	P	Q	A	M	D	I	S	R	R	Q	M	L	G	N	T
T	A	L	T	T	V	P	Q	A	M	D	I	S	R	R	Q	M	L	G	N	T
T	A	L	T	T	V	P	Q	A	M	D	I	S	R	R	Q	M	L	G	N	T
T	A	L	T	T	V	P	Q	A	M	D	I	S	R	R	Q	M	L	G	N	T
T	A	L	T	T	V	P	Q	A	M	D	I	S	R	R	Q	M	L	G	N	T
T	A	L	T	T	V	P	Q	A	M	D	I	S	R	R	Q	M	L	G	N	T
T	A	L	T	T	V	P	Q	A	I	D	I	S	R	R	Q	M	L	G	N	T
T	A	L	T	T	V	P	Q	A	M	D	I	S	R	R	Q	M	L	G	N	T
T	A	L	T	T	V	P	Q	A	M	D	I	S	R	R	Q	M	L	G	N	T
T	A	L	T	T	V	P	Q	A	M	D	I	S	R	R	Q	M	L	G	N	T
T	A	L	T	T	V	P	Q	A	I	D	I	S	R	R	Q	M	L	G	N	T
T	A	L	T	T	V	P	Q	A	M	D	I	S	R	R	Q	M	L	G	N	T
T	A	L	T	T	V	P	Q	A	M	D	I	S	R	R	Q	M	L	G	N	T
T	A	L	T	T	V	P	Q	A	M	D	I	S	R	R	Q	M	L	G	N	T
T	A	L	T	T	V	P	Q	A	M	D	I	S	R	R	Q	M	L	G	N	T
T	A	L	T	T	V	P	Q	A	M	D	I	S	R	R	Q	M	L	G	N	T
T	A	L	T	T	V	P	Q	A	M	D	I	S	R	R	Q	M	L	G	N	T
T	A	L	T	T	V	P	Q	A	M	D	I	S	C	R	Q	M	L	G	N	S
T	A	L	T	T	V	P	Q	A	M	D	I	S	R	R	Q	M	L	G	N	T
T	A	L	T	T	V	P	Q	A	M	D	I	S	C	R	Q	M	L	G	N	S
T	A	L	T	T	V	P	Q	A	M	D	I	S	R	R	K	M	L	G	N	T
T	A	L	T	T	V	P	Q	A	M	D	I	S	R	R	Q	M	L	G	N	T
T	A	L	T	T	V	P	Q	A	M	D	I	S	R	R	Q	M	L	G	N	T
T	A	L	T	T	V	P	Q	A	M	D	I	S	R	R	Q	M	L	G	N	T

T	A	L	T	T	V	P	Q	A	M	D	I	S	R	R	Q	M	L	G	N	T
T	A	L	T	T	V	P	Q	A	M	D	I	S	R	R	Q	M	L	G	N	T
T	A	L	T	T	V	P	L	A	M	D	I	S	R	R	Q	M	L	G	N	T
T	A	L	T	T	V	P	Q	A	M	D	I	S	R	R	Q	M	L	G	N	T
T	A	L	T	T	V	P	Q	A	M	D	I	S	R	R	Q	M	L	G	N	T
T	A	L	T	T	V	P	Q	A	M	D	I	S	R	R	Q	M	L	G	N	T
T	A	L	T	T	V	P	Q	A	M	D	I	S	R	R	Q	M	L	G	N	T
T	A	L	T	T	V	P	Q	A	M	D	I	S	R	R	Q	M	L	G	N	T
T	A	L	T	T	V	P	Q	A	M	D	I	S	R	R	Q	M	L	G	N	T
T	A	L	T	T	V	P	Q	A	M	D	I	S	R	R	Q	M	L	G	N	T
T	A	L	T	T	V	P	Q	A	M	D	I	S	R	R	Q	M	L	G	N	T
T	A	L	T	T	V	P	Q	A	M	D	I	S	R	R	Q	M	L	G	N	T
T	A	L	T	T	V	P	Q	A	M	D	I	S	R	C	Q	M	L	G	N	T
T	A	L	T	T	V	P	Q	A	M	D	I	S	R	R	Q	M	L	G	N	T
T	A	L	T	T	V	P	Q	A	M	D	I	S	R	R	Q	M	L	G	N	T
T	A	L	T	T	V	P	Q	A	M	D	I	S	R	R	Q	M	L	G	N	T
T	A	L	T	T	V	P	Q	A	M	D	I	S	R	R	Q	M	L	G	N	T
T	A	L	T	T	V	P	Q	A	M	D	I	S	R	R	Q	M	L	G	N	T
T	A	L	T	T	V	P	Q	A	M	D	I	S	R	R	Q	M	L	G	N	T
T	A	L	T	T	V	P	Q	A	M	D	I	S	R	R	Q	M	L	G	N	T
T	A	L	T	T	V	P	Q	A	M	D	I	S	R	R	Q	M	L	G	N	T
T	A	L	T	T	V	P	Q	A	M	D	I	S	R	R	Q	M	L	G	N	T
T	A	L	T	T	V	P	Q	A	M	D	I	S	R	R	Q	M	L	G	N	T
T	A	L	T	T	V	P	Q	A	M	D	I	S	R	R	Q	M	L	G	N	T
T	A	L	T	T	V	P	Q	A	M	D	I	S	R	R	Q	M	L	G	N	T
T	A	L	T	T	V	P	Q	A	M	D	I	S	R	R	Q	M	L	G	N	T
T	A	L	T	T	V	P	Q	A	M	D	I	S	R	R	Q	M	L	G	N	T
T	A	L	T	T	V	P	Q	A	M	D	I	S	R	R	Q	M	L	G	N	T
T	A	L	T	T	V	P	Q	A	M	D	I	S	R	R	Q	M	L	G	N	T
T	A	L	T	T	V	P	Q	A	M	D	I	S	R	R	Q	M	L	G	N	T
T	A	L	T	T	V	P	Q	A	M	D	I	S	R	R	Q	M	L	G	N	T
T	A	L	T	T	V	P	Q	A	M	D	I	S	R	R	Q	M	L	G	N	T
T	A	L	T	T	V	P	Q	A	M	D	I	S	R	R	Q	M	L	G	N	T
T	A	L	T	T	V	P	Q	A	M	D	I	S	R	R	Q	M	L	G	N	T
T	A	L	T	T	V	P	Q	A	M	D	I	S	R	R	Q	M	L	G	N	T
T	A	L	T	T	V	P	Q	A	M	D	I	S	R	R	Q	M	L	G	N	T
T	A	L	T	T	V	P	Q	A	M	D	I	S	R	R	Q	M	L	G	N	T
T	A	L	T	T	V	P	Q	A	M	D	I	S	R	R	Q	M	L	G	N	T
T	A	L	T	T	V	P	Q	A	M	D	I	S	R	R	Q	M	L	G	N	T
T	A	L	T	T	V	P	Q	A	M	D	I	S	R	R	Q	M	L	G	N	T
T	A	L	T	T	V	P	Q	A	M	D	I	S	R	R	Q	M	L	G	N	T
T	A	L	T	T	V	P	Q	A	M	D	I	S	R	R	Q	M	L	G	N	T
T	A	L	T	T	V	S	Q	A	M	D	I	S	R	R	Q	M	L	G	N	T
T	A	L	T	T	V	P	Q	A	M	D	I	S	R	R	Q	M	L	G	N	T
T	A	L	T	T	V	P	Q	A	M	D	I	S	R	R	Q	M	L	G	N	T
T	A	L	T	T	V	P	Q	A	M	D	I	S	R	R	Q	M	L	G	N	T
T	A	L	T	T	V	P	Q	A	M	D	I	S	R	R	Q	M	L	G	N	T
T	A	L	T	T	V	P	Q	A	M	D	I	S	R	R	Q	M	L	G	N	T
T	A	L	T	I	V	P	Q	A	M	D	I	S	R	R	Q	M	L	G	N	T
T	A	L	T	I	V	P	Q	A	M	D	I	S	R	R	Q	M	L	G	N	T
T	A	L	T	T	V	P	Q	A	M	D	I	S	R	R	Q	M	L	G	N	T
T	A	L	T	T	V	P	Q	A	M	D	I	S	R	R	Q	M	L	G	N	T
T	A	L	T	T	V	P	Q	A	M	D	I	S	R	R	Q	M	L	G	N	T
T	A	L	T	T	V	P	Q	A	M	D	I	S	R	R	Q	M	L	G	N	T
T	A	L	T	T	V	P	Q	A	M	Y	I	S	R	R	Q	M	L	G	N	T
T	A	L	T	T	V	P	Q	A	M	D	I	S	R	R	Q	M	L	G	N	T
T	A	L	T	T	V	P	Q	A	M	D	I	S	R	R	Q	M	L	G	N	T
T	A	L	T	T	V	P	Q	A	M	D	I	S	R	R	Q	M	L	G	N	T
T	A	L	T	T	V	P	Q	A	M	D	I	S	R	R	Q	M	L	G	N	T
T	A	L	T	T	V	P	Q	A	M	D	I	S	R	R	Q	M	L	G	N	T
T	A	L	T	T	V	P	Q	A	M	D	I	S	R	R	Q	M	L	G	N	T
T	A	L	T	T	V	P	Q	A	M	D	I	S	R	R	Q	M	L	G	N	T
T	A	L	T	T	V	P	Q	A	M	D	I	S	R	R	Q	M	L	G	N	T
T	A	L	T	T	V	P	Q	A	M	D	I	S	R	R	Q	M	L	G	N	T
T	A	L	T	T	V	P	Q	A	M	D	I	S	R	R	Q	M	L	D	N	T
T	A	L	T	T	V	P	Q	A	M	D	I	S	R	R	Q	M	L	G	N	T
T	A	L	T	T	V	P	Q	A	M	D	I	S	R	R	Q	M	L	G	N	T

T	A	L	T	T	V	P	Q	A	M	D	I	S	R	R	Q	M	L	G	N	T
T	A	L	T	T	V	P	Q	A	M	D	I	S	R	R	Q	M	L	G	N	T
T	A	L	T	T	V	P	Q	A	M	D	I	S	R	R	Q	M	L	G	N	T
T	A	L	T	T	V	P	Q	A	M	D	I	S	R	R	Q	M	L	G	N	T
T	A	L	T	T	V	P	Q	A	M	D	I	S	R	R	Q	M	L	G	N	T
T	A	L	T	T	V	P	Q	A	M	D	I	S	R	R	Q	M	L	G	N	T
T	A	L	T	T	V	P	Q	V	M	D	I	S	R	R	Q	M	L	G	N	T
T	A	L	T	T	V	P	Q	A	M	D	I	S	R	R	Q	M	L	G	N	T
T	A	L	T	T	V	P	Q	A	M	D	I	S	R	R	Q	M	L	G	N	T
T	A	L	T	T	V	P	Q	A	M	D	I	S	R	R	Q	M	L	G	N	T
T	A	L	T	T	V	P	Q	A	M	D	I	S	R	R	Q	M	L	G	N	T
T	A	L	T	T	V	P	Q	A	M	D	I	S	R	R	Q	M	L	G	N	T
T	A	L	T	T	V	P	Q	A	M	D	I	S	R	R	Q	M	L	G	N	T
T	A	L	T	T	V	P	Q	A	M	D	I	S	R	R	Q	M	L	G	N	T
T	A	L	T	T	V	P	Q	A	M	D	I	S	R	R	Q	M	L	G	N	T
T	A	L	T	T	V	P	Q	A	M	D	I	S	R	R	Q	M	L	G	N	T
T	A	L	T	T	V	P	Q	A	M	D	I	S	R	R	Q	M	L	G	N	T
T	A	L	T	T	V	P	Q	A	M	D	I	S	R	R	Q	M	L	G	N	T
T	A	L	T	T	V	P	Q	A	M	D	I	S	R	R	Q	M	L	G	N	T
T	A	L	T	T	V	P	Q	A	M	D	I	S	R	R	Q	M	L	G	N	T
T	A	L	T	T	V	P	Q	A	M	D	I	S	R	R	Q	M	L	G	N	T
T	A	L	T	T	V	P	Q	A	M	D	I	S	R	R	Q	M	L	G	N	T
T	A	L	T	T	V	P	Q	A	M	D	I	S	R	R	Q	M	L	G	N	T
T	A	L	T	T	V	P	Q	A	M	D	I	S	R	R	Q	M	L	G	N	T
T	A	L	T	T	V	P	Q	A	M	D	I	S	R	R	Q	M	L	G	N	T
T	A	L	T	T	V	P	Q	A	M	D	I	S	R	R	Q	M	L	G	N	T
T	A	L	T	T	V	P	Q	A	M	D	I	S	R	R	Q	M	L	G	N	T
T	A	L	T	T	V	P	Q	A	M	D	I	S	R	R	Q	M	L	G	N	T
T	A	L	T	T	V	P	Q	A	M	D	I	S	R	R	Q	M	L	G	N	T
T	A	L	T	T	V	P	Q	A	M	D	I	S	R	R	Q	M	L	G	N	T
T	A	L	T	T	V	P	Q	A	M	D	I	S	R	R	Q	M	L	G	N	T
T	A	L	T	T	V	P	Q	A	M	D	I	S	R	R	Q	M	L	G	N	T
T	A	L	T	T	V	P	Q	A	M	D	I	S	R	R	Q	M	L	G	N	T
T	A	L	T	T	V	P	Q	A	M	D	I	S	R	R	Q	M	L	G	N	T
T	A	L	T	T	V	P	Q	A	M	D	I	S	R	R	Q	M	L	G	N	T
T	A	L	T	T	V	P	Q	A	M	D	I	S	R	R	Q	M	L	G	N	T
T	A	L	T	T	V	P	Q	A	M	D	I	S	R	R	Q	M	L	G	N	T
T	A	L	T	T	V	P	Q	A	M	D	I	S	R	R	Q	M	L	G	N	T
T	A	L	T	T	V	P	Q	A	M	D	I	S	R	R	Q	M	L	G	N	T
T	A	L	T	T	V	P	Q	A	M	D	I	S	R	R	Q	M	L	G	N	T
T	A	L	T	T	V	P	Q	A	M	D	I	S	R	R	Q	M	L	G	N	T
T	A	L	T	T	V	P	Q	A	M	D	I	S	R	R	Q	M	L	G	N	T
T	A	L	T	T	V	P	Q	A	M	D	I	S	R	R	Q	M	L	G	N	T
T	A	L	T	T	V	P	Q	A	M	D	I	S	R	R	Q	M	L	G	N	T
T	A	L	T	T	V	P	Q	A	M	D	I	S	R	R	Q	M	L	G	N	T
T	A	L	T	T	V	P	Q	A	M	D	I	S	R	R	Q	M	L	G	N	T
T	A	L	T	T	V	P	Q	A	M	D	I	S	R	R	Q	M	L	G	N	T
T	A	L	T	T	V	P	Q	A	M	D	I	S	R	R	Q	M	L	G	N	T
T	A	L	T	T	V	P	Q	A	M	D	I	S	R	R	Q	M	L	G	N	T
T	A	L	T	T	V	P	Q	A	M	D	I	S	R	R	Q	M	L	G	N	T
T	A	L	T	T	V	P	Q	A	M	D	I	S	R	R	Q	M	L	G	N	T
T	A	L	T	T	V	P	Q	A	M	D	I	S	R	R	Q	M	L	G	N	T
T	A	L	T	T	V	P	Q	A	M	D	I	S	R	R	Q	M	L	G	N	T
T	A	L	T	T	V	P	Q	A	M	D	I	S	R	R	Q	M	L	G	N	T
T	A	L	T	T	V	P	Q	A	M	D	I	S	R	R	Q	M	L	G	N	T
T	A	L	T	T	V	P	Q	A	M	D	I	S	R	R	Q	M	L	G	N	T
T	A	L	T	T	V	P	Q	A	M	D	I	S	R	R	Q	M	L	G	N	T
T	A	L	T	T	V	P	Q	A	M	D	I	S	R	R	Q	M	L	G	N	T
T	A	L	T	T	V	P	Q	A	M	D	I	S	R	R	Q	M	L	G	N	T
T	A	L	T	T	V	P	Q	A	M	D	I	S	R	R	Q	M	L	G	N	T
T	A	L	T	T	V	P	Q	A	M	D	I	S	R	R	Q	M	L	G	N	T
T	A	L	T	T	V	P	Q	A	M	D	I	S	R	R	Q	M	L	G	N	T
T	A	L	T	T	V	P	Q	A	M	D	I	S	R	R	Q	M	L	G	N	T
T	A	L	T	T	V	P	Q	A	V	D	I	S	R	R	Q	M	L	G	N	T
T	A	L	T	T	V	P	Q	A	V	D	I	S	R	R	Q	M	L	G	N	T
T	A	L	T	T	V	P	Q	A	V	D	I	S	R	R	Q	M	L	G	N	T
T	A	L	T	T	V	P	Q	A	M	D	I	S	R	R	Q	M	L	G	N	T

T	A	L	T	T	V	P	Q	A	M	D	I	S	R	R	Q	M	L	G	N	T
T	A	L	T	T	L	P	Q	A	M	D	I	S	R	R	Q	M	L	G	N	T
T	A	L	T	T	L	P	Q	A	M	D	I	S	R	R	Q	M	L	G	N	T
T	A	L	T	T	L	P	Q	A	M	D	I	S	R	R	Q	M	L	G	N	T
T	A	L	T	T	V	P	Q	A	M	D	I	S	R	R	Q	M	L	G	N	T
T	A	L	T	T	L	P	Q	A	M	D	I	S	R	R	Q	M	L	G	N	T
T	A	L	T	T	V	P	Q	A	M	D	I	S	R	R	Q	M	L	G	N	T
T	A	L	T	T	V	P	Q	A	M	D	I	S	R	R	Q	M	L	G	N	T
T	A	L	T	T	V	P	Q	A	M	D	I	S	R	R	Q	M	L	G	N	T
T	A	L	T	T	V	P	Q	A	M	D	I	S	R	R	Q	M	L	G	N	T
T	A	L	I	T	L	P	Q	A	M	D	I	S	R	R	Q	M	L	G	N	T
T	A	L	T	T	L	P	Q	A	M	D	I	S	R	R	Q	M	L	G	N	T
T	A	L	T	T	L	P	Q	A	M	D	I	S	R	R	Q	M	L	G	N	T
T	A	L	T	T	L	P	Q	A	M	D	I	S	R	R	Q	M	L	G	N	T
T	A	L	T	T	L	P	Q	A	M	D	I	S	R	R	Q	M	L	G	N	T
T	A	L	T	T	V	P	Q	A	M	D	I	S	R	R	Q	M	L	G	N	T
T	A	L	T	T	V	P	Q	A	M	D	I	S	R	R	Q	M	L	G	N	T
T	A	L	T	T	V	P	Q	A	M	D	I	S	R	R	Q	M	L	G	N	T

wze																			wchB	wc
52	62	111	121	153	155	162	172	177	179	180	186	187	197	198	211	212	217	221	269	128
T	R	I	S	T	P	A	S	A	G	E	V	Q	G	E	S	V	V	K	H	A
T	R	I	S	T	P	A	S	A	D	E	V	Q	G	K	S	V	V	K	H	A
T	R	I	S	T	P	A	S	A	G	E	V	Q	G	K	S	V	V	K	H	A
T	R	I	S	T	P	A	S	A	G	E	V	Q	G	K	S	V	V	K	H	A
T	R	I	S	T	P	A	S	A	G	E	V	Q	G	K	S	V	V	K	H	A
T	R	I	S	T	P	A	S	A	G	E	V	Q	G	K	S	V	V	K	H	A
T	R	I	S	T	P	A	S	A	G	E	V	Q	G	K	S	V	V	K	H	A
T	R	I	S	T	P	A	S	A	G	E	V	Q	G	K	S	V	V	K	H	A
T	R	I	S	T	P	A	S	A	G	E	V	Q	G	K	S	V	V	K	H	A
T	R	I	S	T	P	A	S	A	G	E	V	Q	G	K	S	V	V	K	H	A
T	R	I	S	T	P	A	S	A	G	E	V	Q	G	K	S	V	V	K	H	A
T	R	I	S	T	P	A	S	A	G	E	V	Q	G	K	S	V	V	K	H	A
T	R	I	S	T	P	A	S	A	G	E	V	Q	G	K	S	V	V	K	H	A
T	R	I	S	T	P	A	S	A	G	E	V	Q	G	K	S	V	V	K	H	A
T	R	I	S	T	P	A	S	A	G	E	V	Q	G	K	S	V	V	K	H	A
T	R	I	S	T	P	A	S	A	G	E	V	Q	G	K	S	V	V	K	H	A
T	R	I	S	T	P	A	S	A	G	E	V	Q	G	K	S	V	V	K	H	A
T	R	I	S	T	P	A	S	A	G	E	V	Q	G	K	S	V	V	K	H	A
T	R	I	S	T	P	A	S	A	G	E	V	Q	G	K	S	V	V	K	H	A
T	R	I	S	T	P	A	S	A	G	E	V	Q	G	K	S	V	V	K	H	A
T	R	I	S	T	P	A	S	A	G	E	V	Q	G	K	S	V	V	K	H	A
T	R	I	S	T	P	A	S	A	G	E	V	Q	G	K	S	V	V	K	H	A
T	R	I	S	T	P	A	S	A	G	E	V	Q	G	K	S	V	V	K	H	A
T	R	I	S	T	P	A	S	A	G	E	V	Q	G	K	S	V	V	K	H	A
T	R	I	S	T	P	A	S	A	G	E	V	Q	G	K	S	V	V	K	H	A
T	R	I	S	T	P	A	S	A	G	E	V	Q	G	K	S	V	V	K	H	A
T	R	I	S	T	P	A	S	A	G	E	V	Q	G	K	S	V	V	K	H	A
T	R	I	S	T	P	A	S	A	G	E	V	Q	G	K	S	V	V	K	H	A
T	R	I	S	T	P	A	S	A	G	E	V	Q	G	K	S	V	V	K	H	A
T	R	I	S	T	P	A	S	A	G	E	V	Q	G	K	S	V	V	K	H	A
T	R	I	S	T	P	A	S	A	G	E	V	Q	G	K	S	V	V	K	H	A
T	R	I	S	T	P	A	S	A	G	E	V	Q	G	K	S	V	V	K	H	A
T	R	I	S	T	P	A	S	A	G	E	V	Q	G	K	S	V	V	K	H	A
T	R	I	S	T	P	A	S	A	G	E	V	Q	G	K	S	V	V	K	H	A
T	R	I	S	T	P	A	S	A	G	E	V	Q	G	K	S	V	V	K	H	A
T	R	I	S	T	P	A	S	A	G	E	V	Q	G	K	S	V	V	K	H	A
T	R	I	S	T	P	A	S	A	G	E	V	Q	G	K	S	V	V	K	H	A
T	R	I	S	T	P	A	S	A	G	E	V	Q	G	K	S	V	V	K	H	A
T	R	I	S	T	P	A	S	A	G	E	V	Q	G	K	S	V	V	K	H	A
T	R	I	S	T	P	A	S	A	G	E	V	Q	G	K	S	V	V	K	H	A
T	R	I	S	T	P	A	S	A	G	E	V	Q	G	K	S	V	V	K	H	A
T	R	I	S	T	P	A	S	A	G	E	V	Q	G	K	S	V	V	K	H	A
T	R	I	S	T	P	A	S	A	G	E	V	Q	G	K	S	V	V	K	H	A
T	R	I	S	T	P	A	S	A	G	E	V	Q	G	K	S	V	V	K	H	A
T	R	I	S	T	P	A	S	A	G	E	V	Q	G	K	S	V	V	K	H	A
T	R	I	S	T	P	A	S	A	G	E	V	Q	G	K	S	V	V	K	H	A
T	R	I	S	T	P	A	S	A	G	E	V	Q	G	K	S	V	V	K	H	A
T	R	I	S	T	P	A	S	A	G	E	V	Q	G	K	S	V	V	K	H	A
T	R	I	S	T	P	A	S	A	G	E	V	Q	G	K	S	V	V	K	H	A
T	R	V	S	T	P	A	S	A	G	E	V	Q	G	K	S	V	V	K	H	A
T	R	I	S	T	P	A	S	A	G	E	V	Q	G	K	S	V	V	K	H	A
T	R	I	S	T	P	A	S	A	G	E	V	Q	G	K	S	V	V	K	H	A
T	R	I	S	T	P	A	S	A	G	E	V	Q	G	K	S	V	V	K	H	A
T	R	I	S	T	P	A	S	A	G	E	V	Q	G	K	S	V	V	K	H	A
T	R	I	S	T	P	A	S	A	G	E	V	Q	G	K	S	V	V	K	H	A
T	R	I	S	T	P	A	S	A	G	E	V	Q	G	K	S	V	V	K	H	A
T	R	I	S	T	P	A	S	A	G	E	V	Q	G	K	S	V	V	K	H	A
T	R	I	S	T	P	A	S	A	G	E	V	Q	G	K	S	V	V	K	H	A
T	R	I	S	T	P	A	S	A	G	E	V	Q	G	K	S	V	V	K	H	A
T	R	V	S	T	P	A	S	A	G	E	V	Q	G	K	S	V	V	K	H	A
T	R	I	S	T	P	A	S	A	G	E	V	Q	G	K	S	V	V	K	H	A
T	R	I	S	T	P	A	S	A	G	E	V	Q	G	K	S	V	V	K	H	A
T	R	I	S	T	P	A	S	A	G	E	V	Q	G	K	S	V	V	K	H	A

T	R	I	S	T	P	A	S	A	G	E	V	Q	G	K	S	V	V	K	H	A
T	R	I	S	T	P	A	S	A	G	E	V	Q	G	K	S	V	V	K	H	A
T	R	I	S	T	P	A	S	A	G	E	V	Q	G	K	S	V	V	K	H	A
T	R	I	S	T	P	A	S	A	G	E	V	Q	G	K	S	V	V	K	H	A
T	R	I	S	T	P	A	S	A	G	E	V	Q	G	K	S	V	V	K	H	A
T	R	I	S	T	P	A	S	A	G	E	V	Q	G	K	S	V	V	K	H	A
T	R	I	S	T	P	A	S	A	G	E	V	Q	G	K	S	V	V	K	H	A
T	R	I	S	T	P	A	S	A	G	E	V	Q	G	K	S	V	V	K	H	A
T	R	I	S	T	P	A	S	A	G	E	V	Q	G	K	S	V	V	K	H	A
T	R	I	S	T	P	A	S	A	G	E	V	Q	G	K	S	V	V	K	H	A
T	R	I	S	T	P	A	S	A	G	E	V	Q	G	K	S	V	V	K	H	A
T	R	I	S	T	P	A	S	A	G	E	V	Q	G	K	S	V	V	K	H	A
T	R	I	S	T	P	A	S	A	G	E	V	Q	G	K	S	V	V	K	H	A
T	R	I	S	T	P	A	S	A	G	E	V	Q	G	K	S	V	V	K	H	A
T	R	I	S	T	P	A	S	A	G	E	V	Q	G	K	S	V	V	K	H	A
T	R	I	S	T	P	A	S	A	G	E	V	Q	G	K	S	A	V	K	H	A
T	R	I	S	A	P	A	S	A	G	E	V	Q	G	K	P	V	V	R	H	A
T	R	I	S	A	P	A	S	A	G	E	V	Q	G	K	P	V	V	R	H	A
T	R	I	S	A	P	A	S	A	G	E	V	Q	G	K	P	V	V	R	H	A
T	R	I	S	A	P	A	S	A	G	E	V	Q	G	K	P	V	V	R	H	A
T	R	I	S	T	P	A	S	A	G	E	V	Q	G	K	S	A	V	K	H	A
T	R	I	S	T	P	A	S	A	G	E	V	Q	G	K	S	V	V	K	H	A
T	R	I	S	T	P	A	S	A	G	E	V	Q	G	K	S	V	V	K	H	A
T	R	I	S	T	P	A	S	A	G	E	V	Q	G	K	S	V	V	K	H	A
T	R	I	S	T	P	A	S	A	G	E	V	Q	G	K	S	V	V	K	H	A
T	R	I	S	T	P	A	S	A	G	E	V	Q	G	K	S	V	V	K	H	A
T	R	I	S	T	P	A	S	A	G	E	V	Q	G	K	S	V	V	K	H	A
T	R	I	S	T	P	A	S	A	G	E	V	Q	G	K	S	V	V	K	H	A
T	R	I	S	T	P	A	S	A	G	E	V	Q	G	K	S	V	V	K	H	A
T	R	I	S	T	P	A	S	A	G	E	V	Q	G	K	S	V	V	K	H	A
T	R	I	S	T	P	A	S	A	G	E	V	Q	G	K	S	V	V	K	H	A
T	R	I	S	T	P	A	S	A	G	E	V	Q	G	K	S	V	V	K	H	A
T	R	I	S	T	P	A	S	A	G	D	V	Q	G	K	S	V	V	K	Y	A
T	R	I	S	T	P	A	S	A	G	E	V	Q	G	K	S	V	V	K	H	A
T	R	I	S	T	P	A	S	A	G	E	V	Q	G	K	S	V	V	K	H	A
T	R	I	S	T	P	A	S	A	G	E	V	Q	G	K	S	V	V	K	H	A
T	R	I	S	T	P	A	S	A	G	E	V	Q	G	K	S	V	V	K	H	A
T	R	I	S	T	P	A	S	A	G	E	V	Q	G	K	S	V	V	K	H	A
T	R	I	S	T	P	A	S	A	G	E	V	Q	G	K	S	V	V	K	H	A
T	R	I	S	T	P	A	S	A	G	E	V	Q	G	K	S	V	V	K	H	A
T	R	I	S	T	P	A	S	A	G	D	V	Q	G	K	S	V	V	K	Y	A
T	R	I	S	T	P	A	S	A	G	E	V	Q	G	K	S	V	V	K	H	A
T	R	I	S	T	P	A	S	A	G	E	V	Q	G	K	S	V	V	K	H	A
T	R	I	S	T	P	A	S	A	G	E	V	Q	G	K	S	V	V	K	H	A
T	R	I	S	T	P	A	S	A	G	E	V	Q	G	K	S	V	V	K	H	A
T	R	I	S	T	P	A	S	A	G	E	V	Q	G	K	S	V	V	K	H	A
T	R	I	S	T	P	A	S	A	G	E	V	Q	G	K	S	V	V	K	H	A
T	R	I	S	T	P	A	S	A	G	E	V	Q	G	K	S	V	V	K	H	A
T	R	I	S	T	P	A	S	A	G	D	V	Q	G	K	S	V	V	K	Y	A
T	R	I	S	T	P	A	S	A	G	E	V	Q	G	K	S	V	V	K	H	A
T	R	I	S	T	P	A	S	A	G	E	V	Q	G	K	S	V	V	K	H	A
T	R	I	S	T	P	A	S	A	G	E	V	Q	G	K	S	V	V	K	H	A
T	R	I	S	T	P	A	S	A	G	E	V	Q	G	K	S	V	V	K	H	A
T	R	I	S	T	P	A	S	A	G	E	V	Q	G	K	S	V	V	K	H	A
T	R	I	S	T	P	A	S	A	G	E	V	Q	G	K	S	V	V	K	H	A
T	R	I	S	T	P	A	S	A	G	E	V	Q	G	K	S	V	V	K	H	A
T	R	I	S	T	P	A	S	A	G	E	V	Q	G	K	S	V	V	K	H	A
T	R	I	S	T	P	A	S	A	G	E	V	Q	G	K	S	V	V	K	H	A
T	R	I	S	T	P	A	S	A	G	E	V	Q	G	K	S	V	V	K	H	A
T	R	I	S	T	P	A	S	A	G	E	V	Q	G	K	S	V	V	K	H	A
T	R	I	S	T	P	A	S	A	G	E	V	Q	G	K	S	V	V	K	H	A
T	R	I	S	T	P	A	S	A	G	D	V	Q	G	K	S	V	V	K	H	A
T	R	I	S	T	P	A	S	A	G	E	V	Q	G	K	S	V	V	K	H	A
T	R	I	S	T	P	A	S	A	G	E	V	Q	G	K	S	V	V	K	H	A
T	R	I	S	T	P	A	S	A	G	E	V	Q	G	K	S	V	V	K	H	A
T	R	I	S	T	P	A	S	A	G	E	V	Q	G	K	S	V	V	K	H	A
T	R	I	S	T	P	A	S	A	G	E	V	Q	G	K	S	V	V	K	H	A

T	R	I	S	T	P	A	S	A	G	E	V	Q	G	K	S	V	V	K	H	A
T	R	I	S	T	P	A	S	A	G	E	V	Q	G	K	S	V	V	K	H	A
T	R	I	S	T	P	A	S	A	G	E	V	Q	G	K	S	V	V	K	H	A
T	R	I	S	T	P	A	S	A	G	E	V	Q	G	K	S	V	V	K	H	A
T	R	I	S	T	P	A	S	A	G	E	V	Q	G	K	S	V	V	K	H	A
T	R	I	S	T	P	A	S	A	G	E	V	Q	G	K	S	V	V	K	H	A
T	R	I	S	T	P	A	S	A	G	E	V	Q	G	K	S	V	V	K	H	A
T	R	I	S	T	P	A	S	A	G	E	V	Q	G	K	S	V	V	K	H	A
T	R	I	S	T	P	A	S	A	G	E	V	Q	G	K	S	V	V	K	H	A
T	R	I	S	T	P	A	S	A	G	E	V	Q	G	K	S	V	V	K	H	A
T	R	I	S	T	P	A	S	A	G	E	V	Q	G	K	S	V	V	K	H	A
T	R	I	S	T	P	A	S	A	G	E	V	Q	G	K	S	V	V	K	H	A
T	R	I	S	T	P	A	S	A	G	D	V	Q	G	K	S	V	V	K	H	A
T	R	I	S	T	P	A	S	A	G	E	V	Q	G	K	S	V	V	K	H	A
T	R	I	S	T	P	A	S	A	G	E	V	Q	G	K	S	V	V	K	H	A
T	R	I	S	T	P	A	S	A	G	E	V	Q	G	K	S	V	V	K	H	A
T	R	I	S	T	P	T	S	A	G	E	V	Q	G	K	S	V	V	K	H	A
T	R	I	S	T	P	A	S	A	G	E	I	Q	G	K	S	V	V	K	H	A
T	R	I	S	T	P	A	S	A	G	E	V	Q	G	K	S	V	V	K	H	A
T	R	I	S	T	P	A	S	A	G	E	V	Q	G	K	S	V	V	K	H	A
T	R	I	S	T	P	A	S	A	G	E	V	Q	G	K	S	V	V	K	H	A
T	R	I	S	T	P	A	S	A	G	E	V	Q	G	K	S	V	V	K	H	A
T	R	I	S	T	P	A	S	A	G	E	V	Q	G	K	S	V	V	K	H	A
T	R	I	S	T	P	A	S	A	G	E	V	Q	G	K	S	V	V	K	H	A
T	R	I	S	T	P	A	S	A	G	E	V	Q	G	K	S	V	V	K	H	A
T	R	I	S	T	P	A	S	A	G	E	V	Q	G	K	S	V	V	K	H	A
T	R	I	S	T	P	A	S	A	G	E	V	Q	G	K	S	V	V	K	H	A
T	R	I	S	T	P	A	S	A	G	E	V	Q	G	K	S	V	V	K	H	A
T	R	I	S	T	P	A	S	A	G	E	V	Q	G	K	S	V	V	K	H	A
I	R	I	S	T	P	A	S	A	G	E	V	Q	G	N	S	V	V	K	H	A
T	R	I	S	T	P	A	S	A	G	E	V	Q	G	K	S	V	V	K	H	A
T	R	I	S	T	P	A	S	A	G	E	V	Q	G	K	S	V	V	K	H	A
T	R	I	S	T	P	A	S	A	G	E	V	Q	G	K	S	V	V	K	H	A
T	R	I	S	T	P	A	S	A	G	E	V	Q	G	K	S	V	V	K	H	A
T	R	I	S	T	P	A	S	A	G	E	V	Q	G	K	S	V	D	K	H	A
T	R	I	S	T	P	A	S	A	G	E	V	Q	G	K	S	V	V	K	H	A
T	R	I	S	T	P	A	S	A	G	E	V	Q	G	K	S	V	V	K	H	A
T	R	I	S	T	P	A	S	A	G	E	V	Q	G	K	S	V	V	K	H	A
T	R	I	S	T	P	A	S	S	G	E	V	Q	G	K	S	V	V	K	H	A
T	R	I	S	T	P	A	S	A	G	E	V	Q	G	K	S	V	V	K	H	A
T	R	I	S	T	P	A	S	A	G	E	V	Q	G	K	S	V	V	K	H	A
T	R	I	S	T	P	A	S	A	G	E	V	Q	G	K	S	V	V	K	H	A
T	R	I	S	T	P	A	S	A	G	E	V	Q	G	K	S	V	V	K	H	A
T	R	I	S	T	P	A	S	A	G	E	V	Q	G	K	S	V	V	K	H	A
T	R	I	S	T	P	A	S	A	G	E	V	Q	G	K	S	V	V	K	H	A
T	R	I	S	T	P	A	S	A	G	E	V	Q	G	K	S	V	V	K	H	A
T	R	I	S	T	P	A	S	A	G	E	V	Q	G	K	S	V	V	K	H	A
T	R	I	T	T	P	A	S	A	G	E	V	Q	G	K	S	V	V	K	H	A
T	R	I	S	T	P	A	S	A	G	E	V	Q	G	K	S	V	V	K	H	A
T	R	I	S	T	P	A	S	A	G	E	V	Q	E	K	S	V	V	K	H	A
T	R	I	S	T	P	A	S	A	G	E	V	Q	G	K	S	V	V	K	H	A
T	R	I	S	T	P	A	S	A	G	E	V	Q	G	K	S	V	V	K	H	A
T	R	I	S	T	P	A	S	A	G	E	V	Q	G	K	S	V	V	K	H	A
T	R	I	S	T	P	A	S	A	G	E	V	Q	E	K	S	V	V	K	H	A
T	R	I	S	T	P	A	S	A	G	E	V	Q	G	K	S	V	V	K	H	A
T	R	I	S	T	P	A	S	A	G	E	V	Q	G	K	S	V	V	K	H	A
T	R	I	S	T	P	A	S	A	G	E	V	Q	G	K	S	V	V	K	H	A
T	R	I	S	T	P	A	S	S	G	E	V	Q	G	K	S	V	V	K	H	A
T	R	I	S	T	P	A	S	A	G	E	V	Q	G	K	S	V	V	K	H	A
T	R	I	S	T	P	A	S	A	G	E	V	Q	G	K	S	V	V	K	H	V
T	R	I	S	T	P	A	T	A	G	E	V	Q	G	K	S	V	V	K	H	A
T	R	I	S	T	P	A	S	A	G	E	V	Q	G	K	S	V	V	K	H	A
T	R	I	S	T	P	A	T	A	G	E	V	Q	G	K	S	V	V	K	H	A
T	L	I	S	T	P	A	S	A	G	E	V	Q	G	K	S	V	V	K	H	A
T	R	I	S	T	P	A	S	A	G	E	V	Q	G	K	S	V	V	K	H	A
T	R	I	S	T	P	A	S	A	G	E	V	Q	G	K	S	V	V	K	H	A
T	R	I	S	T	P	A	S	A	G	E	V	Q	G	K	S	V	V	K	H	A

T	R	I	S	T	P	A	S	A	G	E	V	Q	G	K	S	V	V	K	H	A
T	R	I	S	T	P	A	S	A	G	E	V	Q	G	K	S	V	V	K	H	A
T	R	I	S	T	P	A	S	A	G	E	V	Q	G	K	S	V	V	K	H	A
T	R	I	S	T	P	A	S	A	G	E	V	Q	G	K	S	V	V	K	H	A
T	R	I	S	T	P	A	S	A	G	E	V	Q	G	K	S	V	V	K	H	A
T	R	I	S	T	P	A	S	A	G	E	V	Q	G	K	S	V	V	K	H	A
T	R	I	S	T	P	A	S	A	G	E	V	Q	G	K	S	V	V	K	H	A
T	R	I	S	T	P	A	S	A	G	E	V	Q	G	K	S	V	V	K	H	A
T	R	I	S	T	P	A	S	A	G	E	V	Q	G	K	S	V	V	K	H	A
T	R	I	S	T	P	A	S	A	G	E	V	Q	G	K	S	V	V	K	H	A
T	R	I	S	T	P	A	S	A	G	E	V	Q	G	K	S	V	V	K	H	A
T	R	I	S	T	P	A	S	A	G	E	V	Q	G	K	S	V	V	K	H	A
T	R	I	S	T	P	A	S	A	G	E	V	Q	G	K	S	V	V	K	H	A
T	R	I	S	T	P	A	S	A	G	E	V	Q	G	K	S	V	V	K	H	A
T	R	I	S	T	P	A	S	A	G	E	V	Q	G	K	S	V	V	K	H	A
T	R	I	S	T	P	A	S	A	G	E	V	Q	G	K	S	V	V	K	H	A
T	R	I	S	T	P	A	S	A	G	E	V	Q	G	K	S	V	V	K	H	A
T	R	I	S	T	P	A	S	A	G	E	V	Q	G	K	S	V	V	K	H	A
T	R	I	S	T	S	A	S	A	G	E	V	Q	G	K	S	V	V	K	H	A
T	R	I	S	T	P	A	S	A	G	E	V	Q	G	K	S	V	V	K	H	A
T	R	I	S	T	P	A	S	A	G	E	V	Q	G	K	S	V	V	K	H	A
T	R	I	S	T	P	A	S	A	G	E	V	Q	G	K	S	V	V	K	H	A
T	R	I	S	T	P	A	S	A	G	E	V	Q	G	K	S	V	V	K	H	A
T	R	I	S	T	P	A	S	A	G	E	V	Q	G	K	S	V	V	K	H	A
T	R	I	S	T	P	A	S	A	G	E	I	Q	G	K	S	V	V	K	H	A
T	R	I	S	T	P	A	S	A	G	E	V	Q	G	K	S	V	V	K	H	A
T	R	I	S	T	P	A	S	A	G	E	V	Q	G	K	S	V	V	K	H	A
T	R	I	S	T	P	A	S	A	G	E	V	Q	G	K	S	V	V	K	H	A
T	R	I	S	T	P	A	S	A	G	E	I	Q	G	K	S	V	V	K	H	A
T	R	I	S	T	P	A	S	A	G	E	V	Q	G	K	S	V	V	K	H	A
T	R	I	S	T	P	A	S	A	G	E	I	Q	G	K	S	V	V	K	H	A
T	R	I	S	T	P	A	S	A	G	E	V	Q	G	K	S	V	V	K	H	A
T	R	I	S	T	P	A	S	A	G	E	V	Q	G	K	S	V	V	K	H	A
T	R	I	S	T	P	A	S	A	G	E	V	Q	G	K	S	V	V	K	H	A
T	R	I	S	T	P	A	S	A	G	E	V	Q	G	K	S	V	V	K	H	A
T	R	I	S	T	P	A	S	A	G	E	V	Q	G	K	S	V	V	K	H	A
T	R	I	S	T	P	A	S	A	G	E	V	Q	G	K	S	V	V	K	H	A
T	R	I	S	T	P	A	S	A	G	E	V	Q	G	K	S	V	V	K	H	A
T	R	I	S	T	P	A	S	A	G	E	V	Q	G	K	S	V	V	K	H	A
T	R	I	S	T	P	A	S	A	G	E	V	Q	G	K	S	V	V	K	H	A
T	R	I	S	T	P	A	S	A	G	E	V	Q	G	K	S	V	V	K	H	A
T	R	I	S	T	P	A	S	A	G	E	V	Q	G	K	S	V	V	K	H	A
T	R	I	S	T	P	A	S	A	G	E	V	Q	G	K	S	V	V	K	H	A
T	R	I	S	T	P	A	S	A	G	E	V	Q	G	K	S	V	V	K	H	A
T	R	I	S	T	P	A	S	A	G	E	V	Q	G	K	S	V	V	K	H	A
T	R	I	S	T	P	A	S	A	G	E	V	Q	G	K	S	V	V	K	H	A
T	R	I	S	T	P	A	S	A	G	E	V	Q	G	K	S	V	V	K	H	A
T	R	I	S	T	P	A	S	A	G	E	V	Q	G	K	S	V	V	K	H	A
T	R	I	S	T	P	A	S	A	G	E	V	K	G	K	S	V	V	K	H	A
T	R	I	S	T	P	A	S	A	G	E	V	K	G	K	S	V	V	K	H	A
T	R	I	S	T	P	A	S	A	G	E	V	Q	G	K	S	V	V	K	H	A
T	R	I	S	T	P	A	S	A	G	E	V	Q	G	K	S	V	V	K	H	A
T	R	I	S	T	P	A	S	A	G	E	V	Q	G	K	S	V	V	K	H	A
T	R	I	S	T	P	A	S	A	G	E	V	Q	G	K	S	V	V	K	H	A
T	R	I	S	T	P	A	S	A	G	E	V	Q	G	K	S	V	V	K	H	A
T	R	I	S	T	P	A	S	A	G	E	V	Q	G	K	S	V	V	K	H	A
T	R	I	S	T	P	A	S	A	G	E	V	Q	G	K	S	V	V	K	H	A
T	R	I	S	T	P	A	S	A	G	E	V	Q	G	K	S	V	V	K	H	A
T	R	I	S	T	P	A	S	A	G	E	V	Q	G	K	S	V	V	K	H	A
T	R	I	S	T	P	A	S	A	G	E	V	Q	G	K	S	V	V	K	H	A
T	R	I	S	T	P	A	S	A	G	E	V	Q	G	K	S	V	V	K	H	A
T	R	I	S	T	P	A	S	A	G	E	V	Q	G	K	S	V	V	K	H	A
T	R	I	S	T	P	A	S	A	G	E	V	Q	G	K	S	V	V	K	H	A
T	R	I	S	T	P	A	S	A	G	E	V	Q	G	K	S	V	V	K	H	A
T	R	I	S	T	P	A	S	A	G	E	V	Q	G	K	S	V	V	K	H	A
T	R	I	S	T	P	A	S	A	G	E	V	Q	G	K	S	V	V	K	H	A
T	R	I	S	T	P	A	S	A	G	E	V	Q	G	K	S	V	V	K	H	A

T	R	I	S	T	P	A	S	A	G	E	V	Q	R	K	S	V	V	K	H	A
T	R	I	S	T	P	A	S	A	G	E	V	Q	G	K	S	V	V	K	H	A
T	R	I	S	T	P	A	S	A	G	E	V	Q	G	K	S	V	V	K	H	A
T	R	I	T	T	P	A	S	A	G	E	V	Q	G	K	S	V	V	K	H	A
T	R	I	S	T	P	A	S	A	G	E	V	Q	G	K	S	V	V	K	H	A
T	R	I	S	T	P	A	S	A	G	E	V	Q	G	K	S	V	V	K	H	A
T	R	I	S	T	P	A	S	A	G	E	V	Q	G	K	S	V	V	K	H	A
T	R	I	S	T	P	A	S	A	G	E	V	Q	G	K	S	V	V	K	H	A
T	R	I	S	T	P	A	S	A	G	E	V	Q	G	K	S	V	V	K	H	A
T	R	I	S	T	P	A	S	A	G	E	V	Q	G	K	S	V	V	K	H	A
T	R	I	S	T	P	A	S	A	G	E	V	Q	G	K	S	V	V	K	H	A
T	R	I	S	T	P	A	S	A	G	E	V	Q	G	K	S	V	V	K	H	A
T	R	I	S	T	P	A	S	A	G	E	V	Q	G	K	S	V	V	K	H	A
T	R	I	S	T	P	A	S	A	G	E	V	Q	G	K	S	V	V	K	H	A
T	R	I	S	T	P	A	S	A	G	E	V	Q	G	K	S	V	V	K	H	A
T	R	I	S	T	P	A	S	A	G	E	V	Q	G	K	S	V	V	K	H	A
T	R	I	S	T	P	A	S	A	G	E	V	Q	G	K	S	V	V	K	H	A
T	R	I	S	T	P	A	S	A	G	E	V	Q	G	K	S	V	V	K	H	A
T	R	I	S	T	P	A	S	A	G	E	V	Q	G	K	S	V	V	K	H	A
T	R	I	S	T	P	A	S	A	G	E	V	Q	G	K	S	V	V	K	H	A
T	R	I	S	T	P	A	S	A	G	E	V	Q	G	K	S	V	V	K	H	A
T	R	I	S	T	P	A	S	A	G	E	V	Q	G	K	S	V	V	K	H	A
T	R	I	S	T	P	A	S	A	G	E	V	Q	G	K	S	V	V	K	H	A
T	R	I	S	T	P	A	S	A	G	E	V	Q	G	K	S	V	V	K	H	A
T	R	I	S	T	P	A	S	A	G	E	V	Q	G	K	S	V	V	K	H	A
T	R	I	S	T	P	A	S	A	G	E	V	Q	G	K	S	V	V	K	H	A
T	R	I	S	T	P	A	S	A	G	E	V	Q	G	K	S	V	V	K	H	A
T	R	I	S	T	P	A	S	A	G	E	V	Q	G	K	S	V	V	K	H	A
T	R	I	S	T	P	A	S	A	G	E	V	Q	G	K	S	V	V	K	H	A
T	R	I	S	T	P	A	S	A	G	E	V	Q	G	K	S	V	V	K	H	A
T	R	I	S	T	P	A	S	A	G	E	V	Q	G	K	S	V	V	K	H	A
T	R	I	S	T	P	A	S	A	G	E	V	Q	G	K	S	V	V	K	H	A
T	R	I	S	T	P	A	S	A	G	E	V	Q	G	K	S	V	V	K	H	A
T	R	I	S	T	P	A	S	A	G	E	V	Q	G	K	S	V	V	K	H	A
T	R	I	S	T	P	A	S	A	G	E	V	Q	G	K	S	V	V	K	H	A
T	R	I	S	T	P	A	S	A	G	E	V	Q	G	K	S	V	V	K	H	A
T	R	I	S	T	P	A	S	A	G	E	V	Q	G	K	S	V	V	K	H	A
T	R	I	S	T	P	A	S	A	G	E	V	Q	G	K	S	V	V	K	H	A
T	R	I	S	T	P	A	S	A	G	E	V	Q	G	K	S	V	V	K	H	A
T	R	I	S	T	P	A	S	A	G	E	V	Q	G	K	S	V	V	K	H	A
T	R	I	S	T	P	A	S	A	G	E	V	Q	G	K	S	V	V	K	H	A
T	R	I	S	T	P	A	S	A	G	E	V	Q	G	K	S	V	V	K	H	A
T	R	I	S	T	P	A	S	A	G	E	V	Q	G	K	S	V	V	K	H	A
T	R	I	S	T	P	A	S	A	G	E	V	Q	G	K	S	V	V	K	H	A
T	R	I	S	T	P	A	S	A	G	E	V	Q	G	K	S	V	V	K	H	A
T	R	I	S	T	P	A	S	A	G	E	V	Q	G	K	S	V	V	K	H	A
T	R	I	S	T	P	A	S	A	G	E	V	Q	G	K	S	V	V	K	H	A
T	R	I	S	T	P	A	S	A	G	E	V	Q	G	K	S	V	V	K	H	A
T	R	I	S	T	P	A	S	A	G	E	V	Q	G	K	S	V	V	K	H	A
T	R	I	S	T	P	A	S	A	G	E	V	Q	G	K	S	V	V	K	H	A
T	R	I	S	T	P	A	S	A	G	E	V	Q	G	K	S	V	V	K	H	A
T	R	I	S	T	P	A	S	A	G	E	V	Q	G	K	S	V	V	K	H	A
T	R	I	S	T	P	A	S	A	G	E	V	Q	G	K	S	V	V	K	H	A
T	R	I	S	T	P	A	S	A	G	E	V	Q	G	K	S	V	V	K	H	A
T	R	I	S	T	P	A	S	A	G	E	V	Q	G	K	S	V	V	K	H	A
T	R	I	S	T	P	A	S	A	G	E	V	Q	G	K	S	V	V	K	H	A
T	R	I	S	T	P	A	S	A	G	E	V	Q	G	K	S	V	V	K	H	A
T	R	I	S	T	P	A	S	A	G	E	V	Q	G	K	S	V	V	K	H	A
T	R	I	S	T	P	A	S	A	G	E	V	Q	G	K	S	V	V	K	H	A
T	R	I	S	T	P	A	S	A	G	E	V	Q	G	K	S	V	V	K	H	A
T	R	I	S	T	P	A	S	A	G	E	V	Q	G	K	S	V	V	K	H	A
T	R	I	S	T	P	A	S	A	G	E	V	Q	G	K	S	V	V	K	H	A
T	R	I	S	T	P	A	S	A	G	E	V	Q	G	K	S	V	V	K	H	A
T	R	I	S	T	P	A	S	A	G	E	V	Q	G	E	S	V	V	K	H	A
T	R	I	S	T	P	A	S	A	G	E	V	Q	G	E	S	V	V	K	H	A
T	R	I	S	T	P	A	S	A	G	E	V	Q	G	E	S	V	V	K	H	A
T	R	I	S	T	P	A	S	A	G	E	V	Q	G	E	S	V	V	K	H	A

T	R	I	S	T	P	A	S	A	G	E	V	Q	G	E	S	V	V	K	H	A
T	R	I	S	T	P	A	S	A	G	E	V	Q	G	E	S	V	V	K	H	A
T	R	I	S	T	P	A	S	A	G	E	V	Q	G	E	S	V	V	K	H	A
T	R	I	S	T	P	A	S	A	G	E	V	Q	G	E	S	V	V	K	H	A
T	R	I	S	T	P	A	S	A	G	E	V	Q	G	E	S	V	V	K	H	A
T	R	I	S	T	P	A	S	A	G	E	V	Q	G	E	S	V	V	K	H	A
T	R	I	S	T	P	A	S	A	G	E	V	Q	G	E	S	V	V	K	H	A
T	R	I	S	T	P	A	S	A	G	G	V	Q	G	E	S	V	V	K	H	A
T	R	I	S	T	P	A	S	A	G	E	V	Q	G	E	S	V	V	K	H	A
T	R	I	S	T	P	A	S	A	G	E	V	Q	G	E	S	V	V	K	H	A
T	R	I	S	T	P	A	S	A	G	G	V	Q	G	E	S	V	V	K	H	A
T	R	I	S	T	P	A	S	A	G	E	V	Q	G	E	S	V	V	K	H	A
T	R	I	S	T	P	A	S	A	G	E	V	Q	G	E	S	V	V	K	H	A
T	R	I	S	T	P	A	S	A	G	E	V	Q	G	E	S	V	V	K	H	A
T	R	I	S	T	P	A	S	A	G	E	V	Q	G	E	S	V	V	K	H	A
T	R	I	S	T	P	A	S	A	G	E	V	Q	G	K	S	V	V	K	H	A
T	R	I	S	T	P	A	S	A	G	E	V	Q	G	K	S	V	V	K	H	A
T	R	I	S	T	P	A	S	A	G	E	V	Q	G	K	S	V	V	K	H	A

hC	wchD	wzy			wzx	gla	ugd		rmlA						rm
168		6	135	422	145		126	132	25	68	141	192	199	245	152
A		Q	T	M	S		S	L	L	G	V	E	A	A	P
A		Q	T	M	S		S	L	L	G	V	E	A	A	P
A		Q	T	M	S		S	L	L	G	V	E	A	A	P
A		Q	T	M	S		S	L	L	G	V	E	A	A	P
A		Q	T	M	S		S	L	L	G	V	E	A	A	P
A		Q	T	M	S		S	L	L	G	V	E	A	A	P
A		Q	T	M	S		S	L	L	G	V	E	A	A	P
A		Q	T	M	S		S	L	L	G	V	E	A	A	P
A		Q	T	M	S		S	L	L	G	V	E	A	A	P
A		Q	T	M	S		S	L	L	G	V	E	A	A	P
A		Q	T	M	S		S	L	L	G	V	E	A	A	P
A		Q	T	M	S		S	L	L	G	V	E	A	A	P
A		Q	T	M	S		S	L	L	G	V	E	A	A	P
A		Q	T	M	S		S	L	L	G	V	E	A	A	P
A		Q	T	M	S		S	L	L	G	V	E	A	A	P
A		Q	T	M	S		S	L	L	G	V	E	A	A	P
A		Q	T	M	S		S	L	L	G	V	E	A	A	P
A		Q	T	M	S		S	L	L	G	V	E	A	A	P
A		Q	T	M	S		S	L	L	G	V	E	A	A	P
A		Q	T	M	S		S	L	L	G	V	E	A	A	P
A		Q	T	M	S		S	L	L	G	V	E	A	A	P
A		Q	T	M	S		S	L	L	G	V	E	A	A	P
A		Q	T	M	S		S	L	L	G	V	E	A	A	P
A		Q	T	M	S		S	L	L	G	V	E	A	A	P
A		Q	T	M	S		S	L	L	G	V	E	A	A	P
A		Q	T	M	S		S	L	L	G	V	E	A	A	P
A		Q	T	M	S		S	L	L	G	V	E	A	A	P
A		Q	T	M	S		S	L	L	G	V	E	A	A	P
A		Q	T	M	S		S	L	L	G	V	E	A	A	P
A		Q	T	M	S		S	L	L	G	V	E	A	A	P
A		Q	T	M	S		S	L	L	G	V	E	A	A	P
A		Q	T	M	S		S	L	L	G	V	E	A	A	P
A		Q	T	M	S		S	L	L	G	V	E	A	A	P
A		Q	T	M	S		S	L	L	G	V	E	A	A	P
A		Q	T	M	S		S	L	L	G	V	E	A	A	P
A		Q	T	M	S		S	L	L	G	V	E	A	A	P
A		Q	T	M	S		S	L	L	G	V	E	A	A	P
A		Q	T	M	S		S	L	L	G	V	E	A	A	P
A		Q	T	M	S		S	L	L	G	V	E	A	A	P
A		Q	T	M	S		S	L	L	G	V	E	A	A	P
A		Q	T	M	S		S	L	L	G	V	E	A	A	P
A		Q	T	M	S		S	L	L	G	V	E	A	A	S
A		Q	T	M	S		S	L	L	G	V	E	A	A	P
A		Q	T	M	S		S	L	L	G	V	E	A	A	S
A		Q	T	M	S		S	L	L	G	V	E	A	A	P
A		Q	T	M	S		S	L	L	G	V	E	A	A	P
A		Q	T	M	S		S	L	L	G	V	E	A	A	P
A		Q	T	M	S		S	L	L	G	V	E	A	A	P
A		Q	T	M	S		S	L	L	G	V	E	A	A	P
A		Q	T	M	S		S	L	L	G	V	E	A	A	P
A		Q	T	M	S		S	L	L	G	V	E	A	A	P
A		Q	T	M	S		S	L	L	G	V	E	A	A	P
A		Q	T	M	S		S	L	L	G	V	E	A	A	P
A		Q	T	M	S		S	L	L	G	V	E	A	A	P
A		Q	T	M	S		S	L	L	G	V	E	A	A	P
A		Q	T	M	S		S	L	L	G	V	E	A	A	P
A		Q	T	M	S		S	L	L	G	V	E	A	A	P
A		Q	T	M	S		S	L	L	G	V	E	A	A	P
A		Q	T	M	S		S	L	L	G	V	E	A	A	P
A		Q	T	M	S		S	L	L	G	V	E	A	A	P
A		Q	T	M	S		S	L	L	G	V	E	A	A	P
A		Q	T	M	S		S	L	L	G	V	E	A	A	P
A		Q	T	M	S		S	L	L	G	V	E	A	A	P

A		Q	T	M	S		S	L	L	G	V	E	A	A	P
A		Q	T	M	S		S	L	L	G	V	E	A	A	P
A		Q	T	M	S		S	L	L	G	V	E	A	A	P
A		Q	T	M	S		S	L	L	G	V	E	A	A	P
A		Q	T	M	S		S	L	L	G	V	E	A	A	P
A		Q	T	M	S		S	L	L	G	V	E	A	A	P
A		Q	T	M	S		S	L	L	G	V	E	A	A	P
A		Q	T	M	S		S	L	L	G	V	E	V	A	P
A		Q	T	M	S		S	L	L	G	V	E	A	A	P
A		Q	T	M	S		S	L	L	G	V	E	A	A	P
A		Q	T	M	S		S	L	L	G	V	E	A	A	P
A		Q	T	M	S		S	L	L	G	V	E	A	A	P
A		Q	T	M	S		S	L	L	G	V	E	A	A	P
A		Q	T	M	S		S	L	L	G	V	E	A	A	P
A		Q	T	M	S		S	L	L	G	V	E	A	A	P
A		Q	T	M	S		S	L	L	G	V	E	V	A	P
A		Q	T	M	S		S	L	L	G	V	E	V	A	P
A		Q	T	M	S		S	L	L	G	V	E	V	A	P
A		Q	T	M	S		S	L	L	G	V	E	V	A	P
A		Q	T	M	S		S	L	L	G	V	E	V	A	P
A		Q	T	M	S		S	L	L	G	V	E	V	A	P
A		Q	T	M	S		S	L	L	G	V	E	V	A	P
A		Q	T	M	S		S	L	L	G	V	E	V	A	P
A		Q	T	M	S		S	L	L	G	V	E	A	A	P
A		Q	T	M	S		S	L	L	G	V	E	A	A	P
A		Q	T	M	S		S	L	L	G	V	E	A	A	P
A		Q	T	M	S		S	L	L	G	V	E	A	A	P
A		Q	T	M	S		S	L	L	G	V	E	V	A	P
A		Q	T	M	S		S	L	L	G	V	E	V	A	P
A		Q	T	M	S		S	L	L	G	V	E	A	A	P
A		Q	T	M	S		S	L	L	G	V	E	A	A	P
A		Q	T	M	S		S	L	L	G	V	E	A	A	P
A		Q	T	M	S		S	L	L	G	V	E	A	A	P
A		Q	T	M	S		S	L	L	G	V	E	A	A	P
A		Q	T	M	S		S	L	L	G	V	E	A	A	P
A		Q	T	M	S		S	L	L	G	V	E	A	A	P
A		Q	T	M	S		S	L	L	G	V	E	A	A	P
A		Q	T	M	S		S	L	L	G	V	E	A	A	P
A		Q	T	M	S		S	L	L	G	V	E	A	A	P
A		Q	T	M	S		S	L	L	G	V	E	A	A	P
A		Q	T	M	S		S	L	L	G	V	E	A	A	P
A		Q	T	M	S		S	L	L	G	V	E	A	A	P
A		Q	T	M	S		S	L	L	G	V	E	A	A	P
A		Q	T	M	S		S	L	L	G	V	E	A	A	P
A		Q	T	M	S		S	L	L	G	V	E	A	A	P
A		Q	T	M	S		S	L	L	G	V	E	A	A	P
A		Q	T	M	S		S	L	L	G	V	E	V	A	P
A		Q	T	M	S		S	L	L	G	V	E	A	A	P
A		Q	T	M	S		S	L	L	G	V	E	A	A	P
A		Q	T	M	S		S	L	L	G	V	E	A	A	P
A		Q	T	M	S		S	L	L	G	V	E	A	A	P
A		Q	T	M	S		S	L	L	G	V	E	A	A	P
A		Q	T	M	S		S	L	L	G	V	E	A	A	P
A		Q	T	M	S		S	L	L	G	V	E	A	A	P
A		Q	T	M	S		S	L	L	G	V	E	A	A	P
A		Q	T	M	S		S	L	L	G	V	E	A	A	P
A		Q	T	M	S		S	L	L	G	V	E	V	A	P
A		Q	T	M	S		S	L	L	G	V	E	V	A	P
A		Q	T	M	S		S	L	L	G	V	E	A	A	P
A		Q	T	M	S		S	L	L	G	V	E	A	A	P
A		Q	T	M	S		S	L	L	G	V	E	A	A	P
A		Q	T	M	S		S	L	L	G	V	E	A	A	P
A		Q	T	M	S		S	L	L	G	V	E	A	A	P
A		Q	T	M	S		S	L	L	G	V	E	A	A	P
A		Q	T	M	S		S	L	L	G	V	E	A	A	P
A		Q	T	M	S		S	L	L	G	V	E	A	A	P
A		Q	T	M	S		S	L	L	G	V	E	A	A	P

A		Q	T	M	S		S	L	L	G	V	E	A	A	P
A		Q	T	M	S		S	L	L	G	V	E	A	A	P
A		Q	T	M	S		S	L	L	G	V	E	A	A	P
A		Q	T	M	S		S	L	L	G	V	E	A	A	P
A		Q	T	M	S		S	L	L	G	V	E	A	A	P
A		Q	T	M	S		S	L	L	G	V	E	A	A	P
A		Q	T	M	S		S	L	L	G	V	E	A	A	P
A		Q	T	M	S		S	L	L	G	V	E	V	A	P
A		Q	T	M	S		S	L	L	G	V	E	V	A	P
A		Q	T	M	S		S	L	L	G	V	E	V	A	P
A		Q	T	M	S		S	L	L	G	V	E	V	A	P
A		Q	T	M	S		S	L	L	G	V	E	V	A	P
A		Q	T	M	S		S	L	L	G	V	E	V	A	P
A		Q	T	M	S		S	L	L	G	V	E	V	A	P
A		Q	T	M	S		S	L	L	G	V	E	V	A	P
A		Q	T	M	S		S	L	L	G	V	E	V	A	P
A		Q	T	M	S		S	L	L	G	V	E	V	A	P
A		Q	T	M	S		S	L	L	G	V	E	V	A	P
A		Q	T	M	S		S	L	L	G	V	E	V	A	P
A		Q	T	M	S		S	L	L	G	V	E	V	A	P
A		Q	T	M	S		S	L	L	G	L	E	V	A	P
A		Q	S	M	S		S	L	L	G	V	E	V	A	P
A		Q	T	M	S		S	L	I	G	V	E	V	A	P
A		Q	T	M	S		S	L	L	G	V	E	V	A	P
A		Q	T	M	S		S	L	L	G	V	E	V	A	P
A		Q	T	M	S		S	L	L	G	V	E	V	A	P
A		Q	T	M	S		S	L	L	G	V	E	V	A	P
A		Q	T	M	S		S	L	L	G	V	E	V	A	P
A		Q	T	M	S		S	L	L	G	V	E	V	A	P
A		Q	T	M	S		S	L	L	G	V	A	V	A	P
A		Q	T	M	S		S	L	L	G	V	E	V	A	P
A		Q	T	M	S		S	L	L	G	V	E	A	A	P
A		Q	T	M	S		S	L	L	G	V	E	V	A	P
A		Q	T	M	S		S	L	L	G	V	E	V	A	P
A		Q	T	M	S		S	L	L	G	V	E	V	A	P
A		Q	T	M	S		S	L	L	G	V	E	V	A	P
A		H	T	M	S		S	L	L	G	V	E	V	A	P
A		Q	T	M	S		S	L	L	G	V	E	V	A	P
A		Q	T	M	S		S	L	L	G	V	E	V	A	P
A		Q	T	M	S		S	L	L	G	V	E	V	A	P
A		Q	T	M	S		S	L	L	G	V	E	V	A	P
A		Q	T	M	S		S	L	L	G	V	E	V	A	P
A		Q	T	M	S		S	L	L	G	V	E	V	A	P
A		Q	T	M	S		S	L	L	G	V	E	V	A	P
A		Q	T	M	S		S	L	L	G	V	E	V	A	P
A		Q	T	M	S		S	L	L	G	V	E	V	A	P
A		Q	T	M	S		S	L	L	G	V	E	A	A	P
A		Q	T	M	S		S	L	L	G	V	E	V	A	P
A		Q	T	M	S		S	L	L	G	V	E	V	A	P
A		Q	T	M	S		S	L	L	G	V	E	V	A	P
A		Q	T	M	S		S	L	L	G	V	E	V	A	P
A		Q	T	M	S		S	L	L	G	V	E	V	A	P
A		Q	T	M	C		S	L	L	G	V	E	V	A	P
A		Q	T	M	S		S	L	L	G	V	E	V	A	P
A		Q	T	M	S		S	L	L	G	V	E	V	A	P
A		Q	T	M	S		S	L	L	G	V	E	V	A	P
A		Q	T	M	S		S	L	L	G	V	E	V	A	P
A		Q	T	M	S		S	L	L	G	V	E	V	A	P
A		Q	T	M	S		S	L	L	G	V	E	A	A	P
A		Q	T	M	S		S	L	L	G	V	E	V	A	P
A		Q	T	M	S		S	L	L	G	V	E	V	A	P
A		Q	T	M	S		S	L	L	G	V	E	V	A	P
A		Q	T	M	S		S	L	L	G	V	E	V	A	P
A		Q	T	M	S		S	L	L	G	V	E	V	A	P
A		Q	T	M	S		S	L	L	G	V	E	V	A	P
A		Q	T	M	S		S	L	L	G	V	E	V	A	P
A		Q	T	M	S		S	L	L	G	V	E	V	A	P

A		Q	T	M	S		S	L	L	G	V	E	V	A	P
A		Q	T	M	S		S	L	L	G	V	E	V	A	P
A		Q	T	M	S		S	F	L	E	V	E	V	A	P
A		Q	T	M	S		S	L	L	G	V	E	V	A	P
A		Q	T	M	S		S	L	L	G	V	E	V	A	P
A		Q	T	M	S		S	L	L	G	V	E	V	A	P
A		Q	T	M	S		S	L	L	G	V	E	V	A	P
A		Q	T	M	S		S	L	L	G	V	E	V	A	P
A		Q	T	M	S		S	L	L	G	V	E	V	A	P
A		Q	T	M	S		S	L	L	G	V	E	V	A	P
A		Q	T	M	S		S	L	L	G	V	E	V	A	P
A		Q	T	M	S		S	L	L	G	V	E	V	A	P
A		Q	T	M	S		S	L	L	G	V	E	V	A	P
A		Q	T	M	S		S	L	L	G	V	E	V	A	P
A		Q	T	M	S		S	L	L	G	V	E	V	A	P
A		Q	T	M	S		S	L	L	G	V	E	V	A	P
A		Q	T	M	S		S	L	L	G	V	E	V	A	P
A		Q	T	M	S		S	L	L	G	V	E	V	A	P
A		Q	T	M	S		S	L	L	G	V	E	V	A	P
A		Q	T	M	S		S	L	L	G	V	E	V	A	P
A		Q	T	M	S		S	L	L	G	V	E	V	A	P
A		Q	T	M	S		S	L	L	G	V	E	V	A	P
A		Q	T	M	S		S	L	L	G	V	E	V	A	P
A		Q	T	M	S		S	L	L	G	V	E	V	A	P
A		Q	T	M	S		S	L	L	G	V	E	V	A	P
A		Q	T	M	S		S	L	L	G	V	E	A	A	P
A		Q	T	M	S		S	L	L	G	V	E	A	A	P
S		Q	T	M	S		S	L	L	G	V	E	A	A	P
A		Q	T	M	S		S	L	L	G	V	E	A	A	P
A		Q	T	M	S		S	L	L	G	V	E	A	A	P
A		Q	T	M	S		S	L	L	G	V	E	A	A	P
A		Q	T	M	S		S	L	L	G	V	E	A	A	P
A		Q	T	M	S		S	L	L	G	V	E	A	A	P
A		Q	T	M	S		S	L	L	G	V	E	A	A	P
A		Q	T	M	S		S	L	L	G	V	E	A	A	P
A		Q	T	M	S		S	L	L	G	V	E	A	A	P
A		Q	T	M	S		S	L	L	G	V	E	A	A	P
A		Q	T	M	S		S	L	L	G	V	E	A	A	P
A		Q	T	M	S		S	L	L	G	V	E	A	A	P
A		Q	T	M	S		S	L	L	G	V	E	A	A	P
A		Q	T	M	S		S	L	L	G	V	E	A	A	P
A		Q	T	M	S		S	L	L	G	V	E	A	A	P
A		Q	T	M	S		S	L	L	G	V	E	A	A	P
A		Q	T	M	S		S	L	L	G	V	E	A	A	P
A		Q	T	M	S		S	L	L	G	V	E	A	A	P
A		Q	T	M	S		S	L	L	G	V	E	A	V	P
A		Q	T	M	S		S	L	L	G	V	E	A	A	P
A		Q	T	M	S		S	L	L	G	V	E	A	A	P
A		Q	T	M	S		S	L	L	G	V	E	V	A	P
A		Q	T	M	S		S	L	L	G	V	E	V	A	P
A		Q	T	M	S		S	L	L	G	V	E	A	A	P
A		Q	T	M	S		S	L	L	G	V	E	A	A	P
A		Q	T	M	S		S	L	L	G	V	E	A	A	P
A		Q	T	M	S		S	L	L	G	V	E	V	A	P
A		Q	T	M	S		S	L	L	G	V	E	V	A	P
A		Q	T	M	S		S	L	L	G	V	E	V	A	P
A		Q	T	M	S		S	L	L	G	V	E	V	A	P
A		Q	T	M	S		S	L	L	G	V	E	V	A	P
A		Q	T	M	S		S	L	L	G	V	E	A	A	P
A		Q	T	M	S		S	L	L	G	V	E	A	A	P
A		Q	T	M	S		S	L	L	G	V	E	A	A	P
A		Q	T	M	S		S	L	L	G	V	E	A	A	S
A		Q	T	M	S		S	L	L	G	V	E	A	A	P
A		Q	T	M	S		S	L	L	G	V	E	A	A	P
A		Q	T	M	S		S	L	L	G	V	E	A	A	P
A		Q	T	M	S		S	L	L	G	V	E	A	A	P
A		Q	T	M	S		S	L	L	G	V	E	A	A	P

A		Q	T	M	S		S	L	L	G	V	E	A	A	P
A		Q	T	M	S		S	L	L	G	V	E	A	A	P
A		Q	T	M	S		S	L	L	G	V	E	A	A	P
A		Q	T	M	S		S	L	L	G	V	E	V	A	P
A		Q	T	M	S		S	L	L	G	V	E	V	A	P
A		Q	T	M	S		S	L	L	G	V	E	A	A	P
A		Q	T	M	S		S	L	L	G	V	E	V	A	P
A		Q	T	M	S		S	L	L	G	V	E	A	A	P
A		Q	T	M	S		S	L	L	G	V	E	A	A	P
A		Q	T	M	S		S	L	L	G	V	E	A	A	P
A		Q	T	M	S		S	L	L	G	V	E	A	A	P
A		Q	T	M	S		S	L	L	G	V	E	A	A	P
A		Q	T	M	S		S	L	L	G	V	E	A	A	P
A		Q	T	M	S		S	L	L	G	V	E	A	A	P
A		Q	T	M	S		S	L	L	G	V	E	A	A	P
A		Q	T	M	S		S	L	L	G	V	E	A	A	P
A		Q	T	M	S		S	L	L	G	V	E	A	A	P
A		Q	T	M	S		S	L	L	G	V	E	A	A	P
A		Q	T	M	S		S	L	L	G	V	E	A	A	P
A		Q	T	M	S		L	L	L	G	V	E	A	A	P
A		Q	T	M	S		L	L	L	G	V	E	A	A	P
A		Q	T	M	S		L	L	L	G	V	E	A	A	P
A		Q	T	M	S		S	L	L	G	V	E	A	A	S
A		Q	T	M	S		L	L	L	G	V	E	A	A	P
A		Q	T	M	S		L	L	L	G	V	E	A	A	P
A		Q	T	M	S		S	L	L	G	V	E	A	A	P
A		Q	T	M	S		L	L	L	G	V	E	A	A	P
A		Q	T	M	S		L	L	L	G	V	E	A	A	P
A		Q	T	M	S		L	L	L	G	V	E	A	A	P
A		Q	T	M	S		S	L	L	G	V	E	A	A	S
A		Q	T	M	S		S	L	L	G	V	E	A	A	P
A		Q	T	M	S		S	L	L	G	V	E	A	A	P
A		Q	T	M	S		S	L	L	G	V	E	A	A	P
A		Q	T	M	S		S	L	L	G	V	E	A	A	P
A		Q	T	M	S		S	L	L	G	V	E	A	A	P
A		Q	T	M	S		S	L	L	G	V	E	A	A	P
A		Q	T	M	S		S	L	L	G	V	E	A	A	P
A		Q	T	M	S		S	L	L	G	V	E	A	A	P
A		Q	T	M	S		S	L	L	G	V	E	A	A	P
A		Q	T	M	S		S	L	L	G	V	E	A	A	P
A		Q	T	M	S		S	L	L	G	V	E	A	A	S
A		Q	T	M	S		S	L	L	G	V	E	A	A	P
A		Q	T	M	S		S	L	L	G	V	E	A	A	P
A		Q	T	M	S		S	L	L	G	V	E	A	A	S
A		Q	T	M	S		S	L	L	G	V	E	A	A	P
A		Q	T	M	S		S	L	L	G	V	E	A	A	P
A		Q	T	M	S		S	L	L	G	V	E	A	A	P
A		Q	T	M	S		S	L	L	G	V	E	A	A	S
A		Q	T	M	S		S	L	L	G	V	E	A	A	P
A		Q	T	M	S		S	L	L	G	V	E	A	A	P
A		Q	T	M	S		S	L	L	G	V	E	A	A	P
A		Q	T	M	S		S	L	L	G	V	E	A	A	P
A		Q	T	M	S		S	L	L	G	V	E	V	A	P
A		Q	T	M	S		S	L	L	G	V	E	A	A	P
A		Q	T	M	S		S	L	L	G	V	E	V	A	P
A		Q	T	M	S		S	L	L	G	V	E	V	A	P
A		Q	T	M	S		S	L	L	G	V	E	V	A	P
A		Q	T	M	S		S	L	L	G	V	E	V	A	P
A		Q	T	K	S		S	L	L	G	V	E	V	A	P
A		Q	T	M	S		S	L	L	G	V	E	V	A	P
A		Q	T	M	S		S	L	L	G	V	E	V	A	P
A		Q	T	M	S		S	L	L	G	V	E	V	A	P
A		Q	T	M	S		S	L	L	G	V	E	V	A	P
A		Q	T	M	S		S	L	L	G	V	E	A	A	P
A		Q	T	M	S		S	L	L	G	V	E	A	A	P
A		Q	T	M	S		S	L	L	G	V	E	A	A	P
A		Q	T	M	S		S	L	L	G	V	E	A	A	P

A		Q	T	M	S		S	L	L	G	V	E	A	A	P
A		Q	T	M	S		S	L	L	G	V	E	A	A	P
A		Q	T	M	S		S	L	L	G	V	E	A	A	P
A		Q	T	M	S		S	L	L	G	V	E	A	A	P
A		Q	T	M	S		S	L	L	G	V	E	A	A	P
A		Q	T	M	S		S	L	L	G	V	E	A	A	P
A		Q	T	M	S		S	L	L	G	V	E	A	A	P
A		Q	T	M	S		S	L	L	G	V	E	A	A	P
A		Q	T	M	S		S	L	L	G	V	E	A	A	P
A		Q	T	M	S		S	L	L	G	V	E	A	A	P
A		Q	T	M	S		S	L	L	G	V	E	A	A	P
A		Q	T	M	S		S	L	L	G	V	E	A	A	P
A		Q	T	M	S		S	L	L	G	V	E	A	A	P
A		Q	T	M	S		S	L	L	G	V	E	A	A	P
A		Q	T	M	S		S	L	L	G	V	E	A	A	P
A		Q	T	M	S		S	L	L	G	V	E	A	A	P
A		Q	T	M	S		S	L	L	G	V	E	A	A	P
A		Q	T	M	S		S	L	L	G	V	E	A	A	P

lC	rmlB			
197	140	203	217	299
L	G	P	L	R
L	D	P	L	R
L	D	P	L	R
L	D	P	L	R
L	D	P	L	R
L	D	P	L	R
L	D	P	L	R
L	D	P	L	R
L	D	P	L	R
L	D	P	L	R
L	D	P	L	R
L	D	P	L	R
L	D	P	L	R
L	D	P	L	R
L	D	P	L	R
L	D	P	L	R
L	D	P	L	R
L	D	P	L	C
L	D	P	L	R
L	D	P	L	R
L	D	P	L	R
L	D	P	L	C
L	D	P	L	R
L	G	P	L	R
L	D	P	L	R
L	D	P	L	C
L	D	P	L	R
L	D	P	L	R
L	D	P	L	R
L	D	P	L	R
L	G	P	L	R
L	G	P	L	R
L	G	P	L	R
L	G	P	L	R
L	G	P	L	R
L	G	P	L	R
L	G	P	L	R
L	G	P	L	R
L	G	P	L	R
L	G	P	L	R
L	G	P	L	R
L	D	P	L	R
L	G	P	L	R
L	D	P	L	R
L	G	P	L	R
L	G	P	L	R
L	D	P	L	R
L	D	P	L	C
L	D	P	L	R
L	D	P	L	R
L	D	P	L	R
L	D	P	L	R
L	D	P	L	R
L	D	P	L	R
L	D	P	L	R
L	D	P	L	R
L	D	P	L	R
L	D	P	L	R
L	D	P	L	R
L	D	P	L	R
L	D	P	L	R
L	D	P	L	R
L	D	P	L	R

L	D	P	L	R
L	D	P	L	R
L	D	P	L	R
L	G	P	L	R
L	G	P	L	R
L	G	P	L	R
L	G	P	L	R
L	G	P	L	R
L	D	P	L	R
L	G	P	L	R
L	G	P	L	R
L	G	P	L	R
L	G	P	L	R
L	G	P	L	R
L	G	P	L	R
L	G	P	L	R
L	G	P	L	R
L	G	P	L	R
L	G	P	L	R
L	G	P	L	R
L	G	P	L	R
L	G	P	L	R
L	G	P	L	R
L	G	P	L	R
L	G	P	L	R
L	G	P	L	R
L	G	P	L	R
L	G	P	L	R
L	G	P	L	R
L	G	P	L	R
L	G	P	L	R
L	G	P	L	R
L	G	P	L	R
L	G	P	L	R
L	G	P	L	R
L	G	P	L	R
L	G	P	L	R
L	G	P	L	R
L	G	P	L	R
L	G	P	L	R
L	G	P	L	R
L	G	P	L	R
L	G	P	L	R
L	G	P	L	R
L	G	P	L	R
L	G	P	L	R
L	G	P	L	R
L	G	P	L	R
L	G	P	L	R
L	D	P	L	R
L	G	P	L	R
L	G	P	L	R
L	G	P	L	R
L	G	P	L	R
L	G	P	L	R
L	G	P	L	R
L	G	P	L	R
L	G	P	L	R
L	G	P	L	R
L	G	P	L	R
L	G	P	L	R
L	G	P	L	R
L	G	P	L	R
L	D	P	L	R
L	G	P	L	R
L	G	P	L	R
L	G	P	L	R

L	D	P	L	R
L	D	P	L	R
L	D	P	L	R
L	D	P	L	R
L	D	P	L	R
L	D	P	L	R
L	D	P	L	R
L	G	P	L	R
L	G	P	L	R
L	G	P	L	R
L	G	P	L	R
L	G	P	L	R
L	G	P	L	R
L	G	P	L	R
L	G	P	L	R
L	G	P	L	R
L	G	P	L	R
L	G	P	L	R
L	G	P	L	R
L	G	P	L	R
L	G	P	L	R
L	G	P	L	R
L	G	P	L	R
L	G	P	L	R
L	G	P	L	R
L	G	P	L	R
L	G	P	L	R
L	G	P	L	R
L	G	P	L	R
L	G	P	L	R
L	G	P	L	R
L	G	P	L	R
L	G	P	L	R
L	G	P	L	R
L	G	P	L	R
L	G	P	L	R
L	G	P	L	R
L	G	P	L	R
L	G	P	L	R
L	G	P	L	R
L	G	P	L	R
L	G	P	L	R
L	G	P	L	R
L	G	P	L	R
L	G	P	L	R
L	G	P	L	R
L	G	P	L	R
L	G	P	L	R
L	G	P	L	R
L	G	P	L	R
L	G	P	L	R
L	G	P	L	R
L	G	P	L	R
L	G	P	L	R
L	G	P	L	R
L	G	P	L	R
L	G	P	L	R
L	G	P	L	R
L	G	P	L	R
L	G	P	L	R
L	G	P	L	R
L	G	P	L	R
L	G	P	L	R
L	G	P	L	R
L	G	P	L	R
L	G	P	L	R
L	G	P	L	R

L	G	P	L	R
L	G	P	L	R
L	G	P	L	R
L	G	P	L	R
L	G	P	L	R
L	G	P	L	R
L	G	P	L	R
L	G	P	L	R
L	G	P	L	R
L	G	P	L	R
L	G	P	L	R
L	G	P	L	R
L	G	P	L	R
L	G	P	L	R
L	G	P	L	R
L	G	P	I	R
L	G	P	I	R
L	G	P	L	R
L	G	P	L	R
L	G	P	L	R
L	G	P	L	R
L	G	P	L	R
L	G	P	L	R
L	G	P	L	R
L	G	P	L	R
L	G	P	L	R
L	G	P	L	R
L	G	P	L	R
L	G	P	L	R
L	G	P	L	R
L	G	P	L	R
L	G	P	L	R
L	G	P	L	R
L	G	P	L	R
L	G	P	L	R
L	G	P	L	R
L	G	P	L	R
L	G	P	L	R
L	G	L	L	R
L	G	P	L	R
L	G	P	L	R
L	G	L	L	R
L	G	P	L	R
L	G	P	L	R
L	G	P	L	R
L	G	P	L	R
L	G	P	L	R
L	G	P	L	R
L	G	P	L	R
L	G	P	L	R
L	G	P	L	R
L	G	P	L	R
L	G	P	L	R
L	G	P	L	R
L	G	P	L	R
L	G	P	L	R
L	G	P	L	R
L	G	P	L	R
L	D	P	L	R
L	D	P	L	R
L	D	P	L	R
L	D	P	L	R
L	D	P	L	R
L	D	P	L	R
L	D	P	L	R
L	G	P	L	R
L	G	P	L	R

L		G	P	L	R
L		G	P	L	R
L		G	P	L	R
L		G	P	L	R
L		G	P	L	R
L		G	P	L	R
L		G	P	L	R
L		G	P	L	R
L		G	P	L	R
L		G	P	L	R
L		D	P	L	R
L		D	P	L	R
L		G	P	L	R
L		G	P	L	R
F		G	P	L	R
L		G	P	L	R
L		G	P	L	R
L		G	P	L	R
L		D	P	L	R
L		G	P	L	R
L		G	P	L	R
L		G	P	L	R
L		D	P	L	R
L		G	P	L	R
L		G	P	L	R
L		D	P	L	R
L		G	P	L	R
L		G	P	L	R
L		G	P	L	R
L		D	P	L	R
L		D	P	L	R
L		D	P	L	R
L		D	P	L	R
L		D	P	L	R
L		D	P	L	R
L		G	P	L	R
L		G	P	L	R
L		G	P	L	R
L		G	P	L	R
L		D	P	L	R
L		D	P	L	R
L		G	P	L	R
L		G	P	L	R
L		D	P	L	R
L		G	P	L	R
L		D	P	L	R
L		D	P	L	R
L		D	P	L	R
L		D	P	L	R
L		D	P	L	R
L		D	P	L	R
L		D	P	L	R
L		G	P	L	R
L		G	P	L	R
L		G	P	L	R
L		G	P	L	R
L		G	P	L	R
L		G	P	L	R
L		G	P	L	R
L		G	P	L	R
L		G	P	L	R
L		G	P	L	R
L		G	P	L	R
L		G	P	L	R
L		G	P	L	R
L		G	P	L	R
L		G	P	L	R

L
L
L
L
L
L
L
L
L
L
L
L
L
L
L
L
L
L

G	P	L	R
G	P	L	R
G	P	L	R
G	P	L	R
G	P	L	R
G	P	L	R
G	P	L	R
G	P	L	R
G	P	L	R
G	P	L	R
G	P	L	R
G	P	L	R
G	P	L	R
G	P	L	R
G	P	L	R
G	P	L	R
G	P	L	R
G	P	L	R

rmlst ID	PubMLST id	ENA	Name	ST	CC	Orig_sero	Orig_sero_method
N/A	35815	N/A	3/2	378	378	3	Quellung
N/A	15515	N/A	3/3	7244	378	3	Quellung
N/A	35816	N/A	3/4	378	378	3	Quellung
N/A	15481	N/A	3/6	7211	1024	3	Quellung
N/A	35817	N/A	3/7	1377	378	3	Quellung
N/A	35818	N/A	3/8	1377	378	3	Quellung
32180	29638	ERR069748	65645	180	180	3	Quellung
33263	29502	ERR069750	72511	180	180	3	Quellung
32211	29488	ERR069832	369820	180	180	3	Quellung
N/A	2910	N/A	A45	6934	Sing6934	3	unknown
32849	30476	ERR026232	BHN035	180	180	3	unknown
32830	30477	ERR025297	BHN037	1826	180	3	unknown
32831	30478	ERR025298	BHN167	180	180	3	unknown
55122	30479	ERR025283	BHN341	180	180	3	unknown
31913	30480	ERR025301	BHN369	180	180	3	unknown
32832	30481	ERR025302	BHN383	180	180	3	unknown
31920	30482	ERR026215	BHN413	180	180	3	unknown
40058	30483	ERR026216	BHN570	180	180	3	unknown
31185	30484	ERR026217	BHN571	180	180	3	unknown
123296	30485	ERR026218	BHN572	180	180	3	unknown
123297	30486	ERR026219	BHN573	180	180	3	unknown
18528	30487	ERR026220	BHN574	180	180	3	unknown
18602	30488	ERR026222	BHN575	180	180	3	unknown
18573	30489	ERR026213	BHN577	180	180	3	unknown
32845	30490	ERR026214	BHN578	180	180	3	unknown
32847	30491	ERR026224	BHN579	180	180	3	unknown
31921	30492	ERR026228	BHN580	180	180	3	unknown
33816	30493	ERR026230	BHN582	180	180	3	unknown
31922	30494	ERR026231	BHN583	180	180	3	unknown
44810	30495	ERR026233	BHN584	180	180	3	unknown
32850	30496	ERR026234	BHN585	180	180	3	unknown
31923	30497	ERR026235	BHN586	505	180	3	unknown
115781	30498	ERR026225	BHN587	180	180	3	unknown
33815	30499	ERR026226	BHN588	180	180	3	unknown
123257	30500	ERR026227	BHN589	180	180	3	unknown
31924	30501	ERR026237	BHN590	180	180	3	unknown
18425	30502	ERR026241	BHN591	180	180	3	unknown
123298	30503	ERR026242	BHN592	180	180	3	unknown
123299	30504	ERR026243	BHN593	180	180	3	unknown
32853	30505	ERR026244	BHN594	180	180	3	unknown
31926	30506	ERR026245	BHN595	180	180	3	unknown
18461	30507	ERR026246	BHN596	180	180	3	unknown
31927	30508	ERR026247	BHN597	180	180	3	unknown
32854	30509	ERR026248	BHN598	180	180	3	unknown
32851	30510	ERR026238	BHN599	180	180	3	unknown
31925	30511	ERR026239	BHN600	180	180	3	unknown
32852	30512	ERR026240	BHN601	180	180	3	unknown
40045	30513	ERR025280	BHN602	180	180	3	unknown
32825	30514	ERR025284	BHN603	SLV180	180	3	unknown
32826	30515	ERR025285	BHN604	180	180	3	unknown
32827	30516	ERR025286	BHN605	180	180	3	unknown
31909	30517	ERR025287	BHN606	180	180	3	unknown
32828	30518	ERR025288	BHN607	180	180	3	unknown
31910	30519	ERR025289	BHN608	180	180	3	unknown
33793	30520	ERR025290	BHN609	180	180	3	unknown
33794	30521	ERR025291	BHN610	SLV180	180	3	unknown
18436	30522	ERR025281	BHN611	505	180	3	unknown
32824	30523	ERR025282	BHN612	180	180	3	unknown

33795	30524	ERR025293	BHN616	180	180	3	unknown
33797	30525	ERR025303	BHN621	180	180	3	unknown
33798	30526	ERR025304	BHN622	180	180	3	unknown
32829	30527	ERR025294	BHN623	180	180	3	unknown
33796	30528	ERR025295	BHN624	180	180	3	unknown
31911	30529	ERR025296	BHN625	180	180	3	unknown
33799	30530	ERR025306	BHN626	180	180	3	unknown
44808	30531	ERR025309	BHN628	180	180	3	unknown
33801	30532	ERR025310	BHN629	180	180	3	unknown
32834	30533	ERR025311	BHN630	180	180	3	unknown
32835	30534	ERR025312	BHN631	180	180	3	unknown
33802	30535	ERR025313	BHN632	180	180	3	unknown
32836	30536	ERR025314	BHN633	SLV180	180	3	unknown
33803	30537	ERR025315	BHN636	180	180	3	unknown
32837	30538	ERR025316	BHN637	180	180	3	unknown
32833	30539	ERR025307	BHN638	180	180	3	unknown
33800	30540	ERR025308	BHN640	180	180	3	unknown
123295	30541	ERR025318	BHN642	180	180	3	unknown
44142	30542	ERR025321	BHN643	180	180	3	unknown
33805	30543	ERR025322	BHN644	180	180	3	unknown
33806	30544	ERR025323	BHN645	180	180	3	unknown
40046	30545	ERR025324	BHN646	180	180	3	unknown
33807	30546	ERR025325	BHN647	180	180	3	unknown
32838	30547	ERR025326	BHN650	180	180	3	unknown
33808	30548	ERR025327	BHN651	180	180	3	unknown
33804	30549	ERR025319	BHN654	180	180	3	unknown
44141	30550	ERR025320	BHN657	180	180	3	unknown
N/A	35960	N/A	GA19690	180	180	3	unknown
32478	29319	ERR129180	MD5090	180	180	3	Quellung
32499	29639	ERR129212	ND6097	180	180	3	Quellung
32076	29317	ERR067983	R34-3078	180	180	3	Quellung
32081	29487	ERR067989	R34-3084	180	180	3	Quellung
31353	29162	ERR068006	R34-3101	180	180	3	Quellung
32141	29637	ERR069689	R34-3190	180	180	3	Quellung
32147	29318	ERR069701	R34-3202	180	180	3	Quellung
31413	29406	ERR069716	R34-3217	180	180	3	Quellung
32977	26568	ERR051605	SMRU1059	180	180	3	latex & Quellung
115541	26569	ERR054583	SMRU1131	4389	Sing4389	3	latex & Quellung
82242	26570	ERR054295	SMRU1202	4389	Sing4389	3	latex & Quellung
82243	26571	ERR054297	SMRU1204	4389	Sing4389	3	latex & Quellung
109679	26548	ERR047925	SMRU128	8799	1765/8799	3	latex & Quellung
115522	26574	ERR054279	SMRU1402	4389	Sing4389	3	latex & Quellung
111535	26573	ERR054282	SMRU1405	6012	180	3	latex & Quellung
113807	26576	ERR056770	SMRU1432	4389	Sing4389	3	latex & Quellung
82302	26575	ERR056772	SMRU1434	4389	Sing4389	3	latex & Quellung
109846	26577	ERR056793	SMRU1455	180	180	3	latex & Quellung
33112	26578	ERR054434	SMRU1485	4389	Sing4389	3	latex & Quellung
83244	26549	ERR049108	SMRU174	458	458	3	latex & Quellung
111451	26579	ERR060001	SMRU1773	4389	Sing4389	3	latex & Quellung
111445	26580	ERR060018	SMRU1814	4389	Sing4389	3	latex & Quellung
83263	26550	ERR050059	SMRU196	4389	Sing4389	3	latex & Quellung
82379	26581	ERR063831	SMRU2038	4389	Sing4389	3	latex & Quellung
115502	26551	ERR050070	SMRU207	4389	Sing4389	3	latex & Quellung
113095	26552	ERR049941	SMRU222	4389	Sing4389	3	latex & Quellung
109958	26582	ERR064159	SMRU2421	458	458	3	latex & Quellung
115513	26553	ERR052591	SMRU254	4389	Sing4389	3	latex & Quellung
82593	26583	ERR067851	SMRU2628	10441	458	3	latex & Quellung
55131	26554	ERR047953	SMRU276	4389	Sing4389	3	latex & Quellung
32925	26555	ERR047970	SMRU293	4389	Sing4389	3	latex & Quellung

32926	26556	ERR047971	SMRU294	4389	Sing4389	3	latex & Quellung
109687	26557	ERR048063	SMRU338	180	180	3	latex & Quellung
109688	26558	ERR048076	SMRU351	458	458	3	latex & Quellung
83223	26559	ERR048079	SMRU354	458	458	3	latex & Quellung
40162	26560	ERR047993	SMRU364	4389	Sing4389	3	latex & Quellung
115479	26562	ERR048003	SMRU374	180	180	3	latex & Quellung
40167	26561	ERR048005	SMRU376	4389	Sing4389	3	latex & Quellung
33908	26563	ERR048116	SMRU439	458	458	3	latex & Quellung
113760	26564	ERR048175	SMRU498	4389	Sing4389	3	latex & Quellung
33732	26565	ERR273494	SMRU543	458	458	3	latex & Quellung
115519	26566	ERR052646	SMRU573	4389	Sing4389	3	latex & Quellung
40215	26567	ERR051460	SMRU650	4389	Sing4389	3	latex & Quellung
N/A	35993	N/A	Sp02-1198	180	180	3	unknown
N/A	35933	N/A	Sp03-4156	180	180	3	unknown
N/A	35934	N/A	Sp03-4183	180	180	3	unknown
N/A	35992	N/A	Sp07-2838	SLV180	180	3	unknown
N/A	35931	N/A	SP3-BS71	180	180	3	unknown
N/A	35935	N/A	Sp99_4038	180	180	3	unknown
N/A	35936	N/A	Sp99_4039	180	180	3	unknown
N/A	35884	N/A	SPnOXC141	180	180	3	Quellung
121379	33823	ERR044882	UoS0103	180	180	unknown	WGS-based
55921	33073	ERR044929	UoS0272	180	180	unknown	WGS-based
123245	34169	ERR048220	UoS2370	180	180	unknown	WGS-based
121360	33804	ERR046055	UoS3033	180	180	unknown	WGS-based
121377	33821	ERR046102	UoS3205	180	180	unknown	WGS-based
123225	34149	ERR046150	UoS4027	180	180	unknown	WGS-based
32916	32921	ERR046176	UoS4133	180	180	unknown	WGS-based
123209	34133	ERR045405	UoS5023	180	180	unknown	WGS-based
N/A	34555	ERR388818	VICE0178	180	180	not done	--
N/A	33496	ERR388872	VICE0179	180	180	not done	--
N/A	33120	ERR388873	VICE0180	180	180	not done	--
N/A	33092	ERR388896	VICE0213	180	180	not done	--
N/A	33622	ERR540444	VICE0232	180	180	not done	--
N/A	33625	ERR540447	VICE0257	180	180	not done	--
N/A	33630	ERR540455	VICE0291	180	180	not done	--
N/A	33631	ERR540456	VICE0296	180	180	not done	--
N/A	33170	ERR449756	VICE0320	180	180	not done	--
N/A	33525	ERR470262	VICE0322	180	180	not done	--
N/A	33402	ERR460407	VICE0486	180	180	not done	--
N/A	33266	ERR460425	VICE0516	180	180	not done	--
N/A	33376	ERR460439	VICE0536	180	180	not done	--
N/A	33642	ERR540473	VICE0549	180	180	not done	--
N/A	33533	ERR470291	VICE0561	180	180	not done	--
N/A	33384	ERR460486	VICE0605	180	180	not done	--
N/A	33388	ERR460492	VICE0617	180	180	not done	--
N/A	33157	ERR470296	VICE0647	260	260	not done	--
N/A	33186	ERR449846	VICE0670	1377	378	not done	--
N/A	33148	ERR470258	VICE0689	505	180	not done	--
N/A	33159	ERR470303	VICE0718	180	180	not done	--
N/A	34280	ERR540487	VICE0723	180	180	not done	--
N/A	33263	ERR470387	VICE0744	180	180	not done	--
N/A	33265	ERR470399	VICE0764	180	180	not done	--
N/A	33398	ERR470417	VICE0793	180	180	not done	--
N/A	33582	ERR540500	VICE0795	180	180	not done	--
N/A	33162	ERR470314	VICE0852	505	180	not done	--
N/A	33662	ERR540508	VICE0853	505	180	not done	--
N/A	33163	ERR470317	VICE0858	505	180	not done	--
N/A	33583	ERR540513	VICE0882	180	180	not done	--
N/A	34447	ERR540535	VICE0965	180	180	not done	--

N/A	34402	ERR540551	VICE1024	180	180	not done	--
N/A	34529	ERR690901	VICE1052	180	180	not done	--
N/A	33692	ERR540590	VICE1078a	180	180	not done	--
N/A	33587	ERR540591	VICE1081	180	180	not done	--
N/A	34500	ERR690910	VICE1086	180	180	not done	--
N/A	34489	ERR540616	VICE1115a	180	180	not done	--
N/A	33700	ERR540619	VICE1117	180	180	not done	--
N/A	33701	ERR540623	VICE1121	180	180	not done	--
N/A	33456	ERR570731	VICE1126	180	180	not done	--
N/A	34531	ERR690915	VICE1162	505	180	not done	--
N/A	34544	ERR690917	VICE1174	180	180	not done	--
N/A	34532	ERR690919	VICE1188	180	180	not done	--
N/A	34545	ERR690920	VICE1189b	180	180	not done	--
N/A	36008	ERR755394	VICE1206	180	180	not done	--
N/A	36012	ERR755398	VICE1248	180	180	not done	--
N/A	34505	ERR690929	VICE1276b	180	180	not done	--
N/A	33571	ERR540412	VICE1330	180	180	not done	--
N/A	34587	ERR697214	VICE1478	180	180	not done	--
N/A	34471	ERR697223	VICE1488a	180	180	not done	--
N/A	36029	ERR755405	VICE1498	180	180	not done	--
N/A	34552	ERR697278	VICE1546	180	180	not done	--
N/A	36040	ERR755416	VICE1582	180	180	not done	--
N/A	36051	ERR755431	VICE1598	180	180	not done	--
N/A	36060	ERR755441	VICE1610	180	180	not done	--
N/A	36071	ERR755452	VICE1622	180	180	not done	--
N/A	36130	ERR755356	VICE1693	180	180	not done	--
N/A	36190	ERR755504	VICE1768a	180	180	not done	--
N/A	36197	ERR755512	VICE1778	505	180	not done	--
N/A	36253	ERR755581	VICE1864	180	180	not done	--

Seq_sero	Hybrid	Hybrid type	Country	Region	carriage/disease	Source
3	No	N/A	USA	unknown	unknown	unknown
3	No	N/A	USA	unknown	unknown	unknown
3	No	N/A	Denmark	unknown	unknown	unknown
3	No	N/A	Denmark	unknown	unknown	unknown
3	No	N/A	Denmark	unknown	unknown	unknown
3	No	N/A	Denmark	unknown	unknown	unknown
3	No	N/A	USA	Massachusetts	carriage	nasopharynx
3	No	N/A	USA	Massachusetts	carriage	nasopharynx
3	No	N/A	USA	Massachusetts	carriage	nasopharynx
3	No	N/A	United Kingdom	unknown	disease	unknown
3	No	N/A	Spain	unknown	disease	unknown
3	No	N/A	Sweden	unknown	disease	unknown
3	No	N/A	Sweden	unknown	disease	unknown
3	No	N/A	Sweden	unknown	disease	unknown
3	No	N/A	Sweden	unknown	disease	unknown
3	No	N/A	Sweden	unknown	disease	unknown
3	No	N/A	USA	unknown	disease	unknown
3	No	N/A	USA	unknown	disease	unknown
3	No	N/A	USA	unknown	disease	unknown
3	No	N/A	USA	unknown	disease	unknown
3	No	N/A	USA	unknown	disease	unknown
3	No	N/A	USA	unknown	disease	unknown
3	No	N/A	Sweden	unknown	disease	unknown
3	No	N/A	Sweden	unknown	disease	unknown
3	No	N/A	Sweden	unknown	disease	unknown
3	No	N/A	Sweden	unknown	disease	unknown
3	No	N/A	Sweden	unknown	disease	unknown
3	No	N/A	United Kingdom	England	disease	unknown
3	No	N/A	Sweden	unknown	disease	unknown
3	No	N/A	Spain	unknown	disease	unknown
3	No	N/A	Czech Republic	unknown	disease	CSF
3	No	N/A	Czech Republic	unknown	disease	CSF
3	No	N/A	Czech Republic	unknown	disease	CSF
3	No	N/A	Czech Republic	unknown	disease	CSF
3	No	N/A	Czech Republic	unknown	disease	CSF
3	No	N/A	Czech Republic	unknown	disease	CSF
3	No	N/A	Czech Republic	unknown	disease	CSF
3	No	N/A	Czech Republic	unknown	disease	CSF
3	No	N/A	Czech Republic	unknown	disease	CSF
3	No	N/A	Czech Republic	unknown	disease	CSF
3	No	N/A	Czech Republic	unknown	disease	CSF
3	No	N/A	Czech Republic	unknown	disease	CSF
3	No	N/A	Czech Republic	unknown	disease	CSF
3	No	N/A	Czech Republic	unknown	disease	CSF
3	No	N/A	Czech Republic	unknown	disease	CSF
3	No	N/A	Czech Republic	unknown	disease	CSF
3	No	N/A	Czech Republic	unknown	disease	CSF
3	No	N/A	Czech Republic	unknown	disease	CSF
3	No	N/A	Czech Republic	unknown	disease	CSF
3	No	N/A	Czech Republic	unknown	disease	CSF
3	No	N/A	Czech Republic	unknown	disease	CSF
3	No	N/A	Czech Republic	unknown	disease	CSF
3	No	N/A	Czech Republic	unknown	disease	CSF
3	No	N/A	Czech Republic	unknown	disease	CSF
3	No	N/A	Czech Republic	unknown	disease	CSF
3	No	N/A	Czech Republic	unknown	disease	CSF
3	No	N/A	Czech Republic	unknown	disease	CSF
3	No	N/A	Czech Republic	unknown	disease	CSF
3	No	N/A	Czech Republic	unknown	disease	CSF

3	No	N/A	Sweden	unknown	disease	unknown
3	No	N/A	Sweden	unknown	disease	unknown
3	No	N/A	Sweden	unknown	disease	unknown
3	No	N/A	Sweden	unknown	disease	unknown
3	No	N/A	Sweden	unknown	disease	unknown
3	No	N/A	Sweden	unknown	disease	unknown
3	No	N/A	Sweden	unknown	disease	unknown
3	No	N/A	Sweden	unknown	disease	unknown
3	No	N/A	Sweden	unknown	disease	unknown
3	No	N/A	Sweden	unknown	disease	unknown
3	No	N/A	Sweden	unknown	disease	unknown
3	No	N/A	Sweden	unknown	disease	unknown
3	No	N/A	Sweden	unknown	disease	unknown
3	No	N/A	Sweden	unknown	disease	unknown
3	No	N/A	Sweden	unknown	disease	unknown
3	No	N/A	Sweden	unknown	disease	unknown
3	No	N/A	Sweden	unknown	disease	unknown
3	No	N/A	Sweden	unknown	disease	unknown
3	No	N/A	Sweden	unknown	disease	unknown
3	No	N/A	Sweden	unknown	disease	unknown
3	No	N/A	Sweden	unknown	disease	unknown
3	No	N/A	Sweden	unknown	disease	unknown
3	No	N/A	Sweden	unknown	disease	CSF
3	No	N/A	Sweden	unknown	disease	CSF
3	No	N/A	Sweden	unknown	disease	CSF
3	No	N/A	Sweden	unknown	disease	CSF
3	No	N/A	Sweden	unknown	disease	CSF
3	No	N/A	USA	Georgia	disease	blood
3	No	N/A	USA	Massachusetts	carriage	nasopharynx
3	No	N/A	USA	Massachusetts	carriage	nasopharynx
3	No	N/A	USA	Massachusetts	carriage	nasopharynx
3	No	N/A	USA	Massachusetts	carriage	nasopharynx
3	No	N/A	USA	Massachusetts	carriage	nasopharynx
3	No	N/A	USA	Massachusetts	carriage	nasopharynx
3	No	N/A	USA	Massachusetts	carriage	nasopharynx
3	No	N/A	USA	Massachusetts	carriage	nasopharynx
3	No	N/A	Thailand	Maela	carriage	nasopharynx
3	No	N/A	Thailand	Maela	carriage	nasopharynx
3	No	N/A	Thailand	Maela	carriage	nasopharynx
3	No	N/A	Thailand	Maela	carriage	nasopharynx
3	No	N/A	Thailand	Maela	carriage	nasopharynx
3	No	N/A	Thailand	Maela	carriage	nasopharynx
3	No	N/A	Thailand	Maela	carriage	nasopharynx
3	No	N/A	Thailand	Maela	carriage	nasopharynx
3	No	N/A	Thailand	Maela	carriage	nasopharynx
3	No	N/A	Thailand	Maela	carriage	nasopharynx
3	No	N/A	Thailand	Maela	carriage	nasopharynx
3	No	N/A	Thailand	Maela	carriage	nasopharynx
3	No	N/A	Thailand	Maela	carriage	nasopharynx
3	No	N/A	Thailand	Maela	carriage	nasopharynx
3	No	N/A	Thailand	Maela	carriage	nasopharynx
3	No	N/A	Thailand	Maela	carriage	nasopharynx
3	No	N/A	Thailand	Maela	carriage	nasopharynx
3	No	N/A	Thailand	Maela	carriage	nasopharynx
3	No	N/A	Thailand	Maela	carriage	nasopharynx
3	No	N/A	Thailand	Maela	carriage	nasopharynx
3	No	N/A	Thailand	Maela	carriage	nasopharynx
3	No	N/A	Thailand	Maela	carriage	nasopharynx
3	No	N/A	Thailand	Maela	carriage	nasopharynx

3	No	N/A	Thailand	Maela	carriage	nasopharynx
3	No	N/A	Thailand	Maela	carriage	nasopharynx
3	No	N/A	Thailand	Maela	carriage	nasopharynx
3	No	N/A	Thailand	Maela	carriage	nasopharynx
3	No	N/A	Thailand	Maela	carriage	nasopharynx
3	No	N/A	Thailand	Maela	carriage	nasopharynx
3	No	N/A	Thailand	Maela	carriage	nasopharynx
3	No	N/A	Thailand	Maela	carriage	nasopharynx
3	No	N/A	Thailand	Maela	carriage	nasopharynx
3	No	N/A	Thailand	Maela	carriage	nasopharynx
3	No	N/A	Thailand	Maela	carriage	nasopharynx
3	No	N/A	Thailand	Maela	carriage	nasopharynx
3	No	N/A	United Kingdom	unknown	disease	unknown
3	No	N/A	Netherlands	unknown	carriage	nasopharynx
3	No	N/A	Netherlands	unknown	carriage	nasopharynx
3	No	N/A	Bolivia	unknown	carriage	nasopharynx
3	No	N/A	USA	Pennsylvania	disease	nasopharynx
3	No	N/A	United Kingdom	Scotland	disease	blood
3	No	N/A	United Kingdom	Scotland	disease	CSF
3	No	N/A	United Kingdom	Oxford	carriage	nasopharynx
3	No	N/A	United Kingdom	Southampton	carriage	nasopharynx
3	No	N/A	United Kingdom	Southampton	carriage	nasopharynx
3	No	N/A	United Kingdom	Southampton	carriage	nasopharynx
3	No	N/A	United Kingdom	Southampton	carriage	nasopharynx
3	No	N/A	United Kingdom	Southampton	carriage	nasopharynx
3	No	N/A	United Kingdom	Southampton	carriage	nasopharynx
3	No	N/A	United Kingdom	Southampton	carriage	nasopharynx
3	No	N/A	United Kingdom	Southampton	carriage	nasopharynx
3	No	N/A	Iceland	unknown	disease	blood
3	No	N/A	Iceland	unknown	disease	blood
3	No	N/A	Iceland	Vík	disease	blood
3	No	N/A	Iceland	Reykjavík	disease	blood
3	No	N/A	Iceland	unknown	disease	blood
3	No	N/A	Iceland	Kópavogur	disease	blood
3	No	N/A	Iceland	Reykjavík	disease	middle ear fluid
3	No	N/A	Iceland	Reykjavík	disease	middle ear fluid
3	No	N/A	Iceland	Mosfellsbær	disease	middle ear fluid
3	No	N/A	Iceland	Reykjavík	disease	middle ear fluid
3	No	N/A	Iceland	Hafnarfjörður	disease	middle ear fluid
3	No	N/A	Iceland	Reykjavík	disease	middle ear fluid
3	No	N/A	Iceland	Reykjavík	disease	middle ear fluid
3	No	N/A	Iceland	Reykjavík	disease	middle ear fluid
3	No	N/A	Iceland	Reykjavík	disease	middle ear fluid
3	No	N/A	Iceland	Reykjavík	disease	middle ear fluid
3	No	N/A	Iceland	Reykjavík	disease	middle ear fluid
3	No	N/A	Iceland	Reykjavík	disease	sputum
3	No	N/A	Iceland	Reykjavík	disease	sputum
3	No	N/A	Iceland	Reykjavík	disease	sputum
3	No	N/A	Iceland	Reykjavík	disease	sputum
3	No	N/A	Iceland	Reykjavík	disease	sputum
3	No	N/A	Iceland	Reykjavík	disease	sputum
3	No	N/A	Iceland	Garðabær	disease	sputum
3	No	N/A	Iceland	Reykjavík	disease	sputum
3	No	N/A	Iceland	Snæfellsbær	disease	sputum
3	No	N/A	Iceland	Reykjavík	carriage	nasopharynx
3	No	N/A	Iceland	Reykjavík	carriage	nasopharynx
3	No	N/A	Iceland	Reykjavík	carriage	nasopharynx
3	No	N/A	Iceland	Reykjavík	carriage	nasopharynx
3	No	N/A	Iceland	Hafnarfjörður	carriage	nasopharynx

3	No	N/A	Iceland	Kópavogur	carriage	nasopharynx
3	No	N/A	Iceland	Hafnarfjörður	carriage	nasopharynx
3	No	N/A	Iceland	Hafnarfjörður	carriage	nasopharynx
3	No	N/A	Iceland	Hafnarfjörður	carriage	nasopharynx
3	No	N/A	Iceland	Hafnarfjörður	carriage	nasopharynx
3	No	N/A	Iceland	Kópavogur	carriage	nasopharynx
3	No	N/A	Iceland	Kópavogur	carriage	nasopharynx
3	No	N/A	Iceland	Kópavogur	carriage	nasopharynx
3	No	N/A	Iceland	Kópavogur	carriage	nasopharynx
3	No	N/A	Iceland	Reykjavík	carriage	nasopharynx
3	No	N/A	Iceland	Reykjavík	carriage	nasopharynx
3	No	N/A	Iceland	Hafnarfjörður	carriage	nasopharynx
3	No	N/A	Iceland	unknown	carriage	nasopharynx
3	No	N/A	Iceland	Hafnarfjörður	carriage	nasopharynx
3	No	N/A	Iceland	Kópavogur	carriage	nasopharynx
3	No	N/A	Iceland	unknown	carriage	nasopharynx
3	No	N/A	Iceland	Hafnarfjörður	carriage	nasopharynx
3	No	N/A	Iceland	Kópavogur	carriage	nasopharynx
3	No	N/A	Iceland	Kópavogur	carriage	nasopharynx
3	No	N/A	Iceland	Kópavogur	carriage	nasopharynx
3	No	N/A	Iceland	Kópavogur	carriage	nasopharynx
3	No	N/A	Iceland	Reykjavík	carriage	nasopharynx
3	No	N/A	Iceland	Borgarnes	disease	lung aspirate
3	No	N/A	Iceland	Kópavogur	disease	middle ear fluid
3	No	N/A	Iceland	Reykjavík	disease	middle ear fluid
3	No	N/A	Iceland	Garðabær	disease	sputum
3	No	N/A	Iceland	Hafnarfjörður	carriage	nasopharynx
3	No	N/A	Iceland	Kópavogur	carriage	nasopharynx
3	No	N/A	Iceland	unknown	carriage	nasopharynx

Year	Publication	Reference	vaccine status
1973	Historical	this publication	pre
1971	Historical	this publication	pre
1961	Historical	this publication	pre
1962	Historical	this publication	pre
1961	Historical	this publication	pre
1961	Historical	this publication	pre
2001	Massachusetts	PMID: 23644493	pre
2001	Massachusetts	PMID: 23644493	pre
2001	Massachusetts	PMID: 23644493	pre
unknown	Genbank (336)	N/A	unknown
1993-1995	Croucher Serotype 3	PMID: 24130509	pre
1998-2001	Croucher Serotype 3	PMID: 24130509	pre
1998-2001	Croucher Serotype 3	PMID: 24130509	pre
2001	Croucher Serotype 3	PMID: 24130509	pre
2000	Croucher Serotype 3	PMID: 24130509	pre
2001	Croucher Serotype 3	PMID: 24130509	pre
1993	Croucher Serotype 3	PMID: 24130509	pre
1994	Croucher Serotype 3	PMID: 24130509	pre
1995	Croucher Serotype 3	PMID: 24130509	pre
1994	Croucher Serotype 3	PMID: 24130509	pre
1994	Croucher Serotype 3	PMID: 24130509	pre
1995	Croucher Serotype 3	PMID: 24130509	pre
1994	Croucher Serotype 3	PMID: 24130509	pre
1995	Croucher Serotype 3	PMID: 24130509	pre
1993-1995	Croucher Serotype 3	PMID: 24130509	pre
1993-1995	Croucher Serotype 3	PMID: 24130509	pre
1993-1995	Croucher Serotype 3	PMID: 24130509	pre
1993-1995	Croucher Serotype 3	PMID: 24130509	pre
1995	Croucher Serotype 3	PMID: 24130509	pre
1993-1995	Croucher Serotype 3	PMID: 24130509	pre
1997	Croucher Serotype 3	PMID: 24130509	pre
1995	Croucher Serotype 3	PMID: 24130509	pre
2001	Croucher Serotype 3	PMID: 24130509	pre
1998	Croucher Serotype 3	PMID: 24130509	pre
1996	Croucher Serotype 3	PMID: 24130509	pre
2002	Croucher Serotype 3	PMID: 24130509	pre
2003	Croucher Serotype 3	PMID: 24130509	pre
2000	Croucher Serotype 3	PMID: 24130509	pre
2001	Croucher Serotype 3	PMID: 24130509	pre
2003	Croucher Serotype 3	PMID: 24130509	pre
2001	Croucher Serotype 3	PMID: 24130509	pre
2003	Croucher Serotype 3	PMID: 24130509	pre
2000	Croucher Serotype 3	PMID: 24130509	pre
2000	Croucher Serotype 3	PMID: 24130509	pre
2001	Croucher Serotype 3	PMID: 24130509	pre
2003	Croucher Serotype 3	PMID: 24130509	pre
1995	Croucher Serotype 3	PMID: 24130509	pre
2003	Croucher Serotype 3	PMID: 24130509	pre
2002	Croucher Serotype 3	PMID: 24130509	pre
2003	Croucher Serotype 3	PMID: 24130509	pre
2000	Croucher Serotype 3	PMID: 24130509	pre
2001	Croucher Serotype 3	PMID: 24130509	pre
1999	Croucher Serotype 3	PMID: 24130509	pre
2003	Croucher Serotype 3	PMID: 24130509	pre
2004	Croucher Serotype 3	PMID: 24130509	pre
2000	Croucher Serotype 3	PMID: 24130509	pre
2002	Croucher Serotype 3	PMID: 24130509	pre
1999	Croucher Serotype 3	PMID: 24130509	pre

1998-2001	Croucher Serotype 3	PMID: 24130509	pre
1998	Croucher Serotype 3	PMID: 24130509	pre
1999	Croucher Serotype 3	PMID: 24130509	pre
1999	Croucher Serotype 3	PMID: 24130509	pre
1999	Croucher Serotype 3	PMID: 24130509	pre
1999	Croucher Serotype 3	PMID: 24130509	pre
1999	Croucher Serotype 3	PMID: 24130509	pre
2000	Croucher Serotype 3	PMID: 24130509	pre
2000	Croucher Serotype 3	PMID: 24130509	pre
2000	Croucher Serotype 3	PMID: 24130509	pre
2000	Croucher Serotype 3	PMID: 24130509	pre
1998	Croucher Serotype 3	PMID: 24130509	pre
2000	Croucher Serotype 3	PMID: 24130509	pre
2001	Croucher Serotype 3	PMID: 24130509	pre
2001	Croucher Serotype 3	PMID: 24130509	pre
1998	Croucher Serotype 3	PMID: 24130509	pre
1998	Croucher Serotype 3	PMID: 24130509	pre
1998	Croucher Serotype 3	PMID: 24130509	pre
1998	Croucher Serotype 3	PMID: 24130509	pre
2000	Croucher Serotype 3	PMID: 24130509	pre
2001	Croucher Serotype 3	PMID: 24130509	pre
2001	Croucher Serotype 3	PMID: 24130509	pre
2007	Croucher Serotype 3	PMID: 24130509	pre
1997	Croucher Serotype 3	PMID: 24130509	pre
1997	Croucher Serotype 3	PMID: 24130509	pre
2003	Croucher Serotype 3	PMID: 24130509	pre
2006	Croucher Serotype 3	PMID: 24130509	pre
2004	Genbank (336)	N/A	post
2007	Massachusetts	PMID: 23644493	post
2007	Massachusetts	PMID: 23644493	post
2004	Massachusetts	PMID: 23644493	post
2004	Massachusetts	PMID: 23644493	post
2004	Massachusetts	PMID: 23644493	post
2004	Massachusetts	PMID: 23644493	post
2004	Massachusetts	PMID: 23644493	post
2004	Massachusetts	PMID: 23644493	post
2008	Maela	PMID: 24509479	pre
2009	Maela	PMID: 24509479	pre
2009	Maela	PMID: 24509479	pre
2009	Maela	PMID: 24509479	pre
2008	Maela	PMID: 24509479	pre
2009	Maela	PMID: 24509479	pre
2009	Maela	PMID: 24509479	pre
2009	Maela	PMID: 24509479	pre
2009	Maela	PMID: 24509479	pre
2009	Maela	PMID: 24509479	pre
2009	Maela	PMID: 24509479	pre
2008	Maela	PMID: 24509479	pre
2009	Maela	PMID: 24509479	pre
2009	Maela	PMID: 24509479	pre
2008	Maela	PMID: 24509479	pre
2009	Maela	PMID: 24509479	pre
2008	Maela	PMID: 24509479	pre
2008	Maela	PMID: 24509479	pre
2010	Maela	PMID: 24509479	pre
2008	Maela	PMID: 24509479	pre
2010	Maela	PMID: 24509479	pre
2008	Maela	PMID: 24509479	pre
2008	Maela	PMID: 24509479	pre

2008	Maela	PMID: 24509479	pre
2008	Maela	PMID: 24509479	pre
2008	Maela	PMID: 24509479	pre
2008	Maela	PMID: 24509479	pre
2008	Maela	PMID: 24509479	pre
2008	Maela	PMID: 24509479	pre
2008	Maela	PMID: 24509479	pre
2008	Maela	PMID: 24509479	pre
2008	Maela	PMID: 24509479	pre
2008	Maela	PMID: 24509479	pre
2008	Maela	PMID: 24509479	pre
2008	Maela	PMID: 24509479	pre
2002	Genbank (336)	N/A	pre
2003	Genbank (336)	N/A	pre
2003	Genbank (336)	N/A	pre
2007	Genbank (336)	N/A	pre
unknown	Genbank (336)	N/A	unknown
1999	Genbank (336)	N/A	pre
1999	Genbank (336)	N/A	pre
2001	Genbank (336)	N/A	pre
2006-2011	Gladstone UK PCV	PMID: 25776920	post
2006-2011	Gladstone UK PCV	PMID: 25776920	post
2006-2011	Gladstone UK PCV	PMID: 25776920	post
2006-2011	Gladstone UK PCV	PMID: 25776920	post
2006-2011	Gladstone UK PCV	PMID: 25776920	post
2006-2011	Gladstone UK PCV	PMID: 25776920	post
2006-2011	Gladstone UK PCV	PMID: 25776920	post
2006-2011	Gladstone UK PCV	PMID: 25776920	post
2010	VICE	this publication	pre
2010	VICE	this publication	pre
2010	VICE	this publication	pre
2011	VICE	this publication	pre
2011	VICE	this publication	pre
2012	VICE	this publication	post
2009	VICE	this publication	pre
2009	VICE	this publication	pre
2009	VICE	this publication	pre
2009	VICE	this publication	pre
2011	VICE	this publication	pre
2011	VICE	this publication	pre
2011	VICE	this publication	pre
2011	VICE	this publication	pre
2011	VICE	this publication	pre
2012	VICE	this publication	post
2012	VICE	this publication	post
2009	VICE	this publication	pre
2009	VICE	this publication	pre
2010	VICE	this publication	pre
2010	VICE	this publication	pre
2010	VICE	this publication	pre
2011	VICE	this publication	pre
2012	VICE	this publication	post
2012	VICE	this publication	post
2012	VICE	this publication	post
2009	VICE	this publication	pre
2009	VICE	this publication	pre
2009	VICE	this publication	pre
2009	VICE	this publication	pre
2009	VICE	this publication	pre

2009	VICE	this publication	pre
2010	VICE	this publication	pre
2010	VICE	this publication	pre
2010	VICE	this publication	pre
2010	VICE	this publication	pre
2010	VICE	this publication	pre
2010	VICE	this publication	pre
2010	VICE	this publication	pre
2010	VICE	this publication	pre
2010	VICE	this publication	pre
2010	VICE	this publication	pre
2011	VICE	this publication	pre
2011	VICE	this publication	pre
2011	VICE	this publication	pre
2011	VICE	this publication	pre
2011	VICE	this publication	pre
2012	VICE	this publication	post
2013	VICE	this publication	post
2013	VICE	this publication	post
2013	VICE	this publication	post
2013	VICE	this publication	post
2013	VICE	this publication	post
2013	VICE	this publication	post
2013	VICE	this publication	post
2013	VICE	this publication	post
2013	VICE	this publication	post
2014	VICE	this publication	post
2014	VICE	this publication	post
2014	VICE	this publication	post

SEROTYPE 3 ALL ISOLATES

rmlst ID	BIGS id	Name	CC	Seq_sero	wzd 46	wzd 218	ugd 3	ugd 4	ugd 57	ugd 212
		3 reference			P	T	I	A	N	T
32180	N/A	65645	180	3	P	T	I	A	K	T
33263	N/A	72511	180	3	P	T	I	A	K	T
32211	N/A	369820	180	3	P	T	I	A	K	T
32849	N/A	BHN035	180	3	P	T	I	A	K	T
32830	N/A	BHN037	180	3	P	T	I	A	K	T
32831	N/A	BHN167	180	3	P	T	I	A	K	T
55122	N/A	BHN341	180	3	P	T	I	A	K	T
31913	N/A	BHN369	180	3	P	T	I	A	K	T
32832	N/A	BHN383	180	3	P	T	I	A	K	T
31920	N/A	BHN413	180	3	P	T	I	A	K	T
40058	N/A	BHN570	180	3	P	T	I	A	K	T
31185	N/A	BHN571	180	3	P	T	I	A	K	T
123296	N/A	BHN572	180	3	P	T	I	A	K	T
123297	N/A	BHN573	180	3	P	T	I	A	K	T
18528	N/A	BHN574	180	3	P	T	I	A	K	T
18602	N/A	BHN575	180	3	P	T	I	A	K	T
18573	N/A	BHN577	180	3	P	T	I	A	K	T
32845	N/A	BHN578	180	3	P	T	I	A	K	T
32847	N/A	BHN579	180	3	P	T	I	A	K	T
31921	N/A	BHN580	180	3	P	T	I	A	K	T
33816	N/A	BHN582	180	3	P	T	I	A	K	T
31922	N/A	BHN583	180	3	P	T	I	A	K	T
44810	N/A	BHN584	180	3	P	T	I	A	K	T
32850	N/A	BHN585	180	3	P	T	I	A	K	T
31923	N/A	BHN586	180	3	P	T	I	A	K	T
115781	N/A	BHN587	180	3	P	T	I	A	K	T
33815	N/A	BHN588	180	3	P	T	I	A	K	T
123257	N/A	BHN589	180	3	P	T	I	A	K	T
31924	N/A	BHN590	180	3	P	T	I	A	K	T
18425	N/A	BHN591	180	3	P	T	I	A	K	T
123298	N/A	BHN592	180	3	P	T	I	A	K	T
123299	N/A	BHN593	180	3	P	T	I	A	K	T
32853	N/A	BHN594	180	3	P	T	I	A	K	T
31926	N/A	BHN595	180	3	P	T	I	A	K	T
18461	N/A	BHN596	180	3	P	T	I	A	K	T
31927	N/A	BHN597	180	3	P	T	I	A	K	T
32854	N/A	BHN598	180	3	P	T	I	A	K	T
32851	N/A	BHN599	180	3	P	T	I	A	K	T
31925	N/A	BHN600	180	3	P	T	I	A	K	T
32852	N/A	BHN601	180	3	P	T	I	A	K	T
40045	N/A	BHN602	180	3	P	T	I	A	K	T
32825	N/A	BHN603	180	3	P	T	I	A	K	T
32826	N/A	BHN604	180	3	P	T	I	A	K	T
32827	N/A	BHN605	180	3	P	T	I	A	K	T
31909	N/A	BHN606	180	3	P	T	I	A	K	T
32828	N/A	BHN607	180	3	P	T	I	A	K	T
31910	N/A	BHN608	180	3	P	T	I	A	K	T
33793	N/A	BHN609	180	3	P	T	I	A	K	T
33794	N/A	BHN610	180	3	P	T	I	A	K	T
18436	N/A	BHN611	180	3	P	T	I	A	K	T
32824	N/A	BHN612	180	3	P	T	I	A	K	T
33795	N/A	BHN616	180	3	P	T	I	A	K	T
33797	N/A	BHN621	180	3	P	T	I	A	K	T
33798	N/A	BHN622	180	3	P	T	I	A	K	T
32829	N/A	BHN623	180	3	P	T	I	A	K	T
33796	N/A	BHN624	180	3	P	T	I	A	K	T
31911	N/A	BHN625	180	3	P	T	I	A	K	T
33799	N/A	BHN626	180	3	P	T	I	A	K	T
44808	N/A	BHN628	180	3	P	T	I	A	K	T
33801	N/A	BHN629	180	3	P	T	I	A	K	T
32834	N/A	BHN630	180	3	P	T	I	A	K	T
32835	N/A	BHN631	180	3	P	T	I	A	K	T

33802	N/A	BHN632	180	3	P	T	I	A	K	T
32836	N/A	BHN633	180	3	P	T	I	A	K	T
33803	N/A	BHN636	180	3	P	T	I	A	K	T
32837	N/A	BHN637	180	3	P	T	I	A	K	T
32833	N/A	BHN638	180	3	P	T	I	A	K	T
33800	N/A	BHN640	180	3	P	T	I	A	K	T
123295	N/A	BHN642	180	3	P	T	I	A	K	T
44142	N/A	BHN643	180	3	P	T	I	A	K	T
33805	N/A	BHN644	180	3	P	T	I	A	K	T
33806	N/A	BHN645	180	3	P	T	I	A	K	T
40046	N/A	BHN646	180	3	P	T	I	A	K	T
33807	N/A	BHN647	180	3	P	T	I	A	K	T
32838	N/A	BHN650	180	3	P	T	I	A	K	I
33808	N/A	BHN651	180	3	P	T	I	A	K	T
33804	N/A	BHN654	180	3	P	T	I	A	K	T
44141	N/A	BHN657	180	3	P	T	I	A	K	T
N/A	1146	GA19690	180	3	P	T	I	A	K	T
32478	N/A	MD5090	180	3	P	T	I	A	K	T
32499	N/A	ND6097	180	3	P	T	I	A	K	T
32076	N/A	R34-3078	180	3	P	T	I	A	K	T
32081	N/A	R34-3084	180	3	P	T	I	A	K	T
31353	N/A	R34-3101	180	3	P	T	I	A	K	T
32141	N/A	R34-3190	180	3	P	T	I	A	K	T
32147	N/A	R34-3202	180	3	P	T	I	A	K	T
31413	N/A	R34-3217	180	3	P	T	I	A	K	T
32977	N/A	SMRU1059	180	3	P	T	I	A	K	T
111535	N/A	SMRU1405	180	3	P	T	I	A	K	T
109846	N/A	SMRU1455	180	3	P	T	I	A	K	T
109687	N/A	SMRU338	180	3	P	T	I	A	K	T
115479	N/A	SMRU374	180	3	P	T	I	A	K	T
N/A	1195	Sp02-1198	180	3	P	T	I	A	K	T
N/A	1110	Sp03-4156	180	3	P	T	I	A	K	T
N/A	1111	Sp03-4183	180	3	P	T	I	A	K	T
N/A	1194	Sp07-2838	180	3	P	T	I	A	K	T
N/A	1107	SP3-BS71	180	3	P	T	I	A	K	T
N/A	1112	Sp99_4038	180	3	P	T	I	A	K	T
N/A	1113	Sp99_4039	180	3	P	T	I	A	K	T
N/A	317	SPnOXC141	180	3	P	T	I	A	K	T
121379	N/A	UoS0103	180	3	P	T	I	A	K	T
55921	N/A	UoS0272	180	3	P	T	I	A	K	T
123245	N/A	UoS2370	180	3	P	T	I	A	K	T
121360	N/A	UoS3033	180	3	P	T	I	A	K	T
121377	N/A	UoS3205	180	3	P	T	I	A	K	T
123225	N/A	UoS4027	180	3	P	T	I	A	K	T
32916	N/A	UoS4133	180	3	P	T	I	A	K	T
123209	N/A	UoS5023	180	3	P	T	I	A	K	T
N/A	499	VICE0178	180	3	P	T	I	A	K	T
N/A	500	VICE0179	180	3	P	T	I	A	K	T
N/A	501	VICE0180	180	3	P	T	I	A	K	T
N/A	534	VICE0213	180	3	P	T	I	A	K	T
N/A	553	VICE0232	180	3	P	T	I	A	K	T
N/A	579	VICE0257	180	3	P	T	I	A	K	T
N/A	613	VICE0291	180	3	P	T	I	A	K	T
N/A	618	VICE0296	180	3	P	T	I	A	K	T
N/A	642	VICE0320	180	3	P	T	I	A	K	T
N/A	644	VICE0322	180	3	P	T	I	A	K	T
N/A	808	VICE0486	180	3	P	T	I	A	K	T
N/A	838	VICE0516	180	3	P	T	I	A	K	T
N/A	858	VICE0536	180	3	P	T	I	A	K	T
N/A	871	VICE0549	180	3	P	T	I	A	K	T
N/A	883	VICE0561	180	3	P	T	I	A	K	T
N/A	927	VICE0605	180	3	P	T	I	A	K	T
N/A	939	VICE0617	180	3	P	T	I	A	K	T
N/A	1283	VICE0689	180	3	P	T	I	A	K	T
N/A	1312	VICE0718	180	3	P	T	I	A	K	T
N/A	1317	VICE0723	180	3	P	T	I	A	K	T
N/A	1338	VICE0744	180	3	P	T	I	A	K	T

N/A	1358	VICE0764	180	3	P	T	I	A	K	T
N/A	1387	VICE0793	180	3	P	T	I	A	K	T
N/A	1389	VICE0795	180	3	P	T	I	A	K	T
N/A	345	VICE0852	180	3	P	T	I	A	K	T
N/A	349	VICE0853	180	3	P	T	I	A	K	T
N/A	370	VICE0858	180	3	P	T	I	A	K	T
N/A	436	VICE0882	180	3	P	T	I	A	K	T
N/A	1516	VICE0965	180	3	P	T	I	A	K	T
N/A	1575	VICE1024	180	3	P	T	I	A	K	T
N/A	1603	VICE1052	180	3	P	T	I	A	K	T
N/A	1629	VICE1078a	180	3	P	T	I	A	K	T
N/A	1632	VICE1081	180	3	P	T	I	A	K	T
N/A	1637	VICE1086	180	3	P	T	I	A	K	T
N/A	1666	VICE1115a	180	3	P	T	I	A	K	T
N/A	1668	VICE1117	180	3	P	T	I	A	K	T
N/A	1672	VICE1121	180	3	P	T	I	A	K	T
N/A	1677	VICE1126	180	3	P	T	I	A	K	T
N/A	2040	VICE1162	180	3	P	T	I	A	K	T
N/A	2052	VICE1174	180	3	P	T	I	A	K	T
N/A	2066	VICE1188	180	3	P	T	I	A	K	T
N/A	2490	VICE1189b	180	3	P	T	I	A	K	T
N/A	2084	VICE1206	180	3	P	T	I	A	K	T
N/A	2126	VICE1248	180	3	P	T	I	A	K	T
N/A	2508	VICE1276b	180	3	P	T	I	A	K	T
N/A	2208	VICE1330	180	3	P	T	I	A	K	T
N/A	2356	VICE1478	180	3	P	T	I	A	K	T
N/A	2366	VICE1488a	180	3	P	T	I	A	K	T
N/A	2376	VICE1498	180	3	P	T	I	A	K	T
N/A	2424	VICE1546	180	3	P	T	I	A	K	T
N/A	2460	VICE1582	180	3	P	T	I	A	K	T
N/A	2551	VICE1598	180	3	P	T	I	A	K	T
N/A	2563	VICE1610	180	3	P	T	I	A	K	T
N/A	2575	VICE1622	180	3	P	T	I	A	K	T
N/A	2646	VICE1693	180	3	P	T	I	A	K	T
N/A	2725	VICE1768a	180	3	P	T	I	A	K	T
N/A	2736	VICE1778	180	3	P	T	I	A	K	T
N/A	2829	VICE1864	180	3	P	T	I	A	K	T
N/A	1241	VICE0647	260	3	P	T	I	A	K	T
N/A	10	3/2	378	3	P	T	T	A	K	T
N/A	11	3/3	378	3	P	T	I	G	K	T
N/A	12	3/4	378	3	P	T	I	A	K	T
N/A	15	3/7	378	3	P	T	I	A	K	T
N/A	16	3/8	378	3	P	T	I	A	K	T
N/A	1264	VICE0670	378	3	P	T	I	A	K	T
83244	N/A	SMRU174	458	3	P	T	I	A	K	T
109958	N/A	SMRU2421	458	3	P	T	I	A	K	T
82593	N/A	SMRU2628	458	3	P	T	I	A	K	T
109688	N/A	SMRU351	458	3	P	T	I	A	K	T
83223	N/A	SMRU354	458	3	P	T	I	A	K	T
33908	N/A	SMRU439	458	3	P	T	I	A	K	T
33732	N/A	SMRU543	458	3	P	T	I	A	K	T
N/A	2889	3/6	1024	3	P	T	I	A	K	T
109679	N/A	SMRU128	1765/8799	3	S	T	I	A	K	T
115541	N/A	SMRU1131	Sing4389	3	P	T	I	A	K	T
82242	N/A	SMRU1202	Sing4389	3	P	T	I	A	K	T
82243	N/A	SMRU1204	Sing4389	3	P	T	I	A	K	T
115522	N/A	SMRU1402	Sing4389	3	P	T	I	A	K	T
113807	N/A	SMRU1432	Sing4389	3	P	T	I	A	K	T
82302	N/A	SMRU1434	Sing4389	3	P	T	I	A	K	T
33112	N/A	SMRU1485	Sing4389	3	P	T	I	A	K	T
111451	N/A	SMRU1773	Sing4389	3	P	T	I	A	K	T
111445	N/A	SMRU1814	Sing4389	3	P	T	I	A	K	T
83263	N/A	SMRU196	Sing4389	3	P	T	I	A	K	T
82379	N/A	SMRU2038	Sing4389	3	P	T	I	A	K	T
115502	N/A	SMRU207	Sing4389	3	P	T	I	A	K	T
113095	N/A	SMRU222	Sing4389	3	P	T	I	A	K	T
115513	N/A	SMRU254	Sing4389	3	P	T	I	A	K	T

55131	N/A	SMRU276	Sing4389	3	P	T	I	A	K	T
32925	N/A	SMRU293	Sing4389	3	P	T	I	A	K	T
32926	N/A	SMRU294	Sing4389	3	P	T	I	A	K	T
40162	N/A	SMRU364	Sing4389	3	P	T	I	A	K	T
40167	N/A	SMRU376	Sing4389	3	P	T	I	A	K	T
113760	N/A	SMRU498	Sing4389	3	P	T	I	A	K	T
115519	N/A	SMRU573	Sing4389	3	P	T	I	A	K	T
40215	N/A	SMRU650	Sing4389	3	P	T	I	A	K	T
N/A	1196	A45	Sing6934	3	P	I	I	A	K	T

			wchE									galU
284	336	375	20	25	28	37	305	341	342	367	379	58
Q	K	N	F	V	L	H	R	A	I	H	V	E
Q	K	H	F	V	L	H	R	T	I	N	V	G
Q	K	H	F	V	L	H	R	T	I	N	V	G
Q	K	H	F	V	L	H	R	T	I	N	V	G
Q	K	H	F	V	L	H	R	T	I	N	V	G
Q	K	H	F	V	L	H	R	T	I	N	V	G
Q	K	H	F	V	L	H	R	T	I	N	V	G
Q	K	H	F	V	L	H	R	T	I	N	V	G
Q	K	H	F	V	L	H	R	T	I	N	V	G
Q	K	H	F	V	L	H	R	T	I	N	V	G
Q	K	H	F	V	L	H	R	T	I	N	V	G
Q	K	H	F	V	L	H	R	T	I	N	V	G
Q	K	H	F	V	L	H	R	T	I	N	V	G
Q	K	H	F	V	L	H	R	T	I	N	V	G
Q	K	H	F	V	L	H	R	T	I	N	V	G
Q	K	H	F	V	L	H	R	T	I	N	V	G
Q	K	H	F	V	L	H	R	T	I	N	V	G
Q	K	H	F	V	L	H	R	T	I	N	V	G
Q	K	H	F	V	L	H	R	T	I	N	V	G
Q	K	H	F	V	L	H	R	T	I	N	V	G
Q	K	H	F	V	L	H	R	T	I	N	V	G
Q	K	H	F	V	L	H	R	T	I	N	V	G
Q	K	H	F	V	L	H	R	T	I	N	V	G
Q	K	H	F	V	L	H	R	T	I	N	V	G
Q	K	H	F	V	L	H	R	T	I	N	V	G
Q	K	H	F	V	L	H	R	T	I	N	V	G
Q	K	H	F	V	L	H	R	T	I	N	V	G
Q	K	H	F	V	L	H	R	T	I	N	V	G
Q	K	H	F	V	L	H	R	T	I	N	V	G
Q	K	H	F	V	L	H	R	T	I	N	V	G
Q	K	H	F	V	L	H	R	T	I	N	V	G
Q	K	H	F	V	L	H	R	T	I	N	V	G
Q	K	H	F	V	L	H	R	T	I	N	V	G
Q	K	H	F	V	L	H	R	T	I	N	V	G
Q	K	H	F	V	L	H	R	T	I	N	V	G
Q	K	H	F	V	L	H	R	T	I	N	V	G
Q	K	H	F	V	L	H	R	T	I	N	V	G
Q	K	H	F	V	L	H	R	T	I	N	V	G
Q	K	H	F	V	L	H	R	T	I	N	V	G
Q	K	H	F	V	L	H	R	T	I	N	V	G
Q	K	H	F	V	L	H	R	T	I	N	V	G
Q	K	H	F	V	L	H	R	T	I	N	V	G
Q	K	H	F	V	L	H	R	T	I	N	V	G
Q	K	H	F	V	L	H	R	T	I	N	V	G
Q	K	H	F	V	L	H	R	T	I	N	V	G
Q	K	H	F	V	L	H	R	T	I	N	V	G
Q	K	H	F	V	L	H	R	T	I	N	V	G
Q	K	H	F	V	L	H	R	T	N	N	V	G
Q	K	H	F	V	L	H	R	T	I	N	V	G
Q	K	H	F	V	L	H	R	T	I	N	V	G
Q	K	H	F	V	L	H	R	T	I	N	V	G
Q	K	H	F	V	L	H	R	T	I	N	V	G
Q	K	H	F	V	L	H	R	T	I	N	V	G
Q	K	H	F	V	L	H	R	T	I	N	V	G
Q	K	H	F	V	L	H	R	T	I	N	V	G
Q	K	H	F	V	L	H	R	T	I	N	V	G
Q	K	H	F	V	L	H	R	T	I	N	V	G
Q	K	H	F	V	L	H	R	T	I	N	V	G
Q	K	H	F	V	L	Y	R	T	I	N	V	G
Q	K	H	F	V	L	H	R	T	I	N	V	G
Q	K	H	F	V	L	H	R	T	I	N	V	G
Q	K	H	F	V	L	H	R	T	I	N	V	G
Q	K	H	F	V	L	H	R	T	I	N	V	G

Q	K	H	F	V	L	H	R	T	I	N	V	G
Q	K	H	F	V	L	H	R	T	I	N	V	G
Q	K	H	F	V	L	H	R	T	I	N	V	G
Q	K	H	F	V	L	H	R	T	I	N	V	G
Q	K	H	F	V	L	H	R	T	I	N	V	G
Q	K	H	F	V	L	H	R	T	I	N	V	G
Q	K	H	F	V	L	H	R	T	I	N	V	G
Q	K	H	F	V	L	H	R	T	I	N	V	G
Q	K	H	F	V	L	H	R	T	I	N	V	G
Q	K	H	F	V	L	H	R	T	I	N	V	G
Q	K	H	F	V	L	H	R	T	I	N	V	G
Q	K	H	F	V	L	H	R	T	I	N	V	G
Q	E	H	F	V	L	H	R	T	I	N	V	G
Q	K	H	F	V	L	H	R	T	I	N	V	G
Q	K	H	F	V	L	H	R	T	I	N	V	G
Q	K	H	F	V	L	H	R	T	I	N	V	G
Q	K	H	F	V	L	H	R	T	I	N	V	G
Q	K	H	F	V	L	H	R	T	I	N	V	G
Q	K	H	L	V	L	H	R	T	I	N	V	G
Q	K	H	F	V	L	H	R	T	I	N	V	G
Q	K	H	F	V	L	H	R	T	I	N	V	G
Q	K	H	F	V	L	H	R	T	I	N	V	G
Q	K	H	F	V	L	H	R	T	I	N	V	G
Q	K	H	F	V	L	H	R	T	I	N	V	G
Q	K	H	F	V	L	H	R	T	I	N	V	G
Q	K	H	F	V	L	H	R	T	I	N	V	G
Q	K	H	F	V	L	H	R	T	I	N	V	G
Q	K	H	F	V	L	H	R	T	I	N	V	G
Q	K	H	F	V	L	H	R	T	I	N	V	G
Q	K	H	F	V	L	H	R	T	I	N	V	G
Q	K	H	F	V	L	H	R	T	I	N	V	G
Q	K	H	F	V	L	H	R	T	I	N	V	G
Q	K	H	F	V	L	H	R	T	I	N	V	G
Q	K	H	F	V	L	H	R	T	I	N	V	G
Q	K	H	F	V	L	H	R	T	I	N	V	G
Q	K	H	F	V	L	H	R	T	I	N	V	G
Q	K	H	F	V	L	H	R	T	I	N	V	G
Q	K	H	F	V	L	H	R	T	I	N	V	G
Q	K	H	F	V	L	H	R	T	I	N	V	G
Q	K	H	F	V	L	H	R	T	I	N	V	G
Q	K	H	F	V	L	H	R	T	I	N	V	G
Q	K	H	F	V	L	H	R	T	I	N	V	G
Q	K	H	F	V	L	H	R	T	I	N	V	G
Q	K	H	F	V	L	H	R	T	I	N	V	G
Q	K	H	F	V	L	H	R	T	I	N	V	G
Q	K	H	F	V	L	H	R	T	I	N	V	G
Q	K	H	F	V	L	H	R	T	I	N	V	G
Q	K	H	F	V	L	H	R	T	I	N	V	G
Q	K	H	F	V	L	H	R	T	I	N	V	G
Q	K	H	F	V	L	H	R	T	I	N	V	G
Q	K	H	F	F	L	H	R	T	I	N	V	G
Q	K	H	F	V	L	H	R	T	I	N	V	G
Q	K	H	F	V	L	H	R	T	I	N	V	G
Q	K	H	F	V	L	H	R	T	I	N	V	G
Q	K	H	F	V	L	H	R	T	I	N	V	G
Q	K	H	F	V	L	H	R	T	I	N	V	G
Q	K	H	F	V	L	H	R	T	I	N	V	G
Q	K	H	F	V	L	H	R	T	I	N	V	G
Q	K	H	F	V	L	H	R	T	I	N	V	G
Q	K	H	F	V	L	H	R	T	I	N	V	G
Q	K	H	F	V	L	H	R	T	I	N	V	G
Q	K	H	F	V	L	H	R	T	I	N	V	G
Q	K	H	F	V	L	H	R	T	I	N	V	G
Q	K	H	F	V	L	H	R	T	I	N	V	G
Q	K	H	F	V	L	H	R	T	I	N	V	G
Q	K	H	F	V	L	H	R	T	I	N	V	G
Q	K	H	F	V	L	H	R	T	I	N	V	

Q	K	H	F	V	L	H	R	T	I	N	V	G
Q	K	H	F	V	L	H	R	T	I	N	V	G
Q	K	H	F	V	L	H	R	T	I	N	V	G
Q	K	H	F	V	L	H	R	T	I	N	V	G
Q	K	H	F	V	L	H	R	T	I	N	V	G
Q	K	H	F	V	L	H	R	T	I	N	V	G
Q	K	H	F	V	L	H	R	T	I	N	V	G
Q	K	H	F	V	L	H	R	T	I	N	V	G
Q	K	H	F	V	L	H	R	T	I	N	V	G
Q	K	H	F	V	L	H	R	T	I	N	V	G
Q	K	H	F	V	L	H	R	T	I	N	V	G
Q	K	H	F	V	L	H	R	T	I	N	V	G
Q	K	H	F	V	L	H	R	T	I	N	V	G
Q	K	H	F	V	L	H	R	T	I	N	V	G
Q	K	H	F	V	L	H	R	T	I	N	V	G
Q	K	H	F	V	L	H	R	T	I	N	V	G
Q	K	H	F	V	L	H	R	T	I	N	V	G
Q	K	H	F	V	L	H	R	T	I	N	V	G
Q	K	H	F	V	L	H	R	T	I	N	V	G
Q	K	H	F	V	L	H	R	T	I	N	V	G
Q	K	H	F	V	L	H	R	T	I	N	V	G
Q	K	H	F	V	L	H	R	T	I	N	V	G
Q	K	H	F	V	L	H	R	T	I	N	V	G
Q	K	H	F	V	L	H	R	T	I	N	V	G
Q	K	H	F	V	L	H	R	T	I	N	V	G
Q	K	H	F	V	L	H	R	T	I	N	V	G
Q	K	H	F	V	L	H	R	T	I	N	V	G
Q	K	H	F	V	L	H	R	T	I	N	V	G
Q	K	H	F	V	L	H	R	T	I	N	V	G
Q	K	H	F	V	L	H	R	T	I	N	V	G
Q	K	H	F	V	L	H	R	T	I	N	V	G
Q	K	H	F	V	L	H	R	T	I	N	V	G
Q	K	H	F	V	L	H	R	T	I	N	V	G
Q	K	H	F	V	L	H	R	T	I	N	V	G
Q	K	H	F	V	L	H	R	T	I	N	V	G
Q	K	H	F	V	L	H	R	T	I	N	V	G
Q	K	H	F	V	L	H	R	T	I	N	V	G
Q	K	N	F	V	L	H	R	T	I	N	V	G
Q	K	N	F	V	L	H	R	T	I	N	V	G
Q	K	N	F	V	L	H	R	T	I	N	V	G
Q	K	N	F	V	L	H	R	T	I	N	V	G
Q	K	N	F	V	L	H	R	T	I	N	V	G
Q	K	N	F	V	L	H	R	T	I	N	V	G
R	K	N	F	V	L	H	R	T	I	N	A	G
Q	K	N	F	V	L	H	K	T	I	N	V	E
Q	K	N	F	V	L	H	K	T	I	N	V	E
Q	K	N	F	V	L	H	K	T	I	N	V	E
Q	K	N	F	V	L	H	K	T	I	N	V	E
Q	K	N	F	V	L	H	K	T	I	N	V	E
Q	K	N	F	V	L	H	K	T	I	N	V	E
Q	K	N	F	V	L	H	K	T	I	N	V	E
Q	K	N	F	V	L	H	R	A	I	H	V	E
Q	K	N	F	V	L	H	R	T	I	N	V	E
Q	K	H	F	V	L	H	R	T	I	N	V	G
Q	K	H	F	V	L	H	R	T	I	N	V	G
Q	K	H	F	V	L	H	R	T	I	N	V	G
Q	K	H	F	V	L	H	R	T	I	N	V	G
Q	K	H	F	V	L	H	R	T	I	N	V	G
Q	K	H	F	V	L	H	R	T	I	N	V	G
Q	K	H	F	V	L	H	R	T	I	N	V	G
Q	K	H	F	V	L	H	R	T	I	N	V	G
Q	K	H	F	V	L	H	R	T	I	N	V	G
Q	K	H	F	V	L	H	R	T	I	N	V	G
Q	K	H	F	V	L	H	R	T	I	N	V	G
Q	K	H	F	V	L	H	R	T	I	N	V	G
Q	K	H	F	V	L	H	R	T	I	N	V	G
Q	K	H	F	V	L	H	R	T	I	N	V	G

Q	K	H
Q	K	H
Q	K	H
Q	K	H
Q	K	H
Q	K	H
Q	K	H
Q	K	H
Q	K	N

F	V	L	H	R	T	I	N	V
F	V	L	H	R	T	I	N	V
F	V	L	H	R	T	I	N	V
F	V	L	H	R	T	I	N	V
F	V	L	H	R	T	I	N	V
F	V	L	H	R	T	I	N	V
F	V	L	H	R	T	I	N	V
F	V	L	H	R	T	I	N	V
F	V	F	H	R	T	I	N	V

G
G
G
G
G
G
G
G
G

rmlst ID	PubMLST id	ENA	Name	ST	CC	Orig_sero
N/A	35875	ERR025268	Netherlands7F-39	191	191	7F
N/A	35840	N/A	ICE22	191	191	7F
N/A	35841	N/A	ICE23	218	218	7F
N/A	35987	N/A	GA47439	1176	218	7F
N/A	35877	N/A	CDC1087-00	191	191	7F
121465	33909	ERR045020	UoS2235	1797	1797	unknown
44823	33053	ERR044948	UoS0307	191	191	unknown
123222	34146	ERR046112	UoS3245	191	191	unknown
121365	33809	ERR046181	UoS4151	191	191	unknown
44827	33057	ERR046198	UoS4223	191	191	unknown
121364	33808	ERR047117	UoS5220	191	191	unknown
N/A	36290	N/A	7A/2	191	191	7A
N/A	15445	N/A	7C/2	7175	15	7C
N/A	35821	N/A	7F/3	191	191	7F
N/A	36292	N/A	7F/5	191	191	7F
N/A	35820	N/A	7B/3	230	230	7B
N/A	15480	N/A	7F/6	7210	218	7F
N/A	15478	N/A	7F/4	7208	191	7F
N/A	35819	N/A	7A/3	5994	Sing5994	7A
N/A	15450	N/A	7B/2	7180	66	7B
N/A	15441	N/A	7C/3	7171	15	7C
N/A	36291	N/A	7F/2	7210	218	7F
109724	28600	ERR051643	SMRU1097	230	230	7B
115544	28601	ERR054614	SMRU1162	230	230	7B
83450	28602	ERR063965	SMRU2172	1701	230	7B
44985	28603	ERR066310	SMRU2343	1701	230	7B
34186	28604	ERR064031	SMRU2526	1701	230	7B
83538	28607	ERR067858	SMRU2635	1701	230	7B
83468	28605	ERR064112	SMRU2710	1701	230	7B
83469	28606	ERR064113	SMRU2711	1701	230	7B
115592	28608	ERR064118	SMRU2716	1701	230	7B
109954	28599	ERR064131	SMRU2729	1701	230	7B
34306	28609	ERR069630	SMRU2780	1701	230	7B
82298	28612	ERR056759	SMRU1421	10386	Sing10386	7B
83318	28613	ERR054473	SMRU1525	2758	2758	7B
82078	28610	ERR039565	SMRU6	2758	2758	7B
82079	28611	ERR039566	SMRU7	2758	2758	7B
33948	28621	ERR051573	SMRU1003	3545	3545/5265	7F
32975	28622	ERR051603	SMRU1057	3545	3545/5265	7F
109799	28624	ERR054388	SMRU1295	3735	3324/5735/6853	7F
115561	28625	ERR056809	SMRU1663	3545	3545/5265	7F
83417	28626	ERR063804	SMRU1795	3545	3545/5265	7F
115503	28616	ERR050071	SMRU208	3545	3545/5265	7F
109672	28615	ERR039586	SMRU27	3545	3545/5265	7F
31973	28617	ERR047984	SMRU307	3545	3545/5265	7F
44844	28618	ERR048104	SMRU427	3545	3545/5265	7F
82151	28614	ERR048204	SMRU527	3545	3545/5265	7F
32394	29669	ERR129064	BR1113	1176	218	7A
32397	29365	ERR129067	BR1116	191	191	7A
33393	29671	ERR124224	ND6120	191	191	7A
32356	29499	ERR124292	PT8039	191	191	7A
33429	29672	ERR124312	PT8094	191	191	7A
31320	29666	ERR067961	R34-3163	191	191	7A
32158	29667	ERR069718	R34-3219	191	191	7A
32186	29363	ERR069758	95344	199	199	7C
32383	29497	ERR129047	BR1074	3284	1797	7C
33452	29498	ERR129187	ND6000	1797	1797	7C
32403	29670	ERR129073	CH2008	191	191	7F

32319	29668	ERR124228	ND6130	191	191	7F
32330	29364	ERR124243	ND6158	191	191	7F
32347	29401	ERR124276	NP7118	191	191	7F
N/A	33091	ERR388881	VICE0191	191	191	not done
N/A	34570	ERR388805	VICE0138	191	191	not done
N/A	33114	ERR388863	VICE0161	191	191	not done
N/A	34273	ERR388866	VICE0165	191	191	not done
N/A	33122	ERR388876	VICE0185	191	191	not done
N/A	33088	ERR388926	VICE0274	191	191	not done
N/A	33396	ERR470408	VICE0780	191	191	not done
N/A	33563	ERR540388	VICE1300	191	191	not done
N/A	36054	ERR755435	VICE1603	191	191	not done
N/A	34576	ERR388814	VICE0164	191	191	not done
N/A	33119	ERR388871	VICE0176	191	191	not done
N/A	34579	ERR388817	VICE0177	191	191	not done
N/A	34398	ERR388894	VICE0211	191	191	not done
N/A	33149	ERR470260	VICE0231	191	191	not done
N/A	36281	ERR755619	VICE1902	1589	191	not done

Orig_sero_method	Seq_sero	Hybrid	Hybrid type	Country	Region	carriage/disease
Quellung	7F		N/A	Netherlands	unknown	disease
Latex/PCR	7F		N/A	Iceland	unknown	disease
Latex/PCR	7F	Yes	7F/19F	Iceland	unknown	disease
unknown	7F	Yes	7F/19F	USA	Georgia	disease
unknown	7F		N/A	USA	Maryland	disease
WGS-based	7C		N/A	United Kingdom	Southampton	carriage
WGS-based	7F		N/A	United Kingdom	Southampton	carriage
WGS-based	7F		N/A	United Kingdom	Southampton	carriage
WGS-based	7F		N/A	United Kingdom	Southampton	carriage
WGS-based	7F		N/A	United Kingdom	Southampton	carriage
WGS-based	7F		N/A	United Kingdom	Southampton	carriage
Quellung	7A		N/A	Denmark	unknown	unknown
Quellung	7C		N/A	Denmark	unknown	unknown
Quellung	7F		N/A	Denmark	unknown	unknown
Quellung	7F		N/A	Denmark	unknown	unknown
Quellung	7B		N/A	France	unknown	unknown
Quellung	7F	Yes	7F/19F	Sweden	unknown	unknown
Quellung	7F		N/A	United Kingdom	unknown	unknown
Quellung	7F	Yes	7F/19F	USA	unknown	unknown
Quellung	7B		N/A	USA	unknown	unknown
Quellung	7C		N/A	USA	unknown	unknown
Quellung	7F	Yes	7F/19F	USA	unknown	unknown
latex & Quellung	7B		N/A	Thailand	Maela	Carriage
latex & Quellung	7B		N/A	Thailand	Maela	Carriage
latex & Quellung	7B		N/A	Thailand	Maela	Carriage
latex & Quellung	7B		N/A	Thailand	Maela	Carriage
latex & Quellung	7B		N/A	Thailand	Maela	Carriage
latex & Quellung	7B		N/A	Thailand	Maela	Carriage
latex & Quellung	7B		N/A	Thailand	Maela	Carriage
latex & Quellung	7B		N/A	Thailand	Maela	Carriage
latex & Quellung	7B		N/A	Thailand	Maela	Carriage
latex & Quellung	7B		N/A	Thailand	Maela	Carriage
latex & Quellung	7B		N/A	Thailand	Maela	Carriage
latex & Quellung	7C		N/A	Thailand	Maela	Carriage
latex & Quellung	7C		N/A	Thailand	Maela	Carriage
latex & Quellung	7C		N/A	Thailand	Maela	Carriage
latex & Quellung	7C		N/A	Thailand	Maela	Carriage
latex & Quellung	7F	Yes	7F/19F	Thailand	Maela	Carriage
latex & Quellung	7F	Yes	7F/19F	Thailand	Maela	Carriage
latex & Quellung	7F	Yes	7F/19F	Thailand	Maela	Carriage
latex & Quellung	7F	Yes	7F/19F	Thailand	Maela	Carriage
latex & Quellung	7F	Yes	7F/19F	Thailand	Maela	Carriage
latex & Quellung	7F	Yes	7F/19F	Thailand	Maela	Carriage
latex & Quellung	7F	Yes	7F/19F	Thailand	Maela	Carriage
latex & Quellung	7F	Yes	7F/19F	Thailand	Maela	Carriage
latex & Quellung	7F	Yes	7F/19F	Thailand	Maela	Carriage
latex & Quellung	7F	Yes	7F/19F	Thailand	Maela	Carriage
Quellung	7F	Yes	7F/19F	USA	Massachusetts	Carriage
Quellung	7F		N/A	USA	Massachusetts	Carriage
Quellung	7F		N/A	USA	Massachusetts	Carriage
Quellung	7F		N/A	USA	Massachusetts	Carriage
Quellung	7F		N/A	USA	Massachusetts	Carriage
Quellung	7F		N/A	USA	Massachusetts	Carriage
Quellung	7F		N/A	USA	Massachusetts	Carriage
Quellung	7C		N/A	USA	Massachusetts	Carriage
Quellung	7C		N/A	USA	Massachusetts	Carriage
Quellung	7C		N/A	USA	Massachusetts	Carriage
Quellung	7F		N/A	USA	Massachusetts	Carriage

Quellung	7F	N/A	USA	Massachusetts	Carriage
Quellung	7F	N/A	USA	Massachusetts	Carriage
Quellung	7F	N/A	USA	Massachusetts	Carriage
--	7F	N/A	Iceland	Búðardalur	disease
--	7F	N/A	Iceland	Egilsstaðir	disease
--	7F	N/A	Iceland	Reykjavík	disease
--	7F	N/A	Iceland	Reykjavík	disease
--	7F	N/A	Iceland	Reykjavík	disease
--	7F	N/A	Iceland	Reykjavík	disease
--	7F	N/A	Iceland	Reykjavík	disease
--	7F	N/A	Iceland	Reykjavík	carriage
--	7F	N/A	Iceland	Reykjavík	disease
--	7F	N/A	Iceland	unknown	disease
--	7F	N/A	Iceland	unknown	disease
--	7F	N/A	Iceland	unknown	disease
--	7F	N/A	Iceland	unknown	disease
--	7F	N/A	Iceland	unknown	disease
--	7F	N/A	Iceland	unknown	disease

Source	Year	Publication	Reference	vaccine status
CSF	1984	Capsule switching	PMID: 23175765	pre
blood	1993	Genbank (336)	N/A	pre
blood	1993	Genbank (336)	N/A	pre
blood	2006	Genbank (336)	N/A	post
blood	1999	Genbank (336)	N/A	pre
nasopharynx	2006-2011	Gladstone UK PCV	PMID: 25776920	unknown
nasopharynx	2006-2011	Gladstone UK PCV	PMID: 25776920	unknown
nasopharynx	2006-2011	Gladstone UK PCV	PMID: 25776920	unknown
nasopharynx	2006-2011	Gladstone UK PCV	PMID: 25776920	unknown
nasopharynx	2006-2011	Gladstone UK PCV	PMID: 25776920	unknown
nasopharynx	2006-2011	Gladstone UK PCV	PMID: 25776920	unknown
unknown	1937	Historical	this publication	pre
unknown	1939	Historical	this publication	pre
unknown	1962	Historical	this publication	pre
unknown	1962	Historical	this publication	pre
unknown	1996	Historical	this publication	pre
unknown	1996	Historical	this publication	pre
unknown	1986	Historical	this publication	pre
unknown	1968	Historical	this publication	pre
unknown	1952	Historical	this publication	pre
unknown	1971	Historical	this publication	pre
unknown	1952	Historical	this publication	pre
nasopharynx	2008	Maela	PMID: 24509479	pre
nasopharynx	2009	Maela	PMID: 24509479	pre
nasopharynx	2009	Maela	PMID: 24509479	pre
nasopharynx	2010	Maela	PMID: 24509479	pre
nasopharynx	2010	Maela	PMID: 24509479	pre
nasopharynx	2010	Maela	PMID: 24509479	pre
nasopharynx	2010	Maela	PMID: 24509479	pre
nasopharynx	2010	Maela	PMID: 24509479	pre
nasopharynx	2010	Maela	PMID: 24509479	pre
nasopharynx	2010	Maela	PMID: 24509479	pre
nasopharynx	2010	Maela	PMID: 24509479	pre
nasopharynx	2009	Maela	PMID: 24509479	pre
nasopharynx	2009	Maela	PMID: 24509479	pre
nasopharynx	2007	Maela	PMID: 24509479	pre
nasopharynx	2007	Maela	PMID: 24509479	pre
nasopharynx	2008	Maela	PMID: 24509479	pre
nasopharynx	2008	Maela	PMID: 24509479	pre
nasopharynx	2009	Maela	PMID: 24509479	pre
nasopharynx	2009	Maela	PMID: 24509479	pre
nasopharynx	2009	Maela	PMID: 24509479	pre
nasopharynx	2008	Maela	PMID: 24509479	pre
nasopharynx	2007	Maela	PMID: 24509479	pre
nasopharynx	2008	Maela	PMID: 24509479	pre
nasopharynx	2008	Maela	PMID: 24509479	pre
nasopharynx	2008	Maela	PMID: 24509479	pre
nasopharynx	2007	Massachusetts	PMID: 23644493	post
nasopharynx	2007	Massachusetts	PMID: 23644493	post
nasopharynx	2007	Massachusetts	PMID: 23644493	post
nasopharynx	2007	Massachusetts	PMID: 23644493	post
nasopharynx	2007	Massachusetts	PMID: 23644493	post
nasopharynx	2004	Massachusetts	PMID: 23644493	post
nasopharynx	2004	Massachusetts	PMID: 23644493	post
nasopharynx	2001	Massachusetts	PMID: 23644493	pre
nasopharynx	2007	Massachusetts	PMID: 23644493	post
nasopharynx	2007	Massachusetts	PMID: 23644493	post
nasopharynx	2007	Massachusetts	PMID: 23644493	post

nasopharynx	2007	Massachusetts	PMID: 23644493	post
nasopharynx	2007	Massachusetts	PMID: 23644493	post
nasopharynx	2007	Massachusetts	PMID: 23644493	post
blood	2010	VICE	this publication	post
blood	2009	VICE	this publication	post
blood	2009	VICE	this publication	post
blood	2009	VICE	this publication	post
blood	2010	VICE	this publication	post
blood	2013	VICE	this publication	post
sputum	2012	VICE	this publication	post
nasopharynx	2014	VICE	this publication	post
middle ear fluid	2013	VICE	this publication	post
blood	2009	VICE	this publication	post
CSF	2010	VICE	this publication	post
blood	2010	VICE	this publication	post
blood	2011	VICE	this publication	post
blood	2011	VICE	this publication	post
blood	2014	VICE	this publication	post

SEROTYPE 7B ALL ISOLATES

		7B reference		
N/A	30	7B/3	230	7B
N/A	2895	7B/2	66	7B
34186	N/A	SMRU2526	230	7B
34306	N/A	SMRU2780	230	7B
44985	N/A	SMRU2343	230	7B
83450	N/A	SMRU2172	230	7B
83468	N/A	SMRU2710	230	7B
83469	N/A	SMRU2711	230	7B
83538	N/A	SMRU2635	230	7B
109724	N/A	SMRU1097	230	7B
109954	N/A	SMRU2729	230	7B
115544	N/A	SMRU1162	230	7B
115592	N/A	SMRU2716	230	7B

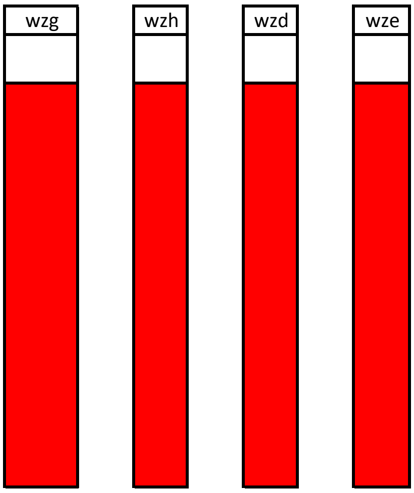

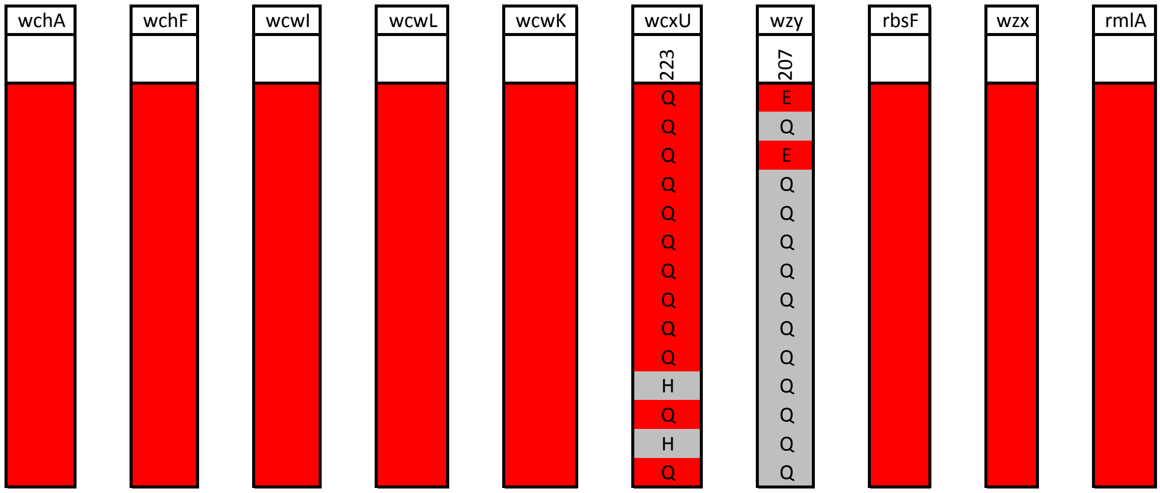

wchA
wchF
wcwI
wcwL
wcwK
wcxU
223
Q
Q
Q
Q
Q
Q
Q
Q
Q
Q
H
Q
H
Q
wzy
207
E
Q
E
Q
Q
Q
Q
Q
Q
Q
Q
Q
Q
Q
rbsF
wzx
rmlA

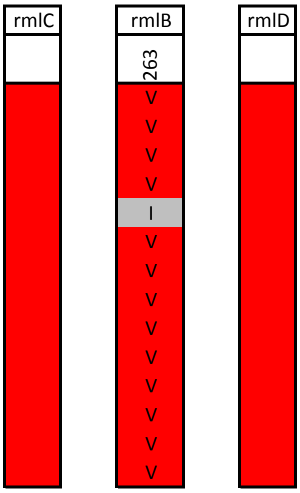
rmlC
rmlB
263
V
V
V
V
I
V
V
V
V
V
V
V
V
V
rmlD

SEROTYPE 7C ALL ISOLATES

		7C reference		
N/A	31	7C/2	15	7C
N/A	2896	7C/3	15	7C
32186	N/A	95344	199	7C
32383	N/A	BR1074	1797	7C
33452	N/A	ND6000	1797	7C
82078	N/A	SMRU6	2758	7C
82079	N/A	SMRU7	2758	7C
82298	N/A	SMRU1421	Sing10386	7C
83318	N/A	SMRU1525	2758	7C
121465	N/A	UoS2235	1797	7C

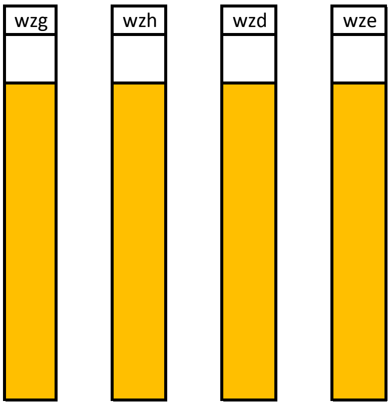

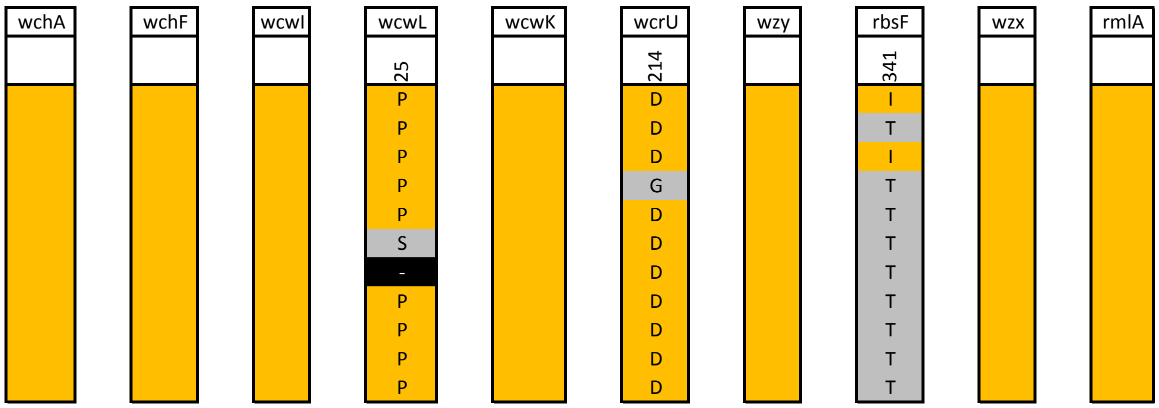
wchA
wchF
wcwI
wcwL
25
P
P
P
P
P
S
-
P
P
P
P
wcwK
wcrU
214
D
D
D
G
D
D
D
D
D
D
D
wzy
rbsF
341
I
T
I
T
T
T
T
T
T
T
T
wzx
rmlA

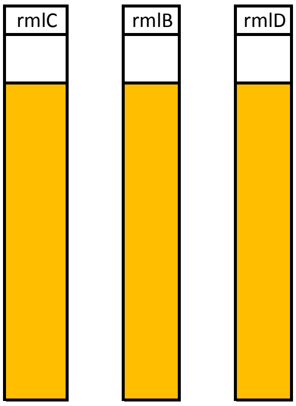
rmlC
rmlB
rmlD

SEROTYPE 7F ALL ISOLATES

					3	134	170	386	394	400
		7F reference			G	T	T	L	A	T
N/A	28	7A/3	Sing5994	7F	R	A	A	I	V	S
N/A	37	7F/6	218	7F	R	T	T	I	V	S
N/A	218	ICE23	218	7F	R	T	T	I	V	S
N/A	1179	GA47439	218	7F	R	T	T	I	V	S
N/A	2897	7F/2	218	7F	R	T	T	I	V	S
31973	N/A	SMRU307	3545/5265	7F	R	T	T	I	V	S
32394	N/A	BR1113	218	7F	R	T	T	I	V	S
32975	N/A	SMRU1057	3545/5265	7F	R	T	T	I	V	S
33948	N/A	SMRU1003	3545/5265	7F	R	T	T	I	V	S
44844	N/A	SMRU427	3545/5265	7F	R	T	T	I	V	S
82151	N/A	SMRU527	3545/5265	7F	R	T	T	I	V	S
83417	N/A	SMRU1795	3545/5265	7F	R	T	T	I	V	S
109672	N/A	SMRU27	3545/5265	7F	R	T	T	I	V	S
109799	N/A	SMRU1295	3324/5735/6853	7F	R	T	T	I	V	S
115503	N/A	SMRU208	3545/5265	7F	R	T	T	I	V	S
115561	N/A	SMRU1663	3545/5265	7F	R	T	T	I	V	S
N/A	34	7F/3	191	7F	R	T	T	L	A	T
N/A	217	ICE22	191	7F	R	T	T	L	A	T
N/A	299	Netherlands7F-39	191	7F	R	T	T	L	A	T
N/A	305	CDC1087-00	191	7F	R	T	T	L	A	T
N/A	459	VICE0138	191	7F	R	T	T	L	A	T
N/A	482	VICE0161	191	7F	R	T	T	L	A	T
N/A	485	VICE0164	191	7F	R	T	T	L	A	T
N/A	486	VICE0165	191	7F	R	T	T	L	A	T
N/A	497	VICE0176	191	7F	R	T	T	L	A	T
N/A	498	VICE0177	191	7F	R	T	T	L	A	T
N/A	506	VICE0185	191	7F	R	T	T	L	A	T
N/A	512	VICE0191	191	7F	R	T	T	L	A	T
N/A	532	VICE0211	191	7F	R	T	T	L	A	T
N/A	552	VICE0231	191	7F	R	T	T	L	A	T
N/A	596	VICE0274	191	7F	R	T	T	L	A	T
N/A	1374	VICE0780	191	7F	R	T	T	L	A	T
N/A	2178	VICE1300	191	7F	R	T	T	L	A	T
N/A	2556	VICE1603	191	7F	R	T	T	L	A	T
N/A	2874	VICE1902	191	7F	R	T	T	L	A	T
N/A	2898	7F/4	191	7F	R	T	T	L	A	T
N/A	2899	7F/5	191	7F	R	T	T	L	A	T
31320	N/A	R34-3163	191	7F	R	T	T	L	A	T
32158	N/A	R34-3219	191	7F	R	T	T	L	A	T
32319	N/A	ND6130	191	7F	R	T	T	L	A	T
32330	N/A	ND6158	191	7F	R	T	T	L	A	T
32347	N/A	NP7118	191	7F	R	T	T	L	A	T
32356	N/A	PT8039	191	7F	R	T	T	L	A	T
32397	N/A	BR1116	191	7F	R	T	T	L	A	T
32403	N/A	CH2008	191	7F	R	T	T	L	A	T
33393	N/A	ND6120	191	7F	R	T	T	L	A	T
33429	N/A	PT8094	191	7F	R	T	T	L	A	T
44823	N/A	UoS0307	191	7F	R	T	T	L	A	T
44827	N/A	UoS4223	191	7F	R	T	T	L	A	T
121364	N/A	UoS5220	191	7F	R	T	T	L	A	T
121365	N/A	UoS4151	191	7F	R	T	T	L	A	T
123222	N/A	UoS3245	191	7F	R	T	T	L	A	T

SEROTYPE 7F/19F HYBRID SEQUENCE

				3	12	53	123	134	170
			7F reference	G	K	V	E	T	T
			19F reference	R	R	A	D	T	A
N/A	28	7A/3	Sing5994	R	K	V	E	A	A
N/A	37	7F/6	218	R	K	V	E	T	T
N/A	218	ICE23	218	R	K	V	E	T	T
N/A	1179	GA47439	218	R	K	V	E	T	T
N/A	2897	7F/2	218	R	K	V	E	T	T
31973	N/A	SMRU307	3545/5265	R	K	V	E	T	T
32394	N/A	BR1113	218	R	K	V	E	T	T
32975	N/A	SMRU1057	3545/5265	R	K	V	E	T	T
33948	N/A	SMRU1003	3545/5265	R	K	V	E	T	T
44844	N/A	SMRU427	3545/5265	R	K	V	E	T	T
82151	N/A	SMRU527	3545/5265	R	K	V	E	T	T
83417	N/A	SMRU1795	3545/5265	R	K	V	E	T	T
109672	N/A	SMRU27	3545/5265	R	K	V	E	T	T
109799	N/A	SMRU1295	3324/5735/6853	R	K	V	E	T	T
115503	N/A	SMRU208	3545/5265	R	K	V	E	T	T
115561	N/A	SMRU1663	3545/5265	R	K	V	E	T	T

SEROTYPE 7F ISOLATES (NO HYBRID SEQUENCES)

					wzg	wzh	wzd
					3		197
		7F reference			G		L
N/A	34	7F/3	191	7F	R		L
N/A	217	ICE22	191	7F	R		L
N/A	299	Netherlands7F-39	191	7F	R		L
N/A	305	CDC1087-00	191	7F	R		L
N/A	459	VICE0138	191	7F	R		L
N/A	482	VICE0161	191	7F	R		L
N/A	485	VICE0164	191	7F	R		L
N/A	486	VICE0165	191	7F	R		L
N/A	497	VICE0176	191	7F	R		L
N/A	498	VICE0177	191	7F	R		L
N/A	506	VICE0185	191	7F	R		L
N/A	512	VICE0191	191	7F	R		L
N/A	532	VICE0211	191	7F	R		L
N/A	552	VICE0231	191	7F	R		L
N/A	596	VICE0274	191	7F	R		L
N/A	1374	VICE0780	191	7F	R		L
N/A	2178	VICE1300	191	7F	R		L
N/A	2556	VICE1603	191	7F	R		L
N/A	2874	VICE1902	191	7F	R		L
N/A	2898	7F/4	191	7F	R		L
N/A	2899	7F/5	191	7F	R		L
31320	N/A	R34-3163	191	7F	R		L
32158	N/A	R34-3219	191	7F	R		I
32319	N/A	ND6130	191	7F	R		L
32330	N/A	ND6158	191	7F	R		I
32347	N/A	NP7118	191	7F	R		I
32356	N/A	PT8039	191	7F	R		L
32397	N/A	BR1116	191	7F	R		I
32403	N/A	CH2008	191	7F	R		I
33393	N/A	ND6120	191	7F	R		I
33429	N/A	PT8094	191	7F	R		L
44823	N/A	UoS0307	191	7F	R		L
44827	N/A	UoS4223	191	7F	R		L
121364	N/A	UoS5220	191	7F	R		L
121365	N/A	UoS4151	191	7F	R		L

123222	N/A	UoS3245	191	7F	R		L

wzg							wzh						wzd	wze	wch
402	403	404	409	415	420	424	34	71	85	87	89	95	197	111	11
I	N	S	I	L	N	A	V	D	L	A	E	E	L	T	A
L	Q	G	L	I	D	T	V	S	P	V	D	R	L	I	G
L	Q	G	L	I	D	T	V	D	L	A	E	E	L	T	A
L	Q	G	L	I	D	T	V	D	L	A	E	E	L	T	A
L	Q	G	L	I	D	T	V	D	L	A	E	-	L	T	A
L	Q	G	L	I	D	T	V	D	L	A	E	E	L	T	A
L	Q	G	L	I	D	T	G	D	L	A	E	E	L	T	A
L	Q	G	L	I	D	T	V	D	L	A	E	E	L	T	A
L	Q	G	L	I	D	T	V	D	L	A	E	E	L	T	A
L	Q	G	L	I	D	T	V	D	L	A	E	E	L	T	A
L	Q	G	L	I	D	T	V	D	L	A	E	E	L	T	A
L	Q	G	L	I	D	T	V	D	L	A	E	E	L	T	A
L	Q	G	L	I	D	T	V	D	L	A	E	E	L	T	A
L	Q	G	L	I	D	T	V	D	L	A	E	E	L	T	A
L	Q	G	L	I	D	T	V	D	L	A	E	E	L	T	A
L	Q	G	L	I	D	T	V	D	L	A	E	E	L	T	A
L	Q	G	L	I	D	T	V	D	L	A	E	E	L	T	A
I	N	S	I	L	N	A	V	D	L	A	E	E	L	T	A
I	N	S	I	L	N	A	V	D	L	A	E	E	L	T	A
I	N	S	I	L	N	A	V	D	L	A	E	E	L	T	A
I	N	S	I	L	N	A	V	D	L	A	E	E	L	T	A
I	N	S	I	L	N	A	V	D	L	A	E	E	L	T	A
I	N	S	I	L	N	A	V	D	L	A	E	E	L	T	A
I	N	S	I	L	N	A	V	D	L	A	E	E	L	T	A
I	N	S	I	L	N	A	V	D	L	A	E	E	L	T	A
I	N	S	I	L	N	A	V	D	L	A	E	E	L	T	A
I	N	S	I	L	N	A	V	D	L	A	E	E	L	T	A
I	N	S	I	L	N	A	V	D	L	A	E	E	L	T	A
I	N	S	I	L	N	A	V	D	L	A	E	E	L	T	A
I	N	S	I	L	N	A	V	D	L	A	E	E	L	T	A
I	N	S	I	L	N	A	V	D	L	A	E	E	L	T	A
I	N	S	I	L	N	A	V	D	L	A	E	E	L	T	A
I	N	S	I	L	N	A	V	D	L	A	E	E	L	T	A
I	N	S	I	L	N	A	V	D	L	A	E	E	L	T	A
I	N	S	I	L	N	A	V	D	L	A	E	E	L	T	A
I	N	S	I	L	N	A	V	D	L	A	E	E	L	T	A
I	N	S	I	L	N	A	V	D	L	A	E	E	L	T	A
I	N	S	I	L	N	A	V	D	L	A	E	E	L	T	A
I	N	S	I	L	N	A	V	D	L	A	E	E	L	T	A
I	N	S	I	L	N	A	V	D	L	A	E	E	I	T	A
I	N	S	I	L	N	A	V	D	L	A	E	E	L	T	A
I	N	S	I	L	N	A	V	D	L	A	E	E	I	T	A
I	N	S	I	L	N	A	V	D	L	A	E	E	I	T	A
I	N	S	I	L	N	A	V	D	L	A	E	E	L	T	A
I	N	S	I	L	N	A	V	D	L	A	E	E	I	T	A
I	N	S	I	L	N	A	V	D	L	A	E	E	I	T	A
I	N	S	I	L	N	A	V	D	L	A	E	E	I	T	A
I	N	S	I	L	N	A	V	D	L	A	E	E	L	T	A
I	N	S	I	L	N	A	V	D	L	A	E	E	L	T	A
I	N	S	I	L	N	A	V	D	L	A	E	E	L	T	A
I	N	S	I	L	N	A	V	D	L	A	E	E	L	T	A
I	N	S	I	L	N	A	V	D	L	A	E	E	L	T	A
I	N	S	I	L	N	A	V	D	L	A	E	E	L	T	A

wzg												
259	269	339	386	394	400	402	403	404	409	415	420	424
R	S	P	L	A	T	I	N	S	I	L	N	A
K	A	L	I	V	S	L	Q	G	L	I	D	T
R	S	P	I	V	S	L	Q	G	L	I	D	T
R	S	P	I	V	S	L	Q	G	L	I	D	T
R	S	P	I	V	S	L	Q	G	L	I	D	T
R	S	P	I	V	S	L	Q	G	L	I	D	T
R	S	P	I	V	S	L	Q	G	L	I	D	T
R	S	P	I	V	S	L	Q	G	L	I	D	T
R	S	P	I	V	S	L	Q	G	L	I	D	T
R	S	P	I	V	S	L	Q	G	L	I	D	T
R	S	P	I	V	S	L	Q	G	L	I	D	T
R	S	P	I	V	S	L	Q	G	L	I	D	T
R	S	P	I	V	S	L	Q	G	L	I	D	T
R	S	P	I	V	S	L	Q	G	L	I	D	T
R	S	P	I	V	S	L	Q	G	L	I	D	T
R	S	P	I	V	S	L	Q	G	L	I	D	T
R	S	P	I	V	S	L	Q	G	L	I	D	T
R	S	P	I	V	S	L	Q	G	L	I	D	T

wzh					
27	34	71	85	87	89
E	V	D	L	A	E
A	V	D	L	A	E
E	V	S	P	V	D
E	V	D	L	A	E
E	V	D	L	A	E
E	V	D	L	A	E
E	V	D	L	A	E
E	G	D	L	A	E
E	V	D	L	A	E
E	V	D	L	A	E
E	V	D	L	A	E
E	V	D	L	A	E
E	V	D	L	A	E
E	V	D	L	A	E
E	V	D	L	A	E
E	V	D	L	A	E
E	V	D	L	A	E
E	V	D	L	A	E

wze	wchA	wchF	wcwA	wcwC	wcwD	wcwF	wcwG	wcwH	wzy
	217		79				114		
	M		S				N		
	M		S				N		
	M		S				N		
	M		S				N		
	M		S				N		
	M		S				N		
	M		S				N		
	M		S				N		
	M		S				N		
	M		S				N		
	M		S				N		
	V		S				N		
	M		S				N		
	M		S				N		
	M		S				N		
	M		S				N		
	M		S				N		
	M		S				N		
	M		S				N		
	M		S				D		
	M		S				N		
	M		S				N		
	M		S				N		
	M		F				N		
	M		S				N		
	M		F				N		
	M		F				N		
	M		S				N		
	M		F				N		
	M		F				N		
	M		F				N		
	M		S				N		
	M		S				N		
	M		S				N		
	M		S				N		
	M		S				N		

	M		S				N		

A	wchF		wcwA		wcwC	wcwD		wcwF	wcwG		wcwH	
217	41	294	79	229		25	245	227	114	259	166	289
M	A	Y	S	Q		A	Y	I	N	I	S	E
M	V	H	S	Q		A	C	F	N	L	P	E
M	A	Y	S	K		V	Y	I	N	I	S	E
M	A	Y	S	K		V	Y	I	N	I	S	D
M	A	Y	S	K		V	Y	I	N	I	S	D
M	A	Y	S	K		V	Y	I	N	I	S	E
M	A	Y	S	Q		A	Y	I	N	I	S	-
M	A	Y	S	K		V	Y	I	N	I	S	D
M	A	Y	S	Q		A	Y	I	N	I	S	E
M	A	Y	S	Q		A	Y	I	N	I	S	E
M	A	Y	S	Q		A	Y	I	N	I	S	E
M	A	Y	S	Q		A	Y	I	N	I	S	E
M	A	Y	S	Q		A	Y	I	N	I	S	E
M	A	Y	S	Q		A	Y	I	N	I	S	E
M	A	Y	S	Q		A	Y	I	N	I	S	E
M	A	Y	S	Q		A	Y	I	N	I	S	E
M	A	Y	S	Q		A	Y	I	N	-	S	E
M	A	Y	S	Q		A	Y	I	N	I	S	E
M	A	Y	S	Q		A	Y	I	N	I	S	E
M	A	Y	S	Q		A	Y	I	N	I	S	E
M	A	Y	S	Q		A	Y	I	N	I	S	E
M	A	Y	S	Q		A	Y	I	N	I	S	E
M	A	Y	S	Q		A	Y	I	N	I	S	E
M	A	Y	S	Q		A	Y	I	N	I	S	E
M	A	Y	S	Q		A	Y	I	N	I	S	E
M	A	Y	S	Q		A	Y	I	N	I	S	E
M	A	Y	S	Q		A	Y	I	N	I	S	E
V	A	Y	S	Q		A	Y	I	N	I	S	E
M	A	Y	S	Q		A	Y	I	N	I	S	E
M	A	Y	S	Q		A	Y	I	N	I	S	E
M	A	Y	S	Q		A	Y	I	N	I	S	E
M	A	Y	S	Q		A	Y	I	N	I	S	E
M	A	Y	S	Q		A	Y	I	N	I	S	E
M	A	Y	S	Q		A	Y	I	N	I	S	E
M	A	Y	S	Q		A	Y	I	N	I	S	E
M	A	Y	S	Q		A	Y	I	D	I	S	E
M	A	Y	S	Q		A	Y	I	N	I	S	E
M	A	Y	S	Q		A	Y	I	N	I	S	E
M	A	Y	S	Q		A	Y	I	N	I	S	E
M	A	Y	F	Q		A	Y	I	N	I	S	E
M	A	Y	S	Q		A	Y	I	N	I	S	E
M	A	Y	F	Q		A	Y	I	N	I	S	E
M	A	Y	F	Q		A	Y	I	N	I	S	E
M	A	Y	S	Q		A	Y	I	N	I	S	E
M	A	Y	F	Q		A	Y	I	N	I	S	E
M	A	Y	F	Q		A	Y	I	N	I	S	E
M	A	Y	F	Q		A	Y	I	N	I	S	E
M	A	Y	S	Q		A	Y	I	N	I	S	E
M	A	Y	S	Q		A	Y	I	N	I	S	E
M	A	Y	S	Q		A	Y	I	N	I	S	E
M	A	Y	S	Q		A	Y	I	N	I	S	E
M	A	Y	S	Q		A	Y	I	N	I	S	E
M	A	Y	S	Q		A	Y	I	N	I	S	E

95	165	167	223	226
E	H	I	V	A
E	Y	V	A	V
R	H	I	V	A
E	H	I	V	A
E	H	I	V	A
-	H	I	V	A
E	H	I	V	A
E	H	I	V	A
E	H	I	V	A
E	H	I	V	A
E	H	I	V	A
E	H	I	V	A
E	H	I	V	A
E	H	I	V	A
E	H	I	V	A
E	H	I	V	A
E	H	I	V	A
E	H	I	V	A

wzx	rmlA	rmlC	rmlB	rmlD
				192
				A
				A
				A
				A
				A
				V
				A
				V
				V
				A
				A
				V
				A
				V
				V
				V
				V
				V
				A
				A
				A
				A
				A
				A
				A
				A
				A
				A
				A
				A
				A
				A
				A
				A
				A
				A

wzy	wzx	rmlA	rmlC						rmlB		rmlD		
365		268	36	136	165	171	172	175	30	34	142	192	236
G		T	F	V	G	I	A	E	G	T	V	A	N
S		A	F	A	S	L	E	D	D	I	A	A	K
G		T	F	V	G	I	A	E	G	T	V	A	N
G		T	F	V	G	I	A	E	G	T	V	A	N
G		T	F	V	G	I	A	E	G	T	V	A	N
G		T	F	V	G	I	A	E	G	T	V	A	N
G		T	F	V	G	I	A	E	G	T	V	A	N
G		T	F	V	G	I	A	E	G	T	V	A	N
G		T	F	V	G	I	A	E	G	T	V	A	N
G		T	V	V	G	I	A	E	G	T	V	A	N
G		T	F	V	G	I	A	E	G	T	V	A	N
G		T	F	V	G	I	A	E	G	T	V	A	N
G		T	F	V	G	I	A	E	G	T	V	A	N
G		T	F	V	G	I	A	E	G	T	V	A	N
G		T	F	V	G	I	A	E	G	T	V	A	N
G		T	F	V	G	I	A	E	G	T	V	A	N
G		T	F	V	G	I	A	E	G	T	V	A	N
G		T	F	V	G	I	A	E	G	T	V	A	N
G		T	F	V	G	I	A	E	G	T	V	A	N
G		T	F	V	G	I	A	E	G	T	V	A	N
G		T	F	V	G	I	A	E	G	T	V	A	N
G		T	F	V	G	I	A	E	G	T	V	V	N
G		T	F	V	G	I	A	E	G	T	V	A	N
G		T	F	V	G	I	A	E	G	T	V	V	N
G		T	F	V	G	I	A	E	G	T	V	V	N
G		T	F	V	G	I	A	E	G	T	V	A	N
G		T	F	V	G	I	A	E	G	T	V	A	N
G		T	F	V	G	I	A	E	G	T	V	V	N
G		T	F	V	G	I	A	E	G	T	V	A	N
G		T	F	V	G	I	A	E	G	T	V	V	N
G		T	F	V	G	I	A	E	G	T	V	V	N
G		T	F	V	G	I	A	E	G	T	V	V	N
G		T	F	V	G	I	A	E	G	T	V	V	N
G		T	F	V	G	I	A	E	G	T	V	V	N
G		T	F	V	G	I	A	E	G	T	V	A	N
G		T	F	V	G	I	A	E	G	T	V	A	N
G		T	F	V	G	I	A	E	G	T	V	A	N
G		T	F	V	G	I	A	E	G	T	V	A	N
G		T	F	V	G	I	A	E	G	T	V	A	N
G		T	F	V	G	I	A	E	G	T	V	A	N
G		T	F	V	G	I	A	E	G	T	V	A	N
G		T	F	V	G	I	A	E	G	T	V	A	N
G		T	F	V	G	I	A	E	G	T	V	A	N
G		T	F	V	G	I	A	E	G	T	V	A	N
G		T	F	V	G	I	A	E	G	T	V	A	N
G		T	F	V	G	I	A	E	G	T	V	A	N
G		T	F	V	G	I	A	E	G	T	V	A	N
G		T	F	V	G	I	A	E	G	T	V	A	N
G		T	F	V	G	I	A	E	G	T	V	A	N
G		T	F	V	G	I	A	E	G	T	V	A	N
G		T	F	V	G	I	A	E	G	T	V	A	N
G		T	F	V	G	I	A	E	G	T	V	A	N
G		T	F	V	G	I	A	E	G	T	V	A	N

rmlst ID	PubMLST id	ENA	Name	ST	CC	Orig_sero	Orig_sero_method
N/A	35860	N/A	Spain9V-3	156	156/162	9V	Quellung
N/A	35932	N/A	SP9-BS68	1269	156/162	unknown	unknown
N/A	35854	N/A	USA8	8119	66	9N	unknown
N/A	35943	N/A	GA08780	156	156/162	9V	unknown
N/A	35957	N/A	GA17570	156	156/162	9V	unknown
121380	33824	ERR044876	UoS0080	405	218	unknown	WGS-based
121523	33967	ERR046057	UoS3042	66	66	unknown	WGS-based
121506	33950	ERR045361	UoS4311	66	66	unknown	WGS-based
123201	34125	ERR045374	UoS4350	66	66	unknown	WGS-based
121598	34042	ERR044860	UoS0014	162	156/162	unknown	WGS-based
123304	34171	ERR044903	UoS0176	162	156/162	unknown	WGS-based
121583	34027	ERR044905	UoS0196	162	156/162	unknown	WGS-based
121533	33977	ERR046061	UoS3059	4424	Sing4424	unknown	WGS-based
N/A	15474	N/A	9L/3	7204	292	9L	Quellung
N/A	15511	N/A	9L/4	7240	2174	9L	Quellung
N/A	35822	N/A	9N/3	3983	3983	9N	Quellung
N/A	36295	N/A	9N/4	71	66	9N	Quellung
N/A	35823	N/A	9N/5	71	66	9N	Quellung
N/A	36296	N/A	9N/6	71	66	9N	Quellung
N/A	36297	N/A	9V/4	123	113	9V	Quellung
N/A	36298	N/A	9V/5	162	156/162	9V	Quellung
N/A	36299	N/A	9V/6	162	156/162	9V	Quellung
N/A	36293	N/A	9A/1	312	156/162	9A	Quellung
N/A	36294	N/A	9L/2	5979	124	9L	Quellung
N/A	15475	N/A	9N/2	7205	3983	9N	Quellung
109896	28627	ERR057948	SMRU1967	5873	5873	9L	latex & Quellung
40235	28629	ERR051615	SMRU1069	5873	5873	9N	latex & Quellung
111495	28630	ERR054612	SMRU1160	3983	3983	9N	latex & Quellung
113114	28631	ERR054293	SMRU1416	3983	3983	9N	latex & Quellung
109847	28633	ERR056795	SMRU1457	5873	5873	9N	latex & Quellung
55943	28634	ERR054426	SMRU1477	5873	5873	9N	latex & Quellung
40351	28635	ERR054455	SMRU1507	3983	3983	9N	latex & Quellung
83370	28636	ERR056830	SMRU1684	5873	5873	9N	latex & Quellung
82540	28639	ERR066350	SMRU2383	5873	5873	9N	latex & Quellung
44955	28641	ERR064034	SMRU2529	3983	3983	9N	latex & Quellung
82576	28642	ERR067821	SMRU2598	5873	5873	9N	latex & Quellung
83527	28643	ERR067824	SMRU2601	5873	5873	9N	latex & Quellung
113852	28640	ERR064110	SMRU2708	5873	5873	9N	latex & Quellung
83470	28644	ERR064115	SMRU2713	5873	5873	9N	latex & Quellung
109951	28645	ERR064117	SMRU2715	5873	5873	9N	latex & Quellung
82475	28646	ERR064125	SMRU2723	5873	5873	9N	latex & Quellung
82638	28647	ERR069591	SMRU2741	5873	5873	9N	latex & Quellung
83569	28648	ERR069682	SMRU2832	5873	5873	9N	latex & Quellung
34279	28649	ERR067935	SMRU2904	5873	5873	9N	latex & Quellung
115548	28628	ERR056701	SMRU835	3983	3983	9N	latex & Quellung
31724	28656	ERR273536	SMRU1017	156	156/162	9V	latex & Quellung
31734	28655	ERR273551	SMRU1032	156	156/162	9V	latex & Quellung
109828	28667	ERR054626	SMRU1174	280	280	9V	latex & Quellung
82244	28657	ERR054307	SMRU1214	156	156/162	9V	latex & Quellung
111546	28668	ERR054240	SMRU1363	280	280	9V	latex & Quellung
82388	28669	ERR063858	SMRU2065	280	280	9V	latex & Quellung
82427	28658	ERR063948	SMRU2155	156	156/162	9V	latex & Quellung
83247	28662	ERR049953	SMRU234	280	280	9V	latex & Quellung
113097	28652	ERR049957	SMRU238	156	156/162	9V	latex & Quellung
44960	28659	ERR064156	SMRU2418	156	156/162	9V	latex & Quellung
83524	28670	ERR067813	SMRU2590	280	280	9V	latex & Quellung
109964	28660	ERR066185	SMRU2650	156	156/162	9V	latex & Quellung
83557	28661	ERR069588	SMRU2738	156	156/162	9V	latex & Quellung

82611	28671	ERR067892	SMRU2837	280	280	9V	latex & Quellung
83546	28673	ERR067926	SMRU2895	280	280	9V	latex & Quellung
82622	28672	ERR067927	SMRU2896	280	280	9V	latex & Quellung
82118	28651	ERR048056	SMRU331	156	156/162	9V	latex & Quellung
113088	28664	ERR048160	SMRU483	280	280	9V	latex & Quellung
82148	28650	ERR048198	SMRU521	156	156/162	9V	latex & Quellung
111553	28654	ERR051449	SMRU615	156	156/162	9V	latex & Quellung
83271	28653	ERR051450	SMRU616	156	156/162	9V	latex & Quellung
82193	28666	ERR050047	SMRU976	280	280	9V	latex & Quellung
31506	29402	ERR124320	422264	156	156/162	9A	Quellung
32192	29366	ERR069769	132571	405	218	9N	Quellung
32065	29673	ERR065958	436915	66	66	9N	Quellung
32466	29500	ERR129162	MD5007	1797	66	9N	Quellung
32318	29367	ERR124226	ND6123	405	218	9N	Quellung
31436	29368	ERR069799	234323	156	156/162	9V	Quellung
31453	29369	ERR069818	312942	156	156/162	9V	Quellung
32406	29403	ERR129077	CH2029	162	156/162	9V	Quellung
31309	29675	ERR065342	R34-3016	156	156/162	9V	Quellung
31274	29674	ERR065301	R34-3045	156	156/162	9V	Quellung
N/A	33244	ERR449852	VICE0676	1269	156/162	not done	--
N/A	33107	ERR388848	VICE0132	66	66	not done	--
N/A	29948	ERR460432	VICE0527	10344	66	not done	--
N/A	33118	ERR388870	VICE0175	66	66	not done	--
N/A	33089	ERR388927	VICE0275	66	66	not done	--
N/A	36191	ERR755505	VICE1770	66	66	not done	--
N/A	36192	ERR755506	VICE1771	66	66	not done	--
N/A	33474	ERR470340	VICE0894a	66	66	not done	--
N/A	34396	ERR388851	VICE0136	66	66	not done	--
N/A	33545	ERR470332	VICE0881	66	66	not done	--
N/A	33559	ERR540377	VICE1287	66	66	not done	--
N/A	33562	ERR540385	VICE1296	66	66	not done	--
N/A	34300	ERR689505	VICE1426	66	66	not done	--
N/A	34459	ERR697244	VICE1511	66	66	not done	--
N/A	34262	ERR697285	VICE1553	66	66	not done	--
N/A	34315	ERR697286	VICE1554a	66	66	not done	--
N/A	34316	ERR697287	VICE1554b	66	66	not done	--
N/A	34435	ERR690898	VICE1025b	66	66	not done	--
N/A	33109	ERR388854	VICE0141	162	156/162	not done	--
N/A	33111	ERR388859	VICE0156	162	156/162	not done	--
N/A	34542	ERR690877	VICE0797	162	156/162	not done	--
N/A	33112	ERR388860	VICE0157	162	156/162	not done	--
N/A	33634	ERR540461	VICE0367	162	156/162	not done	--
N/A	33245	ERR449857	VICE0526	162	156/162	not done	--
N/A	33422	ERR470369	VICE0713	1269	156/162	not done	--
N/A	33356	ERR470380	VICE0729	162	156/162	not done	--
N/A	34498	ERR690889	VICE0945	162	156/162	not done	--
N/A	33673	ERR540532	VICE0958	162	156/162	not done	--
N/A	33674	ERR540533	VICE0959	162	156/162	not done	--
N/A	34214	ERR540536	VICE0967	162	156/162	not done	--
N/A	33676	ERR540537	VICE0968	162	156/162	not done	--
N/A	34486	ERR540538	VICE0972	162	156/162	not done	--
N/A	34285	ERR540547	VICE1016	162	156/162	not done	--
N/A	33697	ERR540601	VICE1096	162	156/162	not done	--
N/A	33457	ERR540410	VICE1328a	162	156/162	not done	--
N/A	34274	ERR388868	VICE0167	162	156/162	not done	--
N/A	33635	ERR540462	VICE0441	162	156/162	not done	--
N/A	33191	ERR449856	VICE0681	156	156/162	not done	--
N/A	33326	ERR470362	VICE0704	156	156/162	not done	--
N/A	33329	ERR470396	VICE0759	156	156/162	not done	--

N/A	33342	ERR470407	VICE0779	162	156/162	not done	--
N/A	34255	ERR697247	VICE1514	162	156/162	not done	--
N/A	34571	ERR388806	VICE0142	162	156/162	not done	--
N/A	34397	ERR388862	VICE0160	1269	156/162	not done	--
N/A	34212	ERR540442	VICE0173	1269	156/162	not done	--
N/A	33401	ERR449763	VICE0328	1269	156/162	not done	--
N/A	33354	ERR470171	VICE0391	162	156/162	not done	--
N/A	33115	ERR388864	VICE0162	162	156/162	not done	--
N/A	33116	ERR388865	VICE0163	1269	156/162	not done	--

Seq_sero	Hybrid	Hybrid type	Country	Region	carriage/disease	Source
9V	No	N/A	France	Toulouse	disease	unknown
9V	No	N/A	USA	Pennsylvania	disease	unknown
9N	No	N/A	USA	Maryland	disease	blood
9V	No	N/A	USA	Georgia	disease	blood
9A	No	N/A	USA	Georgia	disease	blood
9N	No	N/A	United Kingdom	Southampton	carriage	nasopharynx
9N	No	N/A	United Kingdom	Southampton	carriage	nasopharynx
9N	No	N/A	United Kingdom	Southampton	carriage	nasopharynx
9N	No	N/A	United Kingdom	Southampton	carriage	nasopharynx
9V	No	N/A	United Kingdom	Southampton	carriage	nasopharynx
9V	No	N/A	United Kingdom	Southampton	carriage	nasopharynx
9V	No	N/A	United Kingdom	Southampton	carriage	nasopharynx
9Vii	No	N/A	United Kingdom	Southampton	carriage	nasopharynx
9L	No	N/A	Denmark	unknown	unknown	unknown
9L	No	N/A	Denmark	unknown	unknown	unknown
9N	No	N/A	Denmark	unknown	unknown	unknown
9N	No	N/A	Denmark	unknown	unknown	unknown
9N	No	N/A	Denmark	unknown	unknown	unknown
9N	No	N/A	Denmark	unknown	unknown	unknown
9V	No	N/A	Denmark	unknown	unknown	unknown
9V	No	N/A	Denmark	unknown	unknown	unknown
9V	No	N/A	Denmark	unknown	unknown	unknown
9A	No	N/A	USA	unknown	unknown	unknown
9L	No	N/A	USA	unknown	unknown	unknown
9N	No	N/A	USA	unknown	unknown	unknown
9Lii	No	N/A	Thailand	Maela	carriage	nasopharynx
9Lii	No	N/A	Thailand	Maela	carriage	nasopharynx
9N	No	N/A	Thailand	Maela	carriage	nasopharynx
9N	No	N/A	Thailand	Maela	carriage	nasopharynx
9L	No	N/A	Thailand	Maela	carriage	nasopharynx
9Lii	No	N/A	Thailand	Maela	carriage	nasopharynx
9N	No	N/A	Thailand	Maela	carriage	nasopharynx
9Lii	No	N/A	Thailand	Maela	carriage	nasopharynx
9Lii	No	N/A	Thailand	Maela	carriage	nasopharynx
9N	No	N/A	Thailand	Maela	carriage	nasopharynx
9Lii	No	N/A	Thailand	Maela	carriage	nasopharynx
9Lii	No	N/A	Thailand	Maela	carriage	nasopharynx
9L	No	N/A	Thailand	Maela	carriage	nasopharynx
9L	No	N/A	Thailand	Maela	carriage	nasopharynx
9L	No	N/A	Thailand	Maela	carriage	nasopharynx
9L	No	N/A	Thailand	Maela	carriage	nasopharynx
9L	No	N/A	Thailand	Maela	carriage	nasopharynx
9L	No	N/A	Thailand	Maela	carriage	nasopharynx
9L	No	N/A	Thailand	Maela	carriage	nasopharynx
9N	No	N/A	Thailand	Maela	carriage	nasopharynx
9V	No	N/A	Thailand	Maela	carriage	nasopharynx
9V	No	N/A	Thailand	Maela	carriage	nasopharynx
9Vii	No	N/A	Thailand	Maela	carriage	nasopharynx
9V	No	N/A	Thailand	Maela	carriage	nasopharynx
9Vii	No	N/A	Thailand	Maela	carriage	nasopharynx
9Vii	No	N/A	Thailand	Maela	carriage	nasopharynx
9V	No	N/A	Thailand	Maela	carriage	nasopharynx
9Vii	No	N/A	Thailand	Maela	carriage	nasopharynx
9V	No	N/A	Thailand	Maela	carriage	nasopharynx
9V	No	N/A	Thailand	Maela	carriage	nasopharynx
9Vii	No	N/A	Thailand	Maela	carriage	nasopharynx
9V	No	N/A	Thailand	Maela	carriage	nasopharynx
9V	No	N/A	Thailand	Maela	carriage	nasopharynx

9Vii	No	N/A	Thailand	Maela	carriage	nasopharynx
9Vii	No	N/A	Thailand	Maela	carriage	nasopharynx
9Vii	No	N/A	Thailand	Maela	carriage	nasopharynx
9V	No	N/A	Thailand	Maela	carriage	nasopharynx
9Vii	No	N/A	Thailand	Maela	carriage	nasopharynx
9V	No	N/A	Thailand	Maela	carriage	nasopharynx
9V	No	N/A	Thailand	Maela	carriage	nasopharynx
9V	No	N/A	Thailand	Maela	carriage	nasopharynx
9Vii	No	N/A	Thailand	Maela	carriage	nasopharynx
9V	No	N/A	USA	Massachusetts	carriage	nasopharynx
9N	No	N/A	USA	Massachusetts	carriage	nasopharynx
9N	No	N/A	USA	Massachusetts	carriage	nasopharynx
9N	No	N/A	USA	Massachusetts	carriage	nasopharynx
9N	No	N/A	USA	Massachusetts	carriage	nasopharynx
9V	No	N/A	USA	Massachusetts	carriage	nasopharynx
9V	No	N/A	USA	Massachusetts	carriage	nasopharynx
9V	No	N/A	USA	Massachusetts	carriage	nasopharynx
9V	No	N/A	USA	Massachusetts	carriage	nasopharynx
9V	No	N/A	USA	Massachusetts	carriage	nasopharynx
9A	No	N/A	Iceland	Kópavogur	disease	sputum
9N	No	N/A	Iceland	Borgarnes	disease	blood
9N	No	N/A	Iceland	Garðabær	disease	middle ear fluid
9N	No	N/A	Iceland	Hafnarfjörður	disease	blood
9N	No	N/A	Iceland	Hafnarfjörður	disease	blood
9N	No	N/A	Iceland	Hafnarfjörður	carriage	nasopharynx
9N	No	N/A	Iceland	Hafnarfjörður	carriage	nasopharynx
9N	No	N/A	Iceland	Kópavogur	carriage	nasopharynx
9N	No	N/A	Iceland	Reykjavík	disease	blood
9N	No	N/A	Iceland	Reykjavík	carriage	nasopharynx
9N	No	N/A	Iceland	Reykjavík	carriage	nasopharynx
9N	No	N/A	Iceland	Reykjavík	carriage	nasopharynx
9N	No	N/A	Iceland	Reykjavík	carriage	nasopharynx
9N	No	N/A	Iceland	Reykjavík	disease	blood
9N	No	N/A	Iceland	Reykjavík	carriage	nasopharynx
9N	No	N/A	Iceland	Reykjavík	carriage	nasopharynx
9N	No	N/A	Iceland	Reykjavík	carriage	nasopharynx
9N	No	N/A	Iceland	unknown	carriage	nasopharynx
9V	No	N/A	Iceland	Akranes	disease	blood
9V	No	N/A	Iceland	Akranes	disease	CSF
9V	No	N/A	Iceland	Garðabær	disease	sputum
9V	No	N/A	Iceland	Hafnarfjörður	disease	blood
9V	No	N/A	Iceland	Hafnarfjörður	disease	middle ear fluid
9V	No	N/A	Iceland	Hafnarfjörður	disease	middle ear fluid
9V	No	N/A	Iceland	Hafnarfjörður	disease	sputum
9V	No	N/A	Iceland	Hafnarfjörður	disease	sputum
9V	No	N/A	Iceland	Hafnarfjörður	carriage	nasopharynx
9V	No	N/A	Iceland	Hafnarfjörður	carriage	nasopharynx
9V	No	N/A	Iceland	Hafnarfjörður	carriage	nasopharynx
9V	No	N/A	Iceland	Hafnarfjörður	carriage	nasopharynx
9V	No	N/A	Iceland	Hafnarfjörður	carriage	nasopharynx
9V	No	N/A	Iceland	Hafnarfjörður	carriage	nasopharynx
9V	No	N/A	Iceland	Hafnarfjörður	carriage	nasopharynx
9V	No	N/A	Iceland	Hafnarfjörður	carriage	nasopharynx
9V	No	N/A	Iceland	Hafnarfjörður	carriage	nasopharynx
9V	No	N/A	Iceland	Kópavogur	disease	blood
9V	No	N/A	Iceland	Kópavogur	disease	middle ear fluid
9V	No	N/A	Iceland	Kópavogur	disease	sputum
9V	No	N/A	Iceland	Kópavogur	disease	sputum
9V	No	N/A	Iceland	Kópavogur	disease	sputum

9V	No	N/A	Iceland	Kópavogur	disease	sputum
9V	No	N/A	Iceland	Kópavogur	disease	blood
9V	No	N/A	Iceland	Reykjavík	disease	blood
9V	No	N/A	Iceland	Reykjavík	disease	blood
9V	No	N/A	Iceland	Reykjavík	disease	blood
9V	No	N/A	Iceland	Reykjavík	disease	middle ear fluid
9V	No	N/A	Iceland	Reykjavík	disease	middle ear fluid
9V	No	N/A	Iceland	unknown	disease	blood
9V	No	N/A	Iceland	unknown	disease	blood

Year	Publication	Reference	vaccine status
1993	Capsule switching	PMID: 23175765	pre
unknown	Genbank (336)	N/A	post
2001	Genbank (336)	N/A	post
1997	Genbank (336)	N/A	pre
2001	Genbank (336)	N/A	post
2006-2011	Gladstone UK PCV	PMID: 25776920	unknown
2006-2011	Gladstone UK PCV	PMID: 25776920	unknown
2006-2011	Gladstone UK PCV	PMID: 25776920	unknown
2006-2011	Gladstone UK PCV	PMID: 25776920	unknown
2006-2011	Gladstone UK PCV	PMID: 25776920	unknown
2006-2011	Gladstone UK PCV	PMID: 25776920	unknown
2006-2011	Gladstone UK PCV	PMID: 25776920	unknown
2006-2011	Gladstone UK PCV	PMID: 25776920	unknown
1941	Historical	this publication	pre
1968	Historical	this publication	pre
1938	Historical	this publication	pre
1962	Historical	this publication	pre
1962	Historical	this publication	pre
1960	Historical	this publication	pre
1968	Historical	this publication	pre
1991	Historical	this publication	pre
1994	Historical	this publication	pre
1962	Historical	this publication	pre
1952	Historical	this publication	pre
1952	Historical	this publication	pre
2009	Maela	PMID: 24509479	pre
2008	Maela	PMID: 24509479	pre
2009	Maela	PMID: 24509479	pre
2009	Maela	PMID: 24509479	pre
2009	Maela	PMID: 24509479	pre
2009	Maela	PMID: 24509479	pre
2009	Maela	PMID: 24509479	pre
2009	Maela	PMID: 24509479	pre
2010	Maela	PMID: 24509479	pre
2010	Maela	PMID: 24509479	pre
2010	Maela	PMID: 24509479	pre
2010	Maela	PMID: 24509479	pre
2010	Maela	PMID: 24509479	pre
2010	Maela	PMID: 24509479	pre
2010	Maela	PMID: 24509479	pre
2010	Maela	PMID: 24509479	pre
2010	Maela	PMID: 24509479	pre
2010	Maela	PMID: 24509479	pre
2010	Maela	PMID: 24509479	pre
2008	Maela	PMID: 24509479	pre
2008	Maela	PMID: 24509479	pre
2008	Maela	PMID: 24509479	pre
2009	Maela	PMID: 24509479	pre
2009	Maela	PMID: 24509479	pre
2009	Maela	PMID: 24509479	pre
2009	Maela	PMID: 24509479	pre
2009	Maela	PMID: 24509479	pre
2008	Maela	PMID: 24509479	pre
2008	Maela	PMID: 24509479	pre
2010	Maela	PMID: 24509479	pre
2010	Maela	PMID: 24509479	pre
2010	Maela	PMID: 24509479	pre
2010	Maela	PMID: 24509479	pre

2010	Maela	PMID: 24509479	pre
2010	Maela	PMID: 24509479	pre
2010	Maela	PMID: 24509479	pre
2008	Maela	PMID: 24509479	pre
2008	Maela	PMID: 24509479	pre
2008	Maela	PMID: 24509479	pre
2008	Maela	PMID: 24509479	pre
2008	Maela	PMID: 24509479	pre
2008	Maela	PMID: 24509479	pre
2001	Massachusetts	PMID: 23644493	pre
2001	Massachusetts	PMID: 23644493	pre
2001	Massachusetts	PMID: 23644493	pre
2007	Massachusetts	PMID: 23644493	post
2007	Massachusetts	PMID: 23644493	post
2001	Massachusetts	PMID: 23644493	pre
2001	Massachusetts	PMID: 23644493	pre
2007	Massachusetts	PMID: 23644493	post
2004	Massachusetts	PMID: 23644493	post
2004	Massachusetts	PMID: 23644493	post
2009	VICE	this publication	pre
2009	VICE	this publication	pre
2011	VICE	this publication	post
2010	VICE	this publication	pre
2013	VICE	this publication	post
2014	VICE	this publication	post
2014	VICE	this publication	post
2009	VICE	this publication	pre
2009	VICE	this publication	pre
2009	VICE	this publication	pre
2011	VICE	this publication	post
2011	VICE	this publication	post
2012	VICE	this publication	post
2014	VICE	this publication	post
2013	VICE	this publication	post
2013	VICE	this publication	post
2013	VICE	this publication	post
2009	VICE	this publication	pre
2009	VICE	this publication	pre
2009	VICE	this publication	pre
2012	VICE	this publication	post
2009	VICE	this publication	pre
2009	VICE	this publication	pre
2011	VICE	this publication	post
2010	VICE	this publication	pre
2011	VICE	this publication	post
2009	VICE	this publication	pre
2009	VICE	this publication	pre
2009	VICE	this publication	pre
2009	VICE	this publication	pre
2009	VICE	this publication	pre
2009	VICE	this publication	pre
2009	VICE	this publication	pre
2010	VICE	this publication	pre
2012	VICE	this publication	post
2009	VICE	this publication	pre
2010	VICE	this publication	pre
2010	VICE	this publication	pre
2010	VICE	this publication	pre
2011	VICE	this publication	post

2012	VICE	this publication	post
2014	VICE	this publication	post
2009	VICE	this publication	pre
2009	VICE	this publication	pre
2010	VICE	this publication	pre
2009	VICE	this publication	pre
2010	VICE	this publication	pre
2009	VICE	this publication	pre
2009	VICE	this publication	pre

SEROTYPE 9A ALL ISOLATES

					wzg					wzh
					9	80	106	123	162	
		9A reference			G	L	A	E	N	
N/A	1141	GA17570	156/162	9A	G	L	A	E	N	
N/A	1270	VICE0676	156/162	9A	R	V	T	D	D	
N/A	2901	9A/1	156/162	9A	G	L	A	E	N	

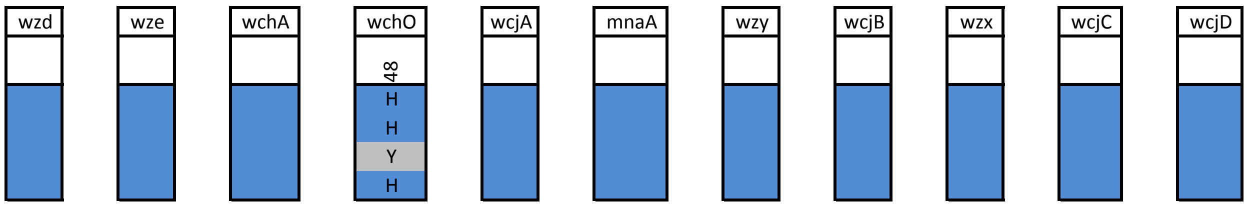
wzd
wze
wchA
wchO
48
H
H
Y
H
wcjA
mnaA
wzy
wcjB
wzx
wcjC
wcjD

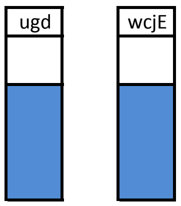
ugd
wcjE

SEROTYPE 9L ALL ISOLATES

					wzg				wzh
					9	16	138	305	
		9L reference			G	S	E	V	
N/A	44	9L/3	292	9L	G	S	G	V	
N/A	2902	9L/2	124	9L	G	S	G	V	
N/A	2903	9L/4	2174	9L	G	S	E	V	
34279	N/A	SMRU2904	5873	9L	G	S	G	V	
82475	N/A	SMRU2723	5873	9L	G	S	G	V	
82638	N/A	SMRU2741	5873	9L	G	S	G	V	
83470	N/A	SMRU2713	5873	9L	G	S	G	V	
83569	N/A	SMRU2832	5873	9L	G	S	G	V	
109847	N/A	SMRU1457	5873	9L	G	S	G	V	
109951	N/A	SMRU2715	5873	9L	G	S	G	V	
113852	N/A	SMRU2708	5873	9L	G	S	G	V	
40235	N/A	SMRU1069	5873	9L	S	N	G	I	
55943	N/A	SMRU1477	5873	9L	S	N	G	I	
82540	N/A	SMRU2383	5873	9L	S	N	G	I	
82576	N/A	SMRU2598	5873	9L	S	N	G	I	
83370	N/A	SMRU1684	5873	9L	S	N	G	I	
83527	N/A	SMRU2601	5873	9L	S	N	G	I	
109896	N/A	SMRU1967	5873	9L	S	N	G	I	

98.7

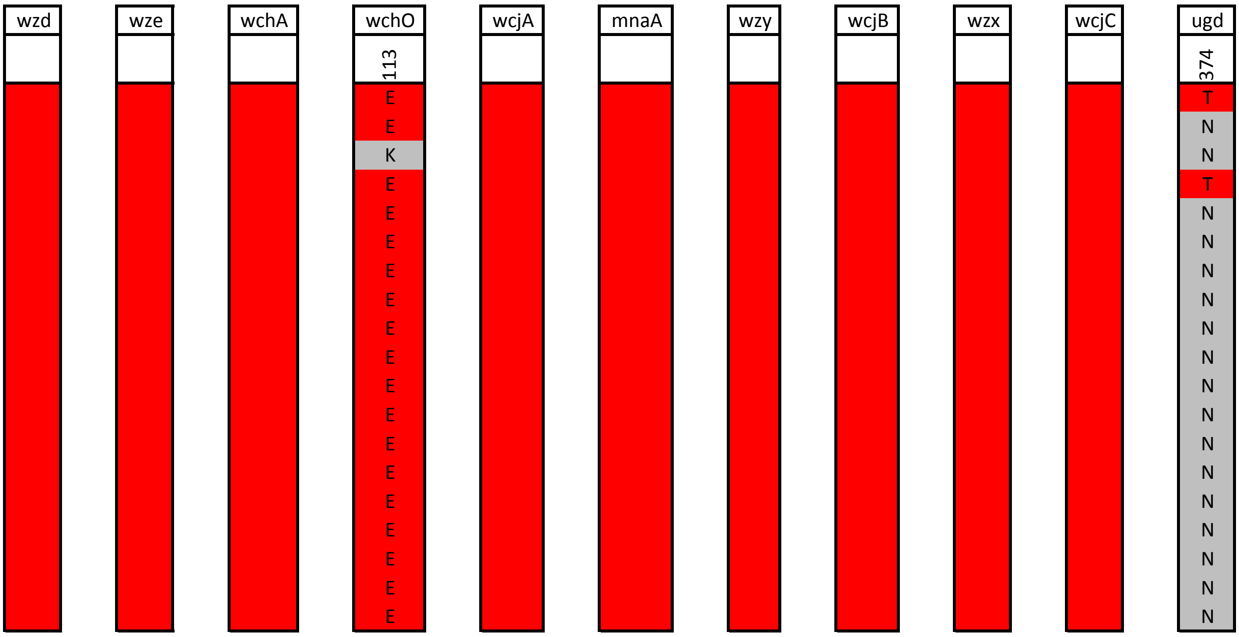
wzd
wze
wchA
wchO
wcjA
mnaA
wzy
wcjB
wzx
wcjC
ugd
113
E E K E E E E E E E E E E E E E E E E
374
T N N T N N N N N N N N N N N N N N N

SEROTYPE 9N ALL ISOLATES

					wzg	wzh	wzd	w[illegible]
								172
		9N reference						S
N/A	47	9N/3	3983	9N				S
N/A	49	9N/5	66	9N				S
N/A	246	USA8	66	9N				S
N/A	435	VICE0881	66	9N				S
N/A	453	VICE0132	66	9N				S
N/A	457	VICE0136	66	9N				S
N/A	496	VICE0175	66	9N				S
N/A	597	VICE0275	66	9N				S
N/A	849	VICE0527	66	9N				S
N/A	1445	VICE0894a	66	9N				S
N/A	2165	VICE1287	66	9N				S
N/A	2174	VICE1296	66	9N				S
N/A	2304	VICE1426	66	9N				S
N/A	2389	VICE1511	66	9N				S
N/A	2431	VICE1553	66	9N				S
N/A	2432	VICE1554a	66	9N				S
N/A	2475	VICE1025b	66	9N				S
N/A	2536	VICE1554b	66	9N				S
N/A	2728	VICE1770	66	9N				S
N/A	2729	VICE1771	66	9N				S
N/A	2904	9N/2	3983	9N				S
N/A	2905	9N/4	66	9N				F
N/A	2906	9N/6	66	9N				S
32065	N/A	436915	66	9N				S
32192	N/A	132571	218	9N				S
32318	N/A	ND6123	218	9N				S
32466	N/A	MD5007	66	9N				S
40351	N/A	SMRU1507	3983	9N				S
44955	N/A	SMRU2529	3983	9N				S
111495	N/A	SMRU1160	3983	9N				S
113114	N/A	SMRU1416	3983	9N				S
115548	N/A	SMRU835	3983	9N				S
121380	N/A	UoS0080	218	9N				S
121506	N/A	UoS4311	66	9N				S
121523	N/A	UoS3042	66	9N				S
123201	N/A	UoS4350	66	9N				S

ze	wchA			wchO	wcjA	mnaA	wzy	wcjB	wzx	wcjC	ugd
219	167	234	271	38						91	
G	D	R	T	G						A	
G	D	R	T	G						A	
G	D	R	T	G						A	
G	D	R	A	G						A	
G	D	R	T	G						A	
G	D	R	T	G						A	
G	D	R	T	G						A	
G	D	R	T	G						A	
G	D	R	T	G						A	
G	D	R	T	G						A	
G	D	R	T	G						A	
G	D	R	T	G						A	
G	D	R	T	G						A	
G	D	R	T	G						A	
G	D	S	T	G						A	
G	D	R	T	G						A	
G	D	R	T	G						A	
G	D	R	T	G						A	
G	D	R	T	E						A	
G	D	R	T	G						A	
G	D	R	T	G						A	
G	D	R	T	G						A	
G	D	R	T	G						A	
G	D	R	T	G						A	
G	D	R	T	G						A	
G	D	R	T	G						A	
G	D	R	T	G						A	
G	D	R	T	G						A	
G	D	R	T	G						S	
G	D	R	T	G						S	
G	D	R	T	G						S	
G	D	R	T	G						S	
G	D	R	T	G						S	
G	D	R	T	G						A	
G	N	R	T	G						A	
G	D	R	T	G						A	
S	D	R	T	G						A	

SEROTYPE 9V ALL ISOLATES

					wzg						
					9	80	106	120	121	123	162
		9V reference			G	L	A	A	D	E	N
N/A	263	Spain9V-3	156/162	9V	G	L	A	A	D	E	N
N/A	462	VICE0141	156/162	9V	G	L	A	A	D	E	N
N/A	463	VICE0142	156/162	9V	G	L	A	A	D	E	N
N/A	477	VICE0156	156/162	9V	G	L	A	A	D	E	N
N/A	478	VICE0157	156/162	9V	G	L	A	A	D	E	N
N/A	483	VICE0162	156/162	9V	G	L	A	A	D	E	N
N/A	488	VICE0167	156/162	9V	G	L	A	A	D	E	N
N/A	689	VICE0367	156/162	9V	G	L	A	A	D	E	N
N/A	713	VICE0391	156/162	9V	G	L	A	A	D	E	N
N/A	763	VICE0441	156/162	9V	G	L	A	A	D	E	N
N/A	848	VICE0526	156/162	9V	G	L	A	A	D	E	N
N/A	1109	SP9-BS68	156/162	9V	G	L	A	A	D	E	N
N/A	1123	GA08780	156/162	9V	G	L	A	A	D	E	N
N/A	1275	VICE0681	156/162	9V	G	L	A	A	D	E	N
N/A	1298	VICE0704	156/162	9V	G	L	A	A	D	E	N
N/A	1323	VICE0729	156/162	9V	G	L	A	A	D	E	N
N/A	1353	VICE0759	156/162	9V	G	L	A	A	D	E	N
N/A	1373	VICE0779	156/162	9V	G	L	A	A	D	E	N
N/A	1391	VICE0797	156/162	9V	G	L	A	A	D	E	N
N/A	1496	VICE0945	156/162	9V	G	L	A	A	D	E	N
N/A	1509	VICE0958	156/162	9V	G	L	A	A	D	E	N
N/A	1510	VICE0959	156/162	9V	G	L	A	A	D	E	N
N/A	1518	VICE0967	156/162	9V	G	L	A	A	D	E	N
N/A	1519	VICE0968	156/162	9V	G	L	A	A	D	E	N
N/A	1523	VICE0972	156/162	9V	G	L	A	A	D	E	N
N/A	1567	VICE1016	156/162	9V	G	L	A	A	D	E	N
N/A	1647	VICE1096	156/162	9V	G	L	A	A	D	E	N
N/A	2206	VICE1328a	156/162	9V	G	L	A	A	D	E	N
N/A	2392	VICE1514	156/162	9V	G	L	A	A	D	E	N
N/A	2907	9V/4	113	9V	G	L	A	A	D	E	N
N/A	2908	9V/5	156/162	9V	G	L	A	A	D	E	N
N/A	2909	9V/6	156/162	9V	G	L	A	A	D	E	N
31274	N/A	R34-3045	156/162	9V	G	L	A	A	D	E	N
31309	N/A	R34-3016	156/162	9V	G	L	A	A	D	E	N
31436	N/A	234323	156/162	9V	G	L	A	A	D	E	N
31453	N/A	312942	156/162	9V	G	L	A	A	D	E	N
31506	N/A	422264	156/162	9V	G	L	A	A	D	E	N
31724	N/A	SMRU1017	156/162	9V	G	L	A	A	D	E	N
31734	N/A	SMRU1032	156/162	9V	G	L	A	A	D	E	N
32406	N/A	CH2029	156/162	9V	G	L	A	A	D	E	N
44960	N/A	SMRU2418	156/162	9V	G	L	A	A	D	E	N
82118	N/A	SMRU331	156/162	9V	G	L	A	A	D	E	N
82148	N/A	SMRU521	156/162	9V	G	L	A	A	D	E	N
82244	N/A	SMRU1214	156/162	9V	G	L	A	A	D	E	N
82427	N/A	SMRU2155	156/162	9V	G	L	A	A	D	E	N
83271	N/A	SMRU616	156/162	9V	G	L	A	A	D	E	N
83557	N/A	SMRU2738	156/162	9V	G	L	A	A	D	E	N
109964	N/A	SMRU2650	156/162	9V	G	L	A	A	D	E	N
111553	N/A	SMRU615	156/162	9V	G	L	A	A	D	E	N
113097	N/A	SMRU238	156/162	9V	G	L	A	A	D	E	N
121583	N/A	UoS0196	156/162	9V	G	L	A	A	D	E	N
121598	N/A	UoS0014	156/162	9V	G	L	A	A	D	E	N
123304	N/A	UoS0176	156/162	9V	G	L	A	A	D	E	N
N/A	481	VICE0160	156/162	9V	R	V	T	A	D	D	D
N/A	484	VICE0163	156/162	9V	R	V	T	A	D	D	D
N/A	494	VICE0173	156/162	9V	R	V	T	A	D	D	D
N/A	650	VICE0328	156/162	9V	R	V	T	A	D	D	D
N/A	1307	VICE0713	156/162	9V	R	V	T	A	D	D	D
82193	N/A	SMRU976	280	9V	R	V	T	T	E	E	N
82388	N/A	SMRU2065	280	9V	R	V	T	T	E	E	N
82611	N/A	SMRU2837	280	9V	R	V	T	T	E	E	N
82622	N/A	SMRU2896	280	9V	R	V	T	T	E	E	N

83247	N/A	SMRU234	280	9V	R	V	T	T	E	E	N
83524	N/A	SMRU2590	280	9V	R	V	T	T	E	E	N
83546	N/A	SMRU2895	280	9V	R	V	T	T	E	E	N
109828	N/A	SMRU1174	280	9V	R	V	T	T	E	E	N
111546	N/A	SMRU1363	280	9V	R	V	T	T	E	E	N
113088	N/A	SMRU483	280	9V	R	V	T	T	E	E	N
121533	N/A	UoS3059	Sing4424	9V	R	V	T	T	E	E	N

####

	wzh	wzd	wze	wchA						wchO	wcjA	wzy	
460	238	197	163	161	173	203	210	212	268	48		179	355
L	V	F	A	I	H	E	L	S	V	H		T	V
L	V	F	A	I	H	E	L	S	V	H		T	V
L	V	F	A	I	H	E	L	S	V	H		T	V
L	V	F	A	I	H	E	L	S	V	H		T	V
L	V	F	A	I	H	E	L	S	V	H		T	V
L	V	F	A	I	H	E	L	S	V	H		T	V
L	V	F	A	I	H	E	L	S	V	H		T	V
L	V	F	A	I	H	E	L	S	V	H		T	V
L	V	F	A	I	H	E	L	S	V	H		T	V
L	V	F	A	I	H	E	L	S	V	H		T	V
L	V	F	A	I	H	E	L	S	V	H		T	V
L	V	F	A	I	H	E	L	S	V	H		T	V
L	V	F	A	I	H	E	L	S	V	H		T	V
L	V	F	A	I	H	E	L	S	V	H		T	V
L	V	F	V	I	H	E	L	S	V	H		T	V
L	V	F	V	I	H	E	L	S	V	H		T	V
L	V	F	A	I	H	E	L	S	V	H		T	V
L	V	F	V	I	H	E	L	S	V	H		T	V
L	V	F	A	I	H	E	L	S	V	H		T	V
L	V	F	A	I	H	E	L	S	V	H		T	V
L	V	F	A	I	H	E	L	S	V	H		I	V
L	V	F	A	I	H	E	L	S	V	H		T	V
L	V	F	A	I	H	E	L	S	V	H		T	V
L	V	F	A	I	H	E	L	S	V	H		T	V
L	V	F	A	I	H	E	L	S	V	H		T	V
L	V	F	A	I	H	E	L	S	V	H		T	V
L	V	F	A	I	H	E	L	S	V	H		T	V
L	V	F	A	I	H	E	L	S	V	H		T	V
L	V	F	A	I	H	E	L	S	V	H		T	V
L	V	F	A	I	H	E	L	S	V	H		T	V
L	V	F	A	I	H	E	L	S	V	H		T	V
L	V	F	A	I	H	E	L	S	V	H		T	V
L	V	F	A	I	H	E	L	S	V	H		T	V
L	V	F	A	I	H	E	L	S	V	H		T	V
L	V	F	A	I	H	E	L	S	V	H		T	V
L	V	F	A	I	H	E	L	S	V	H		T	V
L	V	F	A	I	H	E	L	S	V	H		T	V
L	V	F	A	I	H	E	L	S	V	H		T	V
L	V	F	A	I	H	E	L	S	V	H		T	V
L	V	F	A	I	H	E	L	S	V	H		T	V
L	V	F	A	I	H	E	L	S	V	H		T	V
L	V	F	A	I	H	E	L	S	V	H		T	V
L	V	F	A	I	H	E	L	S	V	H		T	V
L	V	F	A	I	H	E	L	S	V	H		T	V
L	V	F	A	I	H	E	L	S	V	H		T	V
L	V	F	A	I	H	E	L	S	V	H		T	V
L	V	F	A	I	H	E	L	S	V	H		T	V
L	V	F	A	I	H	E	L	S	V	H		T	V
L	V	F	A	I	H	E	L	S	V	H		T	V
L	V	F	A	I	H	E	L	S	V	H		T	V
L	V	F	A	I	H	E	L	S	V	H		T	V
L	V	F	A	I	H	E	L	S	V	H		T	V
L	V	F	A	I	H	E	L	S	V	H		T	V
L	V	F	A	I	H	E	L	S	V	H		T	V
L	V	F	A	I	H	E	L	S	V	Y		T	V
L	V	F	A	I	H	E	L	S	V	Y		T	V
L	V	F	A	I	H	E	L	S	V	Y		T	V
L	V	F	A	I	H	E	L	S	V	Y		T	V
L	V	F	A	I	H	E	L	S	V	Y		T	V
M	I	L	A	V	N	G	F	Y	M	H		T	I
M	I	L	A	V	N	G	F	Y	M	H		T	I
M	I	L	A	V	N	E	F	S	M	H		T	I
M	I	L	A	V	N	E	F	S	M	H		T	I

M	I	L	A	V	N	E	F	S	M	H		T	I
M	I	L	A	V	N	G	F	Y	M	H		T	I
M	I	L	A	V	N	E	F	S	M	H		T	I
M	I	L	A	V	N	G	F	Y	M	H		T	I
M	I	L	A	V	N	G	F	Y	M	H		T	I
M	I	L	A	V	N	E	F	S	M	H		T	I
M	I	L	A	V	N	E	F	S	M	H		T	I

99.4

wcjB	wzx	wcjC				wcjD	ugd	wcjE		
81	399	109	167	325	349			86	198	264
K	V	F	K	G	Q			I	D	P
E	V	F	K	G	Q			I	D	P
E	V	F	K	G	Q			I	D	P
E	V	F	K	G	Q			I	D	P
E	V	F	K	G	Q			I	D	P
E	V	F	K	G	Q			I	D	P
E	V	F	K	G	Q			I	D	P
E	V	F	K	G	Q			I	D	P
E	V	F	K	G	Q			I	D	P
E	V	F	K	G	Q			I	D	P
E	V	F	K	G	Q			I	D	P
E	V	F	K	G	Q			I	D	P
E	V	F	K	G	Q			I	D	P
E	V	F	K	G	Q			I	D	P
E	V	F	K	G	Q			I	D	P
E	V	F	K	G	Q			I	D	P
E	V	F	K	G	Q			I	D	P
E	V	F	K	G	Q			I	D	-
E	V	F	K	G	Q			I	D	P
E	V	F	K	G	Q			I	D	P
E	V	F	K	G	Q			I	D	P
E	V	F	K	G	Q			I	D	P
E	V	F	K	G	Q			I	D	P
E	V	F	K	G	Q			I	D	P
E	V	F	K	G	Q			I	D	P
E	V	F	K	G	Q			I	D	P
E	V	F	K	G	Q			I	D	P
E	V	F	K	G	Q			I	D	P
E	V	F	K	G	Q			I	D	P
E	V	F	K	G	Q			I	D	P
K	V	F	K	G	Q			I	D	P
E	V	F	K	G	Q			I	D	P
E	V	F	K	G	Q			I	D	P
E	V	F	K	G	Q			I	D	P
E	V	F	K	G	Q			I	D	P
E	V	F	K	G	Q			I	D	P
E	V	F	K	G	Q			I	D	P
E	V	F	K	G	Q			I	D	P
E	V	F	K	G	Q			I	D	P
E	V	F	K	G	Q			I	D	P
E	V	F	K	G	Q			I	D	P
E	V	F	K	G	Q			I	D	P
E	V	F	K	G	Q			I	D	P
E	V	F	K	G	Q			I	D	P
E	V	F	K	G	Q			I	D	P
E	V	F	K	G	Q			I	D	P
E	V	F	K	G	Q			I	D	P
E	V	F	K	G	Q			I	D	P
E	V	F	K	G	Q			I	D	P
E	V	F	K	G	Q			I	D	P
E	V	F	K	G	Q			I	D	P
E	V	F	K	G	Q			I	D	P
E	V	F	K	G	Q			I	D	P
E	V	F	K	G	Q			I	D	P
E	V	F	K	G	Q			I	D	P
E	V	F	K	G	Q			I	D	P
E	V	F	K	G	Q			I	D	P
E	V	F	K	G	Q			I	D	P
E	V	F	K	G	Q			I	D	P
E	V	L	R	C	R			V	G	S
E	V	L	R	C	R			V	G	S
E	V	L	R	C	R			V	G	S
E	V	L	R	C	R			V	G	S

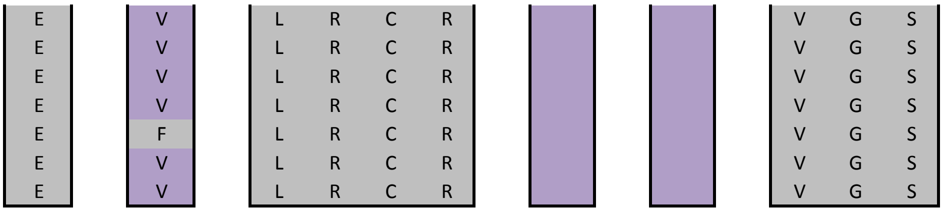
E V L R C R V G S
E V L R C R V G S
E V L R C R V G S
E V L R C R V G S
E F L R C R V G S
E V L R C R V G S
E V L R C R V G S

99.7

rmlst ID	PubMLST id	ENA	Name	ST	CC	Orig_sero	Orig_sero_method
N/A	3099	N/A	AP200	62	62	11A	unknown
N/A	15518	N/A	SA53	7247	1010	11A	unknown
N/A	35881	N/A	MLV-016	62	62	11A	unknown
N/A	35926	N/A	SP11-BS70	62	62	11	unknown
32922	32927	ERR047144	UoS5113	62	62	unknown	WGS-based
40133	33014	ERR044997	UoS2130	62	62	unknown	WGS-based
40134	33015	ERR044998	UoS2131	62	62	unknown	WGS-based
40137	33018	ERR046143	UoS4002	62	62	unknown	WGS-based
40140	33021	ERR046199	UoS4226	62	62	unknown	WGS-based
55125	33063	ERR044944	UoS0302	62	62	unknown	WGS-based
115828	33766	ERR044992	UoS2082	4695	62	unknown	WGS-based
115838	33776	ERR045012	UoS2205	62	62	unknown	WGS-based
115855	33792	ERR047145	UoS5114	62	62	unknown	WGS-based
121366	33810	ERR046069	UoS3077	62	62	unknown	WGS-based
121367	33811	ERR048225	UoS3006	62	62	unknown	WGS-based
121368	33812	ERR046078	UoS3115	62	62	unknown	WGS-based
121369	33813	ERR045394	UoS5003	62	62	unknown	WGS-based
121370	33814	ERR045370	UoS4339	62	62	unknown	WGS-based
121371	33815	ERR046165	UoS4083	62	62	unknown	WGS-based
121373	33817	ERR046118	UoS3252	62	62	unknown	WGS-based
121382	33826	ERR045419	UoS5122	62	62	unknown	WGS-based
121384	33828	ERR046190	UoS4180	62	62	unknown	WGS-based
121385	33829	ERR045364	UoS4320	62	62	unknown	WGS-based
121386	33830	ERR046136	UoS3301	62	62	unknown	WGS-based
121387	33831	ERR046148	UoS4020	62	62	unknown	WGS-based
121388	33832	ERR046146	UoS4012	62	62	unknown	WGS-based
121389	33833	ERR045387	UoS4382	62	62	unknown	WGS-based
121390	33834	ERR045047	UoS2315	62	62	unknown	WGS-based
121397	33841	ERR044971	UoS2027	4019	62	unknown	WGS-based
121445	33889	ERR046149	UoS4024	62	62	unknown	WGS-based
121449	33893	ERR046151	UoS4028	62	62	unknown	WGS-based
121455	33899	ERR045392	UoS4393	62	62	unknown	WGS-based
121458	33902	ERR044976	UoS2037	62	62	unknown	WGS-based
121459	33903	ERR044975	UoS2034	62	62	unknown	WGS-based
123192	34116	ERR044896	UoS0160	62	62	unknown	WGS-based
123215	34139	ERR045428	UoS5152	9137	62	unknown	WGS-based
123218	34142	ERR046048	UoS3021	62	62	unknown	WGS-based
123228	34152	ERR046174	UoS4109	62	62	unknown	WGS-based
123239	34163	ERR047158	UoS5275	62	62	unknown	WGS-based
123318	34185	ERR046095	UoS3176	62	62	unknown	WGS-based
123332	34199	ERR047141	UoS5108	62	62	unknown	WGS-based
N/A	15485	N/A	11A/2	7214	Sing7214	11A	Quellung
N/A	15435	N/A	11B/2	7165	Sing7165	11B	Quellung
N/A	36300	N/A	11D/1	62	62	11D	Quellung
N/A	15483	N/A	11F/2	7212	5995/7212	11F	Quellung
N/A	15471	N/A	11C/1	7201	124	11C	Quellung
32689	27168	ERR273516	SMRU637	99	99	11A	latex & Quellung
32928	27159	ERR047973	SMRU296	99	99	11A	latex & Quellung
32929	27160	ERR047974	SMRU297	99	99	11A	latex & Quellung
33114	27169	ERR054515	SMRU775	99	99	11A	latex & Quellung
33872	27157	ERR047948	SMRU271	99	99	11A	latex & Quellung
33916	27151	ERR049107	SMRU173	99	99	11A	latex & Quellung
34118	27174	ERR054520	SMRU780	99	99	11A	latex & Quellung
34158	27186	ERR057943	SMRU1962	99	99	11A	latex & Quellung
40117	27149	ERR039634	SMRU75	99	99	11A	latex & Quellung
40158	27158	ERR047949	SMRU272	99	99	11A	latex & Quellung
40169	27161	ERR048008	SMRU379	99	99	11A	latex & Quellung
40186	27162	ERR048130	SMRU453	99	99	11A	latex & Quellung

40195	27152	ERR049117	SMRU183	99	99	11A	latex & Quellung
40236	27176	ERR051616	SMRU1070	99	99	11A	latex & Quellung
40239	27177	ERR051670	SMRU1124	99	99	11A	latex & Quellung
40599	27200	ERR072204	SMRU3055	99	99	11A	latex & Quellung
44871	27175	ERR051614	SMRU1068	99	99	11A	latex & Quellung
44984	27191	ERR066300	SMRU2333	99	99	11A	latex & Quellung
55133	27178	ERR051664	SMRU1118	99	99	11A	latex & Quellung
55160	27193	ERR066333	SMRU2366	99	99	11A	latex & Quellung
55963	27198	ERR084171	SMRU2945	10480	99	11A	latex & Quellung
82100	27147	ERR039619	SMRU60	99	99	11A	latex & Quellung
82101	27148	ERR039620	SMRU61	99	99	11A	latex & Quellung
82137	27163	ERR048152	SMRU475	99	99	11A	latex & Quellung
82224	27156	ERR052586	SMRU249	99	99	11A	latex & Quellung
82349	27183	ERR057877	SMRU1896	99	99	11A	latex & Quellung
82424	27187	ERR063944	SMRU2151	99	99	11A	latex & Quellung
82480	27194	ERR064144	SMRU2406	10477	99	11A	latex & Quellung
82577	27196	ERR067823	SMRU2600	99	99	11A	latex & Quellung
82729	27201	ERR084227	SMRU3001	99	99	11A	latex & Quellung
82736	27189	ERR084264	SMRU2246	99	99	11A	latex & Quellung
83323	27181	ERR054489	SMRU1541	99	99	11A	latex & Quellung
83492	27190	ERR066278	SMRU2311	99	99	11A	latex & Quellung
83496	27192	ERR066302	SMRU2335	99	99	11A	latex & Quellung
109675	27150	ERR039643	SMRU84	99	99	11A	latex & Quellung
109745	27165	ERR052626	SMRU553	99	99	11A	latex & Quellung
109746	27166	ERR052627	SMRU554	99	99	11A	latex & Quellung
109845	27180	ERR056779	SMRU1441	99	99	11A	latex & Quellung
109957	27195	ERR064158	SMRU2420	99	99	11A	latex & Quellung
109992	27155	ERR084300	SMRU3098	99	99	11A	latex & Quellung
109996	27167	ERR084307	SMRU3105	99	99	11A	latex & Quellung
111417	27199	ERR084194	SMRU2968	99	99	11A	latex & Quellung
111466	27184	ERR057886	SMRU1905	10480	99	11A	latex & Quellung
111531	27179	ERR054306	SMRU1213	99	99	11A	latex & Quellung
111560	27153	ERR050063	SMRU200	99	99	11A	latex & Quellung
113126	27185	ERR057885	SMRU1904	10480	99	11A	latex & Quellung
113755	27164	ERR048165	SMRU488	99	99	11A	latex & Quellung
115501	27154	ERR050062	SMRU199	99	99	11A	latex & Quellung
115518	27172	ERR052624	SMRU767	99	99	11A	latex & Quellung
31281	29509	ERR065308	R34-3052	62	62	11A	Quellung
31283	29375	ERR065310	R34-3054	62	62	11A	Quellung
31298	29176	ERR065326	R34-3071	62	62	11A	Quellung
31327	29510	ERR067968	R34-3170	62	62	11A	Quellung
31348	29511	ERR068000	R34-3095	62	62	11A	Quellung
31380	29415	ERR068040	R34-3147	1913	62	11A	Quellung
31381	29512	ERR068041	R34-3149	408	62	11A	Quellung
31385	29513	ERR068047	R34-3156	62	62	11A	Quellung
31438	29177	ERR069801	242588	62	62	11A	Quellung
31440	29514	ERR069804	263137	62	62	11A	Quellung
31444	29416	ERR069808	277394	10505	1934/10505	11A	Quellung
31457	29417	ERR069822	335574	62	62	11A	Quellung
31468	29418	ERR069837	386329	1913	62	11A	Quellung
31469	29515	ERR069839	389109	62	62	11A	Quellung
31544	29516	ERR129037	BR1039	62	62	11A	Quellung
31554	29517	ERR129154	LE4111	62	62	11A	Quellung
31556	29518	ERR129159	LE4127	62	62	11A	Quellung
32078	29519	ERR067985	R34-3080	62	62	11A	Quellung
32079	29520	ERR067986	R34-3081	62	62	11A	Quellung
32094	29521	ERR068046	R34-3155	62	62	11A	Quellung
32148	29419	ERR069702	R34-3203	62	62	11A	Quellung
32152	29178	ERR069707	R34-3208	62	62	11A	Quellung

32167	29179	ERR069727	R34-3228	62	62	11A	Quellung
32188	29522	ERR069760	102720	62	62	11A	Quellung
32328	29420	ERR124240	ND6151	62	62	11A	Quellung
32329	29180	ERR124242	ND6156	62	62	11A	Quellung
32344	29523	ERR124268	NP7095	62	62	11A	Quellung
32355	29181	ERR124291	PT8037	62	62	11A	Quellung
32360	29182	ERR124302	PT8072	62	62	11A	Quellung
32387	29524	ERR129053	BR1085	156	156/162	11A	Quellung
32398	29183	ERR129068	BR1117	62	62	11A	Quellung
32408	29525	ERR129079	CH2053	62	62	11A	Quellung
32409	29421	ERR129080	CH2054	3276	62	11A	Quellung
32446	29184	ERR129127	LE4005	62	62	11A	Quellung
32455	29422	ERR129139	LE4050	62	62	11A	Quellung
32475	29423	ERR129177	MD5070	62	62	11A	Quellung
32476	29186	ERR129178	MD5071	62	62	11A	Quellung
32498	29526	ERR129211	ND6085	62	62	11A	Quellung
32500	29376	ERR129214	ND6103	62	62	11A	Quellung
32501	29424	ERR129215	ND6104	62	62	11A	Quellung
33179	29425	ERR065964	459747	62	62	11A	Quellung
33260	29527	ERR069713	R34-3214	62	62	11A	Quellung
33264	29426	ERR069752	74124	1913	62	11A	Quellung
33420	29187	ERR124296	PT8053	62	62	11A	Quellung
33422	29188	ERR124298	PT8063	62	62	11A	Quellung
33440	29377	ERR129093	CH2091	62	62	11A	Quellung
33445	29528	ERR129131	LE4018	62	62	11A	Quellung
33457	29189	ERR129210	ND6073	62	62	11A	Quellung
35279	29190	ERR124246	NP7010	62	62	11A	Quellung
N/A	33400	ERR470448	VICE0281	62	62	not done	--
N/A	36122	ERR755348	VICE1684	62	62	not done	--
N/A	34491	ERR690868	VICE0616	62	62	not done	--
N/A	33689	ERR540578	VICE1063	62	62	not done	--
N/A	34430	ERR540585	VICE1072	62	62	not done	--
N/A	33691	ERR540587	VICE1075	62	62	not done	--
N/A	33694	ERR540597	VICE1091	62	62	not done	--
N/A	34217	ERR540675	VICE1187	62	62	not done	--
N/A	34407	ERR540676	VICE1190	62	62	not done	--
N/A	33599	ERR540680	VICE1198	62	62	not done	--
N/A	34437	ERR690923	VICE1199a	62	62	not done	--
N/A	33600	ERR540681	VICE1200	62	62	not done	--
N/A	33565	ERR540394	VICE1309a	62	62	not done	--
N/A	34508	ERR690935	VICE1310a	62	62	not done	--
N/A	33615	ERR540396	VICE1312	62	62	not done	--
N/A	33617	ERR540417	VICE1337	62	62	not done	--
N/A	33577	ERR540423	VICE1344a	62	62	not done	--
N/A	34547	ERR690946	VICE1357	62	62	not done	--
N/A	34533	ERR690948	VICE1359	62	62	not done	--
N/A	34243	ERR689530	VICE1454	62	62	not done	--
N/A	34417	ERR689543	VICE1469	62	62	not done	--
N/A	36174	ERR755487	VICE1752	62	62	not done	--
N/A	36175	ERR755488	VICE1753	62	62	not done	--
N/A	36177	ERR755490	VICE1755	62	62	not done	--
N/A	36178	ERR755491	VICE1756	62	62	not done	--
N/A	36179	ERR755492	VICE1757	62	62	not done	--
N/A	36180	ERR755493	VICE1758	62	62	not done	--
N/A	36187	ERR755501	VICE1764	62	62	not done	--
N/A	33322	ERR460484	VICE0603	62	62	not done	--
N/A	36124	ERR755351	VICE1687	62	62	not done	--
N/A	33671	ERR540522	VICE0923	62	62	not done	--
N/A	34496	ERR690885	VICE0924a	62	62	not done	--

N/A	34451	ERR690891	VICE0953	62	62	not done	--
N/A	33698	ERR540604	VICE1100	62	62	not done	--
N/A	34442	ERR540335	VICE1241a	62	62	not done	--
N/A	33548	ERR540342	VICE1250	62	62	not done	--
N/A	34253	ERR697239	VICE1504	62	62	not done	--
N/A	34311	ERR697271	VICE1539a	62	62	not done	--
N/A	36201	ERR755516	VICE1784	62	62	not done	--
N/A	29949	ERR755520	VICE1791a	10345	62	not done	--
N/A	34524	ERR690863	VICE0259	62	62	not done	--
N/A	33264	ERR449827	VICE0649	62	62	not done	--
N/A	33225	ERR470261	VICE0316	62	62	not done	--
N/A	33206	ERR470201	VICE0421	62	62	not done	--
N/A	33373	ERR460421	VICE0507	62	62	not done	--
N/A	33387	ERR460491	VICE0615	62	62	not done	--
N/A	34278	ERR540479	VICE0633	62	62	not done	--
N/A	34279	ERR540480	VICE0648	62	62	not done	--
N/A	33187	ERR449847	VICE0671	62	62	not done	--
N/A	33581	ERR540483	VICE0685	62	62	not done	--
N/A	34541	ERR690872	VICE0715	62	62	not done	--
N/A	33164	ERR470324	VICE0870	62	62	not done	--
N/A	33677	ERR540541	VICE0978	62	62	not done	--
N/A	33465	ERR540645	VICE1144	62	62	not done	--
N/A	33708	ERR540658	VICE1158	62	62	not done	--
N/A	33555	ERR540372	VICE1282a	62	62	not done	--
N/A	34413	ERR689488	VICE1408	62	62	not done	--
N/A	34230	ERR689494	VICE1415	62	62	not done	--
N/A	34421	ERR697249	VICE1516	62	62	not done	--
N/A	34266	ERR697293	VICE1561	62	62	not done	--
N/A	36050	ERR755429	VICE1596	62	62	not done	--
N/A	36094	ERR755317	VICE1651a	62	62	not done	--
N/A		ERR755318	VICE1651b	62	62	not done	--
N/A	33204	ERR470192	VICE0412	62	62	not done	--
N/A	34504	ERR690928	VICE1241b	62	62	not done	--
N/A	33556	ERR540373	VICE1282b	62	62	not done	--
N/A	36226	ERR755548	VICE1828	62	62	not done	--
N/A	36233	ERR755557	VICE1839	62	62	not done	--
N/A	36242	ERR755567	VICE1850	62	62	not done	--
N/A	36243	ERR755568	VICE1851	62	62	not done	--
N/A	36244	ERR755569	VICE1852	62	62	not done	--
N/A	36259	ERR755587	VICE1870	62	62	not done	--
N/A	36263	ERR755592	VICE1876	62	62	not done	--
N/A	36277	ERR755615	VICE1897	62	62	not done	--
N/A	36278	ERR755616	VICE1898	62	62	not done	--
N/A	36283	ERR755621	VICE1904	62	62	not done	--
N/A	33626	ERR540448	VICE0262	62	62	not done	--
N/A	34580	ERR388819	VICE0181	1009	Sing1009	not done	--

Seq_sero	Hybrid	Hybrid type	Country	Region	carriage/disease	Source
11A	No	N/A	Italy	unknown	disease	CSF
11A	No	N/A	South Africa	KwaZulu	disease	blood
11A	No	N/A	unknown	unknown	carriage	nasopharynx
11A	No	N/A	USA	Pennsylvania	disease	nasopharynx
11A	No	N/A	United Kingdom	Southampton	carriage	nasopharynx
11A	No	N/A	United Kingdom	Southampton	carriage	nasopharynx
11A	No	N/A	United Kingdom	Southampton	carriage	nasopharynx
11A	No	N/A	United Kingdom	Southampton	carriage	nasopharynx
11A	No	N/A	United Kingdom	Southampton	carriage	nasopharynx
11A	No	N/A	United Kingdom	Southampton	carriage	nasopharynx
11A	No	N/A	United Kingdom	Southampton	carriage	nasopharynx
11A	No	N/A	United Kingdom	Southampton	carriage	nasopharynx
11A	No	N/A	United Kingdom	Southampton	carriage	nasopharynx
11A	No	N/A	United Kingdom	Southampton	carriage	nasopharynx
11A	No	N/A	United Kingdom	Southampton	carriage	nasopharynx
11A	No	N/A	United Kingdom	Southampton	carriage	nasopharynx
11A	No	N/A	United Kingdom	Southampton	carriage	nasopharynx
11A	No	N/A	United Kingdom	Southampton	carriage	nasopharynx
11A	No	N/A	United Kingdom	Southampton	carriage	nasopharynx
11A	No	N/A	United Kingdom	Southampton	carriage	nasopharynx
11A	No	N/A	United Kingdom	Southampton	carriage	nasopharynx
11A	No	N/A	United Kingdom	Southampton	carriage	nasopharynx
11A	No	N/A	United Kingdom	Southampton	carriage	nasopharynx
11A	No	N/A	United Kingdom	Southampton	carriage	nasopharynx
11A	No	N/A	United Kingdom	Southampton	carriage	nasopharynx
11A	No	N/A	United Kingdom	Southampton	carriage	nasopharynx
11A	No	N/A	United Kingdom	Southampton	carriage	nasopharynx
11A	No	N/A	United Kingdom	Southampton	carriage	nasopharynx
11A	No	N/A	United Kingdom	Southampton	carriage	nasopharynx
11A	No	N/A	United Kingdom	Southampton	carriage	nasopharynx
11A	No	N/A	United Kingdom	Southampton	carriage	nasopharynx
11A	No	N/A	United Kingdom	Southampton	carriage	nasopharynx
11A	No	N/A	United Kingdom	Southampton	carriage	nasopharynx
11A	No	N/A	United Kingdom	Southampton	carriage	nasopharynx
11A	No	N/A	United Kingdom	Southampton	carriage	nasopharynx
11A	No	N/A	United Kingdom	Southampton	carriage	nasopharynx
11A	No	N/A	United Kingdom	Southampton	carriage	nasopharynx
11A	No	N/A	United Kingdom	Southampton	carriage	nasopharynx
11A	No	N/A	United Kingdom	Southampton	carriage	nasopharynx
11A	No	N/A	United Kingdom	Southampton	carriage	nasopharynx
11A	No	N/A	United Kingdom	Southampton	carriage	nasopharynx
11A	No	N/A	Denmark	unknown	unknown	unknown
11B	No	N/A	Denmark	unknown	unknown	unknown
11D	No	N/A	Denmark	unknown	unknown	unknown
11F	No	N/A	Denmark	unknown	unknown	unknown
11C	No	N/A	USA	unknown	unknown	unknown
11X	No	N/A	Thailand	Maela	carriage	nasopharynx
11X	No	N/A	Thailand	Maela	carriage	nasopharynx
11X	No	N/A	Thailand	Maela	carriage	nasopharynx
11X	No	N/A	Thailand	Maela	carriage	nasopharynx
11X	No	N/A	Thailand	Maela	carriage	nasopharynx
11X	No	N/A	Thailand	Maela	carriage	nasopharynx
11X	No	N/A	Thailand	Maela	carriage	nasopharynx
11X	No	N/A	Thailand	Maela	carriage	nasopharynx
11X	No	N/A	Thailand	Maela	carriage	nasopharynx
11X	No	N/A	Thailand	Maela	carriage	nasopharynx
11X	No	N/A	Thailand	Maela	carriage	nasopharynx
11X	No	N/A	Thailand	Maela	carriage	nasopharynx

11X	No	N/A	Thailand	Maela	carriage	nasopharynx
11X	No	N/A	Thailand	Maela	carriage	nasopharynx
11X	No	N/A	Thailand	Maela	carriage	nasopharynx
11X	No	N/A	Thailand	Maela	carriage	nasopharynx
11X	No	N/A	Thailand	Maela	carriage	nasopharynx
11X	No	N/A	Thailand	Maela	carriage	nasopharynx
11X	No	N/A	Thailand	Maela	carriage	nasopharynx
11X	No	N/A	Thailand	Maela	carriage	nasopharynx
11X	No	N/A	Thailand	Maela	carriage	nasopharynx
11X	No	N/A	Thailand	Maela	carriage	nasopharynx
11X	No	N/A	Thailand	Maela	carriage	nasopharynx
11X	No	N/A	Thailand	Maela	carriage	nasopharynx
11X	No	N/A	Thailand	Maela	carriage	nasopharynx
11X	No	N/A	Thailand	Maela	carriage	nasopharynx
11X	No	N/A	Thailand	Maela	carriage	nasopharynx
11X	No	N/A	Thailand	Maela	carriage	nasopharynx
11X	No	N/A	Thailand	Maela	carriage	nasopharynx
11X	No	N/A	Thailand	Maela	carriage	nasopharynx
11X	No	N/A	Thailand	Maela	carriage	nasopharynx
11X	No	N/A	Thailand	Maela	carriage	nasopharynx
11X	No	N/A	Thailand	Maela	carriage	nasopharynx
11X	No	N/A	Thailand	Maela	carriage	nasopharynx
11X	No	N/A	Thailand	Maela	carriage	nasopharynx
11X	No	N/A	Thailand	Maela	carriage	nasopharynx
11X	No	N/A	Thailand	Maela	carriage	nasopharynx
11X	No	N/A	Thailand	Maela	carriage	nasopharynx
11X	No	N/A	Thailand	Maela	carriage	nasopharynx
11X	No	N/A	Thailand	Maela	carriage	nasopharynx
11X	No	N/A	Thailand	Maela	carriage	nasopharynx
11X	No	N/A	Thailand	Maela	carriage	nasopharynx
11X	No	N/A	Thailand	Maela	carriage	nasopharynx
11X	No	N/A	Thailand	Maela	carriage	nasopharynx
11X	No	N/A	Thailand	Maela	carriage	nasopharynx
11X	No	N/A	Thailand	Maela	carriage	nasopharynx
11X	No	N/A	Thailand	Maela	carriage	nasopharynx
11X	No	N/A	Thailand	Maela	carriage	nasopharynx
11X	No	N/A	Thailand	Maela	carriage	nasopharynx
11A	No	N/A	USA	Massachusetts	carriage	nasopharynx
11A	No	N/A	USA	Massachusetts	carriage	nasopharynx
11A	No	N/A	USA	Massachusetts	carriage	nasopharynx
11A	No	N/A	USA	Massachusetts	carriage	nasopharynx
11A	No	N/A	USA	Massachusetts	carriage	nasopharynx
11A	No	N/A	USA	Massachusetts	carriage	nasopharynx
11A	No	N/A	USA	Massachusetts	carriage	nasopharynx
11A	No	N/A	USA	Massachusetts	carriage	nasopharynx
11A	No	N/A	USA	Massachusetts	carriage	nasopharynx
11A	No	N/A	USA	Massachusetts	carriage	nasopharynx
11A	Yes	11A/11nov	USA	Massachusetts	carriage	nasopharynx
11A	No	N/A	USA	Massachusetts	carriage	nasopharynx
11A	No	N/A	USA	Massachusetts	carriage	nasopharynx
11A	No	N/A	USA	Massachusetts	carriage	nasopharynx
11A	No	N/A	USA	Massachusetts	carriage	nasopharynx
11A	No	N/A	USA	Massachusetts	carriage	nasopharynx
11A	No	N/A	USA	Massachusetts	carriage	nasopharynx
11A	No	N/A	USA	Massachusetts	carriage	nasopharynx
11A	No	N/A	USA	Massachusetts	carriage	nasopharynx
11A	No	N/A	USA	Massachusetts	carriage	nasopharynx
11A	No	N/A	USA	Massachusetts	carriage	nasopharynx
11A	No	N/A	USA	Massachusetts	carriage	nasopharynx

11A	No	N/A	USA	Massachusetts	carriage	nasopharynx
11A	No	N/A	USA	Massachusetts	carriage	nasopharynx
11A	No	N/A	USA	Massachusetts	carriage	nasopharynx
11A	No	N/A	USA	Massachusetts	carriage	nasopharynx
11A	No	N/A	USA	Massachusetts	carriage	nasopharynx
11A	No	N/A	USA	Massachusetts	carriage	nasopharynx
11A	No	N/A	USA	Massachusetts	carriage	nasopharynx
11A	No	N/A	USA	Massachusetts	carriage	nasopharynx
11A	No	N/A	USA	Massachusetts	carriage	nasopharynx
11A	No	N/A	USA	Massachusetts	carriage	nasopharynx
11A	No	N/A	USA	Massachusetts	carriage	nasopharynx
11A	No	N/A	USA	Massachusetts	carriage	nasopharynx
11A	No	N/A	USA	Massachusetts	carriage	nasopharynx
11A	No	N/A	USA	Massachusetts	carriage	nasopharynx
11A	No	N/A	USA	Massachusetts	carriage	nasopharynx
11A	No	N/A	USA	Massachusetts	carriage	nasopharynx
11A	No	N/A	USA	Massachusetts	carriage	nasopharynx
11A	No	N/A	USA	Massachusetts	carriage	nasopharynx
11A	No	N/A	USA	Massachusetts	carriage	nasopharynx
11A	No	N/A	USA	Massachusetts	carriage	nasopharynx
11A	No	N/A	USA	Massachusetts	carriage	nasopharynx
11A	No	N/A	USA	Massachusetts	carriage	nasopharynx
11A	No	N/A	USA	Massachusetts	carriage	nasopharynx
11A	No	N/A	USA	Massachusetts	carriage	nasopharynx
11A	No	N/A	USA	Massachusetts	carriage	nasopharynx
11A	No	N/A	USA	Massachusetts	carriage	nasopharynx
11A	No	N/A	USA	Massachusetts	carriage	nasopharynx
11A	No	N/A	Iceland	Akureyri	disease	middle ear fluid
11A	No	N/A	Iceland	Akureyri	disease	sputum
11A	No	N/A	Iceland	Hafnarfjörður	disease	middle ear fluid
11A	No	N/A	Iceland	Hafnarfjörður	carriage	nasopharynx
11A	No	N/A	Iceland	Hafnarfjörður	carriage	nasopharynx
11A	No	N/A	Iceland	Hafnarfjörður	carriage	nasopharynx
11A	No	N/A	Iceland	Hafnarfjörður	carriage	nasopharynx
11A	No	N/A	Iceland	Hafnarfjörður	carriage	nasopharynx
11A	No	N/A	Iceland	Hafnarfjörður	carriage	nasopharynx
11A	No	N/A	Iceland	Hafnarfjörður	carriage	nasopharynx
11A	No	N/A	Iceland	Hafnarfjörður	carriage	nasopharynx
11A	No	N/A	Iceland	Hafnarfjörður	carriage	nasopharynx
11A	No	N/A	Iceland	Hafnarfjörður	carriage	nasopharynx
11A	No	N/A	Iceland	Hafnarfjörður	carriage	nasopharynx
11A	No	N/A	Iceland	Hafnarfjörður	carriage	nasopharynx
11A	No	N/A	Iceland	Hafnarfjörður	carriage	nasopharynx
11A	No	N/A	Iceland	Hafnarfjörður	carriage	nasopharynx
11A	No	N/A	Iceland	Hafnarfjörður	carriage	nasopharynx
11A	No	N/A	Iceland	Hafnarfjörður	carriage	nasopharynx
11A	No	N/A	Iceland	Hafnarfjörður	carriage	nasopharynx
11A	No	N/A	Iceland	Hafnarfjörður	carriage	nasopharynx
11A	No	N/A	Iceland	Hafnarfjörður	carriage	nasopharynx
11A	No	N/A	Iceland	Hafnarfjörður	carriage	nasopharynx
11A	No	N/A	Iceland	Hafnarfjörður	carriage	nasopharynx
11A	No	N/A	Iceland	Hafnarfjörður	carriage	nasopharynx
11A	No	N/A	Iceland	Hafnarfjörður	carriage	nasopharynx
11A	No	N/A	Iceland	Hafnarfjörður	carriage	nasopharynx
11A	No	N/A	Iceland	Hnífsdalur	disease	middle ear fluid
11A	No	N/A	Iceland	Kópavogur	disease	sputum
11A	No	N/A	Iceland	Kópavogur	carriage	nasopharynx
11A	No	N/A	Iceland	Kópavogur	carriage	nasopharynx

11A	No	N/A	Iceland	Kópavogur	carriage	nasopharynx
11A	No	N/A	Iceland	Kópavogur	carriage	nasopharynx
11A	No	N/A	Iceland	Kópavogur	carriage	nasopharynx
11A	No	N/A	Iceland	Kópavogur	carriage	nasopharynx
11A	No	N/A	Iceland	Kópavogur	carriage	nasopharynx
11A	No	N/A	Iceland	Kópavogur	carriage	nasopharynx
11A	No	N/A	Iceland	Kópavogur	carriage	nasopharynx
11A	No	N/A	Iceland	Kópavogur	carriage	nasopharynx
11A	No	N/A	Iceland	Mosfellsbær	disease	blood
11A	No	N/A	Iceland	Mosfellsbær	disease	sputum
11A	No	N/A	Iceland	Ólafsfjörður	disease	middle ear fluid
11A	No	N/A	Iceland	Reykjavík	disease	middle ear fluid
11A	No	N/A	Iceland	Reykjavík	disease	middle ear fluid
11A	No	N/A	Iceland	Reykjavík	disease	middle ear fluid
11A	No	N/A	Iceland	Reykjavík	disease	sputum
11A	No	N/A	Iceland	Reykjavík	disease	sputum
11A	No	N/A	Iceland	Reykjavík	disease	sputum
11A	No	N/A	Iceland	Reykjavík	disease	sputum
11A	No	N/A	Iceland	Reykjavík	disease	sputum
11A	No	N/A	Iceland	Reykjavík	carriage	nasopharynx
11A	No	N/A	Iceland	Reykjavík	carriage	nasopharynx
11A	No	N/A	Iceland	Reykjavík	carriage	nasopharynx
11A	No	N/A	Iceland	Reykjavík	carriage	nasopharynx
11A	No	N/A	Iceland	Reykjavík	carriage	nasopharynx
11A	No	N/A	Iceland	Reykjavík	carriage	nasopharynx
11A	No	N/A	Iceland	Reykjavík	carriage	nasopharynx
11A	No	N/A	Iceland	Reykjavík	disease	blood
11A	No	N/A	Iceland	Reykjavík	carriage	nasopharynx
11A	No	N/A	Iceland	Reykjavík	disease	lung aspirate
11A	No	N/A	Iceland	Reykjavík	disease	middle ear fluid
11A	No	N/A	Iceland	Reykjavík	disease	middle ear fluid
11A	No	N/A	Iceland	Selfoss	disease	middle ear fluid
11A	No	N/A	Iceland	unknown	carriage	nasopharynx
11A	No	N/A	Iceland	unknown	carriage	nasopharynx
11A	No	N/A	Iceland	unknown	carriage	nasopharynx
11A	No	N/A	Iceland	unknown	carriage	nasopharynx
11A	No	N/A	Iceland	unknown	carriage	nasopharynx
11A	No	N/A	Iceland	unknown	carriage	nasopharynx
11A	No	N/A	Iceland	unknown	carriage	nasopharynx
11A	No	N/A	Iceland	unknown	carriage	nasopharynx
11A	No	N/A	Iceland	unknown	carriage	nasopharynx
11A	No	N/A	Iceland	unknown	carriage	nasopharynx
11A	No	N/A	Iceland	unknown	carriage	nasopharynx
11A	No	N/A	Iceland	unknown	disease	blood
11E	No	N/A	Iceland	Reykjavík	disease	blood
11B	No	N/A	Iceland	Reykjavík	disease	blood

Year	Publication	Reference	vaccine status
2003	Genbank (336)	N/A	pre
1983	Genbank (336)	N/A	pre
unknown	Genbank (336)	N/A	unknown
unknown	Genbank (336)	N/A	unknown
2006-2011	Gladstone UK PCV	PMID: 25776920	unknown
2006-2011	Gladstone UK PCV	PMID: 25776920	unknown
2006-2011	Gladstone UK PCV	PMID: 25776920	unknown
2006-2011	Gladstone UK PCV	PMID: 25776920	unknown
2006-2011	Gladstone UK PCV	PMID: 25776920	unknown
2006-2011	Gladstone UK PCV	PMID: 25776920	unknown
2006-2011	Gladstone UK PCV	PMID: 25776920	unknown
2006-2011	Gladstone UK PCV	PMID: 25776920	unknown
2006-2011	Gladstone UK PCV	PMID: 25776920	unknown
2006-2011	Gladstone UK PCV	PMID: 25776920	unknown
2006-2011	Gladstone UK PCV	PMID: 25776920	unknown
2006-2011	Gladstone UK PCV	PMID: 25776920	unknown
2006-2011	Gladstone UK PCV	PMID: 25776920	unknown
2006-2011	Gladstone UK PCV	PMID: 25776920	unknown
2006-2011	Gladstone UK PCV	PMID: 25776920	unknown
2006-2011	Gladstone UK PCV	PMID: 25776920	unknown
2006-2011	Gladstone UK PCV	PMID: 25776920	unknown
2006-2011	Gladstone UK PCV	PMID: 25776920	unknown
2006-2011	Gladstone UK PCV	PMID: 25776920	unknown
2006-2011	Gladstone UK PCV	PMID: 25776920	unknown
2006-2011	Gladstone UK PCV	PMID: 25776920	unknown
2006-2011	Gladstone UK PCV	PMID: 25776920	unknown
2006-2011	Gladstone UK PCV	PMID: 25776920	unknown
2006-2011	Gladstone UK PCV	PMID: 25776920	unknown
2006-2011	Gladstone UK PCV	PMID: 25776920	unknown
2006-2011	Gladstone UK PCV	PMID: 25776920	unknown
2006-2011	Gladstone UK PCV	PMID: 25776920	unknown
2006-2011	Gladstone UK PCV	PMID: 25776920	unknown
2006-2011	Gladstone UK PCV	PMID: 25776920	unknown
2006-2011	Gladstone UK PCV	PMID: 25776920	unknown
2006-2011	Gladstone UK PCV	PMID: 25776920	unknown
2006-2011	Gladstone UK PCV	PMID: 25776920	unknown
2006-2011	Gladstone UK PCV	PMID: 25776920	unknown
2006-2011	Gladstone UK PCV	PMID: 25776920	unknown
2006-2011	Gladstone UK PCV	PMID: 25776920	unknown
2006-2011	Gladstone UK PCV	PMID: 25776920	unknown
2006-2011	Gladstone UK PCV	PMID: 25776920	unknown
1939	Historical	this publication	pre
1940	Historical	this publication	pre
1986	Historical	this publication	pre
1952	Historical	this publication	pre
1957	Historical	this publication	pre
2008	Maela	PMID: 24509479	pre
2008	Maela	PMID: 24509479	pre
2008	Maela	PMID: 24509479	pre
2008	Maela	PMID: 24509479	pre
2008	Maela	PMID: 24509479	pre
2008	Maela	PMID: 24509479	pre
2008	Maela	PMID: 24509479	pre
2009	Maela	PMID: 24509479	pre
2008	Maela	PMID: 24509479	pre
2008	Maela	PMID: 24509479	pre
2008	Maela	PMID: 24509479	pre
2008	Maela	PMID: 24509479	pre

2008	Maela	PMID: 24509479	pre
2008	Maela	PMID: 24509479	pre
2009	Maela	PMID: 24509479	pre
2010	Maela	PMID: 24509479	pre
2008	Maela	PMID: 24509479	pre
2010	Maela	PMID: 24509479	pre
2009	Maela	PMID: 24509479	pre
2010	Maela	PMID: 24509479	pre
2010	Maela	PMID: 24509479	pre
2008	Maela	PMID: 24509479	pre
2008	Maela	PMID: 24509479	pre
2008	Maela	PMID: 24509479	pre
2008	Maela	PMID: 24509479	pre
2009	Maela	PMID: 24509479	pre
2009	Maela	PMID: 24509479	pre
2010	Maela	PMID: 24509479	pre
2010	Maela	PMID: 24509479	pre
2010	Maela	PMID: 24509479	pre
2009	Maela	PMID: 24509479	pre
2009	Maela	PMID: 24509479	pre
2010	Maela	PMID: 24509479	pre
2010	Maela	PMID: 24509479	pre
2008	Maela	PMID: 24509479	pre
2008	Maela	PMID: 24509479	pre
2008	Maela	PMID: 24509479	pre
2009	Maela	PMID: 24509479	pre
2010	Maela	PMID: 24509479	pre
2008	Maela	PMID: 24509479	pre
2008	Maela	PMID: 24509479	pre
2010	Maela	PMID: 24509479	pre
2009	Maela	PMID: 24509479	pre
2009	Maela	PMID: 24509479	pre
2008	Maela	PMID: 24509479	pre
2009	Maela	PMID: 24509479	pre
2008	Maela	PMID: 24509479	pre
2008	Maela	PMID: 24509479	pre
2008	Maela	PMID: 24509479	pre
2004	Massachusetts	PMID: 23644493	post
2004	Massachusetts	PMID: 23644493	post
2004	Massachusetts	PMID: 23644493	post
2004	Massachusetts	PMID: 23644493	post
2004	Massachusetts	PMID: 23644493	post
2004	Massachusetts	PMID: 23644493	post
2004	Massachusetts	PMID: 23644493	post
2004	Massachusetts	PMID: 23644493	post
2001	Massachusetts	PMID: 23644493	pre
2001	Massachusetts	PMID: 23644493	pre
2001	Massachusetts	PMID: 23644493	pre
2001	Massachusetts	PMID: 23644493	pre
2001	Massachusetts	PMID: 23644493	pre
2001	Massachusetts	PMID: 23644493	pre
2007	Massachusetts	PMID: 23644493	post
2007	Massachusetts	PMID: 23644493	post
2007	Massachusetts	PMID: 23644493	post
2004	Massachusetts	PMID: 23644493	post
2004	Massachusetts	PMID: 23644493	post
2004	Massachusetts	PMID: 23644493	post
2004	Massachusetts	PMID: 23644493	post
2004	Massachusetts	PMID: 23644493	post

2004	Massachusetts	PMID: 23644493	post
2001	Massachusetts	PMID: 23644493	pre
2007	Massachusetts	PMID: 23644493	post
2007	Massachusetts	PMID: 23644493	post
2007	Massachusetts	PMID: 23644493	post
2007	Massachusetts	PMID: 23644493	post
2007	Massachusetts	PMID: 23644493	post
2007	Massachusetts	PMID: 23644493	post
2007	Massachusetts	PMID: 23644493	post
2007	Massachusetts	PMID: 23644493	post
2007	Massachusetts	PMID: 23644493	post
2007	Massachusetts	PMID: 23644493	post
2007	Massachusetts	PMID: 23644493	post
2007	Massachusetts	PMID: 23644493	post
2007	Massachusetts	PMID: 23644493	post
2007	Massachusetts	PMID: 23644493	post
2007	Massachusetts	PMID: 23644493	post
2007	Massachusetts	PMID: 23644493	post
2001	Massachusetts	PMID: 23644493	pre
2004	Massachusetts	PMID: 23644493	post
2001	Massachusetts	PMID: 23644493	pre
2007	Massachusetts	PMID: 23644493	post
2007	Massachusetts	PMID: 23644493	post
2007	Massachusetts	PMID: 23644493	post
2007	Massachusetts	PMID: 23644493	post
2007	Massachusetts	PMID: 23644493	post
2007	Massachusetts	PMID: 23644493	post
2009	VICE	this publication	pre
2013	VICE	this publication	post
2012	VICE	this publication	post
2010	VICE	this publication	pre
2010	VICE	this publication	pre
2010	VICE	this publication	pre
2010	VICE	this publication	pre
2011	VICE	this publication	post
2011	VICE	this publication	post
2011	VICE	this publication	post
2011	VICE	this publication	post
2011	VICE	this publication	post
2012	VICE	this publication	post
2012	VICE	this publication	post
2012	VICE	this publication	post
2012	VICE	this publication	post
2012	VICE	this publication	post
2012	VICE	this publication	post
2012	VICE	this publication	post
2013	VICE	this publication	post
2013	VICE	this publication	post
2014	VICE	this publication	post
2014	VICE	this publication	post
2014	VICE	this publication	post
2014	VICE	this publication	post
2014	VICE	this publication	post
2014	VICE	this publication	post
2014	VICE	this publication	post
2012	VICE	this publication	post
2013	VICE	this publication	post
2009	VICE	this publication	pre
2009	VICE	this publication	pre

2009	VICE	this publication	pre
2010	VICE	this publication	pre
2011	VICE	this publication	post
2011	VICE	this publication	post
2013	VICE	this publication	post
2013	VICE	this publication	post
2014	VICE	this publication	post
2014	VICE	this publication	post
2012	VICE	this publication	post
2009	VICE	this publication	pre
2009	VICE	this publication	pre
2010	VICE	this publication	pre
2011	VICE	this publication	post
2012	VICE	this publication	post
2009	VICE	this publication	pre
2009	VICE	this publication	pre
2009	VICE	this publication	pre
2010	VICE	this publication	pre
2010	VICE	this publication	pre
2009	VICE	this publication	pre
2009	VICE	this publication	pre
2010	VICE	this publication	pre
2010	VICE	this publication	pre
2011	VICE	this publication	post
2012	VICE	this publication	post
2012	VICE	this publication	post
2014	VICE	this publication	post
2013	VICE	this publication	post
2013	VICE	this publication	post
2013	VICE	this publication	post
2013	VICE	this publication	post
2010	VICE	this publication	pre
2011	VICE	this publication	post
2011	VICE	this publication	post
2014	VICE	this publication	post
2014	VICE	this publication	post
2014	VICE	this publication	post
2014	VICE	this publication	post
2014	VICE	this publication	post
2014	VICE	this publication	post
2014	VICE	this publication	post
2014	VICE	this publication	post
2014	VICE	this publication	post
2014	VICE	this publication	post
2012	VICE	this publication	post
2010	VICE	this publication	pre

SEROTYPE 11A ALL ISOLATES

rmlst ID	BIGS id	Name	CC	Seq_sero	wzg 19	wzg 105	wzg 255	wzg 353	wzg 448	wzh	6
		11A Reference			I	A	R	D	P		T
N/A	311	MLV-016	62	11A	I	A	R	D	P		T
N/A	403	VICE0870	62	11A	I	A	R	D	P		T
N/A	581	VICE0259	62	11A	I	A	R	D	P		A
N/A	603	VICE0281	62	11A	I	A	R	D	P		T
N/A	638	VICE0316	62	11A	I	A	R	D	P		T
N/A	734	VICE0412	62	11A	I	A	R	D	P		T
N/A	743	VICE0421	62	11A	I	V	R	D	P		T
N/A	829	VICE0507	62	11A	I	A	R	D	P		T
N/A	925	VICE0603	62	11A	I	A	R	D	P		T
N/A	937	VICE0615	62	11A	I	A	R	D	P		T
N/A	938	VICE0616	62	11A	I	A	R	D	P		T
N/A	955	VICE0633	62	11A	I	V	R	D	P		T
N/A	1063	SA53	1010	11A	I	A	R	D	P		T
N/A	1101	SP11-BS70	62	11A	I	A	R	D	P		T
N/A	1116	AP200	62	11A	I	A	R	D	P		T
N/A	1242	VICE0648	62	11A	I	A	R	D	P		T
N/A	1243	VICE0649	62	11A	I	A	R	D	P		T
N/A	1265	VICE0671	62	11A	I	A	R	D	P		T
N/A	1279	VICE0685	62	11A	I	A	R	D	P		T
N/A	1309	VICE0715	62	11A	I	A	R	D	P		T
N/A	1474	VICE0923	62	11A	I	A	R	D	P		T
N/A	1475	VICE0924a	62	11A	I	A	R	D	P		T
N/A	1504	VICE0953	62	11A	I	V	R	D	P		T
N/A	1529	VICE0978	62	11A	I	A	R	D	P		T
N/A	1614	VICE1063	62	11A	I	A	R	D	P		T
N/A	1623	VICE1072	62	11A	I	A	R	D	P		T
N/A	1626	VICE1075	62	11A	I	A	R	D	P		T
N/A	1642	VICE1091	62	11A	I	A	R	D	P		T
N/A	1651	VICE1100	62	11A	I	A	R	D	P		T
N/A	2022	VICE1144	62	11A	I	A	R	D	P		T
N/A	2036	VICE1158	62	11A	I	A	R	D	P		T
N/A	2065	VICE1187	62	11A	I	A	R	D	P		T
N/A	2068	VICE1190	62	11A	I	A	R	D	P		T
N/A	2076	VICE1198	62	11A	I	A	R	D	P		T
N/A	2077	VICE1199a	62	11A	I	A	R	D	P		T
N/A	2078	VICE1200	62	11A	I	A	R	D	P		T
N/A	2119	VICE1241a	62	11A	I	A	R	D	P		T
N/A	2128	VICE1250	62	11A	I	A	R	D	P		T
N/A	2160	VICE1282a	62	11A	I	A	R	D	P		T
N/A	2187	VICE1309a	62	11A	I	A	R	D	P		T
N/A	2188	VICE1310a	62	11A	I	A	R	D	P		T
N/A	2190	VICE1312	62	11A	I	A	R	D	P		T
N/A	2215	VICE1337	62	11A	I	A	R	D	P		T
N/A	2222	VICE1344a	62	11A	I	A	R	D	P		T
N/A	2235	VICE1357	62	11A	I	A	R	D	P		T
N/A	2237	VICE1359	62	11A	I	A	R	D	P		T
N/A	2286	VICE1408	62	11A	I	V	R	D	P		T
N/A	2293	VICE1415	62	11A	I	A	R	D	P		T
N/A	2332	VICE1454	62	11A	I	V	R	D	P		T
N/A	2347	VICE1469	62	11A	I	A	R	D	P		T
N/A	2382	VICE1504	62	11A	I	V	R	D	P		T
N/A	2394	VICE1516	62	11A	I	A	R	D	P		T
N/A	2417	VICE1539a	62	11A	I	A	R	D	P		T
N/A	2439	VICE1561	62	11A	I	V	R	D	P		T
N/A	2499	VICE1241b	62	11A	I	A	R	D	P		T
N/A	2509	VICE1282b	62	11A	I	A	R	D	P		T
N/A	2549	VICE1596	62	11A	I	A	R	D	P		T
N/A	2604	VICE1651a	62	11A	I	A	R	D	P		T
N/A	2637	VICE1684	62	11A	I	A	R	D	P		T
N/A	2640	VICE1687	62	11A	I	A	R	D	P		T
N/A	2707	VICE1752	62	11A	I	A	R	D	P		T
N/A	2708	VICE1753	62	11A	I	A	R	D	P		T

N/A	2710	VICE1755	62	11A	I	A	R	D	P			T
N/A	2711	VICE1756	62	11A	I	A	R	D	P			T
N/A	2712	VICE1757	62	11A	I	A	R	D	P			T
N/A	2713	VICE1758	62	11A	I	A	R	D	P			T
N/A	2721	VICE1764	62	11A	I	A	R	D	P			T
N/A	2743	VICE1784	62	11A	I	A	R	D	P			T
N/A	2750	VICE1791a	62	11A	I	A	R	D	P			T
N/A	2789	VICE1828	62	11A	I	A	R	D	P			T
N/A	2802	VICE1839	62	11A	I	A	R	D	P			T
N/A	2815	VICE1850	62	11A	I	A	R	D	P			T
N/A	2816	VICE1851	62	11A	I	A	R	D	P			T
N/A	2817	VICE1852	62	11A	I	A	R	D	P			T
N/A	2835	VICE1870	62	11A	I	A	R	D	P			T
N/A	2842	VICE1876	62	11A	I	A	R	D	P			T
N/A	2869	VICE1897	62	11A	I	A	R	D	P			T
N/A	2870	VICE1898	62	11A	I	A	R	D	P			T
N/A	2876	VICE1904	62	11A	I	A	R	D	P			T
N/A	2882	VICE1651b	62	11A	I	A	R	D	P			T
N/A	2914	11A/2	Sing7214	11A	I	A	R	D	P			T
31281	N/A	R34-3052	62	11A	I	A	R	D	P			T
31283	N/A	R34-3054	62	11A	I	A	R	D	P			T
31298	N/A	R34-3071	62	11A	I	A	R	D	P			T
31327	N/A	R34-3170	62	11A	I	A	R	D	P			T
31348	N/A	R34-3095	62	11A	I	A	R	D	P			T
31380	N/A	R34-3147	62	11A	I	A	R	D	P			T
31381	N/A	R34-3149	62	11A	I	A	R	D	P			T
31385	N/A	R34-3156	62	11A	I	A	R	D	P			T
31438	N/A	242588	62	11A	I	A	R	D	P			T
31440	N/A	263137	62	11A	I	A	R	D	P			T
31457	N/A	335574	62	11A	I	A	R	D	P			T
31468	N/A	386329	62	11A	I	A	R	D	P			T
31469	N/A	389109	62	11A	I	A	R	D	P			T
31544	N/A	BR1039	62	11A	I	A	R	D	P			T
31554	N/A	LE4111	62	11A	I	A	R	D	P			T
31556	N/A	LE4127	62	11A	I	A	R	D	P			T
32078	N/A	R34-3080	62	11A	I	A	R	D	P			T
32079	N/A	R34-3081	62	11A	I	A	R	D	P			T
32094	N/A	R34-3155	62	11A	I	A	R	D	P			T
32148	N/A	R34-3203	62	11A	I	A	R	D	P			T
32152	N/A	R34-3208	62	11A	I	A	R	D	P			T
32167	N/A	R34-3228	62	11A	I	A	R	D	P			T
32188	N/A	102720	62	11A	I	A	R	D	P			T
32328	N/A	ND6151	62	11A	I	A	R	D	P			T
32329	N/A	ND6156	62	11A	I	A	R	D	P			T
32344	N/A	NP7095	62	11A	I	A	R	D	P			T
32355	N/A	PT8037	62	11A	I	A	R	D	P			T
32360	N/A	PT8072	62	11A	I	A	R	D	P			T
32387	N/A	BR1085	156/162	11A	I	A	R	D	P			T
32398	N/A	BR1117	62	11A	I	A	R	D	P			T
32408	N/A	CH2053	62	11A	I	A	R	D	P			T
32409	N/A	CH2054	62	11A	I	A	R	D	P			T
32446	N/A	LE4005	62	11A	I	A	R	D	P			T
32455	N/A	LE4050	62	11A	I	A	R	D	P			T
32475	N/A	MD5070	62	11A	I	A	R	D	P			T
32476	N/A	MD5071	62	11A	I	A	R	D	P			T
32498	N/A	ND6085	62	11A	I	A	R	D	P			T
32500	N/A	ND6103	62	11A	I	A	R	D	P			T
32501	N/A	ND6104	62	11A	I	A	R	D	P			T
32922	N/A	UoS5113	62	11A	I	A	R	D	P			T
33179	N/A	459747	62	11A	I	A	R	D	P			T
33260	N/A	R34-3214	62	11A	I	A	R	D	P			T
33264	N/A	74124	62	11A	I	A	R	D	P			T
33420	N/A	PT8053	62	11A	I	A	R	D	P			T
33422	N/A	PT8063	62	11A	I	A	R	D	P			T
33440	N/A	CH2091	62	11A	I	A	R	D	P			T
33445	N/A	LE4018	62	11A	I	A	R	D	P			T
33457	N/A	ND6073	62	11A	I	A	R	D	P			T

					wzg					wzh	
35279	N/A	NP7010	62	11A	I	A	R	D	P		T
40133	N/A	UoS2130	62	11A	I	A	R	D	P		T
40134	N/A	UoS2131	62	11A	I	A	R	D	P		T
40137	N/A	UoS4002	62	11A	I	A	R	D	P		T
40140	N/A	UoS4226	62	11A	I	A	R	G	P		T
55125	N/A	UoS0302	62	11A	I	A	R	D	P		T
115828	N/A	UoS2082	62	11A	I	A	R	D	P		T
115838	N/A	UoS2205	62	11A	I	A	R	D	P		T
115855	N/A	UoS5114	62	11A	I	A	R	D	P		T
121366	N/A	UoS3077	62	11A	I	A	R	D	P		T
121367	N/A	UoS3006	62	11A	I	A	Q	D	P		T
121368	N/A	UoS3115	62	11A	I	A	R	D	P		T
121369	N/A	UoS5003	62	11A	I	A	R	D	P		T
121370	N/A	UoS4339	62	11A	I	A	R	D	P		T
121371	N/A	UoS4083	62	11A	I	A	R	D	P		T
121373	N/A	UoS3252	62	11A	I	A	R	D	P		T
121382	N/A	UoS5122	62	11A	I	A	R	D	P		T
121384	N/A	UoS4180	62	11A	I	A	R	D	P		T
121385	N/A	UoS4320	62	11A	I	A	R	D	P		T
121386	N/A	UoS3301	62	11A	I	A	R	D	P		T
121387	N/A	UoS4020	62	11A	I	A	R	D	L		T
121388	N/A	UoS4012	62	11A	I	A	R	D	P		T
121389	N/A	UoS4382	62	11A	I	A	R	D	P		T
121390	N/A	UoS2315	62	11A	I	A	R	D	P		T
121397	N/A	UoS2027	62	11A	M	A	R	D	P		T
121445	N/A	UoS4024	62	11A	I	A	R	D	P		T
121449	N/A	UoS4028	62	11A	I	A	R	D	P		T
121455	N/A	UoS4393	62	11A	I	A	R	D	P		T
121458	N/A	UoS2037	62	11A	I	A	R	D	P		T
121459	N/A	UoS2034	62	11A	I	A	R	D	P		T
123192	N/A	UoS0160	62	11A	I	A	R	D	P		T
123215	N/A	UoS5152	62	11A	I	A	R	D	P		T
123218	N/A	UoS3021	62	11A	I	A	R	D	P		T
123228	N/A	UoS4109	62	11A	I	A	R	D	P		T
123239	N/A	UoS5275	62	11A	I	A	R	D	P		T
123318	N/A	UoS3176	62	11A	I	A	R	D	P		T
123332	N/A	UoS5108	62	11A	I	A	R	D	P		T
31444	N/A	277394	1934/10505	11A	I	A	R	D	P		T

SEROTYPE 11A/11X HYBRID SEQUENCE

					wzg								
					9	80	123	162	328	403	463	469	472
		11A reference			G	V	E	N	I	D	N	S	T
32689		11X	99	11nov	R	L	D	D	V	N	D	V	A
31444	N/A	277394	1934/10505	11hy	G	V	E	N	I	D	N	S	T

SEROTYPE 11A ISOLATES (NO HYBRID SEQUENCES)

					wzg					wzh	
rmlst ID	BIGS id	Name	CC	Seq_sero	19	105	255	353	448		6
		11A Reference			I	A	R	D	P		T
N/A	1063	SA53	1010	11A	I	A	R	D	P		T
N/A	2914	11A/2	Sing7214	11A	I	A	R	D	P		T
32387	N/A	BR1085	156/162	11A	I	A	R	D	P		T
N/A	311	MLV-016	62	11A	I	A	R	D	P		T
N/A	403	VICE0870	62	11A	I	A	R	D	P		T
N/A	581	VICE0259	62	11A	I	A	R	D	P		A
N/A	603	VICE0281	62	11A	I	A	R	D	P		T
N/A	638	VICE0316	62	11A	I	A	R	D	P		T
N/A	734	VICE0412	62	11A	I	A	R	D	P		T
N/A	743	VICE0421	62	11A	I	V	R	D	P		T
N/A	829	VICE0507	62	11A	I	A	R	D	P		T
N/A	925	VICE0603	62	11A	I	A	R	D	P		T

N/A	937	VICE0615	62	11A	I	A	R	D	P	T
N/A	938	VICE0616	62	11A	I	A	R	D	P	T
N/A	955	VICE0633	62	11A	I	V	R	D	P	T
N/A	1101	SP11-BS70	62	11A	I	A	R	D	P	T
N/A	1116	AP200	62	11A	I	A	R	D	P	T
N/A	1242	VICE0648	62	11A	I	A	R	D	P	T
N/A	1243	VICE0649	62	11A	I	A	R	D	P	T
N/A	1265	VICE0671	62	11A	I	A	R	D	P	T
N/A	1279	VICE0685	62	11A	I	A	R	D	P	T
N/A	1309	VICE0715	62	11A	I	A	R	D	P	T
N/A	1474	VICE0923	62	11A	I	A	R	D	P	T
N/A	1475	VICE0924a	62	11A	I	A	R	D	P	T
N/A	1504	VICE0953	62	11A	I	V	R	D	P	T
N/A	1529	VICE0978	62	11A	I	A	R	D	P	T
N/A	1614	VICE1063	62	11A	I	A	R	D	P	T
N/A	1623	VICE1072	62	11A	I	A	R	D	P	T
N/A	1626	VICE1075	62	11A	I	A	R	D	P	T
N/A	1642	VICE1091	62	11A	I	A	R	D	P	T
N/A	1651	VICE1100	62	11A	I	A	R	D	P	T
N/A	2022	VICE1144	62	11A	I	A	R	D	P	T
N/A	2036	VICE1158	62	11A	I	A	R	D	P	T
N/A	2065	VICE1187	62	11A	I	A	R	D	P	T
N/A	2068	VICE1190	62	11A	I	A	R	D	P	T
N/A	2076	VICE1198	62	11A	I	A	R	D	P	T
N/A	2077	VICE1199a	62	11A	I	A	R	D	P	T
N/A	2078	VICE1200	62	11A	I	A	R	D	P	T
N/A	2119	VICE1241a	62	11A	I	A	R	D	P	T
N/A	2128	VICE1250	62	11A	I	A	R	D	P	T
N/A	2160	VICE1282a	62	11A	I	A	R	D	P	T
N/A	2187	VICE1309a	62	11A	I	A	R	D	P	T
N/A	2188	VICE1310a	62	11A	I	A	R	D	P	T
N/A	2190	VICE1312	62	11A	I	A	R	D	P	T
N/A	2215	VICE1337	62	11A	I	A	R	D	P	T
N/A	2222	VICE1344a	62	11A	I	A	R	D	P	T
N/A	2235	VICE1357	62	11A	I	A	R	D	P	T
N/A	2237	VICE1359	62	11A	I	A	R	D	P	T
N/A	2286	VICE1408	62	11A	I	V	R	D	P	T
N/A	2293	VICE1415	62	11A	I	A	R	D	P	T
N/A	2332	VICE1454	62	11A	I	V	R	D	P	T
N/A	2347	VICE1469	62	11A	I	A	R	D	P	T
N/A	2382	VICE1504	62	11A	I	V	R	D	P	T
N/A	2394	VICE1516	62	11A	I	A	R	D	P	T
N/A	2417	VICE1539a	62	11A	I	A	R	D	P	T
N/A	2439	VICE1561	62	11A	I	V	R	D	P	T
N/A	2499	VICE1241b	62	11A	I	A	R	D	P	T
N/A	2509	VICE1282b	62	11A	I	A	R	D	P	T
N/A	2549	VICE1596	62	11A	I	A	R	D	P	T
N/A	2604	VICE1651a	62	11A	I	A	R	D	P	T
N/A	2637	VICE1684	62	11A	I	A	R	D	P	T
N/A	2640	VICE1687	62	11A	I	A	R	D	P	T
N/A	2707	VICE1752	62	11A	I	A	R	D	P	T
N/A	2708	VICE1753	62	11A	I	A	R	D	P	T
N/A	2710	VICE1755	62	11A	I	A	R	D	P	T
N/A	2711	VICE1756	62	11A	I	A	R	D	P	T
N/A	2712	VICE1757	62	11A	I	A	R	D	P	T
N/A	2713	VICE1758	62	11A	I	A	R	D	P	T
N/A	2721	VICE1764	62	11A	I	A	R	D	P	T
N/A	2743	VICE1784	62	11A	I	A	R	D	P	T
N/A	2750	VICE1791a	62	11A	I	A	R	D	P	T
N/A	2789	VICE1828	62	11A	I	A	R	D	P	T
N/A	2802	VICE1839	62	11A	I	A	R	D	P	T
N/A	2815	VICE1850	62	11A	I	A	R	D	P	T
N/A	2816	VICE1851	62	11A	I	A	R	D	P	T
N/A	2817	VICE1852	62	11A	I	A	R	D	P	T
N/A	2835	VICE1870	62	11A	I	A	R	D	P	T
N/A	2842	VICE1876	62	11A	I	A	R	D	P	T
N/A	2869	VICE1897	62	11A	I	A	R	D	P	T

N/A	2870	VICE1898	62	11A	I	A	R	D	P		T
N/A	2876	VICE1904	62	11A	I	A	R	D	P		T
N/A	2882	VICE1651b	62	11A	I	A	R	D	P		T
31281	N/A	R34-3052	62	11A	I	A	R	D	P		T
31283	N/A	R34-3054	62	11A	I	A	R	D	P		T
31298	N/A	R34-3071	62	11A	I	A	R	D	P		T
31327	N/A	R34-3170	62	11A	I	A	R	D	P		T
31348	N/A	R34-3095	62	11A	I	A	R	D	P		T
31380	N/A	R34-3147	62	11A	I	A	R	D	P		T
31381	N/A	R34-3149	62	11A	I	A	R	D	P		T
31385	N/A	R34-3156	62	11A	I	A	R	D	P		T
31438	N/A	242588	62	11A	I	A	R	D	P		T
31440	N/A	263137	62	11A	I	A	R	D	P		T
31457	N/A	335574	62	11A	I	A	R	D	P		T
31468	N/A	386329	62	11A	I	A	R	D	P		T
31469	N/A	389109	62	11A	I	A	R	D	P		T
31544	N/A	BR1039	62	11A	I	A	R	D	P		T
31554	N/A	LE4111	62	11A	I	A	R	D	P		T
31556	N/A	LE4127	62	11A	I	A	R	D	P		T
32078	N/A	R34-3080	62	11A	I	A	R	D	P		T
32079	N/A	R34-3081	62	11A	I	A	R	D	P		T
32094	N/A	R34-3155	62	11A	I	A	R	D	P		T
32148	N/A	R34-3203	62	11A	I	A	R	D	P		T
32152	N/A	R34-3208	62	11A	I	A	R	D	P		T
32167	N/A	R34-3228	62	11A	I	A	R	D	P		T
32188	N/A	102720	62	11A	I	A	R	D	P		T
32328	N/A	ND6151	62	11A	I	A	R	D	P		T
32329	N/A	ND6156	62	11A	I	A	R	D	P		T
32344	N/A	NP7095	62	11A	I	A	R	D	P		T
32355	N/A	PT8037	62	11A	I	A	R	D	P		T
32360	N/A	PT8072	62	11A	I	A	R	D	P		T
32398	N/A	BR1117	62	11A	I	A	R	D	P		T
32408	N/A	CH2053	62	11A	I	A	R	D	P		T
32409	N/A	CH2054	62	11A	I	A	R	D	P		T
32446	N/A	LE4005	62	11A	I	A	R	D	P		T
32455	N/A	LE4050	62	11A	I	A	R	D	P		T
32475	N/A	MD5070	62	11A	I	A	R	D	P		T
32476	N/A	MD5071	62	11A	I	A	R	D	P		T
32498	N/A	ND6085	62	11A	I	A	R	D	P		T
32500	N/A	ND6103	62	11A	I	A	R	D	P		T
32501	N/A	ND6104	62	11A	I	A	R	D	P		T
32922	N/A	UoS5113	62	11A	I	A	R	D	P		T
33179	N/A	459747	62	11A	I	A	R	D	P		T
33260	N/A	R34-3214	62	11A	I	A	R	D	P		T
33264	N/A	74124	62	11A	I	A	R	D	P		T
33420	N/A	PT8053	62	11A	I	A	R	D	P		T
33422	N/A	PT8063	62	11A	I	A	R	D	P		T
33440	N/A	CH2091	62	11A	I	A	R	D	P		T
33445	N/A	LE4018	62	11A	I	A	R	D	P		T
33457	N/A	ND6073	62	11A	I	A	R	D	P		T
35279	N/A	NP7010	62	11A	I	A	R	D	P		T
40133	N/A	UoS2130	62	11A	I	A	R	D	P		T
40134	N/A	UoS2131	62	11A	I	A	R	D	P		T
40137	N/A	UoS4002	62	11A	I	A	R	D	P		T
40140	N/A	UoS4226	62	11A	I	A	R	G	P		T
55125	N/A	UoS0302	62	11A	I	A	R	D	P		T
115828	N/A	UoS2082	62	11A	I	A	R	D	P		T
115838	N/A	UoS2205	62	11A	I	A	R	D	P		T
115855	N/A	UoS5114	62	11A	I	A	R	D	P		T
121366	N/A	UoS3077	62	11A	I	A	R	D	P		T
121367	N/A	UoS3006	62	11A	I	A	Q	D	P		T
121368	N/A	UoS3115	62	11A	I	A	R	D	P		T
121369	N/A	UoS5003	62	11A	I	A	R	D	P		T
121370	N/A	UoS4339	62	11A	I	A	R	D	P		T
121371	N/A	UoS4083	62	11A	I	A	R	D	P		T
121373	N/A	UoS3252	62	11A	I	A	R	D	P		T
121382	N/A	UoS5122	62	11A	I	A	R	D	P		T

121384	N/A	UoS4180	62	11A	I	A	R	D	P		T
121385	N/A	UoS4320	62	11A	I	A	R	D	P		T
121386	N/A	UoS3301	62	11A	I	A	R	D	P		T
121387	N/A	UoS4020	62	11A	I	A	R	D	L		T
121388	N/A	UoS4012	62	11A	I	A	R	D	P		T
121389	N/A	UoS4382	62	11A	I	A	R	D	P		T
121390	N/A	UoS2315	62	11A	I	A	R	D	P		T
121397	N/A	UoS2027	62	11A	M	A	R	D	P		T
121445	N/A	UoS4024	62	11A	I	A	R	D	P		T
121449	N/A	UoS4028	62	11A	I	A	R	D	P		T
121455	N/A	UoS4393	62	11A	I	A	R	D	P		T
121458	N/A	UoS2037	62	11A	I	A	R	D	P		T
121459	N/A	UoS2034	62	11A	I	A	R	D	P		T
123192	N/A	UoS0160	62	11A	I	A	R	D	P		T
123215	N/A	UoS5152	62	11A	I	A	R	D	P		T
123218	N/A	UoS3021	62	11A	I	A	R	D	P		T
123228	N/A	UoS4109	62	11A	I	A	R	D	P		T
123239	N/A	UoS5275	62	11A	I	A	R	D	P		T
123318	N/A	UoS3176	62	11A	I	A	R	D	P		T
123332	N/A	UoS5108	62	11A	I	A	R	D	P		T

wzd					wze															
29	51	91	135	224	7	23	51	118	119	163	185	207	213	217	8	78	154	155	188	212
A	T	V	A	G	S	A	T	Q	A	A	D	K	E	S	P	A	N	E	A	S
A	T	V	A	G	S	A	T	Q	A	A	D	K	E	S	P	A	N	E	A	S
A	T	V	A	G	S	A	T	Q	A	A	D	K	E	S	P	A	N	E	A	S
A	T	V	A	G	S	A	T	Q	A	A	D	K	E	S	P	A	N	E	A	S
A	T	V	A	G	S	A	T	Q	A	A	D	K	E	S	P	A	N	E	A	S
A	T	V	A	G	S	A	T	Q	A	A	D	K	E	S	P	A	N	E	A	S
A	T	V	A	G	S	A	T	Q	A	A	D	K	E	S	P	A	N	E	A	S
A	T	V	V	G	S	A	T	Q	A	A	D	K	E	S	P	A	N	E	A	S
A	T	V	A	G	S	A	T	Q	A	A	D	K	E	S	P	A	N	E	A	S
A	T	V	A	G	S	A	T	Q	A	A	D	K	E	S	P	A	N	E	A	S
A	T	V	A	G	S	A	T	Q	A	A	D	K	E	S	P	A	N	E	A	S
A	T	V	A	G	S	A	M	Q	A	A	D	K	E	S	P	A	N	E	A	S
A	T	V	V	G	S	A	T	Q	A	A	D	K	E	S	P	A	N	E	A	S
A	T	V	A	G	S	A	T	Q	A	A	D	K	E	S	P	A	N	E	A	S
A	T	V	A	G	S	A	T	Q	A	A	D	K	E	S	P	A	N	E	A	S
A	T	V	A	G	S	A	T	Q	A	A	D	K	E	S	P	A	N	E	A	S
A	T	V	A	G	S	A	T	Q	A	A	D	K	E	S	P	A	N	E	A	S
A	T	V	A	G	S	A	T	Q	A	A	D	K	E	S	P	A	N	E	A	S
A	T	V	A	G	S	A	T	Q	A	A	D	K	E	S	P	A	N	E	A	S
A	T	V	A	G	S	A	T	Q	A	A	D	K	E	S	P	A	N	E	A	S
A	T	V	A	G	S	A	T	Q	A	A	D	K	E	S	P	A	N	E	A	S
A	T	V	A	G	S	A	T	Q	A	A	D	K	E	S	P	A	N	E	A	S
A	T	V	A	G	S	A	T	Q	A	A	D	K	E	S	P	A	N	E	A	S
A	T	V	V	G	S	A	T	Q	A	A	D	K	E	S	P	A	N	E	A	S
A	T	V	A	G	S	T	T	Q	A	A	D	K	E	S	P	A	N	E	A	S
A	T	V	A	G	S	A	T	Q	A	A	D	K	E	S	P	A	N	E	A	S
A	T	V	A	G	S	A	T	Q	A	A	D	K	E	S	P	A	N	E	A	S
A	T	V	A	G	S	A	T	Q	A	A	D	K	E	S	P	A	N	E	A	S
A	T	V	A	G	S	A	T	Q	A	A	D	K	E	S	P	A	N	E	A	S
A	T	V	A	G	S	A	T	Q	A	A	D	K	E	S	P	A	N	E	A	S
A	T	V	A	G	S	A	T	Q	A	A	D	K	E	S	P	A	N	E	A	S
A	T	V	A	G	S	A	T	Q	A	A	D	K	E	S	P	A	N	E	A	S
A	T	V	A	G	S	A	T	Q	A	A	D	K	E	S	P	A	N	E	A	S
A	T	V	A	G	S	A	T	Q	A	A	D	K	E	S	P	A	N	E	A	S
A	T	V	A	G	S	A	T	Q	A	A	D	K	E	S	P	A	N	E	A	S
A	T	V	A	G	S	A	T	Q	A	A	D	K	E	S	P	A	N	E	A	S
A	T	V	A	G	S	A	T	Q	A	A	D	K	E	S	P	A	N	E	A	S
A	T	V	A	G	S	A	T	Q	A	A	D	K	E	S	P	A	N	E	A	S
A	T	V	A	G	S	A	T	Q	A	A	D	K	E	S	P	A	N	E	A	S
A	T	V	A	G	S	A	T	Q	A	A	D	K	E	S	P	A	N	E	A	S
A	T	V	A	G	S	A	T	Q	A	A	D	K	E	S	P	A	N	E	A	S
A	T	V	A	G	S	A	T	Q	A	A	D	K	E	S	P	A	N	E	A	S
A	T	V	A	G	S	A	T	Q	A	A	D	K	E	S	P	A	N	E	A	S
A	T	V	A	G	S	A	T	Q	A	A	D	K	E	S	P	A	N	E	A	S
A	T	V	A	G	S	A	T	Q	A	A	D	K	E	S	P	A	N	E	A	S
A	T	V	A	G	S	A	T	Q	A	A	D	K	E	S	P	A	N	E	A	S
A	T	V	A	G	S	A	T	Q	A	A	D	K	E	S	P	A	N	E	A	S
A	T	V	V	G	S	A	T	Q	A	A	D	K	E	S	P	A	N	E	A	S
A	T	V	A	G	S	A	T	Q	A	A	D	K	E	S	P	A	N	E	A	S
A	T	V	V	G	S	A	T	Q	A	A	D	K	E	S	P	A	N	E	A	S
A	T	V	A	G	S	A	T	Q	A	A	D	K	E	S	P	A	N	E	A	S
A	T	V	V	G	S	A	T	Q	A	A	D	K	E	S	P	A	N	E	A	S
A	T	V	A	G	S	A	T	Q	A	A	D	K	E	S	P	A	N	E	A	S
A	T	V	A	G	S	A	T	Q	A	A	D	K	E	S	P	A	N	E	A	S
A	T	V	V	G	S	A	T	Q	A	A	D	K	E	S	P	A	N	E	A	S
A	T	V	A	G	S	A	T	Q	A	A	D	K	E	S	P	A	N	E	A	S
A	T	V	A	G	S	A	T	Q	A	A	D	K	E	S	P	A	N	E	A	S
A	T	V	A	G	S	A	T	Q	A	A	D	K	E	S	P	A	N	E	A	S
V	T	V	A	G	S	A	T	Q	A	A	D	K	E	S	P	A	N	E	A	S
A	T	V	A	G	S	A	T	Q	A	A	D	K	E	S	P	A	N	E	A	S
T	T	V	A	G	S	A	T	Q	A	A	D	K	E	S	P	A	N	E	A	S
A	T	V	A	G	S	A	T	Q	A	A	D	K	E	S	P	A	N	E	A	S
A	T	V	A	G	S	A	T	Q	A	A	D	K	E	S	P	A	N	E	A	S

A	T	V	A	G	S	A	T	Q	A	A	D	K	E	S	P	A	N	E	A	S
A	T	V	A	G	S	A	T	Q	A	A	D	K	E	S	P	A	N	E	A	S
A	T	V	A	G	S	A	T	Q	A	A	D	K	E	S	P	A	N	E	A	S
T	T	V	A	G	S	A	T	Q	A	A	D	K	E	S	P	A	N	E	A	S
A	T	V	A	G	S	A	M	Q	A	A	D	K	E	S	P	A	N	E	A	S
A	T	V	A	G	S	A	T	Q	A	A	D	K	V	S	P	A	N	E	A	F
A	T	V	A	G	S	A	T	Q	A	A	D	K	E	S	P	A	N	E	A	S
A	T	V	A	G	S	A	T	Q	A	A	D	K	E	S	P	A	N	E	A	S
A	T	V	A	G	S	A	T	Q	A	A	D	K	E	S	P	A	N	E	A	S
A	T	V	A	G	S	A	T	Q	A	A	D	K	E	S	P	A	N	E	A	S
A	T	V	A	G	S	A	T	Q	A	A	D	K	E	S	P	A	N	E	A	S
A	T	V	A	G	S	A	T	Q	A	A	D	K	E	S	P	A	N	E	A	S
A	T	V	A	G	S	A	T	Q	A	A	D	K	E	S	P	A	N	E	A	S
A	T	V	A	G	S	A	T	Q	A	A	D	K	E	S	P	A	N	E	A	S
A	T	V	A	G	S	A	T	Q	A	A	D	K	E	S	P	A	N	E	A	S
A	T	V	A	G	S	A	T	Q	A	A	D	K	E	S	P	A	N	E	A	S
A	T	V	A	G	S	A	T	Q	A	A	D	K	E	S	P	A	N	E	A	S
V	T	V	A	G	S	A	T	Q	A	A	D	K	E	S	P	A	N	E	A	S
A	T	V	A	G	S	A	T	Q	A	A	D	K	E	S	P	A	N	E	A	S
A	T	V	A	G	S	A	T	Q	A	A	D	K	E	S	P	A	N	E	A	S
A	T	V	A	G	S	A	T	Q	A	A	D	K	E	S	P	A	N	E	A	S
A	T	V	A	G	S	A	T	Q	A	A	D	K	E	S	P	A	N	E	A	S
A	T	V	A	G	S	A	T	Q	A	A	D	K	E	S	P	A	N	E	A	S
A	T	V	A	G	S	A	T	Q	A	A	D	K	E	S	P	A	N	E	A	S
A	T	V	A	G	S	A	T	Q	A	A	D	K	E	S	P	A	N	E	A	S
A	T	V	A	G	S	A	T	Q	A	A	D	K	E	S	P	A	N	E	A	S
A	T	V	A	G	S	A	T	Q	A	A	D	K	E	S	P	A	N	E	A	S
A	T	V	A	G	S	A	T	Q	A	A	D	K	E	S	P	A	N	E	A	S
A	T	V	A	G	S	A	T	Q	A	A	D	K	E	S	P	A	N	E	A	S
A	T	V	A	G	S	A	T	Q	A	A	D	K	E	S	P	A	N	E	A	S
A	T	V	A	G	S	A	T	Q	A	A	D	K	E	S	P	A	N	E	A	S
A	T	V	A	G	S	A	T	Q	A	A	D	K	E	S	P	A	N	E	A	S
A	T	V	A	G	S	A	T	Q	A	A	D	K	E	S	P	A	N	E	A	S
A	T	V	A	G	S	A	T	Q	A	A	D	K	E	S	P	A	N	E	A	S
A	T	V	A	G	S	A	T	Q	A	A	D	K	E	S	P	A	N	E	A	S
A	T	V	A	G	S	A	T	Q	A	A	D	K	E	S	P	A	N	E	A	S
A	I	V	A	G	S	A	T	Q	A	A	D	K	E	S	P	A	N	E	A	S
A	T	V	A	G	S	A	T	Q	A	A	D	K	E	S	P	A	N	E	A	S
A	T	V	A	G	S	A	T	Q	A	A	D	K	E	S	P	A	N	E	A	S
A	T	V	A	G	S	A	T	Q	A	A	D	K	E	S	P	A	N	E	A	S
A	T	V	A	G	S	A	T	Q	A	A	D	K	E	S	P	A	N	E	A	S
A	T	V	A	G	S	A	T	Q	A	A	D	K	E	S	P	A	N	E	A	S
A	T	V	A	G	S	A	T	Q	A	A	D	K	E	S	P	A	N	E	A	S
A	T	V	A	G	S	A	T	Q	T	A	D	K	E	S	P	A	N	E	A	S
A	T	V	A	G	S	A	T	Q	A	A	D	K	E	S	P	A	N	E	A	S
A	T	V	A	G	S	A	T	Q	A	A	D	K	E	S	P	A	N	E	A	S
A	T	V	A	G	S	A	T	Q	A	A	D	K	E	S	P	A	N	E	A	S
A	T	V	A	G	S	A	T	Q	A	A	D	K	E	S	P	A	N	E	A	S
A	T	V	A	G	S	A	T	Q	A	A	D	K	E	S	P	A	N	E	A	S
A	T	V	A	G	S	A	T	Q	A	A	D	K	E	S	P	A	N	E	A	S
A	T	V	A	G	S	A	T	Q	A	A	D	K	E	S	P	A	N	E	A	S
A	T	V	A	G	S	A	T	Q	A	A	D	K	E	S	P	A	N	E	A	S
A	T	V	A	G	S	A	T	Q	A	A	D	K	E	S	P	A	N	E	A	S
A	T	V	A	G	S	A	T	Q	A	A	D	K	E	S	P	A	N	E	A	S
A	T	V	A	G	S	A	T	Q	A	A	D	K	E	S	P	A	N	E	A	S
A	T	V	A	R	S	A	T	Q	A	A	D	K	E	S	P	A	N	E	A	S
A	T	V	A	G	S	A	T	Q	A	A	D	K	E	S	P	A	N	E	A	S
A	T	V	A	G	S	A	T	Q	A	A	D	K	E	S	P	A	N	E	A	S
A	T	V	A	G	S	A	T	Q	A	V	D	K	E	S	P	A	N	E	A	S
A	T	V	A	G	S	A	T	Q	A	A	D	K	E	S	P	A	N	E	A	S
A	T	V	A	G	S	A	T	Q	A	A	D	K	E	S	P	A	N	E	A	S
A	T	V	A	G	L	A	T	Q	A	A	D	K	E	S	P	A	N	E	A	S
A	T	V	A	G	S	A	T	Q	A	A	D	K	E	S	P	A	N	E	A	S
A	T	V	A	G	S	A	T	H	A	A	E	K	E	S	P	V	N	E	A	S
A	T	V	A	G	S	A	T	Q	A	A	D	K	E	S	P	A	N	E	A	S
A	T	V	A	G	S	A	T	Q	A	A	D	K	E	S	P	A	N	E	A	S

A	T	V	A	G
A	T	V	A	G
A	T	V	A	G
A	T	V	A	G
A	T	V	A	G
A	T	V	A	G
A	T	V	A	G
A	T	V	A	G
A	T	V	A	G
A	T	V	A	G
A	T	V	A	G
A	T	V	A	G
A	T	V	A	G
A	T	V	A	G
A	T	V	A	G
A	T	V	A	G
A	T	V	A	G
A	T	V	A	G
A	T	V	A	G
A	T	V	A	G
A	T	V	V	G
A	T	V	A	G
A	T	V	A	G
A	T	V	A	G
A	T	V	A	G
A	T	I	A	G
A	T	V	A	G
A	T	V	A	G
A	T	V	A	G
A	T	V	A	G
A	T	V	A	G
A	T	V	A	G
A	T	V	A	G
A	T	V	A	G
A	T	V	A	G
A	T	V	A	G
A	T	V	A	G
A	T	V	A	G

S	A	T	Q	A	A	D	K	E	S
S	A	T	Q	A	A	D	E	E	S
S	A	T	Q	A	A	D	E	E	S
S	A	T	Q	A	A	D	K	E	S
S	A	T	Q	A	A	D	K	E	S
S	A	T	Q	A	A	D	K	E	S
S	A	T	Q	A	A	D	K	E	S
S	A	T	Q	A	A	D	K	E	S
S	A	T	Q	A	A	D	K	E	S
S	A	T	Q	A	A	D	K	E	S
S	A	T	Q	A	A	D	K	E	S
S	A	T	Q	A	A	D	K	E	S
S	A	T	Q	A	A	D	K	E	S
S	A	T	Q	A	A	D	K	E	S
S	A	T	Q	A	A	D	K	E	S
S	A	T	Q	A	A	D	K	E	S
S	A	T	Q	A	A	D	K	E	S
S	A	T	Q	A	A	D	K	E	S
S	A	T	Q	A	A	D	K	E	S
S	A	T	Q	A	A	D	K	E	S
S	A	T	Q	A	A	D	K	E	S
S	A	T	Q	A	A	D	K	E	S
S	A	T	Q	A	A	D	K	E	S
S	A	T	Q	A	A	D	K	E	S
S	A	T	Q	A	A	D	K	E	S
S	A	T	Q	A	A	D	K	E	S
S	A	T	Q	A	A	D	K	E	S
S	A	T	Q	A	A	D	K	E	S
S	A	T	Q	A	A	D	K	E	S
S	A	T	Q	A	A	D	K	E	S
S	A	T	Q	A	A	D	K	E	S
S	A	T	Q	A	A	D	K	E	S
S	A	T	Q	A	A	E	K	E	F
S	A	T	Q	A	A	D	K	E	S
S	A	T	Q	A	A	D	K	E	S
S	A	T	Q	A	A	D	K	E	S
S	A	T	Q	A	A	D	K	E	S
S	A	T	Q	A	A	D	K	E	S

P	A	N	E	A	S
P	A	N	E	A	S
P	A	N	E	A	S
P	A	N	E	A	S
P	A	N	E	A	S
P	A	N	E	A	S
P	A	N	E	A	S
P	A	N	E	A	S
P	A	N	E	A	S
P	A	N	E	A	S
P	A	N	E	A	S
P	A	N	E	A	S
S	A	N	E	V	S
P	A	N	E	A	S
P	A	N	E	A	S
P	A	N	E	A	S
P	A	N	E	A	S
P	A	N	E	A	S
P	A	N	E	A	S
P	A	N	E	A	S
P	A	N	E	A	S
P	A	N	E	A	S
P	A	N	E	A	S
P	A	N	E	A	S
P	A	N	E	A	S
P	A	N	E	A	S
P	A	N	E	A	S
P	A	N	E	A	S
P	A	N	E	A	S
P	A	N	E	A	S
P	A	N	E	A	S
P	A	N	E	A	S
P	A	N	E	A	S
P	A	N	E	A	S
P	A	N	E	A	S
P	A	N	E	A	S
P	A	N	E	A	S
P	A	D	D	A	S

478
L
M
L

wzh										
27	34	94	101	123	170	190	203	205	218	235
T	V	N	N	I	S	R	V	S	S	R
A	M	K	D	S	P	Q	L	G	T	Q
T	V	N	N	I	S	R	V	S	S	R

14	15	21	24	27	41	60	76	87
S	L	Y	I	L	T	N	T	L
L	F	R	M	I	A	D	S	F
S	L	Y	I	L	T	N	T	L

wzd				
29	51	91	135	224
A	T	V	A	G
A	T	V	A	G
A	T	V	A	G
A	T	V	A	G
A	T	V	A	G
A	T	V	A	G
A	T	V	A	G
A	T	V	A	G
A	T	V	A	G
A	T	V	A	G
A	T	V	V	G
A	T	V	A	G
A	T	V	A	G

wze									
7	23	51	118	119	163	185	207	213	217
S	A	T	Q	A	A	D	K	E	S
S	A	T	Q	A	A	D	K	E	S
S	A	T	Q	A	A	D	K	E	S
S	A	T	Q	A	A	D	K	E	S
S	A	T	Q	A	A	D	K	E	S
S	A	T	Q	A	A	D	K	E	S
S	A	T	Q	A	A	D	K	E	S
S	A	T	Q	A	A	D	K	E	S
S	A	T	Q	A	A	D	K	E	S
S	A	T	Q	A	A	D	K	E	S
S	A	T	Q	A	A	D	K	E	S
S	A	T	Q	A	A	D	K	E	S
S	A	T	Q	A	A	D	K	E	S

					wchA
8	78	188	212	244	257
P	A	A	S	V	M
P	A	A	S	V	M
P	A	A	S	V	M
P	A	A	S	V	M
P	A	A	S	V	V
P	A	A	S	V	V
P	A	A	S	V	V
P	A	A	S	V	V
P	A	A	S	V	V
P	A	A	S	V	V
P	A	A	S	V	V
P	A	A	S	V	V
P	A	A	S	V	V

A	T	V	A	G	S	A	T	Q	A	A	D	K	E	S	P	A	A	S	V	V
A	T	V	A	G	S	A	M	Q	A	A	D	K	E	S	P	A	A	S	V	V
A	T	V	V	G	S	A	T	Q	A	A	D	K	E	S	P	A	A	S	V	V
A	T	V	A	G	S	A	T	Q	A	A	D	K	E	S	P	A	A	S	V	V
A	T	V	A	G	S	A	T	Q	A	A	D	K	E	S	P	A	A	S	V	V
A	T	V	A	G	S	A	T	Q	A	A	D	K	E	S	P	A	A	S	V	V
A	T	V	A	G	S	A	T	Q	A	A	D	K	E	S	P	A	A	S	V	V
A	T	V	A	G	S	A	T	Q	A	A	D	K	E	S	P	A	A	S	V	V
A	T	V	A	G	S	A	T	Q	A	A	D	K	E	S	P	A	A	S	V	V
A	T	V	A	G	S	A	T	Q	A	A	D	K	E	S	P	A	A	S	V	V
A	T	V	A	G	S	A	T	Q	A	A	D	K	E	S	P	A	A	S	V	V
A	T	V	A	G	S	A	T	Q	A	A	D	K	E	S	P	A	A	S	V	V
A	T	V	V	G	S	A	T	Q	A	A	D	K	E	S	P	A	A	S	V	V
A	T	V	A	G	S	T	T	Q	A	A	D	K	E	S	P	A	A	S	V	V
A	T	V	A	G	S	A	T	Q	A	A	D	K	E	S	P	A	A	S	V	V
A	T	V	A	G	S	A	T	Q	A	A	D	K	E	S	P	A	A	S	V	V
A	T	V	A	G	S	A	T	Q	A	A	D	K	E	S	P	A	A	S	V	V
A	T	V	A	G	S	A	T	Q	A	A	D	K	E	S	P	A	A	S	V	V
A	T	V	A	G	S	A	T	Q	A	A	D	K	E	S	P	A	A	S	V	V
A	T	V	A	G	S	A	T	Q	A	A	D	K	E	S	P	A	A	S	V	V
A	T	V	A	G	S	A	T	Q	A	A	D	K	E	S	P	A	A	S	V	V
A	T	V	A	G	S	A	T	Q	A	A	D	K	E	S	P	A	A	S	V	V
A	T	V	A	G	S	A	T	Q	A	A	D	K	E	S	P	A	A	S	V	V
A	T	V	A	G	S	A	T	Q	A	A	D	K	E	S	P	A	A	S	V	V
A	T	V	A	G	S	A	T	Q	A	A	D	K	E	S	P	A	A	S	V	V
A	T	V	A	G	S	A	T	Q	A	A	D	K	E	S	P	A	A	S	V	V
A	T	V	A	G	S	A	T	Q	A	A	D	K	E	S	P	A	A	S	V	V
A	T	V	A	G	S	A	T	Q	A	A	D	K	E	S	P	A	A	S	V	V
A	T	V	A	G	S	A	T	Q	A	A	D	K	E	S	P	A	A	S	V	V
A	T	V	A	G	S	A	T	Q	A	A	D	K	E	S	P	A	A	S	V	V
A	T	V	A	G	S	A	T	Q	A	A	D	K	E	S	P	A	A	S	V	V
A	T	V	A	G	S	A	T	Q	A	A	D	K	E	S	P	A	A	S	V	V
A	T	V	A	G	S	A	T	Q	A	A	D	K	E	S	P	A	A	S	V	V
A	T	V	A	G	S	A	T	Q	A	A	D	K	E	S	P	A	A	S	V	V
A	T	V	A	G	S	A	T	Q	A	A	D	K	E	S	P	A	A	S	V	V
A	T	V	A	G	S	A	T	Q	A	A	D	K	E	S	P	A	A	S	V	V
A	T	V	V	G	S	A	T	Q	A	A	D	K	E	S	P	A	A	S	V	V
A	T	V	A	G	S	A	T	Q	A	A	D	K	E	S	P	A	A	S	V	V
A	T	V	V	G	S	A	T	Q	A	A	D	K	E	S	P	A	A	S	V	V
A	T	V	A	G	S	A	T	Q	A	A	D	K	E	S	P	A	A	S	V	V
A	T	V	V	G	S	A	T	Q	A	A	D	K	E	S	P	A	A	S	V	V
A	T	V	A	G	S	A	T	Q	A	A	D	K	E	S	P	A	A	S	V	V
A	T	V	A	G	S	A	T	Q	A	A	D	K	E	S	P	A	A	S	V	V
A	T	V	V	G	S	A	T	Q	A	A	D	K	E	S	P	A	A	S	V	V
A	T	V	A	G	S	A	T	Q	A	A	D	K	E	S	P	A	A	S	V	V
A	T	V	A	G	S	A	T	Q	A	A	D	K	E	S	P	A	A	S	V	V
A	T	V	A	G	S	A	T	Q	A	A	D	K	E	S	P	A	A	S	V	V
V	T	V	A	G	S	A	T	Q	A	A	D	K	E	S	P	A	A	S	V	V
A	T	V	A	G	S	A	T	Q	A	A	D	K	E	S	P	A	A	S	V	V
T	T	V	A	G	S	A	T	Q	A	A	D	K	E	S	P	A	A	S	V	V
A	T	V	A	G	S	A	T	Q	A	A	D	K	E	S	P	A	A	S	V	V
A	T	V	A	G	S	A	T	Q	A	A	D	K	E	S	P	A	A	S	V	V
A	T	V	A	G	S	A	T	Q	A	A	D	K	E	S	P	A	A	S	V	V
A	T	V	A	G	S	A	T	Q	A	A	D	K	E	S	P	A	A	S	V	V
A	T	V	A	G	S	A	T	Q	A	A	D	K	E	S	P	A	A	S	V	V
T	T	V	A	G	S	A	T	Q	A	A	D	K	E	S	P	A	A	S	V	V
A	T	V	A	G	S	A	M	Q	A	A	D	K	E	S	P	A	A	S	V	V
A	T	V	A	G	S	A	T	Q	A	A	D	K	V	S	P	A	A	F	V	V
A	T	V	A	G	S	A	T	Q	A	A	D	K	E	S	P	A	A	S	V	V
A	T	V	A	G	S	A	T	Q	A	A	D	K	E	S	P	A	A	S	V	V
A	T	V	A	G	S	A	T	Q	A	A	D	K	E	S	P	A	A	S	V	V
A	T	V	A	G	S	A	T	Q	A	A	D	K	E	S	P	A	A	S	V	V
A	T	V	A	G	S	A	T	Q	A	A	D	K	E	S	P	A	A	S	V	V
A	T	V	A	G	S	A	T	Q	A	A	D	K	E	S	P	A	A	S	V	V
A	T	V	A	G	S	A	T	Q	A	A	D	K	E	S	P	A	A	S	V	V
A	T	V	A	G	S	A	T	Q	A	A	D	K	E	S	P	A	A	S	V	V
A	T	V	A	G	S	A	T	Q	A	A	D	K	E	S	P	A	A	S	V	V

A	T	V	A	G	S	A	T	Q	A	A	D	K	E	S	P	A	A	S	V	V
A	T	V	A	G	S	A	T	Q	A	A	D	K	E	S	P	A	A	S	V	V
V	T	V	A	G	S	A	T	Q	A	A	D	K	E	S	P	A	A	S	V	V
A	T	V	A	G	S	A	T	Q	A	A	D	K	E	S	P	A	A	S	V	V
A	T	V	A	G	S	A	T	Q	A	A	D	K	E	S	P	A	A	S	V	V
A	T	V	A	G	S	A	T	Q	A	A	D	K	E	S	P	A	A	S	V	V
A	T	V	A	G	S	A	T	Q	A	A	D	K	E	S	P	A	A	S	V	V
A	T	V	A	G	S	A	T	Q	A	A	D	K	E	S	P	A	A	S	V	V
A	T	V	A	G	S	A	T	Q	A	A	D	K	E	S	P	A	A	S	V	V
A	T	V	A	G	S	A	T	Q	A	A	D	K	E	S	P	A	A	S	V	V
A	T	V	A	G	S	A	T	Q	A	A	D	K	E	S	P	A	A	S	V	V
A	T	V	A	G	S	A	T	Q	A	A	D	K	E	S	P	A	A	S	V	V
A	T	V	A	G	S	A	T	Q	A	A	D	K	E	S	P	A	A	S	V	V
A	T	V	A	G	S	A	T	Q	A	A	D	K	E	S	P	A	A	S	V	V
A	T	V	A	G	S	A	T	Q	A	A	D	K	E	S	P	A	A	S	V	V
A	T	V	A	G	S	A	T	Q	A	A	D	K	E	S	P	A	A	S	V	V
A	T	V	A	G	S	A	T	Q	A	A	D	K	E	S	P	A	A	S	V	V
A	T	V	A	G	S	A	T	Q	A	A	D	K	E	S	P	A	A	S	V	V
A	T	V	A	G	S	A	T	Q	A	A	D	K	E	S	P	A	A	S	V	V
A	T	V	A	G	S	A	T	Q	A	A	D	K	E	S	P	A	A	S	V	V
A	I	V	A	G	S	A	T	Q	A	A	D	K	E	S	P	A	A	S	V	V
A	T	V	A	G	S	A	T	Q	A	A	D	K	E	S	P	A	A	S	V	V
A	T	V	A	G	S	A	T	Q	A	A	D	K	E	S	P	A	A	S	V	V
A	T	V	A	G	S	A	T	Q	A	A	D	K	E	S	P	A	A	S	V	V
A	T	V	A	G	S	A	T	Q	A	A	D	K	E	S	P	A	A	S	V	V
A	T	V	A	G	S	A	T	Q	A	A	D	K	E	S	P	A	A	S	V	V
A	T	V	A	G	S	A	T	Q	A	A	D	K	E	S	P	A	A	S	V	V
A	T	V	A	G	S	A	T	Q	T	A	D	K	E	S	P	A	A	S	V	V
A	T	V	A	G	S	A	T	Q	A	A	D	K	E	S	P	A	A	S	V	V
A	T	V	A	G	S	A	T	Q	A	A	D	K	E	S	P	A	A	S	V	V
A	T	V	A	G	S	A	T	Q	A	A	D	K	E	S	P	A	A	S	V	V
A	T	V	A	G	S	A	T	Q	A	A	D	K	E	S	P	A	A	S	V	V
A	T	V	A	G	S	A	T	Q	A	A	D	K	E	S	P	A	A	S	V	V
A	T	V	A	G	S	A	T	Q	A	A	D	K	E	S	P	A	A	S	V	V
A	T	V	A	G	S	A	T	Q	A	A	D	K	E	S	P	A	A	S	V	V
A	T	V	A	G	S	A	T	Q	A	A	D	K	E	S	P	A	A	S	V	V
A	T	V	A	G	S	A	T	Q	A	A	D	K	E	S	P	A	A	S	V	V
A	T	V	A	G	S	A	T	Q	A	A	D	K	E	S	P	A	A	S	V	V
A	T	V	A	G	S	A	T	Q	A	A	D	K	E	S	P	A	A	S	V	V
A	T	V	A	R	S	A	T	Q	A	A	D	K	E	S	P	A	A	S	V	V
A	T	V	A	G	S	A	T	Q	A	A	D	K	E	S	P	A	A	S	V	V
A	T	V	A	G	S	A	T	Q	A	A	D	K	E	S	P	A	A	S	V	V
A	T	V	A	G	S	A	T	Q	A	V	D	K	E	S	P	A	A	S	V	V
A	T	V	A	G	S	A	T	Q	A	A	D	K	E	S	P	A	A	S	V	V
A	T	V	A	G	S	A	T	Q	A	A	D	K	E	S	P	A	A	S	V	V
A	T	V	A	G	L	A	T	Q	A	A	D	K	E	S	P	A	A	S	V	V
A	T	V	A	G	S	A	T	Q	A	A	D	K	E	S	P	A	A	S	V	V
A	T	V	A	G	S	A	T	H	A	A	E	K	E	S	P	V	A	S	V	V
A	T	V	A	G	S	A	T	Q	A	A	D	K	E	S	P	A	A	S	V	V
A	T	V	A	G	S	A	T	Q	A	A	D	K	E	S	P	A	A	S	V	V
A	T	V	A	G	S	A	T	Q	A	A	D	K	E	S	P	A	A	S	V	V
A	T	V	A	G	S	A	T	Q	A	A	D	E	E	S	P	A	A	S	V	V
A	T	V	A	G	S	A	T	Q	A	A	D	E	E	S	P	A	A	S	V	V
A	T	V	A	G	S	A	T	Q	A	A	D	K	E	S	P	A	A	S	V	V
A	T	V	A	G	S	A	T	Q	A	A	D	K	E	S	P	A	A	S	V	V
A	T	V	A	G	S	A	T	Q	A	A	D	K	E	S	P	A	A	S	V	V
A	T	V	A	G	S	A	T	Q	A	A	D	K	E	S	P	A	A	S	V	V
A	T	V	A	G	S	A	T	Q	A	A	D	K	E	S	P	A	A	S	V	V
A	T	V	A	G	S	A	T	Q	A	A	D	K	E	S	P	A	A	S	V	V
A	T	V	A	G	S	A	T	Q	A	A	D	K	E	S	P	A	A	S	V	V
A	T	V	A	G	S	A	T	Q	A	A	D	K	E	S	P	A	A	S	V	V
A	T	V	A	G	S	A	T	Q	A	A	D	K	E	S	P	A	A	S	V	V
A	T	V	A	G	S	A	T	Q	A	A	D	K	E	S	S	A	V	S	V	V
A	T	V	A	G	S	A	T	Q	A	A	D	K	E	S	P	A	A	S	V	V
A	T	V	A	G	S	A	T	Q	A	A	D	K	E	S	P	A	A	S	V	V
A	T	V	A	G	S	A	T	Q	A	A	D	K	E	S	P	A	A	S	V	V
A	T	V	A	G	S	A	T	Q	A	A	D	K	E	S	P	A	A	S	I	V

A	T	V	A	G
A	T	V	A	G
A	T	V	A	G
A	T	V	V	G
A	T	V	A	G
A	T	V	A	G
A	T	V	A	G
A	T	V	A	G
A	T	I	A	G
A	T	V	A	G
A	T	V	A	G
A	T	V	A	G
A	T	V	A	G
A	T	V	A	G
A	T	V	A	G
A	T	V	A	G
A	T	V	A	G
A	T	V	A	G
A	T	V	A	G
A	T	V	A	G

S	A	T	Q	A	A	D	K	E	S
S	A	T	Q	A	A	D	K	E	S
S	A	T	Q	A	A	D	K	E	S
S	A	T	Q	A	A	D	K	E	S
S	A	T	Q	A	A	D	K	E	S
S	A	T	Q	A	A	D	K	E	S
S	A	T	Q	A	A	D	K	E	S
S	A	T	Q	A	A	D	K	E	S
S	A	T	Q	A	A	D	K	E	S
S	A	T	Q	A	A	D	K	E	S
S	A	T	Q	A	A	D	K	E	S
S	A	T	Q	A	A	D	K	E	S
S	A	T	Q	A	A	D	K	E	S
S	A	T	Q	A	A	D	K	E	S
S	A	T	Q	A	A	D	K	E	S
S	A	T	Q	A	A	E	K	E	F
S	A	T	Q	A	A	D	K	E	S
S	A	T	Q	A	A	D	K	E	S
S	A	T	Q	A	A	D	K	E	S
S	A	T	Q	A	A	D	K	E	S

P	A	A	S	V	V
P	A	A	S	V	V
P	A	A	S	V	V
P	A	A	S	V	V
P	A	A	S	V	V
P	A	A	S	V	V
P	A	A	S	V	V
P	A	A	S	V	V
P	A	A	S	V	V
P	A	A	S	V	V
P	A	A	S	V	V
P	A	A	S	V	V
P	A	A	S	V	V
P	A	A	S	V	V
P	A	A	S	V	V
P	A	A	S	V	V
P	A	A	S	V	V
P	A	A	S	V	V
P	A	A	S	V	V
P	A	A	S	V	V

wchA							wchJ	wchK	wcyK	wcwC		wcrL		
244	257	261	310	347	354	452		60	72	16	196	153	159	160
V	M	R	Y	K	K	A		R	Y	P	N	T	L	G
V	V	W	Y	K	K	A		R	Y	P	D	T	L	G
V	V	W	Y	K	K	A		R	Y	P	D	T	L	G
V	V	W	Y	K	K	A		R	Y	P	D	T	L	G
V	V	W	Y	K	K	A		R	Y	P	D	T	L	G
V	V	W	Y	K	K	A		R	Y	P	D	T	L	G
V	V	W	Y	K	K	A		R	Y	P	D	T	L	G
V	V	W	Y	K	K	A		R	Y	P	D	T	L	G
V	V	W	Y	K	K	A		R	Y	P	D	T	L	G
V	V	W	Y	K	K	A		R	Y	P	D	T	L	G
V	V	W	Y	K	K	A		R	Y	P	D	T	L	G
V	V	W	Y	K	K	A		R	Y	P	D	T	L	G
V	V	W	Y	K	K	A		R	Y	P	D	T	L	G
V	M	R	Y	K	K	A		R	Y	P	N	T	L	G
V	V	W	Y	K	K	A		R	Y	P	D	T	L	G
V	V	W	Y	K	K	A		R	Y	P	D	T	L	G
V	V	W	Y	K	K	A		R	Y	P	D	T	L	G
V	V	W	Y	K	K	A		R	Y	P	D	T	L	G
V	V	W	Y	K	K	A		R	Y	P	D	T	L	G
V	V	W	Y	K	K	A		R	Y	P	D	T	L	G
V	V	W	Y	K	K	A		R	Y	P	D	T	L	G
V	V	W	Y	K	K	A		R	Y	P	D	T	L	G
V	V	W	Y	K	K	A		R	Y	P	D	T	L	G
V	V	W	Y	K	K	A		R	Y	P	D	T	L	G
V	V	W	Y	T	K	A		R	Y	P	D	T	L	G
V	V	W	Y	K	K	A		R	Y	P	D	T	L	G
V	V	W	Y	K	K	A		R	Y	P	D	T	L	G
V	V	W	Y	K	K	A		R	Y	P	D	T	L	G
V	V	W	Y	K	K	A		R	Y	P	D	T	L	G
V	V	W	Y	K	K	A		R	Y	P	D	T	L	G
V	V	W	Y	K	K	A		R	Y	P	D	T	L	G
V	V	W	Y	K	K	A		R	Y	P	D	T	L	G
V	V	W	Y	K	K	A		R	Y	P	D	T	L	G
V	V	W	Y	K	K	A		R	Y	P	D	T	L	G
V	V	W	Y	K	K	A		R	Y	P	D	T	L	G
V	V	W	Y	K	K	A		R	Y	P	D	T	L	G
V	V	W	Y	K	K	A		R	Y	P	D	T	L	G
V	V	W	Y	K	K	A		R	Y	P	D	T	L	G
V	V	W	Y	K	K	A		R	Y	P	D	T	L	G
V	V	W	Y	K	K	A		R	Y	P	D	T	L	G
V	V	W	Y	K	K	A		R	Y	P	D	T	L	G
V	V	W	Y	K	K	A		R	Y	P	D	T	L	G
V	V	W	Y	K	K	A		R	Y	P	D	T	L	G
V	V	W	Y	K	K	A		R	Y	P	D	T	L	G
V	V	W	Y	K	K	A		R	Y	P	D	T	L	G
V	V	W	Y	K	K	A		R	Y	P	D	T	L	G
V	V	W	Y	K	K	A		R	Y	P	D	T	L	G
V	V	W	Y	K	K	A		R	Y	P	D	T	L	G
V	V	W	Y	K	K	A		R	Y	P	D	T	L	G
V	V	W	Y	K	K	A		R	Y	P	D	T	L	G
V	V	W	Y	K	K	A		R	Y	P	D	T	L	G
V	V	W	Y	K	K	A		R	Y	P	D	T	L	G
V	V	W	Y	K	K	A		R	Y	P	D	T	L	G
V	V	W	Y	K	K	A		R	Y	P	D	T	L	G
V	V	W	Y	K	K	A		R	Y	P	D	T	L	G
V	V	W	Y	K	K	A		R	Y	P	D	T	L	G
V	V	W	Y	K	K	A		R	Y	P	D	T	L	G
V	V	W	Y	K	K	A		R	Y	P	D	T	L	G
V	V	W	Y	K	K	A		R	Y	P	D	T	L	G
V	V	W	Y	K	K	A		R	Y	P	D	T	L	G
V	V	W	Y	K	K	A		R	Y	P	D	T	L	G
V	V	W	Y	K	K	A		R	Y	P	D	T	L	G
V	V	W	Y	K	K	A		R	Y	P	D	T	L	G

V	V	W	Y	K	K	A		R	Y	P	D	T	L	G
V	V	W	Y	K	K	A		R	Y	P	D	T	L	G
V	V	W	Y	K	K	A		R	Y	P	D	T	L	G
V	V	W	Y	K	K	A		R	Y	P	D	T	L	G
V	V	W	Y	K	K	A		R	Y	P	D	T	L	G
V	V	W	Y	K	K	A		R	Y	P	D	T	L	G
V	V	W	Y	K	K	A		R	Y	P	D	T	L	G
V	V	W	Y	K	K	A		R	Y	P	D	T	L	G
V	V	W	Y	K	K	A		R	Y	P	D	T	L	G
V	V	W	Y	K	K	A		R	Y	P	D	T	L	D
V	V	W	Y	K	K	A		R	Y	P	D	T	L	D
V	V	W	Y	K	K	A		R	Y	P	D	T	L	D
V	V	W	Y	K	K	A		R	Y	P	D	T	L	G
V	V	W	Y	K	K	A		R	Y	P	D	T	L	G
V	V	W	Y	K	K	A		R	Y	P	D	T	L	G
V	V	W	Y	K	K	A		R	Y	P	D	T	L	G
V	V	W	Y	K	K	S		R	Y	P	D	T	L	G
V	V	W	Y	K	K	A		R	Y	P	D	T	L	G
V	M	R	Y	K	K	A		R	Y	P	N	T	L	G
V	V	W	Y	K	K	A		R	Y	P	D	T	L	G
V	V	W	Y	K	K	A		R	Y	P	D	T	L	G
V	V	W	Y	K	K	A		R	Y	P	D	T	L	G
V	V	W	Y	K	K	A		R	Y	P	D	T	L	G
V	V	W	Y	K	K	A		R	Y	P	D	T	L	G
V	V	W	Y	K	K	A		R	Y	P	D	T	L	G
V	V	W	Y	K	N	A		R	Y	P	D	T	L	G
V	V	W	Y	K	K	A		R	Y	P	D	T	L	G
V	V	W	Y	K	K	A		R	Y	P	D	T	L	G
V	V	W	Y	K	K	A		R	Y	P	D	T	L	G
V	V	W	Y	K	K	A		R	Y	P	D	T	L	G
V	V	W	Y	K	K	A		R	Y	P	D	T	L	G
V	V	W	Y	K	K	A		R	Y	P	D	T	L	G
V	V	W	Y	K	K	A		R	Y	P	D	T	L	G
V	V	W	Y	K	K	A		R	Y	P	D	T	L	G
V	V	W	Y	K	K	A		R	Y	P	D	T	L	G
V	V	W	Y	K	K	A		R	Y	P	D	T	L	G
V	V	W	Y	K	K	A		R	Y	P	D	T	L	G
V	V	W	Y	K	K	A		R	Y	P	D	T	L	G
V	V	W	Y	K	K	A		R	Y	P	D	T	L	G
V	V	W	Y	K	K	A		R	Y	P	D	T	L	G
V	V	W	Y	K	K	A		R	Y	P	D	T	L	G
V	V	W	Y	K	K	A		R	Y	P	D	T	L	G
V	V	W	Y	K	K	A		R	Y	P	D	T	L	G
V	V	W	Y	K	K	A		R	Y	P	D	T	L	G
V	V	W	Y	K	K	A		R	Y	P	D	T	L	G
V	V	W	C	K	K	A		R	Y	P	D	T	L	G
V	V	W	Y	K	K	A		R	Y	P	D	T	L	G
V	M	R	Y	K	K	A		R	Y	S	N	T	L	G
V	V	W	Y	K	K	A		R	Y	P	D	T	L	G
V	V	W	Y	K	K	A		R	Y	P	D	T	L	G
V	V	W	Y	K	K	A		R	Y	P	D	T	L	G
V	V	W	Y	K	K	A		R	Y	P	D	T	L	G
V	V	W	Y	K	K	A		R	Y	P	D	T	L	G
V	V	W	Y	K	K	A		R	Y	P	D	T	L	G
V	V	W	Y	K	K	A		R	Y	P	D	T	L	G
V	V	W	Y	K	K	A		R	Y	P	D	T	L	G
V	V	W	Y	K	K	A		R	Y	P	D	T	L	G
V	V	W	Y	K	K	A		R	Y	P	D	T	L	G
V	V	W	Y	K	K	A		R	Y	P	D	I	F	G
V	V	W	Y	K	K	A		R	Y	P	D	T	L	G
V	V	W	Y	K	K	A		R	Y	P	D	T	L	G
V	V	W	Y	K	K	A		R	Y	P	D	T	L	G
V	V	W	Y	K	K	A		R	Y	P	D	T	L	G
V	V	W	Y	K	K	A		R	Y	P	D	T	L	G
V	V	W	Y	K	K	A		R	Y	P	D	T	L	G
V	V	W	Y	K	K	A		R	Y	P	D	T	L	G
V	V	W	Y	K	K	A		R	Y	P	D	T	L	G

V	V	W	Y	K	K	A		R	Y	P	D	T	L	G
V	V	W	Y	K	K	A		R	Y	P	D	T	L	G
V	V	W	Y	K	K	A		R	Y	P	D	T	L	G
V	V	W	Y	K	K	A		C	Y	P	D	T	L	G
V	V	W	Y	K	K	A		R	Y	P	D	T	L	G
V	V	W	Y	K	K	A		R	Y	P	D	T	L	G
V	V	W	Y	K	K	A		R	Y	P	D	T	L	G
V	V	W	Y	K	K	A		R	Y	P	D	T	L	G
V	V	W	Y	K	K	A		R	Y	P	D	T	L	G
V	V	W	Y	K	K	A		R	Y	P	D	T	L	G
V	V	W	Y	K	K	A		R	Y	P	D	T	L	G
V	V	W	Y	K	K	A		R	Y	P	D	T	L	G
V	V	W	Y	K	K	A		R	Y	P	D	T	L	G
V	V	W	Y	K	K	A		R	Y	P	D	T	L	G
V	V	W	Y	K	K	A		R	Y	P	D	T	L	G
V	V	W	Y	K	K	A		R	Y	P	D	T	L	G
I	V	W	Y	K	K	A		R	Y	P	D	T	L	G
V	V	W	Y	K	K	A		R	Y	P	D	T	L	G
V	V	W	Y	K	K	A		R	Y	P	D	T	L	G
V	V	W	Y	K	K	A		R	Y	P	D	T	L	G
V	V	W	Y	K	K	A		R	Y	P	D	T	L	G
V	V	W	Y	K	K	A		R	Y	P	D	T	L	G
V	V	W	Y	K	K	A		R	Y	P	D	T	L	G
V	V	W	Y	K	K	A		R	Y	P	D	T	L	G
V	V	W	Y	K	K	A		R	Y	P	D	T	L	G
V	V	W	Y	K	K	A		R	Y	P	D	T	L	G
V	V	W	Y	K	K	A		R	Y	P	D	T	L	G
V	V	W	Y	K	K	A		R	Y	P	D	T	L	G
V	V	W	Y	K	K	A		R	Y	P	D	T	L	G
V	V	W	Y	K	K	A		R	Y	P	D	T	L	G
V	V	W	Y	K	K	A		R	Y	P	D	T	L	G
V	V	W	Y	K	K	A		R	Y	P	D	T	L	G
V	V	W	Y	K	K	A		R	Y	P	D	T	L	G
V	V	W	Y	K	K	A		R	Y	P	D	T	L	G
V	V	W	Y	K	K	A		R	Y	P	D	T	L	G
V	V	W	Y	K	K	A		R	Y	P	D	T	L	G
V	V	W	Y	K	K	A		R	Y	P	D	T	L	G
V	V	R	Y	K	K	A		R	C	P	N	T	L	G

wzd										
103	109	151	180	182	189	193	194	217	225	226
I	T	I	T	V	A	I	T	M	D	F
M	A	V	S	F	V	S	A	I	N	L
I	T	I	T	V	A	I	T	M	D	F

9	12	13	14	45	50	62	72	74
V	E	L	A	A	T	R	A	M
A	D	F	V	L	S	H	G	I
V	E	L	A	A	T	R	A	M

					wchJ	wchK	wcyK	wcwC		wcrL			wzy
261	310	347	354	452		60		16	196	153	159	160	303
R	Y	K	K	A		R		P	N	T	L	G	I
R	Y	K	K	A		R		P	N	T	L	G	I
R	Y	K	K	A		R		P	N	T	L	G	I
R	Y	K	K	A		R		S	N	T	L	G	I
W	Y	K	K	A		R		P	D	T	L	G	I
W	Y	K	K	A		R		P	D	T	L	G	I
W	Y	K	K	A		R		P	D	T	L	G	I
W	Y	K	K	A		R		P	D	T	L	G	I
W	Y	K	K	A		R		P	D	T	L	G	I
W	Y	K	K	A		R		P	D	T	L	G	I
W	Y	K	K	A		R		P	D	T	L	G	I
W	Y	K	K	A		R		P	D	T	L	G	I
W	Y	K	K	A		R		P	D	T	L	G	I

W	Y	K	K	A		R		P	D	T	L	G	I
W	Y	K	K	A		R		P	D	T	L	G	I
W	Y	K	K	A		R		P	D	T	L	G	I
W	Y	K	K	A		R		P	D	T	L	G	I
W	Y	K	K	A		R		P	D	T	L	G	I
W	Y	K	K	A		R		P	D	T	L	G	I
W	Y	K	K	A		R		P	D	T	L	G	I
W	Y	K	K	A		R		P	D	T	L	G	I
W	Y	K	K	A		R		P	D	T	L	G	I
W	Y	K	K	A		R		P	D	T	L	G	I
W	Y	K	K	A		R		P	D	T	L	G	I
W	Y	K	K	A		R		P	D	T	L	G	I
W	Y	K	K	A		R		P	D	T	L	G	I
W	Y	T	K	A		R		P	D	T	L	G	I
W	Y	K	K	A		R		P	D	T	L	G	I
W	Y	K	K	A		R		P	D	T	L	G	I
W	Y	K	K	A		R		P	D	T	L	G	I
W	Y	K	K	A		R		P	D	T	L	G	I
W	Y	K	K	A		R		P	D	T	L	G	I
W	Y	K	K	A		R		P	D	T	L	G	I
W	Y	K	K	A		R		P	D	T	L	G	I
W	Y	K	K	A		R		P	D	T	L	G	I
W	Y	K	K	A		R		P	D	T	L	G	I
W	Y	K	K	A		R		P	D	T	L	G	I
W	Y	K	K	A		R		P	D	T	L	G	I
W	Y	K	K	A		R		P	D	T	L	G	I
W	Y	K	K	A		R		P	D	T	L	G	I
W	Y	K	K	A		R		P	D	T	L	G	I
W	Y	K	K	A		R		P	D	T	L	G	I
W	Y	K	K	A		R		P	D	T	L	G	I
W	Y	K	K	A		R		P	D	T	L	G	I
W	Y	K	K	A		R		P	D	T	L	G	I
W	Y	K	K	A		R		P	D	T	L	G	I
W	Y	K	K	A		R		P	D	T	L	G	I
W	Y	K	K	A		R		P	D	T	L	G	I
W	Y	K	K	A		R		P	D	T	L	G	I
W	Y	K	K	A		R		P	D	T	L	G	I
W	Y	K	K	A		R		P	D	T	L	G	I
W	Y	K	K	A		R		P	D	T	L	G	I
W	Y	K	K	A		R		P	D	T	L	G	I
W	Y	K	K	A		R		P	D	T	L	G	I
W	Y	K	K	A		R		P	D	T	L	G	I
W	Y	K	K	A		R		P	D	T	L	G	I
W	Y	K	K	A		R		P	D	T	L	G	I
W	Y	K	K	A		R		P	D	T	L	G	I
W	Y	K	K	A		R		P	D	T	L	G	I
W	Y	K	K	A		R		P	D	T	L	G	I
W	Y	K	K	A		R		P	D	T	L	G	I
W	Y	K	K	A		R		P	D	T	L	G	I
W	Y	K	K	A		R		P	D	T	L	G	I
W	Y	K	K	A		R		P	D	T	L	G	I
W	Y	K	K	A		R		P	D	T	L	G	I
W	Y	K	K	A		R		P	D	T	L	G	I
W	Y	K	K	A		R		P	D	T	L	G	I
W	Y	K	K	A		R		P	D	T	L	G	I
W	Y	K	K	A		R		P	D	T	L	G	I
W	Y	K	K	A		R		P	D	T	L	G	I
W	Y	K	K	A		R		P	D	T	L	G	I
W	Y	K	K	A		R		P	D	T	L	G	I
W	Y	K	K	A		R		P	D	T	L	G	I
W	Y	K	K	A		R		P	D	T	L	G	I
W	Y	K	K	A		R		P	D	T	L	D	I
W	Y	K	K	A		R		P	D	T	L	D	I
W	Y	K	K	A		R		P	D	T	L	D	I
W	Y	K	K	A		R		P	D	T	L	G	I
W	Y	K	K	A		R		P	D	T	L	G	I
W	Y	K	K	A		R		P	D	T	L	G	I

W	Y	K	K	A		R		P	D	T	L	G	I
W	Y	K	K	S		R		P	D	T	L	G	I
W	Y	K	K	A		R		P	D	T	L	G	I
W	Y	K	K	A		R		P	D	T	L	G	I
W	Y	K	K	A		R		P	D	T	L	G	I
W	Y	K	K	A		R		P	D	T	L	G	I
W	Y	K	K	A		R		P	D	T	L	G	I
W	Y	K	K	A		R		P	D	T	L	G	I
W	Y	K	K	A		R		P	D	T	L	G	I
W	Y	K	N	A		R		P	D	T	L	G	I
W	Y	K	K	A		R		P	D	T	L	G	I
W	Y	K	K	A		R		P	D	T	L	G	I
W	Y	K	K	A		R		P	D	T	L	G	T
W	Y	K	K	A		R		P	D	T	L	G	I
W	Y	K	K	A		R		P	D	T	L	G	I
W	Y	K	K	A		R		P	D	T	L	G	I
W	Y	K	K	A		R		P	D	T	L	G	I
W	Y	K	K	A		R		P	D	T	L	G	I
W	Y	K	K	A		R		P	D	T	L	G	I
W	Y	K	K	A		R		P	D	T	L	G	I
W	Y	K	K	A		R		P	D	T	L	G	I
W	Y	K	K	A		R		P	D	T	L	G	I
W	Y	K	K	A		R		P	D	T	L	G	I
W	Y	K	K	A		R		P	D	T	L	G	I
W	Y	K	K	A		R		P	D	T	L	G	I
W	Y	K	K	A		R		P	D	T	L	G	I
W	Y	K	K	A		R		P	D	T	L	G	I
W	Y	K	K	A		R		P	D	T	L	G	I
W	Y	K	K	A		R		P	D	T	L	G	I
W	C	K	K	A		R		P	D	T	L	G	I
W	Y	K	K	A		R		P	D	T	L	G	I
W	Y	K	K	A		R		P	D	T	L	G	I
W	Y	K	K	A		R		P	D	T	L	G	I
W	Y	K	K	A		R		P	D	T	L	G	I
W	Y	K	K	A		R		P	D	T	L	G	I
W	Y	K	K	A		R		P	D	T	L	G	I
W	Y	K	K	A		R		P	D	T	L	G	I
W	Y	K	K	A		R		P	D	T	L	G	I
W	Y	K	K	A		R		P	D	T	L	G	I
W	Y	K	K	A		R		P	D	T	L	G	I
W	Y	K	K	A		R		P	D	T	L	G	I
W	Y	K	K	A		R		P	D	I	F	G	I
W	Y	K	K	A		R		P	D	T	L	G	I
W	Y	K	K	A		R		P	D	T	L	G	I
W	Y	K	K	A		R		P	D	T	L	G	I
W	Y	K	K	A		R		P	D	T	L	G	I
W	Y	K	K	A		R		P	D	T	L	G	I
W	Y	K	K	A		R		P	D	T	L	G	I
W	Y	K	K	A		R		P	D	T	L	G	I
W	Y	K	K	A		R		P	D	T	L	G	I
W	Y	K	K	A		R		P	D	T	L	G	I
W	Y	K	K	A		R		P	D	T	L	G	I
W	Y	K	K	A		R		P	D	T	L	G	I
W	Y	K	K	A		C		P	D	T	L	G	I
W	Y	K	K	A		R		P	D	T	L	G	I
W	Y	K	K	A		R		P	D	T	L	G	I
W	Y	K	K	A		R		P	D	T	L	G	I
W	Y	K	K	A		R		P	D	T	L	G	I
W	Y	K	K	A		R		P	D	T	L	G	I
W	Y	K	K	A		R		P	D	T	L	G	I
W	Y	K	K	A		R		P	D	T	L	G	I
W	Y	K	K	A		R		P	D	T	L	G	I
W	Y	K	K	A		R		P	D	T	L	G	I
W	Y	K	K	A		R		P	D	T	L	G	I
W	Y	K	K	A		R		P	D	T	L	G	I
W	Y	K	K	A		R		P	D	T	L	G	I
W	Y	K	K	A		R		P	D	T	L	G	I

W	Y	K	K	A		R		P	D	T	L	G	I
W	Y	K	K	A		R		P	D	T	L	G	I
W	Y	K	K	A		R		P	D	T	L	G	I
W	Y	K	K	A		R		P	D	T	L	G	I
W	Y	K	K	A		R		P	D	T	L	G	I
W	Y	K	K	A		R		P	D	T	L	G	I
W	Y	K	K	A		R		P	D	T	L	G	I
W	Y	K	K	A		R		P	D	T	L	G	I
W	Y	K	K	A		R		P	D	T	L	G	I
W	Y	K	K	A		R		P	D	T	L	G	I
W	Y	K	K	A		R		P	D	T	L	G	I
W	Y	K	K	A		R		P	D	T	L	G	I
W	Y	K	K	A		R		P	D	T	L	G	I
W	Y	K	K	A		R		P	D	T	L	G	I
W	Y	K	K	A		R		P	D	T	L	G	I
W	Y	K	K	A		R		P	D	T	L	G	I
W	Y	K	K	A		R		P	D	T	L	G	I
W	Y	K	K	A		R		P	D	T	L	G	I
W	Y	K	K	A		R		P	D	T	L	G	I
W	Y	K	K	A		R		P	D	T	L	G	I

wzy		wcwT			wcwU														
83	303	26	131	158	43	84	141	171	193	201	235	242	253	282	284	288	290	332	345
I	I	G	I	Q	N	V	S	G	P	N	E	N	A	D	N	T	L	L	K
I	I	G	I	Q	N	V	S	G	P	N	E	N	A	D	N	T	L	L	K
I	I	G	I	Q	N	V	S	G	P	N	E	N	A	D	N	T	L	L	K
I	I	G	I	Q	N	V	S	G	P	N	E	N	A	D	N	T	L	L	K
I	I	G	I	Q	N	V	S	G	P	N	E	N	A	D	N	T	L	L	K
I	I	G	I	Q	N	V	S	G	P	N	E	N	A	D	N	T	L	L	K
I	I	G	I	Q	N	V	S	G	P	N	E	N	A	D	N	T	L	L	K
I	I	G	I	Q	N	V	S	G	P	N	E	N	A	D	N	T	L	L	K
I	I	G	I	Q	N	V	S	G	P	N	E	N	A	D	N	T	L	L	K
I	I	G	I	Q	N	V	S	G	P	N	E	N	A	D	N	T	L	L	K
I	I	G	I	Q	N	V	S	G	P	N	E	N	A	D	N	T	L	L	K
I	I	G	I	Q	N	V	S	G	P	N	E	N	A	D	N	T	L	L	K
I	I	G	I	Q	N	V	S	G	P	N	E	N	A	D	N	T	L	L	K
I	I	G	I	Q	N	V	S	G	P	N	E	N	A	D	N	T	L	L	K
I	I	G	I	Q	N	V	S	G	P	N	E	N	A	D	N	T	L	L	K
I	I	G	I	Q	N	V	S	G	P	N	E	N	A	D	N	T	L	L	K
I	I	G	I	Q	N	V	S	G	P	N	E	N	A	D	N	T	L	L	K
I	I	G	I	Q	N	V	S	G	P	N	E	N	A	D	N	T	L	L	K
I	I	G	I	Q	N	V	S	G	P	N	E	N	A	D	N	T	L	L	K
I	I	G	I	Q	N	V	S	G	P	N	E	N	A	D	N	T	L	L	K
I	I	G	I	Q	N	V	S	G	P	N	E	N	A	D	N	T	L	L	K
I	I	G	I	Q	N	V	S	G	P	N	E	N	A	D	N	T	L	L	K
I	I	G	I	Q	N	V	S	G	P	N	E	N	A	D	N	T	L	L	K
I	I	G	I	Q	N	V	S	G	P	N	E	N	A	D	N	T	L	L	K
I	I	G	I	Q	N	V	S	G	P	N	E	N	A	D	N	T	L	L	K
I	I	G	I	Q	N	V	S	G	P	N	E	N	A	D	N	T	L	L	K
I	I	G	I	Q	N	V	S	G	P	N	E	N	A	D	N	T	L	L	K
I	I	G	I	Q	N	V	S	G	P	N	E	N	A	D	N	T	L	L	K
I	I	G	I	Q	N	V	S	G	P	N	E	N	A	D	N	T	L	L	K
I	I	G	I	Q	N	V	S	G	P	N	E	N	A	D	N	T	L	L	K
I	I	G	I	Q	N	V	S	G	P	N	E	N	A	D	N	T	L	L	K
I	I	G	I	Q	N	V	S	G	P	N	E	N	A	D	N	T	L	L	R
I	I	G	I	Q	N	V	S	G	P	N	E	N	A	D	N	T	L	L	K
I	I	G	I	Q	N	V	S	G	P	N	E	N	A	D	N	T	L	L	K
I	I	G	I	Q	N	V	S	G	P	N	E	N	A	D	N	T	L	L	K
I	I	G	I	Q	N	V	S	G	P	N	E	N	A	D	N	T	L	L	K
I	I	G	I	Q	N	V	S	G	P	N	E	N	A	D	N	T	L	L	K
I	I	G	I	Q	N	V	S	G	P	N	E	N	A	D	N	T	L	L	K
I	I	G	I	Q	N	V	S	G	P	N	E	N	A	D	N	T	L	L	K
I	I	G	I	Q	N	V	S	G	P	N	E	N	A	D	N	T	L	L	K
I	I	G	I	Q	N	V	S	G	P	N	E	N	A	D	N	T	L	L	K
I	I	G	I	Q	N	V	S	G	P	N	E	N	A	D	N	T	L	L	K
I	I	G	I	Q	N	V	S	G	P	N	E	N	A	D	N	T	L	L	K
I	I	G	I	Q	N	V	S	G	P	N	E	N	A	D	N	T	L	L	K
I	I	G	I	Q	N	V	S	G	P	N	E	N	A	D	N	T	L	L	K
I	I	G	I	Q	N	V	S	G	P	N	E	N	A	D	N	T	L	L	K
I	I	G	I	Q	N	V	S	G	P	N	E	N	A	D	N	T	L	L	K
I	I	G	I	Q	N	V	S	G	P	N	E	N	A	D	N	T	L	L	K
I	I	G	I	Q	N	V	S	G	P	N	E	N	A	D	N	T	L	L	K
I	I	G	I	Q	N	V	S	G	P	N	E	N	A	D	N	T	L	L	K
I	I	G	I	Q	N	V	S	G	P	N	E	N	A	D	N	T	L	L	K
I	I	G	I	Q	N	V	S	G	P	N	E	N	A	D	N	T	L	L	K
I	I	G	I	Q	N	V	S	G	P	N	E	N	A	D	N	T	L	L	K
I	I	G	I	Q	N	V	S	G	P	N	E	N	A	D	N	T	L	L	K
I	I	G	I	Q	N	V	S	G	P	N	E	N	A	D	N	T	L	L	K
I	I	G	I	Q	N	V	S	G	P	N	E	N	A	D	N	T	L	L	K
I	I	G	I	Q	N	V	S	G	P	N	E	N	A	D	N	T	L	L	K
I	I	G	I	Q	N	V	S	G	P	N	E	N	A	D	N	T	L	L	K
I	I	G	I	Q	N	V	S	G	P	N	E	N	A	D	N	T	L	L	K
I	I	G	I	Q	N	V	S	G	P	N	E	N	A	D	N	T	L	L	K
I	I	G	I	Q	N	V	S	G	P	N	E	N	A	D	N	T	L	L	K
I	I	G	I	Q	N	V	S	G	P	N	E	N	A	D	N	T	L	L	K
I	I	G	I	Q	N	V	S	G	P	N	E	N	A	D	N	T	L	L	K

I	I	G	I	Q	N	V	S	G	P	N	E	N	A	D	N	T	L	L	K
I	I	G	I	Q	N	V	S	G	P	N	E	N	A	D	N	T	L	L	K
I	I	G	I	Q	N	V	S	G	P	N	E	N	A	D	N	T	L	L	K
I	I	G	I	Q	N	V	S	G	P	N	E	N	A	D	N	T	L	L	K
I	I	G	I	Q	N	V	S	G	P	N	E	N	A	D	N	T	L	L	K
I	I	G	I	Q	N	V	S	G	P	N	E	N	A	D	N	T	L	L	K
I	I	G	I	Q	N	V	S	G	P	N	E	N	A	D	N	T	L	L	K
I	I	G	I	Q	N	V	S	G	P	N	E	N	A	D	N	T	L	L	K
I	I	G	I	Q	N	V	S	G	P	N	E	N	A	D	N	T	L	L	K
I	I	G	I	Q	N	V	S	G	P	N	E	N	A	D	N	T	L	L	K
I	I	G	I	Q	N	V	S	G	P	N	E	N	A	D	N	T	L	L	K
I	I	G	I	Q	N	V	S	G	P	N	E	N	A	D	N	T	L	L	K
I	I	G	I	Q	N	V	S	G	P	N	E	N	A	D	N	T	L	L	K
I	I	G	I	Q	N	V	S	G	P	N	E	N	A	D	N	T	L	L	K
I	I	G	I	Q	N	V	S	G	P	N	E	N	A	D	N	T	L	L	K
I	I	G	I	Q	N	V	S	G	P	N	E	N	A	D	N	T	L	L	K
I	I	G	I	Q	N	V	S	G	P	N	E	N	A	D	N	T	L	L	K
I	I	G	I	Q	N	V	S	G	P	N	E	N	A	D	N	T	L	L	K
I	I	G	I	Q	N	V	S	G	P	N	E	N	A	D	N	T	L	L	K
I	I	G	I	Q	N	V	S	G	P	N	E	N	A	D	N	T	L	L	K
I	I	G	I	Q	N	V	S	G	P	N	E	N	A	D	N	T	L	L	K
I	I	G	I	Q	N	V	S	G	P	N	E	N	A	D	N	T	L	L	K
I	I	G	I	Q	N	V	S	G	P	N	E	N	A	D	N	T	L	L	K
I	I	G	I	Q	N	V	S	E	P	N	E	N	A	D	N	T	L	L	K
I	I	G	I	Q	N	V	S	G	P	N	E	N	A	D	N	T	L	L	K
I	I	G	I	Q	N	V	S	G	P	N	E	N	A	D	N	T	L	L	K
I	I	G	I	Q	N	V	S	G	P	N	E	N	A	D	N	T	L	L	K
I	I	G	I	Q	N	V	S	G	P	N	E	N	A	D	N	T	L	L	K
I	T	G	I	Q	N	V	S	G	P	N	E	N	A	D	N	T	L	L	K
I	I	G	I	Q	N	V	S	G	P	N	E	N	A	D	N	T	L	L	K
I	I	G	I	Q	N	V	S	G	P	N	E	N	A	D	N	T	L	L	K
I	I	G	I	Q	N	V	S	E	P	N	E	N	A	D	N	T	L	L	K
I	I	G	I	Q	N	V	S	G	P	N	E	N	A	D	N	T	L	L	K
I	I	G	I	Q	N	V	S	G	P	N	E	N	A	D	N	T	L	L	K
I	I	G	I	Q	N	V	S	G	P	N	E	N	A	D	N	T	L	L	K
I	I	G	I	Q	N	V	S	G	P	N	E	N	A	D	N	T	L	L	K
I	I	G	I	Q	N	V	S	G	P	N	E	N	A	D	N	T	L	L	K
I	I	G	I	Q	N	V	S	G	P	N	E	N	A	D	N	T	L	L	K
I	I	G	I	Q	N	V	S	G	P	N	E	N	A	D	N	T	L	L	K
I	I	G	I	Q	N	V	S	G	P	N	E	N	A	D	N	T	L	L	K
I	I	G	I	Q	N	V	S	G	P	N	E	N	A	D	N	T	L	L	K
I	I	G	I	Q	N	V	S	G	P	N	E	N	A	D	N	T	L	L	K
I	I	G	I	Q	N	V	S	G	P	N	E	N	A	D	N	T	L	L	K
I	I	G	I	Q	N	V	S	G	P	N	E	N	A	D	N	T	L	L	K
I	I	G	I	Q	N	V	S	G	P	N	E	N	A	D	N	T	L	L	K
I	I	G	I	Q	N	V	S	G	P	N	E	N	A	D	N	T	L	L	K
I	I	G	I	Q	N	V	S	G	P	N	E	N	A	D	N	T	L	L	K
I	I	G	I	Q	N	V	S	G	P	N	E	N	A	D	N	T	L	L	K
I	I	G	I	Q	N	V	S	G	P	N	E	N	A	D	N	T	L	L	K
I	I	G	I	Q	N	V	S	G	P	N	E	N	A	D	N	T	L	L	K
I	I	G	I	Q	N	V	S	G	P	N	E	N	A	D	N	T	L	L	K
I	I	G	I	Q	N	V	S	G	P	N	E	N	A	D	N	T	L	L	K
I	I	G	I	Q	N	V	S	G	P	N	E	N	A	D	N	T	L	L	K
I	I	G	I	Q	N	V	S	G	P	N	E	N	A	D	N	T	L	L	K
I	I	G	I	Q	N	V	S	G	P	N	E	N	A	D	N	T	L	L	K
I	I	G	I	Q	N	V	S	G	P	N	E	N	A	D	N	T	L	L	K
I	I	G	I	Q	N	V	S	G	P	N	E	N	A	D	N	T	L	L	K
I	I	G	I	Q	N	V	S	G	P	N	E	N	A	D	N	T	L	L	K
I	I	G	I	Q	N	V	S	G	P	N	E	N	A	D	N	T	L	L	K
I	I	G	I	Q	N	V	S	G	P	N	E	N	A	D	N	T	L	L	K
I	I	G	I	Q	N	V	S	G	P	N	E	N	A	D	N	T	L	L	K
I	I	G	I	Q	N	V	S	G	P	N	E	N	A	D	N	T	L	L	K
I	I	G	I	Q	N	V	S	G	P	N	E	N	A	D	N	T	L	L	K
I	I	G	I	Q	N	V	S	G	P	N	E	N	A	D	N	T	L	L	K
I	I	G	I	Q	N	V	S	G	P	N	E	N	A	D	N	T	L	L	K
I	I	G	I	Q	N	V	S	G	P	N	E	N	A	D	N	T	L	L	K
I	I	G	I	Q	N	V	S	G	P	N	E	N	A	D	N	T	L	L	K

I	I
I	I
I	I
I	I
I	I
I	I
I	I
I	I
I	I
I	I
I	I
I	I
I	I
I	I
I	I
I	I
I	I
I	I
I	I
I	I
I	I
I	I
I	I
I	I
I	I
I	I
I	I
I	I
I	I
I	I
I	I
I	I
I	I
I	I
I	I
I	I
I	I
M	I

G	I	Q
G	I	Q
G	I	Q
G	I	Q
G	I	Q
G	I	Q
G	I	Q
G	I	Q
G	I	Q
G	I	Q
G	I	Q
G	I	Q
G	I	Q
G	I	Q
G	I	Q
G	I	Q
G	I	Q
G	I	Q
G	I	Q
G	I	Q
G	I	Q
G	I	Q
G	I	Q
G	I	Q
G	I	Q
G	I	Q
G	I	Q
G	I	Q
G	I	Q
G	I	Q
G	I	Q
G	I	Q
G	I	Q
G	I	Q
G	I	Q
G	I	Q
G	I	Q
S	A	R

N	V	S	G	P	N	E	N	A	D	N	T	L	L	K
N	V	S	G	P	N	E	N	A	D	N	T	L	L	K
N	V	S	G	P	N	E	N	A	D	N	T	L	L	K
N	V	S	G	P	N	E	N	A	D	N	T	L	L	K
N	V	S	G	P	N	E	N	A	D	N	T	L	L	K
N	V	S	G	P	N	E	N	A	D	N	T	L	L	K
N	V	S	G	P	N	E	N	A	D	N	T	L	L	K
N	V	S	G	P	N	E	N	A	D	N	T	L	L	K
N	V	S	G	P	N	E	N	A	D	N	T	L	L	K
N	V	S	G	P	N	E	N	A	D	N	T	L	L	K
N	V	S	G	P	N	E	N	A	D	N	T	L	L	K
N	V	S	G	P	N	E	N	A	D	N	T	L	L	K
N	V	S	G	P	N	E	N	A	D	N	T	L	L	K
N	V	S	G	P	N	E	N	A	D	N	T	L	L	K
N	V	S	G	P	N	E	N	A	D	N	T	L	L	K
N	V	S	G	P	N	E	N	A	D	N	T	L	L	K
N	V	S	G	P	N	E	N	A	D	N	T	L	L	K
N	V	S	G	P	N	E	N	A	D	N	T	L	L	K
N	V	S	G	P	N	E	N	A	D	N	T	L	L	K
N	V	S	G	P	N	E	N	A	D	N	T	L	L	K
N	V	S	G	P	N	E	N	A	D	N	T	L	L	K
N	V	S	G	P	N	E	N	A	D	N	T	L	L	K
N	V	S	G	P	N	E	N	A	D	N	T	L	L	K
N	V	S	G	P	N	E	N	A	D	N	T	L	L	K
N	V	S	G	P	N	E	N	A	D	N	T	L	L	K
N	V	S	G	P	N	E	N	A	D	N	T	L	L	K
N	V	S	G	P	N	E	N	A	D	N	T	L	L	K
N	V	S	G	P	N	E	N	A	D	N	T	L	L	K
N	V	S	G	P	N	E	N	A	D	N	T	L	L	K
N	V	S	G	P	N	E	N	A	D	N	T	L	L	K
N	V	S	G	P	N	E	N	A	D	N	T	L	L	K
N	V	S	G	P	N	E	N	A	D	N	T	L	L	K
N	V	S	G	P	N	E	N	A	D	N	T	L	L	K
N	V	S	G	P	N	E	N	A	D	N	T	L	L	K
N	V	S	G	P	N	E	N	A	D	N	T	L	L	K
N	V	S	G	P	N	E	N	A	D	N	T	L	L	K
N	V	S	G	P	N	E	N	A	D	N	T	L	L	K
D	S	N	G	T	D	G	H	V	N	K	I	M	F	K

wze																				
80	85	87	88	140	150	156	158	166	167	177	181	182	186	195	199	200	213	223	225	227
S	S	E	R	G	V	I	I	M	Q	K	T	K	L	Q	S	C	E	N	R	Q
L	A	D	K	E	I	V	V	T	R	E	I	N	I	H	P	F	D	D	G	N
S	S	E	R	G	V	I	I	M	Q	K	T	K	L	Q	S	C	E	N	R	Q

wcwT

wcwU

wzx

gct

wcjE							
30	76	182	185	193	196	234	287
S	H	V	C	V	N	S	S
S	H	V	C	V	N	S	S
S	H	V	C	V	N	S	S
S	H	V	C	V	N	S	S
S	Y	I	F	I	D	I	S
S	Y	I	F	I	D	I	S
S	Y	I	F	I	D	I	S
S	Y	I	F	I	D	I	S
S	Y	I	F	I	D	I	S
S	Y	I	F	I	D	I	S
S	Y	I	F	I	D	I	S
S	Y	I	F	I	D	I	S
S	Y	I	F	I	D	I	S

S	Y	I	F	I	D	I	S
S	Y	I	F	I	D	I	S
S	Y	I	F	I	D	I	S
S	Y	I	F	I	D	I	S
S	Y	I	F	I	D	I	S
S	Y	I	F	I	D	I	S
S	Y	I	F	I	D	I	S
S	Y	I	F	I	D	I	S
S	Y	I	F	I	D	I	S
S	Y	I	F	I	D	I	S
S	Y	I	F	I	D	I	S
S	Y	I	F	I	D	I	S
S	Y	I	F	I	D	I	S
S	Y	I	F	I	D	I	S
S	Y	I	F	I	D	I	S
S	Y	I	F	I	D	I	S
S	Y	I	F	I	D	I	S
S	Y	I	F	I	D	I	S
S	Y	I	F	I	D	I	S
S	Y	I	F	I	D	I	S
S	Y	I	F	I	D	I	S
S	Y	I	F	I	D	I	S
S	Y	I	F	I	D	I	S
S	Y	I	F	I	D	I	S
S	Y	I	F	I	D	I	S
S	Y	I	F	I	D	I	S
S	Y	I	F	I	D	I	S
S	Y	I	F	I	D	I	S
S	Y	I	F	I	D	I	S
S	Y	I	F	I	D	I	S
S	Y	I	F	I	D	I	S
S	Y	I	F	I	D	I	S
S	Y	I	F	I	D	I	S
S	Y	I	F	I	D	I	S
S	Y	I	F	I	D	I	S
S	Y	I	F	I	D	I	S
S	Y	I	F	I	D	I	S
S	Y	I	F	I	D	I	S
S	Y	I	F	I	D	I	S
S	Y	I	F	I	D	I	S
S	Y	I	F	I	D	I	S
S	Y	I	F	I	D	I	S
S	Y	I	F	I	D	I	S
S	Y	I	F	I	D	I	S
S	Y	I	F	I	D	I	S
S	Y	I	F	I	D	I	S
S	Y	I	F	I	D	I	S
S	Y	I	F	I	D	I	S
S	Y	I	F	I	D	I	S
S	Y	I	F	I	D	I	S
S	Y	I	F	I	D	I	S
S	Y	I	F	I	D	I	S
S	Y	I	F	I	D	I	S
S	Y	I	F	I	D	I	S
S	Y	I	F	I	D	I	S
S	Y	I	F	I	D	I	S
S	Y	I	F	I	D	I	S
S	Y	I	F	I	D	I	S
S	Y	I	F	I	D	I	S
S	Y	I	F	I	D	I	S
S	Y	I	F	I	D	I	S
S	Y	I	F	I	D	I	S
S	Y	I	F	I	D	I	S
S	Y	I	F	I	D	I	S
S	Y	I	F	I	D	I	S
S	Y	I	F	I	D	I	S
S	Y	I	F	I	D	I	S

S	Y	I	F	I	D	I	S
S	Y	I	F	I	D	I	N
S	Y	I	F	I	D	I	S
S	Y	I	F	I	D	I	S
S	Y	I	F	I	D	I	S
S	Y	I	F	I	D	I	S
S	Y	I	F	I	D	I	S
S	Y	I	F	I	D	I	S
S	Y	I	F	I	D	I	S
S	Y	I	F	I	D	I	S
S	Y	I	F	I	D	I	S
S	Y	I	F	I	D	I	S
S	Y	I	F	I	D	I	S
S	Y	I	F	I	D	I	S
S	Y	I	F	I	D	I	S
S	Y	I	F	I	D	I	S
S	Y	I	F	I	D	I	S
S	Y	I	F	I	D	I	S
S	Y	I	F	I	D	I	S
S	Y	I	F	I	D	I	S
S	Y	I	F	I	D	I	S
S	Y	I	F	I	D	I	S
S	Y	I	F	I	D	I	S
S	Y	I	F	I	D	I	S
S	Y	I	F	I	D	I	S
S	Y	I	F	I	D	I	S
S	Y	I	F	I	D	I	S
S	Y	I	F	I	D	I	S
S	Y	I	F	I	D	I	S
S	Y	I	F	I	D	I	S
S	Y	I	F	I	D	I	S
S	Y	I	F	I	D	I	S
S	Y	I	F	I	D	I	S
S	Y	I	F	I	D	I	S
S	Y	I	F	I	D	I	S
S	Y	I	F	I	D	I	S
S	Y	I	F	I	D	I	S
S	Y	I	F	I	D	I	S
S	Y	I	F	I	D	I	S
S	Y	I	F	I	D	I	S
S	Y	I	F	I	D	I	S
S	Y	I	F	I	D	I	S
S	Y	I	F	I	D	I	S
S	Y	I	F	I	D	I	S
S	Y	I	F	I	D	I	S
S	Y	I	F	I	D	I	S
S	Y	I	F	I	D	I	S
S	Y	I	F	I	D	I	S
S	Y	I	F	I	D	I	S
S	Y	I	F	I	D	I	S
S	Y	I	F	I	D	I	S
S	Y	I	F	I	D	I	S
S	Y	I	F	I	D	I	S
S	Y	I	F	I	D	I	S
S	Y	I	F	I	D	I	S
S	Y	I	F	I	D	I	S
S	Y	I	F	I	D	I	S
S	Y	I	F	I	D	I	S
S	Y	I	F	I	D	I	S
S	Y	I	F	I	D	I	S
S	Y	I	F	I	D	I	S
S	Y	I	F	I	D	I	S
S	Y	I	F	I	D	I	S
S	Y	I	F	I	D	I	S
S	Y	I	F	I	D	I	S
S	Y	I	F	I	D	I	S
S	Y	I	F	I	D	I	S

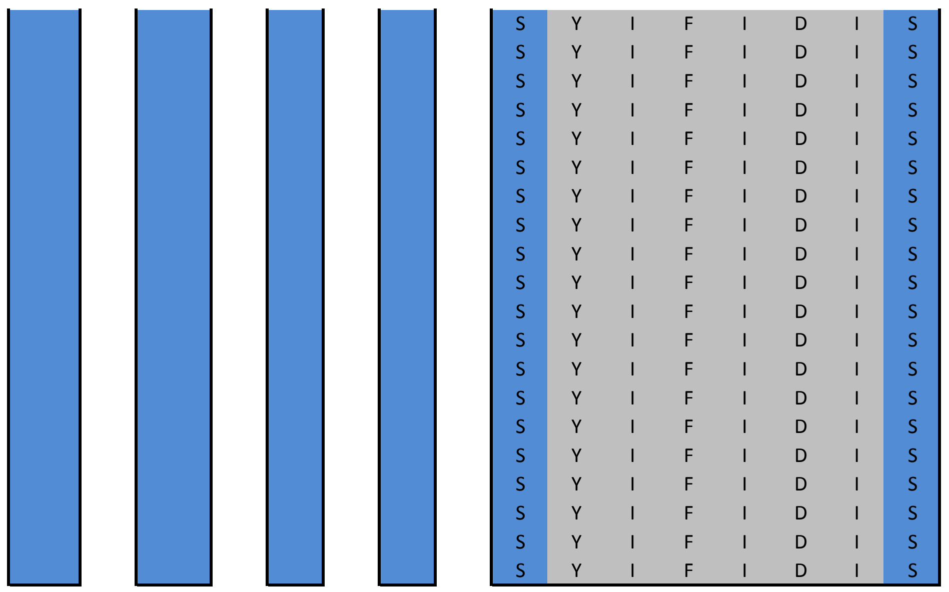
S Y I F I D I S
S Y I F I D I S
S Y I F I D I S
S Y I F I D I S
S Y I F I D I S
S Y I F I D I S
S Y I F I D I S
S Y I F I D I S
S Y I F I D I S
S Y I F I D I S
S Y I F I D I S
S Y I F I D I S
S Y I F I D I S
S Y I F I D I S
S Y I F I D I S
S Y I F I D I S
S Y I F I D I S
S Y I F I D I S
S Y I F I D I S
S Y I F I D I S

											wzx										
347	351	5	11	12	104	129	165	166	170	181	234	287	298	309	310	314	316	398	444	457	464
L	N	H	K	S	T	S	T	F	F	A	I	H	C	V	V	I	A	A	I	I	A
L	N	H	K	S	T	S	T	F	F	A	I	H	C	V	V	I	A	A	I	I	A
L	N	H	K	S	T	S	T	F	F	A	I	H	C	V	V	I	A	A	I	I	A
L	N	H	K	S	T	S	T	F	F	A	I	H	C	V	V	I	A	A	I	I	A
L	N	H	K	S	T	S	T	F	F	A	I	H	C	V	V	I	A	A	I	I	A
L	N	H	K	S	T	S	T	F	F	A	I	H	C	V	V	I	A	A	I	I	A
L	N	H	K	S	T	S	T	F	F	A	I	H	C	V	V	I	A	A	I	I	A
L	N	H	K	S	T	S	T	F	F	A	I	H	C	V	V	I	A	A	I	I	A
L	N	H	K	S	T	S	T	F	F	A	I	H	C	V	V	I	A	A	I	I	A
L	N	H	K	S	T	S	T	F	F	A	I	H	C	V	V	I	A	A	I	I	A
L	N	H	K	S	T	S	T	F	F	A	I	H	C	V	V	I	A	A	I	I	A
L	N	H	K	S	T	S	T	F	F	A	I	H	C	V	V	I	A	A	I	I	A
L	N	H	K	S	T	S	T	F	F	A	I	H	C	V	V	I	A	A	I	I	A
L	N	H	K	S	T	S	T	F	F	A	I	H	C	V	V	I	A	A	I	I	A
L	N	H	K	S	T	S	T	F	F	A	I	H	C	V	V	I	A	A	I	I	A
L	N	H	K	S	T	S	T	F	F	A	I	H	C	V	V	I	A	A	I	I	A
L	N	H	K	S	T	S	T	F	F	A	I	H	C	V	V	I	A	A	I	I	A
L	N	H	K	S	T	S	T	F	F	A	I	H	C	V	V	I	A	A	I	I	A
L	N	H	K	S	T	S	T	F	F	A	I	H	C	V	V	I	A	A	I	I	A
L	N	H	K	S	T	S	T	F	F	A	I	H	C	V	V	I	A	A	I	I	A
L	N	H	K	S	T	S	T	F	F	A	I	H	C	V	V	I	A	A	I	I	A
L	N	H	K	S	T	S	T	F	F	A	I	H	C	V	V	I	A	A	I	I	A
L	N	H	K	S	T	S	T	F	F	A	I	H	C	V	V	I	A	A	I	I	A
L	N	H	K	S	T	S	T	F	F	A	I	H	C	V	V	I	A	A	I	I	A
L	N	H	K	S	T	S	T	F	F	A	I	H	C	V	V	I	A	A	I	I	A
L	N	H	K	S	T	S	T	F	F	A	I	H	C	V	V	I	A	A	I	I	A
L	N	H	K	S	T	S	T	F	F	A	I	H	C	V	V	I	A	A	I	I	A
L	N	H	K	S	T	S	T	F	F	A	I	H	C	V	V	I	A	A	I	I	A
L	N	H	K	S	T	S	T	F	F	A	I	H	C	V	V	I	A	A	I	I	A
L	N	H	K	S	T	S	T	F	F	A	I	H	C	V	V	I	A	A	I	I	A
L	N	H	K	S	T	S	T	F	F	A	I	H	C	V	V	I	A	A	I	I	A
L	N	H	K	S	T	S	T	F	F	A	I	H	C	V	V	I	A	A	I	I	A
L	N	H	K	S	T	S	T	F	F	A	I	H	C	V	V	I	A	A	I	I	A
L	N	H	K	S	T	S	T	F	F	A	I	H	C	V	V	I	A	A	I	I	A
L	N	H	K	S	T	S	T	F	F	A	I	H	C	V	V	I	A	A	I	I	A
L	N	H	K	S	T	S	T	F	F	A	I	H	C	V	V	I	A	A	I	I	A
L	N	H	K	S	T	S	T	F	F	A	I	H	C	V	V	I	A	A	I	I	A
L	N	H	K	S	T	S	T	F	F	A	I	H	C	V	V	I	A	A	I	I	A
L	N	H	K	S	T	S	T	F	F	A	I	H	C	V	V	I	A	A	I	I	A
L	N	H	K	S	T	S	T	F	F	A	I	H	C	V	V	I	A	A	I	I	A
L	N	H	K	S	T	S	T	F	F	A	I	H	C	V	V	I	A	A	I	I	A
L	N	H	K	S	T	S	T	F	F	A	I	H	C	V	V	I	A	A	I	I	A
L	N	H	K	S	T	S	T	F	F	A	I	H	C	V	V	I	A	A	I	I	A
L	N	H	K	S	T	S	T	F	F	A	I	H	C	V	V	I	A	A	I	I	A
L	N	H	K	S	T	S	T	F	F	A	I	H	C	V	V	I	A	A	I	I	A
L	N	H	K	S	T	S	T	F	F	A	I	H	C	V	V	I	A	A	I	I	A
L	N	H	K	S	T	S	T	F	F	A	I	H	C	V	V	I	A	A	I	I	A
L	N	H	K	S	T	S	T	F	F	A	I	H	C	V	V	I	A	A	I	I	A
L	N	H	K	S	T	S	T	F	F	A	I	H	C	V	V	I	A	A	I	I	A
L	N	H	K	S	T	S	T	F	F	A	I	H	C	V	V	I	A	A	I	I	A
L	N	H	K	S	T	S	T	F	F	A	I	H	C	V	V	I	A	A	I	I	A
L	N	H	K	S	T	S	T	F	F	A	I	H	C	V	V	I	A	A	I	I	A
L	N	H	K	S	T	S	T	F	F	A	I	H	C	V	V	I	A	A	I	I	A
L	N	H	K	S	T	S	T	F	F	A	I	H	C	V	V	I	A	A	I	I	A
L	N	H	K	S	T	S	T	F	F	A	I	H	C	V	V	I	A	A	I	I	A
L	N	H	K	S	T	S	T	F	F	A	I	H	C	V	V	I	A	A	I	I	A
L	N	H	K	S	T	S	T	F	F	A	I	H	C	V	V	I	A	A	I	I	A
L	N	H	K	S	T	S	T	F	F	A	I	H	C	V	V	I	A	A	I	I	A
L	N	H	K	S	T	S	T	F	F	A	I	H	C	V	V	I	A	A	I	I	A
L	N	H	K	S	T	S	T	F	F	A	I	H	C	V	V	I	A	A	I	I	A
L	N	H	K	S	T	S	T	F	F	A	I	H	C	V	V	I	A	A	I	I	A
L	N	H	K	S	T	S	T	F	F	A	I	H	C	V	V	I	A	A	I	I	A
L	N	H	K	S	T	S	T	F	F	A	I	H	C	V	V	I	A	A	I	I	A

L	N	H	K	S	T	S	T	F	F	A	I	H	C	V	V	I	A	A	I	I	A
L	N	H	K	S	T	S	T	F	F	A	I	H	C	V	V	I	A	A	I	I	A
L	N	H	K	S	T	S	T	F	F	A	I	H	C	V	V	I	A	A	I	I	A
L	N	H	K	S	T	S	T	F	F	A	I	H	C	V	V	I	A	A	I	I	A
L	N	H	K	S	T	S	T	F	F	A	I	H	C	V	V	I	A	A	I	I	A
L	N	H	K	S	T	S	T	F	F	A	I	H	C	V	V	I	A	A	I	I	A
L	N	H	K	S	T	S	T	F	F	A	I	H	C	V	V	I	A	A	I	I	A
L	N	H	K	S	T	S	T	F	F	A	I	H	C	V	V	I	A	A	I	I	A
L	N	H	K	S	T	S	T	F	F	A	I	H	C	V	V	I	A	A	I	I	A
L	N	H	K	S	T	S	T	F	F	A	I	H	C	V	V	I	A	A	I	I	A
L	N	H	K	S	T	S	T	F	F	A	I	H	C	V	V	I	A	A	I	I	A
L	N	H	K	S	T	S	T	F	F	A	I	H	C	V	V	I	A	A	I	I	A
L	N	H	K	S	T	S	T	F	F	A	I	H	C	V	V	I	A	A	I	I	A
L	N	H	K	S	T	S	T	F	F	A	I	H	C	V	V	I	A	A	I	I	A
L	N	H	K	S	T	S	T	F	F	A	I	H	C	V	V	I	A	A	I	I	A
L	N	H	K	S	T	S	T	F	F	A	I	H	C	V	V	I	A	A	I	I	A
L	N	H	K	S	T	S	T	F	F	A	I	H	C	V	V	I	A	A	I	I	A
L	N	H	K	S	T	S	T	F	F	A	I	H	C	V	V	I	A	A	I	I	A
L	N	H	K	S	T	S	T	F	F	A	I	H	C	V	V	I	A	A	I	I	A
L	N	H	K	S	T	S	T	F	F	A	I	H	C	V	V	I	A	A	I	I	A
L	N	H	K	S	T	S	T	F	F	A	I	H	C	V	V	I	A	A	I	I	A
L	N	H	K	S	T	S	T	F	F	A	I	H	C	V	V	I	A	A	I	I	A
L	N	H	K	S	T	S	T	F	F	A	I	H	C	V	V	I	A	A	I	I	A
L	N	H	K	S	T	S	T	F	F	A	I	H	C	V	V	I	A	A	I	I	A
L	N	H	K	S	T	S	T	F	F	A	I	H	C	V	V	I	A	A	I	I	A
L	N	H	K	S	T	S	T	F	F	A	I	H	C	V	V	I	A	A	I	I	A
L	N	H	K	S	T	S	T	F	F	A	I	H	C	V	V	I	A	A	I	I	A
L	N	H	K	S	T	S	T	F	F	A	I	H	C	V	V	I	A	A	I	I	A
L	N	H	K	S	T	S	T	F	F	A	I	H	C	V	V	I	A	A	I	I	A
L	N	H	K	S	T	S	T	F	F	A	I	H	C	V	V	I	A	A	I	I	A
L	N	H	K	S	T	S	T	F	F	A	I	H	C	V	V	I	A	A	I	I	A
L	N	H	K	S	T	S	T	F	F	A	I	H	C	V	V	I	A	A	I	I	A
L	N	H	K	S	T	S	T	F	F	A	I	H	C	V	V	I	A	A	I	I	A
L	N	H	K	S	T	S	T	F	F	A	I	H	C	V	V	I	A	A	I	I	A
L	N	H	K	S	T	S	T	F	F	A	I	H	C	V	V	I	A	A	I	I	A
L	N	H	K	S	T	S	T	F	F	A	I	H	C	V	V	I	A	A	I	I	A
L	N	H	K	S	T	S	T	F	F	A	I	H	C	V	V	I	A	A	I	I	A
L	N	H	K	S	T	S	T	F	F	A	I	H	C	V	V	I	A	A	I	I	A
L	N	H	K	S	T	S	T	F	F	A	I	H	C	V	V	I	A	A	I	I	A
L	N	H	K	S	T	S	T	F	F	A	I	H	C	V	V	I	A	A	I	I	A
L	N	H	K	S	T	S	T	F	F	A	I	H	C	V	V	I	A	A	I	I	A
L	N	H	K	S	T	S	T	F	F	A	I	H	C	V	V	I	A	A	I	I	A
L	N	H	K	S	T	S	T	F	F	A	I	H	C	V	V	I	A	A	I	I	A
L	N	H	K	S	T	S	T	F	F	A	I	H	C	V	V	I	A	A	I	I	A
L	N	H	K	S	T	S	T	F	F	A	I	H	C	V	V	I	A	A	I	I	A
L	N	H	K	S	T	S	T	F	F	A	I	H	C	V	V	I	A	A	I	I	A
L	N	H	K	S	T	S	T	F	F	A	I	H	C	V	V	I	A	A	I	I	A
L	N	H	K	S	T	S	T	F	F	A	I	H	C	V	V	I	A	A	I	I	A
L	N	H	K	S	T	S	T	F	F	A	I	H	C	V	V	I	A	A	I	I	A
L	N	H	K	S	T	S	T	F	F	A	I	H	C	V	V	I	A	A	I	I	A
L	N	H	K	S	T	S	T	F	F	A	I	H	C	V	V	I	A	A	I	I	A
L	N	H	K	S	T	S	T	F	F	A	I	H	C	V	V	I	A	A	I	I	A
L	N	H	K	S	T	S	T	F	F	A	I	H	C	V	V	I	A	A	I	I	A
L	N	H	K	S	T	S	T	F	F	A	I	H	C	V	V	I	A	A	I	I	A
L	N	H	K	S	T	S	T	F	F	A	I	H	C	V	V	I	A	A	I	I	A
L	N	H	K	S	T	S	T	F	F	A	I	H	C	V	V	I	A	A	I	I	A
L	N	H	K	S	T	S	T	F	F	A	I	H	C	V	V	I	A	A	I	I	A
L	N	H	K	S	T	S	T	F	F	A	I	H	C	V	V	I	A	A	I	I	A
L	N	H	K	S	T	S	T	F	F	A	I	H	C	V	V	I	A	A	I	I	A
L	N	H	K	S	T	S	T	F	F	A	I	H	C	V	V	I	A	A	I	I	A
L	N	H	K	S	T	S	T	F	F	A	I	H	C	V	V	I	A	A	I	I	A
L	N	H	K	S	T	S	T	F	F	A	I	H	C	V	V	I	A	A	I	I	A
L	N	H	K	S	T	S	T	F	F	A	I	H	C	V	V	I	A	A	I	I	A
L	N	H	K	S	T	S	T	F	F	A	I	H	C	V	V	I	A	A	I	I	A
L	N	H	K	S	T	S	T	F	F	A	I	H	C	V	V	I	A	A	I	I	A
L	N	H	K	S	T	S	T	F	F	A	I	H	C	V	V	I	A	A	I	I	A
L	N	H	K	S	T	S	T	F	F	A	I	H	C	V	V	I	A	A	I	I	A

L	N	H	K	S	T	S	T	F	F	A	I	H	C	V	V	I	A	A	I	I	A
L	N	H	K	S	T	S	T	F	F	A	I	H	C	V	V	I	A	A	I	I	A
L	N	H	K	S	T	S	T	F	F	A	I	H	C	V	V	I	A	A	I	I	A
L	N	H	K	S	T	S	T	F	F	A	I	H	C	V	V	I	A	A	I	I	A
L	N	H	K	S	T	S	T	F	F	A	I	H	C	V	V	I	A	A	I	I	A
L	N	H	K	S	T	S	T	F	F	A	I	H	C	V	V	I	A	A	I	I	A
L	N	H	K	S	T	S	T	F	F	A	I	H	C	V	V	I	A	A	I	I	A
L	N	H	K	S	T	S	T	F	F	A	I	H	C	V	V	I	A	A	I	I	A
L	N	H	K	S	T	S	T	F	F	A	I	H	C	V	V	I	A	A	I	I	A
L	N	H	K	S	T	S	T	F	F	A	I	H	C	V	V	I	A	A	I	I	A
L	N	H	K	S	T	S	T	F	F	A	I	H	C	V	V	I	A	A	I	I	A
L	N	H	K	S	T	S	T	F	F	A	I	H	C	V	V	I	A	A	I	I	A
L	N	H	K	S	T	S	T	F	F	A	I	H	C	V	V	I	A	A	I	I	A
L	N	H	K	S	T	S	T	F	F	A	I	H	C	V	V	I	A	A	I	I	A
L	N	H	K	S	T	S	T	F	F	A	I	H	C	V	V	I	A	A	I	I	A
L	N	H	K	S	T	S	T	F	F	A	I	H	C	V	V	I	A	A	I	I	A
L	N	H	K	S	T	S	T	F	F	A	I	H	C	V	V	I	A	A	I	I	A
L	N	H	K	S	T	S	T	F	F	A	I	H	C	V	V	I	A	A	I	I	A
L	N	H	K	S	T	S	T	F	F	A	I	H	C	V	V	I	A	A	I	I	A
L	N	H	K	S	T	S	T	F	F	A	I	H	C	V	V	I	A	A	I	I	A
L	N	H	K	S	T	S	T	F	F	A	I	H	C	V	V	I	A	A	I	I	A
L	N	H	K	S	T	S	T	F	F	A	I	H	C	V	V	I	A	A	I	I	A
L	N	H	K	S	T	S	T	F	F	A	I	H	C	V	V	I	A	A	I	I	A
L	N	H	K	S	T	S	T	F	F	A	I	H	C	V	V	I	A	A	I	I	A
L	N	H	K	S	T	S	T	F	F	A	I	H	C	V	V	I	A	A	I	I	A
L	N	H	K	S	T	S	T	F	F	A	I	H	C	V	V	I	A	A	I	I	A
L	N	H	K	S	T	S	T	F	F	A	I	H	C	V	V	I	A	A	I	I	A
L	N	H	K	S	T	S	T	F	F	A	I	H	C	V	V	I	A	A	I	I	A
L	N	H	K	S	T	S	T	F	F	A	I	H	C	V	V	I	A	A	I	I	A
L	N	H	K	S	T	S	T	F	F	A	I	H	C	V	V	I	A	A	I	I	A
L	N	H	K	S	T	S	T	F	F	A	I	H	C	V	V	I	A	A	I	I	A
L	N	H	K	S	T	S	T	F	F	A	I	H	C	V	V	I	A	A	I	I	A
L	N	H	K	S	T	S	T	F	F	A	I	H	C	V	V	I	A	A	I	I	A
L	N	H	K	S	T	S	T	F	F	A	I	H	C	V	V	I	A	A	I	I	A
L	N	H	K	S	T	S	T	F	F	A	I	H	C	V	V	I	A	A	I	I	A
L	N	H	K	S	T	S	T	F	F	A	I	H	C	V	V	I	A	A	I	I	A
L	N	H	K	S	T	S	T	F	F	A	I	H	C	V	V	I	A	A	I	I	A
M	D	P	R	R	I	G	I	S	L	V	M	Y	R	I	T	V	V	S	T	F	V

														wchA								
2	3	5	6	8	10	13	31	40	56	104	105	107	108	134	135	142	154	155	175	203	259	270
S	E	L	A	P	V	V	T	V	R	L	L	V	F	V	F	H	N	E	Y	E	M	S
N	G	I	V	S	L	I	A	A	Q	T	V	A	L	I	L	R	N	E	C	K	V	A
S	E	L	A	P	V	V	T	V	R	L	L	V	F	V	F	H	D	D	Y	E	V	S

gct		wcjE																		
71	111	30	76	102	114	121	122	145	154	159	182	185	193	196	200	203	205	234	287
S	I	S	H	L	H	R	Y	I	F	I	V	C	V	N	L	F	F	S	S
S	I	S	Y	L	H	R	Y	I	F	I	I	F	I	D	L	F	F	I	S
S	I	S	Y	L	H	R	Y	I	F	I	I	F	I	D	L	F	F	I	S
S	I	S	Y	L	H	R	Y	I	F	I	I	F	I	D	L	F	F	I	S
S	I	S	Y	L	H	R	Y	I	F	I	I	F	I	D	L	F	F	I	S
S	I	S	Y	L	H	R	Y	I	F	I	I	F	I	D	L	F	F	I	S
S	I	S	Y	L	H	R	Y	I	F	I	I	F	I	D	L	F	F	I	S
S	I	S	Y	L	H	R	Y	I	F	I	I	F	I	D	L	F	F	I	S
S	I	S	Y	L	H	R	Y	I	F	I	I	F	I	D	L	F	F	I	S
S	I	S	Y	L	H	R	Y	I	F	I	I	F	I	D	L	F	F	I	S
S	I	S	Y	L	H	R	Y	I	F	I	I	F	I	D	L	F	F	I	S
S	I	S	Y	L	H	R	Y	I	F	I	I	F	I	D	L	F	F	I	S
S	I	S	Y	L	H	R	Y	I	F	I	I	F	I	D	L	F	F	I	S
S	I	S	H	L	H	R	Y	I	F	I	V	C	V	N	L	F	F	S	S
S	I	S	Y	L	H	R	Y	I	F	I	I	F	I	D	L	F	F	I	S
S	I	S	Y	L	H	R	Y	I	F	I	I	F	I	D	L	F	F	I	S
S	I	S	Y	L	H	R	Y	I	F	I	I	F	I	D	L	F	F	I	S
S	I	S	Y	L	H	R	Y	I	F	I	I	F	I	D	L	F	F	I	S
S	I	S	Y	L	H	R	Y	I	F	I	I	F	I	D	L	F	F	I	S
S	I	S	Y	L	H	R	Y	I	F	I	I	F	I	D	L	F	F	I	S
S	I	S	Y	L	H	R	Y	I	F	I	I	F	I	D	L	F	F	I	S
S	I	S	Y	L	H	R	Y	I	F	I	I	F	I	D	L	F	F	I	S
S	I	S	Y	L	H	R	Y	I	F	I	I	F	I	D	L	F	F	I	S
S	I	S	Y	L	H	R	Y	I	F	I	I	F	I	D	L	F	F	I	S
S	I	S	Y	L	H	R	Y	I	F	I	I	F	I	D	L	F	F	I	S
S	I	S	Y	L	H	R	Y	I	F	I	I	F	I	D	L	F	F	I	S
S	I	S	Y	L	H	R	Y	I	F	I	I	F	I	D	L	F	F	I	S
S	I	S	Y	L	H	R	Y	I	F	I	I	F	I	D	L	F	F	I	S
S	I	S	Y	L	H	R	Y	I	F	I	I	F	I	D	L	F	F	I	S
S	I	S	Y	L	H	R	Y	I	F	I	I	F	I	D	L	F	F	I	S
S	I	S	Y	L	H	R	Y	I	F	I	I	F	I	D	L	F	F	I	S
S	I	S	Y	L	H	R	Y	I	F	I	I	F	I	D	L	F	F	I	S
S	I	S	Y	L	H	R	Y	I	F	I	I	F	I	D	L	F	F	I	S
S	I	S	Y	L	H	R	Y	I	F	I	I	F	I	D	L	F	F	I	S
S	I	S	Y	L	H	R	Y	I	F	I	I	F	I	D	L	F	F	I	S
S	I	S	Y	L	H	R	Y	I	F	I	I	F	I	D	L	F	F	I	S
S	I	S	Y	L	H	R	Y	I	F	I	I	F	I	D	L	F	F	I	S
S	I	S	Y	L	H	R	Y	I	F	I	I	F	I	D	L	F	F	I	S
S	I	S	Y	L	H	R	Y	I	F	I	I	F	I	D	L	F	F	I	S
S	I	S	Y	L	H	R	Y	I	F	I	I	F	I	D	L	F	F	I	S
S	I	S	Y	L	H	R	Y	I	F	I	I	F	I	D	L	F	F	I	S
S	I	S	Y	L	H	R	Y	I	F	I	I	F	I	D	L	F	F	I	S
S	I	S	Y	L	H	R	Y	I	F	I	I	F	I	D	L	F	F	I	S
S	I	S	Y	L	H	R	Y	I	F	I	I	F	I	D	L	F	F	I	S
S	I	S	Y	L	H	R	Y	I	F	I	I	F	I	D	L	F	F	I	S
S	I	S	Y	L	H	R	Y	I	F	I	I	F	I	D	L	F	F	I	S
S	I	S	Y	L	H	R	Y	I	F	I	I	F	I	D	L	F	F	I	S
S	I	S	Y	L	H	R	Y	I	F	I	I	F	I	D	L	F	F	I	S
S	I	S	Y	L	H	R	Y	I	F	I	I	F	I	D	L	F	F	I	S
S	I	S	Y	L	H	R	Y	I	F	I	I	F	I	D	L	F	F	I	S
S	I	S	Y	L	H	R	Y	I	F	I	I	F	I	D	L	F	F	I	S
S	I	S	Y	L	H	R	Y	I	F	I	I	F	I	D	L	F	F	I	S
S	I	S	Y	L	H	R	Y	I	F	I	I	F	I	D	L	F	F	I	S
S	I	S	Y	L	H	R	Y	I	F	I	I	F	I	D	L	F	F	I	S
S	I	S	Y	L	H	R	Y	I	F	I	I	F	I	D	L	F	F	I	S
S	I	S	Y	L	H	R	Y	I	F	I	I	F	I	D	L	F	F	I	S
S	I	S	Y	L	H	R	Y	I	F	I	I	F	I	D	L	F	F	I	S
S	I	S	Y	L	H	R	Y	I	F	I	I	F	I	D	L	F	F	I	S
S	I	S	Y	L	H	R	Y	I	F	I	I	F	I	D	L	F	F	I	S
S	I	S	Y	L	H	R	Y	I	F	I	I	F	I	D	L	F	F	I	S
S	I	S	Y	L	H	R	Y	I	F	I	I	F	I	D	L	F	F	I	S
S	I	S	Y	L	H	R	Y	I	F	I	I	F	I	D	L	F	F	I	S
S	I	S	Y	L	H	R	Y	I	F	I	I	F	I	D	L	F	F	I	S

S	I	S	Y	L	H	R	Y	I	F	I	I	F	I	D	L	F	F	I	S
S	I	S	Y	L	H	R	Y	I	F	I	I	F	I	D	L	F	F	I	S
S	I	S	Y	L	H	R	Y	I	F	I	I	F	I	D	L	F	F	I	S
S	I	S	Y	L	H	R	Y	I	F	I	I	F	I	D	L	F	F	I	S
S	I	S	Y	L	H	R	Y	I	F	I	I	F	I	D	L	F	F	I	S
S	I	S	Y	L	H	R	Y	I	F	I	I	F	I	D	L	F	F	I	S
S	I	S	Y	L	H	R	Y	I	F	I	I	F	I	D	L	F	F	I	S
S	I	S	Y	L	H	R	Y	I	F	I	I	F	I	D	L	F	F	I	S
S	I	S	Y	L	H	R	Y	I	F	I	I	F	I	D	L	F	F	I	S
S	I	S	Y	L	H	R	Y	I	F	I	I	F	I	D	L	F	F	I	S
S	I	S	Y	L	H	R	Y	I	F	I	I	F	I	D	L	F	F	I	S
S	I	S	Y	L	H	R	Y	I	F	I	I	F	I	D	L	F	F	I	S
S	I	S	Y	L	H	R	Y	I	F	I	I	F	I	D	L	F	F	I	S
S	I	S	Y	L	H	R	Y	I	F	I	I	F	I	D	L	F	F	I	S
S	I	S	Y	L	H	R	Y	I	F	I	I	F	I	D	L	F	F	I	S
S	I	S	Y	L	H	R	Y	I	F	I	I	F	I	D	L	F	F	I	S
S	I	S	Y	L	H	R	Y	I	F	I	I	F	I	D	L	F	F	I	N
S	I	S	Y	L	H	R	Y	I	F	I	I	F	I	D	L	F	F	I	S
S	I	S	H	L	H	R	Y	I	F	I	V	C	V	N	L	F	F	S	S
S	I	S	Y	L	H	R	Y	I	F	I	I	F	I	D	L	F	F	I	S
S	I	S	Y	L	H	R	Y	I	F	I	I	F	I	D	L	F	F	I	S
S	I	S	Y	L	H	R	Y	I	F	I	I	F	I	D	L	F	F	I	S
S	I	S	Y	L	H	R	Y	I	F	I	I	F	I	D	L	F	F	I	S
S	I	S	Y	L	H	R	Y	I	F	I	I	F	I	D	L	F	F	I	S
S	I	S	Y	L	H	R	Y	I	F	I	I	F	I	D	L	F	F	I	S
S	I	S	Y	L	H	R	Y	I	F	I	I	F	I	D	L	F	F	I	S
S	I	S	Y	L	H	R	Y	I	F	I	I	F	I	D	L	F	F	I	S
S	I	S	Y	L	H	R	Y	I	F	I	I	F	I	D	L	F	F	I	S
S	I	S	Y	L	H	R	Y	I	F	I	I	F	I	D	L	F	F	I	S
S	I	S	Y	L	H	R	Y	I	F	I	I	F	I	D	L	F	F	I	S
S	I	S	Y	L	H	R	Y	I	F	I	I	F	I	D	L	F	F	I	S
S	I	S	Y	L	H	R	Y	I	F	I	I	F	I	D	L	F	F	I	S
S	I	S	Y	L	H	R	Y	I	F	I	I	F	I	D	L	F	F	I	S
S	I	S	Y	L	H	R	Y	I	F	I	I	F	I	D	L	F	F	I	S
S	I	S	Y	L	H	R	Y	I	F	I	I	F	I	D	L	F	F	I	S
S	I	S	Y	L	H	R	Y	I	F	I	I	F	I	D	L	F	F	I	S
S	I	S	Y	L	H	R	Y	I	F	I	I	F	I	D	L	F	F	I	S
S	I	S	Y	L	H	R	Y	I	F	I	I	F	I	D	L	F	F	I	S
S	I	S	Y	L	H	R	Y	I	F	I	I	F	I	D	L	F	F	I	S
S	I	S	Y	L	H	R	Y	I	F	I	I	F	I	D	L	F	F	I	S
S	I	S	Y	L	H	R	Y	I	F	I	I	F	I	D	L	F	F	I	S
S	I	S	Y	L	H	R	Y	I	F	I	I	F	I	D	L	F	F	I	S
S	I	S	Y	L	H	R	Y	I	F	I	I	F	I	D	L	F	F	I	S
S	I	S	Y	L	H	R	Y	I	F	I	I	F	I	D	L	F	F	I	S
S	I	S	Y	L	H	R	Y	I	F	I	I	F	I	D	L	F	F	I	S
S	I	S	Y	L	H	R	Y	I	F	I	I	F	I	D	L	F	F	I	S
S	I	S	H	L	H	R	Y	I	F	I	V	C	V	N	L	F	F	S	S
S	I	S	Y	L	H	R	Y	I	F	I	I	F	I	D	L	F	F	I	S
S	I	S	Y	L	H	R	Y	I	F	I	I	F	I	D	L	F	F	I	S
S	I	S	Y	L	H	R	Y	I	F	I	I	F	I	D	L	F	F	I	S
S	I	S	Y	L	H	R	Y	I	F	I	I	F	I	D	L	F	F	I	S
S	I	S	Y	L	H	R	Y	I	F	I	I	F	I	D	L	F	F	I	S
S	I	S	Y	L	H	R	Y	I	F	I	I	F	I	D	L	F	F	I	S
S	I	S	Y	L	H	R	Y	I	F	I	I	F	I	D	L	F	F	I	S
S	I	S	Y	L	H	R	Y	I	F	I	I	F	I	D	L	F	F	I	S
S	I	S	Y	L	H	R	Y	I	F	I	I	F	I	D	L	F	F	I	S
S	I	S	Y	L	H	R	Y	I	F	I	I	F	I	D	L	F	F	I	S
S	I	S	Y	L	H	R	Y	I	F	I	I	F	I	D	L	F	F	I	S
S	I	S	Y	L	H	R	Y	I	F	I	I	F	I	D	L	F	F	I	S
S	I	S	Y	L	H	R	Y	I	F	I	I	F	I	D	L	F	F	I	S
S	I	S	Y	L	H	R	Y	I	F	I	I	F	I	D	L	F	F	I	S
S	I	S	Y	L	H	R	Y	I	F	I	I	F	I	D	L	F	F	I	S
S	I	S	Y	L	H	R	Y	I	F	I	I	F	I	D	L	F	F	I	S
S	I	S	Y	L	H	R	Y	I	F	I	I	F	I	D	L	F	F	I	S
S	I	S	Y	L	H	R	Y	I	F	I	I	F	I	D	L	F	F	I	S
S	I	S	Y	L	H	R	Y	I	F	I	I	F	I	D	L	F	F	I	S
S	I	S	Y	L	H	R	Y	I	F	I	I	F	I	D	L	F	F	I	S

S	I
S	I
S	I
S	I
S	I
S	I
S	I
S	I
S	I
S	I
S	I
S	I
S	I
S	I
S	I
S	I
S	I
S	I
S	I
S	I
S	I
S	I
S	I
S	I
S	I
S	I
S	I
S	I
S	I
S	I
S	I
S	I
S	I
S	I
S	I
S	I
S	I
P	V

S	Y	L	H	R	Y	I	F	I	I	F	I	D	L	F	F	I	S
S	Y	L	H	R	Y	I	F	I	I	F	I	D	L	F	F	I	S
S	Y	L	H	R	Y	I	F	I	I	F	I	D	L	F	F	I	S
S	Y	L	H	R	Y	I	F	I	I	F	I	D	L	F	F	I	S
S	Y	L	H	R	Y	I	F	I	I	F	I	D	L	F	F	I	S
S	Y	L	H	R	Y	I	F	I	I	F	I	D	L	F	F	I	S
S	Y	L	H	R	Y	I	F	I	I	F	I	D	L	F	F	I	S
S	Y	L	H	R	Y	I	F	I	I	F	I	D	L	F	F	I	S
S	Y	L	H	R	Y	I	F	I	I	F	I	D	L	F	F	I	S
S	Y	L	H	R	Y	I	F	I	I	F	I	D	L	F	F	I	S
S	Y	L	H	R	Y	I	F	I	I	F	I	D	L	F	F	I	S
S	Y	L	H	R	Y	I	F	I	I	F	I	D	L	F	F	I	S
S	Y	L	H	R	Y	I	F	I	I	F	I	D	L	F	F	I	S
S	Y	L	H	R	Y	I	F	I	I	F	I	D	L	F	F	I	S
S	Y	L	H	R	Y	I	F	I	I	F	I	D	L	F	F	I	S
S	Y	L	H	R	Y	I	F	I	I	F	I	D	L	F	F	I	S
S	Y	L	H	R	Y	I	F	I	I	F	I	D	L	F	F	I	S
S	Y	L	H	R	Y	I	F	I	I	F	I	D	L	F	F	I	S
S	Y	L	H	R	Y	I	F	I	I	F	I	D	L	F	F	I	S
S	Y	L	H	R	Y	I	F	I	I	F	I	D	L	F	F	I	S
S	Y	L	H	R	Y	I	F	I	I	F	I	D	L	F	F	I	S
S	Y	L	H	R	Y	I	F	I	I	F	I	D	L	F	F	I	S
S	Y	L	H	R	Y	I	F	I	I	F	I	D	L	F	F	I	S
S	Y	L	H	R	Y	I	F	I	I	F	I	D	L	F	F	I	S
S	Y	L	H	R	Y	I	F	I	I	F	I	D	L	F	F	I	S
S	Y	L	H	R	Y	I	F	I	I	F	I	D	L	F	F	I	S
S	Y	L	H	R	Y	I	F	I	I	F	I	D	L	F	F	I	S
S	Y	L	H	R	Y	I	F	I	I	F	I	D	L	F	F	I	S
S	Y	L	H	R	Y	I	F	I	I	F	I	D	L	F	F	I	S
S	Y	L	H	R	Y	I	F	I	I	F	I	D	L	F	F	I	S
S	Y	L	H	R	Y	I	F	I	I	F	I	D	L	F	F	I	S
S	Y	L	H	R	Y	I	F	I	I	F	I	D	L	F	F	I	S
S	Y	L	H	R	Y	I	F	I	I	F	I	D	L	F	F	I	S
S	Y	L	H	R	Y	I	F	I	I	F	I	D	L	F	F	I	S
S	Y	L	H	R	Y	I	F	I	I	F	I	D	L	F	F	I	S
S	Y	L	H	R	Y	I	F	I	I	F	I	D	L	F	F	I	S
S	Y	L	H	R	Y	I	F	I	I	F	I	D	L	F	F	I	S
Y	H	V	F	K	F	V	L	T	I	L	I	D	F	I	V	S	S

285	296	308	323	352	409	449	451
V	F	H	D	H	V	L	K
L	I	Q	A	R	I	F	R
V	F	H	D	H	V	L	K

										wchJ			
36	38	41	43	44	46	47	51	58	66	69	70	73	79
E	T	I	G	N	T	F	H	L	V	F	N	R	V
A	A	V	S	E	V	V	Y	I	F	Y	K	K	L
E	T	I	G	N	T	F	H	L	V	F	N	R	V

87	94	100	116	120	127	138
V	L	A	L	I	K	V
I	I	S	I	L	R	I
V	L	A	L	I	K	V

6	14	20	25	27	28	31	32	39	41	43	45	48	50	51
I	N	V	G	G	F	D	D	F	D	E	V	Q	K	N
V	D	F	E	N	L	E	E	Y	N	V	K	K	E	K
I	N	V	G	G	F	D	D	F	D	E	V	Q	K	N

wchK																	
52	56	57	60	61	63	66	67	69	80	86	93	106	112	144	145	149	153
L	D	E	R	Y	D	N	I	I	V	I	L	E	L	T	C	K	T
I	E	K	Q	F	E	D	T	V	I	T	Q	N	S	A	N	Q	I
L	D	E	R	Y	D	N	I	I	V	I	L	E	L	T	C	K	T

41	42	52	71
S	P	C	V
P	L	Y	I
S	P	C	V

wcyK											
72	77	78	104	157	167	204	212	220	288	303	340
Y	I	I	V	K	R	C	K	I	E	G	K
Y	L	M	I	E	K	R	R	L	K	E	R
C	I	I	V	K	R	C	K	I	E	G	K

wcwC									
37	98	127	131	137	138	139	144	151	156
R	R	K	L	A	N	Q	E	N	E
K	K	N	I	S	S	M	K	K	K
R	R	K	L	A	N	Q	E	N	E

205	212	218	219
R	T	D	Q
I	A	N	K
R	T	D	Q

17	28	47	54	58	59	74	88	91	94	97	99	119	123	124	131	132	136
S	K	E	Q	R	D	I	V	N	K	E	K	S	T	S	N	L	I
P	R	Q	K	K	N	V	I	K	L	D	T	A	S	F	D	S	V
S	K	E	Q	R	D	I	V	N	K	E	K	S	T	S	N	L	I

wcrL																				
137	140	141	154	155	158	162	165	166	168	170	171	189	191	205	212	214	215	217	228	229
E	L	S	P	V	R	T	D	C	V	N	E	G	E	S	R	Q	M	S	I	L
K	I	N	Q	I	Q	V	N	S	I	S	Q	E	A	N	M	R	V	R	V	S
E	L	S	P	V	R	T	D	C	V	N	E	G	E	S	R	Q	M	S	I	L

43
N
K
N

wzy																					
44	54	83	89	95	97	132	141	151	165	166	206	213	221	234	286	297	301	310	342	343	345
G	L	I	V	V	L	P	V	I	V	I	V	V	R	V	L	K	V	V	S	T	S
E	S	M	L	D	V	A	I	V	I	L	A	I	K	I	V	E	I	I	P	A	G
G	L	M	V	V	L	P	V	I	V	I	V	V	R	V	L	K	V	V	S	T	S

wcwT		
26	131	158
G	I	Q
S	A	R
S	A	R

wcwU														
43	84	141	193	201	235	242	253	282	284	288	290	332	347	351
N	V	S	P	N	E	N	A	D	N	T	L	L	L	N
D	S	N	T	D	G	H	V	N	K	I	M	F	M	D
D	S	N	T	D	G	H	V	N	K	I	M	F	M	D

5	11	12
H	K	S
P	R	R
P	R	R

wzx																		gct		
104	129	165	166	170	181	234	287	298	309	310	314	316	398	444	457	464	467	71	111	30
T	S	T	F	F	A	I	H	C	V	V	I	A	A	I	I	A	L	S	I	S
I	G	I	S	L	V	M	Y	R	I	T	V	V	S	T	F	V	V	P	V	Y
I	G	I	S	L	V	M	Y	R	I	T	V	V	S	T	F	V	L	P	V	Y

wcjE													
102	114	121	122	145	154	169	182	185	193	196	200	203	205
L	H	R	Y	I	F	I	V	C	V	N	L	F	F
V	F	K	F	V	L	T	I	L	I	D	F	I	V
V	F	K	F	V	L	T	I	L	I	D	F	I	V

SEROTYPE 11X ALL ISOLATES (11A REFERENCE)

					wzg						
					9	80	123	162	328	403	463
		11A reference			G	V	E	N	I	D	N
32689	N/A	SMRU637	99	11nov	R	L	D	D	V	N	D
32928	N/A	SMRU296	99	11nov	R	L	D	D	V	N	D
32929	N/A	SMRU297	99	11nov	R	L	D	D	V	N	D
33114	N/A	SMRU775	99	11nov	R	L	D	D	V	N	D
33872	N/A	SMRU271	99	11nov	R	L	D	D	V	N	D
33916	N/A	SMRU173	99	11nov	R	L	D	D	V	N	D
34118	N/A	SMRU780	99	11nov	R	L	D	D	V	N	D
34158	N/A	SMRU1962	99	11nov	R	L	D	D	V	N	D
40117	N/A	SMRU75	99	11nov	R	L	D	D	V	N	D
40158	N/A	SMRU272	99	11nov	R	L	D	D	V	N	D
40169	N/A	SMRU379	99	11nov	R	L	D	D	V	N	D
40186	N/A	SMRU453	99	11nov	R	L	D	D	V	N	D
40195	N/A	SMRU183	99	11nov	R	L	D	D	V	N	D
40236	N/A	SMRU1070	99	11nov	R	L	D	D	V	N	D
40239	N/A	SMRU1124	99	11nov	R	L	D	D	V	N	D
40599	N/A	SMRU3055	99	11nov	R	L	D	D	V	N	D
44871	N/A	SMRU1068	99	11nov	R	L	D	D	V	N	D
44984	N/A	SMRU2333	99	11nov	R	L	D	D	V	N	D
55133	N/A	SMRU1118	99	11nov	R	L	D	D	V	N	D
55160	N/A	SMRU2366	99	11nov	R	L	D	D	V	N	D
55963	N/A	SMRU2945	99	11nov	R	L	D	D	V	N	D
82100	N/A	SMRU60	99	11nov	R	L	D	D	V	N	D
82101	N/A	SMRU61	99	11nov	R	L	D	D	V	N	D
82137	N/A	SMRU475	99	11nov	R	L	D	D	V	N	D
82224	N/A	SMRU249	99	11nov	R	L	D	D	V	N	D
82349	N/A	SMRU1896	99	11nov	R	L	D	D	V	N	D
82424	N/A	SMRU2151	99	11nov	R	L	D	D	V	N	D
82480	N/A	SMRU2406	99	11nov	R	L	D	D	V	N	D
82577	N/A	SMRU2600	99	11nov	R	L	D	D	V	N	D
82729	N/A	SMRU3001	99	11nov	R	L	D	D	V	N	D
82736	N/A	SMRU2246	99	11nov	R	L	D	D	V	N	D
83323	N/A	SMRU1541	99	11nov	R	L	D	D	V	N	D
83492	N/A	SMRU2311	99	11nov	R	L	D	D	V	N	D
83496	N/A	SMRU2335	99	11nov	R	L	D	D	V	N	D
109675	N/A	SMRU84	99	11nov	R	L	D	D	V	N	D
109745	N/A	SMRU553	99	11nov	R	L	D	D	V	N	D
109746	N/A	SMRU554	99	11nov	R	L	D	D	V	N	D
109845	N/A	SMRU1441	99	11nov	R	L	D	D	V	N	D
109957	N/A	SMRU2420	99	11nov	R	L	D	D	V	N	D
109992	N/A	SMRU3098	99	11nov	R	L	D	D	V	N	D
109996	N/A	SMRU3105	99	11nov	R	L	D	D	V	N	D
111417	N/A	SMRU2968	99	11nov	R	L	D	D	V	N	D
111466	N/A	SMRU1905	99	11nov	R	L	D	D	V	N	D
111531	N/A	SMRU1213	99	11nov	R	L	D	D	V	N	D
111560	N/A	SMRU200	99	11nov	R	L	D	D	V	N	D
113126	N/A	SMRU1904	99	11nov	R	L	D	D	V	N	D
113755	N/A	SMRU488	99	11nov	R	L	D	D	V	N	D
115501	N/A	SMRU199	99	11nov	R	L	D	D	V	N	D
115518	N/A	SMRU767	99	11nov	R	L	D	D	V	N	D

SEROTYPE 11X ALL ISOLATES (ALL SEROGROUP 11 REFERENCES)

					9	51	54	61	65	80	91
		11A reference			G	V	L	V	L	V	F
		11B reference			G	A	F	V	S	V	L
		11C reference			G	A	F	I	S	V	L
		11D reference			G	V	L	V	L	V	F
		11F reference			G	V	L	V	L	L	F
32689	N/A	SMRU637	99	11nov	R	V	L	V	L	L	F

32928	N/A	SMRU296	99	11nov	R	V	L	V	L	L	F
32929	N/A	SMRU297	99	11nov	R	V	L	V	L	L	F
33114	N/A	SMRU775	99	11nov	R	V	L	V	L	L	F
33872	N/A	SMRU271	99	11nov	R	V	L	V	L	L	F
33916	N/A	SMRU173	99	11nov	R	V	L	V	L	L	F
34118	N/A	SMRU780	99	11nov	R	V	L	V	L	L	F
34158	N/A	SMRU1962	99	11nov	R	V	L	V	L	L	F
40117	N/A	SMRU75	99	11nov	R	V	L	V	L	L	F
40158	N/A	SMRU272	99	11nov	R	V	L	V	L	L	F
40169	N/A	SMRU379	99	11nov	R	V	L	V	L	L	F
40186	N/A	SMRU453	99	11nov	R	V	L	V	L	L	F
40195	N/A	SMRU183	99	11nov	R	V	L	V	L	L	F
40236	N/A	SMRU1070	99	11nov	R	V	L	V	L	L	F
40239	N/A	SMRU1124	99	11nov	R	V	L	V	L	L	F
40599	N/A	SMRU3055	99	11nov	R	V	L	V	L	L	F
44871	N/A	SMRU1068	99	11nov	R	V	L	V	L	L	F
44984	N/A	SMRU2333	99	11nov	R	V	L	V	L	L	F
55133	N/A	SMRU1118	99	11nov	R	V	L	V	L	L	F
55160	N/A	SMRU2366	99	11nov	R	V	L	V	L	L	F
55963	N/A	SMRU2945	99	11nov	R	V	L	V	L	L	F
82100	N/A	SMRU60	99	11nov	R	V	L	V	L	L	F
82101	N/A	SMRU61	99	11nov	R	V	L	V	L	L	F
82137	N/A	SMRU475	99	11nov	R	V	L	V	L	L	F
82224	N/A	SMRU249	99	11nov	R	V	L	V	L	L	F
82349	N/A	SMRU1896	99	11nov	R	V	L	V	L	L	F
82424	N/A	SMRU2151	99	11nov	R	V	L	V	L	L	F
82480	N/A	SMRU2406	99	11nov	R	V	L	V	L	L	F
82577	N/A	SMRU2600	99	11nov	R	V	L	V	L	L	F
82729	N/A	SMRU3001	99	11nov	R	V	L	V	L	L	F
82736	N/A	SMRU2246	99	11nov	R	V	L	V	L	L	F
83323	N/A	SMRU1541	99	11nov	R	V	L	V	L	L	F
83492	N/A	SMRU2311	99	11nov	R	V	L	V	L	L	F
83496	N/A	SMRU2335	99	11nov	R	V	L	V	L	L	F
109675	N/A	SMRU84	99	11nov	R	V	L	V	L	L	F
109745	N/A	SMRU553	99	11nov	R	V	L	V	L	L	F
109746	N/A	SMRU554	99	11nov	R	V	L	V	L	L	F
109845	N/A	SMRU1441	99	11nov	R	V	L	V	L	L	F
109957	N/A	SMRU2420	99	11nov	R	V	L	V	L	L	F
109992	N/A	SMRU3098	99	11nov	R	V	L	V	L	L	F
109996	N/A	SMRU3105	99	11nov	R	V	L	V	L	L	F
111417	N/A	SMRU2968	99	11nov	R	V	L	V	L	L	F
111466	N/A	SMRU1905	99	11nov	R	V	L	V	L	L	F
111531	N/A	SMRU1213	99	11nov	R	V	L	V	L	L	F
111560	N/A	SMRU200	99	11nov	R	V	L	V	L	L	F
113126	N/A	SMRU1904	99	11nov	R	V	L	V	L	L	F
113755	N/A	SMRU488	99	11nov	R	V	L	V	L	L	F
115501	N/A	SMRU199	99	11nov	R	V	L	V	L	L	F
115518	N/A	SMRU767	99	11nov	R	V	L	V	L	L	F

469	472	478
S	T	L
V	A	M
V	A	M
V	A	M
V	A	M
V	A	M
V	A	M
V	A	M
V	A	M
V	A	M
V	A	M
V	A	M
V	A	M
V	A	M
V	A	M
V	A	M
V	A	M
V	A	M
V	A	M
V	A	M
V	A	M
V	A	M
V	A	M
V	A	M
V	A	M
V	A	M
V	A	M
V	A	M
V	A	M
V	A	M
V	A	M
V	A	M
V	A	M
V	A	M
V	A	M
V	A	M
V	A	M
V	A	M
V	A	M
V	A	M
V	A	M
V	A	M
V	A	M
V	A	M
V	A	M
V	A	M
V	A	M
V	A	M
V	A	M
V	A	M

wzh										
27	34	94	101	123	170	190	203	205	218	235
T	V	N	N	I	S	R	V	S	S	R
A	M	K	D	S	P	Q	L	G	T	Q
A	M	K	D	S	P	Q	L	G	T	Q
A	M	K	D	S	P	Q	L	G	T	Q
A	M	K	D	S	P	Q	L	G	T	Q
A	M	K	D	S	P	Q	L	G	T	Q
A	M	K	D	S	P	Q	L	G	T	Q
A	M	K	D	S	P	Q	L	G	T	Q
A	M	K	D	S	P	Q	L	G	T	Q
A	M	K	D	S	P	Q	L	G	T	Q
A	M	K	D	S	P	Q	L	G	T	Q
A	M	K	D	S	P	Q	L	G	T	Q
A	M	K	D	S	P	Q	L	G	T	Q
A	M	K	D	S	P	Q	L	G	T	Q
A	M	K	D	S	P	Q	L	G	T	Q
A	M	K	D	S	P	Q	L	G	T	Q
A	M	K	D	S	P	Q	L	G	T	Q
A	M	K	D	S	P	Q	L	G	T	Q
A	M	K	D	S	P	Q	L	G	T	Q
A	M	K	D	S	P	Q	L	G	T	Q
A	M	K	D	S	P	Q	L	G	T	Q
A	M	K	D	S	P	Q	L	G	T	Q
A	M	K	D	S	P	Q	L	G	T	Q
A	M	K	D	S	P	Q	L	G	T	Q
A	M	K	D	S	P	Q	L	G	T	Q
A	M	K	D	S	P	Q	L	G	T	Q
A	M	K	D	S	P	Q	L	G	T	Q
A	M	K	D	S	P	Q	L	G	T	Q
A	M	K	D	S	P	Q	L	G	T	Q
A	M	K	D	S	P	Q	L	G	T	Q
A	M	K	D	S	P	Q	L	G	T	Q
A	M	K	D	S	P	Q	L	G	T	Q
A	M	K	D	S	P	Q	L	G	T	Q
A	M	K	D	S	P	Q	L	G	T	Q
A	M	K	D	S	P	Q	L	G	T	Q
A	M	K	D	S	P	Q	L	G	T	Q
A	M	K	D	S	P	Q	L	G	T	Q
A	M	K	D	S	P	Q	L	G	T	Q
A	M	K	D	S	P	Q	L	G	T	Q
A	M	K	D	S	P	Q	L	G	T	Q
A	M	K	D	S	P	Q	L	G	T	Q
A	M	K	D	S	P	Q	L	G	T	Q
A	M	K	D	S	P	Q	L	G	T	Q
A	M	K	D	S	P	Q	L	G	T	Q
A	M	K	D	S	P	Q	L	G	T	Q
A	M	K	D	S	P	Q	L	G	T	Q
A	M	K	D	S	P	Q	L	G	T	Q
A	M	K	D	S	P	Q	L	G	T	Q
A	M	K	D	S	P	Q	L	G	T	Q

14	15	21	24	27	29	41
S	L	Y	I	L	A	T
L	F	R	M	I	A	A
L	F	R	M	I	A	A
L	F	R	M	I	A	A
L	F	R	M	I	A	A
L	F	R	M	I	A	A
L	F	R	M	I	A	A
L	F	R	M	I	A	A
L	F	R	M	I	A	A
L	F	R	M	I	A	A
L	F	R	M	I	A	A
L	F	R	M	I	A	A
L	F	R	M	I	A	A
L	F	R	M	I	A	A
L	F	R	M	I	A	A
L	F	R	M	I	A	A
L	F	R	M	I	A	A
L	F	R	M	I	A	A
L	F	R	M	I	A	A
L	F	R	M	I	A	A
L	F	R	M	I	A	A
L	F	R	M	I	A	A
L	F	R	M	I	V	A
L	F	R	M	I	V	A
L	F	R	M	I	A	A
L	F	R	M	I	A	A
L	F	R	M	I	A	A
L	F	R	M	I	A	A
L	F	R	M	I	A	A
L	F	R	M	I	A	A
L	F	R	M	I	A	A
L	F	R	M	I	A	A
L	F	R	M	I	A	A
L	F	R	M	I	A	A
L	F	R	M	I	A	A
L	F	R	M	I	A	A
L	F	R	M	I	A	A
L	F	R	M	I	A	A
L	F	R	M	I	A	A
L	F	R	M	I	A	A
L	F	R	M	I	A	A
L	F	R	M	I	A	A
L	F	R	M	I	A	A
L	F	R	M	I	A	A
L	F	R	M	I	A	A
L	F	R	M	I	A	A
L	F	R	M	I	A	A
L	F	R	M	I	A	A
L	F	R	M	I	A	A
L	F	R	M	I	A	A

wzg																						
92	114	123	157	162	178	199	308	321	328	331	337	340	341	343	345	346	347	356	359	391	399	400
A	I	E	T	N	E	Y	N	M	I	H	S	H	G	F	F	P	V	Q	G	A	S	T
A	L	E	I	N	E	H	H	L	V	Y	T	A	N	K	Y	S	I	E	A	V	S	R
A	L	E	I	N	E	H	H	L	V	Y	T	A	N	K	Y	S	I	E	A	V	S	R
A	I	E	T	N	E	Y	N	M	I	H	S	H	G	F	F	P	V	Q	G	A	S	T
T	I	E	T	N	D	Y	N	L	V	H	S	H	G	F	F	P	V	Q	G	A	G	T
A	I	D	T	D	E	Y	N	M	V	H	S	H	G	F	F	P	V	Q	G	A	S	T

A	I	D	T	D	E	Y	N	M	V	H	S	H	G	F	F	P	V	Q	G	A	S	T
A	I	D	T	D	E	Y	N	M	V	H	S	H	G	F	F	P	V	Q	G	A	S	T
A	I	D	T	D	E	Y	N	M	V	H	S	H	G	F	F	P	V	Q	G	A	S	T
A	I	D	T	D	E	Y	N	M	V	H	S	H	G	F	F	P	V	Q	G	A	S	T
A	I	D	T	D	E	Y	N	M	V	H	S	H	G	F	F	P	V	Q	G	A	S	T
A	I	D	T	D	E	Y	N	M	V	H	S	H	G	F	F	P	V	Q	G	A	S	T
A	I	D	T	D	E	Y	N	M	V	H	S	H	G	F	F	P	V	Q	G	A	S	T
A	I	D	T	D	E	Y	N	M	V	H	S	H	G	F	F	P	V	Q	G	A	S	T
A	I	D	T	D	E	Y	N	M	V	H	S	H	G	F	F	P	V	Q	G	A	S	T
A	I	D	T	D	E	Y	N	M	V	H	S	H	G	F	F	P	V	Q	G	A	S	T
A	I	D	T	D	E	Y	N	M	V	H	S	H	G	F	F	P	V	Q	G	A	S	T
A	I	D	T	D	E	Y	N	M	V	H	S	H	G	F	F	P	V	Q	G	A	S	T
A	I	D	T	D	E	Y	N	M	V	H	S	H	G	F	F	P	V	Q	G	A	S	T
A	I	D	T	D	E	Y	N	M	V	H	S	H	G	F	F	P	V	Q	G	A	S	T
A	I	D	T	D	E	Y	N	M	V	H	S	H	G	F	F	P	V	Q	G	A	S	T
A	I	D	T	D	E	Y	N	M	V	H	S	H	G	F	F	P	V	Q	G	A	S	T
A	I	D	T	D	E	Y	N	M	V	H	S	H	G	F	F	P	V	Q	G	A	S	T
A	I	D	T	D	E	Y	N	M	V	H	S	H	G	F	F	P	V	Q	G	A	S	T
A	I	D	T	D	E	Y	N	M	V	H	S	H	G	F	F	P	V	Q	G	A	S	T
A	I	D	T	D	E	Y	N	M	V	H	S	H	G	F	F	P	V	Q	G	A	S	T
A	I	D	T	D	E	Y	N	M	V	H	S	H	G	F	F	P	V	Q	G	A	S	T
A	I	D	T	D	E	Y	N	M	V	H	S	H	G	F	F	P	V	Q	G	A	S	T
A	I	D	T	D	E	Y	N	M	V	H	S	H	G	F	F	P	V	Q	G	A	S	T
A	I	D	T	D	E	Y	N	M	V	H	S	H	G	F	F	P	V	Q	G	A	S	T
A	I	D	T	D	E	Y	N	M	V	H	S	H	G	F	F	P	V	Q	G	A	S	T
A	I	D	T	D	E	Y	N	M	V	H	S	H	G	F	F	P	V	Q	G	A	S	T
A	I	D	T	D	E	Y	N	M	V	H	S	H	G	F	F	P	V	Q	G	A	S	T
A	I	D	T	D	E	Y	N	M	V	H	S	H	G	F	F	P	V	Q	G	A	S	T
A	I	D	T	D	E	Y	N	M	V	H	S	H	G	F	F	P	V	Q	G	A	S	T
A	I	D	T	D	E	Y	N	M	V	H	S	H	G	F	F	P	V	Q	G	A	S	T
A	I	D	T	D	E	Y	N	M	V	H	S	H	G	F	F	P	V	Q	G	A	S	T
A	I	D	T	D	E	Y	N	M	V	H	S	H	G	F	F	P	V	Q	G	A	S	T
A	I	D	T	D	E	Y	N	M	V	H	S	H	G	F	F	P	V	Q	G	A	S	T
A	I	D	T	D	E	Y	N	M	V	H	S	H	G	F	F	P	V	Q	G	A	S	T
A	I	D	T	D	E	Y	N	M	V	H	S	H	G	F	F	P	V	Q	G	A	S	T
A	I	D	T	D	E	Y	N	M	V	H	S	H	G	F	F	P	V	Q	G	A	S	T
A	I	D	T	D	E	Y	N	M	V	H	S	H	G	F	F	P	V	Q	G	A	S	T
A	I	D	T	D	E	Y	N	M	V	H	S	H	G	F	F	P	V	Q	G	A	S	T
A	I	D	T	D	E	Y	N	M	V	H	S	H	G	F	F	P	V	Q	G	A	S	T
A	I	D	T	D	E	Y	N	M	V	H	S	H	G	F	F	P	V	Q	G	A	S	T
A	I	D	T	D	E	Y	N	M	V	H	S	H	G	F	F	P	V	Q	G	A	S	T
A	I	D	T	D	E	Y	N	M	V	H	S	H	G	F	F	P	V	Q	G	A	S	T
A	I	D	T	D	E	Y	N	M	V	H	S	H	G	F	F	P	V	Q	G	A	S	T
A	I	D	T	D	E	Y	N	M	V	H	S	H	G	F	F	P	V	Q	G	A	S	T
A	I	D	T	D	E	Y	N	M	V	H	S	H	G	F	F	P	V	Q	G	A	S	T
A	I	D	T	D	E	Y	N	M	V	H	S	H	G	F	F	P	V	Q	G	A	S	T
A	I	D	T	D	E	Y	N	M	V	H	S	H	G	F	F	P	V	Q	G	A	S	T
A	I	D	T	D	E	Y	N	M	V	H	S	H	G	F	F	P	V	Q	G	A	S	T

wzd													
60	76	87	103	109	151	180	182	189	193	194	217	225	225
N	T	L	I	T	I	T	V	A	I	T	M	D	F
D	S	F	M	A	V	S	F	V	S	A	I	N	L
D	S	L	M	A	V	S	F	V	S	A	I	N	L
D	S	L	M	A	V	S	F	V	S	A	I	N	L
D	S	L	M	A	V	S	F	V	S	A	I	N	L
D	S	L	M	A	V	S	F	V	S	A	I	N	L
D	S	L	M	A	V	S	F	V	S	A	I	N	L
D	S	L	M	A	V	S	F	V	S	A	I	N	L
D	S	L	M	A	V	S	F	V	S	A	I	N	L
D	S	L	M	A	V	S	F	V	S	A	I	N	L
D	S	L	M	A	V	S	F	V	S	A	I	N	L
D	S	L	M	A	V	S	F	V	S	A	I	N	L
D	S	L	M	A	V	S	F	V	S	A	I	N	L
D	S	L	M	A	V	S	F	V	S	A	I	N	L
D	S	L	M	A	V	S	F	V	S	A	I	N	L
D	S	L	M	A	V	S	F	V	S	A	I	N	L
D	S	L	M	A	V	S	F	V	S	A	I	N	L
D	S	L	M	A	V	S	F	V	S	A	I	N	L
D	S	L	M	A	V	S	F	V	S	A	I	N	L
D	S	L	M	A	V	S	F	V	S	A	I	N	L
D	S	L	M	A	V	S	F	V	S	A	I	N	L
D	S	L	M	A	V	S	F	V	S	A	I	N	L
D	S	L	M	A	V	S	F	V	S	A	I	N	L
D	S	L	M	A	V	S	F	V	S	A	I	N	L
D	S	L	M	A	V	S	F	V	S	A	I	N	L
D	S	L	M	A	V	S	F	V	S	A	I	N	L
D	S	L	M	A	V	S	F	V	S	A	I	N	L
D	S	L	M	A	V	S	F	V	S	A	I	N	L
D	S	L	M	A	V	S	F	V	S	A	I	N	L
D	S	L	M	A	V	S	F	V	S	A	I	N	L
D	S	L	M	A	V	S	F	V	S	A	I	N	L
D	S	L	M	A	V	S	F	V	S	A	I	N	L
D	S	L	M	A	V	S	F	V	S	A	I	N	L
D	S	L	M	A	V	S	F	V	S	A	I	N	L
D	S	L	M	A	V	S	F	V	S	A	I	N	L
D	S	L	M	A	V	S	F	V	S	A	I	N	L
D	S	L	M	A	V	S	F	V	S	A	I	N	L
D	S	L	M	A	V	S	F	V	S	A	I	N	L
D	S	L	M	A	V	S	F	V	S	A	I	N	L
D	S	L	M	A	V	S	F	V	S	A	I	N	L
D	S	L	M	A	V	S	F	V	S	A	I	N	L
D	S	L	M	A	V	S	F	V	S	A	I	N	L
D	S	L	M	A	V	S	F	V	S	A	I	N	L
D	S	L	M	A	V	S	F	V	S	A	I	N	L
D	S	L	M	A	V	S	F	V	S	A	I	N	L
D	S	L	M	A	V	S	F	V	S	A	I	N	L
D	S	L	M	A	V	S	F	V	S	A	I	N	L
D	S	L	M	A	V	S	F	V	S	A	I	N	L
D	S	L	M	A	V	S	F	V	S	A	I	N	L

9	12	13	14	45	50	62	72
V	E	L	A	A	T	R	A
A	D	F	V	L	S	H	G
A	D	F	V	L	S	H	G
A	D	F	V	L	S	H	G
A	D	F	V	L	S	H	G
A	D	F	V	L	S	H	G
A	D	F	V	L	S	H	G
A	D	F	V	L	S	H	G
A	D	F	V	L	S	H	G
A	D	F	V	L	S	H	G
A	D	F	V	L	S	H	G
A	D	F	V	L	S	H	G
A	D	F	V	L	S	H	G
A	D	F	V	L	S	H	G
A	D	F	V	L	S	H	G
A	D	F	V	L	S	H	G
A	D	F	V	L	S	H	G
A	D	F	V	L	S	H	G
A	D	F	V	L	S	H	G
A	D	F	V	L	S	H	G
A	D	F	V	L	S	H	G
A	D	F	V	L	S	H	G
A	D	F	V	L	S	H	G
A	D	F	V	L	S	H	G
A	D	F	V	L	S	H	G
A	D	F	V	L	S	H	G
A	D	F	V	L	S	H	G
A	D	F	V	L	S	H	G
A	D	F	V	L	S	H	G
A	D	F	V	L	S	H	G
A	D	F	V	L	S	H	G
A	D	F	V	L	S	H	G
A	D	F	V	L	S	H	G
A	D	F	V	L	S	H	G
A	D	F	V	L	S	H	G
A	D	F	V	L	S	H	G
A	D	F	V	L	S	H	G
A	D	F	V	L	S	H	G
A	D	F	V	L	S	H	G
A	D	F	V	L	S	H	G
A	D	F	V	L	S	H	G
A	D	F	V	L	S	H	G
A	D	F	V	L	S	H	G
A	D	F	V	L	S	H	G
A	D	F	V	L	S	H	G
A	D	F	V	L	S	H	G
A	D	F	V	L	S	H	G
A	D	F	V	L	S	H	G
A	D	F	V	L	S	H	G
A	D	F	V	L	S	H	G

403	407	420	424	431	442	445	459	460	463	469	472	478
D	D	N	A	N	T	T	V	M	N	S	T	L
D	N	N	A	T	R	T	M	M	N	S	T	L
D	N	N	A	T	R	T	M	M	N	S	T	L
D	D	N	A	N	T	T	V	M	N	S	T	L
N	D	D	T	K	T	M	V	L	D	V	A	M
N	D	N	A	N	T	T	V	M	D	V	A	M

27	29	34	93	94	101	119	123	130
T	A	V	K	N	N	H	I	I
T	A	V	N	N	N	R	S	I
T	A	V	N	N	N	R	S	I
T	A	V	K	N	N	H	I	I
A	S	V	K	K	D	H	S	V
A	A	M	K	K	D	H	S	I

N	D	N	A	N	T	T	V	M	D	V	A	M	A	A	M	K	K	D	H	S	I
N	D	N	A	N	T	T	V	M	D	V	A	M	A	A	M	K	K	D	H	S	I
N	D	N	A	N	T	T	V	M	D	V	A	M	A	A	M	K	K	D	H	S	I
N	D	N	A	N	T	T	V	M	D	V	A	M	A	A	M	K	K	D	H	S	I
N	D	N	A	N	T	T	V	M	D	V	A	M	A	A	M	K	K	D	H	S	I
N	D	N	A	N	T	T	V	M	D	V	A	M	A	A	M	K	K	D	H	S	I
N	D	N	A	N	T	T	V	M	D	V	A	M	A	A	M	K	K	D	H	S	I
N	D	N	A	N	T	T	V	M	D	V	A	M	A	A	M	K	K	D	H	S	I
N	D	N	A	N	T	T	V	M	D	V	A	M	A	A	M	K	K	D	H	S	I
N	D	N	A	N	T	T	V	M	D	V	A	M	A	A	M	K	K	D	H	S	I
N	D	N	A	N	T	T	V	M	D	V	A	M	A	A	M	K	K	D	H	S	I
N	D	N	A	N	T	T	V	M	D	V	A	M	A	A	M	K	K	D	H	S	I
N	D	N	A	N	T	T	V	M	D	V	A	M	A	A	M	K	K	D	H	S	I
N	D	N	A	N	T	T	V	M	D	V	A	M	A	A	M	K	K	D	H	S	I
N	D	N	A	N	T	T	V	M	D	V	A	M	A	A	M	K	K	D	H	S	I
N	D	N	A	N	T	T	V	M	D	V	A	M	A	A	M	K	K	D	H	S	I
N	D	N	A	N	T	T	V	M	D	V	A	M	A	A	M	K	K	D	H	S	I
N	D	N	A	N	T	T	V	M	D	V	A	M	A	A	M	K	K	D	H	S	I
N	D	N	A	N	T	T	V	M	D	V	A	M	A	A	M	K	K	D	H	S	I
N	D	N	A	N	T	T	V	M	D	V	A	M	A	A	M	K	K	D	H	S	I
N	D	N	A	N	T	T	V	M	D	V	A	M	A	A	M	K	K	D	H	S	I
N	D	N	A	N	T	T	V	M	D	V	A	M	A	A	M	K	K	D	H	S	I
N	D	N	A	N	T	T	V	M	D	V	A	M	A	A	M	K	K	D	H	S	I
N	D	N	A	N	T	T	V	M	D	V	A	M	A	A	M	K	K	D	H	S	I
N	D	N	A	N	T	T	V	M	D	V	A	M	A	A	M	K	K	D	H	S	I
N	D	N	A	N	T	T	V	M	D	V	A	M	A	A	M	K	K	D	H	S	I
N	D	N	A	N	T	T	V	M	D	V	A	M	A	A	M	K	K	D	H	S	I
N	D	N	A	N	T	T	V	M	D	V	A	M	A	A	M	K	K	D	H	S	I
N	D	N	A	N	T	T	V	M	D	V	A	M	A	A	M	K	K	D	H	S	I
N	D	N	A	N	T	T	V	M	D	V	A	M	A	A	M	K	K	D	H	S	I
N	D	N	A	N	T	T	V	M	D	V	A	M	A	A	M	K	K	D	H	S	I
N	D	N	A	N	T	T	V	M	D	V	A	M	A	A	M	K	K	D	H	S	I
N	D	N	A	N	T	T	V	M	D	V	A	M	A	A	M	K	K	D	H	S	I
N	D	N	A	N	T	T	V	M	D	V	A	M	A	A	M	K	K	D	H	S	I
N	D	N	A	N	T	T	V	M	D	V	A	M	A	A	M	K	K	D	H	S	I
N	D	N	A	N	T	T	V	M	D	V	A	M	A	A	M	K	K	D	H	S	I
N	D	N	A	N	T	T	V	M	D	V	A	M	A	A	M	K	K	D	H	S	I
N	D	N	A	N	T	T	V	M	D	V	A	M	A	A	M	K	K	D	H	S	I
N	D	N	A	N	T	T	V	M	D	V	A	M	A	A	M	K	K	D	H	S	I
N	D	N	A	N	T	T	V	M	D	V	A	M	A	A	M	K	K	D	H	S	I
N	D	N	A	N	T	T	V	M	D	V	A	M	A	A	M	K	K	D	H	S	I
N	D	N	A	N	T	T	V	M	D	V	A	M	A	A	M	K	K	D	H	S	I
N	D	N	A	N	T	T	V	M	D	V	A	M	A	A	M	K	K	D	H	S	I
N	D	N	A	N	T	T	V	M	D	V	A	M	A	A	M	K	K	D	H	S	I
N	D	N	A	N	T	T	V	M	D	V	A	M	A	A	M	K	K	D	H	S	I
N	D	N	A	N	T	T	V	M	D	V	A	M	A	A	M	K	K	D	H	S	I
N	D	N	A	N	T	T	V	M	D	V	A	M	A	A	M	K	K	D	H	S	I
N	D	N	A	N	T	T	V	M	D	V	A	M	A	A	M	K	K	D	H	S	I

wze																						
74	80	85	87	88	140	150	156	158	166	167	177	181	182	186	195	199	200	211	213	223	225	227
M	S	S	E	R	G	V	I	I	M	Q	K	T	K	L	Q	S	C	S	E	N	R	Q
I	L	A	D	K	E	I	V	V	T	R	E	I	N	I	H	P	F	S	D	D	G	N
I	L	A	D	K	E	I	V	V	T	R	E	I	N	I	H	P	F	S	D	D	G	N
I	L	A	D	K	E	I	V	V	T	R	E	I	N	I	H	P	F	S	D	D	G	N
I	L	A	D	K	E	I	V	V	T	R	E	I	N	I	H	P	F	S	D	D	G	N
I	L	A	D	K	E	I	V	V	T	R	E	I	N	I	H	P	F	S	D	D	G	N
I	L	A	D	K	E	I	V	V	T	R	E	I	N	I	H	P	F	S	D	D	G	N
I	L	A	D	K	E	I	V	V	T	R	E	I	N	I	H	P	F	S	D	D	G	N
I	L	A	D	K	E	I	V	V	T	R	E	I	N	I	H	P	F	S	D	D	G	N
I	L	A	D	K	E	I	V	V	T	R	E	I	N	I	H	P	F	S	D	D	G	N
I	L	A	D	K	E	I	V	V	T	R	E	I	N	I	H	P	F	S	D	D	G	N
I	L	A	D	K	E	I	V	V	T	R	E	I	N	I	H	P	F	S	D	D	G	N
I	L	A	D	K	E	I	V	V	T	R	E	I	N	I	H	P	F	S	D	D	G	N
I	L	A	D	K	E	I	V	V	T	R	E	I	N	I	H	P	F	S	D	D	G	N
I	L	A	D	K	E	I	V	V	T	R	E	I	N	I	H	P	F	S	D	D	G	N
I	L	A	D	K	E	I	V	V	T	R	E	I	N	I	H	P	F	S	D	D	G	N
I	L	A	D	K	E	I	V	V	T	R	E	I	N	I	H	P	F	S	D	D	G	N
I	L	A	D	K	E	I	V	V	T	R	E	I	N	I	H	P	F	S	D	D	G	N
I	L	A	D	K	E	I	V	V	T	R	E	I	N	I	H	P	F	S	D	D	G	N
I	L	A	D	K	E	I	V	V	T	R	E	I	N	I	H	P	F	S	D	D	G	N
I	L	A	D	K	E	I	V	V	T	R	E	I	N	I	H	P	F	S	D	D	G	N
I	L	A	D	K	E	I	V	V	T	R	E	I	N	I	H	P	F	S	D	D	G	N
I	L	A	D	K	E	I	V	V	T	R	E	I	N	I	H	P	F	S	D	D	G	N
I	L	A	D	K	E	I	V	V	T	R	E	I	N	I	H	P	F	S	D	D	G	N
I	L	A	D	K	E	I	V	V	T	R	E	I	N	I	H	P	F	S	D	D	G	N
I	L	A	D	K	E	I	V	V	T	R	E	I	N	I	H	P	F	S	D	D	G	N
I	L	A	D	K	E	I	V	V	T	R	E	I	N	I	H	P	F	S	D	D	G	N
I	L	A	D	K	E	I	V	V	T	R	E	I	N	I	H	P	F	S	D	D	G	N
I	L	A	D	K	E	I	V	V	T	R	E	I	N	I	H	P	F	S	D	D	G	N
I	L	A	D	K	E	I	V	V	T	R	E	I	N	I	H	P	F	S	D	D	G	N
I	L	A	D	K	E	I	V	V	T	R	E	I	N	I	H	P	F	S	D	D	G	N
I	L	A	D	K	E	I	V	V	T	R	E	I	N	I	H	P	F	S	D	D	G	N
I	L	A	D	K	E	I	V	V	T	R	E	I	N	I	H	P	F	S	D	D	G	N
I	L	A	D	K	E	I	V	V	T	R	E	I	N	I	H	P	F	S	D	D	G	N
I	L	A	D	K	E	I	V	V	T	R	E	I	N	I	H	P	F	L	D	D	G	N
I	L	A	D	K	E	I	V	V	T	R	E	I	N	I	H	P	F	S	D	D	G	N
I	L	A	D	K	E	I	V	V	T	R	E	I	N	I	H	P	F	S	D	D	G	N
I	L	A	D	K	E	I	V	V	T	R	E	I	N	I	H	P	F	S	D	D	G	N
I	L	A	D	K	E	I	V	V	T	R	E	I	N	I	H	P	F	S	D	D	G	N
I	L	A	D	K	E	I	V	V	T	R	E	I	N	I	H	P	F	S	D	D	G	N
I	L	A	D	K	E	I	V	V	T	R	E	I	N	I	H	P	F	S	D	D	G	N
I	L	A	D	K	E	I	V	V	T	R	E	I	N	I	H	P	F	S	D	D	G	N
I	L	A	D	K	E	I	V	V	T	R	E	I	N	I	H	P	F	S	D	D	G	N
I	L	A	D	K	E	I	V	V	T	R	E	I	N	I	H	P	F	S	D	D	G	N
I	L	A	D	K	E	I	V	V	T	R	E	I	N	I	H	P	F	S	D	D	G	N
I	L	A	D	K	E	I	V	V	T	R	E	I	N	I	H	P	F	S	D	D	G	N
I	L	A	D	K	E	I	V	V	T	R	E	I	N	I	H	P	F	S	D	D	G	N
I	L	A	D	K	E	I	V	V	T	R	E	I	N	I	H	P	F	S	D	D	G	N
I	L	A	D	K	E	I	V	V	T	R	E	I	N	I	H	P	F	S	D	D	G	N

wzh										
170	176	177	184	190	203	205	209	218	228	235
S	R	Y	A	R	V	S	H	S	E	R
S	P	D	A	R	V	G	Y	S	D	Q
S	P	D	A	R	V	G	Y	S	D	Q
S	R	Y	A	R	V	S	H	S	E	R
S	R	Y	V	Q	L	G	H	T	E	R
P	R	Y	A	Q	L	G	H	T	E	Q

14	15	21	24	27	29	35	38	41	49	60
S	L	Y	I	L	A	G	A	T	T	N
S	L	H	I	L	A	V	A	T	M	N
S	L	H	I	L	A	V	A	T	M	N
S	L	Y	I	L	A	G	A	T	T	N
L	F	R	M	I	A	G	V	T	T	N
L	F	R	M	I	A	G	A	A	T	D

P	R	Y	A	Q	L	G	H	T	E	Q	L	F	R	M	I	A	G	A	A	T	D
P	R	Y	A	Q	L	G	H	T	E	Q	L	F	R	M	I	A	G	A	A	T	D
P	R	Y	A	Q	L	G	H	T	E	Q	L	F	R	M	I	A	G	A	A	T	D
P	R	Y	A	Q	L	G	H	T	E	Q	L	F	R	M	I	A	G	A	A	T	D
P	R	Y	A	Q	L	G	H	T	E	Q	L	F	R	M	I	A	G	A	A	T	D
P	R	Y	A	Q	L	G	H	T	E	Q	L	F	R	M	I	A	G	A	A	T	D
P	R	Y	A	Q	L	G	H	T	E	Q	L	F	R	M	I	A	G	A	A	T	D
P	R	Y	A	Q	L	G	H	T	E	Q	L	F	R	M	I	A	G	A	A	T	D
P	R	Y	A	Q	L	G	H	T	E	Q	L	F	R	M	I	A	G	A	A	T	D
P	R	Y	A	Q	L	G	H	T	E	Q	L	F	R	M	I	A	G	A	A	T	D
P	R	Y	A	Q	L	G	H	T	E	Q	L	F	R	M	I	A	G	A	A	T	D
P	R	Y	A	Q	L	G	H	T	E	Q	L	F	R	M	I	A	G	A	A	T	D
P	R	Y	A	Q	L	G	H	T	E	Q	L	F	R	M	I	A	G	A	A	T	D
P	R	Y	A	Q	L	G	H	T	E	Q	L	F	R	M	I	A	G	A	A	T	D
P	R	Y	A	Q	L	G	H	T	E	Q	L	F	R	M	I	A	G	A	A	T	D
P	R	Y	A	Q	L	G	H	T	E	Q	L	F	R	M	I	A	G	A	A	T	D
P	R	Y	A	Q	L	G	H	T	E	Q	L	F	R	M	I	A	G	A	A	T	D
P	R	Y	A	Q	L	G	H	T	E	Q	L	F	R	M	I	A	G	A	A	T	D
P	R	Y	A	Q	L	G	H	T	E	Q	L	F	R	M	I	A	G	A	A	T	D
P	R	Y	A	Q	L	G	H	T	E	Q	L	F	R	M	I	A	G	A	A	T	D
P	R	Y	A	Q	L	G	H	T	E	Q	L	F	R	M	I	V	G	A	A	T	D
P	R	Y	A	Q	L	G	H	T	E	Q	L	F	R	M	I	V	G	A	A	T	D
P	R	Y	A	Q	L	G	H	T	E	Q	L	F	R	M	I	A	G	A	A	T	D
P	R	Y	A	Q	L	G	H	T	E	Q	L	F	R	M	I	A	G	A	A	T	D
P	R	Y	A	Q	L	G	H	T	E	Q	L	F	R	M	I	A	G	A	A	T	D
P	R	Y	A	Q	L	G	H	T	E	Q	L	F	R	M	I	A	G	A	A	T	D
P	R	Y	A	Q	L	G	H	T	E	Q	L	F	R	M	I	A	G	A	A	T	D
P	R	Y	A	Q	L	G	H	T	E	Q	L	F	R	M	I	A	G	A	A	T	D
P	R	Y	A	Q	L	G	H	T	E	Q	L	F	R	M	I	A	G	A	A	T	D
P	R	Y	A	Q	L	G	H	T	E	Q	L	F	R	M	I	A	G	A	A	T	D
P	R	Y	A	Q	L	G	H	T	E	Q	L	F	R	M	I	A	G	A	A	T	D
P	R	Y	A	Q	L	G	H	T	E	Q	L	F	R	M	I	A	G	A	A	T	D
P	R	Y	A	Q	L	G	H	T	E	Q	L	F	R	M	I	A	G	A	A	T	D
P	R	Y	A	Q	L	G	H	T	E	Q	L	F	R	M	I	A	G	A	A	T	D
P	R	Y	A	Q	L	G	H	T	E	Q	L	F	R	M	I	A	G	A	A	T	D
P	R	Y	A	Q	L	G	H	T	E	Q	L	F	R	M	I	A	G	A	A	T	D
P	R	Y	A	Q	L	G	H	T	E	Q	L	F	R	M	I	A	G	A	A	T	D
P	R	Y	A	Q	L	G	H	T	E	Q	L	F	R	M	I	A	G	A	A	T	D
P	R	Y	A	Q	L	G	H	T	E	Q	L	F	R	M	I	A	G	A	A	T	D
P	R	Y	A	Q	L	G	H	T	E	Q	L	F	R	M	I	A	G	A	A	T	D
P	R	Y	A	Q	L	G	H	T	E	Q	L	F	R	M	I	A	G	A	A	T	D
P	R	Y	A	Q	L	G	H	T	E	Q	L	F	R	M	I	A	G	A	A	T	D
P	R	Y	A	Q	L	G	H	T	E	Q	L	F	R	M	I	A	G	A	A	T	D
P	R	Y	A	Q	L	G	H	T	E	Q	L	F	R	M	I	A	G	A	A	T	D
P	R	Y	A	Q	L	G	H	T	E	Q	L	F	R	M	I	A	G	A	A	T	D
P	R	Y	A	Q	L	G	H	T	E	Q	L	F	R	M	I	A	G	A	A	T	D
P	R	Y	A	Q	L	G	H	T	E	Q	L	F	R	M	I	A	G	A	A	T	D
P	R	Y	A	Q	L	G	H	T	E	Q	L	F	R	M	I	A	G	A	A	T	D

wchA																					
2	3	5	6	8	10	13	31	40	56	104	105	107	108	134	135	141	142	175	203	259	270
S	E	L	A	P	V	V	T	V	R	L	L	V	F	V	F	S	H	Y	E	M	S
N	G	I	V	S	L	I	A	A	Q	T	V	A	L	I	L	S	R	C	K	V	A
N	G	I	V	S	L	I	A	A	Q	T	V	A	L	I	L	S	R	C	K	V	A
N	G	I	V	S	L	I	A	A	Q	T	V	A	L	I	L	S	R	C	K	V	A
N	G	I	V	S	L	I	A	A	Q	T	V	A	L	I	L	S	R	C	K	V	A
N	G	I	V	S	L	I	A	A	Q	T	V	A	L	I	L	S	R	C	K	V	A
N	G	I	V	S	L	I	A	A	Q	T	V	A	L	I	L	S	R	C	K	V	A
N	G	I	V	S	L	I	A	A	Q	T	V	A	L	I	L	S	R	C	K	V	A
N	G	I	V	S	L	I	A	A	Q	T	V	A	L	I	L	S	R	C	K	V	A
N	G	I	V	S	L	I	A	A	Q	T	V	A	L	I	L	S	R	C	K	V	A
N	G	I	V	S	L	I	A	A	Q	T	V	A	L	I	L	S	R	C	K	V	A
N	G	I	V	S	L	I	A	A	Q	T	V	A	L	I	L	S	R	C	K	V	A
N	G	I	V	S	L	I	A	A	Q	T	V	A	L	I	L	S	R	C	K	V	A
N	G	I	V	S	L	I	A	A	Q	T	V	A	L	I	L	S	R	C	K	V	A
N	G	I	V	S	L	I	A	A	Q	T	V	A	L	I	L	S	R	C	K	V	A
N	G	I	V	S	L	I	A	A	Q	T	V	A	L	I	L	S	R	C	K	V	A
N	G	I	V	S	L	I	A	A	Q	T	V	A	L	I	L	S	R	C	K	V	A
N	G	I	V	S	L	I	A	A	Q	T	V	A	L	I	L	S	R	C	K	V	A
N	G	I	V	S	L	I	A	A	Q	T	V	A	L	I	L	S	R	C	K	V	A
N	G	I	V	S	L	I	A	A	Q	T	V	A	L	I	L	S	R	C	K	V	A
N	G	I	V	S	L	I	A	A	Q	T	V	A	L	I	L	S	R	C	K	V	A
N	G	I	V	S	L	I	A	A	Q	T	V	A	L	I	L	S	R	C	K	V	A
N	G	I	V	S	L	I	A	A	Q	T	V	A	L	I	L	S	R	C	K	V	A
N	G	I	V	S	L	I	A	A	Q	T	V	A	L	I	L	S	R	C	K	V	A
N	G	I	V	S	L	I	A	A	Q	T	V	A	L	I	L	S	R	C	K	V	A
N	G	I	V	S	L	I	A	A	Q	T	V	A	L	I	L	S	R	C	K	V	A
N	G	I	V	S	L	I	A	A	Q	T	V	A	L	I	L	S	R	C	K	V	A
N	G	I	V	S	L	I	A	A	Q	T	V	A	L	I	L	S	R	C	K	V	A
N	G	I	V	S	L	I	A	A	Q	T	V	A	L	I	L	S	R	C	K	V	A
N	G	I	V	S	L	I	A	A	Q	T	V	A	L	I	L	S	R	C	K	V	A
N	G	I	V	S	L	I	A	A	Q	T	V	A	L	I	L	S	R	C	K	V	A
N	G	I	V	S	L	I	A	A	Q	T	V	A	L	I	L	S	R	C	K	V	A
N	G	I	V	S	L	I	A	A	Q	T	V	A	L	I	L	S	R	C	K	V	A
N	G	I	V	S	L	I	A	A	Q	T	V	A	L	I	L	F	R	C	K	V	A
N	G	I	V	S	L	I	A	A	Q	T	V	A	L	I	L	S	R	C	K	V	A
N	G	I	V	S	L	I	A	A	Q	T	V	A	L	I	L	S	R	C	K	V	A
N	G	I	V	S	L	I	A	A	Q	T	V	A	L	I	L	S	R	C	K	V	A
N	G	I	V	S	L	I	A	A	Q	T	V	A	L	I	L	S	R	C	K	V	A
N	G	I	V	S	L	I	A	A	Q	T	V	A	L	I	L	S	R	C	K	V	A
N	G	I	V	S	L	I	A	A	Q	T	V	A	L	I	L	S	R	C	K	V	A
N	G	I	V	S	L	I	A	A	Q	T	V	A	L	I	L	S	R	C	K	V	A
N	G	I	V	S	L	I	A	A	Q	T	V	A	L	I	L	S	R	C	K	V	A
N	G	I	V	S	L	I	A	A	Q	T	V	A	L	I	L	S	R	C	K	V	A
N	G	I	V	S	L	I	A	A	Q	T	V	A	L	I	L	S	R	C	K	V	A
N	G	I	V	S	L	I	A	A	Q	T	V	A	L	I	L	S	R	C	K	V	A
N	G	I	V	S	L	I	A	A	Q	T	V	A	L	I	L	S	R	C	K	V	A
N	G	I	V	S	L	I	A	A	Q	T	V	A	L	I	L	S	R	C	K	V	A
N	G	I	V	S	L	I	A	A	Q	T	V	A	L	I	L	S	R	C	K	V	A
N	G	I	V	S	L	I	A	A	Q	T	V	A	L	I	L	S	R	C	K	V	A

wzd																	
76	87	91	103	109	115	151	180	182	188	189	193	194	217	222	225	226	229
T	L	V	I	T	T	I	T	V	A	A	I	T	M	V	D	F	M
A	L	V	M	T	T	I	T	V	A	A	I	T	M	V	D	F	M
A	L	V	M	T	T	I	T	V	A	A	I	T	M	V	D	F	M
T	L	V	I	T	T	I	T	V	A	A	I	T	M	V	D	F	M
S	L	A	M	A	A	V	S	F	T	V	I	A	I	L	D	L	I
S	F	V	M	A	T	V	S	F	A	V	S	A	I	V	N	L	M

9	12	13	14
V	E	L	A
A	E	P	M
A	E	P	V
V	E	L	A
A	D	S	V
A	D	F	V

S	L	V	M	A	T	V	S	F	A	V	S	A	I	V	N	L	M	A	D	F	V
S	L	V	M	A	T	V	S	F	A	V	S	A	I	V	N	L	M	A	D	F	V
S	L	V	M	A	T	V	S	F	A	V	S	A	I	V	N	L	M	A	D	F	V
S	L	V	M	A	T	V	S	F	A	V	S	A	I	V	N	L	M	A	D	F	V
S	L	V	M	A	T	V	S	F	A	V	S	A	I	V	N	L	M	A	D	F	V
S	L	V	M	A	T	V	S	F	A	V	S	A	I	V	N	L	M	A	D	F	V
S	L	V	M	A	T	V	S	F	A	V	S	A	I	V	N	L	M	A	D	F	V
S	L	V	M	A	T	V	S	F	A	V	S	A	I	V	N	L	M	A	D	F	V
S	L	V	M	A	T	V	S	F	A	V	S	A	I	V	N	L	M	A	D	F	V
S	L	V	M	A	T	V	S	F	A	V	S	A	I	V	N	L	M	A	D	F	V
S	L	V	M	A	T	V	S	F	A	V	S	A	I	V	N	L	M	A	D	F	V
S	L	V	M	A	T	V	S	F	A	V	S	A	I	V	N	L	M	A	D	F	V
S	L	V	M	A	T	V	S	F	A	V	S	A	I	V	N	L	M	A	D	F	V
S	L	V	M	A	T	V	S	F	A	V	S	A	I	V	N	L	M	A	D	F	V
S	L	V	M	A	T	V	S	F	A	V	S	A	I	V	N	L	M	A	D	F	V
S	L	V	M	A	T	V	S	F	A	V	S	A	I	V	N	L	M	A	D	F	V
S	L	V	M	A	T	V	S	F	A	V	S	A	I	V	N	L	M	A	D	F	V
S	L	V	M	A	T	V	S	F	A	V	S	A	I	V	N	L	M	A	D	F	V
S	L	V	M	A	T	V	S	F	A	V	S	A	I	V	N	L	M	A	D	F	V
S	L	V	M	A	T	V	S	F	A	V	S	A	I	V	N	L	M	A	D	F	V
S	L	V	M	A	T	V	S	F	A	V	S	A	I	V	N	L	M	A	D	F	V
S	L	V	M	A	T	V	S	F	A	V	S	A	I	V	N	L	M	A	D	F	V
S	L	V	M	A	T	V	S	F	A	V	S	A	I	V	N	L	M	A	D	F	V
S	L	V	M	A	T	V	S	F	A	V	S	A	I	V	N	L	M	A	D	F	V
S	L	V	M	A	T	V	S	F	A	V	S	A	I	V	N	L	M	A	D	F	V
S	L	V	M	A	T	V	S	F	A	V	S	A	I	V	N	L	M	A	D	F	V
S	L	V	M	A	T	V	S	F	A	V	S	A	I	V	N	L	M	A	D	F	V
S	L	V	M	A	T	V	S	F	A	V	S	A	I	V	N	L	M	A	D	F	V
S	L	V	M	A	T	V	S	F	A	V	S	A	I	V	N	L	M	A	D	F	V
S	L	V	M	A	T	V	S	F	A	V	S	A	I	V	N	L	M	A	D	F	V
S	L	V	M	A	T	V	S	F	A	V	S	A	I	V	N	L	M	A	D	F	V
S	L	V	M	A	T	V	S	F	A	V	S	A	I	V	N	L	M	A	D	F	V
S	L	V	M	A	T	V	S	F	A	V	S	A	I	V	N	L	M	A	D	F	V
S	L	V	M	A	T	V	S	F	A	V	S	A	I	V	N	L	M	A	D	F	V
S	L	V	M	A	T	V	S	F	A	V	S	A	I	V	N	L	M	A	D	F	V
S	L	V	M	A	T	V	S	F	A	V	S	A	I	V	N	L	M	A	D	F	V
S	L	V	M	A	T	V	S	F	A	V	S	A	I	V	N	L	M	A	D	F	V
S	L	V	M	A	T	V	S	F	A	V	S	A	I	V	N	L	M	A	D	F	V
S	L	V	M	A	T	V	S	F	A	V	S	A	I	V	N	L	M	A	D	F	V
S	L	V	M	A	T	V	S	F	A	V	S	A	I	V	N	L	M	A	D	F	V
S	L	V	M	A	T	V	S	F	A	V	S	A	I	V	N	L	M	A	D	F	V
S	L	V	M	A	T	V	S	F	A	V	S	A	I	V	N	L	M	A	D	F	V
S	L	V	M	A	T	V	S	F	A	V	S	A	I	V	N	L	M	A	D	F	V
S	L	V	M	A	T	V	S	F	A	V	S	A	I	V	N	L	M	A	D	F	V
S	L	V	M	A	T	V	S	F	A	V	S	A	I	V	N	L	M	A	D	F	V
S	L	V	M	A	T	V	S	F	A	V	S	A	I	V	N	L	M	A	D	F	V
S	L	V	M	A	T	V	S	F	A	V	S	A	I	V	N	L	M	A	D	F	V
S	L	V	M	A	T	V	S	F	A	V	S	A	I	V	N	L	M	A	D	F	V

285	296	308	323	352	409	449	451
V	F	H	D	H	V	L	K
L	I	Q	A	R	I	F	R
L	I	Q	A	R	I	F	R
L	I	Q	A	R	I	F	R
L	I	Q	A	R	I	F	R
L	I	Q	A	R	I	F	R
L	I	Q	A	R	I	F	R
L	I	Q	A	R	I	F	R
L	I	Q	A	R	I	F	R
L	I	Q	A	R	I	F	R
L	I	Q	A	R	I	F	R
L	I	Q	A	R	I	F	R
L	I	Q	A	R	I	F	R
L	I	Q	A	R	I	F	R
L	I	Q	A	R	I	F	R
L	I	Q	A	R	I	F	R
L	I	Q	A	R	I	F	R
L	I	Q	A	R	I	F	R
L	I	Q	A	R	I	F	R
L	I	Q	A	R	I	F	R
L	I	Q	A	R	I	F	R
L	I	Q	A	R	I	F	R
L	I	Q	A	R	I	F	R
L	I	Q	A	R	I	F	R
L	I	Q	A	R	I	F	R
L	I	Q	A	R	I	F	R
L	I	Q	A	R	I	F	R
L	I	Q	A	R	I	F	R
L	I	Q	A	R	I	F	R
L	I	Q	A	R	I	F	R
L	I	Q	A	R	I	F	R
L	I	Q	A	R	I	F	R
L	I	Q	A	R	I	F	R
L	I	Q	A	R	I	F	R
L	I	Q	A	R	I	F	R
L	I	Q	A	R	I	F	R
L	I	Q	A	R	I	F	R
L	I	Q	A	R	I	F	R
L	I	Q	A	R	I	F	R
L	I	Q	A	R	I	F	R
L	I	Q	A	R	I	F	R
L	I	Q	A	R	I	F	R
L	I	Q	A	R	I	F	R
L	I	Q	A	R	I	F	R
L	I	Q	A	R	I	F	R
L	I	Q	A	R	I	F	R
L	I	Q	A	R	I	F	R
L	I	Q	A	R	I	F	R
L	I	Q	A	R	I	F	R
L	I	Q	A	R	I	F	R
L	I	Q	A	R	I	F	R

wchJ													
36	38	41	43	44	46	47	51	58	66	69	70	73	79
E	T	I	G	N	T	F	H	L	V	F	N	R	V
A	A	V	S	E	V	V	Y	I	F	Y	K	K	L
A	A	V	S	E	V	V	Y	I	F	Y	K	K	L
A	A	V	S	E	V	V	Y	I	F	Y	K	K	L
A	A	V	S	E	V	V	Y	I	F	Y	K	K	L
A	A	V	S	E	V	V	Y	I	F	Y	K	K	L
A	A	V	S	E	V	V	Y	I	F	Y	K	K	L
A	A	V	S	E	V	V	Y	I	F	Y	K	K	L
A	A	V	S	E	V	V	Y	I	F	Y	K	K	L
A	A	V	S	E	V	V	Y	I	F	Y	K	K	L
A	A	V	S	E	V	V	Y	I	F	Y	K	K	L
A	A	V	S	E	V	V	Y	I	F	Y	K	K	L
A	A	V	S	E	V	V	Y	I	F	Y	K	K	L
A	A	V	S	E	V	V	Y	I	F	Y	K	K	L
A	A	V	S	E	V	V	Y	I	F	Y	K	K	L
A	A	V	S	E	V	V	Y	I	F	Y	K	K	L
A	A	V	S	E	V	V	Y	I	F	Y	K	K	L
A	A	V	S	E	V	V	Y	I	F	Y	K	K	L
A	A	V	S	E	V	V	Y	I	F	Y	K	K	L
A	A	V	S	E	V	V	Y	I	F	Y	K	K	L
A	A	V	S	E	V	V	Y	I	F	Y	K	K	L
A	A	V	S	E	V	V	Y	I	F	Y	K	K	L
A	A	V	S	E	V	V	Y	I	F	Y	K	K	L
A	A	V	S	E	V	V	Y	I	F	Y	K	K	L
A	A	V	S	E	V	V	Y	I	F	Y	K	K	L
A	A	V	S	E	V	V	Y	I	F	Y	K	K	L
A	A	V	S	E	V	V	Y	I	F	Y	K	K	L
A	A	V	S	E	V	V	Y	I	F	Y	K	K	L
A	A	V	S	E	V	V	Y	I	F	Y	K	K	L
A	A	V	S	E	V	V	Y	I	F	Y	K	K	L
A	A	V	S	E	V	V	Y	I	F	Y	K	K	L
A	A	V	S	E	V	V	Y	I	F	Y	K	K	L
A	A	V	S	E	V	V	Y	I	F	Y	K	K	L
A	A	V	S	E	V	V	Y	I	F	Y	K	K	L
A	A	V	S	E	V	V	Y	I	F	Y	K	K	L
A	A	V	S	E	V	V	Y	I	F	Y	K	K	L
A	A	V	S	E	V	V	Y	I	F	Y	K	K	L
A	A	V	S	E	V	V	Y	I	F	Y	K	K	L
A	A	V	S	E	V	V	Y	I	F	Y	K	K	L
A	A	V	S	E	V	V	Y	I	F	Y	K	K	L
A	A	V	S	E	V	V	Y	I	F	Y	K	K	L
A	A	V	S	E	V	V	Y	I	F	Y	K	K	L
A	A	V	S	E	V	V	Y	I	F	Y	K	K	L
A	A	V	S	E	V	V	Y	I	F	Y	K	K	L
A	A	V	S	E	V	V	Y	I	F	Y	K	K	L
A	A	V	S	E	V	V	Y	I	F	Y	K	K	L
A	A	V	S	E	V	V	Y	I	F	Y	K	K	L
A	A	V	S	E	V	V	Y	I	F	Y	K	K	L
A	A	V	S	E	V	V	Y	I	F	Y	K	K	L
A	A	V	S	E	V	V	Y	I	F	Y	K	K	L

wze																						
34	45	50	54	62	72	74	80	85	87	88	136	140	150	156	158	166	167	168	177	181	182	186
D	A	T	T	R	A	M	S	S	E	R	S	G	V	I	I	M	Q	K	K	T	K	L
D	A	T	I	R	A	M	S	P	E	R	S	G	V	I	I	M	Q	K	K	T	K	L
D	A	T	I	R	A	M	S	P	E	R	S	G	V	I	I	M	Q	K	K	T	K	L
D	A	T	T	R	A	M	S	S	E	R	S	G	V	I	I	M	Q	K	K	T	K	L
G	I	S	A	R	G	I	L	A	N	K	T	E	I	V	V	T	R	N	E	I	N	I
D	L	S	T	H	G	I	L	A	D	K	S	E	I	V	V	T	R	K	E	I	N	I

D	L	S	T	H	G	I	L	A	D	K	S	E	I	V	V	T	R	K	E	I	N	I
D	L	S	T	H	G	I	L	A	D	K	S	E	I	V	V	T	R	K	E	I	N	I
D	L	S	T	H	G	I	L	A	D	K	S	E	I	V	V	T	R	K	E	I	N	I
D	L	S	T	H	G	I	L	A	D	K	S	E	I	V	V	T	R	K	E	I	N	I
D	L	S	T	H	G	I	L	A	D	K	S	E	I	V	V	T	R	K	E	I	N	I
D	L	S	T	H	G	I	L	A	D	K	S	E	I	V	V	T	R	K	E	I	N	I
D	L	S	T	H	G	I	L	A	D	K	S	E	I	V	V	T	R	K	E	I	N	I
D	L	S	T	H	G	I	L	A	D	K	S	E	I	V	V	T	R	K	E	I	N	I
D	L	S	T	H	G	I	L	A	D	K	S	E	I	V	V	T	R	K	E	I	N	I
D	L	S	T	H	G	I	L	A	D	K	S	E	I	V	V	T	R	K	E	I	N	I
D	L	S	T	H	G	I	L	A	D	K	S	E	I	V	V	T	R	K	E	I	N	I
D	L	S	T	H	G	I	L	A	D	K	S	E	I	V	V	T	R	K	E	I	N	I
D	L	S	T	H	G	I	L	A	D	K	S	E	I	V	V	T	R	K	E	I	N	I
D	L	S	T	H	G	I	L	A	D	K	S	E	I	V	V	T	R	K	E	I	N	I
D	L	S	T	H	G	I	L	A	D	K	S	E	I	V	V	T	R	K	E	I	N	I
D	L	S	T	H	G	I	L	A	D	K	S	E	I	V	V	T	R	K	E	I	N	I
D	L	S	T	H	G	I	L	A	D	K	S	E	I	V	V	T	R	K	E	I	N	I
D	L	S	T	H	G	I	L	A	D	K	S	E	I	V	V	T	R	K	E	I	N	I
D	L	S	T	H	G	I	L	A	D	K	S	E	I	V	V	T	R	K	E	I	N	I
D	L	S	T	H	G	I	L	A	D	K	S	E	I	V	V	T	R	K	E	I	N	I
D	L	S	T	H	G	I	L	A	D	K	S	E	I	V	V	T	R	K	E	I	N	I
D	L	S	T	H	G	I	L	A	D	K	S	E	I	V	V	T	R	K	E	I	N	I
D	L	S	T	H	G	I	L	A	D	K	S	E	I	V	V	T	R	K	E	I	N	I
D	L	S	T	H	G	I	L	A	D	K	S	E	I	V	V	T	R	K	E	I	N	I
D	L	S	T	H	G	I	L	A	D	K	S	E	I	V	V	T	R	K	E	I	N	I
D	L	S	T	H	G	I	L	A	D	K	S	E	I	V	V	T	R	K	E	I	N	I
D	L	S	T	H	G	I	L	A	D	K	S	E	I	V	V	T	R	K	E	I	N	I
D	L	S	T	H	G	I	L	A	D	K	S	E	I	V	V	T	R	K	E	I	N	I
D	L	S	T	H	G	I	L	A	D	K	S	E	I	V	V	T	R	K	E	I	N	I
D	L	S	T	H	G	I	L	A	D	K	S	E	I	V	V	T	R	K	E	I	N	I
D	L	S	T	H	G	I	L	A	D	K	S	E	I	V	V	T	R	K	E	I	N	I
D	L	S	T	H	G	I	L	A	D	K	S	E	I	V	V	T	R	K	E	I	N	I
D	L	S	T	H	G	I	L	A	D	K	S	E	I	V	V	T	R	K	E	I	N	I
D	L	S	T	H	G	I	L	A	D	K	S	E	I	V	V	T	R	K	E	I	N	I
D	L	S	T	H	G	I	L	A	D	K	S	E	I	V	V	T	R	K	E	I	N	I
D	L	S	T	H	G	I	L	A	D	K	S	E	I	V	V	T	R	K	E	I	N	I
D	L	S	T	H	G	I	L	A	D	K	S	E	I	V	V	T	R	K	E	I	N	I
D	L	S	T	H	G	I	L	A	D	K	S	E	I	V	V	T	R	K	E	I	N	I
D	L	S	T	H	G	I	L	A	D	K	S	E	I	V	V	T	R	K	E	I	N	I
D	L	S	T	H	G	I	L	A	D	K	S	E	I	V	V	T	R	K	E	I	N	I
D	L	S	T	H	G	I	L	A	D	K	S	E	I	V	V	T	R	K	E	I	N	I
D	L	S	T	H	G	I	L	A	D	K	S	E	I	V	V	T	R	K	E	I	N	I
D	L	S	T	H	G	I	L	A	D	K	S	E	I	V	V	T	R	K	E	I	N	I
D	L	S	T	H	G	I	L	A	D	K	S	E	I	V	V	T	R	K	E	I	N	I
D	L	S	T	H	G	I	L	A	D	K	S	E	I	V	V	T	R	K	E	I	N	I
D	L	S	T	H	G	I	L	A	D	K	S	E	I	V	V	T	R	K	E	I	N	I
D	L	S	T	H	G	I	L	A	D	K	S	E	I	V	V	T	R	K	E	I	N	I
D	L	S	T	H	G	I	L	A	D	K	S	E	I	V	V	T	R	K	E	I	N	I

87	94	100	116	120	127	138
V	L	A	L	I	K	V
I	I	S	I	L	R	I
I	I	S	I	L	R	I
I	I	S	I	L	R	I
I	I	S	I	L	R	I
I	I	S	I	L	R	I
I	I	S	I	L	R	I
I	I	S	I	L	R	I
I	I	S	I	L	R	I
I	I	S	I	L	R	I
I	I	S	I	L	R	I
I	I	S	I	L	R	I
I	I	S	I	L	R	I
I	I	S	I	L	R	I
I	I	S	I	L	R	I
I	I	S	I	L	R	I
I	I	S	I	L	R	I
I	I	S	I	L	R	I
I	I	S	I	L	R	I
I	I	S	I	L	R	I
I	I	S	I	L	R	I
I	I	S	I	L	R	I
I	I	S	I	L	R	I
I	I	S	I	L	R	I
I	I	S	I	L	R	I
I	I	S	I	L	R	I
I	I	S	I	L	R	I
I	I	S	I	L	R	I
I	I	S	I	L	R	I
I	I	S	I	L	R	I
I	I	S	I	L	R	I
I	I	S	I	L	R	I
I	I	S	I	L	R	I
I	I	S	I	L	R	I
I	I	S	I	L	R	I
I	I	S	I	L	R	I
I	I	S	I	L	R	I
I	I	S	I	L	R	I
I	I	S	I	L	R	I
I	I	S	I	L	R	I
I	I	S	I	L	R	I
I	I	S	I	L	R	I
I	I	S	I	L	R	I
I	I	S	I	L	R	I
I	I	S	I	L	R	I
I	I	S	I	L	R	I
I	I	S	I	L	R	I
I	I	S	I	L	R	I
I	I	S	I	L	R	I
I	I	S	I	L	R	I

6	14	20	25	27	28	31	32	39	41	43	45	48	50	51
I	N	V	G	G	F	D	D	F	D	E	V	Q	K	N
V	D	F	E	N	L	E	E	Y	N	V	K	K	E	K
V	D	F	E	N	L	E	E	Y	N	V	K	K	E	K
V	D	F	E	N	L	E	E	Y	N	V	K	K	E	K
V	D	F	E	N	L	E	E	Y	N	V	K	K	E	K
V	D	F	E	N	L	E	E	Y	N	V	K	K	E	K
V	D	F	E	N	L	E	E	Y	N	V	K	K	E	K
V	D	F	E	N	L	E	E	Y	N	V	K	K	E	K
V	D	F	E	N	L	E	E	Y	N	V	K	K	E	K
V	D	F	E	N	L	E	E	Y	N	V	K	K	E	K
V	D	F	E	N	L	E	E	Y	N	V	K	K	E	K
V	D	F	E	N	L	E	E	Y	N	V	K	K	E	K
V	D	F	E	N	L	E	E	Y	N	V	K	K	E	K
V	D	F	E	N	L	E	E	Y	N	V	K	K	E	K
V	D	F	E	N	L	E	E	Y	N	V	K	K	E	K
V	D	F	E	N	L	E	E	Y	N	V	K	K	E	K
V	D	F	E	N	L	E	E	Y	N	V	K	K	E	K
V	D	F	E	N	L	E	E	Y	N	V	K	K	E	K
V	D	F	E	N	L	E	E	Y	N	V	K	K	E	K
V	D	F	E	N	L	E	E	Y	N	V	K	K	E	K
V	D	F	E	N	L	E	E	Y	N	V	K	K	E	K
V	D	F	E	N	L	E	E	Y	N	V	K	K	E	K
V	D	F	E	N	L	E	E	Y	N	V	K	K	E	K
V	D	F	E	N	L	E	E	Y	N	V	K	K	E	K
V	D	F	E	N	L	E	E	Y	N	V	K	K	E	K
V	D	F	E	N	L	E	E	Y	N	V	K	K	E	K
V	D	F	E	N	L	E	E	Y	N	V	K	K	E	K
V	D	F	E	N	L	E	E	Y	N	V	K	K	E	K
V	D	F	E	N	L	E	E	Y	N	V	K	K	E	K
V	D	F	E	N	L	E	E	Y	N	V	K	K	E	K
V	D	F	E	N	L	E	E	Y	N	V	K	K	E	K
V	D	F	E	N	L	E	E	Y	N	V	K	K	E	K
V	D	F	E	N	L	E	E	Y	N	V	K	K	E	K
V	D	F	E	N	L	E	E	Y	N	V	K	K	E	K
V	D	F	E	N	L	E	E	Y	N	V	K	K	E	K
V	D	F	E	N	L	E	E	Y	N	V	K	K	E	K
V	D	F	E	N	L	E	E	Y	N	V	K	K	E	K
V	D	F	E	N	L	E	E	Y	N	V	K	K	E	K
V	D	F	E	N	L	E	E	Y	N	V	K	K	E	K
V	D	F	E	N	L	E	E	Y	N	V	K	K	E	K
V	D	F	E	N	L	E	E	Y	N	V	K	K	E	K
V	D	F	E	N	L	E	E	Y	N	V	K	K	E	K
V	D	F	E	N	L	E	E	Y	N	V	K	K	E	K
V	D	F	E	N	L	E	E	Y	N	V	K	K	E	K
V	D	F	E	N	L	E	E	Y	N	V	K	K	E	K
V	D	F	E	N	L	E	E	Y	N	V	K	K	E	K
V	D	F	E	N	L	E	E	Y	N	V	K	K	E	K
V	D	F	E	N	L	E	E	Y	N	V	K	K	E	K
V	D	F	E	N	L	E	E	Y	N	V	K	K	E	K
V	D	F	E	N	L	E	E	Y	N	V	K	K	E	K

195	199	200	203	211	213	226	229	231	233
Q	S	C	V	S	E	S	N	R	Q
Q	S	C	V	L	E	N	N	R	Q
Q	S	C	V	S	E	N	N	R	Q
Q	S	C	V	S	E	S	N	R	Q
H	P	F	I	S	D	N	N	G	N
H	P	F	V	S	D	-	D	G	N

2	3	5	6	7	8	10	11	13	23	31	40
S	E	L	A	K	P	V	A	V	Y	T	V
N	E	L	A	K	L	V	V	A	V	T	V
N	E	L	A	K	L	V	V	A	V	T	V
S	E	L	A	K	P	V	A	V	Y	T	V
N	G	I	L	R	S	L	A	I	Y	T	A
N	G	I	V	K	S	L	A	I	Y	A	A

H	P	F	V	S	D	-	D	G	N	N	G	I	V	K	S	L	A	I	Y	A	A
H	P	F	V	S	D	-	D	G	N	N	G	I	V	K	S	L	A	I	Y	A	A
H	P	F	V	S	D	-	D	G	N	N	G	I	V	K	S	L	A	I	Y	A	A
H	P	F	V	S	D	-	D	G	N	N	G	I	V	K	S	L	A	I	Y	A	A
H	P	F	V	S	D	-	D	G	N	N	G	I	V	K	S	L	A	I	Y	A	A
H	P	F	V	S	D	-	D	G	N	N	G	I	V	K	S	L	A	I	Y	A	A
H	P	F	V	S	D	-	D	G	N	N	G	I	V	K	S	L	A	I	Y	A	A
H	P	F	V	S	D	-	D	G	N	N	G	I	V	K	S	L	A	I	Y	A	A
H	P	F	V	S	D	-	D	G	N	N	G	I	V	K	S	L	A	I	Y	A	A
H	P	F	V	S	D	-	D	G	N	N	G	I	V	K	S	L	A	I	Y	A	A
H	P	F	V	S	D	-	D	G	N	N	G	I	V	K	S	L	A	I	Y	A	A
H	P	F	V	S	D	-	D	G	N	N	G	I	V	K	S	L	A	I	Y	A	A
H	P	F	V	S	D	-	D	G	N	N	G	I	V	K	S	L	A	I	Y	A	A
H	P	F	V	S	D	-	D	G	N	N	G	I	V	K	S	L	A	I	Y	A	A
H	P	F	V	S	D	-	D	G	N	N	G	I	V	K	S	L	A	I	Y	A	A
H	P	F	V	S	D	-	D	G	N	N	G	I	V	K	S	L	A	I	Y	A	A
H	P	F	V	S	D	-	D	G	N	N	G	I	V	K	S	L	A	I	Y	A	A
H	P	F	V	S	D	-	D	G	N	N	G	I	V	K	S	L	A	I	Y	A	A
H	P	F	V	S	D	-	D	G	N	N	G	I	V	K	S	L	A	I	Y	A	A
H	P	F	V	S	D	-	D	G	N	N	G	I	V	K	S	L	A	I	Y	A	A
H	P	F	V	S	D	-	D	G	N	N	G	I	V	K	S	L	A	I	Y	A	A
H	P	F	V	S	D	-	D	G	N	N	G	I	V	K	S	L	A	I	Y	A	A
H	P	F	V	S	D	-	D	G	N	N	G	I	V	K	S	L	A	I	Y	A	A
H	P	F	V	S	D	-	D	G	N	N	G	I	V	K	S	L	A	I	Y	A	A
H	P	F	V	S	D	-	D	G	N	N	G	I	V	K	S	L	A	I	Y	A	A
H	P	F	V	S	D	-	D	G	N	N	G	I	V	K	S	L	A	I	Y	A	A
H	P	F	V	S	D	-	D	G	N	N	G	I	V	K	S	L	A	I	Y	A	A
H	P	F	V	S	D	-	D	G	N	N	G	I	V	K	S	L	A	I	Y	A	A
H	P	F	V	S	D	-	D	G	N	N	G	I	V	K	S	L	A	I	Y	A	A
H	P	F	V	S	D	-	D	G	N	N	G	I	V	K	S	L	A	I	Y	A	A
H	P	F	V	S	D	-	D	G	N	N	G	I	V	K	S	L	A	I	Y	A	A
H	P	F	V	S	D	-	D	G	N	N	G	I	V	K	S	L	A	I	Y	A	A
H	P	F	V	S	D	-	D	G	N	N	G	I	V	K	S	L	A	I	Y	A	A
H	P	F	V	L	D	-	D	G	N	N	G	I	V	K	S	L	A	I	Y	A	A
H	P	F	V	S	D	-	D	G	N	N	G	I	V	K	S	L	A	I	Y	A	A
H	P	F	V	S	D	-	D	G	N	N	G	I	V	K	S	L	A	I	Y	A	A
H	P	F	V	S	D	-	D	G	N	N	G	I	V	K	S	L	A	I	Y	A	A
H	P	F	V	S	D	-	D	G	N	N	G	I	V	K	S	L	A	I	Y	A	A
H	P	F	V	S	D	-	D	G	N	N	G	I	V	K	S	L	A	I	Y	A	A
H	P	F	V	S	D	-	D	G	N	N	G	I	V	K	S	L	A	I	Y	A	A
H	P	F	V	S	D	-	D	G	N	N	G	I	V	K	S	L	A	I	Y	A	A
H	P	F	V	S	D	-	D	G	N	N	G	I	V	K	S	L	A	I	Y	A	A
H	P	F	V	S	D	-	D	G	N	N	G	I	V	K	S	L	A	I	Y	A	A
H	P	F	V	S	D	-	D	G	N	N	G	I	V	K	S	L	A	I	Y	A	A
H	P	F	V	S	D	-	D	G	N	N	G	I	V	K	S	L	A	I	Y	A	A
H	P	F	V	S	D	-	D	G	N	N	G	I	V	K	S	L	A	I	Y	A	A
H	P	F	V	S	D	-	D	G	N	N	G	I	V	K	S	L	A	I	Y	A	A
H	P	F	V	S	D	-	D	G	N	N	G	I	V	K	S	L	A	I	Y	A	A

wchK																	
52	56	57	60	61	63	66	67	69	80	86	93	106	112	144	145	149	153
L	D	E	R	Y	D	N	I	I	V	I	L	E	L	T	C	K	T
I	E	K	Q	F	E	D	T	V	I	T	Q	N	S	A	N	Q	I
I	E	K	Q	F	E	D	T	V	I	T	Q	N	S	A	N	Q	I
I	E	K	Q	F	E	D	T	V	I	T	Q	N	S	A	N	Q	I
I	E	K	Q	F	E	D	T	V	I	T	Q	N	S	A	N	Q	I
I	E	K	Q	F	E	D	T	V	I	T	Q	N	S	A	N	Q	I
I	E	K	Q	F	E	D	T	V	I	T	Q	N	S	A	N	Q	I
I	E	K	Q	F	E	D	T	V	I	T	Q	N	S	A	N	Q	I
I	E	K	Q	F	E	D	T	V	I	T	Q	N	S	A	N	Q	I
I	E	K	Q	F	E	D	T	V	I	T	Q	N	S	A	N	Q	I
I	E	K	Q	F	E	D	T	V	I	T	Q	N	S	A	N	Q	I
I	E	K	Q	F	E	D	T	V	I	T	Q	N	S	A	N	Q	I
I	E	K	Q	F	E	D	T	V	I	T	Q	N	S	A	N	Q	I
I	E	K	Q	F	E	D	T	V	I	T	Q	N	S	A	N	Q	I
I	E	K	Q	F	E	D	T	V	I	T	Q	N	S	A	N	Q	I
I	E	K	Q	F	E	D	T	V	I	T	Q	N	S	A	N	Q	I
I	E	K	Q	F	E	D	T	V	I	T	Q	N	S	A	N	Q	I
I	E	K	Q	F	E	D	T	V	I	T	Q	N	S	A	N	Q	I
I	E	K	Q	F	E	D	T	V	I	T	Q	N	S	A	N	Q	I
I	E	K	Q	F	E	D	T	V	I	T	Q	N	S	A	N	Q	I
I	E	K	Q	F	E	D	T	V	I	T	Q	N	S	A	N	Q	I
I	E	K	Q	F	E	D	T	V	I	T	Q	N	S	A	N	Q	I
I	E	K	Q	F	E	D	T	V	I	T	Q	N	S	A	N	Q	I
I	E	K	Q	F	E	D	T	V	I	T	Q	N	S	A	N	Q	I
I	E	K	Q	F	E	D	T	V	I	T	Q	N	S	A	N	Q	I
I	E	K	Q	F	E	D	T	V	I	T	Q	N	S	A	N	Q	I
I	E	K	Q	F	E	D	T	V	I	T	Q	N	S	A	N	Q	I
I	E	K	Q	F	E	D	T	V	I	T	Q	N	S	A	N	Q	I
I	E	K	Q	F	E	D	T	V	I	T	Q	N	S	A	N	Q	I
I	E	K	Q	F	E	D	T	V	I	T	Q	N	S	A	N	Q	I
I	E	K	Q	F	E	D	T	V	I	T	Q	N	S	A	N	Q	I
I	E	K	Q	F	E	D	T	V	I	T	Q	N	S	A	N	Q	I
I	E	K	Q	F	E	D	T	V	I	T	Q	N	S	A	N	Q	I
I	E	K	Q	F	E	D	T	V	I	T	Q	N	S	A	N	Q	I
I	E	K	Q	F	E	D	T	V	I	T	Q	N	S	A	N	Q	I
I	E	K	Q	F	E	D	T	V	I	T	Q	N	S	A	N	Q	I
I	E	K	Q	F	E	D	T	V	I	T	Q	N	S	A	N	Q	I
I	E	K	Q	F	E	D	T	V	I	T	Q	N	S	A	N	Q	I
I	E	K	Q	F	E	D	T	V	I	T	Q	N	S	A	N	Q	I
I	E	K	Q	F	E	D	T	V	I	T	Q	N	S	A	N	Q	I
I	E	K	Q	F	E	D	T	V	I	T	Q	N	S	A	N	Q	I
I	E	K	Q	F	E	D	T	V	I	T	Q	N	S	A	N	Q	I
I	E	K	Q	F	E	D	T	V	I	T	Q	N	S	A	N	Q	I
I	E	K	Q	F	E	D	T	V	I	T	Q	N	S	A	N	Q	I
I	E	K	Q	F	E	D	T	V	I	T	Q	N	S	A	N	Q	I
I	E	K	Q	F	E	D	T	V	I	T	Q	N	S	A	N	Q	I
I	E	K	Q	F	E	D	T	V	I	T	Q	N	S	A	N	Q	I
I	E	K	Q	F	E	D	T	V	I	T	Q	N	S	A	N	Q	I
I	E	K	Q	F	E	D	T	V	I	T	Q	N	S	A	N	Q	

41	42	52	71
S	P	C	V
P	L	Y	I
P	L	Y	I
P	L	Y	I
P	L	Y	I
P	L	Y	I
P	L	Y	I
P	L	Y	I
P	L	Y	I
P	L	Y	I
P	L	Y	I
P	L	Y	I
P	L	Y	I
P	L	Y	I
P	L	Y	I
P	L	Y	I
P	L	Y	I
P	L	Y	I
P	L	Y	I
P	L	Y	I
P	L	Y	I
P	L	Y	I
P	L	Y	I
P	L	Y	I
P	L	Y	I
P	L	Y	I
P	L	Y	I
P	L	Y	I
P	L	Y	I
P	L	Y	I
P	L	Y	I
P	L	Y	I
P	L	Y	I
P	L	Y	I
P	L	Y	I
P	L	Y	I
P	L	Y	I
P	L	Y	I
P	L	Y	I
P	L	Y	I
P	L	Y	I
P	L	Y	I
P	L	Y	I
P	L	Y	I
P	L	Y	I
P	L	Y	I
P	L	Y	I
P	L	Y	I
P	L	Y	I
P	L	Y	I

																wchA						
56	57	62	88	104	105	106	107	108	122	124	134	135	141	142	143	148	149	154	155	159	175	203
R	N	G	L	L	L	H	V	F	K	A	V	F	S	H	V	D	R	N	E	E	Y	E
R	N	G	S	L	L	Y	V	F	R	A	F	F	S	H	I	D	R	D	D	K	Y	E
R	N	G	S	L	L	Y	V	F	R	A	F	F	S	H	I	D	R	D	D	K	Y	E
R	N	G	L	L	L	H	V	F	K	A	V	F	S	H	V	D	R	N	E	E	Y	E
Q	D	R	L	T	L	Y	A	L	K	T	I	L	S	R	V	H	K	N	E	E	C	G
Q	N	G	L	T	V	H	A	L	K	A	I	L	S	R	V	D	R	N	E	E	C	K

Q	N	G	L	T	V	H	A	L	K	A	I	L	S	R	V	D	R	N	E	E	C	K
Q	N	G	L	T	V	H	A	L	K	A	I	L	S	R	V	D	R	N	E	E	C	K
Q	N	G	L	T	V	H	A	L	K	A	I	L	S	R	V	D	R	N	E	E	C	K
Q	N	G	L	T	V	H	A	L	K	A	I	L	S	R	V	D	R	N	E	E	C	K
Q	N	G	L	T	V	H	A	L	K	A	I	L	S	R	V	D	R	N	E	E	C	K
Q	N	G	L	T	V	H	A	L	K	A	I	L	S	R	V	D	R	N	E	E	C	K
Q	N	G	L	T	V	H	A	L	K	A	I	L	S	R	V	D	R	N	E	E	C	K
Q	N	G	L	T	V	H	A	L	K	A	I	L	S	R	V	D	R	N	E	E	C	K
Q	N	G	L	T	V	H	A	L	K	A	I	L	S	R	V	D	R	N	E	E	C	K
Q	N	G	L	T	V	H	A	L	K	A	I	L	S	R	V	D	R	N	E	E	C	K
Q	N	G	L	T	V	H	A	L	K	A	I	L	S	R	V	D	R	N	E	E	C	K
Q	N	G	L	T	V	H	A	L	K	A	I	L	S	R	V	D	R	N	E	E	C	K
Q	N	G	L	T	V	H	A	L	K	A	I	L	S	R	V	D	R	N	E	E	C	K
Q	N	G	L	T	V	H	A	L	K	A	I	L	S	R	V	D	R	N	E	E	C	K
Q	N	G	L	T	V	H	A	L	K	A	I	L	S	R	V	D	R	N	E	E	C	K
Q	N	G	L	T	V	H	A	L	K	A	I	L	S	R	V	D	R	N	E	E	C	K
Q	N	G	L	T	V	H	A	L	K	A	I	L	S	R	V	D	R	N	E	E	C	K
Q	N	G	L	T	V	H	A	L	K	A	I	L	S	R	V	D	R	N	E	E	C	K
Q	N	G	L	T	V	H	A	L	K	A	I	L	S	R	V	D	R	N	E	E	C	K
Q	N	G	L	T	V	H	A	L	K	A	I	L	S	R	V	D	R	N	E	E	C	K
Q	N	G	L	T	V	H	A	L	K	A	I	L	S	R	V	D	R	N	E	E	C	K
Q	N	G	L	T	V	H	A	L	K	A	I	L	S	R	V	D	R	N	E	E	C	K
Q	N	G	L	T	V	H	A	L	K	A	I	L	S	R	V	D	R	N	E	E	C	K
Q	N	G	L	T	V	H	A	L	K	A	I	L	S	R	V	D	R	N	E	E	C	K
Q	N	G	L	T	V	H	A	L	K	A	I	L	S	R	V	D	R	N	E	E	C	K
Q	N	G	L	T	V	H	A	L	K	A	I	L	S	R	V	D	R	N	E	E	C	K
Q	N	G	L	T	V	H	A	L	K	A	I	L	S	R	V	D	R	N	E	E	C	K
Q	N	G	L	T	V	H	A	L	K	A	I	L	S	R	V	D	R	N	E	E	C	K
Q	N	G	L	T	V	H	A	L	K	A	I	L	S	R	V	D	R	N	E	E	C	K
Q	N	G	L	T	V	H	A	L	K	A	I	L	S	R	V	D	R	N	E	E	C	K
Q	N	G	L	T	V	H	A	L	K	A	I	L	S	R	V	D	R	N	E	E	C	K
Q	N	G	L	T	V	H	A	L	K	A	I	L	S	R	V	D	R	N	E	E	C	K
Q	N	G	L	T	V	H	A	L	K	A	I	L	F	R	V	D	R	N	E	E	C	K
Q	N	G	L	T	V	H	A	L	K	A	I	L	S	R	V	D	R	N	E	E	C	K
Q	N	G	L	T	V	H	A	L	K	A	I	L	S	R	V	D	R	N	E	E	C	K
Q	N	G	L	T	V	H	A	L	K	A	I	L	S	R	V	D	R	N	E	E	C	K
Q	N	G	L	T	V	H	A	L	K	A	I	L	S	R	V	D	R	N	E	E	C	K
Q	N	G	L	T	V	H	A	L	K	A	I	L	S	R	V	D	R	N	E	E	C	K
Q	N	G	L	T	V	H	A	L	K	A	I	L	S	R	V	D	R	N	E	E	C	K
Q	N	G	L	T	V	H	A	L	K	A	I	L	S	R	V	D	R	N	E	E	C	K
Q	N	G	L	T	V	H	A	L	K	A	I	L	S	R	V	D	R	N	E	E	C	K
Q	N	G	L	T	V	H	A	L	K	A	I	L	S	R	V	D	R	N	E	E	C	K
Q	N	G	L	T	V	H	A	L	K	A	I	L	S	R	V	D	R	N	E	E	C	K
Q	N	G	L	T	V	H	A	L	K	A	I	L	S	R	V	D	R	N	E	E	C	K
Q	N	G	L	T	V	H	A	L	K	A	I	L	S	R	V	D	R	N	E	E	C	K
Q	N	G	L	T	V	H	A	L	K	A	I	L	S	R	V	D	R	N	E	E	C	K
Q	N	G	L	T	V	H	A	L	K	A	I	L	S	R	V	D	R	N	E	E	C	K
Q	N	G	L	T	V	H	A	L	K	A	I	L	S	R	V	D	R	N	E	E	C	K

wcyK											
77	78	104	157	167	204	212	220	288	303	340	343
I	I	V	K	R	C	K	I	E	G	K	G
L	M	I	E	K	R	R	L	K	E	R	G
L	M	I	E	K	R	R	L	K	E	R	G
L	M	I	E	K	R	R	L	K	E	R	G
L	M	I	E	K	R	R	L	K	E	R	G
L	M	I	E	K	R	R	L	K	E	R	G
L	M	I	E	K	R	R	L	K	E	R	G
L	M	I	E	K	R	R	L	K	E	R	G
L	M	I	E	K	R	R	L	K	E	R	G
L	M	I	E	K	R	R	L	K	E	R	G
L	M	I	E	K	R	R	L	K	E	R	G
L	M	I	E	K	R	R	L	K	E	R	G
L	M	I	E	K	R	R	L	K	E	R	G
L	M	I	E	K	R	R	L	K	E	R	G
L	M	I	E	K	R	R	L	K	E	R	G
L	M	I	E	K	R	R	L	K	E	R	G
L	M	I	E	K	R	R	L	K	E	R	G
L	M	I	E	K	R	R	L	K	E	R	G
L	M	I	E	K	R	R	L	K	E	R	G
L	M	I	E	K	R	R	L	K	E	R	G
L	M	I	E	K	R	R	L	K	E	R	G
L	M	I	E	K	R	R	L	K	E	R	G
L	M	I	E	K	R	R	L	K	E	R	G
L	M	I	E	K	R	R	L	K	E	R	G
L	M	I	E	K	R	R	L	K	E	R	G
L	M	I	E	K	R	R	L	K	E	R	G
L	M	I	E	K	R	R	L	K	E	R	G
L	M	I	E	K	R	R	L	K	E	R	G
L	M	I	E	K	R	R	L	K	E	R	G
L	M	I	E	K	R	R	L	K	E	R	G
L	M	I	E	K	R	R	L	K	E	R	G
L	M	I	E	K	R	R	L	K	E	R	D
L	M	I	E	K	R	R	L	K	E	R	G
L	M	I	E	K	R	R	L	K	E	R	G
L	M	I	E	K	R	R	L	K	E	R	G
L	M	I	E	K	R	R	L	K	E	R	G
L	M	I	E	K	R	R	L	K	E	R	G
L	M	I	E	K	R	R	L	K	E	R	G
L	M	I	E	K	R	R	L	K	E	R	G
L	M	I	E	K	R	R	L	K	E	R	G
L	M	I	E	K	R	R	L	K	E	R	G
L	M	I	E	K	R	R	L	K	E	R	G
L	M	I	E	K	R	R	L	K	E	R	G
L	M	I	E	K	R	R	L	K	E	R	G
L	M	I	E	K	R	R	L	K	E	R	G
L	M	I	E	K	R	R	L	K	E	R	G
L	M	I	E	K	R	R	L	K	E	R	G
L	M	I	E	K	R	R	L	K	E	R	G
L	M	I	E	K	R	R	L	K	E	R	G
L	M	I	E	K	R	R	L	K	E	R	G

wcwC									
37	98	127	131	137	138	139	144	151	156
R	R	K	L	A	N	Q	E	N	E
K	K	N	I	S	S	M	K	K	K
K	K	N	I	S	S	M	K	K	K
K	K	N	I	S	S	M	K	K	K
K	K	N	I	S	S	M	K	K	K
K	K	N	I	S	S	M	K	K	K
K	K	N	I	S	S	M	K	K	K
K	K	N	I	S	S	M	K	K	K
K	K	N	I	S	S	M	K	K	K
K	K	N	I	S	S	M	K	K	K
K	K	N	I	S	S	M	K	K	K
K	K	N	I	S	S	M	K	K	K
K	K	N	I	S	-	-	-	-	-
K	K	N	I	S	S	M	K	K	K
K	K	N	I	S	S	M	K	K	K
K	K	N	I	S	S	M	K	K	K
K	K	N	I	S	S	M	K	K	K
K	K	N	I	S	S	M	K	K	K
K	K	N	I	S	S	M	K	K	K
K	K	N	I	S	S	M	K	K	K
K	K	N	I	S	S	M	K	K	K
K	K	N	I	S	S	M	K	K	K
K	K	N	I	S	S	M	K	K	K
K	K	N	I	S	S	M	K	K	K
K	K	N	I	S	S	M	K	K	K
K	K	N	I	S	S	M	K	K	K
K	K	N	I	S	S	M	K	K	K
K	K	N	I	S	S	M	K	K	K
K	K	N	I	S	S	M	K	K	K
K	K	N	I	S	S	M	K	K	K
K	K	N	I	S	S	M	K	K	K
K	K	N	I	S	S	M	K	K	K
K	K	N	I	S	S	M	K	K	K
K	K	N	I	S	S	M	K	K	K
K	K	N	I	S	S	M	K	K	K
K	K	N	I	S	S	M	K	K	K
K	K	N	I	S	S	M	K	K	K
K	K	N	I	S	S	M	K	K	K
K	K	N	I	S	S	M	K	K	K
K	K	N	I	S	S	M	K	K	K
K	K	N	I	S	S	M	K	K	K
K	K	N	I	S	S	M	K	K	K
K	K	N	I	S	S	M	K	K	K
K	K	N	I	S	S	M	K	K	K
K	K	N	I	S	S	M	K	K	K
K	K	N	I	S	S	M	K	K	K
K	K	N	I	S	S	M	K	K	K
K	K	N	I	S	S	M	K	K	K
K	K	N	I	S	S	M	K	K	K
K	K	N	I	S	S	M	K	K	K

227	230	231	234	239	259	263	264	268	270	278	282	285	296	308	323	327	342	352	409	449	451
A	-	-	R	R	M	R	I	V	S	G	I	V	F	H	D	E	D	H	V	L	K
A	C	I	H	C	V	R	V	I	S	S	I	V	I	H	E	E	H	R	V	L	K
A	C	I	H	C	V	R	V	I	S	S	I	V	I	H	E	E	H	R	V	L	K
A	-	-	R	R	V	W	I	V	S	G	I	V	F	H	D	E	D	H	V	L	K
T	R	S	R	R	V	R	I	V	A	G	V	V	I	H	D	K	D	R	V	L	K
A	R	S	R	R	V	R	I	V	A	G	I	L	I	Q	A	E	D	R	I	F	R

A	R	S	R	R	V	R	I	V	A	G	I	L	I	Q	A	E	D	R	I	F	R
A	R	S	R	R	V	R	I	V	A	G	I	L	I	Q	A	E	D	R	I	F	R
A	R	S	R	R	V	R	I	V	A	G	I	L	I	Q	A	E	D	R	I	F	R
A	R	S	R	R	V	R	I	V	A	G	I	L	I	Q	A	E	D	R	I	F	R
A	R	S	R	R	V	R	I	V	A	G	I	L	I	Q	A	E	D	R	I	F	R
A	R	S	R	R	V	R	I	V	A	G	I	L	I	Q	A	E	D	R	I	F	R
A	R	S	R	R	V	R	I	V	A	G	I	L	I	Q	A	E	D	R	I	F	R
A	R	S	R	R	V	R	I	V	A	G	I	L	I	Q	A	E	D	R	I	F	R
A	R	S	R	R	V	R	I	V	A	G	I	L	I	Q	A	E	D	R	I	F	R
A	R	S	R	R	V	R	I	V	A	G	I	L	I	Q	A	E	D	R	I	F	R
A	R	S	R	R	V	R	I	V	A	G	I	L	I	Q	A	E	D	R	I	F	R
A	R	S	R	R	V	R	I	V	A	G	I	L	I	Q	A	E	D	R	I	F	R
A	R	S	R	R	V	R	I	V	A	G	I	L	I	Q	A	E	D	R	I	F	R
A	R	S	R	R	V	R	I	V	A	G	I	L	I	Q	A	E	D	R	I	F	R
A	R	S	R	R	V	R	I	V	A	G	I	L	I	Q	A	E	D	R	I	F	R
A	R	S	R	R	V	R	I	V	A	G	I	L	I	Q	A	E	D	R	I	F	R
A	R	S	R	R	V	R	I	V	A	G	I	L	I	Q	A	E	D	R	I	F	R
A	R	S	R	R	V	R	I	V	A	G	I	L	I	Q	A	E	D	R	I	F	R
A	R	S	R	R	V	R	I	V	A	G	I	L	I	Q	A	E	D	R	I	F	R
A	R	S	R	R	V	R	I	V	A	G	I	L	I	Q	A	E	D	R	I	F	R
A	R	S	R	R	V	R	I	V	A	G	I	L	I	Q	A	E	D	R	I	F	R
A	R	S	R	R	V	R	I	V	A	G	I	L	I	Q	A	E	D	R	I	F	R
A	R	S	R	R	V	R	I	V	A	G	I	L	I	Q	A	E	D	R	I	F	R
A	R	S	R	R	V	R	I	V	A	G	I	L	I	Q	A	E	D	R	I	F	R
A	R	S	R	R	V	R	I	V	A	G	I	L	I	Q	A	E	D	R	I	F	R
A	R	S	R	R	V	R	I	V	A	G	I	L	I	Q	A	E	D	R	I	F	R
A	R	S	R	R	V	R	I	V	A	G	I	L	I	Q	A	E	D	R	I	F	R
A	R	S	R	R	V	R	I	V	A	G	I	L	I	Q	A	E	D	R	I	F	R
A	R	S	R	R	V	R	I	V	A	G	I	L	I	Q	A	E	D	R	I	F	R
A	R	S	R	R	V	R	I	V	A	G	I	L	I	Q	A	E	D	R	I	F	R
A	R	S	R	R	V	R	I	V	A	G	I	L	I	Q	A	E	D	R	I	F	R
A	R	S	R	R	V	R	I	V	A	G	I	L	I	Q	A	E	D	R	I	F	R
A	R	S	R	R	V	R	I	V	A	G	I	L	I	Q	A	E	D	R	I	F	R
A	R	S	R	R	V	R	I	V	A	G	I	L	I	Q	A	E	D	R	I	F	R
A	R	S	R	R	V	R	I	V	A	G	I	L	I	Q	A	E	D	R	I	F	R
A	R	S	R	R	V	R	I	V	A	G	I	L	I	Q	A	E	D	R	I	F	R
A	R	S	R	R	V	R	I	V	A	G	I	L	I	Q	A	E	D	R	I	F	R
A	R	S	R	R	V	R	I	V	A	G	I	L	I	Q	A	E	D	R	I	F	R
A	R	S	R	R	V	R	I	V	A	G	I	L	I	Q	A	E	D	R	I	F	R
A	R	S	R	R	V	R	I	V	A	G	I	L	I	Q	A	E	D	R	I	F	R
A	R	S	R	R	V	R	I	V	A	G	I	L	I	Q	A	E	D	R	I	F	R
A	R	S	R	R	V	R	I	V	A	G	I	L	I	Q	A	E	D	R	I	F	R
A	R	S	R	R	V	R	I	V	A	G	I	L	I	Q	A	E	D	R	I	F	R
A	R	S	R	R	V	R	I	V	A	G	I	L	I	Q	A	E	D	R	I	F	R
A	R	S	R	R	V	R	I	V	A	G	I	L	I	Q	A	E	D	R	I	F	R
A	R	S	R	R	V	R	I	V	A	G	I	L	I	Q	A	E	D	R	I	F	R
A	R	S	R	R	V	R	I	V	A	G	I	L	I	Q	A	E	D	R	I	F	R
A	R	S	R	R	V	R	I	V	A	G	I	L	I	Q	A	E	D	R	I	F	R

205	212	218	219	17	28	47	54	58	59	74	88	91	94	97	99	119	123	124	131	132	136
R	T	D	Q	S	K	E	Q	R	D	I	V	N	K	E	K	S	T	S	N	L	I
I	A	N	K	P	R	Q	K	K	N	V	I	K	L	D	T	A	S	F	D	S	V
I	A	N	K	P	R	Q	K	K	N	V	I	K	L	D	T	A	S	F	D	S	V
I	A	N	K	P	R	Q	K	K	N	V	I	K	L	D	T	A	S	F	D	S	V
I	A	N	K	P	R	Q	K	K	N	V	I	K	L	D	T	A	S	F	D	S	V
I	A	N	K	P	R	Q	K	K	N	V	I	K	L	D	T	A	S	F	D	S	V
I	A	N	K	P	R	Q	K	K	N	V	I	K	L	D	T	A	S	F	D	S	V
I	A	N	K	P	R	Q	K	K	N	V	I	K	L	D	T	A	S	F	D	S	V
I	A	N	K	P	R	Q	K	K	N	V	I	K	L	D	T	A	S	F	D	S	V
I	A	N	K	P	R	Q	K	K	N	V	I	K	L	D	T	A	S	F	D	S	V
I	A	N	K	P	R	Q	K	K	N	V	I	K	L	D	T	A	S	F	D	S	V
I	A	N	K	P	R	Q	K	K	N	V	I	K	L	D	T	A	S	F	D	S	V
-	-	-	-	P	R	Q	K	K	N	V	I	K	L	D	T	A	S	F	D	S	V
I	A	N	K	P	R	Q	K	K	N	V	I	K	L	D	T	A	S	F	D	S	V
I	A	N	K	P	R	Q	K	K	N	V	I	K	L	D	T	A	S	F	D	S	V
I	A	N	K	P	R	Q	K	K	N	V	I	K	L	D	T	A	S	F	D	S	V
I	A	N	K	P	R	Q	K	K	N	V	I	K	L	D	T	A	S	F	D	S	V
I	A	N	K	P	R	Q	K	K	N	V	I	K	L	D	T	A	S	F	D	S	V
I	A	N	K	P	R	Q	K	K	N	V	I	K	L	D	T	A	S	F	D	S	V
I	A	N	K	P	R	Q	K	K	N	V	I	K	L	D	T	A	S	F	D	S	V
I	A	N	K	P	R	Q	K	K	N	V	I	K	L	D	T	A	S	F	D	S	V
I	A	N	K	P	R	Q	K	K	N	V	I	K	L	D	T	A	S	F	D	S	V
I	A	N	K	P	R	Q	K	K	N	V	I	K	L	D	T	A	S	F	D	S	V
I	A	N	K	P	R	Q	K	K	N	V	I	K	L	D	T	A	S	F	D	S	V
I	A	N	K	P	R	Q	K	K	N	V	I	K	L	D	T	A	S	F	D	S	V
I	A	N	K	P	R	Q	K	K	N	V	I	K	L	D	T	A	S	F	D	S	V
I	A	N	K	P	R	Q	K	K	N	V	I	K	L	D	T	A	S	F	D	S	V
I	A	N	K	P	R	Q	K	K	N	V	I	K	L	D	T	A	S	F	D	S	V
I	A	N	K	P	R	Q	K	K	N	V	I	K	L	D	T	A	S	F	D	S	V
I	A	N	K	P	R	Q	K	K	N	V	I	K	L	D	T	A	S	F	D	S	V
I	A	N	K	P	R	Q	K	K	N	V	I	K	L	D	T	A	S	F	D	S	V
I	A	N	K	P	R	Q	K	K	N	V	I	K	L	D	T	A	S	F	D	S	V
I	A	N	K	P	R	Q	K	K	N	V	I	K	L	D	T	A	S	F	D	S	V
I	A	N	K	P	R	Q	K	K	N	V	I	K	L	D	T	A	S	F	D	S	V
I	A	N	K	P	R	Q	K	K	N	V	I	K	L	D	T	A	S	F	D	S	V
I	A	N	K	P	R	Q	K	K	N	V	I	K	L	D	T	A	S	F	D	S	V
I	A	N	K	P	R	Q	K	K	N	V	I	K	L	D	T	A	S	F	D	S	V
I	A	N	K	P	R	Q	K	K	N	V	I	K	L	D	T	A	S	F	D	S	V
I	A	N	K	P	R	Q	K	K	N	V	I	K	L	D	T	A	S	F	D	S	V
I	A	N	K	P	R	Q	K	K	N	V	I	K	L	D	T	A	S	F	D	S	V
I	A	N	K	P	R	Q	K	K	N	V	I	K	L	D	T	A	S	F	D	S	V
I	A	N	K	P	R	Q	K	K	N	V	I	K	L	D	T	A	S	F	D	S	V
I	A	N	K	P	R	Q	K	K	N	V	I	K	L	D	T	A	S	F	D	S	V
I	A	N	K	P	R	Q	K	K	N	V	I	K	L	D	T	A	S	F	D	S	V
I	A	N	K	P	R	Q	K	K	N	V	I	K	L	D	T	A	S	F	D	S	V
I	A	N	K	P	R	Q	K	K	N	V	I	K	L	D	T	A	S	F	D	S	V
I	A	N	K	P	R	Q	K	K	N	V	I	K	L	D	T	A	S	F	D	S	V
I	A	N	K	P	R	Q	K	K	N	V	I	K	L	D	T	A	S	F	D	S	V
I	A	N	K	P	R	Q	K	K	N	V	I	K	L	D	T	A	S	F	D	S	V

wchJ																						
36	38	41	43	44	46	47	51	58	66	69	70	73	79	87	94	100	113	116	120	127	133	138
E	T	I	G	N	T	F	H	L	V	F	N	R	V	V	L	A	A	L	I	K	E	V
E	A	I	G	N	T	F	H	L	I	F	N	R	V	V	L	A	T	L	L	K	D	V
E	A	I	G	N	T	F	H	L	I	F	N	R	V	V	L	A	T	L	L	K	D	V
E	T	I	G	N	T	F	H	L	V	F	N	R	V	V	L	A	A	L	I	K	E	V
A	A	V	S	E	V	V	Y	I	F	Y	K	K	L	I	I	S	A	I	L	K	E	I
A	A	V	S	E	V	V	Y	I	F	Y	K	K	L	I	I	S	A	I	L	R	E	I

A	A	V	S	E	V	V	Y	I	F	Y	K	K	L	I	I	S	A	I	L	R	E	I
A	A	V	S	E	V	V	Y	I	F	Y	K	K	L	I	I	S	A	I	L	R	E	I
A	A	V	S	E	V	V	Y	I	F	Y	K	K	L	I	I	S	A	I	L	R	E	I
A	A	V	S	E	V	V	Y	I	F	Y	K	K	L	I	I	S	A	I	L	R	E	I
A	A	V	S	E	V	V	Y	I	F	Y	K	K	L	I	I	S	A	I	L	R	E	I
A	A	V	S	E	V	V	Y	I	F	Y	K	K	L	I	I	S	A	I	L	R	E	I
A	A	V	S	E	V	V	Y	I	F	Y	K	K	L	I	I	S	A	I	L	R	E	I
A	A	V	S	E	V	V	Y	I	F	Y	K	K	L	I	I	S	A	I	L	R	E	I
A	A	V	S	E	V	V	Y	I	F	Y	K	K	L	I	I	S	A	I	L	R	E	I
A	A	V	S	E	V	V	Y	I	F	Y	K	K	L	I	I	S	A	I	L	R	E	I
A	A	V	S	E	V	V	Y	I	F	Y	K	K	L	I	I	S	A	I	L	R	E	I
A	A	V	S	E	V	V	Y	I	F	Y	K	K	L	I	I	S	A	I	L	R	E	I
A	A	V	S	E	V	V	Y	I	F	Y	K	K	L	I	I	S	A	I	L	R	E	I
A	A	V	S	E	V	V	Y	I	F	Y	K	K	L	I	I	S	A	I	L	R	E	I
A	A	V	S	E	V	V	Y	I	F	Y	K	K	L	I	I	S	A	I	L	R	E	I
A	A	V	S	E	V	V	Y	I	F	Y	K	K	L	I	I	S	A	I	L	R	E	I
A	A	V	S	E	V	V	Y	I	F	Y	K	K	L	I	I	S	A	I	L	R	E	I
A	A	V	S	E	V	V	Y	I	F	Y	K	K	L	I	I	S	A	I	L	R	E	I
A	A	V	S	E	V	V	Y	I	F	Y	K	K	L	I	I	S	A	I	L	R	E	I
A	A	V	S	E	V	V	Y	I	F	Y	K	K	L	I	I	S	A	I	L	R	E	I
A	A	V	S	E	V	V	Y	I	F	Y	K	K	L	I	I	S	A	I	L	R	E	I
A	A	V	S	E	V	V	Y	I	F	Y	K	K	L	I	I	S	A	I	L	R	E	I
A	A	V	S	E	V	V	Y	I	F	Y	K	K	L	I	I	S	A	I	L	R	E	I
A	A	V	S	E	V	V	Y	I	F	Y	K	K	L	I	I	S	A	I	L	R	E	I
A	A	V	S	E	V	V	Y	I	F	Y	K	K	L	I	I	S	A	I	L	R	E	I
A	A	V	S	E	V	V	Y	I	F	Y	K	K	L	I	I	S	A	I	L	R	E	I
A	A	V	S	E	V	V	Y	I	F	Y	K	K	L	I	I	S	A	I	L	R	E	I
A	A	V	S	E	V	V	Y	I	F	Y	K	K	L	I	I	S	A	I	L	R	E	I
A	A	V	S	E	V	V	Y	I	F	Y	K	K	L	I	I	S	A	I	L	R	E	I
A	A	V	S	E	V	V	Y	I	F	Y	K	K	L	I	I	S	A	I	L	R	E	I
A	A	V	S	E	V	V	Y	I	F	Y	K	K	L	I	I	S	A	I	L	R	E	I
A	A	V	S	E	V	V	Y	I	F	Y	K	K	L	I	I	S	A	I	L	R	E	I
A	A	V	S	E	V	V	Y	I	F	Y	K	K	L	I	I	S	A	I	L	R	E	I
A	A	V	S	E	V	V	Y	I	F	Y	K	K	L	I	I	S	A	I	L	R	E	I
A	A	V	S	E	V	V	Y	I	F	Y	K	K	L	I	I	S	A	I	L	R	E	I
A	A	V	S	E	V	V	Y	I	F	Y	K	K	L	I	I	S	A	I	L	R	E	I
A	A	V	S	E	V	V	Y	I	F	Y	K	K	L	I	I	S	A	I	L	R	E	I
A	A	V	S	E	V	V	Y	I	F	Y	K	K	L	I	I	S	A	I	L	R	E	I
A	A	V	S	E	V	V	Y	I	F	Y	K	K	L	I	I	S	A	I	L	R	E	I
A	A	V	S	E	V	V	Y	I	F	Y	K	K	L	I	I	S	A	I	L	R	E	I
A	A	V	S	E	V	V	Y	I	F	Y	K	K	L	I	I	S	A	I	L	R	E	I
A	A	V	S	E	V	V	Y	I	F	Y	K	K	L	I	I	S	A	I	L	R	E	I
A	A	V	S	E	V	V	Y	I	F	Y	K	K	L	I	I	S	A	I	L	R	E	I
A	A	V	S	E	V	V	Y	I	F	Y	K	K	L	I	I	S	A	I	L	R	E	I
A	A	V	S	E	V	V	Y	I	F	Y	K	K	L	I	I	S	A	I	L	R	E	I
A	A	V	S	E	V	V	Y	I	F	Y	K	K	L	I	I	S	A	I	L	R	E	I
A	A	V	S	E	V	V	Y	I	F	Y	K	K	L	I	I	S	A	I	L	R	E	I
A	A	V	S	E	V	V	Y	I	F	Y	K	K	L	I	I	S	A	I	L	R	E	I

wcrL																				
137	140	141	154	155	158	162	165	166	168	170	171	189	191	205	212	214	215	217	228	229
E	L	S	P	V	R	T	D	C	V	N	E	G	E	S	R	Q	M	S	I	L
K	I	N	Q	I	Q	V	N	S	I	S	Q	E	A	N	M	R	V	R	V	S
K	I	N	Q	I	Q	V	N	S	I	S	Q	E	A	N	M	R	V	R	V	S
K	I	N	Q	I	Q	V	N	S	I	S	Q	E	A	N	M	R	V	R	V	S
K	I	N	Q	I	Q	V	N	S	I	S	Q	E	A	N	M	R	V	R	V	S
K	I	N	Q	I	Q	V	N	S	I	S	Q	E	A	N	M	R	V	R	V	S
K	I	N	Q	I	Q	V	N	S	I	S	Q	E	A	N	M	R	V	R	V	S
K	I	N	Q	I	Q	V	N	S	I	S	Q	E	A	N	M	R	V	R	V	S
K	I	N	Q	I	Q	V	N	S	I	S	Q	E	A	N	M	R	V	R	V	S
K	I	N	Q	I	Q	V	N	S	I	S	Q	E	A	N	M	R	V	R	V	S
K	I	N	Q	I	Q	V	N	S	I	S	Q	E	A	N	M	R	V	R	V	S
K	I	N	Q	I	Q	V	N	S	I	S	Q	E	A	N	M	R	V	R	V	S
K	I	N	Q	I	Q	V	N	S	I	S	Q	E	A	N	M	R	V	R	V	S
K	I	N	Q	I	Q	V	N	S	I	S	Q	E	A	N	M	R	V	R	V	S
K	I	N	Q	I	Q	V	N	S	I	S	Q	E	A	N	M	R	V	R	V	S
K	I	N	Q	I	Q	V	N	S	I	S	Q	E	A	N	M	R	V	R	V	S
K	I	N	Q	I	Q	V	N	S	I	S	Q	E	A	N	M	R	V	R	V	S
K	I	N	Q	I	Q	V	N	S	I	S	Q	E	A	N	M	R	V	R	V	S
K	I	N	Q	I	Q	V	N	S	I	S	Q	E	A	N	M	R	V	R	V	S
K	I	N	Q	I	Q	V	N	S	I	S	Q	E	A	N	M	R	V	R	V	S
K	I	N	Q	I	Q	V	N	S	I	S	Q	E	A	N	M	R	V	R	V	S
K	I	N	Q	I	Q	V	N	S	I	S	Q	E	A	N	M	R	V	R	V	S
K	I	N	Q	I	Q	V	N	S	I	S	Q	E	A	N	M	R	V	R	V	S
K	I	N	Q	I	Q	V	N	S	I	S	Q	E	A	N	M	R	V	R	V	S
K	I	N	Q	I	Q	V	N	S	I	S	Q	E	A	N	M	R	V	R	V	S
K	I	N	Q	I	Q	V	N	S	I	S	Q	E	A	N	M	R	V	R	V	S
K	I	N	Q	I	Q	V	N	S	I	S	Q	E	A	N	M	R	V	R	V	S
K	I	N	Q	I	Q	V	N	S	I	S	Q	E	A	N	M	R	V	R	V	S
K	I	N	Q	I	Q	V	N	S	I	S	Q	E	A	N	M	R	V	R	V	S
K	I	N	Q	I	Q	V	N	S	I	S	Q	E	A	N	M	R	V	R	V	S
K	I	N	Q	I	Q	V	N	S	I	S	Q	E	A	N	M	R	V	R	V	S
K	I	N	Q	I	Q	V	N	S	I	S	Q	E	A	N	M	R	V	R	V	S
K	I	N	Q	I	Q	V	N	S	I	S	Q	E	A	N	M	R	V	R	V	S
K	I	N	Q	I	Q	V	N	S	I	S	Q	E	A	N	M	R	V	R	V	S
K	I	N	Q	I	Q	V	N	S	I	S	Q	E	A	N	M	R	V	R	V	S
K	I	N	Q	I	Q	V	N	S	I	S	Q	E	A	N	M	R	V	R	V	S
K	I	N	Q	I	Q	V	N	S	I	S	Q	E	A	N	M	R	V	R	V	S
K	I	N	Q	I	Q	V	N	S	I	S	Q	E	A	N	M	R	V	R	V	S
K	I	N	Q	I	Q	V	N	S	I	S	Q	E	A	N	M	R	V	R	V	S
K	I	N	Q	I	Q	V	N	S	I	S	Q	E	A	N	M	R	V	R	V	S
K	I	N	Q	I	Q	V	N	S	I	S	Q	E	A	N	M	R	V	R	V	S
K	I	N	Q	I	Q	V	N	S	I	S	Q	E	A	N	M	R	V	R	V	S
K	I	N	Q	I	Q	V	N	S	I	S	Q	E	A	N	M	R	V	R	V	S
K	I	N	Q	I	Q	V	N	S	I	S	Q	E	A	N	M	R	V	R	V	S
K	I	N	Q	I	Q	V	N	S	I	S	Q	E	A	N	M	R	V	R	V	S
K	I	N	Q	I	Q	V	N	S	I	S	Q	E	A	N	M	R	V	R	V	S
K	I	N	Q	I	Q	V	N	S	I	S	Q	E	A	N	M	R	V	R	V	S
K	I	N	Q	I	Q	V	N	S	I	S	Q	E	A	N	M	R	V	R	V	S
K	I	N	Q	I	Q	V	N	S	I	S	Q	E	A	N	M	R	V	R	V	S

43
N
K
K
K
K
K
K
K
K
K
K
K
K
K
K
K
K
K
K
K
K
K
K
K
K
K
K
K
K
K
K
K
K
K
K
K
K
K
K
K
K
K
K
K
K
K
K
K
K
K

6	14	20	25	27	28	31	32	34	39	41	43	45	46	48	50	51	52	56	57	60	61
I	N	V	G	G	F	D	D	F	F	D	E	V	Y	Q	K	N	L	D	E	R	Y
V	N	V	G	G	L	D	E	I	F	D	E	V	H	Q	K	A	L	D	E	Q	Y
V	N	V	G	G	L	D	E	I	F	D	E	V	H	Q	K	A	L	D	E	Q	Y
I	N	V	G	G	F	D	D	F	F	D	E	V	Y	Q	K	N	L	D	E	R	Y
V	D	V	E	N	L	E	E	F	Y	N	V	K	Y	K	E	K	I	E	K	Q	F
V	D	F	E	N	L	E	E	F	Y	N	V	K	Y	K	E	K	I	E	K	Q	F

V	D	F	E	N	L	E	E	F	Y	N	V	K	Y	K	E	K	I	E	K	Q	F
V	D	F	E	N	L	E	E	F	Y	N	V	K	Y	K	E	K	I	E	K	Q	F
V	D	F	E	N	L	E	E	F	Y	N	V	K	Y	K	E	K	I	E	K	Q	F
V	D	F	E	N	L	E	E	F	Y	N	V	K	Y	K	E	K	I	E	K	Q	F
V	D	F	E	N	L	E	E	F	Y	N	V	K	Y	K	E	K	I	E	K	Q	F
V	D	F	E	N	L	E	E	F	Y	N	V	K	Y	K	E	K	I	E	K	Q	F
V	D	F	E	N	L	E	E	F	Y	N	V	K	Y	K	E	K	I	E	K	Q	F
V	D	F	E	N	L	E	E	F	Y	N	V	K	Y	K	E	K	I	E	K	Q	F
V	D	F	E	N	L	E	E	F	Y	N	V	K	Y	K	E	K	I	E	K	Q	F
V	D	F	E	N	L	E	E	F	Y	N	V	K	Y	K	E	K	I	E	K	Q	F
V	D	F	E	N	L	E	E	F	Y	N	V	K	Y	K	E	K	I	E	K	Q	F
V	D	F	E	N	L	E	E	F	Y	N	V	K	Y	K	E	K	I	E	K	Q	F
V	D	F	E	N	L	E	E	F	Y	N	V	K	Y	K	E	K	I	E	K	Q	F
V	D	F	E	N	L	E	E	F	Y	N	V	K	Y	K	E	K	I	E	K	Q	F
V	D	F	E	N	L	E	E	F	Y	N	V	K	Y	K	E	K	I	E	K	Q	F
V	D	F	E	N	L	E	E	F	Y	N	V	K	Y	K	E	K	I	E	K	Q	F
V	D	F	E	N	L	E	E	F	Y	N	V	K	Y	K	E	K	I	E	K	Q	F
V	D	F	E	N	L	E	E	F	Y	N	V	K	Y	K	E	K	I	E	K	Q	F
V	D	F	E	N	L	E	E	F	Y	N	V	K	Y	K	E	K	I	E	K	Q	F
V	D	F	E	N	L	E	E	F	Y	N	V	K	Y	K	E	K	I	E	K	Q	F
V	D	F	E	N	L	E	E	F	Y	N	V	K	Y	K	E	K	I	E	K	Q	F
V	D	F	E	N	L	E	E	F	Y	N	V	K	Y	K	E	K	I	E	K	Q	F
V	D	F	E	N	L	E	E	F	Y	N	V	K	Y	K	E	K	I	E	K	Q	F
V	D	F	E	N	L	E	E	F	Y	N	V	K	Y	K	E	K	I	E	K	Q	F
V	D	F	E	N	L	E	E	F	Y	N	V	K	Y	K	E	K	I	E	K	Q	F
V	D	F	E	N	L	E	E	F	Y	N	V	K	Y	K	E	K	I	E	K	Q	F
V	D	F	E	N	L	E	E	F	Y	N	V	K	Y	K	E	K	I	E	K	Q	F
V	D	F	E	N	L	E	E	F	Y	N	V	K	Y	K	E	K	I	E	K	Q	F
V	D	F	E	N	L	E	E	F	Y	N	V	K	Y	K	E	K	I	E	K	Q	F
V	D	F	E	N	L	E	E	F	Y	N	V	K	Y	K	E	K	I	E	K	Q	F
V	D	F	E	N	L	E	E	F	Y	N	V	K	Y	K	E	K	I	E	K	Q	F
V	D	F	E	N	L	E	E	F	Y	N	V	K	Y	K	E	K	I	E	K	Q	F
V	D	F	E	N	L	E	E	F	Y	N	V	K	Y	K	E	K	I	E	K	Q	F
V	D	F	E	N	L	E	E	F	Y	N	V	K	Y	K	E	K	I	E	K	Q	F
V	D	F	E	N	L	E	E	F	Y	N	V	K	Y	K	E	K	I	E	K	Q	F
V	D	F	E	N	L	E	E	F	Y	N	V	K	Y	K	E	K	I	E	K	Q	F
V	D	F	E	N	L	E	E	F	Y	N	V	K	Y	K	E	K	I	E	K	Q	F
V	D	F	E	N	L	E	E	F	Y	N	V	K	Y	K	E	K	I	E	K	Q	F
V	D	F	E	N	L	E	E	F	Y	N	V	K	Y	K	E	K	I	E	K	Q	F
V	D	F	E	N	L	E	E	F	Y	N	V	K	Y	K	E	K	I	E	K	Q	F
V	D	F	E	N	L	E	E	F	Y	N	V	K	Y	K	E	K	I	E	K	Q	F
V	D	F	E	N	L	E	E	F	Y	N	V	K	Y	K	E	K	I	E	K	Q	F
V	D	F	E	N	L	E	E	F	Y	N	V	K	Y	K	E	K	I	E	K	Q	F
V	D	F	E	N	L	E	E	F	Y	N	V	K	Y	K	E	K	I	E	K	Q	F
V	D	F	E	N	L	E	E	F	Y	N	V	K	Y	K	E	K	I	E	K	Q	F
V	D	F	E	N	L	E	E	F	Y	N	V	K	Y	K	E	K	I	E	K	Q	F
V	D	F	E	N	L	E	E	F	Y	N	V	K	Y	K	E	K	I	E	K	Q	F
V	D	F	E	N	L	E	E	F	Y	N	V	K	Y	K	E	K	I	E	K	Q	F

wzy																					
44	54	83	89	95	97	132	141	151	165	166	206	213	221	234	286	297	301	310	342	343	345
G	L	I	V	V	L	P	V	I	V	I	V	V	R	V	L	K	V	V	S	T	S
E	S	M	L	D	V	A	I	V	I	L	A	I	K	I	V	E	I	I	P	A	G
E	S	M	L	D	V	A	I	V	I	L	A	I	K	I	V	E	I	I	P	A	G
E	S	M	L	D	V	A	I	V	I	L	A	I	K	I	V	E	I	I	P	A	G
E	S	M	L	D	V	A	I	V	I	L	A	I	K	I	V	E	I	I	P	A	G
E	S	M	L	D	V	A	I	V	I	L	A	I	K	I	V	E	I	I	P	A	G
E	S	M	L	D	V	A	I	V	I	L	A	I	K	I	V	E	I	I	P	A	G
E	S	M	L	D	V	A	I	V	I	L	A	I	K	I	V	E	I	I	P	A	G
E	S	M	L	D	V	A	I	V	I	L	A	I	K	I	V	E	I	I	P	A	G
E	S	M	L	D	V	A	I	V	I	L	A	I	K	I	V	E	I	I	P	A	G
E	S	M	L	D	V	A	I	V	I	L	A	I	K	I	V	E	I	I	P	A	G
E	S	M	L	D	V	A	I	V	I	L	A	I	K	I	V	E	I	I	P	A	G
E	S	M	L	D	V	A	I	V	I	L	A	I	K	I	V	E	I	I	P	A	G
E	S	M	L	D	V	A	I	V	I	L	A	I	K	I	V	E	I	I	P	A	G
E	S	M	L	D	V	A	I	V	I	L	A	I	K	I	V	E	I	I	P	A	G
E	S	M	L	D	V	A	I	V	I	L	A	I	K	I	V	E	I	I	P	A	G
E	S	M	L	D	V	A	I	V	I	L	A	I	K	I	V	E	I	I	P	A	G
E	S	M	L	D	V	A	I	V	I	L	A	I	K	I	V	E	I	I	P	A	G
E	S	M	L	D	V	A	I	V	I	L	A	I	K	I	V	E	I	I	P	A	G
E	S	M	L	D	V	A	I	V	I	L	A	I	K	I	V	E	I	I	P	A	G
E	S	M	L	D	V	A	I	V	I	L	A	I	K	I	V	E	I	I	P	A	G
E	S	M	L	D	V	A	I	V	I	L	A	I	K	I	V	E	I	I	P	A	G
E	S	M	L	D	V	A	I	V	I	L	A	I	K	I	V	E	I	I	P	A	G
E	S	M	L	D	V	A	I	V	I	L	A	I	K	I	V	E	I	I	P	A	G
E	S	M	L	D	V	A	I	V	I	L	A	I	K	I	V	E	I	I	P	A	G
E	S	M	L	D	V	A	I	V	I	L	A	I	K	I	V	E	I	I	P	A	G
E	S	M	L	D	V	A	I	V	I	L	A	I	K	I	V	E	I	I	P	A	G
E	S	M	L	D	V	A	I	V	I	L	A	I	K	I	V	E	I	I	P	A	G
E	S	M	L	D	V	A	I	V	I	L	A	I	K	I	V	E	I	I	P	A	G
E	S	M	L	D	V	A	I	V	I	L	A	I	K	I	V	E	I	I	P	A	G
E	S	M	L	D	V	A	I	V	I	L	A	I	K	I	V	E	I	I	P	A	G
E	S	M	L	D	V	A	I	V	I	L	A	I	K	I	V	E	I	I	P	A	G
E	S	M	L	D	V	A	I	V	I	L	A	I	K	I	V	E	I	I	P	A	G
E	S	M	L	D	V	A	I	V	I	L	A	I	K	I	V	E	I	I	P	A	G
E	S	M	L	D	V	A	I	V	I	L	A	I	K	I	V	E	I	I	P	A	G
E	S	M	L	D	V	A	I	V	I	L	A	I	K	I	V	E	I	I	P	A	G
E	S	M	L	D	V	A	I	V	I	L	A	I	K	I	V	E	I	I	P	A	G
E	S	M	L	D	V	A	I	V	I	L	A	I	K	I	V	E	I	I	P	A	G
E	S	M	L	D	V	A	I	V	I	L	A	I	K	I	V	E	I	I	P	A	G
E	S	M	L	D	V	A	I	V	I	L	A	I	K	I	V	E	I	I	P	A	G
E	S	M	L	D	V	A	I	V	I	L	A	I	K	I	V	E	I	I	P	A	G
E	S	M	L	D	V	A	I	V	I	L	A	I	K	I	V	E	I	I	P	A	G
E	S	M	L	D	V	A	I	V	I	L	A	I	K	I	V	E	I	I	P	A	G
E	S	M	L	D	V	A	I	V	I	L	A	I	K	I	V	E	I	I	P	A	G
E	S	M	L	D	V	A	I	V	I	L	A	I	K	I	V	E	I	I	P	A	G
E	S	M	L	D	V	A	I	V	I	L	A	I	K	I	V	E	I	I	P	A	G
E	S	M	L	D	V	A	I	V	I	L	A	I	K	I	V	E	I	I	P	A	G
E	S	M	L	D	V	A	I	V	I	L	A	I	K	I	V	E	I	I	P	A	G
E	S	M	L	D	V	A	I	V	I	L	A	I	K	I	V	E	I	I	P	A	G

wchK																						
62	63	65	66	67	68	69	79	80	82	86	93	94	106	109	111	112	113	117	119	120	122	123
I	D	A	N	I	I	I	A	V	A	I	L	K	E	S	I	L	S	E	S	L	N	D
M	D	A	D	I	V	I	G	V	S	R	Q	E	E	L	L	L	G	D	V	V	D	E
M	D	A	D	I	V	I	G	V	S	R	Q	E	E	L	L	L	G	D	V	V	D	E
I	D	A	N	I	I	I	A	V	A	I	L	K	E	S	I	L	S	E	S	L	N	D
I	E	S	D	T	I	V	A	I	A	T	Q	K	N	S	I	S	S	E	S	L	N	D
I	E	A	D	T	I	V	A	I	A	T	Q	K	N	S	I	S	S	E	S	L	N	D

I	E	A	D	T	I	V	A	I	A	T	Q	K	N	S	I	S	S	E	S	L	N	D
I	E	A	D	T	I	V	A	I	A	T	Q	K	N	S	I	S	S	E	S	L	N	D
I	E	A	D	T	I	V	A	I	A	T	Q	K	N	S	I	S	S	E	S	L	N	D
I	E	A	D	T	I	V	A	I	A	T	Q	K	N	S	I	S	S	E	S	L	N	D
I	E	A	D	T	I	V	A	I	A	T	Q	K	N	S	I	S	S	E	S	L	N	D
I	E	A	D	T	I	V	A	I	A	T	Q	K	N	S	I	S	S	E	S	L	N	D
I	E	A	D	T	I	V	A	I	A	T	Q	K	N	S	I	S	S	E	S	L	N	D
I	E	A	D	T	I	V	A	I	A	T	Q	K	N	S	I	S	S	E	S	L	N	D
I	E	A	D	T	I	V	A	I	A	T	Q	K	N	S	I	S	S	E	S	L	N	D
I	E	A	D	T	I	V	A	I	A	T	Q	K	N	S	I	S	S	E	S	L	N	D
I	E	A	D	T	I	V	A	I	A	T	Q	K	N	S	I	S	S	E	S	L	N	D
I	E	A	D	T	I	V	A	I	A	T	Q	K	N	S	I	S	S	E	S	L	N	D
I	E	A	D	T	I	V	A	I	A	T	Q	K	N	S	I	S	S	E	S	L	N	D
I	E	A	D	T	I	V	A	I	A	T	Q	K	N	S	I	S	S	E	S	L	N	D
I	E	A	D	T	I	V	A	I	A	T	Q	K	N	S	I	S	S	E	S	L	N	D
I	E	A	D	T	I	V	A	I	A	T	Q	K	N	S	I	S	S	E	S	L	N	D
I	E	A	D	T	I	V	A	I	A	T	Q	K	N	S	I	S	S	E	S	L	N	D
I	E	A	D	T	I	V	A	I	A	T	Q	K	N	S	I	S	S	E	S	L	N	D
I	E	A	D	T	I	V	A	I	A	T	Q	K	N	S	I	S	S	E	S	L	N	D
I	E	A	D	T	I	V	A	I	A	T	Q	K	N	S	I	S	S	E	S	L	N	D
I	E	A	D	T	I	V	A	I	A	T	Q	K	N	S	I	S	S	E	S	L	N	D
I	E	A	D	T	I	V	A	I	A	T	Q	K	N	S	I	S	S	E	S	L	N	D
I	E	A	D	T	I	V	A	I	A	T	Q	K	N	S	I	S	S	E	S	L	N	D
I	E	A	D	T	I	V	A	I	A	T	Q	K	N	S	I	S	S	E	S	L	N	D
I	E	A	D	T	I	V	A	I	A	T	Q	K	N	S	I	S	S	E	S	L	N	D
I	E	A	D	T	I	V	A	I	A	T	Q	K	N	S	I	S	S	E	S	L	N	D
I	E	A	D	T	I	V	A	I	A	T	Q	K	N	S	I	S	S	E	S	L	N	D
I	E	A	D	T	I	V	A	I	A	T	Q	K	N	S	I	S	S	E	S	L	N	D
I	E	A	D	T	I	V	A	I	A	T	Q	K	N	S	I	S	S	E	S	L	N	D
I	E	A	D	T	I	V	A	I	A	T	Q	K	N	S	I	S	S	E	S	L	N	D
I	E	A	D	T	I	V	A	I	A	T	Q	K	N	S	I	S	S	E	S	L	N	D
I	E	A	D	T	I	V	A	I	A	T	Q	K	N	S	I	S	S	E	S	L	N	D
I	E	A	D	T	I	V	A	I	A	T	Q	K	N	S	I	S	S	E	S	L	N	D
I	E	A	D	T	I	V	A	I	A	T	Q	K	N	S	I	S	S	E	S	L	N	D
I	E	A	D	T	I	V	A	I	A	T	Q	K	N	S	I	S	S	E	S	L	N	D
I	E	A	D	T	I	V	A	I	A	T	Q	K	N	S	I	S	S	E	S	L	N	D
I	E	A	D	T	I	V	A	I	A	T	Q	K	N	S	I	S	S	E	S	L	N	D
I	E	A	D	T	I	V	A	I	A	T	Q	K	N	S	I	S	S	E	S	L	N	D
I	E	A	D	T	I	V	A	I	A	T	Q	K	N	S	I	S	S	E	S	L	N	D
I	E	A	D	T	I	V	A	I	A	T	Q	K	N	S	I	S	S	E	S	L	N	D
I	E	A	D	T	I	V	A	I	A	T	Q	K	N	S	I	S	S	E	S	L	N	D
I	E	A	D	T	I	V	A	I	A	T	Q	K	N	S	I	S	S	E	S	L	N	D
I	E	A	D	T	I	V	A	I	A	T	Q	K	N	S	I	S	S	E	S	L	N	D
I	E	A	D	T	I	V	A	I	A	T	Q	K	N	S	I	S	S	E	S	L	N	D
I	E	A	D	T	I	V	A	I	A	T	Q	K	N	S	I	S	S	E	S	L	N	D
I	E	A	D	T	I	V	A	I	A	T	Q	K	N	S	I	S	S	E	S	L	N	D
I	E	A	D	T	I	V	A	I	A	T	Q	K	N	S	I	S	S	E	S	L	N	D
I	E	A	D	T	I	V	A	I	A	T	Q	K	N	S	I	S	S	E	S	L	N	D

wcwT			wcwU																	
26	131	158	43	84	141	193	201	235	242	253	282	284	288	290	332	347	351	5	11	12
G	I	Q	N	V	S	P	N	E	N	A	D	N	T	L	L	L	N	H	K	S
S	A	R	D	S	N	T	D	G	H	V	N	K	I	M	F	M	D	P	R	R
S	A	R	D	S	N	T	D	G	H	V	N	K	I	M	F	M	D	P	R	R
S	A	R	D	S	N	T	D	G	H	V	N	K	I	M	F	M	D	P	R	R
S	A	R	D	S	N	T	D	G	H	V	N	K	I	M	F	M	D	P	R	R
S	A	R	D	S	N	T	D	G	H	V	N	K	I	M	F	M	D	P	R	R
S	A	R	D	S	N	T	D	G	H	V	N	K	I	M	F	M	D	P	R	R
S	A	R	D	S	N	T	D	G	H	V	N	K	I	M	F	M	D	P	R	R
S	A	R	D	S	N	T	D	G	H	V	N	K	I	M	F	M	D	P	R	R
S	A	R	D	S	N	T	D	G	H	V	N	K	I	M	F	M	D	P	R	R
S	A	R	D	S	N	T	D	G	H	V	N	K	I	M	F	M	D	P	R	R
S	A	R	D	S	N	T	D	G	H	V	N	K	I	M	F	M	D	P	R	R
S	A	R	D	S	N	T	D	G	H	V	N	K	I	M	F	M	D	P	R	R
S	A	R	D	S	N	T	D	G	H	V	N	K	I	M	F	M	D	P	R	R
S	A	R	D	S	N	T	D	G	H	V	N	K	I	M	F	M	D	P	R	R
S	A	R	D	S	N	T	D	G	H	V	N	K	I	M	F	M	D	P	R	R
S	A	R	D	S	N	T	D	G	H	V	N	K	I	M	F	M	D	P	R	R
S	A	R	D	S	N	T	D	G	H	V	N	K	I	M	F	M	D	P	R	R
S	A	R	D	S	N	T	D	G	H	V	N	K	I	M	F	M	D	P	R	R
S	A	R	D	S	N	T	D	G	H	V	N	K	I	M	F	M	D	P	R	R
S	A	R	D	S	N	T	D	G	H	V	N	K	I	M	F	M	D	P	R	R
S	A	R	D	S	N	T	D	G	H	V	N	K	I	M	F	M	D	P	R	R
S	A	R	D	S	N	T	D	G	H	V	N	K	I	M	F	M	D	P	R	R
S	A	R	D	S	N	T	D	G	H	V	N	K	I	M	F	M	D	P	R	R
S	A	R	D	S	N	T	D	G	H	V	N	K	I	M	F	M	D	P	R	R
S	A	R	D	S	N	T	D	G	H	V	N	K	I	M	F	M	D	P	R	R
S	A	R	D	S	N	T	D	G	H	V	N	K	I	M	F	M	D	P	R	R
S	A	R	D	S	N	T	D	G	H	V	N	K	I	M	F	M	D	P	R	R
S	A	R	D	S	N	T	D	G	H	V	N	K	I	M	F	M	D	P	R	R
S	A	R	D	S	N	T	D	G	H	V	N	K	I	M	F	M	D	P	R	R
S	A	R	D	S	N	T	D	G	H	V	N	K	I	M	F	M	D	P	R	R
S	A	R	D	S	N	T	D	G	H	V	N	K	I	M	F	M	D	P	R	R
S	A	R	D	S	N	T	D	G	H	V	N	K	I	M	F	M	D	P	R	R
S	A	R	D	S	N	T	D	G	H	V	N	K	I	M	F	M	D	P	R	R
S	A	R	D	S	N	T	D	G	H	V	N	K	I	M	F	M	D	P	R	R
S	A	R	D	S	N	T	D	G	H	V	N	K	I	M	F	M	D	P	R	R
S	A	R	D	S	N	T	D	G	H	V	N	K	I	M	F	M	D	P	R	R
S	A	R	D	S	N	T	D	G	H	V	N	K	I	M	F	M	D	P	R	R
S	A	R	D	S	N	T	D	G	H	V	N	K	I	M	F	M	D	P	R	R
S	A	R	D	S	N	T	D	G	H	V	N	K	I	M	F	M	D	P	R	R
S	A	R	D	S	N	T	D	G	H	V	N	K	I	M	F	M	D	P	R	R
S	A	R	D	S	N	T	D	G	H	V	N	K	I	M	F	M	D	P	R	R
S	A	R	D	S	N	T	D	G	H	V	N	K	I	M	F	M	D	P	R	R
S	A	R	D	S	N	T	D	G	H	V	N	K	I	M	F	M	D	P	R	R
S	A	R	D	S	N	T	D	G	H	V	N	K	I	M	F	M	D	P	R	R
S	A	R	D	S	N	T	D	G	H	V	N	K	I	M	F	M	D	P	R	R
S	A	R	D	S	N	T	D	G	H	V	N	K	I	M	F	M	D	P	R	R
S	A	R	D	S	N	T	D	G	H	V	N	K	I	M	F	M	D	P	R	R
S	A	R	D	S	N	T	D	G	H	V	N	K	I	M	F	M	D	P	R	R

125	126	128	129	130	131	133	134	136	137	138	139	140	142	144	145	147	148	149	152	153	156	157
S	E	Q	Y	S	L	Q	E	K	T	K	F	Y	N	T	C	L	K	K	K	T	D	L
D	K	N	E	N	I	Y	S	V	Q	Q	V	S	H	S	K	T	N	E	D	I	N	M
D	K	N	E	N	I	Y	S	V	Q	Q	V	S	H	S	K	T	N	E	D	I	N	M
S	E	Q	Y	S	L	Q	E	K	T	K	F	Y	N	T	C	L	K	K	K	T	D	L
S	E	Q	Y	S	L	Q	E	K	T	K	F	Y	N	A	N	L	K	Q	K	I	D	L
S	E	Q	Y	S	L	Q	E	K	T	K	F	Y	N	A	N	L	K	Q	K	I	D	L

S	E	Q	Y	S	L	Q	E	K	T	K	F	Y	N	A	N	L	K	Q	K	I	D	L
S	E	Q	Y	S	L	Q	E	K	T	K	F	Y	N	A	N	L	K	Q	K	I	D	L
S	E	Q	Y	S	L	Q	E	K	T	K	F	Y	N	A	N	L	K	Q	K	I	D	L
S	E	Q	Y	S	L	Q	E	K	T	K	F	Y	N	A	N	L	K	Q	K	I	D	L
S	E	Q	Y	S	L	Q	E	K	T	K	F	Y	N	A	N	L	K	Q	K	I	D	L
S	E	Q	Y	S	L	Q	E	K	T	K	F	Y	N	A	N	L	K	Q	K	I	D	L
S	E	Q	Y	S	L	Q	E	K	T	K	F	Y	N	A	N	L	K	Q	K	I	D	L
S	E	Q	Y	S	L	Q	E	K	T	K	F	Y	N	A	N	L	K	Q	K	I	D	L
S	E	Q	Y	S	L	Q	E	K	T	K	F	Y	N	A	N	L	K	Q	K	I	D	L
S	E	Q	Y	S	L	Q	E	K	T	K	F	Y	N	A	N	L	K	Q	K	I	D	L
S	E	Q	Y	S	L	Q	E	K	T	K	F	Y	N	A	N	L	K	Q	K	I	D	L
S	E	Q	Y	S	L	Q	E	K	T	K	F	Y	N	A	N	L	K	Q	K	I	D	L
S	E	Q	Y	S	L	Q	E	K	T	K	F	Y	N	A	N	L	K	Q	K	I	D	L
S	E	Q	Y	S	L	Q	E	K	T	K	F	Y	N	A	N	L	K	Q	K	I	D	L
S	E	Q	Y	S	L	Q	E	K	T	K	F	Y	N	A	N	L	K	Q	K	I	D	L
S	E	Q	Y	S	L	Q	E	K	T	K	F	Y	N	A	N	L	K	Q	K	I	D	L
S	E	Q	Y	S	L	Q	E	K	T	K	F	Y	N	A	N	L	K	Q	K	I	D	L
S	E	Q	Y	S	L	Q	E	K	T	K	F	Y	N	A	N	L	K	Q	K	I	D	L
S	E	Q	Y	S	L	Q	E	K	T	K	F	Y	N	A	N	L	K	Q	K	I	D	L
S	E	Q	Y	S	L	Q	E	K	T	K	F	Y	N	A	N	L	K	Q	K	I	D	L
S	E	Q	Y	S	L	Q	E	K	T	K	F	Y	N	A	N	L	K	Q	K	I	D	L
S	E	Q	Y	S	L	Q	E	K	T	K	F	Y	N	A	N	L	K	Q	K	I	D	L
S	E	Q	Y	S	L	Q	E	K	T	K	F	Y	N	A	N	L	K	Q	K	I	D	L
S	E	Q	Y	S	L	Q	E	K	T	K	F	Y	N	A	N	L	K	Q	K	I	D	L
S	E	Q	Y	S	L	Q	E	K	T	K	F	Y	N	A	N	L	K	Q	K	I	D	L
S	E	Q	Y	S	L	Q	E	K	T	K	F	Y	N	A	N	L	K	Q	K	I	D	L
S	E	Q	Y	S	L	Q	E	K	T	K	F	Y	N	A	N	L	K	Q	K	I	D	L
S	E	Q	Y	S	L	Q	E	K	T	K	F	Y	N	A	N	L	K	Q	K	I	D	L
S	E	Q	Y	S	L	Q	E	K	T	K	F	Y	N	A	N	L	K	Q	K	I	D	L
S	E	Q	Y	S	L	Q	E	K	T	K	F	Y	N	A	N	L	K	Q	K	I	D	L
S	E	Q	Y	S	L	Q	E	K	T	K	F	Y	N	A	N	L	K	Q	K	I	D	L
S	E	Q	Y	S	L	Q	E	K	T	K	F	Y	N	A	N	L	K	Q	K	I	D	L
S	E	Q	Y	S	L	Q	E	K	T	K	F	Y	N	A	N	L	K	Q	K	I	D	L
S	E	Q	Y	S	L	Q	E	K	T	K	F	Y	N	A	N	L	K	Q	K	I	D	L
S	E	Q	Y	S	L	Q	E	K	T	K	F	Y	N	A	N	L	K	Q	K	I	D	L
S	E	Q	Y	S	L	Q	E	K	T	K	F	Y	N	A	N	L	K	Q	K	I	D	L
S	E	Q	Y	S	L	Q	E	K	T	K	F	Y	N	A	N	L	K	Q	K	I	D	L
S	E	Q	Y	S	L	Q	E	K	T	K	F	Y	N	A	N	L	K	Q	K	I	D	L
S	E	Q	Y	S	L	Q	E	K	T	K	F	Y	N	A	N	L	K	Q	K	I	D	L
S	E	Q	Y	S	L	Q	E	K	T	K	F	Y	N	A	N	L	K	Q	K	I	D	L
S	E	Q	Y	S	L	Q	E	K	T	K	F	Y	N	A	N	L	K	Q	K	I	D	L
S	E	Q	Y	S	L	Q	E	K	T	K	F	Y	N	A	N	L	K	Q	K	I	D	L
S	E	Q	Y	S	L	Q	E	K	T	K	F	Y	N	A	N	L	K	Q	K	I	D	L
S	E	Q	Y	S	L	Q	E	K	T	K	F	Y	N	A	N	L	K	Q	K	I	D	L
S	E	Q	Y	S	L	Q	E	K	T	K	F	Y	N	A	N	L	K	Q	K	I	D	L
S	E	Q	Y	S	L	Q	E	K	T	K	F	Y	N	A	N	L	K	Q	K	I	D	L
S	E	Q	Y	S	L	Q	E	K	T	K	F	Y	N	A	N	L	K	Q	K	I	D	L
S	E	Q	Y	S	L	Q	E	K	T	K	F	Y	N	A	N	L	K	Q	K	I	D	L

wzx																		gct		
104	129	165	166	170	181	234	287	298	309	310	314	316	398	444	457	464	467	71	111	30
T	S	T	F	F	A	I	H	C	V	V	I	A	A	I	I	A	L	S	I	S
I	G	I	S	L	V	M	Y	R	I	T	V	V	S	T	F	V	V	P	V	Y
I	G	I	S	L	V	M	Y	R	I	T	V	V	S	T	F	V	V	P	V	Y
I	G	I	S	L	V	M	Y	R	I	T	V	V	S	T	F	V	V	P	V	Y
I	G	I	S	L	V	M	Y	R	I	T	V	V	S	T	F	V	V	P	V	Y
I	G	I	S	L	V	M	Y	R	I	T	V	V	S	T	F	V	V	P	V	Y
I	G	I	S	L	V	M	Y	R	I	T	V	V	S	T	F	V	V	P	V	Y
I	G	I	S	L	V	M	Y	R	I	T	V	V	S	T	F	V	V	P	V	Y
I	G	I	S	L	V	M	Y	R	I	T	V	V	S	T	F	V	V	P	V	Y
I	G	I	S	L	V	M	Y	R	I	T	V	V	S	T	F	V	V	P	V	Y
I	G	I	S	L	V	M	Y	R	I	T	V	V	S	T	F	V	V	P	V	Y
I	G	I	S	L	V	M	Y	R	I	T	V	V	S	T	F	V	V	P	V	Y
I	G	I	S	L	V	M	Y	R	I	T	V	V	S	T	F	V	V	P	V	Y
I	G	I	S	L	V	M	Y	R	I	T	V	V	S	T	F	V	V	P	V	Y
I	G	I	S	L	V	M	Y	R	I	T	V	V	S	T	F	V	V	P	V	Y
I	G	I	S	L	V	M	Y	R	I	T	V	V	S	T	F	V	V	P	V	Y
I	G	I	S	L	V	M	Y	R	I	T	V	V	S	T	F	V	V	P	V	Y
I	G	I	S	L	V	M	Y	R	I	T	V	V	S	T	F	V	V	P	V	Y
I	G	I	S	L	V	M	Y	R	I	T	V	V	S	T	F	V	V	P	V	Y
I	G	I	S	L	V	M	Y	R	I	T	V	V	S	T	F	V	V	P	V	Y
I	G	I	S	L	V	M	Y	R	I	T	V	V	S	T	F	V	V	P	V	Y
I	G	I	S	L	V	M	Y	R	I	T	V	V	S	T	F	V	V	P	V	Y
I	G	I	S	L	V	M	Y	R	I	T	V	V	S	T	F	V	V	P	V	Y
I	G	I	S	L	V	M	Y	R	I	T	V	V	S	T	F	V	V	P	V	Y
I	G	I	S	L	V	M	Y	R	I	T	V	V	S	T	F	V	V	P	V	Y
I	G	I	S	L	V	M	Y	R	I	T	V	V	S	T	F	V	V	P	V	Y
I	G	I	S	L	V	M	Y	R	I	T	V	V	S	T	F	V	V	P	V	Y
I	G	I	S	L	V	M	Y	R	I	T	V	V	S	T	F	V	V	P	V	Y
I	G	I	S	L	V	M	Y	R	I	T	V	V	S	T	F	V	V	P	V	Y
I	G	I	S	L	V	M	Y	R	I	T	V	V	S	T	F	V	V	P	V	Y
I	G	I	S	L	V	M	Y	R	I	T	V	V	S	T	F	V	V	P	V	Y
I	G	I	S	L	V	M	Y	R	I	T	V	V	S	T	F	V	V	P	V	Y
I	G	I	S	L	V	M	Y	R	I	T	V	V	S	T	F	V	V	P	V	Y
I	G	I	S	L	V	M	Y	R	I	T	V	V	S	T	F	V	V	P	V	Y
I	G	I	S	L	V	M	Y	R	I	T	V	V	S	T	F	V	V	P	V	Y
I	G	I	S	L	V	M	Y	R	I	T	V	V	S	T	F	V	V	P	V	Y
I	G	I	S	L	V	M	Y	R	I	T	V	V	S	T	F	V	V	P	V	Y
I	G	I	S	L	V	M	Y	R	I	T	V	V	S	T	F	V	V	P	V	Y
I	G	I	S	L	V	M	Y	R	I	T	V	V	S	T	F	V	V	P	V	Y
I	G	I	S	L	V	M	Y	R	I	T	V	V	S	T	F	V	V	P	V	Y
I	G	I	S	L	V	M	Y	R	I	T	V	V	S	T	F	V	V	P	V	Y
I	G	I	S	L	V	M	Y	R	I	T	V	V	S	T	F	V	V	P	V	Y
I	G	I	S	L	V	M	Y	R	I	T	V	V	S	T	F	V	V	P	V	Y
I	G	I	S	L	V	M	Y	R	I	T	V	V	S	T	F	V	V	P	V	Y
I	G	I	S	L	V	M	Y	R	I	T	V	V	S	T	F	V	V	P	V	Y
I	G	I	S	L	V	M	Y	R	I	T	V	V	S	T	F	V	V	P	V	Y
I	G	I	S	L	V	M	Y	R	I	T	V	V	S	T	F	V	V	P	V	Y
I	G	I	S	L	V	M	Y	R	I	T	V	V	S	T	F	V	V	P	V	Y
I	G	I	S	L	V	M	Y	R	I	T	V	V	S	T	F	V	V	P	V	Y

158	159	26	28	30	32	33	36	41	42	44	45	48	52	55	56	58	59	60	65	66	67
I	V	Y	D	N	Y	D	I	S	P	K	S	E	C	V	K	I	E	I	S	N	L
F	I	C	N	D	W	E	V	I	P	L	E	A	N	I	Q	V	V	L	T	A	I
F	I	C	N	D	W	E	V	I	P	L	E	A	N	I	Q	V	V	L	T	A	I
I	V	Y	D	N	Y	D	I	S	P	K	S	E	C	V	K	I	E	I	S	N	L
I	V	Y	D	N	Y	D	I	P	L	K	S	E	Y	V	K	I	E	I	S	N	L
I	V	Y	D	N	Y	D	I	P	L	K	S	E	Y	V	K	I	E	I	S	N	L

I	V
I	V
I	V
I	V
I	V
I	V
I	V
I	V
I	V
I	V
I	V
I	V
I	V
I	V
I	V
I	V
I	V
I	V
I	V
I	V
I	V
I	V
I	V
I	V
I	V
I	V
I	V
I	V
I	V
I	V
I	V
I	V
I	V
I	V
I	V
I	V
I	V
I	V
I	V
I	V
I	V
I	V
I	V
I	V
I	V
I	V
I	V
I	V

Y	D	N	Y	D	I	P	L	K	S	E	Y	V	K	I	E	I	S	N	L
Y	D	N	Y	D	I	P	L	K	S	E	Y	V	K	I	E	I	S	N	L
Y	D	N	Y	D	I	P	L	K	S	E	Y	V	K	I	E	I	S	N	L
Y	D	N	Y	D	I	P	L	K	S	E	Y	V	K	I	E	I	S	N	L
Y	D	N	Y	D	I	P	L	K	S	E	Y	V	K	I	E	I	S	N	L
Y	D	N	Y	D	I	P	L	K	S	E	Y	V	K	I	E	I	S	N	L
Y	D	N	Y	D	I	P	L	K	S	E	Y	V	K	I	E	I	S	N	L
Y	D	N	Y	D	I	P	L	K	S	E	Y	V	K	I	E	I	S	N	L
Y	D	N	Y	D	I	P	L	K	S	E	Y	V	K	I	E	I	S	N	L
Y	D	N	Y	D	I	P	L	K	S	E	Y	V	K	I	E	I	S	N	L
Y	D	N	Y	D	I	P	L	K	S	E	Y	V	K	I	E	I	S	N	L
Y	D	N	Y	D	I	P	L	K	S	E	Y	V	K	I	E	I	S	N	L
Y	D	N	Y	D	I	P	L	K	S	E	Y	V	K	I	E	I	S	N	L
Y	D	N	Y	D	I	P	L	K	S	E	Y	V	K	I	E	I	S	N	L
Y	D	N	Y	D	I	P	L	K	S	E	Y	V	K	I	E	I	S	N	L
Y	D	N	Y	D	I	P	L	K	S	E	Y	V	K	I	E	I	S	N	L
Y	D	N	Y	D	I	P	L	K	S	E	Y	V	K	I	E	I	S	N	L
Y	D	N	Y	D	I	P	L	K	S	E	Y	V	K	I	E	I	S	N	L
Y	D	N	Y	D	I	P	L	K	S	E	Y	V	K	I	E	I	S	N	L
Y	D	N	Y	D	I	P	L	K	S	E	Y	V	K	I	E	I	S	N	L
Y	D	N	Y	D	I	P	L	K	S	E	Y	V	K	I	E	I	S	N	L
Y	D	N	Y	D	I	P	L	K	S	E	Y	V	K	I	E	I	S	N	L
Y	D	N	Y	D	I	P	L	K	S	E	Y	V	K	I	E	I	S	N	L
Y	D	N	Y	D	I	P	L	K	S	E	Y	V	K	I	E	I	S	N	L
Y	D	N	Y	D	I	P	L	K	S	E	Y	V	K	I	E	I	S	N	L
Y	D	N	Y	D	I	P	L	K	S	E	Y	V	K	I	E	I	S	N	L
Y	D	N	Y	D	I	P	L	K	S	E	Y	V	K	I	E	I	S	N	L
Y	D	N	Y	D	I	P	L	K	S	E	Y	V	K	I	E	I	S	N	L
Y	D	N	Y	D	I	P	L	K	S	E	Y	V	K	I	E	I	S	N	L
Y	D	N	Y	D	I	P	L	K	S	E	Y	V	K	I	E	I	S	N	L
Y	D	N	Y	D	I	P	L	K	S	E	Y	V	K	I	E	I	S	N	L
Y	D	N	Y	D	I	P	L	K	S	E	Y	V	K	I	E	I	S	N	L
Y	D	N	Y	D	I	P	L	K	S	E	Y	V	K	I	E	I	S	N	L
Y	D	N	Y	D	I	P	L	K	S	E	Y	V	K	I	E	I	S	N	L
Y	D	N	Y	D	I	P	L	K	S	E	Y	V	K	I	E	I	S	N	L
Y	D	N	Y	D	I	P	L	K	S	E	Y	V	K	I	E	I	S	N	L
Y	D	N	Y	D	I	P	L	K	S	E	Y	V	K	I	E	I	S	N	L
Y	D	N	Y	D	I	P	L	K	S	E	Y	V	K	I	E	I	S	N	L
Y	D	N	Y	D	I	P	L	K	S	E	Y	V	K	I	E	I	S	N	L
Y	D	N	Y	D	I	P	L	K	S	E	Y	V	K	I	E	I	S	N	L
Y	D	N	Y	D	I	P	L	K	S	E	Y	V	K	I	E	I	S	N	L
Y	D	N	Y	D	I	P	L	K	S	E	Y	V	K	I	E	I	S	N	L
Y	D	N	Y	D	I	P	L	K	S	E	Y	V	K	I	E	I	S	N	L
Y	D	N	Y	D	I	P	L	K	S	E	Y	V	K	I	E	I	S	N	L
Y	D	N	Y	D	I	P	L	K	S	E	Y	V	K	I	E	I	S	N	L
Y	D	N	Y	D	I	P	L	K	S	E	Y	V	K	I	E	I	S	N	L
Y	D	N	Y	D	I	P	L	K	S	E	Y	V	K	I	E	I	S	N	L
Y	D	N	Y	D	I	P	L	K	S	E	Y	V	K	I	E	I	S	N	L

wcjE													
102	114	121	122	145	154	169	182	185	193	196	200	203	205
L	H	R	Y	I	F	I	V	C	V	N	L	F	F
V	F	K	F	V	L	T	I	L	I	D	F	I	V
V	F	K	F	V	L	T	I	L	I	D	F	I	V
V	F	K	F	V	L	T	I	L	I	D	F	I	V
V	F	K	F	V	L	T	I	L	I	D	F	I	V
V	F	K	F	V	L	T	I	L	I	D	F	I	V
V	F	K	F	V	L	T	I	L	I	D	F	I	V
V	F	K	F	V	L	T	I	L	I	D	F	I	V
V	F	K	F	V	L	T	I	L	I	D	F	I	V
V	F	K	F	V	L	T	I	L	I	D	F	I	V
V	F	K	F	V	L	T	I	L	I	D	F	I	V
V	F	K	F	V	L	T	I	L	I	D	F	I	V
V	F	K	F	V	L	T	I	L	I	D	F	I	V
V	F	K	F	V	L	T	I	L	I	D	F	I	V
V	F	K	F	V	L	T	I	L	I	D	F	I	V
V	F	K	F	V	L	T	I	L	I	D	F	I	V
V	F	K	F	V	L	T	I	L	I	D	F	I	V
V	F	K	F	V	L	T	I	L	I	D	F	I	V
V	F	K	F	V	L	T	I	L	I	D	F	I	V
V	F	K	F	V	L	T	I	L	I	D	F	I	V
V	F	K	F	V	L	T	I	L	I	D	F	I	V
V	F	K	F	V	L	T	I	L	I	D	F	I	V
V	F	K	F	V	L	T	I	L	I	D	F	I	V
V	F	K	F	V	L	T	I	L	I	D	F	I	V
V	F	K	F	V	L	T	I	L	I	D	F	I	V
V	F	K	F	V	L	T	I	L	I	D	F	I	V
V	F	K	F	V	L	T	I	L	I	D	F	I	V
V	F	K	F	V	L	T	I	L	I	D	F	I	V
V	F	K	F	V	L	T	I	L	I	D	F	I	V
V	F	K	F	V	L	T	I	L	I	D	F	I	V
V	F	K	F	V	L	T	I	L	I	D	F	I	V
V	F	K	F	V	L	T	I	L	I	D	F	I	V
V	F	K	F	V	L	T	I	L	I	D	F	I	V
V	F	K	F	V	L	T	I	L	I	D	F	I	V
V	F	K	F	V	L	T	I	L	I	D	F	I	V
V	F	K	F	V	L	T	I	L	I	D	F	I	V
V	F	K	F	V	L	T	I	L	I	D	F	I	V
V	F	K	F	V	L	T	I	L	I	D	F	I	V
V	F	K	F	V	L	T	I	L	I	D	F	I	V
V	F	K	F	V	L	T	I	L	I	D	F	I	V
V	F	K	F	V	L	T	I	L	I	D	F	I	V
V	F	K	F	V	L	T	I	L	I	D	F	I	V
V	F	K	F	V	L	T	I	L	I	D	F	I	V
V	F	K	F	V	L	T	I	L	I	D	F	I	V
V	F	K	F	V	L	T	I	L	I	D	F	I	V
V	F	K	F	V	L	T	I	L	I	D	F	I	V
V	F	K	F	V	L	T	I	L	I	D	F	I	V
V	F	K	F	V	L	T	I	L	I	D	F	I	V
V	F	K	F	V	L	T	I	L	I	D	F	I	V
V	F	K	F	V	L	T	I	L	I	D	F	I	V
V	F	K	F	V	L	T	I	L	I	D	F	I	V

68	69	71	72	73	75	77	78	79	80	81	83	93	100	101	104	107	120	122	127	131	133	134
S	K	V	Y	K	K	I	I	K	K	E	F	A	L	T	V	I	E	H	L	I	Y	R
I	H	M	C	Q	Y	L	I	R	A	N	Y	S	S	V	I	V	D	R	V	V	H	K
I	H	M	C	Q	Y	L	I	R	A	N	Y	S	S	V	I	V	D	R	V	V	H	K
S	K	V	Y	K	K	I	I	K	K	E	F	A	L	T	V	I	E	H	L	I	Y	R
S	K	V	Y	R	K	L	I	K	K	E	F	A	L	T	I	I	E	H	L	I	Y	R
S	K	I	Y	K	K	L	M	K	K	E	F	A	L	T	I	I	E	H	L	I	Y	R

S	K	I	Y	K	K	L	M	K	K	E	F	A	L	T	I	I	E	H	L	I	Y	R
S	K	I	Y	K	K	L	M	K	K	E	F	A	L	T	I	I	E	H	L	I	Y	R
S	K	I	Y	K	K	L	M	K	K	E	F	A	L	T	I	I	E	H	L	I	Y	R
S	K	I	Y	K	K	L	M	K	K	E	F	A	L	T	I	I	E	H	L	I	Y	R
S	K	I	Y	K	K	L	M	K	K	E	F	A	L	T	I	I	E	H	L	I	Y	R
S	K	I	Y	K	K	L	M	K	K	E	F	A	L	T	I	I	E	H	L	I	Y	R
S	K	I	Y	K	K	L	M	K	K	E	F	A	L	T	I	I	E	H	L	I	Y	R
S	K	I	Y	K	K	L	M	K	K	E	F	A	L	T	I	I	E	H	L	I	Y	R
S	K	I	Y	K	K	L	M	K	K	E	F	A	L	T	I	I	E	H	L	I	Y	R
S	K	I	Y	K	K	L	M	K	K	E	F	A	L	T	I	I	E	H	L	I	Y	R
S	K	I	Y	K	K	L	M	K	K	E	F	A	L	T	I	I	E	H	L	I	Y	R
S	K	I	Y	K	K	L	M	K	K	E	F	A	L	T	I	I	E	H	L	I	Y	R
S	K	I	Y	K	K	L	M	K	K	E	F	A	L	T	I	I	E	H	L	I	Y	R
S	K	I	Y	K	K	L	M	K	K	E	F	A	L	T	I	I	E	H	L	I	Y	R
S	K	I	Y	K	K	L	M	K	K	E	F	A	L	T	I	I	E	H	L	I	Y	R
S	K	I	Y	K	K	L	M	K	K	E	F	A	L	T	I	I	E	H	L	I	Y	R
S	K	I	Y	K	K	L	M	K	K	E	F	A	L	T	I	I	E	H	L	I	Y	R
S	K	I	Y	K	K	L	M	K	K	E	F	A	L	T	I	I	E	H	L	I	Y	R
S	K	I	Y	K	K	L	M	K	K	E	F	A	L	T	I	I	E	H	L	I	Y	R
S	K	I	Y	K	K	L	M	K	K	E	F	A	L	T	I	I	E	H	L	I	Y	R
S	K	I	Y	K	K	L	M	K	K	E	F	A	L	T	I	I	E	H	L	I	Y	R
S	K	I	Y	K	K	L	M	K	K	E	F	A	L	T	I	I	E	H	L	I	Y	R
S	K	I	Y	K	K	L	M	K	K	E	F	A	L	T	I	I	E	H	L	I	Y	R
S	K	I	Y	K	K	L	M	K	K	E	F	A	L	T	I	I	E	H	L	I	Y	R
S	K	I	Y	K	K	L	M	K	K	E	F	A	L	T	I	I	E	H	L	I	Y	R
S	K	I	Y	K	K	L	M	K	K	E	F	A	L	T	I	I	E	H	L	I	Y	R
S	K	I	Y	K	K	L	M	K	K	E	F	A	L	T	I	I	E	H	L	I	Y	R
S	K	I	Y	K	K	L	M	K	K	E	F	A	L	T	I	I	E	H	L	I	Y	R
S	K	I	Y	K	K	L	M	K	K	E	F	A	L	T	I	I	E	H	L	I	Y	R
S	K	I	Y	K	K	L	M	K	K	E	F	A	L	T	I	I	E	H	L	I	Y	R
S	K	I	Y	K	K	L	M	K	K	E	F	A	L	T	I	I	E	H	L	I	Y	R
S	K	I	Y	K	K	L	M	K	K	E	F	A	L	T	I	I	E	H	L	I	Y	R
S	K	I	Y	K	K	L	M	K	K	E	F	A	L	T	I	I	E	H	L	I	Y	R
S	K	I	Y	K	K	L	M	K	K	E	F	A	L	T	I	I	E	H	L	I	Y	R
S	K	I	Y	K	K	L	M	K	K	E	F	A	L	T	I	I	E	H	L	I	Y	R
S	K	I	Y	K	K	L	M	K	K	E	F	A	L	T	I	I	E	H	L	I	Y	R
S	K	I	Y	K	K	L	M	K	K	E	F	A	L	T	I	I	E	H	L	I	Y	R
S	K	I	Y	K	K	L	M	K	K	E	F	A	L	T	I	I	E	H	L	I	Y	R
S	K	I	Y	K	K	L	M	K	K	E	F	A	L	T	I	I	E	H	L	I	Y	R
S	K	I	Y	K	K	L	M	K	K	E	F	A	L	T	I	I	E	H	L	I	Y	R
S	K	I	Y	K	K	L	M	K	K	E	F	A	L	T	I	I	E	H	L	I	Y	R
S	K	I	Y	K	K	L	M	K	K	E	F	A	L	T	I	I	E	H	L	I	Y	R
S	K	I	Y	K	K	L	M	K	K	E	F	A	L	T	I	I	E	H	L	I	Y	R
S	K	I	Y	K	K	L	M	K	K	E	F	A	L	T	I	I	E	H	L	I	Y	R
S	K	I	Y	K	K	L	M	K	K	E	F	A	L	T	I	I	E	H	L	I	Y	R
S	K	I	Y	K	K	L	M	K	K	E	F	A	L	T	I	I	E	H	L	I	Y	R
S	K	I	Y	K	K	L	M	K	K	E	F	A	L	T	I	I	E	H	L	I	Y	R
S	K	I	Y	K	K	L	M	K	K	E	F	A	L	T	I	I	E	H	L	I	Y	R

135	140	143	144	145	148	152	153	154	155	157	164	165	166	167	170	171	172	173	176	177	178	180
V	C	S	V	S	Q	E	E	K	N	K	D	V	N	R	Y	D	A	I	K	K	L	E
L	W	G	E	G	K	T	N	R	E	T	N	I	K	R	F	S	E	S	Q	R	F	K
L	W	G	E	G	K	T	N	R	E	T	N	I	K	R	F	S	E	S	Q	R	F	K
V	C	S	V	S	Q	E	E	K	N	K	D	V	N	R	Y	D	A	I	K	K	L	E
V	C	S	V	S	Q	E	E	K	N	K	D	V	N	K	Y	D	A	I	K	K	L	E
V	C	S	V	S	Q	E	E	K	N	E	D	V	N	K	Y	D	A	I	K	K	L	E

V	C	S	V	S	Q	E	E	K	N	E	D	V	N	K	Y	D	A	I	K	K	L	E
V	C	S	V	S	Q	E	E	K	N	E	D	V	N	K	Y	D	A	I	K	K	L	E
V	C	S	V	S	Q	E	E	K	N	E	D	V	N	K	Y	D	A	I	K	K	L	E
V	C	S	V	S	Q	E	E	K	N	E	D	V	N	K	Y	D	A	I	K	K	L	E
V	C	S	V	S	Q	E	E	K	N	E	D	V	N	K	Y	D	A	I	K	K	L	E
V	C	S	V	S	Q	E	E	K	N	E	D	V	N	K	Y	D	A	I	K	K	L	E
V	C	S	V	S	Q	E	E	K	N	E	D	V	N	K	Y	D	A	I	K	K	L	E
V	C	S	V	S	Q	E	E	K	N	E	D	V	N	K	Y	D	A	I	K	K	L	E
V	C	S	V	S	Q	E	E	K	N	E	D	V	N	K	Y	D	A	I	K	K	L	E
V	C	S	V	S	Q	E	E	K	N	E	D	V	N	K	Y	D	A	I	K	K	L	E
V	C	S	V	S	Q	E	E	K	N	E	D	V	N	K	Y	D	A	I	K	K	L	E
V	C	S	V	S	Q	E	E	K	N	E	D	V	N	K	Y	D	A	I	K	K	L	E
V	C	S	V	S	Q	E	E	K	N	E	D	V	N	K	Y	D	A	I	K	K	L	E
V	C	S	V	S	Q	E	E	K	N	E	D	V	N	K	Y	D	A	I	K	K	L	E
V	C	S	V	S	Q	E	E	K	N	E	D	V	N	K	Y	D	A	I	K	K	L	E
V	C	S	V	S	Q	E	E	K	N	E	D	V	N	K	Y	D	A	I	K	K	L	E
V	C	S	V	S	Q	E	E	K	N	E	D	V	N	K	Y	D	A	I	K	K	L	E
V	C	S	V	S	Q	E	E	K	N	E	D	V	N	K	Y	D	A	I	K	K	L	E
V	C	S	V	S	Q	E	E	K	N	E	D	V	N	K	Y	D	A	I	K	K	L	E
V	C	S	V	S	Q	E	E	K	N	E	D	V	N	K	Y	D	A	I	K	K	L	E
V	C	S	V	S	Q	E	E	K	N	E	D	V	N	K	Y	D	A	I	K	K	L	E
V	C	S	V	S	Q	E	E	K	N	E	D	V	N	K	Y	D	A	I	K	K	L	E
V	C	S	V	S	Q	E	E	K	N	E	D	V	N	K	Y	D	A	I	K	K	L	E
V	C	S	V	S	Q	E	E	K	N	E	D	V	N	K	Y	D	A	I	K	K	L	E
V	C	S	V	S	Q	E	E	K	N	E	D	V	N	K	Y	D	A	I	K	K	L	E
V	C	S	V	S	Q	E	E	K	N	E	D	V	N	K	Y	D	A	I	K	K	L	E
V	C	S	V	S	Q	E	E	K	N	E	D	V	N	K	Y	D	A	I	K	K	L	E
V	C	S	V	S	Q	E	E	K	N	E	D	V	N	K	Y	D	A	I	K	K	L	E
V	C	S	V	S	Q	E	E	K	N	E	D	V	N	K	Y	D	A	I	K	K	L	E
V	C	S	V	S	Q	E	E	K	N	E	D	V	N	K	Y	D	A	I	K	K	L	E
V	C	S	V	S	Q	E	E	K	N	E	D	V	N	K	Y	D	A	I	K	K	L	E
V	C	S	V	S	Q	E	E	K	N	E	D	V	N	K	Y	D	A	I	K	K	L	E
V	C	S	V	S	Q	E	E	K	N	E	D	V	N	K	Y	D	A	I	K	K	L	E
V	C	S	V	S	Q	E	E	K	N	E	D	V	N	K	Y	D	A	I	K	K	L	E
V	C	S	V	S	Q	E	E	K	N	E	D	V	N	K	Y	D	A	I	K	K	L	E
V	C	S	V	S	Q	E	E	K	N	E	D	V	N	K	Y	D	A	I	K	K	L	E
V	C	S	V	S	Q	E	E	K	N	E	D	V	N	K	Y	D	A	I	K	K	L	E
V	C	S	V	S	Q	E	E	K	N	E	D	V	N	K	Y	D	A	I	K	K	L	E
V	C	S	V	S	Q	E	E	K	N	E	D	V	N	K	Y	D	A	I	K	K	L	E
V	C	S	V	S	Q	E	E	K	N	E	D	V	N	K	Y	D	A	I	K	K	L	E
V	C	S	V	S	Q	E	E	K	N	E	D	V	N	K	Y	D	A	I	K	K	L	E
V	C	S	V	S	Q	E	E	K	N	E	D	V	N	K	Y	D	A	I	K	K	L	E
V	C	S	V	S	Q	E	E	K	N	E	D	V	N	K	Y	D	A	I	K	K	L	E
V	C	S	V	S	Q	E	E	K	N	E	D	V	N	K	Y	D	A	I	K	K	L	E
V	C	S	V	S	Q	E	E	K	N	E	D	V	N	K	Y	D	A	I	K	K	L	E
V	C	S	V	S	Q	E	E	K	N	E	D	V	N	K	Y	D	A	I	K	K	L	E
V	C	S	V	S	Q	E	E	K	N	E	D	V	N	K	Y	D	A	I	K	K	L	E
V	C	S	V	S	Q	E	E	K	N	E	D	V	N	K	Y	D	A	I	K	K	L	E

wcyK																						
181	183	185	187	189	199	203	204	208	210	212	216	217	218	220	227	228	232	233	237	238	247	248
E	L	S	E	I	T	Q	C	S	M	K	K	N	I	I	D	R	G	R	I	S	N	I
K	S	K	T	L	V	H	E	L	M	N	Q	G	L	V	E	G	I	K	Y	G	R	V
K	S	K	T	L	V	H	E	L	M	N	Q	G	L	V	E	G	I	K	Y	G	R	V
E	L	S	E	I	T	Q	C	S	M	K	K	N	I	I	D	R	G	R	I	S	N	I
E	L	S	E	I	T	Q	R	S	I	R	K	N	I	L	D	G	R	R	I	S	N	I
E	L	S	E	I	T	Q	R	S	M	R	K	N	I	L	D	G	R	R	I	S	N	I

E	L	S	E	I	T	Q	R	S	M	R	K	N	I	L	D	G	R	R	I	S	N	I
E	L	S	E	I	T	Q	R	S	M	R	K	N	I	L	D	G	R	R	I	S	N	I
E	L	S	E	I	T	Q	R	S	M	R	K	N	I	L	D	G	R	R	I	S	N	I
E	L	S	E	I	T	Q	R	S	M	R	K	N	I	L	D	G	R	R	I	S	N	I
E	L	S	E	I	T	Q	R	S	M	R	K	N	I	L	D	G	R	R	I	S	N	I
E	L	S	E	I	T	Q	R	S	M	R	K	N	I	L	D	G	R	R	I	S	N	I
E	L	S	E	I	T	Q	R	S	M	R	K	N	I	L	D	G	R	R	I	S	N	I
E	L	S	E	I	T	Q	R	S	M	R	K	N	I	L	D	G	R	R	I	S	N	I
E	L	S	E	I	T	Q	R	S	M	R	K	N	I	L	D	G	R	R	I	S	N	I
E	L	S	E	I	T	Q	R	S	M	R	K	N	I	L	D	G	R	R	I	S	N	I
E	L	S	E	I	T	Q	R	S	M	R	K	N	I	L	D	G	R	R	I	S	N	I
E	L	S	E	I	T	Q	R	S	M	R	K	N	I	L	D	G	R	R	I	S	N	I
E	L	S	E	I	T	Q	R	S	M	R	K	N	I	L	D	G	R	R	I	S	N	I
E	L	S	E	I	T	Q	R	S	M	R	K	N	I	L	D	G	R	R	I	S	N	I
E	L	S	E	I	T	Q	R	S	M	R	K	N	I	L	D	G	R	R	I	S	N	I
E	L	S	E	I	T	Q	R	S	M	R	K	N	I	L	D	G	R	R	I	S	N	I
E	L	S	E	I	T	Q	R	S	M	R	K	N	I	L	D	G	R	R	I	S	N	I
E	L	S	E	I	T	Q	R	S	M	R	K	N	I	L	D	G	R	R	I	S	N	I
E	L	S	E	I	T	Q	R	S	M	R	K	N	I	L	D	G	R	R	I	S	N	I
E	L	S	E	I	T	Q	R	S	M	R	K	N	I	L	D	G	R	R	I	S	N	I
E	L	S	E	I	T	Q	R	S	M	R	K	N	I	L	D	G	R	R	I	S	N	I
E	L	S	E	I	T	Q	R	S	M	R	K	N	I	L	D	G	R	R	I	S	N	I
E	L	S	E	I	T	Q	R	S	M	R	K	N	I	L	D	G	R	R	I	S	N	I
E	L	S	E	I	T	Q	R	S	M	R	K	N	I	L	D	G	R	R	I	S	N	I
E	L	S	E	I	T	Q	R	S	M	R	K	N	I	L	D	G	R	R	I	S	N	I
E	L	S	E	I	T	Q	R	S	M	R	K	N	I	L	D	G	R	R	I	S	N	I
E	L	S	E	I	T	Q	R	S	M	R	K	N	I	L	D	G	R	R	I	S	N	I
E	L	S	E	I	T	Q	R	S	M	R	K	N	I	L	D	G	R	R	I	S	N	I
E	L	S	E	I	T	Q	R	S	M	R	K	N	I	L	D	G	R	R	I	S	N	I
E	L	S	E	I	T	Q	R	S	M	R	K	N	I	L	D	G	R	R	I	S	N	I
E	L	S	E	I	T	Q	R	S	M	R	K	N	I	L	D	G	R	R	I	S	N	I
E	L	S	E	I	T	Q	R	S	M	R	K	N	I	L	D	G	R	R	I	S	N	I
E	L	S	E	I	T	Q	R	S	M	R	K	N	I	L	D	G	R	R	I	S	N	I
E	L	S	E	I	T	Q	R	S	M	R	K	N	I	L	D	G	R	R	I	S	N	I
E	L	S	E	I	T	Q	R	S	M	R	K	N	I	L	D	G	R	R	I	S	N	I
E	L	S	E	I	T	Q	R	S	M	R	K	N	I	L	D	G	R	R	I	S	N	I
E	L	S	E	I	T	Q	R	S	M	R	K	N	I	L	D	G	R	R	I	S	N	I
E	L	S	E	I	T	Q	R	S	M	R	K	N	I	L	D	G	R	R	I	S	N	I
E	L	S	E	I	T	Q	R	S	M	R	K	N	I	L	D	G	R	R	I	S	N	I
E	L	S	E	I	T	Q	R	S	M	R	K	N	I	L	D	G	R	R	I	S	N	I
E	L	S	E	I	T	Q	R	S	M	R	K	N	I	L	D	G	R	R	I	S	N	I
E	L	S	E	I	T	Q	R	S	M	R	K	N	I	L	D	G	R	R	I	S	N	I
E	L	S	E	I	T	Q	R	S	M	R	K	N	I	L	D	G	R	R	I	S	N	I
E	L	S	E	I	T	Q	R	S	M	R	K	N	I	L	D	G	R	R	I	S	N	I
E	L	S	E	I	T	Q	R	S	M	R	K	N	I	L	D	G	R	R	I	S	N	I
E	L	S	E	I	T	Q	R	S	M	R	K	N	I	L	D	G	R	R	I	S	N	I
E	L	S	E	I	T	Q	R	S	M	R	K	N	I	L	D	G	R	R	I	S	N	I
E	L	S	E	I	T	Q	R	S	M	R	K	N	I	L	D	G	R	R	I	S	N	I

249	253	254	255	263	288	289	290	295	296	298	307	308	309	310	314	315	316	324	326	327	328	329
V	Q	F	D	S	V	E	S	R	V	Q	N	C	S	T	S	I	D	E	L	N	L	S
F	H	L	N	A	I	Q	C	T	I	K	T	S	L	P	P	L	Y	A	R	D	F	C
F	H	L	N	A	I	Q	C	T	I	K	T	S	L	P	P	L	Y	A	R	D	F	C
V	Q	F	D	S	V	E	S	R	V	Q	N	C	S	T	S	I	D	E	L	N	L	S
V	Q	F	D	S	V	K	S	R	V	Q	N	C	S	T	S	I	D	E	L	N	L	S
V	Q	F	D	S	V	K	S	R	V	Q	N	C	S	T	S	I	D	E	L	N	L	S

V	Q	F	D	S	V	K	S	R	V	Q	N	C	S	T	S	I	D	E	L	N	L	S
V	Q	F	D	S	V	K	S	R	V	Q	N	C	S	T	S	I	D	E	L	N	L	S
V	Q	F	D	S	V	K	S	R	V	Q	N	C	S	T	S	I	D	E	L	N	L	S
V	Q	F	D	S	V	K	S	R	V	Q	N	C	S	T	S	I	D	E	L	N	L	S
V	Q	F	D	S	V	K	S	R	V	Q	N	C	S	T	S	I	D	E	L	N	L	S
V	Q	F	D	S	V	K	S	R	V	Q	N	C	S	T	S	I	D	E	L	N	L	S
V	Q	F	D	S	V	K	S	R	V	Q	N	C	S	T	S	I	D	E	L	N	L	S
V	Q	F	D	S	V	K	S	R	V	Q	N	C	S	T	S	I	D	E	L	N	L	S
V	Q	F	D	S	V	K	S	R	V	Q	N	C	S	T	S	I	D	E	L	N	L	S
V	Q	F	D	S	V	K	S	R	V	Q	N	C	S	T	S	I	D	E	L	N	L	S
V	Q	F	D	S	V	K	S	R	V	Q	N	C	S	T	S	I	D	E	L	N	L	S
V	Q	F	D	S	V	K	S	R	V	Q	N	C	S	T	S	I	D	E	L	N	L	S
V	Q	F	D	S	V	K	S	R	V	Q	N	C	S	T	S	I	D	E	L	N	L	S
V	Q	F	D	S	V	K	S	R	V	Q	N	C	S	T	S	I	D	E	L	N	L	S
V	Q	F	D	S	V	K	S	R	V	Q	N	C	S	T	S	I	D	E	L	N	L	S
V	Q	F	D	S	V	K	S	R	V	Q	N	C	S	T	S	I	D	E	L	N	L	S
V	Q	F	D	S	V	K	S	R	V	Q	N	C	S	T	S	I	D	E	L	N	L	S
V	Q	F	D	S	V	K	S	R	V	Q	N	C	S	T	S	I	D	E	L	N	L	S
V	Q	F	D	S	V	K	S	R	V	Q	N	C	S	T	S	I	D	E	L	N	L	S
V	Q	F	D	S	V	K	S	R	V	Q	N	C	S	T	S	I	D	E	L	N	L	S
V	Q	F	D	S	V	K	S	R	V	Q	N	C	S	T	S	I	D	E	L	N	L	S
V	Q	F	D	S	V	K	S	R	V	Q	N	C	S	T	S	I	D	E	L	N	L	S
V	Q	F	D	S	V	K	S	R	V	Q	N	C	S	T	S	I	D	E	L	N	L	S
V	Q	F	D	S	V	K	S	R	V	Q	N	C	S	T	S	I	D	E	L	N	L	S
V	Q	F	D	S	V	K	S	R	V	Q	N	C	S	T	S	I	D	E	L	N	L	S
V	Q	F	D	S	V	K	S	R	V	Q	N	C	S	T	S	I	D	E	L	N	L	S
V	Q	F	D	S	V	K	S	R	V	Q	N	C	S	T	S	I	D	E	L	N	L	S
V	Q	F	D	S	V	K	S	R	V	Q	N	C	S	T	S	I	D	E	L	N	L	S
V	Q	F	D	S	V	K	S	R	V	Q	N	C	S	T	S	I	D	E	L	N	L	S
V	Q	F	D	S	V	K	S	R	V	Q	N	C	S	T	S	I	D	E	L	N	L	S
V	Q	F	D	S	V	K	S	R	V	Q	N	C	S	T	S	I	D	E	L	N	L	S
V	Q	F	D	S	V	K	S	R	V	Q	N	C	S	T	S	I	D	E	L	N	L	S
V	Q	F	D	S	V	K	S	R	V	Q	N	C	S	T	S	I	D	E	L	N	L	S
V	Q	F	D	S	V	K	S	R	V	Q	N	C	S	T	S	I	D	E	L	N	L	S
V	Q	F	D	S	V	K	S	R	V	Q	N	C	S	T	S	I	D	E	L	N	L	S
V	Q	F	D	S	V	K	S	R	V	Q	N	C	S	T	S	I	D	E	L	N	L	S
V	Q	F	D	S	V	K	S	R	V	Q	N	C	S	T	S	I	D	E	L	N	L	S
V	Q	F	D	S	V	K	S	R	V	Q	N	C	S	T	S	I	D	E	L	N	L	S
V	Q	F	D	S	V	K	S	R	V	Q	N	C	S	T	S	I	D	E	L	N	L	S
V	Q	F	D	S	V	K	S	R	V	Q	N	C	S	T	S	I	D	E	L	N	L	S
V	Q	F	D	S	V	K	S	R	V	Q	N	C	S	T	S	I	D	E	L	N	L	S
V	Q	F	D	S	V	K	S	R	V	Q	N	C	S	T	S	I	D	E	L	N	L	S
V	Q	F	D	S	V	K	S	R	V	Q	N	C	S	T	S	I	D	E	L	N	L	S
V	Q	F	D	S	V	K	S	R	V	Q	N	C	S	T	S	I	D	E	L	N	L	S
V	Q	F	D	S	V	K	S	R	V	Q	N	C	S	T	S	I	D	E	L	N	L	S
V	Q	F	D	S	V	K	S	R	V	Q	N	C	S	T	S	I	D	E	L	N	L	S
V	Q	F	D	S	V	K	S	R	V	Q	N	C	S	T	S	I	D	E	L	N	L	S
V	Q	F	D	S	V	K	S	R	V	Q	N	C	S	T	S	I	D	E	L	N	L	S

330	331	334	336	338	339	341	342	344	345	346	348	352	355	356	357	358	359	360	369	371	375
T	S	E	V	L	Q	I	Q	I	K	N	G	S	V	K	E	I	E	E	L	Y	N
P	E	L	Q	I	E	Q	R	A	R	E	G	E	L	V	K	L	D	T	I	D	K
P	E	L	Q	I	E	Q	R	A	R	E	G	E	L	V	K	L	D	T	I	D	K
T	S	E	V	L	Q	I	Q	I	K	N	G	S	V	K	E	I	E	E	L	Y	N
T	S	E	V	L	Q	I	Q	I	R	N	G	S	V	K	E	I	E	E	L	Y	N
T	S	E	V	L	Q	I	Q	I	R	N	G	S	V	K	E	I	E	E	L	Y	N

T	S	E	V	L	Q	I	Q	I	R	N	G	S	V	K	E	I	E	E	L	Y	N
T	S	E	V	L	Q	I	Q	I	R	N	G	S	V	K	E	I	E	E	L	Y	N
T	S	E	V	L	Q	I	Q	I	R	N	G	S	V	K	E	I	E	E	L	Y	N
T	S	E	V	L	Q	I	Q	I	R	N	G	S	V	K	E	I	E	E	L	Y	N
T	S	E	V	L	Q	I	Q	I	R	N	G	S	V	K	E	I	E	E	L	Y	N
T	S	E	V	L	Q	I	Q	I	R	N	G	S	V	K	E	I	E	E	L	Y	N
T	S	E	V	L	Q	I	Q	I	R	N	G	S	V	K	E	I	E	E	L	Y	N
T	S	E	V	L	Q	I	Q	I	R	N	G	S	V	K	E	I	E	E	L	Y	N
T	S	E	V	L	Q	I	Q	I	R	N	G	S	V	K	E	I	E	E	L	Y	N
T	S	E	V	L	Q	I	Q	I	R	N	G	S	V	K	E	I	E	E	L	Y	N
T	S	E	V	L	Q	I	Q	I	R	N	G	S	V	K	E	I	E	E	L	Y	N
T	S	E	V	L	Q	I	Q	I	R	N	G	S	V	K	E	I	E	E	L	Y	N
T	S	E	V	L	Q	I	Q	I	R	N	G	S	V	K	E	I	E	E	L	Y	N
T	S	E	V	L	Q	I	Q	I	R	N	G	S	V	K	E	I	E	E	L	Y	N
T	S	E	V	L	Q	I	Q	I	R	N	G	S	V	K	E	I	E	E	L	Y	N
T	S	E	V	L	Q	I	Q	I	R	N	G	S	V	K	E	I	E	E	L	Y	N
T	S	E	V	L	Q	I	Q	I	R	N	G	S	V	K	E	I	E	E	L	Y	N
T	S	E	V	L	Q	I	Q	I	R	N	G	S	V	K	E	I	E	E	L	Y	N
T	S	E	V	L	Q	I	Q	I	R	N	G	S	V	K	E	I	E	E	L	Y	N
T	S	E	V	L	Q	I	Q	I	R	N	G	S	V	K	E	I	E	E	L	Y	N
T	S	E	V	L	Q	I	Q	I	R	N	G	S	V	K	E	I	E	E	L	Y	N
T	S	E	V	L	Q	I	Q	I	R	N	G	S	V	K	E	I	E	E	L	Y	N
T	S	E	V	L	Q	I	Q	I	R	N	G	S	V	K	E	I	E	E	L	Y	N
T	S	E	V	L	Q	I	Q	I	R	N	G	S	V	K	E	I	E	E	L	Y	N
T	S	E	V	L	Q	I	Q	I	R	N	G	S	V	K	E	I	E	E	L	Y	N
T	S	E	V	L	Q	I	Q	I	R	N	G	S	V	K	E	I	E	E	L	Y	N
T	S	E	V	L	Q	I	Q	I	R	N	G	S	V	K	E	I	E	E	L	Y	N
T	S	E	V	L	Q	I	Q	I	R	N	G	S	V	K	E	I	E	E	L	Y	N
T	S	E	V	L	Q	I	Q	I	R	N	G	S	V	K	E	I	E	E	L	Y	N
T	S	E	V	L	Q	I	Q	I	R	N	D	S	V	K	E	I	E	E	L	Y	N
T	S	E	V	L	Q	I	Q	I	R	N	G	S	V	K	E	I	E	E	L	Y	N
T	S	E	V	L	Q	I	Q	I	R	N	G	S	V	K	E	I	E	E	L	Y	N
T	S	E	V	L	Q	I	Q	I	R	N	G	S	V	K	E	I	E	E	L	Y	N
T	S	E	V	L	Q	I	Q	I	R	N	G	S	V	K	E	I	E	E	L	Y	N
T	S	E	V	L	Q	I	Q	I	R	N	G	S	V	K	E	I	E	E	L	Y	N
T	S	E	V	L	Q	I	Q	I	R	N	G	S	V	K	E	I	E	E	L	Y	N
T	S	E	V	L	Q	I	Q	I	R	N	G	S	V	K	E	I	E	E	L	Y	N
T	S	E	V	L	Q	I	Q	I	R	N	G	S	V	K	E	I	E	E	L	Y	N
T	S	E	V	L	Q	I	Q	I	R	N	G	S	V	K	E	I	E	E	L	Y	N
T	S	E	V	L	Q	I	Q	I	R	N	G	S	V	K	E	I	E	E	L	Y	N
T	S	E	V	L	Q	I	Q	I	R	N	G	S	V	K	E	I	E	E	L	Y	N
T	S	E	V	L	Q	I	Q	I	R	N	G	S	V	K	E	I	E	E	L	Y	N
T	S	E	V	L	Q	I	Q	I	R	N	G	S	V	K	E	I	E	E	L	Y	N
T	S	E	V	L	Q	I	Q	I	R	N	G	S	V	K	E	I	E	E	L	Y	N
T	S	E	V	L	Q	I	Q	I	R	N	G	S	V	K	E	I	E	E	L	Y	N
T	S	E	V	L	Q	I	Q	I	R	N	G	S	V	K	E	I	E	E	L	Y	N
T	S	E	V	L	Q	I	Q	I	R	N	G	S	V	K	E	I	E	E	L	Y	N
T	S	E	V	L	Q	I	Q	I	R	N	G	S	V	K	E	I	E	E	L	Y	N

wcwC																						
10	23	33	37	46	54	56	83	86	98	99	102	110	111	115	118	127	131	137	138	139	144	151
M	P	R	R	N	T	G	L	F	R	K	E	N	S	T	D	K	L	A	N	Q	E	N
[redacted]																						
M	P	R	R	N	T	G	L	F	R	K	E	N	S	T	D	K	L	A	N	Q	E	N
T	S	K	K	K	R	A	M	Y	K	D	G	V	T	E	T	K	I	V	N	Q	E	N
M	P	R	K	N	T	G	L	F	K	K	E	N	S	T	D	N	I	S	S	M	K	K

M	P	R	K	N	T	G	L	F	K	K	E	N	S	T	D	N	I	S	S	M	K	K
M	P	R	K	N	T	G	L	F	K	K	E	N	S	T	D	N	I	S	S	M	K	K
M	P	R	K	N	T	G	L	F	K	K	E	N	S	T	D	N	I	S	S	M	K	K
M	P	R	K	N	T	G	L	F	K	K	E	N	S	T	D	N	I	S	S	M	K	K
M	P	R	K	N	T	G	L	F	K	K	E	N	S	T	D	N	I	S	S	M	K	K
M	P	R	K	N	T	G	L	F	K	K	E	N	S	T	D	N	I	S	S	M	K	K
M	P	R	K	N	T	G	L	F	K	K	E	N	S	T	D	N	I	S	S	M	K	K
M	P	R	K	N	T	G	L	F	K	K	E	N	S	T	D	N	I	S	S	M	K	K
M	P	R	K	N	T	G	L	F	K	K	E	N	S	T	D	N	I	S	S	M	K	K
M	P	R	K	N	T	G	L	F	K	K	E	N	S	T	D	N	I	S	S	M	K	K
M	P	R	K	N	T	G	L	F	K	K	E	N	S	T	D	N	I	S	-	-	-	-
M	P	R	K	N	T	G	L	F	K	K	E	N	S	T	D	N	I	S	S	M	K	K
M	P	R	K	N	T	G	L	F	K	K	E	N	S	T	D	N	I	S	S	M	K	K
M	P	R	K	N	T	G	L	F	K	K	E	N	S	T	D	N	I	S	S	M	K	K
M	P	R	K	N	T	G	L	F	K	K	E	N	S	T	D	N	I	S	S	M	K	K
M	P	R	K	N	T	G	L	F	K	K	E	N	S	T	D	N	I	S	S	M	K	K
M	P	R	K	N	T	G	L	F	K	K	E	N	S	T	D	N	I	S	S	M	K	K
M	P	R	K	N	T	G	L	F	K	K	E	N	S	T	D	N	I	S	S	M	K	K
M	P	R	K	N	T	G	L	F	K	K	E	N	S	T	D	N	I	S	S	M	K	K
M	P	R	K	N	T	G	L	F	K	K	E	N	S	T	D	N	I	S	S	M	K	K
M	P	R	K	N	T	G	L	F	K	K	E	N	S	T	D	N	I	S	S	M	K	K
M	P	R	K	N	T	G	L	F	K	K	E	N	S	T	D	N	I	S	S	M	K	K
M	P	R	K	N	T	G	L	F	K	K	E	N	S	T	D	N	I	S	S	M	K	K
M	P	R	K	N	T	G	L	F	K	K	E	N	S	T	D	N	I	S	S	M	K	K
M	P	R	K	N	T	G	L	F	K	K	E	N	S	T	D	N	I	S	S	M	K	K
M	P	R	K	N	T	G	L	F	K	K	E	N	S	T	D	N	I	S	S	M	K	K
M	P	R	K	N	T	G	L	F	K	K	E	N	S	T	D	N	I	S	S	M	K	K
M	P	R	K	N	T	G	L	F	K	K	E	N	S	T	D	N	I	S	S	M	K	K
M	P	R	K	N	T	G	L	F	K	K	E	N	S	T	D	N	I	S	S	M	K	K
M	P	R	K	N	T	G	L	F	K	K	E	N	S	T	D	N	I	S	S	M	K	K
M	P	R	K	N	T	G	L	F	K	K	E	N	S	T	D	N	I	S	S	M	K	K
M	P	R	K	N	T	G	L	F	K	K	E	N	S	T	D	N	I	S	S	M	K	K
M	P	R	K	N	T	G	L	F	K	K	E	N	S	T	D	N	I	S	S	M	K	K
M	P	R	K	N	T	G	L	F	K	K	E	N	S	T	D	N	I	S	S	M	K	K
M	P	R	K	N	T	G	L	F	K	K	E	N	S	T	D	N	I	S	S	M	K	K
M	P	R	K	N	T	G	L	F	K	K	E	N	S	T	D	N	I	S	S	M	K	K
M	P	R	K	N	T	G	L	F	K	K	E	N	S	T	D	N	I	S	S	M	K	K
M	P	R	K	N	T	G	L	F	K	K	E	N	S	T	D	N	I	S	S	M	K	K
M	P	R	K	N	T	G	L	F	K	K	E	N	S	T	D	N	I	S	S	M	K	K
M	P	R	K	N	T	G	L	F	K	K	E	N	S	T	D	N	I	S	S	M	K	K
M	P	R	K	N	T	G	L	F	K	K	E	N	S	T	D	N	I	S	S	M	K	K
M	P	R	K	N	T	G	L	F	K	K	E	N	S	T	D	N	I	S	S	M	K	K
M	P	R	K	N	T	G	L	F	K	K	E	N	S	T	D	N	I	S	S	M	K	K
M	P	R	K	N	T	G	L	F	K	K	E	N	S	T	D	N	I	S	S	M	K	K
M	P	R	K	N	T	G	L	F	K	K	E	N	S	T	D	N	I	S	S	M	K	K
M	P	R	K	N	T	G	L	F	K	K	E	N	S	T	D	N	I	S	S	M	K	K
M	P	R	K	N	T	G	L	F	K	K	E	N	S	T	D	N	I	S	S	M	K	K
M	P	R	K	N	T	G	L	F	K	K	E	N	S	T	D	N	I	S	S	M	K	K

156	170	171	178	181	203	205	212	218	219
E	L	K	T	L	N	R	T	D	Q
[redacted]									
E	L	K	T	L	D	R	T	D	Q
E	F	E	N	F	N	R	T	D	Q
K	L	K	T	L	N	I	A	N	K

14	17	18	22	25	28	33	38	40	42	45	47
K	S	N	R	A	K	D	E	T	K	C	E
G	P	T	K	D	R	N	R	D	S	I	S
G	P	T	K	D	R	N	R	D	S	I	S
K	S	N	R	A	K	D	E	T	K	C	E
K	S	S	R	A	K	D	E	T	K	C	E
K	P	N	R	A	R	D	E	T	K	C	Q

K	L	K	T	L	N	I	A	N	K
K	L	K	T	L	N	I	A	N	K
K	L	K	T	L	N	I	A	N	K
K	L	K	T	L	N	I	A	N	K
K	L	K	T	L	N	I	A	N	K
K	L	K	T	L	N	I	A	N	K
K	L	K	T	L	N	I	A	N	K
K	L	K	T	L	N	I	A	N	K
K	L	K	T	L	N	I	A	N	K
K	L	K	T	L	N	I	A	N	K
-	-	-	-	-	-	-	-	-	-
K	L	K	T	L	N	I	A	N	K
K	L	K	T	L	N	I	A	N	K
K	L	K	T	L	N	I	A	N	K
K	L	K	T	L	N	I	A	N	K
K	L	K	T	L	N	I	A	N	K
K	L	K	T	L	N	I	A	N	K
K	L	K	T	L	N	I	A	N	K
K	L	K	T	L	N	I	A	N	K
K	L	K	T	L	N	I	A	N	K
K	L	K	T	L	N	I	A	N	K
K	L	K	T	L	N	I	A	N	K
K	L	K	T	L	N	I	A	N	K
K	L	K	T	L	N	I	A	N	K
K	L	K	T	L	N	I	A	N	K
K	L	K	T	L	N	I	A	N	K
K	L	K	T	L	N	I	A	N	K
K	L	K	T	L	N	I	A	N	K
K	L	K	T	L	N	I	A	N	K
K	L	K	T	L	N	I	A	N	K
K	L	K	T	L	N	I	A	N	K
K	L	K	T	L	N	I	A	N	K
K	L	K	T	L	N	I	A	N	K
K	L	K	T	L	N	I	A	N	K
K	L	K	T	L	N	I	A	N	K
K	L	K	T	L	N	I	A	N	K
K	L	K	T	L	N	I	A	N	K
K	L	K	T	L	N	I	A	N	K
K	L	K	T	L	N	I	A	N	K
K	L	K	T	L	N	I	A	N	K
K	L	K	T	L	N	I	A	N	K
K	L	K	T	L	N	I	A	N	K
K	L	K	T	L	N	I	A	N	K
K	L	K	T	L	N	I	A	N	K
K	L	K	T	L	N	I	A	N	K
K	L	K	T	L	N	I	A	N	K
K	L	K	T	L	N	I	A	N	K
K	L	K	T	L	N	I	A	N	K

K	P	N	R	A	R	D	E	T	K	C	Q
K	P	N	R	A	R	D	E	T	K	C	Q
K	P	N	R	A	R	D	E	T	K	C	Q
K	P	N	R	A	R	D	E	T	K	C	Q
K	P	N	R	A	R	D	E	T	K	C	Q
K	P	N	R	A	R	D	E	T	K	C	Q
K	P	N	R	A	R	D	E	T	K	C	Q
K	P	N	R	A	R	D	E	T	K	C	Q
K	P	N	R	A	R	D	E	T	K	C	Q
K	P	N	R	A	R	D	E	T	K	C	Q
K	P	N	R	A	R	D	E	T	K	C	Q
K	P	N	R	A	R	D	E	T	K	C	Q
K	P	N	R	A	R	D	E	T	K	C	Q
K	P	N	R	A	R	D	E	T	K	C	Q
K	P	N	R	A	R	D	E	T	K	C	Q
K	P	N	R	A	R	D	E	T	K	C	Q
K	P	N	R	A	R	D	E	T	K	C	Q
K	P	N	R	A	R	D	E	T	K	C	Q
K	P	N	R	A	R	D	E	T	K	C	Q
K	P	N	R	A	R	D	E	T	K	C	Q
K	P	N	R	A	R	D	E	T	K	C	Q
K	P	N	R	A	R	D	E	T	K	C	Q
K	P	N	R	A	R	D	E	T	K	C	Q
K	P	N	R	A	R	D	E	T	K	C	Q
K	P	N	R	A	R	D	E	T	K	C	Q
K	P	N	R	A	R	D	E	T	K	C	Q
K	P	N	R	A	R	D	E	T	K	C	Q
K	P	N	R	A	R	D	E	T	K	C	Q
K	P	N	R	A	R	D	E	T	K	C	Q
K	P	N	R	A	R	D	E	T	K	C	Q
K	P	N	R	A	R	D	E	T	K	C	Q
K	P	N	R	A	R	D	E	T	K	C	Q
K	P	N	R	A	R	D	E	T	K	C	Q
K	P	N	R	A	R	D	E	T	K	C	Q
K	P	N	R	A	R	D	E	T	K	C	Q
K	P	N	R	A	R	D	E	T	K	C	Q
K	P	N	R	A	R	D	E	T	K	C	Q
K	P	N	R	A	R	D	E	T	K	C	Q
K	P	N	R	A	R	D	E	T	K	C	Q
K	P	N	R	A	R	D	E	T	K	C	Q
K	P	N	R	A	R	D	E	T	K	C	Q
K	P	N	R	A	R	D	E	T	K	C	Q
K	P	N	R	A	R	D	E	T	K	C	Q
K	P	N	R	A	R	D	E	T	K	C	Q
K	P	N	R	A	R	D	E	T	K	C	Q
K	P	N	R	A	R	D	E	T	K	C	Q
K	P	N	R	A	R	D	E	T	K	C	Q
K	P	N	R	A	R	D	E	T	K	C	Q

48	51	54	55	58	59	72	74	88	90	91	94	97	98	99	104	107	109	112	119	120	123	124
N	Q	Q	D	R	D	K	I	V	K	N	K	E	N	K	V	V	H	N	S	E	T	S
D	N	Q	T	E	N	R	I	L	R	N	E	E	H	A	A	I	G	A	S	K	T	K
D	N	Q	T	E	N	R	I	L	R	N	E	E	H	A	A	I	G	A	S	K	T	K
N	Q	Q	D	R	D	K	I	V	K	N	K	E	N	K	V	V	H	S	S	E	T	S
N	Q	Q	D	R	D	K	I	V	K	N	K	E	N	K	V	V	H	A	S	E	T	F
N	Q	K	D	K	N	K	V	I	K	K	L	D	N	T	V	V	H	N	A	E	S	F

N	Q	K	D	K	N	K	V	I	K	K	L	D	N	T	V	V	H	N	A	E	S	F
N	Q	K	D	K	N	K	V	I	K	K	L	D	N	T	V	V	H	N	A	E	S	F
N	Q	K	D	K	N	K	V	I	K	K	L	D	N	T	V	V	H	N	A	E	S	F
N	Q	K	D	K	N	K	V	I	K	K	L	D	N	T	V	V	H	N	A	E	S	F
N	Q	K	D	K	N	K	V	I	K	K	L	D	N	T	V	V	H	N	A	E	S	F
N	Q	K	D	K	N	K	V	I	K	K	L	D	N	T	V	V	H	N	A	E	S	F
N	Q	K	D	K	N	K	V	I	K	K	L	D	N	T	V	V	H	N	A	E	S	F
N	Q	K	D	K	N	K	V	I	K	K	L	D	N	T	V	V	H	N	A	E	S	F
N	Q	K	D	K	N	K	V	I	K	K	L	D	N	T	V	V	H	N	A	E	S	F
N	Q	K	D	K	N	K	V	I	K	K	L	D	N	T	V	V	H	N	A	E	S	F
N	Q	K	D	K	N	K	V	I	K	K	L	D	N	T	V	V	H	N	A	E	S	F
N	Q	K	D	K	N	K	V	I	K	K	L	D	N	T	V	V	H	N	A	E	S	F
N	Q	K	D	K	N	K	V	I	K	K	L	D	N	T	V	V	H	N	A	E	S	F
N	Q	K	D	K	N	K	V	I	K	K	L	D	N	T	V	V	H	N	A	E	S	F
N	Q	K	D	K	N	K	V	I	K	K	L	D	N	T	V	V	H	N	A	E	S	F
N	Q	K	D	K	N	K	V	I	K	K	L	D	N	T	V	V	H	N	A	E	S	F
N	Q	K	D	K	N	K	V	I	K	K	L	D	N	T	V	V	H	N	A	E	S	F
N	Q	K	D	K	N	K	V	I	K	K	L	D	N	T	V	V	H	N	A	E	S	F
N	Q	K	D	K	N	K	V	I	K	K	L	D	N	T	V	V	H	N	A	E	S	F
N	Q	K	D	K	N	K	V	I	K	K	L	D	N	T	V	V	H	N	A	E	S	F
N	Q	K	D	K	N	K	V	I	K	K	L	D	N	T	V	V	H	N	A	E	S	F
N	Q	K	D	K	N	K	V	I	K	K	L	D	N	T	V	V	H	N	A	E	S	F
N	Q	K	D	K	N	K	V	I	K	K	L	D	N	T	V	V	H	N	A	E	S	F
N	Q	K	D	K	N	K	V	I	K	K	L	D	N	T	V	V	H	N	A	E	S	F
N	Q	K	D	K	N	K	V	I	K	K	L	D	N	T	V	V	H	N	A	E	S	F
N	Q	K	D	K	N	K	V	I	K	K	L	D	N	T	V	V	H	N	A	E	S	F
N	Q	K	D	K	N	K	V	I	K	K	L	D	N	T	V	V	H	N	A	E	S	F
N	Q	K	D	K	N	K	V	I	K	K	L	D	N	T	V	V	H	N	A	E	S	F
N	Q	K	D	K	N	K	V	I	K	K	L	D	N	T	V	V	H	N	A	E	S	F
N	Q	K	D	K	N	K	V	I	K	K	L	D	N	T	V	V	H	N	A	E	S	F
N	Q	K	D	K	N	K	V	I	K	K	L	D	N	T	V	V	H	N	A	E	S	F
N	Q	K	D	K	N	K	V	I	K	K	L	D	N	T	V	V	H	N	A	E	S	F
N	Q	K	D	K	N	K	V	I	K	K	L	D	N	T	V	V	H	N	A	E	S	F
N	Q	K	D	K	N	K	V	I	K	K	L	D	N	T	V	V	H	N	A	E	S	F
N	Q	K	D	K	N	K	V	I	K	K	L	D	N	T	V	V	H	N	A	E	S	F
N	Q	K	D	K	N	K	V	I	K	K	L	D	N	T	V	V	H	N	A	E	S	F
N	Q	K	D	K	N	K	V	I	K	K	L	D	N	T	V	V	H	N	A	E	S	F
N	Q	K	D	K	N	K	V	I	K	K	L	D	N	T	V	V	H	N	A	E	S	F
N	Q	K	D	K	N	K	V	I	K	K	L	D	N	T	V	V	H	N	A	E	S	F
N	Q	K	D	K	N	K	V	I	K	K	L	D	N	T	V	V	H	N	A	E	S	F
N	Q	K	D	K	N	K	V	I	K	K	L	D	N	T	V	V	H	N	A	E	S	F
N	Q	K	D	K	N	K	V	I	K	K	L	D	N	T	V	V	H	N	A	E	S	F
N	Q	K	D	K	N	K	V	I	K	K	L	D	N	T	V	V	H	N	A	E	S	F
N	Q	K	D	K	N	K	V	I	K	K	L	D	N	T	V	V	H	N	A	E	S	F
N	Q	K	D	K	N	K	V	I	K	K	L	D	N	T	V	V	H	N	A	E	S	F
N	Q	K	D	K	N	K	V	I	K	K	L	D	N	T	V	V	H	N	A	E	S	F
N	Q	K	D	K	N	K	V	I	K	K	L	D	N	T	V	V	H	N	A	E	S	F
N	Q	K	D	K	N	K	V	I	K	K	L	D	N	T	V	V	H	N	A	E	S	F

wcrL																						
131	132	135	136	137	140	141	144	147	150	153	154	155	157	158	159	163	164	166	167	168	169	170
N	L	E	I	E	L	S	D	L	E	T	P	V	K	R	L	T	Y	D	C	V	V	K
R	L	N	T	D	P	C	N	A	I	S	F	V	T	D	F	P	S	N	-	I	V	Q
R	L	N	T	D	P	C	N	A	I	S	F	V	T	D	F	P	S	N	-	I	V	Q
N	L	E	I	E	L	S	D	L	E	T	P	V	K	R	L	T	Y	D	C	V	V	K
N	L	E	I	E	L	R	D	L	E	T	P	V	K	R	L	T	Y	D	C	V	V	K
D	S	E	V	K	I	N	D	L	E	T	Q	I	K	Q	L	V	Y	N	S	V	I	K

D	S	E	V	K	I	N	D	L	E	T	Q	I	K	Q	L	V	Y	N	S	V	I	K
D	S	E	V	K	I	N	D	L	E	T	Q	I	K	Q	L	V	Y	N	S	V	I	K
D	S	E	V	K	I	N	D	L	E	T	Q	I	K	Q	L	V	Y	N	S	V	I	K
D	S	E	V	K	I	N	D	L	E	T	Q	I	K	Q	L	V	Y	N	S	V	I	K
D	S	E	V	K	I	N	D	L	E	T	Q	I	K	Q	L	V	Y	N	S	V	I	K
D	S	E	V	K	I	N	D	L	E	T	Q	I	K	Q	L	V	Y	N	S	V	I	K
D	S	E	V	K	I	N	D	L	E	T	Q	I	K	Q	L	V	Y	N	S	V	I	K
D	S	E	V	K	I	N	D	L	E	T	Q	I	K	Q	L	V	Y	N	S	V	I	K
D	S	E	V	K	I	N	D	L	E	T	Q	I	K	Q	L	V	Y	N	S	V	I	K
D	S	E	V	K	I	N	D	L	E	T	Q	I	K	Q	L	V	Y	N	S	V	I	K
D	S	E	V	K	I	N	D	L	E	T	Q	I	K	Q	L	V	Y	N	S	V	I	K
D	S	E	V	K	I	N	D	L	E	T	Q	I	K	Q	L	V	Y	N	S	V	I	K
D	S	E	V	K	I	N	D	L	E	T	Q	I	K	Q	L	V	Y	N	S	V	I	K
D	S	E	V	K	I	N	D	L	E	T	Q	I	K	Q	L	V	Y	N	S	V	I	K
D	S	E	V	K	I	N	D	L	E	T	Q	I	K	Q	L	V	Y	N	S	V	I	K
D	S	E	V	K	I	N	D	L	E	T	Q	I	K	Q	L	V	Y	N	S	V	I	K
D	S	E	V	K	I	N	D	L	E	T	Q	I	K	Q	L	V	Y	N	S	V	I	K
D	S	E	V	K	I	N	D	L	E	T	Q	I	K	Q	L	V	Y	N	S	V	I	K
D	S	E	V	K	I	N	D	L	E	T	Q	I	K	Q	L	V	Y	N	S	V	I	K
D	S	E	V	K	I	N	D	L	E	T	Q	I	K	Q	L	V	Y	N	S	V	I	K
D	S	E	V	K	I	N	D	L	E	T	Q	I	K	Q	L	V	Y	N	S	V	I	K
D	S	E	V	K	I	N	D	L	E	T	Q	I	K	Q	L	V	Y	N	S	V	I	K
D	S	E	V	K	I	N	D	L	E	T	Q	I	K	Q	L	V	Y	N	S	V	I	K
D	S	E	V	K	I	N	D	L	E	T	Q	I	K	Q	L	V	Y	N	S	V	I	K
D	S	E	V	K	I	N	D	L	E	T	Q	I	K	Q	L	V	Y	N	S	V	I	K
D	S	E	V	K	I	N	D	L	E	T	Q	I	K	Q	L	V	Y	N	S	V	I	K
D	S	E	V	K	I	N	D	L	E	T	Q	I	K	Q	L	V	Y	N	S	V	I	K
D	S	E	V	K	I	N	D	L	E	T	Q	I	K	Q	L	V	Y	N	S	V	I	K
D	S	E	V	K	I	N	D	L	E	T	Q	I	K	Q	L	V	Y	N	S	V	I	K
D	S	E	V	K	I	N	D	L	E	T	Q	I	K	Q	L	V	Y	N	S	V	I	K
D	S	E	V	K	I	N	D	L	E	T	Q	I	K	Q	L	V	Y	N	S	V	I	K
D	S	E	V	K	I	N	D	L	E	T	Q	I	K	Q	L	V	Y	N	S	V	I	K
D	S	E	V	K	I	N	D	L	E	T	Q	I	K	Q	L	V	Y	N	S	V	I	K
D	S	E	V	K	I	N	D	L	E	T	Q	I	K	Q	L	V	Y	N	S	V	I	K
D	S	E	V	K	I	N	D	L	E	T	Q	I	K	Q	L	V	Y	N	S	V	I	K
D	S	E	V	K	I	N	D	L	E	T	Q	I	K	Q	L	V	Y	N	S	V	I	K
D	S	E	V	K	I	N	D	L	E	T	Q	I	K	Q	L	V	Y	N	S	V	I	K
D	S	E	V	K	I	N	D	L	E	T	Q	I	K	Q	L	V	Y	N	S	V	I	K
D	S	E	V	K	I	N	D	L	E	T	Q	I	K	Q	L	V	Y	N	S	V	I	K
D	S	E	V	K	I	N	D	L	E	T	Q	I	K	Q	L	V	Y	N	S	V	I	K
D	S	E	V	K	I	N	D	L	E	T	Q	I	K	Q	L	V	Y	N	S	V	I	K
D	S	E	V	K	I	N	D	L	E	T	Q	I	K	Q	L	V	Y	N	S	V	I	K
D	S	E	V	K	I	N	D	L	E	T	Q	I	K	Q	L	V	Y	N	S	V	I	K
D	S	E	V	K	I	N	D	L	E	T	Q	I	K	Q	L	V	Y	N	S	V	I	K
D	S	E	V	K	I	N	D	L	E	T	Q	I	K	Q	L	V	Y	N	S	V	I	K
D	S	E	V	K	I	N	D	L	E	T	Q	I	K	Q	L	V	Y	N	S	V	I	K
D	S	E	V	K	I	N	D	L	E	T	Q	I	K	Q	L	V	Y	N	S	V	I	K
D	S	E	V	K	I	N	D	L	E	T	Q	I	K	Q	L	V	Y	N	S	V	I	K

	171	172	179	186	187	189	190	191	192	194	197	200	206	211	212	213	214	215	216	218	223	224	227
	N	E	E	S	L	D	G	V	E	I	D	F	S	S	L	R	N	Q	M	S	K	L	S
	T	E	A	Y	V	A	N	A	R	L	E	Y	S	P	V	R	V	R	I	N	Y	I	D
	T	E	A	Y	V	A	N	A	R	L	E	Y	S	P	V	R	V	R	I	N	Y	I	D
	N	E	E	S	L	D	G	V	E	I	D	F	S	S	L	R	N	Q	M	S	K	L	S
	N	E	E	S	L	Y	G	V	E	I	D	F	S	S	L	R	D	Q	M	S	K	F	G
	S	Q	E	S	L	D	E	V	A	I	D	F	N	S	L	M	N	R	V	R	K	L	S

S	Q	E	S	L	D	E	V	A	I	D	F	N	S	L	M	N	R	V	R	K	L	S
S	Q	E	S	L	D	E	V	A	I	D	F	N	S	L	M	N	R	V	R	K	L	S
S	Q	E	S	L	D	E	V	A	I	D	F	N	S	L	M	N	R	V	R	K	L	S
S	Q	E	S	L	D	E	V	A	I	D	F	N	S	L	M	N	R	V	R	K	L	S
S	Q	E	S	L	D	E	V	A	I	D	F	N	S	L	M	N	R	V	R	K	L	S
S	Q	E	S	L	D	E	V	A	I	D	F	N	S	L	M	N	R	V	R	K	L	S
S	Q	E	S	L	D	E	V	A	I	D	F	N	S	L	M	N	R	V	R	K	L	S
S	Q	E	S	L	D	E	V	A	I	D	F	N	S	L	M	N	R	V	R	K	L	S
S	Q	E	S	L	D	E	V	A	I	D	F	N	S	L	M	N	R	V	R	K	L	S
S	Q	E	S	L	D	E	V	A	I	D	F	N	S	L	M	N	R	V	R	K	L	S
S	Q	E	S	L	D	E	V	A	I	D	F	N	S	L	M	N	R	V	R	K	L	S
S	Q	E	S	L	D	E	V	A	I	D	F	N	S	L	M	N	R	V	R	K	L	S
S	Q	E	S	L	D	E	V	A	I	D	F	N	S	L	M	N	R	V	R	K	L	S
S	Q	E	S	L	D	E	V	A	I	D	F	N	S	L	M	N	R	V	R	K	L	S
S	Q	E	S	L	D	E	V	A	I	D	F	N	S	L	M	N	R	V	R	K	L	S
S	Q	E	S	L	D	E	V	A	I	D	F	N	S	L	M	N	R	V	R	K	L	S
S	Q	E	S	L	D	E	V	A	I	D	F	N	S	L	M	N	R	V	R	K	L	S
S	Q	E	S	L	D	E	V	A	I	D	F	N	S	L	M	N	R	V	R	K	L	S
S	Q	E	S	L	D	E	V	A	I	D	F	N	S	L	M	N	R	V	R	K	L	S
S	Q	E	S	L	D	E	V	A	I	D	F	N	S	L	M	N	R	V	R	K	L	S
S	Q	E	S	L	D	E	V	A	I	D	F	N	S	L	M	N	R	V	R	K	L	S
S	Q	E	S	L	D	E	V	A	I	D	F	N	S	L	M	N	R	V	R	K	L	S
S	Q	E	S	L	D	E	V	A	I	D	F	N	S	L	M	N	R	V	R	K	L	S
S	Q	E	S	L	D	E	V	A	I	D	F	N	S	L	M	N	R	V	R	K	L	S
S	Q	E	S	L	D	E	V	A	I	D	F	N	S	L	M	N	R	V	R	K	L	S
S	Q	E	S	L	D	E	V	A	I	D	F	N	S	L	M	N	R	V	R	K	L	S
S	Q	E	S	L	D	E	V	A	I	D	F	N	S	L	M	N	R	V	R	K	L	S
S	Q	E	S	L	D	E	V	A	I	D	F	N	S	L	M	N	R	V	R	K	L	S
S	Q	E	S	L	D	E	V	A	I	D	F	N	S	L	M	N	R	V	R	K	L	S
S	Q	E	S	L	D	E	V	A	I	D	F	N	S	L	M	N	R	V	R	K	L	S
S	Q	E	S	L	D	E	V	A	I	D	F	N	S	L	M	N	R	V	R	K	L	S
S	Q	E	S	L	D	E	V	A	I	D	F	N	S	L	M	N	R	V	R	K	L	S
S	Q	E	S	L	D	E	V	A	I	D	F	N	S	L	M	N	R	V	R	K	L	S
S	Q	E	S	L	D	E	V	A	I	D	F	N	S	L	M	N	R	V	R	K	L	S
S	Q	E	S	L	D	E	V	A	I	D	F	N	S	L	M	N	R	V	R	K	L	S
S	Q	E	S	L	D	E	V	A	I	D	F	N	S	L	M	N	R	V	R	K	L	S
S	Q	E	S	L	D	E	V	A	I	D	F	N	S	L	M	N	R	V	R	K	L	S
S	Q	E	S	L	D	E	V	A	I	D	F	N	S	L	M	N	R	V	R	K	L	S
S	Q	E	S	L	D	E	V	A	I	D	F	N	S	L	M	N	R	V	R	K	L	S
S	Q	E	S	L	D	E	V	A	I	D	F	N	S	L	M	N	R	V	R	K	L	S
S	Q	E	S	L	D	E	V	A	I	D	F	N	S	L	M	N	R	V	R	K	L	S
S	Q	E	S	L	D	E	V	A	I	D	F	N	S	L	M	N	R	V	R	K	L	S
S	Q	E	S	L	D	E	V	A	I	D	F	N	S	L	M	N	R	V	R	K	L	S
S	Q	E	S	L	D	E	V	A	I	D	F	N	S	L	M	N	R	V	R	K	L	S
S	Q	E	S	L	D	E	V	A	I	D	F	N	S	L	M	N	R	V	R	K	L	S
S	Q	E	S	L	D	E	V	A	I	D	F	N	S	L	M	N	R	V	R	K	L	S
S	Q	E	S	L	D	E	V	A	I	D	F	N	S	L	M	N	R	V	R	K	L	S
S	Q	E	S	L	D	E	V	A	I	D	F	N	S	L	M	N	R	V	R	K	L	S

228	229	230	231	234	237	238
N	I	L	E	R	K	G
K	I	L	L	K	E	R
K	I	L	L	K	E	R
N	I	L	E	R	K	G
N	V	L	K	R	K	G
N	V	S	E	R	K	G

2	5	8	9	11	12	14	15	16	19	20	23	28	30	31
R	P	L	I	A	F	S	S	F	L	V	F	F	G	H
K	L	V	F	L	Q	C	A	L	F	T	Y	T	T	Y
K	L	V	F	L	Q	C	A	L	F	T	Y	T	T	Y
R	P	L	I	A	F	S	S	F	L	V	F	F	G	H
R	P	L	I	A	F	S	S	F	L	V	F	F	G	H
R	P	L	I	A	F	S	S	F	L	V	F	F	G	H

N	V	S	E	R	K	G
N	V	S	E	R	K	G
N	V	S	E	R	K	G
N	V	S	E	R	K	G
N	V	S	E	R	K	G
N	V	S	E	R	K	G
N	V	S	E	R	K	G
N	V	S	E	R	K	G
N	V	S	E	R	K	G
N	V	S	E	R	K	G
N	V	S	E	R	K	G
N	V	S	E	R	K	G
N	V	S	E	R	K	G
N	V	S	E	R	K	G
N	V	S	E	R	K	G
N	V	S	E	R	K	G
N	V	S	E	R	K	G
N	V	S	E	R	K	G
N	V	S	E	R	K	G
N	V	S	E	R	K	G
N	V	S	E	R	K	G
N	V	S	E	R	K	G
N	V	S	E	R	K	G
N	V	S	E	R	K	G
N	V	S	E	R	K	G
N	V	S	E	R	K	G
N	V	S	E	R	K	G
N	V	S	E	R	K	G
N	V	S	E	R	K	G
N	V	S	E	R	K	G
N	V	S	E	R	K	G
N	V	S	E	R	K	G
N	V	S	E	R	K	G
N	V	S	E	R	K	G
N	V	S	E	R	K	G
N	V	S	E	R	K	G
N	V	S	E	R	K	G
N	V	S	E	R	K	G
N	V	S	E	R	K	G
N	V	S	E	R	K	G
N	V	S	E	R	K	G
N	V	S	E	R	K	G
N	V	S	E	R	K	G
N	V	S	E	R	K	G
N	V	S	E	R	K	G
N	V	S	E	R	K	G
N	V	S	E	R	K	G
N	V	S	E	R	K	G

R	P	L	I	A	F	S	S	F	L	V	F	F	G	H
R	P	L	I	A	F	S	S	F	L	V	F	F	G	H
R	P	L	I	A	F	S	S	F	L	V	F	F	G	H
R	P	L	I	A	F	S	S	F	L	V	F	F	G	H
R	P	L	I	A	F	S	S	F	L	V	F	F	G	H
R	P	L	I	A	F	S	S	F	L	V	F	F	G	H
R	P	L	I	A	F	S	S	F	L	V	F	F	G	H
R	P	L	I	A	F	S	S	F	L	V	F	F	G	H
R	P	L	I	A	F	S	S	F	L	V	F	F	G	H
R	P	L	I	A	F	S	S	F	L	V	F	F	G	H
R	P	L	I	A	F	S	S	F	L	V	F	F	G	H
R	P	L	I	A	F	S	S	F	L	V	F	F	G	H
R	P	L	I	A	F	S	S	F	L	V	F	F	G	H
R	P	L	I	A	F	S	S	F	L	V	F	F	G	H
R	P	L	I	A	F	S	S	F	L	V	F	F	G	H
R	P	L	I	A	F	S	S	F	L	V	F	F	G	H
R	P	L	I	A	F	S	S	F	L	V	F	F	G	H
R	P	L	I	A	F	S	S	F	L	V	F	F	G	H
R	P	L	I	A	F	S	S	F	L	V	F	F	G	H
R	P	L	I	A	F	S	S	F	L	V	F	F	G	H
R	P	L	I	A	F	S	S	F	L	V	F	F	G	H
R	P	L	I	A	F	S	S	F	L	V	F	F	G	H
R	P	L	I	A	F	S	S	F	L	V	F	F	G	H
R	P	L	I	A	F	S	S	F	L	V	F	F	G	H
R	P	L	I	A	F	S	S	F	L	V	F	F	G	H
R	P	L	I	A	F	S	S	F	L	V	F	F	G	H
R	P	L	I	A	F	S	S	F	L	V	F	F	G	H
R	P	L	I	A	F	S	S	F	L	V	F	F	G	H
R	P	L	I	A	F	S	S	F	L	V	F	F	G	H
R	P	L	I	A	F	S	S	F	L	V	F	F	G	H
R	P	L	I	A	F	S	S	F	L	V	F	F	G	H
R	P	L	I	A	F	S	S	F	L	V	F	F	G	H
R	P	L	I	A	F	S	S	F	L	V	F	F	G	H
R	P	L	I	A	F	S	S	F	L	V	F	F	G	H
R	P	L	I	A	F	S	S	F	L	V	F	F	G	H
R	P	L	I	A	F	S	S	F	L	V	F	F	G	H
R	P	L	I	A	F	S	S	F	L	V	F	F	G	H
R	P	L	I	A	F	S	S	F	L	V	F	F	G	H
R	P	L	I	A	F	S	S	F	L	V	F	F	G	H
R	P	L	I	A	F	S	S	F	L	V	F	F	G	H
R	P	L	I	A	F	S	S	F	L	V	F	F	G	H
R	P	L	I	A	F	S	S	F	L	V	F	F	G	H
R	P	L	I	A	F	S	S	F	L	V	F	F	G	H
R	P	L	I	A	F	S	S	F	L	V	F	F	G	H
R	P	L	I	A	F	S	S	F	L	V	F	F	G	H
R	P	L	I	A	F	S	S	F	L	V	F	F	G	H
R	P	L	I	A	F	S	S	F	L	V	F	F	G	H
R	P	L	I	A	F	S	S	F	L	V	F	F	G	H

33	34	36	37	38	39	40	43	44	45	48	49	51	52	53	54	57	58	59	63	65	66	67
T	L	S	R	L	W	I	N	G	S	L	S	L	L	G	L	V	I	L	S	K	Y	R
P	S	D	F	F	T	L	K	F	C	F	T	A	A	S	I	I	F	I	Y	R	L	H
P	S	D	F	F	T	L	K	F	C	F	T	A	A	S	I	I	F	I	Y	R	L	H
T	L	S	R	L	W	I	N	G	S	L	S	L	L	G	L	V	I	L	S	K	Y	R
T	L	S	R	L	W	I	K	E	S	L	S	L	L	G	S	V	I	L	S	K	Y	R
T	L	S	R	L	W	I	K	E	S	L	S	L	L	G	S	V	I	L	S	K	Y	R

T	L	S	R	L	W	I	K	E	S	L	S	L	L	G	S	V	I	L	S	K	Y	R
T	L	S	R	L	W	I	K	E	S	L	S	L	L	G	S	V	I	L	S	K	Y	R
T	L	S	R	L	W	I	K	E	S	L	S	L	L	G	S	V	I	L	S	K	Y	R
T	L	S	R	L	W	I	K	E	S	L	S	L	L	G	S	V	I	L	S	K	Y	R
T	L	S	R	L	W	I	K	E	S	L	S	L	L	G	S	V	I	L	S	K	Y	R
T	L	S	R	L	W	I	K	E	S	L	S	L	L	G	S	V	I	L	S	K	Y	R
T	L	S	R	L	W	I	K	E	S	L	S	L	L	G	S	V	I	L	S	K	Y	R
T	L	S	R	L	W	I	K	E	S	L	S	L	L	G	S	V	I	L	S	K	Y	R
T	L	S	R	L	W	I	K	E	S	L	S	L	L	G	S	V	I	L	S	K	Y	R
T	L	S	R	L	W	I	K	E	S	L	S	L	L	G	S	V	I	L	S	K	Y	R
T	L	S	R	L	W	I	K	E	S	L	S	L	L	G	S	V	I	L	S	K	Y	R
T	L	S	R	L	W	I	K	E	S	L	S	L	L	G	S	V	I	L	S	K	Y	R
T	L	S	R	L	W	I	K	E	S	L	S	L	L	G	S	V	I	L	S	K	Y	R
T	L	S	R	L	W	I	K	E	S	L	S	L	L	G	S	V	I	L	S	K	Y	R
T	L	S	R	L	W	I	K	E	S	L	S	L	L	G	S	V	I	L	S	K	Y	R
T	L	S	R	L	W	I	K	E	S	L	S	L	L	G	S	V	I	L	S	K	Y	R
T	L	S	R	L	W	I	K	E	S	L	S	L	L	G	S	V	I	L	S	K	Y	R
T	L	S	R	L	W	I	K	E	S	L	S	L	L	G	S	V	I	L	S	K	Y	R
T	L	S	R	L	W	I	K	E	S	L	S	L	L	G	S	V	I	L	S	K	Y	R
T	L	S	R	L	W	I	K	E	S	L	S	L	L	G	S	V	I	L	S	K	Y	R
T	L	S	R	L	W	I	K	E	S	L	S	L	L	G	S	V	I	L	S	K	Y	R
T	L	S	R	L	W	I	K	E	S	L	S	L	L	G	S	V	I	L	S	K	Y	R
T	L	S	R	L	W	I	K	E	S	L	S	L	L	G	S	V	I	L	S	K	Y	R
T	L	S	R	L	W	I	K	E	S	L	S	L	L	G	S	V	I	L	S	K	Y	R
T	L	S	R	L	W	I	K	E	S	L	S	L	L	G	S	V	I	L	S	K	Y	R
T	L	S	R	L	W	I	K	E	S	L	S	L	L	G	S	V	I	L	S	K	Y	R
T	L	S	R	L	W	I	K	E	S	L	S	L	L	G	S	V	I	L	S	K	Y	R
T	L	S	R	L	W	I	K	E	S	L	S	L	L	G	S	V	I	L	S	K	Y	R
T	L	S	R	L	W	I	K	E	S	L	S	L	L	G	S	V	I	L	S	K	Y	R
T	L	S	R	L	W	I	K	E	S	L	S	L	L	G	S	V	I	L	S	K	Y	R
T	L	S	R	L	W	I	K	E	S	L	S	L	L	G	S	V	I	L	S	K	Y	R
T	L	S	R	L	W	I	K	E	S	L	S	L	L	G	S	V	I	L	S	K	Y	R
T	L	S	R	L	W	I	K	E	S	L	S	L	L	G	S	V	I	L	S	K	Y	R
T	L	S	R	L	W	I	K	E	S	L	S	L	L	G	S	V	I	L	S	K	Y	R
T	L	S	R	L	W	I	K	E	S	L	S	L	L	G	S	V	I	L	S	K	Y	R
T	L	S	R	L	W	I	K	E	S	L	S	L	L	G	S	V	I	L	S	K	Y	R
T	L	S	R	L	W	I	K	E	S	L	S	L	L	G	S	V	I	L	S	K	Y	R
T	L	S	R	L	W	I	K	E	S	L	S	L	L	G	S	V	I	L	S	K	Y	R
T	L	S	R	L	W	I	K	E	S	L	S	L	L	G	S	V	I	L	S	K	Y	R
T	L	S	R	L	W	I	K	E	S	L	S	L	L	G	S	V	I	L	S	K	Y	R
T	L	S	R	L	W	I	K	E	S	L	S	L	L	G	S	V	I	L	S	K	Y	R
T	L	S	R	L	W	I	K	E	S	L	S	L	L	G	S	V	I	L	S	K	Y	R
T	L	S	R	L	W	I	K	E	S	L	S	L	L	G	S	V	I	L	S	K	Y	R
T	L	S	R	L	W	I	K	E	S	L	S	L	L	G	S	V	I	L	S	K	Y	R
T	L	S	R	L	W	I	K	E	S	L	S	L	L	G	S	V	I	L	S	K	Y	R
T	L	S	R	L	W	I	K	E	S	L	S	L	L	G	S	V	I	L	S	K	Y	R
T	L	S	R	L	W	I	K	E	S	L	S	L	L	G	S	V	I	L	S	K	Y	R
T	L	S	R	L	W	I	K	E	S	L	S	L	L	G	S	V	I	L	S	K	Y	R

68	69	72	75	76	77	78	80	81	82	83	84	85	86	87	88	89	90	91	92	95	96	97
H	S	C	H	L	T	I	F	Y	C	N	Y	F	N	F	H	G	K	W	V	C	W	A
L	A	Y	Y	I	A	V	Y	C	F	Y	S	Y	Y	I	A	F	N	R	H	F	G	F
L	A	Y	Y	I	A	V	Y	C	F	Y	S	Y	Y	I	A	F	N	R	H	F	G	F
H	S	C	H	L	T	I	F	Y	C	N	Y	F	N	F	H	G	K	W	V	C	W	A
H	S	C	Y	L	T	I	F	Y	C	N	D	F	N	F	Y	V	K	W	V	*	W	G
H	S	C	Y	L	T	I	F	Y	C	N	D	F	N	F	Y	V	K	W	V	*	W	G

H	S	C	Y	L	T	I	F	Y	C	N	D	F	N	F	Y	V	K	W	V	*	W	G
H	S	C	Y	L	T	I	F	Y	C	N	D	F	N	F	Y	V	K	W	V	*	W	G
H	S	C	Y	L	T	I	F	Y	C	N	D	F	N	F	Y	V	K	W	V	*	W	G
H	S	C	Y	L	T	I	F	Y	C	N	D	F	N	F	Y	V	K	W	V	*	W	G
H	S	C	Y	L	T	I	F	Y	C	N	D	F	N	F	Y	V	K	W	V	*	W	G
H	S	C	Y	L	T	I	F	Y	C	N	D	F	N	F	Y	V	K	W	V	*	W	G
H	S	C	Y	L	T	I	F	Y	C	N	D	F	N	F	Y	V	K	W	V	*	W	G
H	S	C	Y	L	T	I	F	Y	C	N	D	F	N	F	Y	V	K	W	V	*	W	G
H	S	C	Y	L	T	I	F	Y	C	N	D	F	N	F	Y	V	K	W	V	*	W	G
H	S	C	Y	L	T	I	F	Y	C	N	D	F	N	F	Y	V	K	W	V	*	W	G
H	S	C	Y	L	T	I	F	Y	C	N	D	F	N	F	Y	V	K	W	V	*	W	G
H	S	C	Y	L	T	I	F	Y	C	N	D	F	N	F	Y	V	K	W	V	*	W	G
H	S	C	Y	L	T	I	F	Y	C	N	D	F	N	F	Y	V	K	W	V	*	W	G
H	S	C	Y	L	T	I	F	Y	C	N	D	F	N	F	Y	V	K	W	V	*	W	G
H	S	C	Y	L	T	I	F	Y	C	N	D	F	N	F	Y	V	K	W	V	*	W	G
H	S	C	Y	L	T	I	F	Y	C	N	D	F	N	F	Y	V	K	W	V	*	W	G
H	S	C	Y	L	T	I	F	Y	C	N	D	F	N	F	Y	V	K	W	V	*	W	G
H	S	C	Y	L	T	I	F	Y	C	N	D	F	N	F	Y	V	K	W	V	*	W	G
H	S	C	Y	L	T	I	F	Y	C	N	D	F	N	F	Y	V	K	W	V	*	W	G
H	S	C	Y	L	T	I	F	Y	C	N	D	F	N	F	Y	V	K	W	V	*	W	G
H	S	C	Y	L	T	I	F	Y	C	N	D	F	N	F	Y	V	K	W	V	*	W	G
H	S	C	Y	L	T	I	F	Y	C	N	D	F	N	F	Y	V	K	W	V	*	W	G
H	S	C	Y	L	T	I	F	Y	C	N	D	F	N	F	Y	V	K	W	V	*	W	G
H	S	C	Y	L	T	I	F	Y	C	N	D	F	N	F	Y	V	K	W	V	*	W	G
H	S	C	Y	L	T	I	F	Y	C	N	D	F	N	F	Y	V	K	W	V	*	W	G
H	S	C	Y	L	T	I	F	Y	C	N	D	F	N	F	Y	V	K	W	V	*	W	G
H	S	C	Y	L	T	I	F	Y	C	N	D	F	N	F	Y	V	K	W	V	*	W	G
H	S	C	Y	L	T	I	F	Y	C	N	D	F	N	F	Y	V	K	W	V	*	W	G
H	S	C	Y	L	T	I	F	Y	C	N	D	F	N	F	Y	V	K	W	V	*	W	G
H	S	C	Y	L	T	I	F	Y	C	N	D	F	N	F	Y	V	K	W	V	*	W	G
H	S	C	Y	L	T	I	F	Y	C	N	D	F	N	F	Y	V	K	W	V	*	W	G
H	S	C	Y	L	T	I	F	Y	C	N	D	F	N	F	Y	V	K	W	V	*	W	G
H	S	C	Y	L	T	I	F	Y	C	N	D	F	N	F	Y	V	K	W	V	*	W	G
H	S	C	Y	L	T	I	F	Y	C	N	D	F	N	F	Y	V	K	W	V	*	W	G
H	S	C	Y	L	T	I	F	Y	C	N	D	F	N	F	Y	V	K	W	V	*	W	G
H	S	C	Y	L	T	I	F	Y	C	N	D	F	N	F	Y	V	K	W	V	*	W	G
H	S	C	Y	L	T	I	F	Y	C	N	D	F	N	F	Y	V	K	W	V	*	W	G
H	S	C	Y	L	T	I	F	Y	C	N	D	F	N	F	Y	V	K	W	V	*	W	G
H	S	C	Y	L	T	I	F	Y	C	N	D	F	N	F	Y	V	K	W	V	*	W	G
H	S	C	Y	L	T	I	F	Y	C	N	D	F	N	F	Y	V	K	W	V	*	W	G
H	S	C	Y	L	T	I	F	Y	C	N	D	F	N	F	Y	V	K	W	V	*	W	G
H	S	C	Y	L	T	I	F	Y	C	N	D	F	N	F	Y	V	K	W	V	*	W	G
H	S	C	Y	L	T	I	F	Y	C	N	D	F	N	F	Y	V	K	W	V	*	W	G
H	S	C	Y	L	T	I	F	Y	C	N	D	F	N	F	Y	V	K	W	V	*	W	G
H	S	C	Y	L	T	I	F	Y	C	N	D	F	N	F	Y	V	K	W	V	*	W	G
H	S	C	Y	L	T	I	F	Y	C	N	D	F	N	F	Y	V	K	W	V	*	W	G
H	S	C	Y	L	T	I	F	Y	C	N	D	F	N	F	Y	V	K	W	V	*	W	G
H	S	C	Y	L	T	I	F	Y	C	N	D	F	N	F	Y	V	K	W	V	*	W	G

100	101	104	105	106	107	109	113	117	119	120	121	126	127	128	129	130	131	132	135	137	139	141
D	F	N	I	H	L	I	S	E	N	S	V	L	S	L	Y	T	Y	S	R	Q	D	C
S	L	D	L	Y	I	I	D	G	D	Y	K	I	F	I	L	F	N	S	L	K	F	S
S	L	D	L	Y	I	I	D	G	D	Y	K	I	F	I	L	F	N	S	L	K	F	S
D	F	N	I	H	L	I	S	E	N	S	V	L	S	L	Y	T	Y	S	R	Q	D	C
D	F	N	I	H	L	V	S	E	N	S	V	I	S	L	Y	T	Y	R	R	Q	D	Y
D	F	N	I	H	L	V	S	E	N	S	V	I	S	L	Y	T	Y	R	R	Q	D	Y

D	F	N	I	H	L	V	S	E	N	S	V	I	S	L	Y	T	Y	R	R	Q	D	Y
D	F	N	I	H	L	V	S	E	N	S	V	I	S	L	Y	T	Y	R	R	Q	D	Y
D	F	N	I	H	L	V	S	E	N	S	V	I	S	L	Y	T	Y	R	R	Q	D	Y
D	F	N	I	H	L	V	S	E	N	S	V	I	S	L	Y	T	Y	R	R	Q	D	Y
D	F	N	I	H	L	V	S	E	N	S	V	I	S	L	Y	T	Y	R	R	Q	D	Y
D	F	N	I	H	L	V	S	E	N	S	V	I	S	L	Y	T	Y	R	R	Q	D	Y
D	F	N	I	H	L	V	S	E	N	S	V	I	S	L	Y	T	Y	R	R	Q	D	Y
D	F	N	I	H	L	V	S	E	N	S	V	I	S	L	Y	T	Y	R	R	Q	D	Y
D	F	N	I	H	L	V	S	E	N	S	V	I	S	L	Y	T	Y	R	R	Q	D	Y
D	F	N	I	H	L	V	S	E	N	S	V	I	S	L	Y	T	Y	R	R	Q	D	Y
D	F	N	I	H	L	V	S	E	N	S	V	I	S	L	Y	T	Y	R	R	Q	D	Y
D	F	N	I	H	L	V	S	E	N	S	V	I	S	L	Y	T	Y	R	R	Q	D	Y
D	F	N	I	H	L	V	S	E	N	S	V	I	S	L	Y	T	Y	R	R	Q	D	Y
D	F	N	I	H	L	V	S	E	N	S	V	I	S	L	Y	T	Y	R	R	Q	D	Y
D	F	N	I	H	L	V	S	E	N	S	V	I	S	L	Y	T	Y	R	R	Q	D	Y
D	F	N	I	H	L	V	S	E	N	S	V	I	S	L	Y	T	Y	R	R	Q	D	Y
D	F	N	I	H	L	V	S	E	N	S	V	I	S	L	Y	T	Y	R	R	Q	D	Y
D	F	N	I	H	L	V	S	E	N	S	V	I	S	L	Y	T	Y	R	R	Q	D	Y
D	F	N	I	H	L	V	S	E	N	S	V	I	S	L	Y	T	Y	R	R	Q	D	Y
D	F	N	I	H	L	V	S	E	N	S	V	I	S	L	Y	T	Y	R	R	Q	D	Y
D	F	N	I	H	L	V	S	E	N	S	V	I	S	L	Y	T	Y	R	R	Q	D	Y
D	F	N	I	H	L	V	S	E	N	S	V	I	S	L	Y	T	Y	R	R	Q	D	Y
D	F	N	I	H	L	V	S	E	N	S	V	I	S	L	Y	T	Y	R	R	Q	D	Y
D	F	N	I	H	L	V	S	E	N	S	V	I	S	L	Y	T	Y	R	R	Q	D	Y
D	F	N	I	H	L	V	S	E	N	S	V	I	S	L	Y	T	Y	R	R	Q	D	Y
D	F	N	I	H	L	V	S	E	N	S	V	I	S	L	Y	T	Y	R	R	Q	D	Y
D	F	N	I	H	L	V	S	E	N	S	V	I	S	L	Y	T	Y	R	R	Q	D	Y
D	F	N	I	H	L	V	S	E	N	S	V	I	S	L	Y	T	Y	R	R	Q	D	Y
D	F	N	I	H	L	V	S	E	N	S	V	I	S	L	Y	T	Y	R	R	Q	D	Y
D	F	N	I	H	L	V	S	E	N	S	V	I	S	L	Y	T	Y	R	R	Q	D	Y
D	F	N	I	H	L	V	S	E	N	S	V	I	S	L	Y	T	Y	R	R	Q	D	Y
D	F	N	I	H	L	V	S	E	N	S	V	I	S	L	Y	T	Y	R	R	Q	D	Y
D	F	N	I	H	L	V	S	E	N	S	V	I	S	L	Y	T	Y	R	R	Q	D	Y
D	F	N	I	H	L	V	S	E	N	S	V	I	S	L	Y	T	Y	R	R	Q	D	Y
D	F	N	I	H	L	V	S	E	N	S	V	I	S	L	Y	T	Y	R	R	Q	D	Y
D	F	N	I	H	L	V	S	E	N	S	V	I	S	L	Y	T	Y	R	R	Q	D	Y
D	F	N	I	H	L	V	S	E	N	S	V	I	S	L	Y	T	Y	R	R	Q	D	Y
D	F	N	I	H	L	V	S	E	N	S	V	I	S	L	Y	T	Y	R	R	Q	D	Y
D	F	N	I	H	L	V	S	E	N	S	V	I	S	L	Y	T	Y	R	R	Q	D	Y
D	F	N	I	H	L	V	S	E	N	S	V	I	S	L	Y	T	Y	R	R	Q	D	Y
D	F	N	I	H	L	V	S	E	N	S	V	I	S	L	Y	T	Y	R	R	Q	D	Y
D	F	N	I	H	L	V	S	E	N	S	V	I	S	L	Y	T	Y	R	R	Q	D	Y
D	F	N	I	H	L	V	S	E	N	S	V	I	S	L	Y	T	Y	R	R	Q	D	Y
D	F	N	I	H	L	V	S	E	N	S	V	I	S	L	Y	T	Y	R	R	Q	D	Y
D	F	N	I	H	L	V	S	E	N	S	V	I	S	L	Y	T	Y	R	R	Q	D	Y
D	F	N	I	H	L	V	S	E	N	S	V	I	S	L	Y	T	Y	R	R	Q	D	Y
D	F	N	I	H	L	V	S	E	N	S	V	I	S	L	Y	T	Y	R	R	Q	D	Y
D	F	N	I	H	L	V	S	E	N	S	V	I	S	L	Y	T	Y	R	R	Q	D	Y

																				wzy		
142	143	146	147	149	151	152	153	155	156	158	159	161	162	165	166	167	168	169	170	171	172	173
F	S	H	K	F	Y	Y	F	R	T	S	G	F	P	C	Y	R	I	S	R	I	S	I
Y	D	Y	Q	L	Y	N	L	G	A	P	S	I	T	N	Y	S	F	F	H	F	F	L
Y	D	Y	Q	L	Y	N	L	G	A	P	S	I	T	N	Y	S	F	F	H	F	F	L
F	S	H	K	F	Y	Y	F	R	T	S	G	F	P	C	Y	R	I	S	R	I	S	I
F	S	H	K	F	C	Y	F	R	T	S	G	F	P	Y	S	R	I	S	R	I	S	L
F	S	H	K	F	C	Y	F	R	T	S	G	F	P	Y	S	R	I	S	R	I	S	L

F	S	H	K	F	C	Y	F	R	T	S	G	F	P	Y	S	R	I	S	R	I	S	L
F	S	H	K	F	C	Y	F	R	T	S	G	F	P	Y	S	R	I	S	R	I	S	L
F	S	H	K	F	C	Y	F	R	T	S	G	F	P	Y	S	R	I	S	R	I	S	L
F	S	H	K	F	C	Y	F	R	T	S	G	F	P	Y	S	R	I	S	R	I	S	L
F	S	H	K	F	C	Y	F	R	T	S	G	F	P	Y	S	R	I	S	R	I	S	L
F	S	H	K	F	C	Y	F	R	T	S	G	F	P	Y	S	R	I	S	R	I	S	L
F	S	H	K	F	C	Y	F	R	T	S	G	F	P	Y	S	R	I	S	R	I	S	L
F	S	H	K	F	C	Y	F	R	T	S	G	F	P	Y	S	R	I	S	R	I	S	L
F	S	H	K	F	C	Y	F	R	T	S	G	F	P	Y	S	R	I	S	R	I	S	L
F	S	H	K	F	C	Y	F	R	T	S	G	F	P	Y	S	R	I	S	R	I	S	L
F	S	H	K	F	C	Y	F	R	T	S	G	F	P	Y	S	R	I	S	R	I	S	L
F	S	H	K	F	C	Y	F	R	T	S	G	F	P	Y	S	R	I	S	R	I	S	L
F	S	H	K	F	C	Y	F	R	T	S	G	F	P	Y	S	R	I	S	R	I	S	L
F	S	H	K	F	C	Y	F	R	T	S	G	F	P	Y	S	R	I	S	R	I	S	L
F	S	H	K	F	C	Y	F	R	T	S	G	F	P	Y	S	R	I	S	R	I	S	L
F	S	H	K	F	C	Y	F	R	T	S	G	F	P	Y	S	R	I	S	R	I	S	L
F	S	H	K	F	C	Y	F	R	T	S	G	F	P	Y	S	R	I	S	R	I	S	L
F	S	H	K	F	C	Y	F	R	T	S	G	F	P	Y	S	R	I	S	R	I	S	L
F	S	H	K	F	C	Y	F	R	T	S	G	F	P	Y	S	R	I	S	R	I	S	L
F	S	H	K	F	C	Y	F	R	T	S	G	F	P	Y	S	R	I	S	R	I	S	L
F	S	H	K	F	C	Y	F	R	T	S	G	F	P	Y	S	R	I	S	R	I	S	L
F	S	H	K	F	C	Y	F	R	T	S	G	F	P	Y	S	R	I	S	R	I	S	L
F	S	H	K	F	C	Y	F	R	T	S	G	F	P	Y	S	R	I	S	R	I	S	L
F	S	H	K	F	C	Y	F	R	T	S	G	F	P	Y	S	R	I	S	R	I	S	L
F	S	H	K	F	C	Y	F	R	T	S	G	F	P	Y	S	R	I	S	R	I	S	L
F	S	H	K	F	C	Y	F	R	T	S	G	F	P	Y	S	R	I	S	R	I	S	L
F	S	H	K	F	C	Y	F	R	T	S	G	F	P	Y	S	R	I	S	R	I	S	L
F	S	H	K	F	C	Y	F	R	T	S	G	F	P	Y	S	R	I	S	R	I	S	L
F	S	H	K	F	C	Y	F	R	T	S	G	F	P	Y	S	R	I	S	R	I	S	L
F	S	H	K	F	C	Y	F	R	T	S	G	F	P	Y	S	R	I	S	R	I	S	L
F	S	H	K	F	C	Y	F	R	T	S	G	F	P	Y	S	R	I	S	R	I	S	L
F	S	H	K	F	C	Y	F	R	T	S	G	F	P	Y	S	R	I	S	R	I	S	L
F	S	H	K	F	C	Y	F	R	T	S	G	F	P	Y	S	R	I	S	R	I	S	L
F	S	H	K	F	C	Y	F	R	T	S	G	F	P	Y	S	R	I	S	R	I	S	L
F	S	H	K	F	C	Y	F	R	T	S	G	F	P	Y	S	R	I	S	R	I	S	L
F	S	H	K	F	C	Y	F	R	T	S	G	F	P	Y	S	R	I	S	R	I	S	L
F	S	H	K	F	C	Y	F	R	T	S	G	F	P	Y	S	R	I	S	R	I	S	L
F	S	H	K	F	C	Y	F	R	T	S	G	F	P	Y	S	R	I	S	R	I	S	L
F	S	H	K	F	C	Y	F	R	T	S	G	F	P	Y	S	R	I	S	R	I	S	L
F	S	H	K	F	C	Y	F	R	T	S	G	F	P	Y	S	R	I	S	R	I	S	L
F	S	H	K	F	C	Y	F	R	T	S	G	F	P	Y	S	R	I	S	R	I	S	L
F	S	H	K	F	C	Y	F	R	T	S	G	F	P	Y	S	R	I	S	R	I	S	L
F	S	H	K	F	C	Y	F	R	T	S	G	F	P	Y	S	R	I	S	R	I	S	L
F	S	H	K	F	C	Y	F	R	T	S	G	F	P	Y	S	R	I	S	R	I	S	L
F	S	H	K	F	C	Y	F	R	T	S	G	F	P	Y	S	R	I	S	R	I	S	L
F	S	H	K	F	C	Y	F	R	T	S	G	F	P	Y	S	R	I	S	R	I	S	L
F	S	H	K	F	C	Y	F	R	T	S	G	F	P	Y	S	R	I	S	R	I	S	L
F	S	H	K	F	C	Y	F	R	T	S	G	F	P	Y	S	R	I	S	R	I	S	L

175	176	177	181	182	183	184	185	186	187	188	189	190	192	193	194	197	198	199	202	203	204	206
L	I	R	K	Y	I	S	G	T	V	A	V	D	N	D	Y	F	F	F	L	S	L	S
L	N	D	K	S	Q	D	S	L	P	I	T	N	D	C	A	S	I	C	F	F	I	G
L	N	D	K	S	Q	D	S	L	P	I	T	N	D	C	A	S	I	C	F	F	I	G
L	I	R	K	Y	I	S	G	T	V	A	V	D	N	D	Y	F	F	F	L	S	L	S
S	I	R	E	Y	I	S	R	T	V	A	V	D	N	D	Y	F	F	F	L	S	L	T
S	I	R	E	Y	I	S	R	T	V	A	V	D	N	D	Y	F	F	F	L	S	L	S

S	I	R	E	Y	I	S	R	T	V	A	V	D	N	D	Y	F	F	F	L	S	L	S
S	I	R	E	Y	I	S	R	T	V	A	V	D	N	D	Y	F	F	F	L	S	L	S
S	I	R	E	Y	I	S	R	T	V	A	V	D	N	D	Y	F	F	F	L	S	L	S
S	I	R	E	Y	I	S	R	T	V	A	V	D	N	D	Y	F	F	F	L	S	L	S
S	I	R	E	Y	I	S	R	T	V	A	V	D	N	D	Y	F	F	F	L	S	L	S
S	I	R	E	Y	I	S	R	T	V	A	V	D	N	D	Y	F	F	F	L	S	L	S
S	I	R	E	Y	I	S	R	T	V	A	V	D	N	D	Y	F	F	F	L	S	L	S
S	I	R	E	Y	I	S	R	T	V	A	V	D	N	D	Y	F	F	F	L	S	L	S
S	I	R	E	Y	I	S	R	T	V	A	V	D	N	D	Y	F	F	F	L	S	L	S
S	I	R	E	Y	I	S	R	T	V	A	V	D	N	D	Y	F	F	F	L	S	L	S
S	I	R	E	Y	I	S	R	T	V	A	V	D	N	D	Y	F	F	F	L	S	L	S
S	I	R	E	Y	I	S	R	T	V	A	V	D	N	D	Y	F	F	F	L	S	L	S
S	I	R	E	Y	I	S	R	T	V	A	V	D	N	D	Y	F	F	F	L	S	L	S
S	I	R	E	Y	I	S	R	T	V	A	V	D	N	D	Y	F	F	F	L	S	L	S
S	I	R	E	Y	I	S	R	T	V	A	V	D	N	D	Y	F	F	F	L	S	L	S
S	I	R	E	Y	I	S	R	T	V	A	V	D	N	D	Y	F	F	F	L	S	L	S
S	I	R	E	Y	I	S	R	T	V	A	V	D	N	D	Y	F	F	F	L	S	L	S
S	I	R	E	Y	I	S	R	T	V	A	V	D	N	D	Y	F	F	F	L	S	L	S
S	I	R	E	Y	I	S	R	T	V	A	V	D	N	D	Y	F	F	F	L	S	L	S
S	I	R	E	Y	I	S	R	T	V	A	V	D	N	D	Y	F	F	F	L	S	L	S
S	I	R	E	Y	I	S	R	T	V	A	V	D	N	D	Y	F	F	F	L	S	L	S
S	I	R	E	Y	I	S	R	T	V	A	V	D	N	D	Y	F	F	F	L	S	L	S
S	I	R	E	Y	I	S	R	T	V	A	V	D	N	D	Y	F	F	F	L	S	L	S
S	I	R	E	Y	I	S	R	T	V	A	V	D	N	D	Y	F	F	F	L	S	L	S
S	I	R	E	Y	I	S	R	T	V	A	V	D	N	D	Y	F	F	F	L	S	L	S
S	I	R	E	Y	I	S	R	T	V	A	V	D	N	D	Y	F	F	F	L	S	L	S
S	I	R	E	Y	I	S	R	T	V	A	V	D	N	D	Y	F	F	F	L	S	L	S
S	I	R	E	Y	I	S	R	T	V	A	V	D	N	D	Y	F	F	F	L	S	L	S
S	I	R	E	Y	I	S	R	T	V	A	V	D	N	D	Y	F	F	F	L	S	L	S
S	I	R	E	Y	I	S	R	T	V	A	V	D	N	D	Y	F	F	F	L	S	L	S
S	I	R	E	Y	I	S	R	T	V	A	V	D	N	D	Y	F	F	F	L	S	L	S
S	I	R	E	Y	I	S	R	T	V	A	V	D	N	D	Y	F	F	F	L	S	L	S
S	I	R	E	Y	I	S	R	T	V	A	V	D	N	D	Y	F	F	F	L	S	L	S
S	I	R	E	Y	I	S	R	T	V	A	V	D	N	D	Y	F	F	F	L	S	L	S
S	I	R	E	Y	I	S	R	T	V	A	V	D	N	D	Y	F	F	F	L	S	L	S
S	I	R	E	Y	I	S	R	T	V	A	V	D	N	D	Y	F	F	F	L	S	L	S
S	I	R	E	Y	I	S	R	T	V	A	V	D	N	D	Y	F	F	F	L	S	L	S
S	I	R	E	Y	I	S	R	T	V	A	V	D	N	D	Y	F	F	F	L	S	L	S
S	I	R	E	Y	I	S	R	T	V	A	V	D	N	D	Y	F	F	F	L	S	L	S
S	I	R	E	Y	I	S	R	T	V	A	V	D	N	D	Y	F	F	F	L	S	L	S
S	I	R	E	Y	I	S	R	T	V	A	V	D	N	D	Y	F	F	F	L	S	L	S
S	I	R	E	Y	I	S	R	T	V	A	V	D	N	D	Y	F	F	F	L	S	L	S
S	I	R	E	Y	I	S	R	T	V	A	V	D	N	D	Y	F	F	F	L	S	L	S
S	I	R	E	Y	I	S	R	T	V	A	V	D	N	D	Y	F	F	F	L	S	L	S
S	I	R	E	Y	I	S	R	T	V	A	V	D	N	D	Y	F	F	F	L	S	L	S
S	I	R	E	Y	I	S	R	T	V	A	V	D	N	D	Y	F	F	F	L	S	L	S
S	I	R	E	Y	I	S	R	T	V	A	V	D	N	D	Y	F	F	F	L	S	L	S
S	I	R	E	Y	I	S	R	T	V	A	V	D	N	D	Y	F	F	F	L	S	L	S

208	209	210	211	213	215	216	217	220	222	223	227	229	230	231	232	233	234	235	236	238	239	240
C	F	V	Y	C	Y	Y	F	V	K	T	K	K	E	D	I	C	C	N	F	C	I	C
S	N	L	H	Y	R	I	I	H	E	S	E	D	S	N	F	F	L	D	I	Y	N	Y
S	N	L	H	Y	R	I	I	H	E	S	E	D	S	N	F	F	L	D	I	Y	N	Y
C	F	V	Y	C	Y	Y	F	V	K	T	K	K	E	D	I	C	C	N	F	C	I	C
C	F	V	Y	Y	Y	Y	V	V	K	T	K	K	E	D	I	C	H	N	F	C	I	C
C	F	V	Y	Y	Y	Y	F	V	K	T	K	K	E	D	I	C	Y	N	F	C	I	C

C	F	V	Y	Y	Y	Y	F	V	K	T	K	K	E	D	I	C	Y	N	F	C	I	C
C	F	V	Y	Y	Y	Y	F	V	K	T	K	K	E	D	I	C	Y	N	F	C	I	C
C	F	V	Y	Y	Y	Y	F	V	K	T	K	K	E	D	I	C	Y	N	F	C	I	C
C	F	V	Y	Y	Y	Y	F	V	K	T	K	K	E	D	I	C	Y	N	F	C	I	C
C	F	V	Y	Y	Y	Y	F	V	K	T	K	K	E	D	I	C	Y	N	F	C	I	C
C	F	V	Y	Y	Y	Y	F	V	K	T	K	K	E	D	I	C	Y	N	F	C	I	C
C	F	V	Y	Y	Y	Y	F	V	K	T	K	K	E	D	I	C	Y	N	F	C	I	C
C	F	V	Y	Y	Y	Y	F	V	K	T	K	K	E	D	I	C	Y	N	F	C	I	C
C	F	V	Y	Y	Y	Y	F	V	K	T	K	K	E	D	I	C	Y	N	F	C	I	C
C	F	V	Y	Y	Y	Y	F	V	K	T	K	K	E	D	I	C	Y	N	F	C	I	C
C	F	V	Y	Y	Y	Y	F	V	K	T	K	K	E	D	I	C	Y	N	F	C	I	C
C	F	V	Y	Y	Y	Y	F	V	K	T	K	K	E	D	I	C	Y	N	F	C	I	C
C	F	V	Y	Y	Y	Y	F	V	K	T	K	K	E	D	I	C	Y	N	F	C	I	C
C	F	V	Y	Y	Y	Y	F	V	K	T	K	K	E	D	I	C	Y	N	F	C	I	C
C	F	V	Y	Y	Y	Y	F	V	K	T	K	K	E	D	I	C	Y	N	F	C	I	C
C	F	V	Y	Y	Y	Y	F	V	K	T	K	K	E	D	I	C	Y	N	F	C	I	C
C	F	V	Y	Y	Y	Y	F	V	K	T	K	K	E	D	I	C	Y	N	F	C	I	C
C	F	V	Y	Y	Y	Y	F	V	K	T	K	K	E	D	I	C	Y	N	F	C	I	C
C	F	V	Y	Y	Y	Y	F	V	K	T	K	K	E	D	I	C	Y	N	F	C	I	C
C	F	V	Y	Y	Y	Y	F	V	K	T	K	K	E	D	I	C	Y	N	F	C	I	C
C	F	V	Y	Y	Y	Y	F	V	K	T	K	K	E	D	I	C	Y	N	F	C	I	C
C	F	V	Y	Y	Y	Y	F	V	K	T	K	K	E	D	I	C	Y	N	F	C	I	C
C	F	V	Y	Y	Y	Y	F	V	K	T	K	K	E	D	I	C	Y	N	F	C	I	C
C	F	V	Y	Y	Y	Y	F	V	K	T	K	K	E	D	I	C	Y	N	F	C	I	C
C	F	V	Y	Y	Y	Y	F	V	K	T	K	K	E	D	I	C	Y	N	F	C	I	C
C	F	V	Y	Y	Y	Y	F	V	K	T	K	K	E	D	I	C	Y	N	F	C	I	C
C	F	V	Y	Y	Y	Y	F	V	K	T	K	K	E	D	I	C	Y	N	F	C	I	C
C	F	V	Y	Y	Y	Y	F	V	K	T	K	K	E	D	I	C	Y	N	F	C	I	C
C	F	V	Y	Y	Y	Y	F	V	K	T	K	K	E	D	I	C	Y	N	F	C	I	C
C	F	V	Y	Y	Y	Y	F	V	K	T	K	K	E	D	I	C	Y	N	F	C	I	C
C	F	V	Y	Y	Y	Y	F	V	K	T	K	K	E	D	I	C	Y	N	F	C	I	C
C	F	V	Y	Y	Y	Y	F	V	K	T	K	K	E	D	I	C	Y	N	F	C	I	C
C	F	V	Y	Y	Y	Y	F	V	K	T	K	K	E	D	I	C	Y	N	F	C	I	C
C	F	V	Y	Y	Y	Y	F	V	K	T	K	K	E	D	I	C	Y	N	F	C	I	C
C	F	V	Y	Y	Y	Y	F	V	K	T	K	K	E	D	I	C	Y	N	F	C	I	C
C	F	V	Y	Y	Y	Y	F	V	K	T	K	K	E	D	I	C	Y	N	F	C	I	C
C	F	V	Y	Y	Y	Y	F	V	K	T	K	K	E	D	I	C	Y	N	F	C	I	C
C	F	V	Y	Y	Y	Y	F	V	K	T	K	K	E	D	I	C	Y	N	F	C	I	C
C	F	V	Y	Y	Y	Y	F	V	K	T	K	K	E	D	I	C	Y	N	F	C	I	C
C	F	V	Y	Y	Y	Y	F	V	K	T	K	K	E	D	I	C	Y	N	F	C	I	C
C	F	V	Y	Y	Y	Y	F	V	K	T	K	K	E	D	I	C	Y	N	F	C	I	C
C	F	V	Y	Y	Y	Y	F	V	K	T	K	K	E	D	I	C	Y	N	F	C	I	C
C	F	V	Y	Y	Y	Y	F	V	K	T	K	K	E	D	I	C	Y	N	F	C	I	C
C	F	V	Y	Y	Y	Y	F	V	K	T	K	K	E	D	I	C	Y	N	F	C	I	C
C	F	V	Y	Y	Y	Y	F	V	K	T	K	K	E	D	I	C	Y	N	F	C	I	C
C	F	V	Y	Y	Y	Y	F	V	K	T	K	K	E	D	I	C	Y	N	F	C	I	C
C	F	V	Y	Y	Y	Y	F	V	K	T	K	K	E	D	I	C	Y	N	F	C	I	C
C	F	V	Y	Y	Y	Y	F	V	K	T	K	K	E	D	I	C	Y	N	F	C	I	C

241	242	249	250	254	256	257	265	266	267	271	273	275	276	278	279	282	283	287	289	291	293	298
C	D	L	D	H	L	D	V	D	T	A	C	L	P	F	K	W	F	L	G	Q	K	K
S	A	K	V	D	Y	N	A	A	I	V	L	Q	K	L	D	I	Y	V	S	H	N	D
S	A	K	V	D	Y	N	A	A	I	V	L	Q	K	L	D	I	Y	V	S	H	N	D
C	D	L	D	H	L	D	V	D	T	A	C	L	P	F	K	W	F	L	G	Q	K	K
C	D	L	N	H	L	D	V	D	T	A	C	L	P	L	K	W	F	V	G	Q	K	E
C	D	L	D	H	L	D	V	D	T	A	C	P	P	F	K	W	F	V	G	Q	K	E

C	D	L	D	H	L	D	V	D	T	A	C	P	P	F	K	W	F	V	G	Q	K	E
C	D	L	D	H	L	D	V	D	T	A	C	P	P	F	K	W	F	V	G	Q	K	E
C	D	L	D	H	L	D	V	D	T	A	C	P	P	F	K	W	F	V	G	Q	K	E
C	D	L	D	H	L	D	V	D	T	A	C	P	P	F	K	W	F	V	G	Q	K	E
C	D	L	D	H	L	D	V	D	T	A	C	P	P	F	K	W	F	V	G	Q	K	E
C	D	L	D	H	L	D	V	D	T	A	C	P	P	F	K	W	F	V	G	Q	K	E
C	D	L	D	H	L	D	V	D	T	A	C	P	P	F	K	W	F	V	G	Q	K	E
C	D	L	D	H	L	D	V	D	T	A	C	P	P	F	K	W	F	V	G	Q	K	E
C	D	L	D	H	L	D	V	D	T	A	C	P	P	F	K	W	F	V	G	Q	K	E
C	D	L	D	H	L	D	V	D	T	A	C	P	P	F	K	W	F	V	G	Q	K	E
C	D	L	D	H	L	D	V	D	T	A	C	P	P	F	K	W	F	V	G	Q	K	E
C	D	L	D	H	L	D	V	D	T	A	C	P	P	F	K	W	F	V	G	Q	K	E
C	D	L	D	H	L	D	V	D	T	A	C	P	P	F	K	W	F	V	G	Q	K	E
C	D	L	D	H	L	D	V	D	T	A	C	P	P	F	K	W	F	V	G	Q	K	E
C	D	L	D	H	L	D	V	D	T	A	C	P	P	F	K	W	F	V	G	Q	K	E
C	D	L	D	H	L	D	V	D	T	A	C	P	P	F	K	W	F	V	G	Q	K	E
C	D	L	D	H	L	D	V	D	T	A	C	P	P	F	K	W	F	V	G	Q	K	E
C	D	L	D	H	L	D	V	D	T	A	C	P	P	F	K	W	F	V	G	Q	K	E
C	D	L	D	H	L	D	V	D	T	A	C	P	P	F	K	W	F	V	G	Q	K	E
C	D	L	D	H	L	D	V	D	T	A	C	P	P	F	K	W	F	V	G	Q	K	E
C	D	L	D	H	L	D	V	D	T	A	C	P	P	F	K	W	F	V	G	Q	K	E
C	D	L	D	H	L	D	V	D	T	A	C	P	P	F	K	W	F	V	G	Q	K	E
C	D	L	D	H	L	D	V	D	T	A	C	P	P	F	K	W	F	V	G	Q	K	E
C	D	L	D	H	L	D	V	D	T	A	C	P	P	F	K	W	F	V	G	Q	K	E
C	D	L	D	H	L	D	V	D	T	A	C	P	P	F	K	W	F	V	G	Q	K	E
C	D	L	D	H	L	D	V	D	T	A	C	P	P	F	K	W	F	V	G	Q	K	E
C	D	L	D	H	L	D	V	D	T	A	C	P	P	F	K	W	F	V	G	Q	K	E
C	D	L	D	H	L	D	V	D	T	A	C	P	P	F	K	W	F	V	G	Q	K	E
C	D	L	D	H	L	D	V	D	T	A	C	P	P	F	K	W	F	V	G	Q	K	E
C	D	L	D	H	L	D	V	D	T	A	C	P	P	F	K	W	F	V	G	Q	K	E
C	D	L	D	H	L	D	V	D	T	A	C	P	P	F	K	W	F	V	G	Q	K	E
C	D	L	D	H	L	D	V	D	T	A	C	P	P	F	K	W	F	V	G	Q	K	E
C	D	L	D	H	L	D	V	D	T	A	C	P	P	F	K	W	F	V	G	Q	K	E
C	D	L	D	H	L	D	V	D	T	A	C	P	P	F	K	W	F	V	G	Q	K	E
C	D	L	D	H	L	D	V	D	T	A	C	P	P	F	K	W	F	V	G	Q	K	E
C	D	L	D	H	L	D	V	D	T	A	C	P	P	F	K	W	F	V	G	Q	K	E
C	D	L	D	H	L	D	V	D	T	A	C	P	P	F	K	W	F	V	G	Q	K	E
C	D	L	D	H	L	D	V	D	T	A	C	P	P	F	K	W	F	V	G	Q	K	E
C	D	L	D	H	L	D	V	D	T	A	C	P	P	F	K	W	F	V	G	Q	K	E
C	D	L	D	H	L	D	V	D	T	A	C	P	P	F	K	W	F	V	G	Q	K	E
C	D	L	D	H	L	D	V	D	T	A	C	P	P	F	K	W	F	V	G	Q	K	E
C	D	L	D	H	L	D	V	D	T	A	C	P	P	F	K	W	F	V	G	Q	K	E
C	D	L	D	H	L	D	V	D	T	A	C	P	P	F	K	W	F	V	G	Q	K	E
C	D	L	D	H	L	D	V	D	T	A	C	P	P	F	K	W	F	V	G	Q	K	E
C	D	L	D	H	L	D	V	D	T	A	C	P	P	F	K	W	F	V	G	Q	K	E
C	D	L	D	H	L	D	V	D	T	A	C	P	P	F	K	W	F	V	G	Q	K	E
C	D	L	D	H	L	D	V	D	T	A	C	P	P	F	K	W	F	V	G	Q	K	E
C	D	L	D	H	L	D	V	D	T	A	C	P	P	F	K	W	F	V	G	Q	K	E

299	301	302	307	311	312	316	318	320	321	324	327	328	336	338	339	341	343	344	345	346	350	352
L	E	V	T	V	L	L	S	I	V	I	F	T	S	K	N	Q	S	T	I	S	I	A
S	A	K	A	L	M	I	N	V	I	V	F	I	L	R	K	R	F	R	V	A	L	S
S	A	K	A	L	M	I	N	V	I	V	F	I	L	R	K	R	F	R	V	A	L	S
L	E	V	T	V	L	L	S	I	V	I	F	T	S	K	N	Q	S	T	I	S	I	A
L	E	I	T	V	L	L	S	I	V	I	L	I	S	K	N	Q	P	T	I	S	I	A
L	E	I	T	I	L	L	S	I	V	I	F	T	S	K	N	Q	P	A	I	G	I	A

L	E	I	T	I	L	L	S	I	V	I	F	T	S	K	N	Q	P	A	I	G	I	A
L	E	I	T	I	L	L	S	I	V	I	F	T	S	K	N	Q	P	A	I	G	I	A
L	E	I	T	I	L	L	S	I	V	I	F	T	S	K	N	Q	P	A	I	G	I	A
L	E	I	T	I	L	L	S	I	V	I	F	T	S	K	N	Q	P	A	I	G	I	A
L	E	I	T	I	L	L	S	I	V	I	F	T	S	K	N	Q	P	A	I	G	I	A
L	E	I	T	I	L	L	S	I	V	I	F	T	S	K	N	Q	P	A	I	G	I	A
L	E	I	T	I	L	L	S	I	V	I	F	T	S	K	N	Q	P	A	I	G	I	A
L	E	I	T	I	L	L	S	I	V	I	F	T	S	K	N	Q	P	A	I	G	I	A
L	E	I	T	I	L	L	S	I	V	I	F	T	S	K	N	Q	P	A	I	G	I	A
L	E	I	T	I	L	L	S	I	V	I	F	T	S	K	N	Q	P	A	I	G	I	A
L	E	I	T	I	L	L	S	I	V	I	F	T	S	K	N	Q	P	A	I	G	I	A
L	E	I	T	I	L	L	S	I	V	I	F	T	S	K	N	Q	P	A	I	G	I	A
L	E	I	T	I	L	L	S	I	V	I	F	T	S	K	N	Q	P	A	I	G	I	A
L	E	I	T	I	L	L	S	I	V	I	F	T	S	K	N	Q	P	A	I	G	I	A
L	E	I	T	I	L	L	S	I	V	I	F	T	S	K	N	Q	P	A	I	G	I	A
L	E	I	T	I	L	L	S	I	V	I	F	T	S	K	N	Q	P	A	I	G	I	A
L	E	I	T	I	L	L	S	I	V	I	F	T	S	K	N	Q	P	A	I	G	I	A
L	E	I	T	I	L	L	S	I	V	I	F	T	S	K	N	Q	P	A	I	G	I	A
L	E	I	T	I	L	L	S	I	V	I	F	T	S	K	N	Q	P	A	I	G	I	A
L	E	I	T	I	L	L	S	I	V	I	F	T	S	K	N	Q	P	A	I	G	I	A
L	E	I	T	I	L	L	S	I	V	I	F	T	S	K	N	Q	P	A	I	G	I	A
L	E	I	T	I	L	L	S	I	V	I	F	T	S	K	N	Q	P	A	I	G	I	A
L	E	I	T	I	L	L	S	I	V	I	F	T	S	K	N	Q	P	A	I	G	I	A
L	E	I	T	I	L	L	S	I	V	I	F	T	S	K	N	Q	P	A	I	G	I	A
L	E	I	T	I	L	L	S	I	V	I	F	T	S	K	N	Q	P	A	I	G	I	A
L	E	I	T	I	L	L	S	I	V	I	F	T	S	K	N	Q	P	A	I	G	I	A
L	E	I	T	I	L	L	S	I	V	I	F	T	S	K	N	Q	P	A	I	G	I	A
L	E	I	T	I	L	L	S	I	V	I	F	T	S	K	N	Q	P	A	I	G	I	A
L	E	I	T	I	L	L	S	I	V	I	F	T	S	K	N	Q	P	A	I	G	I	A
L	E	I	T	I	L	L	S	I	V	I	F	T	S	K	N	Q	P	A	I	G	I	A
L	E	I	T	I	L	L	S	I	V	I	F	T	S	K	N	Q	P	A	I	G	I	A
L	E	I	T	I	L	L	S	I	V	I	F	T	S	K	N	Q	P	A	I	G	I	A
L	E	I	T	I	L	L	S	I	V	I	F	T	S	K	N	Q	P	A	I	G	I	A
L	E	I	T	I	L	L	S	I	V	I	F	T	S	K	N	Q	P	A	I	G	I	A
L	E	I	T	I	L	L	S	I	V	I	F	T	S	K	N	Q	P	A	I	G	I	A
L	E	I	T	I	L	L	S	I	V	I	F	T	S	K	N	Q	P	A	I	G	I	A
L	E	I	T	I	L	L	S	I	V	I	F	T	S	K	N	Q	P	A	I	G	I	A
L	E	I	T	I	L	L	S	I	V	I	F	T	S	K	N	Q	P	A	I	G	I	A
L	E	I	T	I	L	L	S	I	V	I	F	T	S	K	N	Q	P	A	I	G	I	A
L	E	I	T	I	L	L	S	I	V	I	F	T	S	K	N	Q	P	A	I	G	I	A
L	E	I	T	I	L	L	S	I	V	I	F	T	S	K	N	Q	P	A	I	G	I	A
L	E	I	T	I	L	L	S	I	V	I	F	T	S	K	N	Q	P	A	I	G	I	A
L	E	I	T	I	L	L	S	I	V	I	F	T	S	K	N	Q	P	A	I	G	I	A
L	E	I	T	I	L	L	S	I	V	I	F	T	S	K	N	Q	P	A	I	G	I	A
L	E	I	T	I	L	L	S	I	V	I	F	T	S	K	N	Q	P	A	I	G	I	A
L	E	I	T	I	L	L	S	I	V	I	F	T	S	K	N	Q	P	A	I	G	I	A
L	E	I	T	I	L	L	S	I	V	I	F	T	S	K	N	Q	P	A	I	G	I	A

359	375	376	377	379	382	373	386	387	388
I	I	V	L	T	L	K	R	E	R
V	L	I	F	I	E	R	Q	V	G
V	L	I	F	I	E	R	Q	V	G
I	I	V	L	T	L	K	R	E	R
I	I	V	L	T	L	K	R	E	R
I	I	V	L	T	L	K	R	E	R

4	8	11	12	13	18	19	24	25	26	39	44
R	N	K	V	Y	I	K	Q	C	G	A	D
S	R	Q	I	F	V	R	V	A	K	S	S
S	R	Q	I	F	V	R	V	A	K	S	S
R	N	K	V	Y	I	K	Q	C	G	A	D
R	N	K	V	Y	I	K	Q	C	G	A	D
R	N	K	V	Y	I	K	Q	C	S	A	D

I	I	V	L	T	L	K	R	E	R
I	I	V	L	T	L	K	R	E	R
I	I	V	L	T	L	K	R	E	R
I	I	V	L	T	L	K	R	E	R
I	I	V	L	T	L	K	R	E	R
I	I	V	L	T	L	K	R	E	R
I	I	V	L	T	L	K	R	E	R
I	I	V	L	T	L	K	R	E	R
I	I	V	L	T	L	K	R	E	R
I	I	V	L	T	L	K	R	E	R
I	I	V	L	T	L	K	R	E	R
I	I	V	L	T	L	K	R	E	R
I	I	V	L	T	L	K	R	E	R
I	I	V	L	T	L	K	R	E	R
I	I	V	L	T	L	K	R	E	R
I	I	V	L	T	L	K	R	E	R
I	I	V	L	T	L	K	R	E	R
I	I	V	L	T	L	K	R	E	R
I	I	V	L	T	L	K	R	E	R
I	I	V	L	T	L	K	R	E	R
I	I	V	L	T	L	K	R	E	R
I	I	V	L	T	L	K	R	E	R
I	I	V	L	T	L	K	R	E	R
I	I	V	L	T	L	K	R	E	R
I	I	V	L	T	L	K	R	E	R
I	I	V	L	T	L	K	R	E	R
I	I	V	L	T	L	K	R	E	R
I	I	V	L	T	L	K	R	E	R
I	I	V	L	T	L	K	R	E	R
I	I	V	L	T	L	K	R	E	R
I	I	V	L	T	L	K	R	E	R
I	I	V	L	T	L	K	R	E	R
I	I	V	L	T	L	K	R	E	R
I	I	V	L	T	L	K	R	E	R
I	I	V	L	T	L	K	R	E	R
I	I	V	L	T	L	K	R	E	R
I	I	V	L	T	L	K	R	E	R
I	I	V	L	T	L	K	R	E	R
I	I	V	L	T	L	K	R	E	R
I	I	V	L	T	L	K	R	E	R
I	I	V	L	T	L	K	R	E	R
I	I	V	L	T	L	K	R	E	R
I	I	V	L	T	L	K	R	E	R
I	I	V	L	T	L	K	R	E	R
I	I	V	L	T	L	K	R	E	R
I	I	V	L	T	L	K	R	E	R
I	I	V	L	T	L	K	R	E	R
I	I	V	L	T	L	K	R	E	R

R	N	K	V	Y	I	K	Q	C	S	A	D
R	N	K	V	Y	I	K	Q	C	S	A	D
R	N	K	V	Y	I	K	Q	C	S	A	D
R	N	K	V	Y	I	K	Q	C	S	A	D
R	N	K	V	Y	I	K	Q	C	S	A	D
R	N	K	V	Y	I	K	Q	C	S	A	D
R	N	K	V	Y	I	K	Q	C	S	A	D
R	N	K	V	Y	I	K	Q	C	S	A	D
R	N	K	V	Y	I	K	Q	C	S	A	D
R	N	K	V	Y	I	K	Q	C	S	A	D
R	N	K	V	Y	I	K	Q	C	S	A	D
R	N	K	V	Y	I	K	Q	C	S	A	D
R	N	K	V	Y	I	K	Q	C	S	A	D
R	N	K	V	Y	I	K	Q	C	S	A	D
R	N	K	V	Y	I	K	Q	C	S	A	D
R	N	K	V	Y	I	K	Q	C	S	A	D
R	N	K	V	Y	I	K	Q	C	S	A	D
R	N	K	V	Y	I	K	Q	C	S	A	D
R	N	K	V	Y	I	K	Q	C	S	A	D
R	N	K	V	Y	I	K	Q	C	S	A	D
R	N	K	V	Y	I	K	Q	C	S	A	D
R	N	K	V	Y	I	K	Q	C	S	A	D
R	N	K	V	Y	I	K	Q	C	S	A	D
R	N	K	V	Y	I	K	Q	C	S	A	D
R	N	K	V	Y	I	K	Q	C	S	A	D
R	N	K	V	Y	I	K	Q	C	S	A	D
R	N	K	V	Y	I	K	Q	C	S	A	D
R	N	K	V	Y	I	K	Q	C	S	A	D
R	N	K	V	Y	I	K	Q	C	S	A	D
R	N	K	V	Y	I	K	Q	C	S	A	D
R	N	K	V	Y	I	K	Q	C	S	A	D
R	N	K	V	Y	I	K	Q	C	S	A	D
R	N	K	V	Y	I	K	Q	C	S	A	D
R	N	K	V	Y	I	K	Q	C	S	A	D
R	N	K	V	Y	I	K	Q	C	S	A	D
R	N	K	V	Y	I	K	Q	C	S	A	D
R	N	K	V	Y	I	K	Q	C	S	A	D
R	N	K	V	Y	I	K	Q	C	S	A	D
R	N	K	V	Y	I	K	Q	C	S	A	D
R	N	K	V	Y	I	K	Q	C	S	A	D
R	N	K	V	Y	I	K	Q	C	S	A	D
R	N	K	V	Y	I	K	Q	C	S	A	D
R	N	K	V	Y	I	K	Q	C	S	A	D
R	N	K	V	Y	I	K	Q	C	S	A	D
R	N	K	V	Y	I	K	Q	C	S	A	D
R	N	K	V	Y	I	K	Q	C	S	A	D
R	N	K	V	Y	I	K	Q	C	S	A	D
R	N	K	V	Y	I	K	Q	C	S	A	D

wcwT																						
55	59	64	70	80	83	85	91	92	99	101	106	116	117	119	123	131	133	136	146	147	149	154
Y	K	K	L	N	L	D	E	S	L	E	V	A	I	E	I	I	R	A	K	P	R	E
F	R	R	I	D	T	T	N	K	I	D	I	S	R	G	V	V	E	P	R	V	K	K
F	R	R	I	D	T	T	N	K	I	D	I	S	R	G	V	V	E	P	R	V	K	K
Y	K	K	L	N	L	D	E	S	L	E	V	A	I	E	I	I	R	A	K	P	R	E
Y	K	K	L	N	L	D	E	S	L	E	V	A	I	E	I	V	R	A	K	P	R	E
Y	K	K	L	N	L	D	E	S	L	E	V	A	I	E	I	A	R	A	K	P	R	E

Y	K	K	L	N	L	D	E	S	L	E	V	A	I	E	I	A	R	A	K	P	R	E
Y	K	K	L	N	L	D	E	S	L	E	V	A	I	E	I	A	R	A	K	P	R	E
Y	K	K	L	N	L	D	E	S	L	E	V	A	I	E	I	A	R	A	K	P	R	E
Y	K	K	L	N	L	D	E	S	L	E	V	A	I	E	I	A	R	A	K	P	R	E
Y	K	K	L	N	L	D	E	S	L	E	V	A	I	E	I	A	R	A	K	P	R	E
Y	K	K	L	N	L	D	E	S	L	E	V	A	I	E	I	A	R	A	K	P	R	E
Y	K	K	L	N	L	D	E	S	L	E	V	A	I	E	I	A	R	A	K	P	R	E
Y	K	K	L	N	L	D	E	S	L	E	V	A	I	E	I	A	R	A	K	P	R	E
Y	K	K	L	N	L	D	E	S	L	E	V	A	I	E	I	A	R	A	K	P	R	E
Y	K	K	L	N	L	D	E	S	L	E	V	A	I	E	I	A	R	A	K	P	R	E
Y	K	K	L	N	L	D	E	S	L	E	V	A	I	E	I	A	R	A	K	P	R	E
Y	K	K	L	N	L	D	E	S	L	E	V	A	I	E	I	A	R	A	K	P	R	E
Y	K	K	L	N	L	D	E	S	L	E	V	A	I	E	I	A	R	A	K	P	R	E
Y	K	K	L	N	L	D	E	S	L	E	V	A	I	E	I	A	R	A	K	P	R	E
Y	K	K	L	N	L	D	E	S	L	E	V	A	I	E	I	A	R	A	K	P	R	E
Y	K	K	L	N	L	D	E	S	L	E	V	A	I	E	I	A	R	A	K	P	R	E
Y	K	K	L	N	L	D	E	S	L	E	V	A	I	E	I	A	R	A	K	P	R	E
Y	K	K	L	N	L	D	E	S	L	E	V	A	I	E	I	A	R	A	K	P	R	E
Y	K	K	L	N	L	D	E	S	L	E	V	A	I	E	I	A	R	A	K	P	R	E
Y	K	K	L	N	L	D	E	S	L	E	V	A	I	E	I	A	R	A	K	P	R	E
Y	K	K	L	N	L	D	E	S	L	E	V	A	I	E	I	A	R	A	K	P	R	E
Y	K	K	L	N	L	D	E	S	L	E	V	A	I	E	I	A	R	A	K	P	R	E
Y	K	K	L	N	L	D	E	S	L	E	V	A	I	E	I	A	R	A	K	P	R	E
Y	K	K	L	N	L	D	E	S	L	E	V	A	I	E	I	A	R	A	K	P	R	E
Y	K	K	L	N	L	D	E	S	L	E	V	A	I	E	I	A	R	A	K	P	R	E
Y	K	K	L	N	L	D	E	S	L	E	V	A	I	E	I	A	R	A	K	P	R	E
Y	K	K	L	N	L	D	E	S	L	E	V	A	I	E	I	A	R	A	K	P	R	E
Y	K	K	L	N	L	D	E	S	L	E	V	A	I	E	I	A	R	A	K	P	R	E
Y	K	K	L	N	L	D	E	S	L	E	V	A	I	E	I	A	R	A	K	P	R	E
Y	K	K	L	N	L	D	E	S	L	E	V	A	I	E	I	A	R	A	K	P	R	E
Y	K	K	L	N	L	D	E	S	L	E	V	A	I	E	I	A	R	A	K	P	R	E
Y	K	K	L	N	L	D	E	S	L	E	V	A	I	E	I	A	R	A	K	P	R	E
Y	K	K	L	N	L	D	E	S	L	E	V	A	I	E	I	A	R	A	K	P	R	E
Y	K	K	L	N	L	D	E	S	L	E	V	A	I	E	I	A	R	A	K	P	R	E
Y	K	K	L	N	L	D	E	S	L	E	V	A	I	E	I	A	R	A	K	P	R	E
Y	K	K	L	N	L	D	E	S	L	E	V	A	I	E	I	A	R	A	K	P	R	E
Y	K	K	L	N	L	D	E	S	L	E	V	A	I	E	I	A	R	A	K	P	R	E
Y	K	K	L	N	L	D	E	S	L	E	V	A	I	E	I	A	R	A	K	P	R	E
Y	K	K	L	N	L	D	E	S	L	E	V	A	I	E	I	A	R	A	K	P	R	E
Y	K	K	L	N	L	D	E	S	L	E	V	A	I	E	I	A	R	A	K	P	R	E
Y	K	K	L	N	L	D	E	S	L	E	V	A	I	E	I	A	R	A	K	P	R	E
Y	K	K	L	N	L	D	E	S	L	E	V	A	I	E	I	A	R	A	K	P	R	E
Y	K	K	L	N	L	D	E	S	L	E	V	A	I	E	I	A	R	A	K	P	R	E
Y	K	K	L	N	L	D	E	S	L	E	V	A	I	E	I	A	R	A	K	P	R	E
Y	K	K	L	N	L	D	E	S	L	E	V	A	I	E	I	A	R	A	K	P	R	E
Y	K	K	L	N	L	D	E	S	L	E	V	A	I	E	I	A	R	A	K	P	R	E
Y	K	K	L	N	L	D	E	S	L	E	V	A	I	E	I	A	R	A	K	P	R	E
Y	K	K	L	N	L	D	E	S	L	E	V	A	I	E	I	A	R	A	K	P	R	E

157	158	162	163	164	166
N	Q	Q	K	K	H
E	E	L	E	Q	Y
E	E	L	E	Q	Y
N	Q	Q	K	K	H
N	R	Q	K	K	H
N	R	Q	K	K	H

4	5	9	12	13	14	15	17	18	19	20	21	24	26	27	28
V	S	K	I	T	H	T	K	I	I	N	E	M	E	L	F
I	R	R	F	R	Y	A	N	V	L	Y	Q	I	K	I	L
I	R	R	F	R	Y	A	N	V	L	Y	Q	I	K	I	L
V	S	K	I	T	H	T	K	I	I	N	E	M	E	L	F
V	S	K	I	T	H	T	K	I	I	N	E	M	E	L	F
V	S	K	I	T	H	T	K	I	I	N	E	M	E	L	F

N	R	Q	K	K	H	V	S	K	I	T	H	T	K	I	I	N	E	M	E	L	F
N	R	Q	K	K	H	V	S	K	I	T	H	T	K	I	I	N	E	M	E	L	F
N	R	Q	K	K	H	V	S	K	I	T	H	T	K	I	I	N	E	M	E	L	F
N	R	Q	K	K	H	V	S	K	I	T	H	T	K	I	I	N	E	M	E	L	F
N	R	Q	K	K	H	V	S	K	I	T	H	T	K	I	I	N	E	M	E	L	F
N	R	Q	K	K	H	V	S	K	I	T	H	T	K	I	I	N	E	M	E	L	F
N	R	Q	K	K	H	V	S	K	I	T	H	T	K	I	I	N	E	M	E	L	F
N	R	Q	K	K	H	V	S	K	I	T	H	T	K	I	I	N	E	M	E	L	F
N	R	Q	K	K	H	V	S	K	I	T	H	T	K	I	I	N	E	M	E	L	F
N	R	Q	K	K	H	V	S	K	I	T	H	T	K	I	I	N	E	M	E	L	F
N	R	Q	K	K	H	V	S	K	I	T	H	T	K	I	I	N	E	M	E	L	F
N	R	Q	K	K	H	V	S	K	I	T	H	T	K	I	I	N	E	M	E	L	F
N	R	Q	K	K	H	V	S	K	I	T	H	T	K	I	I	N	E	M	E	L	F
N	R	Q	K	K	H	V	S	K	I	T	H	T	K	I	I	N	E	M	E	L	F
N	R	Q	K	K	H	V	S	K	I	T	H	T	K	I	I	N	E	M	E	L	F
N	R	Q	K	K	H	V	S	K	I	T	H	T	K	I	I	N	E	M	E	L	F
N	R	Q	K	K	H	V	S	K	I	T	H	T	K	I	I	N	E	M	E	L	F
N	R	Q	K	K	H	V	S	K	I	T	H	T	K	I	I	N	E	M	E	L	F
N	R	Q	K	K	H	V	S	K	I	T	H	T	K	I	I	N	E	M	E	L	F
N	R	Q	K	K	H	V	S	K	I	T	H	T	K	I	I	N	E	M	E	L	F
N	R	Q	K	K	H	V	S	K	I	T	H	T	K	I	I	N	E	M	E	L	F
N	R	Q	K	K	H	V	S	K	I	T	H	T	K	I	I	N	E	M	E	L	F
N	R	Q	K	K	H	V	S	K	I	T	H	T	K	I	I	N	E	M	E	L	F
N	R	Q	K	K	H	V	S	K	I	T	H	T	K	I	I	N	E	M	E	L	F
N	R	Q	K	K	H	V	S	K	I	T	H	T	K	I	I	N	E	M	E	L	F
N	R	Q	K	K	H	V	S	K	I	T	H	T	K	I	I	N	E	M	E	L	F
N	R	Q	K	K	H	V	S	K	I	T	H	T	K	I	I	N	E	M	E	L	F
N	R	Q	K	K	H	V	S	K	I	T	H	T	K	I	I	N	E	M	E	L	F
N	R	Q	K	K	H	V	S	K	I	T	H	T	K	I	I	N	E	M	E	L	F
N	R	Q	K	K	H	V	S	K	I	T	H	T	K	I	I	N	E	M	E	L	F
N	R	Q	K	K	H	V	S	K	I	T	H	T	K	I	I	N	E	M	E	L	F
N	R	Q	K	K	H	V	S	K	I	T	H	T	K	I	I	N	E	M	E	L	F
N	R	Q	K	K	H	V	S	K	I	T	H	T	K	I	I	N	E	M	E	L	F
N	R	Q	K	K	H	V	S	K	I	T	H	T	K	I	I	N	E	M	E	L	F
N	R	Q	K	K	H	V	S	K	I	T	H	T	K	I	I	N	E	M	E	L	F
N	R	Q	K	K	H	V	S	K	I	T	H	T	K	I	I	N	E	M	E	L	F
N	R	Q	K	K	H	V	S	K	I	T	H	T	K	I	I	N	E	M	E	L	F
N	R	Q	K	K	H	V	S	K	I	T	H	T	K	I	I	N	E	M	E	L	F
N	R	Q	K	K	H	V	S	K	I	T	H	T	K	I	I	N	E	M	E	L	F
N	R	Q	K	K	H	V	S	K	I	T	H	T	K	I	I	N	E	M	E	L	F
N	R	Q	K	K	H	V	S	K	I	T	H	T	K	I	I	N	E	M	E	L	F
N	R	Q	K	K	H	V	S	K	I	T	H	T	K	I	I	N	E	M	E	L	F
N	R	Q	K	K	H	V	S	K	I	T	H	T	K	I	I	N	E	M	E	L	F
N	R	Q	K	K	H	V	S	K	I	T	H	T	K	I	I	N	E	M	E	L	F
N	R	Q	K	K	H	V	S	K	I	T	H	T	K	I	I	N	E	M	E	L	F
N	R	Q	K	K	H	V	S	K	I	T	H	T	K	I	I	N	E	M	E	L	F
N	R	Q	K	K	H	V	S	K	I	T	H	T	K	I	I	N	E	M	E	L	F
N	R	Q	K	K	H	V	S	K	I	T	H	T	K	I	I	N	E	M	E	L	F

wcwU																						
31	33	34	38	40	42	43	44	54	66	68	76	81	82	84	85	86	97	106	107	114	119	138
L	S	I	Q	E	R	N	S	E	N	E	V	D	K	V	K	E	R	V	M	T	M	P
I	I	R	F	K	Q	N	I	D	R	N	I	N	R	V	R	T	K	L	L	L	I	G
I	I	R	F	K	Q	N	I	D	R	N	I	N	R	V	R	T	K	L	L	L	I	P
L	S	I	Q	E	R	N	S	E	N	E	V	D	K	V	K	E	R	V	M	T	M	P
L	S	I	Q	E	R	D	S	E	N	E	V	D	K	A	K	E	R	V	M	T	M	G
L	S	I	Q	E	R	D	S	E	N	E	V	D	K	S	K	E	R	V	M	T	M	P

L	S	I	Q	E	R	D	S	E	N	E	V	D	K	S	K	E	R	V	M	T	M	P
L	S	I	Q	E	R	D	S	E	N	E	V	D	K	S	K	E	R	V	M	T	M	P
L	S	I	Q	E	R	D	S	E	N	E	V	D	K	S	K	E	R	V	M	T	M	P
L	S	I	Q	E	R	D	S	E	N	E	V	D	K	S	K	E	R	V	M	T	M	P
L	S	I	Q	E	R	D	S	E	N	E	V	D	K	S	K	E	R	V	M	T	M	P
L	S	I	Q	E	R	D	S	E	N	E	V	D	K	S	K	E	R	V	M	T	M	P
L	S	I	Q	E	R	D	S	E	N	E	V	D	K	S	K	E	R	V	M	T	M	P
L	S	I	Q	E	R	D	S	E	N	E	V	D	K	S	K	E	R	V	M	T	M	P
L	S	I	Q	E	R	D	S	E	N	E	V	D	K	S	K	E	R	V	M	T	M	P
L	S	I	Q	E	R	D	S	E	N	E	V	D	K	S	K	E	R	V	M	T	M	P
L	S	I	Q	E	R	D	S	E	N	E	V	D	K	S	K	E	R	V	M	T	M	P
L	S	I	Q	E	R	D	S	E	N	E	V	D	K	S	K	E	R	V	M	T	M	P
L	S	I	Q	E	R	D	S	E	N	E	V	D	K	S	K	E	R	V	M	T	M	P
L	S	I	Q	E	R	D	S	E	N	E	V	D	K	S	K	E	R	V	M	T	M	P
L	S	I	Q	E	R	D	S	E	N	E	V	D	K	S	K	E	R	V	M	T	M	P
L	S	I	Q	E	R	D	S	E	N	E	V	D	K	S	K	E	R	V	M	T	M	P
L	S	I	Q	E	R	D	S	E	N	E	V	D	K	S	K	E	R	V	M	T	M	P
L	S	I	Q	E	R	D	S	E	N	E	V	D	K	S	K	E	R	V	M	T	M	P
L	S	I	Q	E	R	D	S	E	N	E	V	D	K	S	K	E	R	V	M	T	M	P
L	S	I	Q	E	R	D	S	E	N	E	V	D	K	S	K	E	R	V	M	T	M	P
L	S	I	Q	E	R	D	S	E	N	E	V	D	K	S	K	E	R	V	M	T	M	P
L	S	I	Q	E	R	D	S	E	N	E	V	D	K	S	K	E	R	V	M	T	M	P
L	S	I	Q	E	R	D	S	E	N	E	V	D	K	S	K	E	R	V	M	T	M	P
L	S	I	Q	E	R	D	S	E	N	E	V	D	K	S	K	E	R	V	M	T	M	P
L	S	I	Q	E	R	D	S	E	N	E	V	D	K	S	K	E	R	V	M	T	M	P
L	S	I	Q	E	R	D	S	E	N	E	V	D	K	S	K	E	R	V	M	T	M	P
L	S	I	Q	E	R	D	S	E	N	E	V	D	K	S	K	E	R	V	M	T	M	P
L	S	I	Q	E	R	D	S	E	N	E	V	D	K	S	K	E	R	V	M	T	M	P
L	S	I	Q	E	R	D	S	E	N	E	V	D	K	S	K	E	R	V	M	T	M	P
L	S	I	Q	E	R	D	S	E	N	E	V	D	K	S	K	E	R	V	M	T	M	P
L	S	I	Q	E	R	D	S	E	N	E	V	D	K	S	K	E	R	V	M	T	M	P
L	S	I	Q	E	R	D	S	E	N	E	V	D	K	S	K	E	R	V	M	T	M	P
L	S	I	Q	E	R	D	S	E	N	E	V	D	K	S	K	E	R	V	M	T	M	P
L	S	I	Q	E	R	D	S	E	N	E	V	D	K	S	K	E	R	V	M	T	M	P
L	S	I	Q	E	R	D	S	E	N	E	V	D	K	S	K	E	R	V	M	T	M	P
L	S	I	Q	E	R	D	S	E	N	E	V	D	K	S	K	E	R	V	M	T	M	P
L	S	I	Q	E	R	D	S	E	N	E	V	D	K	S	K	E	R	V	M	T	M	P
L	S	I	Q	E	R	D	S	E	N	E	V	D	K	S	K	E	R	V	M	T	M	P
L	S	I	Q	E	R	D	S	E	N	E	V	D	K	S	K	E	R	V	M	T	M	P
L	S	I	Q	E	R	D	S	E	N	E	V	D	K	S	K	E	R	V	M	T	M	P
L	S	I	Q	E	R	D	S	E	N	E	V	D	K	S	K	E	R	V	M	T	M	P
L	S	I	Q	E	R	D	S	E	N	E	V	D	K	S	K	E	R	V	M	T	M	P
L	S	I	Q	E	R	D	S	E	N	E	V	D	K	S	K	E	R	V	M	T	M	P
L	S	I	Q	E	R	D	S	E	N	E	V	D	K	S	K	E	R	V	M	T	M	P
L	S	I	Q	E	R	D	S	E	N	E	V	D	K	S	K	E	R	V	M	T	M	P
L	S	I	Q	E	R	D	S	E	N	E	V	D	K	S	K	E	R	V	M	T	M	P
L	S	I	Q	E	R	D	S	E	N	E	V	D	K	S	K	E	R	V	M	T	M	P
L	S	I	Q	E	R	D	S	E	N	E	V	D	K	S	K	E	R	V	M	T	M	P

141	150	193	194	201	218	235	242	245	253	261	282	284	288	290	332	347	348	350	351
S	N	P	E	N	Y	E	N	T	A	M	D	N	T	L	L	L	S	N	N
N	D	P	K	D	D	G	H	T	V	M	D	K	T	V	L	M	F	N	D
N	N	P	E	D	Y	E	N	I	V	M	D	N	T	L	L	L	S	E	N
S	N	P	E	N	Y	E	N	T	A	M	D	N	T	L	L	L	S	N	N
N	D	P	K	D	Y	G	H	T	V	I	D	K	T	V	L	M	F	N	D
N	N	T	E	D	Y	G	H	T	V	M	N	K	I	M	F	M	S	N	D

5	11
H	K
P	K
H	K
H	K
P	K
P	R

N	N	T	E	D	Y	G	H	T	V	M	N	K	I	M	F	M	S	N	D	P	R
N	N	T	E	D	Y	G	H	T	V	M	N	K	I	M	F	M	S	N	D	P	R
N	N	T	E	D	Y	G	H	T	V	M	N	K	I	M	F	M	S	N	D	P	R
N	N	T	E	D	Y	G	H	T	V	M	N	K	I	M	F	M	S	N	D	P	R
N	N	T	E	D	Y	G	H	T	V	M	N	K	I	M	F	M	S	N	D	P	R
N	N	T	E	D	Y	G	H	T	V	M	N	K	I	M	F	M	S	N	D	P	R
N	N	T	E	D	Y	G	H	T	V	M	N	K	I	M	F	M	S	N	D	P	R
N	N	T	E	D	Y	G	H	T	V	M	N	K	I	M	F	M	S	N	D	P	R
N	N	T	E	D	Y	G	H	T	V	M	N	K	I	M	F	M	S	N	D	P	R
N	N	T	E	D	Y	G	H	T	V	M	N	K	I	M	F	M	S	N	D	P	R
N	N	T	E	D	Y	G	H	T	V	M	N	K	I	M	F	M	S	N	D	P	R
N	N	T	E	D	Y	G	H	T	V	M	N	K	I	M	F	M	S	N	D	P	R
N	N	T	E	D	Y	G	H	T	V	M	N	K	I	M	F	M	S	N	D	P	R
N	N	T	E	D	Y	G	H	T	V	M	N	K	I	M	F	M	S	N	D	P	R
N	N	T	E	D	Y	G	H	T	V	M	N	K	I	M	F	M	S	N	D	P	R
N	N	T	E	D	Y	G	H	T	V	M	N	K	I	M	F	M	S	N	D	P	R
N	N	T	E	D	Y	G	H	T	V	M	N	K	I	M	F	M	S	N	D	P	R
N	N	T	E	D	Y	G	H	T	V	M	N	K	I	M	F	M	S	N	D	P	R
N	N	T	E	D	Y	G	H	T	V	M	N	K	I	M	F	M	S	N	D	P	R
N	N	T	E	D	Y	G	H	T	V	M	N	K	I	M	F	M	S	N	D	P	R
N	N	T	E	D	Y	G	H	T	V	M	N	K	I	M	F	M	S	N	D	P	R
N	N	T	E	D	Y	G	H	T	V	M	N	K	I	M	F	M	S	N	D	P	R
N	N	T	E	D	Y	G	H	T	V	M	N	K	I	M	F	M	S	N	D	P	R
N	N	T	E	D	Y	G	H	T	V	M	N	K	I	M	F	M	S	N	D	P	R
N	N	T	E	D	Y	G	H	T	V	M	N	K	I	M	F	M	S	N	D	P	R
N	N	T	E	D	Y	G	H	T	V	M	N	K	I	M	F	M	S	N	D	P	R
N	N	T	E	D	Y	G	H	T	V	M	N	K	I	M	F	M	S	N	D	P	R
N	N	T	E	D	Y	G	H	T	V	M	N	K	I	M	F	M	S	N	D	P	R
N	N	T	E	D	Y	G	H	T	V	M	N	K	I	M	F	M	S	N	D	P	R
N	N	T	E	D	Y	G	H	T	V	M	N	K	I	M	F	M	S	N	D	P	R
N	N	T	E	D	Y	G	H	T	V	M	N	K	I	M	F	M	S	N	D	P	R
N	N	T	E	D	Y	G	H	T	V	M	N	K	I	M	F	M	S	N	D	P	R
N	N	T	E	D	Y	G	H	T	V	M	N	K	I	M	F	M	S	N	D	P	R
N	N	T	E	D	Y	G	H	T	V	M	N	K	I	M	F	M	S	N	D	P	R
N	N	T	E	D	Y	G	H	T	V	M	N	K	I	M	F	M	S	N	D	P	R
N	N	T	E	D	Y	G	H	T	V	M	N	K	I	M	F	M	S	N	D	P	R
N	N	T	E	D	Y	G	H	T	V	M	N	K	I	M	F	M	S	N	D	P	R
N	N	T	E	D	Y	G	H	T	V	M	N	K	I	M	F	M	S	N	D	P	R
N	N	T	E	D	Y	G	H	T	V	M	N	K	I	M	F	M	S	N	D	P	R
N	N	T	E	D	Y	G	H	T	V	M	N	K	I	M	F	M	S	N	D	P	R
N	N	T	E	D	Y	G	H	T	V	M	N	K	I	M	F	M	S	N	D	P	R
N	N	T	E	D	Y	G	H	T	V	M	N	K	I	M	F	M	S	N	D	P	R
N	N	T	E	D	Y	G	H	T	V	M	N	K	I	M	F	M	S	N	D	P	R
N	N	T	E	D	Y	G	H	T	V	M	N	K	I	M	F	M	S	N	D	P	R
N	N	T	E	D	Y	G	H	T	V	M	N	K	I	M	F	M	S	N	D	P	R
N	N	T	E	D	Y	G	H	T	V	M	N	K	I	M	F	M	S	N	D	P	R
N	N	T	E	D	Y	G	H	T	V	M	N	K	I	M	F	M	S	N	D	P	R
N	N	T	E	D	Y	G	H	T	V	M	N	K	I	M	F	M	S	N	D	P	R

wzx																						
12	58	78	103	104	129	161	165	166	170	181	213	227	234	257	278	287	298	302	303	309	310	314
S	I	T	K	T	S	V	T	F	F	A	R	L	I	K	N	H	C	G	S	V	V	I
R	M	T	E	I	G	V	I	S	F	A	K	L	I	K	N	Y	R	E	R	I	I	G
S	M	A	K	I	S	I	T	S	F	A	R	F	M	R	S	Y	C	G	S	V	V	I
S	I	T	K	T	S	V	T	F	F	A	R	L	I	K	N	H	C	G	S	V	V	I
R	M	T	E	I	G	V	I	S	F	A	K	L	I	K	N	Y	R	E	R	I	I	G
R	I	T	K	I	G	V	I	S	L	V	R	L	M	K	N	Y	R	G	S	I	T	V

R	I	T	K	I	G	V	I	S	L	V	R	L	M	K	N	Y	R	G	S	I	T	V
R	I	T	K	I	G	V	I	S	L	V	R	L	M	K	N	Y	R	G	S	I	T	V
R	I	T	K	I	G	V	I	S	L	V	R	L	M	K	N	Y	R	G	S	I	T	V
R	I	T	K	I	G	V	I	S	L	V	R	L	M	K	N	Y	R	G	S	I	T	V
R	I	T	K	I	G	V	I	S	L	V	R	L	M	K	N	Y	R	G	S	I	T	V
R	I	T	K	I	G	V	I	S	L	V	R	L	M	K	N	Y	R	G	S	I	T	V
R	I	T	K	I	G	V	I	S	L	V	R	L	M	K	N	Y	R	G	S	I	T	V
R	I	T	K	I	G	V	I	S	L	V	R	L	M	K	N	Y	R	G	S	I	T	V
R	I	T	K	I	G	V	I	S	L	V	R	L	M	K	N	Y	R	G	S	I	T	V
R	I	T	K	I	G	V	I	S	L	V	R	L	M	K	N	Y	R	G	S	I	T	V
R	I	T	K	I	G	V	I	S	L	V	R	L	M	K	N	Y	R	G	S	I	T	V
R	I	T	K	I	G	V	I	S	L	V	R	L	M	K	N	Y	R	G	S	I	T	V
R	I	T	K	I	G	V	I	S	L	V	R	L	M	K	N	Y	R	G	S	I	T	V
R	I	T	K	I	G	V	I	S	L	V	R	L	M	K	N	Y	R	G	S	I	T	V
R	I	T	K	I	G	V	I	S	L	V	R	L	M	K	N	Y	R	G	S	I	T	V
R	I	T	K	I	G	V	I	S	L	V	R	L	M	K	N	Y	R	G	S	I	T	V
R	I	T	K	I	G	V	I	S	L	V	R	L	M	K	N	Y	R	G	S	I	T	V
R	I	T	K	I	G	V	I	S	L	V	R	L	M	K	N	Y	R	G	S	I	T	V
R	I	T	K	I	G	V	I	S	L	V	R	L	M	K	N	Y	R	G	S	I	T	V
R	I	T	K	I	G	V	I	S	L	V	R	L	M	K	N	Y	R	G	S	I	T	V
R	I	T	K	I	G	V	I	S	L	V	R	L	M	K	N	Y	R	G	S	I	T	V
R	I	T	K	I	G	V	I	S	L	V	R	L	M	K	N	Y	R	G	S	I	T	V
R	I	T	K	I	G	V	I	S	L	V	R	L	M	K	N	Y	R	G	S	I	T	V
R	I	T	K	I	G	V	I	S	L	V	R	L	M	K	N	Y	R	G	S	I	T	V
R	I	T	K	I	G	V	I	S	L	V	R	L	M	K	N	Y	R	G	S	I	T	V
R	I	T	K	I	G	V	I	S	L	V	R	L	M	K	N	Y	R	G	S	I	T	V
R	I	T	K	I	G	V	I	S	L	V	R	L	M	K	N	Y	R	G	S	I	T	V
R	I	T	K	I	G	V	I	S	L	V	R	L	M	K	N	Y	R	G	S	I	T	V
R	I	T	K	I	G	V	I	S	L	V	R	L	M	K	N	Y	R	G	S	I	T	V
R	I	T	K	I	G	V	I	S	L	V	R	L	M	K	N	Y	R	G	S	I	T	V
R	I	T	K	I	G	V	I	S	L	V	R	L	M	K	N	Y	R	G	S	I	T	V
R	I	T	K	I	G	V	I	S	L	V	R	L	M	K	N	Y	R	G	S	I	T	V
R	I	T	K	I	G	V	I	S	L	V	R	L	M	K	N	Y	R	G	S	I	T	V
R	I	T	K	I	G	V	I	S	L	V	R	L	M	K	N	Y	R	G	S	I	T	V
R	I	T	K	I	G	V	I	S	L	V	R	L	M	K	N	Y	R	G	S	I	T	V
R	I	T	K	I	G	V	I	S	L	V	R	L	M	K	N	Y	R	G	S	I	T	V
R	I	T	K	I	G	V	I	S	L	V	R	L	M	K	N	Y	R	G	S	I	T	V
R	I	T	K	I	G	V	I	S	L	V	R	L	M	K	N	Y	R	G	S	I	T	V
R	I	T	K	I	G	V	I	S	L	V	R	L	M	K	N	Y	R	G	S	I	T	V
R	I	T	K	I	G	V	I	S	L	V	R	L	M	K	N	Y	R	G	S	I	T	V
R	I	T	K	I	G	V	I	S	L	V	R	L	M	K	N	Y	R	G	S	I	T	V
R	I	T	K	I	G	V	I	S	L	V	R	L	M	K	N	Y	R	G	S	I	T	V
R	I	T	K	I	G	V	I	S	L	V	R	L	M	K	N	Y	R	G	S	I	T	V
R	I	T	K	I	G	V	I	S	L	V	R	L	M	K	N	Y	R	G	S	I	T	V
R	I	T	K	I	G	V	I	S	L	V	R	L	M	K	N	Y	R	G	S	I	T	V
R	I	T	K	I	G	V	I	S	L	V	R	L	M	K	N	Y	R	G	S	I	T	V
R	I	T	K	I	G	V	I	S	L	V	R	L	M	K	N	Y	R	G	S	I	T	V
R	I	T	K	I	G	V	I	S	L	V	R	L	M	K	N	Y	R	G	S	I	T	V

316	346	375	377	398	444	457	464	466	467
A	I	T	A	A	I	I	A	I	L
V	V	T	A	A	T	F	V	I	L
A	I	I	V	A	I	I	A	V	L
A	I	T	A	A	I	I	A	I	L
V	V	T	A	A	T	F	V	I	L
V	I	T	A	S	T	F	V	I	V

gct				
12	71	86	95	111
L	S	H	D	I
F	P	H	D	V
L	P	R	D	V
L	S	H	D	I
F	P	H	N	V
L	P	H	D	V

30	76	102	114	121	122
S	H	L	H	R	Y
[redacted]					
S	Y	L	H	R	Y
Y	H	V	F	K	F
Y	H	V	F	K	F

V	I	T	A	S	T	F	V	I	V	L	P	H	D	V	Y	H	V	F	K	F
V	I	T	A	S	T	F	V	I	V	L	P	H	D	V	Y	H	V	F	K	F
V	I	T	A	S	T	F	V	I	V	L	P	H	D	V	Y	H	V	F	K	F
V	I	T	A	S	T	F	V	I	V	L	P	H	D	V	Y	H	V	F	K	F
V	I	T	A	S	T	F	V	I	V	L	P	H	D	V	Y	H	V	F	K	F
V	I	T	A	S	T	F	V	I	V	L	P	H	D	V	Y	H	V	F	K	F
V	I	T	A	S	T	F	V	I	V	L	P	H	D	V	Y	H	V	F	K	F
V	I	T	A	S	T	F	V	I	V	L	P	H	D	V	Y	H	V	F	K	F
V	I	T	A	S	T	F	V	I	V	L	P	H	D	V	Y	H	V	F	K	F
V	I	T	A	S	T	F	V	I	V	L	P	H	D	V	Y	H	V	F	K	F
V	I	T	A	S	T	F	V	I	V	L	P	H	D	V	Y	H	V	F	K	F
V	I	T	A	S	T	F	V	I	V	L	P	H	D	V	Y	H	V	F	K	F
V	I	T	A	S	T	F	V	I	V	L	P	H	D	V	Y	H	V	F	K	F
V	I	T	A	S	T	F	V	I	V	L	P	H	D	V	Y	H	V	F	K	F
V	I	T	A	S	T	F	V	I	V	L	P	H	D	V	Y	H	V	F	K	F
V	I	T	A	S	T	F	V	I	V	L	P	H	D	V	Y	H	V	F	K	F
V	I	T	A	S	T	F	V	I	V	L	P	H	D	V	Y	H	V	F	K	F
V	I	T	A	S	T	F	V	I	V	L	P	H	D	V	Y	H	V	F	K	F
V	I	T	A	S	T	F	V	I	V	L	P	H	D	V	Y	H	V	F	K	F
V	I	T	A	S	T	F	V	I	V	L	P	H	D	V	Y	H	V	F	K	F
V	I	T	A	S	T	F	V	I	V	L	P	H	D	V	Y	H	V	F	K	F
V	I	T	A	S	T	F	V	I	V	L	P	H	D	V	Y	H	V	F	K	F
V	I	T	A	S	T	F	V	I	V	L	P	H	D	V	Y	H	V	F	K	F
V	I	T	A	S	T	F	V	I	V	L	P	H	D	V	Y	H	V	F	K	F
V	I	T	A	S	T	F	V	I	V	L	P	H	D	V	Y	H	V	F	K	F
V	I	T	A	S	T	F	V	I	V	L	P	H	D	V	Y	H	V	F	K	F
V	I	T	A	S	T	F	V	I	V	L	P	H	D	V	Y	H	V	F	K	F
V	I	T	A	S	T	F	V	I	V	L	P	H	D	V	Y	H	V	F	K	F
V	I	T	A	S	T	F	V	I	V	L	P	H	D	V	Y	H	V	F	K	F
V	I	T	A	S	T	F	V	I	V	L	P	H	D	V	Y	H	V	F	K	F
V	I	T	A	S	T	F	V	I	V	L	P	H	D	V	Y	H	V	F	K	F
V	I	T	A	S	T	F	V	I	V	L	P	H	D	V	Y	H	V	F	K	F
V	I	T	A	S	T	F	V	I	V	L	P	H	D	V	Y	H	V	F	K	F
V	I	T	A	S	T	F	V	I	V	L	P	H	D	V	Y	H	V	F	K	F
V	I	T	A	S	T	F	V	I	V	L	P	H	D	V	Y	H	V	F	K	F
V	I	T	A	S	T	F	V	I	V	L	P	H	D	V	Y	H	V	F	K	F
V	I	T	A	S	T	F	V	I	V	L	P	H	D	V	Y	H	V	F	K	F
V	I	T	A	S	T	F	V	I	V	L	P	H	D	V	Y	H	V	F	K	F
V	I	T	A	S	T	F	V	I	V	L	P	H	D	V	Y	H	V	F	K	F
V	I	T	A	S	T	F	V	I	V	L	P	H	D	V	Y	H	V	F	K	F
V	I	T	A	S	T	F	V	I	V	L	P	H	D	V	Y	H	V	F	K	F
V	I	T	A	S	T	F	V	I	V	L	P	H	D	V	Y	H	V	F	K	F
V	I	T	A	S	T	F	V	I	V	L	P	H	D	V	Y	H	V	F	K	F
V	I	T	A	S	T	F	V	I	V	L	P	H	D	V	Y	H	V	F	K	F
V	I	T	A	S	T	F	V	I	V	L	P	H	D	V	Y	H	V	F	K	F
V	I	T	A	S	T	F	V	I	V	L	P	H	D	V	Y	H	V	F	K	F
V	I	T	A	S	T	F	V	I	V	L	P	H	D	V	Y	H	V	F	K	F
V	I	T	A	S	T	F	V	I	V	L	P	H	D	V	Y	H	V	F	K	F

wcjE											
145	154	169	182	185	193	196	200	203	205	234	249
I	F	I	V	C	V	N	L	F	F	S	V
[redacted]											
I	F	I	I	F	I	D	L	F	F	I	V
V	L	T	I	F	I	D	L	I	F	S	A
V	L	T	I	L	I	D	F	I	V	S	V

V	L	T	I	L	I	D	F	I	V	S	V
V	L	T	I	L	I	D	F	I	V	S	V
V	L	T	I	L	I	D	F	I	V	S	V
V	L	T	I	L	I	D	F	I	V	S	V
V	L	T	I	L	I	D	F	I	V	S	V
V	L	T	I	L	I	D	F	I	V	S	V
V	L	T	I	L	I	D	F	I	V	S	V
V	L	T	I	L	I	D	F	I	V	S	V
V	L	T	I	L	I	D	F	I	V	S	V
V	L	T	I	L	I	D	F	I	V	S	V
V	L	T	I	L	I	D	F	I	V	S	V
V	L	T	I	L	I	D	F	I	V	S	V
V	L	T	I	L	I	D	F	I	V	S	V
V	L	T	I	L	I	D	F	I	V	S	V
V	L	T	I	L	I	D	F	I	V	S	V
V	L	T	I	L	I	D	F	I	V	S	V
V	L	T	I	L	I	D	F	I	V	S	V
V	L	T	I	L	I	D	F	I	V	S	V
V	L	T	I	L	I	D	F	I	V	S	V
V	L	T	I	L	I	D	F	I	V	S	V
V	L	T	I	L	I	D	F	I	V	S	V
V	L	T	I	L	I	D	F	I	V	S	V
V	L	T	I	L	I	D	F	I	V	S	V
V	L	T	I	L	I	D	F	I	V	S	V
V	L	T	I	L	I	D	F	I	V	S	V
V	L	T	I	L	I	D	F	I	V	S	V
V	L	T	I	L	I	D	F	I	V	S	V
V	L	T	I	L	I	D	F	I	V	S	V
V	L	T	I	L	I	D	F	I	V	S	V
V	L	T	I	L	I	D	F	I	V	S	V
V	L	T	I	L	I	D	F	I	V	S	V
V	L	T	I	L	I	D	F	I	V	S	V
V	L	T	I	L	I	D	F	I	V	S	V
V	L	T	I	L	I	D	F	I	V	S	V
V	L	T	I	L	I	D	F	I	V	S	V
V	L	T	I	L	I	D	F	I	V	S	V
V	L	T	I	L	I	D	F	I	V	S	V
V	L	T	I	L	I	D	F	I	V	S	V
V	L	T	I	L	I	D	F	I	V	S	V
V	L	T	I	L	I	D	F	I	V	S	V
V	L	T	I	L	I	D	F	I	V	S	V
V	L	T	I	L	I	D	F	I	V	S	V
V	L	T	I	L	I	D	F	I	V	S	V
V	L	T	I	L	I	D	F	I	V	S	V
V	L	T	I	L	I	D	F	I	V	S	V
V	L	T	I	L	I	D	F	I	V	S	V
V	L	T	I	L	I	D	F	I	V	S	V
V	L	T	I	L	I	D	F	I	V	S	V

rmlst ID	PubMLST id	ENA	Name	ST	CC	Orig_sero	Orig_sero_method	Seq_sero
121361	33805	ERR044901	UoS0174	698	698	unknown	WGS-based	22F
121408	33852	ERR044967	UoS2014	819	433	unknown	WGS-based	22F
123307	34174	ERR045010	UoS2196	698	698	unknown	WGS-based	22F
115837	33775	ERR045011	UoS2199	698	698	unknown	WGS-based	22F
121363	33807	ERR045028	UoS2254	698	698	unknown	WGS-based	22F
121457	33901	ERR045043	UoS2306	433	433	unknown	WGS-based	22F
121450	33894	ERR048227	UoS3016	433	433	unknown	WGS-based	22F
121434	33878	ERR046054	UoS3032	433	433	unknown	WGS-based	22F
123219	34143	ERR046073	UoS3093	698	698	unknown	WGS-based	22F
121442	33886	ERR046076	UoS3108	433	433	unknown	WGS-based	22F
121453	33897	ERR046077	UoS3109	433	433	unknown	WGS-based	22F
121383	33827	ERR046088	UoS3160	433	433	unknown	WGS-based	22F
121507	33951	ERR046106	UoS3226	433	433	unknown	WGS-based	22F
115845	33782	ERR046133	UoS3298	698	698	unknown	WGS-based	22F
115846	33783	ERR046142	UoS3326	433	433	unknown	WGS-based	22F
40138	33019	ERR046144	UoS4008	3734	698	unknown	WGS-based	22F
121451	33895	ERR046154	UoS4036	433	433	unknown	WGS-based	22F
115848	33785	ERR046183	UoS4157	819	433	unknown	WGS-based	22F
115849	33786	ERR046188	UoS4168	698	698	unknown	WGS-based	22F
121413	33857	ERR046189	UoS4169	819	433	unknown	WGS-based	22F
121559	34003	ERR046196	UoS4208	4325	433	unknown	WGS-based	22F
40141	33022	ERR046205	UoS4252	698	698	unknown	WGS-based	22F
121381	33825	ERR045363	UoS4318	433	433	unknown	WGS-based	22F
123202	34126	ERR045375	UoS4356	433	433	unknown	WGS-based	22F
121452	33896	ERR045407	UoS5027	433	433	unknown	WGS-based	22F
121435	33879	ERR047107	UoS5186	433	433	unknown	WGS-based	22F
123238	34162	ERR047148	UoS5248	698	698	unknown	WGS-based	22F
N/A	15452	N/A	22A/2	7181	74	22A	Quellung	22A
N/A	15448	N/A	22A/3	7181	74	22A	Quellung	22A
N/A	15490	N/A	22F/2	7219	Sing7219	22F	Quellung	22F
83281	27971	ERR051586	SMRU1040	910	910/9483	22A	latex & Quellung	22A
33953	27972	ERR051595	SMRU1049	910	910/9483	22A	latex & Quellung	22A
82217	27973	ERR051598	SMRU1052	910	910/9483	22A	latex & Quellung	22A
109833	27974	ERR054644	SMRU1193	910	910/9483	22A	latex & Quellung	22A
109779	27975	ERR054329	SMRU1236	910	910/9483	22A	latex & Quellung	22A
109780	27976	ERR054332	SMRU1239	910	910/9483	22A	latex & Quellung	22A
111518	27977	ERR054393	SMRU1300	910	910/9483	22A	latex & Quellung	22A
111542	27978	ERR054252	SMRU1375	910	910/9483	22A	latex & Quellung	22A
82309	27979	ERR056799	SMRU1461	910	910/9483	22A	latex & Quellung	22A
115537	27980	ERR054475	SMRU1527	910	910/9483	22A	latex & Quellung	22A
33917	27965	ERR049122	SMRU188	910	910/9483	22A	latex & Quellung	22A
111456	27981	ERR057970	SMRU1989	910	910/9483	22A	latex & Quellung	22A
33880	27966	ERR047968	SMRU291	910	910/9483	22A	latex & Quellung	22A
109685	27967	ERR048050	SMRU325	910	910/9483	22A	latex & Quellung	22A
33929	27968	ERR051480	SMRU670	910	910/9483	22A	latex & Quellung	22A
40225	27969	ERR051530	SMRU720	910	910/9483	22A	latex & Quellung	22A
83291	27970	ERR052606	SMRU749	910	910/9483	22A	latex & Quellung	22A
83284	27985	ERR051601	SMRU1055	6709	Sing6709	22F	latex & Quellung	22F
113734	27982	ERR047905	SMRU108	6709	Sing6709	22F	latex & Quellung	22F
32986	27986	ERR051654	SMRU1108	6709	Sing6709	22F	latex & Quellung	22F
109821	27987	ERR054593	SMRU1141	6709	Sing6709	22F	latex & Quellung	22F
83211	27984	ERR047916	SMRU119	6709	Sing6709	22F	latex & Quellung	22F
109772	27988	ERR054304	SMRU1211	6709	Sing6709	22F	latex & Quellung	22F
83322	27989	ERR054480	SMRU1532	6709	Sing6709	22F	latex & Quellung	22F
82509	27990	ERR066235	SMRU2268	1294	1294/4522	22F	latex & Quellung	22F
83503	27991	ERR066335	SMRU2368	1294	1294/4522	22F	latex & Quellung	22F
115589	27992	ERR064072	SMRU2670	6709	Sing6709	22F	latex & Quellung	22F
109949	27993	ERR064109	SMRU2707	6709	Sing6709	22F	latex & Quellung	22F

115610	27995	ERR084246	SMRU3020	1294	1294/4522	22F	latex & Quellung	22F
32901	27983	ERR039600	SMRU41	6709	Sing6709	22F	latex & Quellung	22F
32173	29472	ERR069738	35100	1901	433	22F	Quellung	22F
33267	29474	ERR069767	120581	433	433	22F	Quellung	22F
33271	29612	ERR069778	153408	433	433	22F	Quellung	22F
32201	29610	ERR069783	163286	433	433	22F	Quellung	22F
31467	29605	ERR069836	385385	433	433	22F	Quellung	22F
32421	29611	ERR129096	CH2108	433	433	22F	Quellung	22F
32464	29280	ERR129156	LE4118	433	433	22F	Quellung	22F
31566	29606	ERR129204	ND6047	433	433	22F	Quellung	22F
32333	29473	ERR124248	NP7013	433	433	22F	Quellung	22F
33403	29281	ERR124260	NP7062	1901	433	22F	Quellung	22F
33427	29282	ERR124307	PT8085	433	433	22F	Quellung	22F
31504	29278	ERR124318	PT8116	433	433	22F	Quellung	22F
32058	29607	ERR065338	R34-3012	1337	433	22F	Quellung	22F
31310	29469	ERR065343	R34-3017	433	433	22F	Quellung	22F
32080	29608	ERR067987	R34-3082	1337	433	22F	Quellung	22F
31345	29277	ERR067996	R34-3091	433	433	22F	Quellung	22F
32092	29471	ERR068037	R34-3143	433	433	22F	Quellung	22F
31324	29604	ERR067965	R34-3167	433	433	22F	Quellung	22F
32138	29279	ERR069686	R34-3187	433	433	22F	Quellung	22F
32140	29609	ERR069688	R34-3189	433	433	22F	Quellung	22F
N/A	33223	ERR470252	VICE0481	433	433	not done	--	22F
N/A	33497	ERR388890	VICE0207	433	433	not done	--	22F
N/A	34559	ERR388828	VICE0245	433	433	not done	--	22F
N/A	36052	ERR755433	VICE1601	433	433	not done	--	22F
N/A	33093	ERR388923	VICE0265	433	433	not done	--	22F
N/A	33451	ERR470489	VICE1011a	433	433	not done	--	22F
N/A	33678	ERR540548	VICE1019	433	433	not done	--	22F
N/A	36149	ERR755377	VICE1716	433	433	not done	--	22F
N/A	36150	ERR755378	VICE1717	433	433	not done	--	22F
N/A	36151	ERR755379	VICE1718	433	433	not done	--	22F
N/A	36157	ERR755386	VICE1729	433	433	not done	--	22F
N/A	36159	ERR755388	VICE1731	433	433	not done	--	22F
N/A	36166	ERR755478	VICE1742	433	433	not done	--	22F
N/A	36167	ERR755479	VICE1743	433	433	not done	--	22F
N/A	36182	ERR755495	VICE1759b	433	433	not done	--	22F
N/A	34424	ERR697264	VICE1532	433	433	not done	--	22F
N/A	34440	ERR697265	VICE1533	433	433	not done	--	22F
N/A	34310	ERR697266	VICE1534	433	433	not done	--	22F
N/A	34461	ERR697267	VICE1535	433	433	not done	--	22F
N/A	36205	ERR755523	VICE1793	433	433	not done	--	22F
N/A	36212	ERR755532	VICE1803	433	433	not done	--	22F
N/A	36213	ERR755533	VICE1804	433	433	not done	--	22F
N/A	36217	ERR755538	VICE1814	433	433	not done	--	22F
N/A	36218	ERR755539	VICE1815	433	433	not done	--	22F
N/A	36285	ERR755623	VICE1906	433	433	not done	--	22F
N/A	33628	ERR540453	VICE0277	433	433	not done	--	22F
N/A	33123	ERR388877	VICE0186	433	433	not done	--	22F
N/A	33096	ERR388899	VICE0216	433	433	not done	--	22F
N/A	33623	ERR540445	VICE0243	433	433	not done	--	22F
N/A	33500	ERR388922	VICE0264	433	433	not done	--	22F
N/A	33534	ERR470293	VICE0610	433	433	not done	--	22F
N/A	33506	ERR449849	VICE0673	433	433	not done	--	22F
N/A	33421	ERR470366	VICE0710	433	433	not done	--	22F
N/A	33254	ERR470382	VICE0734	433	433	not done	--	22F
N/A	33278	ERR470383	VICE0735	433	433	not done	--	22F
N/A	33160	ERR470304	VICE0741	433	433	not done	--	22F
N/A	33280	ERR470397	VICE0760	433	433	not done	--	22F

N/A	33318	ERR470423	VICE0804	433	433	not done	--	22F
N/A	33658	ERR540503	VICE0818	433	433	not done	--	22F
N/A	33320	ERR470447	VICE0839	433	433	not done	--	22F
N/A	34207	ERR689462	VICE1374	433	433	not done	--	22F
N/A	34415	ERR689510	VICE1431	433	433	not done	--	22F
N/A	34325	ERR689511	VICE1432	433	433	not done	--	22F
N/A	34263	ERR697288	VICE1555	433	433	not done	--	22F
N/A	34317	ERR697291	VICE1559	433	433	not done	--	22F
N/A	34265	ERR697292	VICE1560	433	433	not done	--	22F
N/A	34462	ERR697297	VICE1565	433	433	not done	--	22F
N/A	36048	ERR755425	VICE1591	433	433	not done	--	22F
N/A	36114	ERR755340	VICE1676	433	433	not done	--	22F
N/A	36250	ERR755576	VICE1859	433	433	not done	--	22F

Hybrid	Hybrid type	Country	Region	carriage/disease	Source	Year
No	N/A	United Kingdom	Southampton	carriage	nasopharynx	2006-2011
No	N/A	United Kingdom	Southampton	carriage	nasopharynx	2006-2011
No	N/A	United Kingdom	Southampton	carriage	nasopharynx	2006-2011
No	N/A	United Kingdom	Southampton	carriage	nasopharynx	2006-2011
No	N/A	United Kingdom	Southampton	carriage	nasopharynx	2006-2011
No	N/A	United Kingdom	Southampton	carriage	nasopharynx	2006-2011
No	N/A	United Kingdom	Southampton	carriage	nasopharynx	2006-2011
No	N/A	United Kingdom	Southampton	carriage	nasopharynx	2006-2011
No	N/A	United Kingdom	Southampton	carriage	nasopharynx	2006-2011
No	N/A	United Kingdom	Southampton	carriage	nasopharynx	2006-2011
No	N/A	United Kingdom	Southampton	carriage	nasopharynx	2006-2011
No	N/A	United Kingdom	Southampton	carriage	nasopharynx	2006-2011
No	N/A	United Kingdom	Southampton	carriage	nasopharynx	2006-2011
No	N/A	United Kingdom	Southampton	carriage	nasopharynx	2006-2011
No	N/A	United Kingdom	Southampton	carriage	nasopharynx	2006-2011
No	N/A	United Kingdom	Southampton	carriage	nasopharynx	2006-2011
No	N/A	United Kingdom	Southampton	carriage	nasopharynx	2006-2011
No	N/A	United Kingdom	Southampton	carriage	nasopharynx	2006-2011
No	N/A	United Kingdom	Southampton	carriage	nasopharynx	2006-2011
No	N/A	United Kingdom	Southampton	carriage	nasopharynx	2006-2011
No	N/A	United Kingdom	Southampton	carriage	nasopharynx	2006-2011
No	N/A	United Kingdom	Southampton	carriage	nasopharynx	2006-2011
No	N/A	United Kingdom	Southampton	carriage	nasopharynx	2006-2011
No	N/A	United Kingdom	Southampton	carriage	nasopharynx	2006-2011
No	N/A	United Kingdom	Southampton	carriage	nasopharynx	2006-2011
No	N/A	United Kingdom	Southampton	carriage	nasopharynx	2006-2011
No	N/A	Denmark	unknown	unknown	unknown	1939
No	N/A	Denmark	unknown	unknown	unknown	1939
No	N/A	Denmark	unknown	unknown	unknown	1940
No	N/A	Thailand	Maela	carriage	nasopharynx	2008
No	N/A	Thailand	Maela	carriage	nasopharynx	2008
No	N/A	Thailand	Maela	carriage	nasopharynx	2008
No	N/A	Thailand	Maela	carriage	nasopharynx	2009
No	N/A	Thailand	Maela	carriage	nasopharynx	2009
No	N/A	Thailand	Maela	carriage	nasopharynx	2009
No	N/A	Thailand	Maela	carriage	nasopharynx	2009
No	N/A	Thailand	Maela	carriage	nasopharynx	2009
No	N/A	Thailand	Maela	carriage	nasopharynx	2009
No	N/A	Thailand	Maela	carriage	nasopharynx	2009
No	N/A	Thailand	Maela	carriage	nasopharynx	2008
No	N/A	Thailand	Maela	carriage	nasopharynx	2009
No	N/A	Thailand	Maela	carriage	nasopharynx	2008
No	N/A	Thailand	Maela	carriage	nasopharynx	2008
No	N/A	Thailand	Maela	carriage	nasopharynx	2008
No	N/A	Thailand	Maela	carriage	nasopharynx	2008
No	N/A	Thailand	Maela	carriage	nasopharynx	2008
Yes	22F/33A	Thailand	Maela	carriage	nasopharynx	2008
Yes	22F/33A	Thailand	Maela	carriage	nasopharynx	2008
Yes	22F/33A	Thailand	Maela	carriage	nasopharynx	2008
Yes	22F/33A	Thailand	Maela	carriage	nasopharynx	2009
Yes	22F/33A	Thailand	Maela	carriage	nasopharynx	2008
Yes	22F/33A	Thailand	Maela	carriage	nasopharynx	2009
Yes	22F/33A	Thailand	Maela	carriage	nasopharynx	2009
Yes	22F/33A	Thailand	Maela	carriage	nasopharynx	2009
Yes	22F/33A	Thailand	Maela	carriage	nasopharynx	2010
Yes	22F/33A	Thailand	Maela	carriage	nasopharynx	2010
Yes	22F/33A	Thailand	Maela	carriage	nasopharynx	2010

Yes	22F/33A	Thailand	Maela	carriage	nasopharynx	2010
Yes	22F/33A	Thailand	Maela	carriage	nasopharynx	2008
No	N/A	USA	Massachusetts	carriage	nasopharynx	2001
No	N/A	USA	Massachusetts	carriage	nasopharynx	2001
No	N/A	USA	Massachusetts	carriage	nasopharynx	2001
No	N/A	USA	Massachusetts	carriage	nasopharynx	2001
No	N/A	USA	Massachusetts	carriage	nasopharynx	2001
No	N/A	USA	Massachusetts	carriage	nasopharynx	2007
No	N/A	USA	Massachusetts	carriage	nasopharynx	2007
No	N/A	USA	Massachusetts	carriage	nasopharynx	2007
No	N/A	USA	Massachusetts	carriage	nasopharynx	2007
No	N/A	USA	Massachusetts	carriage	nasopharynx	2007
No	N/A	USA	Massachusetts	carriage	nasopharynx	2007
No	N/A	USA	Massachusetts	carriage	nasopharynx	2007
No	N/A	USA	Massachusetts	carriage	nasopharynx	2004
No	N/A	USA	Massachusetts	carriage	nasopharynx	2004
No	N/A	USA	Massachusetts	carriage	nasopharynx	2004
No	N/A	USA	Massachusetts	carriage	nasopharynx	2004
No	N/A	USA	Massachusetts	carriage	nasopharynx	2004
No	N/A	USA	Massachusetts	carriage	nasopharynx	2004
No	N/A	USA	Massachusetts	carriage	nasopharynx	2004
No	N/A	USA	Massachusetts	carriage	nasopharynx	2004
No	N/A	Iceland	Akureyri	disease	blood	2013
No	N/A	Iceland	Garðabær	disease	blood	2011
No	N/A	Iceland	Garðabær	disease	blood	2012
No	N/A	Iceland	Garðabær	disease	lung aspirate	2013
No	N/A	Iceland	Grindavík	disease	blood	2012
No	N/A	Iceland	Hafnarfjörður	carriage	nasopharynx	2009
No	N/A	Iceland	Hafnarfjörður	carriage	nasopharynx	2009
No	N/A	Iceland	Hafnarfjörður	carriage	nasopharynx	2014
No	N/A	Iceland	Hafnarfjörður	carriage	nasopharynx	2014
No	N/A	Iceland	Hafnarfjörður	carriage	nasopharynx	2014
No	N/A	Iceland	Hafnarfjörður	carriage	nasopharynx	2014
No	N/A	Iceland	Hafnarfjörður	carriage	nasopharynx	2014
No	N/A	Iceland	Hafnarfjörður	carriage	nasopharynx	2014
No	N/A	Iceland	Hafnarfjörður	carriage	nasopharynx	2014
No	N/A	Iceland	Hafnarfjörður	carriage	nasopharynx	2014
No	N/A	Iceland	Kópavogur	carriage	nasopharynx	2013
No	N/A	Iceland	Kópavogur	carriage	nasopharynx	2013
No	N/A	Iceland	Kópavogur	carriage	nasopharynx	2013
No	N/A	Iceland	Kópavogur	carriage	nasopharynx	2013
No	N/A	Iceland	Kópavogur	carriage	nasopharynx	2014
No	N/A	Iceland	Kópavogur	carriage	nasopharynx	2014
No	N/A	Iceland	Kópavogur	carriage	nasopharynx	2014
No	N/A	Iceland	Kópavogur	carriage	nasopharynx	2014
No	N/A	Iceland	Kópavogur	carriage	nasopharynx	2014
No	N/A	Iceland	Mosfellsbær	disease	blood	2014
No	N/A	Iceland	Reyðarfjörður	disease	blood	2013
No	N/A	Iceland	Reykjavík	disease	blood	2010
No	N/A	Iceland	Reykjavík	disease	blood	2011
No	N/A	Iceland	Reykjavík	disease	blood	2012
No	N/A	Iceland	Reykjavík	disease	blood	2012
No	N/A	Iceland	Reykjavík	disease	middle ear fluid	2012
No	N/A	Iceland	Reykjavík	disease	sputum	2009
No	N/A	Iceland	Reykjavík	disease	sputum	2010
No	N/A	Iceland	Reykjavík	disease	sputum	2011
No	N/A	Iceland	Reykjavík	disease	sputum	2011
No	N/A	Iceland	Reykjavík	disease	sputum	2011
No	N/A	Iceland	Reykjavík	disease	sputum	2011

No	N/A	Iceland	Reykjavík	disease	sputum	2012
No	N/A	Iceland	Reykjavík	disease	lung aspirate	2009
No	N/A	Iceland	Reykjavík	disease	lung aspirate	2012
No	N/A	Iceland	Reykjavík	carriage	nasopharynx	2014
No	N/A	Iceland	Reykjavík	carriage	nasopharynx	2012
No	N/A	Iceland	Reykjavík	carriage	nasopharynx	2012
No	N/A	Iceland	Reykjavík	carriage	nasopharynx	2013
No	N/A	Iceland	Reykjavík	carriage	nasopharynx	2013
No	N/A	Iceland	Reykjavík	carriage	nasopharynx	2013
No	N/A	Iceland	Reykjavík	carriage	nasopharynx	2013
No	N/A	Iceland	Reykjavík	carriage	nasopharynx	2013
No	N/A	Iceland	Reykjavík	disease	middle ear fluid	2014
No	N/A	Iceland	Reykjavík	carriage	nasopharynx	2014

Publication	Reference	vaccine status
Gladstone UK PCV	PMID: 25776920	unknown
Gladstone UK PCV	PMID: 25776920	unknown
Gladstone UK PCV	PMID: 25776920	unknown
Gladstone UK PCV	PMID: 25776920	unknown
Gladstone UK PCV	PMID: 25776920	unknown
Gladstone UK PCV	PMID: 25776920	unknown
Gladstone UK PCV	PMID: 25776920	unknown
Gladstone UK PCV	PMID: 25776920	unknown
Gladstone UK PCV	PMID: 25776920	unknown
Gladstone UK PCV	PMID: 25776920	unknown
Gladstone UK PCV	PMID: 25776920	unknown
Gladstone UK PCV	PMID: 25776920	unknown
Gladstone UK PCV	PMID: 25776920	unknown
Gladstone UK PCV	PMID: 25776920	unknown
Gladstone UK PCV	PMID: 25776920	unknown
Gladstone UK PCV	PMID: 25776920	unknown
Gladstone UK PCV	PMID: 25776920	unknown
Gladstone UK PCV	PMID: 25776920	unknown
Gladstone UK PCV	PMID: 25776920	unknown
Gladstone UK PCV	PMID: 25776920	unknown
Gladstone UK PCV	PMID: 25776920	unknown
Gladstone UK PCV	PMID: 25776920	unknown
Gladstone UK PCV	PMID: 25776920	unknown
Gladstone UK PCV	PMID: 25776920	unknown
Gladstone UK PCV	PMID: 25776920	unknown
Gladstone UK PCV	PMID: 25776920	unknown
Gladstone UK PCV	PMID: 25776920	unknown
Historical	unknown	pre
Historical	unknown	pre
Historical	unknown	pre
Maela	PMID: 24509479	pre
Maela	PMID: 24509479	pre
Maela	PMID: 24509479	pre
Maela	PMID: 24509479	pre
Maela	PMID: 24509479	pre
Maela	PMID: 24509479	pre
Maela	PMID: 24509479	pre
Maela	PMID: 24509479	pre
Maela	PMID: 24509479	pre
Maela	PMID: 24509479	pre
Maela	PMID: 24509479	pre
Maela	PMID: 24509479	pre
Maela	PMID: 24509479	pre
Maela	PMID: 24509479	pre
Maela	PMID: 24509479	pre
Maela	PMID: 24509479	pre
Maela	PMID: 24509479	pre
Maela	PMID: 24509479	pre
Maela	PMID: 24509479	pre
Maela	PMID: 24509479	pre
Maela	PMID: 24509479	pre
Maela	PMID: 24509479	pre
Maela	PMID: 24509479	pre
Maela	PMID: 24509479	pre
Maela	PMID: 24509479	pre
Maela	PMID: 24509479	pre
Maela	PMID: 24509479	pre
Maela	PMID: 24509479	pre

Maela	PMID: 24509479	pre
Maela	PMID: 24509479	pre
Massachusetts	PMID: 23644493	post
Massachusetts	PMID: 23644493	post
Massachusetts	PMID: 23644493	post
Massachusetts	PMID: 23644493	post
Massachusetts	PMID: 23644493	post
Massachusetts	PMID: 23644493	post
Massachusetts	PMID: 23644493	post
Massachusetts	PMID: 23644493	post
Massachusetts	PMID: 23644493	post
Massachusetts	PMID: 23644493	post
Massachusetts	PMID: 23644493	post
Massachusetts	PMID: 23644493	post
Massachusetts	PMID: 23644493	post
Massachusetts	PMID: 23644493	post
Massachusetts	PMID: 23644493	post
Massachusetts	PMID: 23644493	post
Massachusetts	PMID: 23644493	post
Massachusetts	PMID: 23644493	post
Massachusetts	PMID: 23644493	post
Massachusetts	PMID: 23644493	post
VICE	This publication	post
VICE	This publication	post
VICE	This publication	post
VICE	This publication	post
VICE	This publication	post
VICE	This publication	pre
VICE	This publication	pre
VICE	This publication	post
VICE	This publication	post
VICE	This publication	post
VICE	This publication	post
VICE	This publication	post
VICE	This publication	post
VICE	This publication	post
VICE	This publication	post
VICE	This publication	post
VICE	This publication	post
VICE	This publication	post
VICE	This publication	post
VICE	This publication	post
VICE	This publication	post
VICE	This publication	post
VICE	This publication	post
VICE	This publication	post
VICE	This publication	post
VICE	This publication	post
VICE	This publication	pre
VICE	This publication	post
VICE	This publication	post
VICE	This publication	post
VICE	This publication	post
VICE	This publication	pre
VICE	This publication	pre
VICE	This publication	post
VICE	This publication	post
VICE	This publication	post
VICE	This publication	post

VICE	This publication	post
VICE	This publication	pre
VICE	This publication	post
VICE	This publication	post
VICE	This publication	post
VICE	This publication	post
VICE	This publication	post
VICE	This publication	post
VICE	This publication	post
VICE	This publication	post
VICE	This publication	post
VICE	This publication	post
VICE	This publication	post

SEROTYPE 22A ALL ISOLATES

					wzg	wzh	wzd	wze
							99	
		22A reference					P	
	134	22A/3	74	22A			P	
	2957	22A/2	74	22A			P	
33880		SMRU291	910/9483	22A			S	
33917		SMRU188	910/9483	22A			S	
33929		SMRU670	910/9483	22A			S	
33953		SMRU1049	910/9483	22A			S	
40225		SMRU720	910/9483	22A			S	
82217		SMRU1052	910/9483	22A			S	
82309		SMRU1461	910/9483	22A			S	
83281		SMRU1040	910/9483	22A			S	
83291		SMRU749	910/9483	22A			S	
109685		SMRU325	910/9483	22A			S	
109779		SMRU1236	910/9483	22A			S	
109780		SMRU1239	910/9483	22A			S	
109833		SMRU1193	910/9483	22A			S	
111456		SMRU1989	910/9483	22A			S	
111518		SMRU1300	910/9483	22A			S	
111542		SMRU1375	910/9483	22A			S	
115537		SMRU1527	910/9483	22A			S	

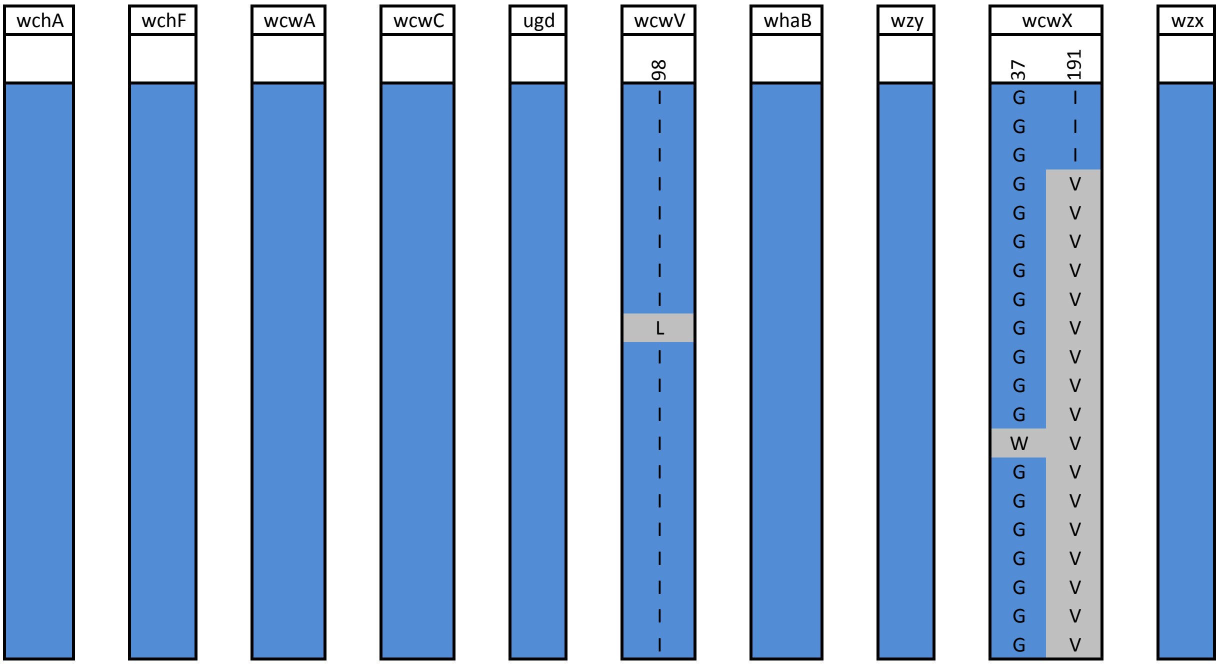
wchA
wchF
wcwA
wcwC
ugd
wcwV
whaB
wzy
wcwX
wzx
98
37
191

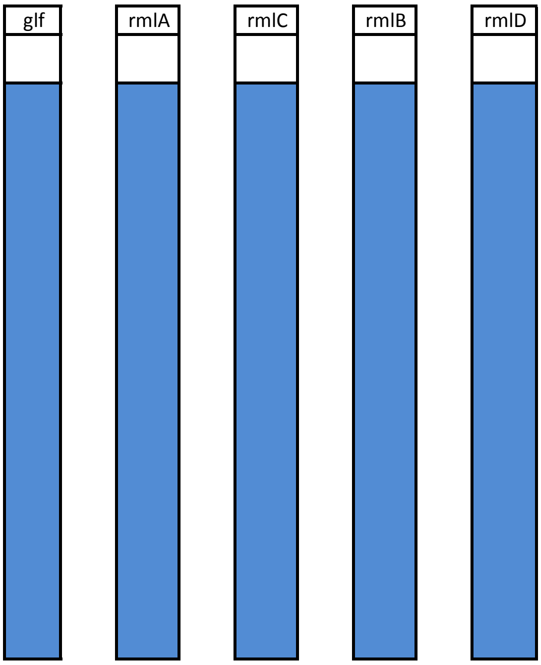
glf
rmlA
rmlC
rmlB
rmlD

SEROTYPE 22F ALL ISOLATES

					6	161	162	255	265	292
		22F reference			S	I	D	R	M	V
32901		SMRU41	Sing6709	22F	G	V	N	Q	T	I
32986		SMRU1108	Sing6709	22F	G	V	N	Q	T	I
82509		SMRU2268	1294/4522	22F	G	V	N	Q	T	I
83211		SMRU119	Sing6709	22F	G	V	N	Q	T	I
83284		SMRU1055	Sing6709	22F	G	V	N	Q	T	I
83322		SMRU1532	Sing6709	22F	G	V	N	Q	T	I
83503		SMRU2368	1294/4522	22F	G	V	N	Q	T	I
109772		SMRU1211	Sing6709	22F	G	V	N	Q	T	I
109821		SMRU1141	Sing6709	22F	G	V	N	Q	T	I
109949		SMRU2707	Sing6709	22F	G	V	N	Q	T	I
113734		SMRU108	Sing6709	22F	G	V	N	Q	T	I
115589		SMRU2670	Sing6709	22F	G	V	N	Q	T	I
115610		SMRU3020	1294/4522	22F	G	V	N	Q	T	I
N/A	507	VICE0186	433	22F	S	I	D	R	T	V
N/A	528	VICE0207	433	22F	S	I	D	R	T	V
N/A	537	VICE0216	433	22F	S	I	D	R	T	V
N/A	564	VICE0243	433	22F	S	I	D	R	T	V
N/A	566	VICE0245	433	22F	S	I	D	R	T	V
N/A	586	VICE0264	433	22F	S	I	D	R	T	V
N/A	587	VICE0265	433	22F	S	I	D	R	T	V
N/A	599	VICE0277	433	22F	S	I	D	R	T	V
N/A	803	VICE0481	433	22F	S	I	D	R	T	V
N/A	932	VICE0610	433	22F	S	I	D	R	T	V
N/A	1267	VICE0673	433	22F	S	I	D	R	T	V
N/A	1304	VICE0710	433	22F	S	I	D	R	T	V
N/A	1328	VICE0734	433	22F	S	I	D	R	T	V
N/A	1329	VICE0735	433	22F	S	I	D	R	T	V
N/A	1335	VICE0741	433	22F	S	I	D	R	T	V
N/A	1354	VICE0760	433	22F	S	I	D	R	T	V
N/A	1398	VICE0804	433	22F	S	I	D	R	T	V
N/A	1412	VICE0818	433	22F	S	I	D	R	T	V
N/A	1433	VICE0839	433	22F	S	I	D	R	T	V
N/A	1562	VICE1011a	433	22F	S	I	D	R	T	V
N/A	1570	VICE1019	433	22F	S	I	D	R	T	V
N/A	2252	VICE1374	433	22F	S	I	D	R	T	V
N/A	2309	VICE1431	433	22F	S	I	D	R	T	V
N/A	2310	VICE1432	433	22F	S	I	D	R	T	V
N/A	2410	VICE1532	433	22F	S	I	D	R	T	V
N/A	2411	VICE1533	433	22F	S	I	D	R	T	V
N/A	2412	VICE1534	433	22F	S	I	D	R	T	V
N/A	2413	VICE1535	433	22F	S	I	D	R	T	V
N/A	2433	VICE1555	433	22F	S	I	D	R	T	V
N/A	2437	VICE1559	433	22F	S	I	D	R	T	V
N/A	2438	VICE1560	433	22F	S	I	D	R	T	V
N/A	2443	VICE1565	433	22F	S	I	D	R	T	V
N/A	2544	VICE1591	433	22F	S	I	D	R	T	V
N/A	2554	VICE1601	433	22F	S	I	D	R	T	V
N/A	2629	VICE1676	433	22F	S	I	D	R	T	V
N/A	2670	VICE1716	433	22F	S	I	D	R	T	V
N/A	2671	VICE1717	433	22F	S	I	D	R	T	V
N/A	2672	VICE1718	433	22F	S	I	D	R	T	V
N/A	2684	VICE1729	433	22F	S	I	D	R	T	V
N/A	2686	VICE1731	433	22F	S	I	D	R	T	V
N/A	2697	VICE1742	433	22F	S	I	D	R	T	V
N/A	2698	VICE1743	433	22F	S	I	D	R	T	V
N/A	2715	VICE1759b	433	22F	S	I	D	R	T	V
N/A	2753	VICE1793	433	22F	S	I	D	R	T	V
N/A	2764	VICE1803	433	22F	S	I	D	R	T	V
N/A	2765	VICE1804	433	22F	S	I	D	R	T	V
N/A	2775	VICE1814	433	22F	S	I	D	R	T	V
N/A	2776	VICE1815	433	22F	S	I	D	R	T	V
N/A	2824	VICE1859	433	22F	S	I	D	R	T	V

N/A	2878	VICE1906	433	22F	S	I	D	R	T	V
N/A	2958	22F/2	Sing7219	22F	S	I	D	R	M	V
31310	N/A	R34-3017	433	22F	S	I	D	R	T	V
31324	N/A	R34-3167	433	22F	S	I	D	R	T	V
31345	N/A	R34-3091	433	22F	S	I	D	R	T	V
31467	N/A	385385	433	22F	S	I	D	R	T	V
31504	N/A	PT8116	433	22F	S	I	D	R	T	V
31566	N/A	ND6047	433	22F	S	I	D	R	T	V
32058	N/A	R34-3012	433	22F	S	I	D	R	T	V
32080	N/A	R34-3082	433	22F	S	I	D	R	T	V
32092	N/A	R34-3143	433	22F	S	I	D	R	T	V
32138	N/A	R34-3187	433	22F	S	I	D	R	T	V
32140	N/A	R34-3189	433	22F	S	I	D	R	T	V
32173	N/A	35100	433	22F	S	I	D	R	T	V
32201	N/A	163286	433	22F	S	I	D	R	T	V
32333	N/A	NP7013	433	22F	S	I	D	R	T	V
32421	N/A	CH2108	433	22F	S	I	D	R	T	V
32464	N/A	LE4118	433	22F	S	I	D	R	T	V
33267	N/A	120581	433	22F	S	I	D	R	T	V
33271	N/A	153408	433	22F	S	I	D	R	T	V
33403	N/A	NP7062	433	22F	S	I	D	R	T	V
33427	N/A	PT8085	433	22F	S	I	D	R	T	V
40138	N/A	UoS4008	698	22F	S	I	D	R	T	V
40141	N/A	UoS4252	698	22F	S	I	D	R	T	V
115837	N/A	UoS2199	698	22F	S	I	D	R	T	V
115845	N/A	UoS3298	698	22F	S	I	D	R	T	V
115846	N/A	UoS3326	433	22F	S	I	D	R	T	V
115848	N/A	UoS4157	433	22F	S	I	D	R	T	V
115849	N/A	UoS4168	698	22F	S	I	D	R	T	V
121361	N/A	UoS0174	698	22F	S	I	D	R	T	V
121363	N/A	UoS2254	698	22F	S	I	D	R	T	V
121381	N/A	UoS4318	433	22F	S	I	D	R	T	V
121383	N/A	UoS3160	433	22F	S	I	D	R	T	V
121408	N/A	UoS2014	433	22F	S	I	D	R	T	V
121413	N/A	UoS4169	433	22F	S	I	D	R	T	V
121434	N/A	UoS3032	433	22F	S	I	D	R	T	V
121435	N/A	UoS5186	433	22F	S	I	D	R	T	V
121442	N/A	UoS3108	433	22F	S	I	D	R	T	V
121450	N/A	UoS3016	433	22F	S	I	D	R	T	V
121451	N/A	UoS4036	433	22F	S	I	D	R	T	V
121452	N/A	UoS5027	433	22F	S	I	D	R	T	V
121453	N/A	UoS3109	433	22F	S	I	D	R	T	V
121457	N/A	UoS2306	433	22F	S	I	D	R	T	V
121507	N/A	UoS3226	433	22F	S	I	D	R	T	V
121559	N/A	UoS4208	433	22F	S	I	D	R	T	V
123202	N/A	UoS4356	433	22F	S	I	D	R	T	V
123219	N/A	UoS3093	698	22F	S	I	D	R	T	V
123238	N/A	UoS5248	698	22F	S	I	D	R	T	V
123307	N/A	UoS2196	698	22F	S	I	D	R	T	V

SEROTYPE 22F/33A HYBRID SEQUENCE

					9	80	161	162	255	265
		22F reference			S	V	I	D	R	M
		33A reference			R	L	V	N	R	T
32901	N/A	SMRU41	Sing6709	22F	G	V	V	N	Q	T
32986	N/A	SMRU1108	Sing6709	22F	G	V	V	N	Q	T
82509	N/A	SMRU2268	1294/4522	22F	G	V	V	N	Q	T
83211	N/A	SMRU119	Sing6709	22F	G	V	V	N	Q	T
83284	N/A	SMRU1055	Sing6709	22F	G	V	V	N	Q	T
83322	N/A	SMRU1532	Sing6709	22F	G	V	V	N	Q	T
83503	N/A	SMRU2368	1294/4522	22F	G	V	V	N	Q	T
109772	N/A	SMRU1211	Sing6709	22F	G	V	V	N	Q	T

109821	N/A	SMRU1141	Sing6709	22F	G	V	V	N	Q	T
109949	N/A	SMRU2707	Sing6709	22F	G	V	V	N	Q	T
113734	N/A	SMRU108	Sing6709	22F	G	V	V	N	Q	T
115589	N/A	SMRU2670	Sing6709	22F	G	V	V	N	Q	T
115610	N/A	SMRU3020	1294/4522	22F	G	V	V	N	Q	T

SEROTYPE 22F ISOLATES (NO HYBRID SEQUENCES)

					wzg	wzh	wzd	
					265	169	41	93
		22F reference			M	K	S	L
N/A	507	VICE0186	433	22F	T	K	S	L
N/A	528	VICE0207	433	22F	T	K	S	L
N/A	537	VICE0216	433	22F	T	K	S	L
N/A	564	VICE0243	433	22F	T	K	S	L
N/A	566	VICE0245	433	22F	T	K	S	L
N/A	586	VICE0264	433	22F	T	K	S	L
N/A	587	VICE0265	433	22F	T	K	S	L
N/A	599	VICE0277	433	22F	T	K	S	L
N/A	803	VICE0481	433	22F	T	K	S	L
N/A	932	VICE0610	433	22F	T	K	S	L
N/A	1267	VICE0673	433	22F	T	K	S	F
N/A	1304	VICE0710	433	22F	T	K	S	L
N/A	1328	VICE0734	433	22F	T	K	S	L
N/A	1329	VICE0735	433	22F	T	K	S	L
N/A	1335	VICE0741	433	22F	T	K	S	L
N/A	1354	VICE0760	433	22F	T	K	S	L
N/A	1398	VICE0804	433	22F	T	K	S	L
N/A	1412	VICE0818	433	22F	T	K	I	L
N/A	1433	VICE0839	433	22F	T	K	S	L
N/A	1562	VICE1011a	433	22F	T	K	S	L
N/A	1570	VICE1019	433	22F	T	K	S	L
N/A	2252	VICE1374	433	22F	T	K	S	L
N/A	2309	VICE1431	433	22F	T	K	S	L
N/A	2310	VICE1432	433	22F	T	K	S	L
N/A	2410	VICE1532	433	22F	T	K	S	L
N/A	2411	VICE1533	433	22F	T	K	S	L
N/A	2412	VICE1534	433	22F	T	K	S	L
N/A	2413	VICE1535	433	22F	T	K	S	L
N/A	2433	VICE1555	433	22F	T	K	S	L
N/A	2437	VICE1559	433	22F	T	K	S	L
N/A	2438	VICE1560	433	22F	T	K	S	L
N/A	2443	VICE1565	433	22F	T	K	S	L
N/A	2544	VICE1591	433	22F	T	K	S	L
N/A	2554	VICE1601	433	22F	T	K	S	L
N/A	2629	VICE1676	433	22F	T	K	S	L
N/A	2670	VICE1716	433	22F	T	K	S	L
N/A	2671	VICE1717	433	22F	T	K	S	L
N/A	2672	VICE1718	433	22F	T	K	S	L
N/A	2684	VICE1729	433	22F	T	K	S	L
N/A	2686	VICE1731	433	22F	T	K	S	L
N/A	2697	VICE1742	433	22F	T	K	S	L
N/A	2698	VICE1743	433	22F	T	K	S	L
N/A	2715	VICE1759b	433	22F	T	K	S	L
N/A	2753	VICE1793	433	22F	T	K	S	L
N/A	2764	VICE1803	433	22F	T	K	S	L
N/A	2765	VICE1804	433	22F	T	K	S	L
N/A	2775	VICE1814	433	22F	T	K	S	L
N/A	2776	VICE1815	433	22F	T	K	S	L
N/A	2824	VICE1859	433	22F	T	K	S	L

N/A	2878	VICE1906	433	22F	T	K	S	L
N/A	2958	22F/2	Sing7219	22F	M	K	S	L
31310	N/A	R34-3017	433	22F	T	K	S	L
31324	N/A	R34-3167	433	22F	T	K	S	L
31345	N/A	R34-3091	433	22F	T	N	S	L
31467	N/A	385385	433	22F	T	K	S	L
31504	N/A	PT8116	433	22F	T	K	S	L
31566	N/A	ND6047	433	22F	T	K	S	L
32058	N/A	R34-3012	433	22F	T	K	S	L
32080	N/A	R34-3082	433	22F	T	K	S	L
32092	N/A	R34-3143	433	22F	T	K	S	L
32138	N/A	R34-3187	433	22F	T	K	S	L
32140	N/A	R34-3189	433	22F	T	K	S	L
32173	N/A	35100	433	22F	T	K	S	L
32201	N/A	163286	433	22F	T	K	S	L
32333	N/A	NP7013	433	22F	T	K	S	L
32421	N/A	CH2108	433	22F	T	K	S	L
32464	N/A	LE4118	433	22F	T	K	S	L
33267	N/A	120581	433	22F	T	K	S	L
33271	N/A	153408	433	22F	T	K	S	L
33403	N/A	NP7062	433	22F	T	K	S	L
33427	N/A	PT8085	433	22F	T	K	S	L
115846	N/A	UoS3326	433	22F	T	K	S	L
115848	N/A	UoS4157	433	22F	T	K	S	L
121381	N/A	UoS4318	433	22F	T	K	S	L
121383	N/A	UoS3160	433	22F	T	K	S	L
121408	N/A	UoS2014	433	22F	T	K	S	L
121413	N/A	UoS4169	433	22F	T	K	S	L
121434	N/A	UoS3032	433	22F	T	K	S	L
121435	N/A	UoS5186	433	22F	T	K	S	L
121442	N/A	UoS3108	433	22F	T	K	S	L
121450	N/A	UoS3016	433	22F	T	K	S	L
121451	N/A	UoS4036	433	22F	T	K	S	L
121452	N/A	UoS5027	433	22F	T	K	S	L
121453	N/A	UoS3109	433	22F	T	K	S	L
121457	N/A	UoS2306	433	22F	T	K	S	L
121507	N/A	UoS3226	433	22F	T	K	S	L
121559	N/A	UoS4208	433	22F	T	K	S	L
123202	N/A	UoS4356	433	22F	T	K	S	L
40138	N/A	UoS4008	698	22F	T	K	S	L
40141	N/A	UoS4252	698	22F	T	K	S	L
115837	N/A	UoS2199	698	22F	T	K	S	L
115845	N/A	UoS3298	698	22F	T	K	S	L
115849	N/A	UoS4168	698	22F	T	K	S	L
121361	N/A	UoS0174	698	22F	T	K	S	L
121363	N/A	UoS2254	698	22F	T	K	S	L
123219	N/A	UoS3093	698	22F	T	K	S	L
123238	N/A	UoS5248	698	22F	T	K	S	L
123307	N/A	UoS2196	698	22F	T	K	S	L

wzg										wzh				wzd					
321	328	331	335	337	346	348	357	411	461	27	36	156	169	41	75	93	104	106	148
M	V	H	E	S	F	V	Q	Q	L	A	I	M	K	S	V	L	M	T	T
L	I	Y	G	T	Y	A	Q	Q	M	T	T	R	K	S	A	L	I	A	A
L	I	Y	G	T	Y	A	Q	Q	M	T	T	R	K	S	A	L	I	A	A
L	I	Y	G	T	Y	A	R	Q	M	T	T	R	K	S	A	L	I	A	A
L	I	Y	G	T	Y	A	Q	Q	M	T	T	R	K	S	A	L	I	A	A
L	I	Y	G	T	Y	A	Q	Q	M	T	T	R	K	S	A	L	I	A	A
L	I	Y	G	T	Y	A	Q	Q	M	T	T	R	K	S	A	L	I	A	A
L	I	Y	G	T	Y	A	R	Q	M	T	T	R	K	S	A	L	I	A	A
L	I	Y	G	T	Y	A	Q	Q	M	T	T	R	K	S	A	L	I	A	A
L	I	Y	G	T	Y	A	Q	Q	M	T	T	R	K	S	A	L	I	A	A
L	I	Y	G	T	Y	A	Q	Q	M	T	T	R	K	S	A	L	I	A	A
L	I	Y	G	T	Y	A	Q	Q	M	T	T	R	K	S	A	L	I	A	A
L	I	Y	G	T	Y	A	Q	Q	M	T	T	R	K	S	A	L	I	A	A
L	I	Y	G	T	Y	A	R	Q	M	T	T	R	K	S	A	L	I	A	A
M	V	H	E	S	F	V	Q	Q	L	A	I	M	K	S	A	L	M	T	A
M	V	H	E	S	F	V	Q	Q	L	A	I	M	K	S	A	L	M	T	A
M	V	H	E	S	F	V	Q	Q	L	A	I	M	K	S	A	L	M	T	A
M	V	H	E	S	F	V	Q	Q	L	A	I	M	K	S	A	L	M	T	A
M	V	H	E	S	F	V	Q	Q	L	A	I	M	K	S	A	L	M	T	A
M	V	H	E	S	F	V	Q	Q	L	A	I	M	K	S	A	L	M	T	A
M	V	H	E	S	F	V	Q	Q	L	A	I	M	K	S	A	L	M	T	A
M	V	H	E	S	F	V	Q	Q	L	A	I	M	K	S	A	L	M	T	A
M	V	H	E	S	F	V	Q	Q	L	A	I	M	K	S	A	L	M	T	A
M	V	H	E	S	F	V	Q	Q	L	A	I	M	K	S	A	L	M	T	A
M	V	H	E	S	F	V	Q	Q	L	A	I	M	K	S	A	F	M	T	A
M	V	H	E	S	F	V	Q	Q	L	A	I	M	K	S	A	L	M	T	A
M	V	H	E	S	F	V	Q	Q	L	A	I	M	K	S	A	L	M	T	A
M	V	H	E	S	F	V	Q	Q	L	A	I	M	K	S	A	L	M	T	A
M	V	H	E	S	F	V	Q	Q	L	A	I	M	K	S	A	L	M	T	A
M	V	H	E	S	F	V	Q	Q	L	A	I	M	K	S	A	L	M	T	A
M	V	H	E	S	F	V	Q	Q	L	A	I	M	K	S	A	L	M	T	A
M	V	H	E	S	F	V	Q	Q	L	A	I	M	K	I	A	L	M	T	A
M	V	H	E	S	F	V	Q	Q	L	A	I	M	K	S	A	L	M	T	A
M	V	H	E	S	F	V	Q	Q	L	A	I	M	K	S	A	L	M	T	A
M	V	H	E	S	F	V	Q	Q	L	A	I	M	K	S	A	L	M	T	A
M	V	H	E	S	F	V	Q	Q	L	A	I	M	K	S	A	L	M	T	A
M	V	H	E	S	F	V	Q	Q	L	A	I	M	K	S	A	L	M	T	A
M	V	H	E	S	F	V	Q	Q	L	A	I	M	K	S	A	L	M	T	A
M	V	H	E	S	F	V	Q	Q	L	A	I	M	K	S	A	L	M	T	A
M	V	H	E	S	F	V	Q	Q	L	A	I	M	K	S	A	L	M	T	A
M	V	H	E	S	F	V	Q	Q	L	A	I	M	K	S	A	L	M	T	A
M	V	H	E	S	F	V	Q	Q	L	A	I	M	K	S	A	L	M	T	A
M	V	H	E	S	F	V	Q	Q	L	A	I	M	K	S	A	L	M	T	A
M	V	H	E	S	F	V	Q	Q	L	A	I	M	K	S	A	L	M	T	A
M	V	H	E	S	F	V	Q	Q	L	A	I	M	K	S	A	L	M	T	A
M	V	H	E	S	F	V	Q	Q	L	A	I	M	K	S	A	L	M	T	A
M	V	H	E	S	F	V	Q	Q	L	A	I	M	K	S	A	L	M	T	A
M	V	H	E	S	F	V	Q	Q	L	A	I	M	K	S	A	L	M	T	A
M	V	H	E	S	F	V	Q	Q	L	A	I	M	K	S	A	L	M	T	A
M	V	H	E	S	F	V	Q	Q	L	A	I	M	K	S	A	L	M	T	A
M	V	H	E	S	F	V	Q	Q	L	A	I	M	K	S	A	L	M	T	A
M	V	H	E	S	F	V	Q	Q	L	A	I	M	K	S	A	L	M	T	A
M	V	H	E	S	F	V	Q	Q	L	A	I	M	K	S	A	L	M	T	A
M	V	H	E	S	F	V	Q	Q	L	A	I	M	K	S	A	L	M	T	A
M	V	H	E	S	F	V	Q	Q	L	A	I	M	K	S	A	L	M	T	A
M	V	H	E	S	F	V	Q	Q	L	A	I	M	K	S	A	L	M	T	A
M	V	H	E	S	F	V	Q	Q	L	A	I	M	K	S	A	L	M	T	A
M	V	H	E	S	F	V	Q	Q	L	A	I	M	K	S	A	L	M	T	A
M	V	H	E	S	F	V	Q	Q	L	A	I	M	K	S	A	L	M	T	A
M	V	H	E	S	F	V	Q	Q	L	A	I	M	K	S	A	L	M	T	A
M	V	H	E	S	F	V	Q	Q	L	A	I	M	K	S	A	L	M	T	A
M	V	H	E	S	F	V	Q	Q	L	A	I	M	K	S	A	L	M	T	A
M	V	H	E	S	F	V	Q	Q	L	A	I	M	K	S	A	L	M	T	A

M	V	H	E	S	F	V	Q	Q	L	A	I	M	K	S	A	L	M	T	A
M	V	H	E	S	F	V	Q	Q	L	A	I	M	K	S	V	L	M	T	T
M	V	H	E	S	F	V	Q	Q	L	A	I	M	K	S	A	L	M	T	A
M	V	H	E	S	F	V	Q	Q	L	A	I	M	K	S	A	L	M	T	A
M	V	H	E	S	F	V	Q	Q	L	A	I	M	N	S	A	L	M	T	A
M	V	H	E	S	F	V	Q	Q	L	A	I	M	K	S	A	L	M	T	A
M	V	H	E	S	F	V	Q	R	L	A	I	M	K	S	A	L	M	T	A
M	V	H	E	S	F	V	Q	Q	L	A	I	M	K	S	A	L	M	T	A
M	V	H	E	S	F	V	Q	Q	L	A	I	M	K	S	A	L	M	T	A
M	V	H	E	S	F	V	Q	Q	L	A	I	M	K	S	A	L	M	T	A
M	V	H	E	S	F	V	Q	Q	L	A	I	M	K	S	A	L	M	T	A
M	V	H	E	S	F	V	Q	Q	L	A	I	M	K	S	A	L	M	T	A
M	V	H	E	S	F	V	Q	Q	L	A	I	M	K	S	A	L	M	T	A
M	V	H	E	S	F	V	Q	Q	L	A	I	M	K	S	A	L	M	T	A
M	V	H	E	S	F	V	Q	Q	L	A	I	M	K	S	A	L	M	T	A
M	V	H	E	S	F	V	Q	Q	L	A	I	M	K	S	A	L	M	T	A
M	V	H	E	S	F	V	Q	Q	L	A	I	M	K	S	A	L	M	T	A
M	V	H	E	S	F	V	Q	Q	L	A	I	M	K	S	A	L	M	T	A
M	V	H	E	S	F	V	Q	Q	L	A	I	M	K	S	A	L	M	T	A
M	V	H	E	S	F	V	Q	Q	L	A	I	M	K	S	A	L	M	T	A
M	V	H	E	S	F	V	Q	Q	L	A	I	M	K	S	A	L	M	T	A
M	V	H	E	S	F	V	Q	Q	L	A	I	M	K	S	A	L	M	T	A
M	V	H	E	S	F	V	Q	Q	L	A	I	M	K	S	A	L	M	T	A
M	V	H	E	S	F	V	Q	Q	L	A	I	M	K	S	A	L	M	T	A
M	V	H	E	S	F	V	Q	Q	L	A	I	M	K	S	A	L	M	T	A
M	V	H	E	S	F	V	Q	Q	L	A	I	M	K	S	A	L	M	T	A
M	V	H	E	S	F	V	Q	Q	L	A	I	M	K	S	A	L	M	T	A
M	V	H	E	S	F	V	Q	Q	L	A	I	M	K	S	A	L	M	T	A
M	V	H	E	S	F	V	Q	Q	L	A	I	M	K	S	A	L	M	T	A
M	V	H	E	S	F	V	Q	Q	L	A	I	M	K	S	A	L	M	T	A
M	V	H	E	S	F	V	Q	Q	L	A	I	M	K	S	A	L	M	T	A
M	V	H	E	S	F	V	Q	Q	L	A	I	M	K	S	A	L	M	T	A
M	V	H	E	S	F	V	Q	Q	L	A	I	M	K	S	A	L	M	T	A
M	V	H	E	S	F	V	Q	Q	L	A	I	M	K	S	A	L	M	T	A
M	V	H	E	S	F	V	Q	Q	L	A	I	M	K	S	A	L	M	T	A
M	V	H	E	S	F	V	Q	Q	L	A	I	M	K	S	A	L	M	T	A
M	V	H	E	S	F	V	Q	Q	L	A	I	M	K	S	A	L	M	T	A
M	V	H	E	S	F	V	Q	Q	L	A	I	M	K	S	A	L	M	T	A
M	V	H	E	S	F	V	Q	Q	L	A	I	M	K	S	A	L	M	T	A
M	V	H	E	S	F	V	Q	Q	L	A	I	M	K	S	A	L	M	T	A
M	V	H	E	S	F	V	Q	Q	L	A	I	M	K	S	A	L	M	T	A
M	V	H	E	S	F	V	Q	Q	L	A	I	M	K	S	A	L	M	T	A
M	V	H	E	S	F	V	Q	Q	L	A	I	M	K	S	A	L	M	T	A
M	V	H	E	S	F	V	Q	Q	L	A	I	M	K	S	A	L	M	T	A
M	V	H	E	S	F	V	Q	Q	L	A	I	M	K	S	A	L	M	T	A
M	V	H	E	S	F	V	Q	Q	L	A	I	M	K	S	A	L	M	T	A
M	V	H	E	S	F	V	Q	Q	L	A	I	M	K	S	A	L	M	T	A
M	V	H	E	S	F	V	Q	Q	L	A	I	M	K	S	A	L	M	T	A
M	V	H	E	S	F	V	Q	Q	L	A	I	M	K	S	A	L	M	T	A

wzg										wzh									
292	321	328	331	335	337	346	348	357	461	27	36	156	195	61	75	91	92	104	106
V	M	V	H	E	S	F	V	Q	L	A	I	M	V	D	V	N	V	M	T
V	L	I	Y	E	T	Y	A	Q	M	T	T	R	I	N	A	D	A	M	A
I	L	I	Y	G	T	Y	A	Q	M	T	T	R	V	D	A	N	V	I	A
I	L	I	Y	G	T	Y	A	Q	M	T	T	R	V	D	A	N	V	I	A
I	L	I	Y	G	T	Y	A	R	M	T	T	R	V	D	A	N	V	I	A
I	L	I	Y	G	T	Y	A	Q	M	T	T	R	V	D	A	N	V	I	A
I	L	I	Y	G	T	Y	A	Q	M	T	T	R	V	D	A	N	V	I	A
I	L	I	Y	G	T	Y	A	Q	M	T	T	R	V	D	A	N	V	I	A
I	L	I	Y	G	T	Y	A	R	M	T	T	R	V	D	A	N	V	I	A
I	L	I	Y	G	T	Y	A	Q	M	T	T	R	V	D	A	N	V	I	A

I	L	I	Y	G	T	Y	A	Q	M	T	T	R	V	D	A	N	V	I	A
I	L	I	Y	G	T	Y	A	Q	M	T	T	R	V	D	A	N	V	I	A
I	L	I	Y	G	T	Y	A	Q	M	T	T	R	V	D	A	N	V	I	A
I	L	I	Y	G	T	Y	A	Q	M	T	T	R	V	D	A	N	V	I	A
I	L	I	Y	G	T	Y	A	R	M	T	T	R	V	D	A	N	V	I	A

	wze					wchA			wchF	wcwA	wcwC	ugd	wcwV	
148	74	119	155	217	222	142	212	427		167			165	248
T	I	T	P	S	G	R	S	A		A			F	N
A	I	A	P	S	G	R	S	A		A			F	N
A	I	A	P	S	G	R	S	A		A			F	N
A	I	A	P	S	G	R	S	A		A			F	N
A	I	A	P	S	G	R	S	A		A			F	N
A	I	A	P	S	G	R	S	A		A			F	N
A	I	A	P	S	G	R	S	A		A			F	N
A	I	A	P	S	G	R	S	A		A			F	N
A	I	A	P	S	G	R	S	A		A			F	N
A	I	A	P	S	G	R	S	A		A			F	N
A	I	A	P	S	G	R	S	A		A			F	N
A	I	A	P	S	G	R	S	A		A			F	N
A	I	A	P	S	G	R	S	A		A			F	N
A	I	A	P	S	G	R	S	A		A			F	N
A	I	A	P	S	R	R	S	A		A			F	N
A	I	A	P	S	G	R	S	A		A			F	N
A	I	A	T	S	G	R	S	A		A			F	N
A	I	A	P	S	G	R	S	A		A			F	N
A	I	A	P	F	G	R	S	A		A			F	N
A	I	A	P	S	G	R	S	A		A			F	N
A	I	A	P	S	G	R	S	A		A			F	N
A	I	A	P	S	G	R	S	A		A			F	N
A	I	A	P	S	G	R	S	A		A			F	N
A	I	A	P	S	G	R	S	A		A			F	N
A	I	A	P	S	G	R	S	A		A			F	N
A	I	A	P	S	G	R	S	A		A			F	N
A	I	A	P	S	G	R	S	A		A			F	N
A	I	A	P	S	G	R	S	A		A			F	N
A	I	A	P	S	G	R	S	A		A			F	N
A	I	A	P	S	G	R	S	A		A			F	N
A	I	A	P	S	G	R	S	A		A			F	N
A	I	A	P	S	G	R	S	A		A			F	N
A	I	A	P	S	G	R	S	A		A			F	N
A	I	A	P	S	G	R	S	A		A			F	N
A	I	A	P	S	G	R	S	A		A			F	N
A	I	A	P	S	G	R	S	A		A			F	N
A	I	A	P	S	G	R	S	A		A			F	N
A	I	A	P	S	G	R	S	A		A			F	N
A	I	A	P	S	G	R	S	A		A			F	N
A	I	A	P	S	G	R	S	A		A			F	N
A	I	A	P	S	G	R	S	A		A			F	N
A	I	A	P	S	G	R	S	A		A			F	N
A	I	A	P	S	G	R	S	A		A			F	N
A	I	A	P	S	G	R	S	A		A			F	N
A	I	A	P	S	G	R	Y	A		A			F	N
A	I	A	P	S	G	R	S	A		A			F	N
A	I	A	P	S	G	R	S	A		A			F	N
A	I	A	P	S	G	R	S	A		A			F	N
A	I	A	P	S	G	R	S	A		A			F	N
A	I	A	P	S	G	R	S	A		A			F	N

A	I	A	P	S	G	R	S	A	A	F	N
T	I	T	P	S	G	R	S	A	A	F	N
A	I	A	P	S	G	R	S	A	A	F	N
A	I	A	P	S	G	R	S	A	A	F	N
A	I	A	P	S	G	R	S	A	A	F	N
A	I	A	P	S	G	R	S	A	A	F	N
A	I	A	P	S	G	R	S	A	A	F	N
A	I	A	P	S	G	R	S	A	A	F	N
A	I	A	P	S	G	R	S	A	A	F	N
A	I	A	P	S	G	R	S	A	A	F	N
A	I	A	P	S	G	R	S	A	A	F	N
A	I	A	P	S	G	R	S	A	A	F	N
A	I	A	P	S	G	C	S	V	A	F	N
A	I	A	P	S	G	R	S	A	A	F	N
A	I	A	P	S	G	R	S	A	A	F	N
A	I	A	P	S	G	R	S	A	A	F	N
A	I	A	P	S	G	R	S	A	A	F	N
A	V	A	P	S	G	R	S	A	A	F	N
A	I	A	P	S	G	R	S	A	A	F	N
A	I	A	P	S	G	R	S	A	A	F	N
A	I	A	P	S	G	R	S	A	A	F	N
A	I	A	P	S	G	R	S	A	A	F	N
A	I	A	P	S	G	R	S	A	A	F	N
A	I	A	P	S	G	R	S	A	A	F	N
A	I	A	P	S	G	R	S	A	A	F	N
A	I	A	P	S	G	R	S	A	A	F	N
A	I	A	P	S	G	R	S	A	A	F	N
A	I	A	P	S	G	R	S	A	A	F	N
A	I	A	P	S	G	R	S	A	A	F	N
A	I	A	P	S	G	R	S	A	A	F	N
A	I	A	P	S	G	R	S	A	A	F	N
A	I	A	P	S	G	R	S	A	A	F	N
A	I	A	P	S	G	R	S	A	A	F	N
A	I	A	P	S	G	R	S	A	A	F	N
A	I	A	P	S	G	R	S	A	A	F	N
A	I	A	P	S	G	R	S	A	A	F	N
A	I	A	P	S	G	R	S	A	A	F	N
A	I	A	P	S	G	R	S	A	A	F	N
A	I	A	P	S	G	R	S	A	A	F	N
A	I	A	P	S	G	R	S	A	A	F	S
A	I	A	P	S	G	R	S	A	A	F	S
A	I	A	P	S	G	R	S	A	V	F	S
A	I	A	P	S	G	R	S	A	A	F	S
A	I	A	P	S	G	R	S	A	A	F	S
A	I	A	P	S	G	R	S	A	A	F	S
A	I	A	P	S	G	R	S	A	A	F	S
A	I	A	P	S	G	R	S	A	A	L	S
A	I	A	P	S	G	R	S	A	A	F	S
A	I	A	P	S	G	R	S	A	A	F	S

	wze					wchA								wchF		
228	74	119	155	217	222	19	82	142	175	212	268	278	427	109	274	316
N	I	T	P	S	G	I	S	R	C	S	M	G	A	I	L	A
D	I	A	P	S	G	V	G	R	Y	S	V	S	A	V	P	S
D	I	A	P	S	G	V	G	R	Y	S	V	S	A	V	P	S
D	I	A	P	S	G	V	G	R	Y	S	V	S	A	V	P	S
D	I	A	P	S	G	V	G	R	Y	S	V	S	A	V	P	S
D	I	A	P	S	G	V	G	R	Y	S	V	S	A	V	P	S
D	I	A	P	S	G	V	G	R	Y	S	V	S	A	V	P	S
D	I	A	P	S	G	V	G	R	Y	S	V	S	A	V	P	S
D	I	A	P	S	G	V	G	R	Y	S	V	S	A	V	P	S
D	I	A	P	S	G	V	G	R	Y	S	V	S	A	V	P	S
D	I	A	P	S	G	V	G	R	Y	S	V	S	A	V	P	S
D	I	A	P	S	G	V	G	R	Y	S	V	S	A	V	P	S
D	I	A	P	S	G	V	G	R	Y	S	V	S	A	V	P	S
D	I	A	P	S	G	V	G	R	Y	S	V	S	A	V	P	S
N	I	A	P	S	G	I	S	R	C	S	M	G	A	I	L	A
N	I	A	P	S	G	I	S	R	C	S	M	G	A	I	L	A
N	I	A	P	S	G	I	S	R	C	S	M	G	A	I	L	A
N	I	A	P	S	G	I	S	R	C	S	M	G	A	I	L	A
N	I	A	P	S	G	I	S	R	C	S	M	G	A	I	L	A
N	I	A	P	S	G	I	S	R	C	S	M	G	A	I	L	A
N	I	A	P	S	G	I	S	R	C	S	M	G	A	I	L	A
N	I	A	P	S	G	I	S	R	C	S	M	G	A	I	L	A
N	I	A	P	S	G	I	S	R	C	S	M	G	A	I	L	A
N	I	A	P	S	G	I	S	R	C	S	M	G	A	I	L	A
N	I	A	P	S	G	I	S	R	C	S	M	G	A	I	L	A
N	I	A	P	S	G	I	S	R	C	S	M	G	A	I	L	A
N	I	A	P	S	G	I	S	R	C	S	M	G	A	I	L	A
N	I	A	P	S	R	I	S	R	C	S	M	G	A	I	L	A
N	I	A	P	S	G	I	S	R	C	S	M	G	A	I	L	A
N	I	A	T	S	G	I	S	R	C	S	M	G	A	I	L	A
N	I	A	P	S	G	I	S	R	C	S	M	G	A	I	L	A
N	I	A	P	F	G	I	S	R	C	S	M	G	A	I	L	A
N	I	A	P	S	G	I	S	R	C	S	M	G	A	I	L	A
N	I	A	P	S	G	I	S	R	C	S	M	G	A	I	L	A
N	I	A	P	S	G	I	S	R	C	S	M	G	A	I	L	A
N	I	A	P	S	G	I	S	R	C	S	M	G	A	I	L	A
N	I	A	P	S	G	I	S	R	C	S	M	G	A	I	L	A
N	I	A	P	S	G	I	S	R	C	S	M	G	A	I	L	A
N	I	A	P	S	G	I	S	R	C	S	M	G	A	I	L	A
N	I	A	P	S	G	I	S	R	C	S	M	G	A	I	L	A
N	I	A	P	S	G	I	S	R	C	S	M	G	A	I	L	A
N	I	A	P	S	G	I	S	R	C	S	M	G	A	I	L	A
N	I	A	P	S	G	I	S	R	C	S	M	G	A	I	L	A
N	I	A	P	S	G	I	S	R	C	S	M	G	A	I	L	A
N	I	A	P	S	G	I	S	R	C	S	M	G	A	I	L	A
N	I	A	P	S	G	I	S	R	C	S	M	G	A	I	L	A
N	I	A	P	S	G	I	S	R	C	S	M	G	A	I	L	A
N	I	A	P	S	G	I	S	R	C	S	M	G	A	I	L	A
N	I	A	P	S	G	I	S	R	C	S	M	G	A	I	L	A
N	I	A	P	S	G	I	S	R	C	S	M	G	A	I	L	A
N	I	A	P	S	G	I	S	R	C	S	M	G	A	I	L	A
N	I	A	P	S	G	I	S	R	C	S	M	G	A	I	L	A
N	I	A	P	S	G	I	S	R	C	S	M	G	A	I	L	A
N	I	A	P	S	G	I	S	R	C	S	M	G	A	I	L	A
N	I	A	P	S	G	I	S	R	C	S	M	G	A	I	L	A
N	I	A	P	S	G	I	S	R	C	S	M	G	A	I	L	A
N	I	A	P	S	G	I	S	R	C	Y	M	G	A	I	L	A
N	I	A	P	S	G	I	S	R	C	S	M	G	A	I	L	A
N	I	A	P	S	G	I	S	R	C	S	M	G	A	I	L	A
N	I	A	P	S	G	I	S	R	C	S	M	G	A	I	L	A
N	I	A	P	S	G	I	S	R	C	S	M	G	A	I	L	A
N	I	A	P	S	G	I	S	R	C	S	M	G	A	I	L	A

N	I	A	P	S	G	I	S	R	C	S	M	G	A	I	L	A
N	I	T	P	S	G	I	S	R	C	S	M	G	A	I	L	A
N	I	A	P	S	G	I	S	R	C	S	M	G	A	I	L	A
N	I	A	P	S	G	I	S	R	C	S	M	G	A	I	L	A
N	I	A	P	S	G	I	S	R	C	S	M	G	A	I	L	A
N	I	A	P	S	G	I	S	R	C	S	M	G	A	I	L	A
N	I	A	P	S	G	I	S	R	C	S	M	G	A	I	L	A
N	I	A	P	S	G	I	S	R	C	S	M	G	A	I	L	A
N	I	A	P	S	G	I	S	R	C	S	M	G	A	I	L	A
N	I	A	P	S	G	I	S	R	C	S	M	G	A	I	L	A
N	I	A	P	S	G	I	S	R	C	S	M	G	A	I	L	A
N	I	A	P	S	G	I	S	R	C	S	M	G	A	I	L	A
N	I	A	P	S	G	I	S	C	C	S	M	G	V	I	L	A
N	I	A	P	S	G	I	S	R	C	S	M	G	A	I	L	A
N	I	A	P	S	G	I	S	R	C	S	M	G	A	I	L	A
N	I	A	P	S	G	I	S	R	C	S	M	G	A	I	L	A
N	I	A	P	S	G	I	S	R	C	S	M	G	A	I	L	A
N	V	A	P	S	G	I	S	R	C	S	M	G	A	I	L	A
N	I	A	P	S	G	I	S	R	C	S	M	G	A	I	L	A
N	I	A	P	S	G	I	S	R	C	S	M	G	A	I	L	A
N	I	A	P	S	G	I	S	R	C	S	M	G	A	I	L	A
N	I	A	P	S	G	I	S	R	C	S	M	G	A	I	L	A
N	I	A	P	S	G	I	S	R	C	S	M	G	A	I	L	A
N	I	A	P	S	G	I	S	R	C	S	M	G	A	I	L	A
N	I	A	P	S	G	I	S	R	C	S	M	G	A	I	L	A
N	I	A	P	S	G	I	S	R	C	S	M	G	A	I	L	A
N	I	A	P	S	G	I	S	R	C	S	M	G	A	I	L	A
N	I	A	P	S	G	I	S	R	C	S	M	G	A	I	L	A
N	I	A	P	S	G	I	S	R	C	S	M	G	A	I	L	A
N	I	A	P	S	G	I	S	R	C	S	M	G	A	I	L	A
N	I	A	P	S	G	I	S	R	C	S	M	G	A	I	L	A
N	I	A	P	S	G	I	S	R	C	S	M	G	A	I	L	A
N	I	A	P	S	G	I	S	R	C	S	M	G	A	I	L	A
N	I	A	P	S	G	I	S	R	C	S	M	G	A	I	L	A
N	I	A	P	S	G	I	S	R	C	S	M	G	A	I	L	A
N	I	A	P	S	G	I	S	R	C	S	M	G	A	I	L	A
N	I	A	P	S	G	I	S	R	C	S	M	G	A	I	L	A
N	I	A	P	S	G	I	S	R	C	S	M	G	A	I	L	A
N	I	A	P	S	G	I	S	R	C	S	M	G	A	I	L	A
N	I	A	P	S	G	I	S	R	C	S	M	G	A	I	L	A
N	I	A	P	S	G	I	S	R	C	S	M	G	A	I	L	A
N	I	A	P	S	G	I	S	R	C	S	M	G	A	I	L	A
N	I	A	P	S	G	I	S	R	C	S	M	G	A	I	L	A
N	I	A	P	S	G	I	S	R	C	S	M	G	A	I	L	A
N	I	A	P	S	G	I	S	R	C	S	M	G	A	I	L	A
N	I	A	P	S	G	I	S	R	C	S	M	G	A	I	L	A
N	I	A	P	S	G	I	S	R	C	S	M	G	A	I	L	A
N	I	A	P	S	G	I	S	R	C	S	M	G	A	I	L	A
N	I	A	P	S	G	I	S	R	C	S	M	G	A	I	L	A

wzd							
114	119	148	217	223	227	228	230
K	T	T	Q	V	L	N	M
Q	A	A	K	L	F	D	I
K	T	A	Q	V	L	D	M
K	T	A	Q	V	L	D	M
K	T	A	Q	V	L	D	M
K	T	A	Q	V	L	D	M
K	T	A	Q	V	L	D	M
K	T	A	Q	V	L	D	M
K	T	A	Q	V	L	D	M
K	T	A	Q	V	L	D	M

wze											
2	13	34	45	54	62	87	111	119	136	168	178
P	F	D	L	T	H	D	V	T	S	K	V
S	S	G	I	A	R	N	I	A	T	N	A
P	F	D	L	T	H	D	V	A	S	K	V
P	F	D	L	T	H	D	V	A	S	K	V
P	F	D	L	T	H	D	V	A	S	K	V
P	F	D	L	T	H	D	V	A	S	K	V
P	F	D	L	T	H	D	V	A	S	K	V
P	F	D	L	T	H	D	V	A	S	K	V
P	F	D	L	T	H	D	V	A	S	K	V
P	F	D	L	T	H	D	V	A	S	K	V

K	T	A	Q	V	L	D	M
K	T	A	Q	V	L	D	M
K	T	A	Q	V	L	D	M
K	T	A	Q	V	L	D	M
K	T	A	Q	V	L	D	M

P	F	D	L	T	H	D	V	A	S	K	V
P	F	D	L	T	H	D	V	A	S	K	V
P	F	D	L	T	H	D	V	A	S	K	V
P	F	D	L	T	H	D	V	A	S	K	V
P	F	D	L	T	H	D	V	A	S	K	V

whaB	wzy	wcwX	wzx	glf	rmlA	rmlC			rmlB	rmlD
9		37		330		21	51	57		27
H		G		A		M	P	E		V
H		G		A		I	P	E		V
H		G		A		M	P	E		V
H		G		A		M	P	E		A
H		G		A		M	P	E		V
H		G		A		M	P	E		V
H		G		A		M	P	E		V
H		G		A		M	P	E		V
N		G		A		M	P	E		V
H		G		A		M	P	E		V
H		G		A		M	P	E		V
H		G		A		M	P	E		V
H		G		A		M	P	E		V
H		G		A		M	P	E		V
H		G		A		M	P	E		V
H		G		A		M	P	E		V
H		G		A		M	P	E		V
H		G		A		M	P	E		V
H		G		A		M	P	E		V
H		G		V		M	P	E		V
H		G		A		M	P	E		V
H		G		A		M	P	E		V
H		G		A		M	P	E		V
H		G		A		M	P	E		V
H		G		A		M	P	E		V
H		G		A		M	P	E		V
H		G		A		M	P	E		V
H		G		A		M	P	E		V
H		G		A		M	P	E		V
H		G		A		M	P	E		V
H		G		A		M	P	E		V
H		G		A		M	P	E		V
H		G		A		M	P	E		V
H		G		A		M	P	E		V
H		G		A		M	P	E		V
H		G		A		M	P	E		V
H		G		A		M	P	E		V
H		G		A		M	P	E		V
H		G		A		M	P	E		V
H		G		A		M	P	E		V
H		G		A		M	P	E		V
H		G		A		M	P	E		V
H		G		A		M	P	E		V
H		G		A		M	P	E		V
H		G		A		M	P	E		V
H		G		V		M	P	E		V
H		G		V		M	P	E		V
H		G		V		M	P	E		V
H		G		V		M	P	E		V
H		G		A		M	P	E		V

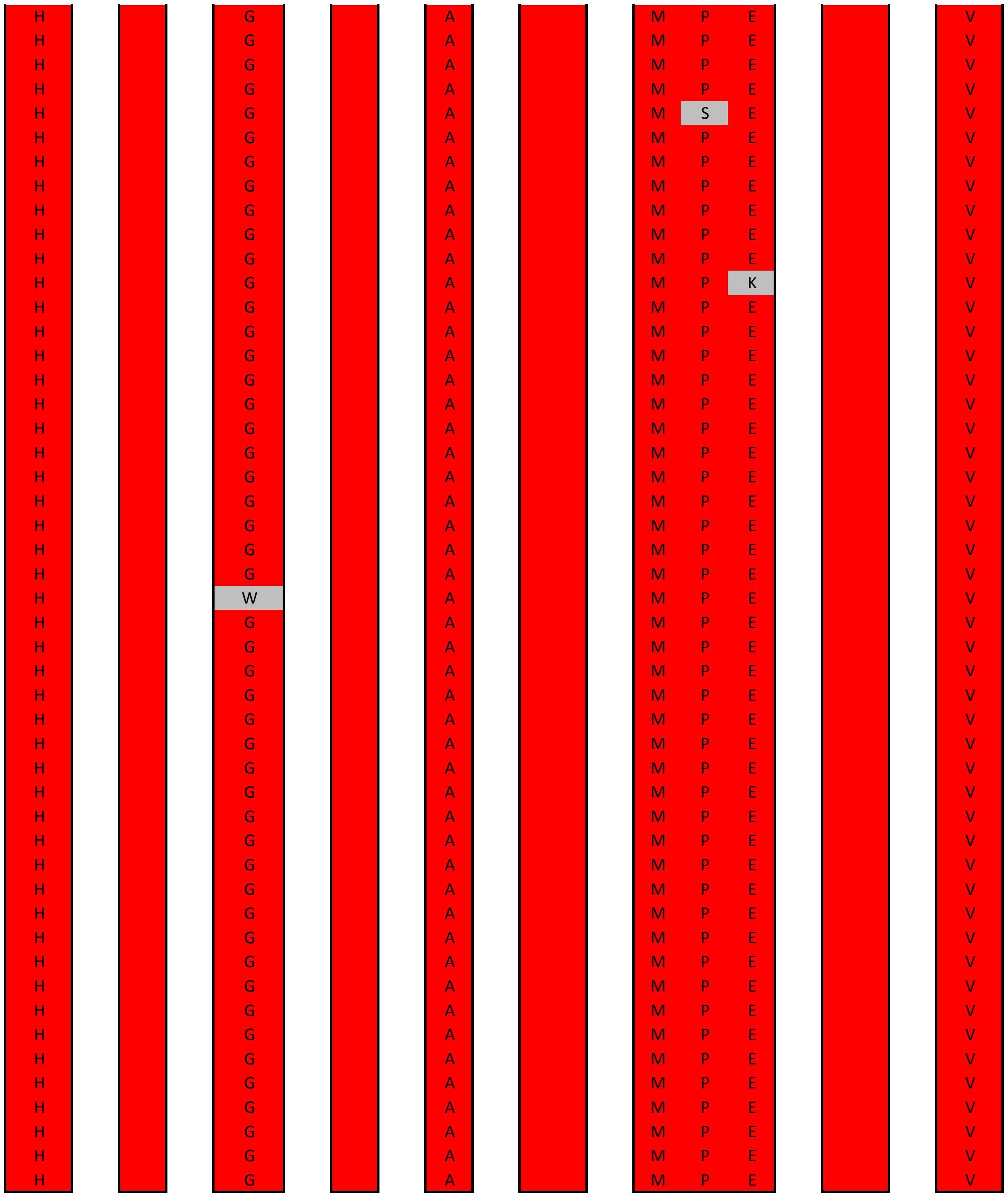
H
G
W
A
M
P
S
E
K
V

wcwA				wcwC	ugd	wcwV			whaB	wzy	wcwX	wzx	g
128	130	131	167	165		165	248	288	9	302	37	77	161
T	F	T	A	V		F	N	F	H	D	G	A	I
T	F	T	A	A		F	N	F	H	G	G	E	T
S	L	K	A	A		F	N	F	H	G	G	E	T
T	F	T	A	A		F	N	F	H	G	G	E	T
T	F	T	A	A		F	N	F	H	G	G	E	T
T	F	T	A	A		F	N	F	H	G	G	E	T
T	F	T	A	A		F	N	F	H	G	G	E	T
T	F	T	A	A		F	N	L	H	G	G	E	T
T	F	T	A	A		F	N	F	H	G	G	E	T
T	F	T	A	A		F	N	F	H	G	G	E	T
T	F	T	A	A		F	N	F	H	G	G	E	T
T	F	T	A	A		F	N	F	H	G	G	E	T
T	F	T	A	A		F	N	F	H	G	G	E	T
T	F	T	A	A		F	N	F	H	G	G	E	T
T	F	T	A	V		F	N	F	H	D	G	A	I
T	F	T	A	V		F	N	F	H	D	G	A	I
T	F	T	A	V		F	N	F	H	D	G	A	I
T	F	T	A	V		F	N	F	H	D	G	A	I
T	F	T	A	V		F	N	F	H	D	G	A	I
T	F	T	A	V		F	N	F	H	D	G	A	I
T	F	T	A	V		F	N	F	H	D	G	A	I
T	F	T	A	V		F	N	F	N	D	G	A	I
T	F	T	A	V		F	N	F	H	D	G	A	I
T	F	T	A	V		F	N	F	H	D	G	A	I
T	F	T	A	V		F	N	F	H	D	G	A	I
T	F	T	A	V		F	N	F	H	D	G	A	I
T	F	T	A	V		F	N	F	H	D	G	A	I
T	F	T	A	V		F	N	F	H	D	G	A	I
T	F	T	A	V		F	N	F	H	D	G	A	I
T	F	T	A	V		F	N	F	H	D	G	A	I
T	F	T	A	V		F	N	F	H	D	G	A	I
T	F	T	A	V		F	N	F	H	D	G	A	I
T	F	T	A	V		F	N	F	H	D	G	A	I
T	F	T	A	V		F	N	F	H	D	G	A	I
T	F	T	A	V		F	N	F	H	D	G	A	I
T	F	T	A	V		F	N	F	H	D	G	A	I
T	F	T	A	V		F	N	F	H	D	G	A	I
T	F	T	A	V		F	N	F	H	D	G	A	I
T	F	T	A	V		F	N	F	H	D	G	A	I
T	F	T	A	V		F	N	F	H	D	G	A	I
T	F	T	A	V		F	N	F	H	D	G	A	I
T	F	T	A	V		F	N	F	H	D	G	A	I
T	F	T	A	V		F	N	F	H	D	G	A	I
T	F	T	A	V		F	N	F	H	D	G	A	I
T	F	T	A	V		F	N	F	H	D	G	A	I
T	F	T	A	V		F	N	F	H	D	G	A	I
T	F	T	A	V		F	N	F	H	D	G	A	I
T	F	T	A	V		F	N	F	H	D	G	A	I
T	F	T	A	V		F	N	F	H	D	G	A	I
T	F	T	A	V		F	N	F	H	D	G	A	I
T	F	T	A	V		F	N	F	H	D	G	A	I
T	F	T	A	V		F	N	F	H	D	G	A	I
T	F	T	A	V		F	N	F	H	D	G	A	I
T	F	T	A	V		F	N	F	H	D	G	A	I
T	F	T	A	V		F	N	F	H	D	G	A	I
T	F	T	A	V		F	N	F	H	D	G	A	I
T	F	T	A	V		F	N	F	H	D	G	A	I
T	F	T	A	V		F	N	F	H	D	G	A	I
T	F	T	A	V		F	N	F	H	D	G	A	I
T	F	T	A	V		F	N	F	H	D	G	A	I
T	F	T	A	V		F	N	F	H	D	G	A	I
T	F	T	A	V		F	N	F	H	D	G	A	I

T	F	T	A	V		F	N	F	H	D	G	A	I
T	F	T	A	V		F	N	F	H	D	G	A	I
T	F	T	A	V		F	N	F	H	D	G	A	I
T	F	T	A	V		F	N	F	H	D	G	A	I
T	F	T	A	V		F	N	F	H	D	G	A	I
T	F	T	A	V		F	N	F	H	D	G	A	I
T	F	T	A	V		F	N	F	H	D	G	A	I
T	F	T	A	V		F	N	F	H	D	G	A	I
T	F	T	A	V		F	N	F	H	D	G	A	I
T	F	T	A	V		F	N	F	H	D	G	A	I
T	F	T	A	V		F	N	F	H	D	G	A	I
T	F	T	A	V		F	N	F	H	D	G	A	I
T	F	T	A	V		F	N	F	H	D	G	A	I
T	F	T	A	V		F	N	F	H	D	G	A	I
T	F	T	A	V		F	N	F	H	D	G	A	I
T	F	T	A	V		F	N	F	H	D	G	A	I
T	F	T	A	V		F	N	F	H	D	G	A	I
T	F	T	A	V		F	N	F	H	D	G	A	I
T	F	T	A	V		F	N	F	H	D	G	A	I
T	F	T	A	V		F	N	F	H	D	G	A	I
T	F	T	A	V		F	N	F	H	D	G	A	I
T	F	T	A	V		F	N	F	H	D	G	A	I
T	F	T	A	V		F	S	F	H	D	G	A	I
T	F	T	A	V		F	S	F	H	D	G	A	I
T	F	T	V	V		F	S	F	H	D	G	A	I
T	F	T	A	V		F	S	F	H	D	G	A	I
T	F	T	A	V		F	N	F	H	D	G	A	I
T	F	T	A	V		F	N	F	H	D	G	A	I
T	F	T	A	V		F	S	F	H	D	G	A	I
T	F	T	A	V		F	S	F	H	D	G	A	I
T	F	T	A	V		F	S	F	H	D	G	A	I
T	F	T	A	V		F	N	F	H	D	W	A	I
T	F	T	A	V		F	N	F	H	D	G	A	I
T	F	T	A	V		F	N	F	H	D	G	A	I
T	F	T	A	V		F	N	F	H	D	G	A	I
T	F	T	A	V		F	N	F	H	D	G	A	I
T	F	T	A	V		F	N	F	H	D	G	A	I
T	F	T	A	V		F	N	F	H	D	G	A	I
T	F	T	A	V		F	N	F	H	D	G	A	I
T	F	T	A	V		F	N	F	H	D	G	A	I
T	F	T	A	V		F	N	F	H	D	G	A	I
T	F	T	A	V		F	N	F	H	D	G	A	I
T	F	T	A	V		F	N	F	H	D	G	A	I
T	F	T	A	V		F	N	F	H	D	G	A	I
T	F	T	A	V		F	N	F	H	D	G	A	I
T	F	T	A	V		F	N	F	H	D	G	A	I
T	F	T	A	V		L	S	F	H	D	G	A	I
T	F	T	A	V		F	S	F	H	D	G	A	I
T	F	T	A	V		F	S	F	H	D	G	A	I

	wchA										
203	8	19	53	82	124	141	175	268	278	352	403
V	S	I	D	S	A	S	C	M	G	C	I
I	P	I	V	S	T	F	C	M	G	R	V
V	S	V	D	G	A	S	Y	V	S	C	I
V	S	V	D	G	A	S	Y	V	S	C	I
V	S	V	D	G	A	S	Y	V	S	C	I
V	S	V	D	G	A	S	Y	V	S	C	I
V	S	V	D	G	A	S	Y	V	S	C	I
V	S	V	D	G	A	S	Y	V	S	C	I
V	S	V	D	G	A	S	Y	V	S	C	I
V	S	V	D	G	A	S	Y	V	S	C	I

V
V
V
V
V

S	V	D	G	A	S	Y	V	S	C	I
S	V	D	G	A	S	Y	V	S	C	I
S	V	D	G	A	S	Y	V	S	C	I
S	V	D	G	A	S	Y	V	S	C	I
S	V	D	G	A	S	Y	V	S	C	I

lf	rmlA	rmlC			rmlB	rmlD
330		21	51	57		27
A		M	P	E		V
A		M	P	E		V
A		M	P	E		V
A		M	P	E		V
A		M	P	E		V
A		M	P	E		V
A		M	P	E		V
A		M	P	E		V
A		M	P	E		V
A		M	P	E		V
A		M	P	E		V
A		M	P	E		V
A		M	P	E		V
A		M	P	E		V
A		I	P	E		V
A		M	P	E		V
A		M	P	E		A
A		M	P	E		V
A		M	P	E		V
A		M	P	E		V
A		M	P	E		V
A		M	P	E		V
A		M	P	E		V
A		M	P	E		V
A		M	P	E		V
A		M	P	E		V
A		M	P	E		V
A		M	P	E		V
A		M	P	E		V
A		M	P	E		V
A		M	P	E		V
A		M	P	E		V
V		M	P	E		V
A		M	P	E		V
A		M	P	E		V
A		M	P	E		V
A		M	P	E		V
A		M	P	E		V
A		M	P	E		V
A		M	P	E		V
A		M	P	E		V
A		M	P	E		V
A		M	P	E		V
A		M	P	E		V
A		M	P	E		V
A		M	P	E		V
A		M	P	E		V
A		M	P	E		V
A		M	P	E		V
A		M	P	E		V
A		M	P	E		V
A		M	P	E		V
A		M	P	E		V
A		M	P	E		V
A		M	P	E		V
A		M	P	E		V
A		M	P	E		V
A		M	P	E		V
V		M	P	E		V
V		M	P	E		V
V		M	P	E		V
V		M	P	E		V
A		M	P	E		V

A		M	P	E		V
A		M	P	E		V
A		M	P	E		V
A		M	P	E		V
A		M	S	E		V
A		M	P	E		V
A		M	P	E		V
A		M	P	E		V
A		M	P	E		V
A		M	P	E		V
A		M	P	E		V
A		M	P	K		V
A		M	P	E		V
A		M	P	E		V
A		M	P	E		V
A		M	P	E		V
A		M	P	E		V
A		M	P	E		V
A		M	P	E		V
A		M	P	E		V
A		M	P	E		V
A		M	P	E		V
A		M	P	E		V
A		M	P	E		V
A		M	P	E		V
A		M	P	E		V
A		M	P	E		V
A		M	P	E		V
A		M	P	E		V
A		M	P	E		V
A		M	P	E		V
A		M	P	E		V
A		M	P	E		V
A		M	P	E		V
A		M	P	E		V
A		M	P	E		V
A		M	P	E		V
A		M	P	E		V
A		M	P	E		V
A		M	P	E		V
A		M	P	E		V
A		M	P	E		V
A		M	P	E		V
A		M	P	E		V
A		M	P	E		V
A		M	P	E		V
A		M	P	E		V
A		M	P	E		V
A		M	P	E		V

rmlst ID	PubMLST id	ENA	Name	ST	CC	Orig_sero
N/A	25262	ERR025271	Finland6B-12	238	273	6B
N/A	25500	ERR025270	ICE11	490	490	6B
N/A	26107	ERR025273	Maryland6B-17	384	384	6B
N/A	25695	ERR025264	Spain6B-2	90	90	6B
N/A	26186	ERR028719	USA22	490	490	6C
N/A	25681	ERR163138	S.Africa6B-8	185	185	6B
N/A	25661	ERR163152	SA11	1094	1094	6A
N/A	25666	ERR163153	SA12	1094	1094	6A
N/A	25658	ERR163154	SA13	1094	1094	6A
N/A	25662	ERR163155	SA14	1094	1094	6A
N/A	25663	ERR163156	SA15	1094	1094	6A
N/A	25667	ERR163157	SA16	1094	1094	6A
N/A	25659	ERR163158	SA17	1094	1094	6A
N/A	25664	ERR163159	SA18	1094	1094	6A
N/A	25665	ERR163160	SA19	1094	1094	6A
N/A	25660	ERR163161	SA20	1094	1094	6A
N/A	25668	ERR163162	SA21	1607	1094	6A
N/A	25686	ERR163169	SA31	1094	1094	6A
N/A	25685	ERR163170	SA32	1094	1094	6A
N/A	25678	ERR163175	SA38	1094	1094	6A
N/A	25684	ERR163178	SA41	1094	1094	6B
N/A	25683	ERR163181	SA44	1094	1094	6A
N/A	25682	ERR163182	SA45	1094	1094	6A
N/A	25680	ERR163186	SA51	185	185	6B
N/A	25671	ERR163187	SA52	2421	2421	6B
N/A	25669	ERR163189	SA54	7084	7084	6B
N/A	25670	ERR163191	SA56	1094	1094	6B
N/A	25672	ERR163192	SA57	185	185	6B
N/A	25674	ERR163194	SA59	7251	1094	6A
N/A	25675	ERR163197	SA62	1094	1094	6B
N/A	25673	ERR163201	SA66	185	185	6B
N/A	25676	ERR163202	SA67	7250	Sing7250	6B
N/A	25677	ERR163203	SA68	7257	1094	6A
N/A	25679	ERR163207	SA72	1094	1094	6A
N/A	26105	ERR163227	USA33	238	273	6B
N/A	26106	ERR163226	USA39	90	90	6B
N/A	25697	unknown	670	90	90	6B
N/A	26191	unknown	07AR0125	1292	1379	6C
N/A	26108	N/A	CDC1873-00	376	2090	6A
N/A	26109	N/A	GA17328	376	2090	6A
N/A	26111	N/A	GA17971	1175	Sing1175	6A
N/A	26153	N/A	GA19077	1296	2090	6A
N/A	26155	N/A	GA41437	376	2090	6A
N/A	26187	N/A	GA47033	4150	1379	6A/C
N/A	26193	N/A	GA52306	3676	1379	6A/C
N/A	26225	N/A	NP127	376	2090	6A
N/A	26224	N/A	SP18-BS74	6214	146	6
N/A	26223	N/A	SP6-BS73	460	460	6A
121648	34092	ERR044861	UoS0015	176	176	unknown
121565	34009	ERR044865	UoS0038	2496	176	unknown
121454	33898	ERR044866	UoS0041	2457	385	unknown
121412	33856	ERR044869	UoS0050	3240	473	unknown
123190	34114	ERR044873	UoS0068	65	460	unknown
121663	34107	ERR044874	UoS0071	469	176	unknown
121595	34039	ERR044875	UoS0075	65	460	unknown
121633	34077	ERR044878	UoS0082	176	176	unknown
121575	34019	ERR044879	UoS0093	176	176	unknown
121593	34037	ERR044884	UoS0113	65	460	unknown

121638	34082	ERR044886	UoS0136	138	138	unknown
121637	34081	ERR044887	UoS0138	138	138	unknown
121635	34079	ERR044888	UoS0140	176	176	unknown
121572	34016	ERR044891	UoS0152	176	176	unknown
121586	34030	ERR044892	UoS0154	65	460	unknown
121468	33912	ERR044895	UoS0159	402	176	unknown
121634	34078	ERR044899	UoS0172	138	138	unknown
121591	34035	ERR044904	UoS0194	65	460	unknown
121564	34008	ERR044906	UoS0205	2496	176	unknown
115801	33739	ERR044911	UoS0220	176	176	unknown
115804	33742	ERR044918	UoS0245	327	395	unknown
121650	34094	ERR044921	UoS0254	176	176	unknown
115805	33743	ERR044925	UoS0263	1714	395	unknown
115806	33744	ERR044926	UoS0266	65	460	unknown
123306	34173	ERR044930	UoS0273	138	138	unknown
121579	34023	ERR044932	UoS0276	176	176	unknown
121577	34021	ERR044937	UoS0285	176	176	unknown
115811	33749	ERR044940	UoS0290	402	176	unknown
33851	32958	ERR044942	UoS0297	138	138	unknown
40131	33012	ERR044943	UoS0301	65	460	unknown
115817	33755	ERR044952	UoS0314	176	176	unknown
121647	34091	ERR044958	UoS0320	402	176	unknown
121649	34093	ERR044959	UoS0321	402	176	unknown
121639	34083	ERR044961	UoS0324	138	138	unknown
121621	34065	ERR044962	UoS2001	65	460	unknown
121620	34064	ERR044963	UoS2002	65	460	unknown
121641	34085	ERR044964	UoS2003	176	176	unknown
121418	33862	ERR044965	UoS2006	42	439	unknown
121571	34015	ERR044968	UoS2018	138	138	unknown
121667	34110	ERR044972	UoS2029	3460	3460/4337	unknown
121661	34105	ERR044973	UoS2030	469	176	unknown
121609	34053	ERR044977	UoS2038	65	460	unknown
115819	33757	ERR044979	UoS2050	138	138	unknown
115822	33760	ERR044983	UoS2055	176	176	unknown
115823	33761	ERR044984	UoS2058	4020	176	unknown
115824	33762	ERR044985	UoS2060	1714	395	unknown
121515	33959	ERR044989	UoS2073	1692	395	unknown
121518	33962	ERR044990	UoS2074	1692	395	unknown
115831	33769	ERR044995	UoS2105	1692	395	unknown
121589	34033	ERR045003	UoS2153	176	176	unknown
121600	34044	ERR045004	UoS2166	65	460	unknown
115835	33773	ERR045007	UoS2180	1692	395	unknown
121584	34028	ERR045014	UoS2215	1714	395	unknown
121475	33919	ERR045017	UoS2225	176	176	unknown
121608	34052	ERR045019	UoS2233	65	460	unknown
32914	32919	ERR045022	UoS2237	138	138	unknown
115840	33778	ERR045024	UoS2243	138	138	unknown
121642	34086	ERR045026	UoS2247	176	176	unknown
121585	34029	ERR045027	UoS2250	402	176	unknown
121652	34096	ERR045034	UoS2279	176	176	unknown
121509	33953	ERR045037	UoS2288	1714	395	unknown
121658	34102	ERR045038	UoS2289	1692	395	unknown
121666	34109	ERR045041	UoS2300	1862	396	unknown
121662	34106	ERR045042	UoS2305	242	242	unknown
121613	34057	ERR045044	UoS2307	460	460	unknown
121651	34095	ERR045045	UoS2308	65	460	unknown
121427	33871	ERR045048	UoS2316	398	473	unknown
121590	34034	ERR045049	UoS2319	65	460	unknown
123242	34166	ERR048206	UoS2323	65	460	unknown

123243	34167	ERR048208	UoS2325	1692	395	unknown
123244	34168	ERR048212	UoS2337	65	460	unknown
123335	34202	ERR048213	UoS2338	1692	395	unknown
121532	33976	ERR048214	UoS2340	327	395	unknown
123336	34203	ERR048215	UoS2343	65	460	unknown
121562	34006	ERR048216	UoS2346	65	460	unknown
123337	34204	ERR048217	UoS2351	1692	395	unknown
121501	33945	ERR048218	UoS2354	1692	395	unknown
121603	34047	ERR048219	UoS2359	65	460	unknown
121581	34025	ERR048221	UoS2371	395	395	unknown
121502	33946	ERR046049	UoS3022	1692	395	unknown
121631	34075	ERR046051	UoS3026	176	176	unknown
121568	34012	ERR046053	UoS3031	138	138	unknown
123310	34177	ERR046059	UoS3050	1600	1379	unknown
123311	34178	ERR046060	UoS3055	1692	395	unknown
121403	33847	ERR046062	UoS3060	1150	172	unknown
123312	34179	ERR046063	UoS3063	65	460	unknown
121493	33937	ERR046064	UoS3064	1692	395	unknown
121582	34026	ERR046068	UoS3074	1714	395	unknown
123315	34182	ERR046072	UoS3088	398	473	unknown
123220	34144	ERR046079	UoS3122	65	460	unknown
121498	33942	ERR046087	UoS3159	1692	395	unknown
121499	33943	ERR046091	UoS3164	395	395	unknown
123317	34184	ERR046094	UoS3171	65	460	unknown
121500	33944	ERR046096	UoS3180	1692	395	unknown
121482	33926	ERR046098	UoS3182	1692	395	unknown
121495	33939	ERR046099	UoS3190	395	395	unknown
121646	34090	ERR046100	UoS3192	176	176	unknown
121592	34036	ERR046101	UoS3200	65	460	unknown
123320	34187	ERR046105	UoS3225	176	176	unknown
123324	34191	ERR046110	UoS3235	65	460	unknown
121484	33928	ERR046127	UoS3277	1692	395	unknown
121602	34046	ERR046129	UoS3284	1692	395	unknown
44824	33054	ERR046132	UoS3293	65	460	unknown
121578	34022	ERR046157	UoS4049	176	176	unknown
121607	34051	ERR046163	UoS4081	65	460	unknown
33859	32966	ERR046166	UoS4084	42	439	unknown
123327	34194	ERR046168	UoS4091	176	176	unknown
123231	34155	ERR046193	UoS4191	1692	395	unknown
121511	33955	ERR046200	UoS4227	1692	395	unknown
33861	32968	ERR046201	UoS4237	65	460	unknown
55126	33064	ERR046202	UoS4242	65	460	unknown
121610	34054	ERR045354	UoS4287	65	460	unknown
123199	34123	ERR045372	UoS4344	1692	395	unknown
121516	33960	ERR045382	UoS4371	1714	395	unknown
121447	33891	ERR045393	UoS4399	1876	473	unknown
121552	33996	ERR045397	UoS5012	8028	1379	unknown
123308	34175	ERR045406	UoS5026	1692	395	unknown
115853	33790	ERR047131	UoS5065	1692	395	unknown
32920	32925	ERR047134	UoS5072	176	176	unknown
115854	33791	ERR047143	UoS5110	1390	1390	unknown
123213	34137	ERR045426	UoS5144	1692	395	unknown
123217	34141	ERR045437	UoS5173	9685	395	unknown
33866	32973	ERR047112	UoS5203	4581	1379	unknown
44830	33060	ERR047118	UoS5222	398	473	unknown
121512	33956	ERR047154	UoS5269	1692	395	unknown
121601	34045	ERR047160	UoS5284	65	460	unknown
N/A	15458	unknown	6A/2	7188	460	6A
N/A	15469	unknown	6B/3	7199	Sing7199	6B

32973	25732	ERR051576	SMRU1006	4404	385	6B
103534	25745	ERR051582	SMRU1036	4420	4420/5228	6C
40234	25784	ERR051596	SMRU1050	4420	4420/5228	6C
83283	25717	ERR051600	SMRU1054	315	315	6B
32976	25708	ERR051604	SMRU1058	315	315	6B
31984	25773	ERR051609	SMRU1063	5477	5477	6A
103587	25759	ERR051627	SMRU1081	4422	4405	6B
103526	25785	ERR051632	SMRU1086	4420	4420/5228	6C
103571	25765	ERR051649	SMRU1103	4936	4936	6A
33956	25749	ERR051651	SMRU1105	4422	4405	6B
32991	25789	ERR051666	SMRU1120	315	315	6B
33961	24403	ERR051667	SMRU1121	10032	5963	6B
31990	24402	ERR051668	SMRU1122	10032	5963	6B
103541	25865	ERR054586	SMRU1134	4422	4405	6B
103561	25810	ERR054590	SMRU1138	315	315	6B
83331	25860	ERR054597	SMRU1145	4422	4405	6B
82280	25856	ERR054608	SMRU1156	4422	4405	6B
103610	25852	ERR054611	SMRU1159	4420	4420/5228	6C
103596	25815	ERR054613	SMRU1161	315	315	6B
103584	25814	ERR054621	SMRU1169	315	315	6B
103603	25885	ERR054623	SMRU1171	4936	4936	6A
103555	25842	ERR054624	SMRU1172	4408	982	6A
103597	25835	ERR054648	SMRU1197	4403	4536/6767	6A
103582	25929	ERR054296	SMRU1203	10032	5963	6B
103581	25813	ERR054311	SMRU1218	315	315	6B
103614	25869	ERR054313	SMRU1220	4422	4405	6B
103809	25886	ERR054317	SMRU1224	4936	4936	6A
103609	25851	ERR054320	SMRU1227	4420	4420/5228	6C
103576	25895	ERR054324	SMRU1231	5111	Singleton5111	6B
103592	25843	ERR054326	SMRU1233	4408	982	6A
103810	25871	ERR054330	SMRU1237	4422	4405	6B
83301	25908	ERR054337	SMRU1244	5477	5477	6A
103562	25915	ERR054338	SMRU1245	6172	4405	6B
103577	25867	ERR054340	SMRU1247	4422	4405	6B
103559	25914	ERR054344	SMRU1251	6172	4405	6B
103585	25881	ERR054353	SMRU1260	4936	4936	6A
103528	25919	ERR054359	SMRU1266	6704	Singleton6704	6B
103591	25821	ERR054368	SMRU1275	982	982	6C
103583	25920	ERR054369	SMRU1276	6704	Singleton6704	6B
103535	25911	ERR054386	SMRU1293	5477	5477	6A
82082	25702	ERR039572	SMRU13	315	315	6B
103560	25809	ERR054399	SMRU1306	315	315	6B
103600	25884	ERR054412	SMRU1319	4936	4936	6A
83212	25734	ERR047930	SMRU133	4404	385	6B
103816	25873	ERR060042	SMRU1334	4422	4405	6B
103839	25874	ERR060044	SMRU1336	4422	4405	6B
103817	25903	ERR060049	SMRU1341	5127	90	6B
103606	25916	ERR054229	SMRU1352	6172	4405	6B
103558	25917	ERR054236	SMRU1359	6703	4405	6B
103831	25836	ERR054247	SMRU1370	4403	4536/6767	6A
103808	25870	ERR054268	SMRU1391	4422	4405	6B
103832	25930	ERR054283	SMRU1406	10032	5963	6B
103594	25882	ERR054287	SMRU1410	4936	4936	6A
83358	25798	ERR056769	SMRU1431	315	315	6B
103543	25912	ERR056782	SMRU1444	5477	5477	6A
82304	25898	ERR056784	SMRU1446	5127	90	6B
83362	25879	ERR056785	SMRU1447	4936	4936	6A
40346	25786	ERR054422	SMRU1473	95	90	6B
40109	24405	ERR039574	SMRU15	10035	10035	6B

82264	25878	ERR054448	SMRU1500	4936	4936	6A
103593	25868	ERR054468	SMRU1520	4422	4405	6B
103622	25913	ERR054481	SMRU1533	5477	5477	6A
103563	25850	ERR054493	SMRU1545	4420	4420/5228	6C
83324	25797	ERR054494	SMRU1546	315	315	6B
103566	25811	ERR054500	SMRU1552	315	315	6B
103554	25834	ERR057764	SMRU1566	4403	4536/6767	6A
83386	25880	ERR057768	SMRU1570	4936	4936	6A
103580	25812	ERR057774	SMRU1576	315	315	6B
34146	25825	ERR057786	SMRU1588	4403	4536/6767	6A
34148	25824	ERR057790	SMRU1592	2528	2528/8011	6A
103553	25833	ERR057808	SMRU1610	4403	4536/6767	6A
82343	25899	ERR057813	SMRU1615	5127	90	6B
103599	25883	ERR057816	SMRU1618	4936	4936	6A
103834	25819	ERR057829	SMRU1631	315	315	6B
66480	25934	ERR059979	SMRU1655	982	982	6A
103525	25807	ERR056819	SMRU1673	315	315	6B
103617	25844	ERR056827	SMRU1681	4408	982	6A
103519	25864	ERR056841	SMRU1695	4422	4405	6B
82317	25828	ERR056860	SMRU1714	4403	4536/6767	6A
103838	25823	ERR060004	SMRU1776	2016	2016	6B
83245	25754	ERR049112	SMRU178	4422	4405	6B
82367	25889	ERR063789	SMRU1780	4966	4107	6A
103825	25704	ERR039577	SMRU18	10031	5477	6A
103601	25817	ERR060010	SMRU1806	315	315	6B
83391	25799	ERR057832	SMRU1851	315	315	6B
103531	25849	ERR057843	SMRU1862	4420	4420/5228	6C
103811	25872	ERR057861	SMRU1880	4422	4405	6B
103570	25866	ERR057864	SMRU1883	4422	4405	6B
83396	25861	ERR057865	SMRU1884	4422	4405	6B
103548	25832	ERR057869	SMRU1888	4403	4536/6767	6A
103835	25925	ERR057878	SMRU1897	10027	Singleton10027	6C
103812	25892	ERR057879	SMRU1898	4966	4107	6A
103527	25725	ERR049124	SMRU190	982	982	6C
103624	25905	ERR057892	SMRU1911	5228	4420/5228	6C
103602	25818	ERR057898	SMRU1917	315	315	6B
103836	25822	ERR057901	SMRU1920	982	982	6A
83400	25800	ERR057902	SMRU1921	315	315	6A
103619	25918	ERR057905	SMRU1924	6703	4405	6B
103813	25931	ERR057909	SMRU1928	10056	Singleton10056	6A
82351	25888	ERR057910	SMRU1929	4966	4107	6A
83405	25801	ERR057916	SMRU1935	315	315	6B
34155	25826	ERR057927	SMRU1946	4403	4536/6767	6A
44153	25922	ERR057937	SMRU1956	6831	4405	6B
103564	25894	ERR057942	SMRU1961	5109	4420/5228	6C
103574	25891	ERR057945	SMRU1964	4966	4107	6A
82364	25932	ERR057954	SMRU1973	10057	315	6B
83409	25802	ERR057955	SMRU1974	315	315	6B
83410	25936	ERR057956	SMRU1975	SLV4403, 6767	4536/6767	6A
40394	25933	ERR057957	SMRU1976	SLV4403, 6767	4536/6767	6A
103814	25853	ERR057960	SMRU1979	4420	4420/5228	6C
44939	25848	ERR057964	SMRU1983	4420	4420/5228	6C
103547	25808	ERR057965	SMRU1984	315	315	6B
103837	25854	ERR057971	SMRU1990	4420	4420/5228	6C
40396	25896	ERR057974	SMRU1993	5127	90	6B
32032	25788	ERR057984	SMRU2003	315	315	6B
34167	25827	ERR063813	SMRU2020	4403	4536/6767	6A
34168	25904	ERR063814	SMRU2021	5228	4420/5228	6C
55947	25790	ERR063819	SMRU2026	315	315	6B

82374	25923	ERR063820	SMRU2027	6831	4405	6B
55948	25876	ERR063821	SMRU2028	4936	4936	6A
82375	25791	ERR063823	SMRU2030	315	315	6B
40401	25846	ERR063826	SMRU2033	4420	4420/5228	6C
82377	25858	ERR063828	SMRU2035	4422	4405	6B
40402	25855	ERR063838	SMRU2045	4422	4405	6B
83422	25803	ERR063842	SMRU2049	315	315	6B
40403	25906	ERR063846	SMRU2053	5477	5477	6A
82386	25792	ERR063849	SMRU2056	315	315	6B
55949	25877	ERR063865	SMRU2072	4936	4936	6A
83426	25804	ERR063867	SMRU2074	315	315	6B
83429	25805	ERR063876	SMRU2083	315	315	6B
103532	25924	ERR063877	SMRU2084	10027	Singleton10027	6C
83430	25902	ERR063881	SMRU2088	5127	90	6B
34174	25845	ERR063883	SMRU2090	4420	4420/5228	6C
82399	25900	ERR063890	SMRU2097	5127	90	6B
103598	25816	ERR063897	SMRU2104	315	315	6B
82402	25793	ERR063899	SMRU2106	315	315	6B
103575	25730	ERR050075	SMRU212	4403	4536/6767	6A
82412	25935	ERR063920	SMRU2127	SLV9601	2782	6B
82423	25820	ERR063940	SMRU2147	982	982	6C
82429	25839	ERR063951	SMRU2158	4408	982	6A
82435	25794	ERR063961	SMRU2168	315	315	6B
82438	25907	ERR063967	SMRU2174	5477	5477	6A
83452	25862	ERR063971	SMRU2178	4422	4405	6B
103516	25831	ERR063977	SMRU2184	4403	4536/6767	6A
40413	25837	ERR063979	SMRU2186	4408	982	6A
82444	25901	ERR063980	SMRU2187	5127	90	6B
34179	25921	ERR063993	SMRU2200	6831	4405	6B
40419	25847	ERR063996	SMRU2203	4420	4420/5228	6C
82506	25795	ERR066206	SMRU2215	315	315	6B
34243	25893	ERR066207	SMRU2216	5109	4420/5228	6C
44977	25875	ERR066216	SMRU2225	4936	4936	6A
83594	25909	ERR084253	SMRU2235	5477	5477	6A
103807	25736	ERR049943	SMRU224	4404	385	6B
83596	25910	ERR084259	SMRU2241	5477	5477	6A
83598	25806	ERR084261	SMRU2243	315	315	6A
103589	25746	ERR049944	SMRU225	4420	4420/5228	6C
82739	25796	ERR084270	SMRU2252	315	315	6B
82740	25830	ERR084271	SMRU2253	4403	4536/6767	6A
83487	25841	ERR066228	SMRU2261	4408	982	6A
44978	25838	ERR066230	SMRU2263	4408	982	6A
82510	25890	ERR066236	SMRU2269	4966	4107	6A
34255	25887	ERR066237	SMRU2270	4966	4107	6A
34260	24404	ERR066247	SMRU2280	10032	5963	6B
82513	25840	ERR066248	SMRU2281	4408	982	6A
82515	26037	ERR066254	SMRU2287	4936	4936	6A
83246	25755	ERR049948	SMRU229	4422	4405	6B
40503	25998	ERR066260	SMRU2293	4403	4536/6767	6A
82516	26058	ERR066261	SMRU2294	5127	90	6B
44982	26051	ERR066274	SMRU2307	5109	4420/5228	6C
40508	26020	ERR066281	SMRU2314	4420	4420/5228	6C
103520	25976	ERR066298	SMRU2331	315	315	6B
103579	25981	ERR066299	SMRU2332	315	315	6B
103556	26042	ERR066308	SMRU2341	4936	4936	6A
83499	25968	ERR066312	SMRU2345	315	315	6B
82529	25955	ERR066313	SMRU2346	315	315	6B
103605	26016	ERR066314	SMRU2347	4408	982	6A
83500	25969	ERR066320	SMRU2353	315	315	6B

82532	25956	ERR066323	SMRU2356	315	315	6B
82534	26085	ERR066327	SMRU2360	10035	10035	6B
103550	26041	ERR066328	SMRU2361	4936	4936	6A
34268	26005	ERR066334	SMRU2367	4408	982	6A
82537	26025	ERR066341	SMRU2374	4420	4420/5228	6C
40517	26021	ERR066346	SMRU2379	4420	4420/5228	6C
44987	26006	ERR066347	SMRU2380	4408	982	6A
40519	24301	ERR066356	SMRU2389	10027	Singleton10027	6C
82548	26086	ERR066365	SMRU2398	10056	Singleton10056	6A
83507	26013	ERR066366	SMRU2399	4408	982	6A
82479	26079	ERR064143	SMRU2405	6831	4405	6B
55155	26052	ERR064145	SMRU2407	5109	4420/5228	6C
34197	26044	ERR064147	SMRU2409	4966	4107	6A
82481	26036	ERR064152	SMRU2414	4936	4936	6A
83475	26039	ERR064153	SMRU2415	4936	4936	6A
82482	25951	ERR064154	SMRU2416	315	315	6A
55953	26087	ERR064164	SMRU2426	10058	10035	6B
82485	25952	ERR064168	SMRU2430	315	315	6B
103840	26088	ERR064171	SMRU2433	10059	4405	6B
82487	25953	ERR064176	SMRU2438	315	315	6B
82488	26091	ERR064179	SMRU2441	SLV9601	2782	6B
32034	25996	ERR064181	SMRU2443	4113	4113	6A
33136	26004	ERR064184	SMRU2446	4408	982	6A
83476	26075	ERR064188	SMRU2450	5477	5477	6A
83477	26083	ERR064212	SMRU2474	10032	5963	6B
83478	26065	ERR064213	SMRU2475	5127	90	6B
83479	26012	ERR064216	SMRU2478	4408	982	6A
82497	26032	ERR064220	SMRU2482	4414	236/271/320	6B
83482	26077	ERR064227	SMRU2489	6703	4405	6B
82447	25948	ERR064002	SMRU2497	315	315	6B
82450	26007	ERR064007	SMRU2502	4408	982	6A
82452	26057	ERR064011	SMRU2506	5127	90	6A
34181	25937	ERR064018	SMRU2513	315	315	6B
40426	26055	ERR064021	SMRU2516	5127	90	6A
83458	26033	ERR064025	SMRU2520	4422	4405	6B
34182	25989	ERR064026	SMRU2521	982	982	6C
34185	26018	ERR064030	SMRU2525	4420	4420/5228	6C
44957	25946	ERR064044	SMRU2539	315	315	6B
44958	25947	ERR064046	SMRU2541	315	315	6B
83509	26048	ERR067775	SMRU2552	4966	4107	6A
83510	25970	ERR067776	SMRU2553	315	315	6B
82560	25957	ERR067790	SMRU2567	315	315	6B
83517	26076	ERR067792	SMRU2569	5477	5477	6A
83520	26002	ERR067803	SMRU2580	4403	4536/6767	6A
82572	25958	ERR067811	SMRU2588	315	315	6B
103557	26028	ERR067815	SMRU2592	4420	4420/5228	6C
82574	26008	ERR067818	SMRU2595	4408	982	6A
83525	26014	ERR067819	SMRU2596	4408	982	6A
82579	26074	ERR067826	SMRU2603	5477	5477	6A
82580	25959	ERR067829	SMRU2606	315	315	6B
82588	26059	ERR067837	SMRU2614	5127	90	6A
83529	26067	ERR067839	SMRU2616	5127	90	6B
82590	26038	ERR067841	SMRU2618	4936	4936	6A
82591	26053	ERR067842	SMRU2619	5109	4420/5228	6C
83532	25992	ERR067844	SMRU2621	982	982	6C
83535	26071	ERR067850	SMRU2627	5228	4420/5228	6C
82599	26092	ERR067861	SMRU2638	SLV9601	2782	6B
103616	26043	ERR066176	SMRU2641	4936	4936	6A
103820	25987	ERR066178	SMRU2643	315	315	6B

82503	25954	ERR066182	SMRU2647	315	315	6B
82504	26047	ERR066189	SMRU2654	4966	4107	6A
83485	26066	ERR066191	SMRU2656	5127	90	6B
103821	26073	ERR066196	SMRU2661	5228	4420/5228	6C
103621	26072	ERR066197	SMRU2662	5228	4420/5228	6C
40484	26019	ERR066198	SMRU2663	4420	4420/5228	6C
83462	25967	ERR064070	SMRU2668	315	315	6B
82470	25950	ERR064071	SMRU2669	315	315	6B
103818	26084	ERR064075	SMRU2673	10032	5963	6B
103536	25978	ERR064077	SMRU2675	315	315	6B
103607	25983	ERR064088	SMRU2686	315	315	6B
103819	26069	ERR064090	SMRU2688	5127	90	6B
103620	25985	ERR064107	SMRU2705	315	315	6B
103590	26029	ERR064116	SMRU2714	4420	4420/5228	6C
82473	26070	ERR064121	SMRU2719	5228	4420/5228	6C
103608	26099	ERR064129	SMRU2727	SLV9601	2782	6B
103612	25984	ERR064137	SMRU2735	315	315	6B
103626	25986	ERR064138	SMRU2736	315	315	6B
82642	25962	ERR069596	SMRU2746	315	315	6B
34303	26045	ERR069606	SMRU2756	4966	4107	6A
83562	25993	ERR069618	SMRU2768	982	982	6C
34304	25940	ERR069622	SMRU2772	315	315	6B
83564	26095	ERR069625	SMRU2775	DLV315	315	6B
34305	25941	ERR069629	SMRU2779	315	315	6B
40580	26000	ERR069643	SMRU2793	4403	4536/6767	6A
40583	25944	ERR069648	SMRU2798	315	315	6B
82086	25703	ERR039587	SMRU28	4420	4420/5228	6C
40589	26030	ERR069656	SMRU2806	4422	4405	6B
82673	25963	ERR069667	SMRU2817	315	315	6B
40593	26056	ERR069669	SMRU2819	5127	90	6A
82677	25964	ERR069673	SMRU2823	315	315	6B
34309	25942	ERR069679	SMRU2829	315	315	6B
82609	26060	ERR067888	SMRU2833	5127	90	6B
40529	25990	ERR067891	SMRU2836	982	982	6C
34275	25938	ERR067896	SMRU2841	315	315	6B
34278	25939	ERR067905	SMRU2850	315	315	6B
40523	25999	ERR067875	SMRU2868	4403	4536/6767	6A
83541	25971	ERR067876	SMRU2869	315	315	6B
40525	26034	ERR067880	SMRU2873	4936	4936	6A
82607	26093	ERR067883	SMRU2876	SLV2912	2912	6C
82616	25960	ERR067915	SMRU2884	315	315	6B
83545	26049	ERR067925	SMRU2894	4966	4107	6A
32924	25707	ERR047967	SMRU290	315	315	6B
83548	25972	ERR067931	SMRU2900	315	315	6B
82625	25961	ERR067933	SMRU2902	315	315	6B
83549	26094	ERR067936	SMRU2905	SLV9601	2782	6B
40542	25943	ERR067941	SMRU2910	315	315	6B
44991	26035	ERR067946	SMRU2915	4936	4936	6A
82631	26026	ERR067948	SMRU2917	4420	4420/5228	6C
40543	26046	ERR067950	SMRU2919	4966	4107	6A
83581	26096	ERR084159	SMRU2933	SLV2912	2912	6C
34319	25997	ERR084160	SMRU2934	4403	4536/6767	6A
103822	26080	ERR084166	SMRU2940	6831	4405	6B
83583	25974	ERR084168	SMRU2942	315	315	6B
83584	26078	ERR084172	SMRU2946	6703	4405	6B
83585	25975	ERR084176	SMRU2950	315	315	6B
103544	25979	ERR084177	SMRU2951	315	315	6B
103623	26017	ERR084182	SMRU2956	4408	982	6A
83587	26015	ERR084186	SMRU2960	4408	982	6A

103586	26098	ERR084190	SMRU2964	DLV315	315	6B
82715	26010	ERR084192	SMRU2966	4408	982	6A
83589	25994	ERR084198	SMRU2972	982	982	6C
82720	26011	ERR084204	SMRU2978	4408	982	6A
103539	26040	ERR084210	SMRU2984	4936	4936	6A
103572	26050	ERR084213	SMRU2987	4966	4107	6A
103521	26089	ERR084216	SMRU2990	10060	90	6B
45004	26031	ERR084219	SMRU2993	4422	4405	6B
103567	25980	ERR084230	SMRU3004	315	315	6B
103823	26081	ERR084232	SMRU3006	6831	4405	6B
103595	25982	ERR084236	SMRU3010	315	315	6B
103618	26003	ERR084242	SMRU3016	4403	4536/6767	6A
103538	26097	ERR084248	SMRU3022	SLV2912	2912	6C
103824	25988	ERR084250	SMRU3024	315	315	6B
34310	26090	ERR072184	SMRU3035	5127	90	6A
82688	25995	ERR072185	SMRU3036	2912	2912	6C
82689	25991	ERR072186	SMRU3037	982	982	6C
82690	26061	ERR072188	SMRU3039	5127	90	6A
82691	26009	ERR072189	SMRU3040	4408	982	6A
82695	26027	ERR072193	SMRU3044	4420	4420/5228	6C
82698	26062	ERR072196	SMRU3047	5127	90	6B
103524	25977	ERR072206	SMRU3057	315	315	6B
82705	25965	ERR072217	SMRU3068	315	315	6B
83578	25973	ERR072221	SMRU3072	315	315	6B
40603	26001	ERR072229	SMRU3080	4403	4536/6767	6A
40608	25945	ERR072240	SMRU3091	315	315	6B
34318	26054	ERR072242	SMRU3093	5127	90	6A
83609	25729	ERR084309	SMRU3107	4403	4536/6767	6A
83601	26068	ERR084278	SMRU3124	5127	90	6B
82747	26063	ERR084283	SMRU3129	5127	90	6A
82748	25966	ERR084285	SMRU3131	315	315	6B
82750	26064	ERR084287	SMRU3133	5127	90	6A
40616	25897	ERR084290	SMRU3136	5127	90	6B
82754	25859	ERR084294	SMRU3140	4422	4405	6B
82756	25787	ERR084296	SMRU3142	95	90	6B
83605	25863	ERR084297	SMRU3143	4422	4405	6B
103542	25720	ERR048040	SMRU315	315	315	6A
82461	26022	ERR064056	SMRU3151	4420	4420/5228	6A
82463	26023	ERR064059	SMRU3154	4420	4420/5228	6A
82465	26024	ERR064061	SMRU3156	4420	4420/5228	6A
103552	25735	ERR048041	SMRU316	4404	385	6B
82469	25949	ERR064066	SMRU3161	315	315	6B
103533	25776	ERR048060	SMRU335	6703	4405	6B
82124	25752	ERR048068	SMRU343	4422	4405	6B
31974	25747	ERR047990	SMRU361	4422	4405	6B
40171	25777	ERR048013	SMRU384	10028	315	6A
31225	25760	ERR048015	SMRU386	4936	4936	6A
33894	25733	ERR048086	SMRU409	4404	385	6B
32949	25738	ERR048095	SMRU418	4407	982	6B
40178	25711	ERR048110	SMRU433	315	315	6A
33910	25710	ERR048121	SMRU444	315	315	6B
82128	25753	ERR048134	SMRU457	4422	4405	6B
83234	25715	ERR048156	SMRU479	315	315	6B
103803	25731	ERR048166	SMRU489	4403	4536/6767	6A
103804	25723	ERR048174	SMRU497	315	315	6B
44819	24400	ERR039609	SMRU50	10030	4420/5228	6C
103805	25779	ERR048180	SMRU503	10028	315	6A
103806	25740	ERR048181	SMRU504	4407	982	6B
83235	25739	ERR048182	SMRU505	4407	982	6B

55917	25774	ERR039611	SMRU52	5477	5477	6A
83297	25719	ERR052643	SMRU570	315	315	6B
103565	25741	ERR052645	SMRU572	4408	982	6A
40211	25766	ERR051417	SMRU583	4966	4107	6A
32952	25748	ERR051426	SMRU592	4422	4405	6B
83270	25716	ERR051441	SMRU607	315	315	6B
31975	25737	ERR051447	SMRU613	4407	982	6B
82201	25713	ERR051455	SMRU621	315	315	6B
82202	25726	ERR051461	SMRU651	4401	5963	6A
82204	25714	ERR051467	SMRU657	315	315	6B
82210	25728	ERR051502	SMRU692	4403	4536/6767	6A
82104	25781	ERR039629	SMRU70	10030	4420/5228	6C
31978	25742	ERR051536	SMRU726	4420	4420/5228	6C
103569	25721	ERR051542	SMRU732	315	315	6B
103537	25764	ERR051544	SMRU734	4936	4936	6A
103604	25722	ERR052602	SMRU745	315	315	6B
103545	25757	ERR052603	SMRU746	4422	4405	6B
83290	25718	ERR052605	SMRU748	315	315	6B
33113	25724	ERR054513	SMRU773	982	982	6C
33116	25743	ERR054517	SMRU777	4420	4420/5228	6C
33117	25709	ERR054518	SMRU778	315	315	6B
40356	25751	ERR054542	SMRU802	4422	4405	6B
103551	25758	ERR056693	SMRU827	4422	4405	6B
83342	25756	ERR056700	SMRU834	4422	4405	6B
83347	25767	ERR056715	SMRU849	4966	4107	6A
34134	25750	ERR054566	SMRU874	4422	4405	6B
40365	25778	ERR054567	SMRU875	10028	315	6A
34135	25763	ERR054571	SMRU879	4936	4936	6A
103625	25775	ERR049965	SMRU894	5477	5477	6A
82172	25770	ERR049978	SMRU907	5116	4405	6B
33919	25761	ERR049993	SMRU922	4936	4936	6A
40201	24401	ERR050004	SMRU933	10031	5477	6A
82180	25783	ERR050009	SMRU938	10031	5477	6A
103827	25771	ERR050016	SMRU945	5116	4405	6B
103828	25768	ERR050019	SMRU948	5105	4405	6B
40202	25772	ERR050021	SMRU950	5124	4420/5228	6C
33922	25762	ERR050022	SMRU951	4936	4936	6A
82190	25744	ERR050036	SMRU965	4420	4420/5228	6C
82195	25712	ERR050052	SMRU981	315	315	6B
33944	25769	ERR051560	SMRU990	5116	4405	6B
33945	25727	ERR051561	SMRU991	4403	4536/6767	6A
18147	25706	ERR051568	SMRU998	315	315	6B
31415	26145	ERR069732	1996	1360	460	6A
31417	26129	ERR069734	15445	1876	473	6A
33261	26134	ERR069735	18044	1518	146	6B
33262	26113	ERR069740	42397	460	460	6A
31418	24406	ERR069741	42861	10036	690	6A
32177	26139	ERR069744	51715	376	2090	6A
32179	26122	ERR069746	62031	460	460	6A
31419	26137	ERR069747	65435	376	2090	6A
32183	26142	ERR069755	89848	460	460	6A
32185	26125	ERR069757	93209	1379	1379	6C
32187	26124	ERR069759	101058	690	690	6A
32189	26140	ERR069763	108480	376	2090	6A
33266	26149	ERR069764	110093	1939	745/1939	6A
32190	26123	ERR069765	118039	460	460	6A
32191	26127	ERR069766	119571	1390	1390	6C
32193	26112	ERR069770	135631	460	460	6A
32199	26128	ERR069779	153438	1827	138	6B

32200	26146	ERR069782	160986	1536	1536	6B
31425	26143	ERR069784	166637	473	473	6A
32203	26144	ERR069786	174702	639	171	6B
31426	26138	ERR069787	179743	376	2090	6A
31427	26147	ERR069788	181272	1876	473	6A
31435	26114	ERR069798	223832	138	138	6B
31447	26126	ERR069811	291880	1390	1390	6C
31448	26133	ERR069812	299801	460	460	6A
31449	26115	ERR069813	302649	146	146	6B
31454	26116	ERR069819	323485	146	146	6B
31458	26119	ERR069823	336850	460	460	6A
31460	26148	ERR069825	342672	1922	Singleton1922	6A
31461	26130	ERR069827	359370	1876	473	6A
31466	26141	ERR069835	383739	460	460	6A
31470	26117	ERR069840	397079	315	315	6B
33175	26131	ERR065953	420881	1940	473	6A
32064	26150	ERR065957	436154	1954	90	6B
32066	26120	ERR065962	446376	460	460	6A
32067	26118	ERR065963	448643	376	2090	6A
32068	26121	ERR065967	472699	460	460	6A
33181	26136	ERR065969	481643	138	138	6B
32070	26135	ERR065971	487827	138	138	6B
33430	26199	ERR129027	BR1008	473	473	6C
32377	26220	ERR129039	BR1045	1390	1390	6C
32381	26196	ERR129044	BR1064	1692	395	6C
33433	26195	ERR129048	BR1076	1379	1379	6C
32418	26205	ERR129092	CH2090	1876	473	6A
32432	26201	ERR129111	GL3032	1379	1379	6C
31549	26198	ERR129115	GL3043	473	473	6C
32447	26207	ERR129128	LE4007	1538	2090	6A
32460	26215	ERR129145	LE4079	1379	1379	6C
31553	26218	ERR129152	LE4100	1390	1390	6C
33451	26197	ERR129168	MD5030	138	138	6C
31559	26219	ERR129169	MD5031	1390	1390	6C
31561	26208	ERR129172	MD5037	138	138	6B
31565	26222	ERR129194	ND6012	1692	395	6C
32493	26204	ERR129203	ND6046	1390	1390	6C
33388	26216	ERR124217	ND6110	1379	1379	6C
33392	26212	ERR124223	ND6119	473	473	6C
32321	26213	ERR124230	ND6133	547	1439	6C
32322	26211	ERR124231	ND6134	460	460	6A
32323	26203	ERR124232	ND6135	1390	1390	6C
32336	26200	ERR124253	NP7028	1379	1379	6C
32337	26206	ERR124254	NP7029	1390	1390	6C
33407	26221	ERR124264	NP7070	1390	1390	6C
32342	26214	ERR124266	NP7078	1379	1379	6C
32361	26210	ERR124303	PT8074	376	2090	6A
33426	26194	ERR124305	PT8081	1379	1379	6C
34393	26217	ERR124310	PT8092	1379	1379	6C
31500	26209	ERR124314	PT8105	376	2090	6A
31503	26202	ERR124317	PT8114	1390	1390	6C
31311	26173	ERR065344	R34-3018	460	460	6A
31317	26157	ERR065352	R34-3026	242	242	6A
31318	26164	ERR065353	R34-3027	473	473	6A
32051	26162	ERR065292	R34-3035	460	460	6A
31270	26185	ERR065295	R34-3038	1930	Singleton1930	6B
32053	26168	ERR065298	R34-3041	1390	1390	6C
31282	26176	ERR065309	R34-3053	1379	1379	NT
31289	26170	ERR065316	R34-3060	1379	1379	6C

31299	26166	ERR065327	R34-3072	1390	1390	6C
31302	26172	ERR065330	R34-3075	460	460	6A
32082	26174	ERR067991	R34-3086	547	1439	6C
31343	26167	ERR067994	R34-3089	1390	1390	6C
32084	26178	ERR067997	R34-3092	1390	1390	6C
31349	26184	ERR068001	R34-3096	1928	473	6A
32085	26183	ERR068004	R34-3099	1927	473	6A
31352	26175	ERR068005	R34-3100	690	690	6B
31368	26177	ERR068025	R34-3125	1390	1390	6C
31382	26179	ERR068043	R34-3151	1917	473	6A
31323	26161	ERR067964	R34-3166	460	460	6A
31325	26171	ERR067966	R34-3168	376	2090	6A
31328	26158	ERR067969	R34-3171	376	2090	6A
31337	26165	ERR067979	R34-3180	690	690	6A
32137	26160	ERR069685	R34-3186	138	138	6B
32143	26169	ERR069693	R34-3194	SLV 170, 176	176	6B
33259	26182	ERR069700	R34-3201	1921	2090	6A
32154	26181	ERR069709	R34-3210	1876	473	6A
31410	26180	ERR069710	R34-3211	1876	473	6A
32159	26159	ERR069719	R34-3220	460	460	6A
32161	26163	ERR069721	R34-3222	460	460	6A
N/A	35869	N/A	Greece6B-22	273	273	6B
18190	26102	ERR016705	DG29	81	81	6A
39946	26103	ERR016706	DG80	81	81	6A
32282	25522	ERR107521	0001+05003	90	90	6B
34379	25523	ERR107523	0003+13150	90	90	6B
33359	25519	ERR107524	0005+24045	90	90	6B
32283	25518	ERR107525	0006+20010	90	90	6B
40675	25521	ERR107528	0009+01095	90	90	6B
40676	25524	ERR107529	0011+09030	90	90	6B
103515	25520	ERR107530	0012+25031	90	90	6B
33360	25525	ERR107531	0101+26271	90	90	6B
33361	25527	ERR107532	0102+01171	90	90	6B
33362	25528	ERR107533	0105+29173	90	90	6B
33363	25529	ERR107534	0106+11154	90	90	6B
40681	25532	ERR107572	0107+17127	90	90	6B
33364	25533	ERR107535	0108+03050	90	90	6B
33365	25526	ERR107536	0109+28217	90	90	6B
33366	25530	ERR107537	0111+21110	90	90	6B
34380	25531	ERR107538	0112+27129	90	90	6B
34381	25536	ERR107539	0202+01134	90	90	6B
103518	25539	ERR107540	0203+19212	90	90	6B
103508	25534	ERR107541	0205+05018	90	90	6B
32285	25540	ERR107542	0206+21189	90	90	6B
33367	25541	ERR107544	0208+13020	90	90	6B
45025	25538	ERR107545	0209+24059	90	90	6B
32286	25535	ERR107546	0211+13275	90	90	6B
34382	25537	ERR107547	0212+05159	90	90	6B
33368	25542	ERR107548	0301+23540	90	90	6B
34383	25544	ERR107549	0302+20271	90	90	6B
33369	25543	ERR107550	0303+13263	90	90	6B
40678	25545	ERR107561	0407+30157	90	90	6B
34386	25547	ERR107562	0409+02133	90	90	6B
103573	25546	ERR107563	0412+17021	90	90	6B
34387	25549	ERR107564	0501+31089	90	90	6B
103513	25550	ERR107565	0502+25231	90	90	6B
33373	25548	ERR107566	0503+03063	90	90	6B
34388	25551	ERR107567	0505+04001	90	90	6B
40679	25554	ERR107568	0506+29167	90	90	6B

34389	25552	ERR107569	0507+08068	90	90	6B
40680	25555	ERR107570	0508+26122	90	90	6B
34390	25553	ERR107571	0509+09128	90	90	6B
33981	25696	ERR051883	29_PMEN2	90	90	6B
33015	26192	ERR051863	6575-07	90	90	6B
103512	25474	ERR107482	9511+06158	90	90	6
103507	25475	ERR107483	9512+07034	90	90	6
33349	25483	ERR107484	9601+30166	90	90	6
40668	25481	ERR107485	9602+15128	90	90	6B
40669	25477	ERR107486	9603+12093	90	90	6
103517	25487	ERR107487	9605+07141	90	90	6B
103510	25486	ERR107489	9607+10105	90	90	6B
33351	25488	ERR107490	9608+02116	90	90	6
103504	25489	ERR107491	9609+24019	90	90	6B
34370	25485	ERR107492	9611+04103	90	90	6B
34371	25476	ERR107493	9612+28032	90	90	6B
34372	25495	ERR107494	9701+10122	90	90	6B
34373	25497	ERR107495	9702+03174	90	90	6B
33352	25490	ERR107496	9703+24150	90	90	6B
33353	25491	ERR107497	9705+05087	90	90	6B
33354	25494	ERR107498	9706+10167	90	90	6B
33355	25498	ERR107499	9707+14101	90	90	6B
32278	25492	ERR107500	9708+26128	90	90	6B
32279	25496	ERR107501	9711+04135	90	90	6B
32280	25493	ERR107502	9712+30001	90	90	6B
40670	25501	ERR107503	9801+23240	90	90	6
34374	25506	ERR107504	9802+26112	90	90	6B
40671	25508	ERR107505	9803+09178	90	90	6B
103505	25507	ERR107506	9806+08086	90	90	6B
40672	25499	ERR107507	9807+09096	90	90	6B
33356	25504	ERR107508	9808+20015	90	90	6B
44157	25502	ERR107509	9809+25147	90	90	6
33357	25505	ERR107510	9811+05053	90	90	6B
31487	25503	ERR107511	9812+11187	SLV90	90	6B
34375	25509	ERR107512	9901+13020	90	90	6B
40673	25510	ERR107513	9903+09147	90	90	6B
44158	25513	ERR107514	9905+21046	90	90	6B
34376	25515	ERR107515	9906+15160	90	90	6B
40674	25511	ERR107516	9907+14172	90	90	6B
34377	25516	ERR107517	9908+13028	90	90	6B
34378	25517	ERR107518	9909+21117	90	90	6B
44159	25514	ERR107519	9911+18139	90	90	6B
32281	25512	ERR107520	9912+17151	90	90	6B
34073	25645	ERR052390	DCC1524	273	273	6B
34074	25646	ERR052391	DCC1738	273	273	6B
34075	25647	ERR052392	DCC1902	273	273	6B
33105	25648	ERR052400	DCC2613	90	90	6B
34078	25649	ERR052401	DCC2623	90	90	6B
33982	26104	ERR051889	DG_25	90	90	6B
32897	25687	ERR029285	K01_071205	90	90	6B
55915	25688	ERR029279	K01_071280	3387	90	6B
32893	25689	ERR029278	K13_0810	90	90	6B
40102	25692	ERR029289	K13_082	4759	90	6B
32899	25691	ERR029287	K13_0820	1624	90	6B
32895	25690	ERR029282	K13_0827	90	90	6B
31946	25693	ERR029283	K13_0913	90	90	6B
32896	25694	ERR029284	K13_0940	90	90	6B
103501	25258	ERR052038	LMG1250	90	90	6B
44887	26152	ERR051938	LMG1417	90	90	6B

103509	25259	ERR052043	LMG1435	90	90	6B
33991	26154	ERR051948	LMG1978	90	90	6B
103502	26101	ERR051950	LMG2050	95	90	6B
33992	26100	ERR051951	LMG2062	95	90	6B
44888	25640	ERR051955	LMG2230	90	90	6B
33995	25641	ERR051960	LMG2290	90	90	6B
40263	25643	ERR051963	LMG2302	90	90	6B
44889	25644	ERR051965	LMG2311	90	90	6B
44890	25639	ERR051966	LMG2888	90	90	6B
103511	25642	ERR051968	LMG3367	90	90	6B
103529	25264	ERR051921	LMG690	90	90	6B
103506	25637	ERR051924	LMG759	90	90	6B
34013	26151	ERR052017	LMG804	90	90	6B
44884	25257	ERR051930	LMG867	90	90	6B
34014	25256	ERR052023	LMG869	90	90	6B
33964	26188	ERR051799	LMG90	90	90	6B
33102	25651	ERR052393	PT1175	273	273	6A
40316	25654	ERR052395	PT1430	273	273	6B
34077	25650	ERR052398	PT2236	90	90	6B
44905	25657	ERR052402	PT3026	273	273	6B
33104	25655	ERR052399	PT3104	90	90	6B
40315	25656	ERR052394	PT3536	273	273	6B
33103	25652	ERR052396	PT582	273	273	6B
34076	25653	ERR052397	PT76	273	273	6B
33984	25260	ERR051898	S-044	90	90	6B
40255	25261	ERR051900	S-112	90	90	6B
103829	25701	ERR052506	SN11927	620	90	6B
103830	25271	ERR052511	SN26575	90	90	6B
103833	25273	ERR055616	SN33007	273	273	6B
103499	25274	ERR055617	SN34677	273	273	6B
34098	25700	ERR052438	SPN11926	620	90	6B
40323	25269	ERR052439	SPN13151	90	90	6B
55939	25270	ERR052440	SPN13522	90	90	6B
44914	25272	ERR052452	SPN22664	273	273	6B
103498	26229	ERR052456	SPN22809	273	273	6B
103503	26227	ERR052331	SPN2906	273	273	6B
103500	26226	ERR052325	SPN3402	90	90	6B
40331	25265	ERR052464	SPN4000	273	273	6B
34101	25266	ERR052465	SPN4488	273	273	6B
33722	26228	ERR271793	SPN4683	90	90	6B
40332	25267	ERR052466	SPN4876	273	273	6B
34102	25268	ERR052467	SPN4901	273	273	6B
31714	25263	ERR271792	SPN8332	90	90	23F
40087	25450	ERR028591	spnIC104	90	90	6B
44813	25454	ERR028592	spnIC116	90	90	6B
32881	25463	ERR028747	spnIC139	90	90	6B
32883	25465	ERR028751	spnIC141	90	90	6B
33827	25464	ERR028752	spnIC145	90	90	6B
33828	25466	ERR028753	spnIC161	90	90	6B
103495	25472	ERR028754	spnIC174	90	90	6B
31216	25467	ERR028756	spnIC178	90	90	6B
31217	25471	ERR028757	spnIC187	90	90	6B
33824	25461	ERR028597	spnIC19	90	90	6B
32884	25468	ERR028758	spnIC190	90	90	6B
40090	25469	ERR028748	spnIC192	90	90	6B
18113	25470	ERR028749	spnIC195	90	90	6B
31938	25459	ERR028600	spnIC2	90	90	6B
33829	25473	ERR028760	spnIC203	90	90	6B
32871	25460	ERR028596	spnIC28	90	90	6B

32870	25457	ERR028595	spnIC38	90	90	6B
44814	25453	ERR028594	spnIC41	90	90	6B
32885	25479	ERR028766	spnIC419	90	90	6B
103514	25458	ERR028590	spnIC42	90	90	6B
18102	25478	ERR028767	spnIC425	90	90	6B
40091	25480	ERR028769	spnIC432	90	90	6B
18276	25482	ERR028770	spnIC434	90	90	6B
33831	25484	ERR028771	spnIC463	90	90	6B
18061	25456	ERR028736	spnIC48	90	90	6B
18112	25455	ERR028735	spnIC49	90	90	6B
31943	25452	ERR028745	spnIC51	90	90	6B
18101	25447	ERR028744	spnIC52	90	90	6B
31942	25451	ERR028743	spnIC54	90	90	6B
31941	25449	ERR028741	spnIC57	90	90	6B
31215	25448	ERR028740	spnIC58	90	90	6B
31940	25636	ERR028739	spnIC59	90	90	6B
31937	25462	ERR028599	spnIC6	90	90	6B
44815	24407	ERR028734	spnSP522	10037	90	6B
103522	25698	ERR028738	spnSP681	90	90	6B
N/A	25558	ERR388804	VICE0133	460	460	not done
N/A	25556	ERR388853	VICE0140	176	176	not done
N/A	25317	ERR388809	VICE0147	176	176	not done
N/A	25275	ERR388810	VICE0148	176	176	not done
N/A	25557	ERR388858	VICE0153	65	460	not done
N/A	25579	ERR470259	VICE0201	3437	176	not done
N/A	25600	ERR388895	VICE0212	386	315	not done
N/A	25601	ERR388901	VICE0219	460	460	not done
N/A	25292	ERR388909	VICE0238	2221	490	not done
N/A	25609	ERR388910	VICE0239	395	395	not done
N/A	25610	ERR388919	VICE0254	2221	490	not done
N/A	25608	ERR388920	VICE0258	2221	490	not done
N/A	25628	ERR540451	VICE0268	138	138	not done
N/A	25629	ERR540452	VICE0272	65	460	not done
N/A	25303	ERR449729	VICE0287	65	460	not done
N/A	25337	ERR449730	VICE0288	90	90	not done
N/A	25295	ERR449734	VICE0293	90	90	not done
N/A	25355	ERR449736	VICE0295	315	315	not done
N/A	25309	ERR449739	VICE0299	90	90	not done
N/A	25404	ERR449746	VICE0308	2221	490	not done
N/A	25310	ERR449749	VICE0311	2221	490	not done
N/A	25318	ERR449753	VICE0317	90	90	not done
N/A	25296	ERR449759	VICE0324	138	138	not done
N/A	25297	ERR449761	VICE0326	176	176	not done
N/A	25278	ERR449768	VICE0337	2221	490	not done
N/A	25298	ERR449771	VICE0340	3981	15	not done
N/A	25304	ERR449780	VICE0351	460	460	not done
N/A	25343	ERR449781	VICE0352	65	460	not done
N/A	25344	ERR449783	VICE0354	2221	490	not done
N/A	25287	ERR449789	VICE0360	138	138	not done
N/A	25313	ERR449801	VICE0375	176	176	not done
N/A	25324	ERR460404	VICE0382	460	460	not done
N/A	25290	ERR470165	VICE0384	460	460	not done
N/A	25349	ERR470170	VICE0390	2221	490	not done
N/A	25284	ERR470173	VICE0393	176	176	not done
N/A	25330	ERR470174	VICE0394	460	460	not done
N/A	25329	ERR470177	VICE0397	176	176	not done
N/A	25389	ERR470178	VICE0398	65	460	not done
N/A	25283	ERR470179	VICE0399	65	460	not done
N/A	25306	ERR470182	VICE0402	460	460	not done

N/A	25276	ERR470195	VICE0415	176	176	not done
N/A	25291	ERR470200	VICE0420	1716	396	not done
N/A	25348	ERR470208	VICE0428	176	176	not done
N/A	25299	ERR470213	VICE0433	3981	15	not done
N/A	25319	ERR470216	VICE0437	138	138	not done
N/A	25305	ERR470218	VICE0439	138	138	not done
N/A	25279	ERR470224	VICE0447	90	90	not done
N/A	25331	ERR470231	VICE0454	639	171	not done
N/A	25583	ERR470234	VICE0458	138	138	not done
N/A	25285	ERR470235	VICE0460	2221	490	not done
N/A	25315	ERR470240	VICE0465	2221	490	not done
N/A	25332	ERR470244	VICE0470	2221	490	not done
N/A	25372	ERR470249	VICE0478	460	460	not done
N/A	25300	ERR460412	VICE0492	65	460	not done
N/A	25301	ERR460416	VICE0500	2340	460	not done
N/A	25281	ERR460422	VICE0508	176	176	not done
N/A	25293	ERR470278	VICE0509	90	90	not done
N/A	25280	ERR460434	VICE0531	135	135	not done
N/A	25311	ERR540472	VICE0539	460	460	not done
N/A	25277	ERR470287	VICE0550	1692	395	not done
N/A	35888	ERR925543	VICE0567	176	176	not done
N/A	35889	ERR975591	VICE0574	386	315	not done
N/A	25367	ERR460462	VICE0577	138	138	not done
N/A	25335	ERR460466	VICE0581	176	176	not done
N/A	25312	ERR460469	VICE0584	460	460	not done
N/A	25350	ERR460474	VICE0590	2221	490	not done
N/A	25294	ERR460476	VICE0592	10029	490	not done
N/A	25336	ERR460477	VICE0593	10393	176	not done
N/A	35890	ERR975592	VICE0595	460	460	not done
N/A	25282	ERR460488	VICE0608	2221	490	not done
N/A	25386	ERR449807	VICE0622	138	138	not done
N/A	25286	ERR449808	VICE0623	2340	460	not done
N/A	25592	ERR470295	VICE0629	90	90	not done
N/A	25578	ERR449815	VICE0634	90	90	not done
N/A	25570	ERR449818	VICE0637	3981	15	not done
N/A	25576	ERR449822	VICE0641	2221	490	not done
N/A	25574	ERR449829	VICE0651	2221	490	not done
N/A	25577	ERR449833	VICE0655	460	460	not done
N/A	25575	ERR449836	VICE0658	176	176	not done
N/A	25573	ERR449842	VICE0666	395	395	not done
N/A	25571	ERR449844	VICE0668	90	90	not done
N/A	25572	ERR449853	VICE0678	395	395	not done
N/A	25595	ERR470253	VICE0682	146	146	not done
N/A	25597	ERR470254	VICE0684	460	460	not done
N/A	25599	ERR470357	VICE0697	138	138	not done
N/A	25598	ERR470365	VICE0709	2221	490	not done
N/A	25594	ERR470374	VICE0721	138	138	not done
N/A	25593	ERR470375	VICE0722	2221	490	not done
N/A	25596	ERR470376	VICE0724	386	315	not done
N/A	25607	ERR470377	VICE0726	3981	15	not done
N/A	25605	ERR470379	VICE0728	176	176	not done
N/A	25606	ERR470384	VICE0736	2221	490	not done
N/A	25624	ERR470403	VICE0769	2221	490	not done
N/A	25626	ERR470406	VICE0773	138	138	not done
N/A	35997	ERR925558	VICE0776	138	138	not done
N/A	25627	ERR470412	VICE0784	2221	490	not done
N/A	25623	ERR470307	VICE0788	176	176	not done
N/A	25625	ERR470419	VICE0798	90	90	not done
N/A	35998	ERR925560	VICE0799	2221	490	not done

N/A	25582	ERR470437	VICE0823	176	176	not done
N/A	25581	ERR470308	VICE0827	90	90	not done
N/A	25289	ERR470440	VICE0828	460	460	not done
N/A	25602	ERR470443	VICE0831	2221	490	not done
N/A	25611	ERR470446	VICE0838	90	90	not done
N/A	25635	ERR470310	VICE0846	1379	1379	not done
N/A	25369	ERR470313	VICE0849	176	176	not done
N/A	25382	ERR470316	VICE0856	315	315	not done
N/A	25431	ERR470318	VICE0859	176	176	not done
N/A	25425	ERR470319	VICE0860	176	176	not done
N/A	25565	ERR690880	VICE0863	2221	490	not done
N/A	35887	ERR1081448	VICE0874	1716	396	not done
N/A	25341	ERR470328	VICE0877	65	460	not done
N/A	25563	ERR470331	VICE0880	90	90	not done
N/A	25397	ERR540514	VICE0887	176	176	not done
N/A	25441	ERR470337	VICE0889	2221	490	not done
N/A	25567	ERR470339	VICE0892	176	176	not done
N/A	25288	ERR470344	VICE0899	460	460	not done
N/A	25314	ERR470346	VICE0901	176	176	not done
N/A	25364	ERR470348	VICE0903	2221	490	not done
N/A	25568	ERR470351	VICE0907	386	315	not done
N/A	25323	ERR690883	VICE0918	386	315	not done
N/A	25405	ERR690886	VICE0926	176	176	not done
N/A	33194	ERR470157	VICE0930a	176	176	not done
N/A	25564	ERR470159	VICE0932	176	176	not done
N/A	35999	ERR925578	VICE0933	2221	490	not done
N/A	25438	ERR470161	VICE0934	3981	15	not done
N/A	33488	ERR540527	VICE0937a	3981	15	not done
N/A	25440	ERR470163	VICE0940	138	138	not done
N/A	25560	ERR470449	VICE0941	176	176	not done
N/A	25569	ERR470450	VICE0942	3981	15	not done
N/A	25430	ERR470451	VICE0943	176	176	not done
N/A	25388	ERR690888	VICE0944	3981	15	not done
N/A	25439	ERR470452	VICE0948	3981	15	not done
N/A	25380	ERR470454	VICE0952	176	176	not done
N/A	25322	ERR470457	VICE0957	138	138	not done
N/A	25356	ERR470462	VICE0966	138	138	not done
N/A	25435	ERR470465	VICE0974	176	176	not done
N/A	36000	ERR755391	VICE0979	90	90	not done
N/A	25419	ERR470471	VICE0985	176	176	not done
N/A	33483	ERR470474	VICE0989a	2221	490	not done
N/A	25328	ERR470477	VICE0993	176	176	not done
N/A	36001	ERR925585	VICE0994	460	460	not done
N/A	25345	ERR470480	VICE0996	138	138	not done
N/A	25384	ERR540543	VICE0998	10393	176	not done
N/A	25421	ERR470481	VICE0999	138	138	not done
N/A	25368	ERR470482	VICE1001	176	176	not done
N/A	25559	ERR470483	VICE1002	176	176	not done
N/A	25379	ERR470484	VICE1004	90	90	not done
N/A	25357	ERR470486	VICE1007	460	460	not done
N/A	25338	ERR470490	VICE1012	10393	176	not done
N/A	25383	ERR470491	VICE1013	2221	490	not done
N/A	25426	ERR470492	VICE1014	2221	490	not done
N/A	25446	ERR470493	VICE1015	2221	490	not done
N/A	25566	ERR470494	VICE1018	460	460	not done
N/A	25370	ERR690899	VICE1030	2221	490	not done
N/A	25584	ERR540558	VICE1034	90	90	not done
N/A	36002	ERR1081451	VICE1040	138	138	not done
N/A	25427	ERR540567	VICE1044	138	138	not done

N/A	36003	ERR1081452	VICE1047	138	138	not done
N/A	25361	ERR690903	VICE1055	2221	490	not done
N/A	25406	ERR690904	VICE1057	176	176	not done
N/A	25442	ERR690905	VICE1058	2221	490	not done
N/A	25590	ERR540579	VICE1064	3981	15	not done
N/A	25408	ERR540586	VICE1074	3981	15	not done
N/A	36004	ERR925595	VICE1077	138	138	not done
N/A	25302	ERR540592	VICE1082	176	176	not done
N/A	33489	ERR540602	VICE1097a	176	176	not done
N/A	25591	ERR540603	VICE1099	176	176	not done
N/A	25325	ERR540605	VICE1102	176	176	not done
N/A	25326	ERR540606	VICE1103	176	176	not done
N/A	25359	ERR540607	VICE1104	176	176	not done
N/A	25589	ERR540608	VICE1106	176	176	not done
N/A	25307	ERR540611	VICE1109	176	176	not done
N/A	25585	ERR540613	VICE1112	176	176	not done
N/A	25443	ERR540626	VICE1124	2221	490	not done
N/A	25586	ERR540627	VICE1125	2221	490	not done
N/A	25432	ERR540628	VICE1127	138	138	not done
N/A	25587	ERR540635	VICE1133	138	138	not done
N/A	33490	ERR540646	VICE1145a	2221	490	not done
N/A	25588	ERR690911	VICE1147	138	138	not done
N/A	33491	ERR540651	VICE1150a	90	90	not done
N/A	36005	ERR975601	VICE1153	176	176	not done
N/A	25422	ERR540657	VICE1157	176	176	not done
N/A	25428	ERR540660	VICE1160	1692	395	not done
N/A	25381	ERR540662	VICE1163b	176	176	not done
N/A	25360	ERR540664	VICE1165	176	176	not done
N/A	25371	ERR540666	VICE1167	176	176	not done
N/A	25444	ERR540669	VICE1170	2221	490	not done
N/A	25412	ERR690916	VICE1171	2221	490	not done
N/A	25402	ERR540672	VICE1176	176	176	not done
N/A	25334	ERR540673	VICE1177	460	460	not done
N/A	36006	ERR925612	VICE1183	2221	490	not done
N/A	25339	ERR540677	VICE1191	2221	490	not done
N/A	25433	ERR540685	VICE1208	460	460	not done
N/A	36009	ERR755395	VICE1211	2221	490	not done
N/A	33492	ERR540693	VICE1223a	3981	15	not done
N/A	25603	ERR540700	VICE1233	176	176	not done
N/A	36010	ERR925623	VICE1243	2221	490	not done
N/A	25423	ERR540339	VICE1246	2221	490	not done
N/A	25436	ERR540343	VICE1251	460	460	not done
N/A	25604	ERR540345	VICE1253	138	138	not done
N/A	36013	ERR975606	VICE1254	2340	460	not done
N/A	36014	ERR925625	VICE1256	2221	490	not done
N/A	25420	ERR540349	VICE1257b	65	460	not done
N/A	33484	ERR540351	VICE1259a	176	176	not done
N/A	25320	ERR540352	VICE1260	176	176	not done
N/A	25366	ERR540361	VICE1269	1692	395	not done
N/A	33485	ERR540362	VICE1270a	176	176	not done
N/A	25333	ERR540364	VICE1271	176	176	not done
N/A	25365	ERR540366	VICE1274	138	138	not done
N/A	36015	ERR925627	VICE1276a	138	138	not done
N/A	25385	ERR540371	VICE1279	176	176	not done
N/A	36016	ERR925628	VICE1281	2221	490	not done
N/A	25437	ERR540383	VICE1294	2221	490	not done
N/A	36017	ERR925630	VICE1299a	138	138	not done
N/A	36018	ERR975607	VICE1303	460	460	not done
N/A	25376	ERR540393	VICE1308	2340	460	not done

N/A	33486	ERR540397	VICE1314a	176	176	not done
N/A	25614	ERR690936	VICE1315	2221	490	not done
N/A	33487	ERR540401	VICE1319a	176	176	not done
N/A	25394	ERR540407	VICE1325	460	460	not done
N/A	25363	ERR540408	VICE1326	460	460	not done
N/A	25352	ERR540409	VICE1327	460	460	not done
N/A	25391	ERR540414	VICE1332	138	138	not done
N/A	36020	ERR975609	VICE1333	2221	490	not done
N/A	36021	ERR975610	VICE1335	2221	490	not done
N/A	25321	ERR540419	VICE1340	176	176	not done
N/A	25316	ERR540420	VICE1341	2221	490	not done
N/A	25616	ERR540425	VICE1345	460	460	not done
N/A	36022	ERR925635	VICE1346	460	460	not done
N/A	25617	ERR690939	VICE1348	460	460	not done
N/A	25622	ERR690940	VICE1349	460	460	not done
N/A	25414	ERR690941	VICE1350	460	460	not done
N/A	36023	ERR975615	VICE1356	460	460	not done
N/A	36024	ERR975617	VICE1362	2221	490	not done
N/A	25327	ERR690950	VICE1363	176	176	not done
N/A	33493	ERR689452	VICE1364a	176	176	not done
N/A	33494	ERR689454	VICE1365a	176	176	not done
N/A	25613	ERR689456	VICE1368	176	176	not done
N/A	25415	ERR689457	VICE1369	2221	490	not done
N/A	25375	ERR689464	VICE1377	2221	490	not done
N/A	25374	ERR689467	VICE1380	65	460	not done
N/A	25413	ERR689468	VICE1381	138	138	not done
N/A	25618	ERR689470	VICE1383	65	460	not done
N/A	36025	ERR925637	VICE1384	460	460	not done
N/A	25399	ERR689484	VICE1404	2221	490	not done
N/A	25351	ERR689490	VICE1410	138	138	not done
N/A	25612	ERR689491	VICE1412	138	138	not done
N/A	25620	ERR689492	VICE1413	2221	490	not done
N/A	25619	ERR689493	VICE1414	138	138	not done
N/A	36027	ERR755402	VICE1416	2221	490	not done
N/A	25398	ERR689501	VICE1423	176	176	not done
N/A	36028	ERR925639	VICE1424	176	176	not done
N/A	25434	ERR689508	VICE1429	138	138	not done
N/A	25621	ERR689509	VICE1430	65	460	not done
N/A	25342	ERR689517	VICE1437	138	138	not done
N/A	25633	ERR689523	VICE1445	2221	490	not done
N/A	25387	ERR689532	VICE1456	138	138	not done
N/A	25411	ERR689537	VICE1463	1692	395	not done
N/A	25631	ERR689538	VICE1464	1692	395	not done
N/A	25396	ERR689540	VICE1466	2221	490	not done
N/A	25418	ERR689542	VICE1468	639	171	not done
N/A	25340	ERR697210	VICE1473	2221	490	not done
N/A	25429	ERR697212	VICE1476	1716	396	not done
N/A	25630	ERR697226	VICE1490	1716	396	not done
N/A	25445	ERR697232	VICE1497	2221	490	not done
N/A	36030	ERR1007867	VICE1507	2221	490	not done
N/A	25353	ERR697250	VICE1518	460	460	not done
N/A	25401	ERR697255	VICE1523	2221	490	not done
N/A	25403	ERR697268	VICE1536	65	460	not done
N/A	25400	ERR697269	VICE1537	138	138	not done
N/A	25395	ERR697270	VICE1538	138	138	not done
N/A	25377	ERR697273	VICE1540	90	90	not done
N/A	25378	ERR697279	VICE1547	386	315	not done
N/A	25354	ERR697284	VICE1552	460	460	not done
N/A	25634	ERR697298	VICE1566	1379	1379	not done

N/A	25632	ERR697299	VICE1567	1379	1379	not done
N/A	25424	ERR697301	VICE1569	1379	1379	not done
N/A	36032	ERR755407	VICE1573	2221	490	not done
N/A	36035	ERR755411	VICE1577	2221	490	not done
N/A	36041	ERR755418	VICE1584	65	460	not done
N/A	36044	ERR755421	VICE1587	90	90	not done
N/A	36045	ERR755422	VICE1588	90	90	not done
N/A	36046	ERR755423	VICE1589	176	176	not done
N/A	29961	ERR755455	VICE1626	10362	315	not done
N/A	36075	ERR755457	VICE1628	1379	1379	not done
N/A	36079	ERR755461	VICE1632	1379	1379	not done
N/A	36081	ERR755463	VICE1634	460	460	not done
N/A	36083	ERR755465	VICE1636	2221	490	not done
N/A	36091	ERR1007876	VICE1647	2340	460	not done
N/A	36092	ERR755315	VICE1648	2221	490	not done
N/A	36099	ERR755325	VICE1658	1533	177	not done
N/A	36102	ERR755329	VICE1663	2221	490	not done
N/A	36107	ERR956423	VICE1668	460	460	not done
N/A	36109	ERR755335	VICE1670	395	395	not done
N/A	36113	ERR755339	VICE1675	386	315	not done
N/A	36116	ERR755342	VICE1678	176	176	not done
N/A	36120	ERR755346	VICE1682	90	90	not done
N/A	36123	ERR755350	VICE1686	90	90	not done
N/A	36125	ERR956424	VICE1688	386	315	not done
N/A	36126	ERR755352	VICE1689	3981	15	not done
N/A	36127	ERR755353	VICE1690	2756	9789	not done
N/A	36137	ERR755363	VICE1702	138	138	not done
N/A	36141	ERR755369	VICE1707	460	460	not done
N/A	36146	ERR755374	VICE1713	2221	490	not done
N/A	36152	ERR755380	VICE1719	138	138	not done
N/A	36154	ERR755382	VICE1723	138	138	not done
N/A	36155	ERR755383	VICE1724	2221	490	not done
N/A	36158	ERR755387	VICE1730	138	138	not done
N/A	36170	ERR755483	VICE1748	1714	395	not done
N/A	36176	ERR755489	VICE1754	386	315	not done
N/A	36196	ERR755511	VICE1776	138	138	not done
N/A	36203	ERR755518	VICE1788	176	176	not done
N/A	36207	ERR1081463	VICE1796	2221	490	not done
N/A	36215	ERR1007887	VICE1808	90	90	not done
N/A	36216	ERR956433	VICE1810	138	138	not done
N/A	36219	ERR755540	VICE1816	395	395	not done
N/A	36220	ERR755541	VICE1817	395	395	not done
N/A	36221	ERR755542	VICE1818	395	395	not done
N/A	36224	ERR755545	VICE1821	395	395	not done
N/A	36225	ERR755546	VICE1826	395	395	not done
N/A	36227	ERR755549	VICE1829	65	460	not done
N/A	36231	ERR1081466	VICE1837	1692	395	not done
N/A	36251	ERR755578	VICE1861	138	138	not done
N/A	36257	ERR755585	VICE1868	2340	460	not done
N/A	36258	ERR755586	VICE1869	2221	490	not done
N/A	36265	ERR755596	VICE1879a	1716	396	not done
N/A	36266	ERR755597	VICE1880a	1692	395	not done
N/A	36274	ERR755612	VICE1894	176	176	not done
N/A	36276	ERR755614	VICE1896	639	171	not done
N/A	36282	ERR755620	VICE1903	2221	490	not done
N/A	36319	ERR956462	VICE1930	386	315	not done
N/A	36320	ERR1081470	VICE1938	386	315	not done
N/A	36321	ERR956473	VICE1943	460	460	not done
N/A	36322	ERR1081472	VICE1951	1379	1379	not done

N/A	36323	ERR956485	VICE1959	176	176	not done
N/A	36324	ERR956508	VICE1983	386	315	not done
N/A	36325	ERR1081474	VICE1984	639	171	not done

Orig_sero_method	Seq_sero	Hybrid	Hybrid type	Country	Region
Quellung	6Bii	No	N/A	Finland	unknown
Latex/PCR	6A	No	N/A	Iceland	unknown
Quellung	6Bii	No	N/A	USA	Maryland
Quellung	6Bii	No	N/A	Spain	unknown
Quellung	6C	No	N/A	USA	unknown
Quellung	6Bii	No	N/A	South Africa	unknown
Quellung/latex	6A	Yes	6A/6Bii	South Africa	unknown
Quellung/latex	6A	Yes	6A/6Bii	South Africa	unknown
Quellung/latex	6A	Yes	6A/6Bii	South Africa	unknown
Quellung/latex	6A	Yes	6A/6Bii	South Africa	unknown
Quellung/latex	6A	Yes	6A/6Bii	South Africa	unknown
Quellung/latex	6A	Yes	6A/6Bii	South Africa	unknown
Quellung/latex	6A	Yes	6A/6Bii	South Africa	unknown
Quellung/latex	6A	Yes	6A/6Bii	South Africa	unknown
Quellung/latex	6A	Yes	6A/6Bii	South Africa	unknown
Quellung/latex	6A	Yes	6A/6Bii	South Africa	unknown
Quellung/latex	6A	Yes	6A/6Bii	South Africa	unknown
Quellung/latex	6A	Yes	6A/6Bii	South Africa	unknown
Quellung/latex	6A	Yes	6A/6Bii	South Africa	unknown
Quellung/latex	6A	Yes	6A/6Bii	South Africa	unknown
Quellung/latex	6Bii	No	N/A	South Africa	unknown
Quellung/latex	6A	Yes	6A/6Bii	South Africa	unknown
Quellung/latex	6A	Yes	6A/6Bii	South Africa	unknown
Quellung/latex	6Bii	No	N/A	South Africa	Gauteng
Quellung/latex	6Bii	No	N/A	South Africa	unknown
Quellung/latex	6Bii	No	N/A	South Africa	unknown
Quellung/latex	6Bii	Yes	6Bii/6A	South Africa	Gauteng
Quellung/latex	6Bii	No	N/A	South Africa	unknown
Quellung/latex	6A	Yes	6A/6Bii	South Africa	Gauteng
Quellung/latex	6Bii	Yes	6Bii/6A	South Africa	unknown
Quellung/latex	6Bii	No	N/A	South Africa	Gauteng
Quellung/latex	6Bii	No	N/A	South Africa	unknown
Quellung/latex	6A	Yes	6A/6Bii	South Africa	unknown
Quellung/latex	6A	Yes	6A/6Bii	South Africa	unknown
unknown	6Bii	No	N/A	USA	unknown
unknown	6Bii	No	N/A	USA	unknown
unknown	6Bii	No	N/A	Spain	unknown
unknown	6C	No	N/A	USA	Massachussetts
unknown	6A	Yes	6A/6Bii	USA	New York
unknown	6A	Yes	6A/6Bii	USA	Georgia
unknown	6A	No	N/A	USA	Georgia
unknown	6A	Yes	6A/6Bii	USA	Georgia
unknown	6A	Yes	6A/6Bii	USA	Georgia
unknown	6C	No	N/A	USA	Georgia
unknown	6C	No	N/A	USA	Georgia
unknown	6A	Yes	6A/6Bii	USA	Georgia
unknown	6Bii	No	N/A	USA	Pennsylvania
unknown	6A	No	N/A	USA	Pennsylvania
WGS-based	6B	No	N/A	UK	Southampton
WGS-based	6B	No	N/A	UK	Southampton
WGS-based	6Bii	No	N/A	UK	Southampton
WGS-based	6A	No	N/A	UK	Southampton
WGS-based	6A	No	N/A	UK	Southampton
WGS-based	6B	No	N/A	UK	Southampton
WGS-based	6A	No	N/A	UK	Southampton
WGS-based	6B	No	N/A	UK	Southampton
WGS-based	6B	No	N/A	UK	Southampton
WGS-based	6A	No	N/A	UK	Southampton

WGS-based	6B	No	N/A	UK	Southampton
WGS-based	6B	No	N/A	UK	Southampton
WGS-based	6B	No	N/A	UK	Southampton
WGS-based	6B	No	N/A	UK	Southampton
WGS-based	6A	No	N/A	UK	Southampton
WGS-based	6B	No	N/A	UK	Southampton
WGS-based	6B	No	N/A	UK	Southampton
WGS-based	6A	No	N/A	UK	Southampton
WGS-based	6B	No	N/A	UK	Southampton
WGS-based	6B	No	N/A	UK	Southampton
WGS-based	6A	Yes	6A/6Bii	UK	Southampton
WGS-based	6B	No	N/A	UK	Southampton
WGS-based	6C	No	N/A	UK	Southampton
WGS-based	6A	No	N/A	UK	Southampton
WGS-based	6B	No	N/A	UK	Southampton
WGS-based	6B	No	N/A	UK	Southampton
WGS-based	6B	No	N/A	UK	Southampton
WGS-based	6B	No	N/A	UK	Southampton
WGS-based	6B	No	N/A	UK	Southampton
WGS-based	6A	No	N/A	UK	Southampton
WGS-based	6B	No	N/A	UK	Southampton
WGS-based	6B	No	N/A	UK	Southampton
WGS-based	6B	No	N/A	UK	Southampton
WGS-based	6B	No	N/A	UK	Southampton
WGS-based	6A	No	N/A	UK	Southampton
WGS-based	6A	No	N/A	UK	Southampton
WGS-based	6B	No	N/A	UK	Southampton
WGS-based	6A	Yes	6A/6Bii	UK	Southampton
WGS-based	6B	No	N/A	UK	Southampton
WGS-based	6D	No	N/A	UK	Southampton
WGS-based	6B	No	N/A	UK	Southampton
WGS-based	6A	No	N/A	UK	Southampton
WGS-based	6B	No	N/A	UK	Southampton
WGS-based	6B	No	N/A	UK	Southampton
WGS-based	6B	No	N/A	UK	Southampton
WGS-based	6C	No	N/A	UK	Southampton
WGS-based	6C	No	N/A	UK	Southampton
WGS-based	6C	No	N/A	UK	Southampton
WGS-based	6C	No	N/A	UK	Southampton
WGS-based	6B	No	N/A	UK	Southampton
WGS-based	6A	No	N/A	UK	Southampton
WGS-based	6C	No	N/A	UK	Southampton
WGS-based	6C	No	N/A	UK	Southampton
WGS-based	6B	No	N/A	UK	Southampton
WGS-based	6A	No	N/A	UK	Southampton
WGS-based	6B	No	N/A	UK	Southampton
WGS-based	6B	No	N/A	UK	Southampton
WGS-based	6B	No	N/A	UK	Southampton
WGS-based	6B	No	N/A	UK	Southampton
WGS-based	6B	No	N/A	UK	Southampton
WGS-based	6C	No	N/A	UK	Southampton
WGS-based	6C	No	N/A	UK	Southampton
WGS-based	6B	Yes	6B/6A	UK	Southampton
WGS-based	6B	Yes	6B/6A	UK	Southampton
WGS-based	6A	No	N/A	UK	Southampton
WGS-based	6A	No	N/A	UK	Southampton
WGS-based	6C	No	N/A	UK	Southampton
WGS-based	6A	No	N/A	UK	Southampton
WGS-based	6A	No	N/A	UK	Southampton

WGS-based	6C	No	N/A	UK	Southampton
WGS-based	6A	No	N/A	UK	Southampton
WGS-based	6C	No	N/A	UK	Southampton
WGS-based	6A	Yes	6A/6Bii	UK	Southampton
WGS-based	6A	No	N/A	UK	Southampton
WGS-based	6A	No	N/A	UK	Southampton
WGS-based	6C	No	N/A	UK	Southampton
WGS-based	6C	No	N/A	UK	Southampton
WGS-based	6A	No	N/A	UK	Southampton
WGS-based	6C	No	N/A	UK	Southampton
WGS-based	6C	No	N/A	UK	Southampton
WGS-based	6B	No	N/A	UK	Southampton
WGS-based	6B	No	N/A	UK	Southampton
WGS-based	6C	No	N/A	UK	Southampton
WGS-based	6C	No	N/A	UK	Southampton
WGS-based	6C	No	N/A	UK	Southampton
WGS-based	6A	No	N/A	UK	Southampton
WGS-based	6C	No	N/A	UK	Southampton
WGS-based	6C	No	N/A	UK	Southampton
WGS-based	6C	No	N/A	UK	Southampton
WGS-based	6A	No	N/A	UK	Southampton
WGS-based	6C	No	N/A	UK	Southampton
WGS-based	6C	No	N/A	UK	Southampton
WGS-based	6A	No	N/A	UK	Southampton
WGS-based	6C	No	N/A	UK	Southampton
WGS-based	6C	No	N/A	UK	Southampton
WGS-based	6C	No	N/A	UK	Southampton
WGS-based	6B	No	N/A	UK	Southampton
WGS-based	6A	No	N/A	UK	Southampton
WGS-based	6B	No	N/A	UK	Southampton
WGS-based	6A	No	N/A	UK	Southampton
WGS-based	6C	No	N/A	UK	Southampton
WGS-based	6C	No	N/A	UK	Southampton
WGS-based	6A	No	N/A	UK	Southampton
WGS-based	6B	No	N/A	UK	Southampton
WGS-based	6A	No	N/A	UK	Southampton
WGS-based	6A	Yes	6A/6Bii	UK	Southampton
WGS-based	6B	No	N/A	UK	Southampton
WGS-based	6C	No	N/A	UK	Southampton
WGS-based	6C	No	N/A	UK	Southampton
WGS-based	6F	No	N/A	UK	Southampton
WGS-based	6A	No	N/A	UK	Southampton
WGS-based	6A	No	N/A	UK	Southampton
WGS-based	6C	No	N/A	UK	Southampton
WGS-based	6C	No	N/A	UK	Southampton
WGS-based	6A	No	N/A	UK	Southampton
WGS-based	6C	No	N/A	UK	Southampton
WGS-based	6C	No	N/A	UK	Southampton
WGS-based	6C	No	N/A	UK	Southampton
WGS-based	6B	No	N/A	UK	Southampton
WGS-based	6C	No	N/A	UK	Southampton
WGS-based	6C	No	N/A	UK	Southampton
WGS-based	6C	No	N/A	UK	Southampton
WGS-based	6C	No	N/A	UK	Southampton
WGS-based	6C	No	N/A	UK	Southampton
WGS-based	6C	No	N/A	UK	Southampton
WGS-based	6A	No	N/A	UK	Southampton
Quellung	6A	No	N/A	USA	unknown
Quellung	6B	No	N/A	Denmark	unknown

latex & Quellung	6Bii	No	N/A	Thailand	Maela
latex & Quellung	6C	Yes	6C/6D	Thailand	Maela
latex & Quellung	6C	Yes	6C/6D	Thailand	Maela
latex & Quellung	6Bii	No	N/A	Thailand	Maela
latex & Quellung	6Bii	No	N/A	Thailand	Maela
latex & Quellung	6A	Yes	6A/6C	Thailand	Maela
latex & Quellung	6Bii	No	N/A	Thailand	Maela
latex & Quellung	6C	Yes	6C/6D	Thailand	Maela
latex & Quellung	6A	Yes	6A/6C/18F	Thailand	Maela
latex & Quellung	6Bii	No	N/A	Thailand	Maela
latex & Quellung	6Bii	No	N/A	Thailand	Maela
latex & Quellung	6Bii	No	N/A	Thailand	Maela
latex & Quellung	6Bii	No	N/A	Thailand	Maela
latex & Quellung	6Bii	No	N/A	Thailand	Maela
latex & Quellung	6Bii	No	N/A	Thailand	Maela
latex & Quellung	6Bii	No	N/A	Thailand	Maela
latex & Quellung	6Bii	No	N/A	Thailand	Maela
latex & Quellung	6C	Yes	6C/6D	Thailand	Maela
latex & Quellung	6Bii	No	N/A	Thailand	Maela
latex & Quellung	6Bii	No	N/A	Thailand	Maela
latex & Quellung	6A	Yes	6A/6C/18F	Thailand	Maela
latex & Quellung	6A	No	N/A	Thailand	Maela
latex & Quellung	6A	No	N/A	Thailand	Maela
latex & Quellung	6Bii	No	N/A	Thailand	Maela
latex & Quellung	6Bii	No	N/A	Thailand	Maela
latex & Quellung	6Bii	No	N/A	Thailand	Maela
latex & Quellung	6A	Yes	6A/6C/18F	Thailand	Maela
latex & Quellung	6C	Yes	6C/6D	Thailand	Maela
latex & Quellung	6Bii	No	N/A	Thailand	Maela
latex & Quellung	6A	No	N/A	Thailand	Maela
latex & Quellung	6Bii	No	N/A	Thailand	Maela
latex & Quellung	6A	Yes	6A/6C	Thailand	Maela
latex & Quellung	6Bii	No	N/A	Thailand	Maela
latex & Quellung	6Bii	No	N/A	Thailand	Maela
latex & Quellung	6Bii	No	N/A	Thailand	Maela
latex & Quellung	6A	Yes	6A/6C/18F	Thailand	Maela
latex & Quellung	6Bii	No	N/A	Thailand	Maela
latex & Quellung	6C	Yes	6C/6D	Thailand	Maela
latex & Quellung	6Bii	No	N/A	Thailand	Maela
latex & Quellung	6A	Yes	6A/6C	Thailand	Maela
latex & Quellung	6Bii	No	N/A	Thailand	Maela
latex & Quellung	6Bii	No	N/A	Thailand	Maela
latex & Quellung	6A	Yes	6A/6C/18F	Thailand	Maela
latex & Quellung	6Bii	No	N/A	Thailand	Maela
latex & Quellung	6Bii	No	N/A	Thailand	Maela
latex & Quellung	6Bii	No	N/A	Thailand	Maela
latex & Quellung	6Bii	No	N/A	Thailand	Maela
latex & Quellung	6Bii	No	N/A	Thailand	Maela
latex & Quellung	6Bii	No	N/A	Thailand	Maela
latex & Quellung	6A	No	N/A	Thailand	Maela
latex & Quellung	6Bii	No	N/A	Thailand	Maela
latex & Quellung	6Bii	No	N/A	Thailand	Maela
latex & Quellung	6A	Yes	6A/6C/18F	Thailand	Maela
latex & Quellung	6Bii	No	N/A	Thailand	Maela
latex & Quellung	6A	Yes	6A/6C	Thailand	Maela
latex & Quellung	6Bii	No	N/A	Thailand	Maela
latex & Quellung	6A	Yes	6A/6C/18F	Thailand	Maela
latex & Quellung	6Bii	No	N/A	Thailand	Maela
latex & Quellung	6Bii	Yes	6Bii/6A	Thailand	Maela

latex & Quellung	6A	Yes	6A/6C/18F	Thailand	Maela
latex & Quellung	6Bii	No	N/A	Thailand	Maela
latex & Quellung	6A	Yes	6A/6C	Thailand	Maela
latex & Quellung	6C	Yes	6C/6D	Thailand	Maela
latex & Quellung	6Bii	No	N/A	Thailand	Maela
latex & Quellung	6Bii	No	N/A	Thailand	Maela
latex & Quellung	6A	No	N/A	Thailand	Maela
latex & Quellung	6A	Yes	6A/6C/18F	Thailand	Maela
latex & Quellung	6Bii	No	N/A	Thailand	Maela
latex & Quellung	6A	No	N/A	Thailand	Maela
latex & Quellung	6A	Yes	6A/6C/23Novel	Thailand	Maela
latex & Quellung	6A	No	N/A	Thailand	Maela
latex & Quellung	6Bii	No	N/A	Thailand	Maela
latex & Quellung	6A	Yes	6A/6C/18F	Thailand	Maela
latex & Quellung	6Bii	No	N/A	Thailand	Maela
latex & Quellung	6A	No	N/A	Thailand	Maela
latex & Quellung	6Bii	No	N/A	Thailand	Maela
latex & Quellung	6A	No	N/A	Thailand	Maela
latex & Quellung	6Bii	No	N/A	Thailand	Maela
latex & Quellung	6A	No	N/A	Thailand	Maela
latex & Quellung	6Bii	No	N/A	Thailand	Maela
latex & Quellung	6Bii	No	N/A	Thailand	Maela
latex & Quellung	6A	Yes	6A/6C	Thailand	Maela
latex & Quellung	6A	Yes	6A/6C	Thailand	Maela
latex & Quellung	6Bii	No	N/A	Thailand	Maela
latex & Quellung	6Bii	No	N/A	Thailand	Maela
latex & Quellung	6C	Yes	6C/6D	Thailand	Maela
latex & Quellung	6Bii	No	N/A	Thailand	Maela
latex & Quellung	6Bii	No	N/A	Thailand	Maela
latex & Quellung	6Bii	No	N/A	Thailand	Maela
latex & Quellung	6A	No	N/A	Thailand	Maela
latex & Quellung	6C	Yes	6C/6D	Thailand	Maela
latex & Quellung	6A	Yes	6A/6C	Thailand	Maela
latex & Quellung	6C	Yes	6C/6D	Thailand	Maela
latex & Quellung	6C	Yes	6C/6D	Thailand	Maela
latex & Quellung	6Bii	No	N/A	Thailand	Maela
latex & Quellung	6A	No	N/A	Thailand	Maela
latex & Quellung	6A	Yes	6A/6Bii/6C	Thailand	Maela
latex & Quellung	6Bii	No	N/A	Thailand	Maela
latex & Quellung	6A	No	N/A	Thailand	Maela
latex & Quellung	6A	Yes	6A/6C	Thailand	Maela
latex & Quellung	6Bii	No	N/A	Thailand	Maela
latex & Quellung	6A	No	N/A	Thailand	Maela
latex & Quellung	6Bii	No	N/A	Thailand	Maela
latex & Quellung	6C	Yes	6C/6D	Thailand	Maela
latex & Quellung	6A	Yes	6A/6C	Thailand	Maela
latex & Quellung	6Bii	No	N/A	Thailand	Maela
latex & Quellung	6Bii	No	N/A	Thailand	Maela
latex & Quellung	6A	No	N/A	Thailand	Maela
latex & Quellung	6A	No	N/A	Thailand	Maela
latex & Quellung	6C	Yes	6C/6D	Thailand	Maela
latex & Quellung	6C	Yes	6C/6D	Thailand	Maela
latex & Quellung	6Bii	No	N/A	Thailand	Maela
latex & Quellung	6C	Yes	6C/6D	Thailand	Maela
latex & Quellung	6Bii	No	N/A	Thailand	Maela
latex & Quellung	6Bii	No	N/A	Thailand	Maela
latex & Quellung	6A	No	N/A	Thailand	Maela
latex & Quellung	6C	Yes	6C/6D	Thailand	Maela
latex & Quellung	6Bii	No	N/A	Thailand	Maela

latex & Quellung	6Bii	No	N/A	Thailand	Maela
latex & Quellung	6A	Yes	6A/6C/18F	Thailand	Maela
latex & Quellung	6Bii	No	N/A	Thailand	Maela
latex & Quellung	6C	Yes	6C/6D	Thailand	Maela
latex & Quellung	6Bii	No	N/A	Thailand	Maela
latex & Quellung	6Bii	No	N/A	Thailand	Maela
latex & Quellung	6Bii	No	N/A	Thailand	Maela
latex & Quellung	6A	Yes	6A/6C	Thailand	Maela
latex & Quellung	6Bii	No	N/A	Thailand	Maela
latex & Quellung	6A	Yes	6A/6C/18F	Thailand	Maela
latex & Quellung	6Bii	No	N/A	Thailand	Maela
latex & Quellung	6Bii	No	N/A	Thailand	Maela
latex & Quellung	6C	Yes	6C/6D	Thailand	Maela
latex & Quellung	6Bii	No	N/A	Thailand	Maela
latex & Quellung	6C	Yes	6C/6D	Thailand	Maela
latex & Quellung	6Bii	No	N/A	Thailand	Maela
latex & Quellung	6Bii	No	N/A	Thailand	Maela
latex & Quellung	6Bii	No	N/A	Thailand	Maela
latex & Quellung	6A	No	N/A	Thailand	Maela
latex & Quellung	6Bii	No	N/A	Thailand	Maela
latex & Quellung	6C	Yes	6C/6D	Thailand	Maela
latex & Quellung	6A	No	N/A	Thailand	Maela
latex & Quellung	6Bii	No	N/A	Thailand	Maela
latex & Quellung	6A	Yes	6A/6C	Thailand	Maela
latex & Quellung	6Bii	No	N/A	Thailand	Maela
latex & Quellung	6A	No	N/A	Thailand	Maela
latex & Quellung	6A	No	N/A	Thailand	Maela
latex & Quellung	6Bii	No	N/A	Thailand	Maela
latex & Quellung	6Bii	No	N/A	Thailand	Maela
latex & Quellung	6C	Yes	6C/6D	Thailand	Maela
latex & Quellung	6Bii	No	N/A	Thailand	Maela
latex & Quellung	6C	Yes	6C/6D	Thailand	Maela
latex & Quellung	6A	Yes	6A/6C/18F	Thailand	Maela
latex & Quellung	6A	Yes	6A/6C	Thailand	Maela
latex & Quellung	6Bii	No	N/A	Thailand	Maela
latex & Quellung	6A	Yes	6A/6C	Thailand	Maela
latex & Quellung	6A	Yes	6A/6Bii/6C	Thailand	Maela
latex & Quellung	6C	Yes	6C/6D	Thailand	Maela
latex & Quellung	6Bii	No	N/A	Thailand	Maela
latex & Quellung	6A	No	N/A	Thailand	Maela
latex & Quellung	6A	No	N/A	Thailand	Maela
latex & Quellung	6A	No	N/A	Thailand	Maela
latex & Quellung	6A	Yes	6A/6C	Thailand	Maela
latex & Quellung	6A	Yes	6A/6C	Thailand	Maela
latex & Quellung	6Bii	No	N/A	Thailand	Maela
latex & Quellung	6A	No	N/A	Thailand	Maela
latex & Quellung	6A	Yes	6A/6C/18F	Thailand	Maela
latex & Quellung	6Bii	No	N/A	Thailand	Maela
latex & Quellung	6A	No	N/A	Thailand	Maela
latex & Quellung	6Bii	No	N/A	Thailand	Maela
latex & Quellung	6C	Yes	6C/6D	Thailand	Maela
latex & Quellung	6C	Yes	6C/6D	Thailand	Maela
latex & Quellung	6Bii	No	N/A	Thailand	Maela
latex & Quellung	6Bii	No	N/A	Thailand	Maela
latex & Quellung	6A	Yes	6A/6C/18F	Thailand	Maela
latex & Quellung	6Bii	No	N/A	Thailand	Maela
latex & Quellung	6Bii	No	N/A	Thailand	Maela
latex & Quellung	6A	No	N/A	Thailand	Maela
latex & Quellung	6Bii	No	N/A	Thailand	Maela

latex & Quellung	6Bii	No	N/A	Thailand	Maela
latex & Quellung	6Bii	Yes	6Bii/6A	Thailand	Maela
latex & Quellung	6A	Yes	6A/6C/18F	Thailand	Maela
latex & Quellung	6A	No	N/A	Thailand	Maela
latex & Quellung	6C	Yes	6C/6D	Thailand	Maela
latex & Quellung	6C	Yes	6C/6D	Thailand	Maela
latex & Quellung	6A	No	N/A	Thailand	Maela
latex & Quellung	6C	Yes	6C/6D	Thailand	Maela
latex & Quellung	6A	No	N/A	Thailand	Maela
latex & Quellung	6A	No	N/A	Thailand	Maela
latex & Quellung	6Bii	No	N/A	Thailand	Maela
latex & Quellung	6C	Yes	6C/6D	Thailand	Maela
latex & Quellung	6A	Yes	6A/6C	Thailand	Maela
latex & Quellung	6A	Yes	6A/6C/18F	Thailand	Maela
latex & Quellung	6A	Yes	6A/6C/18F	Thailand	Maela
latex & Quellung	6A	Yes	6A/6Bii/6C	Thailand	Maela
latex & Quellung	6Bii	Yes	6Bii/6A	Thailand	Maela
latex & Quellung	6Bii	No	N/A	Thailand	Maela
latex & Quellung	6Bii	No	N/A	Thailand	Maela
latex & Quellung	6Bii	No	N/A	Thailand	Maela
latex & Quellung	6Bii	No	N/A	Thailand	Maela
latex & Quellung	6A	No	N/A	Thailand	Maela
latex & Quellung	6A	No	N/A	Thailand	Maela
latex & Quellung	6A	Yes	6A/6C	Thailand	Maela
latex & Quellung	6Bii	No	N/A	Thailand	Maela
latex & Quellung	6Bii	No	N/A	Thailand	Maela
latex & Quellung	6A	No	N/A	Thailand	Maela
latex & Quellung	6Bii	No	N/A	Thailand	Maela
latex & Quellung	6Bii	No	N/A	Thailand	Maela
latex & Quellung	6Bii	No	N/A	Thailand	Maela
latex & Quellung	6A	No	N/A	Thailand	Maela
latex & Quellung	6A	Yes	6A/6Bii	Thailand	Maela
latex & Quellung	6Bii	No	N/A	Thailand	Maela
latex & Quellung	6A	Yes	6A/6Bii	Thailand	Maela
latex & Quellung	6Bii	No	N/A	Thailand	Maela
latex & Quellung	6C	Yes	6C/6D	Thailand	Maela
latex & Quellung	6C	Yes	6C/6D	Thailand	Maela
latex & Quellung	6Bii	No	N/A	Thailand	Maela
latex & Quellung	6Bii	No	N/A	Thailand	Maela
latex & Quellung	6A	Yes	6A/6C	Thailand	Maela
latex & Quellung	6Bii	No	N/A	Thailand	Maela
latex & Quellung	6Bii	No	N/A	Thailand	Maela
latex & Quellung	6A	Yes	6A/6C	Thailand	Maela
latex & Quellung	6A	No	N/A	Thailand	Maela
latex & Quellung	6Bii	No	N/A	Thailand	Maela
latex & Quellung	6C	Yes	6C/6D	Thailand	Maela
latex & Quellung	6A	No	N/A	Thailand	Maela
latex & Quellung	6A	No	N/A	Thailand	Maela
latex & Quellung	6A	Yes	6A/6C	Thailand	Maela
latex & Quellung	6Bii	No	N/A	Thailand	Maela
latex & Quellung	6A	Yes	6A/6Bii	Thailand	Maela
latex & Quellung	6Bii	No	N/A	Thailand	Maela
latex & Quellung	6A	Yes	6A/6C/18F	Thailand	Maela
latex & Quellung	6C	Yes	6C/6D	Thailand	Maela
latex & Quellung	6C	Yes	6C/6D	Thailand	Maela
latex & Quellung	6C	Yes	6C/6D	Thailand	Maela
latex & Quellung	6Bii	No	N/A	Thailand	Maela
latex & Quellung	6A	Yes	6A/6C/18F	Thailand	Maela
latex & Quellung	6Bii	No	N/A	Thailand	Maela

latex & Quellung	6Bii	No	N/A	Thailand	Maela
latex & Quellung	6A	Yes	6A/6C	Thailand	Maela
latex & Quellung	6Bii	No	N/A	Thailand	Maela
latex & Quellung	6C	Yes	6C/6D	Thailand	Maela
latex & Quellung	6C	Yes	6C/6D	Thailand	Maela
latex & Quellung	6C	Yes	6C/6D	Thailand	Maela
latex & Quellung	6Bii	No	N/A	Thailand	Maela
latex & Quellung	6Bii	No	N/A	Thailand	Maela
latex & Quellung	6Bii	No	N/A	Thailand	Maela
latex & Quellung	6Bii	No	N/A	Thailand	Maela
latex & Quellung	6Bii	No	N/A	Thailand	Maela
latex & Quellung	6Bii	No	N/A	Thailand	Maela
latex & Quellung	6Bii	No	N/A	Thailand	Maela
latex & Quellung	6C	Yes	6C/6D	Thailand	Maela
latex & Quellung	6C	Yes	6C/6D	Thailand	Maela
latex & Quellung	6Bii	No	N/A	Thailand	Maela
latex & Quellung	6Bii	No	N/A	Thailand	Maela
latex & Quellung	6Bii	No	N/A	Thailand	Maela
latex & Quellung	6Bii	No	N/A	Thailand	Maela
latex & Quellung	6A	Yes	6A/6C	Thailand	Maela
latex & Quellung	6C	Yes	6C/6D	Thailand	Maela
latex & Quellung	6Bii	No	N/A	Thailand	Maela
latex & Quellung	6Bii	No	N/A	Thailand	Maela
latex & Quellung	6Bii	No	N/A	Thailand	Maela
latex & Quellung	6A	No	N/A	Thailand	Maela
latex & Quellung	6Bii	No	N/A	Thailand	Maela
latex & Quellung	6C	Yes	6C/6D	Thailand	Maela
latex & Quellung	6Bii	No	N/A	Thailand	Maela
latex & Quellung	6Bii	No	N/A	Thailand	Maela
latex & Quellung	6A	Yes	6A/6Bii	Thailand	Maela
latex & Quellung	6Bii	No	N/A	Thailand	Maela
latex & Quellung	6Bii	No	N/A	Thailand	Maela
latex & Quellung	6Bii	No	N/A	Thailand	Maela
latex & Quellung	6C	Yes	6C/6D	Thailand	Maela
latex & Quellung	6Bii	No	N/A	Thailand	Maela
latex & Quellung	6Bii	No	N/A	Thailand	Maela
latex & Quellung	6A	No	N/A	Thailand	Maela
latex & Quellung	6Bii	No	N/A	Thailand	Maela
latex & Quellung	6A	Yes	6A/6C/18F	Thailand	Maela
latex & Quellung	6C	Yes	6C/6D	Thailand	Maela
latex & Quellung	6Bii	No	N/A	Thailand	Maela
latex & Quellung	6A	Yes	6A/6C	Thailand	Maela
latex & Quellung	6Bii	No	N/A	Thailand	Maela
latex & Quellung	6Bii	No	N/A	Thailand	Maela
latex & Quellung	6Bii	No	N/A	Thailand	Maela
latex & Quellung	6Bii	No	N/A	Thailand	Maela
latex & Quellung	6Bii	No	N/A	Thailand	Maela
latex & Quellung	6A	Yes	6A/6C/18F	Thailand	Maela
latex & Quellung	6C	Yes	6C/6D	Thailand	Maela
latex & Quellung	6A	Yes	6A/6C	Thailand	Maela
latex & Quellung	6C	Yes	6C/6D	Thailand	Maela
latex & Quellung	6A	No	N/A	Thailand	Maela
latex & Quellung	6Bii	No	N/A	Thailand	Maela
latex & Quellung	6Bii	No	N/A	Thailand	Maela
latex & Quellung	6Bii	No	N/A	Thailand	Maela
latex & Quellung	6Bii	No	N/A	Thailand	Maela
latex & Quellung	6Bii	No	N/A	Thailand	Maela
latex & Quellung	6A	No	N/A	Thailand	Maela
latex & Quellung	6A	No	N/A	Thailand	Maela

latex & Quellung	6Bii	No	N/A	Thailand	Maela
latex & Quellung	6A	No	N/A	Thailand	Maela
latex & Quellung	6C	Yes	6C/6D	Thailand	Maela
latex & Quellung	6A	No	N/A	Thailand	Maela
latex & Quellung	6A	Yes	6A/6C/18F	Thailand	Maela
latex & Quellung	6A	Yes	6A/6C	Thailand	Maela
latex & Quellung	6Bii	No	N/A	Thailand	Maela
latex & Quellung	6Bii	No	N/A	Thailand	Maela
latex & Quellung	6Bii	No	N/A	Thailand	Maela
latex & Quellung	6Bii	No	N/A	Thailand	Maela
latex & Quellung	6Bii	No	N/A	Thailand	Maela
latex & Quellung	6A	No	N/A	Thailand	Maela
latex & Quellung	6C	Yes	6C/6D	Thailand	Maela
latex & Quellung	6Bii	No	N/A	Thailand	Maela
latex & Quellung	6A	Yes	6A/6Bii	Thailand	Maela
latex & Quellung	6C	Yes	6C/6D	Thailand	Maela
latex & Quellung	6C	Yes	6C/6D	Thailand	Maela
latex & Quellung	6A	Yes	6A/6Bii	Thailand	Maela
latex & Quellung	6A	No	N/A	Thailand	Maela
latex & Quellung	6C	Yes	6C/6D	Thailand	Maela
latex & Quellung	6Bii	No	N/A	Thailand	Maela
latex & Quellung	6Bii	No	N/A	Thailand	Maela
latex & Quellung	6Bii	No	N/A	Thailand	Maela
latex & Quellung	6Bii	No	N/A	Thailand	Maela
latex & Quellung	6A	No	N/A	Thailand	Maela
latex & Quellung	6Bii	No	N/A	Thailand	Maela
latex & Quellung	6A	Yes	6A/6Bii	Thailand	Maela
latex & Quellung	6A	No	N/A	Thailand	Maela
latex & Quellung	6Bii	No	N/A	Thailand	Maela
latex & Quellung	6A	Yes	6A/6Bii	Thailand	Maela
latex & Quellung	6Bii	No	N/A	Thailand	Maela
latex & Quellung	6A	Yes	6A/6Bii	Thailand	Maela
latex & Quellung	6Bii	No	N/A	Thailand	Maela
latex & Quellung	6Bii	No	N/A	Thailand	Maela
latex & Quellung	6Bii	No	N/A	Thailand	Maela
latex & Quellung	6Bii	No	N/A	Thailand	Maela
latex & Quellung	6A	Yes	6A/6Bii/6C	Thailand	Maela
latex & Quellung	6C	Yes	6C/6D	Thailand	Maela
latex & Quellung	6C	Yes	6C/6D	Thailand	Maela
latex & Quellung	6C	Yes	6C/6D	Thailand	Maela
latex & Quellung	6Bii	No	N/A	Thailand	Maela
latex & Quellung	6Bii	No	N/A	Thailand	Maela
latex & Quellung	6Bii	No	N/A	Thailand	Maela
latex & Quellung	6Bii	No	N/A	Thailand	Maela
latex & Quellung	6Bii	No	N/A	Thailand	Maela
latex & Quellung	6A	Yes	6A/6Bii/6C	Thailand	Maela
latex & Quellung	6A	Yes	6A/6C/18F	Thailand	Maela
latex & Quellung	6Bii	No	N/A	Thailand	Maela
latex & Quellung	6D	No	N/A	Thailand	Maela
latex & Quellung	6A	Yes	6A/6Bii/6C	Thailand	Maela
latex & Quellung	6Bii	No	N/A	Thailand	Maela
latex & Quellung	6Bii	No	N/A	Thailand	Maela
latex & Quellung	6Bii	No	N/A	Thailand	Maela
latex & Quellung	6A	No	N/A	Thailand	Maela
latex & Quellung	6Bii	No	N/A	Thailand	Maela
latex & Quellung	6C	Yes	6C/6D	Thailand	Maela
latex & Quellung	6A	Yes	6A/6Bii/6C	Thailand	Maela
latex & Quellung	6D	No	N/A	Thailand	Maela
latex & Quellung	6D	No	N/A	Thailand	Maela

latex & Quellung	6A	Yes	6A/6C	Thailand	Maela
latex & Quellung	6Bii	No	N/A	Thailand	Maela
latex & Quellung	6A	No	N/A	Thailand	Maela
latex & Quellung	6A	Yes	6A/6C	Thailand	Maela
latex & Quellung	6Bii	No	N/A	Thailand	Maela
latex & Quellung	6Bii	No	N/A	Thailand	Maela
latex & Quellung	6D	No	N/A	Thailand	Maela
latex & Quellung	6Bii	No	N/A	Thailand	Maela
latex & Quellung	6A	Yes	6A/6Bii	Thailand	Maela
latex & Quellung	6Bii	No	N/A	Thailand	Maela
latex & Quellung	6A	No	N/A	Thailand	Maela
latex & Quellung	6C	Yes	6C/6D	Thailand	Maela
latex & Quellung	6C	Yes	6C/6D	Thailand	Maela
latex & Quellung	6Bii	No	N/A	Thailand	Maela
latex & Quellung	6A	Yes	6A/6C/18F	Thailand	Maela
latex & Quellung	6Bii	No	N/A	Thailand	Maela
latex & Quellung	6Bii	No	N/A	Thailand	Maela
latex & Quellung	6Bii	No	N/A	Thailand	Maela
latex & Quellung	6C	Yes	6C/6D	Thailand	Maela
latex & Quellung	6C	Yes	6C/6D	Thailand	Maela
latex & Quellung	6Bii	No	N/A	Thailand	Maela
latex & Quellung	6Bii	No	N/A	Thailand	Maela
latex & Quellung	6Bii	No	N/A	Thailand	Maela
latex & Quellung	6Bii	No	N/A	Thailand	Maela
latex & Quellung	6A	Yes	6A/6C	Thailand	Maela
latex & Quellung	6Bii	No	N/A	Thailand	Maela
latex & Quellung	6A	Yes	6A/6Bii/6C	Thailand	Maela
latex & Quellung	6A	Yes	6A/6C/18F	Thailand	Maela
latex & Quellung	6A	Yes	6A/6C	Thailand	Maela
latex & Quellung	6Bii	No	N/A	Thailand	Maela
latex & Quellung	6A	Yes	6A/6C/18F	Thailand	Maela
latex & Quellung	6A	Yes	6A/6C	Thailand	Maela
latex & Quellung	6A	Yes	6A/6C	Thailand	Maela
latex & Quellung	6Bii	No	N/A	Thailand	Maela
latex & Quellung	6Bii	No	N/A	Thailand	Maela
latex & Quellung	6C	Yes	6C/6D	Thailand	Maela
latex & Quellung	6A	Yes	6A/6C/18F	Thailand	Maela
latex & Quellung	6C	Yes	6C/6D	Thailand	Maela
latex & Quellung	6Bii	No	N/A	Thailand	Maela
latex & Quellung	6Bii	No	N/A	Thailand	Maela
latex & Quellung	6A	No	N/A	Thailand	Maela
latex & Quellung	6Bii	No	N/A	Thailand	Maela
Quellung	6A	No	N/A	USA	Massachusetts
Quellung	6A	No	N/A	USA	Massachusetts
Quellung	6Bii	No	N/A	USA	Massachusetts
Quellung	6A	No	N/A	USA	Massachusetts
Quellung	6A	No	N/A	USA	Massachusetts
Quellung	6A	Yes	6A/6Bii	USA	Massachusetts
Quellung	6A	No	N/A	USA	Massachusetts
Quellung	6A	Yes	6A/6Bii	USA	Massachusetts
Quellung	6A	No	N/A	USA	Massachusetts
Quellung	6C	No	N/A	USA	Massachusetts
Quellung	6A	No	N/A	USA	Massachusetts
Quellung	6A	Yes	6A/6Bii	USA	Massachusetts
Quellung	6A	No	N/A	USA	Massachusetts
Quellung	6A	No	N/A	USA	Massachusetts
Quellung	6C	No	N/A	USA	Massachusetts
Quellung	6A	No	N/A	USA	Massachusetts
Quellung	6B	No	N/A	USA	Massachusetts

Quellung	6Bii	No	N/A	USA	Massachusetts
Quellung	6A	No	N/A	USA	Massachusetts
Quellung	6B	No	N/A	USA	Massachusetts
Quellung	6A	Yes	6A/6Bii	USA	Massachusetts
Quellung	6A	No	N/A	USA	Massachusetts
Quellung	6B	No	N/A	USA	Massachusetts
Quellung	6C	No	N/A	USA	Massachusetts
Quellung	6A	No	N/A	USA	Massachusetts
Quellung	6Bii	No	N/A	USA	Massachusetts
Quellung	6Bii	No	N/A	USA	Massachusetts
Quellung	6A	No	N/A	USA	Massachusetts
Quellung	6Bii	No	N/A	USA	Massachusetts
Quellung	6A	No	N/A	USA	Massachusetts
Quellung	6A	No	N/A	USA	Massachusetts
Quellung	6Bii	No	N/A	USA	Massachusetts
Quellung	6A	No	N/A	USA	Massachusetts
Quellung	6Bii	No	N/A	USA	Massachusetts
Quellung	6A	No	N/A	USA	Massachusetts
Quellung	6A	Yes	6A/6Bii	USA	Massachusetts
Quellung	6A	No	N/A	USA	Massachusetts
Quellung	6B	No	N/A	USA	Massachusetts
Quellung	6B	No	N/A	USA	Massachusetts
Quellung	6C	No	N/A	USA	Massachusetts
Quellung	6C	No	N/A	USA	Massachusetts
Quellung	6C	No	N/A	USA	Massachusetts
Quellung	6C	No	N/A	USA	Massachusetts
Quellung	6A	No	N/A	USA	Massachusetts
Quellung	6C	No	N/A	USA	Massachusetts
Quellung	6C	No	N/A	USA	Massachusetts
Quellung	6A	Yes	6A/6Bii	USA	Massachusetts
Quellung	6C	No	N/A	USA	Massachusetts
Quellung	6C	No	N/A	USA	Massachusetts
Quellung	6C	No	N/A	USA	Massachusetts
Quellung	6C	No	N/A	USA	Massachusetts
Quellung	6B	No	N/A	USA	Massachusetts
Quellung	6C	No	N/A	USA	Massachusetts
Quellung	6C	No	N/A	USA	Massachusetts
Quellung	6C	No	N/A	USA	Massachusetts
Quellung	6C	No	N/A	USA	Massachusetts
Quellung	6C	Yes	6C/34	USA	Massachusetts
Quellung	6A	No	N/A	USA	Massachusetts
Quellung	6C	No	N/A	USA	Massachusetts
Quellung	6C	No	N/A	USA	Massachusetts
Quellung	6C	No	N/A	USA	Massachusetts
Quellung	6C	No	N/A	USA	Massachusetts
Quellung	6C	No	N/A	USA	Massachusetts
Quellung	6A	Yes	6A/6Bii	USA	Massachusetts
Quellung	6C	No	N/A	USA	Massachusetts
Quellung	6C	No	N/A	USA	Massachusetts
Quellung	6A	Yes	6A/6Bii	USA	Massachusetts
Quellung	6C	No	N/A	USA	Massachusetts
Quellung	6A	No	N/A	USA	Massachusetts
Quellung	6A	No	N/A	USA	Massachusetts
Quellung	6A	No	N/A	USA	Massachusetts
Quellung	6A	No	N/A	USA	Massachusetts
Quellung	6Bii	No	N/A	USA	Massachusetts
Quellung	6C	No	N/A	USA	Massachusetts
Quellung	6C	No	N/A	USA	Massachusetts
Quellung	6C	No	N/A	USA	Massachusetts

Quellung	6C	No	N/A	USA	Massachusetts
Quellung	6A	No	N/A	USA	Massachusetts
Quellung	6C	Yes	6C/34	USA	Massachusetts
Quellung	6C	No	N/A	USA	Massachusetts
Quellung	6C	No	N/A	USA	Massachusetts
Quellung	6A	No	N/A	USA	Massachusetts
Quellung	6A	No	N/A	USA	Massachusetts
Quellung	6Bii	Yes	6Bii/6A	USA	Massachusetts
Quellung	6C	No	N/A	USA	Massachusetts
Quellung	6A	No	N/A	USA	Massachusetts
Quellung	6A	No	N/A	USA	Massachusetts
Quellung	6A	Yes	6A/6Bii	USA	Massachusetts
Quellung	6A	Yes	6A/6Bii	USA	Massachusetts
Quellung	6A	No	N/A	USA	Massachusetts
Quellung	6B	No	N/A	USA	Massachusetts
Quellung	6B	No	N/A	USA	Massachusetts
Quellung	6A	Yes	6A/6Bii	USA	Massachusetts
Quellung	6A	No	N/A	USA	Massachusetts
Quellung	6A	No	N/A	USA	Massachusetts
Quellung	6A	No	N/A	USA	Massachusetts
Quellung	6A	No	N/A	USA	Massachusetts
Quellung	6Bii	No	N/A	Greece	Patras
Quellung	6A	No	N/A	Turkey	unknown
Quellung	6A	No	N/A	Turkey	unknown
Latex/PCR	6Bii	No	N/A	Iceland	Reykjavík
Latex/PCR	6Bii	No	N/A	Iceland	Reykjavík
Latex/PCR	6Bii	No	N/A	Iceland	Reykjavík
Latex/PCR	6Bii	No	N/A	Iceland	Reykjavík
Latex/PCR	6Bii	No	N/A	Iceland	Reykjavík
Latex/PCR	6Bii	No	N/A	Iceland	Reykjavík
Latex/PCR	6Bii	No	N/A	Iceland	Reykjanesbær
Latex/PCR	6Bii	No	N/A	Iceland	Reykjanesbær
Latex/PCR	6Bii	No	N/A	Iceland	Reykjanesbær
Latex/PCR	6Bii	No	N/A	Iceland	Bessastaðahreppur
Latex/PCR	6Bii	No	N/A	Iceland	Garðabær
Latex/PCR	6Bii	No	N/A	Iceland	Reykjanesbær
Latex/PCR	6Bii	No	N/A	Iceland	Germany
Latex/PCR	6Bii	No	N/A	Iceland	Reykjavík
Latex/PCR	6Bii	No	N/A	Iceland	Akranes
Latex/PCR	6Bii	No	N/A	Iceland	Reykjavík
Latex/PCR	6Bii	No	N/A	Iceland	Kópavogur
Latex/PCR	6Bii	No	N/A	Iceland	Reykjanesbær
Latex/PCR	6Bii	No	N/A	Iceland	Reykjavík
Latex/PCR	6Bii	No	N/A	Iceland	Reykjavík
Latex/PCR	6Bii	No	N/A	Iceland	Seltjarnarnes
Latex/PCR	6Bii	No	N/A	Iceland	Reykjavík
Latex/PCR	6Bii	No	N/A	Iceland	Hafnarfjörður
Latex/PCR	6Bii	No	N/A	Iceland	Reykjavík
Latex/PCR	6Bii	No	N/A	Iceland	Reykjavík
Latex/PCR	6Bii	No	N/A	Iceland	Reykjavík
Latex/PCR	6Bii	No	N/A	Iceland	Reykjavík
Latex/PCR	6Bii	No	N/A	Iceland	Kópavogur
Latex/PCR	6Bii	No	N/A	Iceland	Reykjavík
Latex/PCR	6Bii	No	N/A	Iceland	Akranes
Latex/PCR	6Bii	No	N/A	Iceland	Kópavogur
Latex/PCR	6Bii	No	N/A	Iceland	Kópavogur
Latex/PCR	6Bii	No	N/A	Iceland	Reykjavík
Latex/PCR	6Bii	No	N/A	Iceland	Reykjavík
Latex/PCR	6Bii	No	N/A	Iceland	Hveragerdi

Latex/PCR	6Bii	No	N/A	Iceland	Reykjavík
Latex/PCR	6Bii	No	N/A	Iceland	Reykjavík
Latex/PCR	6Bii	No	N/A	Iceland	Mosfellsbær
Quellung/latex	6Bii	No	N/A	Spain	unknown
Quellung/latex	6Bii	No	N/A	USA	Tennessee
Latex/PCR	6Bii	No	N/A	Iceland	Reykjavík
Latex/PCR	6Bii	No	N/A	Iceland	Reykjavík
Latex/PCR	6Bii	No	N/A	Iceland	Reykjavík
Latex/PCR	6Bii	No	N/A	Iceland	Kópavogur
Latex/PCR	6Bii	No	N/A	Iceland	Reykjavík
Latex/PCR	6Bii	No	N/A	Iceland	Garðabær
Latex/PCR	6Bii	No	N/A	Iceland	Reykjavík
Latex/PCR	6Bii	No	N/A	Iceland	Garðabær
Latex/PCR	6Bii	No	N/A	Iceland	Hveragerdi
Latex/PCR	6Bii	No	N/A	Iceland	Reykjavík
Latex/PCR	6Bii	No	N/A	Iceland	Hafnarfjörður
Latex/PCR	6Bii	No	N/A	Iceland	Akureyri
Latex/PCR	6Bii	No	N/A	Iceland	Reykjavík
Latex/PCR	6Bii	No	N/A	Iceland	Reykjavík
Latex/PCR	6Bii	No	N/A	Iceland	Reykjavík
Latex/PCR	6Bii	No	N/A	Iceland	Kópavogur
Latex/PCR	6Bii	No	N/A	Iceland	Kópavogur
Latex/PCR	6Bii	No	N/A	Iceland	Reykjavík
Latex/PCR	6Bii	No	N/A	Iceland	Mosfellsbær
Latex/PCR	6Bii	No	N/A	Iceland	Reykjanesbær
Latex/PCR	6Bii	No	N/A	Iceland	Reykjavík
Latex/PCR	6Bii	No	N/A	Iceland	Reykjavík
Latex/PCR	6Bii	No	N/A	Iceland	Reykjavík
Latex/PCR	6Bii	No	N/A	Iceland	Reykjavík
Latex/PCR	6Bii	No	N/A	Iceland	Mosfellsbær
Latex/PCR	6Bii	No	N/A	Iceland	Reykjavík
Latex/PCR	6Bii	No	N/A	Iceland	Reykjavík
Latex/PCR	6Bii	No	N/A	Iceland	Reykjavík
Latex/PCR	6Bii	No	N/A	Iceland	Kópavogur
Latex/PCR	6Bii	No	N/A	Iceland	Bessastaðahreppur
Latex/PCR	6Bii	No	N/A	Iceland	Reykjavík
Latex/PCR	6Bii	No	N/A	Iceland	Reykjavík
Latex/PCR	6Bii	No	N/A	Iceland	Reykjavík
Latex/PCR	6Bii	No	N/A	Iceland	Reykjavík
Latex/PCR	6Bii	No	N/A	Iceland	Reykjavík
Latex/PCR	6Bii	No	N/A	Iceland	Grindavik
Latex/PCR	6Bii	No	N/A	Iceland	Seltjarnarnes
Latex/PCR	6Bii	No	N/A	Iceland	Reykjavík
Quellung/latex	6Bii	No	N/A	Portugal	Lisbon
Quellung/latex	6Bii	No	N/A	Portugal	unknown
Quellung/latex	6Bii	No	N/A	Portugal	Lisbon
Quellung/latex	6Bii	No	N/A	Portugal	Lisbon
Quellung/latex	6Bii	No	N/A	Portugal	Lisbon
Quellung/latex	6Bii	No	N/A	Turkey	unknown
Quellung/latex	6Bii	No	N/A	South Korea	unknown
Quellung/latex	6Bii	No	N/A	South Korea	unknown
Quellung/latex	6Bii	No	N/A	South Korea	unknown
Quellung/latex	6Bii	No	N/A	South Korea	unknown
Quellung/latex	6Bii	No	N/A	South Korea	unknown
Quellung/latex	6Bii	No	N/A	South Korea	unknown
Quellung/latex	6Bii	No	N/A	South Korea	unknown
Quellung/latex	6Bii	No	N/A	South Korea	unknown
Quellung/latex	6Bii	No	N/A	China	Hong Kong
Quellung/latex	6Bii	No	N/A	USA	Boston

Quellung/latex	6Bii	No	N/A	China	Hong Kong
Quellung/latex	6Bii	No	N/A	USA	Georgia
Quellung/latex	6Bii	No	N/A	Thailand	unknown
Quellung/latex	6Bii	No	N/A	Thailand	unknown
Quellung/latex	6Bii	No	N/A	Peru	Lima
Quellung/latex	6Bii	No	N/A	Peru	Lima
Quellung/latex	6Bii	No	N/A	Peru	Lima
Quellung/latex	6Bii	No	N/A	Peru	Lima
Quellung/latex	6Bii	No	N/A	Peru	unknown
Quellung/latex	6Bii	No	N/A	Peru	Lima
Quellung/latex	6Bii	No	N/A	France	Paris
Quellung/latex	6Bii	No	N/A	Oman	unknown
Quellung/latex	6Bii	No	N/A	USA	Tennessee
Quellung/latex	6Bii	No	N/A	China	Hong Kong
Quellung/latex	6Bii	No	N/A	China	Hong Kong
Quellung/latex	6Bii	No	N/A	USA	Tennessee
Quellung/latex	6Bii	No	N/A	Portugal	Lisbon
Quellung/latex	6Bii	No	N/A	Portugal	unknown
Quellung/latex	6Bii	No	N/A	Portugal	Lisbon
Quellung/latex	6Bii	No	N/A	Portugal	Lisbon
Quellung/latex	6Bii	No	N/A	Portugal	Lisbon
Quellung/latex	6Bii	No	N/A	Portugal	Lisbon
Quellung/latex	6Bii	No	N/A	Portugal	unknown
Quellung/latex	6Bii	No	N/A	Portugal	unknown
Quellung/latex	6Bii	No	N/A	China	Shanghai
Quellung/latex	6Bii	No	N/A	China	Shanghai
Quellung/latex	6Bii	No	N/A	Spain	unknown
Quellung/latex	6Bii	No	N/A	Germany	unknown
Quellung/latex	6Bii	No	N/A	Germany	unknown
Quellung/latex	6Bii	No	N/A	Germany	unknown
Quellung/latex	6Bii	No	N/A	Spain	unknown
Quellung/latex	6Bii	No	N/A	Germany	unknown
Quellung/latex	6Bii	No	N/A	Germany	unknown
Quellung/latex	6Bii	No	N/A	Germany	unknown
Quellung/latex	6Bii	No	N/A	Unknown	unknown
Quellung/latex	6Bii	No	N/A	Unknown	unknown
Quellung/latex	6Bii	No	N/A	Unknown	unknown
Quellung/latex	6Bii	No	N/A	Germany	unknown
Quellung/latex	6Bii	No	N/A	Germany	unknown
Quellung/latex	6Bii	No	N/A	Unknown	unknown
Quellung/latex	6Bii	No	N/A	Germany	unknown
Quellung/latex	6Bii	No	N/A	Germany	unknown
Quellung/latex	6Bii	No	N/A	France	unknown
Latex/PCR	6Bii	No	N/A	Iceland	Reykjavík
Latex/PCR	6Bii	No	N/A	Iceland	Reydarfjordur
Latex/PCR	6Bii	No	N/A	Iceland	Reykjavík
Latex/PCR	6Bii	No	N/A	Iceland	U.S. Naval Hospital
Latex/PCR	6Bii	No	N/A	Iceland	unknown
Latex/PCR	6Bii	No	N/A	Iceland	Reykjavík
Latex/PCR	6Bii	No	N/A	Iceland	Reykjavík
Latex/PCR	6Bii	No	N/A	Iceland	Reykjavík
Latex/PCR	6Bii	No	N/A	Iceland	Eyjafjordur
Latex/PCR	6Bii	No	N/A	Iceland	Reykjavík
Latex/PCR	6Bii	No	N/A	Iceland	Reykjavík
Latex/PCR	6Bii	No	N/A	Iceland	Eyjafjordur
Latex/PCR	6Bii	No	N/A	Iceland	Reykjavík
Latex/PCR	6Bii	No	N/A	Iceland	Reykjavík
Latex/PCR	6Bii	No	N/A	Iceland	Reykjavík
Latex/PCR	6Bii	No	N/A	Iceland	Reykjavík

Latex/PCR	6Bii	No	N/A	Iceland	Reykjavík
Latex/PCR	6Bii	No	N/A	Iceland	Kópavogur
Latex/PCR	6Bii	No	N/A	Iceland	Reykjavík
Latex/PCR	6Bii	No	N/A	Iceland	Reykjavík
Latex/PCR	6Bii	No	N/A	Iceland	Reykjavík
Latex/PCR	6Bii	No	N/A	Iceland	Reykjavík
Latex/PCR	6Bii	No	N/A	Iceland	Reykjavík
Latex/PCR	6Bii	No	N/A	Iceland	Grindavik
Latex/PCR	6Bii	No	N/A	Iceland	Reykjavík
Latex/PCR	6Bii	No	N/A	Iceland	Reykjavík
Latex/PCR	6Bii	No	N/A	Iceland	Reykjavík
Latex/PCR	6Bii	No	N/A	Iceland	Reykjavík
Latex/PCR	6Bii	No	N/A	Iceland	Laugavatn
Latex/PCR	6Bii	No	N/A	Iceland	Reykjavík
Latex/PCR	6Bii	No	N/A	Iceland	Kópavogur
Latex/PCR	6Bii	No	N/A	Iceland	Reykjavík
Latex/PCR	6Bii	No	N/A	Iceland	Akureyri
Quellung/latex	6Bii	No	N/A	Spain	unknown
Quellung/latex	6Bii	No	N/A	Spain	unknown
--	6A	No	N/A	Iceland	Hveragerði
--	6B	No	N/A	Iceland	Hafnarfjörður
--	6B	No	N/A	Iceland	Reykjavík
--	6B	No	N/A	Iceland	unknown
--	6A	No	N/A	Iceland	Reykjavík
--	6B	No	N/A	Iceland	Seltjarnarnes
--	6C	No	N/A	Iceland	Reykjavík
--	6A	No	N/A	Iceland	Reykjavík
--	6A	No	N/A	Iceland	Álftanes
--	6A	No	N/A	Iceland	Reykjanesbær
--	6A	No	N/A	Iceland	Reykjavík
--	6A	No	N/A	Iceland	Kópavogur
--	6B	No	N/A	Iceland	Garðabær
--	6A	No	N/A	Iceland	Reykjavík
--	6A	No	N/A	Iceland	Reykjavík
--	6Bii	No	N/A	Iceland	Kópavogur
--	6Bii	No	N/A	Iceland	Reykjavík
--	6Bii	No	N/A	Iceland	Sandgerði
--	6Bii	No	N/A	Iceland	Kópavogur
--	6A	No	N/A	Iceland	Reykjavík
--	6A	No	N/A	Iceland	Egilsstaðir
--	6Bii	No	N/A	Iceland	Reykjavík
--	6B	No	N/A	Iceland	Kópavogur
--	6B	No	N/A	Iceland	Kópavogur
--	6A	No	N/A	Iceland	Hafnarfjörður
--	6A	No	N/A	Iceland	Hafnarfjörður
--	6A	No	N/A	Iceland	Reykjavík
--	6A	No	N/A	Iceland	Reykjavík
--	6A	No	N/A	Iceland	Reykjavík
--	6B	No	N/A	Iceland	Reykjavík
--	6B	No	N/A	Iceland	Blönduós
--	6A	No	N/A	Iceland	Reykjavík
--	6A	No	N/A	Iceland	Reykjavík
--	6A	No	N/A	Iceland	Hafnarfjörður
--	6A	No	N/A	Iceland	Reykjavík
--	6A	No	N/A	Iceland	Reykjavík
--	6B	No	N/A	Iceland	Reykjavík
--	6A	No	N/A	Iceland	Hafnarfjörður
--	6A	No	N/A	Iceland	Reykjavík
--	6A	No	N/A	Iceland	Kópavogur

--	6B	No	N/A	Iceland	Grindavík
--	6A	Yes	6A/6Bii	Iceland	Reykjavík
--	6B	No	N/A	Iceland	Reykjavík
--	6A	No	N/A	Iceland	Reykjavík
--	6B	No	N/A	Iceland	Reykjavík
--	6B	No	N/A	Iceland	Grundarfjörður
--	6Bii	No	N/A	Iceland	Kópavogur
--	6B	No	N/A	Iceland	Reykjavík
--	6B	No	N/A	Iceland	Kópavogur
--	6A	No	N/A	Iceland	Reykjavík
--	6A	No	N/A	Iceland	Kópavogur
--	6A	No	N/A	Iceland	Hafnarfjörður
--	6A	No	N/A	Iceland	Reykjavík
--	6A	No	N/A	Iceland	Akureyri
--	6A	No	N/A	Iceland	Garðabær
--	6B	No	N/A	Iceland	Hafnarfjörður
--	6Bii	No	N/A	Iceland	Reykjavík
--	6Bii	No	N/A	Iceland	Hafnarfjörður
--	6A	No	N/A	Iceland	Reykjavík
--	6C	No	N/A	Iceland	Garðabær
--	6B	No	N/A	Iceland	Mosfellsbær
--	6C	No	N/A	Iceland	Borgarnes
--	6B	No	N/A	Iceland	Reykjavík
--	6B	No	N/A	Iceland	Reykjavík
--	6A	No	N/A	Iceland	Kópavogur
--	6A	No	N/A	Iceland	Reykjavík
--	6A	No	N/A	Iceland	Reykjavík
--	6B	No	N/A	Iceland	Garðabær
--	6A	No	N/A	Iceland	Hafnarfjörður
--	6A	No	N/A	Iceland	Reykjavík
--	6B	No	N/A	Iceland	Garðabær
--	6A	No	N/A	Iceland	Reykjavík
--	6Bii	No	N/A	Iceland	Garði
--	6Bii	No	N/A	Iceland	Reykjavík
--	6A	No	N/A	Iceland	Hafnarfjörður
--	6A	No	N/A	Iceland	Hafnarfjörður
--	6A	No	N/A	Iceland	Kópavogur
--	6A	No	N/A	Iceland	Reykjavík
--	6B	No	N/A	Iceland	Reykjavík
--	6A	No	N/A	Iceland	Reykjavík
--	6Bii	No	N/A	Iceland	Reykjavík
--	6A	No	N/A	Iceland	Reykjavík
--	6Bii	No	N/A	Iceland	Reykjavík
--	6A	No	N/A	Iceland	Hafnarfjörður
--	6B	No	N/A	Iceland	Reykjavík
--	6A	No	N/A	Iceland	Reykjavík
--	6B	No	N/A	Iceland	Reykjavík
--	6A	No	N/A	Iceland	Reykjavík
--	6C	No	N/A	Iceland	Álftanes
--	6A	No	N/A	Iceland	Reykjavík
--	6B	No	N/A	Iceland	Mosfellsbær
--	6A	No	N/A	Iceland	Álftanes
--	6A	No	N/A	Iceland	Reykjavík
--	6B	No	N/A	Iceland	Reykjanesbær
--	6B	No	N/A	Iceland	Höfn í Hornafirði
--	6A	No	N/A	Iceland	Kópavogur
--	6B	No	N/A	Iceland	Mosfellsbær
--	6Bii	No	N/A	Iceland	Reykjavík
--	6A	No	N/A	Iceland	Reykjavík

--	6B	No	N/A	Iceland	Reykjavík
--	6Bii	No	N/A	Iceland	Garði
--	6A	No	N/A	Iceland	Reykjavík
--	6A	No	N/A	Iceland	Kópavogur
--	6Bii	No	N/A	Iceland	Reykjanesbær
--	6C	No	N/A	Iceland	Reykjavík
--	6B	No	N/A	Iceland	Reykjavík
--	6Bii	No	N/A	Iceland	Reykjavík
--	6B	No	N/A	Iceland	Reykjavík
--	6B	No	N/A	Iceland	Reykjavík
--	6A	No	N/A	Iceland	Reykjavík
--	6A	Yes	6A/6Bii	Iceland	Reykjavík
--	6A	No	N/A	Iceland	Reykjavík
--	6Bii	No	N/A	Iceland	Reykjavík
--	6B	No	N/A	Iceland	Reykjavík
--	6A	No	N/A	Iceland	Reykjavík
--	6B	No	N/A	Iceland	Reykjavík
--	6A	No	N/A	Iceland	Kópavogur
--	6B	No	N/A	Iceland	Kópavogur
--	6A	No	N/A	Iceland	Kópavogur
--	6C	No	N/A	Iceland	Kópavogur
--	6C	No	N/A	Iceland	Kópavogur
--	6B	No	N/A	Iceland	Kópavogur
--	6B	No	N/A	Iceland	Kópavogur
--	6B	No	N/A	Iceland	Kópavogur
--	6A	No	N/A	Iceland	Kópavogur
--	6A	No	N/A	Iceland	Hafnarfjörður
--	6A	No	N/A	Iceland	Hafnarfjörður
--	6B	No	N/A	Iceland	Hafnarfjörður
--	6B	No	N/A	Iceland	Hafnarfjörður
--	6A	No	N/A	Iceland	Hafnarfjörður
--	6B	No	N/A	Iceland	Hafnarfjörður
--	6A	No	N/A	Iceland	Hafnarfjörður
--	6A	No	N/A	Iceland	Hafnarfjörður
--	6B	No	N/A	Iceland	Hafnarfjörður
--	6B	No	N/A	Iceland	Hafnarfjörður
--	6B	No	N/A	Iceland	Hafnarfjörður
--	6B	No	N/A	Iceland	Kópavogur
--	6Bii	No	N/A	Iceland	Reykjavík
--	6B	No	N/A	Iceland	Hafnarfjörður
--	6A	No	N/A	Iceland	Hafnarfjörður
--	6B	No	N/A	Iceland	Hafnarfjörður
--	6A	No	N/A	Iceland	Hafnarfjörður
--	6B	No	N/A	Iceland	Hafnarfjörður
--	6B	No	N/A	Iceland	Hafnarfjörður
--	6B	No	N/A	Iceland	Hafnarfjörður
--	6B	No	N/A	Iceland	Reykjavík
--	6B	No	N/A	Iceland	Reykjavík
--	6Bii	No	N/A	Iceland	Reykjavík
--	6A	No	N/A	Iceland	Hafnarfjörður
--	6B	No	N/A	Iceland	Hafnarfjörður
--	6A	No	N/A	Iceland	Hafnarfjörður
--	6A	No	N/A	Iceland	Hafnarfjörður
--	6A	No	N/A	Iceland	Hafnarfjörður
--	6A	No	N/A	Iceland	Hafnarfjörður
--	6A	No	N/A	Iceland	Kópavogur
--	6Bii	No	N/A	Iceland	Hafnarfjörður
--	6B	No	N/A	Iceland	Hafnarfjörður
--	6B	No	N/A	Iceland	Hafnarfjörður

--	6B	No	N/A	Iceland	Hafnarfjörður
--	6A	No	N/A	Iceland	Hafnarfjörður
--	6B	No	N/A	Iceland	Hafnarfjörður
--	6A	No	N/A	Iceland	Hafnarfjörður
--	6A	No	N/A	Iceland	Hafnarfjörður
--	6A	No	N/A	Iceland	Hafnarfjörður
--	6B	No	N/A	Iceland	Hafnarfjörður
--	6B	No	N/A	Iceland	Hafnarfjörður
--	6B	No	N/A	Iceland	Kópavogur
--	6B	No	N/A	Iceland	Kópavogur
--	6B	No	N/A	Iceland	Kópavogur
--	6B	No	N/A	Iceland	Kópavogur
--	6B	No	N/A	Iceland	Kópavogur
--	6B	No	N/A	Iceland	Kópavogur
--	6B	No	N/A	Iceland	Kópavogur
--	6B	No	N/A	Iceland	Kópavogur
--	6A	No	N/A	Iceland	Kópavogur
--	6A	No	N/A	Iceland	Kópavogur
--	6B	No	N/A	Iceland	Kópavogur
--	6B	No	N/A	Iceland	Kópavogur
--	6A	No	N/A	Iceland	Reykjavík
--	6B	No	N/A	Iceland	Reykjavík
--	6Bii	No	N/A	Iceland	Reykjavík
--	6B	No	N/A	Iceland	Reykjavík
--	6B	No	N/A	Iceland	Reykjavík
--	6C	No	N/A	Iceland	Reykjavík
--	6B	No	N/A	Iceland	unknown
--	6B	No	N/A	Iceland	Reykjavík
--	6B	No	N/A	Iceland	Reykjavík
--	6A	No	N/A	Iceland	Reykjavík
--	6A	No	N/A	Iceland	Reykjavík
--	6B	No	N/A	Iceland	Reykjavík
--	6A	No	N/A	Iceland	Hafnarfjörður
--	6A	No	N/A	Iceland	Hafnarfjörður
--	6A	No	N/A	Iceland	Hafnarfjörður
--	6A	No	N/A	Iceland	Hafnarfjörður
--	6A	No	N/A	Iceland	Hafnarfjörður
--	6A	No	N/A	Iceland	Hafnarfjörður
--	6B	No	N/A	Iceland	Kópavogur
--	6A	No	N/A	Iceland	Kópavogur
--	6A	No	N/A	Iceland	Kópavogur
--	6A	No	N/A	Iceland	Kópavogur
--	6B	No	N/A	Iceland	Kópavogur
--	6A	No	N/A	Iceland	Kópavogur
--	6A	No	N/A	Iceland	Kópavogur
--	6A	No	N/A	Iceland	unknown
--	6B	No	N/A	Iceland	Kópavogur
--	6B	No	N/A	Iceland	Kópavogur
--	6C	No	N/A	Iceland	Reykjavík
--	6B	No	N/A	Iceland	Reykjavík
--	6B	No	N/A	Iceland	Reykjavík
--	6B	No	N/A	Iceland	Reykjavík
--	6B	No	N/A	Iceland	Reykjavík
--	6B	No	N/A	Iceland	Reykjavík
--	6A	No	N/A	Iceland	Reykjavík
--	6A	No	N/A	Iceland	Reykjavík
--	6B	No	N/A	Iceland	Reykjavík
--	6A	No	N/A	Iceland	Kópavogur
--	6A	No	N/A	Iceland	Kópavogur

--	6B	No	N/A	Iceland	Hafnarfjörður
--	6A	No	N/A	Iceland	Hafnarfjörður
--	6B	No	N/A	Iceland	Hafnarfjörður
--	6A	No	N/A	Iceland	Hafnarfjörður
--	6A	No	N/A	Iceland	Hafnarfjörður
--	6A	No	N/A	Iceland	Hafnarfjörður
--	6B	No	N/A	Iceland	Hafnarfjörður
--	6A	No	N/A	Iceland	Hafnarfjörður
--	6A	No	N/A	Iceland	Hafnarfjörður
--	6A	No	N/A	Iceland	Hafnarfjörður
--	6A	No	N/A	Iceland	Hafnarfjörður
--	6A	No	N/A	Iceland	Hafnarfjörður
--	6A	No	N/A	Iceland	Hafnarfjörður
--	6A	No	N/A	Iceland	Hafnarfjörður
--	6A	No	N/A	Iceland	Hafnarfjörður
--	6A	No	N/A	Iceland	Hafnarfjörður
--	6A	No	N/A	Iceland	Hafnarfjörður
--	6A	No	N/A	Iceland	Kópavogur
--	6B	No	N/A	Iceland	Kópavogur
--	6B	No	N/A	Iceland	Kópavogur
--	6B	No	N/A	Iceland	Kópavogur
--	6B	No	N/A	Iceland	Kópavogur
--	6A	No	N/A	Iceland	Kópavogur
--	6A	No	N/A	Iceland	Kópavogur
--	6A	No	N/A	Iceland	Kópavogur
--	6B	No	N/A	Iceland	Kópavogur
--	6A	No	N/A	Iceland	Kópavogur
--	6A	No	N/A	Iceland	Kópavogur
--	6A	No	N/A	Iceland	Reykjavík
--	6B	No	N/A	Iceland	Reykjavík
--	6B	No	N/A	Iceland	Reykjavík
--	6A	No	N/A	Iceland	Reykjavík
--	6B	No	N/A	Iceland	Reykjavík
--	6A	No	N/A	Iceland	Reykjavík
--	6B	No	N/A	Iceland	Reykjavík
--	6B	No	N/A	Iceland	Reykjavík
--	6B	No	N/A	Iceland	Reykjavík
--	6A	No	N/A	Iceland	Reykjavík
--	6B	No	N/A	Iceland	Hafnarfjörður
--	6A	No	N/A	Iceland	Hafnarfjörður
--	6B	No	N/A	Iceland	Hafnarfjörður
--	6C	No	N/A	Iceland	Kópavogur
--	6C	No	N/A	Iceland	Kópavogur
--	6A	No	N/A	Iceland	Hafnarfjörður
--	6B	No	N/A	Iceland	Hafnarfjörður
--	6A	No	N/A	Iceland	Hafnarfjörður
--	6A	Yes	6A/6Bii	Iceland	Kópavogur
--	6A	Yes	6A/6Bii	Iceland	Kópavogur
--	6A	No	N/A	Iceland	Kópavogur
--	6A	No	N/A	Iceland	Kópavogur
--	6A	No	N/A	Iceland	Hafnarfjörður
--	6A	No	N/A	Iceland	unknown
--	6A	No	N/A	Iceland	Kópavogur
--	6B	No	N/A	Iceland	Kópavogur
--	6B	No	N/A	Iceland	Kópavogur
--	6Bii	No	N/A	Iceland	Kópavogur
--	6C	No	N/A	Iceland	Reykjavík
--	6A	No	N/A	Iceland	Reykjavík
--	6C	No	N/A	Iceland	Reykjavík

--	6C	No	N/A	Iceland	Reykjavík
--	6C	No	N/A	Iceland	Reykjavík
--	6A	No	N/A	Iceland	Reykjavík
--	6A	No	N/A	Iceland	Reykjavík
--	6A	No	N/A	Iceland	Reykjavík
--	6Bii	No	N/A	Iceland	Reykjavík
--	6Bii	No	N/A	Iceland	Reykjavík
--	6A	Yes	6A/6C	Iceland	Reykjavík
--	6C	No	N/A	Iceland	Reykjavík
--	6C	No	N/A	Iceland	Reykjavík
--	6C	No	N/A	Iceland	Reykjavík
--	6A	No	N/A	Iceland	Reykjavík
--	6A	No	N/A	Iceland	Kópavogur
--	6A	No	N/A	Iceland	Reykjavík
--	6A	No	N/A	Iceland	Reykjavík
--	6C	Yes	6C/6D	Iceland	Reykjavík
--	6A	No	N/A	Iceland	Reykjavík
--	6A	No	N/A	Iceland	Garðabær
--	6A	No	N/A	Iceland	Hafnarfjörður
--	6C	No	N/A	Iceland	Mosfellsbær
--	6B	No	N/A	Iceland	Vestmannaeyjum
--	6Bii	No	N/A	Iceland	Vogum
--	6Bii	No	N/A	Iceland	Reykjavík
--	6C	No	N/A	Iceland	Reykjavík
--	6A	No	N/A	Iceland	Kópavogur
--	6A	No	N/A	Iceland	Garðabær
--	6B	No	N/A	Iceland	Kópavogur
--	6A	No	N/A	Iceland	Reykjavík
--	6A	No	N/A	Iceland	Hafnarfjörður
--	6B	No	N/A	Iceland	Hafnarfjörður
--	6B	No	N/A	Iceland	Hafnarfjörður
--	6A	No	N/A	Iceland	Hafnarfjörður
--	6B	No	N/A	Iceland	Hafnarfjörður
--	6C	No	N/A	Iceland	Hafnarfjörður
--	6C	No	N/A	Iceland	Hafnarfjörður
--	6B	No	N/A	Iceland	Kópavogur
--	6B	No	N/A	Iceland	Kópavogur
--	6A	No	N/A	Iceland	Kópavogur
--	6Bii	No	N/A	Iceland	Kópavogur
--	6B	No	N/A	Iceland	Kópavogur
--	6A	No	N/A	Iceland	Kópavogur
--	6A	No	N/A	Iceland	Kópavogur
--	6A	No	N/A	Iceland	Kópavogur
--	6A	No	N/A	Iceland	Kópavogur
--	6A	No	N/A	Iceland	unknown
--	6A	No	N/A	Iceland	unknown
--	6C	No	N/A	Iceland	unknown
--	6B	No	N/A	Iceland	unknown
--	6A	No	N/A	Iceland	unknown
--	6A	No	N/A	Iceland	unknown
--	6A	Yes	6A/6Bii	Iceland	unknown
--	6C	No	N/A	Iceland	unknown
--	6B	No	N/A	Iceland	unknown
--	6B	No	N/A	Iceland	unknown
--	6A	No	N/A	Iceland	unknown
--	6C	No	N/A	Iceland	unknown
--	6C	No	N/A	Iceland	unknown
--	6A	No	N/A	Iceland	unknown
--	6C	No	N/A	Iceland	unknown

--	6B	No	N/A	Iceland	unknown
--	6C	No	N/A	Iceland	unknown
--	6B	No	N/A	Iceland	unknown

carriage/disease	Source	Year	Publication	Reference	vaccine status
disease	unknown	1987	Capsule switching	PMID: 23175765	pre
disease	blood	1998	Capsule switching	PMID: 23175765	pre
disease	unknown	1997	Capsule switching	PMID: 23175765	pre
disease	pus	1988	Capsule switching	PMID: 23175765	pre
disease	blood	2005	Capsule switching	PMID: 23175765	pre
disease	blood	1990	Core Genome	PMID: 25144616	pre
carriage	nasopharynx	1978	Core Genome	PMID: 25144616	pre
carriage	nasopharynx	1978	Core Genome	PMID: 25144616	pre
carriage	nasopharynx	1978	Core Genome	PMID: 25144616	pre
carriage	nasopharynx	1978	Core Genome	PMID: 25144616	pre
carriage	nasopharynx	1978	Core Genome	PMID: 25144616	pre
carriage	nasopharynx	1978	Core Genome	PMID: 25144616	pre
carriage	nasopharynx	1978	Core Genome	PMID: 25144616	pre
carriage	nasopharynx	1978	Core Genome	PMID: 25144616	pre
carriage	nasopharynx	1978	Core Genome	PMID: 25144616	pre
carriage	nasopharynx	1978	Core Genome	PMID: 25144616	pre
carriage	nasopharynx	1978	Core Genome	PMID: 25144616	pre
disease	CSF	1978	Core Genome	PMID: 25144616	pre
disease	CSF	1978	Core Genome	PMID: 25144616	pre
disease	CSF	1987	Core Genome	PMID: 25144616	pre
disease	unknown	unknown	Core Genome	PMID: 25144616	unknown
disease	unknown	unknown	Core Genome	PMID: 25144616	unknown
disease	unknown	unknown	Core Genome	PMID: 25144616	unknown
disease	CSF	1989	Core Genome	PMID: 25144616	pre
disease	sputum	1984	Core Genome	PMID: 25144616	pre
unknown	unknown	1983	Core Genome	PMID: 25144616	pre
disease	CSF	1984	Core Genome	PMID: 25144616	pre
unknown	unknown	1984	Core Genome	PMID: 25144616	pre
disease	blood	1985	Core Genome	PMID: 25144616	pre
unknown	unknown	1985	Core Genome	PMID: 25144616	pre
disease	blood	1985	Core Genome	PMID: 25144616	pre
unknown	unknown	1985	Core Genome	PMID: 25144616	pre
unknown	unknown	1985	Core Genome	PMID: 25144616	pre
unknown	unknown	1988	Core Genome	PMID: 25144616	pre
disease	CSF	1981	Core Genome	PMID: 25144616	pre
disease	blood	1990	Core Genome	PMID: 25144616	pre
disease	unknown	1988	Genbank (336)	N/A	pre
disease	blood	2006	Genbank (336)	N/A	post
disease	blood	1999	Genbank (336)	N/A	pre
disease	blood	2000	Genbank (336)	N/A	pre
disease	blood	2001	Genbank (336)	N/A	post
disease	blood	2002	Genbank (336)	N/A	post
disease	blood	2004	Genbank (336)	N/A	post
disease	blood	2005	Genbank (336)	N/A	post
disease	blood	2007	Genbank (336)	N/A	post
carriage	unknown	unknown	Genbank (336)	N/A	unknown
carriage	nasopharynx	unknown	Genbank (336)	N/A	unknown
carriage	nasopharynx	unknown	Genbank (336)	N/A	unknown
carriage	nasopharynx	2006-2011	Gladstone UK PCV	PMID: 25776920	unknown
carriage	nasopharynx	2006-2011	Gladstone UK PCV	PMID: 25776920	unknown
carriage	nasopharynx	2006-2011	Gladstone UK PCV	PMID: 25776920	unknown
carriage	nasopharynx	2006-2011	Gladstone UK PCV	PMID: 25776920	unknown
carriage	nasopharynx	2006-2011	Gladstone UK PCV	PMID: 25776920	unknown
carriage	nasopharynx	2006-2011	Gladstone UK PCV	PMID: 25776920	unknown
carriage	nasopharynx	2006-2011	Gladstone UK PCV	PMID: 25776920	unknown
carriage	nasopharynx	2006-2011	Gladstone UK PCV	PMID: 25776920	unknown
carriage	nasopharynx	2006-2011	Gladstone UK PCV	PMID: 25776920	unknown
carriage	nasopharynx	2006-2011	Gladstone UK PCV	PMID: 25776920	unknown

carriage	nasopharynx	2006-2011	Gladstone UK PCV	PMID: 25776920	unknown
carriage	nasopharynx	2006-2011	Gladstone UK PCV	PMID: 25776920	unknown
carriage	nasopharynx	2006-2011	Gladstone UK PCV	PMID: 25776920	unknown
carriage	nasopharynx	2006-2011	Gladstone UK PCV	PMID: 25776920	unknown
carriage	nasopharynx	2006-2011	Gladstone UK PCV	PMID: 25776920	unknown
carriage	nasopharynx	2006-2011	Gladstone UK PCV	PMID: 25776920	unknown
carriage	nasopharynx	2006-2011	Gladstone UK PCV	PMID: 25776920	unknown
carriage	nasopharynx	2006-2011	Gladstone UK PCV	PMID: 25776920	unknown
carriage	nasopharynx	2006-2011	Gladstone UK PCV	PMID: 25776920	unknown
carriage	nasopharynx	2006-2011	Gladstone UK PCV	PMID: 25776920	unknown
carriage	nasopharynx	2006-2011	Gladstone UK PCV	PMID: 25776920	unknown
carriage	nasopharynx	2006-2011	Gladstone UK PCV	PMID: 25776920	unknown
carriage	nasopharynx	2006-2011	Gladstone UK PCV	PMID: 25776920	unknown
carriage	nasopharynx	2006-2011	Gladstone UK PCV	PMID: 25776920	unknown
carriage	nasopharynx	2006-2011	Gladstone UK PCV	PMID: 25776920	unknown
carriage	nasopharynx	2006-2011	Gladstone UK PCV	PMID: 25776920	unknown
carriage	nasopharynx	2006-2011	Gladstone UK PCV	PMID: 25776920	unknown
carriage	nasopharynx	2006-2011	Gladstone UK PCV	PMID: 25776920	unknown
carriage	nasopharynx	2006-2011	Gladstone UK PCV	PMID: 25776920	unknown
carriage	nasopharynx	2006-2011	Gladstone UK PCV	PMID: 25776920	unknown
carriage	nasopharynx	2006-2011	Gladstone UK PCV	PMID: 25776920	unknown
carriage	nasopharynx	2006-2011	Gladstone UK PCV	PMID: 25776920	unknown
carriage	nasopharynx	2006-2011	Gladstone UK PCV	PMID: 25776920	unknown
carriage	nasopharynx	2006-2011	Gladstone UK PCV	PMID: 25776920	unknown
carriage	nasopharynx	2006-2011	Gladstone UK PCV	PMID: 25776920	unknown
carriage	nasopharynx	2006-2011	Gladstone UK PCV	PMID: 25776920	unknown
carriage	nasopharynx	2006-2011	Gladstone UK PCV	PMID: 25776920	unknown
carriage	nasopharynx	2006-2011	Gladstone UK PCV	PMID: 25776920	unknown
carriage	nasopharynx	2006-2011	Gladstone UK PCV	PMID: 25776920	unknown
carriage	nasopharynx	2006-2011	Gladstone UK PCV	PMID: 25776920	unknown
carriage	nasopharynx	2006-2011	Gladstone UK PCV	PMID: 25776920	unknown
carriage	nasopharynx	2006-2011	Gladstone UK PCV	PMID: 25776920	unknown
carriage	nasopharynx	2006-2011	Gladstone UK PCV	PMID: 25776920	unknown
carriage	nasopharynx	2006-2011	Gladstone UK PCV	PMID: 25776920	unknown
carriage	nasopharynx	2006-2011	Gladstone UK PCV	PMID: 25776920	unknown
carriage	nasopharynx	2006-2011	Gladstone UK PCV	PMID: 25776920	unknown
carriage	nasopharynx	2006-2011	Gladstone UK PCV	PMID: 25776920	unknown
carriage	nasopharynx	2006-2011	Gladstone UK PCV	PMID: 25776920	unknown
carriage	nasopharynx	2006-2011	Gladstone UK PCV	PMID: 25776920	unknown
carriage	nasopharynx	2006-2011	Gladstone UK PCV	PMID: 25776920	unknown
carriage	nasopharynx	2006-2011	Gladstone UK PCV	PMID: 25776920	unknown
carriage	nasopharynx	2006-2011	Gladstone UK PCV	PMID: 25776920	unknown
carriage	nasopharynx	2006-2011	Gladstone UK PCV	PMID: 25776920	unknown
carriage	nasopharynx	2006-2011	Gladstone UK PCV	PMID: 25776920	unknown
carriage	nasopharynx	2006-2011	Gladstone UK PCV	PMID: 25776920	unknown
carriage	nasopharynx	2006-2011	Gladstone UK PCV	PMID: 25776920	unknown
carriage	nasopharynx	2006-2011	Gladstone UK PCV	PMID: 25776920	unknown
carriage	nasopharynx	2006-2011	Gladstone UK PCV	PMID: 25776920	unknown
carriage	nasopharynx	2006-2011	Gladstone UK PCV	PMID: 25776920	unknown
carriage	nasopharynx	2006-2011	Gladstone UK PCV	PMID: 25776920	unknown
carriage	nasopharynx	2006-2011	Gladstone UK PCV	PMID: 25776920	unknown
carriage	nasopharynx	2006-2011	Gladstone UK PCV	PMID: 25776920	unknown
carriage	nasopharynx	2006-2011	Gladstone UK PCV	PMID: 25776920	unknown
carriage	nasopharynx	2006-2011	Gladstone UK PCV	PMID: 25776920	unknown
carriage	nasopharynx	2006-2011	Gladstone UK PCV	PMID: 25776920	unknown
carriage	nasopharynx	2006-2011	Gladstone UK PCV	PMID: 25776920	unknown
carriage	nasopharynx	2006-2011	Gladstone UK PCV	PMID: 25776920	unknown
carriage	nasopharynx	2006-2011	Gladstone UK PCV	PMID: 25776920	unknown
carriage	nasopharynx	2006-2011	Gladstone UK PCV	PMID: 25776920	unknown

carriage	nasopharynx	2006-2011	Gladstone UK PCV	PMID: 25776920	unknown
carriage	nasopharynx	2006-2011	Gladstone UK PCV	PMID: 25776920	unknown
carriage	nasopharynx	2006-2011	Gladstone UK PCV	PMID: 25776920	unknown
carriage	nasopharynx	2006-2011	Gladstone UK PCV	PMID: 25776920	unknown
carriage	nasopharynx	2006-2011	Gladstone UK PCV	PMID: 25776920	unknown
carriage	nasopharynx	2006-2011	Gladstone UK PCV	PMID: 25776920	unknown
carriage	nasopharynx	2006-2011	Gladstone UK PCV	PMID: 25776920	unknown
carriage	nasopharynx	2006-2011	Gladstone UK PCV	PMID: 25776920	unknown
carriage	nasopharynx	2006-2011	Gladstone UK PCV	PMID: 25776920	unknown
carriage	nasopharynx	2006-2011	Gladstone UK PCV	PMID: 25776920	unknown
carriage	nasopharynx	2006-2011	Gladstone UK PCV	PMID: 25776920	unknown
carriage	nasopharynx	2006-2011	Gladstone UK PCV	PMID: 25776920	unknown
carriage	nasopharynx	2006-2011	Gladstone UK PCV	PMID: 25776920	unknown
carriage	nasopharynx	2006-2011	Gladstone UK PCV	PMID: 25776920	unknown
carriage	nasopharynx	2006-2011	Gladstone UK PCV	PMID: 25776920	unknown
carriage	nasopharynx	2006-2011	Gladstone UK PCV	PMID: 25776920	unknown
carriage	nasopharynx	2006-2011	Gladstone UK PCV	PMID: 25776920	unknown
carriage	nasopharynx	2006-2011	Gladstone UK PCV	PMID: 25776920	unknown
carriage	nasopharynx	2006-2011	Gladstone UK PCV	PMID: 25776920	unknown
carriage	nasopharynx	2006-2011	Gladstone UK PCV	PMID: 25776920	unknown
carriage	nasopharynx	2006-2011	Gladstone UK PCV	PMID: 25776920	unknown
carriage	nasopharynx	2006-2011	Gladstone UK PCV	PMID: 25776920	unknown
carriage	nasopharynx	2006-2011	Gladstone UK PCV	PMID: 25776920	unknown
carriage	nasopharynx	2006-2011	Gladstone UK PCV	PMID: 25776920	unknown
carriage	nasopharynx	2006-2011	Gladstone UK PCV	PMID: 25776920	unknown
carriage	nasopharynx	2006-2011	Gladstone UK PCV	PMID: 25776920	unknown
carriage	nasopharynx	2006-2011	Gladstone UK PCV	PMID: 25776920	unknown
carriage	nasopharynx	2006-2011	Gladstone UK PCV	PMID: 25776920	unknown
carriage	nasopharynx	2006-2011	Gladstone UK PCV	PMID: 25776920	unknown
carriage	nasopharynx	2006-2011	Gladstone UK PCV	PMID: 25776920	unknown
carriage	nasopharynx	2006-2011	Gladstone UK PCV	PMID: 25776920	unknown
carriage	nasopharynx	2006-2011	Gladstone UK PCV	PMID: 25776920	unknown
carriage	nasopharynx	2006-2011	Gladstone UK PCV	PMID: 25776920	unknown
carriage	nasopharynx	2006-2011	Gladstone UK PCV	PMID: 25776920	unknown
carriage	nasopharynx	2006-2011	Gladstone UK PCV	PMID: 25776920	unknown
carriage	nasopharynx	2006-2011	Gladstone UK PCV	PMID: 25776920	unknown
carriage	nasopharynx	2006-2011	Gladstone UK PCV	PMID: 25776920	unknown
carriage	nasopharynx	2006-2011	Gladstone UK PCV	PMID: 25776920	unknown
carriage	nasopharynx	2006-2011	Gladstone UK PCV	PMID: 25776920	unknown
carriage	nasopharynx	2006-2011	Gladstone UK PCV	PMID: 25776920	unknown
carriage	nasopharynx	2006-2011	Gladstone UK PCV	PMID: 25776920	unknown
carriage	nasopharynx	2006-2011	Gladstone UK PCV	PMID: 25776920	unknown
carriage	nasopharynx	2006-2011	Gladstone UK PCV	PMID: 25776920	unknown
carriage	nasopharynx	2006-2011	Gladstone UK PCV	PMID: 25776920	unknown
carriage	nasopharynx	2006-2011	Gladstone UK PCV	PMID: 25776920	unknown
carriage	nasopharynx	2006-2011	Gladstone UK PCV	PMID: 25776920	unknown
carriage	nasopharynx	2006-2011	Gladstone UK PCV	PMID: 25776920	unknown
carriage	nasopharynx	2006-2011	Gladstone UK PCV	PMID: 25776920	unknown
carriage	nasopharynx	2006-2011	Gladstone UK PCV	PMID: 25776920	unknown
carriage	nasopharynx	2006-2011	Gladstone UK PCV	PMID: 25776920	unknown
carriage	nasopharynx	2006-2011	Gladstone UK PCV	PMID: 25776920	unknown
carriage	nasopharynx	2006-2011	Gladstone UK PCV	PMID: 25776920	unknown
carriage	nasopharynx	2006-2011	Gladstone UK PCV	PMID: 25776920	unknown
carriage	nasopharynx	2006-2011	Gladstone UK PCV	PMID: 25776920	unknown
carriage	nasopharynx	2006-2011	Gladstone UK PCV	PMID: 25776920	unknown
carriage	nasopharynx	2006-2011	Gladstone UK PCV	PMID: 25776920	unknown
carriage	nasopharynx	2006-2011	Gladstone UK PCV	PMID: 25776920	unknown
unknown	unknown	1952	Historical	unknown	pre
unknown	unknown	1939	Historical	unknown	pre

carriage	nasopharynx	2008	Maela	PMID: 24509479	pre
carriage	nasopharynx	2008	Maela	PMID: 24509479	pre
carriage	nasopharynx	2008	Maela	PMID: 24509479	pre
carriage	nasopharynx	2008	Maela	PMID: 24509479	pre
carriage	nasopharynx	2008	Maela	PMID: 24509479	pre
carriage	nasopharynx	2008	Maela	PMID: 24509479	pre
carriage	nasopharynx	2008	Maela	PMID: 24509479	pre
carriage	nasopharynx	2008	Maela	PMID: 24509479	pre
carriage	nasopharynx	2008	Maela	PMID: 24509479	pre
carriage	nasopharynx	2008	Maela	PMID: 24509479	pre
carriage	nasopharynx	2009	Maela	PMID: 24509479	pre
carriage	nasopharynx	2009	Maela	PMID: 24509479	pre
carriage	nasopharynx	2009	Maela	PMID: 24509479	pre
carriage	nasopharynx	2009	Maela	PMID: 24509479	pre
carriage	nasopharynx	2009	Maela	PMID: 24509479	pre
carriage	nasopharynx	2009	Maela	PMID: 24509479	pre
carriage	nasopharynx	2009	Maela	PMID: 24509479	pre
carriage	nasopharynx	2009	Maela	PMID: 24509479	pre
carriage	nasopharynx	2009	Maela	PMID: 24509479	pre
carriage	nasopharynx	2009	Maela	PMID: 24509479	pre
carriage	nasopharynx	2009	Maela	PMID: 24509479	pre
carriage	nasopharynx	2009	Maela	PMID: 24509479	pre
carriage	nasopharynx	2009	Maela	PMID: 24509479	pre
carriage	nasopharynx	2009	Maela	PMID: 24509479	pre
carriage	nasopharynx	2009	Maela	PMID: 24509479	pre
carriage	nasopharynx	2009	Maela	PMID: 24509479	pre
carriage	nasopharynx	2009	Maela	PMID: 24509479	pre
carriage	nasopharynx	2009	Maela	PMID: 24509479	pre
carriage	nasopharynx	2009	Maela	PMID: 24509479	pre
carriage	nasopharynx	2009	Maela	PMID: 24509479	pre
carriage	nasopharynx	2009	Maela	PMID: 24509479	pre
carriage	nasopharynx	2009	Maela	PMID: 24509479	pre
carriage	nasopharynx	2009	Maela	PMID: 24509479	pre
carriage	nasopharynx	2009	Maela	PMID: 24509479	pre
carriage	nasopharynx	2009	Maela	PMID: 24509479	pre
carriage	nasopharynx	2009	Maela	PMID: 24509479	pre
carriage	nasopharynx	2009	Maela	PMID: 24509479	pre
carriage	nasopharynx	2009	Maela	PMID: 24509479	pre
carriage	nasopharynx	2009	Maela	PMID: 24509479	pre
carriage	nasopharynx	2009	Maela	PMID: 24509479	pre
carriage	nasopharynx	2007	Maela	PMID: 24509479	pre
carriage	nasopharynx	2009	Maela	PMID: 24509479	pre
carriage	nasopharynx	2009	Maela	PMID: 24509479	pre
carriage	nasopharynx	2008	Maela	PMID: 24509479	pre
carriage	nasopharynx	2009	Maela	PMID: 24509479	pre
carriage	nasopharynx	2009	Maela	PMID: 24509479	pre
carriage	nasopharynx	2009	Maela	PMID: 24509479	pre
carriage	nasopharynx	2009	Maela	PMID: 24509479	pre
carriage	nasopharynx	2009	Maela	PMID: 24509479	pre
carriage	nasopharynx	2009	Maela	PMID: 24509479	pre
carriage	nasopharynx	2009	Maela	PMID: 24509479	pre
carriage	nasopharynx	2009	Maela	PMID: 24509479	pre
carriage	nasopharynx	2009	Maela	PMID: 24509479	pre
carriage	nasopharynx	2009	Maela	PMID: 24509479	pre
carriage	nasopharynx	2009	Maela	PMID: 24509479	pre
carriage	nasopharynx	2009	Maela	PMID: 24509479	pre
carriage	nasopharynx	2009	Maela	PMID: 24509479	pre
carriage	nasopharynx	2009	Maela	PMID: 24509479	pre
carriage	nasopharynx	2007	Maela	PMID: 24509479	pre

carriage	nasopharynx	2009	Maela	PMID: 24509479	pre
carriage	nasopharynx	2009	Maela	PMID: 24509479	pre
carriage	nasopharynx	2009	Maela	PMID: 24509479	pre
carriage	nasopharynx	2009	Maela	PMID: 24509479	pre
carriage	nasopharynx	2009	Maela	PMID: 24509479	pre
carriage	nasopharynx	2009	Maela	PMID: 24509479	pre
carriage	nasopharynx	2009	Maela	PMID: 24509479	pre
carriage	nasopharynx	2009	Maela	PMID: 24509479	pre
carriage	nasopharynx	2009	Maela	PMID: 24509479	pre
carriage	nasopharynx	2009	Maela	PMID: 24509479	pre
carriage	nasopharynx	2009	Maela	PMID: 24509479	pre
carriage	nasopharynx	2009	Maela	PMID: 24509479	pre
carriage	nasopharynx	2009	Maela	PMID: 24509479	pre
carriage	nasopharynx	2009	Maela	PMID: 24509479	pre
carriage	nasopharynx	2009	Maela	PMID: 24509479	pre
carriage	nasopharynx	2009	Maela	PMID: 24509479	pre
carriage	nasopharynx	2009	Maela	PMID: 24509479	pre
carriage	nasopharynx	2009	Maela	PMID: 24509479	pre
carriage	nasopharynx	2009	Maela	PMID: 24509479	pre
carriage	nasopharynx	2009	Maela	PMID: 24509479	pre
carriage	nasopharynx	2009	Maela	PMID: 24509479	pre
carriage	nasopharynx	2008	Maela	PMID: 24509479	pre
carriage	nasopharynx	2009	Maela	PMID: 24509479	pre
carriage	nasopharynx	2007	Maela	PMID: 24509479	pre
carriage	nasopharynx	2009	Maela	PMID: 24509479	pre
carriage	nasopharynx	2009	Maela	PMID: 24509479	pre
carriage	nasopharynx	2009	Maela	PMID: 24509479	pre
carriage	nasopharynx	2009	Maela	PMID: 24509479	pre
carriage	nasopharynx	2009	Maela	PMID: 24509479	pre
carriage	nasopharynx	2009	Maela	PMID: 24509479	pre
carriage	nasopharynx	2009	Maela	PMID: 24509479	pre
carriage	nasopharynx	2009	Maela	PMID: 24509479	pre
carriage	nasopharynx	2009	Maela	PMID: 24509479	pre
carriage	nasopharynx	2008	Maela	PMID: 24509479	pre
carriage	nasopharynx	2009	Maela	PMID: 24509479	pre
carriage	nasopharynx	2009	Maela	PMID: 24509479	pre
carriage	nasopharynx	2009	Maela	PMID: 24509479	pre
carriage	nasopharynx	2009	Maela	PMID: 24509479	pre
carriage	nasopharynx	2009	Maela	PMID: 24509479	pre
carriage	nasopharynx	2009	Maela	PMID: 24509479	pre
carriage	nasopharynx	2009	Maela	PMID: 24509479	pre
carriage	nasopharynx	2009	Maela	PMID: 24509479	pre
carriage	nasopharynx	2009	Maela	PMID: 24509479	pre
carriage	nasopharynx	2009	Maela	PMID: 24509479	pre
carriage	nasopharynx	2009	Maela	PMID: 24509479	pre
carriage	nasopharynx	2009	Maela	PMID: 24509479	pre
carriage	nasopharynx	2009	Maela	PMID: 24509479	pre
carriage	nasopharynx	2009	Maela	PMID: 24509479	pre
carriage	nasopharynx	2009	Maela	PMID: 24509479	pre
carriage	nasopharynx	2009	Maela	PMID: 24509479	pre
carriage	nasopharynx	2009	Maela	PMID: 24509479	pre
carriage	nasopharynx	2009	Maela	PMID: 24509479	pre
carriage	nasopharynx	2009	Maela	PMID: 24509479	pre
carriage	nasopharynx	2009	Maela	PMID: 24509479	pre
carriage	nasopharynx	2009	Maela	PMID: 24509479	pre
carriage	nasopharynx	2009	Maela	PMID: 24509479	pre
carriage	nasopharynx	2009	Maela	PMID: 24509479	pre
carriage	nasopharynx	2009	Maela	PMID: 24509479	pre
carriage	nasopharynx	2009	Maela	PMID: 24509479	pre

carriage	nasopharynx	2009	Maela	PMID: 24509479	pre
carriage	nasopharynx	2009	Maela	PMID: 24509479	pre
carriage	nasopharynx	2009	Maela	PMID: 24509479	pre
carriage	nasopharynx	2009	Maela	PMID: 24509479	pre
carriage	nasopharynx	2009	Maela	PMID: 24509479	pre
carriage	nasopharynx	2009	Maela	PMID: 24509479	pre
carriage	nasopharynx	2009	Maela	PMID: 24509479	pre
carriage	nasopharynx	2009	Maela	PMID: 24509479	pre
carriage	nasopharynx	2009	Maela	PMID: 24509479	pre
carriage	nasopharynx	2009	Maela	PMID: 24509479	pre
carriage	nasopharynx	2009	Maela	PMID: 24509479	pre
carriage	nasopharynx	2009	Maela	PMID: 24509479	pre
carriage	nasopharynx	2009	Maela	PMID: 24509479	pre
carriage	nasopharynx	2009	Maela	PMID: 24509479	pre
carriage	nasopharynx	2009	Maela	PMID: 24509479	pre
carriage	nasopharynx	2009	Maela	PMID: 24509479	pre
carriage	nasopharynx	2009	Maela	PMID: 24509479	pre
carriage	nasopharynx	2009	Maela	PMID: 24509479	pre
carriage	nasopharynx	2008	Maela	PMID: 24509479	pre
carriage	nasopharynx	2009	Maela	PMID: 24509479	pre
carriage	nasopharynx	2009	Maela	PMID: 24509479	pre
carriage	nasopharynx	2009	Maela	PMID: 24509479	pre
carriage	nasopharynx	2009	Maela	PMID: 24509479	pre
carriage	nasopharynx	2009	Maela	PMID: 24509479	pre
carriage	nasopharynx	2009	Maela	PMID: 24509479	pre
carriage	nasopharynx	2009	Maela	PMID: 24509479	pre
carriage	nasopharynx	2009	Maela	PMID: 24509479	pre
carriage	nasopharynx	2009	Maela	PMID: 24509479	pre
carriage	nasopharynx	2009	Maela	PMID: 24509479	pre
carriage	nasopharynx	2009	Maela	PMID: 24509479	pre
carriage	nasopharynx	2009	Maela	PMID: 24509479	pre
carriage	nasopharynx	2009	Maela	PMID: 24509479	pre
carriage	nasopharynx	2009	Maela	PMID: 24509479	pre
carriage	nasopharynx	2009	Maela	PMID: 24509479	pre
carriage	nasopharynx	2008	Maela	PMID: 24509479	pre
carriage	nasopharynx	2009	Maela	PMID: 24509479	pre
carriage	nasopharynx	2009	Maela	PMID: 24509479	pre
carriage	nasopharynx	2008	Maela	PMID: 24509479	pre
carriage	nasopharynx	2009	Maela	PMID: 24509479	pre
carriage	nasopharynx	2009	Maela	PMID: 24509479	pre
carriage	nasopharynx	2009	Maela	PMID: 24509479	pre
carriage	nasopharynx	2009	Maela	PMID: 24509479	pre
carriage	nasopharynx	2009	Maela	PMID: 24509479	pre
carriage	nasopharynx	2009	Maela	PMID: 24509479	pre
carriage	nasopharynx	2009	Maela	PMID: 24509479	pre
carriage	nasopharynx	2009	Maela	PMID: 24509479	pre
carriage	nasopharynx	2010	Maela	PMID: 24509479	pre
carriage	nasopharynx	2008	Maela	PMID: 24509479	pre
carriage	nasopharynx	2010	Maela	PMID: 24509479	pre
carriage	nasopharynx	2010	Maela	PMID: 24509479	pre
carriage	nasopharynx	2010	Maela	PMID: 24509479	pre
carriage	nasopharynx	2010	Maela	PMID: 24509479	pre
carriage	nasopharynx	2010	Maela	PMID: 24509479	pre
carriage	nasopharynx	2010	Maela	PMID: 24509479	pre
carriage	nasopharynx	2010	Maela	PMID: 24509479	pre
carriage	nasopharynx	2010	Maela	PMID: 24509479	pre
carriage	nasopharynx	2010	Maela	PMID: 24509479	pre
carriage	nasopharynx	2010	Maela	PMID: 24509479	pre
carriage	nasopharynx	2010	Maela	PMID: 24509479	pre

carriage	nasopharynx	2010	Maela	PMID: 24509479	pre
carriage	nasopharynx	2010	Maela	PMID: 24509479	pre
carriage	nasopharynx	2010	Maela	PMID: 24509479	pre
carriage	nasopharynx	2010	Maela	PMID: 24509479	pre
carriage	nasopharynx	2010	Maela	PMID: 24509479	pre
carriage	nasopharynx	2010	Maela	PMID: 24509479	pre
carriage	nasopharynx	2010	Maela	PMID: 24509479	pre
carriage	nasopharynx	2010	Maela	PMID: 24509479	pre
carriage	nasopharynx	2010	Maela	PMID: 24509479	pre
carriage	nasopharynx	2010	Maela	PMID: 24509479	pre
carriage	nasopharynx	2010	Maela	PMID: 24509479	pre
carriage	nasopharynx	2010	Maela	PMID: 24509479	pre
carriage	nasopharynx	2010	Maela	PMID: 24509479	pre
carriage	nasopharynx	2010	Maela	PMID: 24509479	pre
carriage	nasopharynx	2010	Maela	PMID: 24509479	pre
carriage	nasopharynx	2010	Maela	PMID: 24509479	pre
carriage	nasopharynx	2010	Maela	PMID: 24509479	pre
carriage	nasopharynx	2010	Maela	PMID: 24509479	pre
carriage	nasopharynx	2010	Maela	PMID: 24509479	pre
carriage	nasopharynx	2010	Maela	PMID: 24509479	pre
carriage	nasopharynx	2010	Maela	PMID: 24509479	pre
carriage	nasopharynx	2010	Maela	PMID: 24509479	pre
carriage	nasopharynx	2010	Maela	PMID: 24509479	pre
carriage	nasopharynx	2010	Maela	PMID: 24509479	pre
carriage	nasopharynx	2010	Maela	PMID: 24509479	pre
carriage	nasopharynx	2010	Maela	PMID: 24509479	pre
carriage	nasopharynx	2010	Maela	PMID: 24509479	pre
carriage	nasopharynx	2010	Maela	PMID: 24509479	pre
carriage	nasopharynx	2010	Maela	PMID: 24509479	pre
carriage	nasopharynx	2010	Maela	PMID: 24509479	pre
carriage	nasopharynx	2010	Maela	PMID: 24509479	pre
carriage	nasopharynx	2010	Maela	PMID: 24509479	pre
carriage	nasopharynx	2010	Maela	PMID: 24509479	pre
carriage	nasopharynx	2010	Maela	PMID: 24509479	pre
carriage	nasopharynx	2010	Maela	PMID: 24509479	pre
carriage	nasopharynx	2010	Maela	PMID: 24509479	pre
carriage	nasopharynx	2010	Maela	PMID: 24509479	pre
carriage	nasopharynx	2010	Maela	PMID: 24509479	pre
carriage	nasopharynx	2010	Maela	PMID: 24509479	pre
carriage	nasopharynx	2010	Maela	PMID: 24509479	pre
carriage	nasopharynx	2010	Maela	PMID: 24509479	pre
carriage	nasopharynx	2010	Maela	PMID: 24509479	pre
carriage	nasopharynx	2010	Maela	PMID: 24509479	pre
carriage	nasopharynx	2010	Maela	PMID: 24509479	pre
carriage	nasopharynx	2010	Maela	PMID: 24509479	pre
carriage	nasopharynx	2010	Maela	PMID: 24509479	pre
carriage	nasopharynx	2010	Maela	PMID: 24509479	pre
carriage	nasopharynx	2010	Maela	PMID: 24509479	pre
carriage	nasopharynx	2010	Maela	PMID: 24509479	pre
carriage	nasopharynx	2010	Maela	PMID: 24509479	pre
carriage	nasopharynx	2010	Maela	PMID: 24509479	pre
carriage	nasopharynx	2010	Maela	PMID: 24509479	pre
carriage	nasopharynx	2010	Maela	PMID: 24509479	pre
carriage	nasopharynx	2010	Maela	PMID: 24509479	pre
carriage	nasopharynx	2010	Maela	PMID: 24509479	pre
carriage	nasopharynx	2010	Maela	PMID: 24509479	pre
carriage	nasopharynx	2010	Maela	PMID: 24509479	pre
carriage	nasopharynx	2010	Maela	PMID: 24509479	pre
carriage	nasopharynx	2010	Maela	PMID: 24509479	pre

carriage	nasopharynx	2010	Maela	PMID: 24509479	pre
carriage	nasopharynx	2010	Maela	PMID: 24509479	pre
carriage	nasopharynx	2010	Maela	PMID: 24509479	pre
carriage	nasopharynx	2010	Maela	PMID: 24509479	pre
carriage	nasopharynx	2010	Maela	PMID: 24509479	pre
carriage	nasopharynx	2010	Maela	PMID: 24509479	pre
carriage	nasopharynx	2010	Maela	PMID: 24509479	pre
carriage	nasopharynx	2010	Maela	PMID: 24509479	pre
carriage	nasopharynx	2010	Maela	PMID: 24509479	pre
carriage	nasopharynx	2010	Maela	PMID: 24509479	pre
carriage	nasopharynx	2010	Maela	PMID: 24509479	pre
carriage	nasopharynx	2010	Maela	PMID: 24509479	pre
carriage	nasopharynx	2010	Maela	PMID: 24509479	pre
carriage	nasopharynx	2010	Maela	PMID: 24509479	pre
carriage	nasopharynx	2010	Maela	PMID: 24509479	pre
carriage	nasopharynx	2010	Maela	PMID: 24509479	pre
carriage	nasopharynx	2010	Maela	PMID: 24509479	pre
carriage	nasopharynx	2010	Maela	PMID: 24509479	pre
carriage	nasopharynx	2010	Maela	PMID: 24509479	pre
carriage	nasopharynx	2010	Maela	PMID: 24509479	pre
carriage	nasopharynx	2010	Maela	PMID: 24509479	pre
carriage	nasopharynx	2010	Maela	PMID: 24509479	pre
carriage	nasopharynx	2010	Maela	PMID: 24509479	pre
carriage	nasopharynx	2010	Maela	PMID: 24509479	pre
carriage	nasopharynx	2010	Maela	PMID: 24509479	pre
carriage	nasopharynx	2010	Maela	PMID: 24509479	pre
carriage	nasopharynx	2007	Maela	PMID: 24509479	pre
carriage	nasopharynx	2010	Maela	PMID: 24509479	pre
carriage	nasopharynx	2010	Maela	PMID: 24509479	pre
carriage	nasopharynx	2010	Maela	PMID: 24509479	pre
carriage	nasopharynx	2010	Maela	PMID: 24509479	pre
carriage	nasopharynx	2010	Maela	PMID: 24509479	pre
carriage	nasopharynx	2010	Maela	PMID: 24509479	pre
carriage	nasopharynx	2010	Maela	PMID: 24509479	pre
carriage	nasopharynx	2010	Maela	PMID: 24509479	pre
carriage	nasopharynx	2010	Maela	PMID: 24509479	pre
carriage	nasopharynx	2010	Maela	PMID: 24509479	pre
carriage	nasopharynx	2010	Maela	PMID: 24509479	pre
carriage	nasopharynx	2010	Maela	PMID: 24509479	pre
carriage	nasopharynx	2010	Maela	PMID: 24509479	pre
carriage	nasopharynx	2010	Maela	PMID: 24509479	pre
carriage	nasopharynx	2010	Maela	PMID: 24509479	pre
carriage	nasopharynx	2008	Maela	PMID: 24509479	pre
carriage	nasopharynx	2010	Maela	PMID: 24509479	pre
carriage	nasopharynx	2010	Maela	PMID: 24509479	pre
carriage	nasopharynx	2010	Maela	PMID: 24509479	pre
carriage	nasopharynx	2010	Maela	PMID: 24509479	pre
carriage	nasopharynx	2010	Maela	PMID: 24509479	pre
carriage	nasopharynx	2010	Maela	PMID: 24509479	pre
carriage	nasopharynx	2010	Maela	PMID: 24509479	pre
carriage	nasopharynx	2010	Maela	PMID: 24509479	pre
carriage	nasopharynx	2010	Maela	PMID: 24509479	pre
carriage	nasopharynx	2010	Maela	PMID: 24509479	pre
carriage	nasopharynx	2010	Maela	PMID: 24509479	pre
carriage	nasopharynx	2010	Maela	PMID: 24509479	pre
carriage	nasopharynx	2010	Maela	PMID: 24509479	pre
carriage	nasopharynx	2010	Maela	PMID: 24509479	pre
carriage	nasopharynx	2010	Maela	PMID: 24509479	pre
carriage	nasopharynx	2010	Maela	PMID: 24509479	pre

carriage	nasopharynx	2010	Maela	PMID: 24509479	pre
carriage	nasopharynx	2010	Maela	PMID: 24509479	pre
carriage	nasopharynx	2010	Maela	PMID: 24509479	pre
carriage	nasopharynx	2010	Maela	PMID: 24509479	pre
carriage	nasopharynx	2010	Maela	PMID: 24509479	pre
carriage	nasopharynx	2010	Maela	PMID: 24509479	pre
carriage	nasopharynx	2010	Maela	PMID: 24509479	pre
carriage	nasopharynx	2010	Maela	PMID: 24509479	pre
carriage	nasopharynx	2010	Maela	PMID: 24509479	pre
carriage	nasopharynx	2010	Maela	PMID: 24509479	pre
carriage	nasopharynx	2010	Maela	PMID: 24509479	pre
carriage	nasopharynx	2010	Maela	PMID: 24509479	pre
carriage	nasopharynx	2010	Maela	PMID: 24509479	pre
carriage	nasopharynx	2010	Maela	PMID: 24509479	pre
carriage	nasopharynx	2010	Maela	PMID: 24509479	pre
carriage	nasopharynx	2010	Maela	PMID: 24509479	pre
carriage	nasopharynx	2010	Maela	PMID: 24509479	pre
carriage	nasopharynx	2010	Maela	PMID: 24509479	pre
carriage	nasopharynx	2010	Maela	PMID: 24509479	pre
carriage	nasopharynx	2010	Maela	PMID: 24509479	pre
carriage	nasopharynx	2010	Maela	PMID: 24509479	pre
carriage	nasopharynx	2010	Maela	PMID: 24509479	pre
carriage	nasopharynx	2010	Maela	PMID: 24509479	pre
carriage	nasopharynx	2010	Maela	PMID: 24509479	pre
carriage	nasopharynx	2010	Maela	PMID: 24509479	pre
carriage	nasopharynx	2010	Maela	PMID: 24509479	pre
carriage	nasopharynx	2010	Maela	PMID: 24509479	pre
carriage	nasopharynx	2008	Maela	PMID: 24509479	pre
carriage	nasopharynx	2010	Maela	PMID: 24509479	pre
carriage	nasopharynx	2010	Maela	PMID: 24509479	pre
carriage	nasopharynx	2010	Maela	PMID: 24509479	pre
carriage	nasopharynx	2010	Maela	PMID: 24509479	pre
carriage	nasopharynx	2009	Maela	PMID: 24509479	pre
carriage	nasopharynx	2009	Maela	PMID: 24509479	pre
carriage	nasopharynx	2009	Maela	PMID: 24509479	pre
carriage	nasopharynx	2009	Maela	PMID: 24509479	pre
carriage	nasopharynx	2008	Maela	PMID: 24509479	pre
carriage	nasopharynx	2010	Maela	PMID: 24509479	pre
carriage	nasopharynx	2010	Maela	PMID: 24509479	pre
carriage	nasopharynx	2010	Maela	PMID: 24509479	pre
carriage	nasopharynx	2008	Maela	PMID: 24509479	pre
carriage	nasopharynx	2010	Maela	PMID: 24509479	pre
carriage	nasopharynx	2008	Maela	PMID: 24509479	pre
carriage	nasopharynx	2008	Maela	PMID: 24509479	pre
carriage	nasopharynx	2008	Maela	PMID: 24509479	pre
carriage	nasopharynx	2008	Maela	PMID: 24509479	pre
carriage	nasopharynx	2008	Maela	PMID: 24509479	pre
carriage	nasopharynx	2008	Maela	PMID: 24509479	pre
carriage	nasopharynx	2008	Maela	PMID: 24509479	pre
carriage	nasopharynx	2008	Maela	PMID: 24509479	pre
carriage	nasopharynx	2008	Maela	PMID: 24509479	pre
carriage	nasopharynx	2008	Maela	PMID: 24509479	pre
carriage	nasopharynx	2008	Maela	PMID: 24509479	pre
carriage	nasopharynx	2008	Maela	PMID: 24509479	pre
carriage	nasopharynx	2008	Maela	PMID: 24509479	pre
carriage	nasopharynx	2008	Maela	PMID: 24509479	pre
carriage	nasopharynx	2008	Maela	PMID: 24509479	pre
carriage	nasopharynx	2008	Maela	PMID: 24509479	pre
carriage	nasopharynx	2008	Maela	PMID: 24509479	pre

carriage	nasopharynx	2008	Maela	PMID: 24509479	pre
carriage	nasopharynx	2008	Maela	PMID: 24509479	pre
carriage	nasopharynx	2008	Maela	PMID: 24509479	pre
carriage	nasopharynx	2008	Maela	PMID: 24509479	pre
carriage	nasopharynx	2008	Maela	PMID: 24509479	pre
carriage	nasopharynx	2008	Maela	PMID: 24509479	pre
carriage	nasopharynx	2008	Maela	PMID: 24509479	pre
carriage	nasopharynx	2008	Maela	PMID: 24509479	pre
carriage	nasopharynx	2008	Maela	PMID: 24509479	pre
carriage	nasopharynx	2008	Maela	PMID: 24509479	pre
carriage	nasopharynx	2008	Maela	PMID: 24509479	pre
carriage	nasopharynx	2008	Maela	PMID: 24509479	pre
carriage	nasopharynx	2008	Maela	PMID: 24509479	pre
carriage	nasopharynx	2008	Maela	PMID: 24509479	pre
carriage	nasopharynx	2008	Maela	PMID: 24509479	pre
carriage	nasopharynx	2008	Maela	PMID: 24509479	pre
carriage	nasopharynx	2008	Maela	PMID: 24509479	pre
carriage	nasopharynx	2008	Maela	PMID: 24509479	pre
carriage	nasopharynx	2008	Maela	PMID: 24509479	pre
carriage	nasopharynx	2008	Maela	PMID: 24509479	pre
carriage	nasopharynx	2008	Maela	PMID: 24509479	pre
carriage	nasopharynx	2008	Maela	PMID: 24509479	pre
carriage	nasopharynx	2008	Maela	PMID: 24509479	pre
carriage	nasopharynx	2008	Maela	PMID: 24509479	pre
carriage	nasopharynx	2008	Maela	PMID: 24509479	pre
carriage	nasopharynx	2008	Maela	PMID: 24509479	pre
carriage	nasopharynx	2008	Maela	PMID: 24509479	pre
carriage	nasopharynx	2008	Maela	PMID: 24509479	pre
carriage	nasopharynx	2008	Maela	PMID: 24509479	pre
carriage	nasopharynx	2008	Maela	PMID: 24509479	pre
carriage	nasopharynx	2008	Maela	PMID: 24509479	pre
carriage	nasopharynx	2008	Maela	PMID: 24509479	pre
carriage	nasopharynx	2008	Maela	PMID: 24509479	pre
carriage	nasopharynx	2008	Maela	PMID: 24509479	pre
carriage	nasopharynx	2008	Maela	PMID: 24509479	pre
carriage	nasopharynx	2008	Maela	PMID: 24509479	pre
carriage	nasopharynx	2008	Maela	PMID: 24509479	pre
carriage	nasopharynx	2008	Maela	PMID: 24509479	pre
carriage	nasopharynx	2008	Maela	PMID: 24509479	pre
carriage	nasopharynx	2008	Maela	PMID: 24509479	pre
carriage	nasopharynx	2008	Maela	PMID: 24509479	pre
carriage	nasopharynx	2008	Maela	PMID: 24509479	pre
carriage	nasopharynx	2001	Massachusetts	PMID: 23644493	post
carriage	nasopharynx	2001	Massachusetts	PMID: 23644493	post
carriage	nasopharynx	2001	Massachusetts	PMID: 23644493	post
carriage	nasopharynx	2001	Massachusetts	PMID: 23644493	post
carriage	nasopharynx	2001	Massachusetts	PMID: 23644493	post
carriage	nasopharynx	2001	Massachusetts	PMID: 23644493	post
carriage	nasopharynx	2001	Massachusetts	PMID: 23644493	post
carriage	nasopharynx	2001	Massachusetts	PMID: 23644493	post
carriage	nasopharynx	2001	Massachusetts	PMID: 23644493	post
carriage	nasopharynx	2001	Massachusetts	PMID: 23644493	post
carriage	nasopharynx	2001	Massachusetts	PMID: 23644493	post
carriage	nasopharynx	2001	Massachusetts	PMID: 23644493	post
carriage	nasopharynx	2001	Massachusetts	PMID: 23644493	post
carriage	nasopharynx	2001	Massachusetts	PMID: 23644493	post
carriage	nasopharynx	2001	Massachusetts	PMID: 23644493	post
carriage	nasopharynx	2001	Massachusetts	PMID: 23644493	post
carriage	nasopharynx	2001	Massachusetts	PMID: 23644493	post

carriage	nasopharynx	2001	Massachusetts	PMID: 23644493	post
carriage	nasopharynx	2001	Massachusetts	PMID: 23644493	post
carriage	nasopharynx	2001	Massachusetts	PMID: 23644493	post
carriage	nasopharynx	2001	Massachusetts	PMID: 23644493	post
carriage	nasopharynx	2001	Massachusetts	PMID: 23644493	post
carriage	nasopharynx	2001	Massachusetts	PMID: 23644493	post
carriage	nasopharynx	2001	Massachusetts	PMID: 23644493	post
carriage	nasopharynx	2001	Massachusetts	PMID: 23644493	post
carriage	nasopharynx	2001	Massachusetts	PMID: 23644493	post
carriage	nasopharynx	2001	Massachusetts	PMID: 23644493	post
carriage	nasopharynx	2001	Massachusetts	PMID: 23644493	post
carriage	nasopharynx	2001	Massachusetts	PMID: 23644493	post
carriage	nasopharynx	2001	Massachusetts	PMID: 23644493	post
carriage	nasopharynx	2001	Massachusetts	PMID: 23644493	post
carriage	nasopharynx	2001	Massachusetts	PMID: 23644493	post
carriage	nasopharynx	2001	Massachusetts	PMID: 23644493	post
carriage	nasopharynx	2001	Massachusetts	PMID: 23644493	post
carriage	nasopharynx	2001	Massachusetts	PMID: 23644493	post
carriage	nasopharynx	2001	Massachusetts	PMID: 23644493	post
carriage	nasopharynx	2001	Massachusetts	PMID: 23644493	post
carriage	nasopharynx	2001	Massachusetts	PMID: 23644493	post
carriage	nasopharynx	2001	Massachusetts	PMID: 23644493	post
carriage	nasopharynx	2007	Massachusetts	PMID: 23644493	post
carriage	nasopharynx	2007	Massachusetts	PMID: 23644493	post
carriage	nasopharynx	2007	Massachusetts	PMID: 23644493	post
carriage	nasopharynx	2007	Massachusetts	PMID: 23644493	post
carriage	nasopharynx	2007	Massachusetts	PMID: 23644493	post
carriage	nasopharynx	2007	Massachusetts	PMID: 23644493	post
carriage	nasopharynx	2007	Massachusetts	PMID: 23644493	post
carriage	nasopharynx	2007	Massachusetts	PMID: 23644493	post
carriage	nasopharynx	2007	Massachusetts	PMID: 23644493	post
carriage	nasopharynx	2007	Massachusetts	PMID: 23644493	post
carriage	nasopharynx	2007	Massachusetts	PMID: 23644493	post
carriage	nasopharynx	2007	Massachusetts	PMID: 23644493	post
carriage	nasopharynx	2007	Massachusetts	PMID: 23644493	post
carriage	nasopharynx	2007	Massachusetts	PMID: 23644493	post
carriage	nasopharynx	2007	Massachusetts	PMID: 23644493	post
carriage	nasopharynx	2007	Massachusetts	PMID: 23644493	post
carriage	nasopharynx	2007	Massachusetts	PMID: 23644493	post
carriage	nasopharynx	2007	Massachusetts	PMID: 23644493	post
carriage	nasopharynx	2007	Massachusetts	PMID: 23644493	post
carriage	nasopharynx	2007	Massachusetts	PMID: 23644493	post
carriage	nasopharynx	2007	Massachusetts	PMID: 23644493	post
carriage	nasopharynx	2007	Massachusetts	PMID: 23644493	post
carriage	nasopharynx	2007	Massachusetts	PMID: 23644493	post
carriage	nasopharynx	2007	Massachusetts	PMID: 23644493	post
carriage	nasopharynx	2007	Massachusetts	PMID: 23644493	post
carriage	nasopharynx	2007	Massachusetts	PMID: 23644493	post
carriage	nasopharynx	2007	Massachusetts	PMID: 23644493	post
carriage	nasopharynx	2007	Massachusetts	PMID: 23644493	post
carriage	nasopharynx	2007	Massachusetts	PMID: 23644493	post
carriage	nasopharynx	2004	Massachusetts	PMID: 23644493	post
carriage	nasopharynx	2004	Massachusetts	PMID: 23644493	post
carriage	nasopharynx	2004	Massachusetts	PMID: 23644493	post
carriage	nasopharynx	2004	Massachusetts	PMID: 23644493	post
carriage	nasopharynx	2004	Massachusetts	PMID: 23644493	post
carriage	nasopharynx	2004	Massachusetts	PMID: 23644493	post
carriage	nasopharynx	2004	Massachusetts	PMID: 23644493	post
carriage	nasopharynx	2004	Massachusetts	PMID: 23644493	post

carriage	nasopharynx	2004	Massachusetts	PMID: 23644493	post
carriage	nasopharynx	2004	Massachusetts	PMID: 23644493	post
carriage	nasopharynx	2004	Massachusetts	PMID: 23644493	post
carriage	nasopharynx	2004	Massachusetts	PMID: 23644493	post
carriage	nasopharynx	2004	Massachusetts	PMID: 23644493	post
carriage	nasopharynx	2004	Massachusetts	PMID: 23644493	post
carriage	nasopharynx	2004	Massachusetts	PMID: 23644493	post
carriage	nasopharynx	2004	Massachusetts	PMID: 23644493	post
carriage	nasopharynx	2004	Massachusetts	PMID: 23644493	post
carriage	nasopharynx	2004	Massachusetts	PMID: 23644493	post
carriage	nasopharynx	2004	Massachusetts	PMID: 23644493	post
carriage	nasopharynx	2004	Massachusetts	PMID: 23644493	post
carriage	nasopharynx	2004	Massachusetts	PMID: 23644493	post
carriage	nasopharynx	2004	Massachusetts	PMID: 23644493	post
carriage	nasopharynx	2004	Massachusetts	PMID: 23644493	post
carriage	nasopharynx	2004	Massachusetts	PMID: 23644493	post
carriage	nasopharynx	2004	Massachusetts	PMID: 23644493	post
carriage	nasopharynx	2004	Massachusetts	PMID: 23644493	post
carriage	nasopharynx	2004	Massachusetts	PMID: 23644493	post
carriage	nasopharynx	2004	Massachusetts	PMID: 23644493	post
carriage	nasopharynx	2004	Massachusetts	PMID: 23644493	post
carriage	nasopharynx	1996	PMEN	unknown	pre
unknown	unknown	2005	PMEN1	PMID: 21273480	pre
unknown	unknown	2005	PMEN1	PMID: 21273480	pre
disease	sputum	2000	PMEN2	PMID: 24957517	pre
disease	sputum	2000	PMEN2	PMID: 24957517	pre
disease	nasopharynx; URT	2000	PMEN2	PMID: 24957517	pre
disease	middle ear fluid	2000	PMEN2	PMID: 24957517	pre
disease	nose; URT	2000	PMEN2	PMID: 24957517	pre
disease	sputum	2000	PMEN2	PMID: 24957517	pre
disease	nasopharynx; URT	2000	PMEN2	PMID: 24957517	pre
disease	blood	2001	PMEN2	PMID: 24957517	pre
disease	nasopharynx; URT	2001	PMEN2	PMID: 24957517	pre
disease	nasopharynx; URT	2001	PMEN2	PMID: 24957517	pre
disease	nasopharynx; URT	2001	PMEN2	PMID: 24957517	pre
disease	nose; URT	2001	PMEN2	PMID: 24957517	pre
disease	sputum	2001	PMEN2	PMID: 24957517	pre
disease	middle ear fluid	2001	PMEN2	PMID: 24957517	post
disease	nasopharynx; URT	2001	PMEN2	PMID: 24957517	pre
disease	nasopharynx; URT	2001	PMEN2	PMID: 24957517	pre
disease	middle ear fluid	2002	PMEN2	PMID: 24957517	pre
disease	nasopharynx; URT	2002	PMEN2	PMID: 24957517	pre
disease	blood	2002	PMEN2	PMID: 24957517	pre
disease	sputum	2002	PMEN2	PMID: 24957517	post
disease	sputum	2002	PMEN2	PMID: 24957517	post
disease	nasopharynx; URT	2002	PMEN2	PMID: 24957517	pre
disease	middle ear fluid	2002	PMEN2	PMID: 24957517	post
disease	middle ear fluid	2002	PMEN2	PMID: 24957517	pre
disease	middle ear fluid	2003	PMEN2	PMID: 24957517	pre
disease	middle ear fluid	2003	PMEN2	PMID: 24957517	pre
disease	middle ear fluid	2003	PMEN2	PMID: 24957517	pre
disease	nasopharynx; URT	2004	PMEN2	PMID: 24957517	pre
disease	sputum	2004	PMEN2	PMID: 24957517	pre
disease	nasopharynx; URT	2004	PMEN2	PMID: 24957517	pre
disease	middle ear fluid	2005	PMEN2	PMID: 24957517	pre
disease	middle ear fluid	2005	PMEN2	PMID: 24957517	pre
disease	middle ear fluid	2005	PMEN2	PMID: 24957517	unknown
disease	nasopharynx; URT	2005	PMEN2	PMID: 24957517	pre
disease	nasopharynx; URT	2005	PMEN2	PMID: 24957517	pre

disease	nasopharynx; URT	2005	PMEN2	PMID: 24957517	pre
disease	nasopharynx; URT	2005	PMEN2	PMID: 24957517	pre
disease	nasopharynx; URT	2005	PMEN2	PMID: 24957517	pre
unknown	unknown	1988	PMEN2	PMID: 24957517	pre
disease	pneumonia	2006	PMEN2	PMID: 24957517	post
disease	nasopharynx; URT	1995	PMEN2	PMID: 24957517	pre
disease	sputum	1995	PMEN2	PMID: 24957517	pre
disease	nasopharynx; URT	1996	PMEN2	PMID: 24957517	pre
disease	middle ear fluid	1996	PMEN2	PMID: 24957517	pre
disease	conjunctiva	1996	PMEN2	PMID: 24957517	pre
disease	nasopharynx; URT	1996	PMEN2	PMID: 24957517	pre
disease	nasopharynx; URT	1996	PMEN2	PMID: 24957517	pre
disease	nose; URT	1996	PMEN2	PMID: 24957517	pre
disease	sputum	1996	PMEN2	PMID: 24957517	pre
disease	nasopharynx; URT	1996	PMEN2	PMID: 24957517	pre
disease	blood	1996	PMEN2	PMID: 24957517	pre
disease	middle ear fluid	1997	PMEN2	PMID: 24957517	pre
disease	nose; URT	1997	PMEN2	PMID: 24957517	pre
disease	blood	1997	PMEN2	PMID: 24957517	pre
disease	blood	1997	PMEN2	PMID: 24957517	pre
disease	middle ear fluid	1997	PMEN2	PMID: 24957517	pre
unknown	unknown	1997	PMEN2	PMID: 24957517	pre
disease	middle ear fluid	1997	PMEN2	PMID: 24957517	pre
disease	nasopharynx; URT	1997	PMEN2	PMID: 24957517	pre
disease	middle ear fluid	1997	PMEN2	PMID: 24957517	pre
disease	middle ear fluid	1998	PMEN2	PMID: 24957517	pre
disease	nasopharynx; URT	1998	PMEN2	PMID: 24957517	pre
disease	sinus	1998	PMEN2	PMID: 24957517	pre
disease	nasopharynx; URT	1998	PMEN2	PMID: 24957517	pre
disease	blood	1998	PMEN2	PMID: 24957517	pre
disease	nasopharynx; URT	1998	PMEN2	PMID: 24957517	pre
disease	middle ear fluid	1998	PMEN2	PMID: 24957517	pre
disease	nasopharynx; URT	1998	PMEN2	PMID: 24957517	pre
disease	middle ear fluid	1998	PMEN2	PMID: 24957517	pre
disease	middle ear fluid	1999	PMEN2	PMID: 24957517	pre
disease	middle ear fluid	1999	PMEN2	PMID: 24957517	pre
disease	nasopharynx; URT	1999	PMEN2	PMID: 24957517	pre
disease	nose; URT	1999	PMEN2	PMID: 24957517	pre
disease	middle ear fluid	1999	PMEN2	PMID: 24957517	pre
disease	urine	1999	PMEN2	PMID: 24957517	pre
disease	nose; URT	1999	PMEN2	PMID: 24957517	pre
disease	nasopharynx; URT	1999	PMEN2	PMID: 24957517	pre
disease	nasopharynx; URT	1999	PMEN2	PMID: 24957517	pre
carriage	carriage	1998	PMEN2	PMID: 24957517	pre
carriage	carriage	1998	PMEN2	PMID: 24957517	pre
carriage	carriage	1998	PMEN2	PMID: 24957517	pre
carriage	carriage	1999	PMEN2	PMID: 24957517	pre
carriage	carriage	1999	PMEN2	PMID: 24957517	pre
unknown	unknown	2007	PMEN2	PMID: 24957517	pre
disease	sputum	2007	PMEN2	PMID: 24957517	post
disease	sputum	2007	PMEN2	PMID: 24957517	post
disease	sputum	2008	PMEN2	PMID: 24957517	post
disease	sputum	2008	PMEN2	PMID: 24957517	post
disease	sputum	2008	PMEN2	PMID: 24957517	pre
disease	sputum	2008	PMEN2	PMID: 24957517	post
disease	sputum	2009	PMEN2	PMID: 24957517	post
disease	sputum	2009	PMEN2	PMID: 24957517	post
unknown	unknown	2001	PMEN2	PMID: 24957517	pre
unknown	unknown	2001	PMEN2	PMID: 24957517	pre

unknown	unknown	2001	PMEN2	PMID: 24957517	pre
disease	pneumonia	2002	PMEN2	PMID: 24957517	pre
unknown	unknown	2008	PMEN2	PMID: 24957517	post
unknown	unknown	2008	PMEN2	PMID: 24957517	pre
disease	pneumonia	2007	PMEN2	PMID: 24957517	pre
disease	pneumonia	2008	PMEN2	PMID: 24957517	pre
disease	pneumonia	2009	PMEN2	PMID: 24957517	post
disease	pneumonia	2009	PMEN2	PMID: 24957517	post
unknown	unknown	2007	PMEN2	PMID: 24957517	post
disease	bacteraemia	2009	PMEN2	PMID: 24957517	post
disease	pneumonia	2001	PMEN2	PMID: 24957517	pre
unknown	unknown	2008	PMEN2	PMID: 24957517	post
unknown	unknown	2001	PMEN2	PMID: 24957517	pre
unknown	unknown	2001	PMEN2	PMID: 24957517	pre
unknown	unknown	2001	PMEN2	PMID: 24957517	post
disease	bacteraemia	2006	PMEN2	PMID: 24957517	pre
carriage	carriage	2001	PMEN2	PMID: 24957517	pre
carriage	carriage	2001	PMEN2	PMID: 24957517	pre
carriage	carriage	2001	PMEN2	PMID: 24957517	pre
carriage	carriage	2002	PMEN2	PMID: 24957517	pre
carriage	carriage	2002	PMEN2	PMID: 24957517	post
carriage	carriage	2002	PMEN2	PMID: 24957517	pre
carriage	carriage	2001	PMEN2	PMID: 24957517	pre
carriage	carriage	2001	PMEN2	PMID: 24957517	pre
unknown	unknown	2004	PMEN2	PMID: 24957517	pre
unknown	unknown	2005	PMEN2	PMID: 24957517	pre
unknown	unknown	unknown	PMEN2	PMID: 24957517	unknown
unknown	unknown	2005	PMEN2	PMID: 24957517	pre
disease	pneumonia	2007	PMEN2	PMID: 24957517	pre
disease	meningitis	2008	PMEN2	PMID: 24957517	post
unknown	unknown	unknown	PMEN2	PMID: 24957517	unknown
disease	bronchitis	1998	PMEN2	PMID: 24957517	pre
unknown	unknown	1999	PMEN2	PMID: 24957517	pre
disease	fever	2005	PMEN2	PMID: 24957517	pre
disease	otitis media	2005	PMEN2	PMID: 24957517	pre
disease	pneumonia	1992	PMEN2	PMID: 24957517	unknown
disease	meningitis	1992	PMEN2	PMID: 24957517	unknown
disease	sepsis	1993	PMEN2	PMID: 24957517	pre
disease	fever	1995	PMEN2	PMID: 24957517	pre
unknown	unknown	1995	PMEN2	PMID: 24957517	unknown
disease	meningitis	1996	PMEN2	PMID: 24957517	pre
unknown	unknown	1996	PMEN2	PMID: 24957517	pre
disease	pharyngitis	2001	PMEN2	PMID: 24957517	pre
disease	middle ear fluid	1989	PMEN2	PMID: 24957517	pre
disease	nasopharynx; URT	1990	PMEN2	PMID: 24957517	pre
disease	middle ear fluid	1992	PMEN2	PMID: 24957517	pre
disease	nasopharynx; URT	1992	PMEN2	PMID: 24957517	pre
disease	middle ear fluid	1992	PMEN2	PMID: 24957517	pre
disease	nasopharynx; URT	1992	PMEN2	PMID: 24957517	pre
disease	nasopharynx; URT	1993	PMEN2	PMID: 24957517	pre
disease	middle ear fluid	1993	PMEN2	PMID: 24957517	pre
disease	nasopharynx; URT	1993	PMEN2	PMID: 24957517	pre
disease	middle ear fluid	1991	PMEN2	PMID: 24957517	pre
disease	middle ear fluid	1993	PMEN2	PMID: 24957517	pre
disease	middle ear fluid	1993	PMEN2	PMID: 24957517	pre
disease	nasopharynx; URT	1993	PMEN2	PMID: 24957517	pre
disease	middle ear fluid	1991	PMEN2	PMID: 24957517	pre
disease	parotid gland	1994	PMEN2	PMID: 24957517	pre
disease	middle ear fluid	1991	PMEN2	PMID: 24957517	pre

disease	sputum	1990	PMEN2	PMID: 24957517	pre
disease	middle ear fluid	1990	PMEN2	PMID: 24957517	pre
disease	middle ear fluid	1996	PMEN2	PMID: 24957517	pre
disease	sputum	1990	PMEN2	PMID: 24957517	pre
disease	middle ear fluid	1996	PMEN2	PMID: 24957517	pre
disease	middle ear fluid	1996	PMEN2	PMID: 24957517	pre
disease	nasopharynx; URT	1996	PMEN2	PMID: 24957517	pre
disease	nasopharynx; URT	1996	PMEN2	PMID: 24957517	pre
disease	sputum	1990	PMEN2	PMID: 24957517	pre
disease	sinus	1990	PMEN2	PMID: 24957517	pre
disease	middle ear fluid	1990	PMEN2	PMID: 24957517	pre
disease	middle ear fluid	1989	PMEN2	PMID: 24957517	pre
disease	sputum	1989	PMEN2	PMID: 24957517	pre
disease	middle ear fluid	1989	PMEN2	PMID: 24957517	pre
disease	middle ear fluid	1989	PMEN2	PMID: 24957517	pre
disease	middle ear fluid	1987-1989	PMEN2	PMID: 24957517	pre
disease	sputum	1991	PMEN2	PMID: 24957517	pre
unknown	unknown	1987-1989	PMEN2	PMID: 24957517	pre
unknown	unknown	1987-1989	PMEN2	PMID: 24957517	pre
disease	blood	2009	VICE	This publication	pre
disease	blood	2009	VICE	This publication	pre
disease	blood	2009	VICE	This publication	pre
disease	blood	2009	VICE	This publication	pre
disease	blood	2009	VICE	This publication	pre
disease	blood	2010	VICE	This publication	pre
disease	blood	2011	VICE	This publication	pre
disease	blood	2011	VICE	This publication	pre
disease	blood	2011	VICE	This publication	pre
disease	blood	2012	VICE	This publication	post
disease	blood	2012	VICE	This publication	post
disease	blood	2012	VICE	This publication	post
disease	blood	2013	VICE	This publication	post
disease	blood	2013	VICE	This publication	post
disease	middle ear fluid	2009	VICE	This publication	pre
disease	middle ear fluid	2009	VICE	This publication	pre
disease	middle ear fluid	2009	VICE	This publication	pre
disease	middle ear fluid	2009	VICE	This publication	pre
disease	middle ear fluid	2009	VICE	This publication	pre
disease	middle ear fluid	2009	VICE	This publication	pre
disease	middle ear fluid	2009	VICE	This publication	pre
disease	middle ear fluid	2009	VICE	This publication	pre
disease	middle ear fluid	2009	VICE	This publication	pre
disease	middle ear fluid	2009	VICE	This publication	pre
disease	middle ear fluid	2009	VICE	This publication	pre
disease	middle ear fluid	2009	VICE	This publication	pre
disease	middle ear fluid	2009	VICE	This publication	pre
disease	middle ear fluid	2009	VICE	This publication	pre
disease	middle ear fluid	2009	VICE	This publication	pre
disease	middle ear fluid	2009	VICE	This publication	pre
disease	middle ear fluid	2009	VICE	This publication	pre
disease	middle ear fluid	2010	VICE	This publication	pre
disease	middle ear fluid	2010	VICE	This publication	pre
disease	middle ear fluid	2010	VICE	This publication	pre
disease	middle ear fluid	2010	VICE	This publication	pre
disease	middle ear fluid	2010	VICE	This publication	pre
disease	middle ear fluid	2010	VICE	This publication	pre
disease	middle ear fluid	2010	VICE	This publication	pre
disease	middle ear fluid	2010	VICE	This publication	pre
disease	middle ear fluid	2010	VICE	This publication	pre

disease	middle ear fluid	2010	VICE	This publication	pre
disease	middle ear fluid	2010	VICE	This publication	pre
disease	middle ear fluid	2010	VICE	This publication	pre
disease	middle ear fluid	2010	VICE	This publication	pre
disease	middle ear fluid	2010	VICE	This publication	pre
disease	middle ear fluid	2010	VICE	This publication	pre
disease	middle ear fluid	2010	VICE	This publication	pre
disease	middle ear fluid	2010	VICE	This publication	pre
disease	middle ear fluid	2010	VICE	This publication	pre
disease	middle ear fluid	2010	VICE	This publication	pre
disease	middle ear fluid	2010	VICE	This publication	pre
disease	middle ear fluid	2011	VICE	This publication	pre
disease	middle ear fluid	2011	VICE	This publication	pre
disease	middle ear fluid	2011	VICE	This publication	pre
disease	middle ear fluid	2011	VICE	This publication	pre
disease	middle ear fluid	2011	VICE	This publication	pre
disease	middle ear fluid	2011	VICE	This publication	pre
disease	middle ear fluid	2011	VICE	This publication	pre
disease	middle ear fluid	2011	VICE	This publication	pre
disease	middle ear fluid	2011	VICE	This publication	pre
disease	middle ear fluid	2012	VICE	This publication	post
disease	middle ear fluid	2012	VICE	This publication	post
disease	middle ear fluid	2012	VICE	This publication	post
disease	middle ear fluid	2012	VICE	This publication	post
disease	middle ear fluid	2012	VICE	This publication	post
disease	middle ear fluid	2012	VICE	This publication	post
disease	middle ear fluid	2012	VICE	This publication	post
disease	middle ear fluid	2012	VICE	This publication	post
disease	middle ear fluid	2012	VICE	This publication	post
disease	middle ear fluid	2012	VICE	This publication	post
disease	middle ear fluid	2012	VICE	This publication	post
disease	middle ear fluid	2012	VICE	This publication	post
disease	pleural fluid	2010	VICE	This publication	pre
disease	sputum	2009	VICE	This publication	pre
disease	sputum	2009	VICE	This publication	pre
disease	sputum	2009	VICE	This publication	pre
disease	sputum	2009	VICE	This publication	pre
disease	sputum	2009	VICE	This publication	pre
disease	sputum	2009	VICE	This publication	pre
disease	sputum	2009	VICE	This publication	pre
disease	sputum	2009	VICE	This publication	pre
disease	sputum	2009	VICE	This publication	pre
disease	sputum	2010	VICE	This publication	pre
disease	sputum	2010	VICE	This publication	pre
disease	sputum	2010	VICE	This publication	pre
disease	sputum	2010	VICE	This publication	pre
disease	sputum	2010	VICE	This publication	pre
disease	sputum	2010	VICE	This publication	pre
disease	sputum	2010	VICE	This publication	pre
disease	sputum	2011	VICE	This publication	pre
disease	sputum	2011	VICE	This publication	pre
disease	sputum	2011	VICE	This publication	pre
disease	sputum	2012	VICE	This publication	post
disease	sputum	2012	VICE	This publication	post
disease	sputum	2012	VICE	This publication	post
disease	sputum	2012	VICE	This publication	post
disease	sputum	2012	VICE	This publication	post
disease	sputum	2012	VICE	This publication	post
disease	sputum	2012	VICE	This publication	post

disease	lung aspirate	2010	VICE	This publication	pre
disease	lung aspirate	2010	VICE	This publication	pre
disease	lung aspirate	2010	VICE	This publication	pre
disease	lung aspirate	2011	VICE	This publication	pre
disease	lung aspirate	2012	VICE	This publication	post
disease	blood	2014	VICE	This publication	post
carriage	nasopharynx	2009	VICE	This publication	pre
carriage	nasopharynx	2009	VICE	This publication	pre
carriage	nasopharynx	2009	VICE	This publication	pre
carriage	nasopharynx	2009	VICE	This publication	pre
carriage	nasopharynx	2009	VICE	This publication	pre
carriage	nasopharynx	2009	VICE	This publication	pre
carriage	nasopharynx	2009	VICE	This publication	pre
carriage	nasopharynx	2009	VICE	This publication	pre
carriage	nasopharynx	2009	VICE	This publication	pre
carriage	nasopharynx	2009	VICE	This publication	pre
carriage	nasopharynx	2009	VICE	This publication	pre
carriage	nasopharynx	2009	VICE	This publication	pre
carriage	nasopharynx	2009	VICE	This publication	pre
carriage	nasopharynx	2009	VICE	This publication	pre
carriage	nasopharynx	2009	VICE	This publication	pre
carriage	nasopharynx	2009	VICE	This publication	pre
carriage	nasopharynx	2009	VICE	This publication	pre
carriage	nasopharynx	2009	VICE	This publication	pre
carriage	nasopharynx	2009	VICE	This publication	pre
carriage	nasopharynx	2009	VICE	This publication	pre
carriage	nasopharynx	2009	VICE	This publication	pre
carriage	nasopharynx	2009	VICE	This publication	pre
carriage	nasopharynx	2009	VICE	This publication	pre
carriage	nasopharynx	2009	VICE	This publication	pre
carriage	nasopharynx	2009	VICE	This publication	pre
carriage	nasopharynx	2009	VICE	This publication	pre
carriage	nasopharynx	2009	VICE	This publication	pre
carriage	nasopharynx	2009	VICE	This publication	pre
carriage	nasopharynx	2009	VICE	This publication	pre
carriage	nasopharynx	2009	VICE	This publication	pre
carriage	nasopharynx	2009	VICE	This publication	pre
carriage	nasopharynx	2009	VICE	This publication	pre
carriage	nasopharynx	2009	VICE	This publication	pre
carriage	nasopharynx	2009	VICE	This publication	pre
carriage	nasopharynx	2009	VICE	This publication	pre
carriage	nasopharynx	2009	VICE	This publication	pre
carriage	nasopharynx	2009	VICE	This publication	pre
carriage	nasopharynx	2009	VICE	This publication	pre
carriage	nasopharynx	2009	VICE	This publication	pre
carriage	nasopharynx	2009	VICE	This publication	pre
carriage	nasopharynx	2009	VICE	This publication	pre
carriage	nasopharynx	2009	VICE	This publication	pre
carriage	nasopharynx	2009	VICE	This publication	pre
carriage	nasopharynx	2009	VICE	This publication	pre
carriage	nasopharynx	2009	VICE	This publication	pre
carriage	nasopharynx	2009	VICE	This publication	pre
carriage	nasopharynx	2009	VICE	This publication	pre
carriage	nasopharynx	2009	VICE	This publication	pre
carriage	nasopharynx	2009	VICE	This publication	pre
carriage	nasopharynx	2009	VICE	This publication	pre
carriage	nasopharynx	2009	VICE	This publication	pre
carriage	nasopharynx	2009	VICE	This publication	pre
carriage	nasopharynx	2010	VICE	This publication	pre
carriage	nasopharynx	2010	VICE	This publication	pre
carriage	nasopharynx	2010	VICE	This publication	pre

carriage	nasopharynx	2010	VICE	This publication	pre
carriage	nasopharynx	2010	VICE	This publication	pre
carriage	nasopharynx	2010	VICE	This publication	pre
carriage	nasopharynx	2010	VICE	This publication	pre
carriage	nasopharynx	2010	VICE	This publication	pre
carriage	nasopharynx	2010	VICE	This publication	pre
carriage	nasopharynx	2010	VICE	This publication	pre
carriage	nasopharynx	2010	VICE	This publication	pre
carriage	nasopharynx	2010	VICE	This publication	pre
carriage	nasopharynx	2010	VICE	This publication	pre
carriage	nasopharynx	2010	VICE	This publication	pre
carriage	nasopharynx	2010	VICE	This publication	pre
carriage	nasopharynx	2010	VICE	This publication	pre
carriage	nasopharynx	2010	VICE	This publication	pre
carriage	nasopharynx	2010	VICE	This publication	pre
carriage	nasopharynx	2010	VICE	This publication	pre
carriage	nasopharynx	2010	VICE	This publication	pre
carriage	nasopharynx	2010	VICE	This publication	pre
carriage	nasopharynx	2010	VICE	This publication	pre
carriage	nasopharynx	2010	VICE	This publication	pre
carriage	nasopharynx	2010	VICE	This publication	pre
carriage	nasopharynx	2010	VICE	This publication	pre
carriage	nasopharynx	2010	VICE	This publication	pre
carriage	nasopharynx	2010	VICE	This publication	pre
carriage	nasopharynx	2010	VICE	This publication	pre
carriage	nasopharynx	2010	VICE	This publication	pre
carriage	nasopharynx	2010	VICE	This publication	pre
carriage	nasopharynx	2010	VICE	This publication	pre
carriage	nasopharynx	2010	VICE	This publication	pre
carriage	nasopharynx	2010	VICE	This publication	pre
carriage	nasopharynx	2010	VICE	This publication	pre
carriage	nasopharynx	2010	VICE	This publication	pre
carriage	nasopharynx	2011	VICE	This publication	pre
carriage	nasopharynx	2011	VICE	This publication	pre
carriage	nasopharynx	2011	VICE	This publication	pre
carriage	nasopharynx	2011	VICE	This publication	pre
carriage	nasopharynx	2011	VICE	This publication	pre
carriage	nasopharynx	2011	VICE	This publication	pre
carriage	nasopharynx	2011	VICE	This publication	pre
carriage	nasopharynx	2011	VICE	This publication	pre
carriage	nasopharynx	2011	VICE	This publication	pre
carriage	nasopharynx	2011	VICE	This publication	pre
carriage	nasopharynx	2011	VICE	This publication	pre
carriage	nasopharynx	2011	VICE	This publication	pre
carriage	nasopharynx	2011	VICE	This publication	pre
carriage	nasopharynx	2011	VICE	This publication	pre
carriage	nasopharynx	2011	VICE	This publication	pre
carriage	nasopharynx	2011	VICE	This publication	pre
carriage	nasopharynx	2011	VICE	This publication	pre
carriage	nasopharynx	2011	VICE	This publication	pre
carriage	nasopharynx	2011	VICE	This publication	pre
carriage	nasopharynx	2011	VICE	This publication	pre
carriage	nasopharynx	2011	VICE	This publication	pre
carriage	nasopharynx	2011	VICE	This publication	pre
carriage	nasopharynx	2011	VICE	This publication	pre
carriage	nasopharynx	2011	VICE	This publication	pre
carriage	nasopharynx	2011	VICE	This publication	pre
carriage	nasopharynx	2012	VICE	This publication	post
carriage	nasopharynx	2012	VICE	This publication	post

carriage	nasopharynx	2012	VICE	This publication	post
carriage	nasopharynx	2012	VICE	This publication	post
carriage	nasopharynx	2012	VICE	This publication	post
carriage	nasopharynx	2012	VICE	This publication	post
carriage	nasopharynx	2012	VICE	This publication	post
carriage	nasopharynx	2012	VICE	This publication	post
carriage	nasopharynx	2012	VICE	This publication	post
carriage	nasopharynx	2012	VICE	This publication	post
carriage	nasopharynx	2012	VICE	This publication	post
carriage	nasopharynx	2012	VICE	This publication	post
carriage	nasopharynx	2012	VICE	This publication	post
carriage	nasopharynx	2012	VICE	This publication	post
carriage	nasopharynx	2012	VICE	This publication	post
carriage	nasopharynx	2012	VICE	This publication	post
carriage	nasopharynx	2012	VICE	This publication	post
carriage	nasopharynx	2012	VICE	This publication	post
carriage	nasopharynx	2012	VICE	This publication	post
carriage	nasopharynx	2012	VICE	This publication	post
carriage	nasopharynx	2012	VICE	This publication	post
carriage	nasopharynx	2012	VICE	This publication	post
carriage	nasopharynx	2012	VICE	This publication	post
carriage	nasopharynx	2012	VICE	This publication	post
carriage	nasopharynx	2012	VICE	This publication	post
carriage	nasopharynx	2012	VICE	This publication	post
carriage	nasopharynx	2012	VICE	This publication	post
carriage	nasopharynx	2012	VICE	This publication	post
carriage	nasopharynx	2012	VICE	This publication	post
carriage	nasopharynx	2012	VICE	This publication	post
carriage	nasopharynx	2012	VICE	This publication	post
carriage	nasopharynx	2012	VICE	This publication	post
carriage	nasopharynx	2012	VICE	This publication	post
carriage	nasopharynx	2012	VICE	This publication	post
carriage	nasopharynx	2012	VICE	This publication	post
carriage	nasopharynx	2012	VICE	This publication	post
carriage	nasopharynx	2012	VICE	This publication	post
carriage	nasopharynx	2012	VICE	This publication	post
carriage	nasopharynx	2012	VICE	This publication	post
carriage	nasopharynx	2012	VICE	This publication	post
carriage	nasopharynx	2013	VICE	This publication	post
carriage	nasopharynx	2013	VICE	This publication	post
carriage	nasopharynx	2013	VICE	This publication	post
carriage	nasopharynx	2013	VICE	This publication	post
carriage	nasopharynx	2013	VICE	This publication	post
carriage	nasopharynx	2013	VICE	This publication	post
carriage	nasopharynx	2013	VICE	This publication	post
carriage	nasopharynx	2013	VICE	This publication	post
carriage	nasopharynx	2013	VICE	This publication	post
carriage	nasopharynx	2013	VICE	This publication	post
carriage	nasopharynx	2013	VICE	This publication	post
carriage	nasopharynx	2013	VICE	This publication	post
carriage	nasopharynx	2013	VICE	This publication	post
carriage	nasopharynx	2013	VICE	This publication	post
carriage	nasopharynx	2013	VICE	This publication	post
carriage	nasopharynx	2013	VICE	This publication	post
carriage	nasopharynx	2013	VICE	This publication	post
carriage	nasopharynx	2013	VICE	This publication	post
carriage	nasopharynx	2013	VICE	This publication	post
carriage	nasopharynx	2013	VICE	This publication	post
carriage	nasopharynx	2013	VICE	This publication	post

carriage	nasopharynx	2013	VICE	This publication	post
carriage	nasopharynx	2013	VICE	This publication	post
carriage	nasopharynx	2013	VICE	This publication	post
carriage	nasopharynx	2013	VICE	This publication	post
carriage	nasopharynx	2013	VICE	This publication	post
carriage	nasopharynx	2013	VICE	This publication	post
carriage	nasopharynx	2013	VICE	This publication	post
carriage	nasopharynx	2013	VICE	This publication	post
disease	middle ear fluid	2013	VICE	This publication	post
disease	middle ear fluid	2013	VICE	This publication	post
disease	middle ear fluid	2013	VICE	This publication	post
disease	middle ear fluid	2013	VICE	This publication	post
disease	middle ear fluid	2013	VICE	This publication	post
disease	middle ear fluid	2013	VICE	This publication	post
disease	middle ear fluid	2013	VICE	This publication	post
disease	middle ear fluid	2013	VICE	This publication	post
disease	middle ear fluid	2013	VICE	This publication	post
disease	middle ear fluid	2013	VICE	This publication	post
disease	middle ear fluid	2013	VICE	This publication	post
disease	middle ear fluid	2013	VICE	This publication	post
disease	sputum	2013	VICE	This publication	post
disease	sputum	2013	VICE	This publication	post
disease	sputum	2013	VICE	This publication	post
disease	sputum	2013	VICE	This publication	post
disease	sputum	2013	VICE	This publication	post
disease	sputum	2013	VICE	This publication	post
disease	sputum	2013	VICE	This publication	post
disease	sputum	2013	VICE	This publication	post
carriage	nasopharynx	2014	VICE	This publication	post
carriage	nasopharynx	2014	VICE	This publication	post
carriage	nasopharynx	2014	VICE	This publication	post
carriage	nasopharynx	2014	VICE	This publication	post
carriage	nasopharynx	2014	VICE	This publication	post
carriage	nasopharynx	2014	VICE	This publication	post
carriage	nasopharynx	2014	VICE	This publication	post
carriage	nasopharynx	2014	VICE	This publication	post
carriage	nasopharynx	2014	VICE	This publication	post
carriage	nasopharynx	2014	VICE	This publication	post
carriage	nasopharynx	2014	VICE	This publication	post
carriage	nasopharynx	2014	VICE	This publication	post
carriage	nasopharynx	2014	VICE	This publication	post
carriage	nasopharynx	2014	VICE	This publication	post
carriage	nasopharynx	2014	VICE	This publication	post
carriage	nasopharynx	2014	VICE	This publication	post
carriage	nasopharynx	2014	VICE	This publication	post
carriage	nasopharynx	2014	VICE	This publication	post
carriage	nasopharynx	2014	VICE	This publication	post
carriage	nasopharynx	2014	VICE	This publication	post
carriage	nasopharynx	2014	VICE	This publication	post
carriage	nasopharynx	2014	VICE	This publication	post
carriage	nasopharynx	2014	VICE	This publication	post
carriage	nasopharynx	2014	VICE	This publication	post
carriage	nasopharynx	2014	VICE	This publication	post
carriage	nasopharynx	2014	VICE	This publication	post
disease	blood	2014	VICE	This publication	post
disease	middle ear fluid	2014	VICE	This publication	post
disease	middle ear fluid	2014	VICE	This publication	post
disease	middle ear fluid	2014	VICE	This publication	post
disease	middle ear fluid	2014	VICE	This publication	post

disease	sputum	2014	VICE	This publication	post
disease	middle ear fluid	2014	VICE	This publication	post
disease	unknown	2014	VICE	This publication	post

SEROTYPE 6A ALL ISOLATES

					6	51	53	54	61	65
		6A reference			S	V	A	L	V	L
31317		R34-3026	242	6A	S	V	A	L	V	L
N/A	1142	GA17971	Sing1175	6A	S	V	A	L	V	L
33136		SMRU2446	982	6A	S	V	A	L	V	L
34268		SMRU2367	982	6A	S	V	A	L	V	L
40413		SMRU2186	982	6A	S	V	A	L	V	L
44978		SMRU2263	982	6A	S	V	A	L	V	L
44987		SMRU2380	982	6A	S	V	A	L	V	L
66480		SMRU1655	982	6A	S	V	A	L	V	L
82429		SMRU2158	982	6A	S	V	A	L	V	L
82450		SMRU2502	982	6A	S	V	A	L	V	L
82513		SMRU2281	982	6A	S	V	A	L	V	L
82548		SMRU2398	Singleton10056	6A	S	V	A	L	V	L
82574		SMRU2595	982	6A	S	V	A	L	V	L
82691		SMRU3040	982	6A	S	V	A	L	V	L
82715		SMRU2966	982	6A	S	V	A	L	V	L
82720		SMRU2978	982	6A	S	V	A	L	V	L
83479		SMRU2478	982	6A	S	V	A	L	V	L
83487		SMRU2261	982	6A	S	V	A	L	V	L
83507		SMRU2399	982	6A	S	V	A	L	V	L
83525		SMRU2596	982	6A	S	V	A	L	V	L
83587		SMRU2960	982	6A	S	V	A	L	V	L
103555		SMRU1172	982	6A	S	V	A	L	V	L
103565		SMRU572	982	6A	S	V	A	L	V	L
103592		SMRU1233	982	6A	S	V	A	L	V	L
103605		SMRU2347	982	6A	S	V	A	L	V	L
103617		SMRU1681	982	6A	S	V	A	L	V	L
103623		SMRU2956	982	6A	S	V	A	L	V	L
103813		SMRU1928	Singleton10056	6A	S	V	A	L	V	L
103836		SMRU1920	982	6A	S	V	A	L	V	L
N/A	662	VICE0340	15	6A	G	V	A	L	V	L
N/A	755	VICE0433	15	6A	G	V	A	L	V	L
N/A	959	VICE0637	15	6A	G	V	A	L	V	L
N/A	1320	VICE0726	15	6A	G	V	A	L	V	L
N/A	1485	VICE0934	15	6A	G	V	A	L	V	L
N/A	1488	VICE0937a	15	6A	G	V	A	L	V	L
N/A	1493	VICE0942	15	6A	G	V	A	L	V	L
N/A	1495	VICE0944	15	6A	G	V	A	L	V	L
N/A	1499	VICE0948	15	6A	G	V	A	L	V	L
N/A	1615	VICE1064	15	6A	G	V	A	L	V	L
N/A	1625	VICE1074	15	6A	G	V	A	L	V	L
N/A	2101	VICE1223a	15	6A	G	V	A	L	V	L
N/A	2642	VICE1689	15	6A	G	V	A	L	V	L
31318		R34-3027	473	6A	G	V	A	L	V	L
31337		R34-3180	690	6A	G	V	A	L	V	L
31349		R34-3096	473	6A	G	V	A	L	V	L
31382		R34-3151	473	6A	G	V	A	L	V	L
31410		R34-3211	473	6A	G	V	A	L	V	L
31417		15445	473	6A	G	V	A	L	V	L
31418		42861	690	6A	G	V	A	L	V	L
31425		166637	473	6A	G	V	A	L	V	L
31427		181272	473	6A	G	V	A	L	V	L
31461		359370	473	6A	G	V	A	L	V	L
32034		SMRU2443	4113	6A	S	V	A	L	V	L
32085		R34-3099	473	6A	G	V	A	L	V	L
32154		R34-3210	473	6A	G	V	A	L	V	L
32187		101058	690	6A	G	V	A	L	V	L
32418		CH2090	473	6A	G	V	A	L	V	L
33175		420881	473	6A	G	V	A	L	V	L
33945		SMRU991	4536/6767	6A	S	V	A	L	V	L
34146		SMRU1588	4536/6767	6A	S	V	A	L	V	L
34155		SMRU1946	4536/6767	6A	S	V	A	L	V	L
34167		SMRU2020	4536/6767	6A	S	V	A	L	V	L

34319		SMRU2934	4536/6767	6A	S	V	A	L	V	L
40394		SMRU1976	4536/6767	6A	S	V	A	L	V	L
40503		SMRU2293	4536/6767	6A	S	V	A	L	V	L
40523		SMRU2868	4536/6767	6A	S	V	A	L	V	L
40580		SMRU2793	4536/6767	6A	S	V	A	L	V	L
40603		SMRU3080	4536/6767	6A	S	V	A	L	V	L
82210		SMRU692	4536/6767	6A	S	V	A	L	V	L
82317		SMRU1714	4536/6767	6A	S	V	A	L	V	L
82740		SMRU2253	4536/6767	6A	S	V	A	L	V	L
83410		SMRU1975	4536/6767	6A	S	V	A	L	V	L
83520		SMRU2580	4536/6767	6A	S	V	A	L	V	L
83609		SMRU3107	4536/6767	6A	S	V	A	L	V	L
103516		SMRU2184	4536/6767	6A	S	V	A	L	V	L
103548		SMRU1888	4536/6767	6A	S	V	A	L	V	L
103553		SMRU1610	4536/6767	6A	S	V	A	L	V	L
103554		SMRU1566	4536/6767	6A	S	V	A	L	V	L
103575		SMRU212	4536/6767	6A	S	V	A	L	V	L
103597		SMRU1197	4536/6767	6A	S	V	A	L	V	L
103618		SMRU3016	4536/6767	6A	S	V	A	L	V	L
103803		SMRU489	4536/6767	6A	S	V	A	L	V	L
103831		SMRU1370	4536/6767	6A	S	V	A	L	V	L
121412		UoS0050	473	6A	G	V	A	L	V	L
121447		UoS4399	473	6A	G	V	A	L	V	L
33266		110093	745/1939	6A	G	V	A	L	V	L
18190		DG29	81	6A	S	V	A	L	V	L
39946		DG80	81	6A	S	V	A	L	V	L
N/A	2643	VICE1690	9789	6A	R	V	A	L	V	L
N/A	420	VICE0877	460	6A	S	V	A	L	V	L
N/A	454	VICE0133	460	6A	S	V	A	L	V	L
N/A	474	VICE0153	460	6A	S	V	A	L	V	L
N/A	540	VICE0219	460	6A	S	V	A	L	V	L
N/A	594	VICE0272	460	6A	S	V	A	L	V	L
N/A	609	VICE0287	460	6A	S	V	A	L	V	L
N/A	673	VICE0351	460	6A	S	V	A	L	V	L
N/A	674	VICE0352	460	6A	S	V	A	L	V	L
N/A	704	VICE0382	460	6A	S	V	A	L	V	L
N/A	706	VICE0384	460	6A	S	V	A	L	V	L
N/A	715	VICE0393	176	6A	S	V	A	L	V	L
N/A	716	VICE0394	460	6A	S	V	A	L	V	L
N/A	720	VICE0398	460	6A	S	V	A	L	V	L
N/A	721	VICE0399	460	6A	S	V	A	L	V	L
N/A	724	VICE0402	460	6A	S	V	A	L	V	L
N/A	800	VICE0478	460	6A	S	V	A	L	V	L
N/A	814	VICE0492	460	6A	S	V	A	L	V	L
N/A	822	VICE0500	460	6A	S	V	A	L	V	L
N/A	861	VICE0539	460	6A	S	V	A	L	V	L
N/A	906	VICE0584	460	6A	S	V	A	L	V	L
N/A	917	VICE0595	460	6A	S	V	A	L	V	L
N/A	945	VICE0623	460	6A	S	V	T	L	V	L
N/A	1108	SP6-BS73	460	6A	S	V	A	L	V	L
N/A	1249	VICE0655	460	6A	S	V	A	L	V	L
N/A	1278	VICE0684	460	6A	S	V	A	L	V	L
N/A	1422	VICE0828	460	6A	S	V	A	L	V	L
N/A	1450	VICE0899	460	6A	S	V	A	L	V	L
N/A	1545	VICE0994	460	6A	S	V	A	L	V	L
N/A	1558	VICE1007	460	6A	S	V	A	L	V	L
N/A	1569	VICE1018	460	6A	S	V	A	L	V	L
N/A	2055	VICE1177	460	6A	S	V	A	L	V	L
N/A	2086	VICE1208	460	6A	S	V	A	L	V	L
N/A	2129	VICE1251	460	6A	S	V	A	L	V	L
N/A	2132	VICE1254	460	6A	S	V	A	L	V	L
N/A	2181	VICE1303	460	6A	S	V	A	L	V	L
N/A	2186	VICE1308	460	6A	S	V	A	L	V	L
N/A	2203	VICE1325	460	6A	S	V	A	L	V	L
N/A	2204	VICE1326	460	6A	S	V	A	L	V	L
N/A	2205	VICE1327	460	6A	S	V	A	L	V	L
N/A	2218	VICE1340	176	6A	S	V	A	L	V	L

N/A	2223	VICE1345	460	6A	S	V	A	L	V	L
N/A	2224	VICE1346	460	6A	S	V	A	L	V	L
N/A	2226	VICE1348	460	6A	S	V	A	L	V	L
N/A	2227	VICE1349	460	6A	S	V	A	L	V	L
N/A	2228	VICE1350	460	6A	S	V	A	L	V	L
N/A	2234	VICE1356	460	6A	S	V	A	L	V	L
N/A	2258	VICE1380	460	6A	S	V	A	L	V	L
N/A	2261	VICE1383	460	6A	S	V	A	L	V	L
N/A	2262	VICE1384	460	6A	S	V	A	L	V	L
N/A	2308	VICE1430	460	6A	S	V	A	L	V	L
N/A	2396	VICE1518	460	6A	S	V	A	L	V	L
N/A	2414	VICE1536	460	6A	S	V	A	L	V	L
N/A	2430	VICE1552	460	6A	S	V	A	L	V	L
N/A	2462	VICE1584	460	6A	S	V	A	L	V	L
N/A	2503	VICE1257b	460	6A	S	V	A	L	V	L
N/A	2587	VICE1634	460	6A	S	V	A	L	V	L
N/A	2600	VICE1647	460	6A	S	V	A	L	V	L
N/A	2621	VICE1668	460	6A	S	V	A	L	V	L
N/A	2660	VICE1707	460	6A	S	V	A	L	V	L
N/A	2790	VICE1829	460	6A	S	V	A	L	V	L
N/A	2833	VICE1868	460	6A	S	V	A	L	V	L
N/A	2892	6A/2	460	6A	S	V	A	L	V	L
N/A	3031	VICE1943	460	6A	S	V	A	L	V	L
31302		R34-3075	460	6A	S	V	A	L	V	L
31311		R34-3018	460	6A	S	V	A	L	V	L
31323		R34-3166	460	6A	S	V	A	L	V	L
31415		1996	460	6A	S	V	A	L	V	L
31448		299801	460	6A	S	V	A	L	V	L
31458		336850	460	6A	S	V	A	L	V	L
31466		383739	460	6A	S	V	A	L	V	L
32051		R34-3035	460	6A	S	V	A	L	V	L
32066		446376	460	6A	S	V	A	L	V	L
32068		472699	460	6A	S	V	A	L	V	L
32159		R34-3220	460	6A	S	V	A	L	V	L
32161		R34-3222	460	6A	S	V	A	L	V	L
32179		62031	460	6A	S	V	A	L	V	L
32183		89848	460	6A	S	V	A	L	V	L
32190		118039	460	6A	S	V	A	L	V	L
32193		135631	460	6A	S	V	A	L	V	L
32322		ND6134	460	6A	S	V	A	L	V	L
33262		42397	460	6A	S	V	A	L	V	L
40131		UoS0301	460	6A	S	V	A	L	V	L
44824		UoS3293	460	6A	S	V	A	L	V	L
55126		UoS4242	460	6A	S	V	A	L	V	L
115806		UoS0266	460	6A	S	V	A	L	V	L
121562		UoS2346	460	6A	S	V	A	L	V	L
121586		UoS0154	460	6A	S	V	A	L	V	L
121590		UoS2319	460	6A	S	V	A	L	V	L
121591		UoS0194	460	6A	S	V	A	L	V	L
121592		UoS3200	460	6A	S	V	A	L	V	L
121593		UoS0113	460	6A	S	V	A	L	V	L
121595		UoS0075	460	6A	S	V	A	L	V	L
121600		UoS2166	460	6A	S	V	A	L	V	L
121601		UoS5284	460	6A	S	V	A	L	V	L
121607		UoS4081	460	6A	S	V	A	L	V	L
121608		UoS2233	460	6A	S	V	A	L	V	L
121609		UoS2038	460	6A	S	V	A	L	V	L
121610		UoS4287	460	6A	S	V	A	L	V	L
121613		UoS2307	460	6A	S	V	A	L	V	L
121620		UoS2002	460	6A	S	V	A	L	V	L
121621		UoS2001	460	6A	S	V	A	L	V	L
121651		UoS2308	460	6A	S	V	A	L	V	L
123190		UoS0068	460	6A	S	V	A	L	V	L
123220		UoS3122	460	6A	S	V	A	L	V	L
123242		UoS2323	460	6A	S	V	A	L	V	L
123244		UoS2337	460	6A	S	V	A	L	V	L
123312		UoS3063	460	6A	S	V	A	L	V	L

123317		UoS3171	460	6A	S	V	A	L	V	L
123324		UoS3235	460	6A	S	V	A	L	V	L
123336		UoS2343	460	6A	S	V	A	L	V	L
121603		UoS2359	460	6A	S	V	A	L	V	L
N/A	216	ICE11	490	6A	G	V	A	L	V	L
N/A	388	VICE0863	490	6A	G	V	A	L	V	L
N/A	559	VICE0238	490	6A	G	V	A	L	V	L
N/A	576	VICE0254	490	6A	G	V	A	L	V	L
N/A	580	VICE0258	490	6A	G	V	A	L	V	L
N/A	630	VICE0308	490	6A	G	V	A	L	V	L
N/A	633	VICE0311	490	6A	G	V	A	L	V	L
N/A	659	VICE0337	490	6A	G	V	A	L	V	L
N/A	676	VICE0354	490	6A	G	V	A	L	V	L
N/A	712	VICE0390	490	6A	G	V	A	L	V	L
N/A	782	VICE0460	490	6A	G	V	A	L	V	L
N/A	787	VICE0465	490	6A	G	V	A	L	V	L
N/A	792	VICE0470	490	6A	G	V	A	L	V	L
N/A	912	VICE0590	490	6A	G	V	A	L	V	L
N/A	914	VICE0592	490	6A	G	V	A	L	V	L
N/A	930	VICE0608	490	6A	G	V	A	L	V	L
N/A	963	VICE0641	490	6A	G	V	A	L	V	L
N/A	1245	VICE0651	490	6A	G	V	A	L	V	L
N/A	1303	VICE0709	490	6A	G	V	A	L	V	L
N/A	1316	VICE0722	490	6A	G	V	A	L	V	L
N/A	1330	VICE0736	490	6A	G	V	A	L	V	L
N/A	1363	VICE0769	490	6A	G	V	A	L	V	L
N/A	1378	VICE0784	490	6A	G	V	A	L	V	L
N/A	1393	VICE0799	490	6A	G	V	A	L	V	L
N/A	1425	VICE0831	490	6A	G	V	A	L	V	L
N/A	1440	VICE0889	490	6A	G	V	A	L	V	L
N/A	1454	VICE0903	490	6A	G	V	A	L	V	L
N/A	1484	VICE0933	490	6A	G	V	A	L	V	L
N/A	1540	VICE0989a	490	6A	G	V	A	L	V	L
N/A	1564	VICE1013	490	6A	G	V	A	L	V	L
N/A	1565	VICE1014	490	6A	G	V	A	L	V	L
N/A	1566	VICE1015	490	6A	G	V	A	L	V	L
N/A	1581	VICE1030	490	6A	G	V	A	L	V	L
N/A	1606	VICE1055	490	6A	G	V	A	L	V	L
N/A	1609	VICE1058	490	6A	G	V	A	L	V	L
N/A	1675	VICE1124	490	6A	G	V	A	L	V	L
N/A	1676	VICE1125	490	6A	G	V	A	L	V	L
N/A	2023	VICE1145a	490	6A	G	V	A	L	V	L
N/A	2048	VICE1170	490	6A	G	V	A	L	V	L
N/A	2049	VICE1171	490	6A	G	V	A	L	V	L
N/A	2061	VICE1183	490	6A	G	V	A	L	V	L
N/A	2069	VICE1191	490	6A	G	V	A	L	V	L
N/A	2089	VICE1211	490	6A	G	V	A	L	V	L
N/A	2121	VICE1243	490	6A	G	V	A	L	V	L
N/A	2124	VICE1246	490	6A	G	V	A	L	V	L
N/A	2134	VICE1256	490	6A	G	V	A	L	V	L
N/A	2159	VICE1281	490	6A	G	V	A	L	V	L
N/A	2172	VICE1294	490	6A	G	V	A	L	V	L
N/A	2193	VICE1315	490	6A	G	V	A	L	V	L
N/A	2211	VICE1333	490	6A	G	V	A	L	V	L
N/A	2213	VICE1335	490	6A	G	V	A	L	V	L
N/A	2219	VICE1341	490	6A	G	V	A	L	V	L
N/A	2240	VICE1362	490	6A	G	V	A	L	V	L
N/A	2247	VICE1369	490	6A	G	V	A	L	V	L
N/A	2255	VICE1377	490	6A	G	V	A	L	V	L
N/A	2282	VICE1404	490	6A	G	V	A	L	V	L
N/A	2291	VICE1413	490	6A	G	V	A	L	V	L
N/A	2294	VICE1416	490	6A	G	V	A	L	V	L
N/A	2323	VICE1445	490	6A	G	V	A	L	V	L
N/A	2344	VICE1466	490	6A	G	V	A	L	V	L
N/A	2351	VICE1473	490	6A	G	V	A	L	V	L
N/A	2375	VICE1497	490	6A	G	V	A	L	V	L
N/A	2385	VICE1507	490	6A	G	V	A	L	V	L

N/A	2401	VICE1523	490	6A	G	V	A	L	V	L
N/A	2451	VICE1573	490	6A	G	V	A	L	V	L
N/A	2455	VICE1577	490	6A	G	V	A	L	V	L
N/A	2589	VICE1636	490	6A	G	V	A	L	V	L
N/A	2601	VICE1648	490	6A	G	V	A	L	V	L
N/A	2616	VICE1663	490	6A	G	V	A	L	V	L
N/A	2667	VICE1713	490	6A	G	V	A	L	V	L
N/A	2679	VICE1724	490	6A	G	V	A	L	V	L
N/A	2756	VICE1796	490	6A	G	V	A	L	V	L
N/A	2834	VICE1869	490	6A	G	V	A	L	V	L
N/A	2875	VICE1903	490	6A	G	V	A	L	V	L
N/A	560	VICE0239	395	6A	G	V	A	L	V	L
N/A	1260	VICE0666	395	6A	G	V	A	L	V	L
N/A	1272	VICE0678	395	6A	G	V	A	L	V	L
N/A	2623	VICE1670	395	6A	G	V	A	L	V	L
N/A	2777	VICE1816	395	6A	G	V	A	L	V	L
N/A	2778	VICE1817	395	6A	G	V	A	L	V	L
N/A	2779	VICE1818	395	6A	G	V	A	L	V	L
N/A	2782	VICE1821	395	6A	G	V	A	L	V	L
N/A	2787	VICE1826	395	6A	G	V	A	L	V	L
40171		SMRU384	315	6A	G	A	A	F	I	S
40178		SMRU433	315	6A	G	A	A	F	I	S
40365		SMRU875	315	6A	G	A	A	F	I	S
82482		SMRU2416	315	6A	G	A	A	F	I	S
83400		SMRU1921	315	6A	G	A	A	F	I	S
83598		SMRU2243	315	6A	G	A	A	F	I	S
103542		SMRU315	315	6A	G	A	A	F	I	S
103805		SMRU503	315	6A	G	A	A	F	I	S
82202		SMRU651	5963	6A	R	V	A	L	V	S
N/A	1003	SA11	1094	6A	G	A	A	F	I	S
N/A	1004	SA12	1094	6A	G	A	A	F	I	S
N/A	1005	SA13	1094	6A	G	A	A	F	I	S
N/A	1006	SA14	1094	6A	G	A	A	F	I	S
N/A	1007	SA15	1094	6A	G	A	A	F	I	S
N/A	1008	SA16	1094	6A	G	A	A	F	I	S
N/A	1009	SA17	1094	6A	G	A	A	F	I	S
N/A	1010	SA18	1094	6A	G	A	A	F	I	S
N/A	1011	SA19	1094	6A	G	A	A	F	I	S
N/A	1012	SA20	1094	6A	G	A	A	F	I	S
N/A	1013	SA21	1094	6A	G	A	A	F	I	S
N/A	1031	SA31	1094	6A	G	A	A	F	I	S
N/A	1032	SA32	1094	6A	G	A	A	F	I	S
N/A	1038	SA38	1094	6A	G	A	A	F	I	S
N/A	1044	SA44	1094	6A	G	A	A	F	I	S
N/A	1045	SA45	1094	6A	G	A	A	F	I	S
N/A	1069	SA59	1094	6A	G	A	A	F	I	S
N/A	1078	SA68	1094	6A	G	A	A	F	I	S
N/A	1082	SA72	1094	6A	G	A	A	F	I	S
34310		SMRU3035	90	6A	G	A	A	F	I	S
34318		SMRU3093	90	6A	G	A	A	F	I	S
40426		SMRU2516	90	6A	G	A	A	F	I	S
40593		SMRU2819	90	6A	G	A	A	F	I	S
82452		SMRU2506	90	6A	G	A	A	F	I	S
82588		SMRU2614	90	6A	G	A	A	F	I	S
82690		SMRU3039	90	6A	G	A	A	F	I	S
82747		SMRU3129	90	6A	G	A	A	F	I	S
82750		SMRU3133	90	6A	G	A	A	F	I	S
N/A	2542	VICE1589	176	6A	G	V	A	L	V	L
31984		SMRU1063	5477	6A	G	V	A	L	V	L
40201		SMRU933	5477	6A	G	V	A	L	V	L
40403		SMRU2053	5477	6A	G	V	A	L	V	L
55917		SMRU52	5477	6A	G	V	A	L	V	L
82180		SMRU938	5477	6A	G	V	A	L	V	L
82438		SMRU2174	5477	6A	G	V	A	L	V	L
82579		SMRU2603	5477	6A	G	V	A	L	V	L
83301		SMRU1244	5477	6A	G	V	A	L	V	L
83476		SMRU2450	5477	6A	G	V	A	L	V	L

83517		SMRU2569	5477	6A	G	V	A	L	V	L
83594		SMRU2235	5477	6A	G	V	A	L	V	L
83596		SMRU2241	5477	6A	G	V	A	L	V	L
103535		SMRU1293	5477	6A	G	V	A	L	V	L
103543		SMRU1444	5477	6A	G	V	A	L	V	L
103622		SMRU1533	5477	6A	G	V	A	L	V	L
103625		SMRU894	5477	6A	G	V	A	L	V	L
103825		SMRU18	5477	6A	G	V	A	L	V	L
34197		SMRU2409	4107	6A	G	V	A	L	V	L
34255		SMRU2270	4107	6A	G	V	A	L	V	L
40211		SMRU583	4107	6A	G	V	A	L	V	L
40543		SMRU2919	4107	6A	G	V	A	L	V	L
82351		SMRU1929	4107	6A	G	V	A	L	V	L
82367		SMRU1780	4107	6A	G	V	A	L	V	L
82510		SMRU2269	4107	6A	G	V	A	L	V	L
83347		SMRU849	4107	6A	G	V	A	L	V	L
83509		SMRU2552	4107	6A	G	V	A	L	V	L
83545		SMRU2894	4107	6A	G	V	A	L	V	L
103572		SMRU2987	4107	6A	G	V	A	L	V	L
103574		SMRU1964	4107	6A	G	V	A	L	V	L
103812		SMRU1898	4107	6A	G	V	A	L	V	L
34303		SMRU2756	4107	6A	G	V	A	L	V	L
82504		SMRU2654	4107	6A	G	V	A	L	V	L
34148		SMRU1592	2528/8011	6A	G	V	A	L	V	L
N/A	306	CDC1873-00	2090	6A	G	V	A	L	V	L
N/A	1140	GA17328	2090	6A	G	V	A	L	V	L
N/A	1144	GA19077	2090	6A	G	V	A	L	V	L
N/A	1151	GA41437	2090	6A	G	V	A	L	V	L
N/A	1193	NP127	2090	6A	G	V	A	L	V	L
31325		R34-3168	2090	6A	G	V	A	L	V	L
31328		R34-3171	2090	6A	G	V	A	L	V	L
31419		65435	2090	6A	G	V	A	L	V	L
31426		179743	2090	6A	G	V	A	L	V	L
31500		PT8105	2090	6A	G	V	A	L	V	L
32067		448643	2090	6A	G	V	A	L	V	L
32177		51715	2090	6A	G	V	A	L	V	L
32189		108480	2090	6A	G	V	A	L	V	L
32361		PT8074	2090	6A	G	V	A	L	V	L
32447		LE4007	2090	6A	G	V	A	L	V	L
33259		R34-3201	2090	6A	G	V	A	L	V	L
N/A	414	VICE0874	396	6A	G	V	A	L	V	L
N/A	742	VICE0420	396	6A	G	V	A	L	V	L
N/A	2354	VICE1476	396	6A	G	V	A	L	V	L
N/A	2368	VICE1490	396	6A	G	V	A	L	V	L
N/A	2846	VICE1879a	396	6A	G	V	A	L	V	L
115804		UoS0245	395	6A	G	V	A	L	V	L
121532		UoS2340	395	6A	G	V	A	L	V	L
33859		UoS4084	439	6A	G	V	A	L	V	L
121418		UoS2006	439	6A	G	V	A	L	V	L
31225		SMRU386	4936	6A	S	V	A	L	V	L
33919		SMRU922	4936	6A	S	V	A	L	V	L
33922		SMRU951	4936	6A	S	V	A	L	V	L
34135		SMRU879	4936	6A	S	V	A	L	V	L
40525		SMRU2873	4936	6A	S	V	A	L	V	L
44977		SMRU2225	4936	6A	S	V	A	L	V	L
44991		SMRU2915	4936	6A	S	V	A	L	V	L
55948		SMRU2028	4936	6A	S	V	A	L	V	L
55949		SMRU2072	4936	6A	S	V	A	L	V	L
82264		SMRU1500	4936	6A	S	V	A	L	V	L
82481		SMRU2414	4936	6A	S	V	A	L	V	L
82515		SMRU2287	4936	6A	S	V	A	L	V	L
82590		SMRU2618	4936	6A	S	V	A	L	V	L
83362		SMRU1447	4936	6A	S	V	A	L	V	L
83386		SMRU1570	4936	6A	S	V	A	L	V	L
83475		SMRU2415	4936	6A	S	V	A	L	V	L
103537		SMRU734	4936	6A	S	V	A	L	V	L
103539		SMRU2984	4936	6A	S	V	A	L	V	L

103550		SMRU2361	4936	6A	S	V	A	L	V	L
103556		SMRU2341	4936	6A	S	V	A	L	V	L
103571		SMRU1103	4936	6A	S	V	A	L	V	L
103585		SMRU1260	4936	6A	S	V	A	L	V	L
103594		SMRU1410	4936	6A	S	V	A	L	V	L
103599		SMRU1618	4936	6A	S	V	A	L	V	L
103600		SMRU1319	4936	6A	S	V	A	L	V	L
103603		SMRU1171	4936	6A	S	V	A	L	V	L
103616		SMRU2641	4936	6A	S	V	A	L	V	L
103809		SMRU1224	4936	6A	S	V	A	L	V	L

SEROTYPE 6A/6Bii/6C HYBRID SEQUENCE

					6	51	54	61	65	81
		6A reference			S	V	L	V	L	F
		6Bii reference			G	A	F	I	S	L
		6C reference			G	V	L	V	L	F
40171		SMRU384	315	6A	G	A	F	I	S	L
40178		SMRU433	315	6A	G	A	F	I	S	L
40365		SMRU875	315	6A	G	A	F	I	S	L
82482		SMRU2416	315	6A	G	A	F	I	S	L
83400		SMRU1921	315	6A	G	A	F	I	S	L
83598		SMRU2243	315	6A	G	A	F	I	S	L
103542		SMRU315	315	6A	G	A	F	I	S	L
103805		SMRU503	315	6A	G	A	F	I	S	L

SEROTYPE 6A/6Bii HYBRID SEQUENCE

					6	51	54	61	65	81
		6A reference			S	V	L	V	L	F
		6Bii reference			G	A	F	I	S	L
N/A	1003	SA11	1094	6A	G	A	F	I	S	L
N/A	1004	SA12	1094	6A	G	A	F	I	S	L
N/A	1005	SA13	1094	6A	G	A	F	I	S	L
N/A	1006	SA14	1094	6A	G	A	F	I	S	L
N/A	1007	SA15	1094	6A	G	A	F	I	S	L
N/A	1008	SA16	1094	6A	G	A	F	I	S	L
N/A	1009	SA17	1094	6A	G	A	F	I	S	L
N/A	1010	SA18	1094	6A	G	A	F	I	S	L
N/A	1011	SA19	1094	6A	G	A	F	I	S	L
N/A	1012	SA20	1094	6A	G	A	F	I	S	L
N/A	1013	SA21	1094	6A	G	A	F	I	S	L
N/A	1031	SA31	1094	6A	G	A	F	I	S	L
N/A	1032	SA32	1094	6A	G	A	F	I	S	L
N/A	1038	SA38	1094	6A	G	A	F	I	S	L
N/A	1044	SA44	1094	6A	G	A	F	I	S	L
N/A	1045	SA45	1094	6A	G	A	F	I	S	L
N/A	1069	SA59	1094	6A	G	A	F	I	S	L
N/A	1078	SA68	1094	6A	G	A	F	I	S	L
N/A	1082	SA72	1094	6A	G	A	F	I	S	L
34310		SMRU3035	90	6A	G	A	F	I	S	L
34318		SMRU3093	90	6A	G	A	F	I	S	L
40426		SMRU2516	90	6A	G	A	F	I	S	L
40593		SMRU2819	90	6A	G	A	F	I	S	L
82202		SMRU651	5963	6A	R	V	L	V	S	F
82452		SMRU2506	90	6A	G	A	F	I	S	L
82588		SMRU2614	90	6A	G	A	F	I	S	L
82690		SMRU3039	90	6A	G	A	F	I	S	L
82747		SMRU3129	90	6A	G	A	F	I	S	L
82750		SMRU3133	90	6A	G	A	F	I	S	L

SEROTYPE 6A/6C HYBRID SEQUENCE

						9	129	321	328	331
		6A reference				S	Q	M	V	H
		6C reference				G	H	L	I	Y
	2542	VICE1589	176	6A	2	G	Q	L	I	Y
31984		SMRU1063	5477	6A	2	G	Q	L	I	Y
34197		SMRU2409	4107	6A	2	G	Q	L	I	Y
34255		SMRU2270	4107	6A	2	G	Q	L	I	Y
34303		SMRU2756	4107	6A	2	G	Q	L	I	Y
40201		SMRU933	5477	6A	2	G	Q	L	I	Y
40211		SMRU583	4107	6A	2	G	Q	L	I	Y
40403		SMRU2053	5477	6A	2	G	Q	L	I	Y
40543		SMRU2919	4107	6A	2	G	Q	L	I	Y
55917		SMRU52	5477	6A	2	G	Q	L	I	Y
82180		SMRU938	5477	6A	2	G	Q	L	I	Y
82351		SMRU1929	4107	6A	2	G	Q	L	I	Y
82367		SMRU1780	4107	6A	2	G	Q	L	I	Y
82438		SMRU2174	5477	6A	2	G	Q	L	I	Y
82504		SMRU2654	4107	6A	2	G	Q	L	I	Y
82510		SMRU2269	4107	6A	2	G	Q	L	I	Y
82579		SMRU2603	5477	6A	2	G	Q	L	I	Y
83301		SMRU1244	5477	6A	2	G	Q	L	I	Y
83347		SMRU849	4107	6A	2	G	Q	L	I	Y
83476		SMRU2450	5477	6A	2	G	Q	L	I	Y
83509		SMRU2552	4107	6A	2	G	Q	L	I	Y
83517		SMRU2569	5477	6A	2	G	Q	L	I	Y
83545		SMRU2894	4107	6A	2	G	Q	L	I	Y
83594		SMRU2235	5477	6A	2	G	Q	L	I	Y
83596		SMRU2241	5477	6A	2	G	Q	L	I	Y
103535		SMRU1293	5477	6A	2	G	Q	L	I	Y
103543		SMRU1444	5477	6A	2	G	Q	L	I	Y
103572		SMRU2987	4107	6A	2	G	Q	L	I	Y
103574		SMRU1964	4107	6A	2	G	Q	L	I	Y
103622		SMRU1533	5477	6A	2	G	Q	L	I	Y
103625		SMRU894	5477	6A	2	G	Q	L	I	Y
103812		SMRU1898	4107	6A	2	G	Q	L	I	Y
103825		SMRU18	5477	6A	2	G	Q	L	I	Y

SEROTYPE 6A/6C/23X HYBRID SEQUENCE

						9	47	68	72	81
		6A reference				S	L	Y	E	F
		6C reference				G	L	Y	E	F
82249	N/A	SMRU1268	6707/10567	23X		G	F	H	A	L
34148		SMRU1592	2528/8011	6A	2	G	L	Y	E	F

					9	51	54	61	65	81
		6A reference			S	V	L	V	L	F
		6Bii reference			G	A	F	I	S	L
N/A	306	CDC1873-00	2090	6A	G	V	L	V	L	F
N/A	414	VICE0874	396	6A	G	V	L	V	L	F
N/A	742	VICE0420	396	6A	G	V	L	V	L	F
N/A	1140	GA17328	2090	6A	G	V	L	V	L	F
N/A	1144	GA19077	2090	6A	G	V	L	V	L	F
N/A	1151	GA41437	2090	6A	G	V	L	V	L	F
N/A	1193	NP127	2090	6A	G	V	L	V	L	F
N/A	2354	VICE1476	396	6A	G	V	L	V	L	F
N/A	2368	VICE1490	396	6A	G	V	L	V	L	F
N/A	2846	VICE1879a	396	6A	G	V	L	V	L	F
31325		R34-3168	2090	6A	G	V	L	V	L	F
31328		R34-3171	2090	6A	G	V	L	V	L	F
31419		65435	2090	6A	G	V	L	V	L	F
31426		179743	2090	6A	G	V	L	V	L	F
31500		PT8105	2090	6A	G	V	L	V	L	F
32067		448643	2090	6A	G	V	L	V	L	F
32177		51715	2090	6A	G	V	L	V	L	F
32189		108480	2090	6A	G	V	L	V	L	F
32361		PT8074	2090	6A	G	V	L	V	L	F
32447		LE4007	2090	6A	G	V	L	V	L	F
33259		R34-3201	2090	6A	G	V	L	V	L	F
33859		UoS4084	439	6A	G	V	L	V	L	F
115804		UoS0245	395	6A	G	V	L	V	L	F
121418		UoS2006	439	6A	G	V	L	V	L	F
121532		UoS2340	395	6A	G	V	L	V	L	F

SEROTYPE 6A/6Bii HYBRID SEQUENCE

				9	129	160	162	306
	6A reference			S	Q	T	D	D
	6C reference			G	H	T	D	D
	18F reference			G	Q	T	N	D
31225	SMRU386	4936	6A	S	Q	M	N	N
33919	SMRU922	4936	6A	S	Q	M	N	N
33922	SMRU951	4936	6A	S	Q	M	N	N
34135	SMRU879	4936	6A	S	Q	M	N	N
40525	SMRU2873	4936	6A	S	Q	M	N	N
44977	SMRU2225	4936	6A	S	Q	M	N	N
44991	SMRU2915	4936	6A	S	Q	M	N	N
55948	SMRU2028	4936	6A	S	Q	M	N	N
55949	SMRU2072	4936	6A	S	Q	M	N	N
82264	SMRU1500	4936	6A	S	Q	M	N	N
82481	SMRU2414	4936	6A	S	Q	M	N	N
82515	SMRU2287	4936	6A	S	Q	M	N	N
82590	SMRU2618	4936	6A	S	Q	M	N	N
83362	SMRU1447	4936	6A	S	Q	M	N	N
83386	SMRU1570	4936	6A	S	Q	M	N	N
83475	SMRU2415	4936	6A	S	Q	M	N	N
103537	SMRU734	4936	6A	S	Q	M	N	N
103539	SMRU2984	4936	6A	S	Q	M	N	N
103550	SMRU2361	4936	6A	S	Q	M	N	N
103556	SMRU2341	4936	6A	S	Q	M	N	N
103571	SMRU1103	4936	6A	S	Q	M	N	N
103585	SMRU1260	4936	6A	S	Q	M	N	N
103594	SMRU1410	4936	6A	S	Q	M	N	N
103599	SMRU1618	4936	6A	S	Q	M	N	N
103600	SMRU1319	4936	6A	S	Q	M	N	N
103603	SMRU1171	4936	6A	S	Q	M	N	N

103616		SMRU2641	4936	6A	S	Q	M	N	N	
103809		SMRU1224	4936	6A	S	Q	M	N	N	

SEROTYPE 6A ISOLATES (NO HYBRID SEQUENCES)

					wzg					
					9	53	123	143	160	162
		6A reference			S	A	E	N	T	D
N/A	662	VICE0340	15	6A	G	A	D	N	T	D
N/A	755	VICE0433	15	6A	G	A	D	N	T	D
N/A	959	VICE0637	15	6A	G	A	D	N	T	D
N/A	1320	VICE0726	15	6A	G	A	D	N	T	D
N/A	1485	VICE0934	15	6A	G	A	D	N	T	D
N/A	1488	VICE0937a	15	6A	G	A	D	N	T	D
N/A	1493	VICE0942	15	6A	G	A	D	N	T	D
N/A	1495	VICE0944	15	6A	G	A	D	N	T	D
N/A	1499	VICE0948	15	6A	G	A	D	N	T	D
N/A	1615	VICE1064	15	6A	G	A	D	N	T	D
N/A	1625	VICE1074	15	6A	G	A	D	N	T	D
N/A	2101	VICE1223a	15	6A	G	A	D	N	T	D
N/A	2642	VICE1689	15	6A	G	A	D	N	T	D
31318		R34-3027	473	6A	G	A	D	N	T	D
31337		R34-3180	690	6A	G	A	D	N	T	D
31349		R34-3096	473	6A	G	A	D	N	T	D
31382		R34-3151	473	6A	G	A	D	N	T	D
31410		R34-3211	473	6A	G	A	D	N	T	D
31417		15445	473	6A	G	A	D	N	T	D
31418		42861	690	6A	G	A	D	N	T	D
31425		166637	473	6A	G	A	D	N	T	D
31427		181272	473	6A	G	A	D	N	T	D
31461		359370	473	6A	G	A	D	N	T	D
32085		R34-3099	473	6A	G	A	D	N	T	D
32154		R34-3210	473	6A	G	A	D	N	T	D
32187		101058	690	6A	G	A	D	N	T	D
32418		CH2090	473	6A	G	A	D	N	T	D
33175		420881	473	6A	G	A	D	N	T	D
121412		UoS0050	473	6A	G	A	D	N	T	D
121447		UoS4399	473	6A	G	A	D	N	T	D
33266		110093	745/1939	6A	G	A	D	N	T	D
N/A	216	ICE11	490	6A	G	A	D	N	T	D
N/A	388	VICE0863	490	6A	G	A	D	N	T	D
N/A	559	VICE0238	490	6A	G	A	D	N	T	D
N/A	576	VICE0254	490	6A	G	A	D	N	T	D
N/A	580	VICE0258	490	6A	G	A	D	N	T	D
N/A	630	VICE0308	490	6A	G	A	D	N	T	D
N/A	633	VICE0311	490	6A	G	A	D	N	T	D
N/A	659	VICE0337	490	6A	G	A	D	N	T	D
N/A	676	VICE0354	490	6A	G	A	D	N	T	D
N/A	712	VICE0390	490	6A	G	A	D	N	T	D
N/A	782	VICE0460	490	6A	G	A	D	N	T	D
N/A	787	VICE0465	490	6A	G	A	D	N	T	D
N/A	792	VICE0470	490	6A	G	A	D	N	T	D
N/A	912	VICE0590	490	6A	G	A	D	N	T	D
N/A	914	VICE0592	490	6A	G	A	D	N	T	D
N/A	930	VICE0608	490	6A	G	A	D	N	T	D
N/A	963	VICE0641	490	6A	G	A	D	N	T	D
N/A	1245	VICE0651	490	6A	G	A	D	N	T	D
N/A	1303	VICE0709	490	6A	G	A	D	N	T	D
N/A	1316	VICE0722	490	6A	G	A	D	N	T	D
N/A	1330	VICE0736	490	6A	G	A	D	N	T	D
N/A	1363	VICE0769	490	6A	G	A	D	N	T	D
N/A	1378	VICE0784	490	6A	G	A	D	N	T	D
N/A	1393	VICE0799	490	6A	G	A	D	N	T	D

N/A	1425	VICE0831	490	6A	G	A	D	N	T	D
N/A	1440	VICE0889	490	6A	G	A	D	N	T	D
N/A	1454	VICE0903	490	6A	G	A	D	N	T	D
N/A	1484	VICE0933	490	6A	G	A	D	N	T	D
N/A	1540	VICE0989a	490	6A	G	A	D	N	T	D
N/A	1564	VICE1013	490	6A	G	A	D	N	T	D
N/A	1565	VICE1014	490	6A	G	A	D	N	T	D
N/A	1566	VICE1015	490	6A	G	A	D	N	T	D
N/A	1581	VICE1030	490	6A	G	A	D	N	T	D
N/A	1606	VICE1055	490	6A	G	A	D	N	T	D
N/A	1609	VICE1058	490	6A	G	A	D	N	T	D
N/A	1675	VICE1124	490	6A	G	A	D	N	T	D
N/A	1676	VICE1125	490	6A	G	A	D	N	T	D
N/A	2023	VICE1145a	490	6A	G	A	D	N	T	D
N/A	2048	VICE1170	490	6A	G	A	D	N	T	D
N/A	2049	VICE1171	490	6A	G	A	D	N	T	D
N/A	2061	VICE1183	490	6A	G	A	D	N	T	D
N/A	2069	VICE1191	490	6A	G	A	D	N	T	D
N/A	2089	VICE1211	490	6A	G	A	D	N	T	D
N/A	2121	VICE1243	490	6A	G	A	D	N	T	D
N/A	2124	VICE1246	490	6A	G	A	D	N	T	D
N/A	2134	VICE1256	490	6A	G	A	D	N	T	D
N/A	2159	VICE1281	490	6A	G	A	D	N	T	D
N/A	2172	VICE1294	490	6A	G	A	D	N	T	D
N/A	2193	VICE1315	490	6A	G	A	D	N	T	D
N/A	2211	VICE1333	490	6A	G	A	D	N	T	D
N/A	2213	VICE1335	490	6A	G	A	D	N	T	D
N/A	2219	VICE1341	490	6A	G	A	D	N	T	D
N/A	2240	VICE1362	490	6A	G	A	D	N	T	D
N/A	2247	VICE1369	490	6A	G	A	D	N	T	D
N/A	2255	VICE1377	490	6A	G	A	D	N	T	D
N/A	2282	VICE1404	490	6A	G	A	D	N	T	D
N/A	2291	VICE1413	490	6A	G	A	D	N	T	D
N/A	2294	VICE1416	490	6A	G	A	D	N	T	D
N/A	2323	VICE1445	490	6A	G	A	D	N	T	D
N/A	2344	VICE1466	490	6A	G	A	D	N	T	D
N/A	2351	VICE1473	490	6A	G	A	D	N	T	D
N/A	2375	VICE1497	490	6A	G	A	D	N	T	D
N/A	2385	VICE1507	490	6A	G	A	D	N	T	D
N/A	2401	VICE1523	490	6A	G	A	D	N	T	D
N/A	2451	VICE1573	490	6A	G	A	D	N	T	D
N/A	2455	VICE1577	490	6A	G	A	D	N	T	D
N/A	2589	VICE1636	490	6A	G	A	D	N	T	D
N/A	2601	VICE1648	490	6A	G	A	D	N	T	D
N/A	2616	VICE1663	490	6A	G	A	D	N	T	D
N/A	2667	VICE1713	490	6A	G	A	D	N	T	D
N/A	2679	VICE1724	490	6A	G	A	D	N	T	D
N/A	2756	VICE1796	490	6A	G	A	D	N	T	D
N/A	2834	VICE1869	490	6A	G	A	D	N	T	D
N/A	2875	VICE1903	490	6A	G	A	D	N	T	D
32034		SMRU2443	4113	6A	S	A	E	N	T	D
33945		SMRU991	4536/6767	6A	S	A	E	N	T	D
34146		SMRU1588	4536/6767	6A	S	A	E	N	T	D
34155		SMRU1946	4536/6767	6A	S	A	E	N	T	D
34167		SMRU2020	4536/6767	6A	S	A	E	N	T	D
34319		SMRU2934	4536/6767	6A	S	A	E	N	T	D
40394		SMRU1976	4536/6767	6A	S	A	E	N	T	D
40503		SMRU2293	4536/6767	6A	S	A	E	N	T	D
40523		SMRU2868	4536/6767	6A	S	A	E	N	T	D
40580		SMRU2793	4536/6767	6A	S	A	E	N	T	D
40603		SMRU3080	4536/6767	6A	S	A	E	N	T	D
82210		SMRU692	4536/6767	6A	S	A	E	N	T	D
82317		SMRU1714	4536/6767	6A	S	A	E	N	T	D
82740		SMRU2253	4536/6767	6A	S	A	E	N	T	D
83410		SMRU1975	4536/6767	6A	S	A	E	N	T	D
83520		SMRU2580	4536/6767	6A	S	A	E	N	T	D
83609		SMRU3107	4536/6767	6A	S	A	E	N	T	D

103516		SMRU2184	4536/6767	6A	S	A	E	N	T	D
103548		SMRU1888	4536/6767	6A	S	A	E	N	T	D
103553		SMRU1610	4536/6767	6A	S	A	E	N	T	D
103554		SMRU1566	4536/6767	6A	S	A	E	N	T	D
103575		SMRU212	4536/6767	6A	S	A	E	N	T	D
103597		SMRU1197	4536/6767	6A	S	A	E	N	T	D
103618		SMRU3016	4536/6767	6A	S	A	E	N	T	D
103803		SMRU489	4536/6767	6A	S	A	E	N	T	D
103831		SMRU1370	4536/6767	6A	S	A	E	N	T	D
18190		DG29	81	6A	S	A	E	N	M	N
39946		DG80	81	6A	S	A	E	N	M	N
N/A	2643	VICE1690	9789	6A	R	A	E	N	M	N
N/A	560	VICE0239	395	6A	G	A	E	H	T	N
N/A	1260	VICE0666	395	6A	G	A	E	H	T	N
N/A	1272	VICE0678	395	6A	G	A	E	H	T	N
N/A	2623	VICE1670	395	6A	G	A	E	H	T	N
N/A	2777	VICE1816	395	6A	G	A	E	H	T	N
N/A	2778	VICE1817	395	6A	G	A	E	H	T	N
N/A	2779	VICE1818	395	6A	G	A	E	H	T	N
N/A	2782	VICE1821	395	6A	G	A	E	H	T	N
N/A	2787	VICE1826	395	6A	G	A	E	H	T	N
N/A	1142	GA17971	Sing1175	6A	S	A	E	N	T	D
33136		SMRU2446	982	6A	S	A	E	N	T	D
34268		SMRU2367	982	6A	S	A	E	N	T	D
40413		SMRU2186	982	6A	S	A	E	N	T	D
44978		SMRU2263	982	6A	S	A	E	N	T	D
44987		SMRU2380	982	6A	S	A	E	N	T	D
66480		SMRU1655	982	6A	S	A	E	N	T	D
82429		SMRU2158	982	6A	S	A	E	N	T	D
82450		SMRU2502	982	6A	S	A	E	N	T	D
82513		SMRU2281	982	6A	S	A	E	N	T	D
82548		SMRU2398	Singleton10056	6A	S	A	E	N	T	D
82574		SMRU2595	982	6A	S	A	E	N	T	D
82691		SMRU3040	982	6A	S	A	E	N	T	D
82715		SMRU2966	982	6A	S	A	E	N	T	D
82720		SMRU2978	982	6A	S	A	E	N	T	D
83479		SMRU2478	982	6A	S	A	E	N	T	D
83487		SMRU2261	982	6A	S	A	E	N	T	D
83507		SMRU2399	982	6A	S	A	E	N	T	D
83525		SMRU2596	982	6A	S	A	E	N	T	D
83587		SMRU2960	982	6A	S	A	E	N	T	D
103555		SMRU1172	982	6A	S	A	E	N	T	D
103565		SMRU572	982	6A	S	A	E	N	T	D
103592		SMRU1233	982	6A	S	A	E	N	T	D
103605		SMRU2347	982	6A	S	A	E	N	T	D
103617		SMRU1681	982	6A	S	A	E	N	T	D
103623		SMRU2956	982	6A	S	A	E	N	T	D
103813		SMRU1928	Singleton10056	6A	S	A	E	N	T	D
103836		SMRU1920	982	6A	S	A	E	N	T	D
31317		R34-3026	242	6A	S	A	E	N	T	D
N/A	420	VICE0877	460	6A	S	A	E	N	T	D
N/A	454	VICE0133	460	6A	S	A	E	N	T	D
N/A	474	VICE0153	460	6A	S	A	E	N	T	D
N/A	540	VICE0219	460	6A	S	A	E	N	T	D
N/A	594	VICE0272	460	6A	S	A	E	N	T	D
N/A	609	VICE0287	460	6A	S	A	E	N	T	D
N/A	673	VICE0351	460	6A	S	A	E	N	T	D
N/A	674	VICE0352	460	6A	S	A	E	N	T	D
N/A	704	VICE0382	460	6A	S	A	E	N	T	D
N/A	706	VICE0384	460	6A	S	A	E	N	T	D
N/A	715	VICE0393	176	6A	S	A	E	N	T	D
N/A	716	VICE0394	460	6A	S	A	E	N	T	D
N/A	720	VICE0398	460	6A	S	A	E	N	T	D
N/A	721	VICE0399	460	6A	S	A	E	N	T	D
N/A	724	VICE0402	460	6A	S	A	E	N	T	D
N/A	800	VICE0478	460	6A	S	A	E	N	T	D
N/A	814	VICE0492	460	6A	S	A	E	N	T	D

N/A	822	VICE0500	460	6A	S	A	E	N	T	D
N/A	861	VICE0539	460	6A	S	A	E	N	T	D
N/A	906	VICE0584	460	6A	S	A	E	N	T	D
N/A	917	VICE0595	460	6A	S	A	E	N	T	D
N/A	945	VICE0623	460	6A	S	T	E	N	T	D
N/A	1108	SP6-BS73	460	6A	S	A	E	N	T	D
N/A	1249	VICE0655	460	6A	S	A	E	N	T	D
N/A	1278	VICE0684	460	6A	S	A	E	N	T	D
N/A	1422	VICE0828	460	6A	S	A	E	N	T	D
N/A	1450	VICE0899	460	6A	S	A	E	N	T	D
N/A	1545	VICE0994	460	6A	S	A	E	N	T	D
N/A	1558	VICE1007	460	6A	S	A	E	N	T	D
N/A	1569	VICE1018	460	6A	S	A	E	N	T	D
N/A	2055	VICE1177	460	6A	S	A	E	N	T	D
N/A	2086	VICE1208	460	6A	S	A	E	N	T	D
N/A	2129	VICE1251	460	6A	S	A	E	N	T	D
N/A	2132	VICE1254	460	6A	S	A	E	N	T	D
N/A	2181	VICE1303	460	6A	S	A	E	N	T	D
N/A	2186	VICE1308	460	6A	S	A	E	N	T	D
N/A	2203	VICE1325	460	6A	S	A	E	N	T	D
N/A	2204	VICE1326	460	6A	S	A	E	N	T	D
N/A	2205	VICE1327	460	6A	S	A	E	N	T	D
N/A	2218	VICE1340	176	6A	S	A	E	N	T	D
N/A	2223	VICE1345	460	6A	S	A	E	N	T	D
N/A	2224	VICE1346	460	6A	S	A	E	N	T	D
N/A	2226	VICE1348	460	6A	S	A	E	N	T	D
N/A	2227	VICE1349	460	6A	S	A	E	N	T	D
N/A	2228	VICE1350	460	6A	S	A	E	N	T	D
N/A	2234	VICE1356	460	6A	S	A	E	N	T	D
N/A	2258	VICE1380	460	6A	S	A	E	N	T	D
N/A	2261	VICE1383	460	6A	S	A	E	N	T	D
N/A	2262	VICE1384	460	6A	S	A	E	N	T	D
N/A	2308	VICE1430	460	6A	S	A	E	N	T	D
N/A	2396	VICE1518	460	6A	S	A	E	N	T	D
N/A	2414	VICE1536	460	6A	S	A	E	N	T	D
N/A	2430	VICE1552	460	6A	S	A	E	N	T	D
N/A	2462	VICE1584	460	6A	S	A	E	N	T	D
N/A	2503	VICE1257b	460	6A	S	A	E	N	T	D
N/A	2587	VICE1634	460	6A	S	A	E	N	T	D
N/A	2600	VICE1647	460	6A	S	A	E	N	T	D
N/A	2621	VICE1668	460	6A	S	A	E	N	T	D
N/A	2660	VICE1707	460	6A	S	A	E	N	T	D
N/A	2790	VICE1829	460	6A	S	A	E	N	T	D
N/A	2833	VICE1868	460	6A	S	A	E	N	T	D
N/A	2892	6A/2	460	6A	S	A	E	N	T	D
N/A	3031	VICE1943	460	6A	S	A	E	N	T	D
31302		R34-3075	460	6A	S	A	E	N	T	D
31311		R34-3018	460	6A	S	A	E	N	T	D
31323		R34-3166	460	6A	S	A	E	N	T	D
31415		1996	460	6A	S	A	E	N	T	D
31448		299801	460	6A	S	A	E	N	T	D
31458		336850	460	6A	S	A	E	N	T	D
31466		383739	460	6A	S	A	E	N	T	D
32051		R34-3035	460	6A	S	A	E	N	T	D
32066		446376	460	6A	S	A	E	N	T	D
32068		472699	460	6A	S	A	E	N	T	D
32159		R34-3220	460	6A	S	A	E	N	T	D
32161		R34-3222	460	6A	S	A	E	N	T	D
32179		62031	460	6A	S	A	E	N	T	D
32183		89848	460	6A	S	A	E	N	T	D
32190		118039	460	6A	S	A	E	N	T	D
32193		135631	460	6A	S	A	E	N	T	D
32322		ND6134	460	6A	S	A	E	N	T	D
33262		42397	460	6A	S	A	E	N	T	D
40131		UoS0301	460	6A	S	A	E	N	T	D
44824		UoS3293	460	6A	S	A	E	N	T	D
55126		UoS4242	460	6A	S	A	E	N	T	D

115806	UoS0266	460	6A	S	A	E	N	T	D
121562	UoS2346	460	6A	S	A	E	N	T	D
121586	UoS0154	460	6A	S	A	E	N	T	D
121590	UoS2319	460	6A	S	A	E	N	T	D
121591	UoS0194	460	6A	S	A	E	N	T	D
121592	UoS3200	460	6A	S	A	E	N	T	D
121593	UoS0113	460	6A	S	A	E	N	T	D
121595	UoS0075	460	6A	S	A	E	N	T	D
121600	UoS2166	460	6A	S	A	E	N	T	D
121601	UoS5284	460	6A	S	A	E	N	T	D
121607	UoS4081	460	6A	S	A	E	N	T	D
121608	UoS2233	460	6A	S	A	E	N	T	D
121609	UoS2038	460	6A	S	A	E	N	T	D
121610	UoS4287	460	6A	S	A	E	N	T	D
121613	UoS2307	460	6A	S	A	E	N	T	D
121620	UoS2002	460	6A	S	A	E	N	T	D
121621	UoS2001	460	6A	S	A	E	N	T	D
121651	UoS2308	460	6A	S	A	E	N	T	D
123190	UoS0068	460	6A	S	A	E	N	T	D
123220	UoS3122	460	6A	S	A	E	N	T	D
123242	UoS2323	460	6A	S	A	E	N	T	D
123244	UoS2337	460	6A	S	A	E	N	T	D
123312	UoS3063	460	6A	S	A	E	N	T	D
123317	UoS3171	460	6A	S	A	E	N	T	D
123324	UoS3235	460	6A	S	A	E	N	T	D
123336	UoS2343	460	6A	S	A	E	N	T	D
121603	UoS2359	460	6A	S	A	E	N	T	D

cluster 1 (6A ref)
cluster 2
cluster 3
cluster 4
cluster 5

																					wzg	
81	114	123	143	157	160	162	169	172	178	199	212	306	321	328	331	337	338	339	340	341	342	344
F	I	E	N	T	T	D	A	K	E	Y	T	D	M	V	H	S	T	-	-	H	G	F
F	I	E	N	T	T	D	A	K	E	Y	T	D	M	V	H	S	T	-	-	H	G	F
F	I	E	N	T	T	D	A	K	K	Y	T	D	M	V	H	S	T	-	-	H	G	F
F	I	E	N	T	T	D	A	K	K	Y	T	D	M	V	H	S	T	-	-	H	G	F
F	I	E	N	T	T	D	A	K	K	Y	T	D	M	V	H	S	T	-	-	H	G	F
F	I	E	N	T	T	D	A	K	K	Y	T	D	M	V	H	S	T	-	-	H	G	F
F	I	E	N	T	T	D	A	K	K	Y	T	D	M	V	H	S	T	-	-	H	G	F
F	I	E	N	T	T	D	A	K	K	Y	T	D	M	V	H	S	T	-	-	H	G	F
F	I	E	N	T	T	D	A	K	K	Y	T	D	M	V	H	S	T	-	-	H	G	F
F	I	E	N	T	T	D	A	K	K	Y	T	D	M	V	H	S	T	-	-	H	G	F
F	I	E	N	T	T	D	A	K	K	Y	T	D	M	V	H	S	T	-	-	H	G	F
F	I	E	N	T	T	D	A	K	K	Y	T	D	M	V	H	S	T	-	-	H	G	F
F	I	E	N	T	T	D	A	K	K	Y	T	D	M	V	H	S	T	-	-	H	G	F
F	I	E	N	T	T	D	A	K	K	Y	T	D	M	V	H	S	T	-	-	H	G	F
F	I	E	N	T	T	D	A	K	K	Y	T	D	M	V	H	S	T	-	-	H	G	F
F	I	E	N	T	T	D	A	K	K	Y	T	D	M	V	H	S	T	-	-	H	G	F
F	I	E	N	T	T	D	A	K	K	Y	T	D	M	V	H	S	T	-	-	H	G	F
F	I	E	N	T	T	D	A	K	K	Y	T	D	M	V	H	S	T	-	-	H	G	F
F	I	E	N	T	T	D	A	K	K	Y	T	D	M	V	H	S	T	-	-	H	G	F
F	I	E	N	T	T	D	A	K	K	Y	T	D	M	V	H	S	T	-	-	H	G	F
F	I	E	N	T	T	D	A	K	K	Y	T	D	M	V	H	S	T	-	-	H	G	F
F	I	E	N	T	T	D	A	K	K	Y	T	D	M	V	H	S	T	-	-	H	G	F
F	I	E	N	T	T	D	A	K	K	Y	T	D	M	V	H	S	T	-	-	H	G	F
F	I	E	N	T	T	D	A	K	K	Y	T	D	M	V	H	S	T	-	-	H	G	F
F	I	E	N	T	T	D	A	K	K	Y	T	D	M	V	H	S	T	-	-	H	G	F
F	I	E	N	T	T	D	A	K	K	Y	T	D	M	V	H	S	T	-	-	H	G	F
F	I	E	N	T	T	D	A	K	K	Y	T	D	M	V	H	S	T	-	-	H	G	F
F	I	E	N	T	T	D	A	K	K	Y	T	D	M	V	H	S	T	-	-	H	G	F
F	I	E	N	T	T	D	A	K	K	Y	T	D	M	V	H	S	T	-	-	H	G	F
F	I	E	N	T	T	D	A	K	K	Y	T	D	M	V	H	S	T	-	-	H	G	F
F	I	D	N	T	T	D	A	K	E	Y	T	D	M	V	H	S	T	-	-	H	G	F
F	I	D	N	T	T	D	A	K	E	Y	T	D	M	V	H	S	T	-	-	H	G	F
F	I	D	N	T	T	D	A	K	E	Y	T	D	M	V	H	S	T	-	-	H	G	F
F	I	D	N	T	T	D	A	K	E	Y	T	D	M	V	H	S	T	-	-	H	G	F
F	I	D	N	T	T	D	A	K	E	Y	T	D	M	V	H	S	T	-	-	H	G	F
F	I	D	N	T	T	D	A	K	E	Y	T	D	M	V	H	S	T	-	-	H	G	F
F	I	D	N	T	T	D	A	K	E	Y	T	D	M	V	H	S	T	-	-	H	G	F
F	I	D	N	T	T	D	A	K	E	Y	T	D	M	V	H	S	T	-	-	H	G	F
F	I	D	N	T	T	D	A	K	E	Y	T	D	M	V	H	S	T	-	-	H	G	F
F	I	D	N	T	T	D	A	K	E	Y	T	D	M	V	H	S	T	-	-	H	G	F
F	I	D	N	T	T	D	A	K	E	Y	T	D	M	V	H	S	T	-	-	H	G	F
F	I	D	N	T	T	D	A	K	E	Y	T	D	M	V	H	S	T	-	-	H	G	F
F	I	D	N	T	T	D	A	K	E	Y	T	D	M	V	H	S	T	-	-	H	G	F
F	I	D	N	T	T	D	A	K	E	Y	T	D	M	V	H	S	T	-	-	H	G	F
F	I	D	N	T	T	D	A	K	E	Y	T	D	M	V	H	S	T	-	-	H	G	F
F	I	D	N	T	T	D	A	K	E	Y	T	D	M	V	H	S	T	-	-	H	G	F
F	I	D	N	T	T	D	A	K	E	Y	T	D	M	V	H	S	T	-	-	H	G	F
F	I	D	N	T	T	D	A	K	E	Y	T	D	M	V	H	S	T	-	-	H	G	F
F	I	D	N	T	T	D	A	K	E	Y	T	D	M	V	H	S	T	-	-	H	G	F
F	I	D	N	T	T	D	A	K	E	Y	T	D	M	V	H	S	T	-	-	H	G	F
F	I	D	N	T	T	D	A	K	E	Y	T	D	M	V	H	S	T	-	-	H	G	F
F	I	D	N	T	T	D	A	K	E	Y	T	D	M	V	H	S	T	-	-	H	G	F
F	I	D	N	T	T	D	A	K	E	Y	T	D	M	V	H	S	T	-	-	H	G	F
F	I	E	N	T	T	D	A	K	E	Y	T	D	M	V	H	S	T	-	-	H	G	F
F	I	D	N	T	T	D	A	K	E	Y	T	D	M	V	H	S	T	-	-	H	G	F
F	I	D	N	T	T	D	A	K	E	Y	T	D	M	V	H	S	T	-	-	H	G	F
F	I	D	N	T	T	D	A	K	E	Y	T	D	M	V	H	S	T	-	-	H	G	F
F	I	D	N	T	T	D	A	K	E	Y	T	D	M	V	H	S	T	-	-	H	G	F
F	I	D	N	T	T	D	A	K	E	Y	T	D	M	V	H	S	T	-	-	H	G	F
F	I	E	N	T	T	D	A	K	E	Y	T	D	M	V	H	S	T	-	-	H	G	F
F	I	E	N	T	T	D	A	K	E	Y	T	D	M	V	H	S	T	-	-	H	G	F
F	I	E	N	T	T	D	A	K	E	Y	T	D	M	V	H	S	T	-	-	H	G	F
F	I	E	N	T	T	D	A	K	E	Y	T	D	M	V	H	S	T	-	-	H	G	F

F	I	E	N	T	T	D	A	K	E	Y	T	D	M	V	H	S	T	-	-	H	G	F
F	I	E	N	T	T	D	A	K	E	Y	T	D	M	V	H	S	T	-	-	H	G	F
F	I	E	N	T	T	D	A	K	E	Y	T	D	M	V	H	S	T	-	-	H	G	F
F	I	E	N	T	T	D	A	K	E	Y	T	D	M	V	H	S	T	-	-	H	G	F
F	I	E	N	T	T	D	A	K	E	Y	T	D	M	V	H	S	T	-	-	H	G	F
F	I	E	N	T	T	D	A	K	E	Y	T	D	M	V	H	S	T	-	-	H	G	F
F	I	E	N	T	T	D	A	K	E	Y	T	D	M	V	H	S	T	-	-	H	G	F
F	I	E	N	T	T	D	A	K	E	Y	T	D	M	V	H	S	T	-	-	H	G	F
F	I	E	N	T	T	D	A	K	E	Y	T	D	M	V	H	S	T	-	-	H	G	F
F	I	E	N	T	T	D	A	K	E	Y	T	D	M	V	H	S	T	-	-	H	G	F
F	I	E	N	T	T	D	A	K	E	Y	T	D	M	V	H	S	T	-	-	H	G	F
F	I	E	N	T	T	D	A	K	E	Y	T	D	M	V	H	S	T	-	-	H	G	F
F	I	E	N	T	T	D	A	K	E	Y	T	D	M	V	H	S	T	-	-	H	G	F
F	I	E	N	T	T	D	A	K	E	Y	T	D	M	V	H	S	T	-	-	H	G	F
F	I	E	N	T	T	D	A	K	E	Y	T	D	M	V	H	S	T	-	-	H	G	F
F	I	E	N	T	T	D	A	K	E	Y	T	D	M	V	H	S	T	-	-	H	G	F
F	I	E	N	T	T	D	A	K	E	Y	T	D	M	V	H	S	T	-	-	H	G	F
F	I	E	N	T	T	D	A	K	E	Y	T	D	M	V	H	S	T	-	-	H	G	F
F	I	E	N	T	T	D	A	K	E	Y	T	D	M	V	H	S	T	-	-	H	G	F
F	I	E	N	T	T	D	A	K	E	Y	T	D	M	V	H	S	T	-	-	H	G	F
F	I	E	N	T	T	D	A	K	E	Y	T	D	M	V	H	S	T	-	-	H	G	F
F	I	D	N	T	T	D	A	K	E	Y	T	D	M	V	H	S	T	-	-	H	G	F
F	I	D	N	T	T	D	A	K	E	Y	T	D	M	V	H	S	T	-	-	H	G	F
F	I	D	N	T	T	D	A	K	E	Y	T	D	M	V	H	S	T	-	-	H	G	F
F	I	E	N	T	M	N	A	K	E	Y	T	N	M	V	H	S	T	-	-	H	G	F
F	I	E	N	T	M	N	A	K	E	Y	T	N	M	V	H	S	T	-	-	H	G	F
F	I	E	N	T	M	N	A	K	E	Y	T	N	M	V	H	S	T	-	-	H	G	F
F	I	E	N	T	T	D	A	K	E	Y	T	D	M	V	H	S	T	-	-	H	G	F
F	I	E	N	T	T	D	A	K	E	Y	T	D	M	V	H	S	T	-	-	H	G	F
F	I	E	N	T	T	D	A	K	E	Y	T	D	M	V	H	S	T	-	-	H	G	F
F	I	E	N	T	T	D	A	K	E	Y	T	D	M	V	H	S	T	-	-	H	G	F
F	I	E	N	T	T	D	A	K	E	Y	T	D	M	V	H	S	T	-	-	H	G	F
F	I	E	N	T	T	D	A	K	E	Y	T	D	M	V	H	S	T	-	-	H	G	F
F	I	E	N	T	T	D	A	K	E	Y	T	D	M	V	H	S	T	-	-	H	G	F
F	I	E	N	T	T	D	A	K	E	Y	T	D	M	V	H	S	T	-	-	H	G	F
F	I	E	N	T	T	D	A	K	E	Y	T	D	M	V	H	S	T	-	-	H	G	F
F	I	E	N	T	T	D	A	K	E	Y	T	D	M	V	H	S	T	-	-	H	G	F
F	I	E	N	T	T	D	A	K	E	Y	T	D	M	V	H	S	T	-	-	H	G	F
F	I	E	N	T	T	D	A	K	E	Y	T	D	M	V	H	S	T	-	-	H	G	F
F	I	E	N	T	T	D	A	K	E	Y	T	D	M	V	H	S	T	-	-	H	G	F
F	I	E	N	T	T	D	A	K	E	Y	T	D	M	V	H	S	T	-	-	H	G	F
F	I	E	N	T	T	D	A	K	E	Y	T	D	M	V	H	S	T	-	-	H	G	F
F	I	E	N	T	T	D	A	K	E	Y	T	D	M	V	H	S	T	-	-	H	G	F
F	I	E	N	T	T	D	A	K	E	Y	T	D	M	V	H	S	T	-	-	H	G	F
F	I	E	N	T	T	D	A	K	E	Y	T	D	M	V	H	S	T	-	-	H	G	F
F	I	E	N	T	T	D	A	K	E	Y	T	D	M	V	H	S	T	-	-	H	G	F
F	I	E	N	T	T	D	A	K	E	Y	T	D	M	V	H	S	T	-	-	H	G	F
F	I	E	N	T	T	D	A	K	E	Y	T	D	M	V	H	S	T	-	-	H	G	F
F	I	E	N	T	T	D	A	K	E	Y	T	D	M	V	H	S	T	-	-	H	G	F
F	I	E	N	T	T	D	A	K	E	Y	T	D	M	V	H	S	T	-	-	H	G	F
F	I	E	N	T	T	D	A	K	E	Y	T	D	M	V	H	S	T	-	-	H	G	F
F	I	E	N	T	T	D	A	K	E	Y	T	D	M	V	H	S	T	-	-	H	G	F
F	I	E	N	T	T	D	A	K	E	Y	T	D	M	V	H	S	T	-	-	H	G	F
F	I	E	N	T	T	D	A	K	E	Y	T	D	M	V	H	S	T	-	-	H	G	F
F	I	E	N	T	T	D	A	K	E	Y	T	D	M	V	H	S	T	-	-	H	G	F
F	I	E	N	T	T	D	A	K	E	Y	T	D	M	V	H	S	T	-	-	H	G	F
F	I	E	N	T	T	D	A	K	E	Y	T	D	M	V	H	S	T	-	-	H	G	F
F	I	E	N	T	T	D	A	K	E	Y	T	D	M	V	H	S	T	-	-	H	G	F
F	I	E	N	T	T	D	A	K	E	Y	T	D	M	V	H	S	T	-	-	H	G	F
F	I	E	N	T	T	D	A	K	E	Y	T	D	M	V	H	S	T	-	-	H	G	F
F	I	E	N	T	T	D	A	K	E	Y	T	D	M	V	H	S	T	-	-	H	G	F
F	I	E	N	T	T	D	A	K	E	Y	T	D	M	V	H	S	T	-	-	H	G	F
F	I	E	N	T	T	D	A	K	E	Y	T	D	M	V	H	S	T	-	-	H	G	F
F	I	E	N	T	T	D	A	K	E	Y	T	D	M	V	H	S	T	-	-	H	G	F
F	I	E	N	T	T	D	A	K	E	Y	T	D	M	V	H	S	T	-	-	H	G	F
F	I	E	N	T	T	D	A	K	E	Y	T	D	M	V	H	S	T	-	-	H	G	F
F	I	E	N	T	T	D	A	K	E	Y	T	D	M	V	H	S	T	-	-	H	G	F
F	I	E	N	T	T	D	A	K	E	Y	T	D	M	V	H	S	T	-	-	H	G	F

F	I	E	N	T	T	D	A	K	E	Y	T	D	M	V	H	S	T	-	-	H	G	F
F	I	E	N	T	T	D	A	K	E	Y	T	D	M	V	H	S	T	-	-	H	G	F
F	I	E	N	T	T	D	A	K	E	Y	T	D	M	V	H	S	T	-	-	H	G	F
F	I	E	N	T	T	D	A	K	E	Y	T	D	M	V	H	S	T	-	-	H	G	F
F	I	E	N	T	T	D	A	K	E	Y	T	D	M	V	H	S	T	-	-	H	G	F
F	I	E	N	T	T	D	A	K	E	Y	T	D	M	V	H	S	T	-	-	H	G	F
F	I	E	N	T	T	D	A	K	E	Y	T	D	M	V	H	S	T	-	-	H	G	F
F	I	E	N	T	T	D	A	K	E	Y	T	D	M	V	H	S	T	-	-	H	G	F
F	I	E	N	T	T	D	A	K	E	Y	T	D	M	V	H	S	T	-	-	H	G	F
F	I	E	N	T	T	D	A	K	E	Y	T	D	M	V	H	S	T	-	-	H	G	F
F	I	E	N	T	T	D	A	K	E	Y	T	D	M	V	H	S	T	-	-	H	G	F
F	I	E	N	T	T	D	A	K	E	Y	T	D	M	V	H	S	T	-	-	H	G	F
F	I	E	N	T	T	D	A	K	E	Y	T	D	M	V	H	S	T	-	-	H	G	F
F	I	E	N	T	T	D	A	K	E	Y	T	D	M	V	H	S	T	-	-	H	G	F
F	I	E	N	T	T	D	A	K	E	Y	T	D	M	V	H	S	T	-	-	H	G	F
F	I	E	N	T	T	D	A	K	E	Y	T	D	M	V	H	S	T	-	-	H	G	F
F	I	E	N	T	T	D	A	K	E	Y	T	D	M	V	H	S	T	-	-	H	G	F
F	I	E	N	T	T	D	A	K	E	Y	T	D	M	V	H	S	T	-	-	H	G	F
F	I	E	N	T	T	D	A	K	E	Y	T	D	M	V	H	S	T	-	-	H	G	F
F	I	E	N	T	T	D	A	K	E	Y	T	D	M	V	H	S	T	-	-	H	G	F
F	I	E	N	T	T	D	A	K	E	Y	T	D	M	V	H	S	T	-	-	H	G	F
F	I	E	N	T	T	D	A	K	E	Y	T	D	M	V	H	S	T	-	-	H	G	F
F	I	E	N	T	T	D	A	K	E	Y	T	D	M	V	H	S	T	-	-	H	G	F
F	I	E	N	T	T	D	A	K	E	Y	T	D	M	V	H	S	T	-	-	H	G	F
F	I	E	N	T	T	D	A	K	E	Y	T	D	M	V	H	S	T	-	-	H	G	F
F	I	E	N	T	T	D	A	K	E	Y	T	D	M	V	H	S	T	-	-	H	G	F
F	I	E	N	T	T	D	A	K	E	Y	T	D	M	V	H	S	T	-	-	H	G	F
F	I	E	N	T	T	D	A	K	E	Y	T	D	M	V	H	S	T	-	-	H	G	F
F	I	E	N	T	T	D	A	K	E	Y	T	D	M	V	H	S	T	-	-	H	G	F
F	I	E	N	T	T	D	A	K	E	Y	T	D	M	V	H	S	T	-	-	H	G	F
F	I	E	N	T	T	D	A	K	E	Y	T	D	M	V	H	S	T	-	-	H	G	F
F	I	E	N	T	T	D	A	K	E	Y	T	D	M	V	H	S	T	-	-	H	G	F
F	I	E	N	T	T	D	A	K	E	Y	T	D	M	V	H	S	T	-	-	H	G	F
F	I	E	N	T	T	D	A	K	E	Y	T	D	M	V	H	S	T	-	-	H	G	F
F	I	E	N	T	T	D	A	K	E	Y	T	D	M	V	H	S	T	-	-	H	G	F
F	I	E	N	T	T	D	A	K	E	Y	T	D	M	V	H	S	T	-	-	H	G	F
F	I	E	N	T	T	D	A	K	E	Y	T	D	M	V	H	S	T	-	-	H	G	F
F	I	E	N	T	T	D	A	K	E	Y	T	D	M	V	H	S	T	-	-	H	G	F
F	I	E	N	T	T	D	A	K	E	Y	T	D	M	V	H	S	T	-	-	H	G	F
F	I	E	N	T	T	D	A	K	E	Y	T	D	M	V	H	S	T	-	-	H	G	F
F	I	E	N	T	T	D	A	K	E	Y	T	D	M	V	H	S	T	-	-	H	G	F
F	I	E	N	T	T	D	A	K	E	Y	T	D	M	V	H	S	T	-	-	H	G	F
F	I	E	N	T	T	D	A	K	E	Y	T	D	M	V	H	S	T	-	-	H	G	F
F	I	E	N	T	T	D	A	K	E	Y	T	D	M	V	H	S	T	-	-	H	G	F
F	I	E	N	T	T	D	A	K	E	Y	T	D	M	V	H	S	T	-	-	H	G	F
F	I	E	N	T	T	D	A	K	E	Y	T	D	M	V	H	S	T	-	-	H	G	F
F	I	E	N	T	T	D	A	K	E	Y	T	D	M	V	H	S	T	-	-	H	G	F
F	I	E	N	T	T	D	A	K	E	Y	T	D	M	V	H	S	T	-	-	H	G	F
F	I	E	N	T	T	D	A	K	E	Y	T	D	M	V	H	S	T	-	-	H	G	F
F	I	E	N	T	T	D	A	K	E	Y	T	D	M	V	H	S	T	-	-	H	G	F
F	I	E	N	T	T	D	A	K	E	Y	T	D	M	V	H	S	T	-	-	H	G	F
F	I	E	N	T	T	D	A	K	E	Y	T	D	M	V	H	S	T	-	-	H	G	F
F	I	E	N	T	T	D	A	K	E	Y	T	D	M	V	H	S	T	-	-	H	G	F
F	I	E	N	T	T	D	A	K	E	Y	T	D	M	V	H	S	T	-	-	H	G	F
F	I	E	N	T	T	D	A	K	E	Y	T	D	M	V	H	S	T	-	-	H	G	F
F	I	E	N	T	T	D	A	K	E	Y	T	D	M	V	H	S	T	-	-	H	G	F
F	I	E	N	T	T	D	A	K	E	Y	T	D	M	V	H	S	T	-	-	H	G	F
F	I	E	N	T	T	D	A	K	E	Y	T	D	M	V	H	S	T	-	-	H	G	F
F	I	E	N	T	T	D	A	K	E	Y	T	D	M	V	H	S	T	-	-	H	G	F
F	I	E	N	T	T	D	A	K	E	Y	T	D	M	V	H	S	T	-	-	H	G	F
F	I	E	N	T	T	D	A	K	E	Y	T	D	M	V	H	S	T	-	-	H	G	F
F	I	E	N	T	T	D	A	K	E	Y	T	D	M	V	H	S	T	-	-	H	G	F
F	I	E	N	T	T	D	A	K	E	Y	T	D	M	V	H	S	T	-	-	H	G	F
F	I	E	N	T	T	D	A	K	E	Y	T	D	M	V	H	S	T	-	-	H	G	F
F	I	E	N	T	T	D	A	K	E	Y	T	D	M	V	H	S	T	-	-	H	G	F
F	I	E	N	T	T	D	A	K	E	Y	T	D	M	V	H	S	T	-	-	H	G	F
F	I	E	N	T	T	D	A	K	E	Y	T	D	M	V	H	S	T	-	-	H	G	F

F	I	E	N	T	T	D	A	K	E	Y	T	D	M	V	H	S	T	-	-	H	G	F
F	I	E	N	T	T	D	A	K	E	Y	T	D	M	V	H	S	T	-	-	H	G	F
F	I	E	N	T	T	D	A	K	E	Y	T	D	M	V	H	S	T	-	-	H	G	F
F	I	E	N	T	T	D	A	K	E	Y	T	D	M	V	H	S	T	-	-	H	G	F
F	I	D	N	T	T	D	A	K	E	Y	T	D	M	V	H	S	T	-	-	H	G	F
F	I	D	N	T	T	D	A	K	E	Y	T	D	M	V	H	S	T	-	-	H	G	F
F	I	D	N	T	T	D	A	K	E	Y	T	D	M	V	H	S	T	-	-	H	G	F
F	I	D	N	T	T	D	A	K	E	Y	T	D	M	V	H	S	T	-	-	H	G	F
F	I	D	N	T	T	D	A	K	E	Y	T	D	M	V	H	S	T	-	-	H	G	F
F	I	D	N	T	T	D	A	K	E	Y	T	D	M	V	H	S	T	-	-	H	G	F
F	I	D	N	T	T	D	A	K	E	Y	T	D	M	V	H	S	T	-	-	H	G	F
F	I	D	N	T	T	D	A	K	E	Y	T	D	M	V	H	S	T	-	-	H	G	F
F	I	D	N	T	T	D	A	K	E	Y	T	D	M	V	H	S	T	-	-	H	G	F
F	I	D	N	T	T	D	A	K	E	Y	T	D	M	V	H	S	T	-	-	H	G	F
F	I	D	N	T	T	D	A	K	E	Y	T	D	M	V	H	S	T	-	-	H	G	F
F	I	D	N	T	T	D	A	K	E	Y	T	D	M	V	H	S	T	-	-	H	G	F
F	I	D	N	T	T	D	A	K	E	Y	T	D	M	V	H	S	T	-	-	H	G	F
F	I	D	N	T	T	D	A	K	E	Y	T	D	M	V	H	S	T	-	-	H	G	F
F	I	D	N	T	T	D	A	K	E	Y	T	D	M	V	H	S	T	-	-	H	G	F
F	I	D	N	T	T	D	A	K	E	Y	T	D	M	V	H	S	T	-	-	H	G	F
F	I	D	N	T	T	D	A	K	E	Y	T	D	M	V	H	S	T	-	-	H	G	F
F	I	D	N	T	T	D	A	K	E	Y	T	D	M	V	H	S	T	-	-	H	G	F
F	I	D	N	T	T	D	A	K	E	Y	T	D	M	V	H	S	T	-	-	H	G	F
F	I	D	N	T	T	D	A	K	E	Y	T	D	M	V	H	S	T	-	-	H	G	F
F	I	D	N	T	T	D	A	K	E	Y	T	D	M	V	H	S	T	-	-	H	G	F
F	I	D	N	T	T	D	A	K	E	Y	T	D	M	V	H	S	T	-	-	H	G	F
F	I	D	N	T	T	D	A	K	E	Y	T	D	M	V	H	S	T	-	-	H	G	F
F	I	D	N	T	T	D	A	K	E	Y	T	D	M	V	H	S	T	-	-	H	G	F
F	I	D	N	T	T	D	A	K	E	Y	T	D	M	V	H	S	T	-	-	H	G	F
F	I	D	N	T	T	D	A	K	E	Y	T	D	M	V	H	S	T	-	-	H	G	F
F	I	D	N	T	T	D	A	K	E	Y	T	D	M	V	H	S	T	-	-	H	G	F
F	I	D	N	T	T	D	A	K	E	Y	T	D	M	V	H	S	T	-	-	H	G	F
F	I	D	N	T	T	D	A	K	E	Y	T	D	M	V	H	S	T	-	-	H	G	F
F	I	D	N	T	T	D	A	K	E	Y	T	D	M	V	H	S	T	-	-	H	G	F
F	I	D	N	T	T	D	A	K	E	Y	T	D	M	V	H	S	T	-	-	H	G	F
F	I	D	N	T	T	D	A	K	E	Y	T	D	M	V	H	S	T	-	-	H	G	F
F	I	D	N	T	T	D	A	K	E	Y	T	D	M	V	H	S	T	-	-	H	G	F
F	I	D	N	T	T	D	A	K	E	Y	T	D	M	V	H	S	T	-	-	H	G	F
F	I	D	N	T	T	D	A	K	E	Y	T	D	M	V	H	S	T	-	-	H	G	F
F	I	D	N	T	T	D	A	K	E	Y	T	D	M	V	H	S	T	-	-	H	G	F
F	I	D	N	T	T	D	A	K	E	Y	T	D	M	V	H	S	T	-	-	H	G	F
F	I	D	N	T	T	D	A	K	E	Y	T	D	M	V	H	S	T	-	-	H	G	F
F	I	D	N	T	T	D	A	K	E	Y	T	D	M	V	H	S	T	-	-	H	G	F
F	I	D	N	T	T	D	A	K	E	Y	T	D	M	V	H	S	T	-	-	H	G	F
F	I	D	N	T	T	D	A	K	E	Y	T	D	M	V	H	S	T	-	-	H	G	F
F	I	D	N	T	T	D	A	K	E	Y	T	D	M	V	H	S	T	-	-	H	G	F
F	I	D	N	T	T	D	A	K	E	Y	T	D	M	V	H	S	T	-	-	H	G	F
F	I	D	N	T	T	D	A	K	E	Y	T	D	M	V	H	S	T	-	-	H	G	F
F	I	D	N	T	T	D	A	K	E	Y	T	D	M	V	H	S	T	-	-	H	G	F
F	I	D	N	T	T	D	A	K	E	Y	T	D	M	V	H	S	T	-	-	H	G	F
F	I	D	N	T	T	D	A	K	E	Y	T	D	M	V	H	S	T	-	-	H	G	F
F	I	D	N	T	T	D	A	K	E	Y	T	D	M	V	H	S	T	-	-	H	G	F
F	I	D	N	T	T	D	A	K	E	Y	T	D	M	V	H	S	T	-	-	H	G	F
F	I	D	N	T	T	D	A	K	E	Y	T	D	M	V	H	S	T	-	-	H	G	F
F	I	D	N	T	T	D	A	K	E	Y	T	D	M	V	H	S	T	-	-	H	G	F
F	I	D	N	T	T	D	A	K	E	Y	T	D	M	V	H	S	T	-	-	H	G	F
F	I	D	N	T	T	D	A	K	E	Y	T	D	M	V	H	S	T	-	-	H	G	F
F	I	D	N	T	T	D	A	K	E	Y	T	D	M	V	H	S	T	-	-	H	G	F
F	I	D	N	T	T	D	A	K	E	Y	T	D	M	V	H	S	T	-	-	H	G	F
F	I	D	N	T	T	D	A	K	E	Y	T	D	M	V	H	S	T	-	-	H	G	F
F	I	D	N	T	T	D	A	K	E	Y	T	D	M	V	H	S	T	-	-	H	G	F
F	I	D	N	T	T	D	A	K	E	Y	T	D	M	V	H	S	T	-	-	H	G	F
F	I	D	N	T	T	D	A	K	E	Y	T	D	M	V	H	S	T	-	-	H	G	F
F	I	D	N	T	T	D	A	K	E	Y	T	D	M	V	H	S	T	-	-	H	G	F
F	I	D	N	T	T	D	A	K	E	Y	T	D	M	V	H	S	T	-	-	H	G	F
F	I	D	N	T	T	D	A	K	E	Y	T	D	M	V	H	S	T	-	-	H	G	F
F	I	D	N	T	T	D	A	K	E	Y	T	D	M	V	H	S	T	-	-	H	G	F

F	I	D	N	T	T	D	A	K	E	Y	T	D	M	V	H	S	T	-	-	H	G	F
F	I	D	N	T	T	D	A	K	E	Y	T	D	M	V	H	S	T	-	-	H	G	F
F	I	D	N	T	T	D	A	K	E	Y	T	D	M	V	H	S	T	-	-	H	G	F
F	I	D	N	T	T	D	A	K	E	Y	T	D	M	V	H	S	T	-	-	H	G	F
F	I	D	N	T	T	D	A	K	E	Y	T	D	M	V	H	S	T	-	-	H	G	F
F	I	D	N	T	T	D	A	K	E	Y	T	D	M	V	H	S	T	-	-	H	G	F
F	I	D	N	T	T	D	A	K	E	Y	T	D	M	V	H	S	T	-	-	H	G	F
F	I	D	N	T	T	D	A	K	E	Y	T	D	M	V	H	S	T	-	-	H	G	F
F	I	D	N	T	T	D	A	K	E	Y	T	D	M	V	H	S	T	-	-	H	G	F
F	I	D	N	T	T	D	A	K	E	Y	T	D	M	V	H	S	T	-	-	H	G	F
F	I	D	N	T	T	D	A	K	E	Y	T	D	M	V	H	S	T	-	-	H	G	F
F	I	E	H	T	T	N	A	K	E	Y	T	D	M	V	H	S	T	-	-	H	G	F
F	I	E	H	T	T	N	A	K	E	Y	T	D	M	V	H	S	T	-	-	H	G	F
F	I	E	H	T	T	N	A	K	E	Y	T	D	M	V	H	S	T	-	-	H	G	F
F	I	E	H	T	T	N	A	K	E	Y	T	D	M	V	H	S	T	-	-	H	G	F
F	I	E	H	T	T	N	A	K	E	Y	T	D	M	V	H	S	T	-	-	H	G	F
F	I	E	H	T	T	N	A	K	E	Y	T	D	M	V	H	S	T	-	-	H	G	F
F	I	E	H	T	T	N	A	K	E	Y	T	D	M	V	H	S	T	-	-	H	G	F
F	I	E	H	T	T	N	A	K	E	Y	T	D	M	V	H	S	T	-	-	H	G	F
F	I	E	H	T	T	N	A	K	E	Y	T	D	M	V	H	S	T	-	-	H	G	F
L	L	E	N	I	T	N	S	R	E	H	I	D	L	V	Y	T	A	L	A	N	K	H
L	L	E	N	I	T	N	S	R	E	H	I	D	L	V	Y	T	A	L	A	N	K	H
L	L	E	N	I	T	N	S	R	E	H	I	D	L	V	Y	T	A	L	A	N	K	H
L	L	E	N	I	T	N	S	R	E	H	I	D	L	V	Y	T	A	L	A	N	K	H
L	L	E	N	I	T	N	S	R	E	H	I	D	L	V	Y	T	A	L	A	N	K	H
L	L	E	N	I	T	N	S	R	E	H	I	D	L	V	Y	T	A	L	A	N	K	H
L	L	E	N	I	T	N	S	R	E	H	I	D	L	V	Y	T	A	L	A	N	K	H
L	L	E	N	I	T	N	S	R	E	H	I	D	L	V	Y	T	A	L	A	N	K	H
F	I	E	N	T	T	D	A	K	E	Y	T	D	L	V	Y	T	A	L	A	N	K	H
L	L	E	N	I	T	N	S	R	E	H	I	D	L	V	Y	T	A	L	A	N	K	H
L	L	E	N	I	T	N	S	R	E	H	I	D	L	V	Y	T	A	L	A	N	K	H
L	L	E	N	I	T	N	S	R	E	H	I	D	L	V	Y	T	A	L	A	N	K	H
L	L	E	N	I	T	N	S	R	E	H	I	D	L	V	Y	T	A	L	A	N	K	H
L	L	E	N	I	T	N	S	R	E	H	I	D	L	V	Y	T	A	L	A	N	K	H
L	L	E	N	I	T	N	S	R	E	H	I	D	L	V	Y	T	A	L	A	N	K	H
L	L	E	N	I	T	N	S	R	E	H	I	D	L	V	Y	T	A	L	A	N	K	H
L	L	E	N	I	T	N	S	R	E	H	I	D	L	V	Y	T	A	L	A	N	K	H
L	L	E	N	I	T	N	S	R	E	H	I	D	L	V	Y	T	A	L	A	N	K	H
L	L	E	N	I	T	N	S	R	E	H	I	D	L	V	Y	T	A	L	A	N	K	H
L	L	E	N	I	T	N	S	R	E	H	I	D	L	V	Y	T	A	L	A	N	K	H
L	L	E	N	I	T	N	S	R	E	H	I	D	L	V	Y	T	A	L	A	N	K	H
L	L	E	N	I	T	N	S	R	E	H	I	D	L	V	Y	T	A	L	A	N	K	H
L	L	E	N	I	T	N	S	R	E	H	I	D	L	V	Y	T	A	L	A	N	K	H
L	L	E	N	I	T	N	S	R	E	H	I	D	L	V	Y	T	A	L	A	N	K	H
L	L	E	N	I	T	N	S	R	E	H	I	D	L	V	Y	T	A	L	A	N	K	H
L	L	E	N	I	T	N	S	R	E	H	I	D	L	V	Y	T	A	L	A	N	K	H
L	L	E	N	I	T	N	S	R	E	H	I	D	L	V	Y	T	A	L	A	N	K	H
L	L	E	N	I	T	N	S	R	E	H	I	D	L	V	Y	T	A	L	A	N	K	H
L	L	E	N	I	T	N	S	R	E	H	I	D	L	V	Y	T	A	L	A	N	K	H
L	L	E	N	I	T	N	S	R	E	H	I	D	L	V	Y	T	A	L	A	N	K	H
L	L	E	N	I	T	N	S	R	E	H	I	D	L	V	Y	T	A	L	A	N	K	H
L	L	E	N	I	T	N	S	R	E	H	I	D	L	V	Y	T	A	L	A	N	K	H
L	L	E	N	I	T	N	S	R	E	H	I	D	L	V	Y	T	A	L	A	N	K	H
L	L	E	N	I	T	N	S	R	E	H	I	D	L	V	Y	T	A	L	A	N	K	H
L	L	E	N	I	T	N	S	R	E	H	I	D	L	V	Y	T	A	L	A	N	K	H
L	L	E	N	I	T	N	S	R	E	H	I	D	L	V	Y	T	A	L	A	N	K	H
L	L	E	N	I	T	N	S	R	E	H	I	D	L	V	Y	T	A	L	A	N	K	H
F	I	E	N	T	T	D	A	K	E	Y	T	D	L	I	Y	T	A	H	T	N	G	-
F	I	E	N	T	T	D	A	K	E	Y	T	D	L	I	Y	T	A	H	T	N	G	-
F	I	E	N	T	T	D	A	K	E	Y	T	D	L	I	Y	T	A	H	T	N	G	-
F	I	E	N	T	T	D	A	K	E	Y	T	D	L	I	Y	T	A	H	T	N	G	-
F	I	E	N	T	T	D	A	K	E	Y	T	D	L	I	Y	T	A	H	T	N	G	-
F	I	E	N	T	T	D	A	K	E	Y	T	D	L	I	Y	T	A	H	T	N	G	-
F	I	E	N	T	T	D	A	K	E	Y	T	D	L	I	Y	T	A	H	T	N	G	-
F	I	E	N	T	T	D	A	K	E	Y	T	D	L	I	Y	T	A	H	T	N	G	-
F	I	E	N	T	T	D	A	K	E	Y	T	D	L	I	Y	T	A	H	T	N	G	-
F	I	E	N	T	T	D	A	K	E	Y	T	D	L	I	Y	T	A	H	T	N	G	-

F	I	E	N	T	T	D	A	K	E	Y	T	D	L	I	Y	T	A	H	T	N	G	-
F	I	E	N	T	T	D	A	K	E	Y	T	D	L	I	Y	T	A	H	T	N	G	-
F	I	E	N	T	T	D	A	K	E	Y	T	D	L	I	Y	T	A	H	T	N	G	-
F	I	E	N	T	T	D	A	K	E	Y	T	D	L	I	Y	T	A	H	T	N	G	-
F	I	E	N	T	T	D	A	K	E	Y	T	D	L	I	Y	T	A	H	T	N	G	-
F	I	E	N	T	T	D	A	K	E	Y	T	D	L	I	Y	T	A	H	T	N	G	-
F	I	E	N	T	T	D	A	K	E	Y	T	D	L	I	Y	T	A	H	T	N	G	-
F	I	E	N	T	T	D	A	K	E	Y	T	D	L	I	Y	T	A	H	T	N	G	-
F	I	E	N	T	T	D	A	K	E	Y	T	D	L	I	Y	T	A	H	T	N	G	-
F	I	E	N	T	T	D	A	K	E	Y	T	D	L	I	Y	T	A	H	T	N	G	-
F	I	E	N	T	T	D	A	K	E	Y	T	D	L	I	Y	T	A	H	T	N	G	-
F	I	E	N	T	T	D	A	K	E	Y	T	D	L	I	Y	T	A	H	T	N	G	-
F	I	E	N	T	T	D	A	K	E	Y	T	D	L	I	Y	T	A	H	T	N	G	-
F	I	E	N	T	T	D	A	K	E	Y	T	D	L	I	Y	T	A	H	T	N	G	-
F	I	E	N	T	T	D	A	K	E	Y	T	D	L	I	Y	T	A	H	T	N	G	-
F	I	E	N	T	T	D	A	K	E	Y	T	D	L	I	Y	T	A	H	T	N	G	-
F	I	E	N	T	T	D	A	K	E	Y	T	D	L	I	Y	T	A	H	T	N	G	-
F	I	E	N	T	T	D	A	K	E	Y	T	D	L	I	Y	T	A	H	T	N	G	-
F	I	E	N	T	T	D	A	K	E	Y	T	D	L	I	Y	T	A	H	T	N	G	-
F	I	E	N	T	T	D	A	K	E	Y	T	D	L	I	Y	T	A	H	T	N	G	-
F	I	E	N	T	T	D	A	K	E	Y	T	D	L	I	Y	T	A	H	T	N	G	-
F	I	E	N	T	T	D	A	K	E	Y	T	D	L	I	Y	T	A	H	T	N	G	-
F	I	E	N	T	T	D	A	K	E	Y	T	D	L	I	Y	T	A	H	T	N	G	-
F	I	E	N	T	T	D	A	K	E	Y	T	D	L	I	Y	T	A	H	T	N	G	-
F	I	E	H	T	T	N	A	K	E	Y	T	D	M	V	H	S	T	-	-	H	G	F
F	I	E	H	T	T	N	A	K	E	Y	T	D	M	V	H	S	T	-	-	H	G	F
F	I	E	H	T	T	N	A	K	E	Y	T	D	M	V	H	S	T	-	-	H	G	F
F	I	E	H	T	T	N	A	K	E	Y	T	D	M	V	H	S	T	-	-	H	G	F
F	I	E	H	T	T	N	A	K	E	Y	T	D	M	V	H	S	T	-	-	H	G	F
F	I	E	H	T	T	N	A	K	E	Y	T	D	M	V	H	S	T	-	-	H	G	F
F	I	E	H	T	T	N	A	K	E	Y	T	D	M	V	H	S	T	-	-	H	G	F
F	I	E	H	T	T	N	A	K	E	Y	T	D	M	V	H	S	T	-	-	H	G	F
F	I	E	H	T	T	N	A	K	E	Y	T	D	M	V	H	S	T	-	-	H	G	F
F	I	E	H	T	T	N	A	K	E	Y	T	D	M	V	H	S	T	-	-	H	G	F
F	I	E	H	T	T	N	A	K	E	Y	T	D	M	V	H	S	T	-	-	H	G	F
F	I	E	H	T	T	N	A	K	E	Y	T	D	M	V	H	S	T	-	-	H	G	F
F	I	E	H	T	T	N	A	K	E	Y	T	D	M	V	H	S	T	-	-	H	G	F
F	I	E	H	T	T	N	A	K	E	Y	T	D	M	V	H	S	T	-	-	H	G	F
F	I	E	H	T	T	N	A	K	E	Y	T	D	M	V	H	S	T	-	-	H	G	F
F	I	E	H	T	T	N	A	K	E	Y	T	D	M	V	H	S	T	-	-	H	G	F
F	I	E	H	T	T	N	A	K	E	Y	T	D	M	V	H	S	T	-	-	H	G	F
F	I	E	H	T	T	N	A	K	E	Y	T	D	M	V	H	S	T	-	-	H	G	F
F	I	E	H	T	T	N	A	K	E	Y	T	D	M	V	H	S	T	-	-	H	G	F
F	I	E	H	T	T	N	A	K	E	Y	T	D	M	V	H	S	T	-	-	H	G	F
F	I	E	H	T	T	N	A	K	E	Y	T	D	M	V	H	S	T	-	-	H	G	F
F	I	E	H	T	T	N	A	K	E	Y	T	D	M	V	H	S	T	-	-	H	G	F
F	I	E	H	T	T	N	A	K	E	Y	T	D	M	V	H	S	T	-	-	H	G	F
F	I	E	H	T	T	N	A	K	E	Y	T	D	M	V	H	S	T	-	-	H	G	F
F	I	E	H	T	T	N	A	K	E	Y	T	D	M	V	H	S	T	-	-	H	G	F
F	I	E	N	T	M	N	A	K	E	Y	T	N	M	V	H	S	T	-	-	H	G	F
F	I	E	N	T	M	N	A	K	E	Y	T	N	M	V	H	S	T	-	-	H	G	F
F	I	E	N	T	M	N	A	K	E	Y	T	N	M	V	H	S	T	-	-	H	G	F
F	I	E	N	T	M	N	A	K	E	Y	T	N	M	V	H	S	T	-	-	H	G	F
F	I	E	N	T	M	N	A	K	E	Y	T	N	M	V	H	S	T	-	-	H	G	F
F	I	E	N	T	M	N	A	K	E	Y	T	N	M	V	H	S	T	-	-	H	G	F
F	I	E	N	T	M	N	A	K	E	Y	T	N	M	V	H	S	T	-	-	H	G	F
F	I	E	N	T	M	N	A	K	E	Y	T	N	M	V	H	S	T	-	-	H	G	F
F	I	E	N	T	M	N	A	K	E	Y	T	N	M	V	H	S	T	-	-	H	G	F
F	I	E	N	T	M	N	A	K	E	Y	T	N	M	V	H	S	T	-	-	H	G	F
F	I	E	N	T	M	N	A	K	E	Y	T	N	M	V	H	S	T	-	-	H	G	F
F	I	E	N	T	M	N	A	K	E	Y	T	N	M	V	H	S	T	-	-	H	G	F
F	I	E	N	T	M	N	A	K	E	Y	T	N	M	V	H	S	T	-	-	H	G	F
F	I	E	N	T	M	N	A	K	E	Y	T	N	M	V	H	S	T	-	-	H	G	F
F	I	E	N	T	M	N	A	K	E	Y	T	N	M	V	H	S	T	-	-	H	G	F
F	I	E	N	T	M	N	A	K	E	Y	T	N	M	V	H	S	T	-	-	H	G	F
F	I	E	N	T	M	N	A	K	E	Y	T	N	M	V	H	S	T	-	-	H	G	F
F	I	E	N	T	M	N	A	K	E	Y	T	N	M	V	H	S	T	-	-	H	G	F

F	I	E	N	T	M	N	A	K	E	Y	T	N	M	V	H	S	T	-	-	H	G	F
F	I	E	N	T	M	N	A	K	E	Y	T	N	M	V	H	S	T	-	-	H	G	F
F	I	E	N	T	M	N	A	K	E	Y	T	N	M	V	H	S	T	-	-	H	G	F
F	I	E	N	T	M	N	A	K	E	Y	T	N	M	V	H	S	T	-	-	H	G	F
F	I	E	N	T	M	N	A	K	E	Y	T	N	M	V	H	S	T	-	-	H	G	F
F	I	E	N	T	M	N	A	K	E	Y	T	N	M	V	H	S	T	-	-	H	G	F
F	I	E	N	T	M	N	A	K	E	Y	T	N	M	V	H	S	T	-	-	H	G	F
F	I	E	N	T	M	N	A	K	E	Y	T	N	M	V	H	S	T	-	-	H	G	F
F	I	E	N	T	M	N	A	K	E	Y	T	N	M	V	H	S	T	-	-	H	G	F
F	I	E	N	T	M	N	A	K	E	Y	T	N	M	V	H	S	T	-	-	H	G	F

wzg																						
114	129	157	162	169	172	199	212	321	328	331	337	338	339	340	342	345	346	347	348	357	360	387
I	Q	T	D	A	K	Y	T	M	V	H	S	T	-	-	G	H	F	P	V	Q	G	I
L	Q	I	N	S	R	H	I	L	V	Y	T	A	L	A	K	H	Y	S	I	E	A	L
I	H	T	D	A	K	Y	T	L	I	Y	T	A	H	T	G	Y	Y	P	V	Q	G	I
L	Q	I	N	S	R	H	I	L	V	Y	T	A	L	A	K	H	Y	S	I	E	A	L
L	Q	I	N	S	R	H	I	L	V	Y	T	A	L	A	K	H	Y	S	I	E	A	L
L	Q	I	N	S	R	H	I	L	V	Y	T	A	L	A	K	H	Y	S	I	E	A	L
L	Q	I	N	S	R	H	I	L	V	Y	T	A	L	A	K	H	Y	S	I	E	A	L
L	Q	I	N	S	R	H	I	L	V	Y	T	A	L	A	K	H	Y	S	I	E	A	L
L	Q	I	N	S	R	H	I	L	V	Y	T	A	L	A	K	H	Y	S	I	E	A	L
L	Q	I	N	S	R	H	I	L	V	Y	T	A	L	A	K	H	Y	S	I	E	A	L
L	Q	I	N	S	R	H	I	L	V	Y	T	A	L	A	K	H	Y	S	I	E	A	L

wzg																						
114	157	162	169	172	199	212	321	331	337	338	340	341	343	345	346	347	350	356	359	386	387	400
I	T	D	A	K	Y	T	M	H	S	T	H	G	F	F	P	V	V	Q	G	I	K	S
L	I	N	S	R	H	I	L	Y	T	A	A	N	K	Y	S	I	V	E	A	L	Q	T
L	I	N	S	R	H	I	L	Y	T	A	A	N	K	Y	S	I	V	E	A	L	Q	T
L	I	N	S	R	H	I	L	Y	T	A	A	N	K	Y	S	I	V	E	A	L	Q	T
L	I	N	S	R	H	I	L	Y	T	A	A	N	K	Y	S	I	V	E	A	L	Q	T
L	I	N	S	R	H	I	L	Y	T	A	A	N	K	Y	S	I	V	E	A	L	Q	T
L	I	N	S	R	H	I	L	Y	T	A	A	N	K	Y	S	I	V	E	A	L	Q	T
L	I	N	S	R	H	I	L	Y	T	A	A	N	K	Y	S	I	V	E	A	L	Q	T
L	I	N	S	R	H	I	L	Y	T	A	A	N	K	Y	S	I	V	E	A	L	Q	T
L	I	N	S	R	H	I	L	Y	T	A	A	N	K	Y	S	I	V	E	A	L	Q	T
L	I	N	S	R	H	I	L	Y	T	A	A	N	K	Y	S	I	V	E	A	L	Q	T
L	I	N	S	R	H	I	L	Y	T	A	A	N	K	Y	S	I	V	E	A	L	Q	T
L	I	N	S	R	H	I	L	Y	T	A	A	N	K	Y	S	I	V	E	A	L	Q	T
L	I	N	S	R	H	I	L	Y	T	A	A	N	K	Y	S	I	V	E	A	L	Q	T
L	I	N	S	R	H	I	L	Y	T	A	A	N	K	Y	S	I	V	E	A	L	Q	T
L	I	N	S	R	H	I	L	Y	T	A	A	N	K	Y	S	I	V	E	A	L	Q	T
L	I	N	S	R	H	I	L	Y	T	A	A	N	K	Y	S	I	V	E	A	L	Q	T
L	I	N	S	R	H	I	L	Y	T	A	A	N	K	Y	S	I	V	E	A	L	Q	T
L	I	N	S	R	H	I	L	Y	T	A	A	N	K	Y	S	I	V	E	A	L	Q	T
L	I	N	S	R	H	I	L	Y	T	A	A	N	K	Y	S	I	V	E	A	L	Q	T
L	I	N	S	R	H	I	L	Y	T	A	A	N	K	Y	S	I	V	E	A	L	Q	T
L	I	N	S	R	H	I	L	Y	T	A	A	N	K	Y	S	I	V	E	A	L	Q	T
L	I	N	S	R	H	I	L	Y	T	A	A	N	K	Y	S	I	V	E	A	L	Q	T
L	I	N	S	R	H	I	L	Y	T	A	A	N	K	Y	S	I	V	E	A	L	Q	T
L	I	N	S	R	H	I	L	Y	T	A	A	N	K	Y	S	I	V	E	A	L	Q	T
I	T	D	A	K	Y	T	L	Y	T	A	A	N	K	Y	S	I	I	E	A	I	K	S
L	I	N	S	R	H	I	L	Y	T	A	A	N	K	Y	S	I	V	E	A	L	Q	T
L	I	N	S	R	H	I	L	Y	T	A	A	N	K	Y	S	I	V	E	A	L	Q	T
L	I	N	S	R	H	I	L	Y	T	A	A	N	K	Y	S	I	V	E	A	L	Q	T
L	I	N	S	R	H	I	L	Y	T	A	A	N	K	Y	S	I	V	E	A	L	Q	T
L	I	N	S	R	H	I	L	Y	T	A	A	N	K	Y	S	I	V	E	A	L	Q	T

zg					wzh			wzd		wze								
337	338	346	369	432	92	191	194	182	211	45	175	212	223	11	16	27	31	32
S	T	F	A	N	G	N	Y	F	I	I	S	S	R	A	F	A	T	E
T	A	Y	A	D	G	D	H	F	V	L	M	N	K	V	S	T	A	K
T	A	Y	A	D	E	D	Y	F	I	I	S	S	R	A	F	A	T	E
T	A	Y	A	D	E	D	Y	F	I	I	S	S	R	A	F	A	T	E
T	A	Y	A	D	E	D	Y	F	I	I	S	S	R	A	F	A	T	E
T	A	Y	A	D	E	D	Y	F	I	I	S	S	R	A	F	A	T	E
T	A	Y	A	D	E	D	Y	F	I	I	S	S	R	A	F	A	T	E
T	A	Y	A	D	E	D	Y	F	I	I	S	S	R	A	F	A	T	E
T	A	Y	A	D	E	D	Y	F	I	I	S	S	R	A	F	A	T	E
T	A	Y	A	D	E	D	Y	F	I	I	S	S	R	A	F	A	T	E
T	A	Y	A	D	E	D	Y	F	I	I	S	S	R	A	F	A	T	E
T	A	Y	A	D	E	D	Y	F	I	I	S	S	R	A	F	A	T	E
T	A	Y	A	D	E	D	Y	F	I	I	S	S	R	A	F	A	T	E
T	A	Y	A	D	E	D	Y	F	I	I	S	S	R	A	F	A	T	E
T	A	Y	A	D	E	D	Y	F	I	I	S	S	R	A	F	A	T	E
T	A	Y	A	D	E	D	Y	F	I	I	S	S	R	A	F	A	T	E
T	A	Y	A	D	E	D	Y	F	I	I	S	S	R	A	F	A	T	E
T	A	Y	A	D	E	D	Y	F	I	I	S	S	R	A	F	A	T	E
T	A	Y	A	D	E	D	Y	F	I	I	S	S	R	A	F	A	T	E
T	A	Y	A	D	E	D	Y	F	I	I	S	S	R	A	F	A	T	E
T	A	Y	A	D	E	D	Y	F	I	I	S	S	R	A	F	A	T	E
T	A	Y	A	D	E	D	Y	F	I	I	S	S	R	A	F	A	T	E
T	A	Y	A	D	E	D	Y	F	I	I	S	S	R	A	F	A	T	E
T	A	Y	A	D	E	D	Y	F	I	I	S	S	R	A	F	A	T	E
T	A	Y	A	D	E	D	Y	F	I	I	S	S	R	A	F	A	T	E
T	A	Y	A	D	E	D	Y	F	I	I	S	S	R	A	F	A	T	E
T	A	Y	A	D	E	D	Y	F	I	I	S	S	R	A	F	A	T	E
T	A	Y	A	D	E	D	Y	F	I	I	S	S	R	A	F	A	T	E
T	A	Y	A	D	E	D	Y	F	I	I	S	S	R	A	F	A	T	E
T	A	Y	A	D	E	D	Y	L	I	I	S	S	R	A	F	A	T	E
T	A	Y	A	D	E	D	Y	F	I	I	S	S	R	A	F	A	T	E
T	A	Y	A	D	E	D	Y	F	I	I	S	S	R	A	F	A	T	E
T	A	Y	A	D	E	D	Y	F	I	I	S	S	R	A	F	A	T	E
T	A	Y	A	D	E	D	Y	F	I	I	S	S	R	A	F	A	T	E
T	A	Y	A	D	E	D	Y	F	I	I	S	S	R	A	F	A	T	E

wzg																						
129	146	157	162	308	321	328	331	335	337	338	346	369	373	384	387	388	393	395	401	403	404	405
Q	K	T	D	N	M	V	H	E	S	T	F	A	R	V	I	K	T	V	S	L	Q	G
H	K	T	D	N	L	I	Y	E	T	A	Y	A	R	V	I	K	T	V	S	L	Q	G
Q	E	I	N	H	L	V	Y	D	T	S	F	A	H	A	L	Q	S	A	M	I	D	S
Q	K	T	D	N	L	I	Y	E	T	A	Y	V	R	V	I	K	T	V	S	L	Q	G

wzg

114	143	157	162	169	172	199	212	321	331	337	338	340	341	343	345	346	347	356	359	386	387	400
I	N	T	D	A	K	Y	T	M	H	S	T	H	G	F	F	P	V	Q	G	I	K	S
L	N	I	N	S	R	H	I	L	Y	T	A	A	N	K	Y	S	I	E	A	L	Q	T
I	H	T	N	A	K	Y	T	M	H	S	T	H	G	F	F	P	V	Q	G	I	K	S
I	H	T	N	A	K	Y	T	M	H	S	T	H	G	F	F	P	V	Q	G	I	K	S
I	H	T	N	A	K	Y	T	M	H	S	T	H	G	F	F	P	V	Q	G	I	K	S
I	H	T	N	A	K	Y	T	M	H	S	T	H	G	F	F	P	V	Q	G	I	K	S
I	H	T	N	A	K	Y	T	M	H	S	T	H	G	F	F	P	V	Q	G	I	K	S
I	H	T	N	A	K	Y	T	M	H	S	T	H	G	F	F	P	V	Q	G	I	K	S
I	H	T	N	A	K	Y	T	M	H	S	T	H	G	F	F	P	V	Q	G	I	K	S
I	H	T	N	A	K	Y	T	M	H	S	T	H	G	F	F	P	V	Q	G	I	K	S
I	H	T	N	A	K	Y	T	M	H	S	T	H	G	F	F	P	V	Q	G	I	K	S
I	H	T	N	A	K	Y	T	M	H	S	T	H	G	F	F	P	V	Q	G	I	K	S
I	H	T	N	A	K	Y	T	M	H	S	T	H	G	F	F	P	V	Q	G	I	K	S
I	H	T	N	A	K	Y	T	M	H	S	T	H	G	F	F	P	V	Q	G	I	K	S
I	H	T	N	A	K	Y	T	M	H	S	T	H	G	F	F	P	V	Q	G	I	K	S
I	H	T	N	A	K	Y	T	M	H	S	T	H	G	F	F	P	V	Q	G	I	K	S
I	H	T	N	A	K	Y	T	M	H	S	T	H	G	F	F	P	V	Q	G	I	K	S
I	H	T	N	A	K	Y	T	M	H	S	T	H	G	F	F	P	V	Q	G	I	K	S
I	H	T	N	A	K	Y	T	M	H	S	T	H	G	F	F	P	V	Q	G	I	K	S
I	H	T	N	A	K	Y	T	M	H	S	T	H	G	F	F	P	V	Q	G	I	K	S
I	H	T	N	A	K	Y	T	M	H	S	T	H	G	F	F	P	V	Q	G	I	K	S
I	H	T	N	A	K	Y	T	M	H	S	T	H	G	F	F	P	V	Q	G	I	K	S
I	H	T	N	A	K	Y	T	M	H	S	T	H	G	F	F	P	V	Q	G	I	K	S
I	H	T	N	A	K	Y	T	M	H	S	T	H	G	F	F	P	V	Q	G	I	K	S
I	H	T	N	A	K	Y	T	M	H	S	T	H	G	F	F	P	V	Q	G	I	K	S
I	H	T	N	A	K	Y	T	M	H	S	T	H	G	F	F	P	V	Q	G	I	K	S
I	H	T	N	A	K	Y	T	M	H	S	T	H	G	F	F	P	V	Q	G	I	K	S

									wzg													
321	328	331	337	338	346	387	388	395	401	403	404	405	410	416	421	425	432	446	463	464	470	473
M	V	H	S	T	F	I	K	V	S	L	Q	G	L	I	D	T	N	M	I	D	V	A
L	I	Y	T	A	Y	I	K	V	S	L	Q	G	L	I	D	T	D	M	I	D	V	A
M	I	H	S	A	F	L	Q	A	T	I	D	S	I	L	N	A	N	T	I	N	S	T
M	V	H	S	T	F	I	K	V	S	L	Q	G	L	I	D	T	N	M	V	D	V	A
M	V	H	S	T	F	I	K	V	S	L	Q	G	L	I	D	T	N	M	V	D	V	A
M	V	H	S	T	F	I	K	V	S	L	Q	G	L	I	D	T	N	M	V	D	V	A
M	V	H	S	T	F	I	K	V	S	L	Q	G	L	I	D	T	N	M	V	D	V	A
M	V	H	S	T	F	I	K	V	S	L	Q	G	L	I	D	T	N	M	V	D	V	A
M	V	H	S	T	F	I	K	V	S	L	Q	G	L	I	D	T	N	M	V	D	V	A
M	V	H	S	T	F	I	K	V	S	L	Q	G	L	I	D	T	N	M	V	D	V	A
M	V	H	S	T	F	I	K	V	S	L	Q	G	L	I	D	T	N	M	V	D	V	A
M	V	H	S	T	F	I	K	V	S	L	Q	G	L	I	D	T	N	M	V	D	V	A
M	V	H	S	T	F	I	K	V	S	L	Q	G	L	I	D	T	N	M	V	D	V	A
M	V	H	S	T	F	I	K	V	S	L	Q	G	L	I	D	T	N	M	V	D	V	A
M	V	H	S	T	F	I	K	V	S	L	Q	G	L	I	D	T	N	M	V	D	V	A
M	V	H	S	T	F	I	K	V	S	L	Q	G	L	I	D	T	N	M	V	D	V	A
M	V	H	S	T	F	I	K	V	S	L	Q	G	L	I	D	T	N	M	V	D	V	A
M	V	H	S	T	F	I	K	V	S	L	Q	G	L	I	D	T	N	M	V	D	V	A
M	V	H	S	T	F	I	K	V	S	L	Q	G	L	I	D	T	N	M	V	D	V	A
M	V	H	S	T	F	I	K	V	S	L	Q	G	L	I	D	T	N	M	V	D	V	A
M	V	H	S	T	F	I	K	V	S	L	Q	G	L	I	D	T	N	M	V	D	V	A
M	V	H	S	T	F	I	K	V	S	L	Q	G	L	I	D	T	N	M	V	D	V	A
M	V	H	S	T	F	I	K	V	S	L	Q	G	L	I	D	T	N	M	V	D	V	A
M	V	H	S	T	F	I	K	V	S	L	Q	G	L	I	D	T	N	M	V	D	V	A
M	V	H	S	T	F	I	K	V	S	L	Q	G	L	I	D	T	N	M	V	D	V	A
M	V	H	S	T	F	I	K	V	S	L	Q	G	L	I	D	T	N	M	V	D	V	A
M	V	H	S	T	F	I	K	V	S	L	Q	G	L	I	D	T	N	M	V	D	V	A
M	V	H	S	T	F	I	K	V	S	L	Q	G	L	I	D	T	N	M	V	D	V	A
M	V	H	S	T	F	I	K	V	S	L	Q	G	L	I	D	T	N	M	V	D	V	A

M	V	H	S	T	F	I	K	V	S	L	Q	G	L	I	D	T	N	M	V	D	V	A
M	V	H	S	T	F	I	K	V	S	L	Q	G	L	I	D	T	N	M	V	D	V	A

			wzh						wzd										
178	306	463	51	74	123	171	191	194	34	42	46	65	66	122	129	168	211	227	17
E	D	I	P	V	S	K	N	Y	A	F	P	P	G	I	D	A	I	D	A
E	D	V	P	I	S	K	D	Y	A	F	P	P	G	I	D	A	V	D	A
E	D	V	P	I	S	K	D	Y	A	F	P	P	G	I	D	A	V	D	A
E	D	V	P	I	S	K	D	Y	A	F	P	P	G	I	D	A	V	D	A
E	D	V	P	I	S	K	D	Y	A	F	P	P	G	I	D	A	V	D	A
E	D	V	P	I	S	K	D	Y	A	F	P	P	G	I	D	A	V	D	A
E	D	V	P	I	S	K	D	Y	A	F	P	P	G	I	D	A	V	D	A
E	D	V	P	I	S	K	D	Y	A	F	P	P	G	I	D	A	V	D	A
E	D	V	P	I	S	K	D	Y	A	F	P	P	G	I	D	A	V	D	A
E	D	V	P	I	S	K	D	Y	A	F	P	P	G	I	D	A	V	D	A
E	D	V	P	I	S	K	D	Y	A	F	P	P	G	I	D	A	V	D	A
E	D	V	P	I	S	K	D	Y	A	F	P	P	G	I	D	A	V	D	A
E	D	V	P	I	S	K	D	Y	A	F	P	P	G	I	D	A	V	D	A
E	D	V	P	I	S	K	D	Y	A	F	P	P	G	I	D	A	V	D	A
E	D	V	P	I	S	K	D	Y	A	F	P	P	G	I	D	A	V	D	A
E	D	V	P	I	S	K	D	Y	A	F	P	P	G	I	D	A	V	D	A
E	D	V	P	I	S	K	D	Y	A	F	P	P	G	I	D	A	V	D	A
E	D	V	P	I	S	K	D	Y	A	F	P	P	G	I	D	A	V	D	A
E	D	V	P	I	S	K	D	Y	A	F	P	P	G	I	D	A	V	D	A
E	D	V	P	I	S	K	D	Y	A	F	P	P	G	I	D	A	V	D	A
E	D	V	P	I	S	K	D	Y	A	F	P	P	G	I	D	A	V	D	A
E	D	V	P	I	S	K	D	Y	A	F	P	P	G	I	D	A	V	D	A
E	D	V	P	I	S	K	D	Y	A	F	P	P	G	I	D	A	V	D	A
E	D	V	P	I	S	K	D	Y	A	F	P	P	G	I	D	A	V	D	A
E	D	V	P	I	S	K	D	Y	A	F	P	P	G	I	D	A	V	D	A
E	D	V	L	I	S	K	D	Y	A	F	P	P	G	I	D	A	V	D	A
E	D	V	P	I	S	K	D	Y	A	F	P	P	G	I	D	A	V	D	A
E	D	V	P	I	S	K	D	Y	A	F	P	P	G	I	D	A	V	D	A
E	D	V	P	I	S	K	D	Y	A	F	P	L	G	I	N	A	V	D	A
E	D	V	P	I	S	K	D	Y	A	F	P	P	G	I	D	A	V	D	A
E	D	V	P	I	S	K	D	Y	A	V	P	P	G	I	D	A	V	D	A
E	D	V	P	I	S	K	D	Y	A	F	P	P	G	I	D	A	V	D	V
E	D	V	P	I	S	K	D	Y	A	F	P	P	G	I	D	A	V	D	V
E	D	V	P	I	S	K	D	Y	A	F	P	P	G	I	D	A	V	D	V
E	D	V	P	I	S	K	D	Y	A	F	P	P	G	I	D	A	V	D	V
E	D	V	P	I	S	K	D	Y	A	F	P	P	G	I	D	A	V	D	V
E	D	V	P	I	S	K	D	Y	A	F	P	P	G	I	D	A	V	D	V
E	D	V	P	I	S	K	D	Y	A	F	P	P	G	I	D	A	V	D	V
E	D	V	P	I	S	K	D	Y	A	F	P	P	G	I	D	A	V	D	V
E	D	V	P	I	S	K	D	Y	A	F	P	P	G	I	D	A	V	D	V
E	D	V	P	I	S	K	D	Y	A	F	P	P	G	I	D	A	V	D	V
E	D	V	P	I	S	K	D	Y	A	F	P	P	G	I	D	A	V	D	V
E	D	V	P	I	S	K	D	Y	A	F	P	P	G	I	D	A	V	D	V
E	D	V	P	I	S	K	D	Y	A	F	P	P	G	I	D	A	V	D	V
E	D	V	P	I	S	K	D	Y	A	F	P	P	G	I	D	A	V	D	V
E	D	V	P	I	S	K	D	Y	A	F	P	P	G	I	D	A	V	D	V
E	D	V	P	I	S	K	D	Y	A	F	P	P	G	I	D	A	V	D	V
E	D	V	P	I	S	K	D	Y	A	F	P	P	G	I	D	A	V	D	V
E	D	V	P	I	S	K	D	Y	A	F	P	P	G	I	D	A	V	D	V
E	D	V	P	I	S	K	D	Y	A	F	P	P	G	I	D	A	V	D	V
E	D	V	P	I	S	K	D	Y	A	F	P	P	G	I	D	A	V	D	V
E	D	V	P	I	S	K	D	Y	A	F	P	P	G	I	D	A	V	D	V
E	D	V	P	I	S	K	D	Y	A	F	P	P	G	I	D	A	V	D	V
E	D	V	P	I	S	K	D	Y	A	F	P	P	G	I	D	A	V	D	V
E	D	V	P	I	S	K	D	Y	A	F	P	P	G	I	D	A	V	D	V

E	D	V	P	I	S	K	D	Y	A	F	P	P	G	I	D	V	V	D	V
E	D	V	P	I	S	K	D	Y	A	F	P	P	G	I	D	A	V	D	V
E	D	V	P	I	S	K	D	Y	A	F	P	P	G	I	D	A	V	D	V
E	D	V	P	I	S	K	D	Y	A	F	P	P	G	I	D	A	V	D	V
E	D	V	P	I	S	K	D	Y	A	F	P	P	G	I	D	A	V	D	V
E	D	V	P	I	S	K	D	Y	A	F	P	P	G	I	D	A	V	D	V
E	D	V	P	I	S	K	D	Y	A	F	P	P	G	I	D	A	V	D	V
E	D	V	P	I	S	K	D	Y	A	F	P	P	G	I	D	A	V	D	V
E	D	V	P	I	S	K	D	Y	A	F	P	P	G	I	D	A	V	D	V
E	D	V	P	I	S	K	D	Y	A	F	P	P	G	I	D	A	V	D	V
E	D	V	P	I	S	K	D	Y	A	F	P	P	G	I	D	A	V	D	V
E	D	V	P	I	S	K	D	Y	A	F	P	P	G	I	D	A	V	D	V
E	D	V	P	I	S	K	D	Y	A	F	P	P	G	I	D	A	V	D	V
E	D	V	P	I	S	K	D	Y	A	F	P	P	G	I	D	A	V	D	V
E	D	V	P	I	S	K	D	Y	A	F	P	P	G	I	D	A	V	D	V
E	D	V	P	I	S	K	D	Y	A	F	P	P	G	I	D	A	V	D	V
E	D	V	P	I	S	K	D	Y	A	F	P	P	G	I	D	A	V	D	V
E	D	V	P	I	S	K	D	Y	A	F	P	P	G	I	D	A	V	D	V
E	D	V	P	I	S	K	D	Y	A	F	P	P	G	I	D	A	V	D	V
E	D	V	P	I	S	K	D	Y	A	F	P	P	G	I	D	A	V	D	V
E	D	V	P	I	S	K	D	Y	A	F	P	P	G	I	D	A	V	D	V
E	D	V	P	I	S	K	D	Y	A	F	P	P	G	I	D	A	V	D	V
E	D	V	P	I	S	K	D	Y	A	F	P	P	G	I	D	A	V	D	V
E	D	V	P	I	S	K	D	Y	A	F	P	P	G	I	D	A	V	D	V
E	D	V	P	I	S	K	D	Y	A	F	P	P	G	I	D	A	V	D	V
E	D	V	P	I	S	K	D	Y	A	F	P	P	G	I	D	A	V	D	V
E	D	V	P	I	S	K	D	Y	A	F	P	P	G	I	D	A	V	D	V
E	D	V	P	I	S	K	D	Y	A	F	P	P	G	I	D	A	V	D	V
E	D	V	P	I	S	K	D	Y	A	F	P	P	G	I	D	A	V	D	V
E	D	V	P	I	S	K	D	Y	A	F	P	P	G	I	D	A	V	D	V
E	D	V	P	I	S	K	D	Y	A	F	P	P	G	I	D	A	V	D	V
E	D	V	P	I	S	K	D	Y	A	F	P	P	G	I	D	A	V	D	V
E	D	V	P	I	S	K	D	Y	A	F	P	P	G	I	D	A	V	D	V
E	D	V	P	I	S	K	D	Y	A	F	P	P	G	I	D	A	V	D	V
E	D	V	P	I	S	K	D	Y	A	F	P	P	G	I	D	A	V	D	V
E	D	V	P	I	S	K	D	Y	A	F	P	P	G	I	D	A	V	D	V
E	D	V	P	I	S	K	D	Y	A	F	P	P	G	I	D	A	V	D	V
E	D	V	P	I	S	K	D	Y	A	F	P	P	G	I	D	A	V	D	V
E	D	V	P	I	S	K	D	Y	A	F	P	P	G	I	D	A	V	D	V
E	D	V	P	I	S	K	D	Y	A	F	P	P	G	I	D	A	V	D	V
E	D	V	P	I	S	K	D	Y	A	F	P	P	G	I	D	A	V	D	V
E	D	V	P	I	S	K	D	Y	A	F	P	P	G	I	D	A	V	D	V
E	D	V	P	I	S	K	D	Y	A	F	P	P	G	I	D	A	V	D	V
E	D	V	P	I	S	K	D	Y	A	F	P	P	G	I	D	A	V	D	V
E	D	V	P	I	S	K	D	Y	A	F	P	P	G	I	D	A	V	D	V
E	D	V	P	I	S	K	D	Y	A	F	P	P	G	I	D	A	V	D	V
E	D	V	P	I	S	K	D	Y	A	F	P	P	G	I	D	A	V	D	V
E	D	V	P	I	S	K	D	Y	A	F	P	P	G	I	D	A	V	D	V
E	D	V	P	I	S	K	D	Y	A	F	P	P	G	I	D	A	V	D	V
E	D	V	P	I	S	K	D	Y	A	F	P	P	G	I	D	A	V	D	V
E	D	I	P	V	S	K	D	Y	A	F	P	P	G	I	D	A	I	D	A
E	D	I	P	V	S	K	D	Y	A	F	P	P	G	I	D	A	I	D	A
E	D	I	P	V	S	K	D	Y	A	F	P	P	G	I	D	A	I	D	A
E	D	I	P	V	S	K	D	Y	A	F	P	P	G	I	D	A	I	D	A
E	D	I	P	V	S	K	D	Y	A	F	P	P	G	I	D	A	I	D	A
E	D	I	P	V	S	K	D	Y	A	F	P	P	G	I	D	A	I	D	A
E	D	I	P	V	S	K	D	Y	A	F	P	P	G	I	D	A	I	D	A
E	D	I	P	V	S	K	D	Y	A	F	P	P	G	I	D	A	I	D	A
E	D	I	P	V	S	K	D	Y	A	F	P	P	G	I	D	A	I	D	A
E	D	I	P	V	S	K	D	Y	A	F	P	P	G	I	D	A	I	D	A
E	D	I	P	V	S	K	D	Y	A	F	P	P	G	I	D	A	I	D	A
E	D	I	P	V	S	K	D	Y	A	F	P	P	G	I	D	A	I	D	A
E	D	I	P	V	S	K	D	Y	A	F	P	P	G	I	D	A	I	D	A
E	D	I	P	V	S	K	D	Y	A	F	P	P	G	I	D	A	I	D	A
E	D	I	P	V	S	K	D	Y	A	F	P	P	G	I	D	A	I	D	A
E	D	I	P	V	S	K	D	Y	A	F	P	P	G	I	D	A	I	D	A
E	D	I	P	V	S	K	D	Y	A	F	P	P	G	I	D	A	I	D	A

E	D	I	P	V	S	K	D	Y	A	F	P	P	G	I	D	A	I	D	A
E	D	I	P	V	S	K	D	Y	A	F	P	P	G	I	D	A	I	D	A
E	D	I	P	V	S	K	D	Y	A	F	P	P	G	I	D	A	I	D	A
E	D	I	P	V	S	K	D	Y	A	F	P	P	G	I	D	A	I	D	A
E	D	I	P	V	S	K	D	Y	A	F	P	P	G	I	D	A	I	D	A
E	D	I	P	V	S	K	D	Y	A	F	P	P	G	I	D	A	I	D	A
E	D	I	P	V	S	K	D	Y	A	F	P	P	G	I	D	A	I	D	A
E	D	I	P	V	S	K	D	Y	A	F	P	P	G	I	D	A	I	D	A
E	D	I	P	V	S	K	D	Y	A	F	P	P	G	I	D	A	I	D	A
E	N	V	P	V	S	K	D	H	A	F	P	P	G	I	D	A	V	E	A
E	N	V	P	V	S	K	D	H	A	F	P	P	G	I	D	A	V	E	A
E	N	V	P	V	S	K	D	H	A	F	P	P	G	I	D	A	V	E	A
E	D	I	P	V	N	R	D	Y	V	F	P	P	G	I	D	A	V	D	A
E	D	I	P	V	N	R	D	Y	V	F	P	P	G	I	D	A	V	D	A
E	D	I	P	V	N	R	D	Y	V	F	P	P	G	I	D	A	V	D	A
E	D	I	P	V	N	R	D	Y	V	F	P	P	G	I	D	A	V	D	A
E	D	I	P	V	N	R	D	Y	V	F	P	P	G	I	D	A	V	D	A
E	D	I	P	V	N	R	D	Y	V	F	P	P	G	I	D	A	V	D	A
E	D	I	P	V	N	R	D	Y	V	F	P	P	G	I	D	A	V	D	A
E	D	I	P	V	N	R	D	Y	V	F	P	P	G	I	D	A	V	D	A
E	D	I	P	V	N	R	D	Y	V	F	P	P	G	I	D	A	V	D	A
K	D	I	P	V	N	R	D	Y	V	F	P	P	G	I	D	A	V	D	A
K	D	I	P	V	N	R	D	Y	V	F	P	P	G	I	D	A	V	D	A
K	D	I	P	V	N	R	D	Y	V	F	P	P	G	I	D	A	V	D	A
K	D	I	P	V	N	R	D	Y	V	F	P	P	G	I	D	A	V	D	A
K	D	I	P	V	N	R	D	Y	V	F	P	P	G	I	D	A	V	D	A
K	D	I	P	V	N	R	D	Y	V	F	P	P	G	I	D	A	V	D	A
K	D	I	P	V	N	R	D	Y	V	F	P	P	G	I	D	A	V	D	A
K	D	I	P	V	N	R	D	Y	V	F	P	P	G	I	D	A	V	D	A
K	D	I	P	V	N	R	D	Y	V	F	P	P	G	I	D	A	V	D	A
K	D	I	P	V	N	R	D	Y	V	F	P	P	G	I	D	A	V	D	A
K	D	I	P	V	N	R	D	Y	V	F	P	P	G	I	D	A	V	D	A
K	D	I	P	V	N	R	D	Y	V	F	P	P	G	I	D	A	V	D	A
K	D	I	P	V	N	R	D	Y	V	F	P	P	G	I	D	A	V	D	A
K	D	I	P	V	N	R	D	Y	V	F	P	P	G	I	D	A	V	D	A
K	D	I	P	V	N	R	D	Y	V	F	P	P	G	I	D	A	V	D	A
K	D	I	P	V	N	R	D	Y	V	F	P	P	G	I	D	A	V	D	A
K	D	I	P	V	N	R	D	Y	V	F	P	P	G	I	D	A	V	D	A
K	D	I	P	V	N	R	D	Y	V	F	P	P	G	I	D	A	V	D	A
K	D	I	P	V	N	R	D	Y	V	F	P	P	G	I	D	A	V	D	A
K	D	I	P	V	N	R	D	Y	V	F	P	P	G	I	D	A	V	D	A
K	D	I	P	V	N	R	D	Y	V	F	P	P	G	I	D	A	V	D	A
K	D	I	P	V	N	R	D	Y	V	F	P	P	G	I	D	A	V	D	A
K	D	I	P	V	N	R	D	Y	V	F	P	P	G	I	D	A	V	D	A
K	D	I	P	V	N	R	D	Y	V	F	P	P	G	I	D	A	V	D	A
K	D	I	P	V	N	R	D	Y	V	F	P	P	G	I	D	A	V	D	A
K	D	I	P	V	N	R	D	Y	V	F	P	P	G	I	D	A	V	D	A
K	D	I	P	V	N	R	D	Y	V	F	P	P	G	I	D	A	V	D	A
K	D	I	P	V	N	R	D	Y	V	F	P	P	G	I	D	A	V	D	A
E	D	I	P	V	S	K	N	Y	A	F	P	P	G	I	D	A	I	D	A
E	D	I	P	V	S	K	N	Y	A	F	P	P	G	I	D	A	I	D	A
E	D	I	P	V	S	K	N	Y	A	F	P	L	G	I	D	A	I	D	A
E	D	I	P	V	S	K	N	Y	A	F	P	P	G	V	D	A	I	D	A
E	D	I	P	V	S	K	N	Y	A	F	P	P	G	I	D	A	I	D	A
E	D	I	P	V	S	K	N	Y	A	F	P	P	G	I	D	A	I	D	A
E	D	I	P	V	S	K	N	Y	A	F	P	P	G	I	D	A	I	D	A
E	D	I	P	V	S	K	N	Y	A	F	P	P	G	I	D	A	I	D	A
E	D	I	P	V	S	K	N	Y	A	F	P	P	G	I	D	A	I	D	A
E	D	I	P	V	S	K	N	Y	A	F	P	P	G	I	D	A	I	D	A
E	D	I	P	V	S	K	N	Y	A	F	P	P	G	I	D	A	I	D	A
E	D	I	P	V	S	K	N	Y	A	F	P	P	G	I	D	A	I	D	A
E	D	I	P	V	S	K	N	Y	A	F	P	P	G	I	D	A	I	D	A
E	D	I	P	V	S	K	N	Y	A	F	P	P	G	I	D	A	I	D	A
E	D	I	P	V	S	K	N	Y	A	F	P	P	G	I	D	A	I	D	A
E	D	I	P	V	S	K	N	Y	A	F	P	P	G	I	D	A	I	D	A
E	D	I	P	V	S	K	N	Y	A	F	P	P	G	I	D	A	I	D	A
E	D	I	P	V	S	K	N	Y	A	F	P	P	G	I	D	A	I	D	A

E	D	I	P	V	S	K	N	Y	A	F	P	P	G	I	D	A	I	D	A
E	D	I	P	V	S	K	N	Y	A	F	P	P	G	I	D	A	I	D	A
E	D	I	P	V	S	K	N	Y	A	F	P	P	G	I	D	A	I	D	A
E	D	I	P	V	S	K	N	Y	A	F	P	P	E	I	D	A	I	D	A
E	D	I	P	V	S	K	N	Y	A	F	P	P	G	I	D	A	I	D	A
E	D	I	P	V	S	K	N	Y	A	F	P	P	G	I	D	A	I	D	A
E	D	I	P	V	S	K	N	Y	A	F	P	P	G	I	D	A	I	D	A
E	D	I	P	V	S	K	N	Y	A	F	P	P	G	I	D	A	I	D	A
E	D	I	P	V	S	K	N	Y	A	F	P	P	G	I	D	A	I	D	A
E	D	I	P	V	S	K	N	Y	A	F	P	P	G	I	D	A	I	D	A
E	D	I	P	V	S	K	N	Y	A	F	P	P	G	I	D	A	I	D	A
E	D	I	P	V	S	K	N	Y	A	F	P	P	G	I	D	A	I	D	A
E	D	I	P	V	S	K	N	Y	A	F	P	P	G	I	D	A	I	D	A
E	D	I	P	V	S	K	N	Y	A	F	P	P	G	I	D	A	I	D	A
E	D	I	P	V	S	K	N	Y	A	F	P	P	G	I	D	A	I	D	A
E	D	I	P	V	S	K	N	Y	A	F	P	P	G	I	D	A	I	D	A
E	D	I	P	V	S	K	N	Y	A	F	P	P	G	I	D	A	I	D	A
E	D	I	P	V	S	K	N	Y	A	F	P	P	G	I	D	A	I	D	A
E	D	I	P	V	S	K	N	Y	A	F	P	P	G	I	D	A	I	D	A
E	D	I	P	V	S	K	N	Y	A	F	P	P	G	I	D	A	I	D	A
E	D	I	P	V	S	K	N	Y	A	F	P	P	G	I	D	A	I	D	A
E	D	I	P	V	S	K	N	Y	A	F	P	P	G	I	D	A	I	D	A
E	D	I	P	V	S	K	N	Y	A	F	P	P	G	I	D	A	I	D	A
E	D	I	P	V	S	K	N	Y	A	F	P	P	G	I	D	A	I	D	A
E	D	I	P	V	S	K	N	Y	A	F	P	P	G	I	D	A	I	D	A
E	D	I	P	V	S	K	N	Y	A	F	P	P	G	I	D	A	I	D	A
E	D	I	P	V	S	K	N	Y	A	F	P	P	G	I	D	A	I	D	A
E	D	I	P	V	S	K	N	Y	A	F	P	P	G	I	D	A	I	D	A
E	D	I	P	V	S	K	N	Y	A	F	P	P	G	I	D	A	I	D	A
E	D	I	P	V	S	K	N	Y	A	F	P	P	G	I	D	A	I	D	A
E	D	I	P	V	S	K	N	Y	A	F	P	P	G	I	D	A	I	D	A
E	D	I	P	V	S	K	N	Y	A	F	P	P	G	I	D	A	I	D	A
E	D	I	P	V	S	K	N	Y	A	F	P	P	G	I	D	A	I	D	A
E	D	I	P	V	S	K	N	Y	A	F	P	P	G	I	D	A	I	D	A
E	D	I	P	V	S	K	N	Y	A	F	P	P	G	I	D	A	I	D	A
E	D	I	P	V	S	K	N	Y	A	F	P	P	G	I	D	A	I	D	A
E	D	I	P	V	S	K	N	Y	A	F	P	P	G	I	D	A	I	D	A
E	D	I	P	V	S	K	N	Y	A	F	P	P	G	I	D	A	I	D	A
E	D	I	P	V	S	K	N	Y	A	F	P	P	G	I	D	A	I	D	A
E	D	I	P	V	S	K	N	Y	A	F	P	P	G	I	D	A	I	D	A
E	D	I	P	V	S	K	N	Y	A	F	P	P	G	I	D	A	I	D	A
E	D	I	P	V	S	K	N	Y	A	F	P	P	G	I	D	A	I	D	A
E	D	I	P	V	S	K	N	Y	A	F	P	P	G	I	D	A	I	D	A
E	D	I	P	V	S	K	N	Y	A	F	P	P	G	I	D	A	I	D	A
E	D	I	P	V	S	K	N	Y	A	F	P	P	G	I	D	A	I	D	A
E	D	I	P	V	S	K	N	Y	A	F	P	P	G	I	D	A	I	D	A
E	D	I	P	V	S	K	N	Y	A	F	P	P	G	I	D	A	I	D	A
E	D	I	P	V	S	K	N	Y	A	F	P	P	G	I	D	A	I	D	A
E	D	I	P	V	S	K	N	Y	A	F	P	P	G	I	D	A	I	D	A
E	D	I	P	V	S	K	N	Y	A	F	P	P	G	I	D	A	I	D	A
E	D	I	P	V	S	K	N	Y	A	F	P	P	G	I	D	A	I	D	A
E	D	I	P	V	S	K	N	Y	A	F	P	P	G	I	D	A	I	D	A
E	D	I	P	V	S	K	N	Y	A	F	P	P	G	I	D	A	I	D	A
E	D	I	P	V	S	K	N	Y	A	F	P	P	G	I	D	A	I	D	A
E	D	I	P	V	S	K	N	Y	A	F	P	P	G	I	D	A	I	D	A
E	D	I	P	V	S	K	N	Y	A	F	P	P	G	I	D	A	I	D	A
E	D	I	P	V	S	K	N	Y	A	F	P	P	G	I	D	A	I	D	A
E	D	I	P	V	S	K	N	Y	A	F	P	P	G	I	D	A	I	D	A
E	D	I	P	V	S	K	N	Y	A	F	P	P	G	I	D	A	I	D	A
E	D	I	P	V	S	K	N	Y	A	F	P	P	G	I	D	A	I	D	A
E	D	I	P	V	S	K	N	Y	A	F	P	P	G	I	D	A	I	D	A
E	D	I	P	V	S	K	N	Y	A	F	P	P	G	I	D	A	I	D	A
E	D	I	P	V	S	K	N	Y	A	F	P	P	G	I	D	A	I	D	A
E	D	I	P	V	S	K	N	Y	A	F	P	P	G	I	D	A	I	D	A
E	D	I	P	V	S	K	N	Y	A	F	P	P	G	I	D	A	I	D	A
E	D	I	P	V	S	K	N	Y	A	F	P	P	G	I	D	A	I	D	A
E	D	I	P	V	S	K	N	Y	A	F	P	P	G	I	D	A	I	D	A

E	D	I	P	V	S	K	N	Y	A	F	P	P	G	I	D	A	I	D	A
E	D	I	P	V	S	K	N	Y	A	F	P	P	G	I	D	A	I	D	A
E	D	I	P	V	S	K	N	Y	A	F	P	P	G	I	D	A	I	D	A
E	D	I	P	V	S	K	N	Y	A	F	P	P	G	I	D	A	I	D	A
E	D	I	P	V	S	K	N	Y	A	F	P	P	G	I	D	A	I	D	A
E	D	I	P	V	S	K	N	Y	A	F	P	P	G	I	D	A	I	D	A
E	D	I	P	V	S	K	N	Y	A	F	P	P	G	I	D	A	I	D	A
E	D	I	P	V	S	K	N	Y	A	F	P	P	G	I	D	A	I	D	A
E	D	I	P	V	S	K	N	Y	A	F	P	P	G	I	D	A	I	D	A
E	D	I	P	V	S	K	N	Y	A	F	P	P	G	I	D	A	I	D	A
E	D	I	P	V	S	K	N	Y	A	F	P	P	G	I	D	A	I	D	A
E	D	I	P	V	S	K	N	Y	A	F	P	P	G	I	D	A	I	D	A
E	D	I	P	V	S	K	N	Y	A	F	P	P	G	I	D	A	I	D	A
E	D	I	P	V	S	K	N	Y	A	F	P	P	G	I	D	A	I	D	A
E	D	I	P	V	S	K	N	Y	A	F	P	P	G	I	D	A	I	D	A
E	D	I	P	V	S	K	N	Y	A	F	Q	P	G	I	D	A	I	D	A
E	D	I	P	V	S	K	N	Y	A	F	Q	P	G	I	D	A	I	D	A
E	D	I	P	V	S	K	N	Y	A	F	P	P	G	I	D	A	I	D	A
E	D	I	P	V	S	K	N	Y	A	F	P	P	G	I	D	A	I	D	A
E	D	I	P	V	S	K	N	Y	A	F	P	P	G	I	D	A	I	D	A
E	D	I	P	V	S	K	N	Y	A	F	P	P	G	I	D	A	I	D	A
E	D	I	P	V	S	K	N	Y	A	F	P	P	G	I	D	A	I	D	A
E	D	I	P	V	S	K	N	Y	A	F	P	P	G	I	D	A	I	D	A
E	D	I	P	V	S	K	N	Y	A	F	P	P	G	I	D	A	I	D	A
E	D	I	P	V	S	K	N	Y	A	F	P	P	G	I	D	A	I	D	A
E	D	I	P	V	S	K	N	Y	A	F	P	P	G	I	D	A	I	D	A
E	D	I	P	V	S	K	N	Y	A	F	P	P	G	I	D	A	I	D	A

345	346	348	351	357	360	369	387	388	401	403	404	405	410	416	421	425	432	443	446	463	464	470
H	F	V	V	Q	G	A	I	K	S	L	Q	G	L	I	D	T	N	T	M	I	D	V
H	F	V	V	Q	G	A	I	K	S	L	Q	G	L	I	D	T	N	T	M	I	D	V
H	F	V	V	Q	G	A	I	K	S	L	Q	G	L	I	D	T	N	T	M	I	D	V
H	F	V	V	Q	G	A	I	K	S	L	Q	G	L	I	D	T	N	T	M	I	D	V
H	F	V	V	Q	G	A	I	K	S	L	Q	G	L	I	D	T	N	T	M	I	D	V
H	F	V	V	Q	G	A	I	K	S	L	Q	G	L	I	D	T	N	T	M	I	D	V
H	F	V	V	Q	G	A	I	K	S	L	Q	G	L	I	D	T	N	T	M	I	D	V
H	F	V	V	Q	G	A	I	K	S	L	Q	G	L	I	D	T	N	T	M	I	D	V
H	F	V	V	Q	G	A	I	K	S	L	Q	G	L	I	D	T	N	T	M	I	D	V
H	F	V	V	Q	G	A	I	K	S	L	Q	G	L	I	D	T	N	T	M	I	D	V
H	F	V	V	Q	G	A	I	K	S	L	Q	G	L	I	D	T	N	T	M	I	D	V
H	F	V	V	Q	G	A	I	K	S	L	Q	G	L	I	D	T	N	T	M	I	D	V
H	F	V	V	Q	G	A	I	K	S	L	Q	G	L	I	D	T	N	T	M	I	D	V
H	F	V	V	Q	G	A	I	K	S	L	Q	G	L	I	D	T	N	T	M	I	D	V
H	F	V	V	Q	G	A	I	K	S	L	Q	G	L	I	D	T	N	T	M	I	D	V
H	F	V	V	Q	G	A	I	K	S	L	Q	G	L	I	D	T	N	T	M	I	D	V
H	F	V	V	Q	G	A	I	K	S	L	Q	G	L	I	D	T	N	T	M	I	D	V
H	F	V	V	Q	G	A	I	K	S	L	Q	G	L	I	D	T	N	T	M	I	D	V
H	F	V	V	Q	G	A	I	K	S	L	Q	G	L	I	D	T	N	T	M	I	D	V
H	F	V	V	Q	G	A	I	K	S	L	Q	G	L	I	D	T	N	T	M	I	D	V
H	F	V	V	Q	G	A	I	K	S	L	Q	G	L	I	D	T	N	T	M	I	D	V
H	F	V	V	Q	G	A	I	K	S	L	Q	G	L	I	D	T	N	T	M	I	D	V
H	F	V	V	Q	G	A	I	K	S	L	Q	G	L	I	D	T	N	T	M	I	D	V
H	F	V	V	Q	G	A	I	K	S	L	Q	G	L	I	D	T	N	T	M	I	D	V
H	F	V	V	Q	G	A	I	K	S	L	Q	G	L	I	D	T	N	T	M	I	D	V
H	F	V	V	Q	G	A	I	K	S	L	Q	G	L	I	D	T	N	T	M	I	D	V
H	F	V	V	Q	G	A	I	K	S	L	Q	G	L	I	D	T	N	T	M	I	D	V
H	F	V	V	Q	G	A	I	K	S	L	Q	G	L	I	D	T	N	T	M	I	D	V
H	F	V	V	Q	G	A	I	K	S	L	Q	G	L	I	D	T	N	T	M	I	D	V
H	F	V	V	Q	G	A	I	K	S	L	Q	G	L	I	D	T	N	T	M	I	D	V
H	F	V	V	Q	G	A	I	K	S	L	Q	G	L	I	D	T	N	T	M	V	D	V
H	F	V	V	Q	G	A	I	K	S	L	Q	G	L	I	D	T	N	T	M	V	D	V
H	F	V	V	Q	G	A	I	K	S	L	Q	G	L	I	D	T	N	T	M	V	D	V
H	F	V	V	Q	G	A	I	K	S	L	Q	G	L	I	D	T	N	T	M	V	D	V
H	F	V	V	Q	G	A	I	K	S	L	Q	G	L	I	D	T	N	T	M	V	D	V
H	F	V	V	Q	G	A	I	K	S	L	Q	G	L	I	D	T	N	T	M	V	D	V
H	F	V	V	Q	G	A	I	K	S	L	Q	G	L	I	D	T	N	T	M	V	D	V
H	F	V	V	Q	G	A	I	K	S	L	Q	G	L	I	D	T	N	T	M	V	D	V
H	F	V	V	Q	G	A	I	K	S	L	Q	G	L	I	D	T	N	T	M	V	D	V
H	F	V	V	Q	G	A	I	K	S	L	Q	G	L	I	D	T	N	T	M	V	D	V
H	F	V	V	Q	G	A	I	K	S	L	Q	G	L	I	D	T	N	T	M	V	D	V
H	F	V	V	Q	G	A	I	K	S	L	Q	G	L	I	D	T	N	T	M	V	D	V
H	F	V	V	Q	G	A	I	K	S	L	Q	G	L	I	D	T	N	T	M	V	D	V
H	F	V	V	Q	G	A	I	K	S	L	Q	G	L	I	D	T	N	T	M	V	D	V
H	F	V	V	Q	G	A	I	K	S	L	Q	G	L	I	D	T	N	T	M	V	D	V
H	F	V	V	Q	G	A	I	K	S	L	Q	G	L	I	D	T	N	T	M	V	D	V
H	F	V	V	Q	G	A	I	K	S	L	Q	G	L	I	D	T	N	T	M	V	D	V
H	F	V	V	Q	G	A	I	K	S	L	Q	G	L	I	D	T	N	T	M	V	D	V
H	F	V	V	Q	G	A	I	K	S	L	Q	G	L	I	D	T	N	T	M	V	D	V
H	F	V	V	Q	G	A	I	K	S	L	Q	G	L	I	D	T	N	T	M	V	D	V
H	F	V	V	Q	G	A	I	K	S	L	Q	G	L	I	D	T	N	T	M	V	D	V
H	F	V	V	Q	G	A	I	K	S	L	Q	G	L	I	D	T	N	T	M	V	D	V
H	F	V	V	Q	G	A	I	K	S	L	Q	G	L	I	D	T	N	T	M	V	D	V
H	F	V	V	Q	G	A	I	K	S	L	Q	G	L	I	D	T	N	T	M	I	D	V
H	F	V	V	Q	G	A	I	K	S	L	Q	G	L	I	D	T	N	T	M	V	D	V
H	F	V	V	Q	G	A	I	K	S	L	Q	G	L	I	D	T	N	T	M	V	D	V
H	F	V	V	Q	G	A	I	K	S	L	Q	G	L	I	D	T	N	T	M	V	D	V
H	F	V	V	Q	G	A	I	K	S	L	Q	G	L	I	D	T	N	T	M	V	D	V
H	F	V	V	Q	G	A	I	K	S	L	Q	G	L	I	D	T	N	T	M	V	D	V
H	F	V	V	Q	G	A	I	K	S	L	Q	G	L	I	D	T	N	T	M	I	D	V
H	F	V	V	Q	G	A	I	K	S	L	Q	G	L	I	D	T	N	T	M	I	D	V
H	F	V	V	Q	G	A	I	K	S	L	Q	G	L	I	D	T	N	T	M	I	D	V
H	F	V	V	Q	G	A	I	K	S	L	Q	G	L	I	D	T	N	T	M	I	D	V

H	F	V	V	Q	G	A	I	K	S	L	Q	G	L	I	D	T	N	T	M	I	D	V
H	F	V	V	Q	G	A	I	K	S	L	Q	G	L	I	D	T	N	T	M	I	D	V
H	F	V	V	Q	G	A	I	K	S	L	Q	G	L	I	D	T	N	T	M	I	D	V
H	F	V	V	Q	G	A	I	K	S	L	Q	G	L	I	D	T	N	T	M	I	D	V
H	F	V	V	Q	G	A	I	K	S	L	Q	G	L	I	D	T	N	T	M	I	D	V
H	F	V	V	Q	G	A	I	K	S	L	Q	G	L	I	D	T	N	T	M	I	D	V
H	F	V	V	Q	G	A	I	K	S	L	Q	G	L	I	D	T	N	T	M	I	D	V
H	F	V	V	Q	G	A	I	K	S	L	Q	G	L	I	D	T	N	T	M	I	D	V
H	F	V	V	Q	G	A	I	K	S	L	Q	G	L	I	D	T	N	T	M	I	D	V
H	F	V	V	Q	G	A	I	K	S	L	Q	G	L	I	D	T	N	T	M	I	D	V
H	F	V	V	Q	G	A	I	K	S	L	Q	G	L	I	D	T	N	T	M	I	D	V
H	F	V	V	Q	G	A	I	K	S	L	Q	G	L	I	D	T	N	T	M	I	D	V
H	F	V	V	Q	G	A	I	K	S	L	Q	G	L	I	D	T	N	T	M	I	D	V
H	F	V	V	Q	G	A	I	K	S	L	Q	G	L	I	D	T	N	T	M	I	D	V
H	F	V	V	Q	G	A	I	K	S	L	Q	G	L	I	D	T	N	T	M	I	D	V
H	F	V	V	Q	G	A	I	K	S	L	Q	G	L	I	D	T	N	T	M	I	D	V
H	F	V	V	Q	G	A	I	K	S	L	Q	G	L	I	D	T	N	T	M	I	D	V
H	F	V	V	Q	G	A	I	K	S	L	Q	G	L	I	D	T	N	T	M	I	D	V
H	F	V	V	Q	G	A	I	K	S	L	Q	G	L	I	D	T	N	T	M	I	D	V
H	F	V	V	Q	G	A	I	K	S	L	Q	G	L	I	D	T	N	T	M	I	D	V
H	F	V	V	Q	G	A	I	K	S	L	Q	G	L	I	D	T	N	T	M	I	D	V
H	F	V	V	Q	G	A	I	K	S	L	Q	G	L	I	D	T	N	T	M	V	D	V
H	F	V	V	Q	G	A	I	K	S	L	Q	G	L	I	D	T	N	T	M	V	D	V
H	F	V	V	Q	G	A	I	K	S	L	Q	G	L	I	D	T	N	T	M	V	D	V
H	F	V	V	Q	G	A	I	K	S	L	Q	G	L	I	D	T	N	T	M	V	D	V
H	F	V	V	Q	G	A	I	K	S	L	Q	G	L	I	D	T	N	T	M	V	D	V
H	F	V	V	Q	G	A	I	K	S	L	Q	G	L	I	D	T	N	T	M	V	D	V
H	F	V	V	Q	G	A	I	K	S	L	Q	G	L	I	D	T	N	T	M	I	D	V
H	F	V	V	Q	G	A	I	K	S	L	Q	G	L	I	D	T	N	T	M	I	D	V
H	F	V	V	Q	G	A	I	K	S	L	Q	G	L	I	D	T	N	T	M	I	D	V
H	F	V	V	Q	G	A	I	K	S	L	Q	G	L	I	D	T	N	T	M	I	D	V
H	F	V	V	Q	G	A	I	K	S	L	Q	G	L	I	D	T	N	T	M	I	D	V
H	F	V	V	Q	G	A	I	K	S	L	Q	G	L	I	D	T	N	T	M	I	D	V
H	F	V	V	Q	G	A	I	K	S	L	Q	G	L	I	D	T	N	T	M	I	D	V
H	F	V	V	Q	G	A	I	K	S	L	Q	G	L	I	D	T	N	T	M	I	D	V
H	F	V	V	Q	G	A	I	K	S	L	Q	G	L	I	D	T	N	T	M	I	D	V
H	F	V	V	Q	G	A	I	K	S	L	Q	G	L	I	D	T	N	T	M	I	D	V
H	F	V	V	Q	G	A	I	K	S	L	Q	G	L	I	D	T	N	T	M	I	D	V
H	F	V	V	Q	G	A	I	K	S	L	Q	G	L	I	D	T	N	T	M	I	D	V
H	F	V	V	Q	G	A	I	K	S	L	Q	G	L	I	D	T	N	T	M	I	D	V
H	F	V	V	Q	G	A	I	K	S	L	Q	G	L	I	D	T	N	T	M	I	D	V
H	F	V	V	Q	G	A	I	K	S	L	Q	G	L	I	D	T	N	T	M	I	D	V
H	F	V	V	Q	G	A	I	K	S	L	Q	G	L	I	D	T	N	T	M	I	D	V
H	F	V	V	Q	G	A	I	K	S	L	Q	G	L	I	D	T	N	T	M	I	D	V
H	F	V	V	Q	G	A	I	K	S	L	Q	G	L	I	D	T	N	T	M	I	D	V
H	F	V	V	Q	G	A	I	K	S	L	Q	G	L	I	D	T	N	T	M	I	D	V
H	F	V	V	Q	G	A	I	K	S	L	Q	G	L	I	D	T	N	T	M	I	D	V
H	F	V	V	Q	G	A	I	K	S	L	Q	G	L	I	D	T	N	T	M	I	D	V
H	F	V	V	Q	G	A	I	K	S	L	Q	G	L	I	D	T	N	T	M	I	D	V
H	F	V	V	Q	G	A	I	K	S	L	Q	G	L	I	D	T	N	T	M	I	D	V
H	F	V	V	Q	G	A	I	K	S	L	Q	G	L	I	D	T	N	T	M	I	D	V
H	F	V	V	Q	G	A	I	K	S	L	Q	G	L	I	D	T	N	T	M	I	D	V
H	F	V	V	Q	G	A	I	K	S	L	Q	G	L	I	D	T	N	T	M	I	D	V
H	F	V	V	Q	G	A	I	K	S	L	Q	G	L	I	D	T	N	T	M	I	D	V
H	F	V	V	Q	G	A	I	K	S	L	Q	G	L	I	D	T	N	T	M	I	D	V
H	F	V	V	Q	G	A	I	K	S	L	Q	G	L	I	D	T	N	T	M	I	D	V
H	F	V	V	Q	G	A	I	K	S	L	Q	G	L	I	D	T	N	T	M	I	D	V
H	F	V	V	Q	G	A	I	K	S	L	Q	G	L	I	D	T	N	T	M	I	D	V
H	F	V	V	Q	G	A	I	K	S	L	Q	G	L	I	D	T	N	T	M	I	D	V
H	F	V	V	Q	G	A	I	K	S	L	Q	G	L	I	D	T	N	T	M	I	D	V
H	F	V	V	Q	G	A	I	K	S	L	Q	G	L	I	D	T	N	T	M	I	D	V
H	F	V	V	Q	G	A	I	K	S	L	Q	G	L	I	D	T	N	T	M	I	D	V
H	F	V	V	Q	G	A	I	K	S	L	Q	G	L	I	D	T	N	T	M	I	D	V
H	F	V	V	Q	G	A	I	K	S	L	Q	G	L	I	D	T	N	T	M	I	D	V
H	F	V	V	Q	G	A	I	K	S	L	Q	G	L	I	D	T	N	T	M	I	D	V
H	F	V	V	Q	G	A	I	K	S	L	Q	G	L	I	D	T	N	T	M	I	D	V
H	F	V	V	Q	G	A	I	K	S	L	Q	G	L	I	D	T	N	T	M	I	D	V

H	F	V	V	Q	G	A	I	K	S	L	Q	G	L	I	D	T	N	T	M	I	D	V
H	F	V	V	Q	G	A	I	K	S	L	Q	G	L	I	D	T	N	T	M	I	D	V
H	F	V	V	Q	G	A	I	K	S	L	Q	G	L	I	D	T	N	T	M	I	D	V
H	F	V	V	Q	G	A	I	K	S	L	Q	G	L	I	D	T	N	T	M	I	D	V
H	F	V	V	Q	G	A	I	K	S	L	Q	G	L	I	D	T	N	T	M	I	D	V
H	F	V	V	Q	G	A	I	K	S	L	Q	G	L	I	D	T	N	T	M	I	D	V
H	F	V	V	Q	G	A	I	K	S	L	Q	G	L	I	D	T	N	T	M	I	D	V
H	F	V	V	Q	G	A	I	K	S	L	Q	G	L	I	D	T	N	T	M	I	D	V
H	F	V	V	Q	G	A	I	K	S	L	Q	G	L	I	D	T	N	T	M	I	D	V
H	F	V	V	Q	G	A	I	K	S	L	Q	G	L	I	D	T	N	T	M	I	D	V
H	F	V	V	Q	G	A	I	K	S	L	Q	G	L	I	D	T	N	T	M	I	D	V
H	F	V	V	Q	G	A	I	K	S	L	Q	G	L	I	D	T	N	T	M	I	D	V
H	F	V	V	Q	G	A	I	K	S	L	Q	G	L	I	D	T	N	T	M	I	D	V
H	F	V	V	Q	G	A	I	K	S	L	Q	G	L	I	D	T	N	T	M	I	D	V
H	F	V	V	Q	G	A	I	K	S	L	Q	G	L	I	D	T	N	T	M	I	D	V
H	F	V	V	Q	G	A	I	K	S	L	Q	G	L	I	D	T	N	T	M	I	D	V
H	F	V	V	Q	G	A	I	K	S	L	Q	G	L	I	D	T	N	T	M	I	D	V
H	F	V	V	Q	G	A	I	K	S	L	Q	G	L	I	D	T	N	T	M	I	D	V
H	F	V	V	Q	G	A	I	K	S	L	Q	G	L	I	D	T	N	T	M	I	D	V
H	F	V	V	Q	G	A	I	K	S	L	Q	G	L	I	D	T	N	T	M	I	D	V
H	F	V	V	Q	G	A	I	K	S	L	Q	G	L	I	D	T	N	T	M	I	D	V
H	F	V	V	Q	G	A	I	K	S	L	Q	G	L	I	D	T	N	T	M	I	D	V
H	F	V	V	Q	G	A	I	K	S	L	Q	G	L	I	D	T	N	T	M	I	D	V
H	F	V	V	Q	G	A	I	K	S	L	Q	G	L	I	D	T	N	T	M	I	D	V
H	F	V	V	Q	G	A	I	K	S	L	Q	G	L	I	D	T	N	T	M	I	D	V
H	F	V	V	Q	G	A	I	K	S	L	Q	G	L	I	D	T	N	T	M	I	D	V
H	F	V	V	Q	G	A	I	K	S	L	Q	G	L	I	D	T	N	T	M	I	D	V
H	F	V	V	Q	G	A	I	K	S	L	Q	G	L	I	D	T	N	T	M	I	D	V
H	F	V	V	Q	G	A	I	K	S	L	Q	G	L	I	D	T	N	T	M	I	D	V
H	F	V	V	Q	G	A	I	K	S	L	Q	G	L	I	D	T	N	T	M	I	D	V
H	F	V	V	Q	G	A	I	K	S	L	Q	G	L	I	D	T	N	T	M	I	D	V
H	F	V	V	Q	G	A	I	K	S	L	Q	G	L	I	D	T	N	T	M	I	D	V
H	F	V	V	Q	G	A	I	K	S	L	Q	G	L	I	D	T	N	T	M	I	D	V
H	F	V	V	Q	G	A	I	K	S	L	Q	G	L	I	D	T	N	T	M	I	D	V
H	F	V	V	Q	G	A	I	K	S	L	Q	G	L	I	D	T	N	T	M	I	D	V
H	F	V	V	Q	G	A	I	K	S	L	Q	G	L	I	D	T	N	T	M	I	D	V
H	F	V	V	Q	G	A	I	K	S	L	Q	G	L	I	D	T	N	T	M	I	D	V
H	F	V	V	Q	G	A	I	K	S	L	Q	G	L	I	D	T	N	T	M	I	D	V
H	F	V	V	Q	G	A	I	K	S	L	Q	G	L	I	D	T	N	T	M	I	D	V
H	F	V	V	Q	G	A	I	K	S	L	Q	G	L	I	D	T	N	T	M	I	D	V
H	F	V	V	Q	G	A	I	K	S	L	Q	G	L	I	D	T	N	T	M	I	D	V
H	F	V	V	Q	G	A	I	K	S	L	Q	G	L	I	D	T	N	T	M	I	D	V
H	F	V	V	Q	G	A	I	K	S	L	Q	G	L	I	D	T	N	T	M	I	D	V
H	F	V	V	Q	G	A	I	K	S	L	Q	G	L	I	D	T	N	T	M	I	D	V
H	F	V	V	Q	G	A	I	K	S	L	Q	G	L	I	D	T	N	T	M	I	D	V
H	F	V	V	Q	G	A	I	K	S	L	Q	G	L	I	D	T	N	T	M	I	D	V
H	F	V	V	Q	G	A	I	K	S	L	Q	G	L	I	D	T	N	T	M	I	D	V
H	F	V	V	Q	G	A	I	K	S	L	Q	G	L	I	D	T	N	T	M	I	D	V
H	F	V	V	Q	G	A	I	K	S	L	Q	G	L	I	D	T	N	T	M	I	D	V
H	F	V	V	Q	G	A	I	K	S	L	Q	G	L	I	D	T	N	T	M	I	D	V
H	F	V	V	Q	G	A	I	K	S	L	Q	G	L	I	D	T	N	T	M	I	D	V
H	F	V	V	Q	G	A	I	K	S	L	Q	G	L	I	D	T	N	T	M	I	D	V
H	F	V	V	Q	G	A	I	K	S	L	Q	G	L	I	D	T	N	T	M	I	D	V
H	F	V	V	Q	G	A	I	K	S	L	Q	G	L	I	D	T	N	T	M	I	D	V
H	F	V	V	Q	G	A	I	K	S	L	Q	G	L	I	D	T	N	T	M	I	D	V
H	F	V	V	Q	G	A	I	K	S	L	Q	G	L	I	D	T	N	T	M	I	D	V
H	F	V	V	Q	G	A	I	K	S	L	Q	G	L	I	D	T	N	T	M	I	D	V
H	F	V	V	Q	G	A	I	K	S	L	Q	G	L	I	D	T	N	T	M	I	D	V
H	F	V	V	Q	G	A	I	K	S	L	Q	G	L	I	D	T	N	T	M	I	D	V
H	F	V	V	Q	G	A	I	K	S	L	Q	G	L	I	D	T	N	T	M	I	D	V
H	F	V	V	Q	G	A	I	K	S	L	Q	G	L	I	D	T	N	T	M	I	D	V
H	F	V	V	Q	G	A	I	K	S	L	Q	G	L	I	D	T	N	T	M	I	D	V
H	F	V	V	Q	G	A	I	K	S	L	Q	G	L	I	D	T	N	T	M	I	D	V
H	F	V	V	Q	G	A	I	K	S	L	Q	G	L	I	D	T	N	T	M	I	D	V
H	F	V	V	Q	G	A	I	K	S	L	Q	G	L	I	D	T	N	T	M	I	D	V
H	F	V	V	Q	G	A	I	K	S	L	Q	G	L	I	D	T	N	T	M	I	D	V
H	F	V	V	Q	G	A	I	K	S	L	Q	G	L	I	D	T	N	T	M	I	D	V

H	F	V	V	Q	G	A	I	K	S	L	Q	G	L	I	D	T	N	T	M	I	D	V
H	F	V	V	Q	G	A	I	K	S	L	Q	G	L	I	D	T	N	T	M	I	D	V
H	F	V	V	Q	G	A	I	K	S	L	Q	G	L	I	D	T	N	T	M	I	D	V
H	F	V	V	Q	G	A	I	K	S	L	Q	G	L	I	D	T	N	T	M	I	D	V
H	F	V	V	Q	G	A	I	K	S	L	Q	G	L	I	D	T	N	T	M	V	D	V
H	F	V	V	Q	G	A	I	K	S	L	Q	G	L	I	D	T	N	T	M	V	D	V
H	F	V	V	Q	G	A	I	K	S	L	Q	G	L	I	D	T	N	T	M	V	D	V
H	F	V	V	Q	G	A	I	K	S	L	Q	G	L	I	D	T	N	T	M	V	D	V
H	F	V	V	Q	G	A	I	K	S	L	Q	G	L	I	D	T	N	T	M	V	D	V
H	F	V	V	Q	G	A	I	K	S	L	Q	G	L	I	D	T	N	T	M	V	D	V
H	F	V	V	Q	G	A	I	K	S	L	Q	G	L	I	D	T	N	T	M	V	D	V
H	F	V	V	Q	G	A	I	K	S	L	Q	G	L	I	D	T	N	T	M	V	D	V
H	F	V	V	Q	G	A	I	K	S	L	Q	G	L	I	D	T	N	T	M	V	D	V
H	F	V	V	Q	G	A	I	K	S	L	Q	G	L	I	D	T	N	T	M	V	D	V
H	F	V	V	Q	G	A	I	K	S	L	Q	G	L	I	D	T	N	T	M	V	D	V
H	F	V	V	Q	G	A	I	K	S	L	Q	G	L	I	D	T	N	T	M	V	D	V
H	F	V	V	Q	G	A	I	K	S	L	Q	G	L	I	D	T	N	T	M	V	D	V
H	F	V	V	Q	G	A	I	K	S	L	Q	G	L	I	D	T	N	T	M	V	D	V
H	F	V	V	Q	G	A	I	K	S	L	Q	G	L	I	D	T	N	T	M	V	D	V
H	F	V	V	Q	G	A	I	K	S	L	Q	G	L	I	D	T	N	T	M	V	D	V
H	F	V	V	Q	G	A	I	K	S	L	Q	G	L	I	D	T	N	T	M	V	D	V
H	F	V	V	Q	G	A	I	K	S	L	Q	G	L	I	D	T	N	T	M	V	D	V
H	F	V	V	Q	G	A	I	K	S	L	Q	G	L	I	D	T	N	T	M	V	D	V
H	F	V	V	Q	G	A	I	K	S	L	Q	G	L	I	D	T	N	T	M	V	D	V
H	F	V	V	Q	G	A	I	K	S	L	Q	G	L	I	D	T	N	T	M	V	D	V
H	F	V	V	Q	G	A	I	K	S	L	Q	G	L	I	D	T	N	T	M	V	D	V
H	F	V	V	Q	G	A	I	K	S	L	Q	G	L	I	D	T	N	T	M	V	D	V
H	F	V	V	Q	G	A	I	K	S	L	Q	G	L	I	D	T	N	T	M	V	D	V
H	F	V	V	Q	G	A	I	K	S	L	Q	G	L	I	D	T	N	T	M	V	D	V
H	F	V	V	Q	G	A	I	K	S	L	Q	G	L	I	D	T	N	T	M	V	D	V
H	F	V	V	Q	G	A	I	K	S	L	Q	G	L	I	D	T	N	T	M	V	D	V
H	F	V	V	Q	G	A	I	K	S	L	Q	G	L	I	D	T	N	T	M	V	D	V
H	F	V	V	Q	G	A	I	K	S	L	Q	G	L	I	D	T	N	T	M	V	D	V
H	F	V	V	Q	G	A	I	K	S	L	Q	G	L	I	D	T	N	T	M	V	D	V
H	F	V	V	Q	G	A	I	K	S	L	Q	G	L	I	D	T	N	T	M	V	D	V
H	F	V	V	Q	G	A	I	K	S	L	Q	G	L	I	D	T	N	T	M	V	D	V
H	F	V	V	Q	G	A	I	K	S	L	Q	G	L	I	D	T	N	T	M	V	D	V
H	F	V	V	Q	G	A	I	K	S	L	Q	G	L	I	D	T	N	T	M	V	D	V
H	F	V	V	Q	G	A	I	K	S	L	Q	G	L	I	D	T	N	T	M	V	D	V
H	F	V	V	Q	G	A	I	K	S	L	Q	G	L	I	D	T	N	T	M	V	D	V
H	F	V	V	Q	G	A	I	K	S	L	Q	G	L	I	D	T	N	T	M	V	D	V
H	F	V	V	Q	G	A	I	K	S	L	Q	G	L	I	D	T	N	T	M	V	D	V
H	F	V	V	Q	G	A	I	K	S	L	Q	G	L	I	D	T	N	T	M	V	D	V
H	F	V	V	Q	G	A	I	K	S	L	Q	G	L	I	D	T	N	T	M	V	D	V
H	F	V	V	Q	G	A	I	K	S	L	Q	G	L	I	D	T	N	T	M	V	D	V
H	F	V	V	Q	G	A	I	K	S	L	Q	G	L	I	D	T	N	T	M	V	D	V
H	F	V	V	Q	G	A	I	K	S	L	Q	G	L	I	D	T	N	T	M	V	D	V
H	F	V	V	Q	G	A	I	K	S	L	Q	G	L	I	D	T	N	T	M	V	D	V
H	F	V	V	Q	G	A	I	K	S	L	Q	G	L	I	D	T	N	T	M	V	D	V
H	F	V	V	Q	G	A	I	K	S	L	Q	G	L	I	D	T	N	T	M	V	D	V
H	F	V	V	Q	G	A	I	K	S	L	Q	G	L	I	D	T	N	T	M	V	D	V
H	F	V	V	Q	G	A	I	K	S	L	Q	G	L	I	D	T	N	T	M	V	D	V
H	F	V	V	Q	G	A	I	K	S	L	Q	G	L	I	D	T	N	T	M	V	D	V
H	F	V	V	Q	G	A	I	K	S	L	Q	G	L	I	D	T	N	T	M	V	D	V
H	F	V	V	Q	G	A	I	K	S	L	Q	G	L	I	D	T	N	T	M	V	D	V
H	F	V	V	Q	G	A	I	K	S	L	Q	G	L	I	D	T	N	T	M	V	D	V
H	F	V	V	Q	G	A	I	K	S	L	Q	G	L	I	D	T	N	T	M	V	D	V
H	F	V	V	Q	G	A	I	K	S	L	Q	G	L	I	D	T	N	T	M	V	D	V
H	F	V	V	Q	G	A	I	K	S	L	Q	G	L	I	D	T	N	T	M	V	D	V
H	F	V	V	Q	G	A	I	K	S	L	Q	G	L	I	D	T	N	T	M	V	D	V
H	F	V	V	Q	G	A	I	K	S	L	Q	G	L	I	D	T	N	T	M	V	D	V
H	F	V	V	Q	G	A	I	K	S	L	Q	G	L	I	D	T	N	T	M	V	D	V
H	F	V	V	Q	G	A	I	K	S	L	Q	G	L	I	D	T	N	T	M	V	D	V
H	F	V	V	Q	G	A	I	K	S	L	Q	G	L	I	D	T	N	T	M	V	D	V
H	F	V	V	Q	G	A	I	K	S	L	Q	G	L	I	D	T	N	T	M	V	D	V
H	F	V	V	Q	G	A	I	K	S	L	Q	G	L	I	D	T	N	T	M	V	D	V
H	F	V	V	Q	G	A	I	K	S	L	Q	G	L	I	D	T	N	T	M	V	D	V

H	F	V	V	Q	G	A	I	K	S	L	Q	G	L	I	D	T	N	T	M	V	D	V
H	F	V	V	Q	G	A	I	K	S	L	Q	G	L	I	D	T	N	T	M	V	D	V
H	F	V	V	Q	G	A	I	K	S	L	Q	G	L	I	D	T	N	T	M	V	D	V
H	F	V	V	Q	G	A	I	K	S	L	Q	G	L	I	D	T	N	T	M	V	D	V
H	F	V	V	Q	G	A	I	K	S	L	Q	G	L	I	D	T	N	T	M	V	D	V
H	F	V	V	Q	G	A	I	K	S	L	Q	G	L	I	D	T	N	T	M	V	D	V
H	F	V	V	Q	G	A	I	K	S	L	Q	G	L	I	D	T	N	T	M	V	D	V
H	F	V	V	Q	G	A	I	K	S	L	Q	G	L	I	D	T	N	T	M	V	D	V
H	F	V	V	Q	G	A	I	K	S	L	Q	G	L	I	D	T	N	T	M	V	D	V
H	F	V	V	Q	G	A	I	K	S	L	Q	G	L	I	D	T	N	T	M	V	D	V
H	F	V	V	Q	G	A	I	K	S	L	Q	G	L	I	D	T	N	T	M	V	D	V
H	F	V	V	Q	G	A	I	K	S	L	Q	G	L	I	D	T	N	T	M	I	D	V
H	F	V	V	Q	G	A	I	K	S	L	Q	G	L	I	D	T	N	T	M	I	D	V
H	F	V	V	Q	G	A	I	K	S	L	Q	G	L	I	D	T	N	T	M	I	D	V
H	F	V	V	Q	G	A	I	K	S	L	Q	G	L	I	D	T	N	T	M	I	D	V
H	F	V	V	Q	G	A	I	K	S	L	Q	G	L	I	D	T	N	T	M	I	D	V
H	F	V	V	Q	G	A	I	K	S	L	Q	G	L	I	D	T	N	T	M	I	D	V
H	F	V	V	Q	G	A	I	K	S	L	Q	G	L	I	D	T	N	T	M	I	D	V
H	F	V	V	Q	G	A	I	K	S	L	Q	G	L	I	D	T	N	T	M	I	D	V
H	F	V	V	Q	G	A	I	K	S	L	Q	G	L	I	D	T	N	T	M	I	D	V
Y	-	I	V	E	A	A	L	Q	T	I	D	S	I	L	N	A	T	R	T	I	N	S
Y	-	I	V	E	A	A	L	Q	T	I	D	S	I	L	N	A	T	R	T	I	N	S
Y	-	I	V	E	A	A	L	Q	T	I	D	S	I	L	N	A	T	R	T	I	N	S
Y	-	I	V	E	A	A	L	Q	T	I	D	S	I	L	N	A	T	R	T	I	N	S
Y	-	I	V	E	A	A	L	Q	T	I	D	S	I	L	N	A	T	R	T	I	N	S
Y	-	I	V	E	A	A	L	Q	T	I	D	S	I	L	N	A	T	R	T	I	N	S
Y	-	I	V	E	A	A	L	Q	T	I	D	S	I	L	N	A	T	R	T	I	N	S
Y	-	I	V	E	A	A	L	Q	T	I	D	S	I	L	N	A	T	R	T	I	N	S
Y	-	I	I	E	A	A	I	K	S	L	Q	G	L	I	D	T	N	T	M	V	D	V
Y	-	I	V	E	A	A	L	Q	T	I	D	S	I	L	N	A	T	T	M	V	D	V
Y	-	I	V	E	A	A	L	Q	T	I	D	S	I	L	N	A	T	T	M	V	D	V
Y	-	I	V	E	A	A	L	Q	T	I	D	S	I	L	N	A	T	T	M	V	D	V
Y	-	I	V	E	A	A	L	Q	T	I	D	S	I	L	N	A	T	T	M	V	D	V
Y	-	I	V	E	A	A	L	Q	T	I	D	S	I	L	N	A	T	T	M	V	D	V
Y	-	I	V	E	A	A	L	Q	T	I	D	S	I	L	N	A	T	T	M	V	D	V
Y	-	I	V	E	A	A	L	Q	T	I	D	S	I	L	N	A	T	T	M	V	D	V
Y	-	I	V	E	A	A	L	Q	T	I	D	S	I	L	N	A	T	T	M	V	D	V
Y	-	I	V	E	A	A	L	Q	T	I	D	S	I	L	N	A	T	T	M	V	D	V
Y	-	I	V	E	A	A	L	Q	T	I	D	S	I	L	N	A	T	T	M	V	D	V
Y	-	I	V	E	A	A	L	Q	T	I	D	S	I	L	N	A	T	T	M	V	D	V
Y	-	I	V	E	A	A	L	Q	T	I	D	S	I	L	N	A	T	T	M	V	D	V
Y	-	I	V	E	A	A	L	Q	T	I	D	S	I	L	N	A	T	T	M	V	D	V
Y	-	I	V	E	A	A	L	Q	T	I	D	S	I	L	N	A	T	T	M	V	D	V
Y	-	I	V	E	A	A	L	Q	T	I	D	S	I	L	N	A	T	T	M	V	D	V
Y	-	I	V	E	A	A	L	Q	T	I	D	S	I	L	N	A	T	T	M	V	D	V
Y	-	I	V	E	A	A	L	Q	T	I	D	S	I	L	N	A	T	T	M	V	D	V
Y	-	I	V	E	A	A	L	Q	T	I	D	S	I	L	N	A	T	T	M	V	D	V
Y	-	I	V	E	A	A	L	Q	T	I	D	S	I	L	N	A	T	T	M	V	D	V
Y	-	I	V	E	A	A	L	Q	T	I	D	S	I	L	N	A	T	T	M	I	D	V
Y	-	I	V	E	A	A	L	Q	T	I	D	S	I	L	N	A	T	T	M	I	D	V
Y	-	I	V	E	A	A	L	Q	T	I	D	S	I	L	N	A	T	T	M	I	D	V
Y	-	I	V	E	A	A	L	Q	T	I	D	S	I	L	N	A	T	T	M	I	D	V
Y	-	I	V	E	A	A	L	Q	T	I	D	S	I	L	N	A	T	T	M	I	D	V
Y	-	I	V	E	A	A	L	Q	T	I	D	S	I	L	N	A	T	T	M	I	D	V
Y	-	I	V	E	A	A	L	Q	T	I	D	S	I	L	N	A	T	T	M	I	D	V
Y	-	I	V	E	A	A	L	Q	T	I	D	S	I	L	N	A	T	T	M	I	D	V
Y	-	I	V	E	A	A	L	Q	T	I	D	S	I	L	N	A	T	T	M	I	D	V
-	Y	V	V	Q	G	A	I	K	S	L	Q	G	L	I	D	T	D	T	M	I	D	V
-	Y	V	V	Q	G	A	I	K	S	L	Q	G	L	I	D	T	D	T	M	I	D	V
-	Y	V	V	Q	G	A	I	K	S	L	Q	G	L	I	D	T	D	T	M	I	D	V
-	Y	V	V	Q	G	A	I	K	S	L	Q	G	L	I	D	T	D	T	M	I	D	V
-	Y	V	V	Q	G	A	I	K	S	L	Q	G	L	I	D	T	D	T	M	I	D	V
-	Y	V	V	Q	G	A	I	K	S	L	Q	G	L	I	D	T	D	T	M	I	D	V
-	Y	V	V	Q	G	A	I	K	S	L	Q	G	L	I	D	T	D	T	M	I	D	V
-	Y	V	V	Q	G	A	I	K	S	L	Q	G	L	I	D	T	D	T	M	I	D	V
-	Y	V	V	Q	G	A	I	K	S	L	Q	G	L	I	D	T	D	T	M	I	D	V
-	Y	V	V	Q	G	A	I	K	S	L	Q	G	L	I	D	T	D	T	M	I	D	V

-	Y	V	V	Q	G	A	I	K	S	L	Q	G	L	I	D	T	D	T	M	I	D	V
-	Y	V	V	Q	G	A	I	K	S	L	Q	G	L	I	D	T	D	T	M	I	D	V
-	Y	V	V	Q	G	A	I	K	S	L	Q	G	L	I	D	T	D	T	M	I	D	V
-	Y	V	V	Q	G	A	I	K	S	L	Q	G	L	I	D	T	D	T	M	I	D	V
-	Y	V	V	Q	G	A	I	K	S	L	Q	G	L	I	D	T	D	T	M	I	D	V
-	Y	V	V	Q	G	A	I	K	S	L	Q	G	L	I	D	T	D	T	M	I	D	V
-	Y	V	V	Q	G	A	I	K	S	L	Q	G	L	I	D	T	D	T	M	I	D	V
-	Y	V	V	Q	G	A	I	K	S	L	Q	G	L	I	D	T	D	T	M	I	D	V
-	Y	V	V	Q	G	A	I	K	S	L	Q	G	L	I	D	T	D	T	M	I	D	V
-	Y	V	V	Q	G	A	I	K	S	L	Q	G	L	I	D	T	D	T	M	I	D	V
-	Y	V	V	Q	G	A	I	K	S	L	Q	G	L	I	D	T	D	T	M	I	D	V
-	Y	V	V	Q	G	A	I	K	S	L	Q	G	L	I	D	T	D	T	M	I	D	V
-	Y	V	V	Q	G	A	I	K	S	L	Q	G	L	I	D	T	D	T	M	I	D	V
-	Y	V	V	Q	G	A	I	K	S	L	Q	G	L	I	D	T	D	T	M	I	D	V
-	Y	V	V	Q	G	A	I	K	S	L	Q	G	L	I	D	T	D	T	M	I	D	V
-	Y	V	V	Q	G	A	I	K	S	L	Q	G	L	I	D	T	D	T	M	I	D	V
-	Y	V	V	Q	G	A	I	K	S	L	Q	G	L	I	D	T	D	T	M	I	D	V
-	Y	V	V	Q	G	A	I	K	S	L	Q	G	L	I	D	T	D	T	M	I	D	V
-	Y	V	V	Q	G	A	I	K	S	L	Q	G	L	I	D	T	D	T	M	I	D	V
-	Y	V	V	Q	G	A	I	K	S	L	Q	G	L	I	D	T	D	T	M	I	D	V
-	Y	V	V	Q	G	A	I	K	S	L	Q	G	L	I	D	T	D	T	M	I	D	V
-	Y	V	V	Q	G	A	I	K	S	L	Q	G	L	I	D	T	D	T	M	I	D	V
-	Y	V	V	Q	G	A	I	K	S	L	Q	G	L	I	D	T	D	T	M	I	D	V
-	Y	V	V	Q	G	V	I	K	S	L	Q	G	L	I	D	T	D	T	M	I	D	V
H	F	V	V	Q	G	A	I	K	S	L	Q	G	L	I	D	T	N	T	M	I	D	V
H	F	V	V	Q	G	A	I	K	S	L	Q	G	L	I	D	T	N	T	M	I	D	V
H	F	V	V	Q	G	A	I	K	S	L	Q	G	L	I	D	T	N	T	M	I	D	V
H	F	V	V	Q	G	A	I	K	S	L	Q	G	L	I	D	T	N	T	M	I	D	V
H	F	V	V	Q	G	A	I	K	S	L	Q	G	L	I	D	T	N	T	M	I	D	V
H	F	V	V	Q	G	A	I	K	S	L	Q	G	L	I	D	T	N	T	M	I	D	V
H	F	V	V	Q	G	A	I	K	S	L	Q	G	L	I	D	T	N	T	M	I	D	V
H	F	V	V	Q	G	A	I	K	S	L	Q	G	L	I	D	T	N	T	M	I	D	V
H	F	V	V	Q	G	A	I	K	S	L	Q	G	L	I	D	T	N	T	M	I	D	V
H	F	V	V	Q	G	A	I	K	S	L	Q	G	L	I	D	T	N	T	M	I	D	V
H	F	V	V	Q	G	A	I	K	S	L	Q	G	L	I	D	T	N	T	M	I	D	V
H	F	V	V	Q	G	A	I	K	S	L	Q	G	L	I	D	T	N	T	M	I	D	V
H	F	V	V	Q	G	A	I	K	S	L	Q	G	L	I	D	T	N	T	M	I	D	V
H	F	V	V	Q	G	A	I	K	S	L	Q	G	L	I	D	T	N	T	M	I	D	V
H	F	V	V	Q	G	A	I	K	S	L	Q	G	L	I	D	T	N	T	M	I	D	V
H	F	V	V	Q	G	A	I	K	S	L	Q	G	L	I	D	T	N	T	M	I	D	V
H	F	V	V	Q	G	A	I	K	S	L	Q	G	L	I	D	T	N	T	M	I	D	V
H	F	V	V	Q	G	A	I	K	S	L	Q	G	L	I	D	T	N	T	M	I	D	V
H	F	V	V	Q	G	A	I	K	S	L	Q	G	L	I	D	T	N	T	M	I	D	V
H	F	V	V	Q	G	A	I	K	S	L	Q	G	L	I	D	T	N	T	M	I	D	V
H	F	V	V	Q	G	A	I	K	S	L	Q	G	L	I	D	T	N	T	M	I	D	V
H	F	V	V	Q	G	A	I	K	S	L	Q	G	L	I	D	T	N	T	M	I	D	V
H	F	V	V	Q	G	A	I	K	S	L	Q	G	L	I	D	T	N	T	M	I	D	V
H	F	V	V	Q	G	A	I	K	S	L	Q	G	L	I	D	T	N	T	M	I	D	V
H	F	V	V	Q	G	A	I	K	S	L	Q	G	L	I	D	T	N	T	M	I	D	V
H	F	V	V	Q	G	A	I	K	S	L	Q	G	L	I	D	T	N	T	M	V	D	V
H	F	V	V	Q	G	A	I	K	S	L	Q	G	L	I	D	T	N	T	M	V	D	V
H	F	V	V	Q	G	A	I	K	S	L	Q	G	L	I	D	T	N	T	M	V	D	V
H	F	V	V	Q	G	A	I	K	S	L	Q	G	L	I	D	T	N	T	M	V	D	V
H	F	V	V	Q	G	A	I	K	S	L	Q	G	L	I	D	T	N	T	M	V	D	V
H	F	V	V	Q	G	A	I	K	S	L	Q	G	L	I	D	T	N	T	M	V	D	V
H	F	V	V	Q	G	A	I	K	S	L	Q	G	L	I	D	T	N	T	M	V	D	V
H	F	V	V	Q	G	A	I	K	S	L	Q	G	L	I	D	T	N	T	M	V	D	V
H	F	V	V	Q	G	A	I	K	S	L	Q	G	L	I	D	T	N	T	M	V	D	V
H	F	V	V	Q	G	A	I	K	S	L	Q	G	L	I	D	T	N	T	M	V	D	V
H	F	V	V	Q	G	A	I	K	S	L	Q	G	L	I	D	T	N	T	M	V	D	V
H	F	V	V	Q	G	A	I	K	S	L	Q	G	L	I	D	T	N	T	M	V	D	V
H	F	V	V	Q	G	A	I	K	S	L	Q	G	L	I	D	T	N	T	M	V	D	V
H	F	V	V	Q	G	A	I	K	S	L	Q	G	L	I	D	T	N	T	M	V	D	V
H	F	V	V	Q	G	A	I	K	S	L	Q	G	L	I	D	T	N	T	M	V	D	V
H	F	V	V	Q	G	A	I	K	S	L	Q	G	L	I	D	T	N	T	M	V	D	V
H	F	V	V	Q	G	A	I	K	S	L	Q	G	L	I	D	T	N	T	M	V	D	V
H	F	V	V	Q	G	A	I	K	S	L	Q	G	L	I	D	T	N	T	M	V	D	V

H	F	V	V	Q	G	A	I	K	S	L	Q	G	L	I	D	T	N	T	M	V	D	V
H	F	V	V	Q	G	A	I	K	S	L	Q	G	L	I	D	T	N	T	M	V	D	V
H	F	V	V	Q	G	A	I	K	S	L	Q	G	L	I	D	T	N	T	M	V	D	V
H	F	V	V	Q	G	A	I	K	S	L	Q	G	L	I	D	T	N	T	M	V	D	V
H	F	V	V	Q	G	A	I	K	S	L	Q	G	L	I	D	T	N	T	M	V	D	V
H	F	V	V	Q	G	A	I	K	S	L	Q	G	L	I	D	T	N	T	M	V	D	V
H	F	V	V	Q	G	A	I	K	S	L	Q	G	L	I	D	T	N	T	M	V	D	V
H	F	V	V	Q	G	A	I	K	S	L	Q	G	L	I	D	T	N	T	M	V	D	V
H	F	V	V	Q	G	A	I	K	S	L	Q	G	L	I	D	T	N	T	M	V	D	V
H	F	V	V	Q	G	A	I	K	S	L	Q	G	L	I	D	T	N	T	M	V	D	V

388	401	403	404	405	410	416	421	425	432	443	446	464	470	473	479
K	S	L	Q	G	L	I	D	T	N	T	M	D	V	A	M
Q	T	I	D	S	I	L	N	A	T	R	T	N	S	T	L
K	S	L	Q	G	L	I	D	T	D	T	M	D	V	A	M
Q	T	I	D	S	I	L	N	A	T	R	T	N	S	T	L
Q	T	I	D	S	I	L	N	A	T	R	T	N	S	T	L
Q	T	I	D	S	I	L	N	A	T	R	T	N	S	T	L
Q	T	I	D	S	I	L	N	A	T	R	T	N	S	T	L
Q	T	I	D	S	I	L	N	A	T	R	T	N	S	T	L
Q	T	I	D	S	I	L	N	A	T	R	T	N	S	T	L
Q	T	I	D	S	I	L	N	A	T	R	T	N	S	T	L
Q	T	I	D	S	I	L	N	A	T	R	T	N	S	T	L

27	34	92	93	94	101
A	V	G	K	K	D
T	V	E	N	N	N
A	V	G	K	K	D
T	G	E	N	N	N
T	V	E	N	N	N
T	V	E	N	N	N
T	V	E	N	N	N
T	V	E	N	N	N
T	V	E	N	N	N
T	V	E	N	N	N
T	V	E	N	N	N

402	403	404	409	415	420	424	431	442	445	462	463	469	472	478
L	Q	G	L	I	D	T	N	T	M	I	D	V	A	M
I	D	S	I	L	N	A	T	R	T	I	N	S	T	L
I	D	S	I	L	N	A	T	T	M	V	D	V	A	M
I	D	S	I	L	N	A	T	T	M	V	D	V	A	M
I	D	S	I	L	N	A	T	T	M	V	D	V	A	M
I	D	S	I	L	N	A	T	T	M	V	D	V	A	M
I	D	S	I	L	N	A	T	T	M	V	D	V	A	M
I	D	S	I	L	N	A	T	T	M	V	D	V	A	M
I	D	S	I	L	N	A	T	T	M	V	D	V	A	M
I	D	S	I	L	N	A	T	T	M	V	D	V	A	M
I	D	S	I	L	N	A	T	T	M	V	D	V	A	M
I	D	S	I	L	N	A	T	T	M	V	D	V	A	M
I	D	S	I	L	N	A	T	T	M	V	D	V	A	M
I	D	S	I	L	N	A	T	T	M	V	D	V	A	M
I	D	S	I	L	N	A	T	T	M	V	D	V	A	M
I	D	S	I	L	N	A	T	T	M	V	D	V	A	M
I	D	S	I	L	N	A	T	T	M	V	D	V	A	M
I	D	S	I	L	N	A	T	T	M	V	D	V	A	M
I	D	S	I	L	N	A	T	T	M	V	D	V	A	M
I	D	S	I	L	N	A	T	T	M	V	D	V	A	M
I	D	S	I	L	N	A	T	T	M	V	D	V	A	M
I	D	S	I	L	N	A	T	T	M	I	D	V	A	M
I	D	S	I	L	N	A	T	T	M	I	D	V	A	M
I	D	S	I	L	N	A	T	T	M	I	D	V	A	M
I	D	S	I	L	N	A	T	T	M	I	D	V	A	M
L	Q	G	L	I	D	T	N	T	M	V	D	V	A	M
I	D	S	I	L	N	A	T	T	M	I	D	V	A	M
I	D	S	I	L	N	A	T	T	M	I	D	V	A	M
I	D	S	I	L	N	A	T	T	M	I	D	V	A	M
I	D	S	I	L	N	A	T	T	M	I	D	V	A	M
I	D	S	I	L	N	A	T	T	M	I	D	V	A	M

27	74	92	93	94	101	123
A	V	G	K	K	D	S
T	V	E	N	N	N	N
A	I	G	K	K	D	S
A	I	G	K	K	D	S
A	I	G	K	K	D	S
A	I	G	K	K	D	S
A	I	G	K	K	D	S
A	I	G	K	K	D	S
A	I	G	K	K	D	S
A	I	G	K	K	D	S
A	I	G	K	K	D	S
A	I	G	K	K	D	S
A	I	G	K	K	D	S
A	I	G	K	K	D	S
A	I	G	K	K	D	S
A	I	G	K	K	D	S
A	I	G	K	K	D	S
A	I	G	K	K	D	S
A	I	G	K	K	D	S
A	I	G	K	K	D	S
A	I	G	K	K	D	S
A	V	E	K	K	D	S
A	V	E	K	K	D	S
A	V	E	K	K	D	S
A	V	E	K	K	D	S
A	V	G	K	K	D	S
A	V	E	K	K	D	S
A	V	E	K	K	D	S
A	V	E	K	K	D	S
A	V	E	K	K	D	S
A	V	E	K	K	D	S

wchA										wciN	HG262	wciO						
149	175	178	204	352	367	430	431	449	451			5	6	23	39	136	205	62
R	C	V	E	R	N	N	G	F	R			R	I	A	L	N	T	F
G	Y	I	K	C	N	D	D	L	K			K	L	P	L	T	T	L
R	C	V	E	R	D	N	G	F	R			R	I	A	L	N	T	F
R	C	V	E	R	N	N	G	F	R			R	I	A	L	N	A	F
R	C	V	E	R	N	N	G	F	R			R	I	A	L	N	T	F
R	C	V	E	R	N	N	G	F	R			R	I	A	L	N	T	F
R	C	V	E	R	N	N	G	F	R			R	I	A	L	N	T	F
R	C	V	E	R	N	N	G	F	R			R	I	A	L	N	A	F
R	C	V	E	R	N	N	G	F	R			R	I	A	L	N	T	F
R	C	V	E	R	N	N	G	F	R			R	I	A	L	N	A	F
R	C	V	E	R	N	N	G	F	R			R	I	A	L	N	T	F
R	C	V	E	R	N	N	G	F	R			R	I	A	L	N	A	F
R	C	V	E	R	N	N	G	F	R			R	I	A	L	N	A	F
R	C	V	E	R	N	N	G	F	R			R	I	A	L	N	T	F
R	C	V	E	R	N	N	G	F	R			R	I	A	L	N	T	F
R	C	V	E	R	N	N	G	F	R			R	I	A	L	N	A	F
R	C	V	E	R	N	N	G	F	R			R	I	A	L	N	T	F
R	C	V	E	R	N	N	G	F	R			R	I	A	L	N	T	F
R	C	V	E	R	N	N	G	F	R			R	I	A	L	N	A	F
R	C	V	E	R	N	N	G	F	R			R	I	A	L	N	A	F
R	C	V	E	R	N	N	G	F	R			R	I	A	L	N	T	F
R	C	V	E	R	N	N	G	F	R			R	I	A	L	N	A	F
R	C	V	E	R	N	N	G	F	R			R	I	A	L	N	T	F
R	C	V	E	R	N	N	G	F	R			R	I	A	L	N	A	F
R	C	V	E	R	N	N	G	F	R			R	I	A	L	N	T	F
R	C	V	E	R	N	N	G	F	R			R	I	A	L	N	A	F
R	C	V	E	R	N	N	G	F	R			R	I	A	L	N	A	F
R	C	V	E	R	N	N	G	F	R			R	I	A	L	N	A	F
R	C	V	E	R	N	N	G	F	R			R	I	A	L	N	A	F
R	C	V	E	R	N	N	G	F	R			R	I	A	L	N	T	F
R	C	V	E	R	N	N	G	F	R			R	I	A	L	N	T	F
R	C	V	E	R	N	N	G	F	R			R	I	A	L	N	A	F
R	C	V	E	R	N	N	G	F	R			R	I	A	L	N	A	F
R	C	V	E	R	N	N	G	F	R			R	I	A	L	N	T	F
R	C	V	E	R	N	N	G	F	R			R	I	A	L	N	A	F

410	416	421	425	432	443	446	460	464	470	473	479
L	I	D	T	N	T	M	V	D	V	A	M
L	I	D	T	D	T	M	V	D	V	A	M
I	L	N	A	T	R	T	M	N	S	T	L
L	I	D	T	D	T	M	V	D	V	A	M

wzh									
27	89	92	94	101	123	155	170	176	190
A	D	G	K	D	S	D	P	R	Q
A	D	G	K	D	S	D	P	R	Q
T	G	E	N	N	I	N	S	P	R
A	D	G	K	D	S	D	P	R	Q

402	403	404	409	415	420	424	431	442	445	463	469	472	478
L	Q	G	L	I	D	T	N	T	M	D	V	A	M
I	D	S	I	L	N	A	T	R	T	N	S	T	L
L	Q	G	L	I	D	T	N	T	M	D	V	A	M
L	Q	G	L	I	D	T	N	T	M	D	V	A	M
L	Q	G	L	I	D	T	N	T	M	D	V	A	M
L	Q	G	L	I	D	T	N	T	M	D	V	A	M
L	Q	G	L	I	D	T	N	T	M	D	V	A	M
L	Q	G	L	I	D	T	N	T	M	D	V	A	M
L	Q	G	L	I	D	T	N	T	M	D	V	A	M
L	Q	G	L	I	D	T	N	T	M	D	V	A	M
L	Q	G	L	I	D	T	N	T	M	D	V	A	M
L	Q	G	L	I	D	T	N	T	M	D	V	A	M
L	Q	G	L	I	D	T	N	T	M	D	V	A	M
L	Q	G	L	I	D	T	N	T	M	D	V	A	M
L	Q	G	L	I	D	T	N	T	M	D	V	A	M
L	Q	G	L	I	D	T	N	T	M	D	V	A	M
L	Q	G	L	I	D	T	N	T	M	D	V	A	M
L	Q	G	L	I	D	T	N	T	M	D	V	A	M
L	Q	G	L	I	D	T	N	T	M	D	V	A	M
L	Q	G	L	I	D	T	N	T	M	D	V	A	M
L	Q	G	L	I	D	T	N	T	M	D	V	A	M
L	Q	G	L	I	D	T	N	T	M	D	V	A	M
L	Q	G	L	I	D	T	N	T	M	D	V	A	M
L	Q	G	L	I	D	T	N	T	M	D	V	A	M
L	Q	G	L	I	D	T	N	T	M	D	V	A	M
L	Q	G	L	I	D	T	N	T	M	D	V	A	M
L	Q	G	L	I	D	T	N	T	M	D	V	A	M

27	92	93	94	101	123	142	162
A	G	K	K	D	S	A	V
T	E	N	N	N	N	V	I
A	G	K	K	D	N	A	V
A	G	K	K	D	N	A	V
A	G	K	K	D	N	A	V
A	G	K	K	D	N	A	V
A	G	K	K	D	N	A	V
A	G	K	K	D	N	A	V
A	G	K	K	D	N	A	V
A	G	K	K	D	N	A	V
A	G	K	K	D	N	A	V
A	G	K	K	D	N	A	V
A	G	K	K	D	N	A	V
A	G	K	K	D	N	A	V
A	G	K	K	D	N	A	V
A	G	K	K	D	N	A	V
A	G	K	K	D	N	A	V
A	G	K	K	D	N	A	V
A	G	K	K	D	N	A	V
A	G	K	K	D	N	A	V
A	G	K	K	D	N	A	V
A	G	K	K	D	N	A	V
A	G	K	K	D	N	A	V
A	G	K	K	D	N	A	V
A	G	K	K	D	N	A	V
A	G	K	K	D	N	A	V
A	G	K	K	D	N	A	V

479
M
M
L
M
M
M
M
M
M
M
M
M
M
M
M
M
M
M
M
M
M
M
M
M
M
M
M
M

wzh											
27	92	94	101	123	170	190	191	194	203	205	218
A	G	K	D	S	P	Q	N	Y	L	G	T
A	G	K	D	S	P	Q	D	H	L	G	T
T	E	N	N	I	S	R	D	H	V	S	S
A	G	K	D	S	P	Q	D	Y	L	G	T
A	G	K	D	S	P	Q	D	Y	L	G	T
A	G	K	D	S	P	Q	D	Y	L	G	T
A	G	K	D	S	P	Q	D	Y	L	G	T
A	G	K	D	S	P	Q	D	Y	L	G	T
A	G	K	D	S	P	Q	D	Y	L	G	T
A	G	K	D	S	P	Q	D	Y	L	G	T
A	G	K	D	S	P	Q	D	Y	L	G	T
A	G	K	D	S	P	Q	D	Y	L	G	T
A	G	K	D	S	P	Q	D	Y	L	G	T
A	G	K	D	S	P	Q	D	Y	L	G	T
A	G	K	D	S	P	Q	D	Y	L	G	T
A	G	K	D	S	P	Q	D	Y	L	G	T
A	G	K	D	S	P	Q	D	Y	L	G	T
A	G	K	D	S	P	Q	D	Y	L	G	T
A	G	K	D	S	P	Q	D	Y	L	G	T
A	G	K	D	S	P	Q	D	Y	L	G	T
A	G	K	D	S	P	Q	D	Y	L	G	T
A	G	K	D	S	P	Q	D	Y	L	G	T
A	G	K	D	S	P	Q	D	Y	L	G	T
A	G	K	D	S	P	Q	D	Y	L	G	T
A	G	K	D	S	P	Q	D	Y	L	G	T
A	G	K	D	S	P	Q	D	Y	L	G	T
A	G	K	D	S	P	Q	D	Y	L	G	T
A	G	K	D	S	P	Q	D	Y	L	G	T
A	G	K	D	S	P	Q	D	Y	L	G	T

14	15	20	21	24	33	91	103
L	F	Q	R	M	S	A	M
L	F	Q	R	M	S	A	M
S	L	K	Y	I	G	V	I
L	F	Q	R	M	S	A	M
L	F	Q	R	M	S	A	M
L	F	Q	R	M	S	A	M
L	F	Q	R	M	S	A	M
L	F	Q	R	M	S	A	M
L	F	Q	R	M	S	A	M
L	F	Q	R	M	S	A	M
L	F	Q	R	M	S	A	M
L	F	Q	R	M	S	A	M
L	F	Q	R	M	S	A	M
L	F	Q	R	M	S	A	M
L	F	Q	R	M	S	A	M
L	F	Q	R	M	S	A	M
L	F	Q	R	M	S	A	M
L	F	Q	R	M	S	A	M
L	F	Q	R	M	S	A	M
L	F	Q	R	M	S	A	M
L	F	Q	R	M	S	A	M
L	F	Q	R	M	S	A	M
L	F	Q	R	M	S	A	M
L	F	Q	R	M	S	A	M
L	F	Q	R	M	S	A	M
L	F	Q	R	M	S	A	M
L	F	Q	R	M	S	A	M
L	F	Q	R	M	S	A	M
L	F	Q	R	M	S	A	M

M	A	G	K	D	S	P	Q	D	Y	L	G	T	L	F	Q	R	M	S	A	M
M	A	G	K	D	S	P	Q	D	Y	L	G	T	L	F	Q	R	M	S	A	M

wze											wchA										
45	52	59	124	136	153	158	163	168	209	215	31	45	66	107	124	138	143	175	233	255	359
I	T	A	P	S	T	V	A	K	D	Y	T	H	E	A	A	T	V	C	A	K	L
T	T	A	P	S	T	V	A	N	N	Y	A	H	E	A	A	T	I	Y	A	K	L
T	T	A	P	S	T	V	A	N	N	Y	A	H	E	A	A	T	I	Y	A	K	L
T	T	A	P	S	T	V	A	N	N	Y	A	H	E	A	A	T	I	Y	A	K	L
T	T	A	P	S	T	V	A	N	N	Y	A	H	E	A	A	T	I	Y	A	K	L
T	T	A	P	S	T	V	A	N	N	Y	A	H	E	A	A	T	I	Y	A	K	L
T	T	A	P	S	T	V	A	N	N	Y	A	H	E	A	A	T	I	Y	A	K	L
T	T	A	P	S	T	V	A	N	N	Y	A	H	E	A	A	T	I	Y	A	K	L
T	T	A	P	S	T	V	A	N	N	Y	A	H	E	A	A	T	I	Y	A	K	L
T	T	A	P	S	T	V	A	N	N	Y	A	H	E	A	A	T	I	Y	A	K	L
T	T	A	P	S	T	V	A	N	N	Y	A	H	E	A	A	T	I	Y	A	K	L
T	T	A	P	S	T	V	A	N	N	Y	A	H	E	A	A	T	I	Y	A	K	L
T	T	A	P	S	T	V	A	N	N	Y	A	H	E	A	A	T	I	Y	A	K	L
T	T	A	P	S	T	V	A	N	N	Y	A	H	E	A	A	T	I	Y	A	K	L
T	T	A	P	T	T	V	A	N	N	Y	A	H	E	A	A	T	I	Y	A	K	L
T	T	A	P	T	T	V	A	N	N	Y	A	H	E	A	A	T	I	Y	A	K	L
T	T	A	P	T	T	V	A	N	N	Y	A	H	E	A	A	T	I	Y	A	K	L
T	T	A	P	T	T	V	A	N	N	Y	A	H	E	A	A	T	I	Y	A	K	L
T	T	A	P	T	T	V	A	N	N	Y	A	H	E	A	A	T	I	Y	A	K	L
T	T	A	P	T	T	V	A	N	N	Y	A	H	E	A	A	T	I	Y	A	K	L
T	T	A	P	T	T	V	A	N	N	Y	A	H	E	A	A	T	I	Y	A	K	L
T	T	A	P	T	T	V	A	N	N	Y	A	H	E	A	A	T	I	Y	A	K	L
T	T	A	P	T	T	V	A	N	N	Y	A	H	E	A	A	T	I	Y	A	K	L
T	T	A	P	T	T	V	A	N	N	Y	A	H	E	A	A	T	I	Y	A	K	L
T	T	T	P	T	T	V	A	N	N	Y	A	H	E	A	A	T	I	Y	A	K	L
T	T	A	P	T	T	V	A	N	N	Y	A	H	E	A	A	T	I	Y	A	K	L
T	T	A	P	T	T	V	A	N	N	Y	A	H	E	A	A	T	I	Y	A	K	L
T	T	A	P	T	T	V	A	N	N	Y	A	H	E	A	A	T	I	Y	A	K	L
T	T	A	P	T	T	V	A	N	N	Y	A	H	E	A	A	T	I	Y	A	K	L
T	T	A	P	T	T	V	A	N	N	Y	A	H	E	A	A	T	I	Y	A	K	L
T	T	A	P	T	T	V	A	N	N	Y	A	H	E	A	A	T	I	Y	A	R	L
T	T	A	P	S	T	V	A	N	N	Y	A	H	E	A	A	T	I	Y	A	K	L
T	T	A	P	S	T	V	A	N	N	Y	A	H	E	A	A	T	I	Y	A	K	L
T	T	A	P	S	T	V	A	N	N	Y	A	H	E	A	A	T	I	Y	A	K	L
T	T	A	P	S	A	V	A	N	N	Y	A	H	E	A	A	T	I	Y	A	K	L
T	T	A	P	S	T	V	A	N	N	Y	A	H	E	A	A	T	I	Y	A	K	L
T	T	A	P	S	T	V	A	N	N	Y	A	H	E	A	A	T	I	Y	A	K	L
T	T	A	P	S	T	V	A	N	N	Y	A	H	E	A	A	T	I	Y	A	K	L
T	T	A	P	S	T	V	A	N	N	Y	A	H	E	A	A	T	I	Y	A	K	L
T	T	A	P	S	T	V	A	N	N	Y	A	H	E	A	A	T	I	Y	A	K	L
T	T	A	P	S	T	V	A	N	N	Y	A	H	E	A	A	T	I	Y	A	K	L
T	T	A	P	S	T	V	A	N	N	Y	A	H	E	A	A	T	I	Y	A	K	L
T	T	A	P	S	T	V	A	N	N	Y	A	H	E	A	A	T	I	Y	A	K	L
T	T	A	P	S	T	V	A	N	N	Y	A	H	E	A	A	T	I	Y	A	K	L
T	T	A	P	S	T	V	A	N	N	Y	A	H	E	A	A	T	I	Y	A	K	L
T	T	A	P	S	T	V	A	N	N	Y	A	H	E	A	A	T	I	Y	A	K	L
T	T	A	P	S	T	V	A	N	N	Y	A	H	E	A	A	T	I	Y	A	K	F
T	T	A	P	S	T	V	A	N	N	Y	A	H	E	A	A	T	I	Y	A	K	L
T	T	A	P	S	T	V	A	N	N	Y	A	H	E	A	A	T	I	Y	A	K	L
T	T	A	P	S	T	V	A	N	N	Y	A	H	E	A	A	T	I	Y	A	K	L
T	T	A	P	S	T	V	A	N	N	Y	A	H	E	A	A	T	I	Y	A	K	L
T	T	A	P	S	T	V	A	N	N	Y	A	H	E	A	A	T	I	Y	A	K	L
T	T	A	P	S	T	V	A	N	N	Y	A	H	E	A	A	T	I	Y	A	K	L
T	T	A	P	S	T	V	A	N	N	Y	A	H	E	A	A	T	I	Y	A	K	L
T	T	A	P	S	T	V	A	N	N	Y	A	H	E	A	A	T	I	Y	A	K	L
T	T	A	P	S	T	V	A	N	N	Y	A	H	E	A	A	T	I	Y	A	K	L

T	T	A	P	S	T	V	A	N	N	Y	A	H	E	A	A	T	I	Y	A	K	L
T	T	A	P	S	T	V	A	N	N	Y	A	H	E	A	A	T	I	Y	A	K	L
T	T	A	P	S	T	V	A	N	N	Y	A	H	E	A	A	T	I	Y	A	K	L
T	T	A	P	S	T	V	A	N	N	Y	A	H	E	A	A	T	I	Y	A	K	L
T	T	A	P	S	T	V	A	N	N	Y	A	H	E	A	A	T	I	Y	A	K	L
T	T	A	P	S	T	V	A	N	N	Y	A	H	E	A	A	T	I	Y	A	K	L
T	T	A	P	S	T	V	A	N	N	Y	A	H	E	A	A	T	I	Y	A	K	L
T	T	A	P	S	T	V	A	N	N	Y	A	H	E	A	A	T	I	Y	A	K	L
T	T	A	P	S	T	V	A	N	N	Y	A	H	E	A	A	T	I	Y	A	K	L
T	T	A	P	S	T	V	A	N	N	Y	A	H	E	A	A	T	I	Y	A	K	L
T	T	A	P	S	T	V	A	N	N	Y	A	H	E	A	A	T	I	Y	A	K	L
T	T	A	P	S	T	V	A	N	N	Y	A	H	E	A	A	T	I	Y	A	K	L
T	T	A	P	S	T	V	A	N	N	Y	A	H	E	A	A	T	I	Y	A	K	L
T	T	A	P	S	T	V	A	N	N	Y	A	H	E	A	A	T	I	Y	A	K	L
T	T	A	P	S	T	V	A	N	N	Y	A	H	E	A	A	T	I	Y	A	K	L
T	T	A	P	S	T	V	A	N	N	Y	A	H	E	A	A	T	I	Y	A	K	L
T	T	A	P	S	T	V	A	N	N	Y	A	H	E	A	A	T	I	Y	A	K	L
T	T	A	P	S	T	V	A	N	N	Y	A	H	E	A	A	T	I	Y	A	K	L
T	T	A	P	S	T	V	A	N	N	Y	A	H	E	A	A	T	I	Y	A	K	L
T	T	A	P	S	T	V	A	N	N	Y	A	H	E	A	A	T	I	Y	A	K	L
T	T	A	P	S	T	V	A	N	N	Y	A	H	E	A	A	T	I	Y	A	K	L
T	T	A	P	S	T	V	A	N	N	Y	A	H	E	A	A	T	I	Y	A	K	L
T	T	A	P	S	T	V	A	N	N	Y	A	H	E	A	A	T	I	Y	A	K	L
T	T	A	P	S	T	V	A	N	N	Y	A	H	E	A	A	T	I	Y	A	K	L
T	T	A	P	S	T	V	A	N	N	Y	A	H	E	A	A	T	I	Y	A	K	L
T	T	A	P	S	T	V	A	N	N	Y	A	H	E	A	A	T	I	Y	A	K	L
T	T	A	P	S	T	V	A	N	N	Y	A	H	E	A	A	T	I	Y	A	K	L
T	T	A	P	S	T	V	A	N	N	Y	A	H	E	A	A	T	I	Y	A	K	L
T	T	A	P	S	T	V	A	N	N	Y	A	H	E	A	A	T	I	Y	A	K	L
T	T	A	P	S	T	V	A	N	N	Y	A	H	E	A	A	T	I	Y	A	K	L
T	T	A	P	S	T	V	A	N	N	Y	A	H	E	A	A	T	I	Y	A	K	L
T	T	A	P	S	T	V	A	N	N	Y	A	H	E	A	A	T	I	Y	A	K	L
T	T	A	P	S	T	V	A	N	N	Y	A	H	E	A	A	T	I	Y	A	K	L
T	T	A	P	S	T	V	A	N	N	Y	A	H	E	A	A	T	I	Y	A	K	L
T	T	A	P	S	T	V	A	N	N	Y	A	H	E	A	A	T	I	Y	A	K	L
T	T	A	P	S	T	V	A	N	N	Y	A	H	E	A	A	T	I	Y	A	K	L
T	T	A	P	S	T	V	A	N	N	Y	A	H	E	A	A	T	I	Y	A	K	L
T	T	A	P	S	T	V	A	N	N	Y	A	H	E	A	A	T	I	Y	A	K	L
T	T	A	P	S	T	V	A	N	N	Y	A	H	E	A	A	T	I	Y	A	K	L
T	T	A	P	S	T	V	A	N	N	Y	A	H	E	A	A	T	I	Y	A	K	L
T	T	A	P	S	T	V	A	N	N	Y	A	H	E	A	A	T	I	Y	A	K	L
T	T	A	P	S	T	V	A	N	N	Y	A	H	E	A	A	T	I	Y	A	K	L
T	T	A	P	S	T	V	A	N	N	Y	A	H	E	A	A	T	I	Y	A	K	L
T	T	A	P	S	T	V	A	N	N	Y	A	H	E	A	A	T	I	Y	A	K	L
T	T	A	P	S	T	V	A	N	N	Y	A	H	E	A	A	T	I	Y	A	K	L
T	T	A	P	S	T	V	A	N	N	Y	A	H	E	A	A	T	I	Y	A	K	L
T	T	A	P	S	T	V	A	N	N	Y	A	H	E	A	A	T	I	Y	A	K	L
T	T	A	P	S	T	V	A	N	N	Y	A	H	E	A	V	T	I	Y	A	K	L
T	T	A	P	S	T	V	A	N	N	Y	A	H	E	A	A	T	I	Y	A	K	L
T	T	A	P	S	T	V	A	N	N	Y	A	Y	E	A	A	T	I	Y	A	K	L
I	T	A	P	S	T	V	A	K	D	Y	T	H	G	A	A	T	V	C	A	K	L
I	T	A	P	S	T	V	A	K	D	Y	T	H	G	A	S	T	V	C	A	K	L
I	T	A	P	S	T	V	A	K	D	Y	T	H	G	A	A	T	V	C	A	K	L
I	T	A	P	S	T	V	A	K	D	Y	T	H	G	A	S	T	V	C	A	K	L
I	T	A	P	S	T	V	A	K	D	Y	T	H	G	A	S	T	V	C	A	K	L
I	T	A	P	S	T	V	A	K	D	Y	T	H	G	A	A	T	V	C	A	K	L
I	T	A	P	S	T	V	A	K	D	Y	T	H	G	A	A	T	V	C	A	K	L
I	T	A	P	S	T	V	A	K	D	Y	T	H	G	A	A	T	V	C	A	K	L
I	T	A	P	S	T	V	A	K	D	Y	T	H	G	A	A	T	V	C	A	K	L
I	T	A	P	S	T	V	A	K	D	Y	T	H	G	A	A	T	V	C	A	K	L
I	T	A	P	S	T	V	A	K	D	Y	T	H	G	A	A	T	V	C	A	K	L
I	T	A	P	S	T	V	A	K	D	Y	T	H	G	A	S	T	V	C	A	K	L
I	T	A	P	S	T	V	A	K	D	Y	T	H	G	A	A	T	V	C	A	K	L
I	T	A	P	S	T	V	A	K	D	Y	T	H	G	A	S	T	V	C	A	K	L
I	T	A	P	S	T	V	A	K	D	Y	T	H	G	A	A	T	V	C	A	K	L
I	T	A	P	S	T	V	A	K	D	Y	T	H	G	A	A	T	V	C	A	K	L
I	T	A	P	S	T	V	A	K	D	Y	T	H	G	A	A	T	V	C	A	K	L

I	T	A	P	S	T	V	A	K	D	Y	T	H	G	A	A	T	V	C	A	K	L
I	T	A	P	S	T	V	A	K	D	Y	T	H	G	A	A	T	V	C	A	K	L
I	T	A	P	S	T	V	A	K	D	Y	T	H	G	A	A	T	V	C	A	K	L
I	T	A	P	S	T	V	A	K	D	Y	T	H	G	A	A	T	V	C	A	K	L
I	T	A	P	S	T	V	A	K	D	Y	T	H	G	A	S	T	V	C	A	K	L
I	T	A	P	S	T	V	A	K	D	Y	T	H	G	A	A	T	V	C	A	K	L
I	T	A	P	S	T	V	A	K	D	Y	T	H	G	A	A	T	V	C	A	K	L
I	T	A	P	S	T	V	A	K	D	Y	T	H	G	A	S	T	V	C	A	K	L
I	T	A	P	S	T	V	A	K	D	Y	T	H	G	A	A	T	V	C	A	K	L
T	T	A	L	T	T	V	A	K	N	Y	A	H	E	A	A	A	V	C	A	K	L
T	T	V	P	T	T	V	A	K	N	Y	A	H	E	A	A	A	V	C	A	K	L
T	T	A	P	T	T	V	A	K	N	Y	A	H	E	A	A	A	V	C	A	K	L
T	T	A	P	T	T	V	A	K	N	Y	A	H	E	A	A	T	V	C	A	K	L
T	T	A	P	T	T	V	A	K	N	Y	A	H	E	A	A	T	V	C	A	K	L
T	T	A	P	T	T	V	A	K	N	Y	A	H	E	A	A	T	V	C	A	K	L
T	T	A	P	T	T	V	A	K	N	Y	A	H	E	A	A	T	V	C	A	K	L
T	T	A	P	T	T	V	A	K	N	Y	A	H	E	A	A	T	V	C	A	K	L
T	T	A	P	T	T	V	A	K	N	Y	A	H	E	A	A	T	V	C	A	K	L
T	T	A	P	T	T	V	A	K	N	Y	A	H	E	A	A	T	V	C	A	K	L
T	T	A	P	T	T	V	A	K	N	Y	A	H	E	A	A	T	V	C	A	K	L
T	T	A	P	T	T	V	A	K	N	Y	A	H	E	A	A	T	V	C	A	K	L
T	T	A	P	S	T	V	A	K	N	Y	A	H	E	T	A	T	I	Y	A	K	L
T	T	A	P	S	T	V	A	K	N	Y	A	H	E	T	A	T	I	Y	A	K	L
T	T	A	P	S	T	V	A	K	N	Y	A	H	E	T	A	T	I	Y	A	K	L
T	T	A	P	S	T	V	A	K	N	Y	A	H	E	T	A	T	I	Y	A	K	L
T	T	A	P	S	T	V	A	K	N	Y	A	H	E	T	A	T	I	Y	A	K	L
T	T	A	P	S	T	V	A	K	N	Y	A	H	E	T	A	T	I	Y	A	K	L
T	T	A	P	S	T	V	A	K	N	Y	A	H	E	T	A	T	I	Y	A	K	L
T	T	A	P	S	T	V	A	K	N	Y	A	H	E	T	A	T	I	Y	A	K	L
T	T	A	P	S	T	V	A	K	N	Y	A	H	E	T	A	T	I	Y	A	K	L
T	T	A	P	S	T	V	A	K	N	Y	A	H	E	T	A	T	I	Y	A	K	L
T	T	A	P	S	T	V	A	K	N	Y	A	H	E	T	A	T	I	Y	A	K	L
T	T	A	P	S	T	V	A	K	N	Y	A	H	E	T	A	T	I	Y	A	K	L
T	T	A	P	S	T	V	A	K	N	Y	A	H	E	T	A	T	I	Y	A	K	L
T	T	A	P	S	T	V	A	K	N	Y	A	H	E	T	A	T	I	Y	A	K	L
T	T	A	P	S	T	V	A	K	N	Y	A	H	E	T	A	T	I	Y	A	K	L
T	T	A	P	S	T	V	A	K	N	Y	A	H	E	T	A	T	I	Y	A	K	L
T	T	A	P	S	T	V	A	K	N	Y	A	H	E	T	A	T	I	Y	A	K	L
T	T	A	P	S	T	V	A	K	N	Y	A	H	E	T	A	T	I	Y	A	K	L
T	T	A	P	S	T	V	A	K	N	Y	A	H	E	T	A	T	I	Y	A	K	L
T	T	A	P	S	T	V	A	K	N	Y	A	H	E	T	A	T	I	Y	A	K	L
T	T	A	P	S	T	V	A	K	N	Y	A	H	E	T	A	T	I	Y	A	K	L
T	T	A	P	S	T	V	A	K	N	Y	A	H	E	T	A	T	I	Y	A	K	L
T	T	A	P	S	T	V	A	K	N	Y	A	H	E	T	A	T	I	Y	A	K	L
T	T	A	P	S	T	V	A	K	N	Y	A	H	E	T	A	T	I	Y	A	K	L
T	T	A	P	S	T	V	A	K	N	Y	A	H	E	T	A	T	I	Y	A	K	L
T	T	A	P	S	T	V	A	K	N	Y	A	H	E	T	A	T	I	Y	A	K	L
T	T	A	P	S	T	V	A	K	N	Y	A	H	E	T	A	T	I	Y	A	K	L
I	T	A	P	S	T	V	A	K	D	C	T	H	E	A	A	T	V	C	A	K	L
I	T	A	P	S	T	V	V	K	D	Y	T	H	E	A	A	T	V	C	A	K	L
I	N	A	P	S	T	V	A	K	D	Y	T	H	E	A	A	T	V	C	A	K	L
I	T	A	P	S	T	V	V	K	D	Y	T	H	E	A	A	T	V	C	A	K	L
I	N	A	P	S	T	V	A	K	D	Y	T	H	E	A	A	T	V	C	A	K	L
I	T	A	P	S	T	V	V	K	D	Y	T	H	E	A	A	T	V	C	A	K	L
I	T	A	P	S	T	V	V	K	D	Y	T	H	E	A	A	T	V	C	A	K	L
I	N	A	P	S	T	V	A	K	D	Y	T	H	E	A	A	T	V	C	A	K	L
I	T	A	P	S	T	V	V	K	D	Y	T	H	E	A	A	T	V	C	A	K	L
I	N	A	P	S	T	V	A	K	D	Y	T	H	E	A	A	T	V	C	A	K	L
I	N	A	P	S	T	V	A	K	D	Y	T	H	E	A	A	T	V	C	A	K	L
I	N	A	P	S	T	V	A	K	D	Y	T	H	E	A	A	T	V	C	A	K	L
I	N	A	P	S	T	V	A	K	D	Y	T	H	E	A	A	T	V	C	A	K	L
I	T	A	P	S	T	V	V	K	D	Y	T	H	E	A	A	T	V	C	A	K	L
I	T	A	P	S	T	V	V	K	D	Y	T	H	E	A	A	T	V	C	A	K	L
I	N	A	P	S	T	V	A	K	D	Y	T	H	E	A	A	T	V	C	A	K	L
I	N	A	P	S	T	V	A	K	D	Y	T	H	E	A	A	T	V	C	A	K	L
I	T	A	P	S	T	V	V	K	D	Y	T	H	E	A	A	T	V	C	A	K	L

I	N	A	P	S	T	V	A	K	D	Y	T	H	E	A	A	T	V	C	A	K	L
I	N	A	P	S	T	V	A	K	D	Y	T	H	E	A	A	T	V	C	A	K	L
I	N	A	P	S	T	V	A	K	D	Y	T	H	E	A	A	T	V	C	A	K	L
I	N	A	P	S	T	V	A	K	D	Y	T	H	E	A	A	T	V	C	A	K	L
I	N	A	P	S	T	V	A	K	D	Y	T	H	E	A	A	T	V	C	A	K	L
I	N	A	P	S	T	V	A	K	D	Y	T	H	E	A	A	T	V	C	A	K	L
I	N	A	P	S	T	V	A	K	D	Y	T	H	E	A	A	T	V	C	A	K	L
I	N	A	P	S	T	V	A	K	D	Y	T	H	E	A	A	T	V	C	A	K	L
I	N	A	P	S	T	V	A	K	D	Y	T	H	E	A	A	T	V	C	A	K	L
I	N	A	P	S	T	V	A	K	D	Y	T	H	E	A	A	T	V	C	A	K	L
I	N	A	P	S	T	V	A	K	D	Y	T	H	E	A	A	T	V	C	A	K	L
I	N	A	P	S	T	V	A	K	D	Y	T	H	E	A	A	T	V	C	A	K	L
I	N	A	P	S	T	V	A	K	D	Y	T	H	E	A	A	T	V	C	A	K	L
I	N	A	P	S	T	V	A	K	D	Y	T	H	E	A	A	T	V	C	A	K	L
I	N	A	P	S	T	V	A	K	D	Y	T	H	E	A	A	T	V	C	A	K	L
I	N	A	P	S	T	V	A	K	D	Y	T	H	E	A	A	T	V	C	A	K	L
I	N	A	P	S	T	V	A	K	D	Y	T	H	E	A	A	T	V	C	A	K	L
I	N	A	P	S	T	V	A	K	D	Y	T	H	E	A	A	T	V	C	A	K	L
I	N	A	P	S	T	V	A	K	D	Y	T	H	E	A	A	T	V	C	A	K	L
I	N	A	P	S	T	V	A	K	D	Y	T	H	E	A	A	T	V	C	A	K	L
I	N	A	P	S	T	V	A	K	D	Y	T	H	E	A	A	T	V	C	A	K	L
I	N	A	P	S	T	V	A	K	D	Y	T	H	E	A	A	T	V	C	A	K	L
I	N	A	P	S	T	V	A	K	D	Y	T	H	E	A	A	T	V	C	A	K	L
I	N	A	P	S	T	V	A	K	D	Y	T	H	E	A	A	T	V	C	A	K	L
I	N	A	P	S	T	V	A	K	D	Y	T	H	E	A	A	T	V	C	A	K	L
I	N	A	P	S	T	V	A	K	D	Y	T	H	E	A	A	T	V	C	A	K	L
I	N	A	P	S	T	V	A	K	D	Y	T	H	E	A	A	T	V	C	A	K	L
I	N	A	P	S	T	V	A	K	D	Y	T	H	E	A	A	T	V	C	A	K	L
I	N	A	P	S	T	V	A	K	D	Y	T	H	E	A	A	T	V	C	A	K	L
I	T	A	P	S	T	V	V	K	D	Y	T	H	E	A	A	T	V	C	A	K	L
I	T	A	P	S	T	V	V	K	D	Y	T	H	E	A	A	T	V	C	A	K	L
I	N	A	P	S	T	V	A	K	D	Y	T	H	E	A	A	T	V	C	A	K	L
I	T	A	P	S	T	V	V	K	D	Y	T	H	E	A	A	T	V	C	A	K	L
I	N	A	P	S	T	V	A	K	D	Y	T	H	E	A	A	T	V	C	A	K	L
I	T	A	P	S	T	V	V	K	D	Y	T	H	E	A	A	T	V	C	A	K	L
I	N	A	P	S	T	V	A	K	D	Y	T	H	E	A	A	T	V	C	A	K	L
I	T	A	P	S	T	V	V	K	D	Y	T	H	E	A	A	T	V	C	A	K	L
I	T	A	P	S	T	V	V	K	D	Y	T	H	E	A	A	T	V	C	A	K	L
I	N	A	P	S	T	V	A	K	D	Y	T	H	E	A	A	T	V	C	A	K	L
I	N	A	P	S	T	V	A	K	D	Y	T	H	E	A	A	T	V	C	A	K	L
I	N	A	P	S	T	V	A	K	D	Y	T	H	E	A	A	T	V	C	A	K	L
I	N	A	P	S	T	V	A	K	D	Y	T	H	E	A	A	T	V	C	A	K	L
I	T	A	P	S	T	V	V	K	D	Y	T	H	E	A	A	T	V	C	A	K	L
I	N	A	P	S	T	V	A	K	D	Y	T	H	E	A	A	T	V	C	A	K	L
I	T	A	P	S	T	V	A	K	D	Y	T	H	E	A	A	T	V	C	A	K	L
I	N	A	P	S	T	V	A	K	D	Y	T	H	E	A	A	T	V	C	A	K	L
I	N	A	P	S	T	V	A	K	D	Y	T	H	E	A	A	T	V	C	A	K	L
I	N	A	P	S	T	V	A	K	D	Y	T	H	E	A	A	T	V	C	A	K	L
I	N	A	P	S	T	V	A	K	D	Y	T	H	E	A	A	T	V	C	A	K	L
I	T	A	P	S	T	V	A	K	D	Y	T	H	E	A	A	T	V	C	A	K	L
I	N	A	P	S	T	V	A	K	D	Y	T	H	E	A	A	T	V	C	A	K	L
I	N	A	P	S	T	V	A	K	D	Y	T	H	E	A	A	T	V	C	A	K	L
I	N	A	P	S	T	V	A	K	D	Y	T	H	E	A	A	T	V	C	A	K	L
I	N	A	P	S	T	V	A	K	D	Y	T	H	E	A	A	T	V	C	A	K	L
I	N	A	P	S	T	V	A	K	D	Y	T	H	E	A	A	T	V	C	A	K	L
I	N	A	P	S	T	V	A	K	D	Y	T	H	E	A	A	T	V	C	A	K	L
I	N	A	P	S	T	V	A	K	D	Y	T	H	E	A	A	T	V	C	A	K	L
I	N	A	P	S	T	V	A	K	D	Y	T	H	E	A	A	T	V	C	A	K	L
I	N	A	P	S	T	V	A	K	D	Y	T	H	E	A	A	T	V	C	A	K	L
I	N	A	P	S	T	V	A	K	D	Y	T	H	E	A	A	T	V	C	A	K	L
I	N	A	P	S	T	V	A	K	D	Y	T	H	E	A	A	T	V	C	A	K	L
I	N	A	P	S	T	V	A	K	D	Y	T	H	E	A	A	T	V	C	A	K	L
I	N	A	P	S	T	V	A	K	D	Y	T	H	E	A	A	T	V	C	A	K	L
I	N	A	P	S	T	V	A	K	D	Y	T	H	E	A	A	T	V	C	V	K	L
I	T	A	P	S	T	V	V	K	D	Y	T	H	E	A	A	T	V	C	A	K	L
I	T	A	P	S	T	V	A	K	D	Y	T	H	E	A	A	T	V	C	A	K	L
I	T	A	P	S	T	V	V	K	D	Y	T	H	E	A	A	T	V	C	A	K	L

I	T	A	P	S	T	V	V	K	D	Y
I	T	A	P	S	T	V	A	K	D	Y
I	T	A	P	S	T	V	V	K	D	Y
I	T	A	P	S	T	V	V	K	D	Y
I	T	A	P	S	T	V	V	K	D	Y
I	T	A	P	S	T	V	A	K	D	Y
I	T	A	P	S	T	V	A	K	D	Y
I	T	A	P	S	T	V	A	K	D	Y
I	T	A	P	S	T	V	V	K	D	Y
I	T	A	P	S	T	V	A	K	D	Y
I	T	A	P	S	T	V	V	K	D	Y
I	T	A	P	S	T	V	A	K	D	Y
I	T	A	P	S	T	V	V	K	D	Y
I	T	A	P	S	T	V	V	K	D	Y
I	N	A	P	S	T	V	A	K	D	Y
I	T	A	P	S	T	V	A	K	D	Y
I	T	A	P	S	T	V	A	K	D	Y
I	T	A	P	S	T	V	V	K	D	Y
I	T	A	P	S	T	I	V	K	D	Y
I	T	A	P	S	T	V	V	K	D	Y
I	T	A	P	S	T	V	A	K	D	Y
I	T	A	P	S	T	V	V	K	D	Y
I	T	A	P	S	T	V	V	K	D	Y
I	T	A	P	S	T	V	V	K	D	Y
I	T	A	P	S	T	V	A	K	D	Y
I	T	A	P	S	T	V	V	K	D	Y
I	T	A	P	S	T	V	V	K	D	Y

T	H	E	A	A	T	V	C	A	K	L
T	H	E	A	A	T	V	C	A	K	L
T	H	E	A	A	T	V	C	A	K	L
T	H	E	A	A	T	V	C	A	K	L
T	H	E	A	A	T	V	C	A	K	L
T	H	E	A	A	T	V	C	A	K	L
T	H	E	A	A	T	V	C	A	K	L
T	H	E	A	A	T	V	C	A	K	L
T	H	E	A	A	T	V	C	A	K	L
T	H	E	A	A	T	V	C	A	K	L
T	H	E	A	A	T	V	C	A	K	L
T	H	E	A	A	T	V	C	A	K	L
T	H	E	A	A	T	V	C	A	K	L
T	H	E	A	A	T	V	C	A	K	L
T	H	E	A	A	T	V	C	A	K	L
T	H	E	A	A	T	V	C	A	K	L
T	H	E	A	A	T	V	C	A	K	L
T	H	E	A	A	T	V	C	A	K	L
T	H	E	A	A	T	V	C	A	K	L
T	H	E	A	A	T	V	C	A	K	L
T	H	E	A	A	T	V	C	A	K	L
T	H	E	A	A	T	V	C	A	K	L
T	H	E	A	A	T	V	C	A	K	L
T	H	E	A	A	T	V	C	A	K	L
T	H	E	A	A	T	V	C	A	K	L
T	H	E	A	A	T	V	C	A	K	L
T	H	E	A	A	T	V	C	A	K	L

		wzh																			
473	479	27	34	51	74	92	93	94	101	123	142	162	170	171	176	190	191	194	195	203	218
A	M	A	V	P	V	G	K	K	D	S	A	V	P	K	R	Q	N	Y	V	L	T
A	M	A	V	P	V	G	K	K	D	S	A	V	P	K	R	Q	N	Y	V	L	T
A	M	A	V	P	V	G	K	K	D	N	A	V	P	R	R	Q	D	Y	V	L	T
A	M	A	V	P	V	G	K	K	D	N	A	V	P	R	R	Q	D	Y	V	L	T
A	M	A	V	P	V	G	K	K	D	N	A	V	P	R	R	Q	D	Y	V	L	T
A	M	A	V	P	V	G	K	K	D	N	A	V	P	R	R	Q	D	Y	V	L	T
A	M	A	V	P	V	G	K	K	D	N	A	V	P	R	R	Q	D	Y	V	L	T
A	M	A	V	P	V	G	K	K	D	N	A	V	P	R	R	Q	D	Y	V	L	T
A	M	A	V	P	V	G	K	K	D	N	A	V	P	R	R	Q	D	Y	V	L	T
A	M	A	V	P	V	G	K	K	D	N	A	V	P	R	R	Q	D	Y	V	L	T
A	M	A	V	P	V	G	K	K	D	N	A	V	P	R	R	Q	D	Y	V	L	T
A	M	A	V	P	V	G	K	K	D	N	A	V	P	R	R	Q	D	Y	V	L	T
A	M	A	V	P	V	G	K	K	D	N	A	V	P	R	R	Q	D	Y	V	L	T
A	M	A	V	P	V	G	K	K	D	N	A	V	P	R	R	Q	D	Y	V	L	T
A	M	A	V	P	V	G	K	K	D	N	A	V	P	R	R	Q	D	Y	V	L	T
A	M	A	V	P	V	G	K	K	D	N	A	V	P	R	R	Q	D	Y	V	L	T
A	M	A	V	P	V	G	K	K	D	N	A	V	P	R	R	Q	D	Y	V	L	T
A	M	A	V	P	V	G	K	K	D	N	A	V	P	R	R	Q	D	Y	V	L	T
A	M	A	V	P	V	G	K	K	D	N	A	V	P	R	R	Q	D	Y	V	L	T
A	M	A	V	P	V	G	K	K	D	N	A	V	P	R	R	Q	D	Y	V	L	T
A	M	A	V	P	V	G	K	K	D	N	A	V	P	R	R	Q	D	Y	V	L	T
A	M	A	V	P	V	G	K	K	D	N	A	V	P	R	R	Q	D	Y	V	L	T
A	M	A	V	P	V	G	K	K	D	N	A	V	P	R	R	Q	D	Y	V	L	T
A	M	A	V	P	V	G	K	K	D	N	A	V	P	R	R	Q	D	Y	V	L	T
A	M	A	V	P	V	G	K	K	D	N	A	V	P	R	R	Q	D	Y	V	L	T
A	M	A	V	P	V	G	K	K	D	N	A	V	P	R	R	Q	D	Y	V	L	T
A	M	A	V	P	V	G	K	K	D	N	A	V	P	R	R	Q	D	Y	V	L	T
A	M	A	V	P	V	G	K	K	D	N	A	V	P	R	R	Q	D	Y	V	L	T
A	M	A	V	P	V	G	K	K	D	N	A	V	P	R	R	Q	D	Y	V	L	T
A	M	A	V	P	V	G	K	K	D	N	A	V	P	R	R	Q	D	Y	V	L	T
A	M	A	V	P	I	G	K	K	D	S	A	V	P	K	R	Q	D	Y	V	L	T
A	M	A	V	P	I	G	K	K	D	S	A	V	P	K	R	Q	D	Y	V	L	T
A	M	A	V	P	I	G	K	K	D	S	A	V	P	K	R	Q	D	Y	V	L	T
A	M	A	V	P	I	G	K	K	D	S	A	V	P	K	R	Q	D	Y	V	L	T
A	M	A	V	P	I	G	K	K	D	S	A	V	P	K	R	Q	D	Y	V	L	T
A	M	A	V	P	I	G	K	K	D	S	A	V	P	K	R	Q	D	Y	V	L	T
A	M	A	V	P	I	G	K	K	D	S	A	V	P	K	R	Q	D	Y	V	L	T
A	M	A	V	P	I	G	K	K	D	S	A	V	P	K	R	Q	D	Y	V	L	T
A	M	A	V	P	I	G	K	K	D	S	A	V	P	K	R	Q	D	Y	V	L	T
A	M	A	V	P	I	G	K	K	D	S	A	V	P	K	R	Q	D	Y	V	L	T
A	M	A	V	P	I	G	K	K	D	S	A	V	P	K	R	Q	D	Y	V	L	T
A	M	A	V	P	I	G	K	K	D	S	A	V	P	K	R	Q	D	Y	V	L	T
A	M	A	V	P	I	G	K	K	D	S	A	V	P	K	R	Q	D	Y	V	L	T
A	M	A	V	P	I	G	K	K	D	S	A	V	P	K	R	Q	D	Y	V	L	T
A	M	A	V	P	I	G	K	K	D	S	A	V	P	K	R	Q	D	Y	V	L	T
A	M	A	V	P	I	G	K	K	D	S	A	V	P	K	R	Q	D	Y	V	L	T
A	M	A	V	P	I	G	K	K	D	S	A	V	P	K	R	Q	D	Y	V	L	T
A	M	A	V	P	I	G	K	K	D	S	A	V	P	K	R	Q	D	Y	V	L	T
A	M	A	V	P	I	G	K	K	D	S	A	V	P	K	R	Q	D	Y	V	L	T
A	M	A	V	P	I	G	K	K	D	S	A	V	P	K	R	Q	D	Y	V	L	T
A	M	A	V	P	I	G	K	K	D	S	A	V	P	K	R	Q	D	Y	V	L	T
A	M	A	V	P	I	G	K	K	D	S	A	V	P	K	R	Q	D	Y	V	L	T
A	M	A	V	P	I	G	K	K	D	S	A	V	P	K	R	Q	D	Y	V	L	T
A	M	A	V	P	V	G	K	K	D	S	A	V	P	K	R	Q	D	Y	V	L	T
A	M	A	V	P	I	G	K	K	D	S	A	V	P	K	R	Q	D	Y	V	L	T
A	M	A	V	P	I	G	K	K	D	S	A	V	P	K	R	Q	D	Y	V	L	T
A	M	A	V	L	I	G	K	K	D	S	A	V	P	K	R	Q	D	Y	V	L	T
A	M	A	V	P	I	G	K	K	D	S	A	V	P	K	R	Q	D	Y	V	L	T
A	M	A	V	P	I	G	K	K	D	S	A	V	P	K	R	Q	D	Y	V	L	T
A	M	A	V	P	V	G	K	K	D	S	A	V	P	K	R	Q	D	Y	V	L	T
A	M	A	V	P	V	G	K	K	D	S	A	V	P	K	R	Q	D	Y	V	L	T
A	M	A	V	P	V	G	K	K	D	S	A	V	P	K	R	Q	D	Y	V	L	T
A	M	A	V	P	V	G	K	K	D	S	A	V	P	K	R	Q	D	Y	V	L	T

A	M	A	V	P	V	G	K	K	D	S	A	V	P	K	R	Q	D	Y	V	L	T
A	M	A	V	P	V	G	K	K	D	S	A	V	P	K	R	Q	D	Y	V	L	T
A	M	A	V	P	V	G	K	K	D	S	A	V	P	K	R	Q	D	Y	V	L	T
A	M	A	V	P	V	G	K	K	D	S	A	V	P	K	R	Q	D	Y	V	L	T
A	M	A	V	P	V	G	K	K	D	S	A	V	P	K	R	Q	D	Y	V	L	T
A	M	A	V	P	V	G	K	K	D	S	A	V	P	K	R	Q	D	Y	V	L	T
A	M	A	V	P	V	G	K	K	D	S	A	V	P	K	R	Q	D	Y	V	L	T
A	M	A	V	P	V	G	K	K	D	S	A	V	P	K	R	Q	D	Y	V	L	T
A	M	A	V	P	V	G	K	K	D	S	A	V	P	K	R	Q	D	Y	V	L	T
A	M	A	V	P	V	G	K	K	D	S	A	V	P	K	R	Q	D	Y	V	L	T
A	M	A	V	P	V	G	K	K	D	S	A	V	P	K	R	Q	D	Y	V	L	T
A	M	A	V	P	V	G	K	K	D	S	A	V	P	K	R	Q	D	Y	V	L	T
A	M	A	V	P	V	G	K	K	D	S	A	V	P	K	R	Q	D	Y	V	L	T
A	M	A	V	P	V	G	K	K	D	S	A	V	P	K	R	Q	D	Y	V	L	T
A	M	A	V	P	V	G	K	K	D	S	A	V	P	K	R	Q	D	Y	V	L	T
A	M	A	V	P	V	G	K	K	D	S	A	V	P	K	R	Q	D	Y	V	L	T
A	M	A	V	P	V	G	K	K	D	S	A	V	P	K	R	Q	D	Y	V	L	T
A	M	A	V	P	V	G	K	K	D	S	A	V	P	K	R	Q	D	Y	V	L	T
A	M	A	V	P	V	G	K	K	D	S	A	V	P	K	R	Q	D	Y	V	L	T
A	M	A	V	P	V	G	K	K	D	S	A	V	P	K	R	Q	D	Y	V	L	T
A	M	A	V	P	V	G	K	K	D	S	A	V	P	K	R	Q	D	Y	V	L	T
A	M	A	V	P	I	G	K	K	D	S	A	V	P	K	R	Q	D	Y	V	L	T
A	M	A	V	P	I	G	K	K	D	S	A	V	P	K	R	Q	D	Y	V	L	T
A	M	A	V	P	I	G	K	K	D	S	A	V	P	K	R	Q	D	Y	V	L	T
A	M	A	V	P	V	G	K	K	D	S	A	V	P	K	R	Q	D	H	V	L	T
A	M	A	V	P	V	G	K	K	D	S	A	V	P	K	R	Q	D	H	V	L	T
A	M	A	V	P	V	G	K	K	D	S	A	V	P	K	R	Q	D	H	V	L	T
A	M	A	V	P	V	G	K	K	D	S	A	V	P	K	R	Q	N	Y	V	L	T
A	M	A	V	P	V	G	K	K	D	S	A	V	P	K	R	Q	N	Y	V	L	T
A	M	A	V	P	V	G	K	K	D	S	A	V	P	K	R	Q	N	Y	V	L	T
A	M	A	V	P	V	G	K	K	D	S	A	V	P	K	R	Q	N	Y	V	L	T
A	M	A	V	P	V	G	K	K	D	S	A	V	P	K	R	Q	N	Y	V	L	T
A	M	A	V	P	V	G	K	K	D	S	A	V	P	K	R	Q	N	Y	V	L	T
A	M	A	V	P	V	G	K	K	D	S	A	V	P	K	R	Q	N	Y	V	L	T
A	M	A	V	P	V	G	K	K	D	S	A	V	P	K	R	Q	N	Y	V	L	T
A	M	A	V	P	V	G	K	K	D	S	A	V	P	K	R	Q	N	Y	V	L	T
A	M	A	V	P	V	G	K	K	D	S	A	V	P	K	R	Q	N	Y	V	L	T
A	M	A	V	P	V	G	K	K	D	S	A	V	P	K	R	Q	N	Y	V	L	T
A	M	A	V	P	V	G	K	K	D	S	A	V	P	K	R	Q	N	Y	V	L	T
A	M	A	V	P	V	G	K	K	D	S	A	V	P	K	R	Q	N	Y	V	L	T
A	M	A	V	P	V	G	K	K	D	S	A	V	P	K	R	Q	N	Y	V	L	T
A	M	A	V	P	V	G	K	K	D	S	A	V	P	K	R	Q	N	Y	V	L	T
A	M	A	V	P	V	G	K	K	D	S	A	V	P	K	R	Q	N	Y	V	L	T
A	M	A	V	P	V	G	K	K	D	S	A	V	P	K	R	Q	N	Y	V	L	T
A	M	A	V	P	V	G	K	K	D	S	A	V	P	K	R	Q	N	Y	V	L	T
A	M	A	V	P	V	G	K	K	D	S	A	V	P	K	R	Q	N	Y	V	L	T
A	M	A	V	P	V	G	K	K	D	S	A	V	P	K	R	Q	N	Y	V	L	T
A	M	A	V	P	V	G	K	K	D	S	A	V	P	K	R	Q	N	Y	V	L	T
A	M	A	V	P	V	G	K	K	D	S	A	V	P	K	R	Q	N	Y	V	L	T
A	M	A	V	P	V	G	K	K	D	S	A	V	P	K	R	Q	N	Y	V	L	T
A	M	A	V	P	V	G	K	K	D	S	A	V	P	K	R	Q	N	Y	V	L	T
A	M	A	V	P	V	G	K	K	D	S	A	V	P	K	R	Q	N	Y	V	L	T
A	M	A	V	P	V	G	K	K	D	S	A	V	P	K	R	Q	N	Y	V	L	T
A	M	A	V	P	V	G	K	K	D	S	A	V	P	K	R	Q	N	Y	V	L	T
A	M	A	V	P	V	G	K	K	D	S	A	V	P	K	R	Q	N	Y	V	L	T
A	M	A	V	P	V	G	K	K	D	S	A	V	P	K	R	Q	N	Y	V	L	T
A	M	A	V	P	V	G	K	K	D	S	A	V	P	K	R	Q	N	Y	V	L	T
A	M	A	V	P	V	G	K	K	D	S	A	V	P	K	R	Q	N	Y	V	L	T
A	M	A	V	P	V	G	K	K	D	S	A	V	P	K	R	Q	N	Y	V	L	T
A	M	A	V	P	V	G	K	K	D	S	A	V	P	K	R	Q	N	Y	V	L	T
A	M	A	V	P	V	G	K	K	D	S	A	V	P	K	R	Q	N	Y	V	L	T
A	M	A	V	P	V	G	K	K	D	S	A	V	P	K	R	Q	N	Y	V	L	T
A	M	A	V	P	V	G	K	K	D	S	A	V	P	K	R	Q	N	Y	V	L	T
A	M	A	V	P	V	G	K	K	D	S	A	V	P	K	R	Q	N	Y	V	L	T
A	M	A	V	P	V	G	K	K	D	S	A	V	P	K	R	Q	N	Y	V	L	T
A	M	A	V	P	V	G	K	K	D	S	A	V	P	K	R	Q	N	Y	V	L	T
A	M	A	V	P	V	G	K	K	D	S	A	V	P	K	R	Q	N	Y	V	L	T

A	M	A	V	P	V	G	K	K	D	S	A	V	P	K	R	Q	N	Y	V	L	T
A	M	A	V	P	V	G	K	K	D	S	A	V	P	K	R	Q	N	Y	V	L	T
A	M	A	V	P	V	G	K	K	D	S	A	V	P	K	R	Q	N	Y	V	L	T
A	M	A	V	P	V	G	K	K	D	S	A	V	P	K	R	Q	N	Y	V	L	T
A	M	A	V	P	V	G	K	K	D	S	A	V	P	K	R	Q	N	Y	V	L	T
A	M	A	V	P	V	G	K	K	D	S	A	V	P	K	R	Q	N	Y	V	L	T
A	M	A	V	P	V	G	K	K	D	S	A	V	P	K	R	Q	N	Y	V	L	T
A	M	A	V	P	V	G	K	K	D	S	A	V	P	K	R	Q	N	Y	V	L	T
A	M	A	V	P	V	G	K	K	D	S	A	V	P	K	R	Q	N	Y	V	L	T
A	M	A	V	P	V	G	K	K	D	S	A	V	P	K	R	Q	N	Y	V	L	T
A	M	A	V	P	V	G	K	K	D	S	A	V	P	K	R	Q	N	Y	V	L	T
A	M	A	V	P	V	G	K	K	D	S	A	V	P	K	R	Q	N	Y	V	L	T
A	M	A	V	P	V	G	K	K	D	S	A	V	P	K	R	Q	N	Y	V	L	T
A	M	A	V	P	V	G	K	K	D	S	A	V	P	K	R	Q	N	Y	V	L	T
A	M	A	V	P	V	G	K	K	D	S	A	V	P	K	R	Q	N	Y	V	L	T
A	M	A	V	P	V	G	K	K	D	S	A	V	P	K	R	Q	N	Y	V	L	T
A	M	A	V	P	V	G	K	K	D	S	A	V	P	K	R	Q	N	Y	V	L	T
A	M	A	V	P	V	G	K	K	D	S	A	V	P	K	R	Q	N	Y	V	L	T
A	M	A	V	P	V	G	K	K	D	S	A	V	P	K	R	Q	N	Y	V	L	T
A	M	A	V	P	V	G	K	K	D	S	A	V	P	K	R	Q	N	Y	V	L	T
A	M	A	V	P	V	G	K	K	D	S	A	V	P	K	R	Q	N	Y	V	L	T
A	M	A	V	P	V	G	K	K	D	S	A	V	P	K	R	Q	N	Y	V	L	T
A	M	A	V	P	V	G	K	K	D	S	A	V	P	K	R	Q	N	Y	V	L	T
A	M	A	V	P	V	G	K	K	D	S	A	V	P	K	R	Q	N	Y	V	L	T
A	M	A	V	P	V	G	K	K	D	S	A	V	P	K	R	Q	N	Y	V	L	T
A	M	A	V	P	V	G	K	K	D	S	A	V	P	K	R	Q	N	Y	V	L	T
A	M	A	V	P	V	G	K	K	D	S	A	V	P	K	R	Q	N	Y	V	L	T
A	M	A	V	P	V	G	K	K	D	S	A	V	P	K	R	Q	N	Y	V	L	T
A	M	A	V	P	V	G	K	K	D	S	A	V	P	K	R	Q	N	Y	V	L	T
A	M	A	V	P	V	G	K	K	D	S	A	V	P	K	R	Q	N	Y	V	L	T
A	M	A	V	P	V	G	K	K	D	S	A	V	P	K	R	Q	N	Y	V	L	T
A	M	A	V	P	V	G	K	K	D	S	A	V	P	K	R	Q	N	Y	V	L	T
A	M	A	V	P	V	G	K	K	D	S	A	V	P	K	R	Q	N	Y	V	L	T
A	M	A	V	P	V	G	K	K	D	S	A	V	P	K	R	Q	N	Y	V	L	T
A	M	A	V	P	V	G	K	K	D	S	A	V	P	K	R	Q	N	Y	V	L	T
A	M	A	V	P	V	G	K	K	D	S	A	V	P	K	R	Q	N	Y	V	L	T
A	M	A	V	P	V	G	K	K	D	S	A	V	P	K	R	Q	N	Y	V	L	T
A	M	A	V	P	V	G	K	K	D	S	A	V	P	K	R	Q	N	Y	V	L	T
A	M	A	V	P	V	G	K	K	D	S	A	V	P	K	R	Q	N	Y	V	L	T
A	M	A	V	P	V	G	K	K	D	S	A	V	P	K	R	Q	N	Y	V	L	T
A	M	A	V	P	V	G	K	K	D	S	A	V	P	K	R	Q	N	Y	V	L	T
A	M	A	V	P	V	G	K	K	D	S	A	V	P	K	R	Q	N	Y	V	L	T
A	M	A	V	P	V	G	K	K	D	S	A	V	P	K	R	Q	N	Y	V	L	T
A	M	A	V	P	V	G	K	K	D	S	A	V	P	K	R	Q	N	Y	V	L	T
A	M	A	V	P	V	G	K	K	D	S	A	V	P	K	R	Q	N	Y	V	L	T
A	M	A	V	P	V	G	K	K	D	S	A	V	P	K	R	Q	N	Y	V	L	T
A	M	A	V	P	V	G	K	K	D	S	A	V	P	K	R	Q	N	Y	V	L	T
A	M	A	V	P	V	G	K	K	D	S	A	V	P	K	R	Q	N	Y	V	L	T
A	M	A	V	P	V	G	K	K	D	S	A	V	P	K	R	Q	N	Y	V	L	T
A	M	A	V	P	V	G	K	K	D	S	A	V	P	K	R	Q	N	Y	V	L	T
A	M	A	V	P	V	G	K	K	D	S	A	V	P	K	R	Q	N	Y	V	L	T
A	M	A	V	P	V	G	K	K	D	S	A	V	P	K	R	Q	N	Y	V	L	T
A	M	A	V	P	V	G	K	K	D	S	A	V	P	K	R	Q	N	Y	V	L	T
A	M	A	V	P	V	G	K	K	D	S	A	V	P	K	R	Q	N	Y	V	L	T
A	M	A	V	P	V	G	K	K	D	S	A	V	P	K	R	Q	N	Y	V	L	T
A	M	A	V	P	V	G	K	K	D	S	A	V	P	K	R	Q	N	Y	V	L	T
A	M	A	V	P	V	G	K	K	D	S	A	V	P	K	R	Q	N	Y	V	L	T
A	M	A	V	P	V	G	K	K	D	S	A	V	P	K	R	Q	N	Y	V	L	T
A	M	A	V	P	V	G	K	K	D	S	A	V	P	K	R	Q	N	Y	V	L	T
A	M	A	V	P	V	G	K	K	D	S	A	V	P	K	R	Q	N	Y	V	L	T
A	M	A	V	P	V	G	K	K	D	S	A	V	P	K	R	Q	N	Y	V	L	T
A	M	A	V	P	V	G	K	K	D	S	A	V	P	K	R	Q	N	Y	V	L	T
A	M	A	V	P	V	G	K	K	D	S	A	V	P	K	R	Q	N	Y	V	L	T
A	M	A	V	P	V	G	K	K	D	S	A	V	P	K	R	Q	N	Y	V	L	T
A	M	A	V	P	V	G	K	K	D	S	A	V	P	K	R	Q	N	Y	V	L	T
A	M	A	V	P	V	G	K	K	D	S	A	V	P	K	R	Q	N	Y	V	L	T
A	M	A	V	P	V	G	K	K	D	S	A	V	P	K	R	Q	N	Y	V	L	T

A	M	A	V	P	V	G	K	K	D	S	A	V	P	K	R	Q	N	Y	V	L	T
A	M	A	V	P	V	G	K	K	D	S	A	V	P	K	R	Q	N	Y	V	L	T
A	M	A	V	P	V	G	K	K	D	S	A	V	P	K	R	Q	N	Y	V	L	T
A	M	A	V	P	V	G	K	K	D	S	A	V	P	K	R	Q	N	Y	V	L	T
A	M	A	V	P	I	G	K	K	D	S	A	V	P	K	R	Q	D	Y	V	L	T
A	M	A	V	P	I	G	K	K	D	S	A	V	P	K	R	Q	D	Y	V	L	T
A	M	A	V	P	I	G	K	K	D	S	A	V	P	K	R	Q	D	Y	V	L	T
A	M	A	V	P	I	G	K	K	D	S	A	V	P	K	R	Q	D	Y	V	L	T
A	M	A	V	P	I	G	K	K	D	S	A	V	P	K	R	Q	D	Y	V	L	T
A	M	A	V	P	I	G	K	K	D	S	A	V	P	K	R	Q	D	Y	V	L	T
A	M	A	V	P	I	G	K	K	D	S	A	V	P	K	R	Q	D	Y	V	L	T
A	M	A	V	P	I	G	K	K	D	S	A	V	P	K	R	Q	D	Y	V	L	T
A	M	A	V	P	I	G	K	K	D	S	A	V	P	K	R	Q	D	Y	V	L	T
A	M	A	V	P	I	G	K	K	D	S	A	V	P	K	R	Q	D	Y	V	L	T
A	M	A	V	P	I	G	K	K	D	S	A	V	P	K	R	Q	D	Y	V	L	T
A	M	A	V	P	I	G	K	K	D	S	A	V	P	K	R	Q	D	Y	V	L	T
A	M	A	V	P	I	G	K	K	D	S	A	V	P	K	R	Q	D	Y	V	L	T
A	M	A	V	P	I	G	K	K	D	S	A	V	P	K	R	Q	D	Y	V	L	T
A	M	A	V	P	I	G	K	K	D	S	A	V	P	K	R	Q	D	Y	V	L	T
A	M	A	V	P	I	G	K	K	D	S	A	V	P	K	R	Q	D	Y	V	L	T
A	M	A	V	P	I	G	K	K	D	S	A	V	P	K	R	Q	D	Y	V	L	T
A	M	A	V	P	I	G	K	K	D	S	A	V	P	K	R	Q	D	Y	V	L	T
A	M	A	V	P	I	G	K	K	D	S	A	V	P	K	R	Q	D	Y	V	L	T
A	M	A	V	P	I	G	K	K	D	S	A	V	P	K	R	Q	D	Y	V	L	T
A	M	A	V	P	I	G	K	K	D	S	A	V	P	K	R	Q	D	Y	V	L	T
A	M	A	V	P	I	G	K	K	D	S	A	V	P	K	R	Q	D	Y	V	L	T
A	M	A	V	P	I	G	K	K	D	S	A	V	P	K	R	Q	D	Y	V	L	T
A	M	A	V	P	I	G	K	K	D	S	A	V	P	K	R	Q	D	Y	V	L	T
A	M	A	V	P	I	G	K	K	D	S	A	V	P	K	R	Q	D	Y	V	L	T
A	M	A	V	P	I	G	K	K	D	S	A	V	P	K	R	Q	D	Y	V	L	T
A	M	A	V	P	I	G	K	K	D	S	A	V	P	K	R	Q	D	Y	V	L	T
A	M	A	V	P	I	G	K	K	D	S	A	V	P	K	R	Q	D	Y	V	L	T
A	M	A	V	P	I	G	K	K	D	S	A	V	P	K	R	Q	D	Y	V	L	T
A	M	A	V	P	I	G	K	K	D	S	A	V	P	K	R	Q	D	Y	V	L	T
A	M	A	V	P	I	G	K	K	D	S	A	V	P	K	R	Q	D	Y	V	L	T
A	M	A	V	P	I	G	K	K	D	S	A	V	P	K	R	Q	D	Y	V	L	T
A	M	A	V	P	I	G	K	K	D	S	A	V	P	K	R	Q	D	Y	V	L	T
A	M	A	V	P	I	G	K	K	D	S	A	V	P	K	R	Q	D	Y	V	L	T
A	M	A	V	P	I	G	K	K	D	S	A	V	P	K	R	Q	D	Y	V	L	T
A	M	A	V	P	I	G	K	K	D	S	A	V	P	K	R	Q	D	Y	V	L	T
A	M	A	V	P	I	G	K	K	D	S	A	V	P	K	R	Q	D	Y	V	L	T
A	M	A	V	P	I	G	K	K	D	S	A	V	P	K	R	Q	D	Y	V	L	T
A	M	A	V	P	I	G	K	K	D	S	A	V	P	K	R	Q	D	Y	V	L	T
A	M	A	V	P	I	G	K	K	D	S	A	V	P	K	R	Q	D	Y	V	L	T
A	M	A	V	P	I	G	K	K	D	S	A	V	P	K	R	Q	D	Y	V	L	T
A	M	A	V	P	I	G	K	K	D	S	A	V	P	K	R	Q	D	Y	V	L	T
A	M	A	V	P	I	G	K	K	D	S	A	V	P	K	R	Q	D	Y	V	L	T
A	M	A	V	P	I	G	K	K	D	S	A	V	P	K	R	Q	D	Y	V	L	T
A	M	A	V	P	I	G	K	K	D	S	A	V	P	K	R	Q	D	Y	V	L	T
A	M	A	V	P	I	G	K	K	D	S	A	V	P	K	R	Q	D	Y	V	L	T
A	M	A	V	P	I	G	K	K	D	S	A	V	P	K	R	Q	D	Y	V	L	T
A	M	A	V	P	I	G	K	K	D	S	A	V	P	K	R	Q	D	Y	V	L	T
A	M	A	V	P	I	G	K	K	D	S	A	V	P	K	R	Q	D	Y	V	L	T
A	M	A	V	P	I	G	K	K	D	S	A	V	P	K	R	Q	D	Y	V	L	T
A	M	A	V	P	I	G	K	K	D	S	A	V	P	K	R	Q	D	Y	V	L	T
A	M	A	V	P	I	G	K	K	D	S	A	V	P	K	R	Q	D	Y	V	L	T
A	M	A	V	P	I	G	K	K	D	S	A	V	P	K	R	Q	D	Y	V	L	T
A	M	A	V	P	I	G	K	K	D	S	A	V	P	K	R	Q	D	Y	V	L	T
A	M	A	V	P	I	G	K	K	D	S	A	V	P	K	R	Q	D	Y	V	L	T
A	M	A	V	P	I	G	K	K	D	S	A	V	P	K	R	Q	D	Y	V	L	T
A	M	A	V	P	I	G	K	K	D	S	A	V	P	K	R	Q	D	Y	V	L	T
A	M	A	V	P	I	G	K	K	D	S	A	V	P	K	R	Q	D	Y	V	L	T
A	M	A	V	P	I	G	K	K	D	S	A	V	P	K	R	Q	D	Y	V	L	T
A	M	A	V	P	I	G	K	K	D	S	A	V	P	K	R	Q	D	Y	V	L	T
A	M	A	V	P	I	G	K	K	D	S	A	V	P	K	R	Q	D	Y	V	L	T
A	M	A	V	P	I	G	K	K	D	S	A	V	P	K	R	Q	D	Y	V	L	T
A	M	A	V	P	I	G	K	K	D	S	A	V	P	K	R	Q	D	Y	V	L	T

A	M	A	V	P	I	G	K	K	D	S	A	V	P	K	R	Q	D	Y	V	L	T
A	M	A	V	P	I	G	K	K	D	S	A	V	P	K	R	Q	D	Y	V	L	T
A	M	A	V	P	I	G	K	K	D	S	A	V	P	K	R	Q	D	Y	V	L	T
A	M	A	V	P	I	G	K	K	D	S	A	V	P	K	R	Q	D	Y	V	L	T
A	M	A	V	P	I	G	K	K	D	S	A	V	P	K	R	Q	D	Y	V	L	T
A	M	A	V	P	I	G	K	K	D	S	A	V	P	K	R	Q	D	Y	V	L	T
A	M	A	V	P	I	G	K	K	D	S	A	V	P	K	R	Q	D	Y	V	L	T
A	M	A	V	P	I	G	K	K	D	S	A	V	P	K	R	Q	D	Y	V	L	T
A	M	A	V	P	I	G	K	K	D	S	A	V	P	K	R	Q	D	Y	V	L	T
A	M	A	V	P	I	G	K	K	D	S	A	V	P	K	R	Q	D	Y	V	L	T
A	M	A	V	P	I	G	K	K	D	S	A	V	P	K	R	Q	D	Y	V	L	T
A	M	A	V	P	V	G	K	K	D	N	A	V	P	R	R	Q	D	Y	V	L	T
A	M	A	V	P	V	G	K	K	D	N	A	V	P	R	R	Q	D	Y	V	L	T
A	M	A	V	P	V	G	K	K	D	N	A	V	P	R	R	Q	D	Y	V	L	T
A	M	A	V	P	V	G	K	K	D	N	A	V	P	R	R	Q	D	Y	V	L	T
A	M	A	V	P	V	G	K	K	D	N	A	V	P	R	R	Q	D	Y	V	L	T
A	M	A	V	P	V	G	K	K	D	N	A	V	P	R	R	Q	D	Y	V	L	T
A	M	A	V	P	V	G	K	K	D	N	A	V	P	R	R	Q	D	Y	V	L	T
A	M	A	V	P	V	G	K	K	D	N	A	V	P	R	R	Q	D	Y	V	L	T
A	M	A	V	P	V	G	K	K	D	N	A	V	P	R	R	Q	D	Y	V	L	T
T	L	T	G	P	V	E	N	N	N	N	V	I	S	K	P	R	D	H	I	V	S
T	L	T	V	P	V	E	N	N	N	N	V	I	S	K	P	R	D	H	I	V	S
T	L	T	V	P	V	E	N	N	N	N	V	I	S	K	P	R	D	H	I	V	S
T	L	T	V	P	V	E	N	N	N	N	V	I	S	K	P	R	D	H	I	V	S
T	L	T	V	P	V	E	N	N	N	N	V	I	S	K	P	R	D	H	I	V	S
T	L	T	V	P	V	E	N	N	N	N	V	I	S	K	P	R	D	H	I	V	S
T	L	T	V	P	V	E	N	N	N	N	V	I	S	K	P	R	D	H	I	V	S
T	L	T	V	P	V	E	N	N	N	N	V	I	S	K	P	R	D	H	I	V	S
A	M	A	V	P	V	G	K	K	D	S	A	V	P	K	R	Q	D	Y	V	L	T
A	M	A	V	P	I	G	K	K	D	S	A	V	P	K	R	Q	D	Y	V	L	T
A	M	A	V	P	I	G	K	K	D	S	A	V	P	K	R	Q	D	Y	V	L	T
A	M	A	V	P	I	G	K	K	D	S	A	V	P	K	R	Q	D	Y	V	L	T
A	M	A	V	P	I	G	K	K	D	S	A	V	P	K	R	Q	D	Y	V	L	T
A	M	A	V	P	I	G	K	K	D	S	A	V	P	K	R	Q	D	Y	V	L	T
A	M	A	V	P	I	G	K	K	D	S	A	V	P	K	R	Q	D	Y	V	L	T
A	M	A	V	P	I	G	K	K	D	S	A	V	P	K	R	Q	D	Y	V	L	T
A	M	A	V	P	I	G	K	K	D	S	A	V	P	K	R	Q	D	Y	V	L	T
A	M	A	V	P	I	G	K	K	D	S	A	V	P	K	R	Q	D	Y	V	L	T
A	M	A	V	P	I	G	K	K	D	S	A	V	P	K	R	Q	D	Y	V	L	T
A	M	A	V	P	I	G	K	K	D	S	A	V	P	K	R	Q	D	Y	V	L	T
A	M	A	V	P	I	G	K	K	D	S	A	V	P	K	R	Q	D	Y	V	L	T
A	M	A	V	P	I	G	K	K	D	S	A	V	P	K	R	Q	D	Y	V	L	T
A	M	A	V	P	I	G	K	K	D	S	A	V	P	K	R	Q	D	Y	V	L	T
A	M	A	V	P	I	G	K	K	D	S	A	V	P	K	R	Q	D	Y	V	L	T
A	M	A	V	P	I	G	K	K	D	S	A	V	P	K	R	Q	D	Y	V	L	T
A	M	A	V	P	I	G	K	K	D	S	A	V	P	K	R	Q	D	Y	V	L	T
A	M	A	V	P	I	G	K	K	D	S	A	V	P	K	R	Q	D	Y	V	L	T
A	M	A	V	P	I	G	K	K	D	S	A	V	P	K	R	Q	D	Y	V	L	T
A	M	A	V	P	V	E	K	K	D	S	A	V	P	K	R	Q	D	Y	V	L	T
A	M	A	V	P	V	E	K	K	D	S	A	V	P	K	R	Q	D	Y	V	L	T
A	M	A	V	P	V	E	K	K	D	S	A	V	P	K	R	Q	D	Y	V	L	T
A	M	A	V	P	V	E	K	K	D	S	A	V	P	K	R	Q	D	Y	V	L	T
A	M	A	V	P	V	E	K	K	D	S	A	V	P	K	R	Q	D	Y	V	L	T
A	M	A	V	P	V	E	K	K	D	S	A	V	P	K	R	Q	D	Y	V	L	T
A	M	A	V	P	V	E	K	K	D	S	A	V	P	K	R	Q	D	Y	V	L	T
A	M	A	V	P	V	E	K	K	D	S	A	V	P	K	R	Q	D	Y	V	L	T
A	M	A	V	P	V	E	K	K	D	S	A	V	P	K	R	Q	D	Y	V	L	T
A	M	A	V	P	V	E	K	K	D	S	A	V	P	K	R	Q	D	Y	V	L	T
A	M	A	V	P	V	E	K	K	D	S	A	V	P	K	R	Q	D	Y	V	L	T
A	M	A	V	P	V	E	K	K	D	S	A	V	P	K	R	Q	D	Y	V	L	T
A	M	A	V	P	V	E	K	K	D	S	A	V	P	K	R	Q	D	Y	V	L	T
A	M	A	V	P	V	E	K	K	D	S	A	V	P	K	R	Q	D	Y	V	L	T
A	M	A	V	P	V	E	K	K	D	S	A	V	P	K	R	Q	D	Y	V	L	T
A	M	A	V	P	V	E	K	K	D	S	A	V	P	K	R	Q	D	Y	V	L	T
A	M	A	V	P	V	E	K	K	D	S	A	V	P	K	R	Q	D	Y	V	L	T
A	M	A	V	P	V	E	K	K	D	S	A	V	P	K	R	Q	D	Y	V	L	T
A	M	A	V	P	V	E	K	K	D	S	A	V	P	K	R	Q	D	Y	V	L	T

A	M	A	V	P	V	E	K	K	D	S	A	V	P	K	R	Q	D	Y	V	L	T
A	M	A	V	P	V	E	K	K	D	S	A	V	P	K	R	Q	D	Y	V	L	T
A	M	A	V	P	V	E	K	K	D	S	A	V	P	K	R	Q	D	Y	V	L	T
A	M	A	V	P	V	E	K	K	D	S	A	V	P	K	R	Q	D	Y	V	L	T
A	M	A	V	P	V	E	K	K	D	S	A	V	P	K	R	Q	D	Y	V	L	T
A	M	A	V	P	V	E	K	K	D	S	A	V	P	K	R	Q	D	Y	V	L	T
A	M	A	V	P	V	E	K	K	D	S	A	V	P	K	R	Q	D	Y	V	L	T
A	M	A	V	P	V	E	K	K	D	S	A	V	P	K	R	Q	D	Y	V	L	T
A	M	A	V	P	V	E	K	K	D	S	A	V	P	K	R	Q	D	Y	V	L	T
A	M	A	V	P	V	E	K	K	D	S	A	V	P	K	R	Q	D	Y	V	L	T
A	M	A	V	P	V	E	K	K	D	S	A	V	P	K	R	Q	D	Y	V	L	T
A	M	A	V	P	V	E	K	K	D	S	A	V	P	K	R	Q	D	Y	V	L	T
A	M	A	V	P	V	E	K	K	D	S	A	V	P	K	R	Q	D	Y	V	L	T
A	M	A	V	P	V	E	K	K	D	S	A	V	P	K	R	Q	D	Y	V	L	T
A	M	A	V	P	V	E	K	K	D	S	A	V	P	K	R	Q	D	Y	V	L	T
A	M	A	V	P	V	E	K	K	D	S	A	V	P	K	R	Q	D	Y	V	L	T
A	M	A	V	P	V	E	K	K	D	S	A	V	P	K	R	Q	D	Y	V	L	T
A	M	A	V	P	V	E	K	K	D	S	A	V	P	K	R	Q	D	Y	V	L	T
A	M	A	V	P	V	E	K	K	D	S	A	V	P	K	R	Q	D	Y	V	L	T
A	M	A	V	P	V	E	K	K	D	S	A	V	P	K	R	Q	D	Y	V	L	T
A	M	A	V	P	V	E	K	K	D	S	A	V	P	K	R	Q	D	Y	V	L	T
A	M	A	V	P	V	E	K	K	D	S	A	V	P	K	R	Q	D	Y	V	L	T
A	M	A	V	P	V	E	K	K	D	S	A	V	P	K	R	Q	D	Y	V	L	T
A	M	A	V	P	V	G	K	K	D	S	A	V	P	K	R	Q	D	H	V	L	T
A	M	A	V	P	V	G	K	K	D	N	A	V	P	R	R	Q	D	Y	V	L	T
A	M	A	V	P	V	G	K	K	D	N	A	V	P	R	R	Q	D	Y	V	L	T
A	M	A	V	P	V	G	K	K	D	N	A	V	P	R	R	Q	D	Y	V	L	T
A	M	A	V	P	V	G	K	K	D	N	A	V	P	R	R	Q	D	Y	V	L	T
A	M	A	V	P	V	G	K	K	D	N	A	V	P	R	R	Q	D	Y	V	L	T
A	M	A	V	P	V	G	K	K	D	N	A	V	P	R	R	Q	D	Y	V	L	T
A	M	A	V	P	V	G	K	K	D	N	A	V	P	R	R	Q	D	Y	V	L	T
A	M	A	V	P	V	G	K	K	D	N	A	V	P	R	R	Q	D	Y	V	L	T
A	M	A	V	P	V	G	K	K	D	N	A	V	P	R	R	Q	D	Y	V	L	T
A	M	A	V	P	V	G	K	K	D	N	A	V	P	R	R	Q	D	Y	V	L	T
A	M	A	V	P	V	G	K	K	D	N	A	V	P	R	R	Q	D	Y	V	L	T
A	M	A	V	P	V	G	K	K	D	N	A	V	P	R	R	Q	D	Y	V	L	T
A	M	A	V	P	V	G	K	K	D	N	A	V	P	R	R	Q	D	Y	V	L	T
A	M	A	V	P	V	G	K	K	D	N	A	V	P	R	R	Q	D	Y	V	L	T
A	M	A	V	P	V	G	K	K	D	N	A	V	P	R	R	Q	D	Y	V	L	T
A	M	A	V	P	V	G	K	K	D	N	A	V	P	R	R	Q	D	Y	V	L	T
A	M	A	V	P	V	G	K	K	D	N	A	V	P	R	R	Q	D	Y	V	L	T
A	M	A	V	P	V	G	K	K	D	N	A	V	P	R	R	Q	D	Y	V	L	T
A	M	A	V	P	V	G	K	K	D	N	A	V	P	R	R	Q	D	Y	V	L	T
A	M	A	V	P	V	G	K	K	D	N	A	V	P	R	R	Q	D	Y	V	L	T
A	M	A	V	P	V	G	K	K	D	N	A	V	P	R	R	Q	D	Y	V	L	T
A	M	A	V	P	V	G	K	K	D	N	A	V	P	R	R	Q	D	Y	V	L	T
A	M	A	V	P	V	G	K	K	D	N	A	V	P	R	R	Q	D	Y	V	L	T
A	M	A	V	P	V	G	K	K	D	N	A	V	P	R	R	Q	D	Y	V	L	T
A	M	A	V	P	V	G	K	K	D	N	A	V	P	R	R	Q	D	Y	V	L	T
A	M	A	V	P	V	G	K	K	D	S	A	V	P	K	R	Q	D	Y	V	L	T
A	M	A	V	P	V	G	K	K	D	S	A	V	P	K	R	Q	D	Y	V	L	T
A	M	A	V	P	V	G	K	K	D	S	A	V	P	K	R	Q	D	Y	V	L	T
A	M	A	V	P	V	G	K	K	D	S	A	V	P	K	R	Q	D	Y	V	L	T
A	M	A	V	P	V	G	K	K	D	S	A	V	P	K	R	Q	D	Y	V	L	T
A	M	A	V	P	V	G	K	K	D	S	A	V	P	K	R	Q	D	Y	V	L	T
A	M	A	V	P	V	G	K	K	D	S	A	V	P	K	R	Q	D	Y	V	L	T
A	M	A	V	P	V	G	K	K	D	S	A	V	P	K	R	Q	D	Y	V	L	T
A	M	A	V	P	V	G	K	K	D	S	A	V	P	K	R	Q	D	Y	V	L	T
A	M	A	V	P	V	G	K	K	D	S	A	V	P	K	R	Q	D	Y	V	L	T
A	M	A	V	P	V	G	K	K	D	S	A	V	P	K	R	Q	D	Y	V	L	T
A	M	A	V	P	V	G	K	K	D	S	A	V	P	K	R	Q	D	Y	V	L	T
A	M	A	V	P	V	G	K	K	D	S	A	V	P	K	R	Q	D	Y	V	L	T
A	M	A	V	P	V	G	K	K	D	S	A	V	P	K	R	Q	D	Y	V	L	T
A	M	A	V	P	V	G	K	K	D	S	A	V	P	K	R	Q	D	Y	V	L	T
A	M	A	V	P	V	G	K	K	D	S	A	V	P	K	R	Q	D	Y	V	L	T
A	M	A	V	P	V	G	K	K	D	S	A	V	P	K	R	Q	D	Y	V	L	T
A	M	A	V	P	V	G	K	K	D	S	A	V	P	K	R	Q	D	Y	V	L	T

A	M
A	M
A	M
A	M
A	M
A	M
A	M
A	M
A	M
A	M

A	V	P	V	G	K	K	D	S	A	V	P	K	R	Q	D	Y	V	L	T
A	V	P	V	G	K	K	D	S	A	V	P	K	R	Q	D	Y	V	L	T
A	V	P	V	G	K	K	D	S	A	V	P	K	R	Q	D	Y	V	L	T
A	V	P	V	G	K	K	D	S	A	V	P	K	R	Q	D	Y	V	L	T
A	V	P	V	G	K	K	D	S	A	V	P	K	R	Q	D	Y	V	L	T
A	V	P	V	G	K	K	D	S	A	V	P	K	R	Q	D	Y	V	L	T
A	V	P	V	G	K	K	D	S	A	V	P	K	R	Q	D	Y	V	L	T
A	V	P	V	G	K	K	D	S	A	V	P	K	R	Q	D	Y	V	L	T
A	V	P	V	G	K	K	D	S	A	V	P	K	R	Q	D	Y	V	L	T
A	V	P	V	G	K	K	D	S	A	V	P	K	R	Q	D	Y	V	L	T

wzh										
123	142	162	170	176	190	191	194	195	203	218
S	A	V	P	R	Q	N	Y	V	L	T
N	V	I	S	P	R	D	H	I	V	S
S	A	V	P	R	Q	D	H	V	L	T
N	V	I	S	P	R	D	H	I	V	S
N	V	I	S	P	R	D	H	I	V	S
N	V	I	S	P	R	D	H	I	V	S
N	V	I	S	P	R	D	H	I	V	S
N	V	I	S	P	R	D	H	I	V	S
N	V	I	S	P	R	D	H	I	V	S
N	V	I	S	P	R	D	H	I	V	S
N	V	I	S	P	R	D	H	I	V	S

15	16	21	22	25	34	63	64	66	92	108
L	F	Q	R	M	S	G	D	P	A	T
V	L	K	H	I	G	E	G	S	V	A
L	F	Q	R	M	S	G	D	P	A	T
V	L	K	H	I	G	E	G	S	V	A
V	L	K	H	I	G	E	G	S	V	A
V	L	K	H	I	G	E	G	S	V	A
V	L	K	H	I	G	E	G	S	V	A
V	L	K	H	I	G	E	G	S	V	A
V	L	K	H	I	G	E	G	S	V	A
V	L	K	H	I	G	E	G	S	V	A
V	L	K	H	I	G	E	G	S	V	A

wzh									
142	162	170	176	190	191	194	195	203	218
A	V	P	R	Q	N	Y	V	L	T
V	I	S	P	R	D	H	I	V	S
A	V	P	R	Q	D	Y	V	L	T
A	V	P	R	Q	D	Y	V	L	T
A	V	P	R	Q	D	Y	V	L	T
A	V	P	R	Q	D	Y	V	L	T
A	V	P	R	Q	D	Y	V	L	T
A	V	P	R	Q	D	Y	V	L	T
A	V	P	R	Q	D	Y	V	L	T
A	V	P	R	Q	D	Y	V	L	T
A	V	P	R	Q	D	Y	V	L	T
A	V	P	R	Q	D	Y	V	L	T
A	V	P	R	Q	D	Y	V	L	T
A	V	P	R	Q	D	Y	V	L	T
A	V	P	R	Q	D	Y	V	L	T
A	V	P	R	Q	D	Y	V	L	T
A	V	P	R	Q	D	Y	V	L	T
A	V	P	R	Q	D	Y	V	L	T
A	V	P	R	Q	D	Y	V	L	T
A	V	P	R	Q	D	Y	V	L	T
A	V	P	R	Q	D	Y	V	L	T
A	V	P	R	Q	D	Y	V	L	T
A	V	P	R	Q	D	Y	V	L	T
A	V	P	R	Q	D	Y	V	L	T
A	V	P	R	Q	D	Y	V	L	T
A	V	P	R	Q	D	Y	V	L	T
A	V	P	R	Q	D	Y	V	L	T
A	V	P	R	Q	D	Y	V	L	T
A	V	P	R	Q	D	Y	V	L	T
A	V	P	R	Q	D	Y	V	L	T
A	V	P	R	Q	D	Y	V	L	T

15	16	21	22	25	34	63	64	66	92	108	110
L	F	Q	R	M	S	G	D	P	A	T	A
V	L	K	H	I	G	E	G	S	V	A	T
L	F	Q	R	M	S	G	D	P	A	T	A
L	F	Q	R	M	S	G	D	P	A	T	A
L	F	Q	R	M	S	G	D	P	A	T	A
L	F	Q	R	M	S	G	D	P	A	T	A
L	F	Q	R	M	S	G	D	P	A	T	A
L	F	Q	R	M	S	G	D	P	A	T	A
L	F	Q	R	M	S	G	D	P	A	T	A
L	F	Q	R	M	S	G	D	P	A	T	A
L	F	Q	R	M	S	G	D	P	A	T	A
L	F	Q	R	M	S	G	D	P	A	T	A
L	F	Q	R	M	S	G	D	P	V	T	A
L	F	Q	R	M	S	G	D	P	A	T	A
L	F	Q	R	M	S	G	D	P	A	T	A
L	F	Q	R	M	S	G	D	P	A	T	A
L	F	Q	R	M	S	G	D	P	A	T	A
L	F	Q	R	M	S	G	D	P	A	T	A
L	F	Q	R	M	S	G	D	P	A	T	A
L	F	Q	R	M	S	G	D	P	A	T	A
L	F	Q	R	M	S	G	D	P	A	T	A
L	F	Q	R	M	S	G	D	P	A	T	A
L	F	Q	R	M	S	G	D	P	A	T	A
L	F	Q	R	M	S	G	D	P	A	T	A
L	F	Q	R	M	S	G	D	P	A	T	A
L	F	Q	R	M	S	G	D	P	A	T	A
L	F	Q	R	M	S	G	D	P	A	T	A
L	F	Q	R	M	S	G	D	P	A	T	A
L	F	Q	R	M	S	G	D	P	A	T	A
L	F	Q	R	M	S	G	D	P	A	T	A
L	F	Q	R	M	S	G	D	P	A	T	A

wciP			
126	128	158	318
M	M	H	T
L	T	Q	A
M	M	H	T
M	T	H	T
M	T	H	T
M	T	H	T
M	T	H	T
M	T	H	T
M	T	H	T
M	T	H	T
M	T	H	T
M	T	H	T
M	T	H	T
M	T	H	T
M	T	H	T
M	T	H	T
M	T	H	T
M	T	H	T
M	T	H	T
M	T	H	T
M	T	H	T
M	T	H	T
M	T	H	T
M	T	H	T
M	T	H	T
M	T	H	T
M	T	H	T
M	T	H	T
M	T	H	T
M	T	H	T
M	T	H	T
M	T	H	T
M	T	H	T
M	T	H	T
M	T	H	T

wzy										
34	35	60	70	89	107	117	178	220	250	403
I	A	Y	V	H	T	I	M	S	T	G
L	T	G	I	Y	I	T	I	A	A	S
I	A	Y	V	H	T	I	M	S	T	G
I	A	Y	V	H	T	I	M	S	T	G
I	A	Y	V	H	T	I	M	S	T	G
I	A	Y	V	H	T	I	M	S	T	G
I	A	Y	V	H	T	I	M	S	T	G
I	A	Y	V	H	T	I	M	S	T	G
I	A	Y	V	H	T	I	M	S	T	G
I	A	Y	V	H	T	I	M	S	T	G
I	A	Y	V	H	T	I	M	S	T	G
I	A	Y	V	H	T	I	M	S	T	G
I	A	Y	V	H	T	I	M	S	T	G
I	A	Y	V	H	T	I	M	S	T	G
I	A	Y	V	H	T	I	M	S	T	G
I	A	Y	V	H	T	I	M	S	T	G
I	A	Y	V	H	T	I	M	S	T	G
I	A	Y	V	H	T	I	M	S	T	G
I	A	Y	V	H	T	I	M	S	T	G
I	A	Y	V	H	T	I	M	S	T	G
I	A	Y	V	H	T	I	M	S	T	G
I	A	Y	V	H	T	I	M	S	T	G
I	A	Y	V	H	T	I	M	S	T	G
I	A	Y	V	H	T	I	M	S	T	G
I	A	Y	V	H	T	I	M	S	T	G
I	A	Y	V	H	T	I	M	S	T	G
I	A	Y	V	H	T	I	M	S	T	G
I	A	Y	V	H	T	I	M	S	T	G
I	A	Y	V	H	T	I	M	S	T	G
I	A	Y	V	H	T	I	M	S	T	G
I	A	Y	V	H	T	I	M	S	T	G
I	A	Y	V	H	T	I	M	S	T	G
I	A	Y	V	H	T	I	M	S	T	G
I	A	Y	V	H	T	I	M	S	T	G
I	A	Y	V	H	T	I	M	S	T	G

wzx		
89	254	370
F	T	L
F	M	L
F	T	L
S	T	F
S	T	F
S	T	F
S	T	F
S	T	F
S	T	F
S	T	F
S	T	F
S	T	F
S	T	F
S	T	F
S	T	F
S	T	F
S	T	F
S	T	F
S	T	F
S	T	F
S	T	F
S	T	F
S	T	F
S	T	F
S	T	F
S	T	F
S	T	F
S	T	F
S	T	F
S	T	F
S	T	F
S	T	F
S	T	F
S	T	F
S	T	F

118	136
T	H
K	Q
T	H
K	H
K	H
K	H
K	H
K	H
K	H
K	H
K	H
K	H
K	H
K	H
K	H
K	H
K	H
K	H
K	H
K	H
K	H
K	H
K	H
K	H
K	H
K	H
K	H
K	H
K	H
K	H
K	H
K	H
K	H
K	H
K	H

191	194	203	205	218	224	228
N	Y	L	G	T	A	E
D	H	L	G	T	A	E
D	H	V	S	S	T	D
D	H	L	G	T	A	E

														wzd
15	20	21	24	33	41	62	63	83	91	103	105	109	115	142
F	Q	R	M	S	T	G	D	R	A	M	A	A	A	L
F	Q	R	M	S	T	G	D	R	A	M	A	A	A	L
L	K	H	I	G	I	E	N	H	V	I	V	T	T	I
F	Q	R	M	S	T	G	D	R	A	M	A	A	A	L

wzh

170	171	176	190	191	194	195	203	218
P	K	R	Q	N	Y	V	L	T
S	K	P	R	D	H	I	V	S
P	R	R	Q	D	Y	V	L	T
P	R	R	Q	D	Y	V	L	T
P	R	R	Q	D	Y	V	L	T
P	R	R	Q	D	Y	V	L	T
P	R	R	Q	D	Y	V	L	T
P	R	R	Q	D	Y	V	L	T
P	R	R	Q	D	Y	V	L	T
P	R	R	Q	D	Y	V	L	T
P	R	R	Q	D	Y	V	L	T
P	R	R	Q	D	Y	V	L	T
P	R	R	Q	D	Y	V	L	T
P	R	R	Q	D	Y	V	L	T
P	R	R	Q	D	Y	V	L	T
P	R	R	Q	D	Y	V	L	T
P	R	R	Q	D	Y	V	L	T
P	R	R	Q	D	Y	V	L	T
P	R	R	Q	D	Y	V	L	T
P	R	R	Q	D	Y	V	L	T
P	R	R	Q	D	Y	V	L	T
P	R	R	Q	D	Y	V	L	T
P	R	R	Q	D	Y	V	L	T
P	R	R	Q	D	Y	V	L	T
P	R	R	Q	D	Y	V	L	T
P	R	R	Q	D	Y	V	L	T
P	R	R	Q	D	Y	V	L	T

15	16	21	22	25	34	35	63	64	66	92	108	110
L	F	Q	R	M	S	A	G	D	P	A	T	A
V	L	K	H	I	G	A	E	G	S	V	A	T
L	F	Q	R	M	S	V	G	D	P	A	T	A
L	F	Q	R	M	S	V	G	D	P	A	T	A
L	F	Q	R	M	S	V	G	D	P	A	T	A
L	F	Q	R	M	S	V	G	D	P	A	T	A
L	F	Q	R	M	S	V	G	D	P	A	T	A
L	F	Q	R	M	S	V	G	D	P	A	T	A
L	F	Q	R	M	S	V	G	D	P	A	T	A
L	F	Q	R	M	S	V	G	D	P	A	T	A
L	F	Q	R	M	S	V	G	D	P	A	T	A
L	F	Q	R	M	S	V	G	D	P	A	T	A
L	F	Q	R	M	S	V	G	D	P	A	T	A
L	F	Q	R	M	S	V	G	D	P	A	T	A
L	F	Q	R	M	S	V	G	D	P	A	T	A
L	F	Q	R	M	S	V	G	D	P	A	T	A
L	F	Q	R	M	S	V	G	D	P	A	T	A
L	F	Q	R	M	S	V	G	D	P	A	T	A
L	F	Q	R	M	S	V	G	D	P	A	T	A
L	F	Q	R	M	S	V	G	D	P	A	T	A
L	F	Q	R	M	S	V	G	D	P	A	T	A
L	F	Q	R	M	S	V	G	D	P	A	T	A
L	F	Q	R	M	S	V	G	D	P	A	T	A
L	F	Q	R	M	S	V	G	D	P	A	T	A
L	F	Q	R	M	S	V	G	D	P	A	T	A
L	F	Q	R	M	S	V	G	D	P	A	T	A
L	F	Q	R	M	S	V	G	D	P	A	T	A

	wzd									
109	115	151	180	182	189	194	211	216	217	222
A	A	V	S	F	V	A	I	K	I	L
A	A	V	S	F	V	A	V	K	I	L
T	T	I	T	V	A	T	V	Q	M	V
A	A	V	S	F	V	A	I	K	I	L
A	A	V	S	F	V	A	I	K	I	L
A	A	V	S	F	V	A	I	K	I	L
A	A	V	S	F	V	A	I	K	I	L
A	A	V	S	F	V	A	I	K	I	L
A	A	V	S	F	V	A	I	K	I	L
A	A	V	S	F	V	A	I	K	I	L
A	A	V	S	F	V	A	I	K	I	L
A	A	V	S	F	V	A	I	K	I	L
A	A	V	S	F	V	A	I	K	I	L
A	A	V	S	F	V	A	I	K	I	L
A	A	V	S	F	V	A	I	K	I	L
A	A	V	S	F	V	A	I	K	I	L
A	A	V	S	F	V	A	I	K	I	L
A	A	V	S	F	V	A	I	K	I	L
A	A	V	S	F	V	A	I	K	I	L
A	A	V	S	F	V	A	I	K	I	L
A	A	V	S	F	V	A	I	K	I	L
A	A	V	S	F	V	A	I	K	I	L
A	A	V	S	F	V	A	I	K	I	L
A	A	V	S	F	V	A	I	K	I	L
A	A	V	S	F	V	A	I	K	I	L
A	A	V	S	F	V	A	I	K	I	L
A	A	V	S	F	V	A	I	K	I	L
A	A	V	S	F	V	A	I	K	I	L
A	A	V	S	F	V	A	I	K	I	L

9	12	13	14	34	45	50	72	74	80	85
A	D	S	V	G	I	S	G	I	L	A
A	D	S	V	G	L	S	G	I	L	A
V	E	L	A	D	A	T	A	M	S	S
A	D	S	V	G	I	S	G	I	L	A
A	D	S	V	G	I	S	G	I	L	A
A	D	S	V	G	I	S	G	I	L	A
A	D	S	V	G	I	S	G	I	L	A
A	D	S	V	G	I	S	G	I	L	A
A	D	S	V	G	I	S	G	I	L	A
A	D	S	V	G	I	S	G	I	L	A
A	D	S	V	G	I	S	G	I	L	A
A	D	S	V	G	I	S	G	I	L	A
A	D	S	V	G	I	S	G	I	L	A
A	D	S	V	G	I	S	G	I	L	A
A	D	S	V	G	I	S	G	I	L	A
A	D	S	V	G	I	S	G	I	L	A
A	D	S	V	G	I	S	G	I	L	A
A	D	S	V	G	I	S	G	I	L	A
A	D	S	V	G	I	S	G	I	L	A
A	D	S	V	G	I	S	G	I	L	A
A	D	S	V	G	I	S	G	I	L	A
A	D	S	V	G	I	S	G	I	L	A
A	D	S	V	G	I	S	G	I	L	A
A	D	S	V	G	I	S	G	I	L	A
A	D	S	V	G	I	S	G	I	L	A
A	D	S	V	G	I	S	G	I	L	A
A	D	S	V	G	I	S	G	I	L	A
A	D	S	V	G	I	S	G	I	L	A
A	D	S	V	G	I	S	G	I	L	A

A	A	V	S	F	V	A	I	K	I	L
A	A	V	S	F	V	A	I	K	I	L

A	D	S	V	G	I	S	G	I	L	A
A	D	S	V	G	I	S	G	I	L	A

		wciN		HG262		wciO	wciP					wzy					
384	421	52	289	36	56	76	128	158	254	273	276	70	74	107	178	220	250
D	V	S	L	Y	-	V	M	H	R	S	L	V	L	T	M	S	T
D	V	S	L	Y	-	V	T	H	R	S	L	V	L	T	M	S	T
D	V	S	L	Y	-	V	T	H	R	S	L	V	L	T	M	S	T
D	V	S	L	Y	-	V	T	H	R	S	L	V	L	T	M	S	T
D	V	S	L	Y	-	V	T	H	R	S	L	V	L	T	M	S	T
D	V	S	L	Y	-	V	T	H	R	S	L	V	L	T	M	S	T
D	V	S	L	Y	-	V	T	H	R	S	L	V	L	T	M	S	T
D	V	S	L	Y	-	V	T	H	R	S	L	V	L	T	M	S	T
D	V	S	L	Y	-	V	T	H	R	S	L	V	L	T	M	S	T
D	V	S	L	Y	-	V	T	H	R	S	L	V	L	T	M	S	T
D	V	S	L	Y	-	V	T	H	R	S	L	V	L	T	M	S	T
D	V	S	L	Y	-	V	T	H	R	S	L	V	L	T	M	S	T
D	V	S	L	Y	-	V	T	H	R	S	L	V	L	T	M	S	T
D	V	S	L	Y	-	V	T	H	R	S	L	V	L	T	M	S	T
D	V	S	L	Y	-	V	T	H	M	S	L	V	L	T	M	S	T
D	V	S	L	Y	-	V	T	H	M	S	L	V	L	T	M	S	T
D	V	S	L	Y	-	V	T	H	M	S	L	V	L	T	M	S	T
D	V	S	L	Y	-	V	T	H	M	S	L	V	L	T	M	S	T
D	I	S	L	Y	-	V	T	H	M	S	R	V	L	T	M	S	T
D	V	S	L	Y	-	V	T	Q	M	S	L	V	L	T	M	S	T
D	V	S	L	Y	-	V	T	H	M	S	L	V	L	T	M	S	T
D	V	S	L	Y	-	V	T	H	M	S	L	V	L	T	M	S	T
D	V	S	L	Y	-	V	T	H	M	S	L	V	L	T	M	S	T
D	V	S	L	Y	-	V	T	H	M	S	L	V	L	T	M	S	T
D	V	S	L	Y	-	V	T	H	M	S	L	V	L	T	M	S	T
D	I	S	L	Y	-	V	T	H	M	S	R	V	L	T	M	S	T
D	V	S	L	Y	-	V	T	H	M	S	L	V	L	T	M	S	T
N	V	S	L	Y	-	V	T	H	M	S	L	V	L	T	M	S	T
D	V	S	L	Y	-	V	T	H	M	S	L	V	L	T	M	S	T
D	V	S	L	Y	-	V	T	H	M	S	L	V	L	T	M	S	T
D	V	S	L	Y	-	V	T	H	M	S	L	V	L	T	M	S	T
D	V	S	L	Y	-	V	T	H	R	S	L	V	L	T	M	S	T
D	V	S	L	Y	-	V	T	H	R	S	L	V	L	T	M	S	T
D	V	S	L	Y	-	V	T	H	R	S	L	V	L	T	M	S	T
D	V	S	L	Y	-	V	T	H	R	S	L	V	L	T	M	S	T
D	V	S	L	Y	-	V	T	H	R	S	L	V	F	T	M	S	T
D	V	S	L	Y	-	V	T	H	R	S	L	V	L	T	M	S	T
D	V	S	L	Y	-	V	T	H	R	S	L	V	L	T	M	S	T
D	V	S	L	Y	-	V	T	H	R	S	L	V	L	T	M	S	T
D	V	S	L	Y	-	V	T	H	R	S	L	V	L	T	M	S	T
D	V	S	L	Y	-	V	T	H	R	S	L	V	L	T	M	S	T
D	V	S	L	Y	-	V	T	H	R	S	L	V	L	T	M	S	T
D	V	S	L	Y	-	V	T	H	R	S	L	V	F	T	M	S	T
D	V	S	L	Y	-	V	T	H	R	S	L	V	L	T	M	S	T
D	V	S	L	Y	-	V	T	H	R	S	L	V	L	T	M	S	T
D	V	S	L	Y	-	V	T	H	R	S	L	V	L	T	M	S	T
D	V	S	L	Y	-	V	T	H	R	S	L	V	L	T	M	S	T
D	V	S	L	Y	-	V	T	H	R	S	L	V	F	T	M	S	T
D	V	S	L	Y	-	V	T	H	R	S	L	V	F	T	M	S	T
D	V	S	L	Y	-	V	T	H	R	S	L	V	L	T	M	S	T
D	V	S	L	Y	-	V	T	H	R	S	L	V	L	T	M	S	T
D	V	S	L	Y	-	V	T	H	R	S	L	V	L	T	M	S	T
D	V	S	L	Y	-	V	T	H	R	S	L	V	L	T	M	S	T
D	V	S	L	Y	-	V	T	H	R	S	L	V	L	T	M	S	T
D	V	S	L	Y	-	V	T	H	R	S	L	V	L	T	M	S	T
D	V	S	L	Y	-	V	T	H	R	S	L	V	L	T	M	S	T

D	V	S	L	Y	-	V	T	H	R	S	L	V	L	T	M	S	T
D	V	S	L	Y	-	V	T	H	R	S	L	V	L	T	M	S	T
D	V	S	L	Y	-	V	T	H	R	S	L	V	L	T	M	S	T
D	V	S	L	Y	-	V	T	H	R	S	L	V	L	T	M	S	T
D	V	S	L	Y	-	V	T	H	R	S	L	V	L	T	M	S	T
D	V	S	L	Y	-	V	T	H	R	S	L	V	L	T	M	S	T
D	V	S	L	Y	-	V	T	H	R	S	L	V	L	T	M	S	T
D	V	S	L	Y	-	V	T	H	R	S	L	V	L	T	M	S	T
D	V	S	L	Y	-	V	T	H	R	S	L	V	F	T	M	S	T
D	V	S	L	Y	-	V	T	H	R	S	L	V	L	T	M	S	T
D	V	S	L	Y	-	V	T	H	R	S	L	V	L	T	M	S	T
D	V	S	L	Y	-	V	T	H	R	S	L	V	L	T	M	S	T
D	V	S	L	Y	-	V	T	H	R	S	L	V	F	T	M	S	T
D	V	S	L	Y	-	V	T	H	R	S	L	V	L	T	M	S	T
D	V	S	L	Y	-	V	T	H	R	S	L	V	L	T	M	S	T
D	V	S	L	Y	-	V	T	H	R	S	L	V	L	T	M	S	T
D	V	S	L	Y	-	V	T	H	R	S	L	V	L	T	M	S	T
D	V	S	L	Y	-	V	T	H	R	S	L	V	F	T	M	S	T
D	V	S	L	Y	-	V	T	H	R	S	L	V	L	T	M	S	T
D	V	S	L	Y	-	V	T	H	R	S	L	V	L	T	M	S	T
D	V	S	L	Y	-	V	T	H	R	S	L	V	L	T	M	S	T
D	V	S	L	Y	-	V	T	H	R	S	L	V	F	T	M	S	T
D	V	S	L	Y	-	V	T	H	R	S	L	V	L	T	M	S	T
D	V	S	L	Y	-	V	T	H	R	S	L	V	F	T	M	S	T
D	V	S	L	Y	-	V	T	H	R	S	L	V	L	T	M	S	T
D	V	S	L	Y	-	V	T	H	R	S	L	V	L	T	M	S	T
D	V	S	L	Y	-	V	T	H	R	S	L	V	L	T	M	S	T
D	V	S	L	Y	-	V	T	H	R	S	L	V	L	T	M	S	T
D	V	S	L	Y	-	V	T	H	R	S	L	V	L	T	M	S	T
D	V	S	L	Y	-	V	T	H	R	S	L	V	F	T	M	S	T
D	V	S	L	Y	-	V	T	H	R	S	L	V	F	T	M	S	T
D	V	S	L	Y	-	V	T	H	R	S	L	V	F	T	M	S	T
D	V	S	L	Y	-	V	T	H	R	S	L	V	L	T	M	S	T
D	V	S	L	Y	-	V	T	H	R	S	L	V	L	T	M	S	T
D	V	S	L	Y	-	V	T	H	R	S	L	V	L	T	M	S	T
D	V	S	L	Y	-	V	T	H	R	S	L	V	L	T	M	S	T
D	V	S	L	Y	-	V	T	H	R	S	L	V	L	T	M	S	T
D	V	S	L	Y	-	V	T	H	R	S	L	V	L	T	M	S	T
D	V	S	L	Y	-	V	T	H	R	S	L	V	L	T	M	S	T
D	V	S	L	Y	-	V	T	H	R	S	L	V	L	T	M	S	T
D	V	S	L	Y	-	V	T	H	R	S	L	V	F	T	M	S	T
D	V	S	L	Y	-	V	T	H	R	S	L	V	F	T	M	S	T
D	V	S	L	Y	-	V	T	H	R	S	L	V	L	T	M	S	T
D	V	S	L	Y	-	V	T	H	R	S	L	V	L	T	M	S	T
D	V	S	L	Y	-	V	T	H	R	S	L	V	L	T	M	S	T
D	V	S	L	Y	-	V	T	H	R	S	L	V	L	T	M	S	T
D	V	S	L	Y	-	V	T	H	R	S	L	V	L	T	M	S	T
D	V	S	L	Y	-	V	T	H	R	S	L	V	L	T	M	S	T
D	V	S	L	Y	-	V	T	H	R	S	L	V	L	T	M	S	T
D	V	S	L	Y	-	V	T	H	R	S	L	V	L	T	M	S	T
D	V	S	L	Y	-	V	T	H	R	S	L	V	L	T	M	S	T
D	V	S	L	Y	-	V	T	H	R	S	L	V	L	T	M	S	T
D	V	S	L	Y	-	V	T	H	R	I	L	V	L	T	M	S	T
D	V	S	L	Y	-	V	T	H	R	S	L	V	L	T	M	S	T
D	V	S	L	Y	-	V	T	H	R	S	L	V	L	T	M	S	T
D	V	S	L	Y	-	V	T	H	R	S	L	V	L	T	M	S	T
D	V	S	L	Y	-	V	T	H	R	S	L	V	L	T	M	S	T
D	V	S	L	Y	-	V	T	H	R	S	L	V	L	T	M	S	T
D	V	S	L	Y	-	V	T	H	R	S	L	V	L	T	M	S	T
D	V	S	L	Y	-	V	T	H	R	S	L	V	L	T	M	S	T
D	V	S	L	Y	-	V	T	H	R	S	L	V	L	T	M	S	T
D	V	S	L	Y	-	V	T	H	R	S	L	V	L	T	M	S	T
D	V	S	L	Y	-	V	T	H	R	S	L	V	L	T	M	S	T
D	V	S	L	Y	-	V	T	H	R	S	L	V	L	T	M	S	T
D	V	S	L	Y	-	V	T	H	R	S	L	V	L	T	M	S	T
D	V	S	L	Y	-	V	T	H	R	S	L	V	L	T	M	S	T
D	V	S	L	Y	-	V	T	H	R	S	L	V	L	T	M	S	T

D	V	S	L	Y	-	V	T	H	R	S	L	V	L	T	M	S	T
D	V	S	L	Y	-	V	T	H	R	S	L	V	L	T	M	S	T
D	V	S	L	Y	-	V	T	H	R	S	L	V	L	T	M	S	T
D	V	S	L	Y	-	V	T	H	R	I	L	V	L	T	M	S	T
D	V	S	L	Y	-	V	T	H	R	S	L	V	L	T	M	S	T
D	V	S	L	Y	-	V	T	H	R	S	L	V	L	T	M	S	T
D	V	S	L	Y	-	V	T	H	R	S	L	V	L	T	M	S	T
D	V	S	L	Y	-	V	T	H	R	S	L	V	L	T	M	S	T
D	V	S	L	Y	-	V	T	H	R	S	L	V	L	T	M	S	T
D	V	S	L	Y	-	I	T	H	R	S	L	V	L	T	M	S	T
D	V	S	L	Y	-	I	T	H	R	S	L	V	L	T	M	S	T
D	V	S	L	Y	-	V	T	H	R	S	L	V	L	T	M	S	T
D	V	A	L	Y	D	V	T	H	R	S	L	I	L	I	I	A	A
D	V	A	L	Y	D	V	T	H	R	S	L	I	L	I	I	A	A
D	V	A	L	Y	D	V	T	H	R	S	L	I	L	I	I	A	A
D	V	A	L	Y	D	V	T	H	R	S	L	I	L	I	I	A	A
D	V	A	L	Y	D	V	T	H	R	S	L	I	L	I	I	A	A
D	V	A	L	Y	D	V	T	H	R	S	L	I	L	I	I	A	A
D	V	A	L	Y	D	V	T	H	R	S	L	I	L	I	I	A	A
D	V	A	L	Y	D	V	T	H	R	S	L	I	L	I	I	A	A
D	V	A	L	Y	D	V	T	H	R	S	L	I	L	I	I	A	A
D	V	S	L	D	-	V	T	H	R	S	L	V	L	T	M	S	T
D	V	S	L	D	-	V	T	H	R	S	L	V	L	T	M	S	T
D	V	S	L	D	-	V	T	H	R	S	L	V	L	T	M	S	T
D	V	S	L	D	-	V	T	H	R	S	L	V	L	T	M	S	T
D	V	S	L	D	-	V	T	H	R	S	L	V	L	T	M	S	T
D	V	S	L	D	-	V	T	H	R	S	L	V	L	T	M	S	T
D	V	S	L	D	-	V	T	H	R	S	L	V	L	T	M	S	T
D	V	S	L	D	-	V	T	H	R	S	L	V	L	T	M	S	T
D	V	S	L	D	-	V	T	H	R	S	L	V	L	T	M	S	T
D	V	S	L	D	-	V	T	H	R	S	L	V	L	T	M	S	T
D	V	S	L	D	-	V	T	H	R	S	L	V	L	T	M	S	T
D	V	S	L	D	-	V	T	H	R	S	L	V	L	T	M	S	T
D	V	S	L	D	-	V	T	H	R	S	L	V	L	T	M	S	T
D	V	S	L	D	-	V	T	H	R	S	L	V	L	T	M	S	T
D	V	S	L	D	-	V	T	H	R	S	L	V	L	T	M	S	T
D	V	S	L	D	-	V	T	H	R	S	L	V	L	T	M	S	T
D	V	S	L	D	-	V	T	H	R	S	L	V	L	T	M	S	T
D	V	S	L	D	-	V	T	H	R	S	L	V	L	T	M	S	T
D	V	S	L	D	-	V	T	H	R	S	L	V	L	T	M	S	T
D	V	S	L	D	-	V	T	H	R	S	L	V	L	T	M	S	T
D	V	S	L	D	-	V	T	H	R	S	L	V	L	T	M	S	T
D	V	S	L	D	-	V	T	H	R	S	L	V	L	T	M	S	T
D	V	S	L	D	-	V	T	H	R	S	L	V	L	T	M	S	T
D	V	S	L	D	-	V	T	H	R	S	L	V	L	T	M	S	T
D	V	S	L	D	-	V	T	H	R	S	L	V	L	T	M	S	T
D	V	S	L	D	-	V	T	H	R	S	L	V	L	T	M	S	T
D	V	S	L	D	-	V	T	H	R	S	L	V	L	T	M	S	T
D	V	S	L	D	-	V	T	H	R	S	L	V	L	T	M	S	T
D	V	S	L	Y	-	V	M	H	R	S	L	V	L	T	M	S	T
D	V	S	L	Y	-	V	M	H	R	S	L	V	L	T	M	S	T
D	V	S	L	Y	-	V	M	H	R	S	L	V	L	T	M	S	T
D	V	S	L	Y	-	V	M	H	R	S	L	V	L	T	M	S	T
D	V	S	L	Y	-	V	M	H	R	S	L	V	L	T	M	S	T
D	V	S	L	Y	-	V	M	H	R	S	L	V	L	T	M	S	T
D	V	S	L	Y	-	V	M	H	R	S	L	V	L	T	M	S	T
D	V	S	L	Y	-	V	M	H	R	S	L	V	L	T	M	S	T
D	V	S	L	Y	-	V	M	H	R	S	L	V	L	T	M	S	T
D	V	S	L	Y	-	V	M	H	R	S	L	V	L	T	M	S	T
D	V	S	L	Y	-	V	M	H	R	S	L	V	L	T	M	S	T
D	V	S	L	Y	-	V	M	H	R	S	L	V	L	T	M	S	T
D	V	S	L	Y	-	V	M	H	R	S	L	V	L	T	M	S	T
D	V	S	L	Y	-	V	M	H	R	S	L	V	L	T	M	S	T
D	V	S	L	Y	-	V	M	H	R	S	L	V	L	T	M	S	T
D	V	S	L	Y	-	V	M	H	R	S	L	V	L	T	M	S	T
D	V	S	L	Y	-	V	M	H	R	S	L	V	L	T	M	S	T
D	V	S	L	Y	-	V	M	H	R	S	L	V	L	T	M	S	T
D	V	S	L	Y	-	V	M	H	R	S	L	V	L	T	M	S	T

D	V	S	L	Y	-	V	M	H	R	S	L	V	L	T	M	S	T
D	V	S	L	Y	-	V	M	H	R	S	L	V	L	T	M	S	T
D	V	S	L	Y	-	V	M	H	R	S	L	V	L	T	M	S	T
D	V	S	L	Y	-	V	M	H	R	S	L	V	L	T	M	S	T
D	V	S	L	Y	-	V	M	H	R	S	L	V	L	T	M	S	T
D	V	S	L	Y	-	V	M	H	R	S	L	V	L	T	M	S	T
D	V	S	L	Y	-	V	M	H	R	S	L	V	L	T	M	S	T
D	V	S	L	Y	-	V	M	H	R	S	L	V	L	T	M	S	T
D	V	S	L	Y	-	V	M	H	R	S	L	V	L	T	M	S	T
D	V	S	L	Y	-	V	M	H	R	S	L	V	L	T	M	S	T
D	V	S	L	Y	-	V	M	H	R	S	L	V	L	T	M	S	T
D	V	S	L	Y	-	V	M	H	R	S	L	V	L	T	M	S	T
D	V	S	L	Y	-	V	M	H	R	S	L	V	L	T	M	S	T
D	V	S	L	Y	-	V	M	H	R	S	L	V	L	T	M	S	T
D	V	S	L	Y	-	V	M	H	R	S	L	V	L	T	M	S	T
D	V	S	L	Y	-	V	M	H	R	S	L	V	L	T	M	S	T
D	V	S	L	Y	-	V	M	H	R	S	L	V	L	T	M	S	T
D	V	S	L	Y	-	V	M	H	R	S	L	V	L	T	M	S	T
D	V	S	L	Y	-	V	M	H	R	S	L	V	L	T	M	S	T
D	V	S	L	Y	-	V	M	H	R	S	L	V	L	T	M	S	T
D	V	S	L	Y	-	V	M	H	R	S	L	V	L	T	M	S	T
D	V	S	L	Y	-	V	M	H	R	S	L	V	L	T	M	S	T
D	V	S	L	Y	-	V	M	H	R	S	L	V	L	T	M	S	T
D	V	S	L	Y	-	V	M	H	R	S	L	V	L	T	M	S	T
D	V	S	L	Y	-	V	M	H	R	S	L	V	L	T	M	S	T
D	V	S	L	Y	-	V	M	H	R	S	L	V	L	T	M	S	T
D	V	S	L	Y	-	V	M	H	R	S	L	V	L	T	M	S	T
D	V	S	L	Y	-	V	M	H	R	S	L	V	L	T	M	S	T
D	V	S	L	Y	-	V	M	H	R	S	L	V	L	T	M	S	T
D	V	S	L	Y	-	V	M	H	R	S	L	V	L	T	M	S	T
D	V	S	L	Y	-	V	M	H	R	S	L	V	L	T	M	S	T
D	V	S	L	Y	-	V	M	H	R	S	L	V	L	T	M	S	T
D	V	S	L	Y	-	V	M	H	R	S	L	V	L	T	M	S	T
D	V	S	L	Y	-	V	M	H	R	S	L	V	L	T	M	S	T
D	V	S	L	Y	-	V	M	H	R	S	L	V	L	T	M	S	T
D	V	S	L	Y	-	V	M	H	R	S	L	V	L	T	M	S	T
D	V	S	L	Y	-	V	M	H	R	S	L	V	L	T	M	S	T
D	V	S	L	Y	-	V	M	H	R	S	L	V	L	T	M	S	T
D	V	S	L	Y	-	V	M	H	R	S	L	V	L	T	M	S	T
D	V	S	L	Y	-	V	M	H	R	S	L	V	L	T	M	S	T
D	V	S	L	Y	-	V	M	H	R	S	L	V	L	T	M	S	T
D	V	S	L	Y	-	V	M	H	R	S	L	V	L	T	M	S	T
D	V	S	L	Y	-	V	M	H	R	S	L	V	L	T	M	S	T
D	V	S	L	Y	-	V	M	H	R	S	L	V	L	T	M	S	T
D	V	S	L	Y	-	V	M	H	R	S	L	V	L	T	M	S	T
D	V	S	L	Y	-	V	M	H	R	S	L	V	L	T	M	S	T
D	V	S	L	Y	-	V	M	H	R	S	L	V	L	T	M	S	T
D	V	S	L	Y	-	V	M	H	R	S	L	V	L	T	M	S	T
D	V	S	V	Y	-	V	M	H	R	S	L	V	L	T	M	S	T
D	V	S	L	Y	-	V	M	H	R	S	L	V	L	T	M	S	T
D	V	S	L	Y	-	V	M	H	R	S	L	V	L	T	M	S	T
D	V	S	L	Y	-	V	M	H	R	S	L	V	L	T	M	S	T
D	V	S	L	Y	-	V	M	H	R	S	L	V	L	T	M	S	T
D	V	S	L	Y	-	V	M	H	R	S	L	V	L	T	M	S	T
D	V	S	L	Y	-	V	M	H	R	S	L	V	L	T	M	S	T
D	V	S	L	Y	-	V	M	H	R	S	L	V	L	T	M	S	T
D	V	S	L	Y	-	V	M	H	R	S	L	V	L	T	M	S	T
D	V	S	L	Y	-	V	M	H	R	S	L	V	L	T	M	S	T
D	V	S	L	Y	-	V	M	H	R	S	L	V	L	T	M	S	T
D	V	S	L	Y	-	V	M	H	R	S	L	V	L	T	M	S	T
D	V	S	L	Y	-	V	M	H	R	S	L	V	L	T	M	S	T
D	V	S	L	Y	-	V	M	H	R	S	L	V	L	T	M	S	T
D	V	S	L	Y	-	V	M	H	R	S	L	V	L	T	M	S	T
D	V	S	L	Y	-	V	M	H	R	S	L	V	L	T	M	S	T
D	V	S	L	Y	-	V	M	H	R	S	L	V	L	T	M	S	T
D	V	S	L	Y	-	V	M	H	R	S	L	V	L	T	M	S	T
D	V	S	L	Y	-	V	M	H	R	S	L	V	L	T	M	S	T

D	V	S	L	Y	-	V	M	H	R	S	L	V	L	T	M	S	T
D	V	S	L	Y	-	V	M	H	R	S	L	V	L	T	M	S	T
D	V	S	L	Y	-	V	M	H	R	S	L	V	L	T	M	S	T
D	V	S	L	Y	-	V	M	H	R	S	L	V	L	T	M	S	T
D	V	S	L	Y	-	V	M	H	R	S	L	V	L	T	M	S	T
D	V	S	L	Y	-	V	M	H	R	S	L	V	L	T	M	S	T
D	V	S	L	Y	-	V	M	H	R	S	L	V	L	T	M	S	T
D	V	S	L	Y	-	V	M	H	R	S	L	V	L	T	M	S	T
D	V	S	L	Y	-	V	M	H	R	S	L	V	L	T	M	S	T
D	V	S	L	Y	-	V	M	H	R	S	L	V	L	T	M	S	T
D	V	S	L	Y	-	V	M	H	R	S	L	V	L	T	M	S	T
D	V	S	L	Y	-	V	M	H	R	S	L	V	L	T	M	S	T
D	V	S	L	Y	-	V	M	H	R	S	L	V	L	T	M	S	T
D	V	S	L	Y	-	V	M	H	R	S	L	V	L	T	M	S	T
D	V	S	L	Y	-	V	M	H	R	S	L	V	L	T	M	S	T
D	V	S	L	Y	-	V	M	H	R	S	L	V	L	T	M	S	T
D	V	S	L	Y	-	V	M	H	R	S	L	V	L	T	M	S	T
D	V	S	L	Y	-	V	M	H	R	S	L	V	L	T	M	S	T
D	V	S	L	Y	-	V	M	H	R	S	L	V	L	T	M	S	T
D	V	S	L	Y	-	V	M	H	R	S	L	V	L	T	M	S	T
D	V	S	L	Y	-	V	M	H	R	S	L	V	L	T	M	S	T
D	V	S	L	Y	-	V	M	H	R	S	L	V	L	T	M	S	T
D	V	S	L	Y	-	V	M	H	R	S	L	V	L	T	M	S	T
D	V	S	L	Y	-	V	M	H	R	S	L	V	L	T	M	S	T
D	V	S	L	Y	-	V	M	H	R	S	L	V	L	T	M	S	T
D	V	S	L	Y	-	V	M	H	R	S	L	V	L	T	M	S	T
D	V	S	L	Y	-	V	M	H	R	S	L	V	L	T	M	S	T

																		wzd			
14	15	20	21	24	33	34	42	46	62	63	65	66	91	107	109	114	115	118	122	129	131
L	F	Q	R	M	S	A	F	P	G	D	P	G	A	T	A	V	A	A	I	D	Q
L	F	Q	R	M	S	A	F	P	G	D	P	G	A	T	A	V	A	A	I	D	Q
L	F	Q	R	M	S	V	F	P	G	D	P	G	A	T	A	V	A	A	I	D	Q
L	F	Q	R	M	S	V	F	P	G	D	P	G	A	T	A	V	A	A	I	D	Q
L	F	Q	R	M	S	V	F	P	G	D	P	G	A	T	A	V	A	A	I	D	Q
L	F	Q	R	M	S	V	F	P	G	D	P	G	A	T	A	V	A	A	I	D	Q
L	F	Q	R	M	S	V	F	P	G	D	P	G	A	T	A	V	A	A	I	D	Q
L	F	Q	R	M	S	V	F	P	G	D	P	G	A	T	A	V	A	A	I	D	Q
L	F	Q	R	M	S	V	F	P	G	D	P	G	A	T	A	V	A	A	I	D	Q
L	F	Q	R	M	S	V	F	P	G	D	P	G	A	T	A	V	A	A	I	D	Q
L	F	Q	R	M	S	V	F	P	G	D	P	G	A	T	A	V	A	A	I	D	Q
L	F	Q	R	M	S	V	F	P	G	D	P	G	A	T	A	V	A	A	I	D	Q
L	F	Q	R	M	S	V	F	P	G	D	P	G	A	T	A	V	A	A	I	D	Q
L	F	Q	R	M	S	V	F	P	G	D	P	G	A	T	A	V	A	A	I	D	Q
L	F	Q	R	M	S	V	F	P	G	D	P	G	A	T	A	V	A	A	I	D	Q
L	F	Q	R	M	S	V	F	P	G	D	P	G	A	T	A	V	A	A	I	D	Q
L	F	Q	R	M	S	V	F	P	G	D	P	G	A	T	A	V	A	A	I	D	Q
L	F	Q	R	M	S	V	F	P	G	D	P	G	A	T	A	V	A	A	I	D	Q
L	F	Q	R	M	S	V	F	P	G	D	P	G	A	T	A	V	A	A	I	D	Q
L	F	Q	R	M	S	V	F	P	G	D	P	G	A	T	A	V	A	A	I	D	Q
L	F	Q	R	M	S	V	F	P	G	D	P	G	A	T	A	V	A	A	I	D	Q
L	F	Q	R	M	S	V	F	P	G	D	P	G	A	T	A	V	A	A	I	D	Q
L	F	Q	R	M	S	V	F	P	G	D	P	G	A	T	A	V	A	A	I	D	Q
L	F	Q	R	M	S	V	F	P	G	D	P	G	A	T	A	V	A	A	I	D	Q
L	F	Q	R	M	S	V	F	P	G	D	P	G	A	T	A	V	A	A	I	D	Q
L	F	Q	R	M	S	V	F	P	G	D	P	G	A	T	A	V	A	A	I	D	Q
L	F	Q	R	M	S	V	F	P	G	D	P	G	A	T	A	V	A	A	I	D	Q
L	F	Q	R	M	S	V	F	P	G	D	P	G	A	T	A	V	A	A	I	D	Q
L	F	Q	R	M	S	V	F	P	G	D	P	G	A	T	A	V	A	A	I	D	Q
L	F	Q	R	M	S	V	F	P	G	D	P	G	A	T	A	V	A	A	I	D	Q
L	F	Q	R	M	S	A	F	P	G	D	P	G	A	T	A	V	A	A	I	D	Q
L	F	Q	R	M	S	A	F	P	G	D	P	G	A	T	A	V	A	A	I	D	Q
L	F	Q	R	M	S	A	F	P	G	D	P	G	A	T	A	V	A	A	I	D	Q
L	F	Q	R	M	S	A	F	P	G	D	P	G	A	T	A	V	A	A	I	D	Q
L	F	Q	R	M	S	A	F	P	G	D	P	G	A	T	A	V	A	A	I	D	Q
L	F	Q	R	M	S	A	F	P	G	D	P	G	A	T	A	V	A	A	I	D	Q
L	F	Q	R	M	S	A	F	P	G	D	P	G	A	T	A	V	A	A	I	D	Q
L	F	Q	R	M	S	A	F	P	G	D	P	G	A	T	A	V	A	A	I	D	Q
L	F	Q	R	M	S	A	F	P	G	D	P	G	A	T	A	V	A	A	I	D	Q
L	F	Q	R	M	S	A	F	P	G	D	P	G	A	T	A	V	A	A	I	D	Q
L	F	Q	R	M	S	A	F	P	G	D	P	G	A	T	A	V	A	A	I	D	Q
L	F	Q	R	M	S	A	F	P	G	D	P	G	A	T	A	V	A	A	I	D	Q
L	F	Q	R	M	S	A	F	P	G	D	P	G	A	T	A	V	A	A	I	D	Q
L	F	Q	R	M	S	A	F	P	G	D	P	G	A	T	A	V	A	A	I	D	Q
L	F	Q	R	M	S	A	F	P	G	D	P	G	A	T	A	V	A	A	I	D	Q
L	F	Q	R	M	S	A	F	P	G	D	P	G	A	T	A	V	A	A	I	D	Q
L	F	Q	R	M	S	A	F	P	G	D	P	G	A	T	A	V	A	A	I	D	Q
L	F	Q	R	M	S	A	F	P	G	D	P	G	A	T	A	V	A	A	I	D	Q
L	F	Q	R	M	S	A	F	P	G	D	P	G	A	T	A	V	A	A	I	D	Q
L	F	Q	R	M	S	A	F	P	G	D	P	G	A	T	A	V	A	A	I	D	Q
L	F	Q	R	M	S	A	F	P	G	D	P	G	A	T	A	V	A	A	I	D	Q
L	F	Q	R	M	S	A	F	P	G	D	P	G	A	T	A	V	A	A	I	D	Q
L	F	Q	R	M	S	A	F	P	G	D	P	G	A	T	A	V	A	A	I	D	Q
L	F	Q	R	M	S	A	F	P	G	D	P	G	A	T	A	V	A	A	I	D	Q
L	F	Q	R	M	S	A	F	P	G	D	P	G	A	T	A	V	A	A	I	D	Q
L	F	Q	R	M	S	A	F	P	G	D	P	G	A	T	A	V	A	A	I	D	Q
L	F	Q	R	M	S	A	F	P	G	D	P	G	A	T	A	V	A	A	I	D	Q
L	F	Q	R	M	S	A	F	P	G	D	P	G	A	T	A	V	A	A	I	D	Q
L	F	Q	R	M	S	A	F	P	G	D	P	G	A	T	A	V	A	A	I	D	Q
L	F	Q	R	M	S	A	F	P	G	D	P	G	A	T	A	V	A	A	I	D	Q
L	F	Q	R	M	S	A	F	P	G	D	P	G	A	T	A	V	A	A	I	D	Q
L	F	Q	R	M	S	A	F	P	G	D	P	G	A	T	A	V	A	A	I	D	Q

L	F	Q	R	M	S	A	F	P	G	D	P	G	A	T	A	V	A	A	I	D	Q
L	F	Q	R	M	S	A	F	P	G	D	P	G	A	T	A	V	A	A	I	D	Q
L	F	Q	R	M	S	A	F	P	G	D	P	G	A	T	A	V	A	A	I	D	Q
L	F	Q	R	M	S	A	F	P	G	D	P	G	A	T	A	V	A	A	I	D	Q
L	F	Q	R	M	S	A	F	P	G	D	P	G	A	T	A	V	A	A	I	D	Q
L	F	Q	R	M	S	A	F	P	G	D	P	G	A	T	A	V	A	A	I	D	Q
L	F	Q	R	M	S	A	F	P	G	D	P	G	A	T	A	V	A	A	I	D	Q
L	F	Q	R	M	S	A	F	P	G	D	P	G	A	T	A	V	A	A	I	D	Q
L	F	Q	R	M	S	A	F	P	G	D	P	G	A	T	A	V	A	A	I	D	Q
L	F	Q	R	M	S	A	F	P	G	D	P	G	A	T	A	V	A	A	I	D	Q
L	F	Q	R	M	S	A	F	P	G	D	P	G	A	T	A	V	A	A	I	D	Q
L	F	Q	R	M	S	A	F	P	G	D	P	G	A	T	A	V	A	A	I	D	Q
L	F	Q	R	M	S	A	F	P	G	D	P	G	A	T	A	V	A	A	I	D	Q
L	F	Q	R	M	S	A	F	P	G	D	P	G	A	T	A	V	A	A	I	D	Q
L	F	Q	R	M	S	A	F	P	G	D	P	G	A	T	A	V	A	A	I	D	Q
L	F	Q	R	M	S	A	F	P	G	D	P	G	A	T	A	V	A	A	I	D	Q
L	F	Q	R	M	S	A	F	P	G	D	P	G	A	T	A	V	A	A	I	D	Q
L	F	Q	R	M	S	A	F	P	G	D	P	G	A	T	A	V	A	A	I	D	Q
L	F	Q	R	M	S	A	F	P	G	D	P	G	A	T	A	V	A	A	I	D	Q
L	F	Q	R	M	S	A	F	P	G	D	P	G	A	T	A	V	A	A	I	D	Q
L	F	Q	R	M	S	A	F	P	G	D	P	G	A	T	A	V	A	A	I	D	Q
L	F	Q	R	M	S	A	F	P	G	D	L	G	A	T	A	V	A	A	I	N	Q
L	F	Q	R	M	S	A	F	P	G	D	P	G	A	T	A	V	A	A	I	D	Q
L	F	Q	R	M	S	A	V	P	G	D	P	G	A	T	A	V	A	A	I	D	Q
L	F	Q	R	M	S	A	F	P	G	D	P	G	A	T	A	V	A	A	I	D	Q
L	F	Q	R	M	S	A	F	P	G	D	P	G	A	T	A	V	A	A	I	D	Q
L	F	Q	R	M	S	A	F	P	G	D	P	G	A	T	A	V	A	A	I	D	Q
L	F	Q	R	M	S	A	F	P	G	D	P	G	A	T	A	V	A	A	I	D	Q
L	F	Q	R	M	S	A	F	P	G	D	L	G	A	T	A	V	A	A	I	D	Q
L	F	Q	R	M	S	A	F	P	G	D	P	G	A	T	A	V	A	A	V	D	Q
L	F	Q	R	M	S	A	F	P	G	D	P	G	A	T	A	V	A	A	I	D	Q
L	F	Q	R	M	S	A	F	P	G	D	P	G	A	T	A	V	A	A	I	D	Q
L	F	Q	R	M	S	A	F	P	G	D	P	G	A	T	A	V	A	A	I	D	Q
L	F	Q	R	M	S	A	F	P	G	D	P	G	A	T	A	V	A	A	I	D	Q
L	F	Q	R	M	S	A	F	P	G	D	P	G	A	T	A	V	A	A	I	D	Q
L	F	Q	R	M	S	A	F	P	G	D	P	G	A	T	A	V	A	A	I	D	Q
L	F	Q	R	M	S	A	F	P	G	D	P	G	A	T	A	V	A	A	I	D	Q
L	F	Q	R	M	S	A	F	P	G	D	P	G	A	T	A	V	A	A	I	D	Q
L	F	Q	R	M	S	A	F	P	G	D	P	G	A	T	A	V	A	A	I	D	Q
L	F	Q	R	M	S	A	F	P	G	D	P	G	A	T	A	V	A	A	I	D	Q
L	F	Q	R	M	S	A	F	P	G	D	P	G	A	T	A	V	A	A	I	D	Q
L	F	Q	R	M	S	A	F	P	G	D	P	G	A	T	A	V	A	A	I	D	Q
L	F	Q	R	M	S	A	F	P	G	D	P	G	A	T	A	V	A	A	I	D	Q
L	F	Q	R	M	S	A	F	P	G	N	P	G	A	T	A	V	A	A	I	D	Q
L	F	Q	R	M	S	A	F	P	G	D	P	G	A	T	A	V	A	A	I	D	Q
L	F	Q	R	M	S	A	F	P	G	D	P	G	A	T	A	V	A	A	I	D	Q
L	F	Q	R	M	S	A	F	P	G	D	P	G	A	T	A	V	A	A	I	D	Q
L	F	Q	R	M	S	A	F	P	G	D	P	E	A	T	A	V	A	A	I	D	Q
L	F	Q	R	M	S	A	F	P	G	D	P	G	A	T	A	V	A	A	I	D	Q
L	F	Q	R	M	S	A	F	P	G	D	P	G	A	T	A	V	A	A	I	D	Q
L	F	Q	R	M	S	A	F	P	G	D	P	G	A	T	A	V	A	A	I	D	Q
L	F	Q	R	M	S	A	F	P	G	D	P	G	A	T	A	V	A	A	I	D	Q
L	F	Q	R	M	S	A	F	P	G	D	P	G	A	T	A	V	A	A	I	D	Q
L	F	Q	R	M	S	A	F	P	G	D	P	G	A	T	A	V	A	A	I	D	Q
L	F	Q	R	M	S	A	F	P	G	D	P	G	A	T	A	V	A	A	I	D	Q
L	F	Q	R	M	S	A	F	P	G	D	P	G	A	T	A	V	A	A	I	D	Q
L	F	Q	R	M	S	A	F	P	G	D	P	G	A	T	A	V	A	A	I	D	Q
L	F	Q	R	M	S	A	F	P	G	D	P	G	A	T	A	V	A	A	I	D	Q
L	F	Q	R	M	S	A	F	P	G	D	P	G	A	T	A	V	A	A	I	D	Q
L	F	Q	R	M	S	A	F	P	G	D	P	G	A	T	A	V	A	A	I	D	Q
L	F	Q	R	M	S	A	F	P	G	D	P	G	A	T	A	V	A	A	I	D	Q
L	F	Q	R	M	S	A	F	P	G	D	P	G	A	T	A	V	A	A	I	D	Q
L	F	Q	R	M	S	A	F	P	G	D	P	G	A	T	A	V	A	A	I	D	Q
L	F	Q	R	M	S	A	F	P	G	D	P	G	A	T	A	V	A	A	I	D	Q
L	F	Q	R	M	S	A	F	P	G	D	P	G	A	T	A	V	A	A	I	D	Q
L	F	Q	R	M	S	A	F	P	G	D	P	G	A	T	A	V	A	A	I	D	Q

L	F	Q	R	M	S	A	F	P	G	D	P	G	A	T	A	V	A	A	I	D	Q
L	F	Q	R	M	S	A	F	P	G	D	P	G	A	T	A	V	A	A	I	D	Q
L	F	Q	R	M	S	A	F	P	G	D	P	G	A	T	A	V	A	A	I	D	Q
L	F	Q	R	M	S	A	F	P	G	D	P	G	A	T	A	V	A	A	I	D	Q
L	F	Q	R	M	S	A	F	P	G	D	P	G	A	T	A	V	A	A	I	D	Q
L	F	Q	R	M	S	A	F	P	G	D	P	G	A	T	A	V	A	A	I	D	Q
L	F	Q	R	M	S	A	F	P	G	D	P	G	A	T	A	V	A	A	I	D	Q
L	F	Q	R	M	S	A	F	P	G	D	P	G	A	T	A	V	A	A	I	D	Q
L	F	Q	R	M	S	A	F	P	G	D	P	G	A	T	A	V	A	A	I	D	Q
L	F	Q	R	M	S	A	F	P	G	D	P	G	A	T	A	V	A	A	I	D	Q
L	F	Q	R	M	S	A	F	P	G	D	P	G	A	T	A	V	A	A	I	D	Q
L	F	Q	R	M	S	A	F	P	G	D	P	G	A	T	A	V	A	A	I	D	Q
L	F	Q	R	M	S	A	F	P	G	D	P	G	A	T	A	V	A	A	I	D	Q
L	F	Q	R	M	S	A	F	P	G	D	P	G	A	T	A	V	A	A	I	D	Q
L	F	Q	R	M	S	A	F	P	G	D	P	G	A	T	A	V	A	A	I	D	Q
L	F	Q	R	M	S	A	F	P	G	D	P	G	A	T	A	V	A	A	I	D	Q
L	F	Q	R	M	S	A	F	P	G	D	P	G	A	T	A	V	A	A	I	D	Q
L	F	Q	R	M	S	A	F	P	G	D	P	G	A	T	A	V	A	A	I	D	Q
L	F	Q	R	M	S	A	F	P	G	D	P	G	A	T	A	V	A	A	I	D	Q
L	F	Q	R	M	S	A	F	P	G	D	P	G	A	T	A	V	A	A	I	D	Q
L	F	Q	R	M	S	A	F	P	G	D	P	G	A	T	A	V	A	A	I	D	Q
L	F	Q	R	M	S	A	F	P	G	D	P	G	A	T	A	V	A	A	I	D	Q
L	F	Q	R	M	S	A	F	P	G	D	P	G	A	T	A	V	A	A	I	D	Q
L	F	Q	R	M	S	A	F	P	G	D	P	G	A	T	A	V	A	A	I	D	Q
L	F	Q	R	M	S	A	F	P	G	D	P	G	A	T	A	V	A	A	I	D	Q
L	F	Q	R	M	S	A	F	P	G	D	P	G	A	T	A	V	A	A	I	D	Q
L	F	Q	R	M	S	A	F	P	G	D	P	G	A	T	A	V	A	A	I	D	Q
L	F	Q	R	M	S	A	F	P	G	D	P	G	A	T	A	V	A	A	I	D	Q
L	F	Q	R	M	S	A	F	P	G	D	P	G	A	T	A	V	A	A	I	D	Q
L	F	Q	R	M	S	A	F	P	G	D	P	G	A	T	A	V	A	A	I	D	Q
L	F	Q	R	M	S	A	F	P	G	D	P	G	A	T	A	V	A	A	I	D	Q
L	F	Q	R	M	S	A	F	P	G	D	P	G	A	T	A	V	A	A	I	D	Q
L	F	Q	R	M	S	A	F	P	G	D	P	G	A	T	A	V	A	A	I	D	Q
L	F	Q	R	M	S	A	F	P	G	D	P	G	A	T	A	V	A	A	I	D	Q
L	F	Q	R	M	S	A	F	P	G	D	P	G	A	T	A	V	A	A	I	D	Q
L	F	Q	R	M	S	A	F	P	G	D	P	G	A	T	A	V	A	A	I	D	Q
L	F	Q	R	M	S	A	F	P	G	D	P	G	A	T	A	V	A	A	I	D	Q
L	F	Q	R	M	S	A	F	P	G	D	P	G	A	T	A	V	A	A	I	D	Q
L	F	Q	R	M	S	A	F	P	G	D	P	G	A	T	A	V	A	A	I	D	Q
L	F	Q	R	M	S	A	F	P	G	D	P	G	A	T	A	V	A	A	I	D	Q
L	F	Q	R	M	S	A	F	P	G	D	P	G	A	T	A	V	A	A	I	D	Q
L	F	Q	R	M	S	A	F	P	G	D	P	G	A	T	A	V	A	A	I	D	Q
L	F	Q	R	M	S	A	F	P	G	D	P	G	A	T	A	V	A	A	I	D	Q
L	F	Q	R	M	S	A	F	P	G	D	P	G	A	T	A	V	A	A	I	D	Q
L	F	Q	R	M	S	A	F	P	G	D	P	G	A	T	A	V	A	A	I	D	Q
L	F	Q	R	M	S	A	F	P	G	D	P	G	A	T	A	V	A	A	I	D	Q
L	F	Q	R	M	S	A	F	P	G	D	P	G	A	T	A	V	A	A	I	D	Q
L	F	Q	R	M	S	A	F	P	G	D	P	G	A	T	A	V	A	A	I	D	Q
L	F	Q	R	M	S	A	F	P	G	D	P	G	A	T	A	V	A	A	I	D	Q
L	F	Q	R	M	S	A	F	P	G	D	P	G	A	T	A	V	A	A	I	D	Q
L	F	Q	R	M	S	A	F	P	G	D	P	G	A	T	A	V	A	A	I	D	Q
L	F	Q	R	M	S	A	F	P	G	D	P	G	A	T	A	V	A	A	I	D	Q
L	F	Q	R	M	S	A	F	P	G	D	P	G	A	T	A	V	A	A	I	D	Q
L	F	Q	R	M	S	A	F	P	G	D	P	G	A	T	A	V	A	A	I	D	Q
L	F	Q	R	M	S	A	F	P	G	D	P	G	A	T	A	V	A	A	I	D	Q
L	F	Q	R	M	S	A	F	P	G	D	P	G	A	T	A	V	A	A	I	D	Q
L	F	Q	R	M	S	A	F	P	G	D	P	G	A	T	A	V	A	A	I	D	Q
L	F	Q	R	M	S	A	F	P	G	D	P	G	A	T	A	V	A	A	I	D	Q
L	F	Q	R	M	S	A	F	P	G	D	P	G	A	T	A	V	A	A	I	D	Q
L	F	Q	R	M	S	A	F	Q	G	D	P	G	A	T	A	V	A	A	I	D	Q
L	F	Q	R	M	S	A	F	Q	G	D	P	G	A	T	A	V	A	A	I	D	Q
L	F	Q	R	M	S	A	F	P	G	D	P	G	A	T	A	V	A	A	I	D	Q
L	F	Q	R	M	S	A	F	P	G	D	P	G	A	T	A	V	A	A	I	D	Q
L	F	Q	R	M	S	A	F	P	G	D	P	G	A	T	A	V	A	A	I	D	Q
L	F	Q	R	M	S	A	F	P	G	D	P	G	A	T	A	V	A	A	I	D	Q
L	F	Q	R	M	S	A	F	P	G	D	P	G	A	T	A	V	A	A	I	D	Q
L	F	Q	R	M	S	A	F	P	G	D	P	G	A	T	A	V	A	A	I	D	Q

L	F	Q	R	M	S	A	F	P	G	D	P	G	A	T	A	V	A	A	I	D	Q
L	F	Q	R	M	S	A	F	P	G	D	P	G	A	T	A	V	A	A	I	D	Q
L	F	Q	R	M	S	A	F	P	G	D	P	G	A	T	A	V	A	A	I	D	Q
L	F	Q	R	M	S	A	F	P	G	D	P	G	A	T	A	V	A	A	I	D	Q
L	F	Q	R	M	S	A	F	P	G	D	P	G	A	T	A	V	A	A	I	D	Q
L	F	Q	R	M	S	A	F	P	G	D	P	G	A	T	A	V	A	A	I	D	Q
L	F	Q	R	M	S	A	F	P	G	D	P	G	A	T	A	V	A	A	I	D	Q
L	F	Q	R	M	S	A	F	P	G	D	P	G	A	T	A	V	A	A	I	D	Q
L	F	Q	R	M	S	A	F	P	G	D	P	G	A	T	A	V	A	A	I	D	Q
L	F	Q	R	M	S	A	F	P	G	D	P	G	A	T	A	V	A	A	I	D	Q
L	F	Q	R	M	S	A	F	P	G	D	P	G	A	T	A	V	A	A	I	D	Q
L	F	Q	R	M	S	A	F	P	G	D	P	G	A	T	A	V	A	A	I	D	Q
L	F	Q	R	M	S	A	F	P	G	D	P	G	A	T	A	V	A	A	I	D	Q
L	F	Q	R	M	S	A	F	P	G	D	P	G	A	T	A	V	A	A	I	D	Q
L	F	Q	R	M	S	A	F	P	G	D	P	G	A	T	A	V	A	A	I	D	Q
L	F	Q	R	M	S	A	F	P	G	D	P	G	A	T	A	V	A	A	I	D	Q
L	F	Q	R	M	S	A	F	P	G	D	P	G	A	T	A	V	A	A	I	D	Q
L	F	Q	R	M	S	A	F	P	G	D	P	G	A	T	A	V	A	A	I	D	Q
L	F	Q	R	M	S	A	F	P	G	D	P	G	A	T	A	V	A	A	I	D	Q
L	F	Q	R	M	S	A	F	P	G	D	P	G	A	T	A	V	A	A	I	D	Q
L	F	Q	R	M	S	A	F	P	G	D	P	G	A	T	A	V	A	A	I	D	Q
L	F	Q	R	M	S	A	F	P	G	D	P	G	A	T	A	V	A	A	I	D	Q
L	F	Q	R	M	S	A	F	P	G	D	P	G	A	T	A	V	A	A	I	D	Q
L	F	Q	R	M	S	A	F	P	G	D	P	G	A	T	A	V	A	A	I	D	Q
L	F	Q	R	M	S	A	F	P	G	D	P	G	A	T	A	V	A	A	I	D	Q
L	F	Q	R	M	S	A	F	P	G	D	P	G	A	T	A	V	A	A	I	D	Q
L	F	Q	R	M	S	A	F	P	G	D	P	G	A	T	A	V	A	A	I	D	Q
L	F	Q	R	M	S	A	F	P	G	D	P	G	A	T	A	V	A	A	I	D	Q
L	F	Q	R	M	S	A	F	P	G	D	P	G	A	T	A	V	A	A	I	D	Q
L	F	Q	R	M	S	A	F	P	G	D	P	G	A	T	A	V	A	A	I	D	Q
L	F	Q	R	M	S	A	F	P	G	D	P	G	A	T	A	V	A	A	I	D	Q
L	F	Q	R	M	S	A	F	P	G	D	P	G	A	T	A	V	A	A	I	D	Q
L	F	Q	R	M	S	A	F	P	G	D	P	G	A	T	A	V	A	A	I	D	Q
L	F	Q	R	M	S	A	F	P	G	D	P	G	A	T	A	V	A	A	I	D	Q
L	F	Q	R	M	S	A	F	P	G	D	P	G	A	T	A	V	A	A	I	D	Q
L	F	Q	R	M	S	A	F	P	G	D	P	G	A	T	A	V	A	A	I	D	Q
L	F	Q	R	M	S	A	F	P	G	D	P	G	A	T	A	V	A	A	I	D	Q
L	F	Q	R	M	S	A	F	P	G	D	P	G	A	T	A	V	A	A	I	D	Q
L	F	Q	R	M	S	A	F	P	G	D	P	G	A	T	A	V	A	A	I	D	Q
L	F	Q	R	M	S	A	F	P	G	D	P	G	A	T	A	V	A	A	I	D	Q
L	F	Q	R	M	S	A	F	P	G	D	P	G	A	T	A	V	A	A	I	D	Q
L	F	Q	R	M	S	A	F	P	G	D	P	G	A	T	A	V	A	A	I	D	Q
L	F	Q	R	M	S	A	F	P	G	D	P	G	A	T	A	V	A	A	I	D	Q
L	F	Q	R	M	S	A	F	P	G	D	P	G	A	T	A	V	A	A	I	D	Q
L	F	Q	R	M	S	A	F	P	G	D	P	G	A	T	A	V	A	A	I	D	Q
L	F	Q	R	M	S	A	F	P	G	D	P	G	A	T	A	V	A	A	I	D	Q
L	F	Q	R	M	S	A	F	P	G	D	P	G	A	T	A	V	A	A	I	D	Q
L	F	Q	R	M	S	A	F	P	G	D	P	G	A	T	A	V	A	A	I	D	Q
L	F	Q	R	M	S	A	F	P	G	D	P	G	A	T	A	V	A	A	I	D	Q
L	F	Q	R	M	S	A	F	P	G	D	P	G	A	T	A	V	A	A	I	D	Q
L	F	Q	R	M	S	A	F	P	G	D	P	G	A	T	A	V	A	A	I	D	Q
L	F	Q	R	M	S	A	F	P	G	D	P	G	A	T	A	V	A	A	I	D	Q
L	F	Q	R	M	S	A	F	P	G	D	P	G	A	T	A	V	A	A	I	D	Q
L	F	Q	R	M	S	A	F	P	G	D	P	G	A	T	A	V	A	A	I	D	Q
L	F	Q	R	M	S	A	F	P	G	D	P	G	A	T	A	V	A	A	I	D	Q
L	F	Q	R	M	S	A	F	P	G	D	P	G	A	T	A	V	A	A	I	D	Q
L	F	Q	R	M	S	A	F	P	G	D	P	G	A	T	A	V	A	A	I	D	Q
L	F	Q	R	M	S	A	F	P	G	D	P	G	A	T	A	V	A	A	I	D	Q
L	F	Q	R	M	S	A	F	P	G	D	P	G	A	T	A	V	A	A	I	D	Q
L	F	Q	R	M	S	A	F	P	G	D	P	G	A	T	A	V	A	A	I	D	Q
L	F	Q	R	M	S	A	F	P	G	D	P	G	A	T	A	V	A	A	I	D	Q
L	F	Q	R	M	S	A	F	P	G	D	P	G	A	T	A	V	A	A	I	D	Q
L	F	Q	R	M	S	A	F	P	G	D	P	G	A	T	A	V	A	A	I	D	Q
L	F	Q	R	M	S	A	F	P	G	D	P	G	A	T	A	V	A	A	I	D	Q
L	F	Q	R	M	S	A	F	P	G	D	P	G	A	T	A	V	A	A	I	D	Q
L	F	Q	R	M	S	A	F	P	G	D	P	G	A	T	A	V	A	A	I	D	Q
L	F	Q	R	M	S	A	F	P	G	D	P	G	A	T	A	V	A	A	I	D	Q

L	F	Q	R	M	S	A	F	P	G	D	P	G	A	T	A	V	A	A	I	D	Q
L	F	Q	R	M	S	A	F	P	G	D	P	G	A	T	A	V	A	A	I	D	Q
L	F	Q	R	M	S	A	F	P	G	D	P	G	A	T	A	V	A	A	I	D	Q
L	F	Q	R	M	S	A	F	P	G	D	P	G	A	T	A	V	A	A	I	D	Q
L	F	Q	R	M	S	A	F	P	G	D	P	G	A	T	A	V	A	A	I	D	Q
L	F	Q	R	M	S	A	F	P	G	D	P	G	A	T	A	V	A	A	I	D	Q
L	F	Q	R	M	S	A	F	P	G	D	P	G	A	T	A	V	A	A	I	D	Q
L	F	Q	R	M	S	A	F	P	G	D	P	G	A	T	A	V	A	A	I	D	Q
L	F	Q	R	M	S	A	F	P	G	D	P	G	A	T	A	V	A	A	I	D	Q
L	F	Q	R	M	S	A	F	P	G	D	P	G	A	T	A	V	A	A	I	D	Q
L	F	Q	R	M	S	A	F	P	G	D	P	G	A	T	A	V	A	A	I	D	Q
L	F	Q	R	M	S	V	F	P	G	D	P	G	A	T	A	V	A	A	I	D	Q
L	F	Q	R	M	S	V	F	P	G	D	P	G	A	T	A	V	A	A	I	D	Q
L	F	Q	R	M	S	V	F	P	G	D	P	G	A	T	A	V	A	A	I	D	Q
L	F	Q	R	M	S	V	F	P	G	D	P	G	A	T	A	V	A	A	I	D	Q
L	F	Q	R	M	S	V	F	P	G	D	P	G	A	T	A	V	A	A	I	D	Q
L	F	Q	R	M	S	V	F	P	G	D	P	G	A	T	A	V	A	A	I	D	Q
L	F	Q	R	M	S	V	F	P	G	D	P	G	A	T	A	V	A	A	I	D	Q
L	F	Q	R	M	S	V	F	P	G	D	P	G	A	T	A	V	A	A	I	D	Q
L	F	Q	R	M	S	V	F	P	G	D	P	G	A	T	A	V	A	A	I	D	Q
V	L	K	H	I	G	A	F	P	E	G	S	G	V	A	T	V	T	T	I	D	E
V	L	K	H	I	G	A	F	P	E	G	S	G	V	A	T	V	T	T	I	D	E
V	L	K	H	I	G	A	F	P	E	G	S	G	V	A	T	V	T	T	I	D	E
V	L	K	H	I	G	A	F	P	E	G	S	G	V	A	T	V	T	T	I	D	E
V	L	K	H	I	G	A	F	P	E	G	S	G	V	A	T	V	T	T	I	D	E
V	L	K	H	I	G	A	F	P	E	G	S	G	V	A	T	V	T	T	I	D	E
V	L	K	H	I	G	A	F	P	E	G	S	G	V	A	T	V	T	T	I	D	E
V	L	K	H	I	G	A	F	P	E	G	S	G	V	A	T	V	T	T	I	D	E
L	F	Q	R	M	S	A	F	P	G	D	P	G	A	T	A	V	A	A	I	D	Q
L	F	Q	R	M	S	A	F	P	G	D	P	G	A	T	A	V	A	A	I	D	Q
L	F	Q	R	M	S	A	F	P	G	D	P	G	A	T	A	V	A	A	I	D	Q
L	F	Q	R	M	S	A	F	P	G	D	P	G	A	T	A	V	A	A	I	D	Q
L	F	Q	R	M	S	A	F	P	G	D	P	G	A	T	A	V	A	A	I	D	Q
L	F	Q	R	M	S	A	F	P	G	D	P	G	A	T	A	V	A	A	I	D	Q
L	F	Q	R	M	S	A	F	P	G	D	P	G	A	T	A	V	A	A	I	D	Q
L	F	Q	R	M	S	A	F	P	G	D	P	G	A	T	A	V	A	A	I	D	Q
L	F	Q	R	M	S	A	F	P	G	D	P	G	A	T	A	V	A	A	I	D	Q
L	F	Q	R	M	S	A	F	P	G	D	P	G	A	T	A	V	A	A	I	D	Q
L	F	Q	R	M	S	A	F	P	G	D	P	G	A	T	A	V	A	A	I	D	Q
L	F	Q	R	M	S	A	F	P	G	D	P	G	V	T	A	V	A	A	I	D	Q
L	F	Q	R	M	S	A	F	P	G	D	P	G	A	T	A	V	A	A	I	D	Q
L	F	Q	R	M	S	A	F	P	G	D	P	G	A	T	A	V	A	A	I	D	Q
L	F	Q	R	M	S	A	F	P	G	D	P	G	A	T	A	V	A	A	I	D	Q
L	F	Q	R	M	S	A	F	P	G	D	P	G	A	T	A	V	A	A	I	D	Q
L	F	Q	R	M	S	A	F	P	G	D	P	G	A	T	A	V	A	A	I	D	Q
L	F	Q	R	M	S	A	F	P	G	D	P	G	A	T	A	M	A	A	I	D	Q
L	F	Q	R	M	S	A	F	P	G	D	P	G	A	T	A	V	A	A	I	D	Q
L	F	Q	R	M	S	A	F	P	G	D	P	G	A	T	A	V	A	A	I	D	Q
L	F	Q	R	M	S	A	F	P	G	D	P	G	A	T	A	V	A	A	I	D	Q
L	F	Q	R	M	S	A	F	P	G	D	P	G	A	T	A	V	A	A	I	D	Q
L	F	Q	R	M	S	A	F	P	G	D	P	G	A	T	A	V	A	A	I	D	Q
L	F	Q	R	M	S	A	F	P	G	D	P	G	A	T	A	V	A	A	I	D	Q
L	F	Q	R	M	S	A	F	P	G	D	P	G	A	T	A	V	A	A	I	D	Q
L	F	Q	R	M	S	A	F	P	G	D	P	G	A	T	A	V	A	A	I	D	Q
L	F	Q	R	M	S	A	F	P	G	D	P	G	A	T	A	V	A	A	I	D	Q
L	F	Q	R	M	S	A	F	P	G	D	P	G	A	T	A	V	A	A	I	D	Q
L	F	Q	R	M	S	A	F	P	G	D	P	G	A	T	A	V	A	A	I	D	Q
L	F	Q	R	M	S	A	F	P	G	D	P	G	A	T	A	V	A	A	I	D	Q
L	F	Q	R	M	S	A	F	P	G	D	P	G	A	T	A	V	A	A	I	D	Q
L	F	Q	R	M	S	A	F	P	G	D	P	G	A	T	A	V	A	A	I	D	Q
L	F	Q	R	M	S	A	F	P	G	D	P	G	A	T	A	V	A	A	I	D	Q
L	F	Q	R	M	S	A	F	P	G	D	P	G	A	T	A	V	A	A	I	D	Q
L	F	Q	R	M	S	A	F	P	G	D	P	G	A	T	A	V	A	A	I	D	Q
L	F	Q	R	M	S	A	F	P	G	D	P	G	A	T	A	V	A	A	I	D	Q
L	F	Q	R	M	S	A	F	P	G	D	P	G	A	T	A	V	A	A	I	D	Q
L	F	Q	R	M	S	A	F	P	G	D	P	G	A	T	A	V	A	A	I	D	Q
L	F	Q	R	M	S	A	F	P	G	D	P	G	A	T	A	V	A	A	I	D	Q

L	F	Q	R	M	S	A	F	P	G	D	P	G	A	T	A	V	A	A	I	D	Q
L	F	Q	R	M	S	A	F	P	G	D	P	G	A	T	A	V	A	A	I	D	Q
L	F	Q	R	M	S	A	F	P	G	D	P	G	A	T	A	V	A	A	I	D	Q
L	F	Q	R	M	S	A	F	P	G	D	P	G	A	T	A	V	A	A	I	D	Q
L	F	Q	R	M	S	A	F	P	G	D	P	G	A	T	A	V	A	A	I	D	Q
L	F	Q	R	M	S	A	F	P	G	D	P	G	A	T	A	V	A	A	I	D	Q
L	F	Q	R	M	S	A	F	P	G	D	P	G	A	T	A	V	A	A	I	D	Q
L	F	Q	R	M	S	A	F	P	G	D	P	G	A	T	A	V	A	A	I	D	Q
L	F	Q	R	M	S	A	F	P	G	D	P	G	A	T	A	V	A	A	I	D	Q
L	F	Q	R	M	S	A	F	P	G	D	P	G	A	T	A	V	A	A	I	D	Q
L	F	Q	R	M	S	A	F	P	G	D	P	G	A	T	A	V	A	A	I	D	Q
L	F	Q	R	M	S	A	F	P	G	D	P	G	A	T	A	V	A	A	I	D	Q
L	F	Q	R	M	S	A	F	P	G	D	P	G	A	T	A	V	A	A	I	D	Q
L	F	Q	R	M	S	A	F	P	G	D	P	G	A	T	A	V	A	A	I	D	Q
L	F	Q	R	M	S	A	F	P	G	D	P	G	A	T	A	V	A	A	I	D	Q
L	F	Q	R	M	S	A	F	P	G	D	P	G	A	T	A	V	A	A	I	D	Q
L	F	Q	R	M	S	A	F	P	G	D	P	G	A	T	A	V	A	A	I	D	Q
L	F	Q	R	M	S	A	F	P	G	D	P	G	A	T	A	V	A	A	I	D	Q
L	F	Q	R	M	S	A	F	P	G	D	P	G	A	T	A	V	A	A	I	D	Q
L	F	Q	R	M	S	A	F	P	G	D	P	G	A	T	A	V	A	A	I	D	Q
L	F	Q	R	M	S	A	F	P	G	D	P	G	A	T	A	V	A	A	I	D	Q
L	F	Q	R	M	S	A	F	P	G	D	P	G	A	T	A	V	A	A	I	D	Q
L	F	Q	R	M	S	A	F	P	G	D	P	G	A	T	A	V	A	A	I	D	Q
L	F	Q	R	M	S	A	F	P	G	D	P	G	A	T	A	V	A	A	I	D	Q
L	F	Q	R	M	S	V	F	P	G	D	P	G	A	T	A	V	A	A	I	D	Q
L	F	Q	R	M	S	V	F	P	G	D	P	G	A	T	A	V	A	A	I	D	Q
L	F	Q	R	M	S	V	F	P	G	D	P	G	A	T	A	V	A	A	I	D	Q
L	F	Q	R	M	S	V	F	P	G	D	P	G	A	T	A	V	A	A	I	D	Q
L	F	Q	R	M	S	V	F	P	G	D	P	G	A	T	A	V	A	A	I	D	Q
L	F	Q	R	M	S	V	F	P	G	D	P	G	A	T	A	V	A	A	I	D	Q
L	F	Q	R	M	S	V	F	P	G	D	P	G	A	T	A	V	A	A	I	D	Q
L	F	Q	R	M	S	V	F	P	G	D	P	G	A	T	A	V	A	A	I	D	Q
L	F	Q	R	M	S	V	F	P	G	D	P	G	A	T	A	V	A	A	I	D	Q
L	F	Q	R	M	S	V	F	P	G	D	P	G	A	T	A	V	A	A	I	D	Q
L	F	Q	R	M	S	V	F	P	G	D	P	G	A	T	A	V	A	A	I	D	Q
L	F	Q	R	M	S	V	F	P	G	D	P	G	A	T	A	V	A	A	I	D	Q
L	F	Q	R	M	S	V	F	P	G	D	P	G	A	T	A	V	A	A	I	D	Q
L	F	Q	R	M	S	V	F	P	G	D	P	G	A	T	A	V	A	A	I	D	Q
L	F	Q	R	M	S	V	F	P	G	D	P	G	A	T	A	V	A	A	I	D	Q
L	F	Q	R	M	S	V	F	P	G	D	P	G	A	T	A	V	A	A	I	D	Q
L	F	Q	R	M	S	V	F	P	G	D	P	G	A	T	A	V	A	A	I	D	Q
L	F	Q	R	M	S	V	F	P	G	D	P	G	A	T	A	V	A	A	I	D	Q
L	F	Q	R	M	S	V	F	P	G	D	P	G	A	T	A	V	A	A	I	D	Q
L	F	Q	R	M	S	V	F	P	G	D	P	G	A	T	A	V	A	A	I	D	Q
L	F	Q	R	M	S	V	F	P	G	D	P	G	A	T	A	V	A	A	I	D	Q
L	F	Q	R	M	S	V	F	P	G	D	P	G	A	T	A	V	A	A	I	D	Q
L	F	Q	R	M	S	V	F	P	G	D	P	G	A	T	A	V	A	A	I	D	Q
L	F	Q	R	M	S	V	F	P	G	D	P	G	A	T	A	V	A	A	I	D	Q
L	F	Q	R	M	S	V	F	P	G	D	P	G	A	T	A	V	A	A	I	D	Q
L	F	Q	R	M	S	A	F	P	G	D	P	G	A	T	A	V	A	A	I	D	Q
L	F	Q	R	M	S	A	F	P	G	D	P	G	A	T	A	V	A	A	I	D	Q
L	F	Q	R	M	S	A	F	P	G	D	P	G	A	T	A	V	A	A	I	D	Q
L	F	Q	R	M	S	A	F	P	G	D	P	G	A	T	A	V	A	A	I	D	Q
L	F	Q	R	M	S	A	F	P	G	D	P	G	A	T	A	V	A	A	I	D	Q
L	F	Q	R	M	S	A	F	P	G	D	P	G	A	T	A	V	A	A	I	D	Q
L	F	Q	R	M	S	A	F	P	G	D	P	G	A	T	A	V	A	A	I	D	Q
L	F	Q	R	M	S	A	F	P	G	D	P	G	A	T	A	V	A	A	I	D	Q
L	F	Q	R	M	S	A	F	P	G	D	P	G	A	T	A	V	A	A	I	D	Q
L	F	Q	R	M	S	A	F	P	G	D	P	G	A	T	A	V	A	A	I	D	Q
L	F	Q	R	M	S	A	F	P	G	D	P	G	A	T	A	V	A	A	I	D	Q
L	F	Q	R	M	S	A	F	P	G	D	P	G	A	T	A	V	A	A	I	D	Q
L	F	Q	R	M	S	A	F	P	G	D	P	G	A	T	A	V	A	A	I	D	Q
L	F	Q	R	M	S	A	F	P	G	D	P	G	A	T	A	V	A	A	I	D	Q
L	F	Q	R	M	S	A	F	P	G	D	P	G	A	T	A	V	A	A	I	D	Q
L	F	Q	R	M	S	A	F	P	G	D	P	G	A	T	A	V	A	A	I	D	Q
L	F	Q	R	M	S	A	F	P	G	D	P	G	A	T	A	V	A	A	I	D	Q
L	F	Q	R	M	S	A	F	P	G	D	P	G	A	T	A	V	A	A	I	D	Q

L	F	Q	R	M	S	A	F	P	G	D	P	G	A	T	A	V	A	A	I	D	Q
L	F	Q	R	M	S	A	F	P	G	D	P	G	A	T	A	V	A	A	I	D	Q
L	F	Q	R	M	S	A	F	P	G	D	P	G	A	T	A	V	A	A	I	D	Q
L	F	Q	R	M	S	A	F	P	G	D	P	G	A	T	A	V	A	A	I	D	Q
L	F	Q	R	M	S	A	F	P	G	D	P	G	A	T	A	V	A	A	I	D	Q
L	F	Q	R	M	S	A	F	P	G	D	P	G	A	T	A	V	A	A	I	D	Q
L	F	Q	R	M	S	A	F	P	G	D	P	G	A	T	A	V	A	A	I	D	Q
L	F	Q	R	M	S	A	F	P	G	D	P	G	A	T	A	V	A	A	I	D	Q
L	F	Q	R	M	S	A	F	P	G	D	P	G	A	T	A	V	A	A	I	D	Q
L	F	Q	R	M	S	A	F	P	G	D	P	G	A	T	A	V	A	A	I	D	Q

wzd																					
110	116	119	132	149	181	183	195	211	212	214	217	218	223	227	9	12	13	14	34	45	50
A	A	A	Q	E	S	F	A	D	I	D	K	I	L	F	A	D	S	V	G	I	S
T	T	T	E	G	T	V	T	E	V	E	Q	V	V	L	V	E	L	A	D	P	T
A	A	A	Q	E	S	F	A	D	V	D	K	I	L	F	A	D	S	V	G	L	S
T	T	T	E	G	T	V	T	E	V	E	Q	V	V	L	V	E	L	A	D	P	T
T	T	T	E	G	T	V	T	E	V	E	Q	V	V	L	V	E	L	A	D	P	T
T	T	T	E	G	T	V	T	E	V	E	Q	V	V	L	V	E	L	A	D	P	T
T	T	T	E	G	T	V	T	E	V	E	Q	V	V	L	V	E	L	A	D	P	T
T	T	T	E	G	T	V	T	E	V	E	Q	V	V	L	V	E	L	A	D	P	T
T	T	T	E	G	T	V	T	E	V	E	Q	V	V	L	V	E	L	A	D	P	T
T	T	T	E	G	T	V	T	E	V	E	Q	V	V	L	V	E	L	A	D	P	T
T	T	T	E	G	T	V	T	E	V	E	Q	V	V	L	V	E	L	A	D	P	T

wzd																					
115	116	119	132	136	149	181	183	195	211	212	214	217	218	223	227	9	12	13	14	34	45
V	A	A	Q	A	E	S	F	A	D	I	D	K	I	L	F	A	D	S	V	G	I
V	T	T	E	A	G	T	V	T	E	V	E	Q	V	V	L	V	E	L	A	D	P
V	A	A	Q	A	E	S	F	A	D	V	D	K	I	L	F	A	D	S	V	G	T
V	A	A	Q	A	E	S	F	A	D	V	D	K	I	L	F	A	D	S	V	G	T
V	A	A	Q	A	E	S	F	A	D	V	D	K	I	L	F	A	D	S	V	G	T
V	A	A	Q	A	E	S	F	A	D	V	D	K	I	L	F	A	D	S	V	G	T
V	A	A	Q	A	E	S	F	A	D	V	D	K	I	L	F	A	D	S	V	G	T
V	A	A	Q	A	E	S	F	A	D	V	D	K	I	L	F	A	D	S	V	G	T
V	A	A	Q	A	E	S	F	A	D	V	D	K	I	L	F	A	D	S	V	G	T
V	A	A	Q	A	E	S	F	A	D	V	D	K	I	L	F	A	D	S	V	G	T
V	A	A	Q	A	E	S	F	A	D	V	D	K	I	L	F	A	D	S	V	G	T
V	A	A	Q	A	E	S	F	A	D	V	D	K	I	L	F	A	D	S	V	G	T
V	A	A	Q	V	E	S	F	A	D	V	D	K	I	L	F	A	D	S	V	G	T
V	A	A	Q	A	E	S	F	A	D	V	D	K	I	L	F	A	D	S	V	G	T
V	A	A	Q	A	E	S	F	A	D	V	D	K	I	L	F	A	D	S	V	G	T
V	A	A	Q	A	E	S	F	A	D	V	D	K	I	L	F	A	D	S	V	G	T
V	A	A	Q	A	E	S	F	A	D	V	D	K	I	L	F	A	D	S	V	G	T
V	A	A	Q	A	E	S	F	A	D	V	D	K	I	L	F	A	D	S	V	G	T
M	A	A	Q	A	E	S	F	A	D	V	D	K	I	L	F	A	D	S	V	G	T
V	A	A	Q	A	E	S	F	A	D	V	D	K	I	L	F	A	D	S	V	G	T
V	A	A	Q	A	E	S	F	A	D	V	D	K	I	L	F	A	D	S	V	G	T
V	A	A	Q	A	E	S	F	A	D	I	D	K	I	L	F	A	D	S	V	G	I
V	A	A	Q	A	E	S	F	A	D	I	D	K	I	L	F	A	D	S	V	G	I
V	A	A	Q	A	E	S	F	A	D	I	D	K	I	L	F	A	D	S	V	G	I
V	A	A	Q	A	E	S	F	A	D	I	D	K	I	L	F	A	D	S	V	G	I
V	A	A	Q	A	E	S	F	A	D	I	D	K	I	L	F	A	D	S	V	G	I
V	A	A	Q	A	E	S	F	A	D	I	D	K	I	L	F	A	D	S	V	G	I
V	A	A	Q	A	E	S	F	A	D	I	D	K	I	L	F	A	D	S	V	G	I
V	A	A	Q	A	E	S	F	A	D	I	D	K	I	L	F	A	D	S	V	G	I
V	A	A	Q	A	E	S	F	A	D	I	D	K	I	L	F	A	D	S	V	G	I
V	A	A	Q	A	E	S	F	A	D	I	D	K	I	L	F	A	D	S	V	G	I

rmlA				rmlC	rmlB		rmlD						
150	151	272	286		275	300	33	38	39	71	77	245	262
E	N	S	V		V	E	E	N	E	K	I	V	A
T	D	P	I		A	D	K	N	E	K	I	A	A
E	N	S	V		V	E	E	N	E	K	I	A	A
E	N	S	I		V	D	E	D	A	R	I	A	A
E	N	S	I		V	D	E	D	A	R	I	A	S
E	N	S	I		V	D	E	D	A	R	I	A	S
E	N	S	I		V	D	E	D	A	R	T	A	S
E	N	S	I		V	D	E	D	A	R	I	A	A
E	N	S	I		V	D	E	D	A	R	I	A	S
E	N	S	I		V	D	E	D	A	R	I	A	A
E	N	S	I		V	D	E	D	A	R	I	A	S
E	N	S	I		V	D	E	D	A	R	I	A	A
E	N	S	I		V	D	E	D	A	R	I	A	A
E	N	S	I		V	D	E	D	A	R	I	A	S
E	N	S	I		V	D	E	D	A	R	I	A	S
E	N	S	I		V	D	E	D	A	R	I	A	A
E	N	S	I		V	D	E	D	A	R	T	A	S
E	N	S	I		V	D	E	D	A	R	I	A	S
E	N	S	I		V	D	E	D	A	R	I	A	A
E	N	S	I		V	D	E	D	A	R	I	A	A
E	N	S	I		V	D	E	D	A	R	I	A	S
E	N	S	I		V	D	E	D	A	R	I	A	A
E	N	S	I		V	D	E	D	A	R	I	A	S
E	N	S	I		V	D	E	D	A	R	I	A	A
E	N	S	I		V	D	E	D	A	R	I	A	S
E	N	S	I		V	D	E	D	A	R	I	A	A
E	N	S	I		V	D	E	D	A	R	I	A	A
E	N	S	I		V	D	E	D	A	R	I	A	A
E	N	S	I		V	D	E	D	A	R	I	A	A
E	N	S	I		V	D	E	D	A	R	I	A	S
E	N	S	I		V	D	E	D	A	R	I	A	S
E	N	S	I		V	D	E	D	A	R	I	A	A
E	N	S	I		V	D	E	D	A	R	I	A	A
E	N	S	I		V	D	E	D	A	R	I	A	S
E	N	S	I		V	D	E	D	A	R	I	A	A

151	180	182	187	189	194	201	210	211	216	217	222	227
V	S	F	G	V	A	L	D	I	K	I	L	D
V	S	F	G	V	A	L	D	V	K	I	L	D
I	T	V	A	A	T	F	E	V	Q	M	V	N
V	S	F	G	V	A	L	D	I	K	I	L	D

12	13	14	17	34	41	45	50	62
D	S	V	A	G	T	I	S	R
D	S	V	A	G	T	L	S	R
E	L	A	T	D	S	A	T	H
D	S	V	A	G	T	I	S	R

wzd	

116	119	132	149	181	183	195	211	212	214	217	218	223	227
A	A	Q	E	S	F	A	D	I	D	K	I	L	F
T	T	E	G	T	V	T	E	V	E	Q	V	V	L
A	A	Q	E	S	F	A	D	V	D	K	I	L	F
A	A	Q	E	S	F	A	D	V	D	K	I	L	F
A	A	Q	E	S	F	A	D	V	D	K	I	L	F
A	A	Q	E	S	F	A	D	V	D	K	I	L	F
A	A	Q	E	S	F	A	D	V	D	K	I	L	F
A	A	Q	E	S	F	A	D	V	D	K	I	L	F
A	A	Q	E	S	F	A	D	V	D	K	I	L	F
A	A	Q	E	S	F	A	D	V	D	K	I	L	F
A	A	Q	E	S	F	A	D	V	D	K	I	L	F
A	A	Q	E	S	F	A	D	V	D	K	I	L	F
A	A	Q	E	S	F	A	D	V	D	K	I	L	F
A	A	Q	E	S	F	A	D	V	D	K	I	L	F
A	A	Q	E	S	F	A	D	V	D	K	I	L	F
A	A	Q	E	S	F	A	D	V	D	K	I	L	F
A	A	Q	E	S	F	A	D	V	D	K	I	L	F
A	A	Q	E	S	F	A	D	V	D	K	I	L	F
A	A	Q	E	S	F	A	D	V	D	K	I	L	F
A	A	Q	E	S	F	A	D	V	D	K	I	L	F
A	A	Q	E	S	F	A	D	V	D	K	I	L	F
A	A	Q	E	S	F	A	D	V	D	K	I	L	F
A	A	Q	E	S	F	A	D	V	D	K	I	L	F
A	A	Q	E	S	F	A	D	V	D	K	I	L	F
A	A	Q	E	S	F	A	D	V	D	K	I	L	F
A	A	Q	E	S	F	A	D	V	D	K	I	L	F
A	A	Q	E	S	F	A	D	V	D	K	I	L	F

9	12	13	14	34	45	50	58
A	D	S	V	G	I	S	W
V	E	L	A	D	P	T	R
A	D	S	V	G	T	S	W
A	D	S	V	G	T	S	W
A	D	S	V	G	T	S	W
A	D	S	V	G	T	S	W
A	D	S	V	G	T	S	W
A	D	S	V	G	T	S	W
A	D	S	V	G	T	S	W
A	D	S	V	G	T	S	W
A	D	S	V	G	T	S	W
A	D	S	V	G	T	S	W
A	D	S	V	G	T	S	W
A	D	S	V	G	T	S	W
A	D	S	V	G	T	S	W
A	D	S	V	G	T	S	W
A	D	S	V	G	T	S	W
A	D	S	V	G	T	S	W
A	D	S	V	G	T	S	W
A	D	S	V	G	T	S	W
A	D	S	V	G	T	S	W
A	D	S	V	G	T	S	W
A	D	S	V	G	T	S	W
A	D	S	V	G	T	S	W
A	D	S	V	G	T	S	W
A	D	S	V	G	T	S	W
A	D	S	V	G	T	S	W

wze																						
87	88	136	140	154	156	158	167	172	173	174	175	176	177	178	181	183	186	191	195	199	208	210
D	K	S	E	A	V	V	R	S	I	L	V	T	E	A	I	R	I	E	H	P	F	T
D	K	S	E	A	V	V	R	F	Y	F	M	D	E	A	I	R	I	E	H	P	F	T
E	R	S	G	P	I	I	Q	S	I	L	I	T	A	T	V	K	V	Q	Q	L	L	I
D	K	T	E	A	V	V	R	S	I	L	V	T	E	A	I	R	I	E	H	P	F	T
D	K	T	E	A	V	V	R	S	I	L	V	T	E	A	I	R	I	E	H	P	F	T
D	K	T	E	A	V	V	R	S	I	L	V	T	E	A	I	R	I	E	H	P	F	T
D	K	T	E	A	V	V	R	S	I	L	V	T	K	A	I	R	I	E	H	P	F	T
D	K	T	E	A	V	V	R	S	I	L	V	T	E	A	I	R	I	E	H	P	F	T
D	K	T	E	A	V	V	R	S	I	L	V	T	E	A	I	R	I	E	H	P	F	T
D	K	T	E	A	V	V	R	S	I	L	V	T	E	A	I	R	I	E	H	P	F	T
D	K	T	E	A	V	V	R	S	I	L	V	T	E	A	I	R	I	E	H	P	F	T
D	K	T	E	A	V	V	R	S	I	L	V	T	E	A	I	R	I	E	H	P	F	T
D	K	T	E	A	V	V	R	S	I	L	V	T	E	A	I	R	I	E	H	P	F	T
D	K	T	E	A	V	V	R	S	I	L	V	T	E	A	I	R	I	E	H	P	F	T
D	K	T	E	A	V	V	R	S	I	L	V	T	E	A	I	R	I	E	H	P	F	T
D	K	T	E	A	V	V	R	S	I	L	V	T	E	A	I	R	I	E	H	P	F	T
D	K	T	E	A	V	V	R	S	I	L	V	T	E	A	I	R	I	E	H	P	F	T
D	K	T	E	A	V	V	R	S	I	L	V	T	E	A	I	R	I	E	H	P	F	T
D	K	T	E	A	V	V	R	S	I	L	V	T	E	A	I	R	I	E	H	P	F	T
D	K	T	E	A	V	V	R	S	I	L	V	T	E	A	I	R	I	E	H	P	F	T
D	K	T	E	A	V	V	R	S	I	L	V	T	E	A	I	R	I	E	H	P	F	T
D	K	T	E	A	V	V	R	S	I	L	V	T	E	A	I	R	I	E	H	P	F	T
D	K	T	E	A	V	V	R	S	I	L	V	T	E	A	I	R	I	E	H	P	F	T
D	K	T	E	A	V	V	R	S	I	L	V	T	E	A	I	R	I	E	H	P	F	T
D	K	T	E	A	V	V	R	S	I	L	V	T	E	A	I	R	I	E	H	P	F	T
D	K	T	E	A	V	V	R	S	I	L	V	T	E	A	I	R	I	E	H	P	F	T
D	K	T	E	A	V	V	R	S	I	L	V	T	E	A	I	R	I	E	H	P	F	T
D	K	T	E	A	V	V	R	S	I	L	V	T	E	A	I	R	I	E	H	P	F	T
D	K	T	E	A	V	V	R	S	I	L	V	T	E	A	I	R	I	E	H	P	F	T

D	K	T	E	A	V	V	R	S	I	L	V	T	E	A	I	R	I	E	H	P	F	T
D	K	T	E	A	V	V	R	S	I	L	V	T	E	A	I	R	I	E	H	P	F	T

	wzx						rmlA													
403	35	89	176	203	254	370	114	118	125	128	136	140	150	151	153	163	206	214	268	272
G	M	F	W	E	T	L	P	T	K	K	H	P	E	N	N	Y	D	L	A	S
G	M	S	W	E	T	L	P	K	K	K	Q	P	T	D	N	Y	D	L	A	P
G	M	S	W	E	T	L	P	K	K	K	Q	P	T	D	N	Y	D	L	A	P
G	M	S	W	E	T	L	P	K	K	K	Q	P	T	D	N	Y	D	L	A	P
G	M	S	W	E	M	L	P	K	K	K	Q	P	T	D	N	Y	D	L	A	P
G	M	S	W	E	T	L	P	K	K	K	Q	P	T	D	N	Y	D	L	A	P
G	M	S	W	E	T	L	P	K	K	K	Q	P	T	D	N	Y	D	L	A	P
G	M	S	W	E	T	L	P	K	K	K	Q	P	T	D	N	Y	D	L	A	P
G	M	S	W	E	T	L	P	K	K	K	Q	P	T	D	N	Y	D	L	A	P
G	M	S	W	E	T	L	P	K	K	K	Q	P	T	D	N	Y	D	L	A	P
G	M	S	W	E	T	L	P	K	K	K	Q	P	T	D	N	Y	D	L	A	P
G	M	S	W	E	T	L	P	K	K	K	Q	P	T	D	N	Y	D	L	A	P
G	M	S	W	E	T	L	P	K	K	K	Q	P	T	D	N	Y	D	L	A	P
G	M	S	W	E	T	L	P	K	K	K	Q	P	T	D	N	Y	D	L	A	P
G	M	S	W	E	T	L	P	K	K	K	Q	P	T	D	N	Y	D	L	A	P
G	M	S	W	E	T	L	P	K	K	K	Q	P	T	D	N	Y	D	L	A	P
G	M	S	W	E	T	L	P	K	K	K	Q	P	T	D	N	Y	D	L	A	P
G	M	S	W	E	T	L	P	K	K	K	Q	P	T	D	N	Y	D	L	A	P
G	M	S	W	E	T	L	P	K	K	K	Q	P	T	D	N	Y	D	L	A	P
G	M	S	W	E	T	L	P	K	K	K	Q	P	T	D	N	Y	D	L	A	P
G	I	S	W	E	T	L	P	K	K	K	Q	P	T	D	N	Y	D	L	A	P
G	M	S	W	E	T	L	P	K	K	K	Q	P	T	D	N	Y	D	L	A	P
G	M	S	W	E	T	L	P	K	K	K	Q	P	T	D	N	Y	D	L	A	P
G	M	S	W	E	T	L	P	K	K	K	Q	P	T	D	N	Y	D	L	A	P
G	M	S	W	E	T	L	P	K	K	K	Q	P	T	D	N	Y	D	L	A	P
G	M	S	W	E	T	L	P	K	K	K	Q	P	T	D	N	Y	D	L	A	P
G	M	S	W	E	T	L	P	K	K	K	Q	P	T	D	N	Y	D	L	A	P
G	M	S	W	E	T	L	P	K	K	K	Q	P	T	D	N	Y	D	L	A	P
G	M	S	W	E	T	L	P	K	K	K	Q	P	T	D	N	Y	D	L	A	P
G	M	S	W	E	T	L	P	K	K	K	Q	P	T	D	N	Y	D	L	A	P
G	M	S	W	E	T	L	S	K	S	S	H	S	Q	D	K	Q	E	L	A	P
G	M	S	W	E	T	L	P	K	K	K	Q	P	T	D	N	Y	D	L	A	P
G	M	S	W	E	T	L	P	K	K	K	Q	P	T	D	N	Y	D	L	A	P
G	M	S	W	E	T	L	P	K	K	K	Q	P	T	D	N	Y	D	L	A	P
G	M	S	W	E	T	L	P	K	K	K	Q	P	T	D	N	Y	D	L	A	P
G	M	S	W	E	T	L	P	K	K	K	Q	P	T	D	N	Y	D	L	A	P
G	M	S	W	E	T	L	P	K	K	K	Q	P	T	D	N	Y	D	L	A	P
G	M	S	W	E	T	L	P	K	K	K	Q	P	T	D	N	Y	D	L	A	P
G	M	S	W	E	T	L	P	K	K	K	Q	P	T	D	N	Y	D	L	A	P
G	M	S	W	E	T	L	P	K	K	K	Q	P	T	D	N	Y	D	L	A	P
G	M	S	W	E	T	L	P	K	K	K	Q	P	T	D	N	Y	D	L	A	P
G	M	S	W	E	T	L	P	K	K	K	Q	P	T	D	N	Y	D	L	A	P
G	M	S	W	E	T	L	P	K	K	K	Q	P	T	D	N	Y	D	L	A	P
G	M	S	W	E	T	L	P	K	K	K	Q	P	T	D	N	Y	D	L	A	P
G	M	S	W	E	T	L	P	K	K	K	Q	P	T	D	N	Y	D	L	A	P
G	M	S	W	E	T	L	P	K	K	K	Q	P	T	D	N	Y	D	L	A	P
G	M	S	W	E	T	L	P	K	K	K	Q	P	T	D	N	Y	D	L	A	P
G	M	S	W	E	T	L	P	K	K	K	Q	P	T	D	N	Y	D	L	A	P
G	M	S	W	E	T	L	P	K	K	K	Q	P	T	D	N	Y	D	L	A	P
G	M	S	W	E	T	L	P	K	K	K	Q	P	T	D	N	Y	D	L	A	P
G	M	S	L	E	T	L	P	K	K	K	Q	P	T	D	N	Y	D	L	A	P
G	M	S	W	E	T	L	P	K	K	K	Q	P	T	D	N	Y	D	L	A	P
G	M	S	W	E	T	L	P	K	K	K	Q	P	T	D	N	Y	D	L	A	P
G	M	S	W	E	T	L	P	K	K	K	Q	P	T	D	N	Y	D	L	A	P
G	M	S	W	E	T	L	P	K	K	K	Q	P	T	D	N	Y	D	L	A	P

G	M	S	W	E	T	L	P	K	K	K	Q	P	T	D	N	Y	D	L	A	P
G	M	S	W	E	T	L	P	K	K	K	Q	P	T	D	N	Y	D	L	A	P
G	M	S	L	E	T	L	P	K	K	K	Q	P	T	D	N	Y	D	L	A	P
G	M	S	L	E	T	L	P	K	K	K	Q	P	T	D	N	Y	D	L	A	P
G	M	S	W	E	T	L	P	K	K	K	Q	P	T	D	N	Y	D	L	A	P
G	M	S	W	E	T	L	P	K	K	K	Q	P	T	D	N	Y	D	L	A	P
G	M	S	W	E	T	L	P	K	K	K	Q	P	T	D	N	Y	D	L	A	P
G	M	S	W	E	T	L	P	K	K	K	Q	P	T	D	N	Y	D	L	A	P
G	M	S	W	E	T	L	P	K	K	K	Q	P	T	D	N	Y	D	L	A	P
G	M	S	W	E	T	L	P	K	K	K	Q	P	T	D	N	Y	D	L	A	P
G	M	S	W	E	T	L	P	K	K	K	Q	P	T	D	N	Y	D	L	A	P
G	M	S	W	E	T	L	P	K	K	K	Q	P	T	D	N	Y	D	L	A	P
G	M	S	W	E	T	L	P	K	K	K	Q	P	T	D	N	Y	D	L	A	P
G	M	S	W	E	T	L	P	K	K	K	Q	P	T	D	N	Y	D	L	A	P
G	M	S	W	E	T	L	P	K	K	K	Q	P	T	D	N	Y	D	L	A	P
G	M	S	W	E	T	L	P	K	K	K	Q	P	T	D	N	Y	D	L	A	P
G	M	S	W	E	T	L	P	K	K	K	Q	P	T	D	N	Y	D	L	A	P
G	M	S	W	E	T	L	P	K	K	K	Q	P	T	D	N	Y	D	L	A	P
G	M	S	W	E	T	L	P	K	K	K	Q	P	T	D	N	Y	D	L	A	P
G	M	S	W	E	T	L	P	K	K	K	Q	P	T	D	N	Y	D	L	A	P
G	M	S	W	E	T	L	P	K	K	K	Q	P	T	D	N	Y	D	L	A	P
G	M	S	W	E	T	L	P	K	K	K	Q	P	T	D	N	Y	D	L	A	P
G	M	S	W	E	T	L	P	K	K	K	Q	P	T	D	N	Y	D	L	A	P
G	M	S	W	E	T	L	P	K	K	K	Q	P	T	D	N	Y	D	L	A	P
G	M	S	W	E	T	L	P	K	K	K	Q	P	T	D	N	Y	D	L	A	P
G	M	S	W	E	T	L	P	K	K	K	Q	P	T	D	N	Y	D	L	A	P
G	M	S	W	E	T	L	P	K	K	K	Q	P	T	D	N	Y	D	L	A	P
G	M	S	W	E	T	L	P	K	K	K	Q	P	T	D	N	Y	D	L	A	P
G	M	S	L	E	T	L	P	K	K	K	Q	P	T	D	N	Y	D	L	A	P
G	M	S	W	E	T	L	P	K	K	K	Q	P	T	D	N	Y	D	L	A	P
G	M	S	W	E	T	L	P	K	K	K	Q	P	T	D	N	Y	D	L	A	P
G	M	S	W	E	T	L	P	K	K	K	Q	P	T	D	N	Y	D	L	A	P
G	M	S	W	E	T	L	P	K	K	K	Q	P	T	D	N	Y	D	L	A	P
G	M	S	W	E	T	L	P	K	K	K	Q	P	T	D	N	Y	D	L	A	P
G	M	S	W	E	T	L	P	K	K	K	Q	P	T	D	N	Y	D	L	A	P
G	M	S	W	E	T	L	P	K	K	K	Q	P	T	D	N	Y	D	L	A	P
G	M	S	W	E	T	L	P	K	K	K	Q	P	T	D	N	Y	D	L	A	P
G	M	S	W	E	T	L	P	K	K	K	Q	P	T	D	N	Y	D	L	A	P
G	M	S	W	E	T	L	P	K	K	K	Q	P	T	D	N	Y	D	L	A	P
G	M	S	W	E	T	L	P	K	K	K	Q	P	T	D	N	Y	D	L	A	P
G	M	S	W	E	T	L	P	K	K	K	Q	P	T	D	N	Y	D	L	A	P
G	M	S	W	E	T	L	P	K	K	K	Q	P	T	D	N	Y	D	L	A	P
G	M	S	W	E	T	L	P	K	K	K	Q	P	T	D	N	Y	D	L	A	P
G	M	S	W	E	T	L	P	K	K	K	Q	P	T	D	N	Y	D	L	A	P
G	M	S	W	E	T	L	P	K	K	K	Q	P	T	D	N	Y	D	L	A	P
G	M	S	L	E	T	L	P	K	K	K	Q	P	T	D	N	Y	D	L	A	P
G	M	S	L	E	T	L	P	K	K	K	Q	P	T	D	N	Y	D	L	A	P
G	M	S	W	E	T	L	P	K	K	K	Q	P	T	D	N	Y	D	L	A	P
G	M	S	W	E	T	L	P	K	K	K	Q	P	T	D	N	Y	D	L	A	P
G	M	S	W	E	T	L	P	K	K	K	Q	P	T	D	N	Y	D	L	A	P
G	M	S	W	E	T	F	P	K	K	K	H	P	T	D	N	Y	D	L	T	P
G	M	S	W	E	T	F	P	K	K	K	H	P	T	D	N	Y	D	L	T	P
G	M	S	W	E	T	F	P	K	K	K	H	P	T	D	N	Y	D	L	T	P
G	M	S	W	E	T	F	P	K	K	K	H	P	T	D	N	Y	D	L	T	P
G	M	S	W	E	T	F	P	K	K	K	H	P	T	D	N	Y	D	L	T	P
G	M	S	W	E	T	F	P	K	K	K	H	P	T	D	N	Y	D	L	T	P
G	M	S	W	E	T	F	P	K	K	K	H	P	T	D	N	Y	D	L	T	P
G	M	S	W	E	T	F	P	K	K	K	H	P	T	D	N	Y	D	L	T	P
G	M	S	W	E	T	F	P	K	K	K	H	P	T	D	N	Y	D	L	T	P
G	M	S	W	E	T	F	P	K	K	K	H	P	T	D	N	Y	D	L	T	P
G	M	S	W	E	T	F	P	K	K	K	H	P	T	D	N	Y	D	L	T	P
G	M	S	W	E	T	F	P	K	K	K	H	P	T	D	N	Y	D	L	T	P
G	M	S	W	E	T	F	P	K	K	K	H	P	T	D	N	Y	D	L	T	P
G	M	S	W	E	T	F	P	K	K	K	H	P	T	D	N	Y	D	L	T	P
G	M	S	W	E	T	F	P	K	K	K	H	P	T	D	N	Y	D	L	T	P
G	M	S	W	E	T	F	P	K	K	K	H	P	T	D	N	Y	D	L	T	P
G	M	S	W	E	T	F	P	K	K	K	H	P	T	D	N	Y	D	L	T	P

G	M	S	W	E	T	F	P	K	K	K	H	P	T	D	N	Y	D	L	T	P
G	M	S	W	E	T	F	P	K	K	K	H	P	T	D	N	Y	D	L	T	P
G	M	S	W	E	T	F	P	K	K	K	H	P	T	D	N	Y	D	L	T	P
G	M	S	W	E	T	F	P	K	K	K	H	P	T	D	N	Y	D	L	T	P
G	M	S	W	E	T	F	P	K	K	K	H	P	T	D	N	Y	D	L	T	P
G	M	S	W	E	T	F	P	K	K	K	H	P	T	D	N	Y	D	L	T	P
G	M	S	W	E	T	F	P	K	K	K	H	P	T	D	N	Y	D	L	T	P
G	M	S	W	E	T	F	P	K	K	K	H	P	T	D	N	Y	D	L	T	P
G	M	S	W	E	T	F	P	K	K	K	H	P	T	D	N	Y	D	L	T	P
G	M	S	W	E	T	L	P	T	K	K	H	P	E	N	N	Y	D	L	A	S
G	M	S	W	E	T	L	P	T	K	K	H	P	E	N	N	Y	D	L	A	S
G	M	S	W	E	T	L	P	T	K	K	H	P	E	N	N	Y	D	L	A	S
S	M	S	W	E	T	L	P	T	K	K	H	P	E	N	N	Y	D	L	A	S
S	M	S	W	E	T	L	P	T	K	K	H	P	E	N	N	Y	D	L	A	S
S	M	S	W	E	T	L	P	T	K	K	H	P	E	N	N	Y	D	L	A	S
S	M	S	W	E	T	L	P	T	K	K	H	P	E	N	N	Y	D	L	A	S
S	M	S	W	E	T	L	P	T	K	K	H	P	E	N	N	Y	D	L	A	S
S	M	S	W	E	T	L	P	T	K	K	H	P	E	N	N	Y	D	L	A	S
S	M	S	W	E	T	L	P	T	K	K	H	P	E	N	N	Y	D	L	A	S
S	M	S	W	E	T	L	P	T	K	K	H	P	E	N	N	Y	D	L	A	S
S	M	S	W	E	T	L	P	T	K	K	H	P	E	N	N	Y	D	L	A	S
G	M	F	W	D	T	L	P	K	K	K	Q	P	T	D	N	Y	D	L	A	P
G	M	F	W	D	T	L	P	K	K	K	Q	P	T	D	N	Y	D	L	A	P
G	M	F	W	D	T	L	P	K	K	K	Q	P	T	D	N	Y	D	L	A	P
G	M	F	W	D	T	L	P	K	K	K	Q	P	T	D	N	Y	D	L	A	P
G	M	F	W	D	T	L	P	K	K	K	Q	P	T	D	N	Y	D	L	A	P
G	M	F	W	D	T	L	P	K	K	K	Q	P	T	D	N	Y	D	L	A	P
G	M	F	W	D	T	L	P	K	K	K	Q	P	T	D	N	Y	D	L	A	P
G	M	F	W	D	T	L	P	K	K	K	Q	P	T	D	N	Y	D	L	A	P
G	M	F	W	D	T	L	P	K	K	K	Q	P	T	D	N	Y	D	L	A	P
G	M	F	W	D	T	L	P	K	K	K	Q	P	T	D	N	Y	D	L	A	P
G	M	F	W	D	T	L	P	K	K	K	Q	P	T	D	N	Y	D	L	A	P
G	M	F	W	D	T	L	P	K	K	K	Q	P	T	D	N	Y	D	L	A	P
G	M	F	W	D	T	L	P	K	K	K	Q	P	T	D	N	Y	D	L	A	P
G	M	F	W	D	T	L	P	K	K	K	Q	P	T	D	N	Y	D	L	A	P
G	M	F	W	D	T	L	P	K	K	K	Q	P	T	D	N	Y	D	L	A	P
G	M	F	W	D	T	L	P	K	K	K	Q	P	T	D	N	Y	D	L	A	P
G	M	F	W	D	T	L	P	K	K	K	Q	P	T	D	N	Y	D	L	A	P
G	M	F	W	D	T	L	P	K	K	K	Q	P	T	D	N	Y	D	L	A	P
G	M	F	W	D	T	L	P	K	K	K	Q	P	T	D	N	Y	D	L	A	P
G	M	F	W	D	T	L	P	K	K	K	Q	P	T	D	N	Y	D	L	A	P
G	M	F	W	D	T	L	P	K	K	K	Q	P	T	D	N	Y	D	L	A	P
G	M	F	W	D	T	L	P	K	K	K	Q	P	T	D	N	Y	D	L	A	P
G	M	F	W	D	T	L	P	K	K	K	Q	P	T	D	N	Y	D	L	A	P
G	M	F	W	D	T	L	P	K	K	K	Q	P	T	D	N	Y	D	L	A	P
G	M	F	W	D	T	L	P	K	K	K	Q	P	T	D	N	Y	D	L	A	P
G	M	F	W	D	T	L	P	K	K	K	Q	P	T	D	N	Y	D	L	A	P
G	M	F	W	D	T	L	P	K	K	K	Q	P	T	D	N	Y	D	L	A	P
G	M	F	W	E	T	L	P	T	K	K	H	P	E	N	N	Y	D	V	T	P
G	M	F	W	E	T	L	P	T	K	K	H	P	E	N	N	Y	D	L	A	S
G	M	F	W	E	T	L	P	T	K	K	H	P	E	N	N	Y	D	L	A	S
G	M	F	W	E	T	L	P	T	K	K	H	P	E	N	N	Y	D	L	A	S
G	M	F	W	E	T	L	P	T	K	K	H	P	E	N	N	Y	D	L	A	S
G	M	F	W	E	T	L	P	T	K	K	H	P	E	N	N	Y	D	L	A	S
G	M	F	W	E	T	L	P	T	K	K	H	P	E	N	N	Y	D	L	A	S
G	M	F	W	E	T	L	P	T	K	K	H	P	E	N	N	Y	D	L	A	S
G	M	F	W	E	T	L	P	T	K	K	H	P	E	N	N	Y	D	L	A	S
G	M	F	W	E	T	L	P	T	K	K	H	P	E	N	N	Y	D	L	A	S
G	M	F	W	E	T	L	P	T	K	K	H	P	E	N	N	Y	D	L	A	S
G	M	F	W	E	T	L	P	T	K	K	H	P	E	N	N	Y	D	L	A	S
G	M	F	W	E	T	L	P	T	K	K	H	P	E	N	N	Y	D	L	A	S
G	M	F	W	E	T	L	P	T	K	K	H	P	E	N	N	Y	D	L	A	S
G	M	F	W	E	T	L	P	T	K	K	H	P	E	N	N	Y	D	L	A	S
G	M	F	W	E	T	L	P	T	K	K	H	P	E	N	N	Y	D	L	A	S
G	M	F	W	E	T	L	P	T	K	K	H	P	E	N	N	Y	D	L	A	S
G	M	F	W	E	T	L	P	T	K	K	H	P	E	N	N	Y	D	L	A	S
G	M	F	W	E	T	L	P	T	K	K	H	P	E	N	N	Y	D	L	A	S

G	M	F	W	E	T	L	P	T	K	K	H	P	E	N	N	Y	D	L	A	S
G	M	F	W	E	T	L	P	T	K	K	H	P	E	N	N	Y	D	L	A	S
G	M	F	W	E	T	L	P	T	K	K	H	P	E	N	N	Y	D	L	A	S
G	M	F	W	E	T	L	P	T	K	K	H	P	E	N	N	Y	D	L	A	S
G	M	F	W	E	T	L	P	T	K	K	H	P	E	N	N	Y	D	L	A	S
G	M	F	W	E	T	L	P	T	K	K	H	P	E	N	N	Y	D	L	A	S
G	M	F	W	E	T	L	P	T	K	K	H	P	E	N	N	Y	D	L	A	S
G	M	F	W	E	T	L	P	T	K	K	H	P	E	N	N	Y	D	L	A	S
G	M	F	W	E	T	L	P	T	K	K	H	P	E	N	N	Y	D	L	A	S
G	M	F	W	E	T	L	P	T	K	K	H	P	E	N	N	Y	D	L	A	S
G	M	F	W	E	T	L	P	T	K	K	H	P	E	N	N	Y	D	L	A	S
G	M	F	W	E	T	L	P	T	K	K	H	P	E	N	N	Y	D	L	A	S
G	M	F	W	E	T	L	P	T	K	K	H	P	E	N	N	Y	D	L	A	S
G	M	F	W	E	T	L	P	T	K	K	H	P	E	N	N	Y	D	L	A	S
G	M	F	W	E	T	L	P	T	K	K	H	P	E	N	N	Y	D	L	A	S
G	M	F	W	E	T	L	P	T	K	K	H	P	E	N	N	Y	D	L	A	S
G	M	F	W	E	T	L	P	T	K	K	H	P	E	N	N	Y	D	L	A	S
G	M	F	W	E	T	L	P	T	K	K	H	P	E	N	N	Y	D	L	A	S
G	M	F	W	E	T	L	P	T	K	K	H	P	E	N	N	Y	D	L	A	S
G	M	F	W	E	T	L	P	T	K	K	H	P	E	N	N	Y	D	L	A	S
G	M	F	W	E	T	L	P	T	K	K	H	P	E	N	N	Y	D	L	A	S
G	M	F	W	E	T	L	P	T	K	K	H	P	E	N	N	Y	D	L	A	S
G	M	F	W	E	T	L	P	T	K	K	H	P	E	N	N	Y	D	L	A	S
G	M	F	W	E	T	L	P	T	K	K	H	P	E	N	N	Y	D	L	A	S
G	M	F	W	E	T	L	P	T	K	K	H	P	E	N	N	Y	D	L	A	S
G	M	F	W	E	T	L	P	T	K	K	H	P	E	N	N	Y	D	L	A	S
G	M	F	W	E	T	L	P	T	K	K	H	P	E	N	N	Y	D	L	A	S
G	M	F	W	E	T	L	P	T	K	K	H	P	E	N	N	Y	D	L	A	S
G	M	F	W	E	T	L	P	T	K	K	H	P	E	N	N	Y	D	L	A	S
G	M	F	W	E	T	L	P	T	K	K	H	P	E	N	N	Y	D	L	A	S
G	M	F	W	E	T	L	P	T	K	K	H	P	E	N	N	Y	D	L	A	S
G	M	F	W	E	T	L	P	T	K	K	H	P	E	N	N	Y	D	L	A	S
G	M	F	W	E	T	L	P	T	K	K	H	P	E	N	N	Y	D	L	A	S
G	M	F	W	E	T	L	P	T	K	K	H	P	E	N	N	Y	D	L	A	S
G	M	F	W	E	T	L	P	T	K	K	H	P	E	N	N	Y	D	L	A	S
G	M	F	W	E	T	L	P	T	K	K	H	P	E	N	N	Y	D	L	A	S
G	M	F	W	E	T	L	P	T	K	K	H	P	E	N	N	Y	D	L	A	S
G	M	F	W	E	T	L	P	T	K	K	H	P	E	N	N	Y	D	L	A	S
G	M	F	W	E	T	L	P	T	K	K	H	P	E	N	N	Y	D	L	A	S
G	M	F	W	E	T	L	P	T	K	K	H	P	E	N	N	Y	D	L	A	S
G	M	F	W	E	T	L	P	T	K	K	H	P	E	N	N	Y	D	L	A	S
G	M	F	W	E	T	L	P	T	K	K	H	P	E	N	N	Y	D	L	A	S
G	M	F	W	E	T	L	P	T	K	K	H	P	E	N	N	Y	D	L	A	S
G	M	F	W	E	T	L	P	T	K	K	H	P	E	N	N	Y	D	L	A	S
G	M	F	W	E	T	L	P	T	K	K	H	P	E	N	N	Y	D	L	A	S
G	M	F	W	E	T	L	P	T	K	K	H	P	E	N	N	Y	D	L	A	S
G	M	F	W	E	T	L	P	T	K	K	H	P	E	N	N	Y	D	L	A	S
G	M	F	W	E	T	L	P	T	K	K	H	P	E	N	N	Y	D	L	A	S
G	M	F	W	E	T	L	P	T	K	K	H	P	E	N	N	Y	D	L	A	S
G	M	F	W	E	T	L	P	T	K	K	H	P	E	N	N	Y	D	L	A	S
G	M	F	W	E	T	L	P	T	K	K	H	P	E	N	N	Y	D	L	A	S
G	M	F	W	E	T	L	P	T	K	K	H	P	E	N	N	Y	D	L	A	S
G	M	F	W	E	T	L	P	T	K	K	H	P	E	N	N	Y	D	L	A	S
G	M	F	W	E	T	L	P	T	K	K	H	P	E	N	N	Y	D	L	A	S
G	M	F	W	E	T	L	P	T	K	K	H	P	E	N	N	Y	D	L	A	S
G	M	F	W	E	T	L	P	T	K	K	H	P	E	N	N	Y	D	L	A	S
G	M	F	W	E	T	L	P	T	K	K	H	P	E	N	N	Y	D	L	A	S
G	M	F	W	E	T	L	P	T	K	K	H	P	E	N	N	Y	D	L	A	S
G	M	F	W	E	T	L	P	T	K	K	H	P	E	N	N	Y	D	L	A	S
G	M	F	W	E	T	L	P	T	K	K	H	P	E	N	N	Y	D	L	A	S
G	M	F	W	E	T	L	P	T	K	K	H	P	E	N	N	Y	D	L	A	S
G	M	F	W	E	T	L	P	T	K	K	H	P	E	N	N	Y	D	L	A	S
G	M	F	W	E	T	L	P	T	K	K	H	P	E	N	N	Y	D	L	A	S
G	M	F	W	E	T	L	P	T	K	K	H	P	E	N	N	Y	D	L	A	S
G	M	F	W	E	T	L	P	T	K	K	H	P	E	N	N	Y	D	L	A	S
G	M	F	W	E	T	L	P	T	K	K	H	P	E	N	N	Y	D	L	A	S
G	M	F	W	E	T	L	P	T	K	K	H	P	E	N	N	Y	D	L	A	S

G	M	F	W	E	T	L	P	T	K	K	H	P	E	N	N	Y	D	L	A	S
G	M	F	W	E	T	L	P	T	K	K	H	P	E	N	N	Y	D	L	A	S
G	M	F	W	E	T	L	P	T	K	K	H	P	E	N	N	Y	D	L	A	S
G	M	F	W	E	T	L	P	T	K	K	H	P	E	N	N	Y	D	L	A	S
G	M	F	W	E	T	L	P	T	K	K	H	P	E	N	N	Y	D	L	A	S
G	M	F	W	E	T	L	P	T	K	K	H	P	E	N	N	Y	D	L	A	S
G	M	F	W	E	T	L	P	T	K	K	H	P	E	N	N	Y	D	L	A	S
G	M	F	W	E	T	L	P	T	K	K	H	P	E	N	N	Y	D	L	A	S
G	M	F	W	E	T	L	P	T	K	K	H	P	E	N	N	Y	D	L	A	S
G	M	F	W	E	T	L	P	T	K	K	H	P	E	N	N	Y	D	L	A	S
G	M	F	W	E	T	L	P	T	K	K	H	P	E	N	N	Y	D	L	A	S
G	M	F	W	E	T	L	P	T	K	K	H	P	E	N	N	Y	D	L	A	S
G	M	F	W	E	T	L	P	T	K	K	H	P	E	N	N	Y	D	L	A	S
G	M	F	W	E	T	L	P	T	K	K	H	P	E	N	N	Y	D	L	A	S
G	M	F	W	E	T	L	P	T	K	K	H	P	E	N	N	Y	D	L	A	S
G	M	F	W	E	T	L	P	T	K	K	H	P	E	N	N	Y	D	L	A	S
G	M	F	W	E	T	L	P	T	K	K	H	P	E	N	N	Y	D	L	A	S
G	M	F	W	E	T	L	P	T	K	K	H	P	E	N	N	Y	D	L	A	S
G	M	F	W	E	T	L	P	T	K	K	H	P	E	N	N	Y	D	L	A	S
G	M	F	W	E	T	L	P	T	K	K	H	P	E	N	N	Y	D	L	A	S
G	M	F	W	E	T	L	P	T	K	K	H	P	E	N	N	Y	D	L	A	S
G	M	F	W	E	T	L	P	T	K	K	H	P	E	N	N	Y	D	L	A	S
G	M	F	W	E	T	L	P	T	K	K	H	P	E	N	N	Y	D	L	A	S
G	M	F	W	E	T	L	P	T	K	K	H	P	E	N	N	Y	D	L	A	S
G	M	F	W	E	T	L	P	T	K	K	H	P	E	N	N	Y	D	L	A	S
G	M	F	W	E	T	L	P	T	K	K	H	P	E	N	N	Y	D	L	A	S
G	M	F	W	E	T	L	P	T	K	K	H	P	E	N	N	Y	D	L	A	S

135	148	168	180	182	194	210	211	213	216	217	222	226	227
A	E	A	S	F	A	D	I	D	K	I	L	F	D
A	E	A	S	F	A	D	I	D	K	I	L	F	D
A	E	A	S	F	A	D	V	D	K	I	L	F	D
A	E	A	S	F	A	D	V	D	K	I	L	F	D
A	E	A	S	F	A	D	V	D	K	I	L	F	D
A	E	A	S	F	A	D	V	D	K	I	L	F	D
A	E	A	S	F	A	D	V	D	K	I	L	F	D
A	E	A	S	F	A	D	V	D	K	I	L	F	D
A	E	A	S	F	A	D	V	D	K	I	L	F	D
A	E	A	S	F	A	D	V	D	K	I	L	F	D
A	E	A	S	F	A	D	V	D	K	I	L	F	D
A	E	A	S	F	A	D	V	D	K	I	L	F	D
A	E	A	S	F	A	D	V	D	K	I	L	F	D
A	E	A	S	F	A	D	V	D	K	I	L	F	D
A	E	A	S	F	A	D	V	D	K	I	L	F	D
A	E	A	S	F	A	D	V	D	K	I	L	F	D
A	E	A	S	F	A	D	V	D	K	I	L	F	D
A	E	A	S	F	A	D	V	D	K	I	L	F	D
A	E	A	S	F	A	D	V	D	K	I	L	F	D
A	E	A	S	F	A	D	V	D	K	I	L	F	D
A	E	A	S	F	A	D	V	D	K	I	L	F	D
A	E	A	S	F	A	D	V	D	K	I	L	F	D
A	E	A	S	F	A	D	V	D	K	I	L	F	D
A	E	A	S	F	A	D	V	D	K	I	L	F	D
A	E	A	S	F	A	D	V	D	K	I	L	F	D
A	E	A	S	F	A	D	V	D	K	I	L	F	D
A	E	A	S	F	A	D	V	D	K	I	L	F	D
A	E	A	S	F	A	D	V	D	K	I	L	F	D
A	E	A	S	F	A	D	V	D	K	I	L	F	D
A	E	A	S	F	A	D	V	D	K	I	L	F	D
A	E	A	S	F	A	D	V	D	K	I	L	F	D
A	E	A	S	F	A	D	V	D	K	I	L	F	D
A	E	A	S	F	A	D	V	D	K	I	L	F	D
A	E	A	S	F	A	D	V	D	K	I	L	F	D
A	E	A	S	F	A	D	V	D	K	I	L	F	D
A	E	A	S	F	A	D	V	D	K	I	L	F	D
A	E	A	S	F	A	D	V	D	K	I	L	F	D
A	E	A	S	F	A	D	V	D	K	I	L	F	D
A	E	A	S	F	A	D	V	D	K	I	L	F	D
A	E	A	S	F	A	D	V	D	K	I	L	F	D
A	E	A	S	F	A	D	V	D	K	I	L	F	D
A	E	A	S	F	A	D	V	D	K	I	L	F	D
A	E	A	S	F	A	D	V	D	K	I	L	F	D
A	E	A	S	F	A	D	V	D	K	I	L	F	D
A	E	A	S	F	A	D	V	D	K	I	L	F	D
A	E	A	S	F	A	D	V	D	K	I	L	F	D
A	E	A	S	F	A	D	V	D	K	I	L	F	D
A	E	A	S	F	A	D	V	D	K	I	L	F	D
A	E	A	S	F	A	D	V	D	K	I	L	F	D
A	E	A	S	F	A	D	V	D	K	I	L	F	D
A	E	A	S	F	A	D	V	D	K	I	L	F	D
A	E	A	S	F	A	D	V	D	K	I	L	F	D
A	E	A	S	F	A	D	V	D	K	I	L	F	D
A	E	A	S	F	A	D	I	D	K	I	L	F	D
A	E	A	S	F	A	D	V	D	K	I	L	F	D
A	E	A	S	F	A	D	V	D	K	I	L	F	D
A	E	A	S	F	A	D	V	D	K	I	L	F	D
A	E	A	S	F	A	D	V	D	K	I	L	F	D
A	E	A	S	F	A	D	V	D	K	I	L	F	D
A	E	A	S	F	A	D	I	D	K	I	L	F	D
A	E	A	S	F	A	D	I	D	K	I	L	F	D
A	E	A	S	F	A	D	I	D	K	I	L	F	D
A	E	A	S	F	A	D	I	D	K	I	L	F	D

9	12	13	14	17	34	45	50
A	D	S	V	A	G	I	S
A	D	S	V	A	G	I	S
A	D	S	V	A	G	T	S
A	D	S	V	A	G	T	S
A	D	S	V	A	G	T	S
A	D	S	V	A	G	T	S
A	D	S	V	A	G	T	S
A	D	S	V	A	G	T	S
A	D	S	V	A	G	T	S
A	D	S	V	A	G	T	S
A	D	S	V	A	G	T	S
A	D	S	V	A	G	T	S
A	D	S	V	A	G	T	S
A	D	S	V	A	G	T	S
A	D	S	V	A	G	T	S
A	D	S	V	A	G	T	S
A	D	S	V	A	G	T	S
A	D	S	V	A	G	T	S
A	D	S	V	A	G	T	S
A	D	S	V	A	G	T	S
A	D	S	V	A	G	T	S
A	D	S	V	A	G	T	S
A	D	S	V	A	G	T	S
A	D	S	V	A	G	T	S
A	D	S	V	A	G	T	S
A	D	S	V	A	G	T	S
A	D	S	V	A	G	T	S
A	D	S	V	A	G	T	S
A	D	S	V	A	G	T	S
A	D	S	V	A	G	T	S
A	D	S	V	A	G	T	S
A	D	S	V	A	G	T	S
A	D	S	V	A	G	T	S
A	D	S	V	A	G	T	S
A	D	S	V	A	G	T	S
A	D	S	V	A	G	T	S
A	D	S	V	A	G	T	S
A	D	S	V	A	G	T	S
A	D	S	V	A	G	T	S
A	D	S	V	A	G	T	S
A	D	S	V	A	G	T	S
A	D	S	V	A	G	T	S
A	D	S	V	A	G	T	S
A	D	S	V	A	G	T	S
A	D	S	V	A	G	T	S
A	D	S	V	A	G	T	S
A	D	S	V	A	G	T	S
A	D	S	V	A	G	T	S
A	D	S	V	A	G	T	S
A	D	S	V	A	G	T	S
A	D	S	V	A	G	T	S
A	D	S	V	A	G	T	S
A	D	S	V	A	G	T	S
A	D	S	V	A	G	I	S
A	D	S	V	A	G	T	S
A	D	S	V	A	G	T	S
A	D	S	V	A	G	T	S
A	D	S	V	A	G	T	S
A	D	S	V	A	G	T	S
A	D	S	V	A	G	I	S
A	D	S	V	A	G	I	S
A	D	S	V	A	G	I	S
A	D	S	V	A	G	I	S

A	E	A	S	F	A	D	I	D	K	I	L	F	D
A	E	A	S	F	A	D	I	D	K	I	L	F	D
A	E	A	S	F	A	D	I	D	K	I	L	F	D
A	E	A	S	F	A	D	I	D	K	I	L	F	D
A	E	A	S	F	A	D	I	D	K	I	L	F	D
A	E	A	S	F	A	D	I	D	K	I	L	F	D
A	E	A	S	F	A	D	I	D	K	I	L	F	D
A	E	A	S	F	A	D	I	D	K	I	L	F	D
A	E	A	S	F	A	D	I	D	K	I	L	F	D
A	E	A	S	F	A	D	I	D	K	I	L	F	D
A	E	A	S	F	A	D	I	D	K	I	L	F	D
A	E	A	S	F	A	D	I	D	K	I	L	F	D
A	E	A	S	F	A	D	I	D	K	I	L	F	D
A	E	A	S	F	A	D	I	D	K	I	L	F	D
A	E	A	S	F	A	D	I	D	K	I	L	F	D
A	E	A	S	F	A	D	I	D	K	I	L	F	D
A	E	A	S	F	A	D	I	D	K	I	L	F	D
A	E	A	S	F	A	D	I	D	K	I	L	F	D
A	E	A	S	F	A	D	I	D	K	I	L	F	D
A	E	A	S	F	A	D	I	D	K	I	L	F	D
A	E	A	S	F	A	D	I	D	K	I	L	F	D
A	E	A	S	F	A	D	V	D	K	I	L	F	D
A	E	A	S	F	A	D	V	D	K	I	L	F	D
A	E	A	S	F	A	D	V	D	K	I	L	F	D
A	E	A	S	F	A	D	V	D	K	I	L	F	E
A	E	A	S	F	A	D	V	D	K	I	L	F	E
A	E	A	S	F	A	D	V	D	K	I	L	F	E
A	E	A	S	F	A	D	I	D	K	I	L	F	D
A	E	A	S	F	A	D	I	D	K	I	L	F	D
A	E	A	S	F	A	D	I	D	K	I	L	F	D
A	E	A	S	F	A	D	I	D	K	I	L	F	D
A	E	A	S	F	A	D	I	D	K	I	L	F	D
A	E	A	S	F	A	D	I	D	K	I	L	F	D
A	E	A	S	F	A	D	I	D	K	I	L	F	D
A	E	A	S	F	A	D	I	D	K	I	L	F	D
A	E	A	S	F	A	D	I	D	K	I	L	F	D
A	E	A	S	F	A	D	I	D	K	I	L	F	D
A	E	A	S	F	A	D	I	D	K	I	L	F	D
A	E	A	S	F	A	D	I	D	K	I	L	F	D
A	E	A	S	F	A	D	I	D	K	I	L	F	D
A	E	A	S	F	A	D	I	D	K	I	L	F	D
A	E	A	S	F	A	D	I	D	K	I	L	F	D
A	E	A	S	F	A	D	I	D	K	I	L	F	D
A	E	A	S	F	A	D	I	D	K	I	L	F	D
A	E	A	S	F	A	D	I	D	K	I	L	F	D
A	E	A	S	F	A	D	I	D	K	I	L	F	D
A	E	A	S	F	A	D	I	D	K	I	L	F	D
A	E	A	S	F	A	D	I	D	K	I	L	F	D
A	E	A	S	F	A	D	I	D	K	I	L	F	D
A	E	A	S	F	A	D	I	D	K	I	L	F	D
A	E	A	S	F	A	D	I	D	K	I	L	F	D
A	E	A	S	F	A	D	I	D	K	I	L	F	D
A	E	A	S	F	A	D	I	D	K	I	L	F	D
A	E	A	S	F	A	D	I	D	K	I	L	F	D
A	E	A	S	F	A	D	I	D	K	I	L	F	D
A	E	A	S	F	A	D	I	D	K	I	L	F	D
A	E	A	S	F	A	D	I	D	K	I	L	F	D
A	E	A	S	F	A	D	I	D	K	I	L	F	D
A	E	A	S	F	A	D	I	D	K	I	L	F	D
A	E	A	S	F	A	D	I	D	K	I	L	F	D
A	E	A	S	F	A	D	I	D	K	I	L	F	D
A	E	A	S	F	A	D	I	D	K	I	L	F	D
A	E	A	S	F	A	D	I	D	K	I	L	F	D
A	E	A	S	F	A	D	I	D	K	I	L	F	D
A	E	A	S	F	A	D	I	D	K	I	L	F	D
A	E	A	S	F	A	D	I	D	K	I	L	F	D
A	E	A	S	F	A	D	I	D	K	I	L	F	D

A	D	S	V	A	G	I	S
A	D	S	V	A	G	I	S
A	D	S	V	A	G	I	S
A	D	S	V	A	G	I	S
A	D	S	V	A	G	I	S
A	D	S	V	A	G	I	S
A	D	S	V	A	G	I	S
A	D	S	V	A	G	I	S
A	D	S	V	A	G	I	S
A	D	S	V	A	G	I	S
A	D	S	V	A	G	I	S
A	D	S	V	A	G	I	S
A	D	S	V	A	G	I	S
A	D	S	V	A	G	I	S
A	D	S	V	A	G	I	S
A	D	S	V	A	G	I	S
A	D	S	V	A	G	I	S
A	D	S	V	A	G	I	S
A	D	S	V	A	G	I	S
A	D	S	V	A	G	I	S
A	D	S	V	A	G	I	S
A	D	S	V	A	G	T	S
A	D	S	V	A	G	T	S
A	D	S	V	A	G	T	S
A	D	S	V	A	G	T	S
A	D	S	V	A	G	T	S
A	D	S	V	A	G	T	S
A	D	S	V	A	G	I	S
A	D	S	V	A	G	I	S
A	D	S	V	A	G	I	S
A	D	S	V	A	G	I	S
A	D	S	V	A	G	I	S
A	D	S	V	A	G	I	S
A	D	S	V	A	G	I	S
A	D	S	V	A	G	I	S
A	D	S	V	A	G	I	S
A	D	S	V	A	G	I	S
A	D	S	V	A	G	I	S
A	D	S	V	A	G	I	S
A	D	S	V	A	G	I	S
A	D	S	V	A	G	I	S
A	D	S	V	A	G	I	S
A	D	S	V	A	G	I	S
A	D	S	V	A	G	I	S
A	D	S	V	A	G	I	S
A	D	S	V	A	G	I	S
A	D	S	V	A	G	I	S
A	D	S	V	A	G	I	S
A	D	S	V	A	G	I	S
A	D	S	V	A	G	I	S
A	D	S	V	A	G	I	S
A	D	S	V	A	G	I	S
A	D	S	V	A	G	I	S
A	D	S	V	A	G	I	S
A	D	S	V	A	G	I	S
A	D	S	V	A	G	I	S
A	D	S	V	A	G	I	S
A	D	S	V	A	G	I	S
A	D	S	V	A	G	I	S
A	D	S	V	A	G	I	S
A	D	S	V	A	G	I	S
A	D	S	V	A	G	I	S
A	D	S	V	A	G	I	S
A	D	S	V	A	G	I	S
A	D	S	V	A	G	I	S
A	D	S	V	A	G	I	S
A	D	S	V	A	G	I	S

A	E	A	S	F	A	D	I	D	K	I	L	F	D
A	E	A	S	F	A	D	I	D	K	I	L	F	D
A	E	A	S	F	A	D	I	D	K	I	L	F	D
A	E	A	S	F	A	D	I	D	K	I	L	F	D
A	E	A	S	F	A	D	I	D	K	I	L	F	D
A	E	A	S	F	A	D	I	D	K	I	L	F	D
A	E	A	S	F	A	D	I	D	K	I	L	F	D
A	E	A	S	F	A	D	I	D	K	I	L	F	D
A	E	A	S	F	A	D	I	D	K	I	L	F	D
A	E	A	S	F	A	D	I	D	K	I	L	F	D
A	E	A	S	F	A	D	I	D	K	I	L	F	D
A	E	A	S	F	A	D	I	D	K	I	L	F	D
A	E	A	S	F	A	D	I	D	K	I	L	F	D
A	E	A	S	F	A	D	I	D	K	I	L	F	D
A	E	A	S	F	A	D	I	D	K	I	L	F	D
A	E	A	S	F	A	D	I	D	K	I	L	F	D
A	E	A	S	F	A	D	I	D	K	I	L	F	D
A	E	A	S	F	A	D	I	D	K	I	L	F	D
A	E	A	S	F	A	D	I	D	K	I	L	F	D
A	E	A	S	F	A	D	I	D	K	I	L	F	D
A	E	A	S	F	A	D	I	D	K	I	L	F	D
A	E	A	S	F	A	D	I	D	K	I	L	F	D
A	E	A	S	F	A	D	I	D	K	I	L	F	D
A	E	A	S	F	A	D	I	D	K	I	L	F	D
A	E	A	S	F	A	D	I	D	K	I	L	F	D
A	E	A	S	F	A	D	I	D	K	I	L	F	D
A	E	A	S	F	A	D	I	D	K	I	L	F	D
A	E	A	S	F	A	D	I	D	K	I	L	F	D
A	E	A	S	F	A	D	I	D	K	I	L	F	D
A	E	A	S	F	A	D	I	D	K	I	L	F	D
A	E	A	S	F	A	D	I	D	K	I	L	F	D
A	E	A	S	F	A	D	I	D	K	I	L	F	D
A	E	A	S	F	A	D	I	D	K	I	L	F	D
A	E	A	S	F	A	D	I	D	K	I	L	F	D
A	E	A	S	F	A	D	I	D	K	I	L	F	D
A	E	A	S	F	A	D	I	D	K	I	L	F	D
A	E	A	S	F	A	D	I	D	K	I	L	F	D
A	E	A	S	F	A	D	I	D	K	I	L	F	D
A	E	A	S	F	A	D	I	D	K	I	L	F	D
A	E	A	S	F	A	D	I	D	K	I	L	F	D
A	E	A	S	F	A	D	I	D	K	I	L	L	D
A	E	A	S	F	A	D	I	D	K	I	L	F	D
A	E	A	S	F	A	D	I	D	K	I	L	F	D
A	E	A	S	F	A	D	I	D	K	I	L	F	D
A	E	A	S	F	A	D	I	D	K	I	L	F	D
A	E	A	S	F	A	D	I	D	K	I	L	F	D
A	E	A	S	F	A	D	I	D	K	I	L	F	D
A	E	A	S	F	A	D	I	D	K	I	L	F	D
A	E	A	S	F	A	D	I	D	K	I	L	F	D
A	E	A	S	F	A	D	I	D	K	I	L	F	D
A	E	A	S	F	A	D	I	D	K	I	L	F	D
A	E	A	S	F	A	D	I	D	K	I	L	F	D
A	E	A	S	F	A	D	I	D	K	I	L	F	D
A	E	A	S	F	A	D	I	D	K	I	L	F	D
A	E	A	S	F	A	D	I	D	K	I	L	F	D
A	E	A	S	F	A	D	I	D	K	I	L	F	D
A	E	A	S	F	A	D	I	D	K	I	L	F	D
A	E	A	S	F	A	D	I	D	K	I	L	F	D
A	E	A	S	F	A	D	I	D	K	I	L	F	D
A	E	A	S	F	A	D	I	D	K	I	L	F	D
A	E	A	S	F	A	D	I	D	K	I	L	F	D
A	E	A	S	F	A	D	I	D	K	I	L	F	D
A	E	A	S	F	A	D	I	D	K	I	L	F	D
A	E	A	S	F	A	D	I	D	K	I	L	F	D
A	E	A	S	F	A	D	I	D	K	I	L	F	D
A	E	A	S	F	A	D	I	D	K	I	L	F	D
A	E	A	S	F	A	D	I	D	K	I	L	F	D

A	D	S	V	A	G	I	S
A	D	S	V	A	G	I	S
A	D	S	V	A	G	I	S
A	D	S	V	A	G	I	S
A	D	S	V	A	G	I	S
A	D	S	V	A	G	I	S
A	D	S	V	A	G	I	S
A	D	S	V	A	G	I	S
A	D	S	V	A	G	I	S
A	D	S	V	A	G	I	S
A	D	S	V	A	G	I	S
A	D	S	V	A	G	I	S
A	D	S	V	A	G	I	S
A	D	S	V	A	G	I	S
A	D	S	V	A	G	I	S
A	D	S	V	A	G	I	S
A	D	S	V	A	G	I	S
A	D	S	V	A	G	I	S
A	D	S	V	A	G	I	S
A	D	S	V	A	G	I	S
A	D	S	V	A	G	I	S
A	D	S	V	A	G	I	S
A	D	S	V	A	G	I	S
A	D	S	V	A	G	I	S
A	D	S	V	A	G	I	S
A	D	S	V	A	G	I	S
A	D	S	V	A	G	I	S
A	D	S	V	A	G	I	S
A	D	S	V	A	G	I	S
A	D	S	V	A	G	I	S
A	D	S	V	A	G	I	S
A	D	S	V	A	G	I	S
A	D	S	V	A	G	I	S
A	D	S	V	A	G	I	S
A	D	S	V	A	G	I	S
A	D	S	V	A	G	I	S
A	D	S	V	A	G	I	S
A	D	S	V	A	G	I	S
A	D	S	V	A	G	I	S
A	D	S	V	A	G	I	S
A	D	S	V	A	G	I	S
A	D	S	V	A	G	I	S
A	D	S	V	A	G	I	S
A	D	S	V	A	G	I	S
A	D	S	V	A	G	I	S
A	D	S	V	A	G	I	S
A	D	S	V	A	G	I	S
A	D	S	V	A	G	I	S
A	D	S	V	A	G	I	S
A	D	S	V	A	G	I	S
A	D	S	V	A	G	I	S
A	D	S	V	A	G	I	S
A	D	S	V	A	G	I	S
A	D	S	V	A	G	I	S
A	D	S	V	A	G	I	S
A	D	S	V	A	G	I	S
A	D	S	V	A	G	I	S
A	D	S	V	A	G	I	S
A	D	S	V	A	G	I	S
A	D	S	V	A	G	I	S
A	D	S	V	A	G	I	S
A	D	S	V	A	G	I	S
A	D	S	V	A	G	I	S
A	D	S	V	A	G	I	S
A	D	S	V	A	G	I	S
A	D	S	V	A	G	I	S
A	D	S	V	A	G	I	S

A	E	A	S	F	A	D	I	D	K	I	L	F	D	A	D	S	V	A	G	I	S
A	E	A	S	F	A	D	I	D	K	I	L	F	D	A	D	S	V	A	G	I	S
A	E	A	S	F	A	D	I	D	K	I	L	F	D	A	D	S	V	A	G	I	S
A	E	A	S	F	A	D	I	D	K	I	L	F	D	A	D	S	V	A	G	I	S
A	E	A	S	F	A	D	V	D	K	I	L	F	D	A	D	S	V	V	G	T	S
A	E	A	S	F	A	D	V	D	K	I	L	F	D	A	D	S	V	V	G	T	S
A	E	A	S	F	A	D	V	D	K	I	L	F	D	A	D	S	V	V	G	T	S
A	E	A	S	F	A	D	V	D	K	I	L	F	D	A	D	S	V	V	G	T	S
A	E	A	S	F	A	D	V	D	K	I	L	F	D	A	D	S	V	V	G	T	S
A	E	A	S	F	A	D	V	D	K	I	L	F	D	A	D	S	V	V	G	T	S
A	E	A	S	F	A	D	V	D	K	I	L	F	D	A	D	S	V	V	G	T	S
A	E	A	S	F	A	D	V	D	K	I	L	F	D	A	D	S	V	V	G	T	S
A	E	A	S	F	A	D	V	D	K	I	L	F	D	A	D	S	V	V	G	T	S
A	E	A	S	F	A	D	V	D	K	I	L	F	D	A	D	S	V	V	G	T	S
A	E	A	S	F	A	D	V	D	K	I	L	F	D	A	D	S	V	V	G	T	S
A	E	A	S	F	A	D	V	D	K	I	L	F	D	A	D	S	V	V	G	T	S
A	E	A	S	F	A	D	V	D	K	I	L	F	D	A	D	S	V	V	G	T	S
A	E	A	S	F	A	D	V	D	K	I	L	F	D	A	D	S	V	V	G	T	S
A	E	A	S	F	A	D	V	D	K	I	L	F	D	A	D	S	V	V	G	T	S
A	E	A	S	F	A	D	V	D	K	I	L	F	D	A	D	S	V	V	G	T	S
A	E	A	S	F	A	D	V	D	K	I	L	F	D	A	D	S	V	V	G	T	S
A	E	A	S	F	A	D	V	D	K	I	L	F	D	A	D	S	V	V	G	T	S
A	E	A	S	F	A	D	V	D	K	I	L	F	D	A	D	S	V	V	G	T	S
A	E	A	S	F	A	D	V	D	K	I	L	F	D	A	D	S	V	V	G	T	S
A	E	A	S	F	A	D	V	D	K	I	L	F	D	A	D	S	V	V	G	T	S
A	E	A	S	F	A	D	V	D	K	I	L	F	D	A	D	S	V	V	G	T	S
A	E	A	S	F	A	D	V	D	K	I	L	F	D	A	D	S	V	V	G	T	S
A	E	A	S	F	A	D	V	D	K	I	L	F	D	A	D	S	V	V	G	T	S
A	E	V	S	F	A	D	V	D	K	I	L	F	D	A	D	S	V	V	G	T	S
A	E	A	S	F	A	D	V	D	K	I	L	F	D	A	D	S	V	V	G	T	S
A	E	A	S	F	A	D	V	D	K	I	L	F	D	A	D	S	V	V	G	T	S
A	E	A	S	F	A	D	V	D	K	I	L	F	D	A	D	S	V	V	G	T	S
A	E	A	S	F	A	D	V	D	K	I	L	F	D	A	D	S	V	V	G	T	S
A	E	A	S	F	A	D	V	D	K	I	L	F	D	A	D	S	V	V	G	T	S
A	E	A	S	F	A	D	V	D	K	I	L	F	D	A	D	S	V	V	G	T	S
A	E	A	S	F	A	D	V	D	K	I	L	F	D	A	D	S	V	V	G	T	S
A	E	A	S	F	A	D	V	D	K	I	L	F	D	A	D	S	V	V	G	T	S
A	E	A	S	F	A	D	V	D	K	I	L	F	D	A	D	S	V	V	G	T	S
A	E	A	S	F	A	D	V	D	K	I	L	F	D	A	D	S	V	V	G	T	S
A	E	A	S	F	A	D	V	D	K	I	L	F	D	A	D	S	V	V	G	T	S
A	E	A	S	F	A	D	V	D	K	I	L	F	D	A	D	S	V	V	G	T	S
A	E	A	S	F	A	D	V	D	K	I	L	F	D	A	D	S	V	V	G	T	S
A	E	A	S	F	A	D	V	D	K	I	L	F	D	A	D	S	V	V	G	T	S
A	E	A	S	F	A	D	V	D	K	I	L	F	D	A	D	S	V	V	G	T	S
A	E	A	S	F	A	D	V	D	K	I	L	F	D	A	D	S	V	V	G	T	S
A	E	A	S	F	A	D	V	D	K	I	L	F	D	A	D	S	V	V	G	T	S
A	E	A	S	F	A	D	V	D	K	I	L	F	D	A	D	S	V	V	G	T	S
A	E	A	S	F	A	D	V	D	K	I	L	F	D	A	D	S	V	V	G	T	S
A	E	A	S	F	A	D	V	D	K	I	L	F	D	A	D	S	V	V	G	T	S
A	E	A	S	F	A	D	V	D	K	I	L	F	D	A	D	S	V	V	G	T	S
A	E	A	S	F	A	D	V	D	K	I	L	F	D	A	D	S	V	V	G	T	S
A	E	A	S	F	A	D	V	D	K	I	L	F	D	A	D	S	V	V	G	T	S
A	E	A	S	F	A	D	V	D	K	I	L	F	D	A	D	S	V	V	G	T	S
A	E	A	S	F	A	D	V	D	K	I	L	F	D	A	D	S	V	V	G	T	S
A	E	A	S	F	A	D	V	D	K	I	L	F	D	A	D	S	V	V	G	T	S
A	E	A	S	F	A	D	V	D	K	I	L	F	D	A	D	S	V	V	G	T	S
A	E	A	S	F	A	D	V	D	K	I	L	F	D	A	D	S	V	V	G	T	S
A	E	A	S	F	A	D	V	D	K	I	L	F	D	A	D	S	V	V	G	T	S
A	E	A	S	F	A	D	V	D	K	I	L	F	D	A	D	S	V	V	G	T	S
A	E	A	S	F	A	D	V	D	K	I	L	F	D	A	D	S	V	V	G	T	S
A	E	A	S	F	A	D	V	D	K	I	L	F	D	A	D	S	V	V	G	T	S
A	E	A	S	F	A	D	V	D	K	I	L	F	D	A	D	S	V	V	G	T	S
A	E	A	S	F	A	D	V	D	K	I	L	F	D	A	D	S	V	V	G	T	S
A	E	A	S	F	A	D	V	D	K	I	L	F	D	A	D	S	V	V	G	T	S
A	E	A	S	F	A	D	V	D	K	I	L	F	D	A	D	S	V	V	G	T	S
A	E	A	S	F	A	D	V	D	K	I	L	F	D	A	D	S	V	V	G	T	S
A	E	A	S	F	A	D	V	D	K	I	L	F	D	A	D	S	V	V	G	T	S

A	E	A	S	F	A	D	V	D	K	I	L	F	D	A	D	S	V	V	G	T	S
A	E	A	S	F	A	D	V	D	K	I	L	F	D	A	D	S	V	V	G	T	S
A	E	A	S	F	A	D	V	D	K	I	L	F	D	A	D	S	V	V	G	T	S
A	E	A	S	F	A	D	V	D	K	I	L	F	D	A	D	S	V	V	G	T	S
A	E	A	S	F	A	D	V	D	K	I	L	F	D	A	D	S	V	V	G	T	S
A	E	A	S	F	A	D	V	D	K	I	L	F	D	A	D	S	V	V	G	T	S
A	E	A	S	F	A	D	V	D	K	I	L	F	D	A	D	S	V	V	G	T	S
A	E	A	S	F	A	D	V	D	K	I	L	F	D	A	D	S	V	V	G	T	S
A	E	A	S	F	A	D	V	D	K	I	L	F	D	A	D	S	V	V	G	T	S
A	E	A	S	F	A	D	V	D	K	I	L	F	D	A	D	S	V	V	G	T	S
A	E	A	S	F	A	D	V	D	K	I	L	F	D	A	D	S	V	V	G	T	S
A	E	A	S	F	A	D	V	D	K	I	L	F	D	A	D	S	V	A	G	T	S
A	E	A	S	F	A	D	V	D	K	I	L	F	D	A	D	S	V	A	G	T	S
A	E	A	S	F	A	D	V	D	K	I	L	F	D	A	D	S	V	A	G	T	S
A	E	A	S	F	A	D	V	D	K	I	L	F	D	A	D	S	V	A	G	T	S
A	E	A	S	F	A	D	V	D	K	I	L	F	D	A	D	S	V	A	G	T	S
A	E	A	S	F	A	D	V	D	K	I	L	F	D	A	D	S	V	A	G	T	S
A	E	A	S	F	A	D	V	D	K	I	L	F	D	A	D	S	V	A	G	T	S
A	E	A	S	F	A	D	V	D	K	I	L	F	D	A	D	S	V	A	G	T	S
A	E	A	S	F	A	D	V	D	K	I	L	F	D	A	D	S	V	A	G	T	S
A	G	A	T	V	T	E	V	E	Q	V	V	L	D	V	E	L	A	A	D	P	T
A	G	A	T	V	T	E	V	E	Q	V	V	L	D	V	E	L	A	A	D	P	T
A	G	A	T	V	T	E	V	E	Q	V	V	L	D	V	E	L	A	A	D	P	T
A	G	A	T	V	T	E	V	E	Q	V	V	L	D	V	E	L	A	A	D	P	T
A	G	A	T	V	T	E	V	E	Q	V	V	L	D	V	E	L	A	A	D	P	T
A	G	A	T	V	T	E	V	E	Q	V	V	L	D	V	E	L	A	A	D	P	T
A	G	A	T	V	T	E	V	E	Q	V	V	L	D	V	E	L	A	A	D	P	T
A	G	A	T	V	T	E	V	E	Q	V	V	L	D	V	E	L	A	A	D	P	T
A	E	A	S	F	A	D	I	D	K	I	L	F	D	A	D	S	V	A	G	I	S
A	E	A	S	F	A	D	V	D	K	I	L	F	D	A	D	S	V	A	G	T	S
A	E	A	S	F	A	D	V	D	K	I	L	F	D	A	D	S	V	A	G	T	S
A	E	A	S	F	A	D	V	D	K	I	L	F	D	A	D	S	V	A	G	T	S
A	E	A	S	F	A	D	V	D	K	I	L	F	D	A	D	S	V	A	G	T	S
A	E	A	S	F	A	D	V	D	K	I	L	F	D	A	D	S	V	A	G	T	S
A	E	A	S	F	A	D	V	D	K	I	L	F	D	A	D	S	V	A	G	T	S
A	E	A	S	F	A	D	V	D	K	I	L	F	D	A	D	S	V	A	G	T	S
A	E	A	S	F	A	D	V	D	K	I	L	F	D	A	D	S	V	A	G	T	S
A	E	A	S	F	A	D	V	D	K	I	L	F	D	A	D	S	V	A	G	T	S
A	E	A	S	F	A	D	V	D	K	I	L	F	D	A	D	S	V	A	G	T	S
V	E	A	S	F	A	D	V	D	K	I	L	F	D	A	D	S	V	A	G	T	S
A	E	A	S	F	A	D	V	D	K	I	L	F	D	A	D	S	V	A	G	T	S
A	E	A	S	F	A	D	V	D	K	I	L	F	D	A	D	S	V	A	G	T	S
A	E	A	S	F	A	D	V	D	K	I	L	F	D	A	D	S	V	A	G	T	S
A	E	A	S	F	A	D	V	D	K	I	L	F	D	A	D	S	V	A	G	T	S
A	E	A	S	F	A	D	V	D	K	I	L	F	D	A	D	S	V	A	G	T	S
A	E	A	S	F	A	D	V	D	K	I	L	F	D	A	D	S	V	A	G	T	S
A	E	A	S	F	A	D	V	D	K	I	L	F	D	A	D	S	V	A	G	T	S
A	E	A	S	F	A	D	V	D	K	I	L	F	D	A	D	S	V	A	G	T	S
A	E	A	S	F	A	D	I	D	K	I	L	F	D	A	D	S	V	A	G	I	S
A	E	A	S	F	A	D	I	D	K	I	L	F	D	A	D	S	V	A	G	I	S
A	E	A	S	F	A	D	I	D	K	I	L	F	D	A	D	S	V	A	G	I	S
A	E	A	S	F	A	D	I	D	K	I	L	F	D	A	D	S	V	A	G	I	S
A	E	A	S	F	A	D	I	D	K	I	L	F	D	A	D	S	V	A	G	I	S
A	E	A	S	F	A	D	I	D	K	I	L	F	D	A	D	S	V	A	G	I	S
A	E	A	S	F	A	D	I	D	K	I	L	F	D	A	D	S	V	A	G	I	S
A	E	A	S	F	A	D	I	D	K	I	L	F	D	A	D	S	V	A	G	I	S
A	E	A	S	F	A	D	I	D	K	I	L	F	D	A	D	S	V	A	G	I	S
A	E	A	S	F	A	D	I	D	K	I	L	F	D	A	D	S	V	A	G	I	S
A	E	A	S	F	A	D	I	D	K	I	L	F	D	A	D	S	V	A	G	I	S
A	E	A	S	F	A	D	I	D	K	I	L	F	D	A	D	S	V	A	G	I	S
A	E	A	S	F	A	D	I	D	K	I	L	F	D	A	D	S	V	A	G	I	S
A	E	A	S	F	A	D	I	D	K	I	L	F	D	A	D	S	V	A	G	I	S
A	E	A	S	F	A	D	I	D	K	I	L	F	D	A	D	S	V	A	G	I	S
A	E	A	S	F	A	D	I	D	K	I	L	F	D	A	D	S	V	A	G	I	S
A	E	A	S	F	A	D	I	D	K	I	L	F	D	A	D	S	V	A	G	I	S
A	E	A	S	F	A	D	I	D	K	I	L	F	D	A	D	S	V	A	G	I	S
A	E	A	S	F	A	D	I	D	K	I	L	F	D	A	D	S	V	A	G	I	S

A	E	A	S	F	A	D	I	D	K	I	L	F	D	A	D	S	V	A	G	I	S
A	E	A	S	F	A	D	I	D	K	I	L	F	D	A	D	S	V	A	G	I	S
A	E	A	S	F	A	D	I	D	K	I	L	F	D	A	D	S	V	A	G	I	S
A	E	A	S	F	A	D	I	D	K	I	L	F	D	A	D	S	V	A	G	I	S
A	E	A	S	F	A	D	I	D	K	I	L	F	D	A	D	S	V	A	G	I	S
A	E	A	S	F	A	D	I	D	K	I	L	F	D	A	D	S	V	A	G	I	S
A	E	A	S	F	A	D	I	D	K	I	L	F	D	A	D	S	V	A	G	I	S
A	E	A	S	F	A	D	I	D	K	I	L	F	D	A	D	S	V	A	G	I	S
A	E	A	S	F	A	D	I	D	K	I	L	F	D	A	D	S	V	A	G	I	S
A	E	A	S	F	A	D	I	D	K	I	L	F	D	A	D	S	V	A	G	I	S
A	E	A	S	F	A	D	I	D	K	I	L	F	D	A	D	S	V	A	G	I	S
A	E	A	S	F	A	D	I	D	K	I	L	F	D	A	D	S	V	A	G	I	S
A	E	A	S	F	A	D	I	D	K	I	L	F	D	A	D	S	V	A	G	I	S
A	E	A	S	F	A	D	I	D	K	I	L	F	D	A	D	S	V	A	G	I	S
A	E	A	S	F	A	D	I	D	K	I	L	F	D	A	D	S	V	A	G	I	S
A	E	A	S	F	A	D	I	D	K	I	L	F	D	A	D	S	V	A	G	I	S
A	E	A	S	F	A	D	I	D	K	I	L	F	D	A	D	S	V	A	G	I	S
A	E	A	S	F	A	D	I	D	K	I	L	F	D	A	D	S	V	A	G	I	S
A	E	A	S	L	A	D	I	D	K	I	L	F	D	A	D	S	V	A	G	I	S
A	E	A	S	F	A	D	I	D	K	I	L	F	D	A	D	S	V	A	G	I	S
A	E	A	S	F	A	D	I	D	K	I	L	F	D	A	D	S	V	A	G	I	S
A	E	A	S	F	A	D	I	D	K	I	L	F	D	A	D	S	V	A	G	I	S
A	E	A	S	F	A	D	I	D	K	I	L	F	D	A	D	S	V	A	G	I	S
A	E	A	S	F	A	D	I	D	K	I	L	F	D	A	D	S	V	A	G	I	S
A	E	A	S	F	A	D	V	D	K	I	L	F	D	A	D	S	V	A	G	T	S
A	E	A	S	F	A	D	V	D	K	I	L	F	D	A	D	S	V	A	G	T	S
A	E	A	S	F	A	D	V	D	K	I	L	F	D	A	D	S	V	A	G	T	S
A	E	A	S	F	A	D	V	D	K	I	L	F	D	A	D	S	V	A	G	T	S
A	E	A	S	F	A	D	V	D	K	I	L	F	D	A	D	S	V	A	G	T	S
A	E	A	S	F	A	D	V	D	K	I	L	F	D	A	D	S	V	A	G	T	S
A	E	A	S	F	A	D	V	D	K	I	L	F	D	A	D	S	V	A	G	T	S
A	E	A	S	F	A	D	V	D	K	I	L	F	D	A	D	S	V	A	G	T	S
A	E	A	S	F	A	D	V	D	K	I	L	F	D	A	D	S	V	A	G	T	S
A	E	A	S	F	A	D	V	D	K	I	L	F	D	A	D	S	V	A	G	T	S
A	E	A	S	F	A	D	V	D	K	I	L	F	D	A	D	S	V	A	G	T	S
A	E	A	S	F	A	D	V	D	K	I	L	F	D	A	D	S	V	A	G	T	S
A	E	A	S	F	A	D	V	D	K	I	L	F	D	A	D	S	V	A	G	T	S
A	E	A	S	F	A	D	V	D	K	I	L	F	D	A	D	S	V	A	G	T	S
A	E	A	S	F	A	D	V	D	K	I	L	F	D	A	D	S	V	A	G	T	S
A	E	A	S	F	A	D	V	D	K	I	L	F	D	A	D	S	V	A	G	T	S
A	E	A	S	F	A	D	V	D	K	I	L	F	D	A	D	S	V	A	G	T	S
A	E	A	S	F	A	D	V	D	K	I	L	F	D	A	D	S	V	A	G	T	S
A	E	A	S	F	A	D	V	D	K	I	L	F	D	A	D	S	V	A	G	T	S
A	E	A	S	F	A	D	V	D	K	I	L	F	D	A	D	S	V	A	G	T	S
A	E	A	S	F	A	D	V	D	K	I	L	F	D	A	D	S	V	A	G	T	S
A	E	A	S	F	A	D	V	D	K	I	L	F	D	A	D	S	V	A	G	T	S
A	E	A	S	F	A	D	V	D	K	I	L	F	D	A	D	S	V	A	G	T	S
A	E	A	S	F	A	D	V	D	K	I	L	F	D	A	D	S	V	A	G	T	S
A	E	A	S	F	A	D	V	D	K	I	L	F	D	A	D	S	V	A	G	T	S
A	E	A	S	F	A	D	I	D	K	I	L	F	D	A	D	S	V	A	G	I	S
A	E	A	S	F	A	D	I	D	K	I	L	F	D	A	D	S	V	A	G	I	S
A	E	A	S	F	A	D	I	D	K	I	L	F	D	A	D	S	V	A	G	I	S
A	E	A	S	F	A	D	I	D	K	I	L	F	D	A	D	S	V	A	G	I	S
A	E	A	S	F	A	D	I	D	K	I	L	F	D	A	D	S	V	A	G	I	S
A	E	A	S	F	A	D	I	D	K	I	L	F	D	A	D	S	V	A	G	I	S
A	E	A	S	F	A	D	I	D	K	I	L	F	D	A	D	S	V	A	G	I	S
A	E	A	S	F	A	D	I	D	K	I	L	F	D	A	D	S	V	A	G	I	S
A	E	A	S	F	A	D	I	D	K	I	L	F	D	A	D	S	V	A	G	I	S
A	E	A	S	F	A	D	I	D	K	I	L	F	D	A	D	S	V	A	G	I	S
A	E	A	S	F	A	D	I	D	K	I	L	F	D	A	D	S	V	A	G	I	S
A	E	A	S	F	A	D	I	D	K	I	L	F	D	A	D	S	V	A	G	I	S
A	E	A	S	F	A	D	I	D	K	I	L	F	D	A	D	S	V	A	G	I	S
A	E	A	S	F	A	D	I	D	K	I	L	F	D	A	D	S	V	A	G	I	S
A	E	A	S	F	A	D	I	D	K	I	L	F	D	A	D	S	V	A	G	I	S
A	E	A	S	F	A	D	I	D	K	I	L	F	D	A	D	S	V	A	G	I	S
A	E	A	S	F	A	D	I	D	K	I	L	F	D	A	D	S	V	A	G	I	S
A	E	A	S	F	A	D	I	D	K	I	L	F	D	A	D	S	V	A	G	I	S

A	E	A	S	F	A	D	I	D	K	I	L	F	D
A	E	A	S	F	A	D	I	D	K	I	L	F	D
A	E	A	S	F	A	D	I	D	K	I	L	F	D
A	E	A	S	F	A	D	I	D	K	I	L	F	D
A	E	A	S	F	A	D	I	D	K	I	L	F	D
A	E	A	S	F	A	D	I	D	K	I	L	F	D
A	E	A	S	F	A	D	I	D	K	I	L	F	D
A	E	A	S	F	A	D	I	D	K	I	L	F	D
A	E	A	S	F	A	D	I	D	K	I	L	F	D
A	E	A	S	F	A	D	I	D	K	I	L	F	D

A	D	S	V	A	G	I	S
A	D	S	V	A	G	I	S
A	D	S	V	A	G	I	S
A	D	S	V	A	G	I	S
A	D	S	V	A	G	I	S
A	D	S	V	A	G	I	S
A	D	S	V	A	G	I	S
A	D	S	V	A	G	I	S
A	D	S	V	A	G	I	S
A	D	S	V	A	G	I	S

wze																						
58	72	74	80	85	87	88	133	142	150	167	175	177	183	190	193	194	196	197	202	212	214	223
W	G	I	L	A	D	K	K	L	I	R	S	G	S	K	V	G	H	R	G	S	Q	R
R	A	M	S	S	E	R	R	V	V	Q	S	E	A	E	D	R	N	W	R	S	R	-
W	G	I	L	A	D	K	K	L	I	R	M	-	S	K	V	G	H	R	G	N	Q	K
R	A	M	S	S	E	R	R	V	V	Q	S	E	A	E	D	R	N	W	R	S	R	-
R	A	M	S	S	E	R	R	V	V	Q	S	E	A	E	D	R	N	W	R	S	R	-
R	A	M	S	S	E	R	R	V	V	Q	S	E	A	E	D	R	N	W	R	S	R	-
R	A	M	S	S	E	R	R	V	V	Q	S	E	A	E	D	R	N	W	R	S	R	-
R	A	M	S	S	E	R	R	V	V	Q	S	E	A	E	D	R	N	W	R	S	R	-
R	A	M	S	S	E	R	R	V	V	Q	S	E	A	E	D	R	N	W	R	S	R	-
R	A	M	S	S	E	R	R	V	V	Q	S	E	A	E	D	R	N	W	R	S	R	-
R	A	M	S	S	E	R	R	V	V	Q	S	E	A	E	D	R	N	W	R	S	R	-

wze																						
50	58	72	74	77	80	85	87	88	133	136	142	150	167	168	177	181	182	193	195	200	209	214
S	W	G	I	S	L	A	D	K	K	S	L	I	R	K	E	I	N	L	H	F	D	K
T	R	A	M	S	S	S	E	R	R	S	V	V	Q	K	K	T	K	I	Q	C	D	E
S	W	G	I	S	L	A	D	K	K	S	L	I	R	N	E	I	N	L	H	F	N	K
S	W	G	I	S	L	A	D	K	K	S	L	I	R	N	E	I	N	L	H	F	N	K
S	W	G	I	S	L	A	D	K	K	S	L	I	R	N	E	I	N	L	H	F	N	K
S	W	G	I	S	L	A	D	K	K	S	L	I	R	N	E	I	N	L	H	F	N	K
S	W	G	I	F	L	A	D	K	K	S	L	I	R	N	E	I	N	L	H	F	N	K
S	W	G	I	S	L	A	D	K	K	S	L	I	R	N	E	I	N	L	H	F	N	K
S	W	G	I	S	L	A	D	K	K	S	L	I	R	N	E	I	N	L	H	F	N	K
S	W	G	I	S	L	A	D	K	K	S	L	I	R	N	E	I	N	L	H	F	N	K
S	W	G	I	S	L	A	D	K	K	S	L	I	R	N	E	I	N	L	H	F	N	K
S	W	G	I	S	L	A	D	K	K	S	L	I	R	N	E	I	N	L	H	F	N	K
S	W	G	I	S	L	A	D	K	K	S	L	I	R	N	E	I	N	L	H	F	N	K
S	W	G	I	S	L	A	D	K	K	S	L	I	R	N	E	I	N	L	H	F	N	K
S	W	G	I	S	L	A	D	K	K	S	L	I	R	N	E	I	N	L	H	F	N	K
S	W	G	I	S	L	A	D	K	K	S	L	I	R	N	E	I	N	L	H	F	N	K
S	W	G	I	S	L	A	D	K	K	S	L	I	R	N	E	I	N	L	H	F	N	K
S	W	G	I	S	L	A	D	K	K	S	L	I	R	N	E	I	N	L	H	F	N	K
S	W	G	I	S	L	A	D	K	K	S	L	I	R	N	E	I	N	L	H	F	N	K
S	W	G	I	S	L	A	D	K	K	S	L	I	R	N	E	I	N	L	H	F	N	K
S	W	G	I	S	L	A	D	K	K	S	L	I	R	N	E	I	N	L	H	F	N	K
S	W	G	I	S	L	A	D	K	K	S	L	I	R	K	E	I	N	L	H	F	D	K
S	W	G	I	S	L	A	D	K	K	S	L	I	R	K	K	I	N	L	H	F	D	K
S	W	G	I	S	L	A	D	K	K	S	L	I	R	K	E	I	N	L	H	F	D	K
S	W	G	I	S	L	A	D	K	K	S	L	I	R	K	E	I	N	L	H	F	D	K
S	W	G	I	S	L	A	D	K	K	T	L	I	R	K	E	I	N	L	H	F	D	K
S	W	G	I	S	L	A	D	K	K	S	L	I	R	K	E	I	N	L	H	F	D	K
S	W	G	I	S	L	A	D	K	K	S	L	I	R	K	E	I	N	L	H	F	D	K
S	W	G	I	S	L	A	D	K	K	S	L	I	R	K	E	I	N	L	H	F	D	K
S	W	G	I	S	L	A	D	K	K	S	L	I	R	K	E	I	N	L	H	F	D	K
S	W	G	I	S	L	A	D	K	K	S	L	I	R	K	K	I	N	L	H	F	D	K

wze																						
72	74	80	85	87	88	111	127	136	140	150	156	158	166	167	175	177	183	186	190	192	196	197
G	I	L	A	D	K	I	T	S	E	I	V	V	T	R	S	G	S	Y	K	T	H	R
G	I	L	A	D	K	I	T	S	E	I	V	V	T	R	M	-	S	Y	K	T	H	R
A	M	S	S	E	R	V	I	S	G	V	I	I	M	Q	S	E	T	T	E	T	N	W
G	I	L	A	D	K	I	T	T	E	I	V	V	T	R	S	G	S	Y	K	A	H	R

wze

59	72	74	80	85	87	88	133	142	150	167	177	181	182	193	195	200	209	214	217	220	227
A	G	I	L	A	D	K	K	L	I	R	E	I	N	L	H	F	D	K	S	N	N
A	A	M	S	S	E	R	R	V	V	Q	K	T	K	I	Q	C	D	E	S	S	K
A	G	I	L	A	D	K	K	L	I	R	E	I	N	L	H	F	N	K	F	N	N
A	G	I	L	A	D	K	K	L	I	R	E	I	N	L	H	F	N	K	F	N	N
A	G	I	L	A	D	K	K	L	I	R	E	I	N	L	H	F	N	K	F	N	N
A	G	I	L	A	D	K	K	L	I	R	E	I	N	L	H	F	N	K	F	N	N
A	G	I	L	A	D	K	K	L	I	R	E	I	N	L	H	F	N	K	F	N	N
A	G	I	L	A	D	K	K	L	I	R	E	I	N	L	H	F	N	K	F	N	N
A	G	I	L	A	D	K	K	L	I	R	E	I	N	L	H	F	N	K	F	N	N
A	G	I	L	A	D	K	K	L	I	R	E	I	N	L	H	F	N	K	F	N	N
A	G	I	L	A	D	K	K	L	I	R	E	I	N	L	H	F	N	K	F	N	N
A	G	I	L	A	D	K	K	L	I	R	E	I	N	L	H	F	N	K	F	N	N
A	G	I	L	A	D	K	K	L	I	R	E	I	N	L	H	F	N	K	F	N	N
A	G	I	L	A	D	K	K	L	I	R	E	I	N	L	H	F	N	K	F	N	N
A	G	I	L	A	D	K	K	L	I	R	E	I	N	L	H	F	N	K	F	N	N
A	G	I	L	A	D	K	K	L	I	R	E	I	N	L	H	F	N	K	F	N	N
A	G	I	L	A	D	K	K	L	I	R	E	I	N	L	H	F	N	K	F	N	N
A	G	I	L	A	D	K	K	L	I	R	E	I	N	L	H	F	N	K	F	N	N
A	G	I	L	A	D	K	K	L	I	R	E	I	N	L	H	F	N	K	F	N	N
A	G	I	L	A	D	K	K	L	I	R	E	I	N	L	H	F	N	K	F	N	N
A	G	I	L	A	D	K	K	L	I	R	E	I	N	L	H	F	N	K	F	N	N
A	G	I	L	A	D	K	K	L	I	R	E	I	N	L	H	F	N	K	F	N	N
A	G	I	L	A	D	K	K	L	I	R	E	I	N	L	H	F	N	K	F	N	N
A	G	I	L	A	D	K	K	L	I	R	E	I	N	L	H	F	N	K	F	N	N
T	G	I	L	A	D	K	K	L	I	R	E	I	N	L	H	F	N	K	F	N	N
A	G	I	L	A	D	K	K	L	I	R	E	I	N	L	H	F	N	K	F	N	N
T	G	I	L	A	D	K	K	L	I	R	E	I	N	L	H	F	N	K	F	N	N

212	217	220	223
V	S	N	D
I	S	N	N
V	A	S	N
V	S	N	D
V	S	N	D
V	S	N	D
V	S	N	D
V	S	N	D
V	S	N	D
V	S	N	D
V	S	N	D
V	S	N	D
V	S	N	D
V	S	N	D
V	S	N	D
V	S	N	D
V	S	N	D
V	S	N	D
V	S	N	D
V	S	N	D
V	S	N	D
V	S	N	D
V	S	N	D
V	S	N	D
V	S	N	D
V	S	N	D
V	S	N	D
V	S	N	D
V	S	N	D

2	3	5	6	8	9	11	12	13	16	17	22	24	27	31	32	33	37
N	G	I	V	S	S	A	I	I	F	L	T	L	A	T	E	I	T
N	G	I	V	S	S	V	I	I	S	L	T	L	T	A	K	I	T
D	E	G	L	I	F	A	V	L	I	I	I	F	F	T	E	L	S
N	G	I	V	S	S	A	I	I	F	L	T	L	A	A	E	I	T
N	G	I	V	S	S	A	I	I	F	L	T	L	A	A	E	I	T
N	G	I	V	S	S	A	I	I	F	L	T	L	A	A	E	I	T
N	G	I	V	S	S	A	I	I	F	L	T	L	A	A	E	I	T
N	G	I	V	S	S	A	I	I	F	L	T	L	A	A	E	I	T
N	G	I	V	S	S	A	I	I	F	L	T	L	A	A	E	I	T
N	G	I	V	S	S	A	I	I	F	L	T	L	A	A	E	I	T
N	G	I	V	S	S	A	I	I	F	L	T	L	A	A	E	I	T
N	G	I	V	S	S	A	I	I	F	L	T	L	A	A	E	I	T
N	G	I	V	S	S	A	I	I	F	L	T	L	A	A	E	I	T
N	G	I	V	S	S	A	I	I	F	L	T	L	A	A	E	I	T
N	G	I	V	S	S	A	I	I	F	L	T	L	A	A	E	I	T
N	G	I	V	S	S	A	I	I	F	L	T	L	A	A	E	I	T
N	G	I	V	S	S	A	I	I	F	L	T	L	A	A	E	I	T
N	G	I	V	S	S	A	I	I	F	L	T	L	A	A	E	I	T
N	G	I	V	S	S	A	I	I	F	L	T	L	A	A	E	I	T
N	G	I	V	S	S	A	I	I	F	L	T	L	A	A	E	I	T
N	G	I	V	S	S	A	I	I	F	L	T	L	A	A	E	I	T
N	G	I	V	S	S	A	I	I	F	L	T	L	A	A	E	I	T
N	G	I	V	S	S	A	I	I	F	L	T	L	A	A	E	I	T
N	G	I	V	S	S	A	I	I	F	L	T	L	A	A	E	I	T
N	G	I	V	S	S	A	I	I	F	L	T	L	A	A	E	I	T
N	G	I	V	S	S	A	I	I	F	L	T	L	A	A	E	I	T
N	G	I	V	S	S	A	I	I	F	L	T	L	A	A	E	I	T
N	G	I	V	S	S	A	I	I	F	L	T	L	A	A	E	I	T
N	G	I	V	S	S	A	I	I	F	L	T	L	A	A	E	I	T

V	S	N	D
V	S	N	D

N	G	I	V	S	S	A	I	I	F	L	T	L	A	A	E	I	T
N	G	I	V	S	S	A	I	I	F	L	T	L	A	A	E	I	T

	rmlC							rmlB						rmlD					
286	12	52	66	149	155	167	182	132	209	245	275	300	338	32	33	38	39	69	87
V	A	E	F	E	A	E	N	L	I	Q	V	E	A	A	E	N	E	E	K
I	A	E	F	E	A	E	N	L	I	Q	A	D	A	A	K	N	E	E	K
I	A	E	F	E	A	E	N	L	I	Q	A	D	A	A	K	N	E	E	K
I	A	E	F	E	A	E	N	L	I	Q	A	D	A	A	K	N	E	E	K
I	A	E	F	E	A	E	N	L	I	Q	A	D	A	A	K	N	E	E	K
I	A	E	F	E	A	E	N	L	I	Q	A	D	A	A	K	N	E	E	K
I	A	E	F	E	A	E	N	L	I	Q	A	D	A	A	K	N	E	E	K
I	A	E	F	E	A	E	N	L	I	Q	A	D	A	A	K	N	E	E	K
I	A	E	F	E	A	E	N	L	I	Q	A	D	A	A	K	N	E	E	K
I	A	E	F	E	A	E	N	L	I	Q	A	D	A	A	K	N	E	E	K
I	A	E	F	E	A	E	N	L	I	Q	A	D	A	A	K	N	E	E	K
I	A	E	F	E	A	E	N	L	I	Q	A	D	A	A	K	N	E	E	K
I	A	E	F	E	A	E	N	L	I	Q	A	D	A	A	K	N	E	E	K
I	A	E	F	E	A	E	N	L	I	Q	A	D	A	A	K	N	E	E	K
I	A	E	F	E	A	E	N	L	I	Q	A	D	A	A	K	N	E	E	K
I	A	E	F	E	A	E	N	L	I	Q	A	D	A	A	K	N	E	E	K
I	A	E	F	E	A	E	N	L	I	Q	A	D	A	A	K	N	E	E	K
I	A	E	F	E	A	E	N	L	I	Q	A	D	A	A	K	N	E	E	K
I	A	E	F	E	A	E	N	L	I	Q	A	D	A	A	K	N	E	E	K
I	A	E	F	E	A	E	N	L	I	Q	A	D	A	A	K	N	E	E	K
I	A	E	F	E	A	E	N	L	I	Q	A	D	A	A	K	N	E	E	K
I	A	E	F	E	A	E	N	L	I	Q	A	D	A	A	K	N	E	E	K
I	A	E	F	E	A	E	N	L	I	Q	A	D	A	A	K	N	E	E	K
I	A	E	F	E	A	E	N	L	I	Q	A	D	A	A	K	N	E	E	K
I	A	E	F	E	A	E	N	L	I	Q	A	D	A	A	K	N	E	E	K
I	A	E	F	E	A	E	N	L	I	Q	A	D	A	A	K	N	E	E	K
I	A	E	F	E	A	E	N	L	I	Q	A	D	A	A	K	N	E	E	K
I	A	E	F	E	A	E	N	L	I	Q	A	D	A	A	K	N	E	E	K
I	A	E	F	E	A	E	N	L	N	Q	A	D	A	A	K	N	E	E	K
I	A	E	F	E	A	E	N	L	I	Q	A	D	A	A	K	N	E	E	K
I	A	A	F	E	A	E	H	F	I	K	A	D	A	T	K	N	E	E	K
I	A	E	F	E	S	E	N	L	I	Q	A	D	A	A	K	N	E	E	K
I	A	E	F	E	S	E	N	L	I	Q	A	D	A	A	K	N	E	E	K
I	A	E	F	E	S	E	N	L	I	Q	A	D	A	A	K	N	E	E	K
I	A	E	F	E	S	E	N	L	I	Q	A	D	A	A	K	N	E	E	K
I	A	E	F	E	S	E	N	L	I	Q	A	D	A	A	K	N	E	E	K
I	A	E	F	E	S	E	N	L	I	Q	A	D	A	A	K	N	E	E	K
I	A	E	F	E	S	E	N	L	I	Q	A	D	A	A	K	N	E	E	K
I	A	E	F	E	S	E	N	L	I	Q	A	D	A	A	K	N	E	E	K
I	A	E	F	E	S	E	N	L	I	Q	A	D	A	A	K	N	E	E	K
I	A	E	F	E	S	E	N	L	I	Q	A	D	A	A	K	N	E	E	K
I	A	E	F	E	S	E	N	L	I	Q	A	D	A	A	K	N	E	E	K
I	A	E	F	E	S	E	N	L	I	Q	A	D	A	A	K	N	E	E	K
I	A	E	F	E	S	E	N	L	I	Q	A	D	A	A	K	N	E	E	K
I	A	E	F	E	S	E	N	L	I	Q	A	D	A	A	K	N	E	E	K
I	A	E	F	E	S	E	N	L	I	Q	A	D	A	A	K	N	E	E	K
I	A	E	F	E	S	E	N	L	I	Q	A	D	A	A	K	N	E	E	K
I	A	E	F	E	S	E	N	L	I	Q	A	D	A	A	K	N	E	E	K
I	A	E	F	E	S	E	N	L	I	Q	A	D	A	A	K	N	E	E	K
I	A	E	F	E	S	E	N	L	I	Q	A	D	A	A	K	N	E	E	K
I	A	E	F	E	S	E	N	L	I	Q	A	D	A	A	K	N	E	E	K
I	A	E	F	E	S	E	N	L	I	Q	A	D	A	A	K	N	E	E	K
I	A	E	F	E	S	E	N	L	I	Q	A	D	A	A	K	N	E	E	K
I	A	E	F	E	S	E	N	L	I	Q	A	D	A	A	K	N	E	E	K
I	A	E	F	E	S	E	N	L	I	Q	A	D	A	A	K	N	E	E	K

I	A	E	F	E	S	E	N	L	I	Q	A	D	A	A	K	N	E	E	K
I	A	E	F	E	S	E	N	L	I	Q	A	D	A	A	K	N	E	E	K
I	A	E	F	E	S	E	N	L	I	Q	A	D	A	A	K	N	E	E	K
I	A	E	F	E	S	E	N	L	I	Q	A	D	A	A	K	N	E	E	K
I	A	E	F	E	S	E	N	L	I	Q	A	D	A	A	K	N	E	E	K
I	A	E	F	E	S	E	N	L	I	Q	A	D	A	A	K	N	E	E	K
I	A	E	F	E	S	E	N	L	I	Q	A	D	A	A	K	N	E	E	K
I	A	E	F	E	S	E	N	L	I	Q	A	D	A	A	K	N	E	E	K
I	A	E	F	E	S	E	N	L	I	Q	A	D	A	A	K	N	E	E	K
I	A	E	F	E	S	E	N	L	I	Q	A	D	A	A	K	N	E	E	K
I	A	E	F	E	S	E	N	L	I	Q	A	D	A	A	K	N	E	E	K
I	A	E	F	E	S	E	N	L	I	Q	A	D	A	A	K	N	E	E	K
I	A	E	F	E	S	E	N	L	I	Q	A	D	A	A	K	N	E	E	K
I	A	E	F	E	S	E	N	L	I	Q	A	D	A	A	K	N	E	E	K
I	A	E	F	E	S	E	N	L	I	Q	A	D	A	A	K	N	E	E	K
I	A	E	F	E	S	E	N	L	I	Q	A	D	A	A	K	N	E	E	K
I	A	E	F	E	S	E	N	L	I	Q	A	D	A	A	K	N	E	E	K
I	A	E	F	E	S	E	N	L	I	Q	A	D	A	A	K	N	E	E	K
I	A	E	F	E	S	E	N	L	I	Q	A	D	A	A	K	N	E	E	K
I	A	E	F	E	S	E	N	L	I	Q	A	D	A	A	K	N	E	E	K
I	A	E	F	E	S	E	N	L	I	Q	A	D	A	A	K	N	E	E	K
I	A	E	F	E	S	E	N	L	I	Q	A	D	A	A	K	N	E	E	K
I	A	E	F	E	S	E	N	L	I	Q	A	D	A	A	K	N	E	E	K
I	A	E	F	E	S	E	N	L	I	Q	A	D	A	A	K	N	E	E	K
I	A	E	F	E	S	E	N	L	I	Q	A	D	A	A	K	N	E	E	K
I	A	E	F	E	S	E	N	L	I	Q	A	D	A	A	K	N	E	E	K
I	A	E	F	E	S	E	N	L	I	Q	A	D	A	A	K	N	E	E	K
I	A	E	F	E	S	E	N	L	I	Q	A	D	A	A	K	N	E	E	K
I	A	E	F	E	S	E	N	L	I	Q	A	D	A	A	K	N	E	E	K
I	A	E	F	E	S	E	N	L	I	Q	A	D	A	A	K	N	E	E	K
I	A	E	F	E	S	E	N	L	I	Q	A	D	A	A	K	N	E	E	K
I	A	E	F	E	S	E	N	L	I	Q	A	D	A	A	K	N	E	E	K
I	A	E	F	E	S	E	N	L	I	Q	A	D	A	A	K	N	E	E	K
I	A	E	F	E	S	E	N	L	I	Q	A	D	A	A	K	N	E	E	K
I	A	E	F	E	S	E	N	L	I	Q	A	D	A	A	K	N	E	E	K
I	A	E	F	E	S	E	N	L	I	Q	A	D	A	A	K	N	E	E	K
I	A	E	F	E	S	E	N	L	I	Q	A	D	A	A	K	N	E	E	K
I	A	E	F	E	S	E	N	L	I	Q	A	D	A	A	K	N	E	E	K
I	A	E	F	E	S	E	N	L	I	Q	A	D	A	A	K	N	E	E	K
I	A	E	F	E	S	E	N	L	I	Q	A	D	A	A	K	N	E	E	K
I	A	E	F	E	S	E	N	L	I	Q	A	D	A	A	K	N	E	E	K
I	A	E	F	E	S	E	N	L	I	Q	A	D	A	A	K	N	E	E	K
I	A	E	F	E	S	E	N	L	I	Q	A	D	A	A	K	N	E	E	K
I	A	E	F	E	S	E	N	L	I	Q	A	D	A	A	K	N	E	E	K
I	A	E	F	E	S	E	N	L	I	Q	A	D	A	A	K	N	E	E	K
I	A	E	F	E	S	E	N	L	I	Q	A	D	A	A	K	N	E	E	K
I	A	E	F	E	S	E	N	L	I	Q	A	D	A	A	K	N	E	E	K
I	A	E	F	E	S	E	N	L	I	Q	A	D	A	A	K	N	E	E	K
I	A	E	F	E	S	E	N	L	I	Q	A	D	A	A	K	N	E	E	K
I	A	E	F	E	S	E	N	L	I	Q	A	D	A	A	K	N	E	E	K
I	A	E	F	E	A	E	N	L	I	Q	A	D	S	A	K	N	E	E	K
I	A	E	F	E	A	E	N	L	I	Q	A	D	S	A	K	N	E	E	K
I	A	E	F	E	A	E	N	L	I	Q	A	D	S	A	K	N	E	E	K
I	A	E	F	E	A	E	N	L	I	Q	A	D	S	A	K	N	E	E	K
I	A	E	F	E	A	E	N	L	I	Q	A	D	S	A	K	N	E	E	K
I	A	E	F	E	A	E	N	L	I	Q	A	D	S	A	K	N	E	E	K
I	A	E	F	E	A	E	N	L	I	Q	A	D	S	A	K	N	E	E	K
I	A	E	F	E	A	E	N	L	I	Q	A	D	S	A	K	N	E	E	K
I	A	E	F	E	A	E	N	L	I	Q	A	D	S	A	K	N	E	E	K
I	A	E	F	E	A	E	N	L	I	Q	A	D	S	A	K	N	E	E	K
I	A	E	F	E	A	E	N	L	I	Q	A	D	S	A	K	N	E	E	K
I	A	E	F	E	A	E	N	L	I	Q	A	D	S	A	K	N	E	E	K
I	A	E	F	E	A	E	N	L	I	Q	A	D	S	A	K	N	E	E	K
I	A	E	F	E	A	E	N	L	I	Q	A	D	S	A	K	N	E	E	K
I	A	E	F	E	A	E	N	L	I	Q	A	D	S	A	K	N	E	E	K
I	A	E	F	E	A	E	N	L	I	Q	A	D	S	A	K	N	E	E	K
I	A	E	F	E	A	E	N	L	I	Q	A	D	S	A	K	N	E	E	K

I	A	E	F	E	A	E	N	L	I	Q	A	D	S	A	K	N	E	E	K
I	A	E	F	E	A	E	N	L	I	Q	A	D	S	A	K	N	E	E	K
I	A	E	F	E	A	E	N	L	I	Q	A	D	S	A	K	N	E	E	K
I	A	E	F	E	A	E	N	L	I	Q	A	D	S	A	K	N	E	E	K
I	A	E	F	E	A	E	N	L	I	Q	A	D	S	A	K	N	E	E	K
I	A	E	F	E	A	E	N	L	I	Q	A	D	S	A	K	N	E	E	K
I	A	E	F	E	A	E	N	L	I	Q	A	D	S	A	K	N	E	E	K
I	A	E	F	E	A	E	N	L	I	Q	A	D	S	A	K	N	E	E	K
I	A	E	F	E	A	E	N	L	I	Q	A	D	S	A	K	N	E	E	K
I	A	E	L	E	A	E	H	L	I	Q	A	D	A	A	E	N	E	E	R
I	A	E	L	E	A	E	H	L	I	Q	A	D	A	A	E	N	E	E	R
I	A	E	L	E	A	E	N	L	I	Q	V	E	A	A	E	N	E	E	R
I	A	E	F	E	A	E	N	L	I	Q	V	E	A	A	E	N	E	E	R
I	A	E	F	E	A	E	N	L	I	Q	V	E	A	A	E	N	E	E	R
I	A	E	F	E	A	E	N	L	I	Q	V	E	A	A	E	N	E	E	R
I	A	E	F	E	A	E	N	L	I	Q	V	E	A	A	E	N	E	E	R
I	A	E	F	E	A	E	N	L	I	Q	V	E	A	A	E	N	E	E	R
I	A	E	F	E	A	E	N	L	I	Q	V	E	A	A	E	N	E	E	R
I	A	E	F	E	A	E	N	L	I	Q	V	E	A	A	E	N	E	E	R
I	A	E	F	E	A	E	N	L	I	Q	V	E	A	A	E	N	E	E	R
I	A	E	F	E	A	E	N	L	I	Q	V	E	A	A	E	N	E	E	R
I	A	A	F	E	A	E	N	L	I	Q	V	D	A	A	E	N	E	E	K
I	A	A	F	E	A	E	N	L	I	Q	V	D	A	A	E	N	E	E	K
I	A	A	F	E	A	E	N	L	I	Q	V	D	A	A	E	N	E	E	K
I	A	A	F	E	A	E	N	L	I	Q	V	D	A	A	E	N	E	E	K
I	A	A	F	E	A	E	N	L	I	Q	V	D	A	A	E	N	E	E	K
I	A	A	F	E	A	E	N	L	I	Q	V	D	A	A	E	N	E	E	K
I	A	A	F	E	A	E	N	L	I	Q	V	D	A	A	E	N	E	E	K
I	A	A	F	E	A	E	N	L	I	Q	V	D	A	A	E	N	E	E	K
I	A	A	F	E	A	E	N	L	I	Q	V	D	A	A	E	N	E	E	K
I	A	A	F	E	A	E	N	L	I	Q	V	D	A	A	E	N	E	E	K
I	A	A	F	E	A	E	N	L	I	Q	V	D	A	A	E	N	E	E	K
I	A	A	F	E	A	E	N	L	I	Q	V	D	A	A	E	N	E	E	K
I	A	A	F	E	A	E	N	L	I	Q	V	D	A	A	E	N	E	E	K
I	A	A	F	E	A	E	N	L	I	Q	V	D	A	A	E	N	E	E	K
I	A	A	F	E	A	E	N	L	I	Q	V	D	A	A	E	N	E	E	K
I	A	A	F	E	A	E	N	L	I	Q	V	D	A	A	E	N	E	E	K
I	A	A	F	E	A	E	N	L	I	Q	V	D	A	A	E	N	E	E	K
I	A	A	F	E	A	E	N	L	I	Q	V	D	A	A	E	N	E	E	K
I	A	A	F	E	A	E	N	L	I	Q	V	D	A	A	E	N	E	E	K
I	A	A	F	E	A	E	N	L	I	Q	V	D	A	A	E	N	E	E	K
I	A	A	F	E	A	E	N	L	I	Q	V	D	A	A	E	N	E	E	K
I	A	A	F	E	A	E	N	L	I	Q	V	D	A	A	E	N	E	E	K
I	A	A	F	E	A	E	N	L	I	Q	V	D	A	A	E	N	E	E	K
I	A	A	F	E	A	E	N	L	I	Q	V	D	A	A	E	N	E	E	K
I	A	A	F	E	A	E	N	L	I	Q	V	D	A	A	E	N	E	E	K
I	A	A	F	E	A	E	N	L	I	Q	V	D	A	A	E	N	E	E	K
I	A	A	F	E	A	E	N	L	I	Q	V	D	A	A	E	N	E	E	K
I	A	A	F	E	A	E	N	L	I	Q	V	D	A	A	E	N	E	E	K
I	A	E	F	E	A	K	H	L	I	Q	V	D	A	A	E	D	A	E	K
V	A	E	F	E	A	E	N	L	I	Q	V	E	A	A	E	N	E	E	K
V	A	E	F	E	A	E	N	L	I	Q	V	E	A	A	E	N	E	E	K
V	V	E	F	E	A	E	N	L	I	Q	V	E	A	A	E	N	E	E	K
V	A	E	F	E	A	E	N	L	I	Q	V	E	A	A	E	N	E	E	K
V	V	E	F	E	A	E	N	L	I	Q	V	E	A	A	E	N	E	E	K
V	A	E	F	E	A	E	N	L	I	Q	V	E	A	A	E	N	E	E	K
V	A	E	F	E	A	E	N	L	I	Q	V	E	A	A	E	N	E	E	K
V	V	E	F	E	A	E	N	L	I	Q	V	E	A	A	E	N	E	E	K
V	A	E	F	E	A	E	N	L	I	Q	V	E	A	A	E	N	E	E	K
V	A	E	F	E	A	E	N	L	I	Q	V	E	A	A	E	N	E	E	K
V	A	E	F	E	A	E	N	L	I	Q	V	E	A	A	E	N	E	E	K
V	A	E	F	E	A	E	N	L	I	Q	V	E	A	A	E	N	E	E	K
V	A	E	F	E	A	E	N	L	I	Q	V	E	A	A	E	N	E	E	K
V	A	E	F	E	A	E	N	L	I	Q	V	E	A	A	E	N	E	E	K
V	A	E	F	E	A	E	N	L	I	Q	V	E	A	A	E	N	E	E	K
V	A	E	F	E	A	E	N	L	I	Q	V	E	A	A	E	N	E	E	K
V	V	E	F	E	A	E	N	L	I	Q	V	E	A	A	E	N	E	E	K

V	A	E	F	E	A	E	N	L	I	Q	V	E	A	A	E	N	E	E	K
V	A	E	F	E	A	E	N	L	I	Q	V	E	A	A	E	N	E	E	K
V	A	E	F	E	A	E	N	L	I	Q	V	E	A	A	E	N	E	E	K
V	A	E	F	E	A	E	N	L	I	Q	V	E	A	A	E	N	E	E	K
V	A	E	F	E	A	E	N	L	I	Q	V	E	A	A	E	N	E	E	K
V	A	E	F	E	A	E	N	L	I	Q	V	E	A	A	E	N	E	E	K
V	A	E	F	E	A	E	N	L	I	Q	V	E	A	A	E	N	E	E	K
V	A	E	F	E	A	E	N	L	I	Q	V	E	A	A	E	N	E	E	K
V	A	E	F	E	A	E	N	L	I	Q	V	E	A	A	E	N	E	E	K
V	A	E	F	E	A	E	N	L	I	Q	V	E	A	A	E	N	E	E	K
V	A	E	F	E	A	E	N	L	I	Q	V	E	A	A	E	N	E	E	K
V	A	E	F	E	A	E	N	L	I	Q	V	E	A	A	E	N	E	E	K
V	A	E	F	E	A	E	N	L	I	Q	V	E	A	A	E	N	E	E	K
V	A	E	F	E	A	E	N	L	I	Q	V	E	A	A	E	N	E	E	K
V	A	E	F	E	A	E	N	L	I	Q	V	E	A	A	E	N	E	E	K
V	A	E	F	E	A	E	N	L	I	Q	V	E	A	A	E	N	E	E	K
V	A	E	F	E	A	E	N	L	I	Q	V	E	A	A	E	N	E	E	K
V	A	E	F	E	A	E	N	L	I	Q	V	E	A	A	E	N	E	E	K
V	A	E	F	E	A	E	N	L	I	Q	V	E	A	A	E	N	E	E	K
V	A	E	F	E	A	E	N	L	I	Q	V	E	A	A	E	N	E	E	K
V	A	E	F	E	A	E	N	L	I	Q	V	E	A	A	E	N	E	E	K
V	A	E	F	E	A	E	N	L	I	Q	V	E	A	A	E	N	E	E	K
V	A	E	F	E	A	E	N	L	I	Q	V	E	A	A	E	N	E	E	K
V	A	E	F	E	A	E	N	L	I	Q	V	E	A	A	E	N	E	E	K
V	A	E	F	E	A	E	N	L	I	Q	V	E	A	A	E	N	E	E	K
V	A	E	F	E	A	E	N	L	I	Q	V	E	A	A	E	N	E	E	K
V	A	E	F	E	A	E	N	L	I	Q	V	E	A	A	E	N	E	E	K
V	A	E	F	E	A	E	N	L	I	Q	V	E	A	A	E	N	E	E	K
V	A	E	F	E	A	E	N	L	I	Q	V	E	A	A	E	N	E	E	K
V	V	E	F	E	A	E	N	L	I	Q	V	E	A	A	E	N	E	E	K
V	V	E	F	E	A	E	N	L	I	Q	V	E	A	A	E	N	E	E	K
V	A	E	F	E	A	E	N	L	I	Q	V	E	A	A	E	N	E	E	K
V	V	E	F	E	A	E	N	L	I	Q	V	E	A	A	E	N	E	E	K
V	A	E	F	E	A	E	N	L	I	Q	V	E	A	A	E	N	E	E	K
V	V	E	F	E	A	E	N	L	I	Q	V	E	A	A	E	N	E	E	K
V	A	E	F	E	A	E	N	L	I	Q	V	E	A	A	E	N	E	E	K
V	V	E	F	E	A	E	N	L	I	Q	V	E	A	A	E	N	E	E	K
V	A	E	F	E	A	E	N	L	I	Q	V	E	A	A	E	N	E	E	K
V	A	E	F	E	A	E	N	L	I	Q	V	E	A	A	E	N	E	E	K
V	A	E	F	E	A	E	N	L	I	Q	V	E	A	A	E	N	E	E	K
V	A	E	F	E	A	E	N	L	I	Q	V	E	A	A	E	N	E	E	K
V	A	E	F	E	A	E	N	L	I	Q	V	E	A	A	E	N	E	E	K
V	V	E	F	E	A	E	N	L	I	Q	V	E	A	A	E	N	E	E	K
V	A	E	F	E	A	E	N	L	I	Q	V	E	A	A	E	N	E	E	K
V	A	E	F	E	A	E	N	L	I	Q	V	E	A	A	E	N	E	E	K
V	A	E	F	E	A	E	N	L	I	Q	V	E	A	A	E	N	E	E	K
V	A	E	F	E	A	E	N	L	I	Q	V	E	A	A	E	N	E	E	K
V	A	E	F	E	A	E	N	L	I	Q	V	E	A	A	E	N	E	E	K
V	A	E	F	E	A	E	N	L	I	Q	V	E	A	A	E	N	E	E	K
V	A	E	F	E	A	E	N	L	I	Q	V	E	A	A	E	N	E	E	K
V	A	E	F	E	A	E	N	L	I	Q	V	E	A	A	E	N	E	E	K
V	A	E	F	E	A	E	N	L	I	Q	V	E	A	A	E	N	E	E	K
V	A	E	F	E	A	E	N	L	I	Q	V	E	A	A	E	N	E	E	K
V	A	E	F	K	A	E	N	L	I	Q	V	E	A	A	E	N	E	E	K
V	A	E	F	E	A	E	N	L	I	Q	V	E	A	A	E	N	E	E	K
V	A	E	F	E	A	E	N	L	I	Q	V	E	A	A	E	N	E	E	K
V	A	E	F	E	A	E	N	L	I	Q	V	E	A	A	E	N	E	E	K
V	A	E	F	E	A	E	N	L	I	Q	V	E	A	A	E	N	E	E	K
V	A	E	F	E	A	E	N	L	I	Q	V	E	A	A	E	N	E	E	K
V	A	E	F	E	A	E	N	L	I	Q	V	E	A	A	E	N	E	E	K
V	A	E	F	E	A	E	N	L	I	Q	V	E	A	A	E	N	E	E	K
V	A	E	F	E	A	E	N	L	I	Q	V	E	A	A	E	N	E	E	K
V	A	E	F	E	A	E	N	L	I	Q	V	E	A	A	E	N	E	E	K
V	A	E	F	E	A	E	N	L	I	Q	V	E	A	A	E	N	E	E	K
V	A	E	F	E	A	E	N	L	I	Q	V	E	A	A	E	N	E	E	K
V	A	E	F	E	A	E	N	L	I	Q	V	E	A	A	E	N	E	E	K
V	A	E	F	E	A	E	N	L	I	Q	V	E	A	A	E	N	E	E	K

V	A	E	F	E	A	E	N	L	I	Q	V	E	A	A	E	N	E	E	K
V	A	E	F	E	A	E	N	L	I	Q	V	E	A	A	E	N	E	E	K
V	A	E	F	E	A	E	N	L	I	Q	V	E	A	A	E	N	E	E	K
V	A	E	F	E	A	E	N	L	I	Q	V	E	A	A	E	N	E	E	K
V	A	E	F	E	A	E	N	L	I	Q	V	E	A	A	E	N	E	E	K
V	A	E	F	E	A	E	N	L	I	Q	V	E	A	A	E	N	E	E	K
V	A	E	F	E	A	E	N	L	I	Q	V	E	A	A	E	N	E	E	K
V	A	E	F	E	A	E	N	L	I	Q	V	E	A	A	E	N	E	E	K
V	A	E	F	E	A	E	N	L	I	Q	V	E	A	A	E	N	E	E	K
V	A	E	F	E	A	E	N	L	I	Q	V	E	A	A	E	N	E	E	K
V	A	E	F	E	A	E	N	L	I	Q	V	E	A	A	E	N	E	E	K
V	A	E	F	E	A	E	N	L	I	Q	V	E	A	A	E	N	E	E	K
V	A	E	F	E	A	E	N	L	I	Q	V	E	A	A	E	N	E	E	K
V	A	E	F	E	A	E	N	L	I	Q	V	E	A	A	E	N	E	E	K
V	A	E	F	E	A	E	N	L	I	Q	V	E	A	A	E	N	E	E	K
V	A	E	F	E	A	E	N	L	I	Q	V	E	A	A	E	N	E	E	K
V	A	E	F	E	A	E	N	L	I	Q	V	E	A	A	E	N	E	E	K
V	A	E	F	E	A	E	N	L	I	Q	V	E	A	A	E	N	E	E	K
V	A	E	F	E	A	E	N	L	I	Q	V	E	A	A	E	N	E	E	K
V	A	E	F	E	A	E	N	L	I	Q	V	E	A	A	E	N	E	E	K
V	A	E	F	E	A	E	N	L	I	Q	V	E	A	A	E	N	E	E	K
V	A	E	F	E	A	E	N	L	I	Q	V	E	A	A	E	N	E	E	K
V	A	E	F	E	A	E	N	L	I	Q	V	E	A	A	E	N	E	E	K
V	A	E	F	E	A	E	N	L	I	Q	V	E	A	A	E	N	E	E	K
V	A	E	F	E	A	E	N	L	I	Q	V	E	A	A	E	N	E	E	K
V	A	E	F	E	A	E	N	L	I	Q	V	E	A	A	E	N	E	E	K
V	A	E	F	E	A	E	N	L	I	Q	V	E	A	A	E	N	E	K	K

wze																						
52	58	59	72	74	77	80	85	87	88	124	133	136	142	150	153	158	163	167	168	177	181	182
T	W	A	G	I	S	L	A	D	K	P	K	S	L	I	T	V	A	R	K	E	I	N
T	W	A	G	I	S	L	A	D	K	P	K	S	L	I	T	V	A	R	K	E	I	N
T	W	A	G	I	S	L	A	D	K	P	K	S	L	I	T	V	A	R	K	E	I	N
T	W	A	G	I	S	L	A	D	K	P	K	S	L	I	T	V	A	R	K	E	I	N
T	W	A	G	I	S	L	A	D	K	P	K	S	L	I	T	V	A	R	K	E	I	N
T	W	A	G	I	S	L	A	D	K	P	K	S	L	I	T	V	A	R	K	E	I	N
T	W	A	G	I	S	L	A	D	K	P	K	S	L	I	T	V	A	R	K	E	I	N
T	W	A	G	I	S	L	A	D	K	P	K	S	L	I	T	V	A	R	K	E	I	N
T	W	A	G	I	S	L	A	D	K	P	K	S	L	I	T	V	A	R	K	E	I	N
T	W	A	G	I	S	L	A	D	K	P	K	S	L	I	T	V	A	R	K	E	I	N
T	W	A	G	I	S	L	A	D	K	P	K	S	L	I	T	V	A	R	K	E	I	N
T	W	A	G	I	S	L	A	D	K	P	K	S	L	I	T	V	A	R	K	E	I	N
T	W	A	G	I	S	L	A	D	K	P	K	S	L	I	T	V	A	R	K	E	I	N
T	W	A	G	I	S	L	A	D	K	P	K	S	L	I	T	V	A	R	K	E	I	N
T	W	A	G	I	S	L	A	D	K	P	K	S	L	I	T	V	A	R	K	E	I	N
T	W	A	G	I	S	L	A	D	K	P	K	S	L	I	T	V	A	R	K	E	I	N
T	W	A	G	I	S	L	A	D	K	P	K	S	L	I	T	V	A	R	K	E	I	N
T	W	A	G	I	S	L	A	D	K	P	K	S	L	I	T	V	A	R	K	E	I	N
T	W	A	G	I	S	L	A	D	K	P	K	S	L	I	T	V	A	R	K	E	I	N
T	W	A	G	I	S	L	A	D	K	P	K	S	L	I	T	V	A	R	K	E	I	N
T	W	A	G	I	S	L	A	D	K	P	K	S	L	I	T	V	A	R	K	E	I	N
T	W	A	G	I	S	L	A	D	K	P	K	S	L	I	T	V	A	R	K	E	I	N
T	W	A	G	I	S	L	A	D	K	P	K	S	L	I	T	V	A	R	K	E	I	N
T	W	A	G	I	S	L	A	D	K	P	K	S	L	I	T	V	A	R	K	E	I	N
T	W	A	G	I	S	L	A	D	K	P	K	S	L	I	T	V	A	R	K	E	I	N
T	W	A	G	I	S	L	A	D	K	P	K	S	L	I	T	V	A	R	K	E	I	N
T	W	A	G	I	S	L	A	D	K	P	K	S	L	I	T	V	A	R	K	E	I	N
T	W	A	G	I	S	L	A	D	K	P	K	S	L	I	T	V	A	R	K	E	I	N
T	W	A	G	I	S	L	A	D	K	P	K	S	L	I	T	V	A	R	K	E	I	N
T	W	A	G	I	S	L	A	D	K	P	K	S	L	I	T	V	A	R	K	E	I	N
T	W	A	G	I	S	L	A	D	K	P	K	S	L	I	T	V	A	R	N	E	I	N
T	W	A	G	I	S	L	A	D	K	P	K	S	L	I	T	V	A	R	N	E	I	N
T	W	A	G	I	S	L	A	D	K	P	K	S	L	I	T	V	A	R	N	E	I	N
T	W	A	G	I	S	L	A	D	K	P	K	S	L	I	T	V	A	R	N	E	I	N
T	W	A	G	I	S	L	A	D	K	P	K	S	L	I	T	V	A	R	N	E	I	N
T	W	A	G	I	S	L	A	D	K	P	K	S	L	I	T	V	A	R	N	E	I	N
T	W	A	G	I	S	L	A	D	K	P	K	S	L	I	T	V	A	R	N	E	I	N
T	W	A	G	I	S	L	A	D	K	P	K	S	L	I	T	V	A	R	N	E	I	N
T	W	A	G	I	S	L	A	D	K	P	K	S	L	I	T	V	A	R	N	E	I	N
T	W	A	G	I	S	L	A	D	K	P	K	S	L	I	T	V	A	R	N	E	I	N
T	W	A	G	I	S	L	A	D	K	P	K	S	L	I	T	V	A	R	N	E	I	N
T	W	A	G	I	S	L	A	D	K	P	K	S	L	I	T	V	A	R	N	E	I	N
T	W	A	G	I	S	L	A	D	K	P	K	S	L	I	T	V	A	R	N	E	I	N
T	W	A	G	I	S	L	A	D	K	P	K	T	L	I	T	V	A	R	N	E	I	N
T	W	A	G	I	S	L	A	D	K	P	K	T	L	I	T	V	A	R	N	E	I	N
T	W	A	G	I	S	L	A	D	K	P	K	T	L	I	T	V	A	R	N	E	I	N
T	W	A	G	I	S	L	A	D	K	P	K	T	L	I	T	V	A	R	N	E	I	N
T	W	A	G	I	S	L	A	D	K	P	K	T	L	I	T	V	A	R	N	E	I	N
T	W	A	G	I	S	L	A	D	K	P	K	T	L	I	T	V	A	R	N	E	I	N
T	W	A	G	I	S	L	A	D	K	P	K	T	L	I	T	V	A	R	N	E	I	N
T	W	A	G	I	S	L	A	D	K	P	K	T	L	I	T	V	A	R	N	E	I	N
T	W	A	G	I	S	L	A	D	K	P	K	T	L	I	T	V	A	R	N	E	I	N
T	W	A	G	I	S	L	A	D	K	P	K	T	L	I	T	V	A	R	N	E	I	N
T	W	A	G	I	S	L	A	D	K	P	K	S	L	I	T	V	A	R	K	E	I	N
T	W	T	G	I	S	L	A	D	K	P	K	T	L	I	T	V	A	R	N	E	I	N
T	W	A	G	I	S	L	A	D	K	P	K	T	L	I	T	V	A	R	N	E	I	N
T	W	A	G	I	S	L	A	D	K	P	K	T	L	I	T	V	A	R	N	E	I	N
T	W	A	G	I	S	L	A	D	K	P	K	T	L	I	T	V	A	R	N	E	I	N
T	W	A	G	I	S	L	A	D	K	P	K	T	L	I	T	V	A	R	N	E	I	N
T	W	A	G	I	S	L	A	D	K	P	K	S	L	I	T	V	A	R	K	E	I	N
T	W	A	G	I	S	L	A	D	K	P	K	S	L	I	T	V	A	R	K	E	I	N
T	W	A	G	I	S	L	A	D	K	P	K	S	L	I	T	V	A	R	K	E	I	N
T	W	A	G	I	S	L	A	D	K	P	K	S	L	I	T	V	A	R	K	E	I	N

T	W	A	G	I	S	L	A	D	K	P	K	S	L	I	T	V	A	R	K	E	I	N
T	W	A	G	I	S	L	A	D	K	P	K	S	L	I	T	V	A	R	K	E	I	N
T	W	A	G	I	S	L	A	D	K	P	K	S	L	I	T	V	A	R	K	E	I	N
T	W	A	G	I	S	L	A	D	K	P	K	S	L	I	T	V	A	R	K	E	I	N
T	W	A	G	I	S	L	A	D	K	P	K	S	L	I	T	V	A	R	K	E	I	N
T	W	A	G	I	S	L	A	D	K	P	K	S	L	I	T	V	A	R	K	E	I	N
T	W	A	G	I	S	L	A	D	K	P	K	S	L	I	T	V	A	R	K	E	I	N
T	W	A	G	I	S	L	A	D	K	P	K	S	L	I	T	V	A	R	K	E	I	N
T	W	A	G	I	S	L	A	D	K	P	K	S	L	I	T	V	A	R	K	E	I	N
T	W	A	G	I	S	L	A	D	K	P	K	S	L	I	T	V	A	R	K	E	I	N
T	W	A	G	I	S	L	A	D	K	P	K	S	L	I	T	V	A	R	K	E	I	N
T	W	A	G	I	S	L	A	D	K	P	K	S	L	I	T	V	A	R	K	E	I	N
T	W	A	G	I	S	L	A	D	K	P	K	S	L	I	T	V	A	R	K	E	I	N
T	W	A	G	I	S	L	A	D	K	P	K	S	L	I	T	V	A	R	K	E	I	N
T	W	A	G	I	S	L	A	D	K	P	K	S	L	I	T	V	A	R	K	E	I	N
T	W	A	G	I	S	L	A	D	K	P	K	S	L	I	T	V	A	R	K	E	I	N
T	W	A	G	I	S	L	A	D	K	P	K	S	L	I	T	V	A	R	K	E	I	N
T	W	A	G	I	S	L	A	D	K	P	K	S	L	I	T	V	A	R	K	E	I	N
T	W	A	G	I	S	L	A	D	K	P	K	S	L	I	T	V	A	R	K	E	I	N
T	W	A	G	I	S	L	A	D	K	P	K	S	L	I	T	V	A	R	K	E	I	N
T	W	A	G	I	S	L	A	D	K	P	K	S	L	I	T	V	A	R	K	E	I	N
T	W	A	G	I	S	L	A	D	K	P	K	T	L	I	T	V	A	R	N	E	I	N
T	W	A	G	I	S	L	A	D	K	P	K	T	L	I	T	V	A	R	N	E	I	N
T	W	A	G	I	S	L	A	D	K	P	K	S	L	I	T	V	A	R	N	E	I	N
T	W	A	G	I	S	L	A	D	K	L	K	T	L	I	T	V	A	R	K	E	I	N
T	W	V	G	I	S	L	A	D	K	P	K	T	L	I	T	V	A	R	K	E	I	N
T	W	A	G	I	S	L	A	D	K	P	K	T	L	I	T	V	A	R	K	E	I	N
T	W	A	G	I	S	L	A	D	K	P	K	S	L	I	T	V	V	R	K	E	I	N
N	W	A	G	I	S	L	A	D	K	P	K	S	L	I	T	V	A	R	K	E	I	N
T	W	A	G	I	S	L	A	D	K	P	K	S	L	I	T	V	V	R	K	E	I	N
N	W	A	G	I	S	L	A	D	K	P	K	S	L	I	T	V	A	R	K	E	I	N
T	W	A	G	I	S	L	A	D	K	P	K	S	L	I	T	V	V	R	K	E	I	N
T	W	A	G	I	S	L	A	D	K	P	K	S	L	I	T	V	V	R	K	E	I	N
N	W	A	G	I	S	L	A	D	K	P	K	S	L	I	T	V	A	R	K	E	I	N
T	W	A	G	I	S	L	A	D	K	P	K	S	L	I	T	V	V	R	K	E	I	N
N	W	A	G	I	S	L	A	D	K	P	K	S	L	I	T	V	A	R	K	E	I	N
N	W	A	G	I	S	L	A	D	K	P	K	S	L	I	T	V	A	R	K	E	I	N
N	W	A	G	I	S	L	A	D	K	P	K	S	L	I	T	V	A	R	K	E	I	N
N	W	A	G	I	S	L	A	D	K	P	K	S	L	I	T	V	A	R	K	E	I	N
T	W	A	G	I	S	L	A	D	K	P	K	S	L	I	T	V	V	R	K	E	I	N
T	W	A	G	I	S	L	A	D	K	P	K	S	L	I	T	V	V	R	K	E	I	N
N	W	A	G	I	S	L	A	D	K	P	K	S	L	I	T	V	A	R	K	E	I	N
N	W	A	G	I	S	L	A	D	K	P	K	S	L	I	T	V	A	R	K	E	I	N
T	W	A	G	I	S	L	A	D	K	P	K	S	L	I	T	V	V	R	K	E	I	N
N	W	A	G	I	S	L	A	D	K	P	K	S	L	I	T	V	A	R	K	E	I	N
N	W	A	G	I	S	L	A	D	K	P	K	S	L	I	T	V	A	R	K	E	I	N
N	W	A	G	I	S	L	A	D	K	P	K	S	L	I	T	V	A	R	K	E	I	N
N	W	A	G	I	S	L	A	D	K	P	K	S	L	I	T	V	A	R	K	E	I	N
N	W	A	G	I	S	L	A	D	K	P	K	S	L	I	T	V	A	R	K	E	I	N
N	W	A	G	I	S	L	A	D	K	P	K	S	L	I	T	V	A	R	K	E	I	N
N	W	A	G	I	S	L	A	D	K	P	K	S	L	I	T	V	A	R	K	E	I	N
N	W	A	G	I	S	L	A	D	K	P	K	S	L	I	T	V	A	R	K	E	I	N
N	W	A	G	I	S	L	A	D	K	P	K	S	L	I	T	V	A	R	K	E	I	N
N	W	A	G	I	S	L	A	D	K	P	K	S	L	I	T	V	A	R	K	E	I	N
N	W	A	G	I	S	L	A	D	K	P	K	S	L	I	T	V	A	R	K	E	I	N
N	W	A	G	I	S	L	A	D	K	P	K	S	L	I	T	V	A	R	K	E	I	N
N	W	A	G	I	S	L	A	D	K	P	K	S	L	I	T	V	A	R	K	E	I	N
N	W	A	G	I	S	L	A	D	K	P	K	S	L	I	T	V	A	R	K	E	I	N
N	W	A	G	I	S	L	A	D	K	P	K	S	L	I	T	V	A	R	K	E	I	N
N	W	A	G	I	S	L	A	D	K	P	K	S	L	I	T	V	A	R	K	E	I	N
N	W	A	G	I	S	L	A	D	K	P	K	S	L	I	T	V	A	R	K	E	I	N
N	W	A	G	I	S	L	A	D	K	P	K	S	L	I	T	V	A	R	K	E	I	N
N	W	A	G	I	S	L	A	D	K	P	K	S	L	I	T	V	A	R	K	E	I	N
N	W	A	G	I	S	L	A	D	K	P	K	S	L	I	T	V	A	R	K	E	I	N
N	W	A	G	I	S	L	A	D	K	P	K	S	L	I	T	V	A	R	K	E	I	N
N	W	A	G	I	S	L	A	D	K	P	K	S	L	I	T	V	A	R	K	E	I	N
N	W	A	G	I	S	L	A	D	K	P	K	S	L	I	T	V	A	R	K	E	I	N

N	W	A	G	I	S	L	A	D	K	P	K	S	L	I	T	V	A	R	K	E	I	N
N	W	A	G	I	S	L	A	D	K	P	K	S	L	I	T	V	A	R	K	E	I	N
N	W	A	G	I	S	L	A	D	K	P	K	S	L	I	T	V	A	R	K	E	I	N
N	W	A	G	I	S	L	A	D	K	P	K	S	L	I	T	V	A	R	K	E	I	N
N	W	A	G	I	S	L	A	D	K	P	K	S	L	I	T	V	A	R	K	E	I	N
N	W	A	G	I	S	L	A	D	K	P	K	S	L	I	T	V	A	R	K	E	I	N
T	W	A	G	I	S	L	A	D	K	P	K	S	L	I	T	V	V	R	K	E	I	N
T	W	A	G	I	S	L	A	D	K	P	K	S	L	I	T	V	V	R	K	E	I	N
N	W	A	G	I	S	L	A	D	K	P	K	S	L	I	T	V	A	R	K	E	I	N
T	W	A	G	I	S	L	A	D	K	P	K	S	L	I	T	V	V	R	K	E	I	N
N	W	A	G	I	S	L	A	D	K	P	K	S	L	I	T	V	A	R	K	E	I	N
T	W	A	G	I	S	L	A	D	K	P	K	S	L	I	T	V	V	R	K	E	I	N
N	W	A	G	I	S	L	A	D	K	P	K	S	L	I	T	V	A	R	K	E	I	N
T	W	A	G	I	S	L	A	D	K	P	K	S	L	I	T	V	V	R	K	E	I	N
T	W	A	G	I	S	L	A	D	K	P	K	S	L	I	T	V	V	R	K	E	I	N
N	W	A	G	I	S	L	A	D	K	P	K	S	L	I	T	V	A	R	K	E	I	N
N	W	A	G	I	S	L	A	D	K	P	K	S	L	I	T	V	A	R	K	E	I	N
N	W	A	G	I	S	L	A	D	K	P	K	S	L	I	T	V	A	R	K	E	I	N
N	W	A	G	I	S	L	A	D	K	P	K	S	L	I	T	V	A	R	K	E	I	N
T	W	A	G	I	S	L	A	D	K	P	K	S	L	I	T	V	V	R	K	E	I	N
N	W	A	G	I	S	L	A	D	K	P	K	S	L	I	T	V	A	R	K	E	I	N
T	W	A	G	I	S	L	A	D	K	P	K	S	L	I	T	V	A	R	K	E	I	N
N	W	A	G	I	S	L	A	D	K	P	K	S	L	I	T	V	A	R	K	E	I	N
N	W	A	G	I	S	L	A	D	K	P	K	S	L	I	T	V	A	R	K	E	I	N
N	W	A	G	I	S	L	A	D	K	P	K	S	L	I	T	V	A	R	K	E	I	N
N	W	A	G	I	S	L	A	D	K	P	K	S	L	I	T	V	A	R	K	E	I	N
T	W	A	G	I	S	L	A	D	K	P	K	S	L	I	T	V	A	R	K	E	I	N
N	W	A	G	I	S	L	A	D	K	P	K	S	L	I	T	V	A	R	K	E	I	N
N	W	A	G	I	S	L	A	D	K	P	K	S	L	I	T	V	A	R	K	E	I	N
N	W	A	G	I	S	L	A	D	K	P	K	S	L	I	T	V	A	R	K	E	I	N
N	W	A	G	I	S	L	A	D	K	P	K	S	L	I	T	V	A	R	K	E	I	N
N	W	A	G	I	S	L	A	D	K	P	K	S	L	I	T	V	A	R	K	E	I	N
N	W	A	G	I	S	L	A	D	K	P	K	S	L	I	T	V	A	R	K	E	I	N
N	W	A	G	I	S	L	A	D	K	P	K	S	L	I	T	V	A	R	K	E	I	N
N	W	A	G	I	S	L	A	D	K	P	K	S	L	I	T	V	A	R	K	E	I	N
N	W	A	G	I	S	L	A	D	K	P	K	S	L	I	T	V	A	R	K	E	I	N
N	W	A	G	I	S	L	A	D	K	P	K	S	L	I	T	V	A	R	K	E	I	N
N	W	A	G	I	S	L	A	D	K	P	K	S	L	I	T	V	A	R	K	E	I	N
N	W	A	G	I	S	L	A	D	K	P	K	S	L	I	T	V	A	R	K	E	I	N
N	W	A	G	I	S	L	A	D	K	P	K	S	L	I	T	V	A	R	K	E	I	N
N	W	A	G	I	S	L	A	D	K	P	K	S	L	I	T	V	A	R	K	E	I	N
T	W	A	G	I	S	L	A	D	K	P	K	S	L	I	T	V	V	R	K	E	I	N
T	W	A	G	I	S	L	A	D	K	P	K	S	L	I	T	V	A	R	K	E	I	N
T	W	A	G	I	S	L	A	D	K	P	K	S	L	I	T	V	V	R	K	E	I	N
T	W	A	G	I	S	L	A	D	K	P	K	S	L	I	T	V	V	R	K	E	I	N
T	W	A	G	I	S	L	A	D	K	P	K	S	L	I	T	V	A	R	K	E	I	N
T	W	A	G	I	S	L	A	D	K	P	K	S	L	I	T	V	V	R	K	E	I	N
T	W	A	G	I	S	L	A	D	K	P	K	S	L	I	T	V	V	R	K	E	I	N
T	W	A	G	I	S	L	A	D	K	P	K	S	L	I	T	V	V	R	K	E	I	N
T	W	A	G	I	S	L	A	D	K	P	K	S	L	I	T	V	A	R	K	E	I	N
T	W	A	G	I	S	L	A	D	K	P	K	S	L	I	T	V	A	R	K	E	I	N
T	W	A	G	I	S	L	A	D	K	P	K	S	L	I	T	V	A	R	K	E	I	N
T	W	A	G	I	S	L	A	D	K	P	K	S	L	I	T	V	V	R	K	E	I	N
T	W	A	G	I	S	L	A	D	K	P	K	S	L	I	T	V	A	R	K	E	I	N
T	W	A	G	I	S	L	A	D	K	P	K	S	L	I	T	V	V	R	K	E	I	N
T	W	A	G	I	S	L	A	D	K	P	K	S	L	I	T	V	A	R	K	E	I	N
T	W	A	G	I	S	L	A	D	K	P	K	S	L	I	T	V	V	R	K	E	I	N
T	W	A	G	I	S	L	A	D	K	P	K	S	L	I	T	V	V	R	K	E	I	N
N	W	A	G	I	S	L	A	D	K	P	K	S	L	I	T	V	A	R	K	E	I	N
T	W	A	G	I	S	L	A	D	K	P	K	S	L	I	T	V	A	R	K	E	I	N
T	W	A	G	I	S	L	A	D	K	P	K	S	L	I	T	V	A	R	K	E	I	N
T	W	A	G	I	S	L	A	D	K	P	K	S	L	I	T	V	V	R	K	E	I	N
T	W	A	G	I	S	L	A	D	K	P	K	S	L	I	T	I	V	R	K	E	I	N
T	W	A	G	I	S	L	A	D	K	P	K	S	L	I	T	V	V	R	K	E	I	N
T	W	A	G	I	S	L	A	D	K	P	K	S	L	I	T	V	A	R	K	E	I	N
T	W	A	G	I	S	L	A	D	K	P	K	S	L	I	T	V	V	R	K	E	I	N
T	W	A	G	I	S	L	A	D	K	P	K	S	L	I	T	V	V	R	K	E	I	N

T	W	A	G	I	S	L	A	D	K	P	K	S	L	I	T	V	V	R	K	E	I	N
T	W	A	G	I	S	L	A	D	K	P	K	S	L	I	T	V	A	R	K	E	I	N
T	W	A	G	I	S	L	A	D	K	P	K	S	L	I	T	V	V	R	K	E	I	N
T	W	A	G	I	S	L	A	D	K	P	K	S	L	I	T	V	V	R	K	E	I	N
T	W	A	G	I	S	L	A	D	K	P	K	S	L	I	T	V	A	R	N	E	I	N
T	W	A	G	I	S	L	A	D	K	P	K	S	L	I	T	V	A	R	N	E	I	N
T	W	A	G	I	S	L	A	D	K	P	K	S	L	I	A	V	A	R	N	E	I	N
T	W	A	G	I	S	L	A	D	K	P	K	S	L	I	T	V	A	R	N	E	I	N
T	W	A	G	I	S	L	A	D	K	P	K	S	L	I	T	V	A	R	N	E	I	N
T	W	A	G	I	S	L	A	D	K	P	K	S	L	I	T	V	A	R	N	E	I	N
T	W	A	G	I	S	L	A	D	K	P	K	S	L	I	T	V	A	R	N	E	I	N
T	W	A	G	I	S	L	A	D	K	P	K	S	L	I	T	V	A	R	N	E	I	N
T	W	A	G	I	S	L	A	D	K	P	K	S	L	I	T	V	A	R	N	E	I	N
T	W	A	G	I	S	L	A	D	K	P	K	S	L	I	T	V	A	R	N	E	I	N
T	W	A	G	I	S	L	A	D	K	P	K	S	L	I	T	V	A	R	N	E	I	N
T	W	A	G	I	S	L	A	D	K	P	K	S	L	I	T	V	A	R	N	E	I	N
T	W	A	G	I	S	L	A	D	K	P	K	S	L	I	T	V	A	R	N	E	I	N
T	W	A	G	I	S	L	A	D	K	P	K	S	L	I	T	V	A	R	N	E	I	N
T	W	A	G	I	S	L	A	D	K	P	K	S	L	I	T	V	A	R	N	E	I	N
T	W	A	G	I	S	L	A	D	K	P	K	S	L	I	T	V	A	R	N	E	I	N
T	W	A	G	I	S	L	A	D	K	P	K	S	L	I	T	V	A	R	N	E	I	N
T	W	A	G	I	S	L	A	D	K	P	K	S	L	I	T	V	A	R	N	E	I	N
T	W	A	G	I	S	L	A	D	K	P	K	S	L	I	T	V	A	R	N	E	I	N
T	W	A	G	I	S	L	A	D	K	P	K	S	L	I	T	V	A	R	N	E	I	N
T	W	A	G	I	S	L	A	D	K	P	K	S	L	I	T	V	A	R	N	E	I	N
T	W	A	G	I	S	L	A	D	K	P	K	S	L	I	T	V	A	R	N	E	I	N
T	W	A	G	I	S	L	A	D	K	P	K	S	L	I	T	V	A	R	N	E	I	N
T	W	A	G	I	S	L	A	D	K	P	K	S	L	I	T	V	A	R	N	E	I	N
T	W	A	G	I	S	L	A	D	K	P	K	S	L	I	T	V	A	R	N	E	I	N
T	W	A	G	I	S	L	A	D	K	P	K	S	L	I	T	V	A	R	N	E	I	N
T	W	A	G	I	S	L	A	D	K	P	K	S	L	I	T	V	A	R	N	E	I	N
T	W	A	G	I	S	L	A	D	K	P	K	S	L	I	T	V	A	R	N	E	I	N
T	W	A	G	I	S	L	A	D	K	P	K	S	L	I	T	V	A	R	N	E	I	N
T	W	A	G	I	S	L	A	D	K	P	K	S	L	I	T	V	A	R	N	E	I	N
T	W	A	G	I	S	L	A	D	K	P	K	S	L	I	T	V	A	R	N	E	I	N
T	W	A	G	I	S	L	A	D	K	P	K	S	L	I	T	V	A	R	N	E	I	N
T	W	A	G	I	S	L	A	D	K	P	K	S	L	I	T	V	A	R	N	E	I	N
T	W	A	G	I	S	L	A	D	K	P	K	S	L	I	T	V	A	R	N	E	I	N
T	W	A	G	I	S	L	A	D	K	P	K	S	L	I	T	V	A	R	N	E	I	N
T	W	A	G	I	S	L	A	D	K	P	K	S	L	I	T	V	A	R	N	E	I	N
T	W	A	G	I	S	L	A	D	K	P	K	S	L	I	T	V	A	R	N	E	I	N
T	W	A	G	I	S	L	A	D	K	P	K	S	L	I	T	V	A	R	N	E	I	N
T	W	A	G	I	S	L	A	D	K	P	K	S	L	I	T	V	A	R	N	E	I	N
T	W	A	G	I	S	L	A	D	K	P	K	S	L	I	T	V	A	R	N	E	I	N
T	W	A	G	I	S	L	A	D	K	P	K	S	L	I	T	V	A	R	N	E	I	N
T	W	A	G	I	S	L	A	D	K	P	K	S	L	I	T	V	A	R	N	E	I	N
T	W	A	G	I	S	L	A	D	K	P	K	S	L	I	T	V	A	R	N	E	I	N
T	W	A	G	I	S	L	A	D	K	P	K	S	L	I	T	V	A	R	N	E	I	N
T	W	A	G	I	S	L	A	D	K	P	K	S	L	I	T	V	A	R	N	E	I	N
T	W	A	G	I	S	L	A	D	K	P	K	S	L	I	T	V	A	R	N	E	I	N
T	W	A	G	I	S	L	A	D	K	P	K	S	L	I	T	V	A	R	N	E	I	N
T	W	A	G	I	S	L	A	D	K	P	K	S	L	I	T	V	A	R	N	E	I	N
T	W	A	G	I	S	L	A	D	K	P	K	S	L	I	T	V	A	R	N	E	I	N
T	W	A	G	I	S	L	A	D	K	P	K	S	L	I	T	V	A	R	N	E	I	N
T	W	A	G	I	S	L	A	D	K	P	K	S	L	I	T	V	A	R	N	E	I	N
T	W	A	G	I	S	L	A	D	K	P	K	S	L	I	T	V	A	R	N	E	I	N
T	W	A	G	I	S	L	A	D	K	P	K	S	L	I	T	V	A	R	N	E	I	N
T	W	A	G	I	S	L	A	D	K	P	K	S	L	I	T	V	A	R	N	E	I	N
T	W	A	G	I	S	L	A	D	K	P	K	S	L	I	T	V	A	R	N	E	I	N
T	W	A	G	I	S	L	A	D	K	P	K	S	L	I	T	V	A	R	N	E	I	N
T	W	A	G	I	S	L	A	D	K	P	K	S	L	I	T	V	A	R	N	E	I	N
T	W	A	G	I	S	L	A	D	K	P	K	S	L	I	T	V	A	R	N	E	I	N
T	W	A	G	I	S	L	A	D	K	P	K	S	L	I	T	V	A	R	N	E	I	N
T	W	A	G	I	S	L	A	D	K	P	K	S	L	I	T	V	A	R	N	E	I	N
T	W	A	G	I	S	L	A	D	K	P	K	S	L	I	T	V	A	R	N	E	I	N
T	W	A	G	I	S	L	A	D	K	P	K	S	L	I	T	V	A	R	N	E	I	N
T	W	A	G	I	S	L	A	D	K	P	K	S	L	I	T	V	A	R	N	E	I	N

T	W	A	G	I	S	L	A	D	K	P	K	S	L	I	T	V	A	R	N	E	I	N
T	W	A	G	I	S	L	A	D	K	P	K	S	L	I	T	V	A	R	N	E	I	N
T	W	A	G	I	S	L	A	D	K	P	K	S	L	I	T	V	A	R	N	E	I	N
T	W	A	G	I	S	L	A	D	K	P	K	S	L	I	T	V	A	R	N	E	I	N
T	W	A	G	I	S	L	A	D	K	P	K	S	L	I	T	V	A	R	N	E	I	N
T	W	A	G	I	S	L	A	D	K	P	K	S	L	I	T	V	A	R	N	E	I	N
T	W	A	G	I	S	L	A	D	K	P	K	S	L	I	T	V	A	R	N	E	I	N
T	W	A	G	I	S	L	A	D	K	P	K	S	L	I	T	V	A	R	N	E	I	N
T	W	A	G	I	S	L	A	D	K	P	K	S	L	I	T	V	A	R	N	E	I	N
T	W	A	G	I	S	L	A	D	K	P	K	S	L	I	T	V	A	R	N	E	I	N
T	W	A	G	I	S	L	A	D	K	P	K	S	L	I	T	V	A	R	N	E	I	N
T	W	A	G	I	S	L	A	D	K	P	K	T	L	I	T	V	A	R	K	E	I	N
T	W	A	G	I	S	L	A	D	K	P	K	T	L	I	T	V	A	R	K	E	I	N
T	W	A	G	I	S	L	A	D	K	P	K	T	L	I	T	V	A	R	K	E	I	N
T	W	A	G	I	S	L	A	D	K	P	K	T	L	I	T	V	A	R	K	E	I	N
T	W	A	G	I	S	L	A	D	K	P	K	T	L	I	T	V	A	R	K	E	I	N
T	W	A	G	I	S	L	A	D	K	P	K	T	L	I	T	V	A	R	K	E	I	N
T	W	A	G	I	S	L	A	D	K	P	K	T	L	I	T	V	A	R	K	E	I	N
T	W	A	G	I	S	L	A	D	K	P	K	T	L	I	T	V	A	R	K	E	I	N
T	W	A	G	I	S	L	A	D	K	P	K	T	L	I	T	V	A	R	K	E	I	N
T	R	A	A	M	S	S	S	E	R	P	R	S	V	V	T	V	A	Q	K	K	T	K
T	R	A	A	M	S	S	S	E	R	P	R	S	V	V	T	V	A	Q	K	K	T	K
T	R	A	A	M	S	S	S	E	R	P	R	S	V	V	T	V	A	Q	K	K	T	K
T	R	A	A	M	S	S	S	E	R	P	R	S	V	V	T	V	A	Q	K	K	T	K
T	R	A	A	M	S	S	S	E	R	P	R	S	V	V	T	V	A	Q	K	K	T	K
T	R	A	A	M	S	S	S	E	R	P	R	S	V	V	T	V	A	Q	K	K	T	K
T	R	A	A	M	S	S	S	E	R	P	R	S	V	V	T	V	A	Q	K	K	T	K
T	R	A	A	M	S	S	S	E	R	P	R	S	V	V	T	V	A	Q	K	K	T	K
T	W	A	G	I	S	L	A	D	K	P	K	T	L	I	T	V	A	R	K	E	I	N
T	W	A	G	I	S	L	A	D	K	P	K	S	L	I	T	V	A	R	N	E	I	N
T	W	A	G	I	S	L	A	D	K	P	K	S	L	I	T	V	A	R	N	E	I	N
T	W	A	G	I	S	L	A	D	K	P	K	S	L	I	T	V	A	R	N	E	I	N
T	W	A	G	I	S	L	A	D	K	P	K	S	L	I	T	V	A	R	N	E	I	N
T	W	A	G	I	F	L	A	D	K	P	K	S	L	I	T	V	A	R	N	E	I	N
T	W	A	G	I	S	L	A	D	K	P	K	S	L	I	T	V	A	R	N	E	I	N
T	W	A	G	I	S	L	A	D	K	P	K	S	L	I	T	V	A	R	N	E	I	N
T	W	A	G	I	S	L	A	D	K	P	K	S	L	I	T	V	A	R	N	E	I	N
T	W	A	G	I	S	L	A	D	K	P	K	S	L	I	T	V	A	R	N	E	I	N
T	W	A	G	I	S	L	A	D	K	P	K	S	L	I	T	V	A	R	N	E	I	N
T	W	A	G	I	S	L	A	D	K	P	K	S	L	I	T	V	A	R	N	E	I	N
T	W	A	G	I	S	L	A	D	K	P	K	S	L	I	T	V	A	R	N	E	I	N
T	W	A	G	I	S	L	A	D	K	P	K	S	L	I	T	V	A	R	N	E	I	N
T	W	A	G	I	S	L	A	D	K	P	K	S	L	I	T	V	A	R	N	E	I	N
T	W	A	G	I	S	L	A	D	K	P	K	S	L	I	T	V	A	R	N	E	I	N
T	W	A	G	I	S	L	A	D	K	P	K	S	L	I	T	V	A	R	N	E	I	N
T	W	A	G	I	S	L	A	D	K	P	K	S	L	I	T	V	A	R	N	E	I	N
T	W	A	G	I	S	L	A	D	K	P	K	S	L	I	T	V	A	R	N	E	I	N
T	W	A	G	I	S	L	A	D	K	P	K	S	L	I	T	V	A	R	K	E	I	N
T	W	A	G	I	S	L	A	D	K	P	K	S	L	I	T	V	A	R	K	K	I	N
T	W	A	G	I	S	L	A	D	K	P	K	S	L	I	T	V	A	R	K	E	I	N
T	W	A	G	I	S	L	A	D	K	P	K	S	L	I	T	V	A	R	K	E	I	N
T	W	A	G	I	S	L	A	D	K	P	K	S	L	I	T	V	A	R	K	E	I	N
T	W	A	G	I	S	L	A	D	K	P	K	S	L	I	T	V	A	R	K	E	I	N
T	W	A	G	I	S	L	A	D	K	P	K	S	L	I	T	V	A	R	K	E	I	N
T	W	A	G	I	S	L	A	D	K	P	K	S	L	I	T	V	A	R	K	E	I	N
T	W	A	G	I	S	L	A	D	K	P	K	S	L	I	T	V	A	R	K	K	I	N
T	W	A	G	I	S	L	A	D	K	P	K	S	L	I	T	V	A	R	K	E	I	N
T	W	A	G	I	S	L	A	D	K	P	K	S	L	I	T	V	A	R	K	E	I	N
T	W	A	G	I	S	L	A	D	K	P	K	S	L	I	T	V	A	R	K	E	I	N
T	W	A	G	I	S	L	A	D	K	P	K	S	L	I	T	V	A	R	K	E	I	N
T	W	A	G	I	S	L	A	D	K	P	K	S	L	I	T	V	A	R	K	E	I	N
T	W	A	G	I	S	L	A	D	K	P	K	S	L	I	T	V	A	R	K	E	I	N
T	W	A	G	I	S	L	A	D	K	P	K	S	L	I	T	V	A	R	K	E	I	N
T	W	A	G	I	S	L	A	D	K	P	K	S	L	I	T	V	A	R	K	E	I	N
T	W	A	G	I	S	L	A	D	K	P	K	S	L	I	T	V	A	R	K	E	I	N
T	W	A	G	I	S	L	A	D	K	P	K	S	L	I	T	V	A	R	K	E	I	N

T	W	A	G	I	S	L	A	D	K	P	K	S	L	I	T	V	A	R	K	E	I	N
T	W	A	G	I	S	L	A	D	K	P	K	S	L	I	T	V	A	R	K	E	I	N
T	W	A	G	I	S	L	A	D	K	P	K	S	L	I	T	V	A	R	K	E	I	N
T	W	A	G	I	S	L	A	D	K	P	K	S	L	I	T	V	A	R	K	E	I	N
T	W	A	G	I	S	L	A	D	K	P	K	S	L	I	T	V	A	R	K	E	I	N
T	W	A	G	I	S	L	A	D	K	P	K	S	L	I	T	V	A	R	K	E	I	N
T	W	A	G	I	S	L	A	D	K	P	K	S	L	I	T	V	A	R	K	E	I	N
T	W	A	G	I	S	L	A	D	K	P	K	S	L	I	T	V	A	R	K	E	I	N
T	W	A	G	I	S	L	A	D	K	P	K	S	L	I	T	V	A	R	K	E	I	N
T	W	A	G	I	S	L	A	D	K	P	K	S	L	I	T	V	A	R	K	E	I	N
T	W	A	G	I	S	L	A	D	K	P	K	S	L	I	T	V	A	R	K	E	I	N
T	W	A	G	I	S	L	A	D	K	P	K	S	L	I	T	V	A	R	K	E	I	N
T	W	A	G	I	S	L	A	D	K	P	K	S	L	I	T	V	A	R	K	E	I	N
T	W	A	G	I	S	L	A	D	K	P	K	S	L	I	T	V	A	R	K	E	I	N
T	W	A	G	I	S	L	A	D	K	P	K	S	L	I	T	V	A	R	K	E	I	N
T	W	A	G	I	S	L	A	D	K	P	K	S	L	I	T	V	A	R	K	E	I	N
T	W	A	G	I	S	L	A	D	K	P	K	S	L	I	T	V	A	R	K	E	I	N
T	W	A	G	I	S	L	A	D	K	P	K	S	L	I	T	V	A	R	K	E	I	N
T	W	A	G	I	S	L	A	D	K	P	K	S	L	I	T	V	A	R	K	E	I	N
T	W	A	G	I	S	L	A	D	K	P	K	S	L	I	T	V	A	R	K	E	I	N
T	W	A	G	I	S	L	A	D	K	P	K	S	L	I	T	V	A	R	K	E	I	N
T	W	A	G	I	S	L	A	D	K	P	K	S	L	I	T	V	A	R	K	E	I	N
T	W	A	G	I	S	L	A	D	K	P	K	S	L	I	T	V	A	R	K	E	I	N
T	W	A	G	I	S	L	A	D	K	P	K	T	L	I	T	V	A	R	K	E	I	N
T	W	A	G	I	S	L	A	D	K	P	K	S	L	I	T	V	A	R	K	E	I	N
T	W	A	G	I	S	L	A	D	K	P	K	S	L	I	T	V	A	R	K	E	I	N
T	W	A	G	I	S	L	A	D	K	P	K	S	L	I	T	V	A	R	K	E	I	N
T	W	A	G	I	S	L	A	D	K	P	K	S	L	I	T	V	A	R	K	E	I	N
T	W	A	G	I	S	L	A	D	K	P	K	S	L	I	T	V	A	R	K	E	I	N
T	W	A	G	I	S	L	A	D	K	P	K	S	L	I	T	V	A	R	K	E	I	N
T	W	A	G	I	S	L	A	D	K	P	K	S	L	I	T	V	A	R	K	E	I	N
T	W	A	G	I	S	L	A	D	K	P	K	S	L	I	T	V	A	R	K	E	I	N
T	W	A	G	I	S	L	A	D	K	P	K	S	L	I	T	V	A	R	K	E	I	N
T	W	A	G	I	S	L	A	D	K	P	K	S	L	I	T	V	A	R	K	E	I	N
T	W	A	G	I	S	L	A	D	K	P	K	S	L	I	T	V	A	R	K	E	I	N
T	W	A	G	I	S	L	A	D	K	P	K	S	L	I	T	V	A	R	K	E	I	N
T	W	A	G	I	S	L	A	D	K	P	K	S	L	I	T	V	A	R	K	E	I	N
T	W	A	G	I	S	L	A	D	K	P	K	S	L	I	T	V	A	R	K	E	I	N
T	W	A	G	I	S	L	A	D	K	P	K	S	L	I	T	V	A	R	K	E	I	N
T	W	A	G	I	S	L	A	D	K	P	K	S	L	I	T	V	A	R	K	E	I	N
T	W	A	G	I	S	L	A	D	K	P	K	S	L	I	T	V	A	R	K	E	I	N
T	W	A	G	I	S	L	A	D	K	P	K	S	L	I	T	V	A	R	K	E	I	N
T	W	A	G	I	S	L	A	D	K	P	K	S	L	I	T	V	A	R	K	E	I	N
T	W	A	G	I	S	L	A	D	K	P	K	S	L	I	T	V	A	R	K	E	I	N
T	W	A	G	I	S	L	A	D	K	P	K	S	L	I	T	V	A	R	K	E	I	N
T	W	T	G	I	S	L	A	D	K	P	K	S	L	I	T	V	A	R	K	E	I	N
T	W	T	G	I	S	L	A	D	K	P	K	S	L	I	T	V	A	R	K	E	I	N
T	W	A	G	I	S	L	A	D	K	P	K	S	L	I	T	V	A	R	K	E	I	N
T	W	A	G	I	S	L	A	D	K	P	K	S	L	I	T	V	A	R	K	E	I	N
T	W	A	G	I	S	L	A	D	K	P	K	T	L	I	T	V	A	R	K	E	I	N
T	W	A	G	I	S	L	A	D	K	P	K	T	L	I	T	V	A	R	K	E	I	N
T	W	A	G	I	S	L	A	D	K	P	K	T	L	I	T	V	A	R	K	E	I	N
T	W	A	G	I	S	L	A	D	K	P	K	T	L	I	T	V	A	R	K	K	I	N
T	W	A	G	I	S	L	A	D	K	P	K	T	L	I	T	V	A	R	K	E	I	N
T	W	A	G	I	S	L	A	D	K	P	K	T	L	I	T	V	A	R	K	E	I	N
T	W	A	G	I	S	L	A	D	K	P	K	T	L	I	T	V	A	R	K	E	I	N
T	W	A	G	I	S	L	A	D	K	P	K	T	L	I	T	V	A	R	K	E	I	N
T	W	A	G	I	S	L	A	D	K	P	K	T	L	I	T	V	A	R	K	E	I	N
T	W	A	G	I	S	L	A	D	K	P	K	T	L	I	T	V	A	R	K	E	I	N
T	W	A	G	I	S	L	A	D	K	P	K	T	L	I	T	V	A	R	K	E	I	N
T	W	A	G	I	S	L	A	D	K	P	K	T	L	I	T	V	A	R	K	E	I	N
T	W	A	G	I	S	L	A	D	K	P	K	T	L	I	T	V	A	R	K	E	I	N
T	W	A	G	I	S	L	A	D	K	P	K	T	L	I	T	V	A	R	K	E	I	N
T	W	A	G	I	S	L	A	D	K	P	K	T	L	I	T	V	A	R	K	E	I	N
T	W	A	G	I	S	L	A	D	K	P	K	T	L	I	T	V	A	R	K	E	I	N
T	W	A	G	I	S	L	A	D	K	P	K	T	L	I	T	V	A	R	K	E	I	N
T	W	A	G	I	S	L	A	D	K	P	K	T	L	I	T	V	A	R	K	E	I	N
T	W	A	G	I	S	L	A	D	K	P	K	T	L	I	T	V	A	R	K	E	I	N

T	W	A	G	I	S	L	A	D	K	P	K	T	L	I	T	V	A	R	K	E	I	N
T	W	A	G	I	S	L	A	D	K	P	K	T	L	I	T	V	A	R	K	E	I	N
T	W	A	G	I	S	L	A	D	K	P	K	T	L	I	T	V	A	R	K	E	I	N
T	W	A	G	I	S	L	A	D	K	P	K	T	L	I	T	V	A	R	K	E	I	N
T	W	A	G	I	S	L	A	D	K	P	K	T	L	I	T	V	A	R	K	E	I	N
T	W	A	G	I	S	L	A	D	K	P	K	T	L	I	T	V	A	R	K	E	I	N
T	W	A	G	I	S	L	A	D	K	P	K	T	L	I	T	V	A	R	K	E	I	N
T	W	A	G	I	S	L	A	D	K	P	K	T	L	I	T	V	A	R	K	E	I	N
T	W	A	G	I	S	L	A	D	K	P	K	T	L	I	T	V	A	R	K	E	I	N
T	W	A	G	I	S	L	A	D	K	P	K	T	L	I	T	V	A	R	K	E	I	N

																		wchA			
5	8	11	13	16	22	27	31	32	40	48	104	107	108	115	149	154	155	175	178	219	230
I	S	A	I	F	T	A	T	E	A	V	T	A	L	L	R	N	E	C	V	I	R
L	P	A	V	F	A	T	T	E	V	A	L	V	F	R	R	D	D	Y	V	V	C
I	S	V	I	S	T	T	A	K	A	V	T	A	L	L	G	N	E	Y	I	I	R
L	P	A	V	F	A	T	T	E	V	A	L	V	L	R	R	D	D	Y	V	V	C
L	P	A	V	F	A	T	T	E	V	A	L	V	L	R	R	D	D	Y	V	V	C
L	P	A	V	F	A	T	T	E	V	A	L	V	L	R	R	D	D	Y	V	V	C
L	P	A	V	F	A	T	T	E	V	A	L	V	L	R	R	D	D	Y	V	V	C
L	P	A	V	F	A	T	T	E	V	A	L	V	L	R	R	D	D	Y	V	V	C
L	P	A	V	F	A	T	T	E	V	A	L	V	L	R	R	D	D	Y	V	V	C
L	P	A	V	F	A	T	T	E	V	A	L	V	L	R	R	D	D	Y	V	V	C
L	P	A	V	F	A	T	T	E	V	A	L	V	L	R	R	D	D	Y	V	V	C

217	219	220	227
S	G	N	N
S	G	S	K
F	G	N	N
F	G	N	N
F	G	N	N
F	G	N	N
F	G	N	N
F	G	N	N
F	G	N	N
F	G	N	N
F	G	N	N
F	G	N	N
F	G	N	N
F	G	N	N
F	G	N	N
F	G	N	N
F	G	N	N
F	G	N	N
F	E	N	N
F	G	N	N
F	G	N	N
S	G	N	N
S	G	N	N
S	G	N	N
S	G	N	N
S	G	N	N
S	G	N	N
S	G	N	N
S	G	N	N
S	G	N	N
S	G	N	N

															wchA		
5	8	13	22	27	31	40	48	104	107	108	115	154	155	175	219	230	231
I	S	I	T	A	T	A	V	T	A	L	L	N	E	C	I	R	S
L	P	V	A	T	T	V	A	L	V	F	R	D	D	Y	V	C	I
I	S	I	T	A	A	A	V	T	A	L	L	N	E	C	I	R	S
I	S	I	T	A	A	A	V	T	A	L	L	N	E	C	I	R	S
I	S	I	T	A	A	A	V	T	A	L	L	N	E	C	I	R	S
I	S	I	T	A	A	A	V	T	A	L	L	N	E	C	I	R	S
I	S	I	T	A	A	A	V	T	A	L	L	N	E	C	I	R	S
I	S	I	T	A	A	A	V	T	A	L	L	N	E	C	I	R	S
I	S	I	T	A	A	A	V	T	A	L	L	N	E	C	I	R	S
I	S	I	T	A	A	A	V	T	A	L	L	N	E	C	I	R	S
I	S	I	T	A	A	A	V	T	A	L	L	N	E	C	I	R	S
I	S	I	T	A	A	A	V	T	A	L	L	N	E	C	I	R	S
I	S	I	T	A	A	A	V	T	A	L	L	N	E	C	I	R	S
I	S	I	T	A	A	A	V	T	A	L	L	N	E	C	I	R	S
I	S	I	T	A	A	A	V	T	A	L	L	N	E	C	I	R	S
I	S	I	T	A	A	A	V	T	A	L	L	N	E	C	I	R	S
I	S	I	T	A	A	A	V	T	A	L	L	N	E	C	I	R	S
I	S	I	T	A	A	A	V	T	A	L	L	N	E	C	I	R	S
I	S	I	T	A	A	A	V	T	A	L	L	N	E	C	I	R	S
I	S	I	T	A	A	A	V	T	A	L	L	N	E	C	I	R	S
I	S	I	T	A	A	A	V	T	A	L	L	N	E	C	I	R	S
I	S	I	T	A	T	A	V	T	A	L	L	N	E	C	I	R	S
I	S	I	T	A	T	A	V	T	A	L	L	N	E	C	I	R	S
I	S	I	T	A	T	A	V	T	A	L	L	N	E	C	I	R	S
I	S	I	T	A	T	A	V	T	A	L	L	N	E	C	I	R	S
I	S	I	T	A	A	A	V	T	A	L	L	N	E	C	I	R	S
I	S	I	T	A	T	A	V	T	A	L	L	N	E	C	I	R	S
I	S	I	T	A	T	A	V	T	A	L	L	N	E	C	I	R	S
I	S	I	T	A	T	A	V	T	A	L	L	N	E	C	I	R	S
I	S	I	T	A	T	A	V	T	A	L	L	N	E	C	I	R	S
I	S	I	T	A	T	A	V	T	A	L	L	N	E	C	I	R	S

199	202	212	223
A	G	S	R
A	G	N	K
V	R	S	-
A	G	S	R

wchA																	
11	16	27	31	32	40	56	57	62	104	107	108	112	134	135	142	149	154
A	F	A	T	E	A	Q	D	G	T	A	L	V	I	L	R	R	N
V	S	T	A	K	A	Q	D	G	T	A	L	V	I	L	R	G	N
-	F	A	T	E	V	R	N	R	L	V	F	M	V	F	H	R	D
A	F	A	A	E	A	Q	D	G	T	A	L	V	I	L	R	R	N

wchA

5	8	13	22	27	31	40	48	104	107	108	115	143	154	155	175	219	230	231	234	239	264	268
I	S	I	T	A	T	A	V	T	A	L	L	V	N	E	C	I	R	S	R	R	I	V
L	P	V	A	T	T	V	A	L	V	F	R	V	D	D	Y	V	C	I	H	C	V	I
I	S	I	T	A	A	A	V	T	A	L	L	V	N	E	C	I	R	S	R	R	I	V
I	S	I	T	A	A	A	V	T	T	L	L	I	N	E	Y	I	R	S	R	R	I	V
I	S	I	T	A	A	A	V	T	T	L	L	I	N	E	Y	I	R	S	R	R	I	V
I	S	I	T	A	A	A	V	T	A	L	L	V	N	E	C	I	R	S	R	R	I	V
I	S	I	T	A	A	A	V	T	A	L	L	V	N	E	C	I	R	S	R	R	I	V
I	S	I	T	A	A	A	V	T	A	L	L	V	N	E	C	I	R	S	R	R	I	V
I	S	I	T	A	A	A	V	T	A	L	L	V	N	E	C	I	R	S	R	R	I	V
I	S	I	T	A	A	A	V	T	T	L	L	I	N	E	Y	I	R	S	R	R	I	V
I	S	I	T	A	A	A	V	T	T	L	L	I	N	E	Y	I	R	S	R	R	I	V
I	S	I	T	A	A	A	V	T	T	L	L	I	N	E	Y	I	R	S	R	R	I	V
I	S	I	T	A	A	A	V	T	A	L	L	V	N	E	C	I	R	S	R	R	I	V
I	S	I	T	A	A	A	V	T	A	L	L	V	N	E	C	I	R	S	R	R	I	V
I	S	I	T	A	A	A	V	T	A	L	L	V	N	E	C	I	R	S	R	R	I	V
I	S	I	T	A	A	A	V	T	A	L	L	V	N	E	C	I	R	S	R	R	I	V
I	S	I	T	A	A	A	V	T	A	L	L	V	N	E	C	I	R	S	R	R	I	V
I	S	I	T	A	A	A	V	T	A	L	L	V	N	E	C	I	R	S	R	R	I	V
I	S	I	T	A	A	A	V	T	A	L	L	V	N	E	C	I	R	S	R	R	I	V
I	S	I	T	A	A	A	V	T	A	L	L	V	N	E	C	I	R	S	R	R	I	V
I	S	I	T	A	A	A	V	T	A	L	L	V	N	E	C	I	R	S	R	R	I	V
I	S	I	T	A	A	A	V	T	A	L	L	V	N	E	C	I	R	S	R	R	I	V
I	S	I	T	A	A	A	V	T	A	L	L	V	N	E	C	I	R	S	R	R	I	V
I	S	I	T	A	A	A	V	T	A	L	L	V	N	E	C	I	R	S	R	R	I	V
I	S	I	T	A	A	A	V	T	A	L	L	V	N	E	C	I	R	S	R	R	I	V
I	S	I	T	A	A	A	V	T	A	L	L	V	N	E	C	I	R	S	R	R	I	V
I	S	I	T	A	A	A	V	T	A	L	L	V	N	E	C	I	R	S	R	R	I	V

38	39	44	47	52	54	57	58	66	68	69	70	72	73	76	80	84	85	89	90	91	92	101
T	A	I	Y	I	D	Q	D	I	L	V	Q	L	K	L	L	I	S	L	E	D	R	I
T	A	I	Y	I	D	Q	D	I	L	V	Q	L	K	L	L	I	S	L	E	D	R	I
M	V	L	F	F	S	N	N	V	F	N	S	I	R	F	I	V	L	I	A	E	R	V
T	A	I	Y	I	D	Q	D	I	L	V	Q	L	K	L	L	I	S	L	E	D	Q	I
T	A	I	Y	I	D	Q	D	I	L	V	Q	L	K	L	L	I	S	L	E	D	Q	I
T	A	I	Y	I	D	Q	D	I	L	V	Q	L	K	L	L	I	S	L	E	D	Q	I
T	A	I	Y	I	D	Q	D	I	L	V	Q	L	K	L	L	I	S	L	E	D	Q	I
T	A	I	Y	I	D	Q	D	I	L	V	Q	L	K	L	L	I	S	L	E	D	Q	I
T	A	I	Y	I	D	Q	D	I	L	V	Q	L	K	L	L	I	S	L	E	D	Q	I
T	A	I	Y	I	D	Q	D	I	L	V	Q	L	K	L	L	I	S	L	E	D	Q	I
T	A	I	Y	I	D	Q	D	I	L	V	Q	L	K	L	L	I	S	L	E	D	Q	I
T	A	I	Y	I	D	Q	D	I	L	V	Q	L	K	L	L	I	S	L	E	D	Q	I
T	A	I	Y	I	D	Q	D	I	L	V	Q	L	K	L	L	I	S	L	E	D	Q	I
T	A	I	Y	I	D	Q	D	I	L	V	Q	L	K	L	L	I	S	L	E	D	Q	I
T	A	I	Y	I	D	Q	D	I	L	V	Q	L	K	L	L	I	S	L	E	D	Q	I
T	A	I	Y	I	D	Q	D	I	L	V	Q	L	K	L	L	I	S	L	E	D	Q	I
T	A	I	Y	I	D	Q	D	I	L	V	Q	L	K	L	L	I	S	L	E	D	Q	I
T	A	I	Y	I	D	Q	D	I	L	V	Q	L	K	L	L	I	S	L	E	D	Q	I
T	A	I	Y	I	D	Q	D	I	L	V	Q	L	K	L	L	I	S	L	E	D	Q	I
T	A	I	Y	I	D	Q	D	I	L	V	Q	L	K	L	L	I	S	L	E	D	Q	I
T	A	I	Y	I	D	Q	D	I	L	V	Q	L	K	L	L	I	S	L	E	D	Q	I
T	A	I	Y	I	D	Q	D	I	L	V	Q	L	K	L	L	I	S	L	E	D	Q	I
T	A	I	Y	I	D	Q	D	I	L	V	Q	L	K	L	L	I	S	L	E	D	Q	I
T	A	I	Y	I	D	Q	D	I	L	V	Q	L	K	L	L	I	S	L	E	D	Q	I
T	A	I	Y	I	D	Q	D	I	L	V	Q	L	K	L	L	I	S	L	E	D	Q	I
T	A	I	Y	I	D	Q	D	I	L	V	Q	L	K	L	L	I	S	L	E	D	Q	I
T	A	I	Y	I	D	Q	D	I	L	V	Q	L	K	L	L	I	S	L	E	D	Q	I
T	A	I	Y	I	D	Q	D	I	L	V	Q	L	K	L	L	I	S	L	E	D	Q	I
T	A	I	Y	I	D	Q	D	I	L	V	Q	L	K	L	L	I	S	L	E	D	Q	I

T	A	I	Y	I	D	Q	D	I	L	V	Q	L	K	L	L	I	S	L	E	D	Q	I
T	A	I	Y	I	D	Q	D	I	L	V	Q	L	K	L	L	I	S	L	E	D	Q	I

236	245	258
K	V	A
K	A	A
K	A	A
K	A	A
K	A	A
K	A	A
K	A	A
K	A	A
K	A	A
K	A	A
K	A	A
K	A	A
K	A	A
K	A	A
K	A	A
K	A	A
K	A	A
K	A	A
K	A	A
K	A	A
K	A	A
K	A	A
K	A	A
K	A	A
K	A	A
K	A	A
K	A	A
K	A	A
K	A	A
K	A	A
K	A	A
K	A	A
K	A	T
K	A	T
K	A	T
K	A	T
K	A	T
K	A	T
K	A	T
K	A	T
K	A	T
K	A	T
K	A	T
K	A	T
K	A	T
K	A	T
K	A	T
K	A	T
K	A	T
K	A	T
K	A	T
K	A	T
K	A	T
K	A	T
K	A	T
K	A	T

K	A	T
K	A	T
K	A	T
K	A	T
K	A	T
K	A	T
K	A	T
K	A	T
K	A	T
K	A	T
K	A	T
K	A	T
K	A	T
K	A	T
K	A	T
K	A	T
K	A	T
K	A	T
K	A	T
K	A	T
K	A	T
K	A	T
K	A	T
K	A	T
K	A	T
K	A	T
K	A	T
K	A	T
K	A	T
K	A	T
K	A	T
K	A	T
K	A	T
K	A	T
K	A	T
K	A	T
K	A	T
K	A	T
K	A	T
K	A	T
K	A	T
K	A	T
K	A	T
K	A	T
K	A	T
K	A	T
K	A	T
K	A	T
K	A	T
K	A	T
K	A	A
K	A	A
K	A	A
K	A	A
K	A	A
K	A	A
K	A	A
K	A	A
K	A	A
K	A	A
K	A	A
K	A	A
K	A	A
K	A	A
K	A	A
K	A	A
K	A	A

K	A	A
K	A	A
K	A	A
K	A	A
K	A	A
K	A	A
K	A	A
K	A	A
K	A	A
K	A	A
K	A	A
N	A	A
K	A	A
K	A	A
K	A	A
K	A	A
K	A	A
K	A	A
K	A	A
K	A	A
K	A	A
K	A	A
K	A	A
K	A	A
K	A	A
K	A	A
K	A	A
K	A	A
K	A	A
K	A	A
K	A	A
K	A	A
K	A	A
K	A	A
K	A	A
K	A	A
K	A	A
K	A	A
K	A	A
K	A	A
K	A	A
K	A	A
K	A	A
K	A	A
K	A	A
K	A	A
K	A	A
K	A	A
K	A	A
K	A	A
K	V	A
K	V	A
K	V	A
K	V	A
K	V	A
K	V	A
K	V	A
K	V	A
K	V	A
K	V	A
K	V	A
K	V	A
K	V	A
K	V	A
K	V	A
K	V	A
K	V	A

K	V	A
K	V	A
K	V	A
K	V	A
K	V	A
K	V	A
K	V	A
K	V	A
K	V	A
K	V	A
K	V	A
K	V	A
K	V	A
K	V	A
K	V	A
K	V	A
K	V	A
K	V	A
K	V	A
K	V	A
K	V	A
K	V	A
K	V	A
K	V	A
K	V	A
K	V	A
K	V	A
K	V	A
K	V	A
K	V	A
K	V	A
K	V	A
K	V	A
K	V	A
K	V	A
K	V	A
K	V	A
K	V	A
K	V	A
K	V	A
K	V	A
K	V	A
K	V	A
K	V	A
K	V	A
K	V	A
K	V	A
K	V	A
K	V	A
K	V	A
K	V	A
K	V	A
K	V	A
K	V	A
K	V	A
K	V	A
K	V	A
K	V	A
K	V	A
K	V	A
K	V	A
K	V	A
K	V	A
K	V	A
K	V	A
K	V	A
K	V	A

K	V	A
K	V	A
K	V	A
K	V	A
K	V	A
K	V	A
K	V	A
K	V	A
K	V	A
K	V	A
K	V	A
K	V	A
K	V	A
K	V	A
K	V	A
K	V	A
K	V	A
K	V	A
K	V	A
K	V	A
K	V	A
K	V	A
K	V	A
K	V	A
K	V	A
K	V	A
K	V	A

193	195	200	209	214	215	222	223	230
L	H	F	D	K	Y	G	N	N
L	H	F	D	K	C	G	N	N
L	H	F	N	K	Y	G	N	N
L	H	F	N	K	Y	G	N	N
L	H	F	N	K	Y	G	N	N
L	H	F	N	K	Y	G	N	N
L	H	F	N	K	Y	G	N	N
L	H	F	N	K	Y	G	N	N
L	H	F	N	K	Y	G	N	N
L	H	F	N	K	Y	G	N	N
L	H	F	N	K	Y	G	N	N
L	H	F	N	K	Y	G	N	N
L	H	F	N	K	Y	G	N	N
L	H	F	N	K	Y	G	N	N
L	H	F	N	K	Y	G	N	N
L	H	F	N	K	Y	G	N	N
L	H	F	N	K	Y	G	N	N
L	H	F	N	K	Y	G	N	N
L	H	F	N	K	Y	G	N	N
L	H	F	N	K	Y	G	N	N
L	H	F	N	K	Y	G	N	N
L	H	F	N	K	Y	G	N	N
L	H	F	N	K	Y	G	N	N
L	H	F	N	K	Y	G	N	N
L	H	F	N	K	Y	G	N	N
L	H	F	N	K	Y	G	N	N
L	H	F	N	K	Y	G	N	N
L	H	F	N	K	Y	G	N	N
L	H	F	N	K	Y	G	N	N
L	H	F	N	K	Y	G	N	N
L	H	F	N	K	Y	G	N	N
L	H	F	N	K	Y	G	N	N
L	H	F	N	K	Y	G	N	N
L	H	F	N	K	Y	G	N	N
L	H	F	N	K	Y	G	N	N
L	H	F	N	K	Y	G	N	N
L	H	F	N	K	Y	G	N	N
L	H	F	N	K	Y	G	N	N
L	H	F	N	K	Y	G	N	N
L	H	F	N	K	Y	G	N	N
L	H	F	N	K	Y	G	N	N
L	H	F	N	K	Y	G	N	N
L	H	F	N	K	Y	G	N	N
L	H	F	N	K	Y	G	N	N
L	H	F	N	K	Y	G	N	N
L	H	F	N	K	Y	G	N	N
L	H	F	N	K	Y	G	N	N
L	H	F	N	K	Y	G	N	N
L	H	F	N	K	Y	G	N	N
L	H	F	N	K	Y	G	N	N
L	H	F	N	K	Y	G	N	N
L	H	F	N	K	Y	G	N	N
L	H	F	N	K	Y	G	N	N
L	H	F	D	K	Y	G	N	N
L	H	F	N	K	Y	G	N	N
L	H	F	N	K	Y	G	N	N
L	H	F	N	K	Y	G	N	N
L	H	F	N	K	Y	G	N	N
L	H	F	N	K	Y	G	N	N
L	H	F	D	K	Y	G	N	N
L	H	F	D	K	Y	G	N	N
L	H	F	D	K	Y	G	N	N
L	H	F	D	K	Y	G	N	N

5	8	13	22	27	31	40	45	48	66	91	104	107
I	S	I	T	A	T	A	H	V	E	R	T	A
I	S	I	T	A	T	A	H	V	E	R	T	A
I	S	I	T	A	A	A	H	V	E	R	T	T
I	S	I	T	A	A	A	H	V	E	R	T	T
I	S	I	T	A	A	A	H	V	E	R	T	T
I	S	I	T	A	A	A	H	V	E	R	T	T
I	S	I	T	A	A	A	H	V	E	R	T	T
I	S	I	T	A	A	A	H	V	E	R	T	T
I	S	I	T	A	A	A	H	V	E	R	T	T
I	S	I	T	A	A	A	H	V	E	R	T	T
I	S	I	T	A	A	A	H	V	E	R	T	T
I	S	I	T	A	A	A	H	V	E	R	T	T
I	S	I	T	A	A	A	H	V	E	R	T	T
I	S	I	T	A	A	A	H	V	E	R	T	T
I	S	I	T	A	A	A	H	V	E	R	T	T
I	S	I	T	A	A	A	H	V	E	R	T	T
I	S	I	T	A	A	A	H	V	E	R	T	T
I	S	I	T	A	A	A	H	V	E	R	T	T
I	S	I	T	A	A	A	H	V	E	R	T	T
I	S	I	T	A	A	A	H	V	E	R	T	T
I	S	I	T	A	A	A	H	V	E	R	T	T
I	S	I	T	A	A	A	H	V	E	R	T	T
I	S	I	T	A	A	A	H	V	E	R	T	T
I	S	I	T	A	A	A	H	V	E	R	T	T
I	S	I	T	A	A	A	H	V	E	R	T	T
I	S	I	T	A	A	A	H	V	E	R	T	T
I	S	I	T	A	A	A	H	V	E	R	T	T
I	S	I	T	A	A	A	H	V	E	R	T	T
I	S	I	T	A	A	A	H	V	E	R	T	T
I	S	I	T	A	A	A	H	V	E	R	T	T
I	S	I	T	A	A	A	H	V	E	R	T	A
I	S	I	T	A	A	A	H	V	E	R	T	A
I	S	I	T	A	A	A	H	V	E	R	T	A
I	S	I	T	A	A	A	H	V	E	R	T	A
I	S	I	T	A	A	A	H	V	E	R	T	A
I	S	I	T	A	A	A	H	V	E	R	T	A
I	S	I	T	A	A	A	H	V	E	R	T	A
I	S	I	T	A	A	A	H	V	E	R	T	A
I	S	I	T	A	A	A	H	V	E	R	T	A
I	S	I	T	A	A	A	H	V	E	R	T	A
I	S	I	T	A	A	A	H	V	E	R	T	A
I	S	I	T	A	A	A	H	V	E	R	T	A
I	S	I	T	A	A	A	H	V	E	R	T	A
I	S	I	T	A	A	A	H	V	E	R	T	A
I	S	I	T	A	A	A	H	V	E	R	T	A
I	S	I	T	A	A	A	H	V	E	R	T	A
I	S	I	T	A	A	A	H	V	E	R	T	A
I	S	I	T	A	A	A	H	V	E	R	T	A
I	S	I	T	A	A	A	H	V	E	R	T	A
I	S	I	T	A	A	A	H	V	E	R	T	A
I	S	I	T	A	A	A	H	V	E	R	T	A
I	S	I	T	A	A	A	H	V	E	R	T	A
I	S	I	T	A	A	A	H	V	E	R	T	A
I	S	I	T	A	T	A	H	V	G	R	T	A
I	S	I	T	A	A	A	H	V	E	R	T	A
I	S	I	T	A	A	A	H	V	E	R	T	A
I	S	I	T	A	A	A	H	V	E	R	T	A
I	S	I	T	A	A	A	H	V	E	R	T	A
I	S	I	T	A	A	A	H	V	E	R	T	A
I	S	I	T	A	T	A	H	V	G	R	T	A
I	S	I	T	A	T	A	H	V	G	R	T	A
I	S	I	T	A	T	A	H	V	G	R	T	A
I	S	I	T	A	T	A	H	V	G	R	T	A

L	H	F	D	K	Y	G	N	N	I	S	I	T	A	T	A	H	V	G	R	T	A
L	H	F	D	K	Y	G	N	N	I	S	I	T	A	T	A	H	V	G	R	T	A
L	H	F	D	K	Y	G	N	N	I	S	I	T	A	T	A	H	V	G	R	T	A
L	H	F	D	K	Y	G	N	N	I	S	I	T	A	T	A	H	V	G	R	T	A
L	H	F	D	K	Y	G	N	N	I	S	I	T	A	T	A	H	V	G	R	T	A
L	H	F	D	K	Y	G	N	N	I	S	I	T	A	T	A	H	V	G	R	T	A
L	H	F	D	K	Y	G	N	N	I	S	I	T	A	T	A	H	V	G	R	T	A
L	H	F	D	K	Y	G	N	N	I	S	I	T	A	T	A	H	V	G	R	T	A
L	H	F	D	K	Y	G	N	N	I	S	I	T	A	T	A	H	V	G	R	T	A
L	H	F	D	K	Y	G	N	N	I	S	I	T	A	T	A	H	V	G	R	T	A
L	H	F	D	K	Y	G	N	N	I	S	I	T	A	T	A	H	V	G	R	T	A
L	H	F	D	K	Y	G	N	N	I	S	I	T	A	T	A	H	V	G	R	T	A
L	H	F	D	K	Y	G	N	N	I	S	I	T	A	T	A	H	V	G	R	T	A
L	H	F	D	K	Y	G	N	N	I	S	I	T	A	T	A	H	V	G	R	T	A
L	H	F	D	K	Y	G	N	N	I	S	I	T	A	T	A	H	V	G	R	T	A
L	H	F	D	K	Y	G	N	N	I	S	I	T	A	T	A	H	V	G	R	T	A
L	H	F	D	K	Y	G	N	N	I	S	I	T	A	T	A	H	V	G	R	T	A
L	H	F	D	K	Y	G	N	N	I	S	I	T	A	T	A	H	V	G	R	T	A
L	H	F	D	K	Y	G	N	N	I	S	I	T	A	T	A	H	V	G	R	T	A
L	H	F	D	K	Y	G	N	N	I	S	I	T	A	T	A	H	V	G	R	T	A
L	H	F	D	K	Y	G	N	N	I	S	I	T	A	T	A	H	V	G	R	T	A
L	H	F	N	K	Y	G	N	N	I	S	I	T	A	A	A	H	V	E	R	T	A
L	H	F	N	K	Y	G	N	N	I	S	I	T	A	A	A	H	V	E	R	T	A
L	H	F	N	K	Y	G	N	N	I	S	I	T	A	A	A	H	V	E	R	T	A
L	H	F	N	K	Y	G	N	N	I	S	I	T	A	A	A	H	V	E	R	T	A
L	H	F	N	K	Y	G	N	N	I	S	I	T	A	A	A	H	V	E	R	T	A
L	H	F	N	K	Y	G	N	N	I	S	I	T	A	A	A	H	V	E	R	T	A
L	H	F	D	K	Y	G	N	N	I	S	I	T	A	T	A	H	V	E	R	T	A
L	H	F	D	K	Y	G	N	N	I	S	I	T	A	T	A	H	V	E	R	T	A
L	H	F	D	K	Y	G	N	N	I	S	I	T	A	T	A	H	V	E	R	T	A
L	H	F	D	K	Y	G	N	N	I	S	I	T	A	T	A	H	V	E	R	T	A
L	H	F	D	K	Y	G	N	N	I	S	I	T	A	T	A	H	V	E	R	T	A
L	H	F	D	K	Y	G	N	N	I	S	I	T	A	T	A	H	V	E	R	T	A
L	H	F	D	K	Y	G	N	N	I	S	I	T	A	T	A	H	V	E	R	T	A
L	H	F	D	K	Y	G	N	N	I	S	I	T	A	T	A	H	V	E	R	T	A
L	H	F	D	K	Y	G	N	N	I	S	I	T	A	T	A	H	V	E	R	T	A
L	H	F	D	K	Y	G	N	N	I	S	I	T	A	T	A	H	V	E	R	T	A
L	H	F	D	K	Y	G	N	N	I	S	I	T	A	T	A	H	V	E	R	T	A
L	H	F	D	K	Y	G	N	N	I	S	I	T	A	T	A	H	V	E	R	T	A
L	H	F	D	K	Y	G	N	N	I	S	I	T	A	T	A	H	V	E	R	T	A
L	H	F	D	K	Y	G	N	N	I	S	I	T	A	T	A	H	V	E	R	T	A
L	H	F	D	K	Y	G	N	N	I	S	I	T	A	T	A	H	V	E	R	T	A
L	H	F	D	K	Y	G	N	N	I	S	I	T	A	T	A	H	V	E	R	T	A
L	H	F	D	K	Y	G	N	N	I	S	I	T	A	T	A	H	V	E	R	T	A
L	H	F	D	K	Y	G	N	N	I	S	I	T	A	T	A	H	V	E	R	T	A
L	H	F	D	K	Y	G	N	N	I	S	I	T	A	T	A	H	V	E	R	T	A
L	H	F	D	K	Y	G	N	N	I	S	I	T	A	T	A	H	V	E	R	T	A
L	H	F	D	K	Y	G	N	N	I	S	I	T	A	T	A	H	V	E	R	T	A
L	H	F	D	K	Y	G	N	N	I	S	I	T	A	T	A	H	V	E	R	T	A
L	H	F	D	K	Y	G	N	N	I	S	I	T	A	T	A	H	V	E	R	T	A
L	H	F	D	K	Y	G	N	N	I	S	I	T	A	T	A	H	V	E	R	T	A
L	H	F	D	K	Y	G	N	N	I	S	I	T	A	T	A	H	V	E	R	T	A
L	H	F	D	K	Y	G	N	N	I	S	I	T	A	T	A	H	V	E	R	T	A
L	H	F	D	K	Y	G	N	N	I	S	I	T	A	T	A	H	V	E	R	T	A
L	H	F	D	K	Y	G	N	N	I	S	I	T	A	T	A	H	V	E	R	T	A
L	H	F	D	K	Y	G	N	N	I	S	I	T	A	T	A	H	V	E	R	T	A
L	H	F	D	K	Y	G	N	N	I	S	I	T	A	T	A	H	V	E	R	T	A
L	H	F	D	K	Y	G	N	N	I	S	I	T	A	T	A	H	V	E	R	T	A
L	H	F	D	K	Y	G	N	N	I	S	I	T	A	T	A	H	V	E	R	T	A
L	H	F	D	K	Y	G	N	N	I	S	I	T	A	T	A	H	V	E	R	T	A
L	H	F	D	K	Y	G	N	N	I	S	I	T	A	T	A	H	V	E	R	T	A
L	H	F	D	K	Y	G	N	N	I	S	I	T	A	T	A	H	V	E	R	T	A
L	H	F	D	K	Y	G	N	N	I	S	I	T	A	T	A	H	V	E	R	T	A
L	H	F	D	K	Y	G	N	N	I	S	I	T	A	T	A	H	V	E	R	T	A
L	H	F	D	K	Y	G	N	N	I	S	I	T	A	T	A	H	V	E	R	T	A
L	H	F	D	K	Y	G	N	N	I	S	I	T	A	T	A	H	V	E	R	T	A
L	H	F	D	K	Y	G	N	N	I	S	I	T	A	T	A	H	V	E	R	T	A

L	H	F	D	K	Y	G	N	N
L	H	F	D	K	Y	G	N	N
L	H	F	D	K	Y	G	N	N
L	H	F	D	K	Y	G	N	N
L	H	F	D	K	Y	G	N	N
L	H	F	D	K	Y	G	N	N
L	H	F	D	K	Y	G	N	N
L	H	F	D	K	Y	G	N	N
L	H	F	D	K	Y	G	N	N
L	H	F	D	K	Y	G	N	N
L	H	F	D	K	Y	G	N	N
L	H	F	D	K	Y	G	N	N
L	H	F	D	K	Y	G	N	N
L	H	F	D	K	Y	G	N	N
L	H	F	D	K	Y	G	N	N
L	H	F	D	K	Y	G	N	N
L	H	F	D	K	Y	G	N	N
L	H	F	D	K	Y	G	N	N
L	H	F	D	K	Y	G	N	N
L	H	F	D	K	Y	G	N	N
L	H	F	D	K	Y	G	N	N
L	H	F	D	K	Y	G	N	N
L	H	F	D	K	Y	G	N	N
L	H	F	D	K	Y	G	N	N
L	H	F	D	K	Y	G	N	N
L	H	F	D	K	Y	G	N	N
L	H	F	D	K	Y	G	N	N
L	H	F	D	K	Y	G	N	N
L	H	F	D	K	Y	G	N	N
L	H	F	D	K	Y	G	N	N
L	H	F	D	K	Y	G	N	N
L	H	F	D	K	Y	G	N	N
L	H	F	D	K	Y	G	N	N
L	H	F	D	K	Y	G	N	N
L	H	F	D	K	Y	G	N	N
L	H	F	D	K	Y	G	N	N
L	H	F	D	K	Y	G	N	N
L	H	F	D	K	Y	G	N	N
L	H	F	D	K	Y	G	N	N
L	H	F	D	K	Y	G	N	N
L	H	F	D	K	Y	G	N	N
L	H	F	D	K	Y	G	N	N
L	H	F	D	K	Y	G	N	N
L	H	F	D	K	Y	G	N	N
L	H	F	D	K	Y	G	N	N
L	H	F	D	K	Y	G	N	N
L	H	F	D	K	Y	G	N	N
L	H	F	D	K	Y	G	N	N
L	H	F	D	K	Y	G	N	N
L	H	F	D	K	Y	G	N	N
L	H	F	D	K	Y	G	N	N
L	H	F	D	K	Y	G	N	N
L	H	F	D	K	Y	G	N	N
L	H	F	D	K	Y	G	N	N
L	H	F	D	K	Y	G	N	N
L	H	F	D	K	Y	G	N	N
L	H	F	D	K	Y	G	N	N
L	H	F	D	K	Y	G	N	N
L	H	F	D	K	Y	G	N	N
L	H	F	D	K	Y	G	N	N
L	H	F	D	K	Y	G	N	N
L	H	F	D	K	Y	G	N	N
L	H	F	D	K	Y	G	N	N
L	H	F	D	K	Y	G	N	N
L	H	F	D	K	Y	G	N	N
L	H	F	D	K	Y	G	N	N
L	H	F	D	K	Y	G	N	N

I	S	I	T	A	T	A	H	V	E	R	T	A
I	S	I	T	A	T	A	H	V	E	R	T	A
I	S	I	T	A	T	A	H	V	E	R	T	A
I	S	I	T	A	T	A	H	V	E	R	T	A
I	S	I	T	A	T	A	H	V	E	R	T	A
I	S	I	T	A	T	A	H	V	E	R	T	A
I	S	I	T	A	T	A	H	V	E	R	T	A
I	S	I	T	A	T	A	H	V	E	R	T	A
I	S	I	T	A	T	A	H	V	E	R	T	A
I	S	I	T	A	T	A	H	V	E	R	T	A
I	S	I	T	A	T	A	H	V	E	R	T	A
I	S	I	T	A	T	A	H	V	E	R	T	A
I	S	I	T	A	T	A	H	V	E	R	T	A
I	S	I	T	A	T	A	H	V	E	R	T	A
I	S	I	T	A	T	A	H	V	E	R	T	A
I	S	I	T	A	T	A	H	V	E	R	T	A
I	S	I	T	A	T	A	H	V	E	R	T	A
I	S	I	T	A	T	A	H	V	E	R	T	A
I	S	I	T	A	T	A	H	V	E	R	T	A
I	S	I	T	A	T	A	H	V	E	R	T	A
I	S	I	T	A	T	A	H	V	E	R	T	A
I	S	I	T	A	T	A	H	V	E	R	T	A
I	S	I	T	A	T	A	H	V	E	R	T	A
I	S	I	T	A	T	A	H	V	E	R	T	A
I	S	I	T	A	T	A	H	V	E	R	T	A
I	S	I	T	A	T	A	H	V	E	R	T	A
I	S	I	T	A	T	A	H	V	E	R	T	A
I	S	I	T	A	T	A	H	V	E	R	T	A
I	S	I	T	A	T	A	H	V	E	R	T	A
I	S	I	T	A	T	A	H	V	E	R	T	A
I	S	I	T	A	T	A	H	V	E	R	T	A
I	S	I	T	A	T	A	H	V	E	R	T	A
I	S	I	T	A	T	A	H	V	E	R	T	A
I	S	I	T	A	T	A	H	V	E	R	T	A
I	S	I	T	A	T	A	H	V	E	R	T	A
I	S	I	T	A	T	A	H	V	E	R	T	A
I	S	I	T	A	T	A	H	V	E	R	T	A
I	S	I	T	A	T	A	H	V	E	R	T	A
I	S	I	T	A	T	A	H	V	E	R	T	A
I	S	I	T	A	T	A	H	V	E	R	T	A
I	S	I	T	A	T	A	H	V	E	R	T	A
I	S	I	T	A	T	A	H	V	E	R	T	A
I	S	I	T	A	T	A	H	V	E	R	T	A
I	S	I	T	A	T	A	H	V	E	R	T	A
I	S	I	T	A	T	A	H	V	E	R	T	A
I	S	I	T	A	T	A	H	V	E	R	T	A
I	S	I	T	A	T	A	H	V	E	R	T	A
I	S	I	T	A	T	A	H	V	E	R	T	A
I	S	I	T	A	T	A	H	V	E	R	T	A
I	S	I	T	A	T	A	H	V	E	R	T	A
I	S	I	T	A	T	A	H	V	E	R	T	A
I	S	I	T	A	T	A	H	V	E	R	T	A
I	S	I	T	A	T	A	H	V	E	R	T	A
I	S	I	T	A	T	A	H	V	E	R	T	A
I	S	I	T	A	T	A	H	V	E	R	T	A
I	S	I	T	A	T	A	H	V	E	R	T	A
I	S	I	T	A	T	A	H	V	E	R	T	A
I	S	I	T	A	T	A	H	V	E	R	T	A
I	S	I	T	A	T	A	H	V	E	R	T	A
I	S	I	T	A	T	A	H	V	E	R	T	A
I	S	I	T	A	T	A	H	V	E	R	T	A
I	S	I	T	A	T	A	H	V	E	R	T	A
I	S	I	T	A	T	A	H	V	E	R	T	A
I	S	I	T	A	T	A	H	V	E	R	T	A
I	S	I	T	A	T	A	H	V	E	R	T	A
I	S	I	T	A	T	A	H	V	E	R	T	A
I	S	I	T	A	T	A	H	V	E	R	T	A

L	H	F	D	K	Y	G	N	N	I	S	I	T	A	T	A	H	V	E	R	T	A
L	H	F	D	K	Y	G	N	N	I	S	I	T	A	T	A	H	V	E	R	T	A
L	H	F	D	K	Y	G	N	N	I	S	I	T	A	T	A	H	V	E	R	T	A
L	H	F	D	K	Y	G	N	N	I	S	I	T	A	T	A	H	V	E	R	T	A
L	H	F	N	K	Y	G	N	N	I	S	I	T	A	A	A	H	V	E	R	T	A
L	H	F	N	K	Y	G	N	N	I	S	I	T	A	A	A	H	V	E	R	T	A
L	H	F	N	K	Y	G	N	N	I	S	I	T	A	A	A	H	V	E	R	T	A
L	H	F	N	K	Y	G	N	N	I	S	I	T	A	A	A	H	V	E	R	T	A
L	H	F	N	K	Y	G	N	N	I	S	I	T	A	A	A	H	V	E	R	T	A
L	H	F	N	K	Y	G	N	N	I	S	I	T	A	A	A	H	V	E	R	T	A
L	H	F	N	K	Y	G	N	N	I	S	I	T	A	A	A	H	V	E	R	T	A
L	H	F	N	K	Y	G	N	N	I	S	I	T	A	A	A	H	V	E	R	T	A
L	H	F	N	K	Y	G	N	N	I	S	I	T	A	A	A	H	V	E	R	T	A
L	H	F	N	K	Y	G	N	N	I	S	I	T	A	A	A	H	V	E	R	T	A
L	H	F	N	K	Y	G	N	N	I	S	I	T	A	A	A	H	V	E	R	T	A
L	H	F	N	K	Y	G	N	N	I	S	I	T	A	A	A	H	V	E	R	T	A
L	H	F	N	K	Y	G	N	N	I	S	I	T	A	A	A	H	V	E	R	T	A
L	H	F	N	K	Y	G	N	N	I	S	I	T	A	A	A	H	V	E	R	T	A
L	H	F	N	K	Y	G	N	N	I	S	I	T	A	A	A	H	V	E	R	T	A
L	H	F	N	K	Y	G	N	N	I	S	I	T	A	A	A	H	V	E	R	T	A
L	H	F	N	K	Y	G	N	N	I	S	I	T	A	A	A	H	V	E	R	T	A
L	H	F	N	K	Y	G	N	N	I	S	I	T	A	A	A	H	V	E	R	T	A
L	H	F	N	K	Y	G	N	N	I	S	I	T	A	A	A	H	V	E	R	T	A
L	H	F	N	K	Y	G	N	N	I	S	I	T	A	A	A	H	V	E	R	T	A
L	H	F	N	K	Y	G	N	N	I	S	I	T	A	A	A	H	V	E	R	T	A
L	H	F	N	K	Y	G	N	N	I	S	I	T	A	A	A	H	V	E	R	T	A
L	H	F	N	K	Y	G	N	N	I	S	I	T	A	A	A	H	V	E	R	T	A
L	H	F	N	K	Y	G	N	N	I	S	I	T	A	A	A	H	V	E	R	T	A
L	H	F	N	K	Y	G	N	N	I	S	I	T	A	A	A	H	V	E	R	T	A
L	H	F	N	K	Y	G	N	N	I	S	I	T	A	A	A	H	V	E	R	T	A
L	H	F	N	K	Y	G	N	N	I	S	I	T	A	A	A	H	V	E	R	T	A
L	H	F	N	K	Y	G	N	N	I	S	I	T	A	A	A	H	V	E	R	T	A
L	H	F	N	K	Y	G	N	N	I	S	I	T	A	A	A	H	V	E	R	T	A
L	H	F	N	K	Y	G	N	N	I	S	I	T	A	A	A	H	V	E	R	T	A
L	H	F	N	K	Y	G	N	N	I	S	I	T	A	A	A	H	V	E	R	T	A
L	H	F	N	K	Y	G	N	N	I	S	I	T	A	A	A	H	V	E	R	T	A
L	H	F	N	K	Y	G	N	N	I	S	I	T	A	A	A	H	V	E	R	T	A
L	H	F	N	K	Y	G	N	N	I	S	I	T	A	A	A	H	V	E	R	T	A
L	H	F	N	K	Y	G	N	N	I	S	I	T	A	A	A	H	V	E	R	T	A
L	H	F	N	K	Y	G	N	N	I	S	I	T	A	A	A	H	V	E	R	T	A
L	H	F	N	K	Y	G	N	N	I	S	I	T	A	A	A	H	V	E	R	T	A
L	H	F	N	K	Y	G	N	N	I	S	I	T	A	A	A	H	V	E	R	T	A
L	H	F	N	K	Y	G	N	N	I	S	I	T	A	A	A	H	V	E	R	T	A
L	H	F	N	K	Y	G	N	N	I	S	I	T	A	A	A	H	V	E	R	T	A
L	H	F	N	K	Y	G	N	N	I	S	I	T	A	A	A	H	V	E	R	T	A
L	H	F	N	K	Y	G	N	N	I	S	I	T	A	A	A	H	V	E	R	T	A
L	H	F	N	K	Y	G	N	N	I	S	I	T	A	A	A	H	V	E	R	T	A
L	H	F	N	K	Y	G	N	N	I	S	I	T	A	A	A	H	V	E	R	T	A
L	H	F	N	K	Y	G	N	N	I	S	I	T	A	A	A	H	V	E	R	T	A
L	H	F	N	K	Y	G	N	N	I	S	I	T	A	A	A	H	V	E	R	T	A
L	H	F	N	K	Y	G	N	N	I	S	I	T	A	A	A	H	V	E	R	T	A
L	H	F	N	K	Y	G	N	N	I	S	I	T	A	A	A	H	V	E	R	T	A
L	H	F	N	K	Y	G	N	N	I	S	I	T	A	A	A	H	V	E	R	T	A
L	H	F	N	K	Y	G	N	N	I	S	I	T	A	A	A	H	V	E	R	T	A
L	H	F	N	K	Y	G	N	N	I	S	I	T	A	A	A	H	V	E	R	T	A
L	H	F	N	K	Y	G	N	N	I	S	I	T	A	A	A	H	V	E	R	T	A
L	H	F	N	K	Y	G	N	N	I	S	I	T	A	A	A	H	V	E	R	T	A
L	H	F	N	K	Y	G	N	N	I	S	I	T	A	A	A	H	V	E	R	T	A
L	H	F	N	K	Y	G	N	N	I	S	I	T	A	A	A	H	V	E	R	T	A
L	H	F	N	K	Y	G	N	N	I	S	I	T	A	A	A	H	V	E	R	T	A
L	H	F	N	K	Y	G	N	N	I	S	I	T	A	A	A	H	V	E	R	T	A
L	H	F	N	K	Y	G	N	N	I	S	I	T	A	A	A	H	V	E	R	T	A
L	H	F	N	K	Y	G	N	N	I	S	I	T	A	A	A	H	V	E	R	T	A
L	H	F	N	K	Y	G	N	N	I	S	I	T	A	A	A	H	V	E	R	T	A
L	H	F	N	K	Y	G	N	N	I	S	I	T	A	A	A	H	V	E	R	T	A
L	H	F	N	K	Y	G	N	N	I	S	I	T	A	A	A	H	V	E	R	T	A
L	H	F	N	K	Y	G	N	N	I	S	I	T	A	A	A	H	V	E	R	T	A

L	H	F	N	K	Y	G	N	N	I	S	I	T	A	A	A	H	V	E	R	T	A
L	H	F	N	K	Y	G	N	N	I	S	I	T	A	A	A	H	V	E	R	T	A
L	H	F	N	K	Y	G	N	N	I	S	I	T	A	A	A	H	V	E	R	T	A
L	H	F	N	K	Y	G	N	N	I	S	I	T	A	A	A	H	V	E	R	T	A
L	H	F	N	K	Y	G	N	N	I	S	I	T	A	A	A	H	V	E	R	T	A
L	H	F	N	K	Y	G	N	N	I	S	I	T	A	A	A	H	V	E	R	T	A
L	H	F	N	K	Y	G	N	N	I	S	I	T	A	A	A	H	V	E	R	T	A
L	H	F	N	K	Y	G	N	N	I	S	I	T	A	A	A	H	V	E	R	T	A
L	H	F	N	K	Y	G	N	N	I	S	I	T	A	A	A	H	V	E	R	T	A
L	H	F	N	K	Y	G	N	N	I	S	I	T	A	A	A	H	V	E	R	T	A
L	H	F	N	K	Y	G	N	N	I	S	I	T	A	A	A	Y	V	E	R	T	A
L	H	F	N	K	Y	G	N	N	I	S	I	T	A	A	A	H	V	E	R	T	A
L	H	F	N	K	Y	G	N	N	I	S	I	T	A	A	A	H	V	E	R	T	A
L	H	F	N	K	Y	G	N	N	I	S	I	T	A	A	A	H	V	E	R	T	A
L	H	F	N	K	Y	G	N	N	I	S	I	T	A	A	A	H	V	E	R	T	A
L	H	F	N	K	Y	G	N	N	I	S	I	T	A	A	A	H	V	E	R	T	A
L	H	F	N	K	Y	G	N	N	I	S	I	T	A	A	A	H	V	E	R	T	A
L	H	F	N	K	Y	G	N	N	I	S	I	T	A	A	A	H	V	E	R	T	A
L	H	F	N	K	Y	G	N	N	I	S	I	T	A	A	A	H	V	E	R	T	A
L	H	F	N	K	Y	G	N	N	I	S	I	T	A	A	A	H	V	E	R	T	A
I	Q	C	D	E	Y	G	S	K	L	P	V	A	T	T	V	H	A	E	R	L	V
I	Q	C	D	E	Y	G	S	K	L	P	V	A	T	T	V	H	A	E	R	L	V
I	Q	C	D	E	Y	G	S	K	L	P	V	A	T	T	V	H	A	E	R	L	V
I	Q	C	D	E	Y	G	S	K	L	P	V	A	T	T	V	H	A	E	R	L	V
I	Q	C	D	E	Y	G	S	K	L	P	V	A	T	T	V	H	A	E	R	L	V
I	Q	C	D	E	Y	G	S	K	L	P	V	A	T	T	V	H	A	E	R	L	V
I	Q	C	D	E	Y	G	S	K	L	P	V	A	T	T	V	H	A	E	R	L	V
I	Q	C	D	E	Y	G	S	K	L	P	V	A	T	T	V	H	A	E	R	L	V
L	H	F	D	K	Y	G	N	N	I	S	I	T	A	A	A	H	V	E	R	T	A
L	H	F	N	K	Y	G	N	N	I	S	I	T	A	A	A	H	V	E	R	T	A
L	H	F	N	K	Y	G	N	N	I	S	I	T	A	A	A	H	V	E	R	T	A
L	H	F	N	K	Y	G	N	N	I	S	I	T	A	A	A	H	V	E	R	T	A
L	H	F	N	K	Y	G	N	N	I	S	I	T	A	A	A	H	V	E	R	T	A
L	H	F	N	K	Y	G	N	N	I	S	I	T	A	A	A	H	V	E	R	T	A
L	H	F	N	K	Y	G	N	N	I	S	I	T	A	A	A	H	V	E	R	T	A
L	H	F	N	K	Y	G	N	N	I	S	I	T	A	A	A	H	V	E	R	T	A
L	H	F	N	K	Y	G	N	N	I	S	I	T	A	A	A	H	V	E	R	T	A
L	H	F	N	K	Y	G	N	N	I	S	I	T	A	A	A	H	V	E	R	T	A
L	H	F	N	K	Y	G	N	N	I	S	I	T	A	A	A	H	V	E	R	T	A
L	H	F	N	K	Y	G	N	N	I	S	I	T	A	A	A	H	V	E	R	T	A
L	H	F	N	K	Y	G	N	N	I	S	I	T	A	A	A	H	V	E	R	T	A
L	H	F	N	K	Y	G	N	N	I	S	I	T	A	A	A	H	V	E	R	T	A
L	H	F	N	K	Y	G	N	N	I	S	I	T	A	A	A	H	V	E	R	T	A
L	H	F	N	K	Y	G	N	N	I	S	I	T	A	A	A	H	V	E	R	T	A
L	H	F	N	K	Y	E	N	N	I	S	I	T	A	A	A	H	V	E	R	T	A
L	H	F	N	K	Y	G	N	N	I	S	I	T	A	A	A	H	V	E	R	T	A
L	H	F	N	K	Y	G	N	N	I	S	I	T	A	A	A	H	V	E	R	T	A
L	H	F	D	K	Y	G	N	N	I	S	I	T	A	T	A	H	V	E	R	T	A
L	H	F	D	K	Y	G	N	N	I	S	I	T	A	T	A	H	V	E	R	T	A
L	H	F	D	K	Y	G	N	N	I	S	I	T	A	T	A	H	V	E	R	T	A
L	H	F	D	K	Y	G	N	N	I	S	I	T	A	T	A	H	V	E	R	T	A
L	H	F	D	K	Y	G	N	N	I	S	I	T	A	T	A	H	V	E	R	T	A
L	H	F	D	K	Y	G	N	N	I	S	I	T	A	T	A	H	V	E	R	T	A
L	H	F	D	K	Y	G	N	N	I	S	I	T	A	T	A	H	V	E	R	T	A
L	H	F	D	K	Y	G	N	N	I	S	I	T	A	T	A	H	V	E	R	T	A
L	H	F	D	K	Y	G	N	N	I	S	I	T	A	T	A	H	V	E	R	T	A
L	H	F	D	K	Y	G	N	N	I	S	I	T	A	T	A	H	V	E	R	T	A
L	H	F	D	K	Y	G	N	N	I	S	I	T	A	T	A	H	V	E	R	T	A
L	H	F	D	K	Y	G	N	N	I	S	I	T	A	T	A	H	V	E	R	T	A
L	H	F	D	K	Y	G	N	N	I	S	I	T	A	T	A	H	V	E	R	T	A
L	H	F	D	K	Y	G	N	N	I	S	I	T	A	T	A	H	V	E	R	T	A
L	H	F	D	K	Y	G	N	N	I	S	I	T	A	T	A	H	V	E	R	T	A
L	H	F	D	K	Y	G	N	N	I	S	I	T	A	T	A	H	V	E	R	T	A
L	H	F	D	K	Y	G	N	N	I	S	I	T	A	T	A	H	V	E	R	T	A
L	H	F	D	K	Y	G	N	N	I	S	I	T	A	T	A	H	V	E	R	T	A
L	H	F	D	K	Y	G	N	N	I	S	I	T	A	T	A	H	V	E	R	T	A

L	H	F	D	K	Y	G	N	N	I	S	I	T	A	T	A	H	V	E	R	T	A
L	H	F	D	K	Y	G	N	N	I	S	I	T	A	T	A	H	V	E	R	T	A
L	H	F	D	K	Y	G	N	N	I	S	I	T	A	T	A	H	V	E	R	T	A
L	H	F	D	K	Y	G	N	N	I	S	I	T	A	T	A	H	V	E	R	T	A
L	H	F	D	K	Y	G	N	N	I	S	I	T	A	T	A	H	V	E	R	T	A
L	H	F	D	K	Y	G	N	N	I	S	I	T	A	T	A	H	V	E	R	T	A
L	H	F	D	K	Y	G	N	N	I	S	I	T	A	T	A	H	V	E	R	T	A
L	H	F	D	K	Y	G	N	N	I	S	I	T	A	T	A	H	V	E	R	T	A
L	H	F	D	K	Y	G	N	N	I	S	I	T	A	T	A	H	V	E	R	T	A
L	H	F	D	K	Y	G	N	N	I	S	I	T	A	T	A	H	V	E	R	T	A
L	H	F	D	K	Y	G	N	N	I	S	I	T	A	T	A	H	V	E	R	T	A
L	H	F	D	K	Y	G	N	N	I	S	I	T	A	T	A	H	V	E	R	T	A
L	H	F	D	K	Y	G	N	N	I	S	I	T	A	T	A	H	V	E	R	T	A
L	H	F	D	K	Y	G	N	N	I	S	I	T	A	T	A	H	V	E	R	T	A
L	H	F	D	K	Y	G	N	N	I	S	I	T	A	T	A	H	V	E	R	T	A
L	H	F	D	K	Y	G	N	N	I	S	I	T	A	T	A	H	V	E	R	T	A
L	H	F	D	K	Y	G	N	N	I	S	I	T	A	T	A	H	V	E	R	T	A
L	H	F	D	K	Y	G	N	N	I	S	I	T	A	T	A	H	V	E	R	T	A
L	H	F	D	K	Y	G	N	N	I	S	I	T	A	T	A	H	V	E	R	T	A
L	H	F	D	K	Y	G	N	N	I	S	I	T	A	T	A	H	V	E	R	T	A
L	H	F	D	K	Y	G	N	N	I	S	I	T	A	T	A	H	V	E	R	T	A
L	H	F	D	K	Y	G	N	N	I	S	I	T	A	T	A	H	V	E	R	T	A
L	H	F	D	K	Y	G	N	N	I	S	I	T	A	T	A	H	V	E	R	T	A
L	H	F	D	K	Y	G	N	N	I	S	I	T	A	A	A	H	V	E	R	T	A
L	H	F	N	K	Y	G	N	N	I	S	I	T	A	A	A	H	V	E	R	T	A
L	H	F	N	K	Y	G	N	N	I	S	I	T	A	A	A	H	V	E	R	T	A
L	H	F	N	K	Y	G	N	N	I	S	I	T	A	A	A	H	V	E	R	T	A
L	H	F	N	K	Y	G	N	N	I	S	I	T	A	A	A	H	V	E	R	T	A
L	H	F	N	K	Y	G	N	N	I	S	I	T	A	A	A	H	V	E	R	T	A
L	H	F	N	K	Y	G	N	N	I	S	I	T	A	A	A	H	V	E	R	T	A
L	H	F	N	K	Y	G	N	N	I	S	I	T	A	A	A	H	V	E	R	T	A
L	H	F	N	K	Y	G	N	N	I	S	I	T	A	A	A	H	V	E	R	T	A
L	H	F	N	K	Y	G	N	N	I	S	I	T	A	A	A	H	V	E	R	T	A
L	H	F	N	K	Y	G	N	N	I	S	I	T	A	A	A	H	V	E	R	T	A
L	H	F	N	K	Y	G	N	N	I	S	I	T	A	A	A	H	V	E	R	T	A
L	H	F	N	K	Y	G	N	N	I	S	I	T	A	A	A	H	V	E	R	T	A
L	H	F	N	K	Y	G	N	N	I	S	I	T	A	A	A	H	V	E	R	T	A
L	H	F	N	K	Y	G	N	N	I	S	I	T	A	A	A	H	V	E	R	T	A
L	H	F	N	K	Y	G	N	N	I	S	I	T	A	A	A	H	V	E	R	T	A
L	H	F	N	K	Y	G	N	N	I	S	I	T	A	A	A	H	V	E	R	T	A
L	H	F	N	K	Y	G	N	N	I	S	I	T	A	A	A	H	V	E	R	T	T
L	H	F	N	K	Y	G	N	N	I	S	I	T	A	A	A	H	V	E	R	T	T
L	H	F	N	K	Y	G	N	N	I	S	I	T	A	A	A	H	V	E	R	T	T
L	H	F	N	K	Y	G	N	N	I	S	I	T	A	A	A	H	V	E	R	T	T
L	H	F	N	K	Y	G	N	N	I	S	I	T	A	A	A	H	V	E	R	T	T
L	H	F	N	K	Y	G	N	N	I	S	I	T	A	A	A	H	V	E	R	T	A
L	H	F	N	K	Y	G	N	N	I	S	I	T	A	A	A	H	V	E	R	T	A
L	H	F	N	K	Y	G	N	N	I	S	I	T	A	A	A	H	V	E	R	T	A
L	H	F	N	K	Y	G	N	N	I	S	I	T	A	A	A	H	V	E	R	T	A
L	H	F	D	K	Y	G	N	N	I	S	I	T	A	A	A	H	V	E	Q	T	A
L	H	F	D	K	Y	G	N	N	I	S	I	T	A	A	A	H	V	E	Q	T	A
L	H	F	D	K	Y	G	N	N	I	S	I	T	A	A	A	H	V	E	Q	T	A
L	H	F	D	K	Y	G	N	N	I	S	I	T	A	A	A	H	V	E	Q	T	A
L	H	F	D	K	Y	G	N	N	I	S	I	T	A	A	A	H	V	E	Q	T	A
L	H	F	D	K	Y	G	N	N	I	S	I	T	A	A	A	H	V	E	Q	T	A
L	H	F	D	K	Y	G	N	N	I	S	I	T	A	A	A	H	V	E	Q	T	A
L	H	F	D	K	Y	G	N	N	I	S	I	T	A	A	A	H	V	E	Q	T	A
L	H	F	D	K	Y	G	N	N	I	S	I	T	A	A	A	H	V	E	Q	T	A
L	H	F	D	K	Y	G	N	N	I	S	I	T	A	A	A	H	V	E	Q	T	A
L	H	F	D	K	Y	G	N	N	I	S	I	T	A	A	A	H	V	E	Q	T	A
L	H	F	D	K	Y	G	N	N	I	S	I	T	A	A	A	H	V	E	Q	T	A
L	H	F	D	K	Y	G	N	N	I	S	I	T	A	A	A	H	V	E	Q	T	A
L	H	F	D	K	Y	G	N	N	I	S	I	T	A	A	A	H	V	E	Q	T	A
L	H	F	D	K	Y	G	N	N	I	S	I	T	A	A	A	H	V	E	Q	T	A
L	H	F	D	K	Y	G	N	N	I	S	I	T	A	A	A	H	V	E	Q	T	A
L	H	F	D	K	Y	G	N	N	I	S	I	T	A	A	A	H	V	E	Q	T	A
L	H	F	D	K	Y	G	N	N	I	S	I	T	A	A	A	H	V	E	Q	T	A

L	H	F	D	K	Y	G	N	N
L	H	F	D	K	Y	G	N	N
L	H	F	D	K	Y	G	N	N
L	H	F	D	K	Y	G	N	N
L	H	F	D	K	Y	G	N	N
L	H	F	D	K	Y	G	N	N
L	H	F	D	K	Y	G	N	N
L	H	F	D	K	Y	G	N	N
L	H	F	D	K	Y	G	N	N
L	H	F	D	K	Y	G	N	N

I	S	I	T	A	A	A	H	V	E	Q	T	A
I	S	I	T	A	A	A	H	V	E	Q	T	A
I	S	I	T	A	A	A	H	V	E	Q	T	A
I	S	I	T	A	A	A	H	V	E	Q	T	A
I	S	I	T	A	A	A	H	V	E	Q	T	A
I	S	I	T	A	A	A	H	V	E	Q	T	A
I	S	I	T	A	A	A	H	V	E	Q	T	A
I	S	I	T	A	A	A	H	V	E	Q	T	A
I	S	I	T	A	A	A	H	V	E	Q	T	A
I	S	I	T	A	A	A	H	V	E	Q	T	A

231	234	239	240	264	268	270	308	323	343	352	430	431	449	451
S	R	R	E	I	V	A	Q	A	D	R	N	G	F	R
I	H	C	E	V	I	S	H	E	E	H	D	G	L	K
S	R	R	K	I	V	A	Q	A	D	C	D	D	L	K
I	H	C	E	V	I	S	H	E	E	H	D	G	L	K
I	H	C	E	V	I	S	H	E	E	H	D	G	L	K
I	H	C	E	V	I	S	H	E	E	H	D	G	L	K
I	H	C	E	V	I	S	H	E	E	H	D	G	L	K
I	H	C	E	V	I	S	H	E	E	H	D	G	L	K
I	H	C	E	V	I	S	H	E	E	H	D	G	L	K
I	H	C	E	V	I	S	H	E	E	H	D	G	L	K
I	H	C	E	V	I	S	H	E	E	H	D	G	L	K

2	115	121	168	247	251	257
N	F	G	R	S	K	G
D	L	D	K	C	L	N
[redacted]						
D	L	D	K	C	L	N
D	L	D	K	C	L	N
D	L	D	K	C	L	N
D	L	D	K	C	L	N
D	L	D	K	C	L	N
D	L	D	K	C	L	N
D	L	D	K	C	L	N
D	L	D	K	C	L	N

234	239	264	268	270	308	323	343	352	430	449	451
R	R	I	V	A	Q	A	D	R	N	F	R
H	C	V	I	S	H	E	E	H	D	L	K
R	R	I	V	A	Q	A	D	R	N	F	R
R	R	I	V	A	Q	A	D	R	N	F	R
R	R	I	V	A	Q	A	D	R	N	F	R
R	R	I	V	A	Q	A	D	R	N	F	R
R	R	I	V	A	Q	A	D	R	N	F	R
R	R	I	V	A	Q	A	D	R	N	F	R
R	R	I	V	A	Q	A	D	R	N	F	R
R	R	I	V	A	Q	A	D	R	N	F	R
R	R	I	V	A	Q	A	D	R	N	F	R
R	R	I	V	A	Q	A	D	R	N	F	R
R	R	I	V	A	Q	A	D	R	N	F	R
R	R	I	V	A	Q	A	D	R	N	F	R
R	R	I	V	A	Q	A	D	R	N	F	R
R	R	I	V	A	Q	A	D	R	N	F	R
R	R	I	V	A	Q	A	D	R	N	F	R
R	R	I	V	A	Q	A	D	R	N	F	R
R	R	I	V	A	Q	A	D	R	N	F	R
R	R	I	V	A	Q	A	D	R	N	F	R
R	R	I	V	A	Q	A	D	R	N	F	R
R	R	I	V	A	Q	A	D	R	N	F	R
R	R	I	V	A	Q	A	D	R	N	F	R
R	R	I	V	A	Q	A	D	R	N	F	R
R	R	I	V	A	Q	A	D	R	N	F	R
R	R	I	V	A	Q	A	D	R	N	F	R
R	R	I	V	A	Q	A	D	R	N	F	R
R	R	I	V	A	Q	A	D	R	N	F	R
R	R	I	V	A	Q	A	D	R	N	F	R
R	R	I	V	A	Q	A	D	R	N	F	R
R	R	I	V	A	Q	A	D	R	N	F	R

2	14	115	121	168	247	251	257	259	263
N	V	F	G	R	S	K	G	N	I
D	V	L	D	K	C	L	N	E	V
N	V	F	G	R	S	K	G	N	I
N	V	F	G	R	S	K	G	N	I
N	V	F	G	R	S	K	G	N	I
N	V	F	G	R	S	K	G	N	I
N	V	F	G	R	S	K	G	N	I
N	V	F	G	R	S	K	G	N	I
N	V	F	G	R	S	K	G	N	I
N	V	F	G	R	S	K	G	N	I
N	V	F	G	R	S	K	G	N	I
N	V	F	G	R	S	K	G	N	I
N	V	F	G	R	S	K	G	N	I
N	V	F	G	R	S	K	G	N	I
N	V	F	G	R	S	K	G	N	I
N	A	F	G	R	S	K	G	N	I
N	V	F	G	R	S	K	G	N	I
N	V	F	G	R	S	K	G	N	I
N	V	F	G	R	S	K	G	N	I
N	V	F	G	R	S	K	G	N	I
N	V	F	G	R	S	K	G	N	I
N	V	F	G	R	S	K	G	N	I
N	V	F	G	R	S	K	G	N	I
N	V	F	G	R	S	K	G	N	I
N	V	F	G	R	S	K	G	N	I
N	V	F	G	R	S	K	G	N	I
N	V	F	G	R	S	K	G	N	I
N	V	F	G	R	S	K	G	N	I
N	V	F	G	R	S	K	G	N	I
N	V	F	G	R	S	K	G	N	I
N	V	F	G	R	S	K	G	N	I

155	175	178	190	239	240	264	268	270	308	323	352	427	430	431	449	451
E	C	V	H	R	E	I	V	A	Q	A	R	A	N	G	F	R
E	Y	I	H	R	K	I	V	A	Q	A	C	A	D	D	L	K
D	D	V	R	C	E	V	I	S	H	D	H	V	D	G	L	K
E	C	V	H	R	E	I	V	A	Q	A	R	A	N	G	F	R

wciN

wc		
5	6	23
R	I	A
K	L	P
R	I	A

270	308	323	341	343	352	430	449	451
A	Q	A	V	D	R	N	F	R
S	H	E	V	E	H	D	L	K
A	Q	A	V	D	R	N	F	R
A	Q	A	V	D	R	N	F	R
A	Q	A	V	D	R	N	F	R
A	Q	A	V	D	R	N	F	R
A	Q	A	V	D	R	N	F	R
A	Q	A	V	D	R	N	F	R
A	Q	A	V	D	R	N	F	R
A	Q	A	V	D	R	N	F	R
A	Q	A	V	D	R	N	F	R
A	Q	A	V	D	R	N	F	R
A	Q	A	V	D	R	N	F	R
A	Q	A	V	D	R	N	F	R
A	Q	A	V	D	R	N	F	R
A	Q	A	V	D	R	N	F	R
A	Q	A	V	D	R	N	F	R
A	Q	A	V	D	R	N	F	R
A	Q	A	V	D	R	N	F	R
A	Q	A	V	D	R	N	F	R
A	Q	A	V	D	R	N	F	R
A	Q	A	V	D	R	N	F	R
A	Q	A	V	D	R	N	F	R
A	Q	A	V	D	R	N	F	R
A	Q	A	V	D	R	N	F	R
A	Q	A	A	D	R	N	F	R
A	Q	A	V	D	R	N	F	R

2	52	115	121	168	247	251	257	259	263	264	265	271
N	S	F	G	R	S	K	G	N	I	D	Q	E
D	S	L	D	K	C	L	N	E	V	E	I	R
N	S	F	G	R	S	K	G	N	I	D	Q	E
N	S	F	G	R	S	K	G	N	I	D	Q	E
N	S	F	G	R	S	K	G	N	I	D	Q	E
N	S	F	G	R	S	K	G	N	I	D	Q	E
N	S	F	G	R	S	K	G	N	I	D	Q	E
N	S	F	G	R	S	K	G	N	I	D	Q	E
N	S	F	G	R	S	K	G	N	I	D	Q	E
N	S	F	G	R	S	K	G	N	I	D	Q	E
N	S	F	G	R	S	K	G	N	I	D	Q	E
N	S	F	G	R	S	K	G	N	I	D	Q	E
N	S	F	G	R	S	K	G	N	I	D	Q	E
N	S	F	G	R	S	K	G	N	I	D	Q	E
N	S	F	G	R	S	K	G	N	I	D	Q	E
N	S	F	G	R	S	K	G	N	I	D	Q	E
N	S	F	G	R	S	K	G	N	I	D	Q	E
N	S	F	G	R	S	K	G	N	I	D	Q	E
N	S	F	G	R	S	K	G	N	I	D	Q	E
N	S	F	G	R	S	K	G	N	I	D	Q	E
N	S	F	G	R	S	K	G	N	I	D	Q	E
N	S	F	G	R	S	K	G	N	I	D	Q	E
N	S	F	G	R	S	K	G	N	I	D	Q	E
N	A	F	G	R	S	K	G	N	I	D	Q	E
N	A	F	G	R	S	K	G	N	I	D	Q	E
N	A	F	G	R	S	K	G	N	I	D	Q	E
N	A	F	G	R	S	K	G	N	I	D	Q	E

107	108	109	110	111	113	116	117	118	120	124	125	126	127	129	131	140	142	143	144	150	152	155
H	A	L	L	V	V	L	F	I	W	R	A	Y	P	F	G	A	S	R	V	R	I	N
H	A	L	L	V	V	L	F	I	W	R	A	Y	P	F	G	A	S	R	V	G	I	N
E	G	I	S	L	L	F	L	V	K	H	V	F	F	L	N	V	K	N	M	K	L	D
H	A	L	L	V	V	L	F	I	W	R	A	Y	P	F	G	A	S	R	V	R	I	N
H	A	L	L	V	V	L	F	I	W	R	A	Y	P	F	G	A	S	R	V	R	I	N
H	A	L	L	V	V	L	F	I	W	R	A	Y	P	F	G	A	S	R	V	R	I	N
H	A	L	L	V	V	L	F	I	W	R	A	Y	P	F	G	A	S	R	V	R	I	N
H	A	L	L	V	V	L	F	I	W	R	A	Y	P	F	G	A	S	R	V	R	I	N
H	A	L	L	V	V	L	F	I	W	R	A	Y	P	F	G	A	S	R	V	R	I	N
H	A	L	L	V	V	L	F	I	W	R	A	Y	P	F	G	A	S	R	V	R	I	N
H	A	L	L	V	V	L	F	I	W	R	A	Y	P	F	G	A	S	R	V	R	I	N
H	A	L	L	V	V	L	F	I	W	R	A	Y	P	F	G	A	S	R	V	R	I	N
H	A	L	L	V	V	L	F	I	W	R	A	Y	P	F	G	A	S	R	V	R	I	N
H	A	L	L	V	V	L	F	I	W	R	A	Y	P	F	G	A	S	R	V	R	I	N
H	A	L	L	V	V	L	F	I	W	R	A	Y	P	F	G	A	S	R	V	R	I	N
H	A	L	L	V	V	L	F	I	W	R	A	Y	P	F	G	A	S	R	V	R	I	N
H	A	L	L	V	V	L	F	I	W	R	A	Y	P	F	G	A	S	R	V	R	I	N
H	A	L	L	V	V	L	F	I	W	R	A	Y	P	F	G	A	S	R	V	R	I	N
H	A	L	L	V	V	L	F	I	W	R	A	Y	P	F	G	A	S	R	V	R	I	N
H	A	L	L	V	V	L	F	I	W	R	A	Y	P	F	G	A	S	R	V	R	I	N
H	A	L	L	V	V	L	F	I	W	R	A	Y	P	F	G	A	S	R	V	R	I	N
H	A	L	L	V	V	L	F	I	W	R	A	Y	P	F	G	A	S	R	V	R	I	N
H	A	L	L	V	V	L	F	I	W	R	A	Y	P	F	G	A	S	R	V	R	I	N
H	A	L	L	V	V	L	F	I	W	R	A	Y	P	F	G	A	S	R	V	R	I	N
H	A	L	L	V	V	L	F	I	W	R	A	Y	P	F	G	A	S	R	V	R	I	N
H	A	L	L	V	V	L	F	I	W	R	A	Y	P	F	G	A	S	R	V	R	I	N
H	A	L	L	V	V	L	F	I	W	R	A	Y	P	F	G	A	S	R	V	R	I	N
H	A	L	L	V	V	L	F	I	W	R	A	Y	P	F	G	A	S	R	V	R	I	N
H	A	L	L	V	V	L	F	I	W	R	A	Y	P	F	G	A	S	R	V	R	I	N

H	A	L	L	V	V	L	F	I	W	R	A	Y	P	F	G	A	S	R	V	R	I	N
H	A	L	L	V	V	L	F	I	W	R	A	Y	P	F	G	A	S	R	V	R	I	N

wchA																						
115	124	138	143	154	155	175	219	230	231	233	234	239	241	255	264	268	270	308	323	341	343	352
L	A	T	V	N	E	C	I	R	S	A	R	R	M	K	I	V	A	Q	A	V	D	R
L	A	T	V	N	E	C	I	R	S	A	R	R	M	K	I	V	A	Q	A	V	D	R
L	A	T	I	N	E	Y	I	R	S	A	R	R	M	K	I	V	A	Q	A	V	D	R
L	A	T	I	N	E	Y	I	R	S	A	R	R	M	K	I	V	A	Q	A	V	D	R
L	A	T	I	N	E	Y	I	R	S	A	R	R	M	K	I	V	A	Q	A	V	D	R
L	A	T	I	N	E	Y	I	R	S	A	R	R	M	K	I	V	A	Q	A	V	D	R
L	A	T	I	N	E	Y	I	R	S	A	R	R	M	K	I	V	A	Q	A	V	D	R
L	A	T	I	N	E	Y	I	R	S	A	R	R	M	K	I	V	A	Q	A	V	D	R
L	A	T	I	N	E	Y	I	R	S	A	R	R	M	K	I	V	A	Q	A	V	D	R
L	A	T	I	N	E	Y	I	R	S	A	R	R	M	K	I	V	A	Q	A	V	D	R
L	A	T	I	N	E	Y	I	R	S	A	R	R	M	K	I	V	A	Q	A	V	D	R
L	A	T	I	N	E	Y	I	R	S	A	R	R	M	K	I	V	A	Q	A	V	D	R
L	A	T	I	N	E	Y	I	R	S	A	R	R	M	K	I	V	A	Q	A	V	D	R
L	A	T	I	N	E	Y	I	R	S	A	R	R	M	K	I	V	A	Q	A	V	D	R
L	A	T	I	N	E	Y	I	R	S	A	R	R	M	K	I	V	A	Q	A	V	D	R
L	A	T	I	N	E	Y	I	R	S	A	R	R	M	K	I	V	A	Q	A	V	D	R
L	A	T	I	N	E	Y	I	R	S	A	R	R	M	K	I	V	A	Q	A	V	D	R
L	A	T	I	N	E	Y	I	R	S	A	R	R	M	K	I	V	A	Q	A	V	D	R
L	A	T	I	N	E	Y	I	R	S	A	R	R	M	K	I	V	A	Q	A	V	D	R
L	A	T	I	N	E	Y	I	R	S	A	R	R	M	K	I	V	A	Q	A	V	D	R
L	A	T	I	N	E	Y	I	R	S	A	R	R	M	K	I	V	A	Q	A	V	D	R
L	A	T	I	N	E	Y	I	R	S	A	R	R	M	K	I	V	A	Q	A	V	D	R
L	A	T	I	N	E	Y	I	R	S	A	R	R	M	K	I	V	A	Q	A	V	D	R
L	A	T	I	N	E	Y	I	R	S	A	R	R	M	K	I	V	A	Q	A	V	D	R
L	A	T	I	N	E	Y	I	R	S	A	R	R	M	K	I	V	A	Q	A	V	D	R
L	A	T	I	N	E	Y	I	R	S	A	R	R	M	K	I	V	A	Q	A	V	D	R
L	A	T	I	N	E	Y	I	R	S	A	R	R	M	K	I	V	A	Q	A	V	D	R
L	A	T	I	N	E	Y	I	R	S	A	R	R	M	K	I	V	A	Q	A	V	D	R
L	A	T	I	N	E	Y	I	R	S	A	R	R	M	K	I	V	A	Q	A	V	D	R
L	A	T	I	N	E	Y	I	R	S	A	R	R	M	K	I	V	A	Q	A	V	D	R
L	A	T	I	N	E	Y	I	R	S	A	R	R	M	K	I	V	A	Q	A	V	D	R
L	A	T	I	N	E	Y	I	R	S	A	R	R	M	K	I	V	A	Q	A	V	D	R
L	A	T	I	N	E	Y	I	R	S	A	R	R	M	K	I	V	A	Q	A	V	D	R
L	A	T	I	N	E	Y	I	R	S	A	R	R	M	K	I	V	A	Q	A	V	D	R
L	A	T	I	N	E	Y	I	R	S	A	R	R	M	K	I	V	A	Q	A	V	D	R
L	A	T	I	N	E	Y	I	R	S	A	R	R	M	K	I	V	A	Q	A	V	D	R
L	A	T	I	N	E	Y	I	R	S	A	R	R	M	K	I	V	A	Q	A	V	D	R
L	A	T	I	N	E	Y	I	R	S	A	R	R	M	K	I	V	A	Q	A	V	D	R
L	A	T	I	N	E	Y	I	R	S	A	R	R	M	K	I	V	A	Q	A	V	D	R
L	A	T	I	N	E	Y	I	R	S	A	R	R	M	K	I	V	A	Q	A	V	D	R
L	A	T	I	N	E	Y	I	R	S	A	R	R	M	K	I	V	A	Q	A	V	D	R
L	A	T	I	N	E	Y	I	R	S	A	R	R	M	K	I	V	A	Q	A	V	D	R
L	A	T	I	N	E	Y	I	R	S	A	R	R	M	K	I	V	A	Q	A	V	D	R
L	A	T	I	N	E	Y	I	R	S	A	R	R	M	K	I	V	A	Q	A	V	D	R
L	A	T	I	N	E	Y	I	R	S	A	R	R	M	K	I	V	A	Q	A	V	D	R
L	A	T	I	N	E	Y	I	R	S	A	R	R	M	K	I	V	A	Q	A	V	D	R
L	A	T	I	N	E	Y	I	R	S	A	R	R	M	K	I	V	A	Q	A	V	D	R
L	A	T	I	N	E	Y	I	R	S	A	R	R	M	K	I	V	A	Q	A	V	D	R
L	A	T	I	N	E	Y	I	R	S	A	R	R	M	K	I	V	A	Q	A	V	D	R
L	A	T	I	N	E	Y	I	R	S	A	R	R	M	K	I	V	A	Q	A	V	D	R
L	A	T	I	N	E	Y	I	R	S	A	R	R	M	K	I	V	A	Q	A	V	D	R
L	A	T	I	N	E	Y	I	R	S	A	R	R	M	K	I	V	A	Q	A	V	D	R
L	A	T	I	N	E	Y	I	R	S	A	R	R	M	K	I	V	A	Q	A	V	D	R
L	A	T	I	N	E	Y	I	R	S	A	R	R	M	K	I	V	A	Q	A	V	D	R
L	A	T	V	N	E	C	I	R	S	A	R	R	M	K	I	V	A	Q	A	V	D	R
L	A	T	I	N	E	Y	I	R	S	A	R	R	M	K	I	V	A	Q	A	V	D	R
L	A	T	I	N	E	Y	I	R	S	A	R	R	M	K	I	V	A	Q	A	V	D	R
L	A	T	I	N	E	Y	I	R	S	A	R	R	M	K	I	V	A	Q	A	V	D	R
L	A	T	I	N	E	Y	I	R	S	A	R	R	M	K	I	V	A	Q	A	V	D	R
L	A	T	I	N	E	Y	I	R	S	A	R	R	M	K	I	V	A	Q	A	V	D	R
L	S	T	V	N	E	C	I	R	S	A	R	R	M	K	I	V	A	Q	A	V	D	R
L	A	T	V	N	E	C	I	R	S	A	R	R	M	K	I	V	A	Q	A	V	D	R
L	S	T	V	N	E	C	I	R	S	A	R	R	M	K	I	V	A	Q	A	V	D	R
L	S	T	V	N	E	C	I	R	S	A	R	R	M	K	I	V	A	Q	A	V	D	R

L	A	T	V	N	E	C	I	R	S	A	R	R	M	K	I	V	A	Q	A	V	D	R
L	A	T	V	N	E	C	I	R	S	A	R	R	M	K	I	V	A	Q	A	V	D	R
L	A	T	V	N	E	C	I	R	S	A	R	R	M	K	I	V	A	Q	A	V	D	R
L	A	T	V	N	E	C	I	R	S	A	R	R	M	K	I	V	A	Q	A	V	D	R
L	A	T	V	N	E	C	I	R	S	A	R	R	M	K	I	V	A	Q	A	V	D	R
L	A	T	V	N	E	C	I	R	S	A	R	R	M	K	I	V	A	Q	A	V	D	R
L	S	T	V	N	E	C	I	R	S	A	R	R	M	K	I	V	A	Q	A	V	D	R
L	A	T	V	N	E	C	I	R	S	A	R	R	M	K	I	V	A	Q	A	V	D	R
L	S	T	V	N	E	C	I	R	S	A	R	R	M	K	I	V	A	Q	A	V	D	R
L	A	T	V	N	E	C	I	R	S	A	R	R	M	K	I	V	A	Q	A	V	D	R
L	A	T	V	N	E	C	I	R	S	A	R	R	M	K	I	V	A	Q	A	V	D	R
L	A	T	V	N	E	C	I	R	S	A	R	R	M	K	I	V	A	Q	A	V	D	R
L	A	T	V	N	E	C	I	R	S	A	R	R	M	K	I	V	A	Q	A	V	D	R
L	A	T	V	N	E	C	I	R	S	A	R	R	M	K	I	V	A	Q	A	V	D	R
L	A	T	V	N	E	C	I	R	S	A	R	R	M	K	I	V	A	Q	A	V	D	R
L	A	T	V	N	E	C	I	R	S	A	R	R	M	K	I	V	A	Q	A	V	D	R
L	S	T	V	N	E	C	I	R	S	A	R	R	M	K	I	V	A	Q	A	V	D	R
L	A	T	V	N	E	C	I	R	S	A	R	R	M	K	I	V	A	Q	A	V	D	R
L	A	T	V	N	E	C	I	R	S	A	R	R	M	K	I	V	A	Q	A	V	D	R
L	S	T	V	N	E	C	I	R	S	A	R	R	M	K	I	V	A	Q	A	V	D	R
L	A	T	V	N	E	C	I	R	S	A	R	R	M	K	I	V	A	Q	A	V	D	R
L	A	T	I	N	E	Y	I	R	S	A	R	R	M	K	I	V	A	Q	A	V	D	R
L	A	T	I	N	E	Y	I	R	S	A	R	R	M	R	I	V	A	Q	A	V	D	R
L	A	T	I	N	E	Y	I	R	S	A	R	R	M	K	I	V	A	Q	A	V	D	R
L	A	A	V	N	E	C	I	R	S	A	R	R	M	K	I	V	A	Q	A	V	D	R
L	A	A	V	N	E	C	I	R	S	A	R	R	M	K	I	V	A	Q	A	V	D	R
L	A	A	V	N	E	C	I	R	S	A	R	R	M	K	I	V	A	Q	A	V	D	R
L	A	T	V	N	E	C	I	R	S	A	R	R	M	K	I	V	A	Q	A	V	D	R
L	A	T	V	N	E	C	I	R	S	A	R	R	M	K	I	V	A	Q	A	V	D	R
L	A	T	V	N	E	C	I	R	S	A	R	R	M	K	I	V	A	Q	A	V	D	R
L	A	T	V	N	E	C	I	R	S	A	R	R	M	K	I	V	A	Q	A	V	D	R
L	A	T	V	N	E	C	I	R	S	A	R	R	M	K	I	V	A	Q	A	V	D	R
L	A	T	V	N	E	C	I	R	S	A	R	R	M	K	I	V	A	Q	A	V	D	R
L	A	T	V	N	E	C	I	R	S	A	R	R	M	K	I	V	A	Q	A	V	D	R
L	A	T	V	N	E	C	I	R	S	A	R	R	M	K	I	V	A	Q	A	V	D	R
L	A	T	V	N	E	C	I	R	S	A	R	R	M	K	I	V	A	Q	A	V	D	R
L	A	T	V	N	E	C	I	R	S	A	R	R	M	K	I	V	A	Q	A	V	D	R
L	A	T	V	N	E	C	I	R	S	A	R	R	M	K	I	V	A	Q	A	V	D	R
L	A	T	V	N	E	C	I	R	S	A	R	R	M	K	I	V	A	Q	A	V	D	R
L	A	T	V	N	E	C	I	R	S	A	R	R	M	K	I	V	A	Q	A	V	D	R
L	A	T	V	N	E	C	I	R	S	A	R	R	M	K	I	V	A	Q	A	V	D	R
L	A	T	V	N	E	C	I	R	S	A	R	R	M	K	I	V	A	Q	A	V	D	R
L	A	T	V	N	E	C	I	R	S	A	R	R	M	K	I	V	A	Q	A	V	D	R
L	A	T	V	N	E	C	I	R	S	A	R	R	M	K	I	V	A	Q	A	V	D	R
L	A	T	V	N	E	C	I	R	S	A	R	R	M	K	I	V	A	Q	A	V	D	R
L	A	T	V	N	E	C	I	R	S	A	R	R	M	K	I	V	A	Q	A	V	D	R
L	A	T	V	N	E	C	I	R	S	A	R	R	M	K	I	V	A	Q	A	V	D	R
L	A	T	V	N	E	C	I	R	S	A	R	R	M	K	I	V	A	Q	A	V	D	R
L	A	T	V	N	E	C	I	R	S	A	R	R	M	K	I	V	A	Q	A	V	D	R
L	A	T	V	N	E	C	I	R	S	A	R	R	M	K	I	V	A	Q	A	V	D	R
L	A	T	V	N	E	C	I	R	S	A	R	R	M	K	I	V	A	Q	A	V	D	R
L	A	T	V	N	E	C	I	R	S	A	R	R	M	K	I	V	A	Q	A	V	D	R
L	A	T	V	N	E	C	I	R	S	A	R	R	M	K	I	V	A	Q	A	V	D	R
L	A	T	V	N	E	C	I	R	S	A	R	R	M	K	I	V	A	Q	A	V	D	R
L	A	T	V	N	E	C	I	R	S	A	R	R	M	K	I	V	A	Q	A	V	D	R
L	A	T	V	N	E	C	I	R	S	A	R	R	M	K	I	V	A	Q	A	V	D	R
L	A	T	V	N	E	C	I	R	S	A	R	R	M	K	I	V	A	Q	A	V	D	R
L	A	T	V	N	E	C	I	R	S	A	R	R	M	K	I	V	A	Q	A	V	D	R
L	A	T	V	N	E	C	I	R	S	A	R	R	M	K	I	V	A	Q	A	V	D	R
L	A	T	V	N	E	C	I	R	S	A	R	R	M	K	I	V	A	Q	A	V	D	R
L	A	T	V	N	E	C	I	R	S	A	R	R	M	K	I	V	A	Q	A	V	D	R
L	A	T	V	N	E	C	I	R	S	A	R	R	M	K	I	V	A	Q	A	V	D	R
L	A	T	V	N	E	C	I	R	S	A	R	R	M	K	I	V	A	Q	A	V	D	R
L	A	T	V	N	E	C	I	R	S	A	R	R	M	K	I	V	A	Q	A	V	D	R
L	A	T	V	N	E	C	I	R	S	A	R	R	M	K	I	V	A	Q	A	V	D	R
L	A	T	V	N	E	C	I	R	S	A	R	R	M	K	I	V	A	Q	A	V	D	R
L	A	T	V	N	E	C	I	R	S	A	R	R	M	K	I	V	A	Q	A	V	D	R

L	A	T	V	N	E	C	I	R	S	A	R	R	M	K	I	V	A	Q	A	V	D	R
L	A	T	V	N	E	C	I	R	S	A	R	R	M	K	I	V	A	Q	A	V	D	R
L	A	T	V	N	E	C	I	R	S	A	R	R	M	K	I	V	A	Q	A	V	D	R
L	A	T	V	N	E	C	I	R	S	A	R	R	M	K	I	V	A	Q	A	V	D	R
L	A	T	V	N	E	C	I	R	S	A	R	R	M	K	I	V	A	Q	A	V	D	R
L	A	T	V	N	E	C	I	R	S	A	R	R	M	K	I	V	A	Q	A	V	D	R
L	A	T	V	N	E	C	I	R	S	A	R	R	M	K	I	V	A	Q	A	V	D	R
L	A	T	V	N	E	C	I	R	S	A	R	R	M	K	I	V	A	Q	A	V	D	R
L	A	T	V	N	E	C	I	R	S	A	R	R	M	K	I	V	A	Q	A	V	D	R
L	A	T	V	N	E	C	I	R	S	A	R	R	M	K	I	V	A	Q	A	V	D	R
L	A	T	V	N	E	C	I	R	S	A	R	R	M	K	I	V	A	Q	A	V	D	R
L	A	T	V	N	E	C	I	R	S	A	R	R	M	K	I	V	A	Q	A	V	D	R
L	A	T	V	N	E	C	I	R	S	A	R	R	M	K	I	V	A	Q	A	V	D	R
L	A	T	V	N	E	C	I	R	S	A	R	R	M	K	I	V	A	Q	A	V	D	R
L	A	T	V	N	E	C	I	R	S	A	R	R	M	K	I	V	A	Q	A	V	D	R
L	A	T	V	N	E	C	I	R	S	A	R	R	M	K	I	V	A	Q	A	V	D	R
L	A	T	V	N	E	C	I	R	S	A	R	R	M	K	I	V	A	Q	A	V	D	R
L	A	T	V	N	E	C	I	R	S	A	R	R	M	K	I	V	A	Q	A	V	D	R
L	A	T	V	N	E	C	I	R	S	A	R	R	M	K	I	V	A	Q	A	V	D	R
L	A	T	V	N	E	C	I	R	S	A	R	R	M	K	I	V	A	Q	A	V	D	R
L	A	T	V	N	E	C	I	R	S	A	R	R	M	K	I	V	A	Q	A	V	D	R
L	A	T	V	N	E	C	I	R	S	A	R	R	M	K	I	V	A	Q	A	V	D	R
L	A	T	V	N	E	C	I	R	S	A	R	R	M	K	I	V	A	Q	A	V	D	R
L	A	T	V	N	E	C	I	R	S	A	R	R	M	K	I	V	A	Q	A	V	D	R
L	A	T	V	N	E	C	I	R	S	A	R	R	M	K	I	V	A	Q	A	V	D	R
L	A	T	V	N	E	C	I	R	S	A	R	R	M	K	I	V	A	Q	A	V	D	R
L	A	T	V	N	E	C	I	R	S	A	R	R	M	K	I	V	A	Q	A	V	D	R
L	A	T	V	N	E	C	I	R	S	A	R	R	M	K	I	V	A	Q	A	V	D	R
L	A	T	V	N	E	C	I	R	S	A	R	R	M	K	I	V	A	Q	A	V	D	R
L	A	T	V	N	E	C	I	R	S	A	R	R	M	K	I	V	A	Q	A	V	D	R
L	A	T	V	N	E	C	I	R	S	A	R	R	M	K	I	V	A	Q	A	V	D	R
L	A	T	V	N	E	C	I	R	S	A	R	R	M	K	I	V	A	Q	A	V	D	R
L	A	T	V	N	E	C	I	R	S	A	R	R	M	K	I	V	A	Q	A	V	D	R
L	A	T	V	N	E	C	I	R	S	A	R	R	M	K	I	V	A	Q	A	V	D	R
L	A	T	V	N	E	C	I	R	S	A	R	R	M	K	I	V	A	Q	A	V	D	R
L	A	T	V	N	E	C	I	R	S	A	R	R	M	K	I	V	A	Q	A	V	D	R
L	A	T	V	N	E	C	I	R	S	A	R	R	M	K	I	V	A	Q	A	V	D	R
L	A	T	V	N	E	C	I	R	S	A	R	R	M	K	I	V	A	Q	A	V	D	R
L	A	T	V	N	E	C	I	R	S	A	R	R	M	K	I	V	A	Q	A	V	D	R
L	A	T	V	N	E	C	I	R	S	A	R	R	M	K	I	V	A	Q	A	V	D	R
L	A	T	V	N	E	C	I	R	S	V	R	R	M	K	I	V	A	Q	A	V	D	R
L	A	T	V	N	E	C	I	R	S	A	R	R	M	K	I	V	A	Q	A	V	D	R
L	A	T	V	N	E	C	I	R	S	A	R	R	M	K	I	V	A	Q	A	V	D	R
L	A	T	V	N	E	C	I	R	S	A	R	R	M	K	I	V	A	Q	A	V	D	R
L	A	T	V	N	E	C	I	R	S	A	R	R	M	K	I	V	A	Q	A	V	D	R
L	A	T	V	N	E	C	I	R	S	A	R	R	M	K	I	V	A	Q	A	V	D	R
L	A	T	V	N	E	C	I	R	S	A	R	R	M	K	I	V	A	Q	A	V	D	R
L	A	T	V	N	E	C	I	R	S	A	R	R	M	K	I	V	A	Q	A	V	D	R
L	A	T	V	N	E	C	I	R	S	A	R	R	M	K	I	V	A	Q	A	V	D	R
L	A	T	V	N	E	C	I	R	S	A	R	R	M	K	I	V	A	Q	A	V	D	R
L	A	T	V	N	E	C	I	R	S	A	R	R	M	K	I	V	A	Q	A	V	D	R
L	A	T	V	N	E	C	I	R	S	A	R	R	M	K	I	V	A	Q	A	V	D	R
L	A	T	V	N	E	C	I	R	S	A	R	R	M	K	I	V	A	Q	A	V	D	R
L	A	T	V	N	E	C	I	R	S	A	R	R	M	K	I	V	A	Q	A	V	D	R
L	A	T	V	N	E	C	I	R	S	A	R	R	M	K	I	V	A	Q	A	V	D	R
L	A	T	V	N	E	C	I	R	S	A	R	R	M	K	I	V	A	Q	A	V	D	R
L	A	T	V	N	E	C	I	R	S	A	R	R	M	K	I	V	A	Q	A	V	D	R
L	A	T	V	N	E	C	I	R	S	A	R	R	M	K	I	V	A	Q	A	V	D	R
L	A	T	V	N	E	C	I	R	S	A	R	R	M	K	I	V	A	Q	A	V	D	R
L	A	T	V	N	E	C	I	R	S	A	R	R	M	K	I	V	A	Q	A	V	D	R
L	A	T	V	N	E	C	I	R	S	A	R	R	M	K	I	V	A	Q	A	V	D	R
L	A	T	V	N	E	C	I	R	S	A	R	R	M	K	I	V	A	Q	A	V	D	R
L	A	T	V	N	E	C	I	R	S	A	R	R	M	K	I	V	A	Q	A	V	D	R
L	A	T	V	N	E	C	I	R	S	A	R	R	M	K	I	V	A	Q	A	V	D	R
L	A	T	V	N	E	C	I	R	S	A	R	R	M	K	I	V	A	Q	A	V	D	R
L	A	T	V	N	E	C	I	R	S	A	R	R	M	K	I	V	A	Q	A	V	D	R
L	A	T	V	N	E	C	I	R	S	A	R	R	M	K	I	V	A	Q	A	V	D	R

L	A	T	V	N	E	C	I	R	S	A	R	R	M	K	I	V	A	Q	A	V	D	R
L	A	T	V	N	E	C	I	R	S	A	R	R	M	K	I	V	A	Q	A	V	D	R
L	A	T	V	N	E	C	I	R	S	A	R	R	M	K	I	V	A	Q	A	V	D	R
L	A	T	V	N	E	C	I	R	S	A	R	R	M	K	I	V	A	Q	A	V	D	R
L	A	T	I	N	E	Y	I	R	S	A	R	R	M	K	I	V	A	Q	A	V	D	R
L	A	T	I	N	E	Y	I	R	S	A	R	R	M	K	I	V	A	Q	A	V	D	R
L	A	T	I	N	E	Y	I	R	S	A	R	R	M	K	I	V	A	Q	A	V	D	R
L	A	T	I	N	E	Y	I	R	S	A	R	R	M	K	I	V	A	Q	A	V	D	R
L	A	T	I	N	E	Y	I	R	S	A	R	R	M	K	I	V	A	Q	A	V	D	R
L	A	T	I	N	E	Y	I	R	S	A	R	R	M	K	I	V	A	Q	A	V	D	R
L	A	T	I	N	E	Y	I	R	S	A	R	R	M	K	I	V	A	Q	A	V	D	R
L	A	T	I	N	E	Y	I	R	S	A	R	R	M	K	I	V	A	Q	A	V	D	R
L	A	T	I	N	E	Y	I	R	S	A	R	R	M	K	I	V	A	Q	A	V	D	R
L	A	T	I	N	E	Y	I	R	S	A	R	R	M	K	I	V	A	Q	A	V	D	R
L	A	T	I	N	E	Y	I	R	S	A	R	R	M	K	I	V	A	Q	A	V	D	R
L	A	T	I	N	E	Y	I	R	S	A	R	R	M	K	I	V	A	Q	A	V	D	R
L	A	T	I	N	E	Y	I	R	S	A	R	R	M	K	I	V	A	Q	A	V	D	R
L	A	T	I	N	E	Y	I	R	S	A	R	R	M	K	I	V	A	Q	A	V	D	R
L	A	T	I	N	E	Y	I	R	S	A	R	R	M	K	I	V	A	Q	A	V	D	R
L	A	T	I	N	E	Y	I	R	S	A	R	R	M	K	I	V	A	Q	A	V	D	R
L	A	T	I	N	E	Y	I	R	S	A	R	R	M	K	I	V	A	Q	A	V	D	R
L	A	T	I	N	E	Y	I	R	S	A	R	R	M	K	I	V	A	Q	A	V	D	R
L	A	T	I	N	E	Y	I	R	S	A	R	R	M	K	I	V	A	Q	A	V	D	R
L	A	T	I	N	E	Y	I	R	S	A	R	R	M	K	I	V	A	Q	A	V	D	R
L	A	T	I	N	E	Y	I	R	S	A	R	R	M	K	I	V	A	Q	A	V	D	R
L	A	T	I	N	E	Y	I	R	S	A	R	R	M	K	I	V	A	Q	A	V	D	R
L	A	T	I	N	E	Y	I	R	S	A	R	R	M	K	I	V	A	Q	A	V	D	R
L	A	T	I	N	E	Y	I	R	S	A	R	R	M	K	I	V	A	Q	A	V	D	R
L	A	T	I	N	E	Y	I	R	S	A	R	R	M	K	I	V	A	Q	A	V	D	R
L	A	T	I	N	E	Y	I	R	S	A	R	R	M	K	I	V	A	Q	A	V	D	R
L	A	T	I	N	E	Y	I	R	S	A	R	R	M	K	I	V	A	Q	A	V	D	R
L	A	T	I	N	E	Y	I	R	S	A	R	R	M	K	I	V	A	Q	A	V	D	R
L	A	T	I	N	E	Y	I	R	S	A	R	R	M	K	I	V	A	Q	A	V	D	R
L	A	T	I	N	E	Y	I	R	S	A	R	R	M	K	I	V	A	Q	A	V	D	R
L	A	T	I	N	E	Y	I	R	S	A	R	R	M	K	I	V	A	Q	A	V	D	R
L	A	T	I	N	E	Y	I	R	S	A	R	R	M	K	I	V	A	Q	A	V	D	R
L	A	T	I	N	E	Y	I	R	S	A	R	R	M	K	I	V	A	Q	A	V	D	R
L	A	T	I	N	E	Y	I	R	S	A	R	R	M	K	I	V	A	Q	A	V	D	R
L	A	T	I	N	E	Y	I	R	S	A	R	R	M	K	I	V	A	Q	A	V	D	R
L	A	T	I	N	E	Y	I	R	S	A	R	R	M	K	I	V	A	Q	A	V	D	R
L	A	T	I	N	E	Y	I	R	S	A	R	R	M	K	I	V	A	Q	A	V	D	R
L	A	T	I	N	E	Y	I	R	S	A	R	R	M	K	I	V	A	Q	A	V	D	R
L	A	T	I	N	E	Y	I	R	S	A	R	R	M	K	I	V	A	Q	A	V	D	R
L	A	T	I	N	E	Y	I	R	S	A	R	R	M	K	I	V	A	Q	A	V	D	R
L	A	T	I	N	E	Y	I	R	S	A	R	R	M	K	I	V	A	Q	A	V	D	R
L	A	T	I	N	E	Y	I	R	S	A	R	R	M	K	I	V	A	Q	A	V	D	R
L	A	T	I	N	E	Y	I	R	S	A	R	R	M	K	I	V	A	Q	A	V	D	R
L	A	T	I	N	E	Y	I	R	S	A	R	R	M	K	I	V	A	Q	A	V	D	R
L	A	T	I	N	E	Y	I	R	S	A	R	R	M	K	I	V	A	Q	A	V	D	R
L	A	T	I	N	E	Y	I	R	S	A	R	R	M	K	I	V	A	Q	A	V	D	R
L	A	T	I	N	E	Y	I	R	S	A	R	R	M	K	I	V	A	Q	A	V	D	R
L	A	T	I	N	E	Y	I	R	S	A	R	R	M	K	I	V	A	Q	A	V	D	R
L	A	T	I	N	E	Y	I	R	S	A	R	R	M	K	I	V	A	Q	A	V	D	R
L	A	T	I	N	E	Y	I	R	S	A	R	R	M	K	I	V	A	Q	A	V	D	R
L	A	T	I	N	E	Y	I	R	S	A	R	R	M	K	I	V	A	Q	A	V	D	R
L	A	T	I	N	E	Y	I	R	S	A	R	R	M	K	I	V	A	Q	A	V	D	R
L	A	T	I	N	E	Y	I	R	S	A	R	R	M	K	I	V	A	Q	A	V	D	R
L	A	T	I	N	E	Y	I	R	S	A	R	R	M	K	I	V	A	Q	A	V	D	R
L	A	T	I	N	E	Y	I	R	S	A	R	R	M	K	I	V	A	Q	A	V	D	R
L	A	T	I	N	E	Y	I	R	S	A	R	R	M	K	I	V	A	Q	A	V	D	R
L	A	T	I	N	E	Y	I	R	S	A	R	R	M	K	I	V	A	Q	A	V	D	R
L	A	T	I	N	E	Y	I	R	S	A	R	R	M	K	I	V	A	Q	A	V	D	R
L	A	T	I	N	E	Y	I	R	S	A	R	R	M	K	I	V	A	Q	A	V	D	R
L	A	T	I	N	E	Y	I	R	S	A	R	R	M	K	I	V	A	Q	A	V	D	R
L	A	T	I	N	E	Y	I	R	S	A	R	R	M	K	I	V	A	Q	A	V	D	R
L	A	T	I	N	E	Y	I	R	S	A	R	R	M	K	I	V	A	Q	A	V	D	R
L	A	T	I	N	E	Y	I	R	S	A	R	R	M	K	I	V	A	Q	A	V	D	R

L	A	T	I	N	E	Y	I	R	S	A	R	R	M	K	I	V	A	Q	A	V	D	R
L	A	T	I	N	E	Y	I	R	S	A	R	R	M	K	I	V	A	Q	A	V	D	R
L	A	T	I	N	E	Y	I	R	S	A	R	R	M	K	I	V	A	Q	A	V	D	R
L	A	T	I	N	E	Y	I	R	S	A	R	R	M	K	I	V	A	Q	A	V	D	R
L	A	T	I	N	E	Y	I	R	S	A	R	R	M	K	I	V	A	Q	A	V	D	R
L	A	T	I	N	E	Y	I	R	S	A	R	R	M	K	I	V	A	Q	A	V	D	R
L	A	T	I	N	E	Y	I	R	S	A	R	R	M	K	I	V	A	Q	A	V	D	R
L	A	T	I	N	E	Y	I	R	S	A	R	R	M	K	I	V	A	Q	A	V	D	R
L	V	T	I	N	E	Y	I	R	S	A	R	R	M	K	I	V	A	Q	A	V	D	R
L	A	T	I	N	E	Y	I	R	S	A	R	R	M	K	I	V	A	Q	A	V	D	R
L	A	T	I	N	E	Y	I	R	S	A	R	R	M	K	I	V	A	Q	A	V	D	R
L	A	T	V	N	E	C	I	R	S	A	R	R	M	K	I	V	A	Q	A	V	D	R
L	A	T	V	N	E	C	I	R	S	A	R	R	M	K	I	V	A	Q	A	V	D	R
L	A	T	V	N	E	C	I	R	S	A	R	R	M	K	I	V	A	Q	A	V	D	R
L	A	T	V	N	E	C	I	R	S	A	R	R	M	K	I	V	A	Q	A	V	D	R
L	A	T	V	N	E	C	I	R	S	A	R	R	M	K	I	V	A	Q	A	V	D	R
L	A	T	V	N	E	C	I	R	S	A	R	R	M	K	I	V	A	Q	A	V	D	R
L	A	T	V	N	E	C	I	R	S	A	R	R	M	K	I	V	A	Q	A	V	D	R
L	A	T	V	N	E	C	I	R	S	A	R	R	M	K	I	V	A	Q	A	V	D	R
L	A	T	V	N	E	C	I	R	S	A	R	R	M	K	I	V	A	Q	A	V	D	R
R	A	T	V	D	D	Y	V	C	I	A	H	C	M	K	V	I	S	H	E	V	E	H
R	A	T	V	D	D	Y	V	C	I	A	H	C	M	K	V	I	S	H	E	V	E	H
R	A	T	V	D	D	Y	V	C	I	A	H	C	M	K	V	I	S	H	E	V	E	H
R	A	T	V	D	D	Y	V	C	I	A	H	C	M	K	V	I	S	H	E	V	E	H
R	A	T	V	D	D	Y	V	C	I	A	H	C	M	K	V	I	S	H	E	V	E	H
R	A	T	V	D	D	Y	V	C	I	A	H	C	M	K	V	I	S	H	E	V	E	H
R	A	T	V	D	D	Y	V	C	I	A	H	C	M	K	V	I	S	H	E	V	E	H
R	A	T	V	D	D	Y	V	C	I	A	H	C	M	K	V	I	S	H	E	V	E	H
L	A	T	V	N	E	C	I	R	S	A	R	R	M	K	I	V	A	Q	A	V	D	R
L	A	T	V	N	E	C	I	R	S	A	R	R	M	K	I	V	A	Q	A	V	D	R
L	A	T	V	N	E	C	I	R	S	A	R	R	M	K	I	V	A	Q	A	V	D	R
L	A	T	V	N	E	C	I	R	S	A	R	R	M	K	I	V	A	Q	A	V	D	R
L	A	T	V	N	E	C	I	R	S	A	R	R	M	K	I	V	A	Q	A	V	D	R
L	A	T	V	N	E	C	I	R	S	A	R	R	M	K	I	V	A	Q	A	V	D	R
L	A	T	V	N	E	C	I	R	S	A	R	R	M	K	I	V	A	Q	A	V	D	R
L	A	T	V	N	E	C	I	R	S	A	R	R	M	K	I	V	A	Q	A	V	D	R
L	A	T	V	N	E	C	I	R	S	A	R	R	M	K	I	V	A	Q	A	V	D	R
L	A	T	V	N	E	C	I	R	S	A	R	R	M	K	I	V	A	Q	A	V	D	R
L	A	T	V	N	E	C	I	R	S	A	R	R	M	K	I	V	A	Q	A	V	D	R
L	A	T	V	N	E	C	I	R	S	A	R	R	M	K	I	V	A	Q	A	V	D	R
L	A	T	V	N	E	C	I	R	S	A	R	R	M	K	I	V	A	Q	A	V	D	R
L	A	T	V	N	E	C	I	R	S	A	R	R	M	K	I	V	A	Q	A	V	D	R
L	A	T	V	N	E	C	I	R	S	A	R	R	M	K	I	V	A	Q	A	V	D	R
L	A	T	V	N	E	C	I	R	S	A	R	R	M	K	I	V	A	Q	A	V	D	R
L	A	T	V	N	E	C	I	R	S	A	R	R	M	K	I	V	A	Q	A	V	D	R
L	A	T	V	N	E	C	I	R	S	A	R	R	M	K	I	V	A	Q	A	V	D	R
L	A	T	V	N	E	C	I	R	S	A	R	R	M	K	I	V	A	Q	A	V	D	R
L	A	T	V	N	E	C	I	R	S	A	R	R	M	K	I	V	A	Q	A	V	D	R
L	A	T	V	N	E	C	I	R	S	A	R	R	M	K	I	V	A	Q	A	V	D	R
L	A	T	V	N	E	C	I	R	S	A	R	R	M	K	I	V	A	Q	A	V	D	R
L	A	T	V	N	E	C	I	R	S	A	R	R	M	K	I	V	A	Q	A	V	D	R
L	A	T	V	N	E	C	I	R	S	A	R	R	M	K	I	V	A	Q	A	V	D	R
L	A	T	V	N	E	C	I	R	S	A	R	R	M	K	I	V	A	Q	A	V	D	R
L	A	T	V	N	E	C	I	R	S	A	R	R	M	K	I	V	A	Q	A	V	D	R
L	A	T	V	N	E	C	I	R	S	A	R	R	M	K	I	V	A	Q	A	V	D	R
L	A	T	V	N	E	C	I	R	S	A	R	R	M	K	I	V	A	Q	A	V	D	R
L	A	T	V	N	E	C	I	R	S	A	R	R	M	K	I	V	A	Q	A	V	D	R
L	A	T	V	N	E	C	I	R	S	A	R	R	M	K	I	V	A	Q	A	V	D	R
L	A	T	V	N	E	C	I	R	S	A	R	R	M	K	I	V	A	Q	A	V	D	R
L	A	T	V	N	E	C	I	R	S	A	R	R	M	K	I	V	A	Q	A	V	D	R
L	A	T	V	N	E	C	I	R	S	A	R	R	M	K	I	V	A	Q	A	V	D	R
L	A	T	V	N	E	C	I	R	S	A	R	R	M	K	I	V	A	Q	A	V	D	R
L	A	T	V	N	E	C	I	R	S	A	R	R	M	K	I	V	A	Q	A	V	D	R
L	A	T	V	N	E	C	I	R	S	A	R	R	M	K	I	V	A	Q	A	V	D	R
L	A	T	V	N	E	C	I	R	S	A	R	R	M	K	I	V	A	Q	A	V	D	R
L	A	T	V	N	E	C	I	R	S	A	R	R	M	K	I	V	A	Q	A	V	D	R
L	A	T	V	N	E	C	I	R	S	A	R	R	M	K	I	V	A	Q	A	V	D	R

L	A	T	V	N	E	C	I	R	S	A	R	R	M	K	I	V	A	Q	A	V	D	R
L	A	T	V	N	E	C	I	R	S	A	R	R	M	K	I	V	A	Q	A	V	D	R
L	A	T	V	N	E	C	I	R	S	A	R	R	M	K	I	V	A	Q	A	V	D	R
L	A	T	V	N	E	C	I	R	S	A	R	R	M	K	I	V	A	Q	A	V	D	R
L	A	T	V	N	E	C	I	R	S	A	R	R	M	K	I	V	A	Q	A	V	D	R
L	A	T	V	N	E	C	I	R	S	A	R	R	M	K	I	V	A	Q	A	V	D	R
L	A	T	V	N	E	C	I	R	S	A	R	R	M	K	I	V	A	Q	A	V	D	R
L	A	T	V	N	E	C	I	R	S	A	R	R	M	K	I	V	A	Q	A	V	D	R
L	A	T	V	N	E	C	I	R	S	A	R	R	M	K	I	V	A	Q	A	V	D	R
L	A	T	V	N	E	C	I	R	S	A	R	R	M	K	I	V	A	Q	A	V	D	R
L	A	T	V	N	E	C	I	R	S	A	R	R	M	K	I	V	A	Q	A	V	D	R
L	A	T	V	N	E	C	I	R	S	A	R	R	M	K	I	V	A	Q	A	V	D	R
L	A	T	V	N	E	C	I	R	S	A	R	R	M	K	I	V	A	Q	A	V	D	R
L	A	T	V	N	E	C	I	R	S	A	R	R	M	K	I	V	A	Q	A	V	D	R
L	A	T	V	N	E	C	I	R	S	A	R	R	M	K	I	V	A	Q	A	V	D	R
L	A	T	V	N	E	C	I	R	S	A	R	R	M	K	I	V	A	Q	A	V	D	R
L	A	T	V	N	E	C	I	R	S	A	R	R	M	K	I	V	A	Q	A	V	D	R
L	A	T	V	N	E	C	I	R	S	A	R	R	M	K	I	V	A	Q	A	V	D	R
L	A	T	V	N	E	C	I	R	S	A	R	R	M	K	I	V	A	Q	A	V	D	R
L	A	T	V	N	E	C	I	R	S	A	R	R	M	K	I	V	A	Q	A	V	D	R
L	A	T	V	N	E	C	I	R	S	A	R	R	M	K	I	V	A	Q	A	V	D	R
L	A	T	V	N	E	C	I	R	S	A	R	R	M	K	I	V	A	Q	A	V	D	R
L	A	T	V	N	E	C	I	R	S	A	R	R	M	K	I	V	A	Q	A	V	D	R
L	A	T	V	N	E	C	I	R	S	A	R	R	M	K	I	V	A	Q	A	V	D	R
L	A	T	V	N	E	C	I	R	S	A	R	R	M	K	I	V	A	Q	A	V	D	R
L	A	T	V	N	E	C	I	R	S	A	R	R	M	K	I	V	A	Q	A	V	D	R
L	A	T	V	N	E	C	I	R	S	A	R	R	M	K	I	V	A	Q	A	V	D	R
L	A	T	V	N	E	C	I	R	S	A	R	R	M	K	I	V	A	Q	A	V	D	R
L	A	T	V	N	E	C	I	R	S	A	R	R	M	K	I	V	A	Q	A	V	D	R
L	A	T	V	N	E	C	I	R	S	A	R	R	M	K	I	V	A	Q	A	V	D	R
L	A	T	V	N	E	C	I	R	S	A	R	R	M	K	I	V	A	Q	A	V	D	R
L	A	T	V	N	E	C	I	R	S	A	R	R	M	K	I	V	A	Q	A	V	D	R
L	A	T	V	N	E	C	I	R	S	A	R	R	M	K	I	V	A	Q	A	V	D	R
L	A	T	V	N	E	C	I	R	S	A	R	R	M	K	I	V	A	Q	A	V	D	R
L	A	T	V	N	E	C	I	R	S	A	R	R	M	K	I	V	A	Q	A	V	D	R
L	A	T	V	N	E	C	I	R	S	A	R	R	M	K	I	V	A	Q	A	V	D	R
L	A	T	V	N	E	C	I	R	S	A	R	R	M	K	I	V	A	Q	A	V	D	R
L	A	T	V	N	E	C	I	R	S	A	R	R	M	K	I	V	A	Q	A	V	D	R
L	A	T	V	N	E	C	I	R	S	A	R	R	M	K	I	V	A	Q	A	V	D	R
L	A	T	V	N	E	C	I	R	S	A	R	R	M	K	I	V	A	Q	A	V	D	R
L	A	T	I	N	E	Y	I	R	S	A	R	R	M	K	I	V	A	Q	A	V	D	R
L	A	T	I	N	E	Y	I	R	S	A	R	R	M	K	I	V	A	Q	A	V	D	R
L	A	T	I	N	E	Y	I	R	S	A	R	R	M	K	I	V	A	Q	A	V	D	R
L	A	T	I	N	E	Y	I	R	S	A	R	R	M	K	I	V	A	Q	A	V	D	R
L	A	T	I	N	E	Y	I	R	S	A	R	R	M	K	I	V	A	Q	A	V	D	R
L	A	T	V	N	E	C	I	R	S	A	R	R	M	K	I	V	A	Q	A	V	D	R
L	A	T	V	N	E	C	I	R	S	A	R	R	M	K	I	V	A	Q	A	V	D	R
L	A	T	V	N	E	C	I	R	S	A	R	R	M	K	I	V	A	Q	A	V	D	R
L	A	T	V	N	E	C	I	R	S	A	R	R	M	K	I	V	A	Q	A	A	D	R
L	A	T	V	N	E	C	I	R	S	A	R	R	V	K	I	V	A	Q	A	V	D	R
L	A	T	V	N	E	C	I	R	S	A	R	R	M	K	I	V	A	Q	A	V	D	R
L	A	T	V	N	E	C	I	R	S	A	R	R	M	K	I	V	A	Q	A	V	D	R
L	A	T	V	N	E	C	I	R	S	A	R	R	M	K	I	V	A	Q	A	V	D	R
L	A	T	V	N	E	C	I	R	S	A	R	R	V	K	I	V	A	Q	A	V	D	R
L	A	T	V	N	E	C	I	R	S	A	R	R	M	K	I	V	A	Q	A	V	D	R
L	A	T	V	N	E	C	I	R	S	A	R	R	M	K	I	V	A	Q	A	V	D	R
L	A	T	V	N	E	C	I	R	S	A	R	R	M	K	I	V	A	Q	A	V	D	R
L	A	T	V	N	E	C	I	R	S	A	R	R	M	K	I	V	A	Q	A	V	D	R
L	A	T	V	N	E	C	I	R	S	A	R	R	M	K	I	V	A	Q	A	V	D	R
L	A	T	V	N	E	C	I	R	S	A	R	R	V	K	I	V	A	Q	A	V	D	R
L	A	T	V	N	E	C	I	R	S	A	R	R	V	K	I	V	A	Q	A	V	D	R
L	A	T	V	N	E	C	I	R	S	A	R	R	V	K	I	V	A	Q	A	V	D	R
L	A	T	V	N	E	C	I	R	S	A	R	R	M	K	I	V	A	Q	A	V	D	R
L	A	T	V	N	E	C	I	R	S	A	R	R	M	K	I	V	A	Q	A	V	D	R
L	A	T	V	N	E	C	I	R	S	A	R	R	V	K	I	V	A	Q	A	V	D	R
L	A	T	V	N	E	C	I	R	S	A	R	R	V	K	I	V	A	Q	A	V	D	R
L	A	T	V	N	E	C	I	R	S	A	R	R	M	K	I	V	A	Q	A	V	D	R

L	A	T	V	N	E	C	I	R	S	A	R	R	M	K	I	V	A	Q	A	V	D	R
L	A	T	V	N	E	C	I	R	S	A	R	R	M	K	I	V	A	Q	A	V	D	R
L	A	T	V	N	E	C	I	R	S	A	R	R	M	K	I	V	A	Q	A	V	D	R
L	A	T	V	N	E	C	I	R	S	A	R	R	M	K	I	V	A	Q	A	V	D	R
L	A	T	V	N	E	C	I	R	S	A	R	R	M	K	I	V	A	Q	A	V	D	R
L	A	T	V	N	E	C	I	R	S	A	R	R	M	K	I	V	A	Q	A	V	D	R
L	A	T	V	N	E	C	I	R	S	A	R	R	M	K	I	V	A	Q	A	V	D	R
L	A	T	V	N	E	C	I	R	S	A	R	R	M	K	I	V	A	Q	A	V	D	R
L	A	T	V	N	E	C	I	R	S	A	R	R	M	K	I	V	A	Q	A	V	D	R
L	A	T	V	N	E	C	I	R	S	A	R	R	M	K	I	V	A	Q	A	V	D	R

wciN																					
259	263	264	265	271	273	276	278	279	281	283	284	286	287	288	293	295	296	300	305	2	4
N	I	D	Q	E	S	N	K	E	I	L	K	V	Q	K	C	V	F	I	F	D	I
E	V	E	I	R	T	Q	S	P	K	S	R	Y	K	I	M	L	Y	F	V	A	S
E	V	E	I	R	T	Q	S	P	K	S	R	Y	K	I	M	L	Y	F	V	A	S
E	V	E	I	R	T	Q	S	P	K	S	R	Y	K	I	M	L	Y	F	V	A	S
E	V	E	I	R	T	Q	S	P	K	S	R	Y	K	I	M	L	Y	F	V	A	S
E	V	E	I	R	T	Q	S	P	K	S	R	Y	K	I	M	L	Y	F	V	A	S
E	V	E	I	R	T	Q	S	P	K	S	R	Y	K	I	M	L	Y	F	V	A	S
E	V	E	I	R	T	Q	S	P	K	S	R	Y	K	I	M	L	Y	F	V	A	S
E	V	E	I	R	T	Q	S	P	K	S	R	Y	K	I	M	L	Y	F	V	A	S
E	V	E	I	R	T	Q	S	P	K	S	R	Y	K	I	M	L	Y	F	V	A	S

wciN																					
264	265	271	273	276	278	279	281	283	284	286	287	288	293	295	296	300	305	2	4	6	15
D	Q	E	S	N	K	E	I	L	K	V	Q	K	C	V	F	I	F	D	I	D	I
E	I	R	T	Q	S	P	K	S	R	Y	K	I	M	L	Y	F	V	A	S	G	V
D	Q	E	S	N	K	E	I	L	K	V	Q	K	C	V	F	I	F	D	I	D	I
D	Q	E	S	N	K	E	I	L	K	V	Q	K	C	V	F	I	F	D	I	D	I
D	Q	E	S	N	K	E	I	L	K	V	Q	K	C	V	F	I	F	D	I	D	I
D	Q	E	S	N	K	E	I	L	K	V	Q	K	C	V	F	I	F	D	I	D	I
D	Q	E	S	N	K	E	I	L	K	V	Q	K	C	V	F	I	F	D	I	D	I
D	Q	E	S	N	K	E	I	L	K	V	Q	K	C	V	F	I	F	D	I	D	I
D	Q	E	S	N	K	E	I	L	K	V	Q	K	C	V	F	I	F	D	I	D	I
D	Q	E	S	N	K	E	I	L	K	V	Q	K	C	V	F	I	F	D	I	D	I
D	Q	E	S	N	K	E	I	L	K	V	Q	K	C	V	F	I	F	D	I	D	I
D	Q	E	S	N	K	E	I	L	K	V	Q	K	C	V	F	I	F	D	I	D	I
D	Q	E	S	N	K	E	I	L	K	V	Q	K	C	V	F	I	F	D	I	D	I
D	Q	E	S	N	K	E	I	L	K	V	Q	K	C	V	F	I	F	D	I	D	I
D	Q	E	S	N	K	E	I	L	K	V	Q	K	C	V	F	I	F	D	I	D	I
D	Q	E	S	N	K	E	I	L	K	V	Q	K	C	V	F	I	F	D	I	D	I
D	Q	E	S	N	K	E	I	L	K	V	Q	K	C	V	F	I	F	D	I	D	I
D	Q	E	S	N	K	E	I	L	K	V	Q	K	C	V	F	I	F	D	I	D	I
D	Q	E	S	N	K	E	I	L	K	V	Q	K	C	V	F	I	F	D	I	D	I
D	Q	E	S	N	K	E	I	L	K	V	Q	K	C	V	F	I	F	D	I	D	I
D	Q	E	S	N	K	E	I	L	K	V	Q	K	C	V	F	I	F	D	I	D	I
D	Q	E	S	N	K	E	I	L	K	V	Q	K	C	V	F	I	F	D	I	D	I
D	Q	E	S	N	K	E	I	L	K	V	Q	K	C	V	F	I	F	D	I	D	I
D	Q	E	S	N	K	E	I	L	K	V	Q	K	C	V	F	I	F	D	I	D	I
D	Q	E	S	N	K	E	I	L	K	V	Q	K	C	V	F	I	F	D	I	D	I
D	Q	E	S	N	K	E	I	L	K	V	Q	K	C	V	F	I	F	D	I	D	I
D	Q	E	S	N	K	E	I	L	K	V	Q	K	C	V	F	I	F	D	I	D	I
D	Q	E	S	N	K	E	I	L	K	V	Q	K	C	V	F	I	F	D	I	D	I
D	Q	E	S	N	K	E	I	L	K	V	Q	K	C	V	F	I	F	D	I	D	I
D	Q	E	S	N	K	E	I	L	K	V	Q	K	C	V	F	I	F	D	I	D	I
D	Q	E	S	N	K	E	I	L	K	V	Q	K	C	V	F	I	F	D	I	D	I

:iO		
39	136	212
L	N	V
L	T	V
I	N	I

wciP				
62	126	128	158	318
F	M	M	H	T
L	L	T	Q	A
F	M	T	H	T

wzy											
34	35	60	70	89	107	117	178	220	250	403	444
I	A	Y	V	H	T	I	M	S	T	G	T
L	T	G	I	Y	I	T	I	A	A	S	T
I	A	Y	I	H	I	I	I	A	A	S	M

wciN

HG[illegible]

273	276	278	279	281	283	284	286	287	288	293	295	296	300	305
S	N	K	E	I	L	K	V	Q	K	C	V	F	I	F
T	Q	S	P	K	S	R	Y	K	I	M	L	Y	F	V
S	N	K	E	I	L	K	V	Q	K	C	V	F	I	F
S	N	K	E	I	L	K	V	Q	K	C	V	F	I	F
S	N	K	E	I	L	K	V	Q	K	C	V	F	I	F
S	N	K	E	I	L	K	V	Q	K	C	V	F	I	F
S	N	K	E	I	L	K	V	Q	K	C	V	F	I	F
S	N	K	E	I	L	K	V	Q	K	C	V	F	I	F
S	N	K	E	I	L	K	V	Q	K	C	V	F	I	F
S	N	K	E	I	L	K	V	Q	K	C	V	F	I	F
S	N	K	E	I	L	K	V	Q	K	C	V	F	I	F
S	N	K	E	I	L	K	V	Q	K	C	V	F	I	F
S	N	K	E	I	L	K	V	Q	K	C	V	F	I	F
S	N	K	E	I	L	K	V	Q	K	C	V	F	I	F
S	N	K	E	I	L	K	V	Q	K	C	V	F	I	F
S	N	K	E	I	L	K	V	Q	K	C	V	F	I	F
S	N	K	E	I	L	K	V	Q	K	C	V	F	I	F
S	N	K	E	I	L	K	V	Q	K	C	V	F	I	F
S	N	K	E	I	L	K	V	Q	K	C	V	F	I	F
S	N	K	E	I	L	K	V	Q	K	C	V	F	I	F
S	N	K	E	I	L	K	V	Q	K	C	V	F	I	F
S	N	K	E	I	L	K	V	Q	K	C	V	F	I	F
S	N	K	E	I	L	K	V	Q	K	C	V	F	I	F
S	N	K	E	I	L	K	V	Q	K	C	V	F	I	F
S	N	K	E	I	L	K	V	Q	K	C	V	F	I	F
S	N	K	E	I	L	K	V	Q	K	C	V	F	I	F
S	N	K	E	I	L	K	V	Q	K	C	V	F	I	F

2	4	6	15	18	34	39
D	I	D	I	E	I	L
A	S	G	V	K	M	S
D	I	D	I	E	I	L
D	I	D	I	E	I	L
D	I	D	I	E	I	L
D	I	D	I	E	I	L
D	I	D	I	E	I	L
D	I	D	I	E	I	L
D	I	D	I	E	I	L
D	I	D	I	E	I	L
D	I	D	I	E	I	L
D	I	D	I	E	I	L
D	I	D	I	E	I	L
D	I	D	I	E	I	L
D	I	D	I	E	I	L
D	I	D	I	E	I	L
D	I	D	I	E	I	L
D	I	D	I	E	I	L
D	I	D	I	E	I	L
D	I	D	I	E	I	L
D	I	D	I	E	I	L
D	I	D	I	E	I	L
D	I	D	I	E	I	L
D	I	D	I	E	I	L
D	I	D	I	E	I	L
D	I	D	I	E	I	L
D	I	D	I	E	I	L

		wchA																				
157	158	159	160	170	176	177	178	179	180	181	182	183	184	186	187	199	203	205	207	211	212	214
V	V	G	E	P	C	L	K	V	V	A	E	G	E	V	N	I	S	K	N	L	V	Q
V	V	G	E	P	Y	L	K	I	V	A	E	G	E	V	N	I	S	K	N	L	V	Q
L	S	W	K	S	K	I	P	V	I	E	K	E	K	I	E	V	G	S	D	I	I	R
V	V	G	E	P	C	L	K	V	V	A	E	G	E	V	N	I	S	K	N	L	V	Q
V	V	G	E	P	C	L	K	V	V	A	E	G	E	V	N	I	S	K	N	L	V	Q
V	V	G	E	P	C	L	K	V	V	A	E	G	E	V	N	I	S	K	N	L	V	Q
V	V	G	E	P	C	L	K	V	V	A	E	G	E	V	N	I	S	K	N	L	V	Q
V	V	G	E	P	C	L	K	V	V	A	E	G	E	V	N	I	S	K	N	L	V	Q
V	V	G	E	P	C	L	K	V	V	A	E	G	E	V	N	I	S	K	N	L	V	Q
V	V	G	E	P	C	L	K	V	V	A	E	G	E	V	N	I	S	K	N	L	V	Q
V	V	G	E	P	C	L	K	V	V	A	E	G	E	V	N	I	S	K	N	L	V	Q
V	V	G	E	P	C	L	K	V	V	A	E	G	E	V	N	I	S	K	N	L	V	Q
V	V	G	E	P	C	L	K	V	V	A	E	G	E	V	N	I	S	K	N	L	V	Q
V	V	G	E	P	C	L	K	V	V	A	E	G	E	V	N	I	S	K	N	L	V	Q
V	V	G	E	P	C	L	K	V	V	A	E	G	E	V	N	I	S	K	N	L	V	Q
V	V	G	E	P	C	L	K	V	V	A	E	G	E	V	N	I	S	K	N	L	V	Q
V	V	G	E	P	C	L	K	V	V	A	E	G	E	V	N	I	S	K	N	L	V	Q
V	V	G	E	P	C	L	K	V	V	A	E	G	E	V	N	I	S	K	N	L	V	Q
V	V	G	E	P	C	L	K	V	V	A	E	G	E	V	N	I	S	K	N	L	V	Q
V	V	G	E	P	C	L	K	V	V	A	E	G	E	V	N	I	S	K	N	L	V	Q
V	V	G	E	P	C	L	K	V	V	A	E	G	E	V	N	I	S	K	N	L	V	Q
V	V	G	E	P	C	L	K	V	V	A	E	G	E	V	N	I	S	K	N	L	V	Q
V	V	G	E	P	C	L	K	V	V	A	E	G	E	V	N	I	S	K	N	L	V	Q
V	V	G	E	P	C	L	K	V	V	A	E	G	E	V	N	I	S	K	N	L	V	Q
V	V	G	E	P	C	L	K	V	V	A	E	G	E	V	N	I	S	K	N	L	V	Q
V	V	G	E	P	C	L	K	V	V	A	E	G	E	V	N	I	S	K	N	L	V	Q
V	V	G	E	P	C	L	K	V	V	A	E	G	E	V	N	I	S	K	N	L	V	Q
V	V	G	E	P	C	L	K	V	V	A	E	G	E	V	N	I	S	K	N	L	V	Q
V	V	G	E	P	C	L	K	V	V	A	E	G	E	V	N	I	S	K	N	L	V	Q

V	V	G	E	P	C	L	K	V	V	A	E	G	E	V	N	I	S	K	N	L	V	Q
V	V	G	E	P	C	L	K	V	V	A	E	G	E	V	N	I	S	K	N	L	V	Q

359	367	384	421	430	449	451
L	N	D	V	N	F	R
L	N	D	V	N	F	R
L	N	D	V	N	F	R
L	N	D	V	N	F	R
L	N	D	V	N	F	R
L	N	D	V	N	F	R
L	N	D	V	N	F	R
L	N	D	V	N	F	R
L	N	D	V	N	F	R
L	N	D	V	N	F	R
L	N	D	V	N	F	R
L	N	D	V	N	F	R
L	N	D	V	N	F	R
L	N	D	V	N	F	R
L	N	D	V	N	F	R
L	N	D	V	N	F	R
L	N	D	V	N	F	R
L	N	D	V	N	F	R
L	N	D	V	N	F	R
L	N	D	V	N	F	R
L	N	D	V	N	F	R
L	N	D	V	N	F	R
L	N	D	V	N	F	R
L	N	D	V	N	F	R
L	N	D	V	N	F	R
L	N	D	V	N	F	R
L	N	D	V	N	F	R
L	N	D	V	N	F	R
L	N	D	V	N	F	R
L	N	D	V	N	F	R
L	N	D	V	N	F	R
L	N	D	V	N	F	R
L	N	D	V	N	F	R
L	N	D	V	N	F	R
L	N	D	V	N	F	R
L	N	D	V	N	F	R
L	N	D	V	N	F	R
L	N	D	V	N	F	R
L	N	D	V	N	F	R
L	N	D	V	N	F	R
L	N	D	V	N	F	R
L	N	D	V	N	F	R
L	N	D	V	N	F	R
L	N	D	V	N	F	R
L	N	D	V	N	F	R
L	N	D	V	N	F	R
L	N	D	V	N	F	R
L	N	D	I	N	F	R
L	N	D	V	N	F	R
L	N	D	V	N	F	R
L	N	D	V	N	F	R
L	N	D	V	N	F	R
L	N	D	V	N	F	R
L	N	D	V	N	F	R
L	N	D	V	N	F	R
L	N	D	I	N	F	R
L	N	D	V	N	F	R
L	N	N	V	N	F	R
L	N	D	V	N	F	R
L	N	D	V	N	F	R
L	N	D	V	N	F	R
L	N	D	V	N	F	R
L	N	D	V	N	F	R

														wc
2	14	52	115	121	168	247	251	257	259	263	264	265	271	273
N	V	S	F	G	R	S	K	G	N	I	D	Q	E	S
N	V	S	F	G	R	S	K	G	N	I	D	Q	E	S
N	V	S	F	G	R	S	K	G	N	I	D	Q	E	S
N	V	S	F	G	R	S	K	G	N	I	D	Q	E	S
N	V	S	F	G	R	S	K	G	N	I	D	Q	E	S
N	V	S	F	G	R	S	K	G	N	I	D	Q	E	S
N	V	S	F	G	R	S	K	G	N	I	D	Q	E	S
N	V	S	F	G	R	S	K	G	N	I	D	Q	E	S
N	V	S	F	G	R	S	K	G	N	I	D	Q	E	S
N	V	S	F	G	R	S	K	G	N	I	D	Q	E	S
N	V	S	F	G	R	S	K	G	N	I	D	Q	E	S
N	V	S	F	G	R	S	K	G	N	I	D	Q	E	S
N	V	S	F	G	R	S	K	G	N	I	D	Q	E	S
N	V	S	F	G	R	S	K	G	N	I	D	Q	E	S
N	V	S	F	G	R	S	K	G	N	I	D	Q	E	S
N	V	S	F	G	R	S	K	G	N	I	D	Q	E	S
N	V	S	F	G	R	S	K	G	N	I	D	Q	E	S
N	V	S	F	G	R	S	K	G	N	I	D	Q	E	S
N	V	S	F	G	R	S	K	G	N	I	D	Q	E	S
N	V	S	F	G	R	S	K	G	N	I	D	Q	E	S
N	V	S	F	G	R	S	K	G	N	I	D	Q	E	S
N	V	S	F	G	R	S	K	G	N	I	D	Q	E	S
N	V	S	F	G	R	S	K	G	N	I	D	Q	E	S
N	V	S	F	G	R	S	K	G	N	I	D	Q	E	S
N	V	S	F	G	R	S	K	G	N	I	D	Q	E	S
N	V	S	F	G	R	S	K	G	N	I	D	Q	E	S
N	V	S	F	G	R	S	K	G	N	I	D	Q	E	S
N	V	S	F	G	R	S	K	G	N	I	D	Q	E	S
N	V	S	F	G	R	S	K	G	N	I	D	Q	E	S
N	V	S	F	G	R	S	K	G	N	I	D	Q	E	S
N	V	S	F	G	R	S	K	G	N	I	D	Q	E	S
N	V	S	F	G	R	S	K	G	N	I	D	Q	E	S
N	V	S	F	G	R	S	K	G	N	I	D	Q	E	S
N	V	S	F	G	R	S	K	G	N	I	D	Q	E	S
N	V	S	F	G	R	S	K	G	N	I	D	Q	E	S
N	V	S	F	G	R	S	K	G	N	I	D	Q	E	S
N	V	S	F	G	R	S	K	G	N	I	D	Q	E	S
N	V	S	F	G	R	S	K	G	N	I	D	Q	E	S
N	V	S	F	G	R	S	K	G	N	I	D	Q	E	S
N	V	S	F	G	R	S	K	G	N	I	D	Q	E	S
N	V	S	F	G	R	S	K	G	N	I	D	Q	E	S
N	V	S	F	G	R	S	K	G	N	I	D	Q	E	S
N	V	S	F	G	R	S	K	G	N	I	D	Q	E	S
N	V	S	F	G	R	S	K	G	N	I	D	Q	E	S
N	V	S	F	G	R	S	K	G	N	I	D	Q	E	S
N	V	S	F	G	R	S	K	G	N	I	D	Q	E	S
N	V	S	F	G	R	S	K	G	N	I	D	Q	E	S
N	V	S	F	G	R	S	K	G	N	I	D	Q	E	S
N	V	S	F	G	R	S	K	G	N	I	D	Q	E	S
N	V	S	F	G	R	S	K	G	N	I	D	Q	E	S
N	V	S	F	G	R	S	K	G	N	I	D	Q	E	S
N	V	S	F	G	R	S	K	G	N	I	D	Q	E	S
N	V	S	F	G	R	S	K	G	N	I	D	Q	E	S
N	V	S	F	G	R	S	K	G	N	I	D	Q	E	S
N	V	S	F	G	R	S	K	G	N	I	D	Q	E	S
N	V	S	F	G	R	S	K	G	N	I	D	Q	E	S
N	V	S	F	G	R	S	K	G	N	I	D	Q	E	S
N	V	S	F	G	R	S	K	G	N	I	D	Q	E	S
N	V	S	F	G	R	S	K	G	N	I	D	Q	E	S
N	V	S	F	G	R	S	K	G	N	I	D	Q	E	S
N	V	S	F	G	R	S	K	G	N	I	D	Q	E	S
N	V	S	F	G	R	S	K	G	N	I	D	Q	E	S
N	V	S	F	G	R	S	K	G	N	I	D	Q	E	S
N	V	S	F	G	R	S	K	G	N	I	D	Q	E	S

L	N	D	V	N	F	R	N	V	S	F	G	R	S	K	G	N	I	D	Q	E	S
L	N	D	V	N	F	R	N	V	S	F	G	R	S	K	G	N	I	D	Q	E	S
L	N	D	V	N	F	R	N	V	S	F	G	R	S	K	G	N	I	D	Q	E	S
L	N	D	V	N	F	R	N	V	S	F	G	R	S	K	G	N	I	D	Q	E	S
L	N	D	V	N	F	R	N	V	S	F	G	R	S	K	G	N	I	D	Q	E	S
L	N	D	V	N	F	R	N	V	S	F	G	R	S	K	G	N	I	D	Q	E	S
L	N	D	V	N	F	R	N	V	S	F	G	R	S	K	G	N	I	D	Q	E	S
L	N	D	V	N	F	R	N	V	S	F	G	R	S	K	G	N	I	D	Q	E	S
L	N	D	V	N	F	R	N	V	S	F	G	R	S	K	G	N	I	D	Q	E	S
L	N	D	V	N	F	R	N	V	S	F	G	R	S	K	G	N	I	D	Q	E	S
L	N	D	V	N	F	R	N	V	S	F	G	R	S	K	G	N	I	D	Q	E	S
L	N	D	V	N	F	R	N	V	S	F	G	R	S	K	G	N	I	D	Q	E	S
L	N	D	V	N	F	R	N	V	S	F	G	R	S	K	G	N	I	D	Q	E	S
L	N	D	V	N	F	R	N	V	S	F	G	R	S	K	G	N	I	D	Q	E	S
L	N	D	V	N	F	R	N	V	S	F	G	R	S	K	G	N	I	D	Q	E	S
L	N	D	V	N	F	R	N	V	S	F	G	R	S	K	G	N	I	D	Q	E	S
L	N	D	V	N	F	R	N	V	S	F	G	R	S	K	G	N	I	D	Q	E	S
L	N	D	V	N	F	R	N	V	S	F	G	R	S	K	G	N	I	D	Q	E	S
L	N	D	V	N	F	R	N	V	S	F	G	R	S	K	G	N	I	D	Q	E	S
L	N	D	V	N	F	R	N	V	S	F	G	R	S	K	G	N	I	D	Q	E	S
L	N	D	V	N	F	R	N	V	S	F	G	R	S	K	G	N	I	D	Q	E	S
L	N	D	V	N	F	R	N	V	S	F	G	R	S	K	G	N	I	D	Q	E	S
L	N	D	V	N	F	R	N	V	S	F	G	R	S	K	G	N	I	D	Q	E	S
L	N	D	V	N	F	R	N	V	S	F	G	R	S	K	G	N	I	D	Q	E	S
L	N	D	V	N	F	R	N	V	S	F	D	R	S	K	G	N	I	D	Q	E	S
L	N	D	V	N	F	R	N	V	S	F	D	R	S	K	G	N	I	D	Q	E	S
L	N	D	V	N	F	R	N	V	S	F	D	R	S	K	G	N	I	D	Q	E	S
L	N	D	V	N	F	R	N	V	S	F	G	R	S	K	G	N	I	D	Q	E	S
L	N	D	V	N	F	R	N	V	S	F	G	R	S	K	G	N	I	D	Q	E	S
L	N	D	V	N	F	R	N	V	S	F	G	R	S	K	G	N	I	D	Q	E	S
L	N	D	V	N	F	R	N	V	S	F	G	R	S	K	G	N	I	D	Q	E	S
L	N	D	V	N	F	R	N	V	S	F	G	R	S	K	G	N	I	D	Q	E	S
L	N	D	V	N	F	R	N	V	S	F	G	R	S	K	G	N	I	D	Q	E	S
L	N	D	V	N	F	R	N	V	S	F	G	R	S	K	G	N	I	D	Q	E	S
L	N	D	V	N	F	R	N	V	S	F	G	R	S	K	G	N	I	D	Q	E	S
L	N	D	V	N	F	R	N	V	S	F	G	R	S	K	G	N	I	D	Q	E	S
L	N	D	V	N	F	R	N	V	S	F	G	R	S	K	G	N	I	D	Q	E	S
L	N	D	V	N	F	R	N	V	S	F	G	R	S	K	G	N	I	D	Q	E	S
L	N	D	V	N	F	R	N	V	S	F	G	R	S	K	G	N	I	D	Q	E	S
L	N	D	V	N	F	R	N	V	S	F	G	R	S	K	G	N	I	D	Q	E	S
L	N	D	V	N	F	R	N	V	S	F	G	R	S	K	G	N	I	D	Q	E	S
L	N	D	V	N	F	R	N	V	S	F	G	R	S	K	G	N	I	D	Q	E	S
L	N	D	V	N	F	R	N	V	S	F	G	R	S	K	G	N	I	D	Q	E	S
L	N	D	V	N	F	R	N	V	S	F	G	R	S	K	G	N	I	D	Q	E	S
L	N	D	V	N	F	R	N	V	S	F	G	R	S	K	G	N	I	D	Q	E	S
L	N	D	V	N	F	R	N	V	S	F	G	R	S	K	G	N	I	D	Q	E	S
L	N	D	V	N	F	R	N	V	S	F	G	R	S	K	G	N	I	D	Q	E	S
L	N	D	V	N	F	R	N	V	S	F	G	R	S	K	G	N	I	D	Q	E	S
L	N	D	V	N	F	R	N	V	S	F	G	R	S	K	G	N	I	D	Q	E	S
L	N	D	V	N	F	R	N	V	S	F	G	R	S	K	G	N	I	D	Q	E	S
L	N	D	V	N	F	R	N	V	S	F	G	R	S	K	G	N	I	D	Q	E	S
L	N	D	V	N	F	R	N	V	S	F	G	R	S	K	G	N	I	D	Q	E	S
L	N	D	V	N	F	R	N	V	S	F	G	R	S	K	G	N	I	D	Q	E	S
L	N	D	V	N	F	R	N	V	S	F	G	R	S	K	G	N	I	D	Q	E	S
L	N	D	V	N	F	R	N	V	S	F	G	R	S	K	G	N	I	D	Q	E	S
L	N	D	V	N	F	R	N	V	S	F	G	R	S	K	G	N	I	D	Q	E	S
L	N	D	V	N	F	R	N	V	S	F	G	R	S	K	G	N	I	D	Q	E	S
L	N	D	V	N	F	R	N	V	S	F	G	R	S	K	G	N	I	D	Q	E	S
L	N	D	V	N	F	R	N	V	S	F	G	R	S	K	G	N	I	D	Q	E	S
L	N	D	V	N	F	R	N	V	S	F	G	R	S	K	G	N	I	D	Q	E	S
L	N	D	V	N	F	R	N	V	S	F	G	R	S	K	G	N	I	D	Q	E	S
L	N	D	V	N	F	R	N	V	S	F	G	R	S	K	G	N	I	D	Q	E	S
L	N	D	V	N	F	R	N	V	S	F	G	R	S	K	G	N	I	D	Q	E	S
L	N	D	V	N	F	R	N	V	S	F	G	R	S	K	G	N	I	D	Q	E	S
L	N	D	V	N	F	R	N	V	S	F	G	R	S	K	G	N	I	D	Q	E	S
L	N	D	V	N	F	R	N	V	S	F	G	R	S	K	G	N	I	D	Q	E	S
L	N	D	V	N	F	R	N	V	S	F	G	R	S	K	G	N	I	D	Q	E	S

L	N	D	V	N	F	R	N	V	S	F	G	R	S	K	G	N	I	D	Q	E	S
L	N	D	V	N	F	R	N	V	S	F	G	R	S	K	G	N	I	D	Q	E	S
L	N	D	V	N	F	R	N	V	S	F	G	R	S	K	G	N	I	D	Q	E	S
L	N	D	V	N	F	R	N	V	S	F	G	R	S	K	G	N	I	D	Q	E	S
L	N	D	V	N	F	R	N	V	S	F	G	R	S	K	G	N	I	D	Q	E	S
L	N	D	V	N	F	R	N	V	S	F	G	R	S	K	G	N	I	D	Q	E	S
L	N	D	V	N	F	R	N	V	S	F	G	R	S	K	G	N	I	D	Q	E	S
L	N	D	V	N	F	R	N	V	S	F	G	R	S	K	G	N	I	D	Q	E	S
L	N	D	V	N	F	R	N	V	S	F	G	R	S	K	G	N	I	D	Q	E	S
L	N	D	V	N	F	R	N	V	S	F	G	R	S	K	G	N	I	D	Q	E	S
L	N	D	V	N	F	R	N	V	S	F	G	R	S	K	G	N	I	D	Q	E	S
L	N	D	V	N	F	R	N	V	S	F	G	R	S	K	G	N	I	D	Q	E	S
L	N	D	V	N	F	R	N	V	S	F	G	R	S	K	G	N	I	D	Q	E	S
L	N	D	V	N	F	R	N	V	S	F	G	R	S	K	G	N	I	D	Q	E	S
L	N	D	V	N	F	R	N	V	S	F	G	R	S	K	G	N	I	D	Q	E	S
L	N	D	V	N	F	R	N	V	S	F	G	R	S	K	G	N	I	D	Q	E	S
L	N	D	V	N	F	R	N	V	S	F	G	R	S	K	G	N	I	D	Q	E	S
L	N	D	V	N	F	R	N	V	S	F	G	R	S	K	G	N	I	D	Q	E	S
L	N	D	V	N	F	R	N	V	S	F	G	R	S	K	G	N	I	D	Q	E	S
L	N	D	V	N	F	R	N	V	S	F	G	R	S	K	G	N	I	D	Q	E	S
L	N	D	V	N	F	R	N	V	S	F	G	R	S	K	G	N	I	D	Q	E	S
L	N	D	V	N	F	R	N	V	S	F	G	R	S	K	G	N	I	D	Q	E	S
L	N	D	V	N	F	R	N	V	S	F	G	R	S	K	G	N	I	D	Q	E	S
L	N	D	V	N	F	R	N	V	S	F	G	R	S	K	G	N	I	D	Q	E	S
L	N	D	V	N	F	R	N	V	S	F	G	R	S	K	G	N	I	D	Q	E	S
L	N	D	V	N	F	R	N	V	S	F	G	R	S	K	G	N	I	D	Q	E	S
L	N	D	V	N	F	R	N	V	S	F	G	R	S	K	G	N	I	D	Q	E	S
L	N	D	V	N	F	R	N	V	S	F	G	R	S	K	G	N	I	D	Q	E	S
L	N	D	V	N	F	R	N	V	S	F	G	R	S	K	G	N	I	D	Q	E	S
L	N	D	V	N	F	R	N	V	S	F	G	R	S	K	G	N	I	D	Q	E	S
L	N	D	V	N	F	R	N	V	S	F	G	R	S	K	G	N	I	D	Q	E	S
L	N	D	V	N	F	R	N	V	S	F	G	R	S	K	G	N	I	D	Q	E	S
L	N	D	V	N	F	R	N	V	S	F	G	R	S	K	G	N	I	D	Q	E	S
L	N	D	V	N	F	R	N	V	S	F	G	R	S	K	G	N	I	D	Q	E	S
L	N	D	V	N	F	R	N	V	S	F	G	R	S	K	G	N	I	D	Q	E	S
L	N	D	V	N	F	R	N	V	S	F	G	R	S	K	G	N	I	D	Q	E	S
L	N	D	V	N	F	R	N	V	S	F	G	R	S	K	G	N	I	D	Q	E	S
L	N	D	V	N	F	R	N	V	S	F	G	R	S	K	G	N	I	D	Q	E	S
L	N	D	V	N	F	R	N	V	S	F	G	R	S	K	G	N	I	D	Q	E	S
L	N	D	V	N	F	R	N	V	S	F	G	R	S	K	G	N	I	D	Q	E	S
L	N	D	V	N	F	R	N	V	S	F	G	R	S	K	G	N	I	D	Q	E	S
L	N	D	V	N	F	R	N	V	S	F	G	R	S	K	G	N	I	D	Q	E	S
L	N	D	V	N	F	R	N	V	S	F	G	R	S	K	G	N	I	D	Q	E	S
L	N	D	V	N	F	R	N	V	S	F	G	R	S	K	G	N	I	D	Q	E	S
L	N	D	V	N	F	R	N	V	S	F	G	R	S	K	G	N	I	D	Q	E	S
L	N	D	V	N	F	R	N	V	S	F	G	R	S	K	G	N	I	D	Q	E	S
L	N	D	V	N	F	R	N	V	S	F	G	R	S	K	G	N	I	D	Q	E	S
L	N	D	V	N	F	R	N	V	S	F	G	R	S	K	G	N	I	D	Q	E	S
L	N	D	V	N	F	R	N	V	S	F	G	R	S	K	G	N	I	D	Q	E	S
L	N	D	V	N	F	R	N	V	S	F	G	R	S	K	G	N	I	D	Q	E	S
L	N	D	V	N	F	R	N	V	S	F	G	R	S	K	G	N	I	D	Q	E	S
L	N	D	V	N	F	R	N	V	S	F	G	R	S	K	G	N	I	D	Q	E	S
L	N	D	V	N	F	R	N	V	S	F	G	R	S	K	G	N	I	D	Q	E	S
L	N	D	V	N	F	R	N	V	S	F	G	R	S	K	G	N	I	D	Q	E	S
L	N	D	V	N	F	R	N	V	S	F	G	R	S	K	G	N	I	D	Q	E	S
L	N	D	V	N	F	R	N	V	S	F	G	R	S	K	G	N	I	D	Q	E	S
L	N	D	V	N	F	R	N	V	S	F	G	R	S	K	G	N	I	D	Q	E	S
L	N	D	V	N	F	R	N	V	S	F	G	R	S	K	G	N	I	D	Q	E	S
L	N	D	V	N	F	R	N	V	S	F	G	R	S	K	G	N	I	D	Q	E	S
L	N	D	V	N	F	R	N	V	S	F	G	R	S	K	G	N	I	D	Q	E	S
L	N	D	V	N	F	R	N	V	S	F	G	R	S	K	G	N	I	D	Q	E	S
L	N	D	V	N	F	R	N	V	S	F	G	R	S	K	G	N	I	D	Q	E	S
L	N	D	V	N	F	R	N	V	S	F	G	R	S	K	G	N	I	D	Q	E	S
L	N	D	V	N	F	R	N	V	S	F	G	R	S	K	G	N	I	D	Q	E	S
L	N	D	V	N	F	R	N	V	S	F	G	R	S	K	G	N	I	D	Q	E	S
L	N	D	V	N	F	R	N	V	S	F	G	R	S	K	G	N	I	D	Q	E	S
L	N	D	V	N	F	R	N	V	S	F	G	R	S	K	G	N	I	D	Q	E	S

L	N	D	V	N	F	R	N	V	S	F	G	R	S	K	G	N	I	D	Q	E	S
L	N	D	V	N	F	R	N	V	S	F	G	R	S	K	G	N	I	D	Q	E	S
L	N	D	V	N	F	R	N	V	S	F	G	R	S	K	G	N	I	D	Q	E	S
L	N	D	V	N	F	R	N	V	S	F	G	R	S	K	G	N	I	D	Q	E	S
L	N	D	V	N	F	R	N	V	S	F	G	R	S	K	G	N	I	D	Q	E	S
L	N	D	V	N	F	R	N	V	S	F	G	R	S	K	G	N	I	D	Q	E	S
L	N	D	V	N	F	R	N	V	S	F	G	R	S	K	G	N	I	D	Q	E	S
L	N	D	V	N	F	R	N	V	S	F	G	R	S	K	G	N	I	D	Q	E	S
L	N	D	V	N	F	R	N	V	S	F	G	R	S	K	G	N	I	D	Q	E	S
L	N	D	V	N	F	R	N	V	S	F	G	R	S	K	G	N	I	D	Q	E	S
L	N	D	V	N	F	R	N	V	S	F	G	R	S	K	G	N	I	D	Q	E	S
L	N	D	V	N	F	R	N	V	S	F	G	R	S	K	G	N	I	D	Q	E	S
L	N	D	V	N	F	R	N	V	S	F	G	R	S	K	G	N	I	D	Q	E	S
L	N	D	V	N	F	R	N	V	S	F	G	R	S	K	G	N	I	D	Q	E	S
L	N	D	V	N	F	R	N	V	S	F	G	R	S	K	G	N	I	D	Q	E	S
L	N	D	V	N	F	R	N	V	S	F	G	R	S	K	G	N	I	D	Q	E	S
L	N	D	V	N	F	R	N	V	S	F	G	R	S	K	G	N	I	D	Q	E	S
L	N	D	V	N	F	R	N	V	S	F	G	R	S	K	G	N	I	D	Q	E	S
F	N	D	V	N	F	R	N	V	S	F	G	R	S	K	G	N	I	D	Q	E	S
L	N	D	V	N	F	R	N	V	S	F	G	R	S	K	G	N	I	D	Q	E	S
L	N	D	V	N	F	R	N	V	S	F	G	R	S	K	G	N	I	D	Q	E	S
L	N	D	V	N	F	R	N	V	S	F	G	R	S	K	G	N	I	D	Q	E	S
L	N	D	V	N	F	R	N	V	S	F	G	R	S	K	G	N	I	D	Q	E	S
L	N	D	V	N	F	R	N	V	S	F	G	R	S	K	G	N	I	D	Q	E	S
L	N	D	V	N	F	R	N	V	S	F	G	R	S	K	G	N	I	D	Q	E	S
L	N	D	V	N	F	R	N	V	S	F	G	R	S	K	G	N	I	D	Q	E	S
L	N	D	V	N	F	R	N	V	S	F	G	R	S	K	G	N	I	D	Q	E	S
L	N	D	V	N	F	R	N	V	S	F	G	R	S	K	G	N	I	D	Q	E	S
L	N	D	V	N	F	R	N	V	S	F	G	R	S	K	G	N	I	D	Q	E	S
L	N	D	V	N	F	R	N	V	S	F	G	R	S	K	G	N	I	D	Q	E	S
L	N	D	V	N	F	R	N	V	S	F	G	R	S	K	G	N	I	D	Q	E	S
L	N	D	V	N	F	R	N	V	S	F	G	R	S	K	G	N	I	D	Q	E	S
L	N	D	V	N	F	R	N	V	S	F	G	R	S	K	G	N	I	D	Q	E	S
L	N	D	V	N	F	R	N	V	S	F	G	R	S	K	G	N	I	D	Q	E	S
L	N	D	V	N	F	R	N	V	S	F	G	R	S	K	G	N	I	D	Q	E	S
L	N	D	V	N	F	R	N	V	S	F	G	R	S	K	G	N	I	D	Q	E	S
L	N	D	V	N	F	R	N	V	S	F	G	R	S	K	G	N	I	D	Q	E	S
L	N	D	V	N	F	R	N	V	S	F	G	R	S	K	G	N	I	D	Q	E	S
L	N	D	V	N	F	R	N	V	S	F	G	R	S	K	G	N	I	D	Q	E	S
L	N	D	V	N	F	R	N	V	S	F	G	R	S	K	G	N	I	D	Q	E	S
L	N	D	V	N	F	R	N	V	S	F	G	R	S	K	G	N	I	D	Q	E	S
L	N	D	V	N	F	R	N	V	S	F	G	R	S	K	G	N	I	D	Q	E	S
L	N	D	V	N	F	R	N	V	S	F	G	R	S	K	G	N	I	D	Q	E	S
L	N	D	V	N	F	R	N	V	S	F	G	R	S	K	G	N	I	D	Q	E	S
L	N	D	V	N	F	R	N	V	S	F	G	R	S	K	G	N	I	D	Q	E	S
L	N	D	V	N	F	R	N	V	S	F	G	R	S	K	G	N	I	D	Q	E	S
L	N	D	V	N	F	R	N	V	S	F	G	R	S	K	G	N	I	D	Q	E	S
L	N	D	V	N	F	R	N	V	S	F	G	R	S	K	G	N	I	D	Q	E	S
L	N	D	V	N	F	R	N	V	S	F	G	R	S	K	G	N	I	D	Q	E	S
L	N	D	V	N	F	R	N	V	S	F	G	R	S	K	G	N	I	D	Q	E	S
L	N	D	V	N	F	R	N	V	S	F	G	R	S	K	G	N	I	D	Q	E	S
L	N	D	V	N	F	R	N	V	S	F	G	R	S	K	G	N	I	D	Q	E	S
L	N	D	V	N	F	R	N	V	S	F	G	R	S	K	G	N	I	D	Q	E	S
L	N	D	V	N	F	R	N	V	S	F	G	R	S	K	G	N	I	D	Q	E	S
L	N	D	V	N	F	R	N	V	S	F	G	R	S	K	G	N	I	D	Q	E	S
L	N	D	V	N	F	R	N	V	S	F	G	R	S	K	G	N	I	D	Q	E	S
L	N	D	V	N	F	R	N	V	S	F	G	R	S	K	G	N	I	D	Q	E	S
L	N	D	V	N	F	R	N	V	S	F	G	R	S	K	G	N	I	D	Q	E	S
L	N	D	V	N	F	R	N	V	S	F	G	R	S	K	G	N	I	D	Q	E	S
L	N	D	V	N	F	R	N	V	S	F	G	R	S	K	G	N	I	D	Q	E	S
L	N	D	V	N	F	R	N	V	S	F	G	R	S	K	G	N	I	D	Q	E	S
L	N	D	V	N	F	R	N	V	S	F	G	R	S	K	G	N	I	D	Q	E	S
L	N	D	V	N	F	R	N	V	S	F	G	R	S	K	G	N	I	D	Q	E	S
L	N	D	V	N	F	R	N	V	S	F	G	R	S	K	G	N	I	D	Q	E	S
L	N	D	V	N	F	R	N	V	S	F	G	R	S	K	G	N	I	D	Q	E	S
L	N	D	V	N	F	R	N	V	S	F	G	R	S	K	G	N	I	D	Q	E	S
L	N	D	V	N	F	R	N	V	S	F	G	R	S	K	G	N	I	D	Q	E	S

L	N	D	V	N	F	R	N	V	S	F	G	R	S	K	G	N	I	D	Q	E	S
L	N	D	V	N	F	R	N	V	S	F	G	R	S	K	G	N	I	D	Q	E	S
L	N	D	V	N	F	R	N	V	S	F	G	R	S	K	G	N	I	D	Q	E	S
L	N	D	V	N	F	R	N	V	S	F	G	R	S	K	G	N	I	D	Q	E	S
L	N	D	V	N	F	R	N	V	S	F	G	R	S	K	G	N	I	D	Q	E	S
L	N	D	V	N	F	R	N	V	S	F	G	R	S	K	G	N	I	D	Q	E	S
L	N	D	V	N	F	R	N	V	S	F	G	R	S	K	G	N	I	D	Q	E	S
L	N	D	V	N	F	R	N	V	S	F	G	R	S	K	G	N	I	D	Q	E	S
L	N	D	V	N	F	R	N	V	S	F	G	R	S	K	G	N	I	D	Q	E	S
L	N	D	V	N	F	R	N	V	S	F	G	R	S	K	G	N	I	D	Q	E	S
L	N	D	V	N	F	R	N	V	S	F	G	R	S	K	G	N	I	D	Q	E	S
L	N	D	V	N	F	R	N	V	A	F	G	R	S	K	G	N	I	D	Q	E	S
L	N	D	V	N	F	R	N	V	A	F	G	R	S	K	G	N	I	D	Q	E	S
L	N	D	V	N	F	R	N	V	A	F	G	R	S	K	G	N	I	D	Q	E	S
L	N	D	V	N	F	R	N	V	A	F	G	R	S	K	G	N	I	D	Q	E	S
L	N	D	V	N	F	R	N	V	A	F	G	R	S	K	G	N	I	D	Q	E	S
L	N	D	V	N	F	R	N	V	A	F	G	R	S	K	G	N	I	D	Q	E	S
L	N	D	V	N	F	R	N	V	A	F	G	R	S	K	G	N	I	D	Q	E	S
L	N	D	V	N	F	R	N	V	A	F	G	R	S	K	G	N	I	D	Q	E	S
L	N	D	V	N	F	R	N	V	A	F	G	R	S	K	G	N	I	D	Q	E	S
L	N	D	V	D	L	K	D	V	S	L	D	K	C	L	N	E	V	E	I	R	T
L	N	D	V	D	L	K	D	V	S	L	D	K	C	L	N	E	V	E	I	R	T
L	N	D	V	D	L	K	D	V	S	L	D	K	C	L	N	E	V	E	I	R	T
L	N	D	V	D	L	K	D	V	S	L	D	K	C	L	N	E	V	E	I	R	T
L	N	D	V	D	L	K	D	V	S	L	D	K	C	L	N	E	V	E	I	R	T
L	N	D	V	D	L	K	D	V	S	L	D	K	C	L	N	E	V	E	I	R	T
L	N	D	V	D	L	K	D	V	S	L	D	K	C	L	N	E	V	E	I	R	T
L	N	D	V	D	L	K	D	V	S	L	D	K	C	L	N	E	V	E	I	R	T
L	N	D	V	N	F	R	N	V	S	F	G	R	S	K	G	N	I	D	Q	E	S
L	N	D	V	N	F	R	N	V	S	F	G	R	S	K	G	N	I	D	Q	E	S
L	N	D	V	N	F	R	N	V	S	F	G	R	S	K	G	N	I	D	Q	E	S
L	N	D	V	N	F	R	N	V	S	F	G	R	S	K	G	N	I	D	Q	E	S
L	N	D	V	N	F	R	N	V	S	F	G	R	S	K	G	N	I	D	Q	E	S
L	N	D	V	N	F	R	N	V	S	F	G	R	S	K	G	N	I	D	Q	E	S
L	N	D	V	N	F	R	N	V	S	F	G	R	S	K	G	N	I	D	Q	E	S
L	N	D	V	N	F	R	N	V	S	F	G	R	S	K	G	N	I	D	Q	E	S
L	N	D	V	N	F	R	N	V	S	F	G	R	S	K	G	N	I	D	Q	E	S
L	N	D	V	N	F	R	N	V	S	F	G	R	S	K	G	N	I	D	Q	E	S
L	N	D	V	N	F	R	N	V	S	F	G	R	S	K	G	N	I	D	Q	E	S
L	N	D	V	N	F	R	N	V	S	F	G	R	S	K	G	N	I	D	Q	E	S
L	N	D	V	N	F	R	N	V	S	F	G	R	S	K	G	N	I	D	Q	E	S
L	N	D	V	N	F	R	N	V	S	F	G	R	S	K	G	N	I	D	Q	E	S
L	N	D	V	N	F	R	N	A	S	F	G	R	S	K	G	N	I	D	Q	E	S
L	N	D	V	N	F	R	N	V	S	F	G	R	S	K	G	N	I	D	Q	E	S
L	N	D	V	N	F	R	N	V	S	F	G	R	S	K	G	N	I	D	Q	E	S
L	N	D	V	N	F	R	N	V	S	F	G	R	S	K	G	N	I	D	Q	E	S
L	N	D	V	N	F	R	N	V	S	F	G	R	S	K	G	N	I	D	Q	E	S
L	N	D	V	N	F	R	N	V	S	F	G	R	S	K	G	N	I	D	Q	E	S
L	N	D	V	N	F	R	N	V	S	F	G	R	S	K	G	N	I	D	Q	E	S
L	N	D	V	N	F	R	N	V	S	F	G	R	S	K	G	N	I	D	Q	E	S
L	N	D	V	N	F	R	N	V	S	F	G	R	S	K	G	N	I	D	Q	E	S
L	N	D	V	N	F	R	N	V	S	F	G	R	S	K	G	N	I	D	Q	E	S
L	N	D	V	N	F	R	N	V	S	F	G	R	S	K	G	N	I	D	Q	E	S
L	N	D	V	N	F	R	N	V	S	F	G	R	S	K	G	N	I	D	Q	E	S
L	N	D	V	N	F	R	N	V	S	F	G	R	S	K	G	N	I	D	Q	E	S
L	N	D	V	N	F	R	N	V	S	F	G	R	S	K	G	N	I	D	Q	E	S
L	D	D	V	N	F	R	N	V	S	F	G	R	S	K	G	N	I	D	Q	E	S
L	N	D	V	N	F	R	N	V	S	F	G	R	S	K	G	N	I	D	Q	E	S
L	N	D	V	N	F	R	N	V	S	F	G	R	S	K	G	N	I	D	Q	E	S
L	N	D	V	N	F	R	N	V	S	F	G	R	S	K	G	N	I	D	Q	E	S
L	N	D	V	N	F	R	N	V	S	F	G	R	S	K	G	N	I	D	Q	E	S
L	N	D	V	N	F	R	N	V	S	F	G	R	S	K	G	N	I	D	Q	E	S
L	N	D	V	N	F	R	N	V	S	F	G	R	S	K	G	N	I	D	Q	E	S
L	N	D	V	N	F	R	N	V	S	F	G	R	S	K	G	N	I	D	Q	E	S
L	N	D	V	N	F	R	N	V	S	F	G	R	S	K	G	N	I	D	Q	E	S
L	N	D	V	N	F	R	N	V	S	F	G	R	S	K	G	N	I	D	Q	E	S

L	N	D	V	N	F	R	N	V	S	F	G	R	S	K	G	N	I	D	Q	E	S
L	N	D	V	N	F	R	N	V	S	F	G	R	S	K	G	N	I	D	Q	E	S
L	N	D	V	N	F	R	N	V	S	F	G	R	S	K	G	N	I	D	Q	E	S
L	N	D	V	N	F	R	N	V	S	F	G	R	S	K	G	N	I	D	Q	E	S
L	N	D	V	N	F	R	N	V	S	F	G	R	S	K	G	N	I	D	Q	E	S
L	N	D	V	N	F	R	N	V	S	F	G	R	S	K	G	N	I	D	Q	E	S
L	N	D	V	N	F	R	N	V	S	F	G	R	S	K	G	N	I	D	Q	E	S
L	N	D	V	N	F	R	N	V	S	F	G	R	S	K	G	N	I	D	Q	E	S
L	N	D	V	N	F	R	N	V	S	F	G	R	S	K	G	N	I	D	Q	E	S
L	N	D	V	N	F	R	N	V	S	F	G	R	S	K	G	N	I	D	Q	E	S
L	N	D	V	N	F	R	N	V	S	F	G	R	S	K	G	N	I	D	Q	E	S
L	N	D	V	N	F	R	N	V	S	F	G	R	S	K	G	N	I	D	Q	E	S
L	N	D	V	N	F	R	N	V	S	F	G	R	S	K	G	N	I	D	Q	E	S
L	N	D	V	N	F	R	N	V	S	F	G	R	S	K	G	N	I	D	Q	E	S
L	N	D	V	N	F	R	N	V	S	F	G	R	S	K	G	N	I	D	Q	E	S
L	N	D	V	N	F	R	N	V	S	F	G	R	S	K	G	N	I	D	Q	E	S
L	N	D	V	N	F	R	N	V	S	F	G	R	S	K	G	N	I	D	Q	E	S
L	N	D	V	N	F	R	N	V	S	F	G	R	S	K	G	N	I	D	Q	E	S
L	N	D	V	N	F	R	N	V	S	F	G	R	S	K	G	N	I	D	Q	E	S
L	N	D	V	N	F	R	N	V	S	F	G	R	S	K	G	N	I	D	Q	E	S
L	N	D	V	N	F	R	N	V	S	F	G	R	S	K	G	N	I	D	Q	E	S
L	N	D	V	N	F	R	N	V	S	F	G	R	S	K	G	N	I	D	Q	E	S
L	N	D	V	N	F	R	N	V	S	F	G	R	S	K	G	N	I	D	Q	E	S
L	N	D	V	N	F	R	N	V	S	F	G	R	S	K	G	N	I	D	Q	E	S
L	N	D	V	N	F	R	N	V	S	F	G	R	S	K	G	N	I	D	Q	E	S
L	N	D	V	N	F	R	N	V	S	F	G	R	S	K	G	N	I	D	Q	E	S
L	N	D	V	N	F	R	N	V	S	F	G	R	S	K	G	N	I	D	Q	E	S
L	N	D	V	N	F	R	N	V	S	F	G	R	S	K	G	N	I	D	Q	E	S
L	N	D	V	N	F	R	N	V	S	F	G	R	S	K	G	N	I	D	Q	E	S
L	N	D	V	N	F	R	N	V	S	F	G	R	S	K	G	N	I	D	Q	E	S
L	N	D	V	N	F	R	N	V	S	F	G	R	S	K	G	N	I	D	Q	E	S
L	N	D	V	N	F	R	N	V	S	F	G	R	S	K	G	N	I	D	Q	E	S
L	N	D	V	N	F	R	N	V	S	F	G	R	S	K	G	N	I	D	Q	E	S
L	N	D	V	N	F	R	N	V	S	F	G	R	S	K	G	N	I	D	Q	E	S
L	N	D	V	N	F	R	N	V	S	F	G	R	S	K	G	N	I	D	Q	E	S
L	N	D	V	N	F	R	N	V	S	F	G	R	S	K	G	N	I	D	Q	E	S
L	N	D	V	N	F	R	N	V	S	F	G	R	S	K	G	N	I	D	Q	E	S
L	N	D	V	N	F	R	N	V	S	F	G	R	S	K	G	N	I	D	Q	E	S
L	N	D	V	N	F	R	N	V	S	F	G	R	S	K	G	N	I	D	Q	E	S
L	N	D	V	N	F	R	N	V	S	F	G	R	S	K	G	N	I	D	Q	E	S
L	N	D	V	N	F	R	N	V	S	F	G	R	S	K	G	N	I	D	Q	E	S
L	N	D	V	N	F	R	N	V	S	F	G	R	S	K	G	N	I	D	Q	E	S
L	N	D	V	N	F	R	N	V	S	F	G	R	S	K	G	N	I	D	Q	E	S
L	N	D	V	N	F	R	N	V	S	F	G	R	S	K	G	N	I	D	Q	E	S
L	N	D	V	N	F	R	N	V	S	F	G	R	S	K	G	N	I	D	Q	E	S
L	N	D	V	N	F	R	N	V	A	F	G	R	S	K	G	N	I	D	Q	E	S
L	N	D	V	N	F	R	N	V	A	F	G	R	S	K	G	N	I	D	Q	E	S
L	N	D	V	N	F	R	N	V	A	F	G	R	S	K	G	N	I	D	Q	E	S
L	N	D	V	N	F	R	N	V	A	F	G	R	S	K	G	N	I	D	Q	E	S
L	N	D	V	N	F	R	N	V	S	F	G	R	S	K	G	N	I	D	Q	E	S
L	N	D	V	N	F	R	N	V	S	F	G	R	S	K	G	N	I	D	Q	E	S
L	N	D	V	N	F	R	N	V	S	F	G	R	S	K	G	N	I	D	Q	E	S
L	N	D	V	N	F	R	N	V	S	F	G	R	S	K	G	N	I	D	Q	E	S
L	N	D	V	N	F	R	N	V	S	F	G	R	S	K	G	N	I	D	Q	E	S
L	N	D	V	N	F	R	N	V	S	F	G	R	S	K	G	N	I	D	Q	E	S
L	N	D	V	N	F	R	N	V	S	F	G	R	S	K	G	N	I	D	Q	E	S
L	N	D	V	N	F	R	N	V	S	F	G	R	S	K	G	N	I	D	Q	E	S
L	N	D	V	N	F	R	N	V	S	F	G	R	S	K	G	N	I	D	Q	E	S
L	N	D	V	N	F	R	N	V	S	F	G	R	S	K	G	N	I	D	Q	E	S
L	N	D	V	N	F	R	N	V	S	F	G	R	S	K	G	N	I	D	Q	E	S
L	N	D	V	N	F	R	N	V	S	F	G	R	S	K	G	N	I	D	Q	E	S
L	N	D	V	N	F	R	N	V	S	F	G	R	S	K	G	N	I	D	Q	E	S
L	N	D	V	N	F	R	N	V	S	F	G	R	S	K	G	N	I	D	Q	E	S
L	N	D	V	N	F	R	N	V	S	F	G	R	S	K	G	N	I	D	Q	E	S
L	N	D	V	N	F	R	N	V	S	F	G	R	S	K	G	N	I	D	Q	E	S
L	N	D	V	N	F	R	N	V	S	F	G	R	S	K	G	N	I	D	Q	E	S
L	N	D	V	N	F	R	N	V	S	F	G	R	S	K	G	N	I	D	Q	E	S

L	N	D	V	N	F	R
L	N	D	V	N	F	R
L	N	D	V	N	F	R
L	N	D	V	N	F	R
L	N	D	V	N	F	R
L	N	D	V	N	F	R
L	N	D	V	N	F	R
L	N	D	V	N	F	R
L	N	D	V	N	F	R
L	N	D	V	N	F	R

N	V	S	F	G	R	S	K	G	N	I	D	Q	E	S
N	V	S	F	G	R	S	K	G	N	I	D	Q	E	S
N	V	S	F	G	R	S	K	G	N	I	D	Q	E	S
N	V	S	F	G	R	S	K	G	N	I	D	Q	E	S
N	V	S	F	G	R	S	K	G	N	I	D	Q	E	S
N	V	S	F	G	R	S	K	G	N	I	D	Q	E	S
N	V	S	F	G	R	S	K	G	N	I	D	Q	E	S
N	V	S	F	G	R	S	K	G	N	I	D	Q	E	S
N	V	S	F	G	R	S	K	G	N	I	D	Q	E	S
N	V	S	F	G	R	S	K	G	N	I	D	Q	E	S

HG262										
6	15	18	34	39	45	47	49	60	62	63
D	I	E	I	L	G	V	H	D	S	F
G	V	K	M	S	E	A	Y	A	G	L
G	V	K	M	S	E	A	Y	A	G	L
G	V	K	M	S	E	A	Y	A	G	L
G	V	K	M	S	E	A	Y	A	G	L
G	V	K	M	S	E	A	Y	A	G	L
G	V	K	M	S	E	A	Y	A	G	L
G	V	K	M	S	E	A	Y	A	G	L
G	V	K	M	S	E	A	Y	A	G	L
G	V	K	M	S	E	A	Y	A	G	L

5	6	7	23	45	54	60	64	67	68	70
R	I	C	A	V	I	I	V	I	I	I
K	L	Y	A	L	V	A	L	L	T	T
K	L	C	P	V	I	I	V	I	I	I
K	L	Y	A	L	V	A	L	L	T	T
K	L	Y	A	L	V	A	L	L	T	T
K	L	Y	A	L	V	A	L	L	T	T
K	L	Y	A	L	V	A	L	L	T	T
K	L	Y	A	L	V	A	L	L	T	T
K	L	Y	A	L	V	A	L	L	T	T
K	L	Y	A	L	V	A	L	L	T	T
K	L	Y	A	L	V	A	L	L	T	T

HG262									
18	34	39	45	47	49	56	60	62	63
E	I	L	G	V	H	-	D	S	F
K	M	S	E	A	Y	N	A	G	L
E	I	L	G	V	H	-	D	S	F
E	I	L	G	V	H	-	D	S	F
E	I	L	G	V	H	-	D	S	F
E	I	L	G	V	H	-	D	S	F
E	I	L	G	V	H	-	D	S	F
E	I	L	G	V	H	-	D	S	F
E	I	L	G	V	H	-	D	S	F
E	I	L	G	V	H	-	D	S	F
E	I	L	G	V	H	-	D	S	F
E	I	L	G	V	H	-	D	S	F
E	I	L	G	V	H	-	D	S	F
E	I	L	G	V	H	-	D	S	F
E	I	L	G	V	H	-	D	S	F
E	I	L	G	V	H	-	D	S	F
E	I	L	G	V	H	-	D	S	F
E	I	L	G	V	H	-	D	S	F
E	I	L	G	V	H	-	D	S	F
E	I	L	G	V	H	-	D	S	F
E	I	L	G	V	H	-	D	S	F
E	I	L	G	V	H	-	D	S	F
E	I	L	G	V	H	-	D	S	F
E	I	L	G	V	H	-	D	S	F
E	I	L	G	V	H	-	D	S	F
E	I	L	G	V	H	D	D	S	F
E	I	L	G	V	H	-	D	S	F
E	I	L	G	V	H	-	D	S	F
E	I	L	G	V	H	-	D	S	F
E	I	L	G	V	H	-	D	S	F
E	I	L	G	V	H	-	D	S	F

5	6	7	39	45	54	60	64	67	68	70	74
R	I	C	L	V	I	I	V	I	I	I	D
K	L	Y	L	L	V	A	L	L	T	T	N
R	I	C	L	V	I	I	V	I	I	I	D
R	I	C	L	V	I	I	V	I	I	I	D
R	I	C	L	V	I	I	V	I	I	I	D
R	I	C	L	V	I	I	V	I	I	I	D
R	I	C	L	V	I	I	V	I	I	I	D
R	I	C	L	V	I	I	V	I	I	I	D
R	I	C	L	V	I	I	V	I	I	I	D
R	I	C	L	V	I	I	V	I	I	I	D
R	I	C	L	V	I	I	V	I	I	I	D
R	I	C	L	V	I	I	V	I	I	I	D
R	I	C	L	V	I	I	V	I	I	I	D
R	I	C	L	V	I	I	V	I	I	I	D
R	I	C	L	V	I	I	V	I	I	I	D
R	I	C	L	V	I	I	V	I	I	I	D
R	I	C	L	V	I	I	V	I	I	I	D
R	I	C	L	V	I	I	V	I	I	I	D
R	I	C	L	V	I	I	V	I	I	I	D
R	I	C	L	V	I	I	V	I	I	I	D
R	I	C	L	V	I	I	V	I	I	I	D
R	I	C	L	V	I	I	V	I	I	I	D
R	I	C	L	V	I	I	V	I	I	I	D
R	I	C	L	V	I	I	V	I	I	I	D
R	I	C	L	V	I	I	V	I	I	I	D
R	I	C	I	V	I	I	V	I	I	I	D
R	I	C	L	V	I	I	V	I	I	I	D
R	I	C	L	V	I	I	V	I	I	I	D
R	I	C	L	V	I	I	V	I	I	I	D
R	I	C	L	V	I	I	V	I	I	I	D
R	I	C	L	V	I	I	V	I	I	I	D

wzx				
89	112	254	287	370
F	A	T	I	L
F	A	M	I	L
S	S	M	V	F

rmlA																
47	63	66	91	118	125	136	150	151	163	186	191	200	203	206	255	268
R	E	Q	I	T	K	H	E	N	Y	S	P	V	A	D	A	A
R	E	Q	I	K	K	Q	T	D	Y	S	P	V	A	D	A	A
K	D	L	L	K	Q	Q	T	D	N	G	A	I	V	N	A	E
R	E	Q	I	K	K	H	T	D	Y	S	P	V	A	D	S	E

262

wciO

45	47	49	56	60	62	63
G	V	H	-	D	S	F
E	A	Y	N	A	G	L
G	V	H	D	D	S	F
G	V	H	D	D	S	F
G	V	H	D	D	S	F
G	V	H	D	D	S	F
G	V	H	D	D	S	F
G	V	H	D	D	S	F
G	V	H	D	D	S	F
G	V	H	D	D	S	F
G	V	H	D	D	S	F
G	V	H	D	D	S	F
G	V	H	D	D	S	F
G	V	H	D	D	S	F
G	V	H	D	D	S	F
G	V	H	D	D	S	F
G	V	H	D	D	S	F
G	V	H	D	D	S	F
G	V	H	D	D	S	F
G	V	H	D	D	S	F
G	V	H	D	D	S	F
G	V	H	D	D	S	F
G	V	H	D	D	S	F
G	V	H	D	D	S	F
G	V	H	D	D	S	F
G	V	H	D	D	S	F
G	V	H	D	D	S	F

5	6	7	45	54	60	64	67	68	70	74	76	77	133	137
R	I	C	V	I	I	V	I	I	I	D	V	V	A	D
K	L	Y	L	V	A	L	L	T	T	N	I	L	T	G
R	I	C	V	I	I	V	I	I	I	D	V	V	A	D
R	I	C	V	I	I	V	I	I	I	D	V	V	A	D
R	I	C	V	I	I	V	I	I	I	D	V	V	A	D
R	I	C	V	I	I	V	I	I	I	D	V	V	A	D
R	I	C	V	I	I	V	I	I	I	D	V	V	A	D
R	I	C	V	I	I	V	I	I	I	D	V	V	A	D
R	I	C	V	I	I	V	I	I	I	D	V	V	A	D
R	I	C	V	I	I	V	I	I	I	D	V	V	A	D
R	I	C	V	I	I	V	I	I	I	D	V	V	A	D
R	I	C	V	I	I	V	I	I	I	D	V	V	A	D
R	I	C	V	I	I	V	I	I	I	D	V	V	A	D
R	I	C	V	I	I	V	I	I	I	D	V	V	A	D
R	I	C	V	I	I	V	I	I	I	D	V	V	A	D
R	I	C	V	I	I	V	I	I	I	D	V	V	A	D
R	I	C	V	I	I	V	I	I	I	D	V	V	A	D
R	I	C	V	I	I	V	I	I	I	D	V	V	A	D
R	I	C	V	I	I	V	I	I	I	D	V	V	A	D
R	I	C	V	I	I	V	I	I	I	D	V	V	A	D
R	I	C	V	I	I	V	I	I	I	D	V	V	A	D
R	I	C	V	I	I	V	I	I	I	D	V	V	A	D
R	I	C	V	I	I	V	I	I	I	D	V	V	A	D
R	I	C	V	I	I	V	I	I	I	D	V	V	A	D
R	I	C	V	I	I	V	I	I	I	D	V	V	A	D
R	I	C	V	I	I	V	I	I	I	D	V	V	A	D
R	I	C	V	I	I	V	I	I	I	D	V	V	A	D

227	231	232	233	234	240	241	242	243	246	253	262	266	269	272	273	278	279	280	281	283	295	309
N	R	S	L	A	R	E	M	A	N	T	A	I	V	L	V	C	G	L	V	I	S	Q
N	R	S	L	A	R	K	M	A	N	T	A	I	V	L	V	C	G	L	V	I	S	Q
K	K	D	F	G	H	E	M	V	D	N	S	L	C	T	I	F	A	I	A	L	P	H
N	R	S	L	A	R	E	V	A	N	T	A	I	V	L	V	C	G	L	V	I	S	Q
N	R	S	L	A	R	E	M	A	N	T	A	I	V	L	V	C	G	L	V	I	S	Q
N	R	S	L	A	R	E	M	A	N	T	A	I	V	L	V	C	G	L	V	I	S	Q
N	R	S	L	A	R	E	M	A	N	T	A	I	V	L	V	C	G	L	V	I	S	Q
N	R	S	L	A	R	E	V	A	N	T	A	I	V	L	V	C	G	L	V	I	S	Q
N	R	S	L	A	R	E	M	A	N	T	A	I	V	L	V	C	G	L	V	I	S	Q
N	R	S	L	A	R	E	M	A	N	T	A	I	V	L	V	C	G	L	V	I	S	Q
N	R	S	L	A	R	E	M	A	N	T	A	I	V	L	V	C	G	L	V	I	S	Q
N	R	S	L	A	R	E	M	A	N	T	A	I	V	L	V	C	G	L	V	I	S	Q
N	R	S	L	A	R	E	M	A	N	T	A	I	V	L	V	C	G	L	V	I	S	Q
N	R	S	L	A	R	E	V	A	N	T	A	I	V	L	V	C	G	L	V	I	S	Q
N	R	S	L	A	R	E	V	A	N	T	A	I	V	L	V	C	G	L	V	I	S	Q
N	R	S	L	A	R	E	V	A	N	T	A	I	V	L	V	C	G	L	V	I	S	Q
N	R	S	L	A	R	E	M	A	N	T	A	I	V	L	V	C	G	L	V	I	S	Q
N	R	S	L	A	R	E	M	A	N	T	A	I	V	L	V	C	G	L	V	I	S	Q
N	R	S	L	A	R	E	V	A	N	T	A	I	V	L	V	C	G	L	V	I	S	Q
N	R	S	L	A	R	E	V	A	N	T	A	I	V	L	V	C	G	L	V	I	S	Q
N	R	S	L	A	R	E	M	A	N	T	A	I	V	L	V	C	G	L	V	I	S	Q
N	R	S	L	A	R	E	M	A	N	T	A	I	V	L	V	C	G	L	V	I	S	Q
N	R	S	L	A	R	E	M	A	N	T	A	I	V	L	V	C	G	L	V	I	S	Q
N	R	S	L	A	R	E	M	A	N	T	A	I	V	L	V	C	G	L	V	I	S	Q
N	R	S	L	A	R	E	M	A	N	T	A	I	V	L	V	C	G	L	V	I	S	Q
N	R	S	L	A	R	E	M	A	N	T	A	I	V	L	V	C	G	L	V	I	S	Q
N	R	S	L	A	R	E	M	A	N	T	A	I	V	L	V	C	G	L	V	I	S	Q
N	R	S	L	A	R	E	M	A	N	T	A	I	V	L	V	C	G	L	V	I	S	Q
N	R	S	L	A	R	E	M	A	N	T	A	I	V	L	V	C	G	L	V	I	S	Q

N	R	S	L	A	R	E	M	A	N	T	A	I	V	L	V	C	G	L	V	I	S	Q
N	R	S	L	A	R	E	M	A	N	T	A	I	V	L	V	C	G	L	V	I	S	Q

:iN

276	278	279	281	283	284	286	287	288	289	293	295	296	300	305
N	K	E	I	L	K	V	Q	K	L	C	V	F	I	F
N	K	E	I	L	K	V	Q	K	L	C	V	F	I	F
N	K	E	I	L	K	V	Q	K	L	C	-	F	I	F
N	K	E	I	L	K	V	Q	K	L	C	V	F	I	F
N	K	E	I	L	K	V	Q	K	L	C	V	F	I	F
N	K	E	I	L	K	V	Q	K	L	C	V	F	I	F
N	K	E	I	L	K	V	Q	K	L	C	V	F	I	F
N	K	E	I	L	K	V	Q	K	L	C	V	F	I	F
N	K	E	I	L	K	V	Q	K	L	C	V	F	I	F
N	K	E	I	L	K	V	Q	K	L	C	V	F	I	F
N	K	E	I	L	K	V	Q	K	L	C	V	F	I	F
N	K	E	I	L	K	V	Q	K	L	C	V	F	I	F
N	K	E	I	L	K	V	Q	K	L	C	V	F	I	F
N	K	E	I	L	K	V	Q	K	L	C	V	F	I	F
N	K	E	I	L	K	V	Q	K	L	C	V	F	I	F
N	K	E	I	L	K	V	Q	K	L	C	V	F	I	F
N	K	E	I	L	K	V	Q	K	L	C	V	F	I	F
N	K	E	I	L	K	V	Q	K	L	C	V	F	I	F
N	K	E	I	L	K	V	Q	K	L	C	V	F	I	F
N	K	E	I	L	K	V	Q	K	L	C	V	F	I	F
N	K	E	I	L	K	V	Q	K	L	C	V	F	I	F
N	K	E	I	L	K	V	Q	K	L	C	V	F	I	F
N	K	E	I	L	K	V	Q	K	L	C	V	F	I	F
N	K	E	I	L	K	V	Q	K	L	C	V	F	I	F
N	K	E	I	L	K	V	Q	K	L	C	V	F	I	F
N	K	E	I	L	K	V	Q	K	L	C	V	F	I	F
N	K	E	I	L	K	V	Q	K	L	C	V	F	I	F
N	K	E	I	L	K	V	Q	K	L	C	V	F	I	F
N	K	E	I	L	K	V	Q	K	L	C	V	F	I	F
N	K	E	I	L	K	V	Q	K	L	C	V	F	I	F
N	K	E	I	L	K	V	Q	K	L	C	V	F	I	F
N	K	E	I	L	K	V	Q	K	L	C	V	F	I	F
N	K	E	I	L	K	V	Q	K	L	C	V	F	I	F
N	K	E	I	L	K	V	Q	K	L	C	V	F	I	F
N	K	E	I	L	K	V	Q	K	L	C	V	F	I	F
N	K	E	I	L	K	V	Q	K	L	C	V	F	I	F
N	K	E	I	L	K	V	Q	K	L	C	V	F	I	F
N	K	E	I	L	K	V	Q	K	L	C	V	F	I	F
N	K	E	I	L	K	V	Q	K	L	C	V	F	I	F
N	K	E	I	L	K	V	Q	K	L	C	V	F	I	F
N	K	E	I	L	K	V	Q	K	L	C	V	F	I	F
N	K	E	I	L	K	V	Q	K	L	C	V	F	I	F
N	K	E	I	L	K	V	Q	K	L	C	V	F	I	F
N	K	E	I	L	K	V	Q	K	L	C	V	F	I	F
N	K	E	I	L	K	V	Q	K	L	C	V	F	I	F
N	K	E	I	L	K	V	Q	K	L	C	V	F	I	F
N	K	E	I	L	K	V	Q	K	L	C	V	F	I	F
N	K	E	I	L	K	V	Q	K	L	C	V	F	I	F
N	K	E	I	L	K	V	Q	K	L	C	V	F	I	F
N	K	E	I	L	K	V	Q	K	L	C	V	F	I	F
N	K	E	I	L	K	V	Q	K	L	C	V	F	I	F
N	K	E	I	L	K	V	Q	K	L	C	V	F	I	F
N	K	E	I	L	K	V	Q	K	L	C	V	F	I	F
N	K	E	I	L	K	V	Q	K	L	C	V	F	I	F
N	K	E	I	L	K	V	Q	K	L	C	V	F	I	F
N	K	E	I	L	K	V	Q	K	L	C	V	F	I	F
N	K	E	I	L	K	V	Q	K	L	C	V	F	I	F
N	K	E	I	L	K	V	Q	K	L	C	V	F	I	F
N	K	E	I	L	K	V	Q	K	L	C	V	F	I	F
N	K	E	I	L	K	V	Q	K	L	C	V	F	I	F
N	K	E	I	L	K	V	Q	K	L	C	V	F	I	F
N	K	E	I	L	K	V	Q	K	L	C	V	F	I	F
N	K	E	I	L	K	V	Q	K	L	C	V	F	I	F

2	4	6	15	18	34	36
D	I	D	I	E	I	Y
D	I	D	I	E	I	Y
D	I	D	I	E	I	D
D	I	D	I	E	I	D
D	I	D	I	E	I	D
D	I	D	I	E	I	D
D	I	D	I	E	I	D
D	I	D	I	E	I	D
D	I	D	I	E	I	D
D	I	D	I	E	I	D
D	I	D	I	E	I	D
D	I	D	I	E	I	D
D	I	D	I	E	I	D
D	I	D	I	E	I	D
D	I	D	I	E	I	D
D	I	D	I	E	I	D
D	I	D	I	E	I	D
D	I	D	I	E	I	D
D	I	D	I	E	I	D
D	I	D	I	E	I	D
D	I	D	I	E	I	D
D	I	D	I	E	I	D
D	I	D	I	E	I	D
D	I	D	I	E	I	D
D	I	D	I	E	I	D
D	I	D	I	E	I	D
D	I	D	I	E	I	D
D	I	D	I	E	I	D
D	I	D	I	E	I	D
D	I	D	I	E	I	D
D	I	D	I	E	I	Y
D	I	D	I	E	I	Y
D	I	D	I	E	I	Y
D	I	D	I	E	I	Y
D	I	D	I	E	I	Y
D	I	D	I	E	I	Y
D	I	D	I	E	I	Y
D	I	D	I	E	I	Y
D	I	D	I	E	I	Y
D	I	D	I	E	I	Y
D	I	D	I	E	I	Y
D	I	D	I	E	I	Y
D	I	D	I	E	I	Y
D	I	D	I	E	I	Y
D	I	D	I	E	I	Y
D	I	D	I	E	I	Y
D	I	D	I	E	I	Y
D	I	D	I	E	I	Y
D	I	D	I	E	I	Y
D	I	D	I	E	I	Y
D	I	D	I	E	I	Y
D	I	D	I	E	I	Y
D	I	D	I	E	I	Y
D	I	D	I	E	I	Y
D	I	D	I	E	I	Y
D	I	D	I	E	I	Y
D	I	D	I	E	I	Y
D	I	D	I	E	I	Y
D	I	D	I	E	I	Y
D	I	D	I	E	I	Y
D	I	D	I	E	I	Y
D	I	D	I	E	I	Y
D	I	D	I	E	I	Y

N	K	E	I	L	K	V	Q	K	L	C	V	F	I	F	D	I	D	I	E	I	Y
N	K	E	I	L	K	V	Q	K	L	C	V	F	I	F	D	I	D	I	E	I	Y
N	K	E	I	L	K	V	Q	K	L	C	V	F	I	F	D	I	D	I	E	I	Y
N	K	E	I	L	K	V	Q	K	L	C	V	F	I	F	D	I	D	I	E	I	Y
N	K	E	I	L	K	V	Q	K	L	C	V	F	I	F	D	I	D	I	E	I	Y
N	K	E	I	L	K	V	Q	K	L	C	V	F	I	F	D	I	D	I	E	I	Y
N	K	E	I	L	K	V	Q	K	L	C	V	F	I	F	D	I	D	I	E	I	Y
N	K	E	I	L	K	V	Q	K	L	C	V	F	I	F	D	I	D	I	E	I	Y
N	K	E	I	L	K	V	Q	K	L	C	V	F	I	F	D	I	D	I	E	I	Y
N	K	E	I	L	K	V	Q	K	L	C	V	F	I	F	D	I	D	I	E	I	Y
N	K	E	I	L	K	V	Q	K	L	C	V	F	I	F	D	I	D	I	E	I	Y
N	K	E	I	L	K	V	Q	K	L	C	V	F	I	F	D	I	D	I	E	I	Y
N	K	E	I	L	K	V	Q	K	L	C	V	F	I	F	D	I	D	I	E	I	Y
N	K	E	I	L	K	V	Q	K	L	C	V	F	I	F	D	I	D	I	E	I	Y
N	K	E	I	L	K	V	Q	K	L	C	V	F	I	F	D	I	D	I	E	I	Y
N	K	E	I	L	K	V	Q	K	L	C	V	F	I	F	D	I	D	I	E	I	Y
N	K	E	I	L	K	V	Q	K	L	C	V	F	I	F	D	I	D	I	E	I	Y
N	K	E	I	L	K	V	Q	K	L	C	V	F	I	F	D	I	D	I	E	I	Y
N	K	E	I	L	K	V	Q	K	L	C	V	F	I	F	D	I	D	I	E	I	Y
N	K	E	I	L	K	V	Q	K	L	C	V	F	I	F	D	I	D	I	E	I	Y
N	K	E	I	L	K	V	Q	K	L	C	V	F	I	F	D	I	D	I	E	I	Y
N	K	E	I	L	K	V	Q	K	L	C	V	F	I	F	D	I	D	I	E	I	Y
N	K	E	I	L	K	V	Q	K	L	C	V	F	I	F	D	I	D	I	E	I	Y
N	K	E	I	L	K	V	Q	K	L	C	V	F	I	F	D	I	D	I	E	I	Y
N	K	E	I	L	K	V	Q	K	L	C	V	F	I	F	D	I	D	I	E	I	Y
N	K	E	I	L	K	V	Q	K	L	C	V	F	I	F	D	I	D	I	E	I	Y
N	K	E	I	L	K	V	Q	K	L	C	V	F	I	F	D	I	D	I	E	I	Y
N	K	E	I	L	K	V	Q	K	L	C	V	F	I	F	D	I	D	I	E	I	Y
N	K	E	I	L	K	V	Q	K	L	C	V	F	I	F	D	I	D	I	E	I	Y
N	K	E	I	L	K	V	Q	K	L	C	V	F	I	F	D	I	D	I	E	I	Y
N	K	E	I	L	K	V	Q	K	L	C	V	F	I	F	D	I	D	I	E	I	Y
N	K	E	I	L	K	V	Q	K	L	C	V	F	I	F	D	I	D	I	E	I	Y
N	K	E	I	L	K	V	Q	K	L	C	V	F	I	F	D	I	D	I	E	I	Y
N	K	E	I	L	K	V	Q	K	L	C	V	F	I	F	D	I	D	I	E	I	Y
N	K	E	I	L	K	V	Q	K	L	C	V	F	I	F	D	I	D	I	E	I	Y
N	K	E	I	L	K	V	Q	K	L	C	V	F	I	F	D	I	D	I	E	I	Y
N	K	E	I	L	K	V	Q	K	L	C	V	F	I	F	D	I	D	I	E	I	Y
N	K	E	I	L	K	V	Q	K	L	C	V	F	I	F	D	I	D	I	E	I	Y
N	K	E	I	L	K	V	Q	K	L	C	V	F	I	F	D	I	D	I	E	I	Y
N	K	E	I	L	K	V	Q	K	L	C	V	F	I	F	D	I	D	I	E	I	Y
N	K	E	I	L	K	V	Q	K	L	C	V	F	I	F	D	I	D	I	E	I	Y
N	K	E	I	L	K	V	Q	K	L	C	V	F	I	F	D	I	D	I	E	I	Y
N	K	E	I	L	K	V	Q	K	L	C	V	F	I	F	D	I	D	I	E	I	Y
N	K	E	I	L	K	V	Q	K	L	C	V	F	I	F	D	I	D	I	E	I	Y
N	K	E	I	L	K	V	Q	K	L	C	V	F	I	F	D	I	D	I	E	I	Y
N	K	E	I	L	K	V	Q	K	L	C	V	F	I	F	D	I	D	I	E	I	Y
N	K	E	I	L	K	V	Q	K	L	C	V	F	I	F	D	I	D	I	E	I	Y
N	K	E	I	L	K	V	Q	K	L	C	V	F	I	F	D	I	D	I	E	I	Y
N	K	E	I	L	K	V	Q	K	L	C	V	F	I	F	D	I	D	I	E	I	Y
N	K	E	I	L	K	V	Q	K	L	C	V	F	I	F	D	I	D	I	E	I	Y
N	K	E	I	L	K	V	Q	K	L	C	V	F	I	F	D	I	D	I	E	I	Y
N	K	E	I	L	K	V	Q	K	L	C	V	F	I	F	D	I	D	I	E	I	Y
N	K	E	I	L	K	V	Q	K	L	C	V	F	I	F	D	I	D	I	E	I	Y
N	K	E	I	L	K	V	Q	K	L	C	V	F	I	F	D	I	D	I	E	I	Y
N	K	E	I	L	K	V	Q	K	L	C	V	F	I	F	D	I	D	I	E	I	Y
N	K	E	I	L	K	V	Q	K	L	C	V	F	I	F	D	I	D	I	E	I	Y
N	K	E	I	L	K	V	Q	K	L	C	V	F	I	F	D	I	D	I	E	I	Y
N	K	E	I	L	K	V	Q	K	L	C	V	F	I	F	D	I	D	I	E	I	Y
N	K	E	I	L	K	V	Q	K	L	C	V	F	I	F	D	I	D	I	E	I	Y
N	K	E	I	L	K	V	Q	K	L	C	V	F	I	F	D	I	D	I	E	I	Y
N	K	E	I	L	K	V	Q	K	L	C	V	F	I	F	D	I	D	I	E	I	Y
N	K	E	I	L	K	V	Q	K	L	C	V	F	I	F	D	I	D	I	E	I	Y
N	K	E	I	L	K	V	Q	K	L	C	V	F	I	F	D	I	D	I	E	I	Y
N	K	E	I	L	K	V	Q	K	L	C	V	F	I	F	D	I	D	I	E	I	Y
N	K	E	I	L	K	V	Q	K	L	C	V	F	I	F	D	I	D	I	E	I	Y
N	K	E	I	L	K	V	Q	K	L	C	V	F	I	F	D	I	D	I	E	I	Y
N	K	E	I	L	K	V	Q	K	L	C	V	F	I	F	D	I	D	I	E	I	Y

N	K	E	I	L	K	V	Q	K	L	C	V	F	I	F	D	I	D	I	E	I	Y
N	K	E	I	L	K	V	Q	K	L	C	V	F	I	F	D	I	D	I	E	I	Y
N	K	E	I	L	K	V	Q	K	L	C	V	F	I	F	D	I	D	I	E	I	Y
N	K	E	I	L	K	V	Q	K	L	C	V	F	I	F	D	I	D	I	E	I	Y
N	K	E	I	L	K	V	Q	K	L	C	V	F	I	F	D	I	D	I	E	I	Y
N	K	E	I	L	K	V	Q	K	L	C	V	F	I	F	D	I	D	I	E	I	Y
N	K	E	I	L	K	V	Q	K	L	C	V	F	I	F	D	I	D	I	E	I	Y
N	K	E	I	L	K	V	Q	K	L	C	V	F	I	F	D	I	D	I	E	I	Y
N	K	E	I	L	K	V	Q	K	L	C	V	F	I	F	D	I	D	I	E	I	Y
N	K	E	I	L	K	V	Q	K	L	C	V	F	I	F	D	I	D	I	E	I	Y
N	K	E	I	L	K	V	Q	K	L	C	V	F	I	F	D	I	D	I	E	I	Y
N	K	E	I	L	K	V	Q	K	L	C	V	F	I	F	D	I	D	I	E	I	Y
N	K	E	I	L	K	V	Q	K	L	C	V	F	I	F	D	I	D	I	E	I	Y
N	K	E	I	L	K	V	Q	K	L	C	V	F	I	F	D	I	D	I	E	I	Y
N	K	E	I	L	K	V	Q	K	L	C	V	F	I	F	D	I	D	I	E	I	Y
N	K	E	I	L	K	V	Q	K	L	C	V	F	I	F	D	I	D	I	E	I	Y
N	K	E	I	L	K	V	Q	K	L	C	V	F	I	F	D	I	D	I	E	I	Y
N	K	E	I	L	K	V	Q	K	L	C	V	F	I	F	D	I	D	I	E	I	Y
N	K	E	I	L	K	V	Q	K	L	C	V	F	I	F	D	I	D	I	E	I	Y
N	K	E	I	L	K	V	Q	K	L	C	V	F	I	F	D	I	D	I	E	I	Y
N	K	E	I	L	K	V	Q	K	L	C	V	F	I	F	D	I	D	I	E	I	Y
N	K	E	I	L	K	V	Q	K	L	C	V	F	I	F	D	I	D	I	E	I	Y
N	K	E	I	L	K	V	Q	K	L	C	V	F	I	F	D	I	D	I	E	I	Y
N	K	E	I	L	K	V	Q	K	L	C	V	F	I	F	D	I	D	I	E	I	Y
N	K	E	I	L	K	V	Q	K	L	C	V	F	I	F	D	I	D	I	E	I	Y
N	K	E	I	L	K	V	Q	K	V	C	V	F	I	F	D	I	D	I	E	I	Y
N	K	E	I	L	K	V	Q	K	L	C	V	F	I	F	D	I	D	I	E	I	Y
N	K	E	I	L	K	V	Q	K	L	C	V	F	I	F	D	I	D	I	E	I	Y
N	K	E	I	L	K	V	Q	K	L	C	V	F	I	F	D	I	D	I	E	I	Y
N	K	E	I	L	K	V	Q	K	L	C	V	F	I	F	D	I	D	I	E	I	Y
N	K	E	I	L	K	V	Q	K	L	C	V	F	I	F	D	I	D	I	E	I	Y
N	K	E	I	L	K	V	Q	K	L	C	V	F	I	F	D	I	D	I	E	I	Y
N	K	E	I	L	K	V	Q	K	L	C	V	F	I	F	D	I	D	I	E	I	Y
N	K	E	I	L	K	V	Q	K	L	C	V	F	I	F	D	I	D	I	E	I	Y
N	K	E	I	L	K	V	Q	K	L	C	V	F	I	F	D	I	D	I	E	I	Y
N	K	E	I	L	K	V	Q	K	L	C	V	F	I	F	D	I	D	I	E	I	Y
N	K	E	I	L	K	V	Q	K	L	C	V	F	I	F	D	I	D	I	E	I	Y
N	K	E	I	L	K	V	Q	K	L	C	V	F	I	F	D	I	D	I	E	I	Y
N	K	E	I	L	K	V	Q	K	L	C	V	F	I	F	D	I	D	I	E	I	Y
N	K	E	I	L	K	V	Q	K	L	C	V	F	I	F	D	I	D	I	E	I	Y
N	K	E	I	L	K	V	Q	K	L	C	V	F	I	F	D	I	D	I	E	I	Y
N	K	E	I	L	K	V	Q	K	L	C	V	F	I	F	D	I	D	I	E	I	Y
N	K	E	I	L	K	V	Q	K	L	C	V	F	I	F	D	I	D	I	E	I	Y
N	K	E	I	L	K	V	Q	K	L	C	V	F	I	F	D	I	D	I	E	I	Y
N	K	E	I	L	K	V	Q	K	L	C	V	F	I	F	D	I	D	I	E	I	Y
N	K	E	I	L	K	V	Q	K	L	C	V	F	I	F	D	I	D	I	E	I	Y
N	K	E	I	L	K	V	Q	K	L	C	V	F	I	F	D	I	D	I	E	I	Y
N	K	E	I	L	K	V	Q	K	L	C	V	F	I	F	D	I	D	I	E	I	Y
N	K	E	I	L	K	V	Q	K	L	C	V	F	I	F	D	I	D	I	E	I	Y
N	K	E	I	L	K	V	Q	K	L	C	V	F	I	F	D	I	D	I	E	I	Y
N	K	E	I	L	K	V	Q	K	L	C	V	F	I	F	D	I	D	I	E	I	Y
N	K	E	I	L	K	V	Q	K	L	C	V	F	I	F	D	I	D	I	E	I	Y
N	K	E	I	L	K	V	Q	K	L	C	V	F	I	F	D	I	D	I	E	I	Y
N	K	E	I	L	K	V	Q	K	L	C	V	F	I	F	D	I	D	I	E	I	Y
N	K	E	I	L	K	V	Q	K	L	C	V	F	I	F	D	I	D	I	E	I	Y
N	K	E	I	L	K	V	Q	K	L	C	V	F	I	F	D	I	D	I	E	I	Y
N	K	E	I	L	K	V	Q	K	L	C	V	F	I	F	D	I	D	I	E	I	Y
N	K	E	I	L	K	V	Q	K	L	C	V	F	I	F	D	I	D	I	E	I	Y
N	K	E	I	L	K	V	Q	K	L	C	V	F	I	F	D	I	D	I	E	I	Y
N	K	E	I	L	K	V	Q	K	L	C	V	F	I	F	D	I	D	I	E	I	Y
N	K	E	I	L	K	V	Q	K	L	C	V	F	I	F	D	I	D	I	E	I	Y
N	K	E	I	L	K	V	Q	K	L	C	V	F	I	F	D	I	D	I	E	I	Y
N	K	E	I	L	K	V	Q	K	L	C	V	F	I	F	D	I	D	I	E	I	Y
N	K	E	I	L	K	V	Q	K	L	C	V	F	I	F	D	I	D	I	E	I	Y
N	K	E	I	L	K	V	Q	K	L	C	V	F	I	F	D	I	D	I	E	I	Y
N	K	E	I	L	K	V	Q	K	L	C	V	F	I	F	D	I	D	I	E	I	Y
N	K	E	I	L	K	V	Q	K	L	C	V	F	I	F	D	I	D	I	E	I	Y

N	K	E	I	L	K	V	Q	K	L	C	V	F	I	F	D	I	D	I	E	I	Y
N	K	E	I	L	K	V	Q	K	L	C	V	F	I	F	D	I	D	I	E	I	Y
N	K	E	I	L	K	V	Q	K	L	C	V	F	I	F	D	I	D	I	E	I	Y
N	K	E	I	L	K	V	Q	K	L	C	V	F	I	F	D	I	D	I	E	I	Y
N	K	E	I	L	K	V	Q	K	L	C	V	F	I	F	D	I	D	I	E	I	Y
N	K	E	I	L	K	V	Q	K	L	C	V	F	I	F	D	I	D	I	E	I	Y
N	K	E	I	L	K	V	Q	K	L	C	V	F	I	F	D	I	D	I	E	I	Y
N	K	E	I	L	K	V	Q	K	L	C	V	F	I	F	D	I	D	I	E	I	Y
N	K	E	I	L	K	V	Q	K	L	C	V	F	I	F	D	I	D	I	E	I	Y
N	K	E	I	L	K	V	Q	K	L	C	V	F	I	F	D	I	D	I	E	I	Y
N	K	E	I	L	K	V	Q	K	L	C	V	F	I	F	D	I	D	I	E	I	Y
N	K	E	I	L	K	V	Q	K	L	C	V	F	I	F	D	I	D	I	E	I	Y
N	K	E	I	L	K	V	Q	K	L	C	V	F	I	F	D	I	D	I	E	I	Y
N	K	E	I	L	K	V	Q	K	L	C	V	F	I	F	D	I	D	I	E	I	Y
N	K	E	I	L	K	V	Q	K	L	C	V	F	I	F	D	I	D	I	E	I	Y
N	K	E	I	L	K	V	Q	K	L	C	V	F	I	F	D	I	D	I	E	I	Y
N	K	E	I	L	K	V	Q	K	L	C	V	F	I	F	D	I	D	I	E	I	Y
N	K	E	I	L	K	V	Q	K	L	C	V	F	I	F	D	I	D	I	E	I	Y
N	K	E	I	L	K	V	Q	K	L	C	V	F	I	F	D	I	D	I	E	I	Y
N	K	E	I	L	K	V	Q	K	L	C	V	F	I	F	D	I	D	I	E	I	Y
N	K	E	I	L	K	V	Q	K	L	C	V	F	I	F	D	I	D	I	E	I	Y
N	K	E	I	L	K	V	Q	K	L	C	V	F	I	F	D	I	D	I	E	I	Y
N	K	E	I	L	K	V	Q	K	L	C	V	F	I	F	D	I	D	I	E	I	Y
N	K	E	I	L	K	V	Q	K	L	C	V	F	I	F	D	I	D	I	E	I	Y
N	K	E	I	L	K	V	Q	K	L	C	V	F	I	F	D	I	D	I	E	I	Y
N	K	E	I	L	K	V	Q	K	L	C	V	F	I	F	D	I	D	I	E	I	Y
N	K	E	I	L	K	V	Q	K	L	C	V	F	I	F	D	I	D	I	E	I	Y
N	K	E	I	L	K	V	Q	K	L	C	V	F	I	F	D	I	D	I	E	I	Y
N	K	E	I	L	K	V	Q	K	L	C	V	F	I	F	D	I	D	I	E	I	Y
N	K	E	I	L	K	V	Q	K	L	C	V	F	I	F	D	I	D	I	E	I	Y
N	K	E	I	L	K	V	Q	K	L	C	V	F	I	F	D	I	D	I	E	I	Y
N	K	E	I	L	K	V	Q	K	L	C	V	F	I	F	D	I	D	I	E	I	Y
N	K	E	I	L	K	V	Q	K	L	C	V	F	I	F	D	I	D	I	E	I	Y
N	K	E	I	L	K	V	Q	K	L	C	V	F	I	F	D	I	D	I	E	I	Y
N	K	E	I	L	K	V	Q	K	L	C	V	F	I	F	D	I	D	I	E	I	Y
N	K	E	I	L	K	V	Q	K	L	C	V	F	I	F	D	I	D	I	E	I	Y
N	K	E	I	L	K	V	Q	K	L	C	V	F	I	F	D	I	D	I	E	I	Y
N	K	E	I	L	K	V	Q	K	L	C	V	F	I	F	D	I	D	I	E	I	Y
N	K	E	I	L	K	V	Q	K	L	C	V	F	I	F	D	I	D	I	E	I	Y
N	K	E	I	L	K	V	Q	K	L	C	V	F	I	F	D	I	D	I	E	I	Y
N	K	E	I	L	K	V	Q	K	L	C	V	F	I	F	D	I	D	I	E	I	Y
N	K	E	I	L	K	V	Q	K	L	C	V	F	I	F	D	I	D	I	E	I	Y
N	K	E	I	L	K	V	Q	K	L	C	V	F	I	F	D	I	D	I	E	I	Y
N	K	E	I	L	K	V	Q	K	L	C	V	F	I	F	D	I	D	I	E	I	Y
N	K	E	I	L	K	V	Q	K	L	C	V	F	I	F	D	I	D	I	E	I	Y
N	K	E	I	L	K	V	Q	K	L	C	V	F	I	F	D	I	D	I	E	I	Y
N	K	E	I	L	K	V	Q	K	L	C	V	F	I	F	D	I	D	I	E	I	Y
N	K	E	I	L	K	V	Q	K	L	C	V	F	I	F	D	I	D	I	E	I	Y
N	K	E	I	L	K	V	Q	K	L	C	V	F	I	F	D	I	D	I	E	I	Y
N	K	E	I	L	K	V	Q	K	L	C	V	F	I	F	D	I	D	I	E	I	Y
N	K	E	I	L	K	V	Q	K	L	C	V	F	I	F	D	I	D	I	E	I	Y
N	K	E	I	L	K	V	Q	K	L	C	V	F	I	F	D	I	D	I	E	I	Y
N	K	E	I	L	K	V	Q	K	L	C	V	F	I	F	D	I	D	I	E	I	Y
N	K	E	I	L	K	V	Q	K	L	C	V	F	I	F	D	I	D	I	E	I	Y
N	K	E	I	L	K	V	Q	K	L	C	V	F	I	F	D	I	D	I	E	I	Y
N	K	E	I	L	K	V	Q	K	L	C	V	F	I	F	D	I	D	I	E	I	Y
N	K	E	I	L	K	V	Q	K	L	C	V	F	I	F	D	I	D	I	E	I	Y
N	K	E	I	L	K	V	Q	K	L	C	V	F	I	F	D	I	D	I	E	I	Y
N	K	E	I	L	K	V	Q	K	L	C	V	F	I	F	D	I	D	I	E	I	Y
N	K	E	I	L	K	V	Q	K	L	C	V	F	I	F	D	I	D	I	E	I	Y
N	K	E	I	L	K	V	Q	K	L	C	V	F	I	F	D	I	D	I	E	I	Y
N	K	E	I	L	K	V	Q	K	L	C	V	F	I	F	D	I	D	I	E	I	Y
N	K	E	I	L	K	V	Q	K	L	C	V	F	I	F	D	I	D	I	E	I	Y
N	K	E	I	L	K	V	Q	K	L	C	V	F	I	F	D	I	D	I	E	I	Y
N	K	E	I	L	K	V	Q	K	L	C	V	F	I	F	D	I	D	I	E	I	Y
N	K	E	I	L	K	V	Q	K	L	C	V	F	I	F	D	I	D	I	E	I	Y
N	K	E	I	L	K	V	Q	K	L	C	V	F	I	F	D	I	D	I	E	I	Y

N	K	E	I	L	K	V	Q	K	L	C	V	F	I	F	D	I	D	I	E	I	Y
N	K	E	I	L	K	V	Q	K	L	C	V	F	I	F	D	I	D	I	E	I	Y
N	K	E	I	L	K	V	Q	K	L	C	V	F	I	F	D	I	D	I	E	I	Y
N	K	E	I	L	K	V	Q	K	L	C	V	F	I	F	D	I	D	I	E	I	Y
N	K	E	I	L	K	V	Q	K	L	C	V	F	I	F	D	I	D	I	E	I	Y
N	K	E	I	L	K	V	Q	K	L	C	V	F	I	F	D	I	D	I	E	I	Y
N	K	E	I	L	K	V	Q	K	L	C	V	F	I	F	D	I	D	I	E	I	Y
N	K	E	I	L	K	V	Q	K	L	C	V	F	I	F	D	I	D	I	E	I	Y
N	K	E	I	L	K	V	Q	K	L	C	V	F	I	F	D	I	D	I	E	I	Y
N	K	E	I	L	K	V	Q	K	L	C	V	F	I	F	D	I	D	I	E	I	Y
N	K	E	I	L	K	V	Q	K	L	C	V	F	I	F	D	I	D	I	E	I	Y
N	K	E	I	L	K	V	Q	K	L	C	V	F	I	F	D	I	D	I	E	I	Y
N	K	E	I	L	K	V	Q	K	L	C	V	F	I	F	D	I	D	I	E	I	Y
N	K	E	I	L	K	V	Q	K	L	C	V	F	I	F	D	I	D	I	E	I	Y
N	K	E	I	L	K	V	Q	K	L	C	V	F	I	F	D	I	D	I	E	I	Y
N	K	E	I	L	K	V	Q	K	L	C	V	F	I	F	D	I	D	I	E	I	Y
N	K	E	I	L	K	V	Q	K	L	C	V	F	I	F	D	I	D	I	E	I	Y
N	K	E	I	L	K	V	Q	K	L	C	V	F	I	F	D	I	D	I	E	I	Y
N	K	E	I	L	K	V	Q	K	L	C	V	F	I	F	D	I	D	I	E	I	Y
N	K	E	I	L	K	V	Q	K	L	C	V	F	I	F	D	I	D	I	E	I	Y
Q	S	P	K	S	R	Y	K	I	L	M	L	Y	F	V	A	S	G	V	K	M	Y
Q	S	P	K	S	R	Y	K	I	L	M	L	Y	F	V	A	S	G	V	K	M	Y
Q	S	P	K	S	R	Y	K	I	L	M	L	Y	F	V	A	S	G	V	K	M	Y
Q	S	P	K	S	R	Y	K	I	L	M	L	Y	F	V	A	S	G	V	K	M	Y
Q	S	P	K	S	R	Y	K	I	L	M	L	Y	F	V	A	S	G	V	K	M	Y
Q	S	P	K	S	R	Y	K	I	L	M	L	Y	F	V	A	S	G	V	K	M	Y
Q	S	P	K	S	R	Y	K	I	L	M	L	Y	F	V	A	S	G	V	K	M	Y
Q	S	P	K	S	R	Y	K	I	L	M	L	Y	F	V	A	S	G	V	K	M	Y
N	K	E	I	L	K	V	Q	K	L	C	V	F	I	F	D	I	D	I	E	I	Y
N	K	E	I	L	K	V	Q	K	L	C	V	F	I	F	D	I	D	I	E	I	Y
N	K	E	I	L	K	V	Q	K	L	C	V	F	I	F	D	I	D	I	E	I	Y
N	K	E	I	L	K	V	Q	K	L	C	V	F	I	F	D	I	D	I	E	I	Y
N	K	E	I	L	K	V	Q	K	L	C	V	F	I	F	D	I	D	I	E	I	Y
N	K	E	I	L	K	V	Q	K	L	C	V	F	I	F	D	I	D	I	E	I	Y
N	K	E	I	L	K	V	Q	K	L	C	V	F	I	F	D	I	D	I	E	I	Y
N	K	E	I	L	K	V	Q	K	L	C	V	F	I	F	D	I	D	I	E	I	Y
N	K	E	I	L	K	V	Q	K	L	C	V	F	I	F	D	I	D	I	E	I	Y
N	K	E	I	L	K	V	Q	K	L	C	V	F	I	F	D	I	D	I	E	I	Y
N	K	E	I	L	K	V	Q	K	L	C	V	F	I	F	D	I	D	I	E	I	Y
N	K	E	I	L	K	V	Q	K	L	C	V	F	I	F	D	I	D	I	E	I	Y
N	K	E	I	L	K	V	Q	K	L	C	V	F	I	F	D	I	D	I	E	I	Y
N	K	E	I	L	K	V	Q	K	L	C	V	F	I	F	D	I	D	I	E	I	Y
N	K	E	I	L	K	V	Q	K	L	C	V	F	I	F	D	I	D	I	E	I	Y
N	K	E	I	L	K	V	Q	K	L	C	V	F	I	F	D	I	D	I	E	I	Y
N	K	E	I	L	K	V	Q	K	L	C	V	F	I	F	D	I	D	I	E	I	Y
N	K	E	I	L	K	V	Q	K	L	C	V	F	I	F	D	I	D	I	E	I	Y
N	K	E	I	L	K	V	Q	K	L	C	V	F	I	F	D	I	D	I	E	I	Y
N	K	E	I	L	K	V	Q	K	L	C	V	F	I	F	D	I	D	I	E	I	Y
N	K	E	I	L	K	V	Q	K	L	C	V	F	I	F	D	I	D	I	E	I	Y
N	K	E	I	L	K	V	Q	K	L	C	V	F	I	F	D	I	D	I	E	I	Y
N	K	E	I	L	K	V	Q	K	L	C	V	F	I	F	D	I	D	I	E	I	Y
N	K	E	I	L	K	V	Q	K	L	C	V	F	I	F	D	I	D	I	E	I	Y
N	K	E	I	L	K	V	Q	K	L	C	V	F	I	F	D	I	D	I	E	I	Y
N	K	E	I	L	K	V	Q	K	L	C	V	F	I	F	D	I	D	I	E	I	Y
N	K	E	I	L	K	V	Q	K	L	C	V	F	I	F	D	I	D	I	E	I	Y
N	K	E	I	L	K	V	Q	K	L	C	V	F	I	F	D	I	D	I	E	I	Y
N	K	E	I	L	K	V	Q	K	L	C	V	F	I	F	D	I	D	I	E	I	Y
N	K	E	I	L	K	V	Q	K	L	C	V	F	I	F	D	I	D	I	E	I	Y
N	K	E	I	L	K	V	Q	K	L	C	V	F	I	F	D	I	D	I	E	I	Y
N	K	E	I	L	K	V	Q	K	L	C	V	F	I	F	D	I	D	I	E	I	Y
N	K	E	I	L	K	V	Q	K	L	C	V	F	I	F	D	I	D	I	E	I	Y
N	K	E	I	L	K	V	Q	K	L	C	V	F	I	F	D	I	D	I	E	I	Y
N	K	E	I	L	K	V	Q	K	L	C	V	F	I	F	D	I	D	I	E	I	Y
N	K	E	I	L	K	V	Q	K	L	C	V	F	I	F	D	I	D	I	E	I	Y
N	K	E	I	L	K	V	Q	K	L	C	V	F	I	F	D	I	D	I	E	I	Y
N	K	E	I	L	K	V	Q	K	L	C	V	F	I	F	D	I	D	I	E	I	Y
N	K	E	I	L	K	V	Q	K	L	C	V	F	I	F	D	I	D	I	E	I	Y

N	K	E	I	L	K	V	Q	K	L	C	V	F	I	F	D	I	D	I	E	I	Y
N	K	E	I	L	K	V	Q	K	L	C	V	F	I	F	D	I	D	I	E	I	Y
N	K	E	I	L	K	V	Q	K	L	C	V	F	I	F	D	I	D	I	E	I	Y
N	K	E	I	L	K	V	Q	K	L	C	V	F	I	F	D	I	D	I	E	I	Y
N	K	E	I	L	K	V	Q	K	L	C	V	F	I	F	D	I	D	I	E	I	Y
N	K	E	I	L	K	V	Q	K	L	C	V	F	I	F	D	I	D	I	E	I	Y
N	K	E	I	L	K	V	Q	K	L	C	V	F	I	F	D	I	D	I	E	I	Y
N	K	E	I	L	K	V	Q	K	L	C	V	F	I	F	D	I	D	I	E	I	Y
N	K	E	I	L	K	V	Q	K	L	C	V	F	I	F	D	I	D	I	E	I	Y
N	K	E	I	L	K	V	Q	K	L	C	V	F	I	F	D	I	D	I	E	I	Y
N	K	E	I	L	K	V	Q	K	L	C	V	F	I	F	D	I	D	I	E	I	Y
N	K	E	I	L	K	V	Q	K	L	C	V	F	I	F	D	I	D	I	E	I	Y
N	K	E	I	L	K	V	Q	K	L	C	V	F	I	F	D	I	D	I	E	I	Y
N	K	E	I	L	K	V	Q	K	L	C	V	F	I	F	D	I	D	I	E	I	Y
N	K	E	I	L	K	V	Q	K	L	C	V	F	I	F	D	I	D	I	E	I	Y
N	K	E	I	L	K	V	Q	K	L	C	V	F	I	F	D	I	D	I	E	I	Y
N	K	E	I	L	K	V	Q	K	L	C	V	F	I	F	D	I	D	I	E	I	Y
N	K	E	I	L	K	V	Q	K	L	C	V	F	I	F	D	I	D	I	E	I	Y
N	K	E	I	L	K	V	Q	K	L	C	V	F	I	F	D	I	D	I	E	I	Y
N	K	E	I	L	K	V	Q	K	L	C	V	F	I	F	D	I	D	I	E	I	Y
N	K	E	I	L	K	V	Q	K	L	C	V	F	I	F	D	I	D	I	E	I	Y
N	K	E	I	L	K	V	Q	K	L	C	V	F	I	F	D	I	D	I	E	I	Y
N	K	E	I	L	K	V	Q	K	L	C	V	F	I	F	D	I	D	I	E	I	Y
N	K	E	I	L	K	V	Q	K	L	C	V	F	I	F	D	I	D	I	E	I	Y
N	K	E	I	L	K	V	Q	K	L	C	V	F	I	F	D	I	D	I	E	I	Y
N	K	E	I	L	K	V	Q	K	L	C	V	F	I	F	D	I	D	I	E	I	Y
N	K	E	I	L	K	V	Q	K	L	C	V	F	I	F	D	I	D	I	E	I	Y
N	K	E	I	L	K	V	Q	K	L	C	V	F	I	F	D	I	D	I	E	I	Y
N	K	E	I	L	K	V	Q	K	L	C	V	F	I	F	D	I	D	I	E	I	Y
N	K	E	I	L	K	V	Q	K	L	C	V	F	I	F	D	I	D	I	E	I	Y
N	K	E	I	L	K	V	Q	K	L	C	V	F	I	F	D	I	D	I	E	I	Y
N	K	E	I	L	K	V	Q	K	L	C	V	F	I	F	D	I	D	I	E	I	Y
N	K	E	I	L	K	V	Q	K	L	C	V	F	I	F	D	I	D	I	E	I	Y
N	K	E	I	L	K	V	Q	K	L	C	V	F	I	F	D	I	D	I	E	I	Y
N	K	E	I	L	K	V	Q	K	L	C	V	F	I	F	D	I	D	I	E	I	Y
N	K	E	I	L	K	V	Q	K	L	C	V	F	I	F	D	I	D	I	E	I	Y
N	K	E	I	L	K	V	Q	K	L	C	V	F	I	F	D	I	D	I	E	I	Y
N	K	E	I	L	K	V	Q	K	L	C	V	F	I	F	D	I	D	I	E	I	Y
N	K	E	I	L	K	V	Q	K	L	C	V	F	I	F	D	I	D	I	E	I	Y
N	K	E	I	L	K	V	Q	K	L	C	V	F	I	F	D	I	D	I	E	I	Y
N	K	E	I	L	K	V	Q	K	L	C	V	F	I	F	D	I	D	I	E	I	Y
N	K	E	I	L	K	V	Q	K	L	C	V	F	I	F	D	I	D	I	E	I	Y
N	K	E	I	L	K	V	Q	K	L	C	V	F	I	F	D	I	D	I	E	I	Y
N	K	E	I	L	K	V	Q	K	L	C	V	F	I	F	D	I	D	I	E	I	Y
N	K	E	I	L	K	V	Q	K	L	C	V	F	I	F	D	I	D	I	E	I	Y
N	K	E	I	L	K	V	Q	K	L	C	V	F	I	F	D	I	D	I	E	I	Y
N	K	E	I	L	K	V	Q	K	L	C	V	F	I	F	D	I	D	I	E	I	Y
N	K	E	I	L	K	V	Q	K	L	C	V	F	I	F	D	I	D	I	E	I	Y
N	K	E	I	L	K	V	Q	K	L	C	V	F	I	F	D	I	D	I	E	I	Y
N	K	E	I	L	K	V	Q	K	L	C	V	F	I	F	D	I	D	I	E	I	Y
N	K	E	I	L	K	V	Q	K	L	C	V	F	I	F	D	I	D	I	E	I	Y
N	K	E	I	L	K	V	Q	K	L	C	V	F	I	F	D	I	D	I	E	I	Y
N	K	E	I	L	K	V	Q	K	L	C	V	F	I	F	D	I	D	I	E	I	Y
N	K	E	I	L	K	V	Q	K	L	C	V	F	I	F	D	I	D	I	E	I	Y
N	K	E	I	L	K	V	Q	K	L	C	V	F	I	F	D	I	D	I	E	I	Y
N	K	E	I	L	K	V	Q	K	L	C	V	F	I	F	D	I	D	I	E	I	Y
N	K	E	I	L	K	V	Q	K	L	C	V	F	I	F	D	I	D	I	E	I	Y
N	K	E	I	L	K	V	Q	K	L	C	V	F	I	F	D	I	D	I	E	I	Y
N	K	E	I	L	K	V	Q	K	L	C	V	F	I	F	D	I	D	I	E	I	Y
N	K	E	I	L	K	V	Q	K	L	C	V	F	I	F	D	I	D	I	E	I	Y
N	K	E	I	L	K	V	Q	K	L	C	V	F	I	F	D	I	D	I	E	I	Y
N	K	E	I	L	K	V	Q	K	L	C	V	F	I	F	D	I	D	I	E	I	Y
N	K	E	I	L	K	V	Q	K	L	C	V	F	I	F	D	I	D	I	E	I	Y
N	K	E	I	L	K	V	Q	K	L	C	V	F	I	F	D	I	D	I	E	I	Y
N	K	E	I	L	K	V	Q	K	L	C	V	F	I	F	D	I	D	I	E	I	Y
N	K	E	I	L	K	V	Q	K	L	C	V	F	I	F	D	I	D	I	E	I	Y
N	K	E	I	L	K	V	Q	K	L	C	V	F	I	F	D	I	D	I	E	I	Y

N	K	E	I	L	K	V	Q	K	L	C	V	F	I	F
N	K	E	I	L	K	V	Q	K	L	C	V	F	I	F
N	K	E	I	L	K	V	Q	K	L	C	V	F	I	F
N	K	E	I	L	K	V	Q	K	L	C	V	F	I	F
N	K	E	I	L	K	V	Q	K	L	C	V	F	I	F
N	K	E	I	L	K	V	Q	K	L	C	V	F	I	F
N	K	E	I	L	K	V	Q	K	L	C	V	F	I	F
N	K	E	I	L	K	V	Q	K	L	C	V	F	I	F
N	K	E	I	L	K	V	Q	K	L	C	V	F	I	F
N	K	E	I	L	K	V	Q	K	L	C	V	F	I	F

D	I	D	I	E	I	Y
D	I	D	I	E	I	Y
D	I	D	I	E	I	Y
D	I	D	I	E	I	Y
D	I	D	I	E	I	Y
D	I	D	I	E	I	Y
D	I	D	I	E	I	Y
D	I	D	I	E	I	Y
D	I	D	I	E	I	Y
D	I	D	I	E	I	Y

wciO																	
74	76	77	133	136	137	140	142	168	169	173	174	180	182	186	204	212	213
D	V	V	A	N	D	I	I	Q	D	S	S	D	N	I	L	V	L
N	I	L	T	N	G	M	V	R	N	N	N	N	S	L	V	E	F
D	V	V	A	T	D	I	I	Q	D	S	S	D	N	I	L	V	L
N	I	L	T	N	G	M	V	R	N	N	N	N	S	L	V	E	F
N	I	L	T	N	G	M	V	R	N	N	N	N	S	L	V	E	F
N	I	L	T	N	G	M	V	R	N	N	N	N	S	L	V	E	F
N	I	L	T	N	G	M	V	R	N	N	N	N	S	L	V	E	F
N	I	L	T	N	G	M	V	R	N	N	N	N	S	L	V	E	F
N	I	L	T	N	G	M	V	R	N	N	N	N	S	L	V	E	F
N	I	L	T	N	G	M	V	R	N	N	N	N	S	L	V	E	F
N	I	L	T	N	G	M	V	R	N	N	N	N	S	L	V	E	F

47	62	117	126
I	F	D	M
V	L	E	M
I	L	D	L
I	L	D	M
I	L	D	M
I	L	D	M
I	L	D	M
I	L	D	M
I	L	D	M
I	L	D	M
I	L	D	M

wciO															
76	77	133	137	140	142	168	169	173	174	180	182	186	204	212	213
V	V	A	D	I	I	Q	D	S	S	D	N	I	L	V	L
I	L	T	G	M	V	R	N	N	N	N	S	L	V	E	F
V	V	A	D	I	I	Q	D	S	S	D	N	I	L	V	L
V	V	A	D	I	I	Q	D	S	S	D	N	I	L	V	L
V	V	A	D	I	I	Q	D	S	S	D	N	I	L	V	L
V	V	A	D	I	I	Q	D	S	S	D	N	I	L	V	L
V	V	A	D	I	I	Q	D	S	S	D	N	I	L	V	L
V	V	A	D	I	I	Q	D	S	S	D	N	I	L	V	L
V	V	A	D	I	I	Q	D	S	S	D	N	I	L	V	L
V	V	A	D	I	I	Q	D	S	S	D	N	I	L	V	L
V	V	A	D	I	I	Q	D	S	S	D	N	I	L	V	L
V	V	A	D	I	I	Q	D	S	S	D	N	I	L	V	L
V	V	A	D	I	I	Q	D	S	S	D	N	I	L	V	L
V	V	A	D	I	I	Q	D	S	S	D	N	I	L	V	L
V	V	A	D	I	I	Q	D	S	S	D	N	I	L	V	L
V	V	A	D	I	I	Q	D	S	S	D	N	I	L	V	L
V	V	A	D	I	I	Q	D	S	S	D	N	I	L	V	L
V	V	A	D	I	I	Q	D	S	S	D	N	I	L	V	L
V	V	A	D	I	I	Q	D	S	S	D	N	I	L	V	L
V	V	A	D	I	I	Q	D	S	S	D	N	I	L	V	L
V	V	A	D	I	I	Q	D	S	S	D	N	I	L	V	L
V	V	A	D	I	I	Q	D	S	S	D	N	I	L	V	L
V	V	A	D	I	I	Q	D	S	S	D	N	I	L	V	L
V	V	A	D	I	I	Q	D	S	S	D	N	I	L	V	L
V	V	A	D	I	I	Q	D	S	S	D	N	I	L	V	L
V	V	A	D	I	I	Q	D	S	S	D	N	I	L	I	L
V	V	A	D	I	I	Q	D	S	S	D	N	I	L	V	L
V	V	A	D	I	I	Q	D	S	S	D	N	I	L	V	L
V	V	A	D	I	I	Q	D	S	S	D	N	I	L	V	L
V	V	A	D	I	I	Q	D	S	S	D	N	I	L	V	L
V	V	A	D	I	I	Q	D	S	S	D	N	I	L	V	L

47	62	117	128	132	134
I	F	D	M	S	N
V	L	E	T	N	K
I	F	D	T	S	N
I	F	D	T	S	N
I	F	D	T	S	N
I	F	D	T	S	N
I	F	D	T	S	N
I	F	D	T	S	N
I	F	D	T	S	N
I	F	D	T	S	N
I	F	D	T	S	N
I	F	D	T	S	N
I	F	D	T	S	N
I	F	D	T	S	N
I	F	D	T	S	N
I	F	D	T	S	N
I	F	D	T	S	N
I	F	D	T	S	N
I	F	D	T	S	N
I	F	D	T	S	N
I	F	D	T	S	N
I	F	D	T	S	N
I	F	D	T	S	N
I	F	D	T	S	N
I	F	D	T	S	N
I	F	D	T	S	N
I	F	D	T	S	N
I	F	D	T	S	N
I	F	D	T	S	N
I	F	D	T	S	N
I	F	D	T	S	N

272	286
S	V
P	I
P	I
P	I

rmlC																	
2	11	12	15	16	21	56	57	89	108	112	163	172	182	187	193	195	196
T	A	A	V	E	M	A	E	G	T	V	S	I	N	K	K	E	D
T	A	A	V	E	M	A	E	G	T	V	S	I	N	K	K	E	D
S	A	V	V	D	L	V	A	D	V	E	S	L	H	R	T	D	E
S	V	V	I	D	L	A	A	D	V	E	A	L	H	R	T	D	E

6
N
N
K
K

wciP

140	142	168	169	173	174	180	182	186	204	212	213
I	I	Q	D	S	S	D	N	I	L	V	L
M	V	R	N	N	N	N	S	L	V	E	F
I	I	Q	D	S	S	D	N	I	L	V	L
I	I	Q	D	S	S	D	N	I	L	V	L
I	I	Q	D	S	S	D	N	I	L	V	L
I	I	Q	D	S	S	D	N	I	L	V	L
I	I	Q	D	S	S	D	N	I	L	V	L
I	I	Q	D	S	S	D	N	I	L	V	L
I	I	Q	D	S	S	D	N	I	L	V	L
I	I	Q	D	S	S	D	N	I	L	V	L
I	I	Q	D	S	S	D	N	I	L	V	L
I	I	Q	D	S	S	D	N	I	L	V	L
I	I	Q	D	S	S	D	N	I	L	V	L
I	I	Q	D	S	S	D	N	I	L	V	L
I	I	Q	D	S	S	D	N	I	L	V	L
I	I	Q	D	S	S	D	N	I	L	V	L
I	I	Q	D	S	S	D	N	I	L	V	L
I	I	Q	D	S	S	D	N	I	L	V	L
I	I	Q	D	S	S	D	N	I	L	V	L
I	I	Q	D	S	S	D	N	I	L	V	L
I	I	Q	D	S	S	D	N	I	L	V	L
I	I	Q	D	S	S	D	N	I	L	V	L
I	I	Q	D	S	S	D	N	I	L	V	L
I	I	Q	D	S	S	D	N	I	L	V	L
I	I	Q	D	S	S	D	N	I	L	V	L
I	I	Q	D	S	S	D	N	I	L	V	L
I	I	Q	D	S	S	D	N	I	L	V	L

13	47	62	72	117	128	132	134	142	192
N	I	F	A	D	M	S	N	K	A
N	V	L	A	E	T	N	K	N	S
N	I	F	T	D	T	S	N	K	A
N	I	F	A	D	T	S	N	K	A
N	I	F	A	D	T	S	N	K	A
N	I	F	A	D	T	S	N	K	A
N	I	F	A	D	T	S	N	K	A
N	I	F	A	D	T	S	N	K	A
N	I	F	A	D	T	S	N	K	A
N	I	F	A	D	T	S	N	K	A
N	I	F	A	D	T	S	N	K	A
N	I	F	A	D	T	S	N	K	A
N	I	F	A	D	T	S	N	K	A
N	I	F	A	D	T	S	N	K	A
N	I	F	A	D	T	S	N	K	A
N	I	F	A	D	T	S	N	K	A
N	I	F	A	D	T	S	N	K	A
N	I	F	A	D	T	S	N	K	A
N	I	F	A	D	T	S	N	K	A
N	I	F	A	D	T	S	N	K	A
S	I	F	A	D	T	S	N	K	A
N	I	F	A	D	T	S	N	K	A
N	I	F	A	D	T	S	N	K	A
N	I	F	A	D	T	S	N	K	A
N	I	F	A	D	T	S	N	K	A
N	I	F	A	D	T	S	N	K	A
N	I	F	A	D	T	S	N	K	A

319	320	324	325	327	328	331	342	344	348	353	367	370	371	388	389	415	417	418	421	431	432	450
C	V	A	K	R	E	E	V	D	I	R	Y	L	K	E	H	E	K	N	E	N	G	F
C	V	A	K	R	E	E	V	D	I	C	Y	L	K	E	H	E	K	N	E	D	D	L
R	I	S	I	E	Q	D	I	N	V	R	W	F	I	D	Q	K	T	D	D	D	N	F
C	V	A	K	R	E	E	V	D	I	R	Y	L	K	E	H	E	K	N	E	N	G	F
C	V	A	K	R	E	E	V	D	I	R	Y	L	K	E	H	E	K	N	E	N	G	F
C	V	A	K	R	E	E	V	D	I	R	Y	L	K	E	H	E	K	N	E	N	G	F
C	V	A	K	R	E	E	V	D	I	R	Y	L	K	E	H	E	K	N	E	N	G	F
C	V	A	K	R	E	E	V	D	I	R	Y	L	K	E	H	E	K	N	E	N	G	F
C	V	A	K	R	E	E	V	D	I	R	Y	L	K	E	H	E	K	N	E	N	G	F
C	V	A	K	R	E	E	V	D	I	R	Y	L	K	E	H	E	K	N	E	N	G	F
C	V	A	K	R	E	E	V	D	I	R	Y	L	K	E	H	E	K	N	E	N	G	F
C	V	A	K	R	E	E	V	D	I	R	Y	L	K	E	H	E	K	N	E	N	G	F
C	V	A	K	R	E	E	V	D	I	R	Y	L	K	E	H	E	K	N	E	N	G	F
C	V	A	K	R	E	E	V	D	I	R	Y	L	K	E	H	E	K	N	E	N	G	F
C	V	A	K	R	E	E	V	D	I	R	Y	L	K	E	H	E	K	N	E	N	G	F
C	V	A	K	R	E	E	V	D	I	R	Y	L	K	E	H	E	K	N	E	N	G	F
C	V	A	K	R	E	E	V	D	I	R	Y	L	K	E	H	E	K	N	E	N	G	F
C	V	A	K	R	E	E	V	D	I	R	Y	L	K	E	H	E	K	N	E	N	G	F
C	V	A	K	R	E	E	V	D	I	R	Y	L	K	E	H	E	K	N	E	N	G	F
C	V	A	K	R	E	E	V	D	I	R	Y	L	K	E	H	E	K	N	E	N	G	F
C	V	A	K	R	E	E	V	D	I	R	Y	L	K	E	H	E	K	N	E	N	G	F
C	V	A	K	R	E	E	V	D	I	R	Y	L	K	E	H	E	K	N	E	N	G	F
C	V	A	K	R	E	E	V	D	I	R	Y	L	K	E	H	E	K	N	E	N	G	F
C	V	A	K	R	E	E	V	D	I	R	Y	L	K	E	H	E	K	N	E	N	G	F
C	V	A	K	R	E	E	V	D	I	R	Y	L	K	E	H	E	K	N	E	N	G	F
C	V	A	K	R	E	E	V	D	I	R	Y	L	K	E	H	E	K	N	E	N	G	F
C	V	A	K	R	E	E	V	D	I	R	Y	L	K	E	H	E	K	N	E	N	G	F
C	V	A	K	R	E	E	V	D	I	R	Y	L	K	E	H	E	K	N	E	N	G	F
C	V	A	K	R	E	E	V	D	I	R	Y	L	K	E	H	E	K	N	E	N	G	F

C	V	A	K	R	E	E	V	D	I	R	Y	L	K	E	H	E	K	N	E	N	G	F
C	V	A	K	R	E	E	V	D	I	R	Y	L	K	E	H	E	K	N	E	N	G	F

HG262

39	45	47	49	56	60	62	63
L	G	V	H	-	D	S	F
L	G	V	H	-	D	S	F
L	G	V	H	-	D	S	F
L	G	V	H	-	D	S	F
L	G	V	H	-	D	S	F
L	G	V	H	-	D	S	F
L	G	V	H	-	D	S	F
L	G	V	H	-	D	S	F
L	G	V	H	-	D	S	F
L	G	V	H	-	D	S	F
L	G	V	H	-	D	S	F
L	G	V	H	-	D	S	F
L	G	V	H	-	D	S	F
L	G	V	H	-	D	S	F
L	G	V	H	-	D	S	F
L	G	V	H	-	D	S	F
L	G	V	H	-	D	S	F
L	G	V	H	-	D	S	F
L	G	V	H	-	D	S	F
L	G	V	H	-	D	S	F
L	G	V	H	-	D	S	F
L	G	V	H	-	D	S	F
L	G	V	H	-	D	S	F
L	G	V	H	-	D	S	F
L	G	V	H	-	D	S	F
L	G	V	H	-	D	S	F
L	G	V	H	-	D	S	F
L	G	V	H	-	D	S	F
L	G	V	H	-	D	S	F
L	G	V	H	-	D	S	F
L	G	V	H	-	D	S	F
L	G	V	H	-	D	S	F
L	G	V	H	-	D	S	F
L	G	V	H	-	D	S	F
L	G	V	H	-	D	S	F
L	G	V	H	-	D	S	F
L	G	V	H	-	D	S	F
L	G	V	H	-	D	S	F
L	G	V	H	-	D	S	F
L	G	V	H	-	D	S	F
L	G	V	H	-	D	S	F
L	G	V	H	-	D	S	F
L	G	V	H	-	D	S	F
L	G	V	H	-	D	S	F
L	G	V	H	-	D	S	F
L	G	V	H	-	D	S	F
L	G	V	H	-	D	S	F
L	G	V	H	-	D	S	F
L	G	V	H	-	D	S	F
L	G	V	H	-	D	S	F
L	G	V	H	-	D	S	F
L	G	V	H	-	D	S	F
L	G	V	H	-	D	S	F
L	G	V	H	-	D	S	F
L	G	V	H	-	D	S	F
L	G	V	H	-	D	S	F
L	G	V	H	-	D	S	F
L	G	V	H	-	D	S	F
L	G	V	H	-	D	S	F
L	G	V	H	-	D	S	F
L	G	V	H	-	D	S	F
L	G	V	H	-	D	S	F
L	G	V	H	-	D	S	F

5	6	7	39	45	54	60	64	67	68	70	74	76	77
R	I	C	L	V	I	I	V	I	I	I	D	V	V
R	I	C	L	V	I	I	V	I	I	I	D	V	V
R	I	C	L	V	I	I	V	I	I	I	D	V	V
R	I	C	L	V	I	I	V	I	I	I	D	V	V
R	I	C	L	V	I	I	V	I	I	I	D	V	V
R	I	C	L	V	I	I	V	I	I	I	D	V	V
R	I	C	L	V	I	I	V	I	I	I	D	V	V
R	I	C	L	V	I	I	V	I	I	I	D	V	V
R	I	C	L	V	I	I	V	I	I	I	D	V	V
R	I	C	L	V	I	I	V	I	I	I	D	V	V
R	I	C	L	V	I	I	V	I	I	I	D	V	V
R	I	C	L	V	I	I	V	I	I	I	D	V	V
R	I	C	L	V	I	I	V	I	I	I	D	V	V
R	I	C	L	V	I	I	V	I	I	I	D	V	V
R	I	C	L	V	I	I	V	I	I	I	D	V	V
R	I	C	L	V	I	I	V	I	I	I	D	V	V
R	I	C	L	V	I	I	V	I	I	I	D	V	V
R	I	C	L	V	I	I	V	I	I	I	D	V	V
R	I	C	L	V	I	I	V	I	I	I	D	V	V
R	I	C	L	V	I	I	V	I	I	I	D	V	V
R	I	C	L	V	I	I	V	I	I	I	D	V	V
R	I	C	L	V	I	I	V	I	I	I	D	V	V
R	I	C	L	V	I	I	V	I	I	I	D	V	V
R	I	C	L	V	I	I	V	I	I	I	D	V	V
R	I	C	L	V	I	I	V	I	I	I	D	V	V
R	I	C	L	V	I	I	V	I	I	I	D	V	V
R	I	C	L	V	I	I	V	I	I	I	D	V	V
R	I	C	L	V	I	I	V	I	I	I	D	V	V
R	I	C	L	V	I	I	V	I	I	I	D	V	V
R	I	C	L	V	I	I	V	I	I	I	D	V	V
R	I	C	L	V	I	I	V	I	I	I	D	V	V
R	I	C	L	V	I	I	V	I	I	I	D	V	V
R	I	C	L	V	I	I	V	I	I	I	D	V	V
R	I	C	L	V	I	I	V	I	I	I	D	V	V
R	I	C	L	V	I	I	V	I	I	I	D	V	V
R	I	C	L	V	I	I	V	I	I	I	D	V	V
R	I	C	L	V	I	I	V	I	I	I	D	V	V
R	I	C	L	V	I	I	V	I	I	I	D	V	V
R	I	C	L	V	I	I	V	I	I	I	D	V	V
R	I	C	L	V	I	I	V	I	I	I	D	V	V
R	I	C	L	V	I	I	V	I	I	I	D	V	V
R	I	C	L	V	I	I	V	I	I	I	D	V	V
R	I	C	L	V	I	I	V	I	I	I	D	V	V
R	I	C	L	V	I	I	V	I	I	I	D	V	V
R	I	C	L	V	I	I	V	I	I	I	D	V	V
R	I	C	L	V	I	I	V	I	I	I	D	V	V
R	I	C	L	V	I	I	V	I	I	I	D	V	V
R	I	C	L	V	I	I	V	I	I	I	D	V	V
R	I	C	L	V	I	I	V	I	I	I	D	V	V
R	I	C	L	V	I	I	V	I	I	I	D	V	V
R	I	C	L	V	I	I	V	I	I	I	D	V	V
R	I	C	L	V	I	I	V	I	I	I	D	V	V
R	I	C	L	V	I	I	V	I	I	I	D	V	V
R	I	C	L	V	I	I	V	I	I	I	D	V	V
R	I	C	L	V	I	I	V	I	I	I	D	V	V
R	I	C	L	V	I	I	V	I	I	I	D	V	V
R	I	C	L	V	I	I	V	I	I	I	D	V	V
R	I	C	L	V	I	I	V	I	I	I	D	V	V
R	I	C	L	V	I	I	V	I	I	I	D	V	V
R	I	C	L	V	I	I	V	I	I	I	D	V	V
R	I	C	L	V	I	I	V	I	I	I	D	V	V
R	I	C	L	V	I	I	V	I	I	I	D	V	V
R	I	C	L	V	I	I	V	I	I	I	D	V	V

L	G	V	H	-	D	S	F	R	I	C	L	V	I	I	V	I	I	I	D	V	V
L	G	V	H	-	D	S	F	R	I	C	L	V	I	I	V	I	I	I	D	V	V
L	G	V	H	-	D	S	F	R	I	C	L	V	I	I	V	I	I	I	D	V	V
L	G	V	H	-	D	S	F	R	I	C	L	V	I	I	V	I	I	I	D	V	V
L	G	V	H	-	D	S	F	R	I	C	L	V	I	I	V	I	I	I	D	V	V
L	G	V	H	-	D	S	F	R	I	C	L	V	I	I	V	I	I	I	D	V	V
L	G	V	H	-	D	S	F	R	I	C	L	V	I	I	V	I	I	I	D	V	V
L	G	V	H	-	D	S	F	R	I	C	L	V	I	I	V	I	I	I	D	V	V
L	G	V	H	-	D	S	F	R	I	C	L	V	I	I	V	I	I	I	D	V	V
L	G	V	H	-	D	S	F	R	I	C	L	V	I	I	V	I	I	I	D	V	V
L	G	V	H	-	D	S	F	R	I	C	L	V	I	I	V	I	I	I	D	V	V
L	G	V	H	-	D	S	F	R	I	C	L	V	I	I	V	I	I	I	D	V	V
L	G	V	H	-	D	S	F	R	I	C	L	V	I	I	V	I	I	I	D	V	V
L	G	V	H	-	D	S	F	R	I	C	L	V	I	I	V	I	I	I	D	V	V
L	G	V	H	-	D	S	F	R	I	C	L	V	I	I	V	I	I	I	D	V	V
L	G	V	H	-	D	S	F	R	I	C	L	V	I	I	V	I	I	I	D	V	V
L	G	V	H	-	D	S	F	R	I	C	L	V	I	I	V	I	I	I	D	V	V
L	G	V	H	-	D	S	F	R	I	C	L	V	I	I	V	I	I	I	D	V	V
L	G	V	H	-	D	S	F	R	I	C	L	V	I	I	V	I	I	I	D	V	V
L	G	V	H	-	D	S	F	R	I	C	L	V	I	I	V	I	I	I	D	V	V
L	G	V	H	-	D	S	F	R	I	C	L	V	I	I	V	I	I	I	D	V	V
L	G	V	H	-	D	S	F	R	I	C	L	V	I	I	V	I	I	I	D	V	V
L	G	V	H	-	D	S	F	R	I	C	L	V	I	I	V	I	I	I	D	V	V
L	G	V	H	-	D	S	F	R	I	C	L	V	I	I	V	I	I	I	D	V	V
L	G	V	H	-	D	S	F	R	I	C	L	V	I	I	V	I	I	I	D	I	V
L	G	V	H	-	D	S	F	R	I	C	L	V	I	I	V	I	I	I	D	I	V
L	G	V	H	-	D	S	F	R	I	C	L	V	I	I	V	I	I	I	D	V	V
L	G	V	H	-	D	S	F	R	I	C	L	V	I	I	V	I	I	I	D	V	V
L	G	V	H	-	D	S	F	R	I	C	L	V	I	I	V	I	I	I	D	V	V
L	G	V	H	-	D	S	F	R	I	C	L	V	I	I	V	I	I	I	D	V	V
L	G	V	H	-	D	S	F	R	I	C	L	V	I	I	V	I	I	I	D	V	V
L	G	V	H	-	D	S	F	R	I	C	L	V	I	I	V	I	I	I	D	V	V
L	G	V	H	-	D	S	F	R	I	C	L	V	I	I	V	I	I	I	D	V	V
L	G	V	H	-	D	S	F	R	I	C	L	V	I	I	V	I	I	I	D	V	V
L	G	V	H	-	D	S	F	R	I	C	L	V	I	I	V	I	I	I	D	V	V
L	G	V	H	-	D	S	F	R	I	C	L	V	I	I	V	I	I	I	D	V	V
L	G	V	H	-	D	S	F	R	I	C	L	V	I	I	V	I	I	I	D	V	V
L	G	V	H	-	D	S	F	R	I	C	L	V	I	I	V	I	I	I	D	V	V
L	G	V	H	-	D	S	F	R	I	C	L	V	I	I	V	I	I	I	D	V	V
L	G	V	H	-	D	S	F	R	I	C	L	V	I	I	V	I	I	I	D	V	V
L	G	V	H	-	D	S	F	R	I	C	L	V	I	I	V	I	I	I	D	V	V
L	G	V	H	-	D	S	F	R	I	C	L	V	I	I	V	I	I	I	D	V	V
L	G	V	H	-	D	S	F	R	I	C	L	V	I	I	V	I	I	I	D	V	V
L	G	V	H	-	D	S	F	R	I	C	L	V	I	I	V	I	I	I	D	V	V
L	G	V	H	-	D	S	F	R	I	C	L	V	I	I	V	I	I	I	D	V	V
L	G	V	H	-	D	S	F	R	I	C	L	V	I	I	V	I	I	I	D	V	V
L	G	V	H	-	D	S	F	R	I	C	L	V	I	I	V	I	I	I	D	V	V
L	G	V	H	-	D	S	F	R	I	C	L	V	I	I	V	I	I	I	D	V	V
L	G	V	H	-	D	S	F	R	I	C	L	V	I	I	V	I	I	I	D	V	V
L	G	V	H	-	D	S	F	R	I	C	L	V	I	I	V	I	I	I	D	V	V
L	G	V	H	-	D	S	F	R	I	C	L	V	I	I	V	I	I	I	D	V	V
L	G	V	H	-	D	S	F	R	I	C	L	V	I	I	V	I	I	I	D	V	V
L	G	V	H	-	D	S	F	R	I	C	L	V	I	I	V	I	I	I	D	V	V
L	G	V	H	-	D	S	F	R	I	C	L	V	I	I	V	I	I	I	D	V	V
L	G	V	H	-	D	S	F	R	I	C	L	V	I	I	V	I	I	I	D	V	V
L	G	V	H	-	D	S	F	R	I	C	L	V	I	I	V	I	I	I	D	V	V
L	G	V	H	-	D	S	F	R	I	C	L	V	I	I	V	I	I	I	D	V	V
L	G	V	H	-	D	S	F	R	I	C	L	V	I	I	V	I	I	I	D	V	V
L	G	V	H	-	D	S	F	R	I	C	L	V	I	I	V	I	I	I	D	V	V
L	G	V	H	-	D	S	F	R	I	C	L	V	I	I	V	I	I	I	D	V	V
L	G	V	H	-	D	S	F	R	I	C	L	V	I	I	V	I	I	I	D	V	V
L	G	V	H	-	D	S	F	R	I	C	L	V	I	I	V	I	I	I	D	V	V
L	G	V	H	-	D	S	F	R	I	C	L	V	I	I	V	I	I	I	D	V	V
L	G	V	H	-	D	S	F	R	I	C	L	V	I	I	V	I	I	I	D	V	V
L	G	V	H	-	D	S	F	R	I	C	L	V	I	I	V	I	I	I	D	V	V
L	G	V	H	-	D	S	F	R	I	C	L	V	I	I	V	I	I	I	D	V	V
L	G	V	H	-	D	S	F	R	I	C	L	V	I	I	V	I	I	I	D	V	V

L	G	V	H	-	D	S	F	R	I	C	L	V	I	I	V	I	I	I	D	V	V
L	G	V	H	-	D	S	F	R	I	C	L	V	I	I	V	I	I	I	D	V	V
L	G	V	H	-	D	S	F	R	I	C	L	V	I	I	V	I	I	I	D	V	V
L	G	V	H	-	D	S	F	R	I	C	L	V	I	I	V	I	I	I	D	V	V
L	G	V	H	-	D	S	F	R	I	C	L	V	I	I	V	I	I	I	D	V	V
L	G	V	H	-	D	S	F	R	I	C	L	V	I	I	V	I	I	I	D	V	V
L	G	V	H	-	D	S	F	R	I	C	L	V	I	I	V	I	I	I	D	V	V
L	G	V	H	-	D	S	F	R	I	C	L	V	I	I	V	I	I	I	D	V	V
L	G	V	H	-	D	S	F	R	I	C	L	V	I	I	V	I	I	I	D	V	V
L	G	V	H	-	D	S	F	R	I	C	L	V	I	I	V	I	I	I	D	V	V
L	G	V	H	-	D	S	F	R	I	C	L	V	I	I	V	I	I	I	D	V	V
L	G	V	H	-	D	S	F	R	I	C	L	V	I	I	V	I	I	I	D	V	V
L	G	V	H	-	D	S	F	R	I	C	L	V	I	I	V	I	I	I	D	V	V
L	G	V	H	-	D	S	F	R	I	C	L	V	I	I	V	I	I	I	D	V	V
L	G	V	H	-	D	S	F	R	I	C	L	V	I	I	V	I	I	I	D	V	V
L	G	V	H	-	D	S	F	R	I	C	L	V	I	I	V	I	I	I	D	V	V
L	G	V	H	-	D	S	F	R	I	C	L	V	I	I	V	I	I	I	D	V	V
L	G	V	H	-	D	S	F	R	I	C	L	V	I	I	V	I	I	I	D	V	V
L	G	V	H	-	D	S	F	R	I	C	L	V	I	I	V	I	I	I	D	V	V
L	G	V	H	-	D	S	F	R	I	C	L	V	I	I	V	I	I	I	D	V	V
L	G	V	H	-	D	S	F	R	I	C	L	V	I	I	V	I	I	I	D	V	V
L	G	V	H	-	D	S	F	R	I	C	L	V	I	I	V	I	I	I	D	V	V
L	G	V	H	-	D	S	F	R	I	C	L	V	I	I	V	I	I	I	D	V	V
L	G	V	H	-	D	S	F	R	I	C	L	V	I	I	V	I	I	I	D	V	V
L	G	V	H	-	D	S	F	R	I	C	L	V	I	I	V	I	I	I	D	V	V
L	G	V	H	-	D	S	F	R	I	C	L	V	I	I	V	I	I	I	D	V	V
L	G	V	H	-	D	S	F	R	I	C	L	V	I	I	V	I	I	I	D	V	V
L	G	V	H	-	D	S	F	R	I	C	L	V	I	I	V	I	I	I	D	V	V
L	G	V	H	-	D	S	F	R	I	C	L	V	I	I	V	I	I	I	D	V	V
L	G	V	H	-	D	S	F	R	I	C	L	V	I	I	V	I	I	I	D	V	V
L	G	V	H	-	D	S	F	R	I	C	L	V	I	I	V	I	I	I	D	V	V
L	G	V	H	-	D	S	F	R	I	C	L	V	I	I	V	I	I	I	D	V	V
L	G	V	H	-	D	S	F	R	I	C	L	V	I	I	V	I	I	I	D	V	V
L	G	V	H	-	D	S	F	R	I	C	L	V	I	I	V	I	I	I	D	V	V
L	G	V	H	-	D	S	F	R	I	C	L	V	I	I	V	I	I	I	D	V	V
L	G	V	H	-	D	S	F	R	I	C	L	V	I	I	V	I	I	I	D	V	V
L	G	V	H	-	D	S	F	R	I	C	L	V	I	I	V	I	I	I	D	V	V
L	G	V	H	-	D	S	F	R	I	C	L	V	I	I	V	I	I	I	D	V	V
L	G	V	H	-	D	S	F	R	I	C	L	V	I	I	V	I	I	I	D	V	V
L	G	V	H	-	D	S	F	R	I	C	L	V	I	I	V	I	I	I	D	V	V
L	G	V	H	-	D	S	F	R	I	C	L	V	I	I	V	I	I	I	D	V	V
L	G	V	H	-	D	S	F	R	I	C	L	V	I	I	V	I	I	I	D	V	V
L	G	V	H	-	D	S	F	R	I	C	L	V	I	I	V	I	I	I	D	V	V
L	G	V	H	-	D	S	F	R	I	C	L	V	I	I	V	I	I	I	D	V	V
L	G	V	H	-	D	S	F	R	I	C	L	V	I	I	V	I	I	I	D	V	V
L	G	V	H	-	D	S	F	R	I	C	L	V	I	I	V	I	I	I	D	V	V
L	G	V	H	-	D	S	F	R	I	C	L	V	I	I	V	I	I	I	D	V	V
L	G	V	H	-	D	S	F	R	I	C	L	V	I	I	V	I	I	I	D	V	V
L	G	V	H	-	D	S	F	R	I	C	L	V	I	I	V	I	I	I	D	V	V
L	G	V	H	-	D	S	F	R	I	C	L	V	I	I	V	I	I	I	D	V	V
L	G	V	H	-	D	S	F	R	I	C	L	V	I	I	V	I	I	I	D	V	V
L	G	V	H	-	D	S	F	R	I	C	L	V	I	I	V	I	I	I	D	V	V
L	G	V	H	-	D	S	F	R	I	C	L	V	I	I	V	I	I	I	D	V	V
L	G	V	H	-	D	S	F	R	I	C	L	V	I	I	V	I	I	I	D	V	V
L	G	V	H	-	D	S	F	R	I	C	L	V	I	I	V	I	I	I	D	V	V
L	G	V	H	-	D	S	F	R	I	C	L	V	I	I	V	I	I	I	D	V	V
L	G	V	H	-	D	S	F	R	I	C	L	V	I	I	V	I	I	I	D	V	V
L	G	V	H	-	D	S	F	R	I	C	L	V	I	I	V	I	I	I	D	V	V
L	G	V	H	-	D	S	F	R	I	C	L	V	I	I	V	I	I	I	D	V	V
L	G	V	H	-	D	S	F	R	I	C	L	V	I	I	V	I	I	I	D	V	V
L	G	V	H	-	D	S	F	R	I	C	L	V	I	I	V	I	I	I	D	V	V
L	G	V	H	-	D	S	F	R	I	C	L	V	I	I	V	I	I	I	D	V	V
L	G	V	H	-	D	S	F	R	I	C	L	V	I	I	V	I	I	I	D	V	V
L	G	V	H	-	D	S	F	R	I	C	L	V	I	I	V	I	I	I	D	V	V
L	G	V	H	-	D	S	F	R	I	C	L	V	I	I	V	I	I	I	D	V	V
L	G	V	H	-	D	S	F	R	I	C	L	V	I	I	V	I	I	I	D	V	V
L	G	V	H	-	D	S	F	R	I	C	L	V	I	I	V	I	I	I	D	V	V

L	G	V	H	-	D	S	F	R	I	C	L	V	I	I	V	I	I	I	D	V	V
L	G	V	H	-	D	S	F	R	I	C	L	V	I	I	V	I	I	I	D	V	V
L	G	V	H	-	D	S	F	R	I	C	L	V	I	I	V	I	I	I	D	V	V
L	G	V	H	-	D	S	F	R	I	C	L	V	I	I	V	I	I	I	D	V	V
L	G	V	H	-	D	S	F	R	I	C	L	V	I	I	V	I	I	I	D	V	V
L	G	V	H	-	D	S	F	R	I	C	L	V	I	I	V	I	I	I	D	V	V
L	G	V	H	-	D	S	F	R	I	C	L	V	I	I	V	I	I	I	D	V	V
L	G	V	H	-	D	S	F	R	I	C	L	V	I	I	V	I	I	I	D	V	V
L	G	V	H	-	D	S	F	R	I	C	L	V	I	I	V	I	I	I	D	V	V
L	G	V	H	-	D	S	F	R	I	C	L	V	I	I	V	I	I	I	D	V	V
L	G	V	H	-	D	S	F	R	I	C	L	V	I	I	V	I	I	I	D	V	V
L	G	V	H	-	D	S	F	R	I	C	L	V	I	I	V	I	I	I	D	V	V
L	G	V	H	-	D	S	F	R	I	C	L	V	I	I	V	I	I	I	D	V	V
L	G	V	H	-	D	S	F	R	I	C	L	V	I	I	V	I	I	I	D	V	V
L	G	V	H	-	D	S	F	R	I	C	L	V	I	I	V	I	I	I	D	V	V
L	G	V	H	-	D	S	F	R	I	C	L	V	I	I	V	I	I	I	D	V	V
L	G	V	H	-	D	S	F	R	I	C	L	V	I	I	V	I	I	I	D	V	V
L	G	V	H	-	D	S	F	R	I	C	L	V	I	I	V	I	I	I	D	V	V
L	G	V	H	-	D	S	F	R	I	C	L	V	I	I	V	I	I	I	D	V	V
L	G	V	H	-	D	S	F	R	I	C	L	V	I	I	V	I	I	I	D	V	V
L	G	V	H	-	D	S	F	R	I	C	L	V	I	I	V	I	I	I	D	V	V
L	G	V	H	-	D	S	F	R	I	C	L	V	I	I	V	I	I	I	D	V	V
L	G	V	H	-	D	S	F	R	I	C	L	V	I	I	V	I	I	I	D	V	V
L	G	V	H	-	D	S	F	R	I	C	L	V	I	I	V	I	I	I	D	V	V
L	G	V	H	-	D	S	F	R	I	C	L	V	I	I	V	I	I	I	D	V	V
L	G	V	H	-	D	S	F	R	I	C	L	V	I	I	V	I	I	I	D	V	V
L	G	V	H	-	D	S	F	R	I	C	L	V	I	I	V	I	I	I	D	V	V
L	G	V	H	-	D	S	F	R	I	C	L	V	I	I	V	I	I	I	D	V	V
L	G	V	H	-	D	S	F	R	I	C	L	V	I	I	V	I	I	I	D	V	V
L	G	V	H	-	D	S	F	R	I	C	L	V	I	I	V	I	I	I	D	V	V
L	G	V	H	-	D	S	F	R	I	C	L	V	I	I	V	I	I	I	D	V	V
L	G	V	H	-	D	S	F	R	I	C	L	V	I	I	V	I	I	I	D	V	V
L	G	V	H	-	D	S	F	R	I	C	L	V	I	I	V	I	I	I	D	V	V
L	G	V	H	-	D	S	F	R	I	C	L	V	I	I	V	I	I	I	D	V	V
L	G	V	H	-	D	S	F	R	I	C	L	V	I	I	V	I	I	I	D	V	V
L	G	V	H	-	D	S	F	R	I	C	L	V	I	I	V	I	I	I	D	V	V
L	G	V	H	-	D	S	F	R	I	C	L	V	I	I	V	I	I	I	D	V	V
L	G	V	H	-	D	S	F	R	I	C	L	V	I	I	V	I	I	I	D	V	V
L	G	V	H	-	D	S	F	R	I	C	L	V	I	I	V	I	I	I	D	V	V
L	G	V	H	-	D	S	F	R	I	C	L	V	I	I	V	I	I	I	D	V	V
L	G	V	H	-	D	S	F	R	I	C	L	V	I	I	V	I	I	I	D	V	V
L	G	V	H	-	D	S	F	R	I	C	L	V	I	I	V	I	I	I	D	V	V
L	G	V	H	-	D	S	F	R	I	C	L	V	I	I	V	I	I	I	D	V	V
L	G	V	H	-	D	S	F	R	I	C	L	V	I	I	V	I	I	I	D	V	V
L	G	V	H	-	D	S	F	R	I	C	L	V	I	I	V	I	I	I	D	V	V
L	G	V	H	-	D	S	F	R	I	C	L	V	I	I	V	I	I	I	D	V	V
L	G	V	H	-	D	S	F	R	I	C	L	V	I	I	V	I	I	I	D	V	V
L	G	V	H	-	D	S	F	R	I	C	L	V	I	I	V	I	I	I	D	V	V
L	G	V	H	-	D	S	F	R	I	C	L	V	I	I	V	I	I	I	D	V	V
L	G	V	H	-	D	S	F	R	I	C	L	V	I	I	V	I	I	I	D	V	V
L	G	V	H	-	D	S	F	R	I	C	L	V	I	I	V	I	I	I	D	V	V
L	G	V	H	-	D	S	F	R	I	C	L	V	I	I	V	I	I	I	D	V	V
L	G	V	H	-	D	S	F	R	I	C	L	V	I	I	V	I	I	I	D	V	V
L	G	V	H	-	D	S	F	R	I	C	L	V	I	I	V	I	I	I	D	V	V
L	G	V	H	-	D	S	F	R	I	C	L	V	I	I	V	I	I	I	D	V	V
L	G	V	H	-	D	S	F	R	I	C	L	V	I	I	V	I	I	I	D	V	V
L	G	V	H	-	D	S	F	R	I	C	L	V	I	I	V	I	I	I	D	V	V
L	G	V	H	-	D	S	F	R	I	C	L	V	I	I	V	I	I	I	D	V	V
L	G	V	H	-	D	S	F	R	I	C	L	V	I	I	V	I	I	I	D	V	V
L	G	V	H	-	D	S	F	R	I	C	L	V	I	I	V	I	I	I	D	V	V
L	G	V	H	-	D	S	F	R	I	C	L	V	I	I	V	I	I	I	D	V	V
L	G	V	H	-	D	S	F	R	I	C	L	V	I	I	V	I	I	I	D	V	V
L	G	V	H	-	D	S	F	R	I	C	L	V	I	I	V	I	I	I	D	V	V
L	G	V	H	-	D	S	F	R	I	C	L	V	I	I	V	I	I	I	D	V	V
L	G	V	H	-	D	S	F	R	I	C	L	V	I	I	V	I	I	I	D	V	V
L	G	V	H	-	D	S	F	R	I	C	L	V	I	I	V	I	I	I	D	V	V
L	G	V	H	-	D	S	F	R	I	C	L	V	I	I	V	I	I	I	D	V	V

L	G	V	H	-	D	S	F	R	I	C	L	V	I	I	V	I	I	I	D	V	V
L	G	V	H	-	D	S	F	R	I	C	L	V	I	I	V	I	I	I	D	V	V
L	G	V	H	-	D	S	F	R	I	C	L	V	I	I	V	I	I	I	D	V	V
L	G	V	H	-	D	S	F	R	I	C	L	V	I	I	V	I	I	I	D	V	V
L	G	V	H	-	D	S	F	R	I	C	L	V	I	I	V	I	I	I	D	V	V
L	G	V	H	-	D	S	F	R	I	C	L	V	I	I	V	I	I	I	D	V	V
L	G	V	H	-	D	S	F	R	I	C	L	V	I	I	V	I	I	I	D	V	V
L	G	V	H	-	D	S	F	R	I	C	L	V	I	I	V	I	I	I	D	V	V
L	G	V	H	-	D	S	F	R	I	C	L	V	I	I	V	I	I	I	D	V	V
L	G	V	H	-	D	S	F	R	I	C	L	V	I	I	V	I	I	I	D	V	V
L	G	V	H	-	D	S	F	R	I	C	L	V	I	I	V	I	I	I	D	V	V
L	G	V	H	D	D	S	F	R	I	C	L	V	I	I	V	I	I	I	D	V	V
L	G	V	H	D	D	S	F	R	I	C	L	V	I	I	V	I	I	I	D	V	V
L	G	V	H	D	D	S	F	R	I	C	L	V	I	I	V	I	I	I	D	V	V
L	G	V	H	D	D	S	F	R	I	C	L	V	I	I	V	I	I	I	D	V	V
L	G	V	H	D	D	S	F	R	I	C	L	V	I	I	V	I	I	I	D	V	V
L	G	V	H	D	D	S	F	R	I	C	L	V	I	I	V	I	I	I	D	V	V
L	G	V	H	D	D	S	F	R	I	C	L	V	I	I	V	I	I	I	D	V	V
L	G	V	H	D	D	S	F	R	I	C	L	V	I	I	V	I	I	I	D	V	V
L	G	V	H	D	D	S	F	R	I	C	L	V	I	I	V	I	I	I	D	V	V
S	E	A	Y	N	A	G	L	K	L	Y	L	L	V	A	L	L	T	T	N	I	L
S	E	A	Y	N	A	G	L	K	L	Y	L	L	V	A	L	L	T	T	N	I	L
S	E	A	Y	N	A	G	L	K	L	Y	L	L	V	A	L	L	T	T	N	I	L
S	E	A	Y	N	A	G	L	K	L	Y	L	L	V	A	L	L	T	T	N	I	L
S	E	A	Y	N	A	G	L	K	L	Y	L	L	V	A	L	L	T	T	N	I	L
S	E	A	Y	N	A	G	L	K	L	Y	L	L	V	A	L	L	T	T	N	I	L
S	E	A	Y	N	A	G	L	K	L	Y	L	L	V	A	L	L	T	T	N	I	L
S	E	A	Y	N	A	G	L	K	L	Y	L	L	V	A	L	L	T	T	N	I	L
L	G	V	H	D	D	S	F	R	I	C	I	V	I	I	V	I	I	I	D	V	V
L	G	V	H	-	D	S	F	R	I	C	L	V	I	I	V	I	I	I	D	V	V
L	G	V	H	-	D	S	F	R	I	C	L	V	I	I	V	I	I	I	D	V	V
L	G	V	H	-	D	S	F	R	I	C	L	V	I	I	V	I	I	I	D	V	V
L	G	V	H	-	D	S	F	R	I	C	L	V	I	I	V	I	I	I	D	V	V
L	G	V	H	-	D	S	F	R	I	C	L	V	I	I	V	I	I	I	D	V	V
L	G	V	H	-	D	S	F	R	I	C	L	V	I	I	V	I	I	I	D	V	V
L	G	V	H	-	D	S	F	R	I	C	L	V	I	I	V	I	I	I	D	V	V
L	G	V	H	-	D	S	F	R	I	C	L	V	I	I	V	I	I	I	D	V	V
L	G	V	H	-	D	S	F	R	I	C	L	V	I	I	V	I	I	I	D	V	V
L	G	V	H	-	D	S	F	R	I	C	L	V	I	I	V	I	I	I	D	V	V
L	G	V	H	-	D	S	F	R	I	C	L	V	I	I	V	I	I	I	D	V	V
L	G	V	H	-	D	S	F	R	I	C	L	V	I	I	V	I	I	I	D	V	V
L	G	V	H	-	D	S	F	R	I	C	L	V	I	I	V	I	I	I	D	V	V
L	G	V	H	-	D	S	F	R	I	C	L	V	I	I	V	I	I	I	D	V	V
L	G	V	H	-	D	S	F	R	I	C	L	V	I	I	V	I	I	I	D	V	V
L	G	V	H	-	D	S	F	R	I	C	L	V	I	I	V	I	I	I	D	V	V
L	G	V	H	-	D	S	F	R	I	C	L	V	I	I	V	I	I	I	D	V	V
L	G	V	H	-	D	S	F	R	I	C	L	V	I	I	V	I	I	I	D	V	V
L	G	V	H	-	D	S	F	R	I	C	L	V	I	I	V	I	I	I	D	V	V
L	G	V	H	-	D	S	F	R	I	C	L	V	I	I	V	I	I	I	D	V	V
L	G	V	H	-	D	S	F	R	I	C	L	V	I	I	V	I	I	I	D	V	V
L	G	V	H	-	D	S	F	R	I	C	L	V	I	I	V	I	I	I	D	V	V
L	G	V	H	-	D	S	F	R	I	C	L	V	I	I	V	I	I	I	D	V	V
L	G	V	H	-	D	S	F	R	I	C	L	V	I	I	V	I	I	I	D	V	V
L	G	V	H	-	D	S	F	R	I	C	L	V	I	I	V	I	I	I	D	V	V
L	G	V	H	-	D	S	F	R	I	C	L	V	I	I	V	I	I	I	D	V	V
L	G	V	H	-	D	S	F	R	I	C	L	V	I	I	V	I	I	I	D	V	V
L	G	V	H	-	D	S	F	R	I	C	L	V	I	I	V	I	I	I	D	V	V
L	G	V	H	-	D	S	F	R	I	C	L	V	I	I	V	I	I	I	D	V	V
L	G	V	H	-	D	S	F	R	I	C	L	V	I	I	V	I	I	I	D	V	V
L	G	V	H	-	D	S	F	R	I	C	L	V	I	I	V	I	I	I	D	V	V
L	G	V	H	-	D	S	F	R	I	C	L	V	I	I	V	I	I	I	D	V	V
L	G	V	H	-	D	S	F	R	I	C	L	V	I	I	V	I	I	I	D	V	V
L	G	V	H	-	D	S	F	R	I	C	L	V	I	I	V	I	I	I	D	V	V
L	G	V	H	-	D	S	F	R	I	C	L	V	I	I	V	I	I	I	D	V	V
L	G	V	H	-	D	S	F	R	I	C	L	V	I	I	V	I	I	I	D	V	V
L	G	V	H	-	D	S	F	R	I	C	L	V	I	I	V	I	I	I	D	V	V
L	G	V	H	-	D	S	F	R	I	C	L	V	I	I	V	I	I	I	D	V	V

L	G	V	H	-	D	S	F	R	I	C	L	V	I	I	V	I	I	I	D	V	V
L	G	V	H	-	D	S	F	R	I	C	L	V	I	I	V	I	I	I	D	V	V
L	G	V	H	-	D	S	F	R	I	C	L	V	I	I	V	I	I	I	D	V	V
L	G	V	H	-	D	S	F	R	I	C	L	V	I	I	V	I	I	I	D	V	V
L	G	V	H	-	D	S	F	R	I	C	L	V	I	I	V	I	I	I	D	V	V
L	G	V	H	-	D	S	F	R	I	C	L	V	I	I	V	I	I	I	D	V	V
L	G	V	H	-	D	S	F	R	I	C	L	V	I	I	V	I	I	I	D	V	V
L	G	V	H	-	D	S	F	R	I	C	L	V	I	I	V	I	I	I	D	V	V
L	G	V	H	-	D	S	F	R	I	C	L	V	I	I	V	I	I	I	D	V	V
L	G	V	H	-	D	S	F	R	I	C	L	V	I	I	V	I	I	I	D	V	V
L	G	V	H	-	D	S	F	R	I	C	L	V	I	I	V	I	I	I	D	V	V
L	G	V	H	-	D	S	F	R	I	C	L	V	I	I	V	I	I	I	D	V	V
L	G	V	H	-	D	S	F	R	I	C	L	V	I	I	V	I	I	I	D	V	V
L	G	V	H	-	D	S	F	R	I	C	L	V	I	I	V	I	I	I	D	V	V
L	G	V	H	-	D	S	F	R	I	C	L	V	I	I	V	I	I	I	D	V	V
L	G	V	H	-	D	S	F	R	I	C	L	V	I	I	V	I	I	I	D	V	V
L	G	V	H	-	D	S	F	R	I	C	L	V	I	I	V	I	I	I	D	V	V
L	G	V	H	-	D	S	F	R	I	C	L	V	I	I	V	I	I	I	D	V	V
L	G	V	H	-	D	S	F	R	I	C	L	V	I	I	V	I	I	I	D	V	V
L	G	V	H	-	D	S	F	R	I	C	L	V	I	I	V	I	I	I	D	V	V
L	G	V	H	-	D	S	F	R	I	C	L	V	I	I	V	I	I	I	D	V	V
L	G	V	H	-	D	S	F	R	I	C	L	V	I	I	V	I	I	I	D	V	V
L	G	V	H	-	D	S	F	R	I	C	L	V	I	I	V	I	I	I	D	V	V
L	G	V	H	D	D	S	F	R	I	C	I	V	I	I	V	I	I	I	D	V	V
L	G	V	H	D	D	S	F	R	I	C	L	V	I	I	V	I	I	I	D	V	V
L	G	V	H	D	D	S	F	R	I	C	L	V	I	I	V	I	I	I	D	V	V
L	G	V	H	D	D	S	F	R	I	C	L	V	I	I	V	I	I	I	D	V	V
L	G	V	H	D	D	S	F	R	I	C	L	V	I	I	V	I	I	I	D	V	V
L	G	V	H	D	D	S	F	R	I	C	L	V	I	I	V	I	I	I	D	V	V
L	G	V	H	D	D	S	F	R	I	C	L	V	I	I	V	I	I	I	D	V	V
L	G	V	H	D	D	S	F	R	I	C	L	V	I	I	V	I	I	I	D	V	V
L	G	V	H	D	D	S	F	R	I	C	L	V	I	I	V	I	I	I	D	V	V
L	G	V	H	D	D	S	F	R	I	C	L	V	I	I	V	I	I	I	D	V	V
L	G	V	H	D	D	S	F	R	I	C	L	V	I	I	V	I	I	I	D	V	V
L	G	V	H	D	D	S	F	R	I	C	L	V	I	I	V	I	I	I	D	V	V
L	G	V	H	D	D	S	F	R	I	C	L	V	I	I	V	I	I	I	D	V	V
L	G	V	H	D	D	S	F	R	I	C	L	V	I	I	V	I	I	I	D	V	V
L	G	V	H	D	D	S	F	R	I	C	L	V	I	I	V	I	I	I	D	V	V
L	G	V	H	D	D	S	F	R	I	C	L	V	I	I	V	I	I	I	D	V	V
L	G	V	H	D	D	S	F	R	I	C	L	V	I	I	V	I	I	I	D	V	V
L	G	V	H	D	D	S	F	R	I	C	L	V	I	I	V	I	I	I	D	V	V
L	G	V	H	D	D	S	F	R	I	C	L	V	I	I	V	I	I	I	D	V	V
L	G	V	H	D	D	S	F	R	I	C	L	V	I	I	V	I	I	I	D	V	V
L	G	V	H	D	D	S	F	R	I	C	L	V	I	I	V	I	I	I	D	V	V
L	G	V	H	D	D	S	F	R	I	C	L	V	I	I	V	I	I	I	D	V	V
L	G	V	H	D	D	S	F	R	I	C	L	V	I	I	V	I	I	I	D	V	V
L	G	V	H	D	D	S	F	R	I	C	L	V	I	I	V	I	I	I	D	V	V
L	G	V	H	D	D	S	F	R	I	C	L	V	I	I	V	I	I	I	D	V	V
L	G	V	H	D	D	S	F	R	I	C	L	V	I	I	V	I	I	I	D	V	V
L	G	V	H	D	D	S	F	R	I	C	L	V	I	I	V	I	I	I	D	V	V
L	G	V	H	D	D	S	F	R	I	C	L	V	I	I	V	I	I	I	D	V	V
L	G	V	H	D	D	S	F	R	I	C	L	V	I	I	V	I	I	I	D	V	V
L	G	V	H	D	D	S	F	R	I	C	L	V	I	I	V	I	I	I	D	V	V
L	G	V	H	D	D	S	F	R	I	C	L	V	I	I	V	I	I	I	D	V	V
L	G	V	H	D	D	S	F	R	I	C	L	V	I	I	V	I	I	I	D	V	V
L	G	V	H	D	D	S	F	R	I	C	L	V	I	I	V	I	I	I	D	V	V
L	G	V	H	D	D	S	F	R	I	C	L	V	I	I	V	I	I	I	D	V	V
L	G	V	H	D	D	S	F	R	I	C	L	V	I	I	V	I	I	I	D	V	V
L	G	V	H	D	D	S	F	R	I	C	L	V	I	I	V	I	I	I	D	V	V
L	G	V	H	D	D	S	F	R	I	C	L	V	I	I	V	I	I	I	D	V	V
L	G	V	H	D	D	S	F	R	I	C	L	V	I	I	V	I	I	I	D	V	V
L	G	V	H	D	D	S	F	R	I	C	L	V	I	I	V	I	I	I	D	V	V
L	G	V	H	D	D	S	F	R	I	C	L	V	I	I	V	I	I	I	D	V	V
L	G	V	H	D	D	S	F	R	I	C	L	V	I	I	V	I	I	I	D	V	V
L	G	V	H	D	D	S	F	R	I	C	L	V	I	I	V	I	I	I	D	V	V
L	G	V	H	D	D	S	F	R	I	C	L	V	I	I	V	I	I	I	D	V	V
L	G	V	H	D	D	S	F	R	I	C	L	V	I	I	V	I	I	I	D	V	V

L	G	V	H	D	D	S	F
L	G	V	H	D	D	S	F
L	G	V	H	D	D	S	F
L	G	V	H	D	D	S	F
L	G	V	H	D	D	S	F
L	G	V	H	D	D	S	F
L	G	V	H	D	D	S	F
L	G	V	H	D	D	S	F
L	G	V	H	D	D	S	F
L	G	V	H	D	D	S	F

R	I	C	L	V	I	I	V	I	I	I	D	V	V
R	I	C	L	V	I	I	V	I	I	I	D	V	V
R	I	C	L	V	I	I	V	I	I	I	D	V	V
R	I	C	L	V	I	I	V	I	I	I	D	V	V
R	I	C	L	V	I	I	V	I	I	I	D	V	V
R	I	C	L	V	I	I	V	I	I	I	D	V	V
R	I	C	L	V	I	I	V	I	I	I	D	V	V
R	I	C	L	V	I	I	V	I	I	I	D	V	V
R	I	C	L	V	I	I	V	I	I	I	D	V	V
R	I	C	L	V	I	I	V	I	I	I	D	V	V

wciP														
128	132	134	142	158	192	195	237	238	254	268	270	295	318	328
M	S	N	K	H	A	S	V	F	R	G	N	Y	T	L
T	N	K	N	H	S	N	I	L	G	E	S	C	T	S
T	S	N	K	Q	A	S	V	F	R	G	N	Y	A	L
T	S	N	K	Q	A	S	V	F	R	G	N	Y	A	L
T	S	N	K	Q	A	S	V	F	R	G	N	Y	A	L
T	S	N	K	Q	A	S	V	F	R	G	N	Y	A	L
T	S	N	K	Q	A	S	V	F	R	G	N	Y	A	L
T	S	N	K	Q	A	S	V	F	R	G	N	Y	A	L
T	S	N	K	Q	A	S	V	F	R	G	N	Y	A	L
T	S	N	K	Q	A	S	V	F	R	G	N	Y	A	L
T	S	N	K	Q	A	S	V	F	R	G	N	Y	A	L

9	25	34	35	60	70	81
S	F	I	A	Y	V	R
F	L	L	A	G	I	K
S	F	L	T	G	I	R
S	F	I	A	Y	V	R
S	F	I	A	Y	V	R
S	F	I	A	Y	V	R
S	F	I	A	Y	V	R
S	F	I	A	Y	V	R
S	F	I	A	Y	V	R
S	F	I	A	Y	V	R
S	F	I	A	Y	V	R

wciP									
142	192	195	237	238	254	268	270	295	328
K	A	S	V	F	R	G	N	Y	L
N	S	N	I	L	G	E	S	C	S
K	A	S	V	F	R	G	N	Y	L
K	A	S	V	F	R	G	N	Y	L
K	A	S	V	F	R	G	N	Y	L
K	A	S	V	F	R	G	N	Y	L
K	A	S	V	F	R	G	N	Y	L
K	A	S	V	F	R	G	N	Y	L
K	A	S	V	F	R	G	N	Y	L
K	A	S	V	F	R	G	N	Y	L
K	A	S	V	F	R	G	N	Y	L
K	A	S	V	F	R	G	N	Y	L
K	A	S	V	F	R	G	N	Y	L
K	A	S	V	F	R	G	N	Y	L
K	A	S	V	F	R	G	N	Y	L
K	A	S	V	F	R	G	N	Y	L
K	A	S	V	F	R	G	N	Y	L
K	A	S	V	F	R	G	N	Y	L
K	A	S	V	F	R	G	N	Y	L
K	A	S	V	F	R	G	N	Y	L
K	A	S	V	F	R	G	N	Y	L
K	A	S	V	F	R	G	N	Y	L
K	A	S	V	F	R	G	N	Y	L
K	A	S	V	F	R	G	N	Y	L
K	A	S	V	F	R	G	N	Y	L
K	A	S	V	F	R	G	N	Y	L
K	A	S	V	F	R	G	N	Y	L
K	A	S	V	F	R	G	N	Y	L
K	A	S	V	F	R	G	N	Y	L
K	A	S	V	F	R	G	N	Y	L
K	A	S	V	F	R	G	N	Y	L

9	25	34	60	70	81	89	90	103	107	118	130
S	F	I	Y	V	R	H	E	V	T	R	S
F	L	L	G	I	K	Y	K	G	I	H	G
S	F	I	Y	V	R	H	E	V	T	R	S
S	F	I	Y	V	R	H	E	V	T	R	S
S	F	I	Y	V	R	H	E	V	T	R	S
S	F	I	Y	V	R	H	E	V	T	R	S
S	F	I	Y	V	R	H	E	V	T	R	S
S	F	I	Y	V	R	H	E	V	T	R	S
S	F	I	Y	V	R	H	E	V	T	R	S
S	F	I	Y	V	R	H	E	V	T	R	S
S	F	I	Y	V	R	H	E	V	T	R	S
S	F	I	Y	V	R	H	E	V	T	R	S
S	F	I	Y	V	R	H	E	V	T	R	S
S	F	I	Y	V	R	H	E	V	T	R	S
S	F	I	Y	V	R	H	E	V	T	R	S
S	F	I	Y	V	R	H	E	V	T	R	S
S	F	I	Y	V	R	H	E	V	T	R	S
S	F	I	Y	V	R	H	E	V	T	R	S
S	F	I	Y	V	R	H	E	V	T	R	S
S	F	I	Y	V	R	H	E	V	T	R	S
S	F	I	Y	V	R	H	E	V	T	R	S
S	F	I	Y	V	R	H	E	V	T	R	S
S	F	I	Y	V	R	H	E	V	T	R	S
S	F	I	Y	V	R	H	E	V	T	R	S
S	F	I	Y	V	R	H	E	V	T	R	S
S	F	I	Y	I	R	H	E	V	I	R	S
S	F	I	Y	V	R	H	E	V	T	R	S
S	F	I	Y	V	R	H	E	V	T	R	S
S	F	I	Y	V	R	H	E	V	T	R	S
S	F	I	Y	V	R	H	E	V	T	R	S
S	F	I	Y	V	R	H	E	V	T	R	S

rmlB												
26	29	54	67	75	118	145	274	275	300	317	327	331
E	P	N	E	Q	I	P	A	V	E	A	E	A
E	P	N	E	Q	I	P	A	A	D	A	E	A
N	P	D	A	E	I	L	P	A	D	E	D	S
N	T	D	A	E	L	P	P	A	D	A	D	A

24	25	32	33	39	47	71	83	92
E	E	A	E	E	E	K	K	H
E	E	A	K	E	E	K	K	H
V	D	T	E	S	A	K	E	Y
E	E	A	K	E	E	R	K	H

195	237	238	254	268	270	295	328
S	V	F	R	G	N	Y	L
N	I	L	G	E	S	C	S
S	V	F	R	G	N	Y	L
S	V	F	R	G	N	Y	L
S	V	F	R	G	N	Y	L
S	V	F	R	G	N	Y	L
S	V	F	R	G	N	Y	L
S	V	F	R	G	N	Y	L
S	V	F	R	G	N	Y	L
S	V	F	R	G	N	Y	L
S	V	F	R	G	N	Y	L
S	V	F	R	G	N	Y	L
S	V	F	R	G	N	Y	L
S	V	F	R	G	N	Y	L
S	V	F	R	G	N	Y	L
S	V	F	R	G	N	Y	L
S	V	F	R	G	N	Y	L
S	V	F	R	G	N	Y	L
S	V	F	R	G	N	Y	L
S	V	F	R	G	N	Y	L
S	V	F	R	G	N	Y	L
S	V	F	R	G	N	Y	L
S	V	F	R	G	N	Y	L
S	V	F	R	G	N	Y	L
S	V	F	R	G	N	Y	L
S	V	F	R	G	N	Y	L
S	V	F	R	G	N	Y	L

9	25	34	60	70	81	89	90	103	107	118	130	153	163
S	F	I	Y	V	R	H	E	V	T	R	S	N	S
F	L	L	G	I	K	Y	K	G	I	H	G	D	A
S	F	I	Y	I	R	H	E	V	I	R	S	N	S
S	F	I	Y	I	R	H	E	V	I	R	S	N	S
S	F	I	Y	I	R	H	E	V	I	R	S	N	S
S	F	I	Y	I	R	H	E	V	I	R	S	N	S
S	F	I	Y	I	R	H	E	V	I	R	S	N	S
S	F	I	Y	I	R	H	E	V	I	R	S	N	S
S	F	I	Y	I	R	H	E	V	I	R	S	N	S
S	F	I	Y	I	R	H	E	V	I	R	S	N	S
S	F	I	Y	I	R	H	E	V	I	R	S	N	S
S	F	I	Y	I	R	H	E	V	I	R	S	N	S
S	F	I	Y	I	R	H	E	V	I	R	S	N	S
S	F	I	Y	I	R	H	E	V	I	R	S	N	S
S	F	I	Y	I	R	H	E	V	I	R	S	N	S
S	F	I	Y	I	R	H	E	V	I	R	S	N	S
S	F	I	Y	I	R	H	E	V	I	R	S	N	S
S	F	I	Y	I	R	H	E	V	I	R	S	N	S
S	F	I	Y	I	R	H	E	V	I	R	S	N	S
S	F	I	Y	I	R	H	E	V	I	R	S	N	S
S	F	I	Y	I	R	H	E	V	I	R	S	N	S
S	F	I	Y	I	R	H	E	V	I	R	S	N	S
S	F	I	Y	I	R	H	E	V	I	R	S	N	S
S	F	I	Y	I	R	H	E	V	I	R	S	N	S
S	F	I	Y	I	R	H	E	V	I	R	S	N	S
S	F	I	Y	I	R	H	E	V	I	R	S	N	S
S	F	I	Y	I	R	H	E	V	I	R	S	N	S

	wciN	wciO					wciP					wzy						
452		5	6	23	136	212	62	126	128	158	318	34	35	60	70	89	107	117
R		R	I	A	N	V	F	M	M	H	T	I	A	Y	V	H	T	I
K		K	L	P	T	V	L	L	T	Q	A	L	T	G	I	Y	I	T
R																		
R		R	I	A	N	I	F	M	T	H	T	I	A	Y	I	H	I	I
R		R	I	A	N	I	F	M	T	H	T	I	A	Y	I	H	I	I
R		R	I	A	N	I	F	M	T	H	T	I	A	Y	I	H	I	I
R		R	I	A	N	I	F	M	T	H	T	I	A	Y	I	H	I	I
R		R	I	A	N	I	F	M	T	H	T	I	A	Y	I	H	I	I
R		R	I	A	N	I	F	M	T	H	T	I	A	Y	I	H	I	I
R		R	I	A	N	I	F	M	T	H	T	I	A	Y	I	H	I	I
R		R	I	A	N	I	F	M	T	H	T	I	A	Y	I	H	I	I
R		R	I	A	N	I	F	M	T	H	T	I	A	Y	I	H	I	I
R		R	I	A	N	I	F	M	T	H	T	I	A	Y	I	H	I	I
R		R	I	A	N	I	F	M	T	H	T	I	A	Y	I	H	I	I
R		R	I	A	N	I	F	M	T	H	T	I	A	Y	I	H	I	I
R		R	I	A	N	I	F	M	T	H	T	I	A	Y	I	H	I	I
R		R	I	A	N	I	F	M	T	H	T	I	A	Y	I	H	I	I
R		R	I	A	N	I	F	M	T	H	T	I	A	Y	I	H	I	I
R		R	I	A	N	I	F	M	T	H	T	I	A	Y	I	H	I	I
R		R	I	A	N	I	F	M	T	H	T	I	A	Y	I	H	I	I
R		R	I	A	N	I	F	M	T	H	T	I	A	Y	I	H	I	I
R		R	I	A	N	I	F	M	T	H	T	I	A	Y	I	H	I	I
R		R	I	A	N	I	F	M	T	H	T	I	A	Y	I	H	I	I
R		R	I	A	N	I	F	M	T	H	T	I	A	Y	I	H	I	I
R		R	I	A	N	I	F	M	T	H	T	I	A	Y	I	H	I	I
R		R	I	A	N	I	F	M	T	H	T	I	A	Y	I	H	I	I
R		R	I	A	N	I	F	M	T	H	T	I	A	Y	I	H	I	I
R		R	I	A	N	I	F	M	T	H	T	I	A	Y	I	H	I	I
R		R	I	A	N	I	F	M	T	H	T	I	A	Y	I	H	I	I

R
R

R	I	A	N	I
R	I	A	N	I

F	M	T	H	T
F	M	T	H	T

I	A	Y	I	H	I	I
I	A	Y	I	H	I	I

wciO															wciP						
133	137	140	142	168	169	173	174	180	182	186	204	205	212	213	13	62	72	128	158	254	273
A	D	I	I	Q	D	S	S	D	N	I	L	T	V	L	N	F	A	M	H	R	S
A	D	I	I	Q	D	S	S	D	N	I	L	T	V	L	N	F	A	M	H	R	S
A	D	I	I	Q	D	S	S	D	N	I	L	T	V	L	N	F	A	T	H	R	S
A	D	I	I	Q	D	S	S	D	N	I	L	T	V	L	N	F	A	T	H	R	S
A	D	I	I	Q	D	S	S	D	N	I	L	T	V	L	N	F	A	T	H	R	S
A	D	I	I	Q	D	S	S	D	N	I	L	T	V	L	N	F	A	T	H	R	S
A	D	I	I	Q	D	S	S	D	N	I	L	T	V	L	N	F	A	T	H	R	S
A	D	I	I	Q	D	S	S	D	N	I	L	T	V	L	N	F	A	T	H	R	S
A	D	I	I	Q	D	S	S	D	N	I	L	T	V	L	N	F	A	T	H	R	S
A	D	I	I	Q	D	S	S	D	N	I	L	T	V	L	N	F	A	T	H	R	S
A	D	I	I	Q	D	S	S	D	N	I	L	T	V	L	N	F	A	T	H	R	S
A	D	I	I	Q	D	S	S	D	N	I	L	T	V	L	N	F	A	T	H	R	S
A	D	I	I	Q	D	S	S	D	N	I	L	T	V	L	N	F	A	T	H	R	S
A	D	I	I	Q	D	S	S	D	N	I	L	T	V	L	N	F	A	T	H	R	S
A	D	I	I	Q	D	S	S	D	N	I	L	T	V	L	N	F	A	T	H	R	S
A	D	I	I	Q	D	S	S	D	N	I	L	T	V	L	N	F	A	T	H	R	S
A	D	I	I	Q	D	S	S	D	N	I	L	T	V	L	N	F	A	T	H	R	S
A	D	I	I	Q	D	S	S	D	N	I	L	T	V	L	N	F	A	T	H	R	S
A	D	I	I	Q	D	S	S	D	N	I	L	T	V	L	N	F	A	T	H	R	S
A	D	I	I	Q	D	S	S	D	N	I	L	T	V	L	N	F	A	T	H	R	S
A	D	I	I	Q	D	S	S	D	N	I	L	T	V	L	N	F	A	T	H	R	S
A	D	I	I	Q	D	S	S	D	N	I	L	T	V	L	N	F	A	T	H	R	S
A	D	I	I	Q	D	S	S	D	N	I	L	T	V	L	N	F	A	T	H	R	S
A	D	I	I	Q	D	S	S	D	N	I	L	T	V	L	N	F	A	T	H	R	S
A	D	I	I	Q	D	S	S	D	N	I	L	T	V	L	N	F	A	T	H	R	S
A	D	I	I	Q	D	S	S	D	N	I	L	T	V	L	N	F	A	T	H	R	S
A	D	I	I	Q	D	S	S	D	N	I	L	T	V	L	N	F	A	T	H	R	S
A	D	I	I	Q	D	S	S	D	N	I	L	T	V	L	N	F	A	T	H	R	S
A	D	I	I	Q	D	S	S	D	N	I	L	T	V	L	N	F	A	T	H	R	S
A	D	I	I	Q	D	S	S	D	N	I	L	T	V	L	N	F	A	T	H	R	S
A	D	I	I	Q	D	S	S	D	N	I	L	T	V	L	N	F	A	T	H	R	S
A	D	I	I	Q	D	S	S	D	N	I	L	T	V	L	N	F	A	T	H	R	S
A	D	I	I	Q	D	S	S	D	N	I	L	T	V	L	N	F	A	T	H	R	S
A	D	I	I	Q	D	S	S	D	N	I	L	T	V	L	N	F	A	T	H	R	S
A	D	I	I	Q	D	S	S	D	N	I	L	T	V	L	N	F	A	T	H	R	S
A	D	I	I	Q	D	S	S	D	N	I	L	T	V	L	N	F	A	T	H	R	S
A	D	I	I	Q	D	S	S	D	N	I	L	T	V	L	N	F	A	T	H	R	S
A	D	I	I	Q	D	S	S	D	N	I	L	T	V	L	N	F	A	T	H	R	S
A	D	I	I	Q	D	S	S	D	N	I	L	T	V	L	N	F	A	T	H	R	S
A	D	I	I	Q	D	S	S	D	N	I	L	T	V	L	N	F	A	T	H	R	S
A	D	I	I	Q	D	S	S	D	N	I	L	T	V	L	N	F	A	T	H	R	S
A	D	I	I	Q	D	S	S	D	N	I	L	T	V	L	N	F	A	T	H	R	S
A	D	I	I	Q	D	S	S	D	N	I	L	T	V	L	N	F	A	T	H	M	S
A	D	I	I	Q	D	S	S	D	N	I	L	T	V	L	N	F	A	T	H	M	S
A	D	I	I	Q	D	S	S	D	N	I	L	T	V	L	N	F	A	T	H	M	S
A	D	I	I	Q	D	S	S	D	N	I	L	T	V	L	N	F	A	T	H	M	S
A	D	I	I	Q	D	S	S	D	N	I	L	T	V	L	N	F	A	T	H	M	S
A	D	I	I	Q	D	S	S	D	N	I	L	T	V	L	N	F	A	T	Q	M	S
A	D	I	I	Q	D	S	S	D	N	I	L	T	V	L	N	F	A	T	H	M	S
A	D	I	I	Q	D	S	S	D	N	I	L	T	V	L	N	F	A	T	H	M	S
A	D	I	I	Q	D	S	S	D	N	I	L	T	V	L	N	F	A	T	H	M	S
A	D	I	I	Q	D	S	S	D	N	I	L	T	V	L	N	F	A	T	H	M	S
A	D	I	I	Q	D	S	S	D	N	I	L	T	V	L	N	F	A	T	H	R	S
A	D	I	I	Q	D	S	S	D	N	I	L	T	V	L	N	F	A	T	H	M	S
A	D	I	I	Q	D	S	S	D	N	I	L	T	V	L	N	F	A	T	H	M	S
A	D	I	I	Q	D	S	S	D	N	I	L	T	V	L	N	F	A	T	H	M	S
A	D	I	I	Q	D	S	S	D	N	I	L	T	V	L	N	F	A	T	H	M	S
A	D	I	I	Q	D	S	S	D	N	I	L	T	V	L	N	F	A	T	H	M	S
A	D	I	I	Q	D	S	S	D	N	I	L	T	V	L	N	F	A	T	H	R	S
A	D	I	I	Q	D	S	S	D	N	I	L	T	V	L	N	F	A	T	H	R	I
A	D	I	I	Q	D	S	S	D	N	I	L	T	V	L	N	F	A	T	H	R	S
A	D	I	I	Q	D	S	S	D	N	I	L	T	V	L	N	F	A	T	H	R	S

A	D	I	I	Q	D	S	S	D	N	I	L	T	V	L	N	F	A	T	H	R	S
A	D	I	I	Q	D	S	S	D	N	I	L	T	V	L	N	F	A	T	H	R	S
A	D	I	I	Q	D	S	S	D	N	I	L	T	V	L	N	F	A	T	H	R	S
A	D	I	I	Q	D	S	S	D	N	I	L	T	V	L	N	F	A	T	H	R	S
A	D	I	I	Q	D	S	S	D	N	I	L	T	V	L	N	F	A	T	H	R	S
A	D	I	I	Q	D	S	S	D	N	I	L	T	V	L	N	F	A	T	H	R	S
A	D	I	I	Q	D	S	S	D	N	I	L	T	V	L	N	F	A	T	H	R	S
A	D	I	I	Q	D	S	S	D	N	I	L	T	V	L	N	F	A	T	H	R	S
A	D	I	I	Q	D	S	S	D	N	I	L	T	V	L	N	F	A	T	H	R	S
A	D	I	I	Q	D	S	S	D	N	I	L	T	V	L	N	F	A	T	H	R	S
A	D	I	I	Q	D	S	S	D	N	I	L	T	V	L	N	F	A	T	H	R	S
A	D	I	I	Q	D	S	S	D	N	I	L	T	V	L	N	F	A	T	H	R	S
A	D	I	I	Q	D	S	S	D	N	I	L	T	V	L	N	F	A	T	H	R	S
A	D	I	I	Q	D	S	S	D	N	I	L	T	V	L	N	F	A	T	H	R	S
A	D	I	I	Q	D	S	S	D	N	I	L	T	V	L	N	F	A	T	H	R	S
A	D	I	I	Q	D	S	S	D	N	I	L	T	V	L	N	F	A	T	H	R	I
A	D	I	I	Q	D	S	S	D	N	I	L	T	V	L	N	F	A	T	H	R	S
A	D	I	I	Q	D	S	S	D	N	I	L	T	V	L	N	F	A	T	H	R	S
A	D	I	I	Q	D	S	S	D	N	I	L	T	V	L	N	F	A	T	H	R	S
A	D	I	I	Q	D	S	S	D	N	I	L	T	V	L	N	F	A	T	H	R	S
A	D	I	I	Q	D	S	S	D	N	I	L	T	V	L	N	F	A	T	H	R	S
A	D	I	I	Q	D	S	S	D	N	I	L	T	V	L	N	F	A	T	H	M	S
A	D	I	I	Q	D	S	S	D	N	I	L	T	V	L	N	F	A	T	H	M	S
A	D	I	I	Q	D	S	S	D	N	I	L	T	V	L	N	F	A	T	H	R	S
A	D	I	I	Q	D	S	S	D	N	I	L	T	V	L	N	F	A	T	H	R	S
A	D	I	I	Q	D	S	S	D	N	I	L	T	V	L	N	F	A	T	H	R	S
A	D	I	I	Q	D	S	S	D	N	I	L	T	V	L	N	F	A	M	H	R	S
A	D	I	I	Q	D	S	S	D	N	I	L	T	V	L	N	F	A	M	H	R	S
A	D	I	I	Q	D	S	S	D	N	I	L	T	V	L	N	F	A	M	H	R	S
A	D	I	I	Q	D	S	S	D	N	I	L	T	V	L	N	F	A	M	H	R	S
A	D	I	I	Q	D	S	S	D	N	I	L	T	V	L	N	F	A	M	H	R	S
A	D	I	I	Q	D	S	S	D	N	I	L	T	V	L	N	F	A	M	H	R	S
A	D	I	I	Q	D	S	S	D	N	I	L	T	V	L	N	F	A	M	H	R	S
A	D	I	I	Q	D	S	S	D	N	I	L	T	V	L	N	F	A	M	H	R	S
A	D	I	I	Q	D	S	S	D	N	I	L	T	V	L	N	F	A	M	H	R	S
A	D	I	I	Q	D	S	S	D	N	I	L	T	V	L	N	F	A	M	H	R	S
A	D	I	I	Q	D	S	S	D	N	I	L	T	V	L	N	F	A	M	H	R	S
A	D	I	I	Q	D	S	S	D	N	I	L	T	V	L	N	F	A	M	H	R	S
A	D	I	I	Q	D	S	S	D	N	I	L	T	V	L	N	F	A	M	H	R	S
A	D	I	I	Q	D	S	S	D	N	I	L	T	V	L	N	F	A	M	H	R	S
A	D	I	I	Q	D	S	S	D	N	I	L	T	V	L	N	F	A	M	H	R	S
A	D	I	I	Q	D	S	S	D	N	I	L	T	V	L	N	F	A	M	H	R	S
A	D	I	I	Q	D	S	S	D	N	I	L	T	V	L	N	F	A	M	H	R	S
A	D	I	I	Q	D	S	S	D	N	I	L	T	V	L	N	F	A	M	H	R	S
A	D	I	I	Q	D	S	S	D	N	I	L	T	V	L	N	F	A	M	H	R	S
A	D	I	I	Q	D	S	S	D	N	I	L	T	V	L	N	F	A	M	H	R	S
A	D	I	I	Q	D	S	S	D	N	I	L	T	V	L	N	F	A	M	H	R	S
A	D	I	I	Q	D	S	S	D	N	I	L	T	V	L	N	F	A	M	H	R	S
A	D	I	I	Q	D	S	S	D	N	I	L	T	V	L	N	F	A	M	H	R	S
A	D	I	I	Q	D	S	S	D	N	I	L	T	V	L	N	F	A	M	H	R	S
A	D	I	I	Q	D	S	S	D	N	I	L	T	V	L	N	F	A	M	H	R	S
A	D	I	I	Q	D	S	S	D	N	I	L	T	V	L	N	F	A	M	H	R	S
A	D	I	I	Q	D	S	S	D	N	I	L	T	V	L	N	F	A	M	H	R	S
A	D	I	I	Q	D	S	S	D	N	I	L	T	V	L	N	F	A	M	H	R	S
A	D	I	I	Q	D	S	S	D	N	I	L	T	V	L	N	F	A	M	H	R	S
A	D	I	I	Q	D	S	S	D	N	I	L	T	V	L	N	F	A	M	H	R	S
A	D	I	I	Q	D	S	S	D	N	I	L	T	V	L	N	F	A	M	H	R	S
A	D	I	I	Q	D	S	S	D	N	I	L	T	V	L	N	F	A	M	H	R	S
A	D	I	I	Q	D	S	S	D	N	I	L	T	V	L	N	F	A	M	H	R	S
A	D	I	I	Q	D	S	S	D	N	I	L	T	V	L	N	F	A	M	H	R	S
A	D	I	I	Q	D	S	S	D	N	I	L	T	V	L	N	F	A	M	H	R	S
A	D	I	I	Q	D	S	S	D	N	I	L	T	V	L	N	F	A	M	H	R	S
A	D	I	I	Q	D	S	S	D	N	I	L	T	V	L	N	F	A	M	H	R	S
A	D	I	I	Q	D	S	S	D	N	I	L	T	V	L	N	F	A	M	H	R	S
A	D	I	I	Q	D	S	S	D	N	I	L	T	V	L	N	F	A	M	H	R	S
A	D	I	I	Q	D	S	S	D	N	I	L	T	V	L	N	F	A	M	H	R	S
A	D	I	I	Q	D	S	S	D	N	I	L	T	V	L	N	F	A	M	H	R	S

A	D	I	I	Q	D	S	S	D	N	I	L	T	V	L	N	F	A	M	H	R	S
A	D	I	I	Q	D	S	S	D	N	I	L	T	V	L	N	F	A	M	H	R	S
A	D	I	I	Q	D	S	S	D	N	I	L	T	V	L	N	F	A	M	H	R	S
A	D	I	I	Q	D	S	S	D	N	I	L	T	V	L	N	F	A	M	H	R	S
A	D	I	I	Q	D	S	S	D	N	I	L	T	V	L	N	F	A	M	H	R	S
A	D	I	I	Q	D	S	S	D	N	I	L	T	V	L	N	F	A	M	H	R	S
A	D	I	I	Q	D	S	S	D	N	I	L	T	V	L	N	F	A	M	H	R	S
A	D	I	I	Q	D	S	S	D	N	I	L	T	V	L	N	F	A	M	H	R	S
A	D	I	I	Q	D	S	S	D	N	I	L	T	V	L	N	F	A	M	H	R	S
A	D	I	I	Q	D	S	S	D	N	I	L	T	V	L	N	F	A	M	H	R	S
A	D	I	I	Q	D	S	S	D	N	I	L	T	V	L	N	F	A	M	H	R	S
A	D	I	I	Q	D	S	S	D	N	I	L	T	V	L	N	F	A	M	H	R	S
A	D	I	I	Q	D	S	S	D	N	I	L	T	V	L	N	F	A	M	H	R	S
A	D	I	I	Q	D	S	S	D	N	I	L	T	V	L	N	F	A	M	H	R	S
A	D	I	I	Q	D	S	S	D	N	I	L	T	V	L	N	F	A	M	H	R	S
A	D	I	I	Q	D	S	S	D	N	I	L	T	V	L	N	F	A	M	H	R	S
A	D	I	I	Q	D	S	S	D	N	I	L	T	V	L	N	F	A	M	H	R	S
A	D	I	I	Q	D	S	S	D	N	I	L	T	V	L	N	F	A	M	H	R	S
A	D	I	I	Q	D	S	S	D	N	I	L	T	V	L	N	F	A	M	H	R	S
A	D	I	I	Q	D	S	S	D	N	I	L	T	V	L	N	F	A	M	H	R	S
A	D	I	I	Q	D	S	S	D	N	I	L	T	V	L	N	F	A	M	H	R	S
A	D	I	I	Q	D	S	S	D	N	I	L	T	V	L	N	F	A	M	H	R	S
A	D	I	I	Q	D	S	S	D	N	I	L	T	V	L	N	F	A	M	H	R	S
A	D	I	I	Q	D	S	S	D	N	I	L	T	V	L	N	F	A	M	H	R	S
A	D	I	I	Q	D	S	S	D	N	I	L	T	V	L	N	F	A	M	H	R	S
A	D	I	I	Q	D	S	S	D	N	I	L	T	V	L	N	F	A	M	H	R	S
A	D	I	I	Q	D	S	S	D	N	I	L	T	V	L	N	F	A	M	H	R	S
A	D	I	I	Q	D	S	S	D	N	I	L	T	V	L	N	F	A	M	H	R	S
A	D	I	I	Q	D	S	S	D	N	I	L	T	V	L	N	F	A	M	H	R	S
A	D	I	I	Q	D	S	S	D	N	I	L	T	V	L	N	F	A	M	H	R	S
A	D	I	I	Q	D	S	S	D	N	I	L	T	V	L	N	F	A	M	H	R	S
A	D	I	I	Q	D	S	S	D	N	I	L	T	V	L	N	F	A	M	H	R	S
A	D	I	I	Q	D	S	S	D	N	I	L	T	V	L	N	F	A	M	H	R	S
A	D	I	I	Q	D	S	S	D	N	I	L	T	V	L	N	F	A	M	H	R	S
A	D	I	I	Q	D	S	S	D	N	I	L	T	V	L	N	F	A	M	H	R	S
A	D	I	I	Q	D	S	S	D	N	I	L	T	V	L	N	F	A	M	H	R	S
A	D	I	I	Q	D	S	S	D	N	I	L	T	V	L	N	F	A	M	H	R	S
A	D	I	I	Q	D	S	S	D	N	I	L	T	V	L	N	F	A	M	H	R	S
A	D	I	I	Q	D	S	S	D	N	I	L	T	V	L	N	F	A	M	H	R	S
A	D	I	I	Q	D	S	S	D	N	I	L	T	V	L	N	F	A	M	H	R	S
A	D	I	I	Q	D	S	S	D	N	I	L	T	V	L	N	F	A	M	H	R	S
A	D	I	I	Q	D	S	S	D	N	I	L	T	V	L	N	F	A	M	H	R	S
A	D	I	I	Q	D	S	S	D	N	I	L	T	V	L	N	F	A	M	H	R	S
A	D	I	I	Q	D	S	S	D	N	I	L	T	V	L	N	F	A	M	H	R	S
A	D	I	I	Q	D	S	S	D	N	I	L	T	V	L	N	F	A	M	H	R	S
A	D	I	I	Q	D	S	S	D	N	I	L	T	V	L	N	F	A	M	H	R	S
A	D	I	I	Q	D	S	S	D	N	I	L	T	V	L	N	F	A	M	H	R	S
A	D	I	I	Q	D	S	S	D	N	I	L	T	V	L	N	F	A	M	H	R	S
A	D	I	I	Q	D	S	S	D	N	I	L	T	V	L	N	F	A	M	H	R	S
A	D	I	I	Q	D	S	S	D	N	I	L	T	V	L	N	F	A	M	H	R	S
A	D	I	I	Q	D	S	S	D	N	I	L	T	V	L	N	F	A	M	H	R	S
A	D	I	I	Q	D	S	S	D	N	I	L	T	V	L	N	F	A	M	H	R	S
A	D	I	I	Q	D	S	S	D	N	I	L	T	V	L	N	F	A	M	H	R	S
A	D	I	I	Q	D	S	S	D	N	I	L	T	V	L	N	F	A	M	H	R	S
A	D	I	I	Q	D	S	S	D	N	I	L	T	V	L	N	F	A	M	H	R	S
A	D	I	I	Q	D	S	S	D	N	I	L	T	V	L	N	F	A	M	H	R	S
A	D	I	I	Q	D	S	S	D	N	I	L	T	V	L	N	F	A	M	H	R	S
A	D	I	I	Q	D	S	S	D	N	I	L	T	V	L	N	F	A	M	H	R	S
A	D	I	I	Q	D	S	S	D	N	I	L	T	V	L	N	F	A	M	H	R	S
A	D	I	I	Q	D	S	S	D	N	I	L	T	V	L	N	F	A	M	H	R	S
A	D	I	I	Q	D	S	S	D	N	I	L	T	V	L	N	F	A	M	H	R	S
A	D	I	I	Q	D	S	S	D	N	I	L	T	V	L	N	F	A	M	H	R	S
A	D	I	I	Q	D	S	S	D	N	I	L	T	V	L	N	F	A	M	H	R	S
A	D	I	I	Q	D	S	S	D	N	I	L	T	V	L	N	F	A	M	H	R	S
A	D	I	I	Q	D	S	S	D	N	I	L	T	V	L	N	F	A	M	H	R	S
A	D	I	I	Q	D	S	S	D	N	I	L	T	V	L	N	F	A	M	H	R	S
A	D	I	I	Q	D	S	S	D	N	I	L	T	V	L	N	F	A	M	H	R	S

A	D	I	I	Q	D	S	S	D	N	I	L	T	V	L	N	F	A	M	H	R	S
A	D	I	I	Q	D	S	S	D	N	I	L	T	V	L	N	F	A	M	H	R	S
A	D	I	I	Q	D	S	S	D	N	I	L	T	V	L	N	F	A	M	H	R	S
A	D	I	I	Q	D	S	S	D	N	I	L	T	V	L	N	F	A	M	H	R	S
A	D	I	I	Q	D	S	S	D	N	I	L	T	V	L	N	F	A	T	H	R	S
A	D	I	I	Q	D	S	S	D	N	I	L	T	V	L	N	F	A	T	H	R	S
A	D	I	I	Q	D	S	S	D	N	I	L	T	V	L	N	F	A	T	H	R	S
A	D	I	I	Q	D	S	S	D	N	I	L	T	V	L	N	F	A	T	H	R	S
A	D	I	I	Q	D	S	S	D	N	I	L	T	V	L	N	F	A	T	H	R	S
A	D	I	I	Q	D	S	S	D	N	I	L	T	V	L	N	F	A	T	H	R	S
A	D	I	I	Q	D	S	S	D	N	I	L	T	V	L	N	F	A	T	H	R	S
A	D	I	I	Q	D	S	S	D	N	I	L	T	V	L	N	F	A	T	H	R	S
A	D	I	I	Q	D	S	S	D	N	I	L	T	V	L	N	F	A	T	H	R	S
A	D	I	I	Q	D	S	S	D	N	I	L	T	V	L	N	F	A	T	H	R	S
A	D	I	I	Q	D	S	S	D	N	I	L	T	V	L	N	F	A	T	H	R	S
A	D	I	I	Q	D	S	S	D	N	I	L	T	V	L	N	F	A	T	H	R	S
A	D	I	I	Q	D	S	S	D	N	I	L	T	V	L	N	F	A	T	H	R	S
A	D	I	I	Q	D	S	S	D	N	I	L	T	V	L	N	F	A	T	H	R	S
A	D	I	I	Q	D	S	S	D	N	I	L	T	V	L	N	F	A	T	H	R	S
A	D	I	I	Q	D	S	S	D	N	I	L	T	V	L	N	F	A	T	H	R	S
A	D	I	I	Q	D	S	S	D	N	I	L	T	V	L	N	F	A	T	H	R	S
A	D	I	I	Q	D	S	S	D	N	I	L	T	V	L	N	F	A	T	H	R	S
A	D	I	I	Q	D	S	S	D	N	I	L	T	V	L	N	F	A	T	H	R	S
A	D	I	I	Q	D	S	S	D	N	I	L	T	V	L	N	F	A	T	H	R	S
A	D	I	I	Q	D	S	S	D	N	I	L	T	V	L	N	F	A	T	H	R	S
A	D	I	I	Q	D	S	S	D	N	I	L	T	V	L	N	F	A	T	H	R	S
A	D	I	I	Q	D	S	S	D	N	I	L	T	V	L	N	F	A	T	H	R	S
A	D	I	I	Q	D	S	S	D	N	I	L	T	V	L	N	F	A	T	H	R	S
A	D	I	I	Q	D	S	S	D	N	I	L	T	V	L	N	F	A	T	H	R	S
A	D	I	I	Q	D	S	S	D	N	I	L	T	V	L	N	F	A	T	H	R	S
A	D	I	I	Q	D	S	S	D	N	I	L	T	V	L	N	F	A	T	H	R	S
A	D	I	I	Q	D	S	S	D	N	I	L	T	V	L	N	F	A	T	H	R	S
A	D	I	I	Q	D	S	S	D	N	I	L	T	V	L	N	F	A	T	H	R	S
A	D	I	I	Q	D	S	S	D	N	I	L	T	V	L	N	F	A	T	H	R	S
A	D	I	I	Q	D	S	S	D	N	I	L	T	V	L	N	F	A	T	H	R	S
A	D	I	I	Q	D	S	S	D	N	I	L	T	V	L	N	F	A	T	H	R	S
A	D	I	I	Q	D	S	S	D	N	I	L	T	V	L	N	F	A	T	H	R	S
A	D	I	I	Q	D	S	S	D	N	I	L	T	V	L	N	F	A	T	H	R	S
A	D	I	I	Q	D	S	S	D	N	I	L	T	V	L	N	F	A	T	H	R	S
A	D	I	I	Q	D	S	S	D	N	I	L	T	V	L	N	F	A	T	H	R	S
A	D	I	I	Q	D	S	S	D	N	I	L	T	V	L	N	F	A	T	H	R	S
A	D	I	I	Q	D	S	S	D	N	I	L	T	V	L	N	F	A	T	H	R	S
A	D	I	I	Q	D	S	S	D	N	I	L	T	V	L	N	F	A	T	H	R	S
A	D	I	I	Q	D	S	S	D	N	I	L	T	V	L	N	F	A	T	H	R	S
A	D	I	I	Q	D	S	S	D	N	I	L	T	V	L	N	F	A	T	H	R	S
A	D	I	I	Q	D	S	S	D	N	I	L	T	V	L	N	F	A	T	H	R	S
A	D	I	I	Q	D	S	S	D	N	I	L	T	V	L	N	F	A	T	H	R	S
A	D	I	I	Q	D	S	S	D	N	I	L	T	V	L	N	F	A	T	H	R	S
A	D	I	I	Q	D	S	S	D	N	I	L	T	V	L	N	F	A	T	H	R	S
A	D	I	I	Q	D	S	S	D	N	I	L	T	V	L	N	F	A	T	H	R	S
A	D	I	I	Q	D	S	S	D	N	I	L	T	V	L	N	F	A	T	H	R	S
A	D	I	I	Q	D	S	S	D	N	I	L	T	V	L	N	F	A	T	H	R	S
A	D	I	I	Q	D	S	S	D	N	I	L	T	V	L	N	F	A	T	H	R	S
A	D	I	I	Q	D	S	S	D	N	I	L	T	V	L	N	F	A	T	H	R	S
A	D	I	I	Q	D	S	S	D	N	I	L	T	V	L	N	F	A	T	H	R	S
A	D	I	I	Q	D	S	S	D	N	I	L	T	V	L	N	F	A	T	H	R	S
A	D	I	I	Q	D	S	S	D	N	I	L	T	V	L	N	F	A	T	H	R	S
A	D	I	I	Q	D	S	S	D	N	I	L	T	V	L	N	F	A	T	H	R	S
A	D	I	I	Q	D	S	S	D	N	I	L	T	V	L	N	F	A	T	H	R	S
A	D	I	I	Q	D	S	S	D	N	I	L	T	V	L	N	F	A	T	H	R	S
A	D	I	I	Q	D	S	S	D	N	I	L	T	V	L	N	F	A	T	H	R	S
A	D	I	I	Q	D	S	S	D	N	I	L	T	V	L	N	F	A	T	H	R	S
A	D	I	I	Q	D	S	S	D	N	I	L	T	V	L	N	F	A	T	H	R	S
A	D	I	I	Q	D	S	S	D	N	I	L	T	V	L	N	F	A	T	H	R	S
A	D	I	I	Q	D	S	S	D	N	I	L	T	V	L	N	F	A	T	H	R	S
A	D	I	I	Q	D	S	S	D	N	I	L	T	V	L	N	F	A	T	H	R	S
A	D	I	I	Q	D	S	S	D	N	I	L	T	V	L	N	F	A	T	H	R	S

A	D	I	I	Q	D	S	S	D	N	I	L	T	V	L	N	F	A	T	H	R	S
A	D	I	I	Q	D	S	S	D	N	I	L	T	V	L	N	F	A	T	H	R	S
A	D	I	I	Q	D	S	S	D	N	I	L	T	V	L	N	F	A	T	H	R	S
A	D	I	I	Q	D	S	S	D	N	I	L	T	V	L	N	F	A	T	H	R	S
A	D	I	I	Q	D	S	S	D	N	I	L	T	V	L	N	F	A	T	H	R	S
A	D	I	I	Q	D	S	S	D	N	I	L	T	V	L	N	F	A	T	H	R	S
A	D	I	I	Q	D	S	S	D	N	I	L	T	V	L	N	F	A	T	H	R	S
A	D	I	I	Q	D	S	S	D	N	I	L	T	V	L	N	F	A	T	H	R	S
A	D	I	I	Q	D	S	S	D	N	I	L	T	V	L	N	F	A	T	H	R	S
A	D	I	I	Q	D	S	S	D	N	I	L	T	V	L	N	F	A	T	H	R	S
A	D	I	I	Q	D	S	S	D	N	I	L	T	V	L	N	F	A	T	H	R	S
A	D	I	I	Q	D	S	S	D	N	I	L	T	V	L	N	F	A	T	H	R	S
A	D	I	I	Q	D	S	S	D	N	I	L	T	V	L	N	F	A	T	H	R	S
A	D	I	I	Q	D	S	S	D	N	I	L	T	V	L	N	F	A	T	H	R	S
A	D	I	I	Q	D	S	S	D	N	I	L	T	V	L	N	F	A	T	H	R	S
A	D	I	I	Q	D	S	S	D	N	I	L	T	V	L	N	F	A	T	H	R	S
A	D	I	I	Q	D	S	S	D	N	I	L	T	V	L	N	F	A	T	H	R	S
A	D	I	I	Q	D	S	S	D	N	I	L	T	V	L	N	F	A	T	H	R	S
A	D	I	I	Q	D	S	S	D	N	I	L	T	V	L	N	F	A	T	H	R	S
A	D	I	I	Q	D	S	S	D	N	I	L	T	V	L	N	F	A	T	H	R	S
T	G	M	V	R	N	N	N	N	S	L	V	T	E	F	N	L	A	T	Q	R	S
T	G	M	V	R	N	N	N	N	S	L	V	T	E	F	N	L	A	T	Q	R	S
T	G	M	V	R	N	N	N	N	S	L	V	T	E	F	N	L	A	T	Q	R	S
T	G	M	V	R	N	N	N	N	S	L	V	T	E	F	N	L	A	T	Q	R	S
T	G	M	V	R	N	N	N	N	S	L	V	T	E	F	N	L	A	T	Q	R	S
T	G	M	V	R	N	N	N	N	S	L	V	T	E	F	N	L	A	T	Q	R	S
T	G	M	V	R	N	N	N	N	S	L	V	T	E	F	N	L	A	T	Q	R	S
T	G	M	V	R	N	N	N	N	S	L	V	T	E	F	N	L	A	T	Q	R	S
A	D	I	I	Q	D	S	S	D	N	I	L	T	I	L	N	F	A	T	H	R	S
A	D	I	I	Q	D	S	S	D	N	I	L	T	V	L	N	F	A	T	H	R	S
A	D	I	I	Q	D	S	S	D	N	I	L	T	V	L	N	F	A	T	H	R	S
A	D	I	I	Q	D	S	S	D	N	I	L	T	V	L	N	F	A	T	H	R	S
A	D	I	I	Q	D	S	S	D	N	I	L	T	V	L	N	F	A	T	H	R	S
A	D	I	I	Q	D	S	S	D	N	I	L	T	V	L	N	F	A	T	H	R	S
A	D	I	I	Q	D	S	S	D	N	I	L	T	V	L	N	F	A	T	H	R	S
A	D	I	I	Q	D	S	S	D	N	I	L	T	V	L	N	F	A	T	H	R	S
A	D	I	I	Q	D	S	S	D	N	I	L	T	V	L	N	F	A	T	H	R	S
A	D	I	I	Q	D	S	S	D	N	I	L	T	V	L	N	F	A	T	H	R	S
A	D	I	I	Q	D	S	S	D	N	I	L	T	V	L	N	F	A	T	H	R	S
A	D	I	I	Q	D	S	S	D	N	I	L	T	V	L	N	F	A	T	H	R	S
A	D	I	I	Q	D	S	S	D	N	I	L	T	V	L	N	F	A	T	H	R	S
A	D	I	I	Q	D	S	S	D	N	I	L	T	V	L	N	F	A	T	H	R	S
A	D	I	I	Q	D	S	S	D	N	I	L	T	V	L	N	F	A	T	H	R	S
A	D	I	I	Q	D	S	S	D	N	I	L	T	V	L	N	F	A	T	H	R	S
A	D	I	I	Q	D	S	S	D	N	I	L	T	V	L	N	F	A	T	H	R	S
A	D	I	I	Q	D	S	S	D	N	I	L	T	V	L	N	F	A	T	H	R	S
A	D	I	I	Q	D	S	S	D	N	I	L	T	V	L	N	F	A	T	H	R	S
A	D	I	I	Q	D	S	S	D	N	I	L	T	V	L	N	F	A	T	H	R	S
A	D	I	I	Q	D	S	S	D	N	I	L	T	V	L	N	F	A	T	H	R	S
A	D	I	I	Q	D	S	S	D	N	I	L	T	V	L	N	F	A	T	H	R	S
A	D	I	I	Q	D	S	S	D	N	I	L	T	V	L	N	F	A	T	H	R	S
A	D	I	I	Q	D	S	S	D	N	I	L	T	V	L	N	F	A	T	H	R	S
A	D	I	I	Q	D	S	S	D	N	I	L	T	V	L	N	F	A	T	H	R	S
A	D	I	I	Q	D	S	S	D	N	I	L	T	V	L	N	F	A	T	H	R	S
A	D	I	I	Q	D	S	S	D	N	I	L	T	V	L	N	F	A	T	H	R	S
A	D	I	I	Q	D	S	S	D	N	I	L	T	V	L	N	F	A	T	H	R	S
A	D	I	I	Q	D	S	S	D	N	I	L	T	V	L	N	F	A	T	H	R	S
A	D	I	I	Q	D	S	S	D	N	I	L	T	V	L	N	F	A	M	H	R	S
A	D	I	I	Q	D	S	S	D	N	I	L	A	V	L	N	F	A	T	H	R	S
A	D	I	I	Q	D	S	S	D	N	I	L	A	V	L	N	F	A	T	H	R	S
A	D	I	I	Q	D	S	S	D	N	I	L	A	V	L	N	F	A	T	H	R	S
A	D	I	I	Q	D	S	S	D	N	I	L	A	V	L	N	F	A	T	H	R	S
A	D	I	I	Q	D	S	S	D	N	I	L	A	V	L	N	F	A	T	H	R	S
A	D	I	I	Q	D	S	S	D	N	I	L	A	V	L	N	F	A	T	H	R	S
A	D	I	I	Q	D	S	S	D	N	I	L	A	V	L	N	F	A	T	H	R	S
A	D	I	I	Q	D	S	S	D	N	I	L	A	V	L	N	F	A	T	H	R	S
A	D	I	I	Q	D	S	S	D	N	I	L	A	V	L	N	F	A	T	H	R	S

A	D	I	I	Q	D	S	S	D	N	I	L	A	V	L	N	F	A	T	H	R	S
A	D	I	I	Q	D	S	S	D	N	I	L	A	V	L	N	F	A	T	H	R	S
A	D	I	I	Q	D	S	S	D	N	I	L	A	V	L	N	F	A	T	H	R	S
A	D	I	I	Q	D	S	S	D	N	I	L	A	V	L	N	F	A	T	H	R	S
A	D	I	I	Q	D	S	S	D	N	I	L	A	V	L	N	F	A	T	H	R	S
A	D	I	I	Q	D	S	S	D	N	I	L	A	V	L	N	F	A	T	H	R	S
A	D	I	I	Q	D	S	S	D	N	I	L	A	V	L	N	F	A	T	H	R	S
A	D	I	I	Q	D	S	S	D	N	I	L	A	V	L	N	F	A	T	H	R	S
A	D	I	I	Q	D	S	S	D	N	I	L	T	V	L	N	F	A	T	H	R	S
A	D	I	I	Q	D	S	S	D	N	I	L	T	V	L	N	F	A	T	H	R	S
A	D	I	I	Q	D	S	S	D	N	I	L	T	V	L	N	F	A	T	H	R	S
A	D	I	I	Q	D	S	S	D	N	I	L	T	V	L	N	F	A	T	H	R	S
A	D	I	I	Q	D	S	S	D	N	I	L	T	V	L	N	F	A	T	H	R	S
A	D	I	I	Q	D	S	S	D	N	I	L	T	V	L	N	F	A	T	H	R	S
A	D	I	I	Q	D	S	S	D	N	I	L	T	V	L	N	F	A	T	H	R	S
A	D	I	I	Q	D	S	S	D	N	I	L	T	V	L	N	F	A	T	H	R	S
A	D	I	I	Q	D	S	S	D	N	I	L	T	V	L	N	F	A	T	H	R	S
A	D	I	I	Q	D	S	S	D	N	I	L	T	V	L	N	F	A	T	H	R	S
A	D	I	I	Q	D	S	S	D	N	I	L	T	V	L	N	F	A	T	H	R	S
A	D	I	I	Q	D	S	S	D	N	I	L	T	V	L	N	F	A	T	H	R	S
A	D	I	I	Q	D	S	S	D	N	I	L	T	V	L	N	F	A	T	H	R	S
A	D	I	I	Q	D	S	S	D	N	I	L	T	V	L	N	F	A	T	H	R	S
A	D	I	I	Q	D	S	S	D	N	I	L	T	V	L	N	F	A	T	H	R	S
A	D	I	I	Q	D	S	S	D	N	I	L	T	I	L	N	F	A	T	H	R	S
A	D	I	I	Q	D	S	S	D	N	I	L	T	V	L	N	F	T	T	H	R	S
A	D	I	I	Q	D	S	S	D	N	I	L	T	V	L	N	F	A	T	H	R	S
A	D	I	I	Q	D	S	S	D	N	I	L	T	V	L	N	F	A	T	H	R	S
A	D	I	I	Q	D	S	S	D	N	I	L	T	V	L	N	F	A	T	H	R	S
A	D	I	I	Q	D	S	S	D	N	I	L	T	V	L	N	F	A	T	H	R	S
A	D	I	I	Q	D	S	S	D	N	I	L	T	V	L	N	F	A	T	H	R	S
A	D	I	I	Q	D	S	S	D	N	I	L	T	V	L	N	F	A	T	H	R	S
A	D	I	I	Q	D	S	S	D	N	I	L	T	V	L	N	F	A	T	H	R	S
A	D	I	I	Q	D	S	S	D	N	I	L	T	V	L	N	F	A	T	H	R	S
A	D	I	I	Q	D	S	S	D	N	I	L	T	V	L	N	F	A	T	H	R	S
A	D	I	I	Q	D	S	S	D	N	I	L	T	V	L	N	F	A	T	H	R	S
A	D	I	I	Q	D	S	S	D	N	I	L	T	V	L	N	F	A	T	H	R	S
A	D	I	I	Q	D	S	S	D	N	I	L	T	V	L	N	F	A	T	H	R	S
A	D	I	I	Q	D	S	S	D	N	I	L	T	V	L	S	F	A	T	H	R	S
A	D	I	I	Q	D	S	S	D	N	I	L	T	V	L	N	F	A	T	H	R	S
A	D	I	I	Q	D	S	S	D	N	I	L	T	V	L	N	F	A	T	H	R	S
A	D	I	I	Q	D	S	S	D	N	I	L	T	V	L	N	F	A	T	H	R	S
A	D	I	I	Q	D	S	S	D	N	I	L	T	V	L	N	F	A	T	H	R	S
A	D	I	I	Q	D	S	S	D	N	I	L	T	V	L	N	F	A	T	H	R	S
A	D	I	I	Q	D	S	S	D	N	I	L	T	V	L	N	F	A	T	H	R	S
A	D	I	I	Q	D	S	S	D	N	I	L	T	V	L	N	F	A	T	H	R	S
A	D	I	I	Q	D	S	S	D	N	I	L	T	V	L	N	F	A	T	H	R	S
A	D	I	I	Q	D	S	S	D	N	I	L	T	V	L	N	F	A	T	H	R	S
A	D	I	I	Q	D	S	S	D	N	I	L	T	V	L	N	F	A	T	H	R	S
A	D	I	I	Q	D	S	S	D	N	I	L	T	V	L	N	F	A	T	H	R	S
A	D	I	I	Q	D	S	S	D	N	I	L	T	I	L	N	F	A	T	H	R	S
A	D	I	I	Q	D	S	S	D	N	I	L	T	I	L	N	F	A	T	H	R	S
A	D	I	I	Q	D	S	S	D	N	I	L	T	I	L	N	F	A	T	H	R	S
A	D	I	I	Q	D	S	S	D	N	I	L	T	I	L	N	F	A	T	H	R	S
A	D	I	I	Q	D	S	S	D	N	I	L	T	I	L	N	F	A	T	H	R	S
A	D	I	I	Q	D	S	S	D	N	I	L	T	I	L	N	F	A	T	H	R	S
A	D	I	I	Q	D	S	S	D	N	I	L	T	I	L	N	F	A	T	H	R	S
A	D	I	I	Q	D	S	S	D	N	I	L	T	I	L	N	F	A	T	H	R	S
A	D	I	I	Q	D	S	S	D	N	I	L	T	I	L	N	F	A	T	H	R	S
A	D	I	I	Q	D	S	S	D	N	I	L	T	I	L	N	F	A	T	H	R	S
A	D	I	I	Q	D	S	S	D	N	I	L	T	I	L	N	F	A	T	H	R	S
A	D	I	I	Q	D	S	S	D	N	I	L	T	I	L	N	F	A	T	H	R	S
A	D	I	I	Q	D	S	S	D	N	I	L	T	I	L	N	F	A	T	H	R	S
A	D	I	I	Q	D	S	S	D	N	I	L	T	I	L	N	F	A	T	H	R	S
A	D	I	I	Q	D	S	S	D	N	I	L	T	I	L	N	F	A	T	H	R	S
A	D	I	I	Q	D	S	S	D	N	I	L	T	I	L	N	F	A	T	H	R	S
A	D	I	I	Q	D	S	S	D	N	I	L	T	I	L	N	F	A	T	H	R	S
A	D	I	I	Q	D	S	S	D	N	I	L	T	I	L	N	F	A	T	H	R	S
A	D	I	I	Q	D	S	S	D	N	I	L	T	I	L	N	F	A	T	H	R	S

A	D	I	I	Q	D	S	S	D	N	I	L	T	I	L	N	F	A	T	H	R	S
A	D	I	I	Q	D	S	S	D	N	I	L	T	I	L	N	F	A	T	H	R	S
A	D	I	I	Q	D	S	S	D	N	I	L	T	I	L	N	F	A	T	H	R	S
A	D	I	I	Q	D	S	S	D	N	I	L	T	I	L	N	F	A	T	H	R	S
A	D	I	I	Q	D	S	S	D	N	I	L	T	I	L	N	F	A	T	H	R	S
A	D	I	I	Q	D	S	S	D	N	I	L	T	I	L	N	F	A	T	H	R	S
A	D	I	I	Q	D	S	S	D	N	I	L	T	I	L	N	F	A	T	H	R	S
A	D	I	I	Q	D	S	S	D	N	I	L	T	I	L	N	F	A	T	H	R	S
A	D	I	I	Q	D	S	S	D	N	I	L	T	I	L	N	F	A	T	H	R	S
A	D	I	I	Q	D	S	S	D	N	I	L	T	I	L	N	F	A	T	H	R	S

wzy																						
89	90	103	107	117	118	130	153	163	171	172	178	218	220	233	239	250	253	331	375	378	384	399
H	E	V	T	I	R	S	N	S	L	I	M	V	S	P	K	T	I	V	V	Y	V	I
Y	K	G	I	I	H	G	D	A	F	V	I	I	T	H	R	A	M	I	I	H	G	L
Y	E	V	I	T	R	S	-	S	L	I	I	V	A	P	K	A	I	V	V	Y	V	I
Y	E	V	I	T	R	S	-	S	L	I	I	V	A	P	K	A	I	I	I	Y	V	I
Y	E	V	I	T	R	S	-	S	L	I	I	V	A	P	K	A	I	I	I	Y	V	I
Y	E	V	I	T	R	S	-	S	L	I	I	V	A	P	K	A	I	I	I	Y	V	I
Y	E	V	I	T	R	S	-	S	L	I	I	V	A	P	K	A	I	I	I	Y	V	I
Y	E	V	I	T	R	S	-	S	L	I	I	V	A	P	K	A	I	I	I	Y	V	I
Y	E	V	I	T	R	S	-	S	L	I	I	V	A	P	K	A	I	I	I	Y	V	I
Y	E	V	I	T	R	S	-	S	L	I	I	V	A	P	K	A	I	I	I	Y	V	I
Y	E	V	I	T	R	S	-	S	L	I	I	V	A	P	K	A	I	I	I	Y	V	I

wzy																					
153	163	171	172	178	218	220	233	239	250	253	331	375	378	384	399	403	444	14	22	89	106
N	S	L	I	M	V	S	P	K	T	I	V	V	Y	V	I	G	T	F	V	F	I
D	A	F	V	I	I	T	H	R	A	M	I	I	H	G	L	S	R	L	L	S	T
N	S	L	I	M	V	S	P	K	T	I	V	V	Y	V	I	G	T	F	V	S	I
N	S	L	I	M	V	S	P	K	T	I	V	V	Y	V	I	G	T	F	V	S	I
N	S	L	I	M	V	S	P	K	T	I	V	V	Y	V	I	G	T	F	V	S	I
N	S	L	I	M	V	S	P	K	T	I	V	V	Y	V	I	G	T	F	V	S	I
N	S	L	I	M	V	S	P	K	T	I	V	V	Y	V	I	G	T	F	V	S	I
N	S	L	I	M	V	S	P	K	T	I	V	V	Y	V	I	G	T	F	V	S	I
N	S	L	I	M	V	S	P	K	T	I	V	V	Y	V	I	G	T	F	V	S	I
N	S	L	I	M	V	S	P	K	T	I	V	V	Y	V	I	G	T	F	V	S	I
N	S	L	I	M	V	S	P	K	T	I	V	V	Y	V	I	G	T	F	V	S	I
N	S	L	I	M	V	S	P	K	T	I	V	V	Y	V	I	G	T	F	V	S	I
N	S	L	I	M	V	S	P	K	T	I	V	V	Y	V	I	G	T	F	V	S	I
N	S	L	I	M	V	S	P	K	T	I	V	V	Y	V	I	G	T	F	V	S	I
N	S	L	I	M	V	S	P	K	T	I	V	V	Y	V	I	G	T	F	V	S	I
N	S	L	I	M	V	S	P	K	T	I	V	V	Y	V	I	G	T	F	V	S	I
N	S	L	I	M	V	S	P	K	T	I	V	V	Y	V	I	G	T	F	V	S	I
N	S	L	I	M	V	S	P	K	T	I	V	V	Y	V	I	G	T	F	V	S	I
N	S	L	I	M	V	S	P	K	T	I	V	V	Y	V	I	G	T	F	V	S	I
N	S	L	I	M	V	S	P	K	T	I	V	V	Y	V	I	G	T	F	V	S	I
N	S	L	I	M	V	S	P	K	T	I	V	V	Y	V	I	G	T	F	V	S	I
N	S	L	I	M	V	S	P	K	T	I	V	V	Y	V	I	G	T	F	V	S	I
N	S	L	I	M	V	S	P	K	T	I	V	V	Y	V	I	G	T	F	V	S	I
N	S	L	I	M	V	S	P	K	T	I	V	V	Y	V	I	G	T	F	V	S	I
N	S	L	I	M	V	S	P	K	T	I	V	V	Y	V	I	G	T	F	V	S	I
N	S	L	I	I	V	A	P	K	A	I	V	V	Y	V	I	S	M	F	V	S	I
N	S	L	I	M	V	S	P	K	T	I	V	V	Y	V	I	G	T	F	V	S	I
N	S	L	I	M	V	S	P	K	T	I	V	V	Y	V	I	G	T	F	V	S	I
N	S	L	I	M	V	S	P	K	T	I	V	V	Y	V	I	G	T	F	V	S	I
N	S	L	I	M	V	S	P	K	T	I	V	V	Y	V	I	G	T	F	V	S	I
N	S	L	I	M	V	S	P	K	T	I	V	V	Y	V	I	G	T	F	V	S	I

rmlD																		
95	120	140	141	142	144	145	167	198	199	202	205	215	225	231	236	242	245	275
T	R	K	H	V	N	F	N	L	A	R	F	T	V	T	K	Q	V	Q
T	R	K	H	V	N	F	N	L	A	R	F	T	V	T	K	Q	A	Q
I	I	N	L	T	Q	H	S	V	T	Q	Y	A	S	S	V	K	A	K
T	R	K	H	V	N	F	N	L	A	R	F	T	V	T	K	Q	A	Q

wzy

171	172	178	218	220	233	239	250	253	331	375	378	384	399	403	444
L	I	M	V	S	P	K	T	I	V	V	Y	V	I	G	T
F	V	I	I	T	H	R	A	M	I	I	H	G	L	S	R
L	I	I	V	A	P	K	A	I	V	V	Y	V	I	S	T
L	I	I	V	A	P	K	A	I	V	V	Y	V	I	S	T
L	I	I	V	A	P	K	A	I	V	V	Y	V	I	S	T
L	I	I	V	A	P	K	A	I	V	V	Y	V	I	S	T
L	I	I	V	A	P	K	A	I	V	V	Y	V	I	S	T
L	I	I	V	A	P	K	A	I	V	V	Y	V	I	S	T
L	I	I	V	A	P	K	A	I	V	V	Y	V	I	S	T
L	I	I	V	A	P	K	A	I	V	V	Y	V	I	S	T
L	I	I	V	A	P	K	A	I	V	V	Y	V	I	S	T
L	I	I	V	A	P	K	A	I	V	V	Y	V	I	S	T
L	I	I	V	A	P	K	A	I	V	V	Y	V	I	S	T
L	I	I	V	A	P	K	A	I	V	V	Y	V	I	S	T
L	I	I	V	A	P	K	A	I	V	V	Y	V	I	S	T
L	I	I	V	A	P	K	A	I	V	V	Y	V	I	S	T
L	I	I	V	A	P	K	A	I	V	V	Y	V	I	S	T
L	I	I	V	A	P	K	A	I	V	V	Y	V	I	S	T
L	I	I	V	A	P	K	A	I	V	V	Y	V	I	S	T
L	I	I	V	A	P	K	A	I	V	V	Y	V	I	S	T
L	I	I	V	A	P	K	A	I	V	V	Y	V	I	S	T
L	I	I	V	A	P	K	A	I	V	V	Y	V	I	S	T
L	I	I	V	A	P	K	A	I	V	V	Y	V	I	S	T
L	I	I	V	A	P	K	A	I	V	V	Y	V	I	S	T
L	I	I	V	A	P	K	A	I	V	V	Y	V	I	S	T
L	I	I	V	A	P	K	A	I	V	V	Y	V	I	S	T
L	I	I	V	A	P	K	A	I	V	V	Y	V	I	S	T

14	22	89	106	110	157
F	V	F	I	P	I
L	L	S	T	Q	V
F	V	S	I	P	I
F	V	S	I	P	I
F	V	S	I	P	I
F	V	S	I	P	I
F	V	S	I	P	I
F	V	S	I	P	I
F	V	S	I	P	I
F	V	S	I	P	I
F	V	S	I	P	I
F	V	S	I	P	I
F	V	S	I	P	I
F	V	S	I	P	I
F	V	S	I	P	I
F	V	S	I	P	I
F	V	S	I	P	I
F	V	S	I	P	I
F	V	S	I	P	I
F	V	S	I	P	I
F	V	S	I	P	I
F	V	S	I	P	I
F	V	S	I	P	I
F	V	S	I	P	I
F	V	S	I	P	I
F	V	S	I	P	I
F	V	S	I	P	I

178	220	250	403	444
M	S	T	G	T
I	A	A	S	T
I	A	A	S	M
I	A	A	S	M
I	A	A	S	M
I	A	A	S	M
I	A	A	S	M
I	A	A	S	M
I	A	A	S	M
I	A	A	S	M
I	A	A	S	M
I	A	A	S	M
I	A	A	S	M
I	A	A	S	M
I	A	A	S	M
I	A	A	S	M
I	A	A	S	M
I	A	A	S	M
I	A	A	S	M
I	A	A	S	M
I	A	A	S	M
I	A	A	S	M
I	A	A	S	M
I	A	A	S	M
I	A	A	S	M
I	A	A	S	M
I	A	A	S	M
I	A	A	S	M

wzx				
20	89	112	254	287
T	F	A	T	I
T	F	A	M	I
T	S	S	M	V
T	S	S	M	V
T	S	S	M	V
T	S	S	M	V
T	S	S	M	V
T	S	S	M	V
T	S	S	M	V
T	S	S	M	V
T	S	S	M	V
T	S	S	M	V
T	S	S	M	V
T	S	S	M	V
T	S	S	M	V
T	S	S	M	V
T	S	S	M	V
T	S	S	M	V
T	S	S	M	V
T	S	S	M	V
T	S	S	M	V
I	S	S	M	V
T	S	S	M	V
T	S	S	M	V
T	S	S	M	V
T	S	S	M	V
T	S	S	M	V
T	S	S	M	V

rmlA										
69	95	118	123	124	125	128	136	150	151	153
S	E	T	A	A	K	K	H	E	N	Y
S	E	K	A	A	K	K	Q	T	D	Y
Y	K	K	V	S	S	S	H	K	D	H
S	K	K	V	A	S	S	H	K	D	H
S	K	K	V	A	S	S	H	K	D	H
S	K	K	V	A	S	S	H	K	D	H
S	K	K	V	A	S	S	H	K	D	H
S	K	K	V	A	S	S	H	K	D	H
S	K	K	V	A	S	S	H	K	D	H
S	K	K	V	A	S	S	H	K	D	H
S	K	K	V	A	S	S	H	K	D	H
S	K	K	V	A	S	S	H	K	D	H
S	K	K	V	A	S	S	H	K	D	H
S	K	K	V	A	S	S	H	K	D	H
S	K	K	V	A	S	S	H	K	D	H
S	K	K	V	A	S	S	H	K	D	H
S	K	K	V	A	S	S	H	K	D	H
S	K	K	V	A	S	S	H	K	D	H
S	K	K	V	A	S	S	H	K	D	H
S	K	K	V	A	S	S	H	K	D	H
S	K	K	V	A	S	S	H	K	D	H
S	K	K	V	A	S	S	H	K	D	H
S	K	K	V	A	S	S	H	K	D	H
S	K	K	V	A	S	S	H	K	D	H
S	K	K	V	A	S	S	H	K	D	H
S	K	K	V	A	S	S	H	K	D	H
S	K	K	V	A	S	S	H	K	D	H
S	K	K	V	A	S	S	H	K	D	H
S	K	K	V	A	S	S	H	K	D	H

I	A	A	S	M
I	A	A	S	M

T	S	S	M	V
T	S	S	M	V

S	K	K	V	A	S	S	H	K	D	H
S	K	K	V	A	S	S	H	K	D	H

		wzy																		
276	318	70	74	89	107	117	178	220	250	331	375	403	444	20	35	89	112	176	203	254
L	T	V	L	H	T	I	M	S	T	V	V	G	T	T	M	F	A	W	E	T
L	T	V	L	H	T	I	M	S	T	V	V	G	T	T	M	F	A	W	E	T
L	T	V	L	H	T	I	M	S	T	V	V	G	T	T	M	F	A	W	D	T
L	T	V	L	H	T	I	M	S	T	V	V	G	T	T	M	F	A	W	D	T
L	T	V	L	H	T	I	M	S	T	V	V	G	T	T	M	F	A	W	D	T
L	T	V	L	H	T	I	M	S	T	V	V	G	T	T	M	F	A	W	D	T
L	T	V	L	H	T	I	M	S	T	V	V	G	T	T	M	F	A	W	D	T
L	T	V	L	H	T	I	M	S	T	V	V	G	T	T	M	F	A	W	D	T
L	T	V	L	H	T	I	M	S	T	V	V	G	T	T	M	F	A	W	D	T
L	T	V	L	H	T	I	M	S	T	V	V	G	T	T	M	F	A	W	D	T
L	T	V	L	H	T	I	M	S	T	V	V	G	T	T	M	F	A	W	D	T
L	T	V	L	H	T	I	M	S	T	V	V	G	T	T	M	F	A	W	D	T
L	T	V	L	H	T	I	M	S	T	V	V	G	T	T	M	F	A	W	D	T
L	T	V	L	H	T	I	M	S	T	V	V	G	T	T	M	F	A	W	D	T
L	T	V	L	H	T	I	M	S	T	V	V	G	T	T	M	F	A	W	D	T
L	T	V	L	H	T	I	M	S	T	V	V	G	T	T	M	F	A	W	D	T
L	T	V	L	H	T	I	M	S	T	V	V	G	T	T	M	F	A	W	D	T
L	T	V	L	H	T	I	M	S	T	V	V	G	T	T	M	F	A	W	D	T
L	T	V	L	H	T	I	M	S	T	V	V	G	T	T	M	F	A	W	D	T
L	T	V	L	H	T	I	M	S	T	V	V	G	T	T	M	F	A	W	D	T
L	T	V	L	H	T	I	M	S	T	V	V	G	T	T	M	F	A	W	D	T
L	T	V	L	H	T	I	M	S	T	V	V	G	T	T	M	F	A	W	D	T
L	T	V	L	H	T	I	M	S	T	V	V	G	T	T	M	F	A	W	D	T
L	T	V	L	H	T	I	M	S	T	V	V	G	T	T	M	F	A	W	D	T
L	T	V	L	H	T	I	M	S	T	V	V	G	T	T	M	F	A	W	D	T
L	T	V	L	H	T	I	M	S	T	V	V	G	T	T	M	F	A	W	D	T
L	T	V	L	H	T	I	M	S	T	V	V	G	T	T	M	F	A	W	D	T
L	T	V	L	H	T	I	M	S	T	V	V	G	T	T	M	F	A	W	D	T
L	T	V	L	H	T	I	M	S	T	V	V	G	T	T	M	F	A	W	D	T
L	T	V	L	H	T	I	M	S	T	V	V	G	T	T	M	F	A	W	D	T
L	T	V	L	H	T	I	M	S	T	V	V	G	T	T	M	F	A	W	D	T
L	T	V	L	H	T	I	M	S	T	V	V	G	T	T	M	S	A	W	E	T
L	T	V	L	H	T	I	M	S	T	V	V	G	T	T	M	S	A	W	E	T
L	T	V	L	H	T	I	M	S	T	V	V	G	T	T	M	S	A	W	E	T
L	T	V	L	H	T	I	M	S	T	V	V	G	T	T	M	S	A	W	E	M
L	T	V	L	H	T	I	M	S	T	V	V	G	T	T	M	S	A	W	E	T
L	T	V	L	H	T	I	M	S	T	V	V	G	T	T	M	S	A	W	E	T
L	T	V	L	H	T	I	M	S	T	V	V	G	T	T	M	S	A	W	E	T
L	T	V	L	H	T	I	M	S	T	V	V	G	T	T	M	S	A	W	E	T
L	T	V	L	H	T	I	M	S	T	V	V	G	T	T	M	S	A	W	E	T
L	T	V	L	H	T	I	M	S	T	V	V	G	T	T	M	S	A	W	E	T
L	T	V	L	H	T	I	M	S	T	V	V	G	T	T	M	S	A	W	E	T
L	T	V	L	H	T	I	M	S	T	V	V	G	T	T	M	S	A	W	E	T
L	T	V	L	H	T	I	M	S	T	V	V	G	T	T	M	S	A	W	E	T
L	T	V	L	H	T	I	M	S	T	V	V	G	T	T	M	S	A	W	E	T
L	T	V	L	H	T	I	M	S	T	V	V	G	T	T	M	S	A	W	E	T
L	T	V	L	H	T	I	M	S	T	V	V	G	T	T	M	S	A	W	E	T
L	T	V	L	H	T	I	M	S	T	V	V	G	T	T	M	S	A	W	E	T
R	T	V	L	H	T	I	M	S	T	V	V	G	T	T	M	S	A	W	E	T
L	T	V	L	H	T	I	M	S	T	V	V	G	T	T	M	S	A	W	E	T
L	T	V	L	H	T	I	M	S	T	V	V	G	T	T	I	S	A	W	E	T
L	T	V	L	H	T	I	M	S	T	V	V	G	T	T	M	S	A	W	E	T
L	T	V	L	H	T	I	M	S	T	V	V	G	T	T	M	S	A	W	E	T
L	T	V	L	H	T	I	M	S	T	V	V	G	T	T	M	S	A	W	E	T
L	T	V	L	H	T	I	M	S	T	V	V	G	T	T	M	S	A	W	E	T
L	T	V	L	H	T	I	M	S	T	V	V	G	T	T	M	S	A	W	E	T
R	T	V	L	H	T	I	M	S	T	V	V	G	T	T	M	S	A	W	E	T
L	T	V	L	H	T	I	M	S	T	V	V	G	T	T	M	S	A	W	E	T
L	T	V	L	H	T	I	M	S	T	V	V	G	T	T	M	S	A	W	E	T
L	T	V	L	H	T	I	M	S	T	V	V	G	T	T	M	S	A	W	E	T
L	T	V	L	H	T	I	M	S	T	V	V	G	T	T	M	S	A	W	E	T
L	T	V	L	H	T	I	M	S	T	V	V	G	T	T	M	S	A	W	E	T
L	T	V	L	H	T	I	M	S	T	V	V	G	T	T	M	S	A	W	E	T
L	T	V	L	H	T	I	M	S	T	V	V	G	T	T	M	S	A	W	E	T

L	T	V	L	H	T	I	M	S	T	V	V	G	T	T	M	S	A	W	E	T
L	T	V	L	H	T	I	M	S	T	V	V	G	T	T	M	S	A	W	E	T
L	T	V	L	H	T	I	M	S	T	V	V	G	T	T	M	S	A	W	E	T
L	T	V	L	H	T	I	M	S	T	V	V	G	T	T	M	S	A	W	E	T
L	T	V	L	H	T	I	M	S	T	V	V	G	T	T	M	S	A	W	E	T
L	T	V	L	H	T	I	M	S	T	V	V	G	T	T	M	S	A	W	E	T
L	T	V	L	H	T	I	M	S	T	V	V	G	T	T	M	S	A	W	E	T
L	T	V	L	H	T	I	M	S	T	V	V	G	T	T	M	S	A	W	E	T
L	T	V	L	H	T	I	M	S	T	V	V	G	T	T	M	S	A	W	E	T
L	T	V	L	H	T	I	M	S	T	V	V	G	T	T	M	S	A	W	E	T
L	T	V	L	H	T	I	M	S	T	V	V	G	T	T	M	S	A	W	E	T
L	T	V	L	H	T	I	M	S	T	V	V	G	T	T	M	S	A	W	E	T
L	T	V	L	H	T	I	M	S	T	V	V	G	T	T	M	S	A	W	E	T
L	T	V	L	H	T	I	M	S	T	V	V	G	T	T	M	S	A	W	E	T
L	T	V	L	H	T	I	M	S	T	V	V	G	T	T	M	S	A	W	E	T
L	T	V	L	H	T	I	M	S	T	V	V	G	T	T	M	S	A	W	E	T
L	T	V	L	H	T	I	M	S	T	V	V	G	T	T	M	S	A	W	E	T
L	T	V	L	H	T	I	M	S	T	V	V	G	T	T	M	S	A	W	E	T
L	T	V	L	H	T	I	M	S	T	V	V	G	T	T	M	S	A	W	E	T
L	T	V	L	H	T	I	M	S	T	V	V	G	T	T	M	S	A	W	E	T
L	T	V	L	H	T	I	M	S	T	V	V	G	T	T	M	S	A	W	E	T
L	T	V	L	H	T	I	M	S	T	V	V	G	T	T	M	S	A	W	E	T
L	T	V	L	H	T	I	M	S	T	V	V	G	T	T	M	S	A	W	E	T
L	T	V	L	H	T	I	M	S	T	V	V	G	T	T	M	S	A	W	E	T
L	T	V	L	H	T	I	M	S	T	V	V	G	T	T	M	S	A	W	E	T
L	T	V	L	H	T	I	M	S	T	V	V	G	T	T	M	S	A	W	E	T
L	T	V	L	H	T	I	M	S	T	V	V	G	T	T	M	S	A	W	E	T
L	T	V	L	H	T	I	M	S	T	V	V	G	T	T	M	F	A	W	E	T
L	T	V	L	H	T	I	M	S	T	V	V	G	T	T	M	F	A	W	E	T
L	T	V	L	H	T	I	M	S	T	V	V	G	T	T	M	F	A	W	E	T
L	T	V	L	H	T	I	M	S	T	V	V	G	T	T	M	F	A	W	E	T
L	T	V	L	H	T	I	M	S	T	V	V	G	T	T	M	F	A	W	E	T
L	T	V	L	H	T	I	M	S	T	V	V	G	T	T	M	F	A	W	E	T
L	T	V	L	H	T	I	M	S	T	V	V	G	T	T	M	F	A	W	E	T
L	T	V	L	H	T	I	M	S	T	V	V	G	T	T	M	F	A	W	E	T
L	T	V	L	H	T	I	M	S	T	V	V	G	T	T	M	F	A	W	E	T
L	T	V	L	H	T	I	M	S	T	V	V	G	T	T	M	F	A	W	E	T
L	T	V	L	H	T	I	M	S	T	V	V	G	T	T	M	F	A	W	E	T
L	T	V	L	H	T	I	M	S	T	V	V	G	T	T	M	F	A	W	E	T
L	T	V	L	H	T	I	M	S	T	V	V	G	T	T	M	F	A	W	E	T
L	T	V	L	H	T	I	M	S	T	V	V	G	T	T	M	F	A	W	E	T
L	T	V	L	H	T	I	M	S	T	V	V	G	T	T	M	F	A	W	E	T
L	T	V	L	H	T	I	M	S	T	V	V	G	T	T	M	F	A	W	E	T
L	T	V	L	H	T	I	M	S	T	V	V	G	T	T	M	F	A	W	E	T
L	T	V	L	H	T	I	M	S	T	V	V	G	T	T	M	F	A	W	E	T
L	T	V	L	H	T	I	M	S	T	V	V	G	T	T	M	F	A	W	E	T
L	T	V	L	H	T	I	M	S	T	V	V	G	T	T	M	F	A	W	E	T
L	T	V	L	H	T	I	M	S	T	V	V	G	T	T	M	F	A	W	E	T
L	T	V	L	H	T	I	M	S	T	V	V	G	T	T	M	F	A	W	E	T
L	T	V	L	H	T	I	M	S	T	V	V	G	T	T	M	F	A	W	E	T
L	T	V	L	H	T	I	M	S	T	V	V	G	T	T	M	F	A	W	E	T
L	T	V	L	H	T	I	M	S	T	V	V	G	T	T	M	F	A	W	E	T
L	T	V	L	H	T	I	M	S	T	V	V	G	T	T	M	F	A	W	E	T
L	T	V	L	H	T	I	M	S	T	V	V	G	T	T	M	F	A	W	E	T
L	T	V	L	H	T	I	M	S	T	V	V	G	T	T	M	F	A	W	E	T
L	T	V	L	H	T	I	M	S	T	V	V	G	T	T	M	F	A	W	E	T
L	T	V	L	H	T	I	M	S	T	V	V	G	T	T	M	F	A	W	E	T
L	T	V	L	H	T	I	M	S	T	V	V	G	T	T	M	F	A	W	E	T
L	T	V	L	H	T	I	M	S	T	V	V	G	T	T	M	F	A	W	E	T
L	T	V	L	H	T	I	M	S	T	V	V	G	T	T	M	F	A	W	E	T
L	T	V	L	H	T	I	M	S	T	V	V	G	T	T	M	F	A	W	E	T
L	T	V	L	H	T	I	M	S	T	V	V	G	T	T	M	F	A	W	E	T
L	T	V	L	H	T	I	M	S	T	V	V	G	T	T	M	F	A	W	E	T
L	T	V	L	H	T	I	M	S	T	V	V	G	T	T	M	F	A	W	E	T
L	T	V	L	H	T	I	M	S	T	V	V	G	T	T	M	F	A	W	E	T
L	T	V	L	H	T	I	M	S	T	V	V	G	T	T	M	F	A	W	E	T
L	T	V	L	H	T	I	M	S	T	V	V	G	T	T	M	F	A	W	E	T

L	T	V	L	H	T	I	M	S	T	V	V	G	T	T	M	F	A	W	E	T
L	T	V	L	H	T	I	M	S	T	V	V	G	T	T	M	F	A	W	E	T
L	T	V	L	H	T	I	M	S	T	V	V	G	T	T	M	F	A	W	E	T
L	T	V	L	H	T	I	M	S	T	V	V	G	T	T	M	F	A	W	E	T
L	T	V	L	H	T	I	M	S	T	V	V	G	T	T	M	F	A	W	E	T
L	T	V	L	H	T	I	M	S	T	V	V	G	T	T	M	F	A	W	E	T
L	T	V	L	H	T	I	M	S	T	V	V	G	T	T	M	F	A	W	E	T
L	T	V	L	H	T	I	M	S	T	V	V	G	T	T	M	F	A	W	E	T
L	T	V	L	H	T	I	M	S	T	V	V	G	T	T	M	F	A	W	E	T
L	T	V	L	H	T	I	M	S	T	V	V	G	T	T	M	F	A	W	E	T
L	T	V	L	H	T	I	M	S	T	V	V	G	T	T	M	F	A	W	E	T
L	T	V	L	H	T	I	M	S	T	V	V	G	T	T	M	F	A	W	E	T
L	T	V	L	H	T	I	M	S	T	V	V	G	T	T	M	F	A	W	E	T
L	T	V	L	H	T	I	M	S	T	V	V	G	T	T	M	F	A	W	E	T
L	T	V	L	H	T	I	M	S	T	V	V	G	T	T	M	F	A	W	E	T
L	T	V	L	H	T	I	M	S	T	V	V	G	T	T	M	F	A	W	E	T
L	T	V	L	H	T	I	M	S	T	V	V	G	T	T	M	F	A	W	E	T
L	T	V	L	H	T	I	M	S	T	V	V	G	T	T	M	F	A	W	E	T
L	T	V	L	H	T	I	M	S	T	V	V	G	T	T	M	F	A	W	E	T
L	T	V	L	H	T	I	M	S	T	V	V	G	T	T	M	F	A	W	E	T
L	T	V	L	H	T	I	M	S	T	V	V	G	T	T	M	F	A	W	E	T
L	T	V	L	H	T	I	M	S	T	V	V	G	T	T	M	F	A	W	E	T
L	T	V	L	H	T	I	M	S	T	V	V	G	T	T	M	F	A	W	E	T
L	T	V	L	H	T	I	M	S	T	V	V	G	T	T	M	F	A	W	E	T
L	T	V	L	H	T	I	M	S	T	V	V	G	T	T	M	F	A	W	E	T
L	T	V	L	H	T	I	M	S	T	V	V	G	T	T	M	F	A	W	E	T
L	T	V	L	H	T	I	M	S	T	V	V	G	T	T	M	F	A	W	E	T
L	T	V	L	H	T	I	M	S	T	V	V	G	T	T	M	F	A	W	E	T
L	T	V	L	H	T	I	M	S	T	V	V	G	T	T	M	F	A	W	E	T
L	T	V	L	H	T	I	M	S	T	V	V	G	T	T	M	F	A	W	E	T
L	T	V	L	H	T	I	M	S	T	V	V	G	T	T	M	F	A	W	E	T
L	T	V	L	H	T	I	M	S	T	V	V	G	T	T	M	F	A	W	E	T
L	T	V	L	H	T	I	M	S	T	V	V	G	T	T	M	F	A	W	E	T
L	T	V	L	H	T	I	M	S	T	V	V	G	T	T	M	F	A	W	E	T
L	T	V	L	H	T	I	M	S	T	V	V	G	T	T	M	F	A	W	E	T
L	T	V	L	H	T	I	M	S	T	V	V	G	T	T	M	F	A	W	E	T
L	T	V	L	H	T	I	M	S	T	V	V	G	T	T	M	F	A	W	E	T
L	T	V	L	H	T	I	M	S	T	V	V	G	T	T	M	F	A	W	E	T
L	T	V	L	H	T	I	M	S	T	V	V	G	T	T	M	F	A	W	E	T
L	T	V	L	H	T	I	M	S	T	V	V	G	T	T	M	F	A	W	E	T
L	T	V	L	H	T	I	M	S	T	V	V	G	T	T	M	F	A	W	E	T
L	T	V	L	H	T	I	M	S	T	V	V	G	T	T	M	F	A	W	E	T
L	T	V	L	H	T	I	M	S	T	V	V	G	T	T	M	F	A	W	E	T
L	T	V	L	H	T	I	M	S	T	V	V	G	T	T	M	F	A	W	E	T
L	T	V	L	H	T	I	M	S	T	V	V	G	T	T	M	F	A	W	E	T
L	T	V	L	H	T	I	M	S	T	V	V	G	T	T	M	F	A	W	E	T
L	T	V	L	H	T	I	M	S	T	V	V	G	T	T	M	F	A	W	E	T
L	T	V	L	H	T	I	M	S	T	V	V	G	T	T	M	F	A	W	E	T
L	T	V	L	H	T	I	M	S	T	V	V	G	T	T	M	F	A	W	E	T
L	T	V	L	H	T	I	M	S	T	V	V	G	T	T	M	F	A	W	E	T
L	T	V	L	H	T	I	M	S	T	V	V	G	T	T	M	F	A	W	E	T
L	T	V	L	H	T	I	M	S	T	V	V	G	T	T	M	F	A	W	E	T
L	T	V	L	H	T	I	M	S	T	V	V	G	T	T	M	F	A	W	E	T
L	T	V	L	H	T	I	M	S	T	V	V	G	T	T	M	F	A	W	E	T
L	T	V	L	H	T	I	M	S	T	V	V	G	T	T	M	F	A	W	E	T
L	T	V	L	H	T	I	M	S	T	V	V	G	T	T	M	F	A	W	E	T
L	T	V	L	H	T	I	M	S	T	V	V	G	T	T	M	F	A	W	E	T
L	T	V	L	H	T	I	M	S	T	V	V	G	T	T	M	F	A	W	E	T
L	T	V	L	H	T	I	M	S	T	V	V	G	T	T	M	F	A	W	E	T
L	T	V	L	H	T	I	M	S	T	V	V	G	T	T	M	F	A	W	E	T
L	T	V	L	H	T	I	M	S	T	V	V	G	T	T	M	F	A	W	E	T
L	T	V	L	H	T	I	M	S	T	V	V	G	T	T	M	F	A	W	E	T
L	T	V	L	H	T	I	M	S	T	V	V	G	T	T	M	F	A	W	E	T
L	T	V	L	H	T	I	M	S	T	V	V	G	T	T	M	F	A	W	E	T
L	T	V	L	H	T	I	M	S	T	V	V	G	T	T	M	F	A	W	E	T
L	T	V	L	H	T	I	M	S	T	V	V	G	T	T	M	F	A	W	E	T
L	T	V	L	H	T	I	M	S	T	V	V	G	T	T	M	F	A	W	E	T

L	T	V	L	H	T	I	M	S	T	V	V	G	T	T	M	F	A	W	E	T
L	T	V	L	H	T	I	M	S	T	V	V	G	T	T	M	F	A	W	E	T
L	T	V	L	H	T	I	M	S	T	V	V	G	T	T	M	F	A	W	E	T
L	T	V	L	H	T	I	M	S	T	V	V	G	T	T	M	F	A	W	E	T
L	T	V	L	H	T	I	M	S	T	V	V	G	T	T	M	S	A	W	E	T
L	T	V	L	H	T	I	M	S	T	V	V	G	T	T	M	S	A	W	E	T
L	T	V	L	H	T	I	M	S	T	V	V	G	T	T	M	S	A	W	E	T
L	T	V	F	H	T	I	M	S	T	V	V	G	T	T	M	S	A	W	E	T
L	T	V	L	H	T	I	M	S	T	V	V	G	T	T	M	S	A	W	E	T
L	T	V	L	H	T	I	M	S	T	V	V	G	T	T	M	S	A	W	E	T
L	T	V	L	H	T	I	M	S	T	V	V	G	T	T	M	S	A	W	E	T
L	T	V	L	H	T	I	M	S	T	V	V	G	T	T	M	S	A	W	E	T
L	T	V	L	H	T	I	M	S	T	V	V	G	T	T	M	S	A	W	E	T
L	T	V	L	H	T	I	M	S	T	V	V	G	T	T	M	S	A	W	E	T
L	T	V	F	H	T	I	M	S	T	V	V	G	T	T	M	S	A	W	E	T
L	T	V	L	H	T	I	M	S	T	V	V	G	T	T	M	S	A	W	E	T
L	T	V	L	H	T	I	M	S	T	V	V	G	T	T	M	S	A	W	E	T
L	T	V	L	H	T	I	M	S	T	V	V	G	T	T	M	S	A	W	E	T
L	T	V	L	H	T	I	M	S	T	V	V	G	T	T	M	S	A	W	E	T
L	T	V	F	H	T	I	M	S	T	V	V	G	T	T	M	S	A	W	E	T
L	T	V	F	H	T	I	M	S	T	V	V	G	T	T	M	S	A	W	E	T
L	T	V	L	H	T	I	M	S	T	V	V	G	T	T	M	S	A	W	E	T
L	T	V	L	H	T	I	M	S	T	V	V	G	T	T	M	S	A	W	E	T
L	T	V	L	H	T	I	M	S	T	V	V	G	T	T	M	S	A	L	E	T
L	T	V	L	H	T	I	M	S	T	V	V	G	T	T	M	S	A	W	E	T
L	T	V	L	H	T	I	M	S	T	V	V	G	T	T	M	S	A	W	E	T
L	T	V	L	H	T	I	M	S	T	V	V	G	T	T	M	S	A	W	E	T
L	T	V	L	H	T	I	M	S	T	V	V	G	T	T	M	S	A	W	E	T
L	T	V	L	H	T	I	M	S	T	V	V	G	T	T	M	S	A	W	E	T
L	T	V	L	H	T	I	M	S	T	V	V	G	T	T	M	S	A	W	E	T
L	T	V	L	H	T	I	M	S	T	V	V	G	T	T	M	S	A	L	E	T
L	T	V	L	H	T	I	M	S	T	V	V	G	T	T	M	S	A	L	E	T
L	T	V	L	H	T	I	M	S	T	V	V	G	T	T	M	S	A	W	E	T
L	T	V	L	H	T	I	M	S	T	V	V	G	T	T	M	S	A	W	E	T
L	T	V	L	H	T	I	M	S	T	V	V	G	T	T	M	S	A	W	E	T
L	T	V	L	H	T	I	M	S	T	V	V	G	T	T	M	S	A	W	E	T
L	T	V	F	H	T	I	M	S	T	V	V	G	T	T	M	S	A	W	E	T
L	T	V	L	H	T	I	M	S	T	V	V	G	T	T	M	S	A	W	E	T
L	T	V	L	H	T	I	M	S	T	V	V	G	T	T	M	S	A	W	E	T
L	T	V	F	H	T	I	M	S	T	V	V	G	T	T	M	S	A	W	E	T
L	T	V	L	H	T	I	M	S	T	V	V	G	T	T	M	S	A	W	E	T
L	T	V	L	H	T	I	M	S	T	V	V	G	T	T	M	S	A	W	E	T
L	T	V	L	H	T	I	M	S	T	V	V	G	T	T	M	S	A	W	E	T
L	T	V	L	H	T	I	M	S	T	V	V	G	T	T	M	S	A	W	E	T
L	T	V	F	H	T	I	M	S	T	V	V	G	T	T	M	S	A	W	E	T
L	T	V	L	H	T	I	M	S	T	V	V	G	T	T	M	S	A	W	E	T
L	T	V	L	H	T	I	M	S	T	V	V	G	T	T	M	S	A	W	E	T
L	T	V	L	H	T	I	M	S	T	V	V	G	T	T	M	S	A	W	E	T
L	T	V	F	H	T	I	M	S	T	V	V	G	T	T	M	S	A	W	E	T
L	T	V	L	H	T	I	M	S	T	V	V	G	T	T	M	S	A	W	E	T
L	T	V	F	H	T	I	M	S	T	V	V	G	T	T	M	S	A	W	E	T
L	T	V	L	H	T	I	M	S	T	V	V	G	T	T	M	S	A	W	E	T
L	T	V	L	H	T	I	M	S	T	V	V	G	T	T	M	S	A	W	E	T
L	T	V	L	H	T	I	M	S	T	V	V	G	T	T	M	S	A	W	E	T
L	T	V	L	H	T	I	M	S	T	V	V	G	T	T	M	S	A	W	E	T
L	T	V	L	H	T	I	M	S	T	V	V	G	T	T	M	S	A	L	E	T
L	T	V	F	H	T	I	M	S	T	V	V	G	T	T	M	S	A	W	E	T
L	T	V	F	H	T	I	M	S	T	V	V	G	T	T	M	S	A	W	E	T
L	T	V	F	H	T	I	M	S	T	V	V	G	T	T	M	S	A	W	E	T
L	T	V	L	H	T	I	M	S	T	V	V	G	T	T	M	S	A	W	E	T
L	T	V	L	H	T	I	M	S	T	V	V	G	T	T	M	S	A	W	E	T
L	T	V	L	H	T	I	M	S	T	V	V	G	T	T	M	S	A	W	E	T
L	T	V	L	H	T	I	M	S	T	V	V	G	T	T	M	S	A	W	E	T
L	T	V	L	H	T	I	M	S	T	V	V	G	T	T	M	S	A	W	E	T
L	T	V	L	H	T	I	M	S	T	V	V	G	T	T	M	S	A	W	E	T
L	T	V	L	H	T	I	M	S	T	V	V	G	T	T	M	S	A	W	E	T

L	T	V	L	H	T	I	M	S	T	V	V	G	T	T	M	S	A	W	E	T
L	T	V	F	H	T	I	M	S	T	V	V	G	T	T	M	S	A	W	E	T
L	T	V	F	H	T	I	M	S	T	V	V	G	T	T	M	S	A	W	E	T
L	T	V	L	H	T	I	M	S	T	V	V	G	T	T	M	S	A	W	E	T
L	T	V	L	H	T	I	M	S	T	V	V	G	T	T	M	S	A	W	E	T
L	T	V	L	H	T	I	M	S	T	V	V	G	T	T	M	S	A	W	E	T
L	T	V	L	H	T	I	M	S	T	V	V	G	T	T	M	S	A	L	E	T
L	T	V	L	H	T	I	M	S	T	V	V	G	T	T	M	S	A	L	E	T
L	T	V	L	H	T	I	M	S	T	V	V	G	T	T	M	S	A	W	E	T
L	T	V	L	H	T	I	M	S	T	V	V	G	T	T	M	S	A	W	E	T
L	T	V	L	H	T	I	M	S	T	V	V	G	T	T	M	S	A	W	E	T
L	T	I	L	H	I	I	I	A	A	V	V	S	T	T	M	S	A	W	E	T
L	T	I	L	H	I	I	I	A	A	V	V	S	T	T	M	S	A	W	E	T
L	T	I	L	H	I	I	I	A	A	V	V	S	T	T	M	S	A	W	E	T
L	T	I	L	H	I	I	I	A	A	V	V	S	T	T	M	S	A	W	E	T
L	T	I	L	H	I	I	I	A	A	V	V	S	T	T	M	S	A	W	E	T
L	T	I	L	H	I	I	I	A	A	V	V	S	T	T	M	S	A	W	E	T
L	T	I	L	H	I	I	I	A	A	V	V	S	T	T	M	S	A	W	E	T
L	T	I	L	H	I	I	I	A	A	V	V	S	T	T	M	S	A	W	E	T
L	T	I	L	H	I	I	I	A	A	V	V	S	T	T	M	S	A	W	E	T
L	A	V	L	Y	I	T	I	A	A	I	I	S	T	T	M	F	A	W	E	M
L	A	V	L	Y	I	T	I	A	A	I	I	S	T	T	M	F	A	W	E	M
L	A	V	L	Y	I	T	I	A	A	I	I	S	T	T	M	F	A	W	E	M
L	A	V	L	Y	I	T	I	A	A	I	I	S	T	T	M	F	A	W	E	M
L	A	V	L	Y	I	T	I	A	A	I	I	S	T	T	M	F	A	W	E	M
L	A	V	L	Y	I	T	I	A	A	I	I	S	T	T	M	F	A	W	E	M
L	A	V	L	Y	I	T	I	A	A	I	I	S	T	T	M	F	A	W	E	M
L	A	V	L	Y	I	T	I	A	A	I	I	S	T	T	M	F	A	W	E	M
L	T	I	L	H	I	I	I	A	A	V	V	S	M	T	M	S	S	W	E	T
L	T	V	L	H	T	I	M	S	T	V	V	G	T	T	M	S	A	W	E	T
L	T	V	L	H	T	I	M	S	T	V	V	G	T	T	M	S	A	W	E	T
L	T	V	L	H	T	I	M	S	T	V	V	G	T	T	M	S	A	W	E	T
L	T	V	L	H	T	I	M	S	T	V	V	G	T	T	M	S	A	W	E	T
L	T	V	L	H	T	I	M	S	T	V	V	G	T	T	M	S	A	W	E	T
L	T	V	L	H	T	I	M	S	T	V	V	G	T	T	M	S	A	W	E	T
L	T	V	L	H	T	I	M	S	T	V	V	G	T	T	M	S	A	W	E	T
L	T	V	L	H	T	I	M	S	T	V	V	G	T	T	M	S	A	W	E	T
L	T	V	L	H	T	I	M	S	T	V	V	G	T	T	M	S	A	W	E	T
L	T	V	L	H	T	I	M	S	T	V	V	G	T	T	M	S	A	W	E	T
L	T	V	L	H	T	I	M	S	T	V	V	G	T	T	M	S	A	W	E	T
L	T	V	L	H	T	I	M	S	T	V	V	G	T	T	M	S	A	W	E	T
L	T	V	L	H	T	I	M	S	T	V	V	G	T	T	M	S	A	W	E	T
L	T	V	L	H	T	I	M	S	T	V	V	G	T	T	M	S	A	W	E	T
L	T	V	L	H	T	I	M	S	T	V	V	G	T	T	M	S	A	W	E	T
L	T	V	L	H	T	I	M	S	T	V	V	G	T	T	M	S	A	W	E	T
L	T	V	L	H	T	I	M	S	T	V	V	G	T	T	M	S	A	W	E	T
L	T	V	L	H	T	I	M	S	T	V	V	G	T	T	M	S	A	W	E	T
L	T	V	L	H	T	I	M	S	T	V	V	G	T	T	M	S	A	W	E	T
L	T	V	L	H	T	I	M	S	T	V	V	G	T	T	M	S	A	W	E	T
L	T	V	L	H	T	I	M	S	T	V	V	G	T	T	M	S	A	W	E	T
L	T	V	L	H	T	I	M	S	T	V	V	G	T	T	M	S	A	W	E	T
L	T	V	L	H	T	I	M	S	T	V	V	G	T	T	M	S	A	W	E	T
L	T	V	L	H	T	I	M	S	T	V	V	G	T	T	M	S	A	W	E	T
L	T	V	L	H	T	I	M	S	T	V	V	G	T	T	M	S	A	W	E	T
L	T	V	L	H	T	I	M	S	T	V	V	G	T	T	M	S	A	W	E	T
L	T	V	L	H	T	I	M	S	T	V	V	G	T	T	M	S	A	W	E	T
L	T	V	L	H	T	I	M	S	T	V	V	G	T	T	M	S	A	W	E	T
L	T	V	L	H	T	I	M	S	T	V	V	G	T	T	M	F	A	W	E	T
L	T	V	L	H	T	I	M	S	T	V	V	G	T	T	M	S	A	W	E	T
L	T	V	L	H	T	I	M	S	T	V	V	G	T	T	M	S	A	W	E	T
L	T	V	L	H	T	I	M	S	T	V	V	G	T	T	M	S	A	W	E	T
L	T	V	L	H	T	I	M	S	T	V	V	G	T	T	M	S	A	W	E	T
L	T	V	L	H	T	I	M	S	T	V	V	G	T	T	M	S	A	W	E	T
L	T	V	L	H	T	I	M	S	T	V	V	G	T	T	M	S	A	W	E	T
L	T	V	L	H	T	I	M	S	T	V	V	G	T	T	M	S	A	W	E	T
L	T	V	L	H	T	I	M	S	T	V	V	G	T	T	M	S	A	W	E	T
L	T	V	L	H	T	I	M	S	T	V	V	G	T	T	M	S	A	W	E	T

L	T	V	L	H	T	I	M	S	T	V	V	G	T	T	M	S	A	W	E	T
L	T	V	L	H	T	I	M	S	T	V	V	G	T	T	M	S	A	W	E	T
L	T	V	L	H	T	I	M	S	T	V	V	G	T	T	M	S	A	W	E	T
L	T	V	L	H	T	I	M	S	T	V	V	G	T	T	M	S	A	W	E	T
L	T	V	L	H	T	I	M	S	T	V	V	G	T	T	M	S	A	W	E	T
L	T	V	L	H	T	I	M	S	T	V	V	G	T	T	M	S	A	W	E	T
L	T	V	L	H	T	I	M	S	T	V	V	G	T	T	M	S	A	W	E	T
L	T	V	L	H	T	I	M	S	T	V	V	G	T	T	M	S	A	W	E	T
L	T	V	L	H	T	I	M	S	T	V	V	G	T	T	M	S	A	W	E	T
L	T	V	L	H	T	I	M	S	T	V	V	G	T	T	M	S	A	W	E	T
L	T	V	L	H	T	I	M	S	T	V	V	G	T	T	M	S	A	W	E	T
L	T	V	L	H	T	I	M	S	T	V	V	G	T	T	M	S	A	W	E	T
L	T	V	L	H	T	I	M	S	T	V	V	G	T	T	M	S	A	W	E	T
L	T	V	L	H	T	I	M	S	T	V	V	G	T	T	M	S	A	W	E	T
L	T	V	L	H	T	I	M	S	T	V	V	G	T	T	M	S	A	W	E	T
L	T	V	L	H	T	I	M	S	T	V	V	G	T	T	M	S	A	W	E	T
L	T	V	L	H	T	I	M	S	T	V	V	G	T	T	M	S	A	W	E	T
L	T	V	L	H	T	I	M	S	T	V	V	G	T	T	M	S	A	W	E	T
L	T	V	L	H	T	I	M	S	T	V	V	G	T	T	M	S	A	W	E	T
L	T	V	L	H	T	I	M	S	T	V	V	G	T	T	M	S	A	W	E	T
L	T	V	L	H	T	I	M	S	T	V	V	G	T	T	M	S	A	W	E	T
L	T	V	L	H	T	I	M	S	T	V	V	G	T	T	M	S	A	W	E	T
L	T	V	L	H	T	I	M	S	T	V	V	G	T	T	M	S	A	W	E	T
L	T	I	L	H	I	I	I	A	A	V	V	S	M	T	M	S	S	W	E	M
L	T	I	L	H	I	I	I	A	A	V	V	S	T	T	M	S	A	W	E	T
L	T	I	L	H	I	I	I	A	A	V	V	S	T	T	M	S	A	W	E	T
L	T	I	L	H	I	I	I	A	A	V	V	S	T	T	M	S	A	W	E	T
L	T	I	L	H	I	I	I	A	A	V	V	S	T	T	M	S	A	W	E	T
L	T	I	L	H	I	I	I	A	A	V	V	S	T	T	M	S	A	W	E	T
L	T	I	L	H	I	I	I	A	A	V	V	S	T	T	M	S	A	W	E	T
L	T	I	L	H	I	I	I	A	A	V	V	S	T	T	M	S	A	W	E	T
L	T	I	L	H	I	I	I	A	A	V	V	S	T	T	M	S	A	W	E	T
L	T	I	L	H	I	I	I	A	A	V	V	S	T	T	M	S	A	W	E	T
L	T	I	L	H	I	I	I	A	A	V	V	S	T	T	M	S	A	W	E	T
L	T	I	L	H	I	I	I	A	A	V	V	S	T	T	M	S	A	W	E	T
L	T	I	L	H	I	I	I	A	A	V	V	S	T	T	M	S	A	W	E	T
L	T	I	L	H	I	I	I	A	A	V	V	S	T	T	M	S	A	W	E	T
L	T	I	L	H	I	I	I	A	A	V	V	S	T	T	M	S	A	W	E	T
L	T	I	L	H	I	I	I	A	A	V	V	S	T	T	M	S	A	W	E	T
L	T	I	L	H	I	I	I	A	A	V	V	S	T	T	M	S	A	W	E	T
L	T	I	L	H	I	I	I	A	A	V	V	S	T	T	M	S	A	W	E	T
L	T	I	L	H	I	I	I	A	A	V	V	S	T	T	M	S	A	W	E	T
L	T	I	L	H	I	I	I	A	A	V	V	S	T	T	M	S	A	W	E	T
L	T	I	L	H	I	I	I	A	A	V	V	S	T	T	M	S	A	W	E	T
L	T	I	L	H	I	I	I	A	A	V	V	S	T	T	M	S	A	W	E	T
L	T	I	L	H	I	I	I	A	A	V	V	S	T	T	M	S	A	W	E	T
L	T	I	L	H	I	I	I	A	A	V	V	S	T	T	M	S	A	W	E	T
L	T	I	L	H	I	I	I	A	A	V	V	S	T	T	M	S	A	W	E	T
L	T	I	L	H	I	I	I	A	A	V	V	S	M	T	M	S	S	W	E	M
L	T	I	L	H	I	I	I	A	A	V	V	S	M	T	M	S	S	W	E	M
L	T	I	L	H	I	I	I	A	A	V	V	S	M	T	M	S	S	W	E	M
L	T	I	L	H	I	I	I	A	A	V	V	S	M	T	M	S	S	W	E	M
L	T	I	L	H	I	I	I	A	A	V	V	S	M	T	M	S	S	W	E	M
L	T	I	L	H	I	I	I	A	A	V	V	S	M	T	M	S	S	W	E	M
L	T	I	L	H	I	I	I	A	A	V	V	S	M	T	M	S	S	W	E	M
L	T	I	L	H	I	I	I	A	A	V	V	S	M	T	M	S	S	W	E	M
L	T	I	L	H	I	I	I	A	A	V	V	S	M	T	M	S	S	W	E	M
L	T	I	L	H	I	I	I	A	A	V	V	S	M	T	M	S	S	W	E	M
L	T	I	L	H	I	I	I	A	A	V	V	S	M	T	M	S	S	W	E	M
L	T	I	L	H	I	I	I	A	A	V	V	S	M	T	M	S	S	W	E	M
L	T	I	L	H	I	I	I	A	A	V	V	S	M	T	M	S	S	W	E	M
L	T	I	L	H	I	I	I	A	A	V	V	S	M	T	M	S	S	W	E	M
L	T	I	L	H	I	I	I	A	A	V	V	S	M	T	M	S	S	W	E	M
L	T	I	L	H	I	I	I	A	A	V	V	S	M	T	M	S	S	W	E	M
L	T	I	L	H	I	I	I	A	A	V	V	S	M	T	M	S	S	W	E	M
L	T	I	L	H	I	I	I	A	A	V	V	S	M	T	M	S	S	W	E	M

L	T
L	T
L	T
L	T
L	T
L	T
L	T
L	T
L	T
L	T

I	L	H	I	I	I	A	A	V	V	S	M
I	L	H	I	I	I	A	A	V	V	S	M
I	L	H	I	I	I	A	A	V	V	S	M
I	L	H	I	I	I	A	A	V	V	S	M
I	L	H	I	I	I	A	A	V	V	S	M
I	L	H	I	I	I	A	A	V	V	S	M
I	L	H	I	I	I	A	A	V	V	S	M
I	L	H	I	I	I	A	A	V	V	S	M
I	L	H	I	I	I	A	A	V	V	S	M
I	L	H	I	I	I	A	A	V	V	S	M

T	M	S	S	W	E	M
I	M	S	S	W	E	M
T	M	S	S	W	E	M
T	M	S	S	W	E	M
T	M	S	S	W	E	M
T	M	S	S	W	E	M
T	M	S	S	W	E	M
T	M	S	S	W	E	M
T	M	S	S	W	E	M
T	M	S	S	W	E	M

403	444
G	T
S	R
S	T
S	T
S	T
S	T
S	T
S	T
S	T
S	T
S	T

wzx																			
14	22	89	106	110	157	158	254	305	312	321	328	335	381	387	398	410	413	414	420
F	V	F	I	P	I	F	T	F	I	R	A	I	N	I	I	I	E	Q	L
L	L	S	T	Q	V	I	T	I	V	Q	T	V	S	V	V	V	K	K	F
F	V	F	I	P	I	F	M	F	I	R	A	I	N	I	I	I	E	Q	L
F	V	F	I	P	I	F	M	F	I	Q	T	V	S	V	I	V	K	K	F
F	V	F	I	P	I	F	M	F	I	Q	T	V	S	V	I	V	K	K	F
F	V	F	I	P	I	F	M	F	I	Q	T	V	S	V	I	V	K	K	F
F	V	F	I	P	I	F	M	F	I	Q	T	V	S	V	I	V	K	K	F
F	V	F	I	P	I	F	M	F	I	Q	T	V	S	V	I	V	K	K	F
F	V	F	I	P	I	F	M	F	I	Q	T	V	S	V	I	V	K	K	F
F	V	F	I	P	I	F	M	F	I	Q	T	V	S	V	I	V	K	K	F
F	V	F	I	P	I	F	M	F	I	Q	T	V	S	V	I	V	K	K	F

wzx																				
110	112	157	158	305	312	321	328	335	370	381	387	398	410	413	144	420	422	425	437	457
P	A	I	F	F	I	R	A	I	L	N	I	I	I	E	Q	L	M	I	L	T
Q	A	V	I	I	V	Q	T	V	L	S	V	V	V	K	K	F	L	V	P	I
P	A	I	F	F	I	R	A	I	L	N	I	I	I	E	Q	L	M	I	L	T
P	A	I	F	F	I	R	A	I	L	N	I	I	I	E	Q	L	M	I	L	T
P	A	I	F	F	I	R	A	I	L	N	I	I	I	E	Q	L	M	I	L	T
P	A	I	F	F	I	R	A	I	L	N	I	I	I	E	Q	L	M	I	L	T
P	A	I	F	F	I	R	A	I	L	N	I	I	I	E	Q	L	M	I	L	T
P	A	I	F	F	I	R	A	I	L	N	I	I	I	E	Q	L	M	I	L	T
P	A	I	F	F	I	R	A	I	L	N	I	I	I	E	Q	L	M	I	L	T
P	A	I	F	F	I	R	A	I	L	N	I	I	I	E	Q	L	M	I	L	T
P	A	I	F	F	I	R	A	I	L	N	I	I	I	E	Q	L	M	I	L	T
P	A	I	F	F	I	R	A	I	L	N	I	I	I	E	Q	L	M	I	L	T
P	A	I	F	F	I	R	A	I	L	N	I	I	I	E	Q	L	M	I	L	T
P	A	I	F	F	I	R	A	I	L	N	I	I	I	E	Q	L	M	I	L	T
P	A	I	F	F	I	R	A	I	L	N	I	I	I	E	Q	L	M	I	L	T
P	A	I	F	F	I	R	A	I	L	N	I	I	I	E	Q	L	M	I	L	T
P	A	I	F	F	I	R	A	I	L	N	I	I	I	E	Q	L	M	I	L	T
P	A	I	F	F	I	R	A	I	L	N	I	I	I	E	Q	L	M	I	L	T
P	A	I	F	F	I	R	A	I	L	N	I	I	I	E	Q	L	M	I	L	T
P	A	I	F	F	I	R	A	I	L	N	I	I	I	E	Q	L	M	I	L	T
P	A	I	F	F	I	R	A	I	L	N	I	I	I	E	Q	L	M	I	L	T
P	A	I	F	F	I	R	A	I	F	N	I	I	I	E	Q	L	M	I	L	T
P	A	I	F	F	I	R	A	I	F	N	I	I	I	E	Q	L	M	I	L	T
P	A	I	F	F	I	R	A	I	F	N	I	I	I	E	Q	L	M	I	L	T
P	A	I	F	F	I	R	A	I	F	N	I	I	I	E	Q	L	M	I	L	T
P	S	I	F	F	I	R	A	I	L	N	I	I	I	E	Q	L	M	I	L	T
P	A	I	F	F	I	R	A	I	F	N	I	I	I	E	Q	L	M	I	L	T
P	A	I	F	F	I	R	A	I	F	N	I	I	I	E	Q	L	M	I	L	T
P	A	I	F	F	I	R	A	I	F	N	I	I	I	E	Q	L	M	I	L	T
P	A	I	F	F	I	R	A	I	F	N	I	I	I	E	Q	L	M	I	L	T
P	A	I	F	F	I	R	A	I	F	N	I	I	I	E	Q	L	M	I	L	T

47
R
K
R
R
R
R
R
R
R
R
R
R
R
R
R
R
R
R
R
R
R
R
R
R
R
R
R
R
R
R
R

158	305	312	321	328	335	381	387	392	410	413	414	420	422	425	437	457
F	F	I	R	A	I	N	I	I	I	E	Q	L	M	I	L	T
I	I	V	Q	T	V	S	V	V	V	K	K	F	L	V	P	I
F	F	I	R	A	I	N	I	I	I	E	Q	L	M	I	L	T
F	F	I	R	A	I	N	I	I	I	E	Q	L	M	I	L	T
F	F	I	R	A	I	N	I	I	I	E	Q	L	M	I	L	T
F	F	I	R	A	I	N	I	I	I	E	Q	L	M	I	L	T
F	F	I	R	A	I	N	I	I	I	E	Q	L	M	I	L	T
F	F	I	R	A	I	N	I	I	I	E	Q	L	M	I	L	T
F	F	I	R	A	I	N	I	I	I	E	Q	L	M	I	L	T
F	F	I	R	A	I	N	I	I	I	E	Q	L	M	I	L	T
F	F	I	R	A	I	N	I	I	I	E	Q	L	M	I	L	T
F	F	I	R	A	I	N	I	I	I	E	Q	L	M	I	L	T
F	F	I	R	A	I	N	I	I	I	E	Q	L	M	I	L	T
F	F	I	R	A	I	N	I	I	I	E	Q	L	M	I	L	T
F	F	I	R	A	I	N	I	I	I	E	Q	L	M	I	L	T
F	F	I	R	A	I	N	I	I	I	E	Q	L	M	I	L	T
F	F	I	R	A	I	N	I	I	I	E	Q	L	M	I	L	T
F	F	I	R	A	I	N	I	I	I	E	Q	L	M	I	L	T
F	F	I	R	A	I	N	I	I	I	E	Q	L	M	I	L	T
F	F	I	R	A	I	N	I	I	I	E	Q	L	M	I	L	T
F	F	I	R	A	I	N	I	I	I	E	Q	L	M	I	L	T
F	F	I	R	A	I	N	I	I	I	E	Q	L	M	I	L	T
F	F	I	R	A	I	N	I	I	I	E	Q	L	M	I	L	T
F	F	I	R	A	I	N	I	I	I	E	Q	L	M	I	L	T
F	F	I	R	A	I	N	I	I	I	E	Q	L	M	I	L	T
F	F	I	R	A	I	N	I	I	I	E	Q	L	M	I	L	T
F	F	I	R	A	I	N	I	I	I	E	Q	L	M	I	L	T

20	47	63	66	91
A	R	E	Q	I
A	K	D	L	L
T	R	E	Q	I
A	R	E	Q	I
A	R	E	Q	I
T	R	E	Q	I
T	R	E	Q	I
T	R	E	Q	I
T	R	E	Q	I
A	R	E	Q	I
A	R	E	Q	I
A	R	E	Q	I
T	R	E	Q	I
T	R	E	Q	I
T	R	E	Q	I
T	R	E	Q	I
T	R	E	Q	I
T	R	E	Q	I
T	R	E	Q	I
T	R	E	Q	I
T	R	E	Q	I
T	R	E	Q	I
T	R	E	Q	I
A	R	E	Q	I
A	R	E	Q	I
A	R	E	Q	I
A	R	E	Q	I

214	268	272	286
L	A	S	V
L	A	P	I
V	T	P	I
V	T	P	I
V	T	P	I
V	T	P	I
V	T	P	I
V	T	P	I
V	T	P	I
V	T	P	I
V	T	P	I
V	T	P	I
V	T	P	I
V	T	P	I
V	T	P	I
V	T	P	I
V	T	P	I
V	T	P	I
V	T	P	I
V	T	P	I
V	T	P	I
V	T	P	I
V	T	P	I
V	T	P	I
V	T	P	I
V	T	P	I
V	T	P	I
V	T	P	I
V	T	P	I

rmlC
182
N
N
H
N
N
N
N
N
N
N
N
N
N
N
N
N
N
N
N
N
N
N
N
N
N
N
N
N

rmlB				
30	275	300	338	343
D	V	E	A	T
D	A	D	A	T
G	T	D	A	T
D	A	D	S	T
D	A	D	S	T
D	A	D	S	T
D	A	D	S	T
D	A	D	S	T
D	A	D	S	T
D	A	D	S	T
D	A	D	S	T
D	A	D	S	T
D	A	D	S	T
D	A	D	S	T
D	A	D	S	T
D	A	D	S	T
D	A	D	S	T
D	A	D	S	T
D	A	D	S	T
D	A	D	S	T
D	A	D	S	T
D	A	D	S	T
D	A	D	S	T
D	A	D	S	T
D	A	D	S	T
D	A	D	S	T
D	A	D	S	T
D	A	D	S	T
D	A	D	S	T

rmlD	
33	245
E	V
K	A
E	A
K	A
K	A
K	A
K	A
K	A
K	A
K	A
K	A
K	A
K	A
K	A
K	A
K	A
K	A
K	A
K	A
K	A
K	A
K	A
K	A
K	A
K	A
K	A
K	A
K	A
K	A

V	T	P	I	N	D	A	D	S	I	K	A
V	T	P	I	N	D	A	D	S	T	K	A

wzx																					
281	321	328	335	370	381	387	410	413	414	420	422	425	437	457	20	47	63	66	91	95	114
I	R	A	I	L	N	I	I	E	Q	L	M	I	L	T	A	R	E	Q	I	E	P
I	R	A	I	L	N	I	I	E	Q	L	M	I	L	T	A	R	E	Q	I	E	P
I	R	A	I	L	N	I	I	E	Q	L	M	I	L	T	A	R	E	Q	I	E	P
I	R	A	I	L	N	I	I	E	Q	L	M	I	L	T	A	R	E	Q	I	E	P
I	R	A	I	L	N	I	I	E	Q	L	M	I	L	T	A	R	E	Q	I	E	P
I	R	A	I	L	N	I	I	E	Q	L	M	I	L	T	A	R	E	Q	I	E	P
I	R	A	I	L	N	I	I	E	Q	L	M	I	L	T	A	R	E	Q	I	E	P
I	R	A	I	L	N	I	I	E	Q	L	M	I	L	T	A	R	E	Q	I	E	P
I	R	A	I	L	N	I	I	E	Q	L	M	I	L	T	A	R	E	Q	I	E	P
I	R	A	I	L	N	I	I	E	Q	L	M	I	L	T	A	R	E	Q	I	E	P
I	R	A	I	L	N	I	I	E	Q	L	M	I	L	T	A	R	E	Q	I	E	P
I	R	A	I	L	N	I	I	E	Q	L	M	I	L	T	A	R	E	Q	I	E	P
I	R	A	I	L	N	I	I	E	Q	L	M	I	L	T	A	R	E	Q	I	E	P
I	R	A	I	L	N	I	I	E	Q	L	M	I	L	T	A	R	E	Q	I	E	P
I	R	A	I	L	N	I	I	E	Q	L	M	I	L	T	A	R	E	Q	I	E	P
I	R	A	I	L	N	I	I	E	Q	L	M	I	L	T	A	R	E	Q	I	E	P
I	R	A	I	L	N	I	I	E	Q	L	M	I	L	T	A	R	E	Q	I	E	P
I	R	A	I	L	N	I	I	E	Q	L	M	I	L	T	A	R	E	Q	I	E	P
I	R	A	I	L	N	I	I	E	Q	L	M	I	L	T	A	R	E	Q	I	E	P
I	R	A	I	L	N	I	I	E	Q	L	M	I	L	T	A	R	E	Q	I	E	P
I	R	A	I	L	N	I	I	E	Q	L	M	I	L	T	A	R	E	Q	I	E	P
I	R	A	I	L	N	I	I	E	Q	L	M	I	L	T	A	R	E	Q	I	E	P
I	R	A	I	L	N	I	I	E	Q	L	M	I	L	T	A	R	E	Q	I	E	P
I	R	A	I	L	N	I	I	E	Q	L	M	I	L	T	A	R	E	Q	I	E	P
I	R	A	I	L	N	I	I	E	Q	L	M	I	L	T	A	R	E	Q	I	E	P
I	R	A	I	L	N	I	I	E	Q	L	M	I	L	T	A	R	E	Q	I	E	P
I	R	A	I	L	N	I	I	E	Q	L	M	I	L	T	A	R	E	Q	I	E	P
I	R	A	I	L	N	I	I	E	Q	L	M	I	L	T	A	R	E	Q	I	E	P
I	R	A	I	L	N	I	I	E	Q	L	M	I	L	T	A	R	E	Q	I	E	P
I	R	A	I	L	N	I	I	E	Q	L	M	I	L	T	A	R	E	Q	I	E	P
I	R	A	I	L	N	I	I	E	Q	L	M	I	L	T	A	R	E	Q	I	E	P
I	R	A	I	L	N	I	I	E	Q	L	M	I	L	T	A	R	E	Q	I	E	P
I	R	A	I	L	N	I	I	E	Q	L	M	I	L	T	A	R	E	Q	I	E	P
I	R	A	I	L	N	I	I	E	Q	L	M	I	L	T	A	R	E	Q	I	E	P
I	R	A	I	L	N	I	I	E	Q	L	M	I	L	T	A	R	E	Q	I	E	P
I	R	A	I	L	N	I	I	E	Q	L	M	I	L	T	A	R	E	Q	I	E	P
I	R	A	I	L	N	I	I	E	Q	L	M	I	L	T	A	R	E	Q	I	E	P
I	R	A	I	L	N	I	I	E	Q	L	M	I	L	T	A	R	E	Q	I	E	P
I	R	A	I	L	N	I	I	E	Q	L	M	I	L	T	A	R	E	Q	I	E	P
I	R	A	I	L	N	I	I	E	Q	L	M	I	L	T	A	R	E	Q	I	E	P
I	R	A	I	L	N	I	I	E	Q	L	M	I	L	T	A	R	E	Q	I	E	P
I	R	A	I	L	N	I	I	E	Q	L	M	I	L	T	A	R	E	Q	I	E	P
I	R	A	I	L	N	I	I	E	Q	L	M	I	L	T	A	R	E	Q	I	E	P
I	R	A	I	L	N	I	I	E	Q	L	M	I	L	T	A	R	E	Q	I	E	P
I	R	A	I	L	N	I	I	E	Q	L	M	I	L	T	A	R	E	Q	I	E	P
I	R	A	I	L	N	I	I	E	Q	L	M	I	L	T	A	R	E	Q	I	E	P
I	R	A	I	L	N	I	I	E	Q	L	M	I	L	T	A	R	E	Q	I	E	P
I	R	A	I	L	N	I	I	E	Q	L	M	I	L	T	A	R	E	Q	I	E	P
I	R	A	I	L	N	I	I	E	Q	L	M	I	L	T	A	R	E	Q	I	E	P
I	R	A	I	L	N	I	I	E	Q	L	M	I	L	T	A	R	E	Q	I	E	P
I	R	A	I	L	N	I	I	E	Q	L	M	I	L	T	A	R	E	Q	I	E	P
I	R	A	I	L	N	I	I	E	Q	L	M	I	L	T	A	R	E	Q	I	E	P
I	R	A	I	L	N	I	I	E	Q	L	M	I	L	T	A	R	E	Q	I	E	P
I	R	A	I	F	N	I	I	E	Q	L	M	I	L	T	A	R	E	Q	I	E	P
I	R	A	I	L	N	I	I	E	Q	L	M	I	L	T	A	R	E	Q	I	E	P
I	R	A	I	L	N	I	I	E	Q	L	M	I	L	T	A	R	E	Q	I	E	P
I	R	A	I	L	N	I	I	E	Q	L	M	I	L	T	A	R	E	Q	I	E	P
I	R	A	I	L	N	I	I	E	Q	L	M	I	L	T	A	R	E	Q	I	E	P
I	R	A	I	L	N	I	I	E	Q	L	M	I	L	T	A	R	E	Q	I	E	P
I	R	A	I	F	N	I	I	E	Q	L	M	I	L	T	A	R	E	Q	I	E	P
I	R	A	I	F	N	I	I	E	Q	L	M	I	L	T	A	R	E	Q	I	E	P
I	R	A	I	F	N	I	I	E	Q	L	M	I	L	T	A	R	E	Q	I	E	P
I	R	A	I	F	N	I	I	E	Q	L	M	I	L	T	A	R	E	Q	I	E	P

I	R	A	I	F	N	I	I	E	Q	L	M	I	L	T	A	R	E	Q	I	E	P
I	R	A	I	F	N	I	I	E	Q	L	M	I	L	T	A	R	E	Q	I	E	P
I	R	A	I	F	N	I	I	E	Q	L	M	I	L	T	A	R	E	Q	I	E	P
I	R	A	I	F	N	I	I	E	Q	L	M	I	L	T	A	R	E	Q	I	E	P
I	R	A	I	F	N	I	I	E	Q	L	M	I	L	T	A	R	E	Q	I	E	P
I	R	A	I	F	N	I	I	E	Q	L	M	I	L	T	A	R	E	Q	I	E	P
I	R	A	I	F	N	I	I	E	Q	L	M	I	L	T	A	R	E	Q	I	E	P
I	R	A	I	F	N	I	I	E	Q	L	M	I	L	T	A	R	E	Q	I	E	P
I	R	A	I	F	N	I	I	E	Q	L	M	I	L	T	A	R	E	Q	I	E	P
I	R	A	I	F	N	I	I	E	Q	L	M	I	L	T	A	R	E	Q	I	E	P
I	R	A	I	F	N	I	I	E	Q	L	M	I	L	T	A	R	E	Q	I	E	P
I	R	A	I	F	N	I	I	E	Q	L	M	I	L	T	A	R	E	Q	I	E	P
I	R	A	I	F	N	I	I	E	Q	L	M	I	L	T	A	R	E	Q	I	E	P
I	R	A	I	F	N	I	I	E	Q	L	M	I	L	T	A	R	E	Q	I	E	P
I	R	A	I	F	N	I	I	E	Q	L	M	I	L	T	A	R	E	Q	I	E	P
I	R	A	I	F	N	I	I	E	Q	L	M	I	L	T	A	R	E	Q	I	E	P
I	R	A	I	F	N	I	I	E	Q	L	M	I	L	T	A	R	E	Q	I	E	P
I	R	A	I	F	N	I	I	E	Q	L	M	I	L	T	A	R	E	Q	I	E	P
I	R	A	I	F	N	I	I	E	Q	L	M	I	L	T	A	R	E	Q	I	E	P
I	R	A	I	F	N	I	I	E	Q	L	M	I	L	T	A	R	E	Q	I	E	P
I	R	A	I	F	N	I	I	E	Q	L	M	I	L	T	A	R	E	Q	I	E	P
I	R	A	I	L	N	I	I	E	Q	L	M	I	L	T	A	R	E	Q	I	E	P
I	R	A	I	L	N	I	I	E	Q	L	M	I	L	T	A	R	E	Q	I	E	P
I	R	A	I	L	N	I	I	E	Q	L	M	I	L	T	A	R	E	Q	I	E	S
I	R	A	I	L	N	I	I	E	Q	L	M	I	L	T	A	R	E	Q	I	E	P
I	R	A	I	L	N	I	I	E	Q	L	M	I	L	T	A	R	E	Q	I	E	P
I	R	A	I	L	N	I	I	E	Q	L	M	I	L	T	A	R	E	Q	I	E	P
I	R	A	I	L	N	I	I	E	Q	L	M	I	L	T	A	R	E	Q	I	E	P
I	R	A	I	L	N	I	I	E	Q	L	M	I	L	T	A	R	E	Q	I	E	P
I	R	A	I	L	N	I	I	E	Q	L	M	I	L	T	A	R	E	Q	I	E	P
I	R	A	I	L	N	I	I	E	Q	L	M	I	L	T	A	R	E	Q	I	E	P
I	R	A	I	L	N	I	I	E	Q	L	M	I	L	T	A	R	E	Q	I	E	P
I	R	A	I	L	N	I	I	E	Q	L	M	I	L	T	A	R	E	Q	I	E	P
I	R	A	I	L	N	I	I	E	Q	L	M	I	L	T	A	R	E	Q	I	E	P
I	R	A	I	L	N	I	I	E	Q	L	M	I	L	T	A	R	E	Q	I	E	P
I	R	A	I	L	N	I	I	E	Q	L	M	I	L	T	A	R	E	Q	I	E	P
I	R	A	I	L	N	I	I	E	Q	L	M	I	L	T	A	R	E	Q	I	E	P
I	R	A	I	L	N	I	I	E	Q	L	M	I	L	T	A	R	E	Q	I	E	P
I	R	A	I	L	N	I	I	E	Q	L	M	I	L	T	A	R	E	Q	I	E	P
I	R	A	I	L	N	I	I	E	Q	L	M	I	L	T	A	R	E	Q	I	E	P
I	R	A	I	L	N	I	I	E	Q	L	M	I	L	T	A	R	E	Q	I	E	P
I	R	A	I	L	N	I	I	E	Q	L	M	I	L	T	A	R	E	Q	I	E	P
I	R	A	I	L	N	I	I	E	Q	L	M	I	L	T	A	R	E	Q	I	E	P
I	R	A	I	L	N	I	I	E	Q	L	M	I	L	T	A	R	E	Q	I	E	P
I	R	A	I	L	N	I	I	E	Q	L	M	I	L	T	A	R	E	Q	I	E	P
I	R	A	I	L	N	I	I	E	Q	L	M	I	L	T	A	R	E	Q	I	E	P
I	R	A	I	L	N	I	I	E	Q	L	M	I	L	T	A	R	E	Q	I	E	P
I	R	A	I	L	N	I	I	E	Q	L	M	I	L	T	A	R	E	Q	I	E	P
I	R	A	I	L	N	I	I	E	Q	L	M	I	L	T	A	R	E	Q	I	E	P
I	R	A	I	L	N	I	I	E	Q	L	M	I	L	T	A	R	E	Q	I	E	P
I	R	A	I	L	N	I	I	E	Q	L	M	I	L	T	A	R	E	Q	I	E	P
I	R	A	I	L	N	I	I	E	Q	L	M	I	L	T	A	R	E	Q	I	E	P
I	R	A	I	L	N	I	I	E	Q	L	M	I	L	T	A	R	E	Q	I	E	P
I	R	A	I	L	N	I	I	E	Q	L	M	I	L	T	A	R	E	Q	I	E	P
I	R	A	I	L	N	I	I	E	Q	L	M	I	L	T	A	R	E	Q	I	E	P
I	R	A	I	L	N	I	I	E	Q	L	M	I	L	T	A	R	E	Q	I	E	P
I	R	A	I	L	N	I	I	E	Q	L	M	I	L	T	A	R	E	Q	I	E	P
I	R	A	I	L	N	I	I	E	Q	L	M	I	L	T	A	R	E	Q	I	E	P
I	R	A	I	L	N	I	I	E	Q	L	M	I	L	T	A	R	E	Q	I	E	P
I	R	A	I	L	N	I	I	E	Q	L	M	I	L	T	A	R	E	Q	I	E	P
I	R	A	I	L	N	I	I	E	Q	L	M	I	L	T	A	R	E	Q	I	E	P
I	R	A	I	L	N	I	I	E	Q	L	M	I	L	T	A	R	E	Q	I	E	P
I	R	A	I	L	N	I	I	E	Q	L	M	I	L	T	A	R	E	Q	I	E	P
I	R	A	I	L	N	I	I	E	Q	L	M	I	L	T	A	R	E	Q	I	E	P
I	R	A	I	L	N	I	I	E	Q	L	M	I	L	T	A	R	E	Q	I	E	P
I	R	A	I	L	N	I	I	E	Q	L	M	I	L	T	A	R	E	Q	I	E	P
I	R	A	I	L	N	I	I	E	Q	L	M	I	L	T	A	R	E	Q	I	E	P

I	R	A	I	L	N	I	I	E	Q	L	M	I	L	T	A	R	E	Q	I	E	P
I	R	A	I	L	N	I	I	E	Q	L	M	I	L	T	A	R	E	Q	I	E	P
I	R	A	I	L	N	I	I	E	Q	L	M	I	L	T	A	R	E	Q	I	E	P
I	R	A	I	L	N	I	I	E	Q	L	M	I	L	T	A	R	E	Q	I	E	P
I	R	A	I	L	N	I	I	E	Q	L	M	I	L	T	A	R	E	Q	I	E	P
I	R	A	I	L	N	I	I	E	Q	L	M	I	L	T	A	R	E	Q	I	E	P
I	R	A	I	L	N	I	I	E	Q	L	M	I	L	T	A	R	E	Q	I	E	P
I	R	A	I	L	N	I	I	E	Q	L	M	I	L	T	A	R	E	Q	I	E	P
I	R	A	I	L	N	I	I	E	Q	L	M	I	L	T	A	R	E	Q	I	E	P
I	R	A	I	L	N	I	I	E	Q	L	M	I	L	T	A	R	E	Q	I	E	P
I	R	A	I	L	N	I	I	E	Q	L	M	I	L	T	A	R	E	Q	I	E	P
I	R	A	I	L	N	I	I	E	Q	L	M	I	L	T	A	R	E	Q	I	E	P
I	R	A	I	L	N	I	I	E	Q	L	M	I	L	T	A	R	E	Q	I	E	P
I	R	A	I	L	N	I	I	E	Q	L	M	I	L	T	A	R	E	Q	I	E	P
I	R	A	I	L	N	I	I	E	Q	L	M	I	L	T	A	R	E	Q	I	E	P
I	R	A	I	L	N	I	I	E	Q	L	M	I	L	T	A	R	E	Q	I	E	P
I	R	A	I	L	N	I	I	E	Q	L	M	I	L	T	A	R	E	Q	I	E	P
I	R	A	I	L	N	I	I	E	Q	L	M	I	L	T	A	R	E	Q	I	E	P
I	R	A	I	L	N	I	I	E	Q	L	M	I	L	T	A	R	E	Q	I	E	P
I	R	A	I	L	N	I	I	E	Q	L	M	I	L	T	A	R	E	Q	I	E	P
I	R	A	I	L	N	I	I	E	Q	L	M	I	L	T	A	R	E	Q	I	E	P
I	R	A	I	L	N	I	I	E	Q	L	M	I	L	T	A	R	E	Q	I	E	P
I	R	A	I	L	N	I	I	E	Q	L	M	I	L	T	A	R	E	Q	I	E	P
I	R	A	I	L	N	I	I	E	Q	L	M	I	L	T	A	R	E	Q	I	E	P
I	R	A	I	L	N	I	I	E	Q	L	M	I	L	T	A	R	E	Q	I	E	P
I	R	A	I	L	N	I	I	E	Q	L	M	I	L	T	A	R	E	Q	I	E	P
I	R	A	I	L	N	I	I	E	Q	L	M	I	L	T	A	R	E	Q	I	E	P
I	R	A	I	L	N	I	I	E	Q	L	M	I	L	T	A	R	E	Q	I	E	P
I	R	A	I	L	N	I	I	E	Q	L	M	I	L	T	A	R	E	Q	I	E	P
I	R	A	I	L	N	I	I	E	Q	L	M	I	L	T	A	R	E	Q	I	E	P
I	R	A	I	L	N	I	I	E	Q	L	M	I	L	T	A	R	E	Q	I	E	P
I	R	A	I	L	N	I	I	E	Q	L	M	I	L	T	A	R	E	Q	I	E	P
I	R	A	I	L	N	I	I	E	Q	L	M	I	L	T	A	R	E	Q	I	E	P
I	R	A	I	L	N	I	I	E	Q	L	M	I	L	T	A	R	E	Q	I	E	P
I	R	A	I	L	N	I	I	E	Q	L	M	I	L	T	A	R	E	Q	I	E	P
I	R	A	I	L	N	I	I	E	Q	L	M	I	L	T	A	R	E	Q	I	E	P
I	R	A	I	L	N	I	I	E	Q	L	M	I	L	T	A	R	E	Q	I	E	P
I	R	A	I	L	N	I	I	E	Q	L	M	I	L	T	A	R	E	Q	I	E	P
I	R	A	I	L	N	I	I	E	Q	L	M	I	L	T	A	R	E	Q	I	E	P
I	R	A	I	L	N	I	I	E	Q	L	M	I	L	T	A	R	E	Q	I	E	P
I	R	A	I	L	N	I	I	E	Q	L	M	I	L	T	A	R	E	Q	I	E	P
I	R	A	I	L	N	I	I	E	Q	L	M	I	L	T	A	R	E	Q	I	E	P
I	R	A	I	L	N	I	I	E	Q	L	M	I	L	T	A	R	E	Q	I	E	P
I	R	A	I	L	N	I	I	E	Q	L	M	I	L	T	A	R	E	Q	I	E	P
I	R	A	I	L	N	I	I	E	Q	L	M	I	L	T	A	R	E	Q	I	E	P
I	R	A	I	L	N	I	I	E	Q	L	M	I	L	T	A	R	E	Q	I	E	P
I	R	A	I	L	N	I	I	E	Q	L	M	I	L	T	A	R	E	Q	I	E	P
I	R	A	I	L	N	I	I	E	Q	L	M	I	L	T	A	R	E	Q	I	E	P
I	R	A	I	L	N	I	I	E	Q	L	M	I	L	T	A	R	E	Q	I	E	P
I	R	A	I	L	N	I	I	E	Q	L	M	I	L	T	A	R	E	Q	I	E	P
I	R	A	I	L	N	I	I	E	Q	L	M	I	L	T	A	R	E	Q	I	E	P
I	R	A	I	L	N	I	I	E	Q	L	M	I	L	T	A	R	E	Q	I	E	P
I	R	A	I	L	N	I	I	E	Q	L	M	I	L	T	A	R	E	Q	I	E	P
I	R	A	I	L	N	I	I	E	Q	L	M	I	L	T	A	R	E	Q	I	E	P
I	R	A	I	L	N	I	I	E	Q	L	M	I	L	T	A	R	E	Q	I	E	P
I	R	A	I	L	N	I	I	E	Q	L	M	I	L	T	A	R	E	Q	I	E	P
I	R	A	I	L	N	I	I	E	Q	L	M	I	L	T	A	R	E	Q	I	E	P
I	R	A	I	L	N	I	I	E	Q	L	M	I	L	T	A	R	E	Q	I	E	P
I	R	A	I	L	N	I	I	E	Q	L	M	I	L	T	A	R	E	Q	I	E	P
I	R	A	I	L	N	I	I	E	Q	L	M	I	L	T	A	R	E	Q	I	E	P
I	R	A	I	L	N	I	I	E	Q	L	M	I	L	T	A	R	E	Q	I	E	P
I	R	A	I	L	N	I	I	E	Q	L	M	I	L	T	A	R	E	Q	I	E	P
I	R	A	I	L	N	I	I	E	Q	L	M	I	L	T	A	R	E	Q	I	E	P
I	R	A	I	L	N	I	I	E	Q	L	M	I	L	T	A	R	E	Q	I	E	P
I	R	A	I	L	N	I	I	E	Q	L	M	I	L	T	A	R	E	Q	I	E	P
I	R	A	I	L	N	I	I	E	Q	L	M	I	L	T	A	R	E	Q	I	E	P
I	R	A	I	L	N	I	I	E	Q	L	M	I	L	T	A	R	E	Q	I	E	P

I	R	A	I	L	N	I	I	E	Q	L	M	I	L	T	A	R	E	Q	I	E	P
I	R	A	I	L	N	I	I	E	Q	L	M	I	L	T	A	R	E	Q	I	E	P
I	R	A	I	L	N	I	I	E	Q	L	M	I	L	T	A	R	E	Q	I	E	P
I	R	A	I	L	N	I	I	E	Q	L	M	I	L	T	A	R	E	Q	I	E	P
I	R	A	I	L	N	I	I	E	Q	L	M	I	L	T	A	R	E	Q	I	E	P
I	R	A	I	L	N	I	I	E	Q	L	M	I	L	T	A	R	E	Q	I	E	P
I	R	A	I	L	N	I	I	E	Q	L	M	I	L	T	A	R	E	Q	I	E	P
I	R	A	I	L	N	I	I	E	Q	L	M	I	L	T	A	R	E	Q	I	E	P
I	R	A	I	L	N	I	I	E	Q	L	M	I	L	T	A	R	E	Q	I	E	P
I	R	A	I	L	N	I	I	E	Q	L	M	I	L	T	A	R	E	Q	I	E	P
I	R	A	I	L	N	I	I	E	Q	L	M	I	L	T	A	R	E	Q	I	E	P
I	R	A	I	L	N	I	I	E	Q	L	M	I	L	T	A	R	E	Q	I	E	P
I	R	A	I	L	N	I	I	E	Q	L	M	I	L	T	A	R	E	Q	I	E	P
I	R	A	I	L	N	I	I	E	Q	L	M	I	L	T	A	R	E	Q	I	E	P
I	R	A	I	L	N	I	I	E	Q	L	M	I	L	T	A	R	E	Q	I	E	P
I	R	A	I	L	N	I	I	E	Q	L	M	I	L	T	A	R	E	Q	I	E	P
I	R	A	I	L	N	I	I	E	Q	L	M	I	L	T	A	R	E	Q	I	E	P
I	R	A	I	L	N	I	I	E	Q	L	M	I	L	T	A	R	E	Q	I	E	P
I	R	A	I	L	N	I	I	E	Q	L	M	I	L	T	A	R	E	Q	I	E	P
I	R	A	I	L	N	I	I	E	Q	L	M	I	L	T	A	R	E	Q	I	E	P
I	R	A	I	L	N	I	I	E	Q	L	M	I	L	T	A	R	E	Q	I	E	P
I	R	A	I	L	N	I	I	E	Q	L	M	I	L	T	A	R	E	Q	I	E	P
I	R	A	I	L	N	I	I	E	Q	L	M	I	L	T	A	R	E	Q	I	E	P
I	R	A	I	L	N	I	I	E	Q	L	M	I	L	T	A	R	E	Q	I	E	P
I	R	A	I	L	N	I	I	E	Q	L	M	I	L	T	A	R	E	Q	I	E	P
I	R	A	I	L	N	I	I	E	Q	L	M	I	L	T	A	R	E	Q	I	E	P
I	R	A	I	L	N	I	I	E	Q	L	M	I	L	T	A	R	E	Q	I	E	P
I	R	A	I	L	N	I	I	E	Q	L	M	I	L	T	A	R	E	Q	I	E	P
I	R	A	I	L	N	I	I	E	Q	L	M	I	L	T	A	R	E	Q	I	E	P
I	R	A	I	L	N	I	I	E	Q	L	M	I	L	T	A	R	E	Q	I	E	P
I	R	A	I	L	N	I	I	E	Q	L	M	I	L	T	A	R	E	Q	I	E	P
I	R	A	I	L	N	I	I	E	Q	L	M	I	L	T	A	R	E	Q	I	E	P
I	R	A	I	L	N	I	I	E	Q	L	M	I	L	T	A	R	E	Q	I	E	P
I	R	A	I	L	N	I	I	E	Q	L	M	I	L	T	A	R	E	Q	I	E	P
I	R	A	I	L	N	I	I	E	Q	L	M	I	L	T	A	R	E	Q	I	E	P
I	R	A	I	L	N	I	I	E	Q	L	M	I	L	T	A	R	E	Q	I	E	P
I	R	A	I	L	N	I	I	E	Q	L	M	I	L	T	A	R	E	Q	I	E	P
I	R	A	I	L	N	I	I	E	Q	L	M	I	L	T	A	R	E	Q	I	E	P
I	R	A	I	L	N	I	I	E	Q	L	M	I	L	T	A	R	E	Q	I	E	P
I	R	A	I	L	N	I	I	E	Q	L	M	I	L	T	A	R	E	Q	I	E	P
I	R	A	I	L	N	I	I	E	Q	L	M	I	L	T	A	R	E	Q	I	E	P
I	R	A	I	L	N	I	I	E	Q	L	M	I	L	T	A	R	E	Q	I	E	P
I	R	A	I	L	N	I	I	E	Q	L	M	I	L	T	A	R	E	Q	I	E	P
I	R	A	I	L	N	I	I	E	Q	L	M	I	L	T	A	R	E	Q	I	E	P
I	R	A	I	L	N	I	I	E	Q	L	M	I	L	T	A	R	E	Q	I	E	P
I	R	A	I	L	N	I	I	E	Q	L	M	I	L	T	A	R	E	Q	I	E	P
I	R	A	I	L	N	I	I	E	Q	L	M	I	L	T	A	R	E	Q	I	E	P
I	R	A	I	L	N	I	I	E	Q	L	M	I	L	T	A	R	E	Q	I	E	P
I	R	A	I	L	N	I	I	E	Q	L	M	I	L	T	A	R	E	Q	I	E	P
I	R	A	I	L	N	I	I	E	Q	L	M	I	L	T	A	R	E	Q	I	E	P
I	R	A	I	L	N	I	I	E	Q	L	M	I	L	T	A	R	E	Q	I	E	P
I	R	A	I	L	N	I	I	E	Q	L	M	I	L	T	A	R	E	Q	I	E	P
I	R	A	I	L	N	I	I	E	Q	L	M	I	L	T	A	R	E	Q	I	E	P
I	R	A	I	L	N	I	I	E	Q	L	M	I	L	T	A	R	E	Q	I	E	P
I	R	A	I	L	N	I	I	E	Q	L	M	I	L	T	A	R	E	Q	I	E	P
I	R	A	I	L	N	I	I	E	Q	L	M	I	L	T	A	R	E	Q	I	E	P
I	R	A	I	L	N	I	I	E	Q	L	M	I	L	T	A	R	E	Q	I	E	P
I	R	A	I	L	N	I	I	E	Q	L	M	I	L	T	A	R	E	Q	I	E	P
I	R	A	I	L	N	I	I	E	Q	L	M	I	L	T	A	R	E	Q	I	E	P
I	R	A	I	L	N	I	I	E	Q	L	M	I	L	T	A	R	E	Q	I	E	P
I	R	A	I	L	N	I	I	E	Q	L	M	I	L	T	A	R	E	Q	I	E	P
I	R	A	I	L	N	I	I	E	Q	L	M	I	L	T	A	R	E	Q	I	E	P
I	R	A	I	L	N	I	I	E	Q	L	M	I	L	T	A	R	E	Q	I	E	P
I	R	A	I	L	N	I	I	E	Q	L	M	I	L	T	A	R	E	Q	I	E	P
I	R	A	I	L	N	I	I	E	Q	L	M	I	L	T	A	R	E	Q	I	E	P
I	R	A	I	L	N	I	I	E	Q	L	M	I	L	T	A	R	E	Q	I	E	P
I	R	A	I	L	N	I	I	E	Q	L	M	I	L	T	A	R	E	Q	I	E	P

I	R	A	I	L	N	I	I	E	Q	L	M	I	L	T	A	R	E	Q	I	E	P
I	R	A	I	L	N	I	I	E	Q	L	M	I	L	T	A	R	E	Q	I	E	P
I	R	A	I	L	N	I	I	E	Q	L	M	I	L	T	A	R	E	Q	I	E	P
I	R	A	I	L	N	I	I	E	Q	L	M	I	L	T	A	R	E	Q	I	E	P
I	R	A	I	L	N	I	I	E	Q	L	M	I	L	T	A	R	E	Q	I	E	P
I	R	A	I	L	N	I	I	E	Q	L	M	I	L	T	A	R	E	Q	I	E	P
I	R	A	I	L	N	I	I	E	Q	L	M	I	L	T	A	R	E	Q	I	E	P
I	R	A	I	L	N	I	I	E	Q	L	M	I	L	T	A	R	E	Q	I	E	P
I	R	A	I	L	N	I	I	E	Q	L	M	I	L	T	A	R	E	Q	I	E	P
I	R	A	I	L	N	I	I	E	Q	L	M	I	L	T	A	R	E	Q	I	E	P
I	R	A	I	L	N	I	I	E	Q	L	M	I	L	T	A	R	E	Q	I	E	P
I	R	A	I	L	N	I	I	E	Q	L	M	I	L	T	A	R	E	Q	I	E	P
I	R	A	I	L	N	I	I	E	Q	L	M	I	L	T	A	R	E	Q	I	E	P
I	R	A	I	L	N	I	I	E	Q	L	M	I	L	T	A	R	E	Q	I	E	P
I	R	A	I	L	N	I	I	E	Q	L	M	I	L	T	A	R	E	Q	I	E	P
I	R	A	I	L	N	I	I	E	Q	L	M	I	L	T	A	R	E	Q	I	E	P
I	R	A	I	L	N	I	I	E	Q	L	M	I	L	T	A	R	E	Q	I	E	P
I	R	A	I	L	N	I	I	E	Q	L	M	I	L	T	A	R	E	Q	I	E	P
I	R	A	I	L	N	I	I	E	Q	L	M	I	L	T	A	R	E	Q	I	E	P
I	R	A	I	L	N	I	I	E	Q	L	M	I	L	T	A	R	E	Q	I	E	P
I	Q	T	V	L	S	V	V	K	K	F	L	V	P	I	A	K	D	L	L	E	P
I	Q	T	V	L	S	V	V	K	K	F	L	V	P	I	A	K	D	L	L	E	P
I	Q	T	V	L	S	V	V	K	K	F	L	V	P	I	A	K	D	L	L	E	P
I	Q	T	V	L	S	V	V	K	K	F	L	V	P	I	A	K	D	L	L	E	P
I	Q	T	V	L	S	V	V	K	K	F	L	V	P	I	A	K	D	L	L	E	P
I	Q	T	V	L	S	V	V	K	K	F	L	V	P	I	A	K	D	L	L	E	P
I	Q	T	V	L	S	V	V	K	K	F	L	V	P	I	A	K	D	L	L	E	P
I	Q	T	V	L	S	V	V	K	K	F	L	V	P	I	A	K	D	L	L	E	P
I	R	A	I	L	N	I	I	E	Q	L	M	I	L	T	A	R	E	Q	I	E	P
I	R	A	I	L	N	I	I	E	Q	L	M	I	L	T	A	R	E	Q	I	E	P
I	R	A	I	L	N	I	I	E	Q	L	M	I	L	T	A	R	E	Q	I	E	P
I	R	A	I	L	N	I	I	E	Q	L	M	I	L	T	A	R	E	Q	I	E	P
I	R	A	I	L	N	I	I	E	Q	L	M	I	L	T	A	R	E	Q	I	E	P
I	R	A	I	L	N	I	I	E	Q	L	M	I	L	T	A	R	E	Q	I	E	P
I	R	A	I	L	N	I	I	E	Q	L	M	I	L	T	A	R	E	Q	I	E	P
I	R	A	I	L	N	I	I	E	Q	L	M	I	L	T	A	R	E	Q	I	E	P
I	R	A	I	L	N	I	I	E	Q	L	M	I	L	T	A	R	E	Q	I	E	P
I	R	A	I	L	N	I	I	E	Q	L	M	I	L	T	A	R	E	Q	I	E	P
I	R	A	I	L	N	I	I	E	Q	L	M	I	L	T	A	R	E	Q	I	E	P
I	R	A	I	L	N	I	I	E	Q	L	M	I	L	T	A	R	E	Q	I	E	P
I	R	A	I	L	N	I	I	E	Q	L	M	I	L	T	A	R	E	Q	I	E	P
I	R	A	I	L	N	I	I	E	Q	L	M	I	L	T	A	R	E	Q	I	E	P
I	R	A	I	L	N	I	I	E	Q	L	M	I	L	T	A	R	E	Q	I	E	P
I	R	A	I	L	N	I	I	E	Q	L	M	I	L	T	A	R	E	Q	I	E	P
I	R	A	I	L	N	I	I	E	Q	L	M	I	L	T	A	R	E	Q	I	E	P
I	R	A	I	L	N	I	I	E	Q	L	M	I	L	T	A	R	E	Q	I	E	P
I	R	A	I	L	N	I	I	E	Q	L	M	I	L	T	A	R	E	Q	I	E	P
I	R	A	I	L	N	I	I	E	Q	L	M	I	L	T	A	R	E	Q	I	E	P
I	R	A	I	F	N	I	I	E	Q	L	M	I	L	T	A	R	E	Q	I	E	P
I	R	A	I	F	N	I	I	E	Q	L	M	I	L	T	A	R	E	Q	I	E	P
I	R	A	I	F	N	I	I	E	Q	L	M	I	L	T	A	R	E	Q	I	E	P
I	R	A	I	F	N	I	I	E	Q	L	M	I	L	T	A	R	E	Q	I	E	P
I	R	A	I	F	N	I	I	E	Q	L	M	I	L	T	A	R	E	Q	I	E	P
I	R	A	I	F	N	I	I	E	Q	L	M	I	L	T	A	R	E	Q	I	E	P
I	R	A	I	F	N	I	I	E	Q	L	M	I	L	T	A	R	E	Q	I	E	P
I	R	A	I	F	N	I	I	E	Q	L	M	I	L	T	A	R	E	Q	I	E	P
I	R	A	I	F	N	I	I	E	Q	L	M	I	L	T	A	R	E	Q	I	E	P
I	R	A	I	L	N	I	I	E	Q	L	M	I	L	T	A	R	E	Q	I	E	P
I	R	A	I	F	N	I	I	E	Q	L	M	I	L	T	A	R	E	Q	I	E	P
I	R	A	I	F	N	I	I	E	Q	L	M	I	L	T	A	R	E	Q	I	E	P
I	R	A	I	F	N	I	I	E	Q	L	M	I	L	T	A	R	E	Q	I	E	P
I	R	A	I	F	N	I	I	E	Q	L	M	I	L	T	A	R	E	Q	I	E	P
I	R	A	I	F	N	I	I	E	Q	L	M	I	L	T	A	R	E	Q	I	E	P
I	R	A	I	F	N	I	I	E	Q	L	M	I	L	T	A	R	E	Q	I	E	P
I	R	A	I	F	N	I	I	E	Q	L	M	I	L	T	A	R	E	Q	I	E	P
I	R	A	I	F	N	I	I	E	Q	L	M	I	L	T	A	R	E	Q	I	E	P
I	R	A	I	F	N	I	I	E	Q	L	M	I	L	T	A	R	E	Q	I	E	P

I	R	A	I	F	N	I	I	E	Q	L	M	I	L	T	A	R	E	Q	I	E	P
I	R	A	I	F	N	I	I	E	Q	L	M	I	L	T	A	R	E	Q	I	E	P
I	R	A	I	F	N	I	I	E	Q	L	M	I	L	T	A	R	E	Q	I	E	P
I	R	A	I	F	N	I	I	E	Q	L	M	I	L	T	A	R	E	Q	I	E	P
I	R	A	I	F	N	I	I	E	Q	L	M	I	L	T	A	R	E	Q	I	E	P
I	R	A	I	F	N	I	I	E	Q	L	M	I	L	T	A	R	E	Q	I	E	P
I	R	A	I	F	N	I	I	E	Q	L	M	I	L	T	A	R	E	Q	I	E	P
I	R	A	I	F	N	I	I	E	Q	L	M	I	L	T	A	R	E	Q	I	E	P
I	R	A	I	F	N	I	I	E	Q	L	M	I	L	T	A	R	E	Q	I	E	P
I	R	A	I	F	N	I	I	E	Q	L	M	I	L	T	A	R	E	Q	I	E	P
I	R	A	I	F	N	I	I	E	Q	L	M	I	L	T	A	R	E	Q	I	E	P
I	R	A	I	F	N	I	I	E	Q	L	M	I	L	T	A	R	E	Q	I	E	P
I	R	A	I	F	N	I	I	E	Q	L	M	I	L	T	A	R	E	Q	I	E	P
I	R	A	I	F	N	I	I	E	Q	L	M	I	L	T	A	R	E	Q	I	E	P
I	R	A	I	F	N	I	I	E	Q	L	M	I	L	T	A	R	E	Q	I	E	P
I	R	A	I	F	N	I	I	E	Q	L	M	I	L	T	A	R	E	Q	I	E	P
I	R	A	I	F	N	I	I	E	Q	L	M	I	L	T	A	R	E	Q	I	E	P
I	R	A	I	F	N	I	I	E	Q	L	M	I	L	T	A	R	E	Q	I	E	P
I	R	A	I	F	N	I	I	E	Q	L	M	I	L	T	A	R	E	Q	I	E	P
I	R	A	I	F	N	I	I	E	Q	L	M	I	L	T	A	R	E	Q	I	E	P
I	R	A	I	F	N	I	I	E	Q	L	M	I	L	T	A	R	E	Q	I	E	P
I	R	A	I	F	N	I	I	E	Q	L	M	I	L	T	A	R	E	Q	I	E	P
I	R	A	I	F	N	I	I	E	Q	L	M	I	L	T	A	R	E	Q	I	E	P
V	R	A	I	F	N	I	I	E	Q	L	M	I	L	T	A	R	E	Q	I	E	P
I	R	A	I	L	N	I	I	E	Q	L	M	I	L	T	T	R	E	Q	I	E	P
I	R	A	I	L	N	I	I	E	Q	L	M	I	L	T	T	R	E	Q	I	E	P
I	R	A	I	L	N	I	I	E	Q	L	M	I	L	T	T	R	E	Q	I	E	P
I	R	A	I	L	N	I	I	E	Q	L	M	I	L	T	T	R	E	Q	I	E	P
I	R	A	I	L	N	I	I	E	Q	L	M	I	L	T	T	R	E	Q	I	E	P
I	R	A	I	L	N	I	I	E	Q	L	M	I	L	T	T	R	E	Q	I	E	P
I	R	A	I	L	N	I	I	E	Q	L	M	I	L	T	T	R	E	Q	I	E	P
I	R	A	I	L	N	I	I	E	Q	L	M	I	L	T	T	R	E	Q	I	E	P
I	R	A	I	L	N	I	I	E	Q	L	M	I	L	T	T	R	E	Q	I	E	P
I	R	A	I	L	N	I	I	E	Q	L	M	I	L	T	T	R	E	Q	I	E	P
I	R	A	I	L	N	I	I	E	Q	L	M	I	L	T	T	R	E	Q	I	E	P
I	R	A	I	L	N	I	I	E	Q	L	M	I	L	T	T	R	E	Q	I	E	P
I	R	A	I	L	N	I	I	E	Q	L	M	I	L	T	T	R	E	Q	I	E	P
I	R	A	I	L	N	I	I	E	Q	L	M	I	L	T	T	R	E	Q	I	E	P
I	R	A	I	L	N	I	I	E	Q	L	M	I	L	T	T	R	E	Q	I	E	P
I	R	A	I	L	N	I	I	E	Q	L	M	I	L	T	T	R	E	Q	I	E	P
I	R	A	I	L	N	I	I	E	Q	L	M	I	L	T	A	R	E	Q	I	E	P
I	R	A	I	L	N	I	I	E	Q	L	M	I	L	T	A	R	E	Q	I	E	P
I	R	A	I	L	N	I	I	E	Q	L	M	I	L	T	A	R	E	Q	I	E	P
I	R	A	I	L	N	I	I	E	Q	L	M	I	L	T	A	R	E	Q	I	E	P
I	R	A	I	L	N	I	I	E	Q	L	M	I	L	T	A	R	E	Q	I	E	P
I	R	A	I	L	N	I	I	E	Q	L	M	I	L	T	A	R	E	Q	I	E	P
I	R	A	I	L	N	I	I	E	Q	L	M	I	L	T	A	R	E	Q	I	E	P
I	R	A	I	L	N	I	I	E	Q	L	M	I	L	T	A	R	E	Q	I	E	P
I	R	A	I	L	N	I	I	E	Q	L	M	I	L	T	A	R	E	Q	I	E	P
V	R	A	I	L	N	I	I	E	Q	L	M	I	L	T	A	R	E	Q	I	K	P
V	R	A	I	L	N	I	I	E	Q	L	M	I	L	T	A	R	E	Q	I	K	P
V	R	A	I	L	N	I	I	E	Q	L	M	I	L	T	A	R	E	Q	I	K	P
V	R	A	I	L	N	I	I	E	Q	L	M	I	L	T	A	R	E	Q	I	K	P
V	R	A	I	L	N	I	I	E	Q	L	M	I	L	T	A	R	E	Q	I	K	P
V	R	A	I	L	N	I	I	E	Q	L	M	I	L	T	A	R	E	Q	I	K	P
V	R	A	I	L	N	I	I	E	Q	L	M	I	L	T	A	R	E	Q	I	K	P
V	R	A	I	L	N	I	I	E	Q	L	M	I	L	T	A	R	E	Q	I	K	P
V	R	A	I	L	N	I	I	E	Q	L	M	I	L	T	A	R	E	Q	I	K	P
V	R	A	I	L	N	I	I	E	Q	L	M	I	L	T	A	R	E	Q	I	K	P
V	R	A	I	L	N	I	I	E	Q	L	M	I	L	T	A	R	E	Q	I	K	P
V	R	A	I	L	N	I	I	E	Q	L	M	I	L	T	A	R	E	Q	I	K	P
V	R	A	I	L	N	I	I	E	Q	L	M	I	L	T	A	R	E	Q	I	K	P
V	R	A	I	L	N	I	I	E	Q	L	M	I	L	T	A	R	E	Q	I	K	P
V	R	A	I	L	N	I	I	E	Q	L	M	I	L	T	A	R	E	Q	I	K	P
V	R	A	I	L	N	I	I	E	Q	L	M	I	L	T	A	R	E	Q	I	K	P
V	R	A	I	L	N	I	I	E	Q	L	M	I	L	T	A	R	E	Q	I	K	P
V	R	A	I	L	N	I	I	E	Q	L	M	I	L	T	A	R	E	Q	I	K	P

V	R	A	I	L	N	I	I	E	Q	L	M	I	L	T
V	R	A	I	L	N	I	I	E	Q	L	M	I	L	T
V	R	A	I	L	N	I	I	E	Q	L	M	I	L	T
V	R	A	I	L	N	I	I	E	Q	L	M	I	L	T
V	R	A	I	L	N	I	I	E	Q	L	M	I	L	T
V	R	A	I	L	N	I	I	E	Q	L	M	I	L	T
V	R	A	I	L	N	I	I	E	Q	L	M	I	L	T
V	R	A	I	L	N	I	I	E	Q	L	M	I	L	T
V	R	A	I	L	N	I	I	E	Q	L	M	I	L	T
V	R	A	I	L	N	I	I	E	Q	L	M	I	L	T

A	R	E	Q	I	K	P
A	R	E	Q	I	K	P
A	R	E	Q	I	K	P
A	R	E	Q	I	K	P
A	R	E	Q	I	K	P
A	R	E	Q	I	K	P
A	R	E	Q	I	K	P
A	R	E	Q	I	K	P
A	R	E	Q	I	K	P
A	R	E	Q	I	K	P

422	425	437	457
M	I	L	T
L	V	P	I
M	I	L	T
L	V	P	I
L	V	P	I
L	V	P	I
L	V	P	I
L	V	P	I
L	V	P	I
L	V	P	I
L	V	P	I

rmlA																	
47	63	66	91	118	125	136	150	151	163	186	191	200	206	263	268	272	286
R	E	Q	I	T	K	H	E	N	Y	S	P	V	D	R	A	S	V
K	D	L	L	K	Q	Q	T	D	N	G	A	I	N	C	E	P	I
R	E	Q	I	K	K	Q	T	D	Y	S	P	V	D	R	A	P	I
K	D	L	L	K	Q	Q	T	D	N	G	A	I	N	C	E	P	I
K	D	L	L	K	Q	Q	T	D	N	G	A	I	N	C	E	P	I
K	D	L	L	K	Q	Q	T	D	N	G	A	I	N	C	E	P	I
K	D	L	L	K	Q	Q	T	D	N	G	A	I	N	C	E	P	I
K	D	L	L	K	Q	Q	T	D	N	G	A	I	N	C	E	P	I
K	D	L	L	K	Q	Q	T	D	N	G	A	I	N	C	E	P	I
K	D	L	L	K	Q	Q	T	D	N	G	A	I	N	C	E	P	I
K	D	L	L	K	Q	Q	T	D	N	G	A	I	N	C	E	P	I

rmlA																					
63	66	91	118	125	136	150	151	163	186	191	200	206	214	255	263	268	272	286	2	12	15
E	Q	I	T	K	H	E	N	Y	S	P	V	D	L	A	R	A	S	V	T	A	V
D	L	L	K	Q	Q	T	D	N	G	A	I	N	L	A	C	E	P	I	S	V	I
E	Q	I	K	K	Q	T	D	H	S	P	V	G	V	S	R	A	P	I	S	V	I
E	Q	I	K	K	Q	T	D	H	S	P	V	G	V	S	R	A	P	I	S	V	I
E	Q	I	K	K	Q	T	D	H	S	P	V	G	V	S	R	A	P	I	S	V	I
E	Q	I	K	K	Q	T	D	H	S	P	V	G	V	S	R	A	P	I	S	V	I
E	Q	I	K	K	Q	T	D	H	S	P	V	G	V	S	R	A	P	I	S	V	I
E	Q	I	K	K	Q	T	D	H	S	P	V	G	V	S	R	A	P	I	S	V	I
E	Q	I	K	K	Q	T	D	H	S	P	V	G	V	S	R	A	P	I	S	V	I
E	Q	I	K	K	Q	T	D	H	S	P	V	G	V	S	R	A	P	I	S	V	I
E	Q	I	K	K	Q	T	D	H	S	P	V	G	V	S	R	A	P	I	S	V	I
E	Q	I	K	K	Q	T	D	H	S	P	V	G	V	S	R	A	P	I	S	V	I
E	Q	I	K	K	Q	T	D	H	S	P	V	G	V	S	R	A	P	I	S	V	I
E	Q	I	K	K	Q	T	D	H	S	P	V	G	V	S	R	A	P	I	S	V	I
E	Q	I	K	K	Q	T	D	H	S	P	V	G	V	S	R	A	P	I	S	V	I
E	Q	I	K	K	Q	T	D	H	S	P	V	G	V	S	R	A	P	I	S	V	I
E	Q	I	K	K	Q	T	D	H	S	P	V	G	V	S	R	A	P	I	S	V	I
E	Q	I	K	K	Q	T	D	H	S	P	V	G	V	S	R	A	P	I	S	V	I
E	Q	I	K	K	Q	T	D	H	S	P	V	G	V	S	R	A	P	I	S	V	I
E	Q	I	K	K	Q	T	D	H	S	P	V	G	V	S	R	A	P	I	S	V	I
E	Q	I	K	K	Q	T	D	H	S	P	V	G	V	S	R	A	P	I	S	V	I
E	Q	I	K	K	H	T	D	N	G	A	I	N	L	A	C	E	P	I	S	V	I
E	Q	I	K	K	H	T	D	N	G	A	I	N	L	A	C	E	P	I	S	V	I
E	Q	I	K	K	H	T	D	N	G	A	I	N	L	A	C	E	P	I	S	V	I
E	Q	I	K	K	H	T	D	N	G	A	I	N	L	A	C	E	P	I	S	V	I
E	Q	I	K	K	H	T	D	Y	S	P	V	D	L	A	R	A	P	I	T	A	V
E	Q	I	K	K	H	T	D	N	G	A	I	N	L	A	C	E	P	I	S	V	I
E	Q	I	K	K	H	T	D	N	G	A	I	N	L	A	C	E	P	I	S	V	I
E	Q	I	K	K	H	T	D	N	G	A	I	N	L	A	C	E	P	I	S	V	I
E	Q	I	K	K	H	T	D	N	G	A	I	N	L	A	C	E	P	I	S	V	I
E	Q	I	K	K	H	T	D	N	G	A	I	N	L	A	C	E	P	I	S	V	I

rmlA	rmlC

118	125	136	150	151	163	186	191	200	206	214	255	263	268	272	286
T	K	H	E	N	Y	S	P	V	D	L	A	R	A	S	V
K	Q	Q	T	D	N	G	A	I	N	L	A	C	E	P	I
K	K	Q	T	D	H	S	P	V	G	V	S	R	A	P	I
K	K	Q	T	D	H	S	P	V	G	V	S	R	A	P	I
K	K	Q	T	D	H	S	P	V	G	V	S	R	A	P	I
K	K	Q	T	D	H	S	P	V	G	V	S	R	A	P	I
K	K	Q	T	D	H	S	P	V	G	V	S	R	A	P	I
K	K	Q	T	D	H	S	P	V	G	V	S	R	A	P	I
K	K	Q	T	D	H	S	P	V	G	V	S	R	A	P	I
K	K	Q	T	D	H	S	P	V	G	V	S	R	A	P	I
K	K	Q	T	D	H	S	P	V	G	V	S	R	A	P	I
K	K	Q	T	D	H	S	P	V	G	V	S	R	A	P	I
K	K	Q	T	D	H	S	P	V	G	V	S	R	A	P	I
K	K	Q	T	D	H	S	P	V	G	V	S	R	A	P	I
K	K	Q	T	D	H	S	P	V	G	V	S	R	A	P	I
K	K	Q	T	D	H	S	P	V	G	V	S	R	A	P	I
K	K	Q	T	D	H	S	P	V	G	V	S	R	A	P	I
K	K	Q	T	D	H	S	P	V	G	V	S	R	A	P	I
K	K	Q	T	D	H	S	P	V	G	V	S	R	A	P	I
K	K	Q	T	D	H	S	P	V	G	V	S	R	A	P	I
K	K	Q	T	D	H	S	P	V	G	V	S	R	A	P	I
K	K	Q	T	D	H	S	P	V	G	V	S	R	A	P	I
K	K	Q	T	D	H	S	P	V	G	V	S	R	A	P	I
K	K	Q	T	D	H	S	P	V	G	V	S	R	A	P	I
K	K	Q	T	D	H	S	P	V	G	V	S	R	A	P	I
K	K	Q	T	D	H	S	P	V	G	V	S	R	A	P	I
K	K	Q	T	D	H	S	P	V	G	V	S	R	A	P	I

2	12	15	16	21	46
T	A	V	E	M	L
S	V	I	D	L	E
T	A	V	E	M	L
T	A	V	E	M	L
T	A	V	E	M	L
T	A	V	E	M	L
T	A	V	E	M	L
T	A	V	E	M	L
T	A	V	E	M	L
T	A	V	E	M	L
T	A	V	E	M	L
T	A	V	E	M	L
T	A	V	E	M	L
T	A	V	E	M	L
T	A	V	E	M	L
T	A	V	E	M	L
T	A	V	E	M	L
T	A	V	E	M	L
T	A	V	E	M	L
T	A	V	E	M	L
T	A	V	E	M	L
T	A	V	E	M	L
T	A	V	E	M	L
T	A	V	E	M	L
T	A	V	E	M	L
T	A	V	E	M	L
T	A	V	E	M	L

rmlA																			
118	123	125	128	136	140	150	151	153	163	186	191	200	206	214	255	263	268	272	286
T	A	K	K	H	P	E	N	N	Y	S	P	V	D	L	A	R	A	S	V
T	A	K	K	H	P	E	N	N	Y	S	P	V	D	V	A	R	T	P	I
K	A	K	K	Q	P	T	D	N	Y	S	P	V	D	L	A	R	A	P	I
K	A	K	K	Q	P	T	D	N	Y	S	P	V	D	L	A	R	A	P	I
K	A	K	K	Q	P	T	D	N	Y	S	P	V	D	L	A	R	A	P	I
K	A	K	K	Q	P	T	D	N	Y	S	P	V	D	L	A	R	A	P	I
K	A	K	K	Q	P	T	D	N	Y	S	P	V	D	L	A	R	A	P	I
K	A	K	K	Q	P	T	D	N	Y	S	P	V	D	L	A	R	A	P	I
K	A	K	K	Q	P	T	D	N	Y	S	P	V	D	L	A	R	A	P	I
K	A	K	K	Q	P	T	D	N	Y	S	P	V	D	L	A	R	A	P	I
K	A	K	K	Q	P	T	D	N	Y	S	P	V	D	L	A	R	A	P	I
K	A	K	K	Q	P	T	D	N	Y	S	P	V	D	L	A	R	A	P	I
K	A	K	K	Q	P	T	D	N	Y	S	P	V	D	L	A	R	A	P	I
K	A	K	K	Q	P	T	D	N	Y	S	P	V	D	L	A	R	A	P	I
K	A	K	K	Q	P	T	D	N	Y	S	P	V	D	L	A	R	A	P	I
K	A	K	K	Q	P	T	D	N	Y	S	P	V	D	L	A	R	A	P	I
K	A	K	K	Q	P	T	D	N	Y	S	P	V	D	L	A	R	A	P	I
K	A	K	K	Q	P	T	D	N	Y	S	P	V	D	L	A	R	A	P	I
K	A	K	K	Q	P	T	D	N	Y	S	P	V	D	L	A	R	A	P	I
K	A	K	K	Q	P	T	D	N	Y	S	P	V	D	L	A	R	A	P	I
K	A	K	K	Q	P	T	D	N	Y	S	P	V	D	L	A	R	A	P	I
K	A	K	K	Q	P	T	D	N	Y	S	P	V	D	L	A	R	A	P	I
K	A	K	K	Q	P	T	D	N	Y	S	P	V	D	L	A	R	A	P	I
K	A	K	K	Q	P	T	D	N	Y	S	P	V	D	L	A	R	A	P	I
K	A	K	K	Q	P	T	D	N	Y	S	P	V	D	L	A	R	A	P	I
K	A	K	K	Q	P	T	D	N	Y	S	P	V	D	L	A	R	A	P	I
K	A	K	K	Q	P	T	D	N	Y	S	P	V	D	L	A	R	A	P	I
K	A	K	K	Q	P	T	D	N	Y	S	P	V	D	L	A	R	A	P	I
K	A	K	K	Q	P	T	D	N	Y	S	P	V	D	L	A	R	A	P	I
K	A	K	K	Q	P	T	D	N	Y	S	P	V	D	L	A	R	A	P	I
K	A	K	K	Q	P	T	D	N	Y	S	P	V	D	L	A	R	A	P	I
K	A	K	K	Q	P	T	D	N	Y	S	P	V	D	L	A	R	A	P	I
K	A	K	K	Q	P	T	D	N	Y	S	P	V	D	L	A	R	A	P	I
K	A	K	K	Q	P	T	D	N	Y	S	P	V	D	L	A	R	A	P	I
K	A	K	K	Q	P	T	D	N	Y	S	P	V	D	L	A	R	A	P	I
K	A	K	K	Q	P	T	D	N	Y	S	P	V	D	L	A	R	A	P	I
K	A	K	K	Q	P	T	D	N	Y	S	P	V	D	L	A	R	A	P	I
K	A	K	K	Q	P	T	D	N	Y	S	P	V	D	L	A	R	A	P	I
K	A	K	K	Q	P	T	D	N	Y	S	P	V	D	L	A	R	A	P	I
K	A	K	K	Q	P	T	D	N	Y	S	P	V	D	L	A	R	A	P	I
K	A	K	K	Q	P	T	D	N	Y	S	P	V	D	L	A	R	A	P	I
K	A	K	K	Q	P	T	D	N	Y	S	P	V	D	L	A	R	A	P	I
K	A	K	K	Q	P	T	D	N	Y	S	P	V	D	L	A	R	A	P	I
K	A	K	K	Q	P	T	D	N	Y	S	P	V	D	L	A	R	A	P	I
K	A	K	K	Q	P	T	D	N	Y	S	P	V	D	L	A	R	A	P	I
K	A	K	K	Q	P	T	D	N	Y	S	P	V	D	L	A	R	A	P	I
K	A	K	K	Q	P	T	D	N	Y	S	P	V	D	L	A	R	A	P	I
K	A	K	K	Q	P	T	D	N	Y	S	P	V	D	L	A	R	A	P	I
K	A	K	K	Q	P	T	D	N	Y	S	P	V	D	L	A	R	A	P	I
K	A	K	K	Q	P	T	D	N	Y	S	P	V	D	L	A	R	A	P	I
K	A	K	K	Q	P	T	D	N	Y	S	P	V	D	L	A	R	A	P	I
K	A	K	K	Q	P	T	D	N	Y	S	P	V	D	L	A	R	A	P	I
K	A	K	K	Q	P	T	D	N	Y	S	P	V	D	L	A	R	A	P	I
K	A	K	K	Q	P	T	D	N	Y	S	P	V	D	L	A	R	A	P	I
K	A	K	K	H	P	T	D	N	Y	S	P	V	D	L	A	R	T	P	I
K	A	K	K	Q	P	T	D	N	Y	S	P	V	D	L	A	R	A	P	I
K	A	K	K	Q	P	T	D	N	Y	S	P	V	D	L	A	R	A	P	I
K	A	K	K	Q	P	T	D	N	Y	S	P	V	D	L	A	R	A	P	I
K	A	K	K	Q	P	T	D	N	Y	S	P	V	D	L	A	R	A	P	I
K	A	K	K	Q	P	T	D	N	Y	S	P	V	D	L	A	R	A	P	I
K	A	K	K	H	P	T	D	N	Y	S	P	V	D	L	A	R	T	P	I
K	A	K	K	H	P	T	D	N	Y	S	P	V	D	L	A	R	T	P	I
K	A	K	K	H	P	T	D	N	Y	S	P	V	D	L	A	R	T	P	I
K	A	K	K	H	P	T	D	N	Y	S	P	V	D	L	A	R	T	P	I

2	11
T	A
T	A
T	A
T	A
T	A
T	A
T	A
T	A
T	A
T	A
T	A
T	A
T	A
T	A
T	A
T	A
T	A
T	A
T	A
T	A
T	A
T	A
T	A
T	A
T	A
T	A
T	A
T	A
T	A
T	A
T	A
T	A
T	A
T	A
T	A
T	A
T	A
T	A
T	A
T	A
T	A
T	A
T	A
T	A
T	A
T	A
T	A
T	A
T	A
T	A
T	A
T	A
T	A
T	A
T	A
T	A
T	A
T	A
T	A
T	A
T	A
T	A
T	A

K	A	K	K	H	P	T	D	N	Y	S	P	V	D	L	A	R	T	P	I	T	A
K	A	K	K	H	P	T	D	N	Y	S	P	V	D	L	A	R	T	P	I	T	A
K	A	K	K	H	P	T	D	N	Y	S	P	V	D	L	A	R	T	P	I	T	A
K	A	K	K	H	P	T	D	N	Y	S	P	V	D	L	A	R	T	P	I	T	A
K	A	K	K	H	P	T	D	N	Y	S	P	V	D	L	A	R	T	P	I	T	A
K	A	K	K	H	P	T	D	N	Y	S	P	V	D	L	A	R	T	P	I	T	A
K	A	K	K	H	P	T	D	N	Y	S	P	V	D	L	A	R	T	P	I	T	A
K	A	K	K	H	P	T	D	N	Y	S	P	V	D	L	A	R	T	P	I	T	A
K	A	K	K	H	P	T	D	N	Y	S	P	V	D	L	A	R	T	P	I	T	A
K	A	K	K	H	P	T	D	N	Y	S	P	V	D	L	A	R	T	P	I	T	A
K	A	K	K	H	P	T	D	N	Y	S	P	V	D	L	A	R	T	P	I	T	A
K	A	K	K	H	P	T	D	N	Y	S	P	V	D	L	A	R	T	P	I	T	A
K	A	K	K	H	P	T	D	N	Y	S	P	V	D	L	A	R	T	P	I	T	A
K	A	K	K	H	P	T	D	N	Y	S	P	V	D	L	A	R	T	P	I	T	A
K	A	K	K	H	P	T	D	N	Y	S	P	V	D	L	A	R	T	P	I	T	A
K	A	K	K	H	P	T	D	N	Y	S	P	V	D	L	A	R	T	P	I	T	A
K	A	K	K	H	P	T	D	N	Y	S	P	V	D	L	A	R	T	P	I	T	A
K	A	K	K	H	P	T	D	N	Y	S	P	V	D	L	A	R	T	P	I	T	A
K	A	K	K	H	P	T	D	N	Y	S	P	V	D	L	A	R	T	P	I	T	A
K	A	K	K	H	P	T	D	N	Y	S	P	V	D	L	A	R	T	P	I	T	A
K	A	K	K	H	P	T	D	N	Y	S	P	V	D	L	A	R	T	P	I	T	A
K	A	K	K	Q	P	T	D	N	Y	S	P	V	D	L	A	R	A	P	I	T	A
K	A	K	K	Q	P	T	D	N	Y	S	P	V	D	L	A	R	A	P	I	T	A
K	A	S	S	H	S	Q	D	K	Q	S	P	V	E	L	A	R	A	P	I	T	A
T	A	K	K	H	P	E	N	N	Y	S	P	V	D	L	A	R	A	S	I	T	A
T	A	K	K	H	P	E	N	N	Y	S	P	V	D	L	A	R	A	S	I	T	A
T	A	K	K	H	P	E	N	N	Y	S	P	V	D	L	A	R	A	S	I	T	A
T	A	K	K	H	P	E	N	N	Y	S	P	V	D	L	A	R	A	S	V	T	A
T	A	K	K	H	P	E	N	N	Y	S	P	V	D	L	A	R	A	S	V	T	A
T	A	K	K	H	P	E	N	N	Y	S	P	V	D	L	A	R	A	S	V	T	A
T	A	K	K	H	P	E	N	N	Y	S	P	V	D	L	A	R	A	S	V	T	A
T	A	K	K	H	P	E	N	N	Y	S	P	V	D	L	A	R	A	S	V	T	A
T	A	K	K	H	P	E	N	N	Y	S	P	V	D	L	A	R	A	S	V	T	A
T	A	K	K	H	P	E	N	N	Y	S	P	V	D	L	A	R	A	S	V	T	A
T	A	K	K	H	P	E	N	N	Y	S	P	V	D	L	A	R	A	S	V	T	A
T	A	K	K	H	P	E	N	N	Y	S	P	V	D	L	A	R	A	S	V	T	A
T	A	K	K	H	P	E	N	N	Y	S	P	V	D	L	A	R	A	S	V	T	A
T	A	K	K	H	P	E	N	N	Y	S	P	V	D	L	A	R	A	S	V	T	A
T	A	K	K	H	P	E	N	N	Y	S	P	V	D	L	A	R	A	S	V	T	A
T	A	K	K	H	P	E	N	N	Y	S	P	V	D	L	A	R	A	S	V	T	A
T	A	K	K	H	P	E	N	N	Y	S	P	V	D	L	A	R	A	S	V	T	A
T	A	K	K	H	P	E	N	N	Y	S	P	V	D	L	A	R	A	S	V	T	A
T	A	K	K	H	P	E	N	N	Y	S	P	V	D	L	A	R	A	S	V	T	A
T	A	K	K	H	P	E	N	N	Y	S	P	V	D	L	A	R	A	S	V	T	A
T	A	K	K	H	P	E	N	N	Y	S	P	V	D	L	A	R	A	S	V	T	A
T	A	K	K	H	P	E	N	N	Y	S	P	V	D	L	A	R	A	S	V	T	A
T	A	K	K	H	P	E	N	N	Y	S	P	V	D	L	A	R	A	S	V	T	A
T	A	K	K	H	P	E	N	N	Y	S	P	V	D	L	A	R	A	S	V	T	A
T	A	K	K	H	P	E	N	N	Y	S	P	V	D	L	A	R	A	S	V	T	A
T	A	K	K	H	P	E	N	N	Y	S	P	V	D	L	A	R	A	S	V	T	A
T	A	K	K	H	P	E	N	N	Y	S	P	V	D	L	A	R	A	S	V	T	A
T	A	K	K	H	P	E	N	N	Y	S	P	V	D	L	A	R	A	S	V	T	A
T	A	K	K	H	P	E	N	N	Y	S	P	V	D	L	A	R	A	S	V	T	A
T	A	K	K	H	P	E	N	N	Y	S	P	V	D	L	A	R	A	S	V	T	A
T	A	K	K	H	P	E	N	N	Y	S	P	V	D	L	A	R	A	S	V	T	A
T	A	K	K	H	P	E	N	N	Y	S	P	V	D	L	A	R	A	S	V	T	A
T	A	K	K	H	P	E	N	N	Y	S	P	V	D	L	A	R	A	S	V	T	A
T	A	K	K	H	P	E	N	N	Y	S	P	V	D	L	A	R	A	S	V	T	A
T	A	K	K	H	P	E	N	N	Y	S	P	V	D	L	A	R	A	S	V	T	A
T	A	K	K	H	P	E	N	N	Y	S	P	V	D	L	A	R	A	S	V	T	A
T	A	K	K	H	P	E	N	N	Y	S	P	V	D	L	A	R	A	S	V	T	A
T	A	K	K	H	P	E	N	N	Y	S	P	V	D	L	A	R	A	S	V	T	A
T	A	K	K	H	P	E	N	N	Y	S	P	V	D	L	A	R	A	S	V	T	A
T	A	K	K	H	P	E	N	N	Y	S	P	V	D	L	A	R	A	S	V	T	A
T	A	K	K	H	P	E	N	N	Y	S	P	V	D	L	A	R	A	S	V	T	A
T	A	K	K	H	P	E	N	N	Y	S	P	V	D	L	A	R	A	S	V	T	A
T	A	K	K	H	P	E	N	N	Y	S	P	V	D	L	A	R	A	S	V	T	A

T	A	K	K	H	P	E	N	N	Y	S	P	V	D	L	A	R	A	S	V	T	A
T	A	K	K	H	P	E	N	N	Y	S	P	V	D	L	A	R	A	S	V	T	A
T	A	K	K	H	P	E	N	N	Y	S	P	V	D	L	A	R	A	S	V	T	A
T	A	K	K	H	P	E	N	N	Y	S	P	V	D	L	A	R	A	S	V	T	A
T	A	K	K	H	P	E	N	N	Y	S	P	V	D	L	A	R	A	S	V	T	A
T	A	K	K	H	P	E	N	N	Y	S	P	V	D	L	A	R	A	S	V	T	A
T	A	K	K	H	P	E	N	N	Y	S	P	V	D	L	A	R	A	S	V	T	A
T	A	K	K	H	P	E	N	N	Y	S	P	V	D	L	A	R	A	S	V	T	A
T	A	K	K	H	P	E	N	N	Y	S	P	V	D	L	A	R	A	S	V	T	A
T	A	K	K	H	P	E	N	N	Y	S	P	V	D	L	A	R	A	S	V	T	A
T	A	K	K	H	P	E	N	N	Y	S	P	V	D	L	A	R	A	S	V	T	A
T	A	K	K	H	P	E	N	N	Y	S	P	V	D	L	A	R	A	S	V	T	A
T	A	K	K	H	P	E	N	N	Y	S	P	V	D	L	A	R	A	S	V	T	A
T	A	K	K	H	P	E	N	N	Y	S	P	V	D	L	A	R	A	S	V	T	A
T	A	K	K	H	P	E	N	N	Y	S	P	V	D	L	A	R	A	S	V	T	A
T	A	K	K	H	P	E	N	N	Y	S	P	V	D	L	A	R	A	S	V	T	A
T	A	K	K	H	P	E	N	N	Y	S	P	V	D	L	A	R	A	S	V	T	A
T	A	K	K	H	P	E	N	N	Y	S	P	V	D	L	A	R	A	S	V	T	A
T	A	K	K	H	P	E	N	N	Y	S	P	V	D	L	A	R	A	S	V	T	A
T	A	K	K	H	P	E	N	N	Y	S	P	V	D	L	A	R	A	S	V	T	A
T	A	K	K	H	P	E	N	N	Y	S	P	V	D	L	A	R	A	S	V	T	A
T	A	K	K	H	P	E	N	N	Y	S	P	V	D	L	A	R	A	S	V	T	A
T	A	K	K	H	P	E	N	N	Y	S	P	V	D	L	A	R	A	S	V	T	A
T	A	K	K	H	P	E	N	N	Y	S	P	V	D	L	A	R	A	S	V	T	A
T	A	K	K	H	P	E	N	N	Y	S	P	V	D	L	A	R	A	S	V	T	A
T	A	K	K	H	P	E	N	N	Y	S	P	V	D	L	A	R	A	S	V	T	A
T	A	K	K	H	P	E	N	N	Y	S	P	V	D	L	A	R	A	S	V	T	A
T	A	K	K	H	P	E	N	N	Y	S	P	V	D	L	A	R	A	S	V	T	A
T	A	K	K	H	P	E	N	N	Y	S	P	V	D	L	A	R	A	S	V	T	A
T	A	K	K	H	P	E	N	N	Y	S	P	V	D	L	A	R	A	S	V	T	A
T	A	K	K	H	P	E	N	N	Y	S	P	V	D	L	A	R	A	S	V	T	A
T	A	K	K	H	P	E	N	N	Y	S	P	V	D	L	A	R	A	S	V	T	A
T	A	K	K	H	P	E	N	N	Y	S	P	V	D	L	A	R	A	S	V	T	A
T	A	K	K	H	P	E	N	N	Y	S	P	V	D	L	A	R	A	S	V	T	A
T	A	K	K	H	P	E	N	N	Y	S	P	V	D	L	A	R	A	S	V	T	A
T	A	K	K	H	P	E	N	N	Y	S	P	V	D	L	A	R	A	S	V	T	A
T	A	K	K	H	P	E	N	N	Y	S	P	V	D	L	A	R	A	S	V	T	A
T	A	K	K	H	P	E	N	N	Y	S	P	V	D	L	A	R	A	S	V	T	A
T	A	K	K	H	P	E	N	N	Y	S	P	V	D	L	A	R	A	S	V	T	A
T	A	K	K	H	P	E	N	N	Y	S	P	V	D	L	A	R	A	S	V	T	A
T	A	K	K	H	P	E	N	N	Y	S	P	V	D	L	A	R	A	S	V	T	A
T	A	K	K	H	P	E	N	N	Y	S	P	V	D	L	A	R	A	S	V	T	A
T	A	K	K	H	P	E	N	N	Y	S	P	V	D	L	A	R	A	S	V	T	A
T	A	K	K	H	P	E	N	N	Y	S	P	V	D	L	A	R	A	S	V	T	A
T	A	K	K	H	P	E	N	N	Y	S	P	V	D	L	A	R	A	S	V	T	A
T	A	K	K	H	P	E	N	N	Y	S	P	V	D	L	A	R	A	S	V	T	A
T	A	K	K	H	P	E	N	N	Y	S	P	V	D	L	A	R	A	S	V	T	A
T	A	K	K	H	P	E	N	N	Y	S	P	V	D	L	A	R	A	S	V	T	A
T	A	K	K	H	P	E	N	N	Y	S	P	V	D	L	A	R	A	S	V	T	A
T	A	K	K	H	P	E	N	N	Y	S	P	V	D	L	A	R	A	S	V	T	A
T	A	K	K	H	P	E	N	N	Y	S	P	V	D	L	A	R	A	S	V	T	A
T	A	K	K	H	P	E	N	N	Y	S	P	V	D	L	A	R	A	S	V	T	A
T	A	K	K	H	P	E	N	N	Y	S	P	V	D	L	A	R	A	S	V	T	A
T	A	K	K	H	P	E	N	N	Y	S	P	V	D	L	A	R	A	S	V	T	A
T	A	K	K	H	P	E	N	N	Y	S	P	V	D	L	A	R	A	S	V	T	A
T	A	K	K	H	P	E	N	N	Y	S	P	V	D	L	A	R	A	S	V	T	A
T	A	K	K	H	P	E	N	N	Y	S	P	V	D	L	A	R	A	S	V	T	A
T	A	K	K	H	P	E	N	N	Y	S	P	V	D	L	A	R	A	S	V	T	A
T	A	K	K	H	P	E	N	N	Y	S	P	V	D	L	A	R	A	S	V	T	A
T	A	K	K	H	P	E	N	N	Y	S	P	V	D	L	A	R	A	S	V	T	A
T	A	K	K	H	P	E	N	N	Y	S	P	V	D	L	A	R	A	S	V	T	A
T	A	K	K	H	P	E	N	N	Y	S	P	V	D	L	A	R	A	S	V	T	A
T	A	K	K	H	P	E	N	N	Y	S	P	V	D	L	A	R	A	S	V	T	A
T	A	K	K	H	P	E	N	N	Y	S	P	V	D	L	A	R	A	S	V	T	A
T	A	K	K	H	P	E	N	N	Y	S	P	V	D	L	A	R	A	S	V	T	A
T	A	K	K	H	P	E	N	N	Y	S	P	V	D	L	A	R	A	S	V	T	A
T	A	K	K	H	P	E	N	N	Y	S	P	V	D	L	A	R	A	S	V	T	A

T	A	K	K	H	P	E	N	N	Y	S	P	V	D	L	A	R	A	S	V	T	A
T	A	K	K	H	P	E	N	N	Y	S	P	V	D	L	A	R	A	S	V	T	A
T	A	K	K	H	P	E	N	N	Y	S	P	V	D	L	A	R	A	S	V	T	A
T	A	K	K	H	P	E	N	N	Y	S	P	V	D	L	A	R	A	S	V	T	A
K	A	K	K	Q	P	T	D	N	Y	S	P	V	D	L	A	R	A	P	I	T	A
K	A	K	K	Q	P	T	D	N	Y	S	P	V	D	L	A	R	A	P	I	T	A
K	A	K	K	Q	P	T	D	N	Y	S	P	V	D	L	A	R	A	P	I	T	A
K	A	K	K	Q	P	T	D	N	Y	S	P	V	D	L	A	R	A	P	I	T	A
K	A	K	K	Q	P	T	D	N	Y	S	P	V	D	L	A	R	A	P	I	T	A
K	A	K	K	Q	P	T	D	N	Y	S	P	V	D	L	A	R	A	P	I	T	A
K	A	K	K	Q	P	T	D	N	Y	S	P	V	D	L	A	R	A	P	I	T	A
K	A	K	K	Q	P	T	D	N	Y	S	P	V	D	L	A	R	A	P	I	T	A
K	A	K	K	Q	P	T	D	N	Y	S	P	V	D	L	A	R	A	P	I	T	A
K	A	K	K	Q	P	T	D	N	Y	S	P	V	D	L	A	R	A	P	I	T	A
K	A	K	K	Q	P	T	D	N	Y	S	P	V	D	L	A	R	A	P	I	T	A
K	A	K	K	Q	P	T	D	N	Y	S	P	V	D	L	A	R	A	P	I	T	A
K	A	K	K	Q	P	T	D	N	Y	S	P	V	D	L	A	R	A	P	I	T	A
K	A	K	K	Q	P	T	D	N	Y	S	P	V	D	L	A	R	A	P	I	T	A
K	A	K	K	Q	P	T	D	N	Y	S	P	V	D	L	A	R	A	P	I	T	A
K	A	K	K	Q	P	T	D	N	Y	S	P	V	D	L	A	R	A	P	I	T	A
K	A	K	K	Q	P	T	D	N	Y	S	P	V	D	L	A	R	A	P	I	T	A
K	A	K	K	Q	P	T	D	N	Y	S	P	V	D	L	A	R	A	P	I	T	A
K	A	K	K	Q	P	T	D	N	Y	S	P	V	D	L	A	R	A	P	I	T	A
K	A	K	K	Q	P	T	D	N	Y	S	P	V	D	L	A	R	A	P	I	T	A
K	A	K	K	Q	P	T	D	N	Y	S	P	V	D	L	A	R	A	P	I	T	A
K	A	K	K	Q	P	T	D	N	Y	S	P	V	D	L	A	R	A	P	I	T	A
K	A	K	K	Q	P	T	D	N	Y	S	P	V	D	L	A	R	A	P	I	T	A
K	A	K	K	Q	P	T	D	N	Y	S	P	V	D	L	A	R	A	P	I	T	A
K	A	K	K	Q	P	T	D	N	Y	S	P	V	D	L	A	R	A	P	I	T	A
K	A	K	K	Q	P	T	D	N	Y	S	P	V	D	L	A	R	A	P	I	T	A
K	A	K	K	Q	P	T	D	N	Y	S	P	V	D	L	A	R	A	P	I	T	A
K	A	K	K	Q	P	T	D	N	Y	S	P	V	D	L	A	R	A	P	I	T	A
K	A	K	K	Q	P	T	D	N	Y	S	P	V	D	L	A	R	A	P	I	T	A
K	A	K	K	Q	P	T	D	N	Y	S	P	V	D	L	A	R	A	P	I	T	A
K	A	K	K	Q	P	T	D	N	Y	S	P	V	D	L	A	R	A	P	I	T	A
K	A	K	K	Q	P	T	D	N	Y	S	P	V	D	L	A	R	A	P	I	T	A
K	A	K	K	Q	P	T	D	N	Y	S	P	V	D	L	A	R	A	P	I	T	A
K	A	K	K	Q	P	T	D	N	Y	S	P	V	D	L	A	R	A	P	I	T	A
K	A	K	K	Q	P	T	D	N	Y	S	P	V	D	L	A	R	A	P	I	T	A
K	A	K	K	Q	P	T	D	N	Y	S	P	V	D	L	A	R	A	P	I	T	A
K	A	K	K	Q	P	T	D	N	Y	S	P	V	D	L	A	R	A	P	I	T	A
K	A	K	K	Q	P	T	D	N	Y	S	P	V	D	L	A	R	A	P	I	T	A
K	A	K	K	Q	P	T	D	N	Y	S	P	V	D	L	A	R	A	P	I	T	A
K	A	K	K	Q	P	T	D	N	Y	S	P	V	D	L	A	R	A	P	I	T	A
K	A	K	K	Q	P	T	D	N	Y	S	P	V	D	L	A	R	A	P	I	T	A
K	A	K	K	Q	P	T	D	N	Y	S	P	V	D	L	A	R	A	P	I	T	A
K	A	K	K	Q	P	T	D	N	Y	S	P	V	D	L	A	R	A	P	I	T	A
K	A	K	K	Q	P	T	D	N	Y	S	P	V	D	L	A	R	A	P	I	T	A
K	A	K	K	Q	P	T	D	N	Y	S	P	V	D	L	A	R	A	P	I	T	A
K	A	K	K	Q	P	T	D	N	Y	S	P	V	D	L	A	R	A	P	I	T	A
K	A	K	K	Q	P	T	D	N	Y	S	P	V	D	L	A	R	A	P	I	T	A
K	A	K	K	Q	P	T	D	N	Y	S	P	V	D	L	A	R	A	P	I	T	A
K	A	K	K	Q	P	T	D	N	Y	S	P	V	D	L	A	R	A	P	I	T	A
K	A	K	K	Q	P	T	D	N	Y	S	P	V	D	L	A	R	A	P	I	T	A
K	A	K	K	Q	P	T	D	N	Y	S	P	V	D	L	A	R	A	P	I	T	A
K	A	K	K	Q	P	T	D	N	Y	S	P	V	D	L	A	R	A	P	I	T	A
K	A	K	K	Q	P	T	D	N	Y	S	P	V	D	L	A	R	A	P	I	T	A
K	A	K	K	Q	P	T	D	N	Y	S	P	V	D	L	A	R	A	P	I	T	A
K	A	K	K	Q	P	T	D	N	Y	S	P	V	D	L	A	R	A	P	I	T	A
K	A	K	K	Q	P	T	D	N	Y	S	P	V	D	L	A	R	A	P	I	T	A
K	A	K	K	Q	P	T	D	N	Y	S	P	V	D	L	A	R	A	P	I	T	A
K	A	K	K	Q	P	T	D	N	Y	S	P	V	D	L	A	R	A	P	I	T	A
K	A	K	K	Q	P	T	D	N	Y	S	P	V	D	L	A	R	A	P	I	T	A
K	A	K	K	Q	P	T	D	N	Y	S	P	V	D	L	A	R	A	P	I	T	A
K	A	K	K	Q	P	T	D	N	Y	S	P	V	D	L	A	R	A	P	I	T	A
K	A	K	K	Q	P	T	D	N	Y	S	P	V	D	L	A	R	A	P	I	T	A
K	A	K	K	Q	P	T	D	N	Y	S	P	V	D	L	A	R	A	P	I	T	A
K	A	K	K	Q	P	T	D	N	Y	S	P	V	D	L	A	R	A	P	I	T	A

K	A	K	K	Q	P	T	D	N	Y	S	P	V	D	L	A	R	A	P	I	T	A
K	A	K	K	Q	P	T	D	N	Y	S	P	V	D	L	A	R	A	P	I	T	A
K	A	K	K	Q	P	T	D	N	Y	S	P	V	D	L	A	R	A	P	I	T	A
K	A	K	K	Q	P	T	D	N	Y	S	P	V	D	L	A	R	A	P	I	T	A
K	A	K	K	Q	P	T	D	N	Y	S	P	V	D	L	A	R	A	P	I	T	A
K	A	K	K	Q	P	T	D	N	Y	S	P	V	D	L	A	R	A	P	I	T	A
K	A	K	K	Q	P	T	D	N	Y	S	P	V	D	L	A	R	A	P	I	T	A
K	A	K	K	Q	P	T	D	N	Y	S	P	V	D	L	A	R	A	P	I	T	A
K	A	K	K	Q	P	T	D	N	Y	S	P	V	D	L	A	R	A	P	I	T	A
K	A	K	K	Q	P	T	D	N	Y	S	P	V	D	L	A	R	A	P	I	T	A
K	A	K	K	Q	P	T	D	N	Y	S	P	V	D	L	A	R	A	P	I	T	A
T	A	K	K	H	P	E	N	N	Y	S	P	V	D	L	A	R	A	S	I	T	A
T	A	K	K	H	P	E	N	N	Y	S	P	V	D	L	A	R	A	S	I	T	A
T	A	K	K	H	P	E	N	N	Y	S	P	V	D	L	A	R	A	S	I	T	A
T	A	K	K	H	P	E	N	N	Y	S	P	V	D	L	A	R	A	S	I	T	A
T	A	K	K	H	P	E	N	N	Y	S	P	V	D	L	A	R	A	S	I	T	A
T	A	K	K	H	P	E	N	N	Y	S	P	V	D	L	A	R	A	S	I	T	A
T	A	K	K	H	P	E	N	N	Y	S	P	V	D	L	A	R	A	S	I	T	A
T	A	K	K	H	P	E	N	N	Y	S	P	V	D	L	A	R	A	S	I	T	A
T	A	K	K	H	P	E	N	N	Y	S	P	V	D	L	A	R	A	S	I	T	A
K	A	Q	K	Q	P	T	D	N	N	G	A	I	N	L	A	C	E	P	I	S	A
K	A	Q	K	Q	P	T	D	N	N	G	A	I	N	L	A	C	E	P	I	S	A
K	A	Q	K	Q	P	T	D	N	N	G	A	I	N	L	A	C	E	P	I	S	A
K	A	Q	K	Q	P	T	D	N	N	G	A	I	N	L	A	C	E	P	I	S	A
K	A	Q	K	Q	P	T	D	N	N	G	A	I	N	L	A	C	E	P	I	S	A
K	A	Q	K	Q	P	T	D	N	N	G	A	I	N	L	A	C	E	P	I	S	A
K	A	Q	K	Q	P	T	D	N	N	G	A	I	N	L	A	C	E	P	I	S	A
K	A	Q	K	Q	P	T	D	N	N	G	A	I	N	L	A	C	E	P	I	S	A
K	A	K	K	H	P	T	D	N	Y	S	P	V	D	L	A	R	A	P	I	T	A
K	A	K	K	Q	P	T	D	N	H	S	P	V	G	V	S	R	A	P	I	S	A
K	A	K	K	Q	P	T	D	N	H	S	P	V	G	V	S	R	A	P	I	S	A
K	A	K	K	Q	P	T	D	N	H	S	P	V	G	V	S	R	A	P	I	S	A
K	A	K	K	Q	P	T	D	N	H	S	P	V	G	V	S	R	A	P	I	S	A
K	A	K	K	Q	P	T	D	N	H	S	P	V	G	V	S	R	A	P	I	S	A
K	A	K	K	Q	P	T	D	N	H	S	P	V	G	V	S	R	A	P	I	S	A
K	A	K	K	Q	P	T	D	N	H	S	P	V	G	V	S	R	A	P	I	S	A
K	A	K	K	Q	P	T	D	N	H	S	P	V	G	V	S	R	A	P	I	S	A
K	A	K	K	Q	P	T	D	N	H	S	P	V	G	V	S	R	A	P	I	S	A
K	A	K	K	Q	P	T	D	N	H	S	P	V	G	V	S	R	A	P	I	S	A
K	A	K	K	Q	P	T	D	N	H	S	P	V	G	V	S	R	A	P	I	S	A
K	A	K	K	Q	P	T	D	N	H	S	P	V	G	V	S	R	A	P	I	S	A
K	A	K	K	Q	P	T	D	N	H	S	P	V	G	V	S	R	A	P	I	S	A
K	A	K	K	Q	P	T	D	N	H	S	P	V	G	V	S	R	A	P	I	S	A
K	A	K	K	Q	P	T	D	N	H	S	P	V	G	V	S	R	A	P	I	S	A
K	A	K	K	Q	P	T	D	N	H	S	P	V	G	V	S	R	A	P	I	S	A
K	A	K	K	Q	P	T	D	N	H	S	P	V	G	V	S	R	A	P	I	S	A
K	A	K	K	Q	P	T	D	N	H	S	P	V	G	V	S	R	A	P	I	S	A
K	A	K	K	Q	P	T	D	N	H	S	P	V	G	V	S	R	A	P	I	S	A
K	A	K	K	H	P	T	D	N	N	G	A	I	N	L	A	C	E	P	I	S	A
K	A	K	K	H	P	T	D	N	N	G	A	I	N	L	A	C	E	P	I	S	A
K	A	K	K	H	P	T	D	N	N	G	A	I	N	L	A	C	E	P	I	S	A
K	A	K	K	H	P	T	D	N	N	G	A	I	N	L	A	C	E	P	I	S	A
K	A	K	K	H	P	T	D	N	N	G	A	I	N	L	A	C	E	P	I	S	A
K	A	K	K	H	P	T	D	N	N	G	A	I	N	L	A	C	E	P	I	S	A
K	A	K	K	H	P	T	D	N	N	G	A	I	N	L	A	C	E	P	I	S	A
K	A	K	K	H	P	T	D	N	N	G	A	I	N	L	A	C	E	P	I	S	A
K	A	K	K	H	P	T	D	N	N	G	A	I	N	L	A	C	E	P	I	S	A
T	A	K	K	H	P	E	N	N	Y	S	P	V	D	L	A	R	A	S	V	T	A
K	A	K	K	H	P	E	N	N	Y	S	P	V	D	L	A	R	A	S	I	T	A
K	A	K	K	H	P	E	N	N	Y	S	P	V	D	L	A	R	A	S	I	T	A
K	A	K	K	H	P	E	N	N	Y	S	P	V	D	L	A	R	A	S	I	T	A
K	A	K	K	H	P	E	N	N	Y	S	P	V	D	L	A	R	A	S	I	T	A
K	A	K	K	H	P	E	N	N	Y	S	P	V	D	L	A	R	A	S	I	T	A
K	A	K	K	H	P	E	N	N	Y	S	P	V	D	L	A	R	A	S	I	T	A
K	A	K	K	H	P	E	N	N	Y	S	P	V	D	L	A	R	A	S	I	T	A
K	A	K	K	H	P	E	N	N	Y	S	P	V	D	L	A	R	A	S	I	T	A
K	A	K	K	H	P	E	N	N	Y	S	P	V	D	L	A	R	A	S	I	T	A

K	A	K	K	H	P	E	N	N	Y	S	P	V	D	L	A	R	A	S	I	T	A
K	A	K	K	H	P	E	N	N	Y	S	P	V	D	L	A	R	A	S	I	T	A
K	A	K	K	H	P	E	N	N	Y	S	P	V	D	L	A	R	A	S	I	T	A
K	A	K	K	H	P	E	N	N	Y	S	P	V	D	L	A	R	A	S	I	T	A
K	A	K	K	H	P	E	N	N	Y	S	P	V	D	L	A	R	A	S	I	T	A
K	A	K	K	H	P	E	N	N	Y	S	P	V	D	L	A	R	A	S	I	T	A
K	A	K	K	H	P	E	N	N	Y	S	P	V	D	L	A	R	A	S	I	T	A
K	A	K	K	H	P	E	N	N	Y	S	P	V	D	L	A	R	A	S	I	T	A
K	A	K	K	H	P	E	N	N	Y	S	P	V	D	L	A	R	A	S	I	T	A
K	A	K	K	H	P	E	N	N	Y	S	P	V	D	L	A	R	A	S	I	T	A
K	A	K	K	H	P	E	N	N	Y	S	P	V	D	L	A	R	A	S	I	T	A
K	A	K	K	H	P	E	N	N	Y	S	P	V	D	L	A	R	A	S	I	T	A
K	A	K	K	H	P	E	N	N	Y	S	P	V	D	L	A	R	A	S	I	T	A
K	A	K	K	H	P	E	N	N	Y	S	P	V	D	L	A	R	A	S	I	T	A
K	A	K	K	H	P	E	N	N	Y	S	P	V	D	L	A	R	A	S	I	T	A
K	A	K	K	H	P	E	N	N	Y	S	P	V	D	L	A	R	A	S	I	T	A
K	A	K	K	H	P	E	N	N	Y	S	P	V	D	L	A	R	A	S	I	T	A
K	A	K	K	H	P	E	N	N	Y	S	P	V	D	L	A	R	A	S	I	T	A
K	A	K	K	H	P	E	N	N	Y	S	P	V	D	L	A	R	A	S	I	T	A
K	A	K	K	H	P	E	N	N	Y	S	P	V	D	L	A	R	A	S	I	T	A
K	A	K	K	H	P	E	N	N	Y	S	P	V	D	L	A	R	A	S	I	T	A
K	A	K	K	H	P	E	N	N	Y	S	P	V	D	L	A	R	A	S	I	T	A
K	A	K	K	H	P	E	N	N	Y	S	P	V	D	L	A	R	A	S	I	T	A
K	A	K	K	H	P	T	D	N	Y	S	P	V	D	L	S	R	E	P	I	S	V
K	A	K	K	Q	P	T	D	N	H	S	P	V	G	V	S	R	A	P	I	T	A
K	A	K	K	Q	P	T	D	N	H	S	P	V	G	V	S	R	A	P	I	T	A
K	A	K	K	Q	P	T	D	N	H	S	P	V	G	V	S	R	A	P	I	T	A
K	A	K	K	Q	P	T	D	N	H	S	P	V	G	V	S	R	A	P	I	T	A
K	A	K	K	Q	P	T	D	N	H	S	P	V	G	V	S	R	A	P	I	T	A
K	A	K	K	Q	P	T	D	N	H	S	P	V	G	V	S	R	A	P	I	T	A
K	A	K	K	Q	P	T	D	N	H	S	P	V	G	V	S	R	A	P	I	T	A
K	A	K	K	Q	P	T	D	N	H	S	P	V	G	V	S	R	A	P	I	T	A
K	A	K	K	Q	P	T	D	N	H	S	P	V	G	V	S	R	A	P	I	T	A
K	A	K	K	Q	P	T	D	N	H	S	P	V	G	V	S	R	A	P	I	T	A
K	A	K	K	Q	P	T	D	N	H	S	P	V	G	V	S	R	A	P	I	T	A
K	A	K	K	Q	P	T	D	N	H	S	P	V	G	V	S	R	A	P	I	T	A
K	A	K	K	Q	P	T	D	N	H	S	P	V	G	V	S	R	A	P	I	T	A
K	A	K	K	Q	P	T	D	N	H	S	P	V	G	V	S	R	A	P	I	T	A
K	A	K	K	Q	P	T	D	N	H	S	P	V	G	V	S	R	A	P	I	T	A
K	A	K	K	Q	P	T	D	N	H	S	P	V	G	V	S	R	A	P	I	T	A
K	A	K	K	Q	P	T	D	N	H	S	P	V	G	V	S	R	A	P	I	T	A
K	A	K	K	Q	P	T	D	N	H	S	P	V	G	V	S	R	A	P	I	T	A
K	A	K	K	Q	P	T	D	N	H	S	P	V	G	V	S	R	A	P	I	T	A
K	A	K	K	Q	P	T	D	N	H	S	P	V	G	V	S	R	A	P	I	T	A
K	A	K	K	Q	P	T	D	N	H	S	P	V	G	V	S	R	A	P	I	T	A
K	A	K	K	Q	P	T	D	N	H	S	P	V	G	V	S	R	A	P	I	T	A
K	A	K	K	Q	P	T	D	N	H	S	P	V	G	V	S	R	A	P	I	T	A
K	A	K	K	Q	P	T	D	N	H	S	P	V	G	V	S	R	A	P	I	T	A
K	A	K	K	Q	P	T	D	N	H	S	P	V	G	V	S	R	A	P	I	T	A
K	V	S	S	H	P	K	D	N	H	S	P	V	D	V	A	R	T	P	I	T	A
K	V	S	S	H	P	K	D	N	H	S	P	V	D	V	A	R	T	P	I	T	A
K	V	S	S	H	P	K	D	N	H	S	P	V	D	V	A	R	T	P	I	T	A
K	V	S	S	H	P	K	D	N	H	S	P	V	D	V	A	R	T	P	I	T	A
K	V	S	S	H	P	K	D	N	H	S	P	V	D	V	A	R	T	P	I	T	A
K	V	S	S	H	P	K	D	N	H	S	P	V	D	V	A	R	T	P	I	T	A
K	V	S	S	H	P	K	D	N	H	S	P	V	D	V	A	R	T	P	I	T	A
K	V	S	S	H	P	K	D	N	H	S	P	V	D	V	A	R	T	P	I	T	A
K	V	S	S	H	P	K	D	N	H	S	P	V	D	V	A	R	T	P	I	T	A
K	V	S	S	H	P	K	D	N	H	S	P	V	D	V	A	R	T	P	I	T	A
K	V	S	S	H	P	K	D	N	H	S	P	V	D	V	A	R	T	P	I	T	A
K	V	S	S	H	P	K	D	N	H	S	P	V	D	V	A	R	T	P	I	T	A
K	V	S	S	H	P	K	D	N	H	S	P	V	D	V	A	R	T	P	I	T	A
K	V	S	S	H	P	K	D	N	H	S	P	V	D	V	A	R	T	P	I	T	A
K	V	S	S	H	P	K	D	N	H	S	P	V	D	V	A	R	T	P	I	T	A
K	V	S	S	H	P	K	D	N	H	S	P	V	D	V	A	R	T	P	I	T	A
K	V	S	S	H	P	K	D	N	H	S	P	V	D	V	A	R	T	P	I	T	A
K	V	S	S	H	P	K	D	N	H	S	P	V	D	V	A	R	T	P	I	T	A

K	V	S	S	H	P	K	D	N	H	S	P	V	D	V	A	R	T	P	I	T	A
K	V	S	S	H	P	K	D	N	H	S	P	V	D	V	A	R	T	P	I	T	A
K	V	S	S	H	P	K	D	N	H	S	P	V	D	V	A	R	T	P	I	T	A
K	V	S	S	H	P	K	D	N	H	S	P	V	D	V	A	R	T	P	I	T	A
K	V	S	S	H	P	K	D	N	H	S	P	V	D	V	A	R	T	P	I	T	A
K	V	S	S	H	P	K	D	N	H	S	P	V	D	V	A	R	T	P	I	T	A
K	V	S	S	H	P	K	D	N	H	S	P	V	D	V	A	R	T	P	I	T	A
K	V	S	S	H	P	K	D	N	H	S	P	V	D	V	A	R	T	P	I	T	A
K	V	S	S	H	P	K	D	N	H	S	P	V	D	V	A	R	T	P	I	T	A
K	V	S	S	H	P	K	D	N	H	S	P	V	D	V	A	R	T	P	I	T	A

rmlC								rmlB		rmlD	
2	12	15	16	21	46	57	89	275	300	33	245
T	A	V	E	M	L	E	G	V	E	E	V
S	V	I	D	L	E	A	D	A	D	K	A
T	A	V	E	M	L	E	G	A	D	K	A
S	V	I	D	L	E	A	D	A	D	K	A
S	V	I	D	L	E	A	D	A	D	K	A
S	V	I	D	L	E	A	D	A	D	K	A
S	V	I	D	L	E	A	D	A	D	K	A
S	V	I	D	L	E	A	D	A	D	K	A
S	V	I	D	L	E	A	D	A	D	K	A
S	V	I	D	L	E	A	D	A	D	K	A
S	V	I	D	L	E	A	D	A	D	K	A

rmlC						rmlB			rmlD				
16	21	46	57	89	152	73	275	300	32	33	38	39	245
E	M	L	E	G	P	A	V	E	A	E	N	E	V
D	L	E	A	D	P	A	A	D	A	K	N	E	A
D	L	E	A	D	P	A	A	D	A	K	N	E	A
D	L	E	A	D	P	A	A	D	A	K	N	E	A
D	L	E	A	D	P	A	A	D	A	K	N	E	A
D	L	E	A	D	P	A	A	D	A	K	N	E	A
D	L	E	A	D	P	A	A	D	A	K	N	E	A
D	L	E	A	D	P	A	A	D	A	K	N	E	A
D	L	E	A	D	P	A	A	D	A	K	N	E	A
D	L	E	A	D	P	A	A	D	A	K	N	E	A
D	L	E	A	D	P	A	A	D	A	K	N	E	A
D	L	E	A	D	P	A	A	D	A	K	N	E	A
D	L	E	A	D	P	A	A	D	A	K	N	E	A
D	L	E	A	D	P	A	A	D	A	K	N	E	A
D	L	E	A	D	P	A	A	D	A	K	N	E	A
D	L	E	A	D	P	A	A	D	A	K	N	E	A
D	L	E	A	D	P	A	A	D	A	K	N	E	A
D	L	E	A	D	L	A	A	D	A	K	N	E	A
D	L	E	A	D	P	A	A	D	A	K	N	E	A
D	L	E	A	D	P	A	A	D	A	K	N	E	A
D	L	E	A	D	P	A	A	D	A	K	N	E	A
D	L	E	A	D	P	A	A	D	A	K	N	E	A
D	L	E	A	D	P	A	A	D	A	K	N	E	A
D	L	E	A	D	P	A	A	D	A	K	N	E	A
D	L	E	A	D	P	A	A	D	A	K	N	E	A
E	M	L	E	G	P	V	A	D	V	E	D	A	A
D	L	E	A	D	P	A	A	D	A	K	N	E	A
D	L	E	A	D	P	A	A	D	A	K	N	E	A
D	L	E	A	D	P	A	A	D	A	K	N	E	A
D	L	E	A	D	P	A	A	D	A	K	N	E	A
D	L	E	A	D	P	A	A	D	A	K	N	E	A

	rmlB	rmlD

57	89	182
E	G	N
A	D	N
E	G	H
E	G	H
E	G	H
E	G	H
E	G	H
E	G	H
E	G	H
E	G	H
E	G	H
E	G	H
E	G	H
E	G	H
E	G	H
E	G	H
E	G	H
E	G	H
E	G	H
E	G	H
E	G	H
E	G	H
E	G	H
E	G	H
E	G	H
E	G	H
E	G	H

275	285	300
V	A	E
A	A	D
A	A	D
V	A	D
V	A	D
A	A	D
A	A	D
A	A	D
A	A	D
V	A	D
V	A	D
V	A	D
A	S	D
A	A	D
A	A	D
A	A	D
A	A	D
A	A	D
A	A	D
A	A	D
A	A	D
A	A	D
A	A	D
V	A	D
A	A	E
V	A	D
A	A	E

33	38	39	65	71	87	230	245
E	N	E	A	K	K	D	V
K	N	E	A	K	K	D	A
E	D	A	A	K	K	D	A
E	N	E	A	K	K	D	A
E	N	E	A	K	K	D	A
E	D	A	A	K	K	D	A
E	D	A	A	K	K	D	A
E	D	A	A	K	K	D	A
E	D	A	A	K	K	D	A
E	N	E	A	K	K	D	A
E	N	E	A	K	K	D	A
E	N	E	A	K	K	D	A
E	D	A	A	K	K	D	A
E	D	A	A	K	K	D	A
E	D	A	A	K	K	D	A
E	D	A	A	K	K	D	A
E	D	A	A	K	K	D	A
E	D	A	A	K	K	D	A
E	D	A	A	K	K	D	A
E	D	A	A	K	K	D	A
E	D	A	A	K	K	D	A
E	D	A	A	K	K	D	A
E	D	A	A	K	K	D	A
E	D	A	A	R	K	G	A
E	N	E	A	K	R	D	A
E	D	A	A	R	K	G	A
E	N	E	T	K	R	D	A

rmlC																					
12	15	16	21	46	52	57	66	89	108	112	149	152	155	163	167	171	182	187	193	195	196
A	V	E	M	L	E	E	F	G	T	V	E	P	A	S	E	I	N	K	K	E	D
A	V	E	M	L	E	E	F	G	T	V	E	P	A	S	K	I	H	K	K	E	D
A	V	E	M	L	A	E	F	G	T	V	E	P	A	S	E	I	N	K	K	E	D
A	V	E	M	L	A	E	F	G	T	V	E	P	A	S	E	I	N	K	K	E	D
A	V	E	M	L	A	E	F	G	T	V	E	P	A	S	E	I	N	K	K	E	D
A	V	E	M	L	A	E	F	G	T	V	E	P	A	S	E	I	N	K	K	E	D
A	V	E	M	L	A	E	F	G	T	V	E	P	A	S	E	I	N	K	K	E	D
A	V	E	M	L	A	E	F	G	T	V	E	P	A	S	E	I	N	K	K	E	D
A	V	E	M	L	A	E	F	G	T	V	E	P	A	S	E	I	N	K	K	E	D
A	V	E	M	L	A	E	F	G	T	V	E	P	A	S	E	I	N	K	K	E	D
A	V	E	M	L	A	E	F	G	T	V	E	P	A	S	E	I	N	K	K	E	D
A	V	E	M	L	A	E	F	G	T	V	E	P	A	S	E	I	N	K	K	E	D
A	V	E	M	L	A	E	F	G	T	V	E	P	A	S	E	I	N	K	K	E	D
A	V	E	M	L	A	E	F	G	T	V	E	P	A	S	E	I	N	K	K	E	D
A	V	E	M	L	A	E	F	G	T	V	E	P	A	S	E	I	N	K	K	E	D
A	V	E	M	L	A	E	F	G	T	V	E	P	A	S	E	I	N	K	K	E	D
A	V	E	M	L	A	E	F	G	T	V	E	P	A	S	E	I	N	K	K	E	D
A	V	E	M	L	A	E	F	G	T	V	E	P	A	S	E	I	N	K	K	E	D
A	V	E	M	L	A	E	F	G	T	V	E	P	A	S	E	I	N	K	K	E	D
A	V	E	M	L	A	E	F	G	T	V	E	P	A	S	E	I	N	K	K	E	D
A	V	E	M	L	A	E	F	G	T	V	E	P	A	S	E	I	N	K	K	E	D
A	V	E	M	L	A	E	F	G	T	V	E	P	A	S	E	I	N	K	K	E	D
A	V	E	M	L	A	E	F	G	T	V	E	P	A	S	E	I	N	K	K	E	D
A	V	E	M	L	A	E	F	G	T	V	E	P	A	S	E	I	N	K	K	E	D
A	V	E	M	L	A	E	F	G	T	V	E	P	A	S	E	I	N	K	K	E	D
A	V	E	M	L	A	E	F	G	T	V	E	P	A	S	E	I	N	K	K	E	D
A	V	E	M	L	A	E	F	G	T	V	E	P	A	S	E	I	N	K	K	E	D
A	V	E	M	L	A	E	F	G	T	V	E	P	A	S	E	I	N	K	K	E	D
A	V	E	M	L	A	E	F	G	T	V	E	P	A	S	E	I	N	K	K	E	D
A	V	E	M	L	A	E	F	G	T	V	E	P	A	S	E	I	N	K	K	E	D
A	V	E	M	L	E	E	F	G	T	V	E	P	A	S	E	I	N	K	K	E	D
A	V	E	M	L	E	E	F	G	T	V	E	P	A	S	E	I	N	K	K	E	D
[truncated: 258,346 more chars]
